# Supplementary material for: Micropeptide hSPAR regulates glutamine levels and suppresses mammary tumor growth via a TRIM21-P27KIP1-mTOR axis
Source: EMBO J. 2025 Jan 28;44(5):1414–41. doi: 10.1038/s44318-024-00359-z (PMC11876615; doi:10.1038/s44318-024-00359-z)
Supplement: Supplementary file 4 — Appendix [file 44318_2024_359_MOESM4_ESM.pdf]

Appendix

Micropeptide hSPAR regulates glutamine levels and suppresses mammary tumor growth via a TRIM21-P27KIP1-mTOR axis

Yan Huang[1,2], Hua Lu[1,2#], Yao Liu[3#], Jiabei Wang[3#], Qingan Xia[4#], Xiangmin Shi[1,2#], Yan Jin[1,2], Xiaolin Liang[1,2], Wei Wang[1,2], Xiaopeng Ma[5], Yangyi Wang[5], Meng Gong[1,2], Canjun Li[5], Chunlei Cang[5], Qinghua Cui[6,7], Ceshi Chen[8,9], Tao Shen[10*], Lianxin Liu[3*], Xiangting Wang[1,2,11*]

1 Department of Geriatrics, Gerontology Institute of Anhui Province, Centre for Leading Medicine and Advanced Technologies of IHM, The First Affiliated Hospital of USTC, Division of Life Sciences and Medicine, University of Science and Technology of China, Hefei, Anhui, China

2 Anhui Provincial Key Laboratory of Tumor Immunotherapy and Nutrition Therapy, Hefei, Anhui, China

3 Department of Hepatobiliary Surgery, Centre for Leading Medicine and Advanced Technologies of IHM, The First Affiliated Hospital of USTC, Division of Life Sciences and Medicine, University of Science and Technology of China, Hefei, Anhui, China

4 Department of Pathology, Tangshan Gongren Hospital, Tangshan, Hebei China

5 Division of Life Sciences and Medicine, University of Science and Technology of China, Hefei, Anhui, China

6 School of Sports Medicine, Wuhan Institute of Physical Education, Wuhan, Hubei, China

7 Department of Biomedical Informatics, Centre for Noncoding RNA Medicine, State Key Laboratory of Vascular Homeostasis and Remodeling, School of Basic Medical Sciences, Peking University, Beijing, China

8 Yunnan Key Laboratory of Breast Cancer Precision Medicine, Academy of Biomedical Engineering, Kunming Medical University, Kunming, Yunnan, China

9 Yunnan Key Laboratory of Breast Cancer Precision Medicine, Yunnan Cancer Hospital, The Third Affiliated Hospital of Kunming Medical University,

Peking University Cancer Hospital Yunnan, Kunming, Yunnan, China
10 Anhui Provincial Key Laboratory of Molecular Enzymology and Mechanism of Major Metabolic Diseases, Anhui Provincial Engineering Research Centre for Molecular Detection and Diagnostics, College of Life Sciences, Anhui Normal University, Wuhu, Anhui, China

11 Lead contact
[#]These authors contributed equally.
[*]Correspondence: wangxt11@ustc.edu.cn, Liulx@ustc.edu.cn,
stao@ahnu.edu.cn

Table of contents

Appendix Figure S1 4

Appendix Figure S1

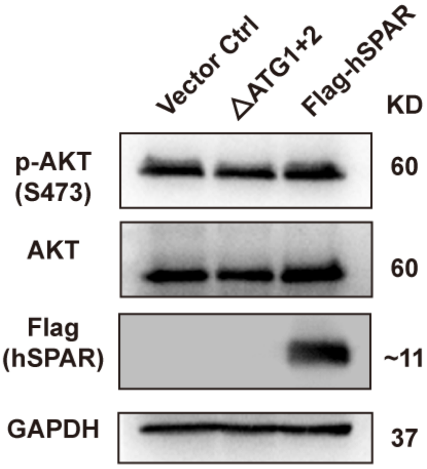

Appendix Figure S1: Overexpression hSPAR does not affect serine 473 phosphorylation of AKT. Immunoblotting against p-AKT, AKT, Flag and GAPDH in extracts from MDA-MB-231 cells transfected with Vector Ctrl, ΔATG1+2, or Flag-hSPAR (n=3 independent biological samples).
