# Supplementary figures and images for: Micropeptide hSPAR regulates glutamine levels and suppresses mammary tumor growth via a TRIM21-P27KIP1-mTOR axis (part 1 of 7)
Source: EMBO J. 2025 Jan 28;44(5):1414–41. doi: 10.1038/s44318-024-00359-z (PMC11876615; doi:10.1038/s44318-024-00359-z)

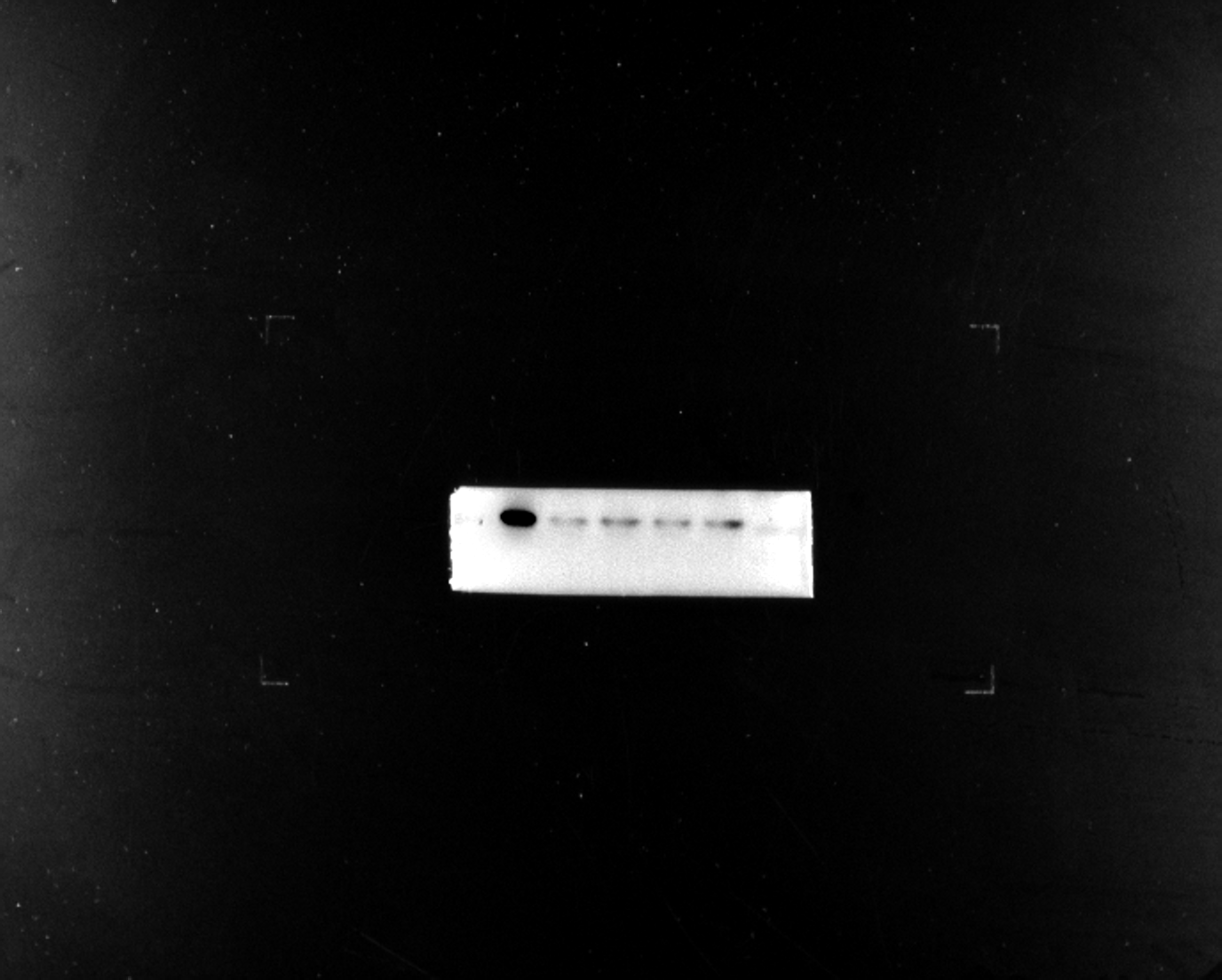

Supplement: Supplementary file 6 — Source data Fig. 1 [file 44318_2024_359_MOESM6_ESM.zip › Figure 1/Fig 1D/1-hSPAR-merge.Tif]

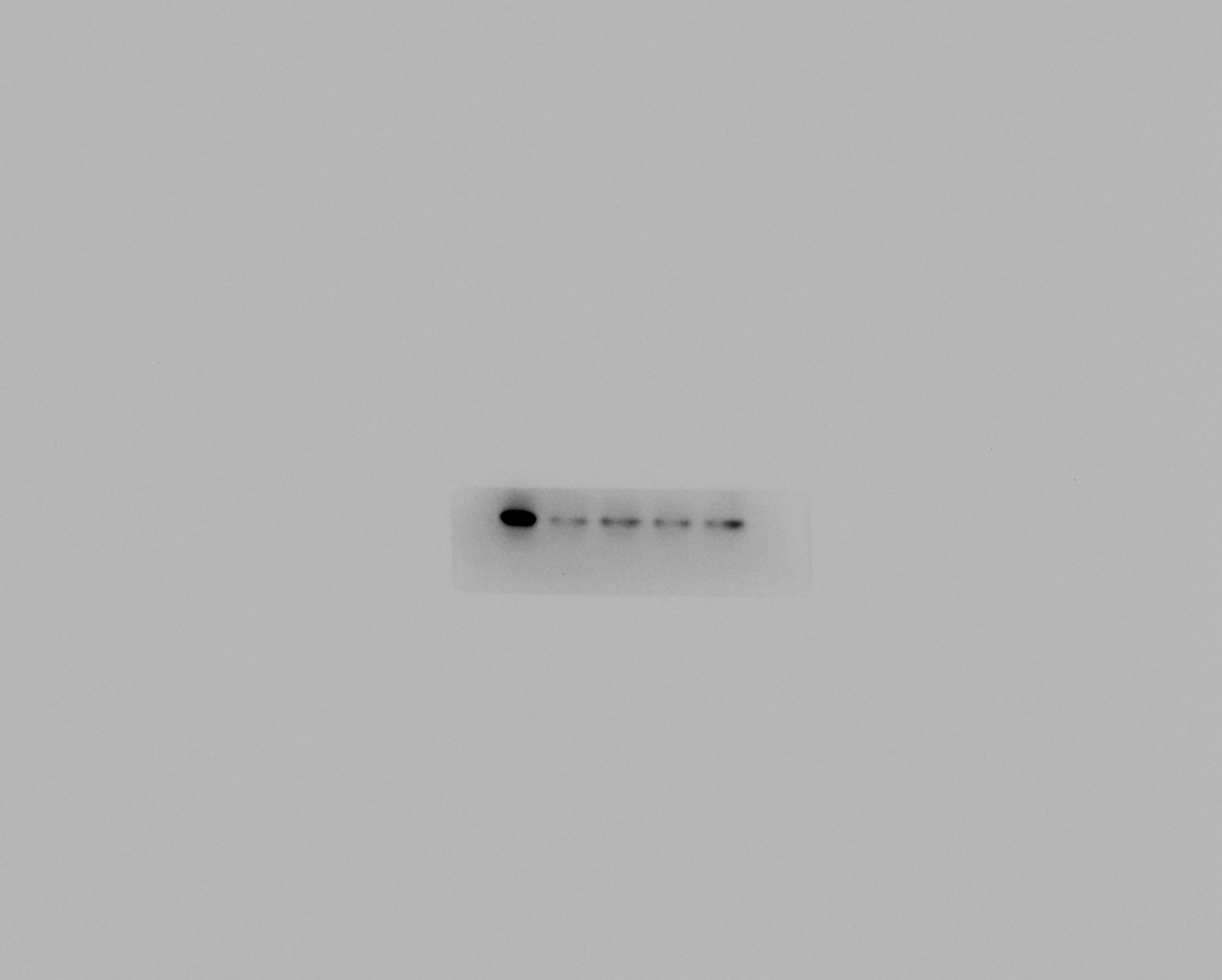

Supplement: Supplementary file 6 — Source data Fig. 1 [file 44318_2024_359_MOESM6_ESM.zip › Figure 1/Fig 1D/1-hSPAR.Tif]

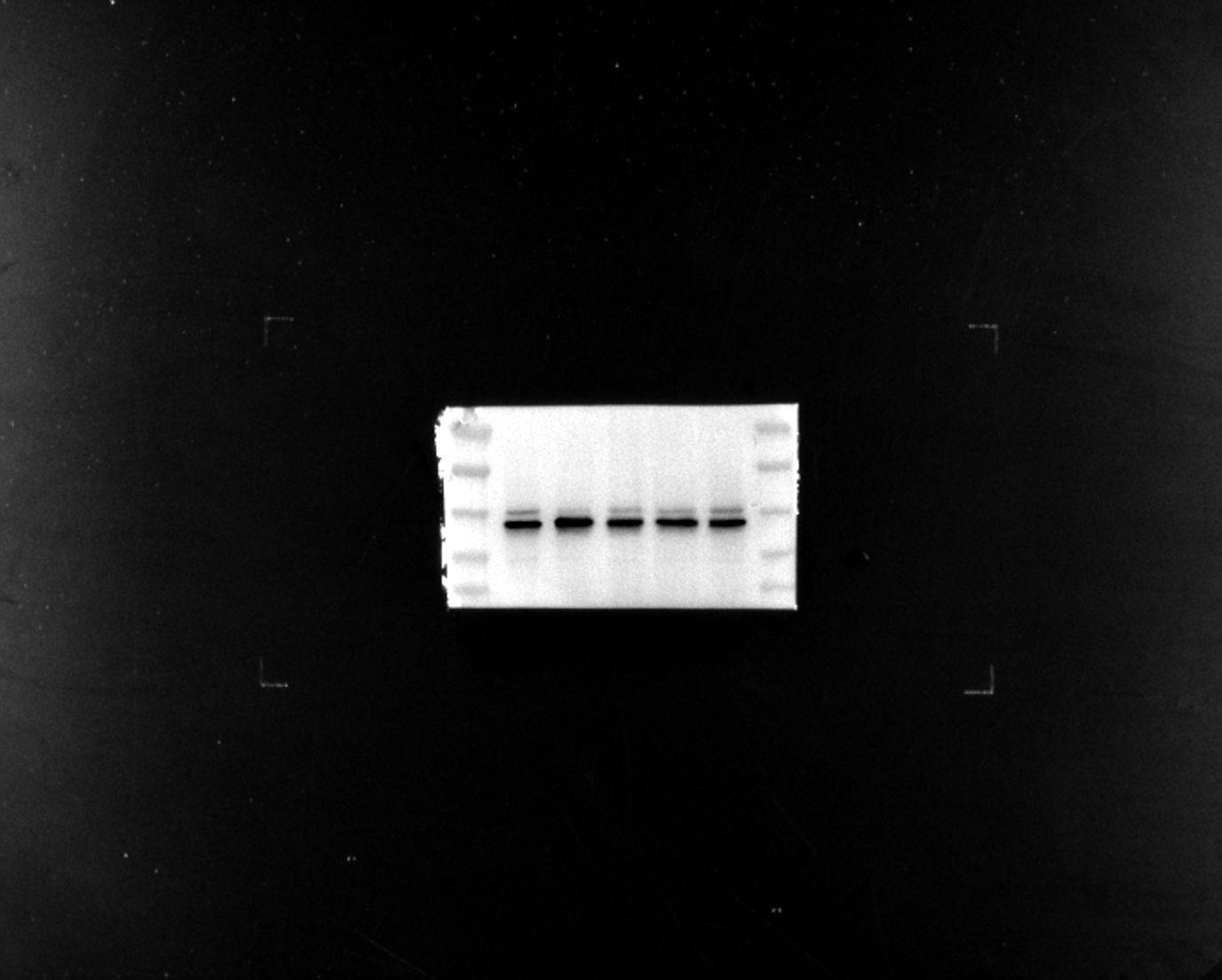

Supplement: Supplementary file 6 — Source data Fig. 1 [file 44318_2024_359_MOESM6_ESM.zip › Figure 1/Fig 1D/2-GAPDH-merge.Tif]

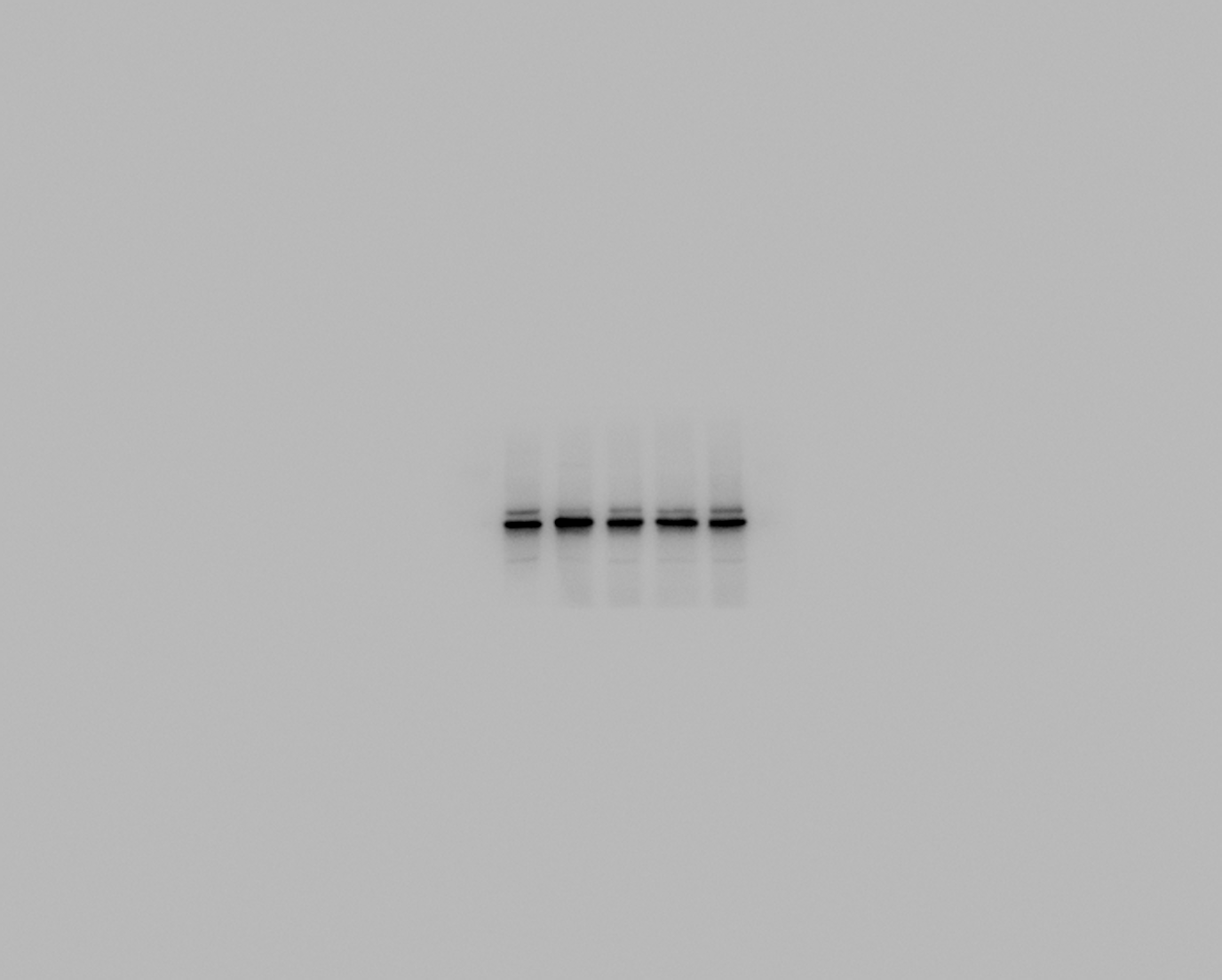

Supplement: Supplementary file 6 — Source data Fig. 1 [file 44318_2024_359_MOESM6_ESM.zip › Figure 1/Fig 1D/2-GAPDH.Tif]

**Fig 1D**

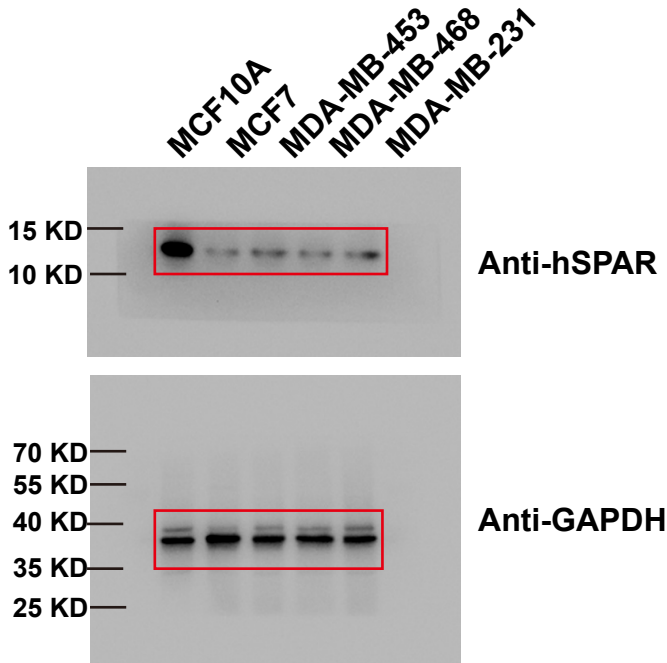

Supplement: Supplementary file 6 — Source data Fig. 1 [file 44318_2024_359_MOESM6_ESM.zip › Figure 1/Fig 1D/Fig 1D.pdf]

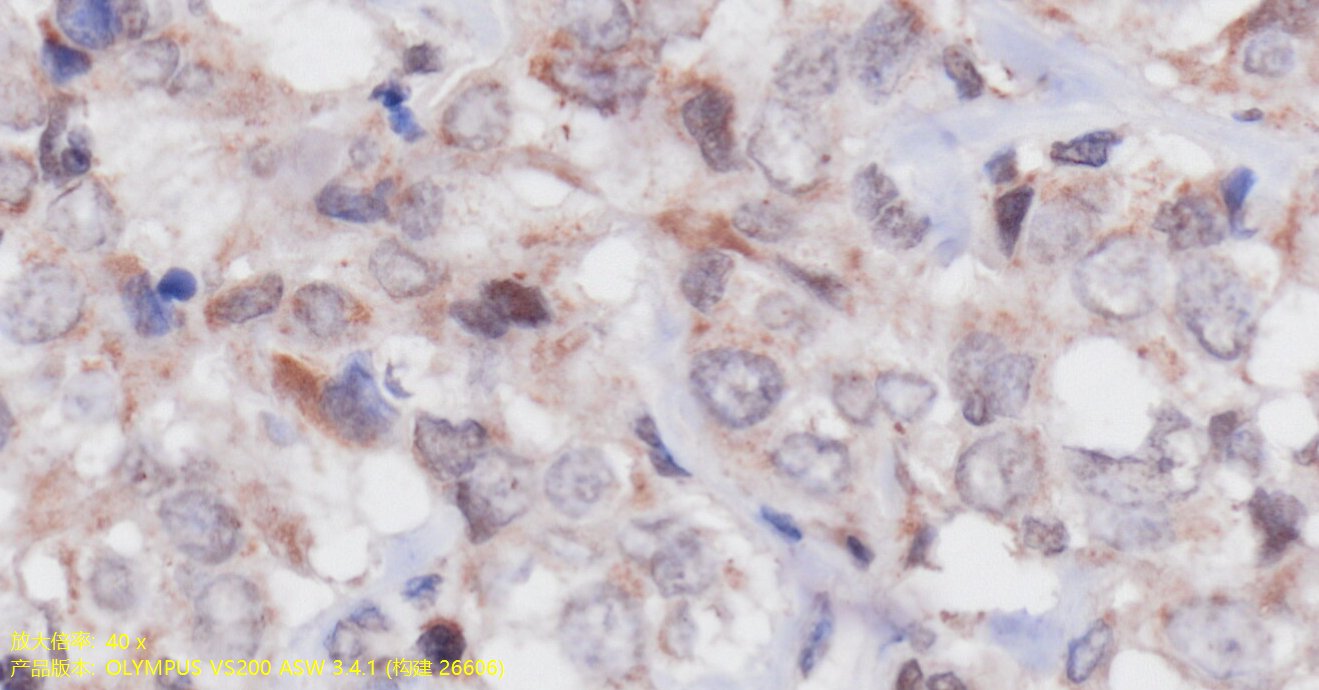

Supplement: Supplementary file 6 — Source data Fig. 1 [file 44318_2024_359_MOESM6_ESM.zip › Figure 1/Fig 1E/1-C.jpg]

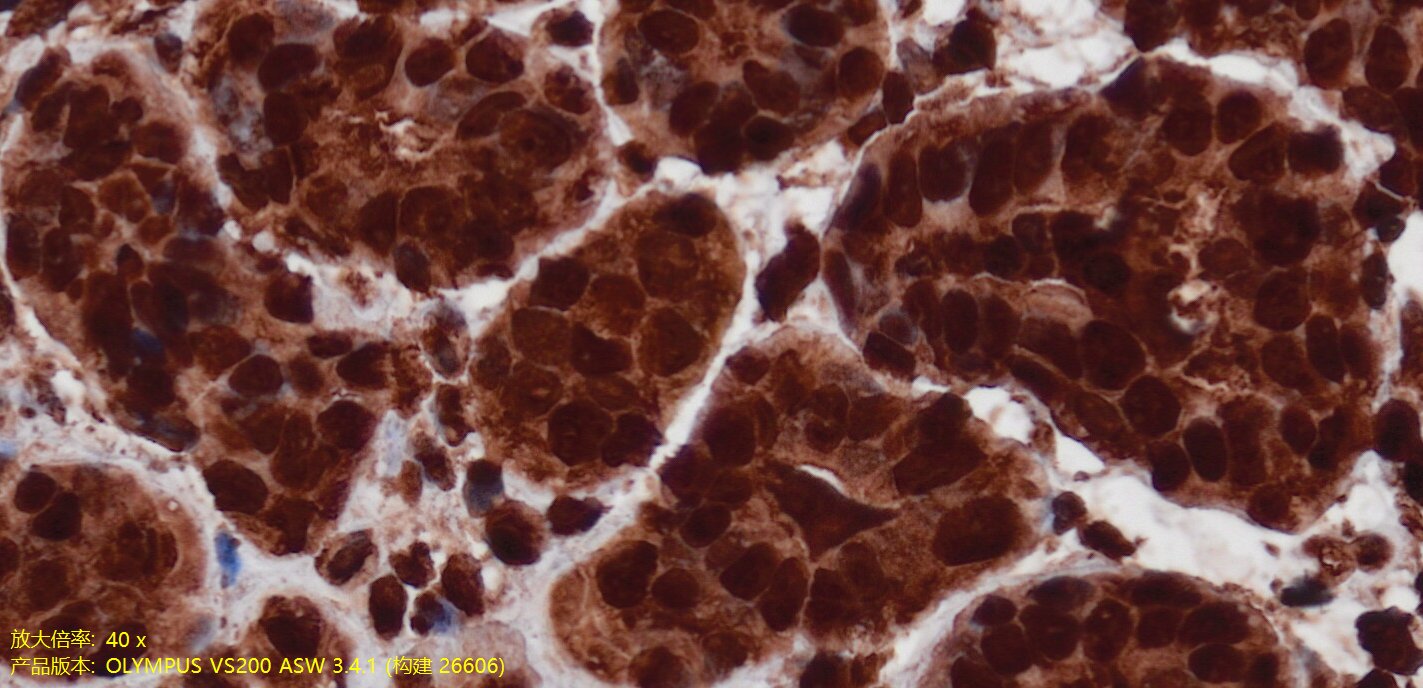

Supplement: Supplementary file 6 — Source data Fig. 1 [file 44318_2024_359_MOESM6_ESM.zip › Figure 1/Fig 1E/1-P.jpg]

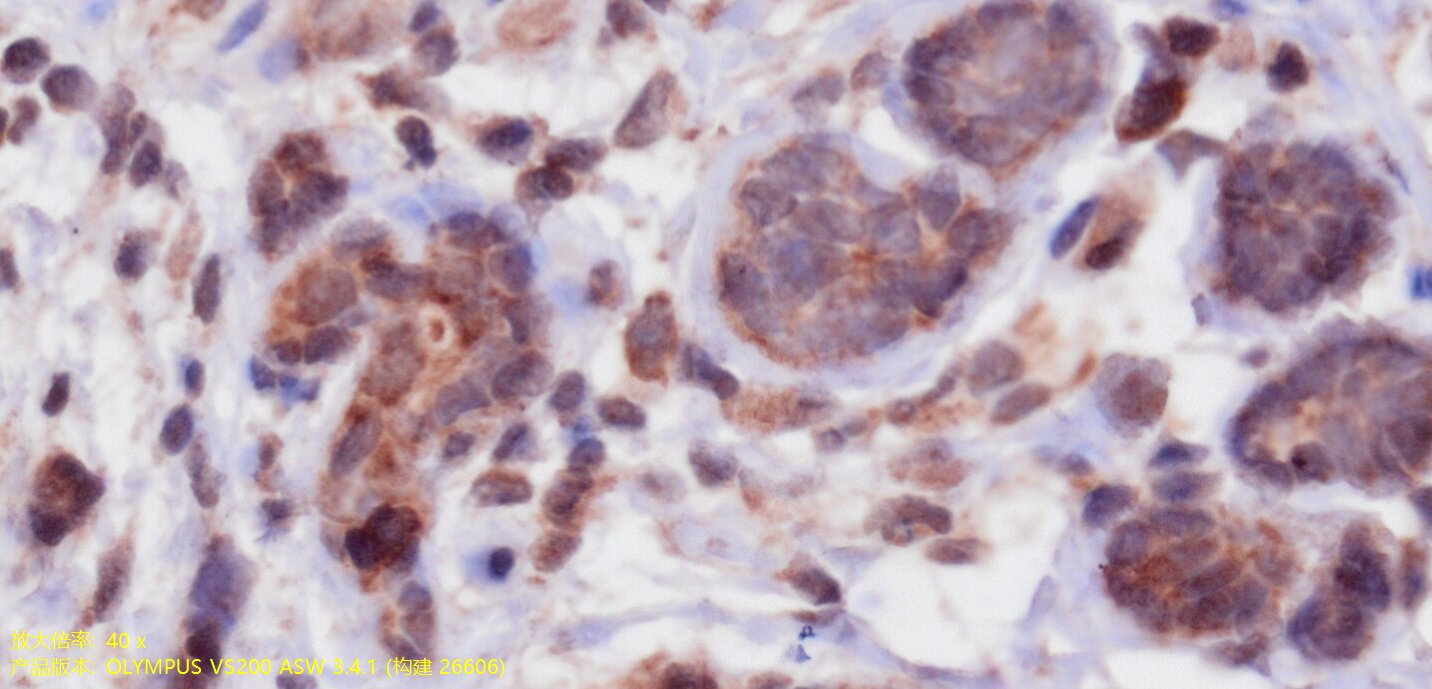

Supplement: Supplementary file 6 — Source data Fig. 1 [file 44318_2024_359_MOESM6_ESM.zip › Figure 1/Fig 1E/10-C.jpg]

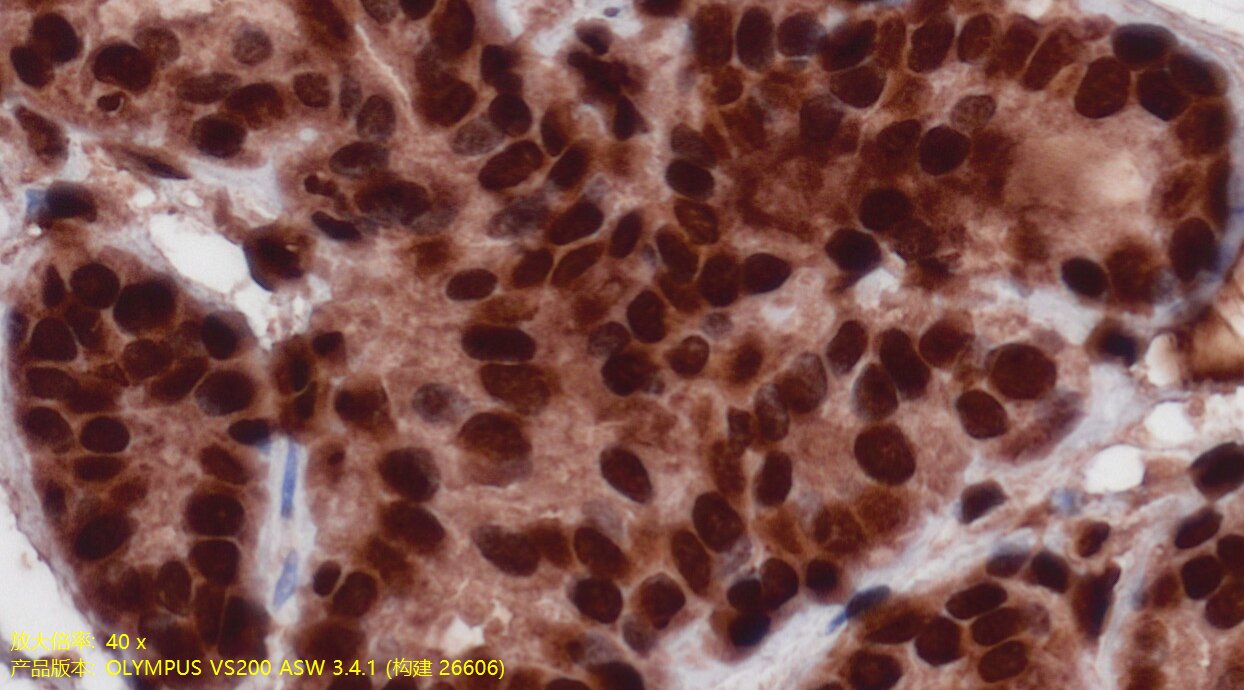

Supplement: Supplementary file 6 — Source data Fig. 1 [file 44318_2024_359_MOESM6_ESM.zip › Figure 1/Fig 1E/10-P.jpg]

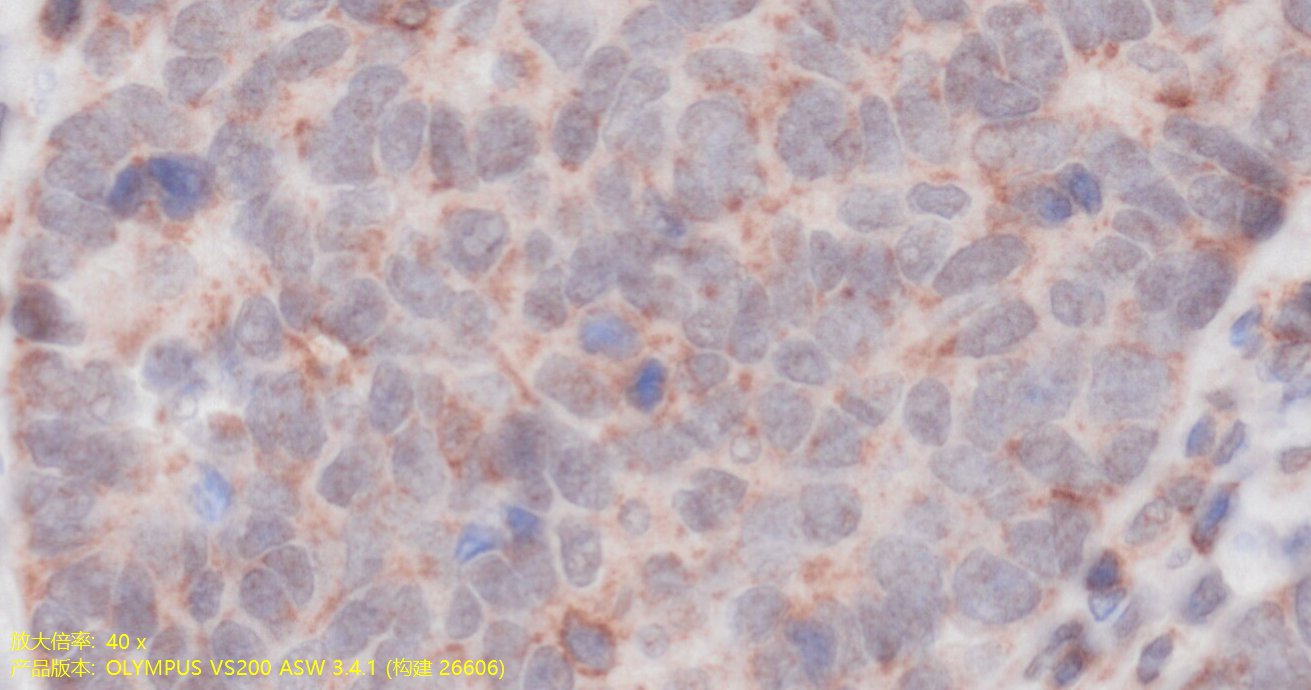

Supplement: Supplementary file 6 — Source data Fig. 1 [file 44318_2024_359_MOESM6_ESM.zip › Figure 1/Fig 1E/2-C.jpg]

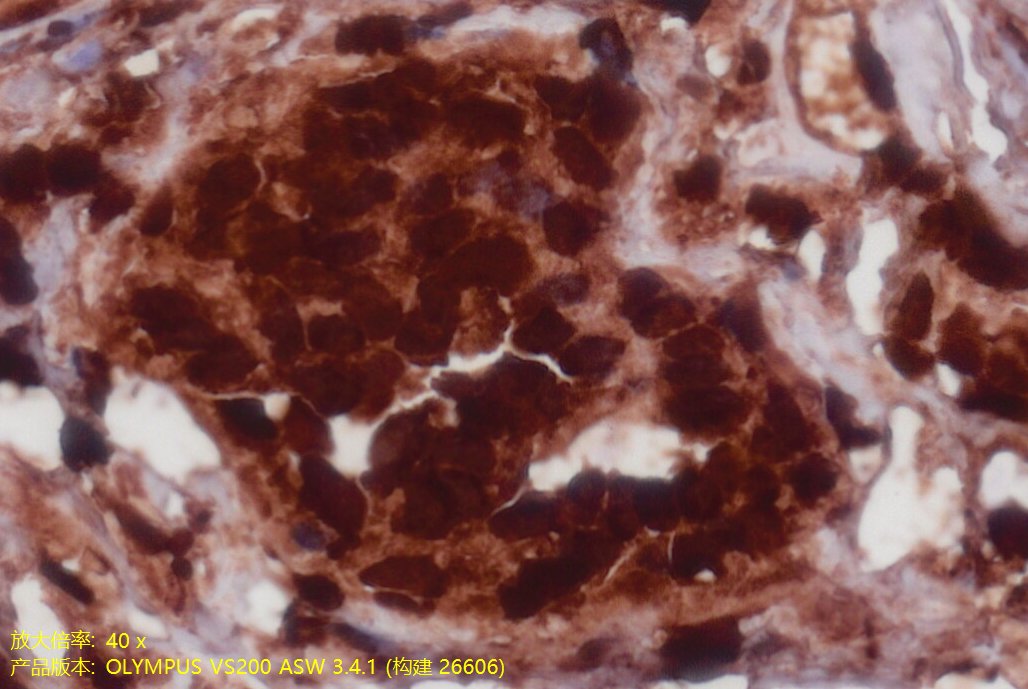

Supplement: Supplementary file 6 — Source data Fig. 1 [file 44318_2024_359_MOESM6_ESM.zip › Figure 1/Fig 1E/2-P.jpg]

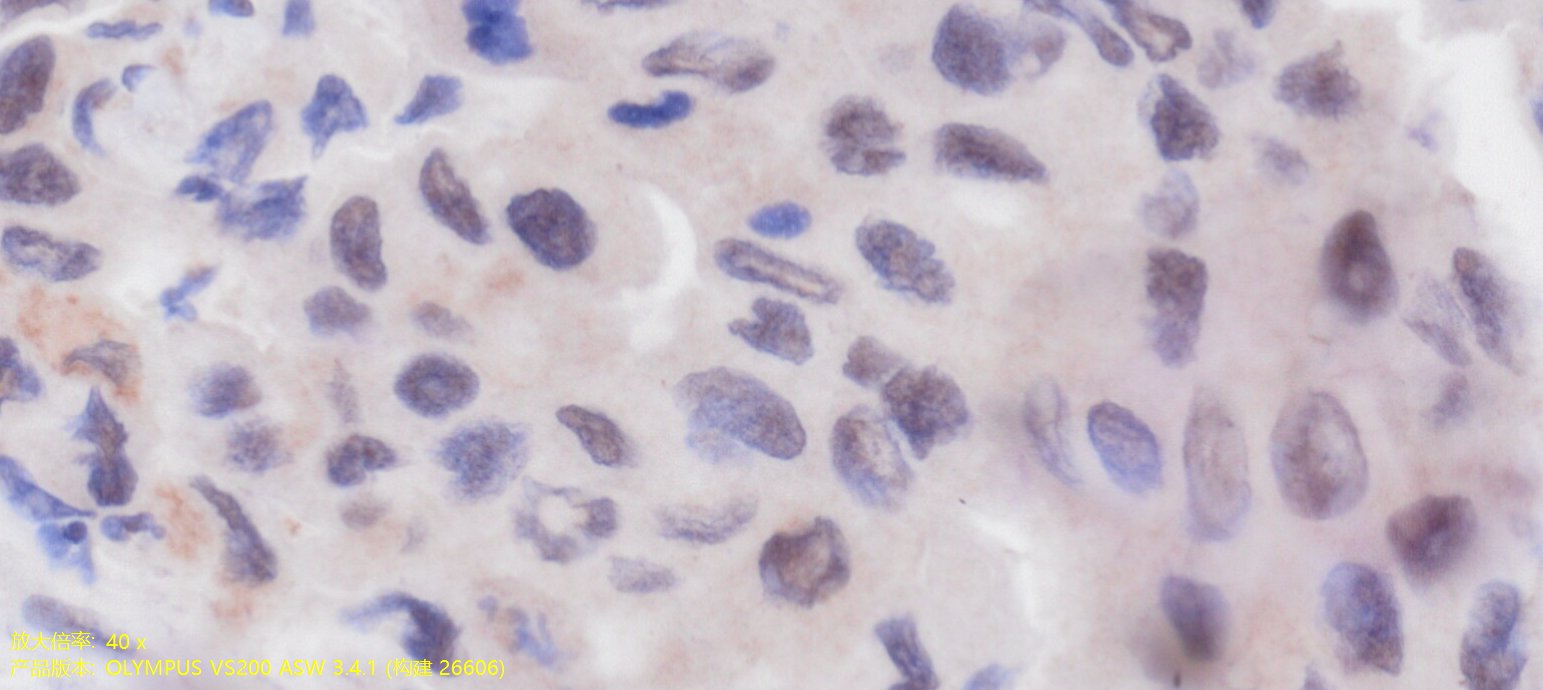

Supplement: Supplementary file 6 — Source data Fig. 1 [file 44318_2024_359_MOESM6_ESM.zip › Figure 1/Fig 1E/3-C.jpg]

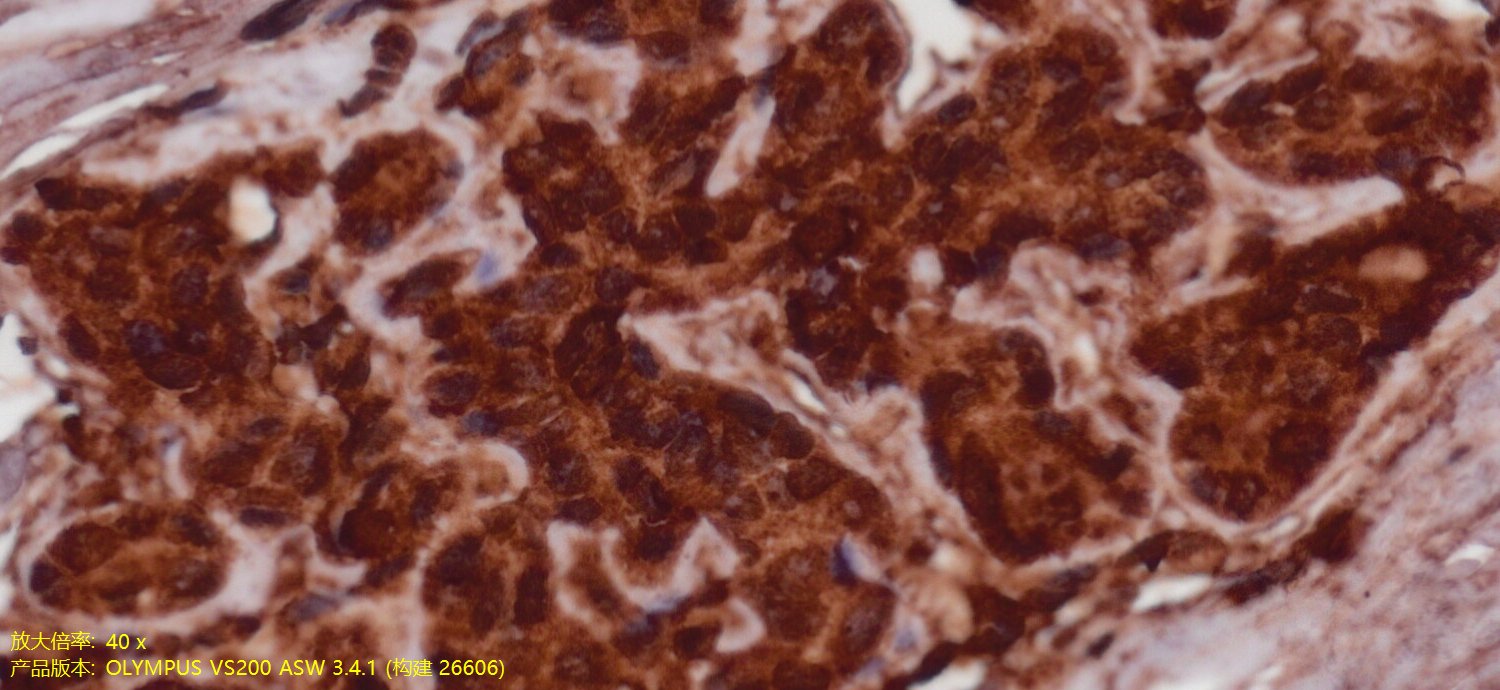

Supplement: Supplementary file 6 — Source data Fig. 1 [file 44318_2024_359_MOESM6_ESM.zip › Figure 1/Fig 1E/3-P.jpg]

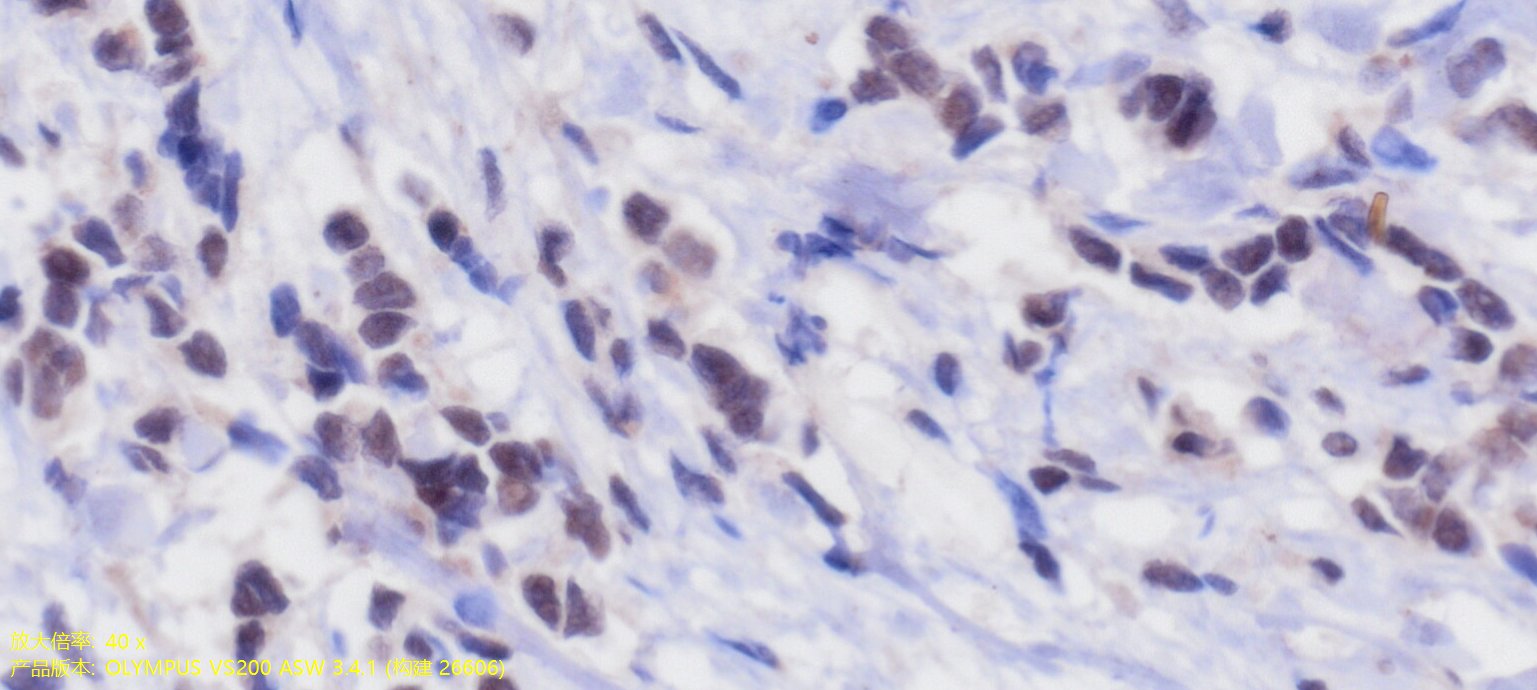

Supplement: Supplementary file 6 — Source data Fig. 1 [file 44318_2024_359_MOESM6_ESM.zip › Figure 1/Fig 1E/4-C.jpg]

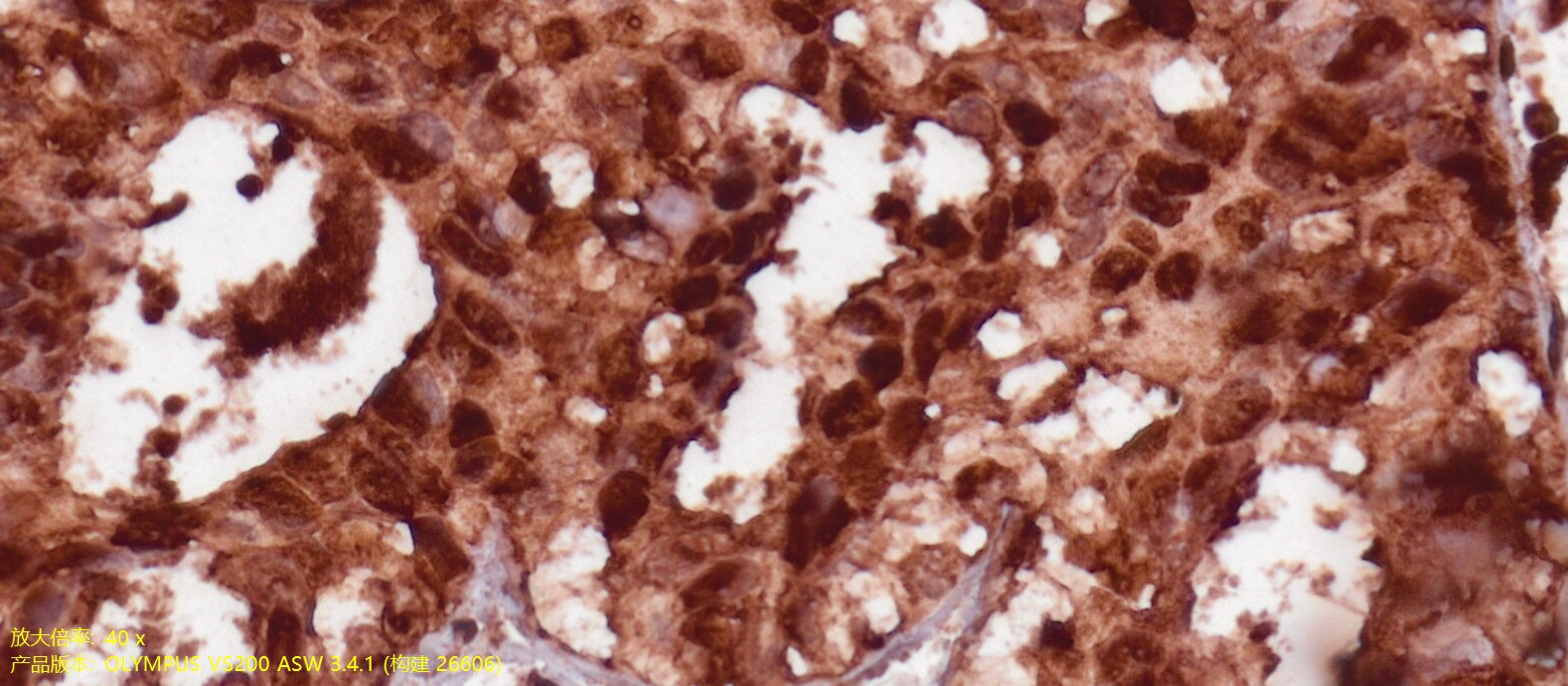

Supplement: Supplementary file 6 — Source data Fig. 1 [file 44318_2024_359_MOESM6_ESM.zip › Figure 1/Fig 1E/4-P.jpg]

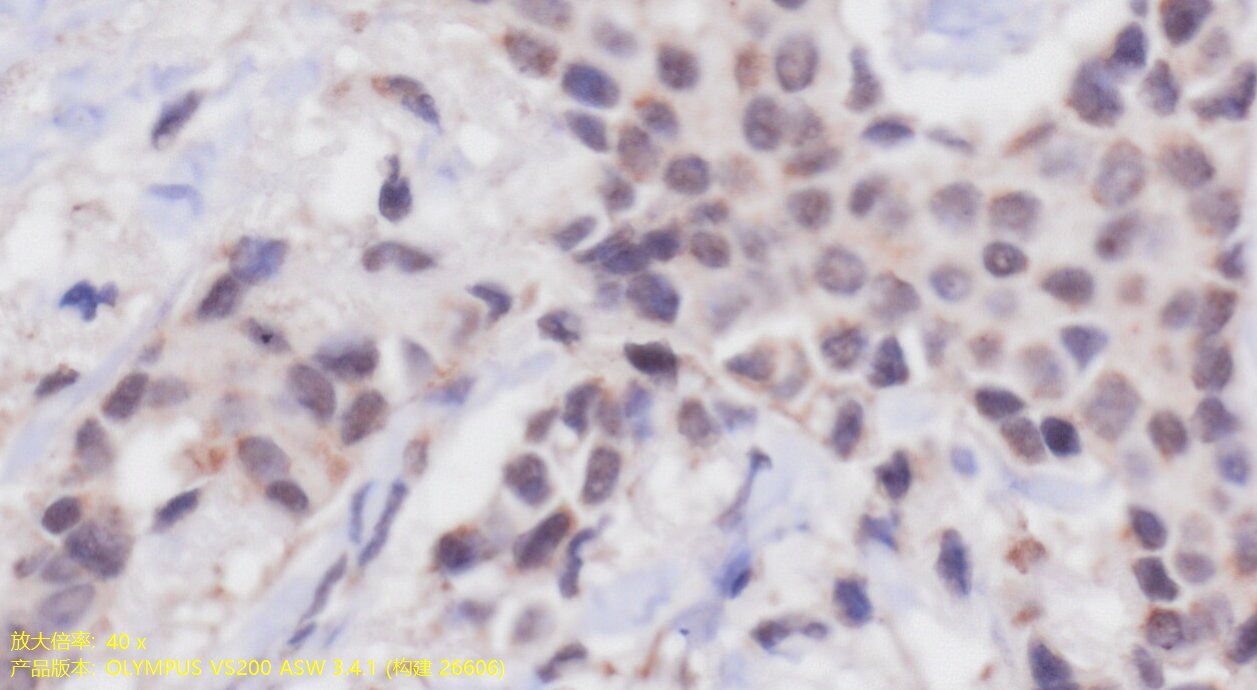

Supplement: Supplementary file 6 — Source data Fig. 1 [file 44318_2024_359_MOESM6_ESM.zip › Figure 1/Fig 1E/5-C.jpg]

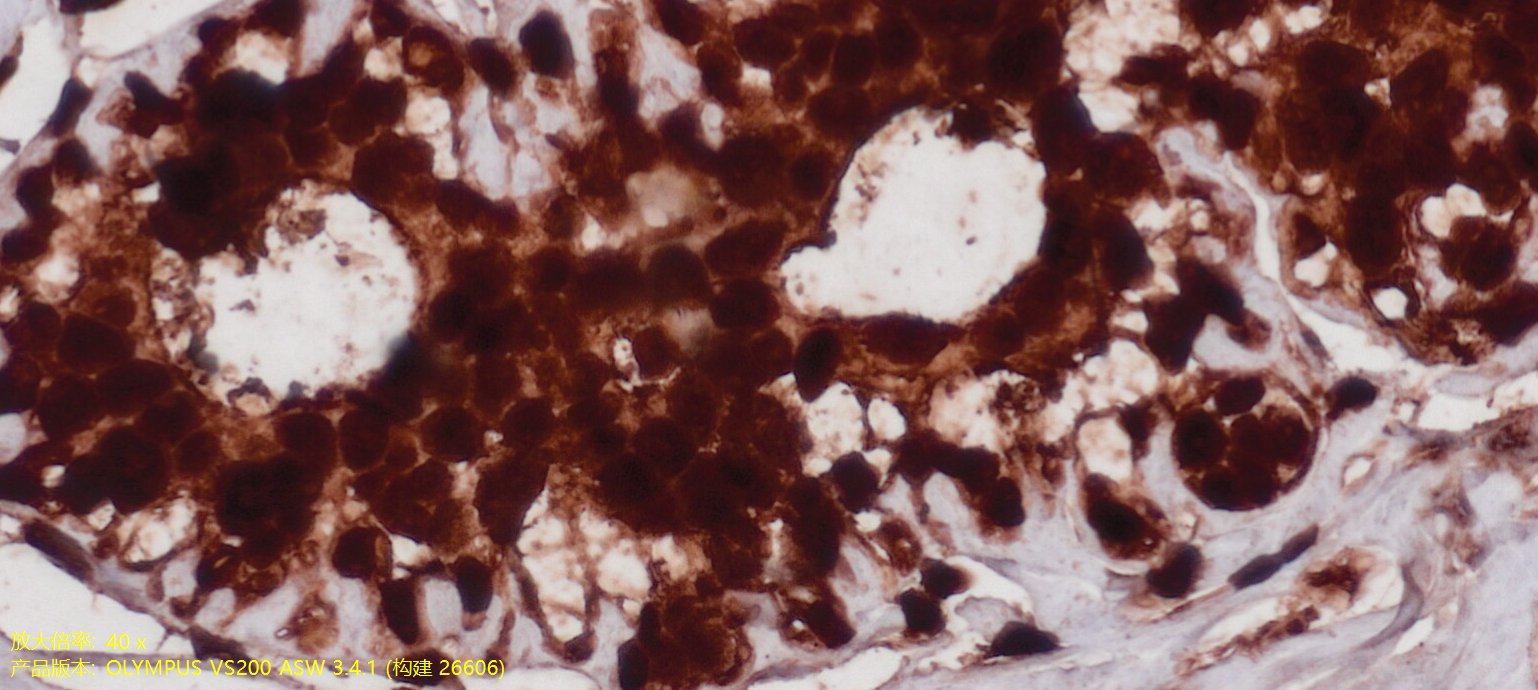

Supplement: Supplementary file 6 — Source data Fig. 1 [file 44318_2024_359_MOESM6_ESM.zip › Figure 1/Fig 1E/5-P.jpg]

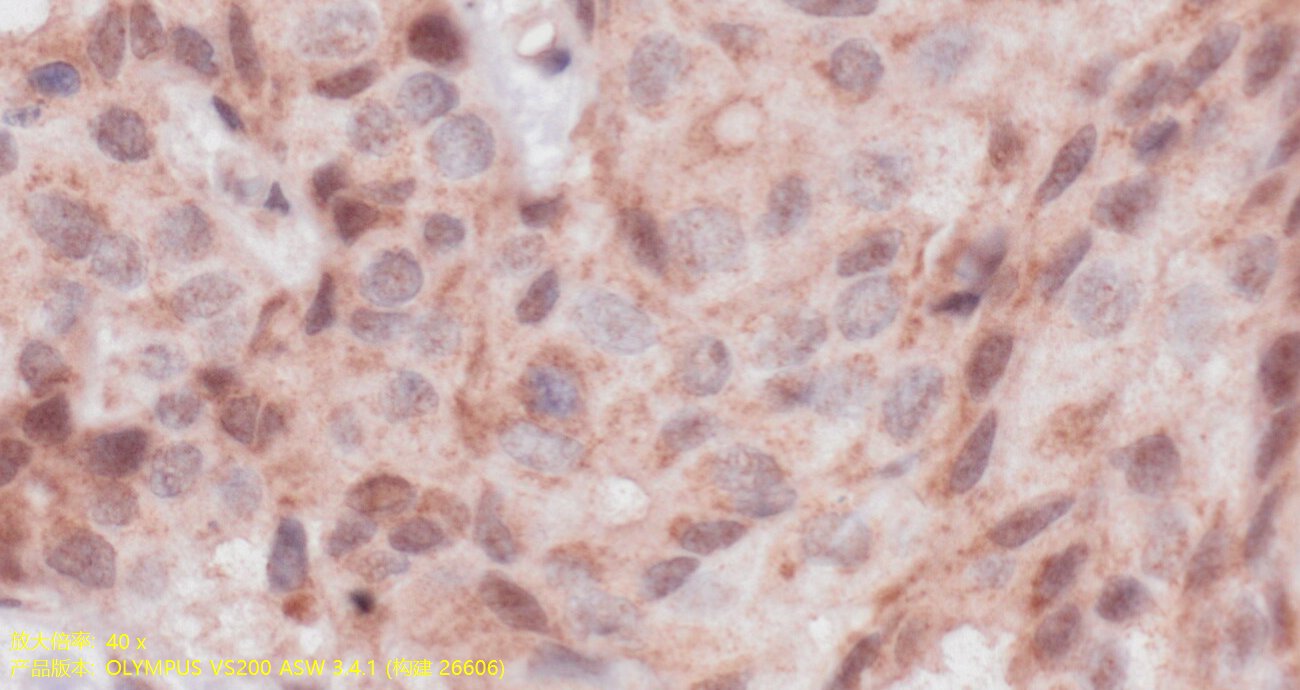

Supplement: Supplementary file 6 — Source data Fig. 1 [file 44318_2024_359_MOESM6_ESM.zip › Figure 1/Fig 1E/6-C.jpg]

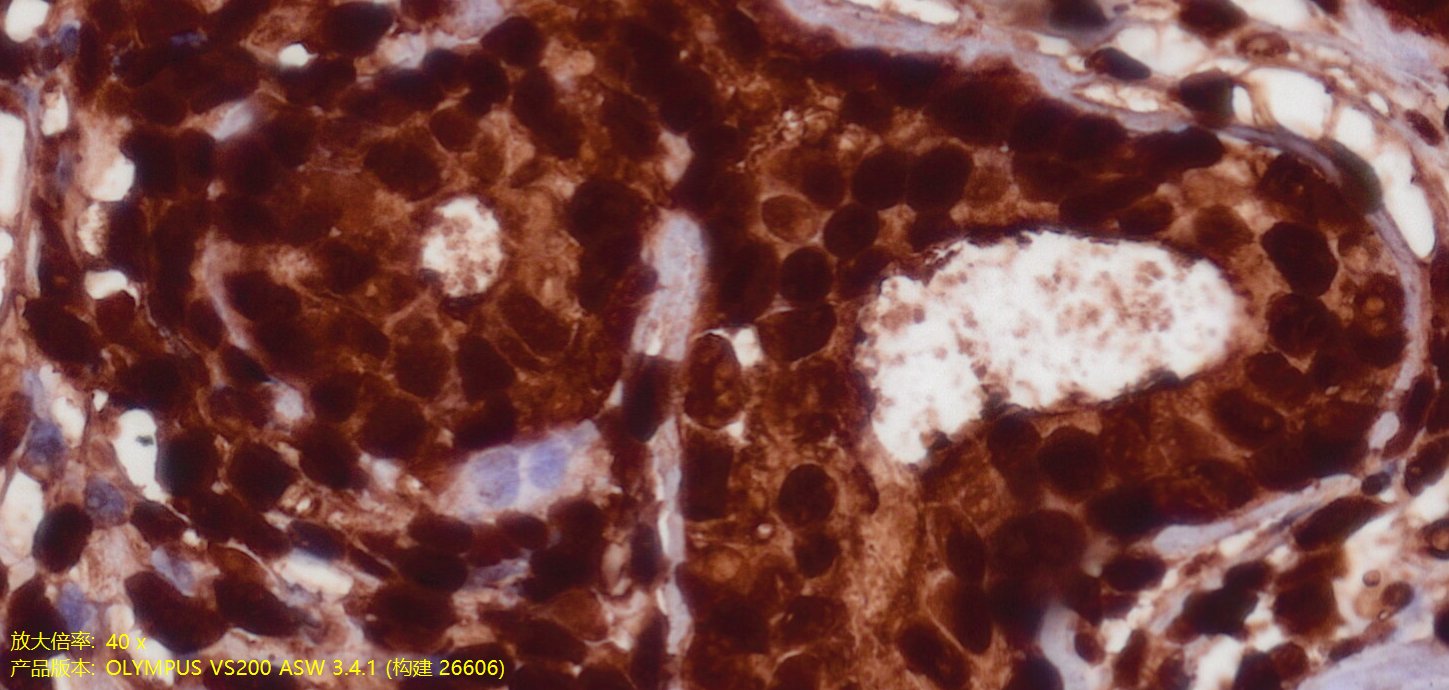

Supplement: Supplementary file 6 — Source data Fig. 1 [file 44318_2024_359_MOESM6_ESM.zip › Figure 1/Fig 1E/6-P.jpg]

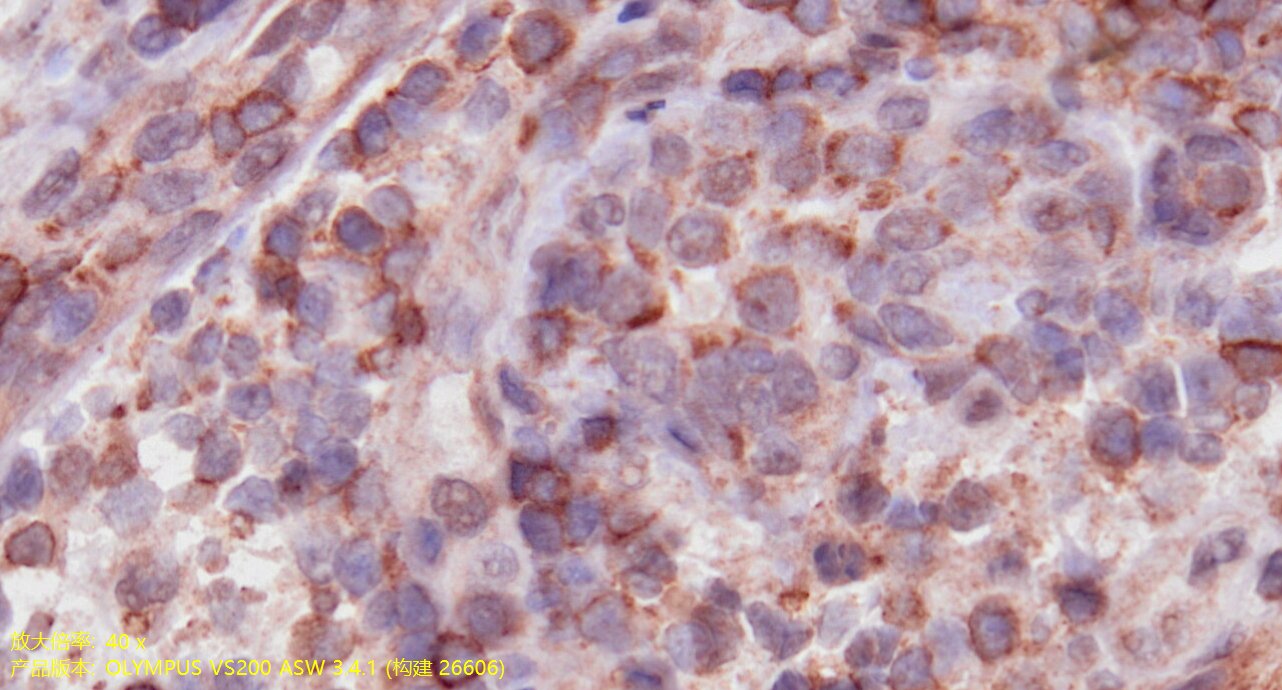

Supplement: Supplementary file 6 — Source data Fig. 1 [file 44318_2024_359_MOESM6_ESM.zip › Figure 1/Fig 1E/7-C.jpg]

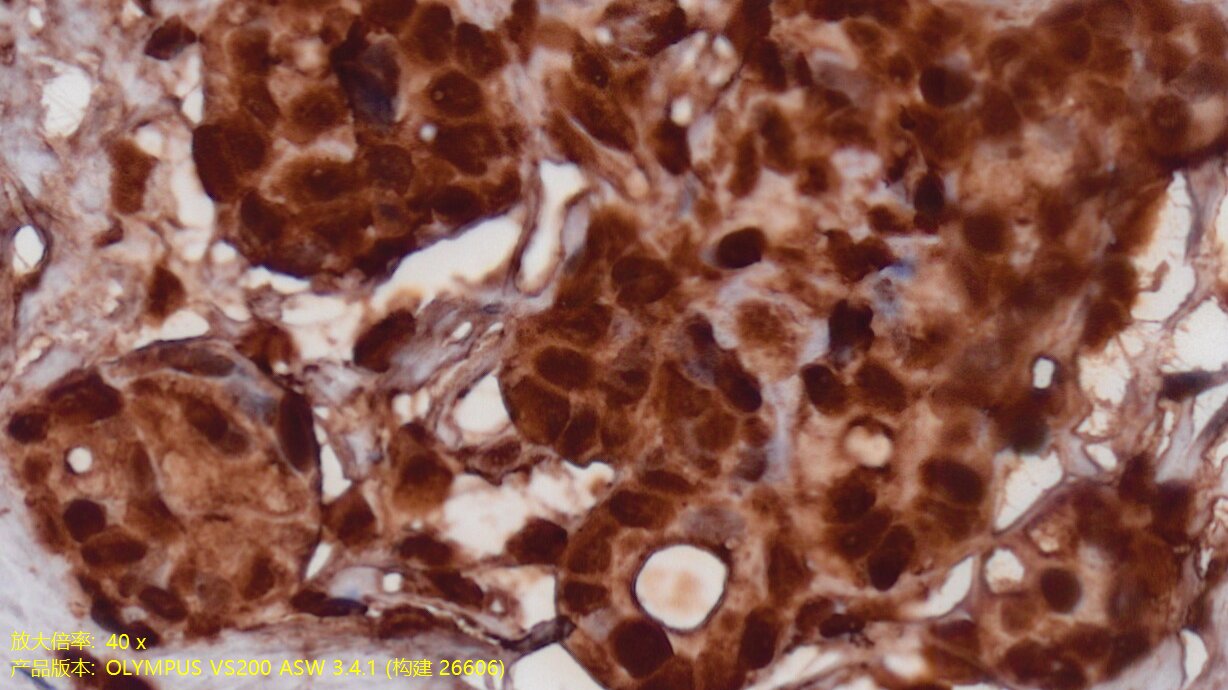

Supplement: Supplementary file 6 — Source data Fig. 1 [file 44318_2024_359_MOESM6_ESM.zip › Figure 1/Fig 1E/7-P.jpg]

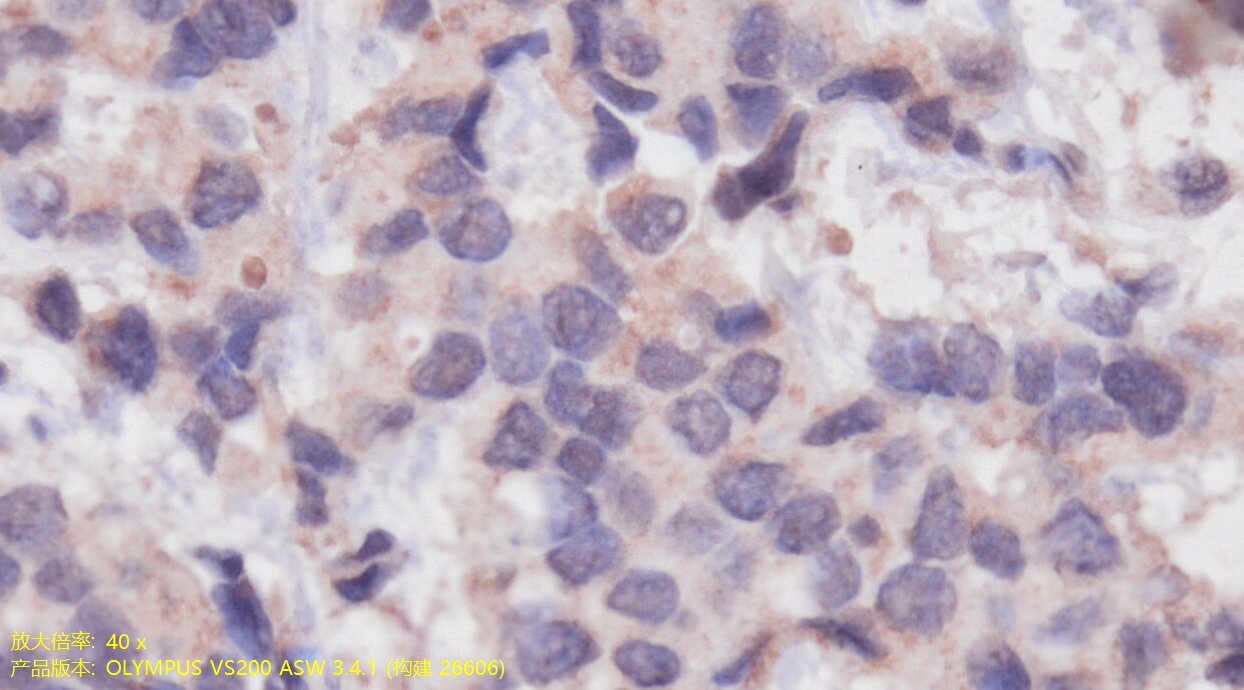

Supplement: Supplementary file 6 — Source data Fig. 1 [file 44318_2024_359_MOESM6_ESM.zip › Figure 1/Fig 1E/8-C.jpg]

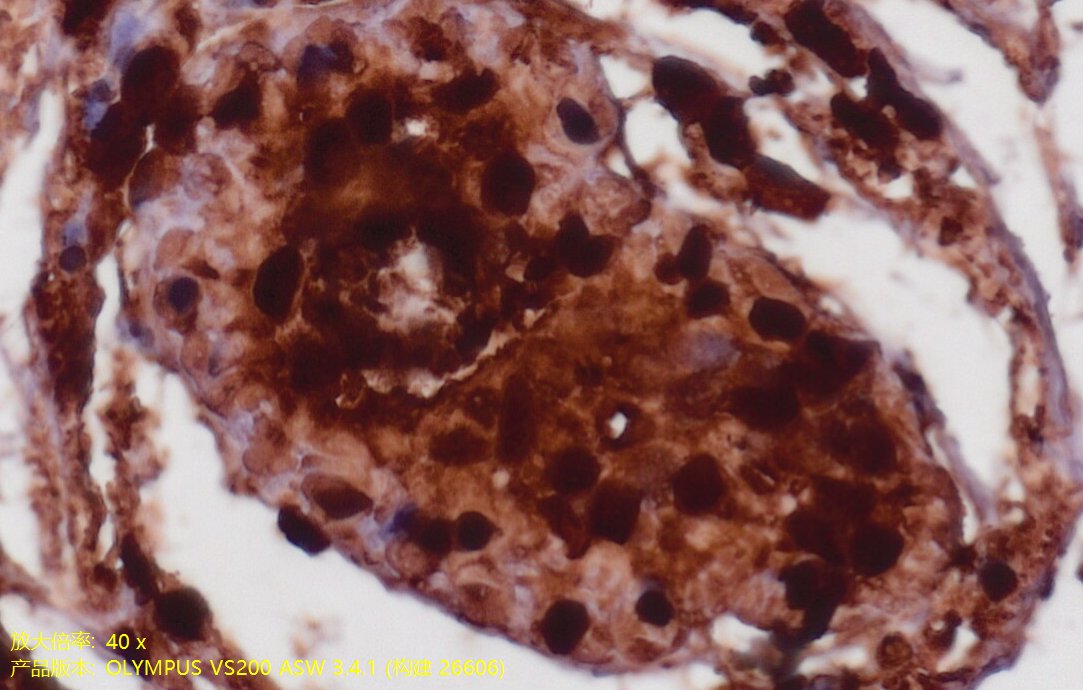

Supplement: Supplementary file 6 — Source data Fig. 1 [file 44318_2024_359_MOESM6_ESM.zip › Figure 1/Fig 1E/8-P.jpg]

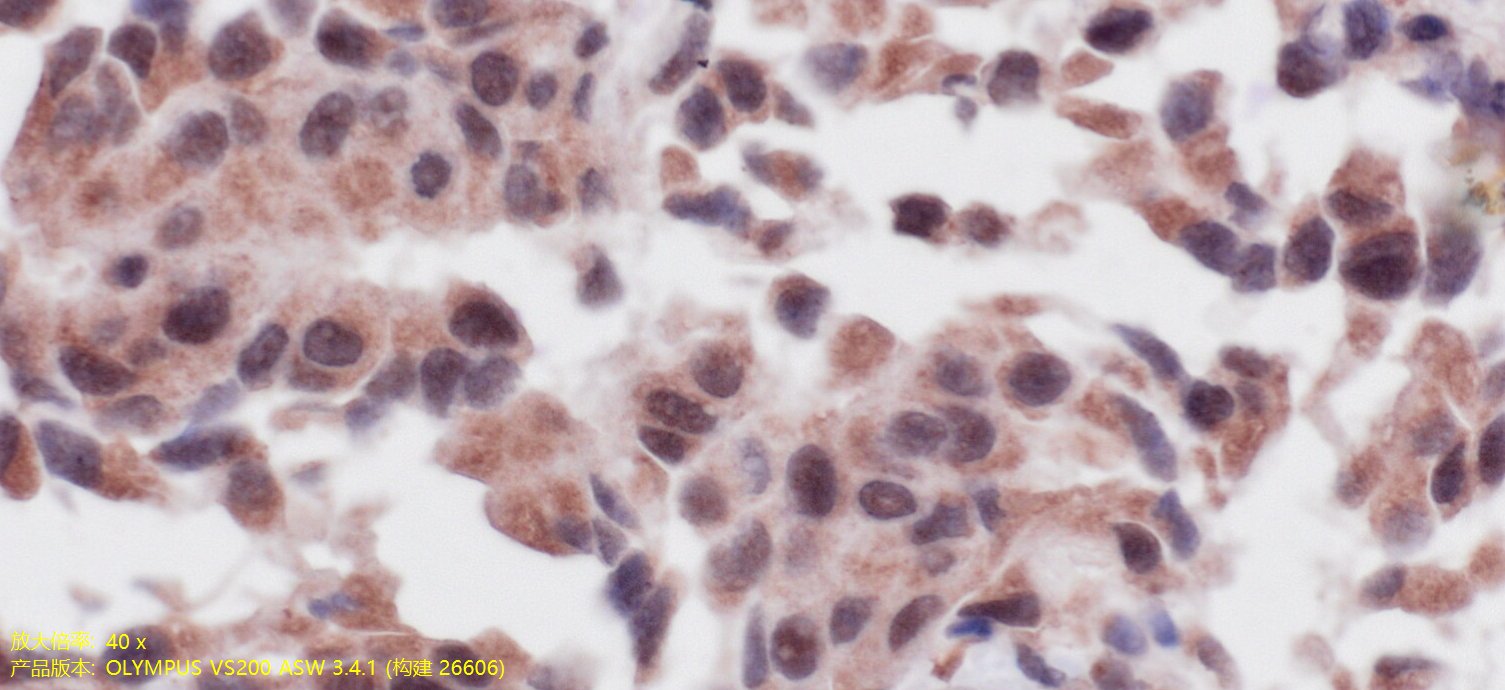

Supplement: Supplementary file 6 — Source data Fig. 1 [file 44318_2024_359_MOESM6_ESM.zip › Figure 1/Fig 1E/9-C.jpg]

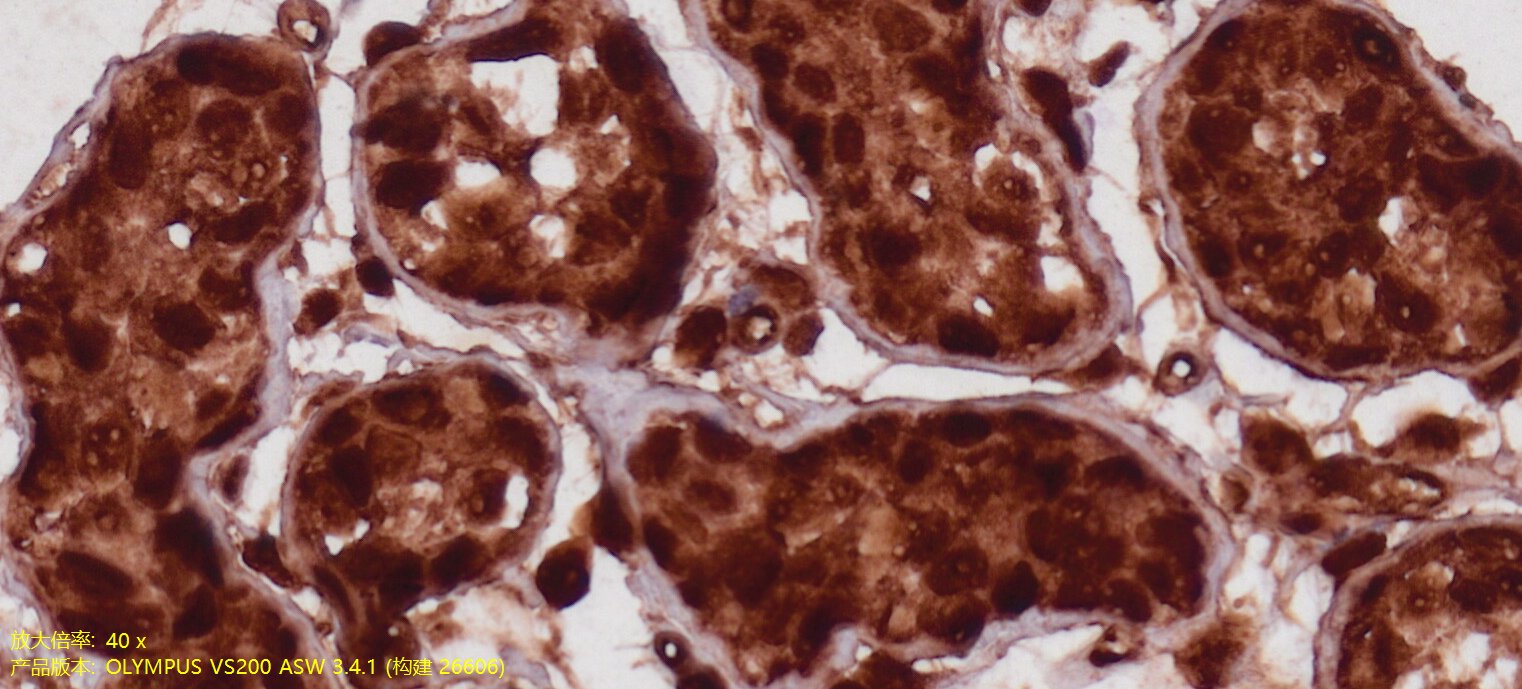

Supplement: Supplementary file 6 — Source data Fig. 1 [file 44318_2024_359_MOESM6_ESM.zip › Figure 1/Fig 1E/9-P.jpg]

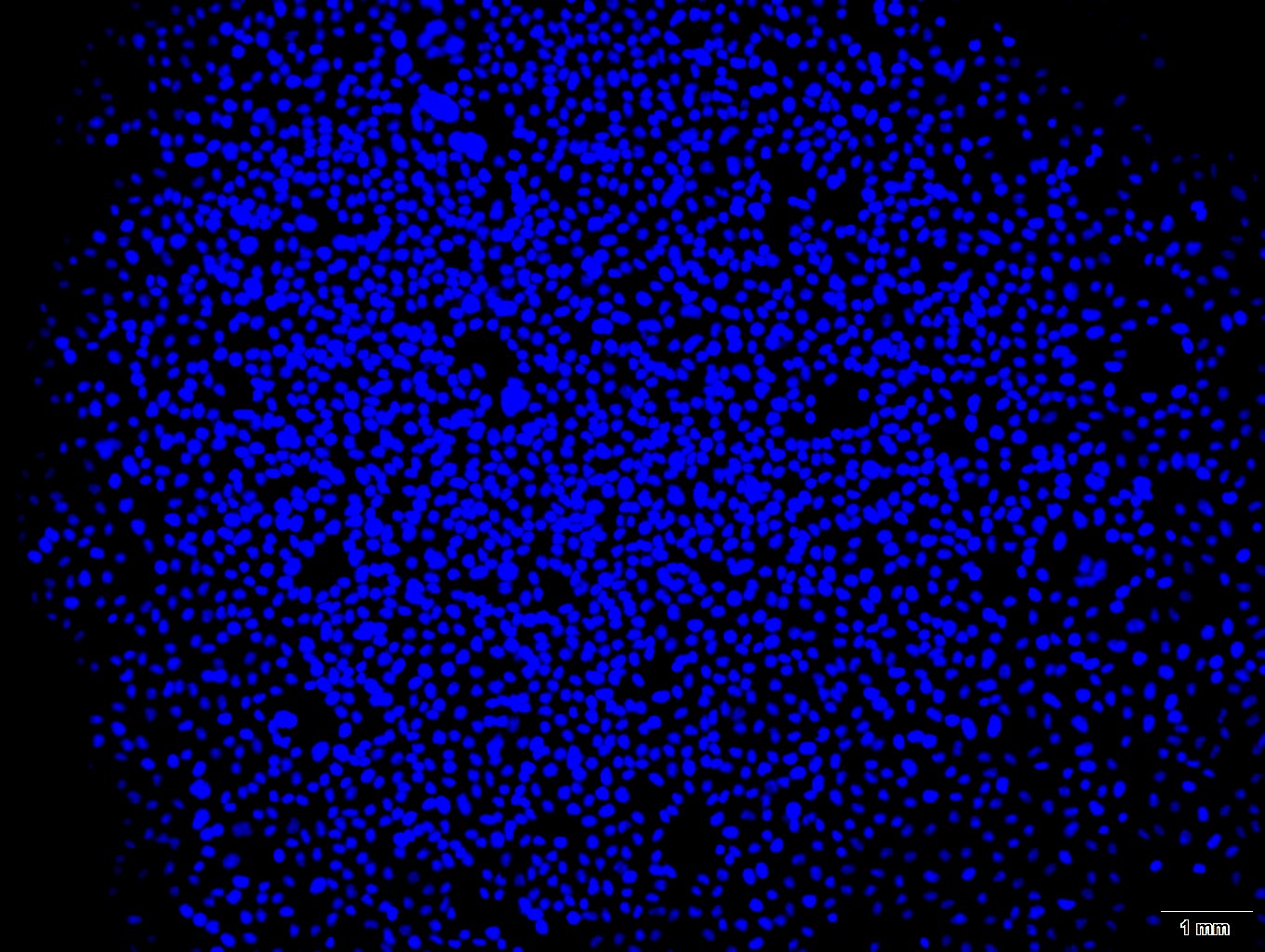

Supplement: Supplementary file 6 — Source data Fig. 1 [file 44318_2024_359_MOESM6_ESM.zip › Figure 1/Fig 1F and 1G/Fig 1F/si-Ctrl/siCtrl-Hoechst.jpg]

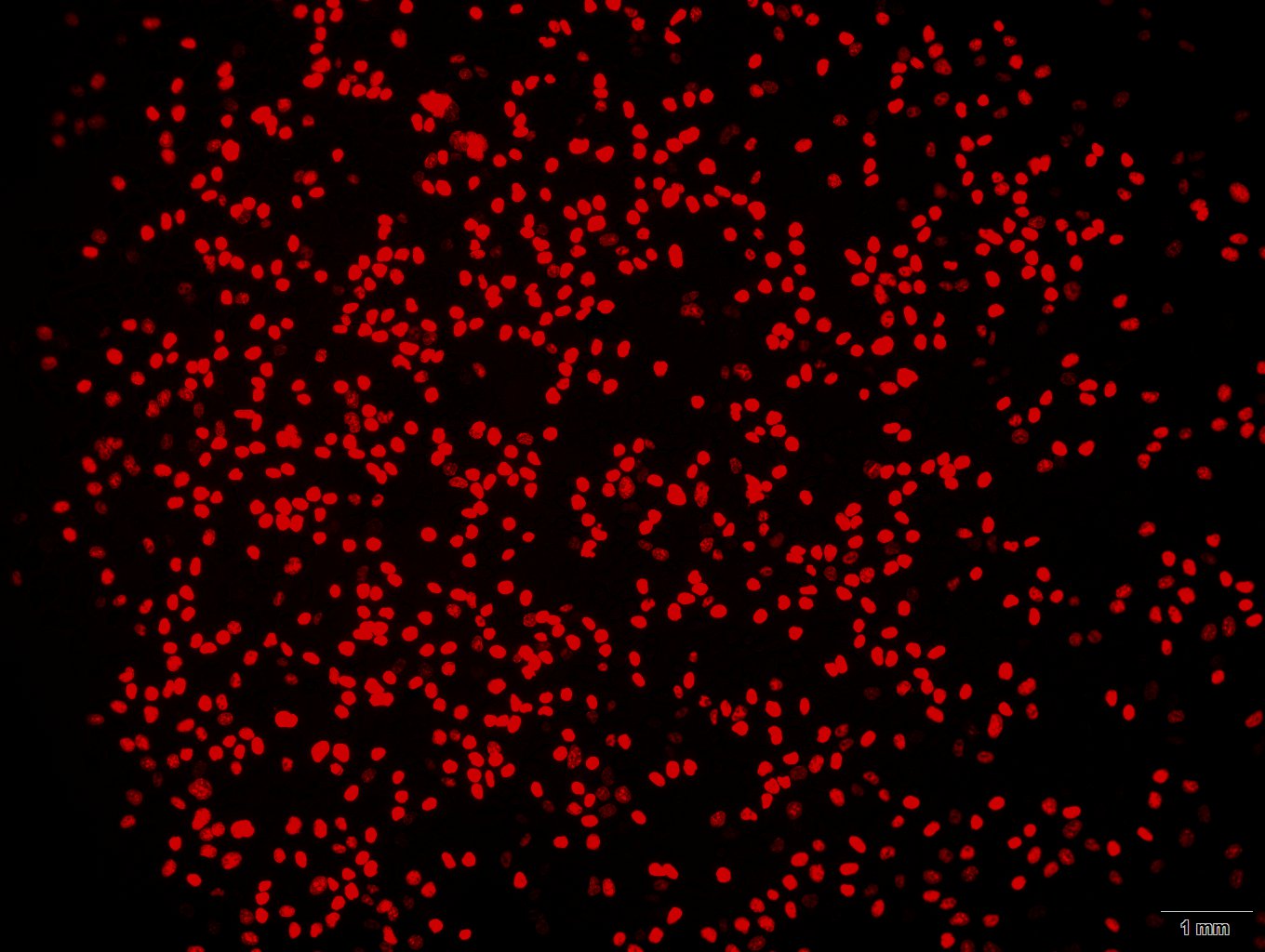

Supplement: Supplementary file 6 — Source data Fig. 1 [file 44318_2024_359_MOESM6_ESM.zip › Figure 1/Fig 1F and 1G/Fig 1F/si-Ctrl/siCtrl-edu.jpg]

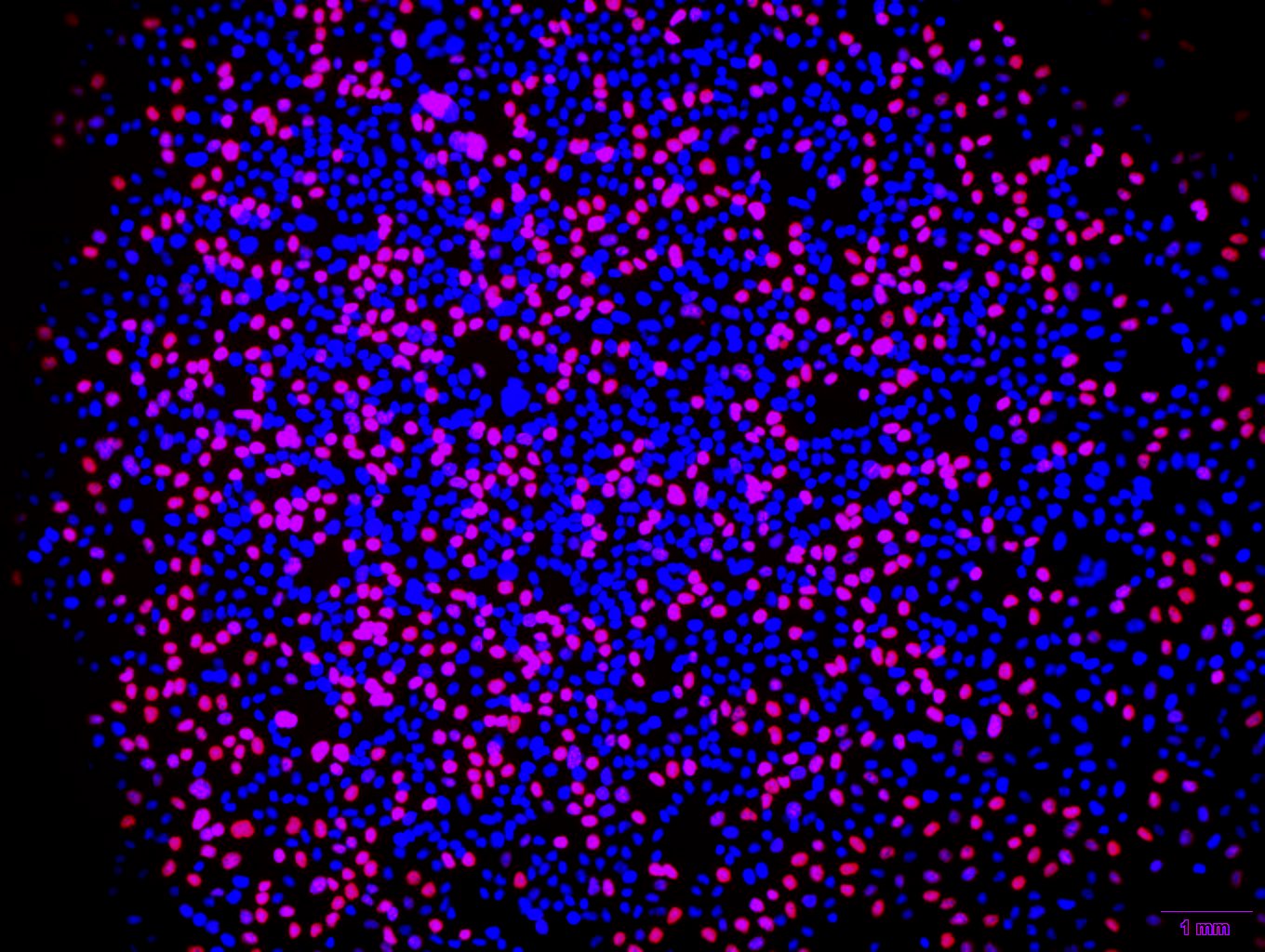

Supplement: Supplementary file 6 — Source data Fig. 1 [file 44318_2024_359_MOESM6_ESM.zip › Figure 1/Fig 1F and 1G/Fig 1F/si-Ctrl/siCtrl-merge.jpg]

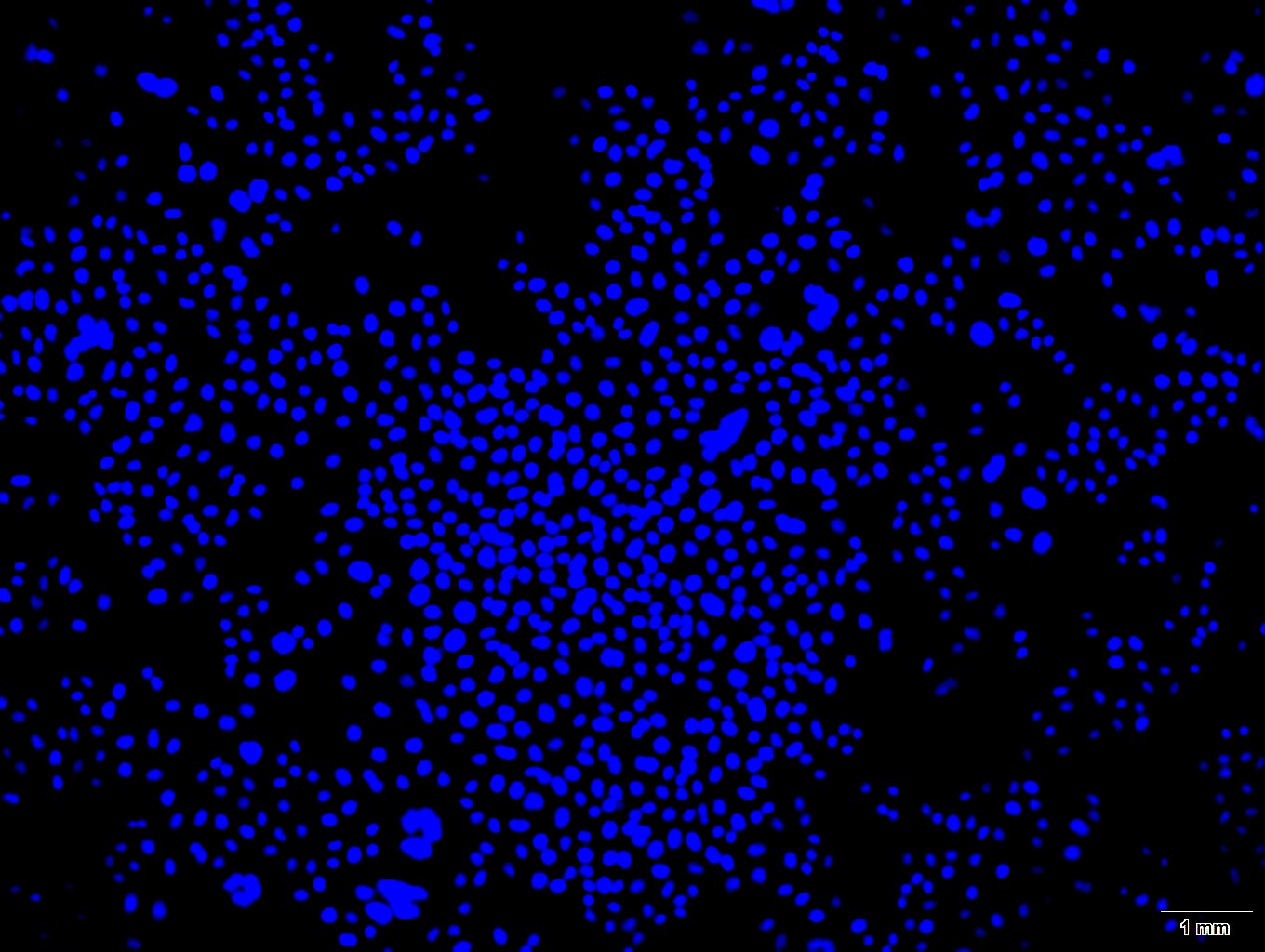

Supplement: Supplementary file 6 — Source data Fig. 1 [file 44318_2024_359_MOESM6_ESM.zip › Figure 1/Fig 1F and 1G/Fig 1F/sihSPAR-1/sihSPAR-1-Hoechst.jpg]

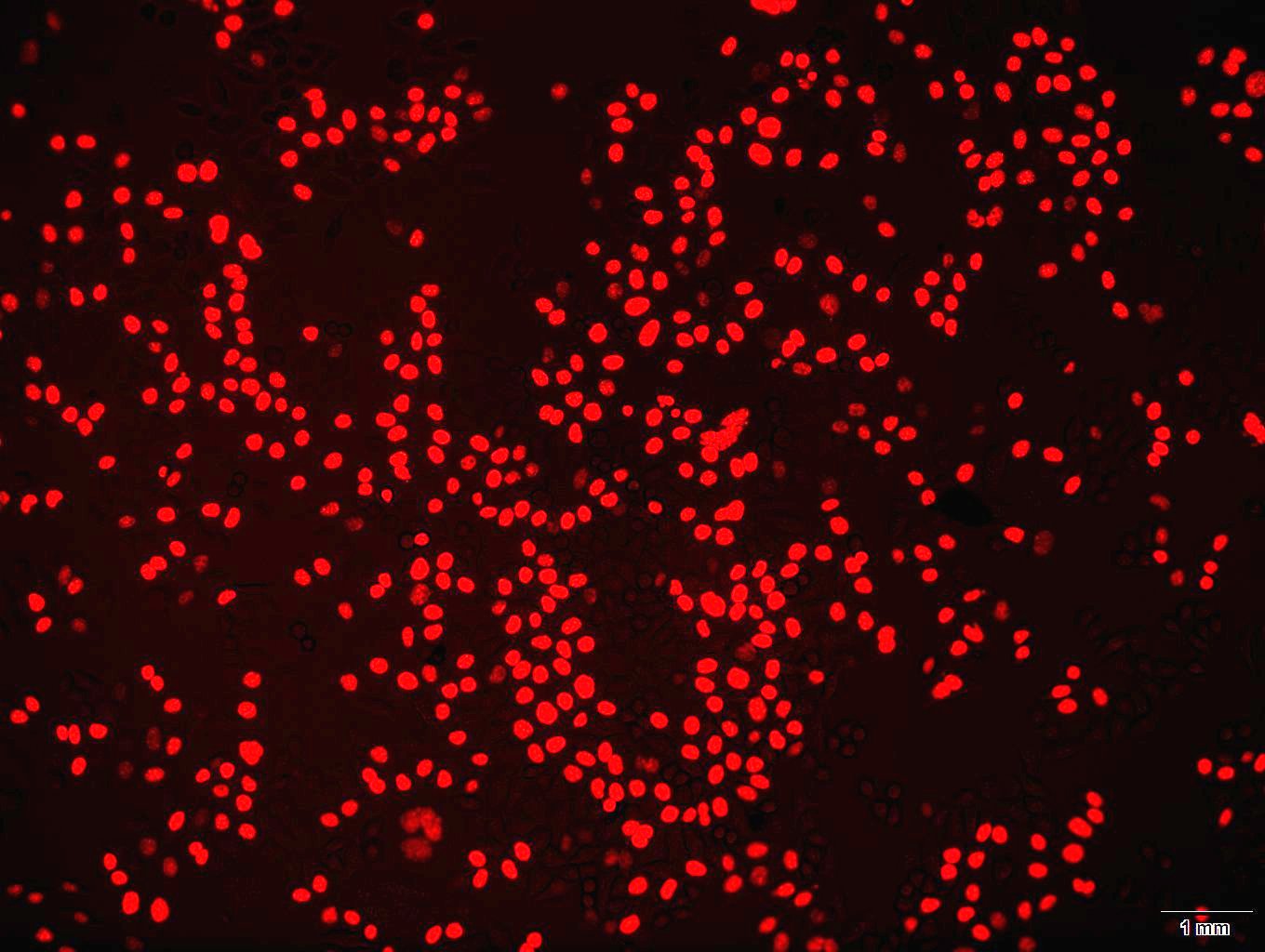

Supplement: Supplementary file 6 — Source data Fig. 1 [file 44318_2024_359_MOESM6_ESM.zip › Figure 1/Fig 1F and 1G/Fig 1F/sihSPAR-1/sihSPAR-1-edu.jpg]

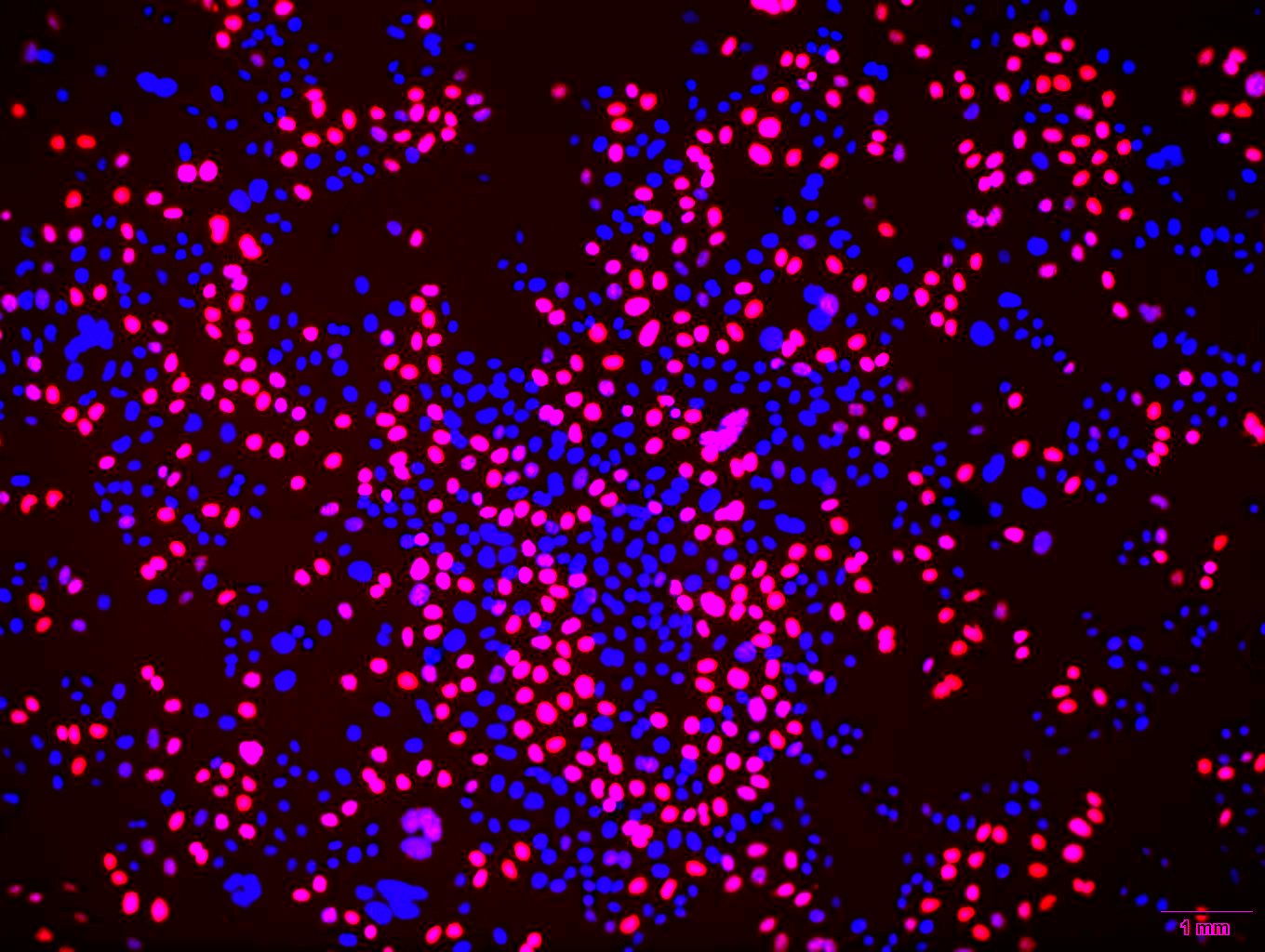

Supplement: Supplementary file 6 — Source data Fig. 1 [file 44318_2024_359_MOESM6_ESM.zip › Figure 1/Fig 1F and 1G/Fig 1F/sihSPAR-1/sihSPAR-1-merge.jpg]

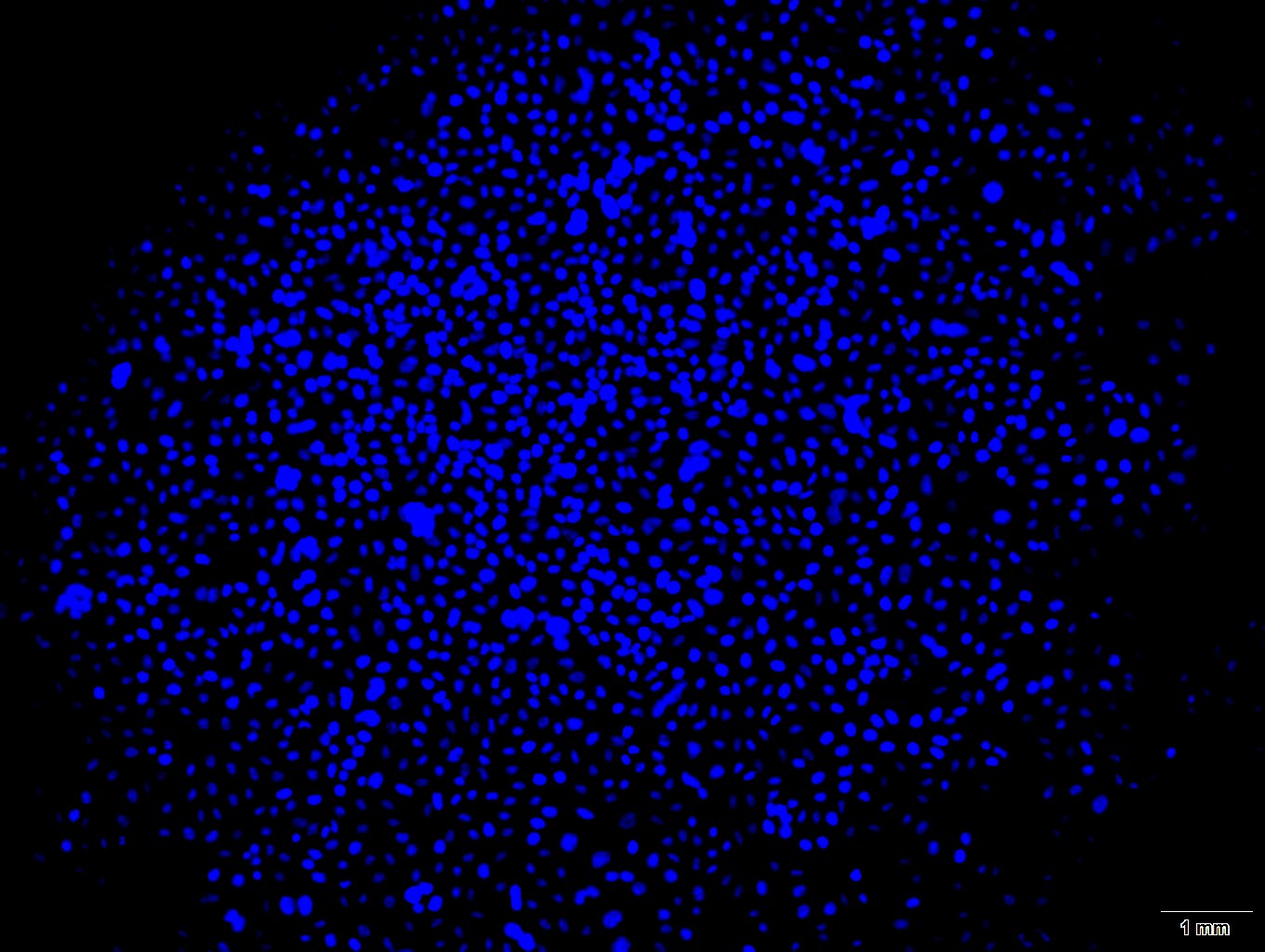

Supplement: Supplementary file 6 — Source data Fig. 1 [file 44318_2024_359_MOESM6_ESM.zip › Figure 1/Fig 1F and 1G/Fig 1F/sihSPAR-2/sihSPAR-2-Hoechst.jpg]

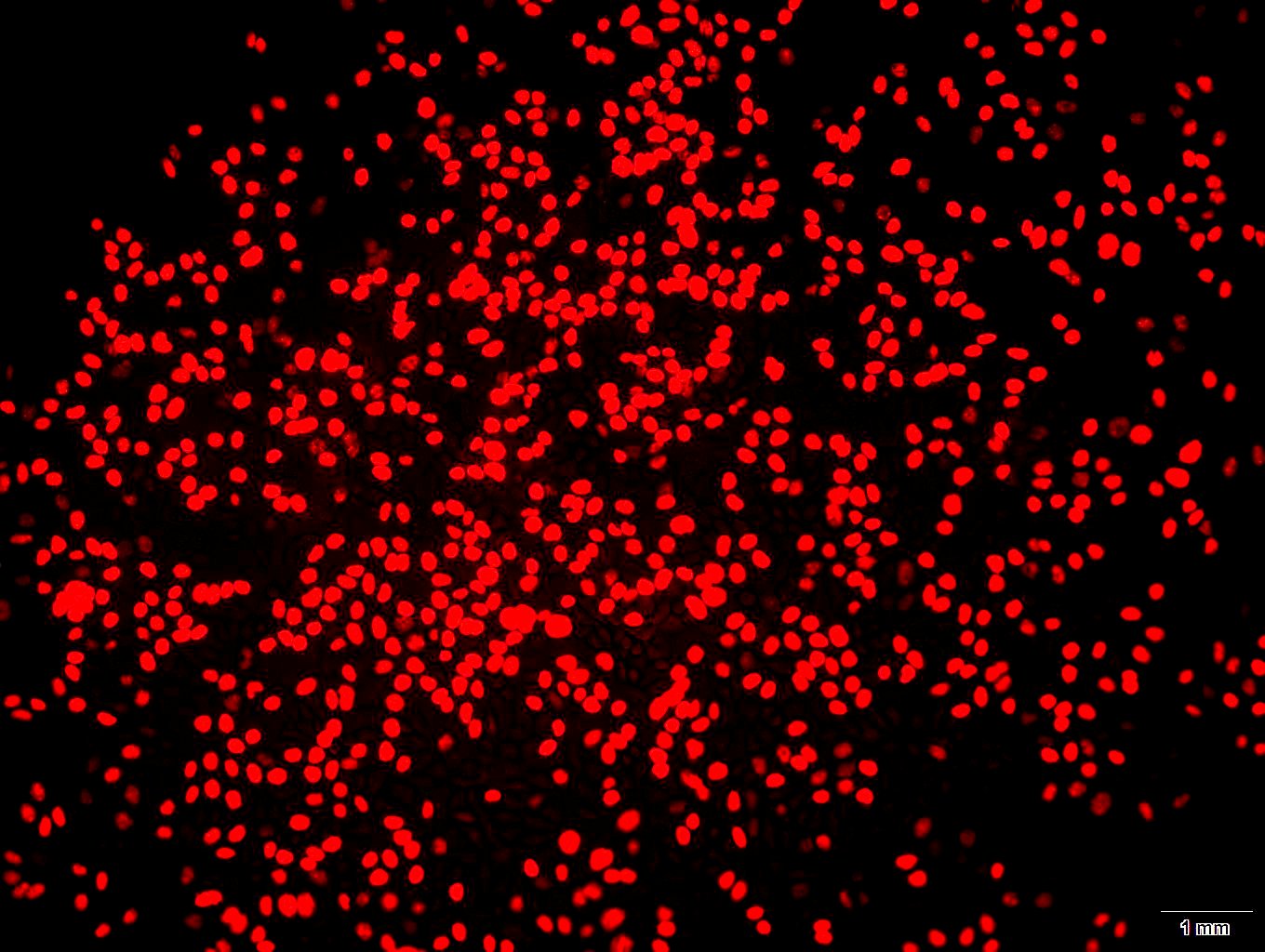

Supplement: Supplementary file 6 — Source data Fig. 1 [file 44318_2024_359_MOESM6_ESM.zip › Figure 1/Fig 1F and 1G/Fig 1F/sihSPAR-2/sihSPAR-2-edu.jpg]

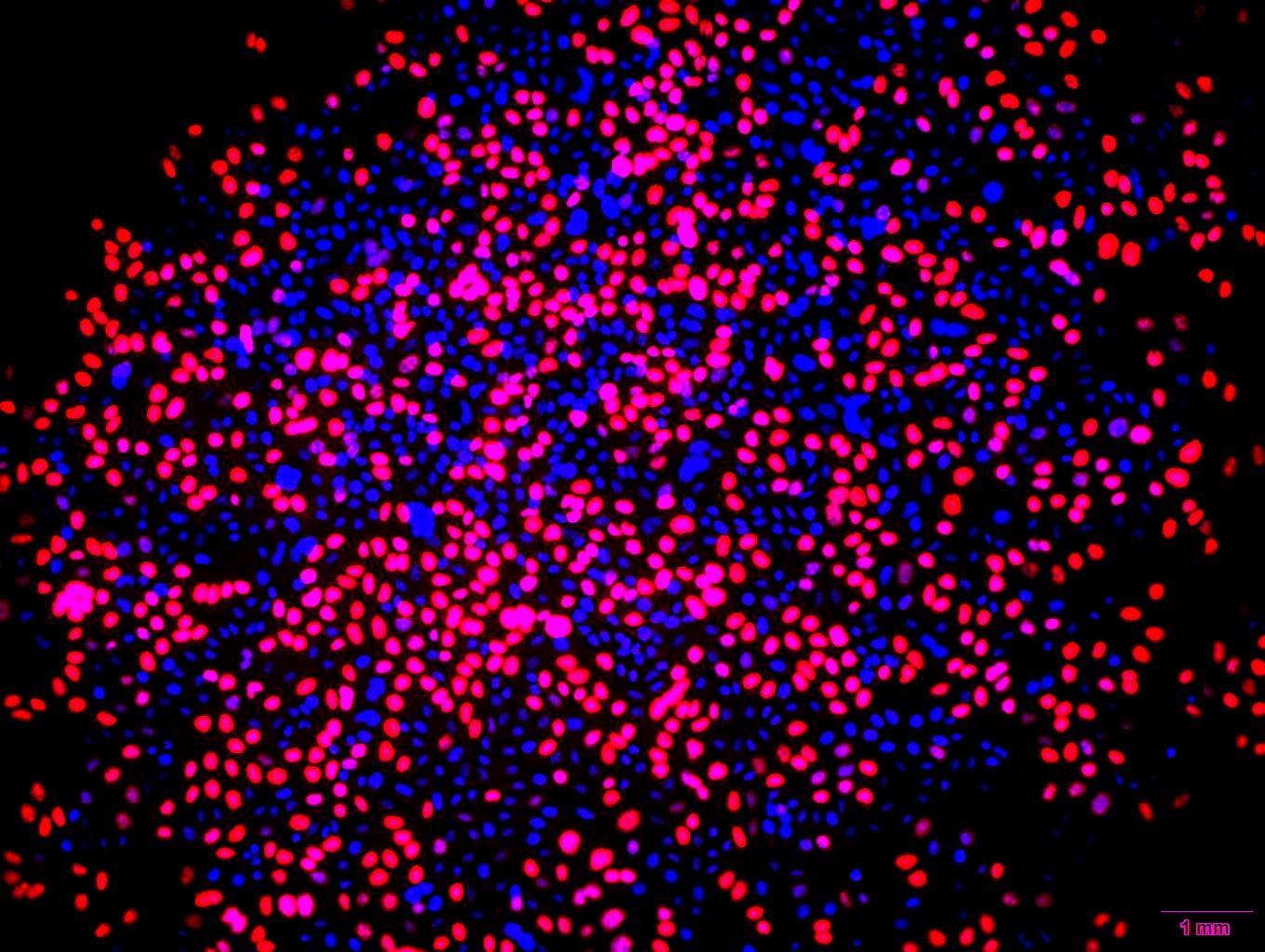

Supplement: Supplementary file 6 — Source data Fig. 1 [file 44318_2024_359_MOESM6_ESM.zip › Figure 1/Fig 1F and 1G/Fig 1F/sihSPAR-2/sihSPAR-2-merge.jpg]

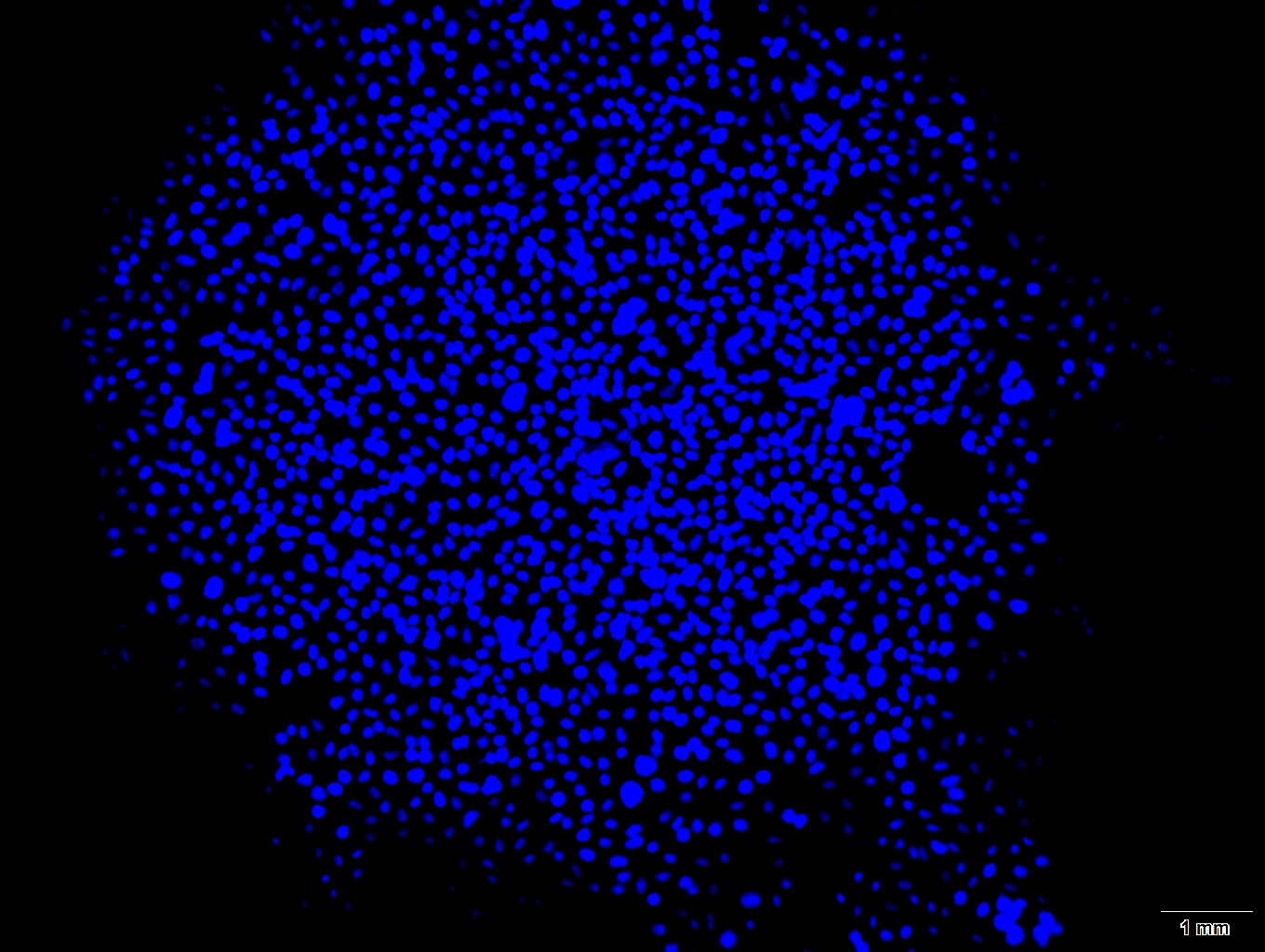

Supplement: Supplementary file 6 — Source data Fig. 1 [file 44318_2024_359_MOESM6_ESM.zip › Figure 1/Fig 1F and 1G/Fig 1F/sihSPAR-3/sihSPAR-3-Hoechst.jpg]

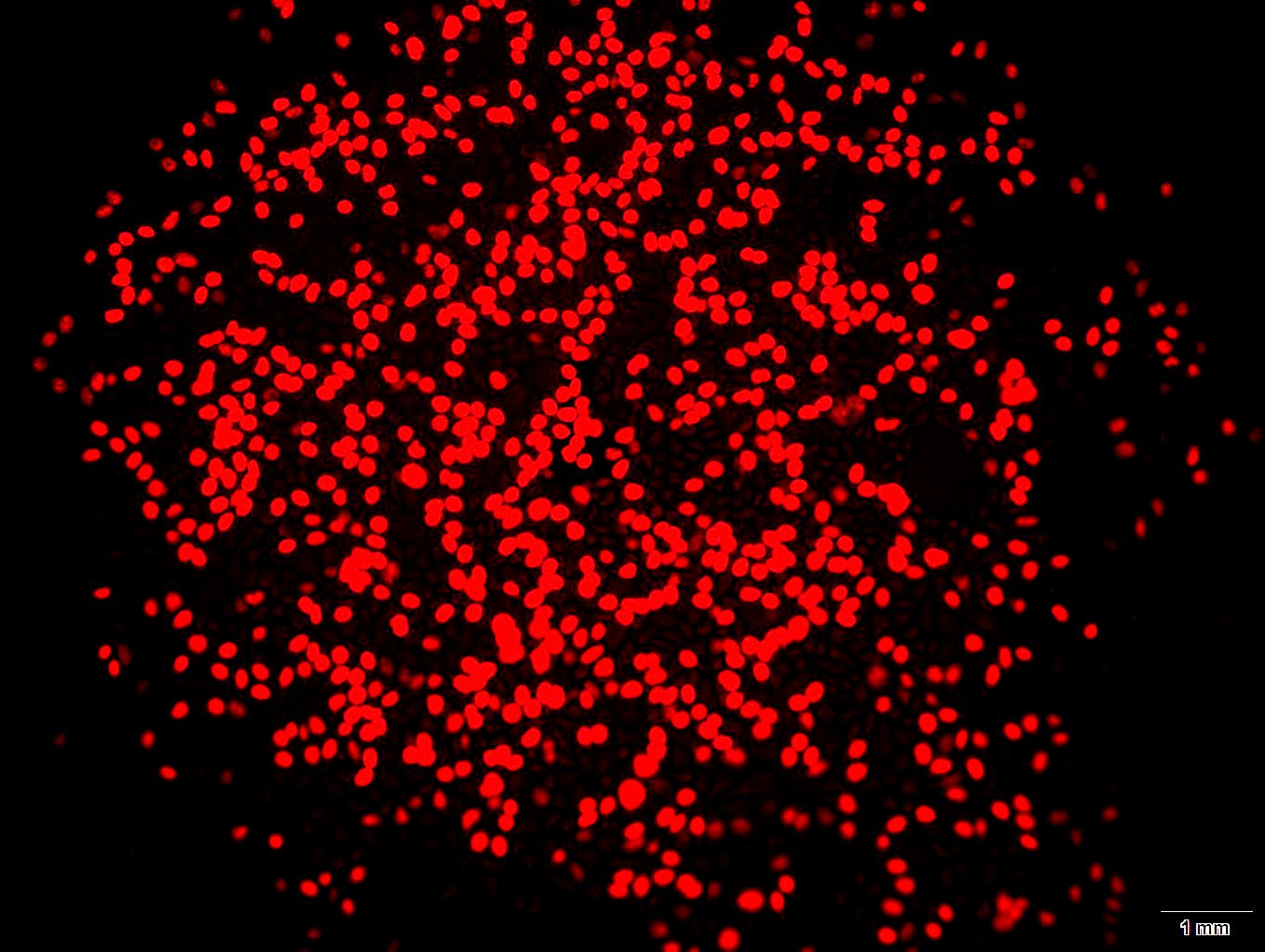

Supplement: Supplementary file 6 — Source data Fig. 1 [file 44318_2024_359_MOESM6_ESM.zip › Figure 1/Fig 1F and 1G/Fig 1F/sihSPAR-3/sihSPAR-3-edu.jpg]

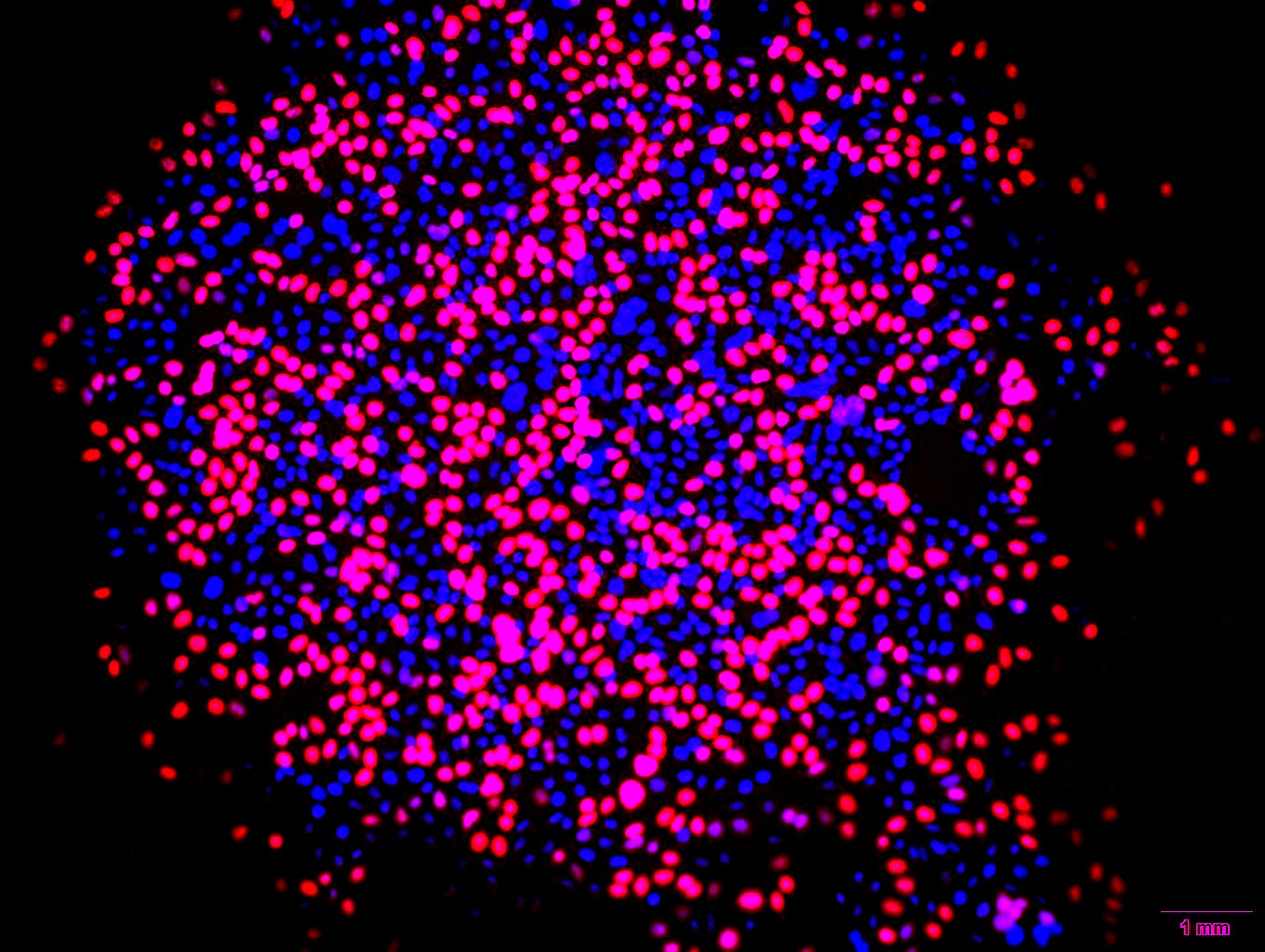

Supplement: Supplementary file 6 — Source data Fig. 1 [file 44318_2024_359_MOESM6_ESM.zip › Figure 1/Fig 1F and 1G/Fig 1F/sihSPAR-3/sihSPAR-3-merge.jpg]

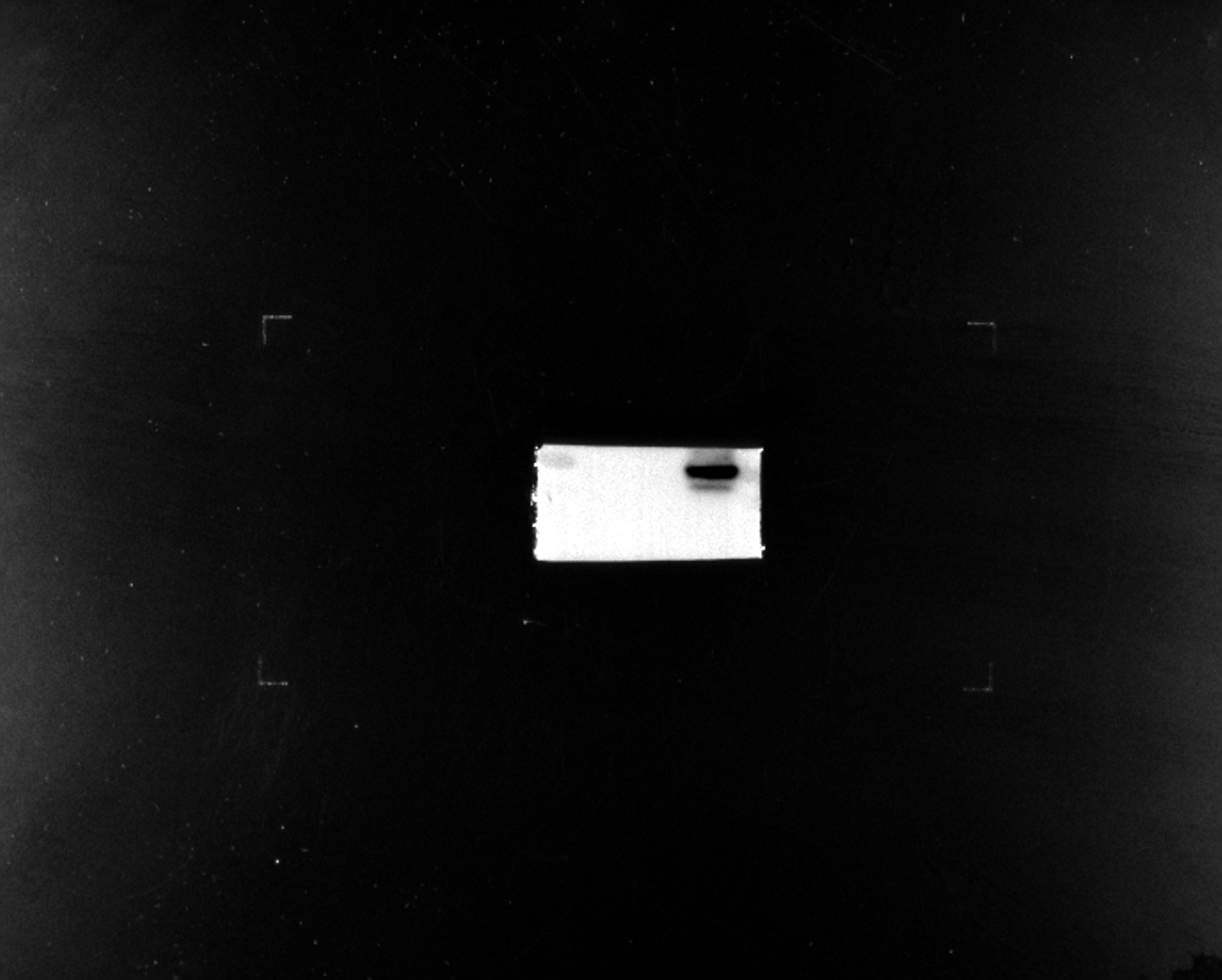

Supplement: Supplementary file 6 — Source data Fig. 1 [file 44318_2024_359_MOESM6_ESM.zip › Figure 1/Fig 1I/1-FLAG-merge.Tif]

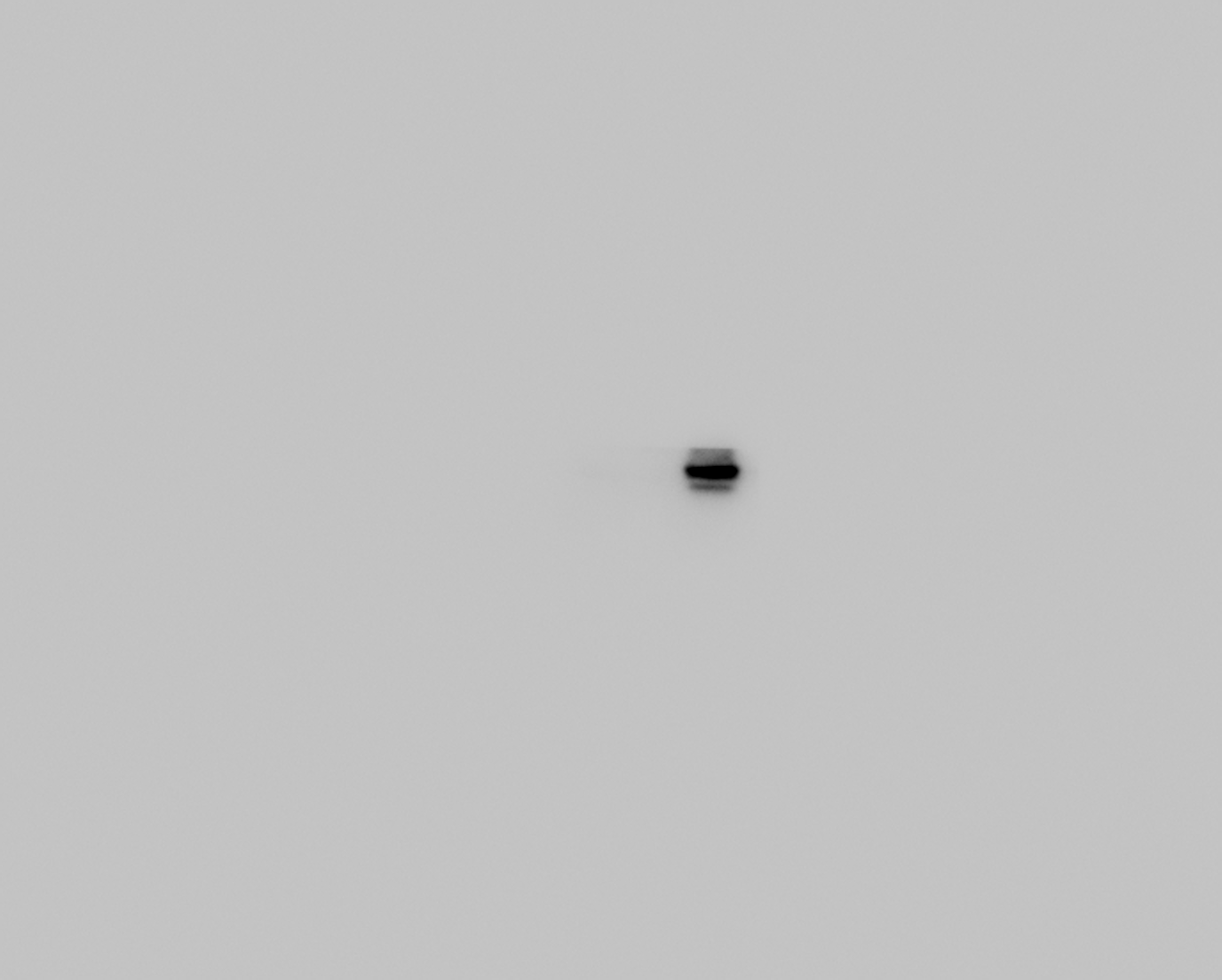

Supplement: Supplementary file 6 — Source data Fig. 1 [file 44318_2024_359_MOESM6_ESM.zip › Figure 1/Fig 1I/1-FLAG.Tif]

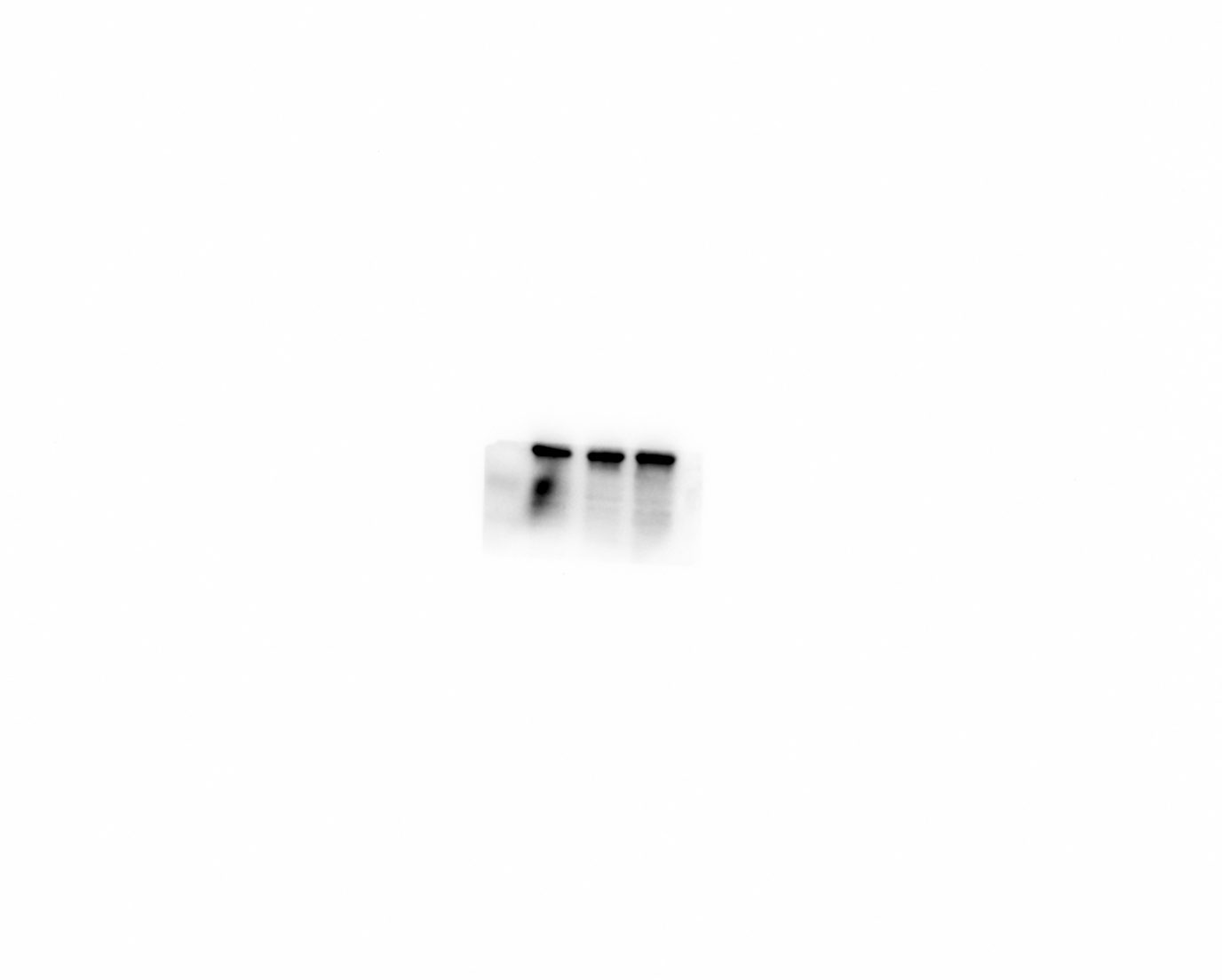

Supplement: Supplementary file 6 — Source data Fig. 1 [file 44318_2024_359_MOESM6_ESM.zip › Figure 1/Fig 1I/2-GAPDH-1.Tif]

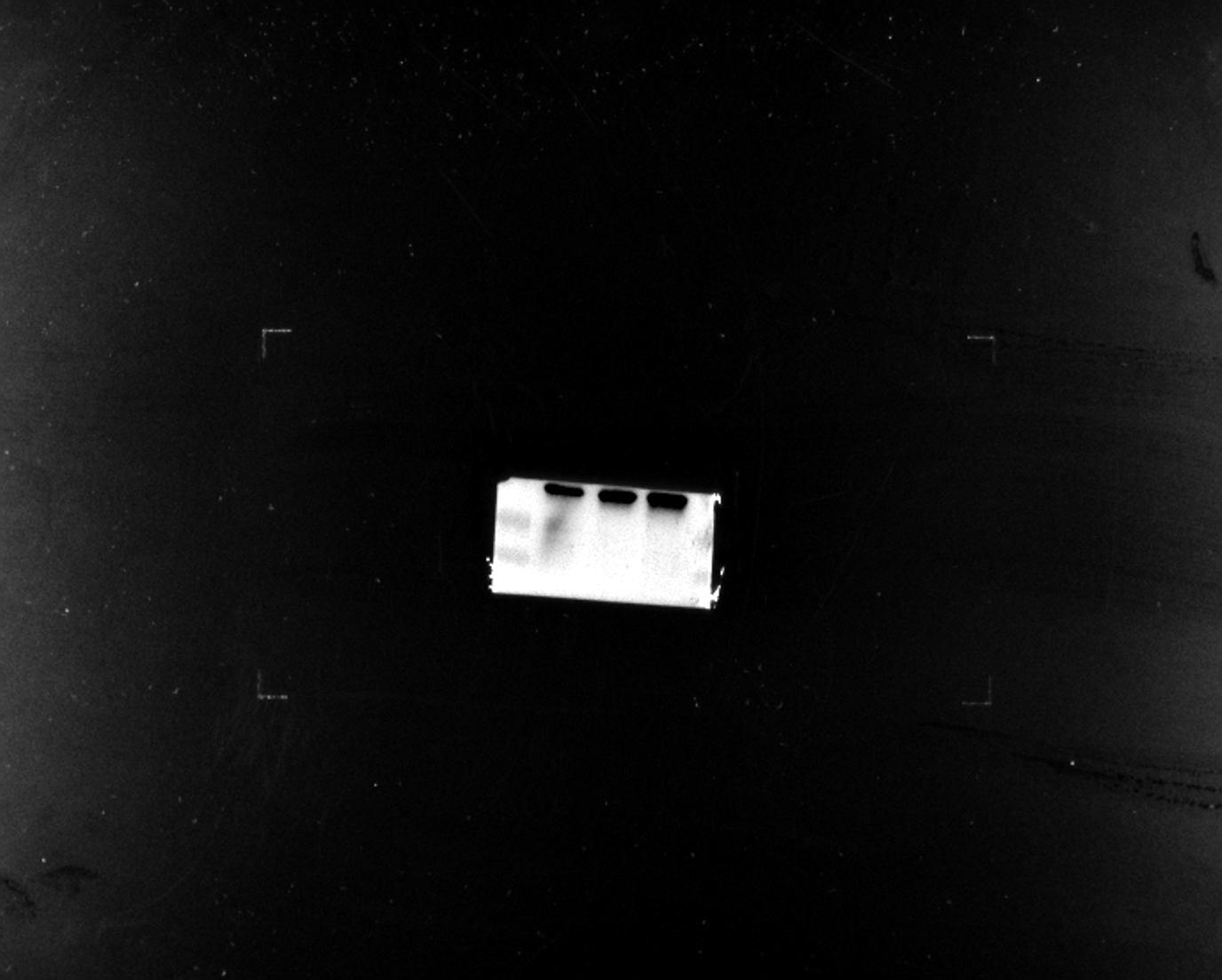

Supplement: Supplementary file 6 — Source data Fig. 1 [file 44318_2024_359_MOESM6_ESM.zip › Figure 1/Fig 1I/2-GAPDH-merge.Tif]

**Fig 1l**

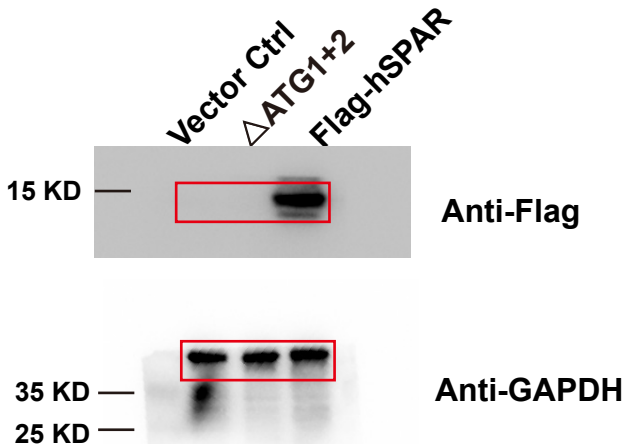

Supplement: Supplementary file 6 — Source data Fig. 1 [file 44318_2024_359_MOESM6_ESM.zip › Figure 1/Fig 1I/Fig 1I.pdf]

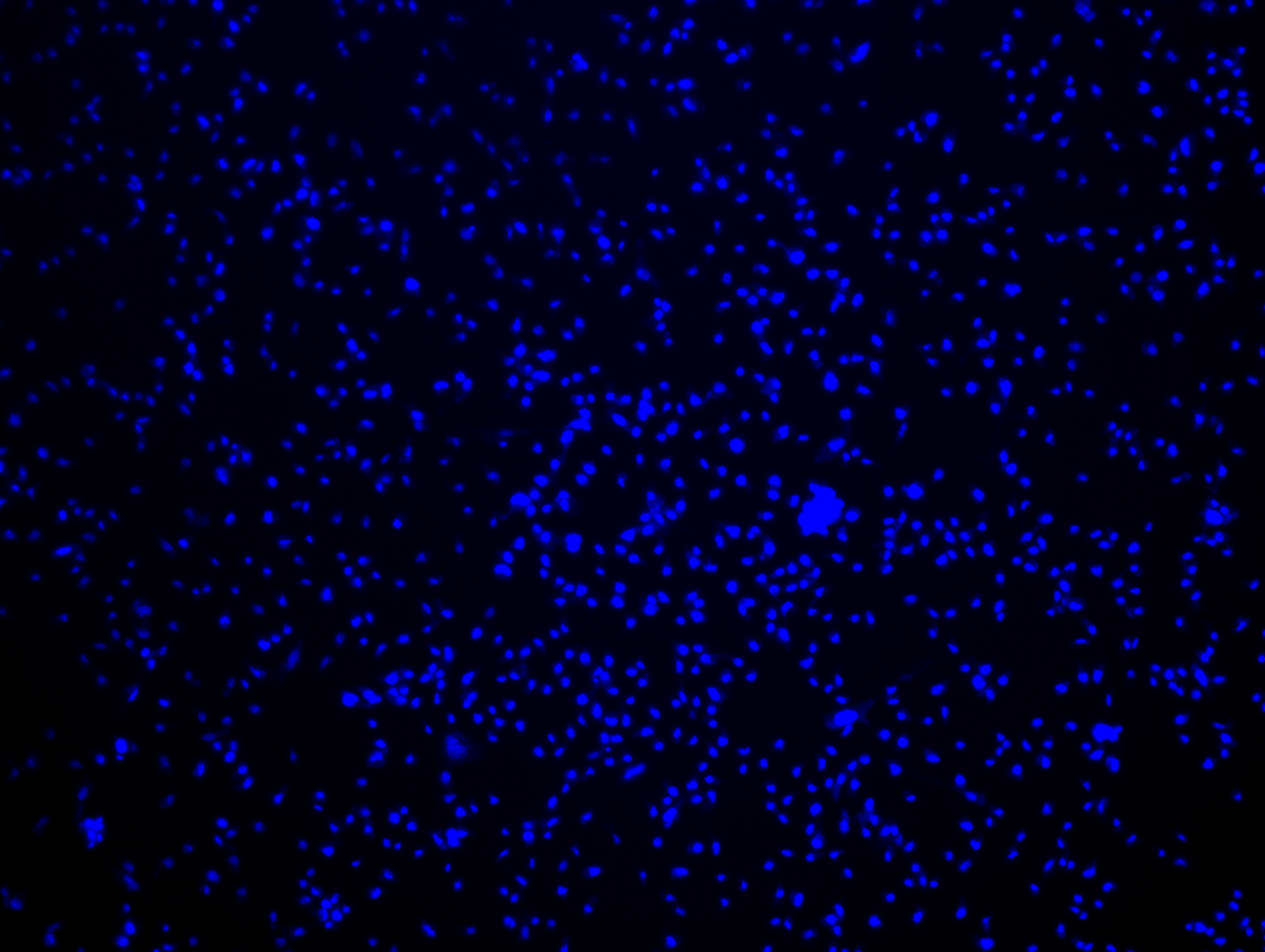

Supplement: Supplementary file 6 — Source data Fig. 1 [file 44318_2024_359_MOESM6_ESM.zip › Figure 1/Fig 1J and 1K/Fig 1J/ATG1+2/ATG1+2-Hoechst.tif]

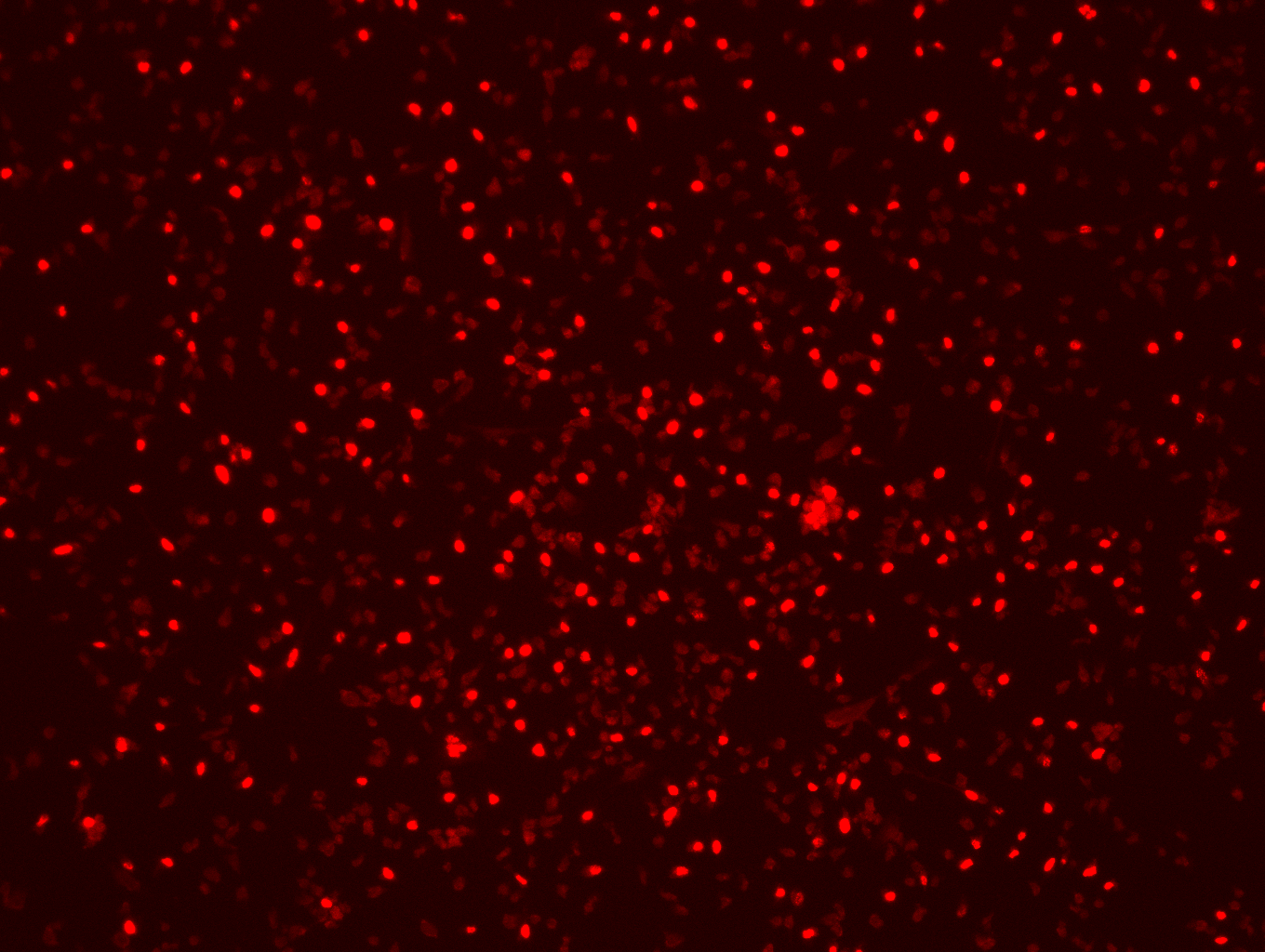

Supplement: Supplementary file 6 — Source data Fig. 1 [file 44318_2024_359_MOESM6_ESM.zip › Figure 1/Fig 1J and 1K/Fig 1J/ATG1+2/ATG1+2-edu.tif]

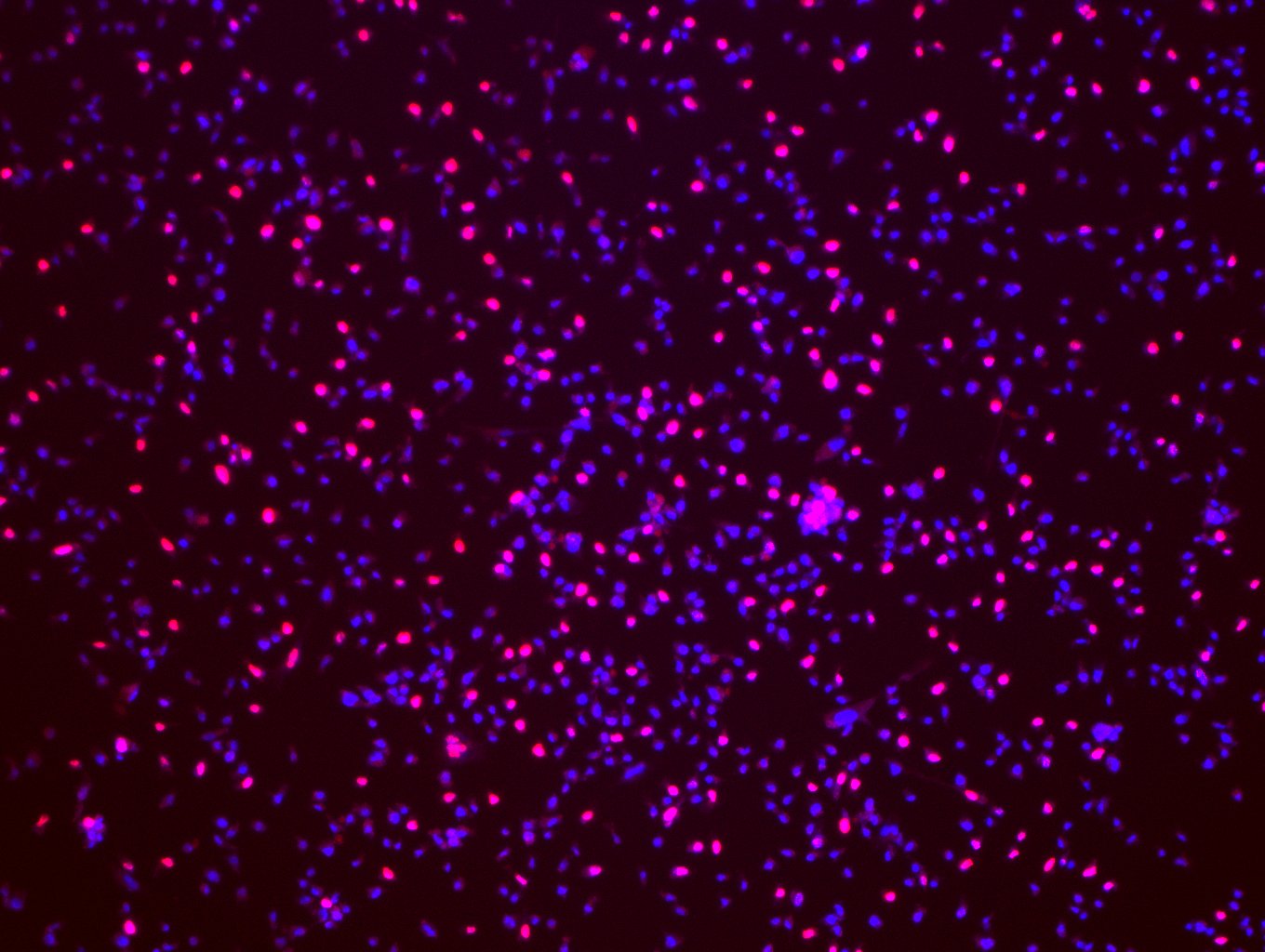

Supplement: Supplementary file 6 — Source data Fig. 1 [file 44318_2024_359_MOESM6_ESM.zip › Figure 1/Fig 1J and 1K/Fig 1J/ATG1+2/ATG1+2-merge.jpg]

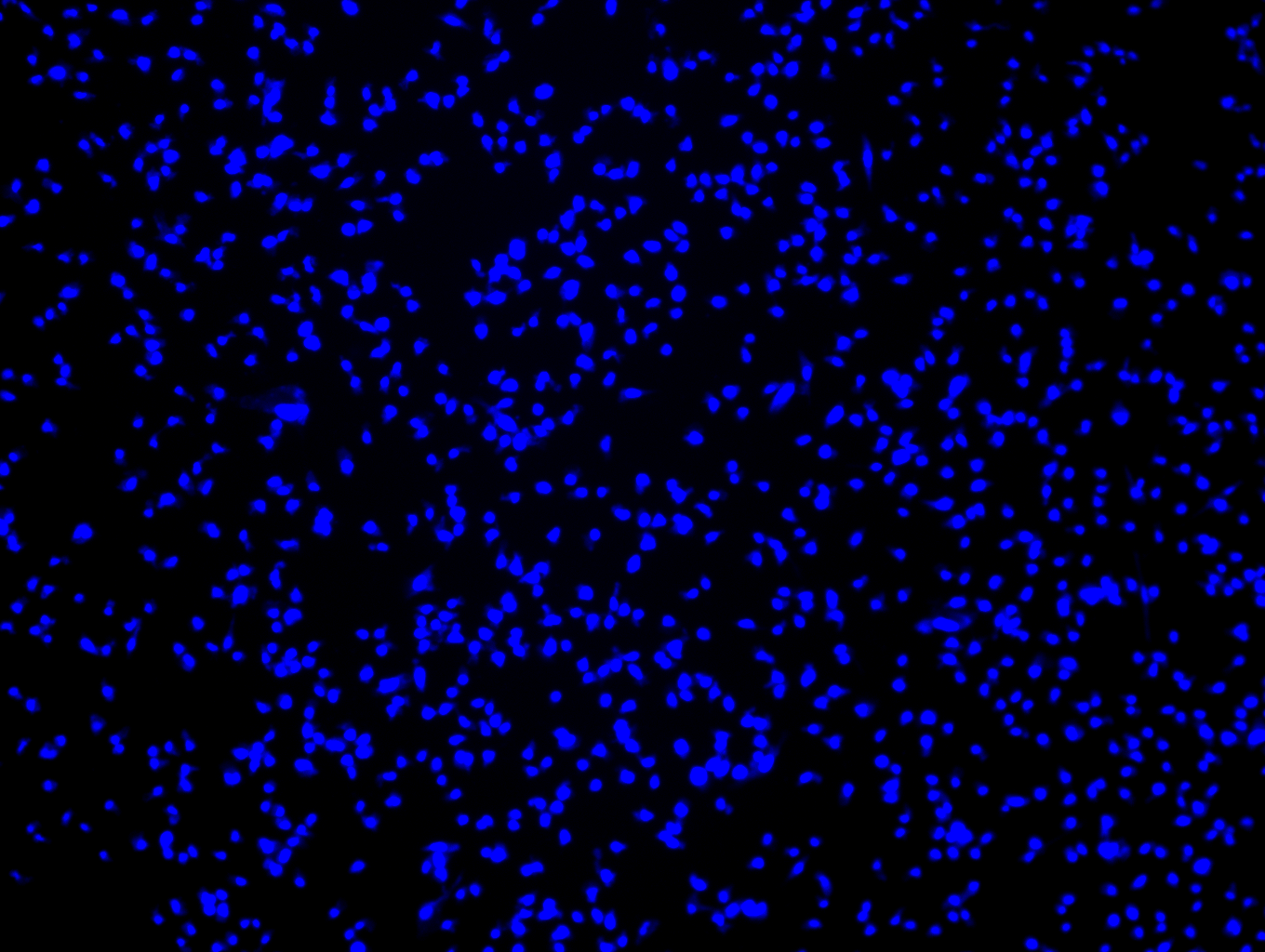

Supplement: Supplementary file 6 — Source data Fig. 1 [file 44318_2024_359_MOESM6_ESM.zip › Figure 1/Fig 1J and 1K/Fig 1J/Flag-hSPAR/hSPAR-Hoechst.tif]

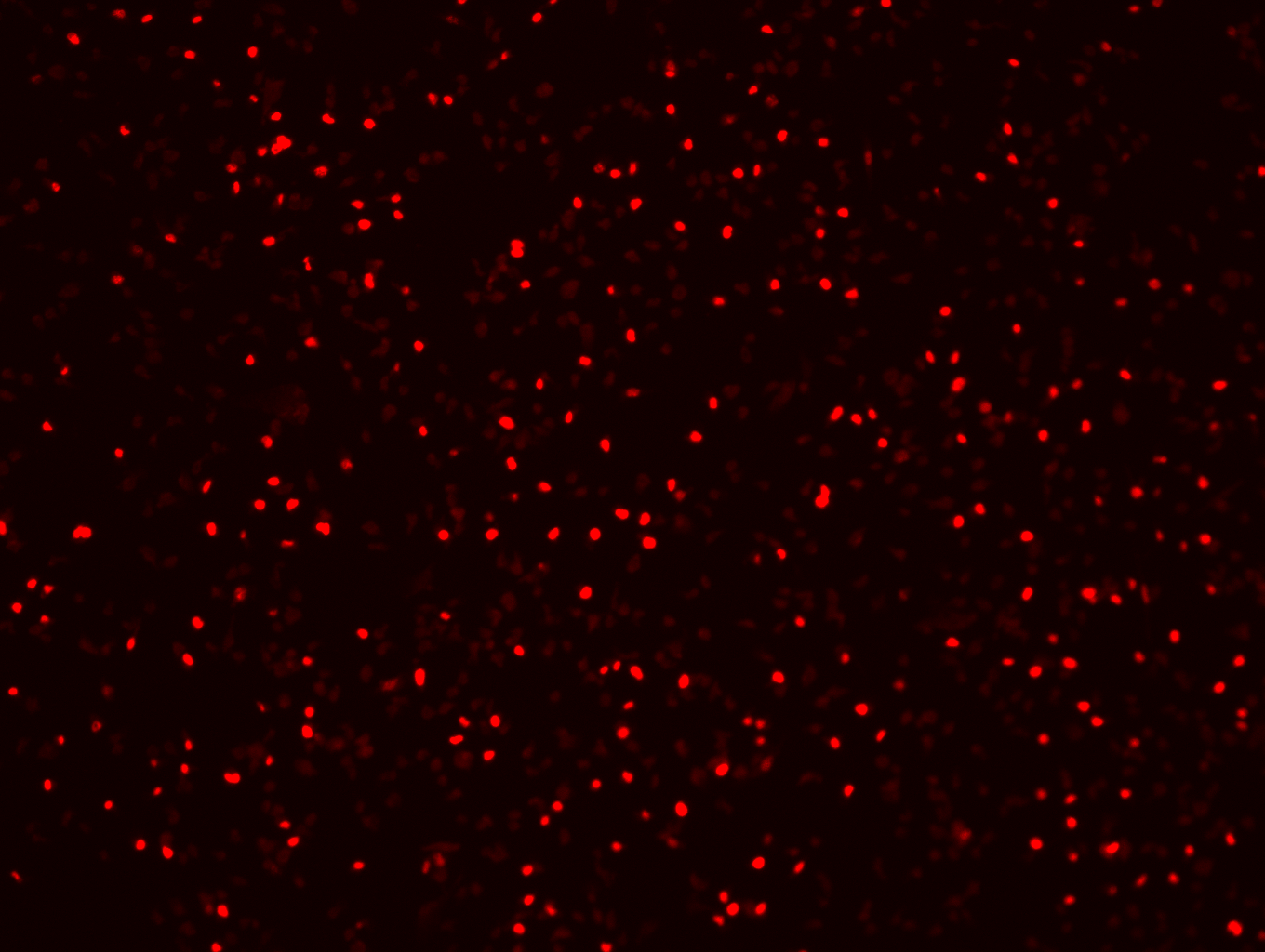

Supplement: Supplementary file 6 — Source data Fig. 1 [file 44318_2024_359_MOESM6_ESM.zip › Figure 1/Fig 1J and 1K/Fig 1J/Flag-hSPAR/hSPAR-edu.tif]

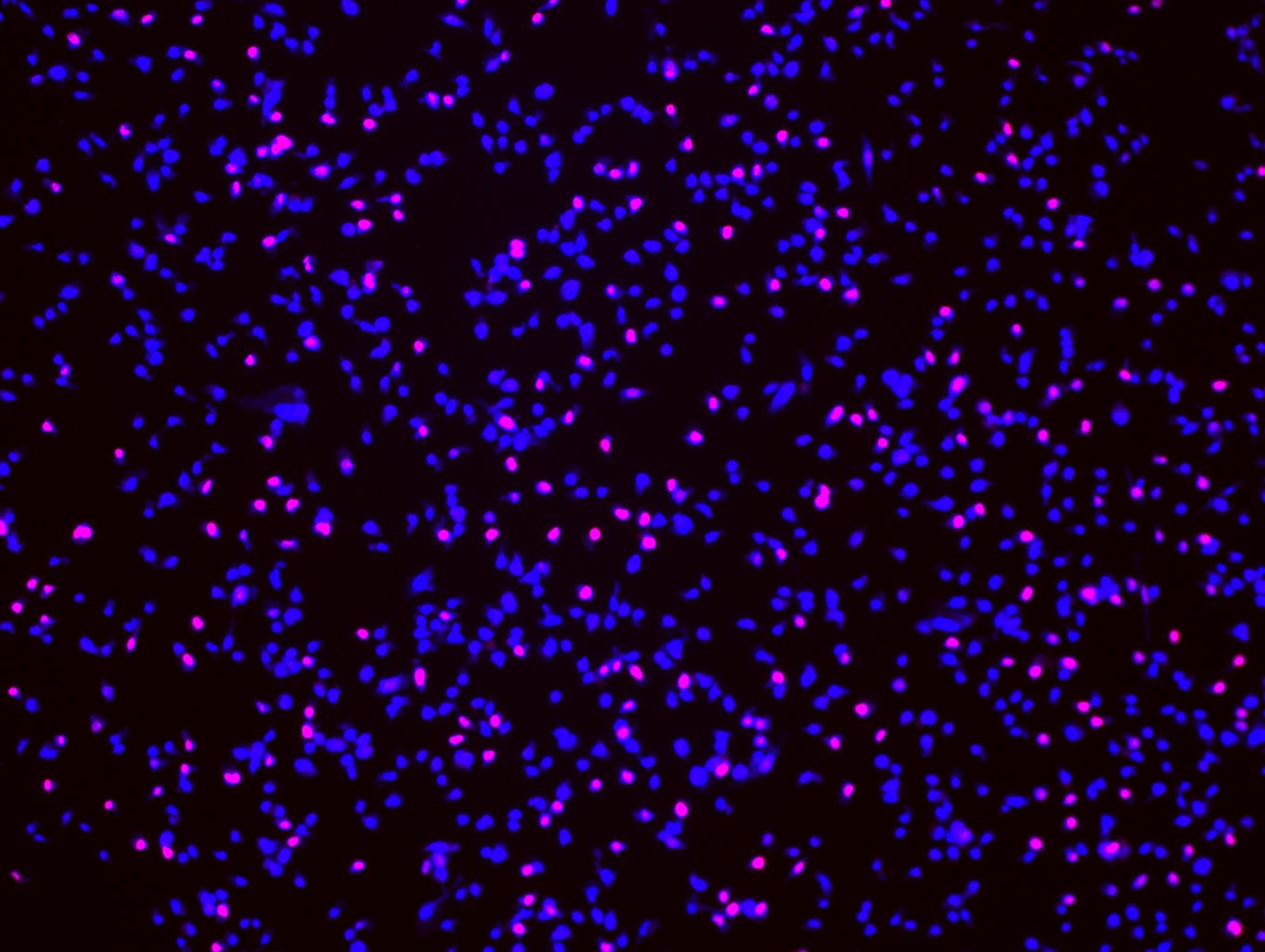

Supplement: Supplementary file 6 — Source data Fig. 1 [file 44318_2024_359_MOESM6_ESM.zip › Figure 1/Fig 1J and 1K/Fig 1J/Flag-hSPAR/hSPAR-merge.jpg]

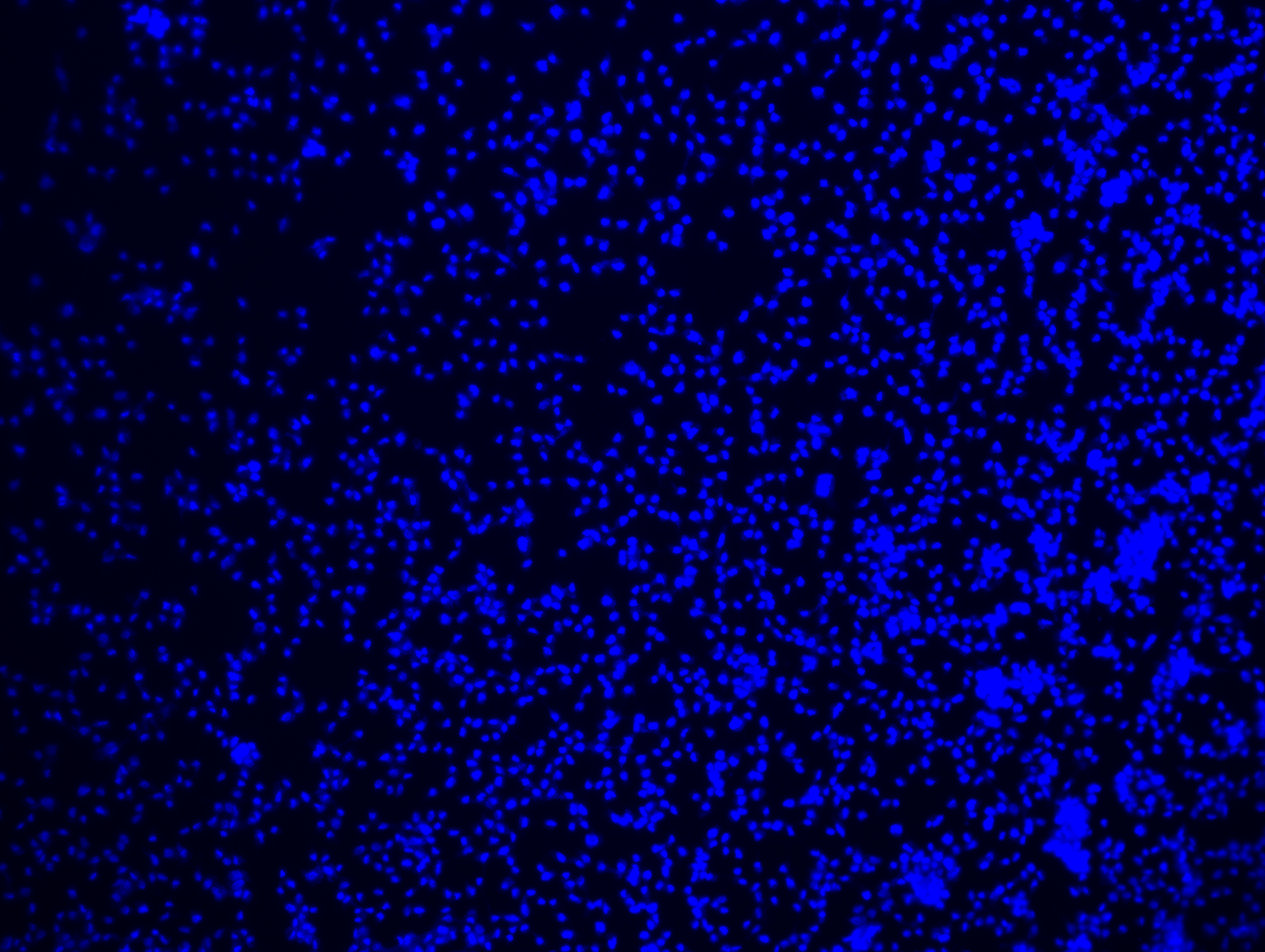

Supplement: Supplementary file 6 — Source data Fig. 1 [file 44318_2024_359_MOESM6_ESM.zip › Figure 1/Fig 1J and 1K/Fig 1J/Vector Ctrl/Vector Ctrl-Hoechst.tif]

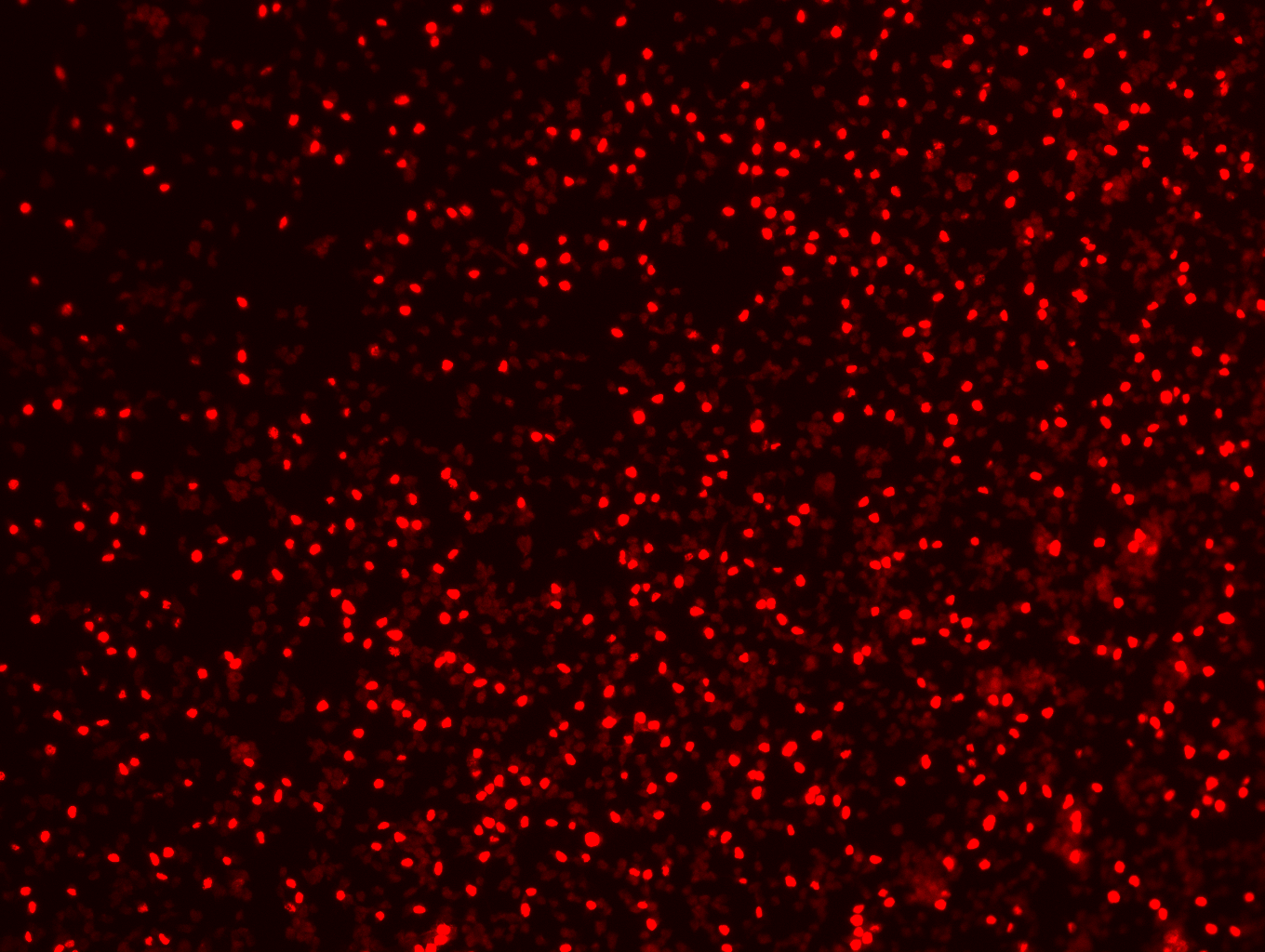

Supplement: Supplementary file 6 — Source data Fig. 1 [file 44318_2024_359_MOESM6_ESM.zip › Figure 1/Fig 1J and 1K/Fig 1J/Vector Ctrl/Vector Ctrl-edu.tif]

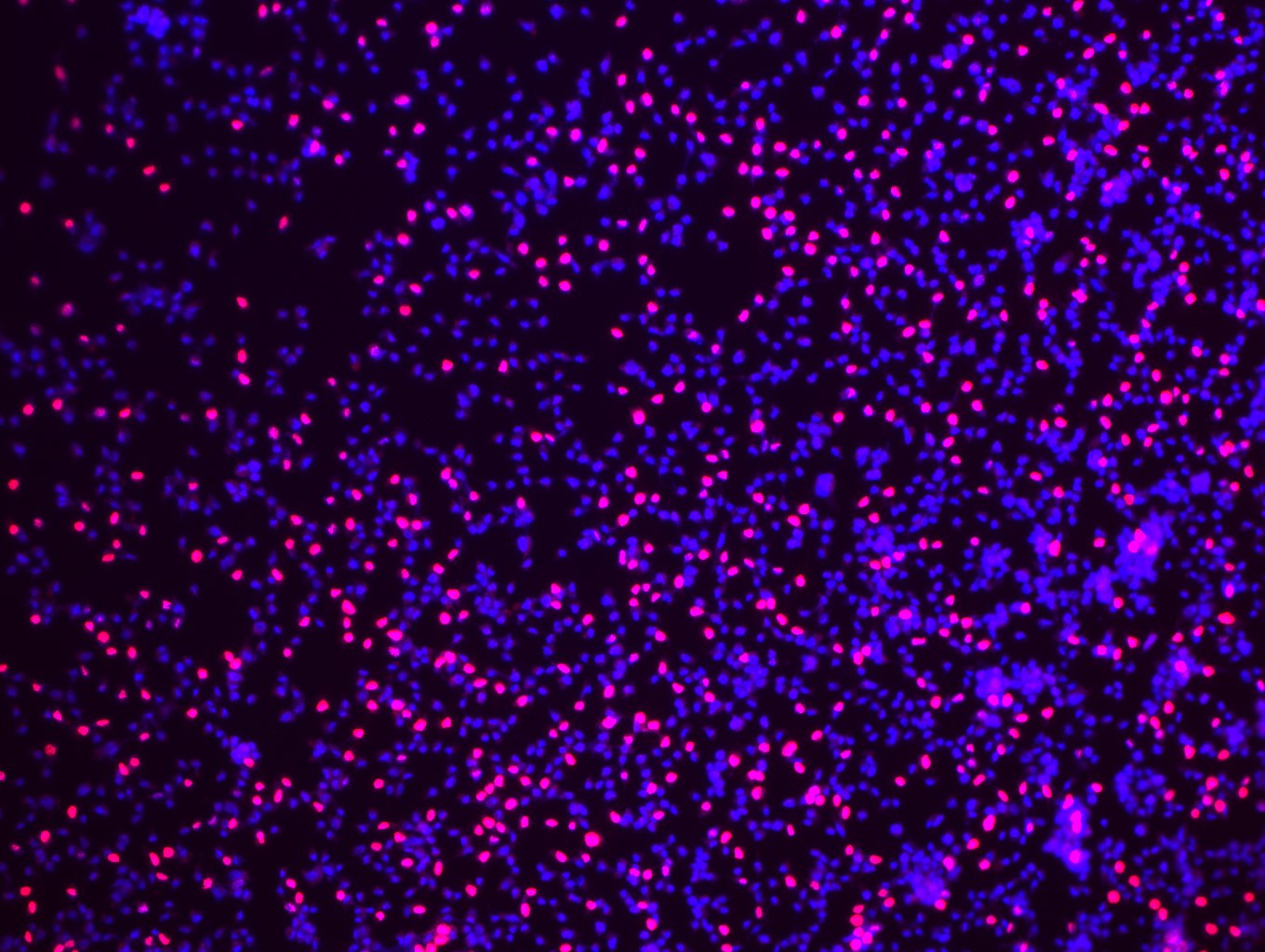

Supplement: Supplementary file 6 — Source data Fig. 1 [file 44318_2024_359_MOESM6_ESM.zip › Figure 1/Fig 1J and 1K/Fig 1J/Vector Ctrl/Vector Ctrl-merge.jpg]

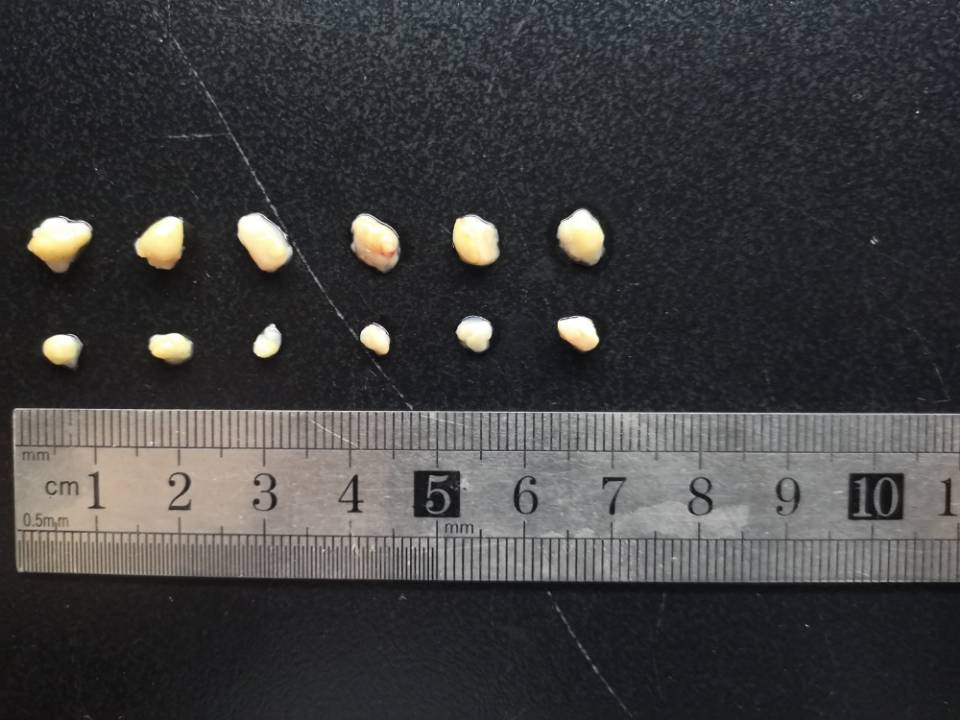

Supplement: Supplementary file 6 — Source data Fig. 1 [file 44318_2024_359_MOESM6_ESM.zip › Figure 1/Fig 1L and 1M/Fig 1L.jpg]

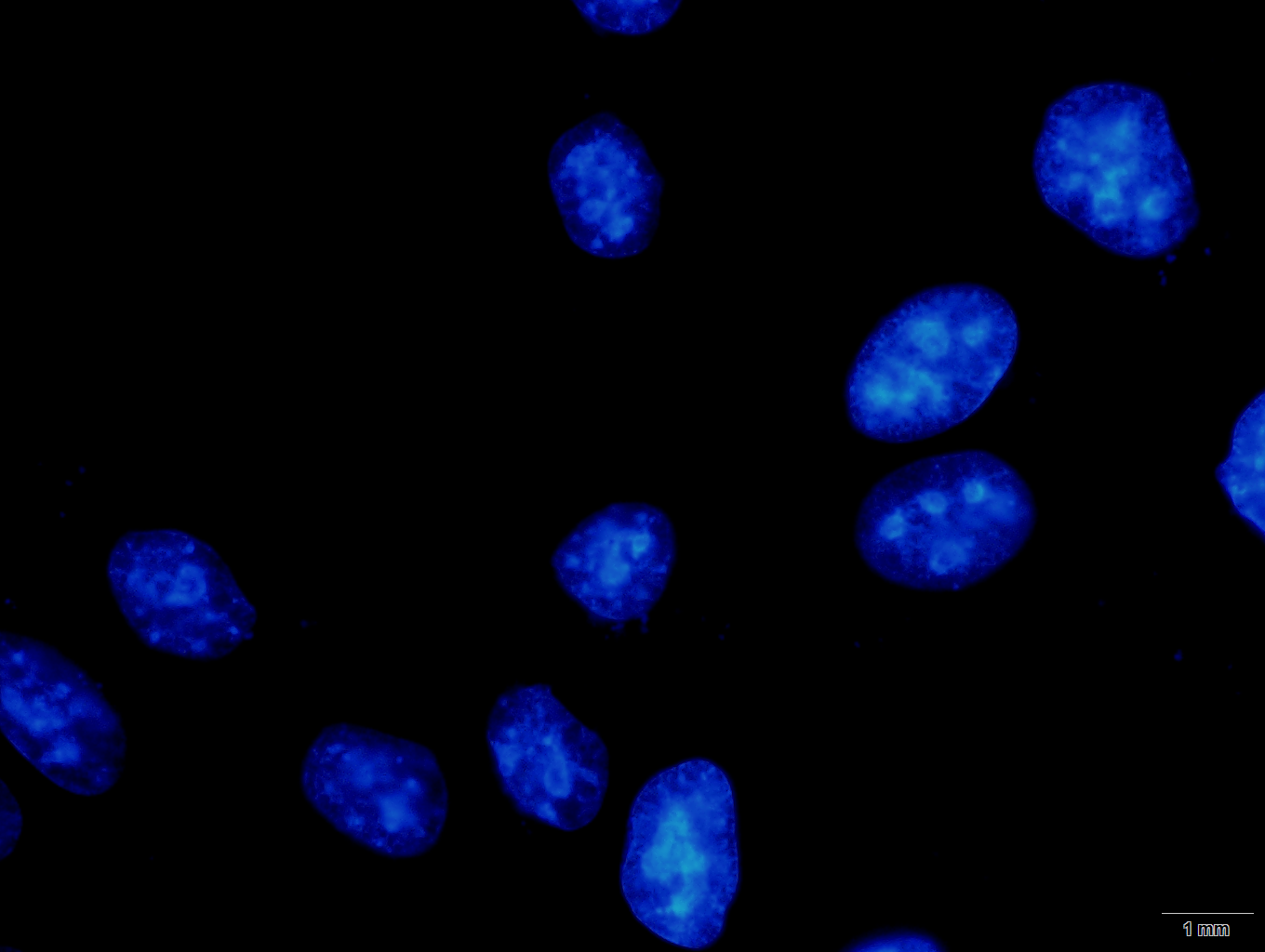

Supplement: Supplementary file 7 — Source data Fig. 2 [file 44318_2024_359_MOESM7_ESM.zip › Figure 2/Fig 2A/Ctrl/Hoechst.png]

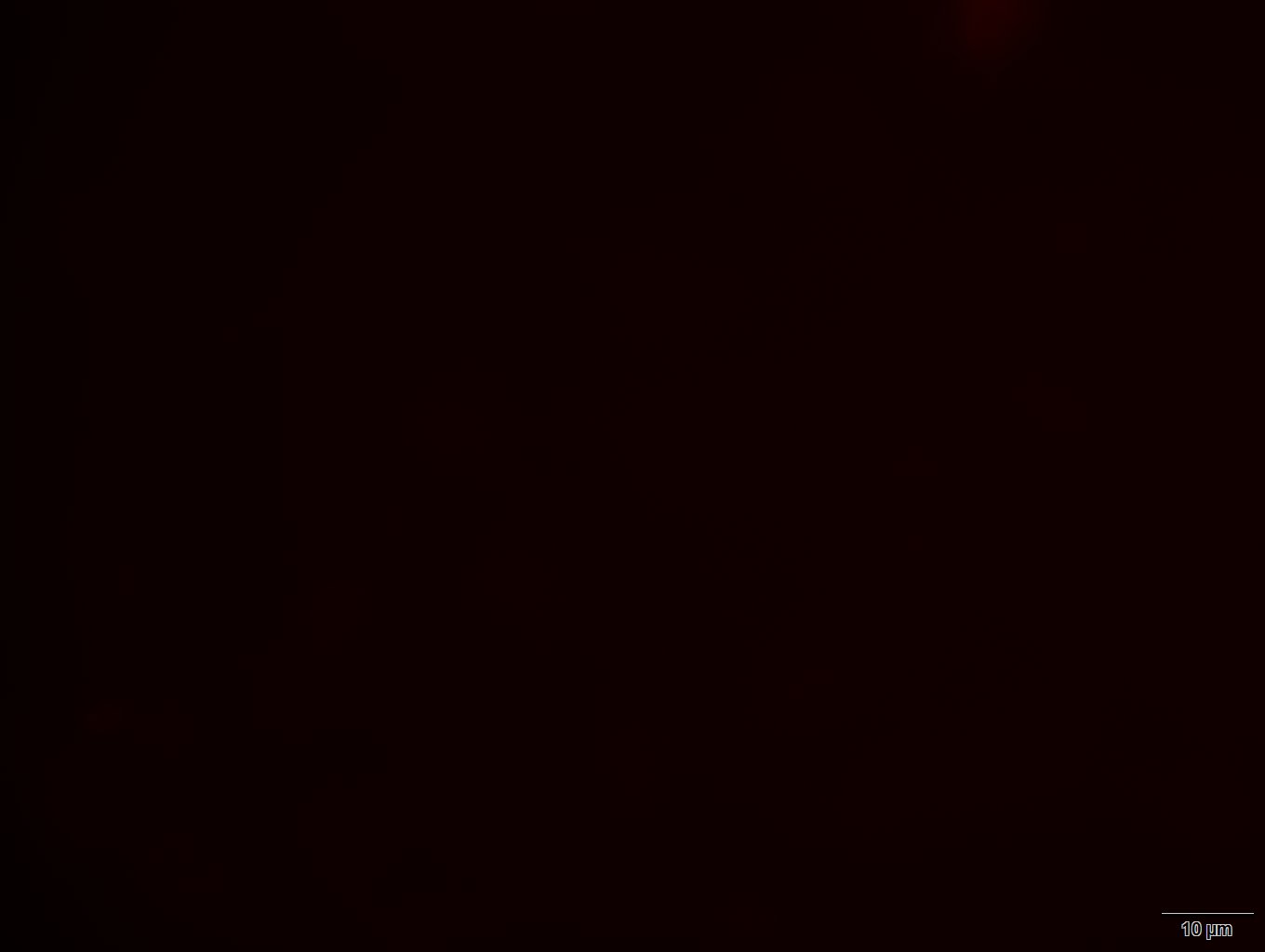

Supplement: Supplementary file 7 — Source data Fig. 2 [file 44318_2024_359_MOESM7_ESM.zip › Figure 2/Fig 2A/Ctrl/No Primary Antibody.jpg]

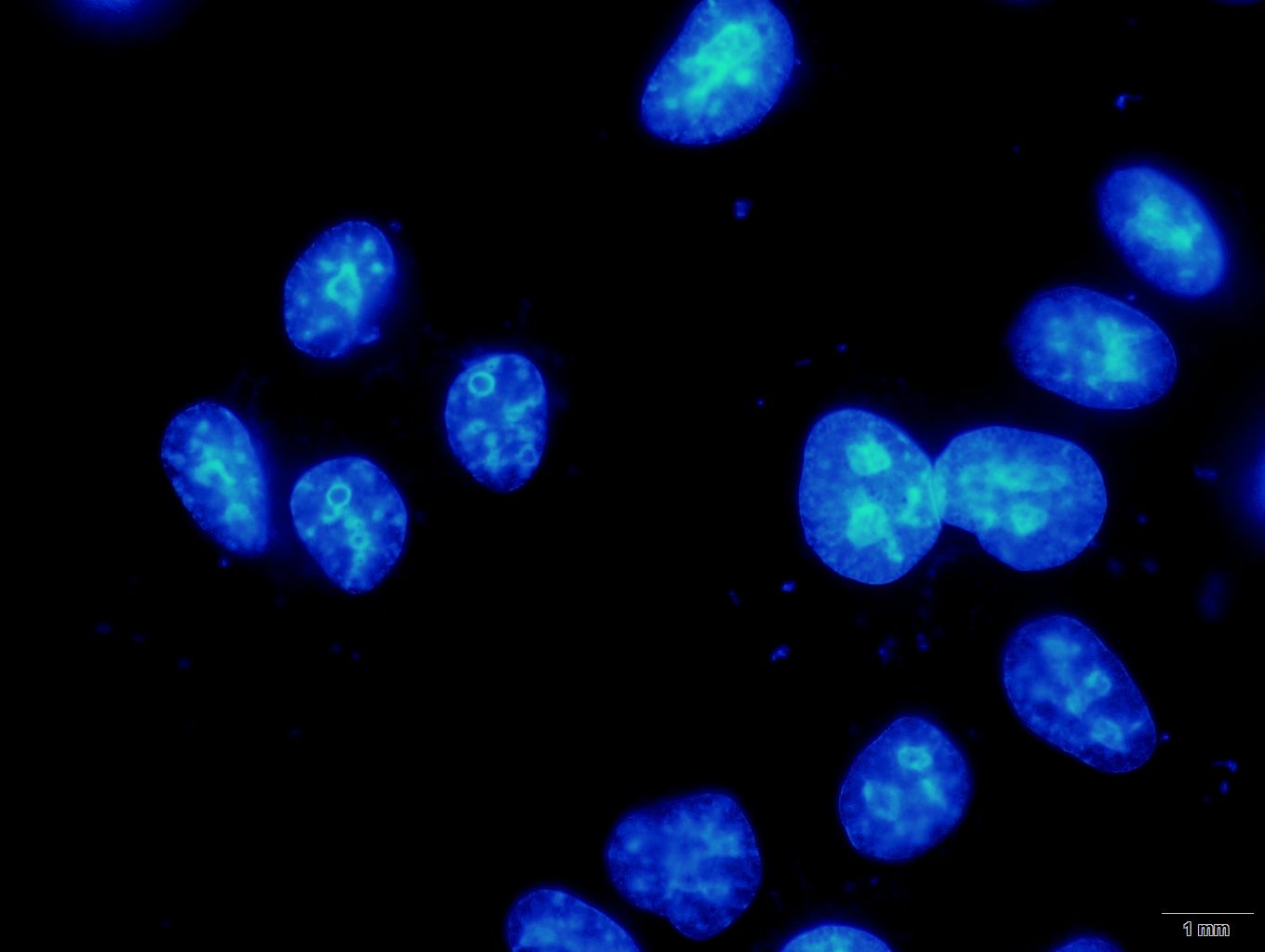

Supplement: Supplementary file 7 — Source data Fig. 2 [file 44318_2024_359_MOESM7_ESM.zip › Figure 2/Fig 2A/hSPAR/Hoechst.png]

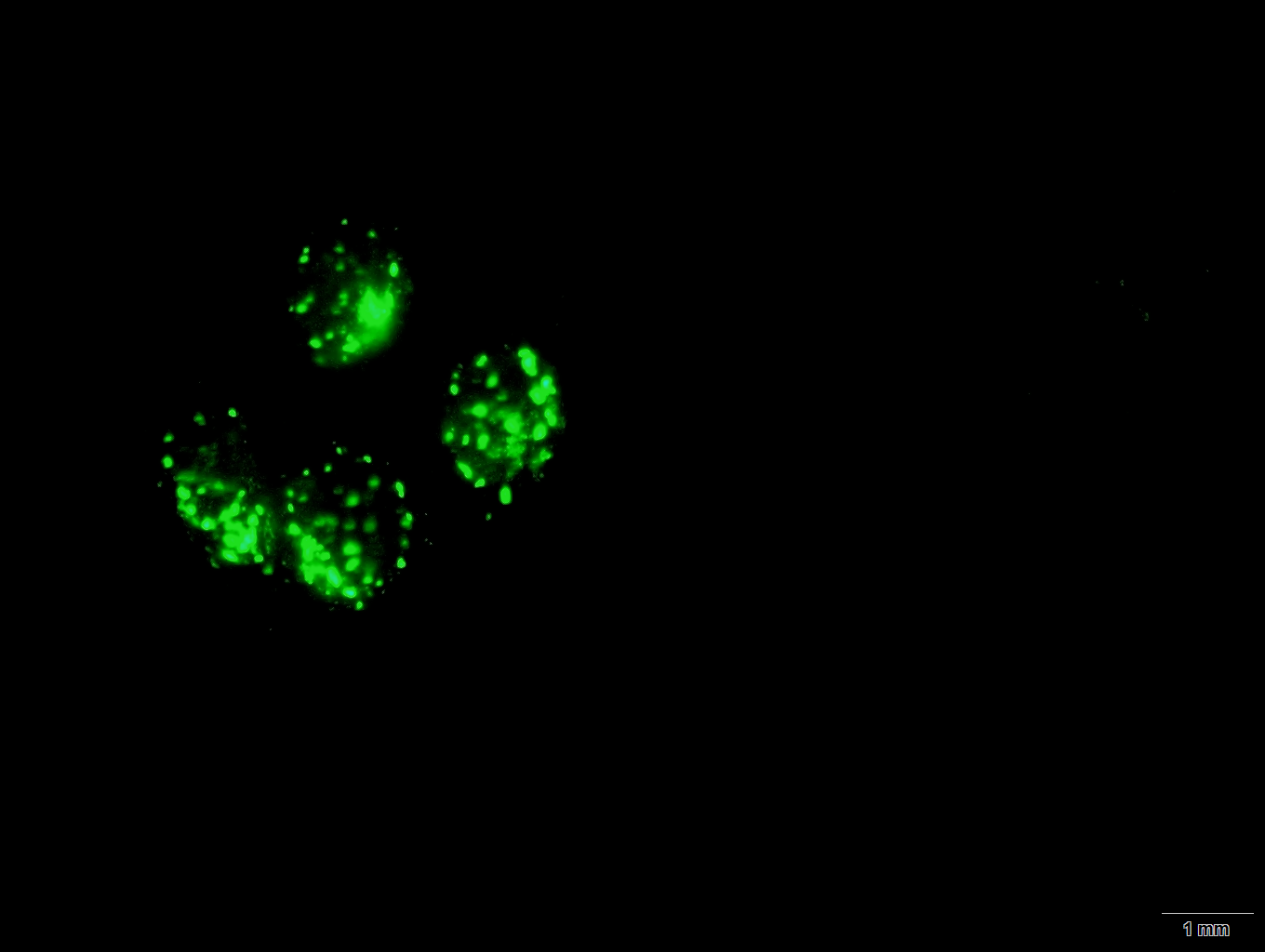

Supplement: Supplementary file 7 — Source data Fig. 2 [file 44318_2024_359_MOESM7_ESM.zip › Figure 2/Fig 2A/hSPAR/hSPAR.png]

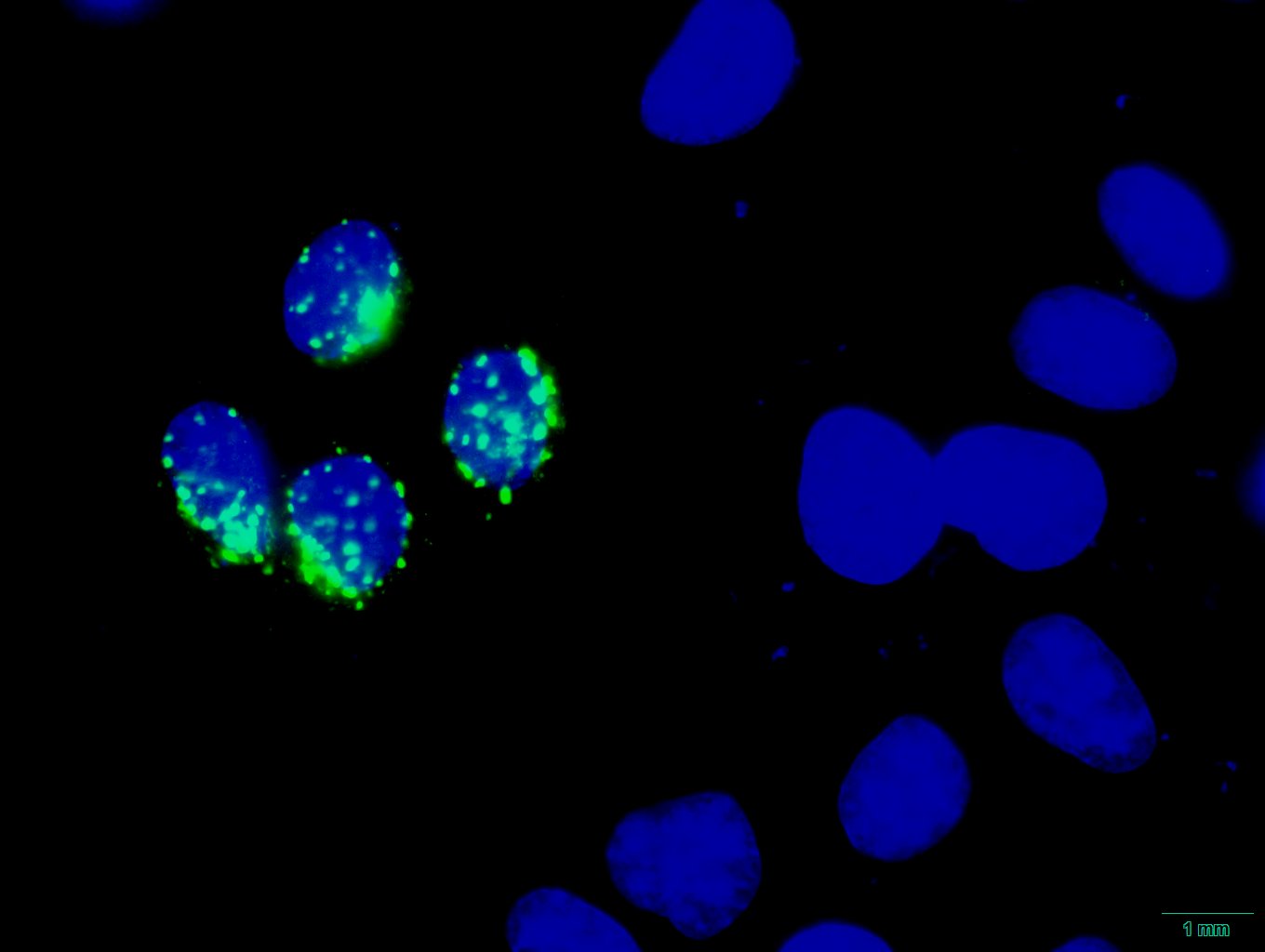

Supplement: Supplementary file 7 — Source data Fig. 2 [file 44318_2024_359_MOESM7_ESM.zip › Figure 2/Fig 2A/hSPAR/merge.jpg]

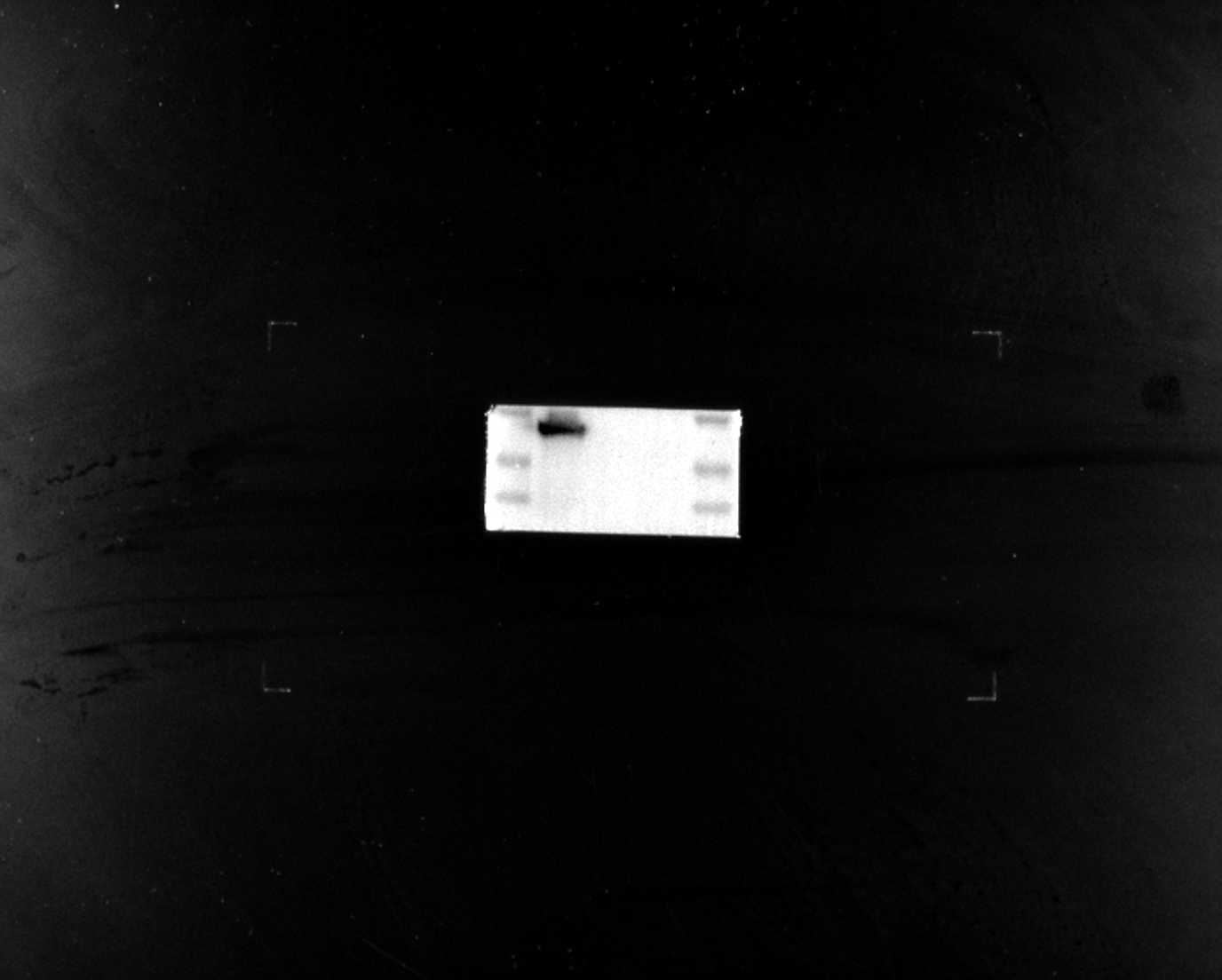

Supplement: Supplementary file 7 — Source data Fig. 2 [file 44318_2024_359_MOESM7_ESM.zip › Figure 2/Fig 2B/1-FBL-merge.Tif]

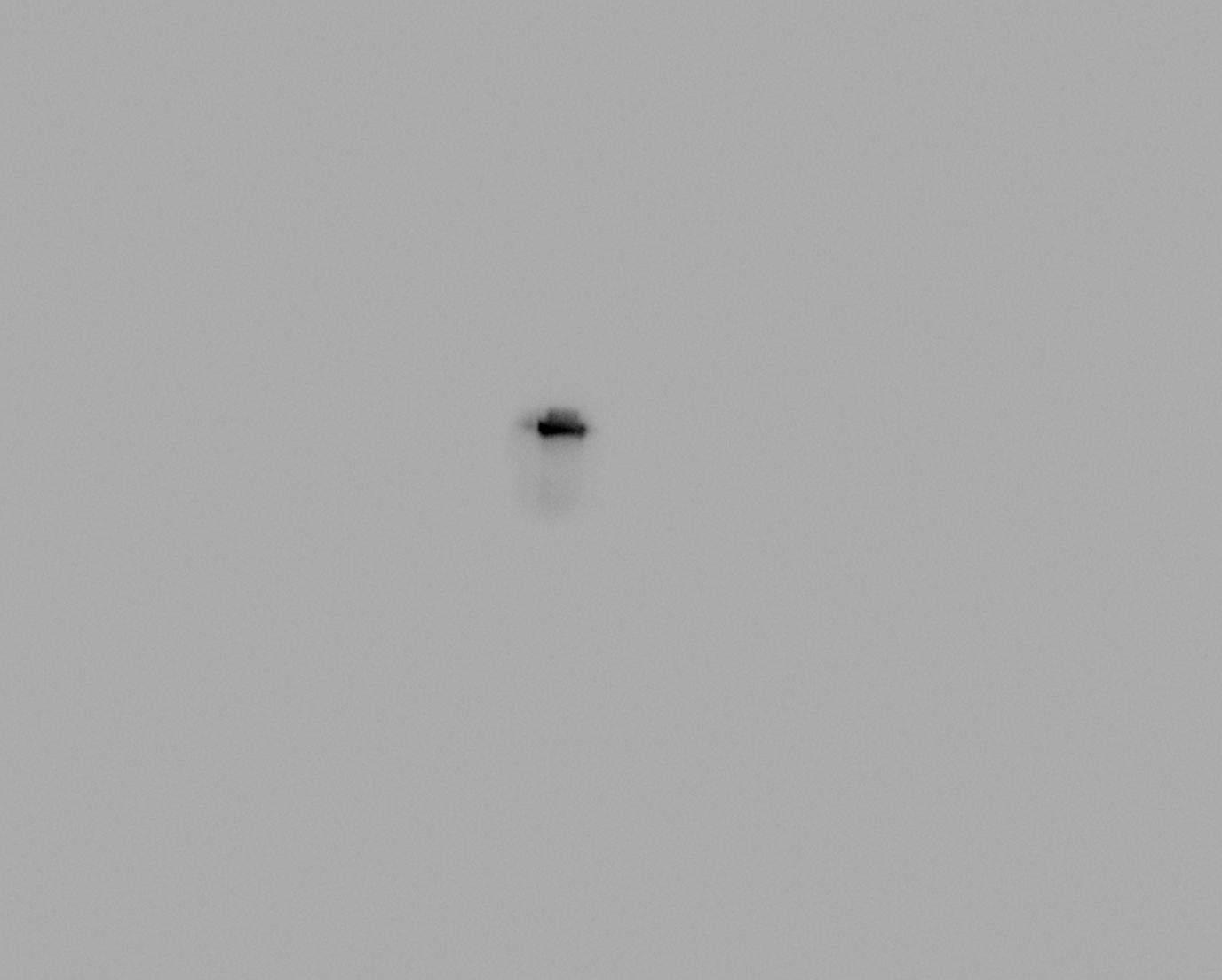

Supplement: Supplementary file 7 — Source data Fig. 2 [file 44318_2024_359_MOESM7_ESM.zip › Figure 2/Fig 2B/1-FBL.Tif]

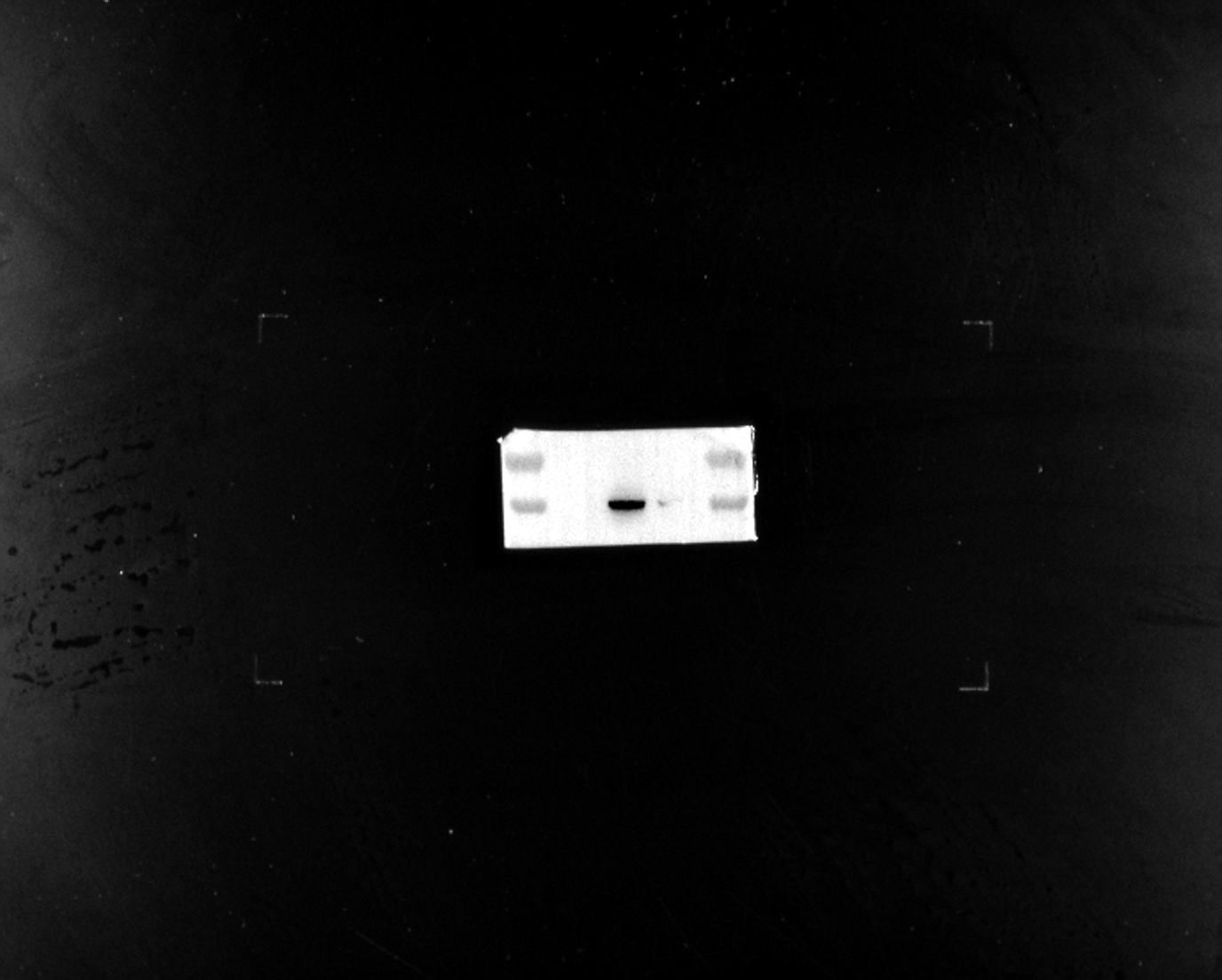

Supplement: Supplementary file 7 — Source data Fig. 2 [file 44318_2024_359_MOESM7_ESM.zip › Figure 2/Fig 2B/2-TUBULIN-merge.Tif]

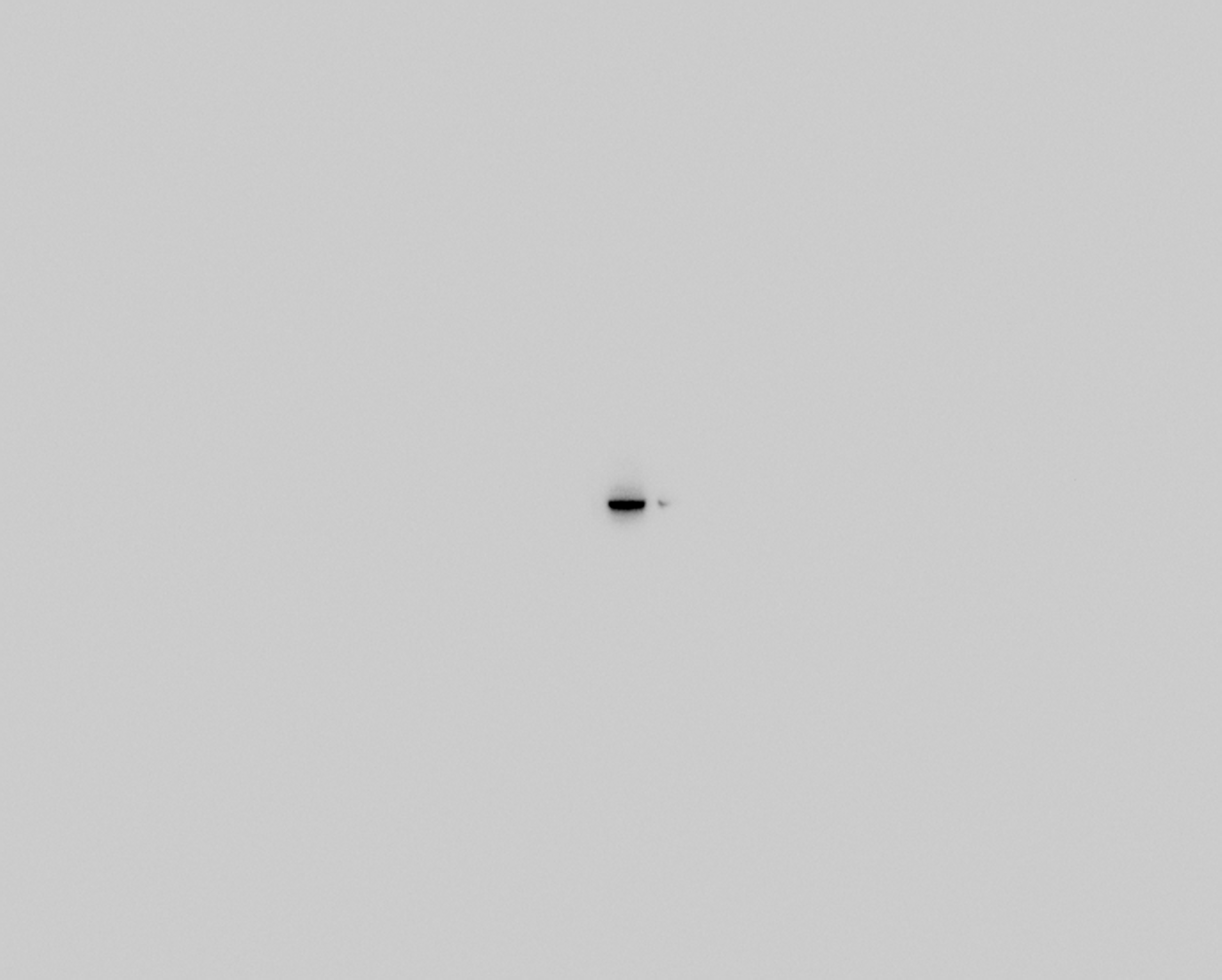

Supplement: Supplementary file 7 — Source data Fig. 2 [file 44318_2024_359_MOESM7_ESM.zip › Figure 2/Fig 2B/2-TUBULIN.Tif]

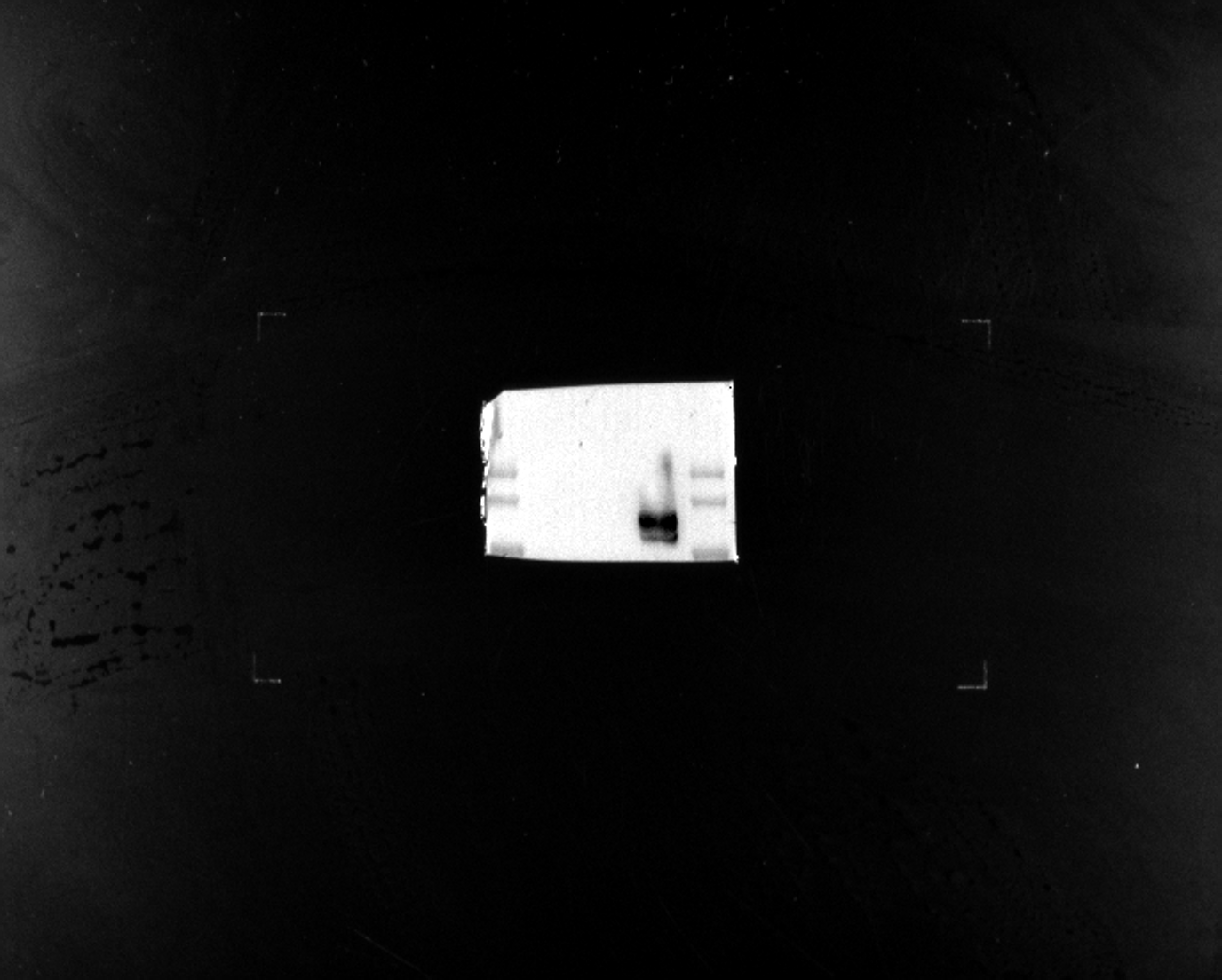

Supplement: Supplementary file 7 — Source data Fig. 2 [file 44318_2024_359_MOESM7_ESM.zip › Figure 2/Fig 2B/3-ATP1V1-merge.Tif]

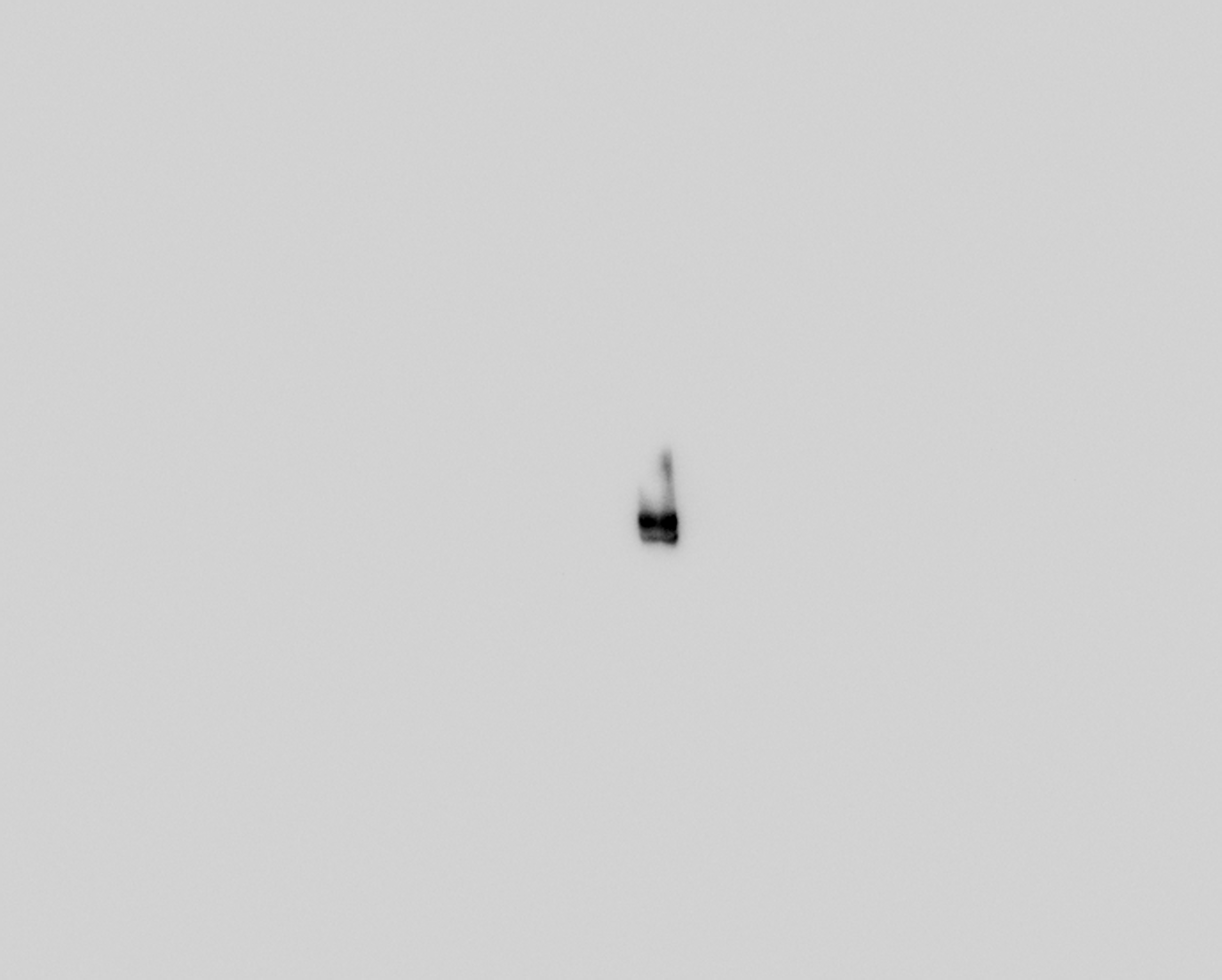

Supplement: Supplementary file 7 — Source data Fig. 2 [file 44318_2024_359_MOESM7_ESM.zip › Figure 2/Fig 2B/3-ATP1V1.Tif]

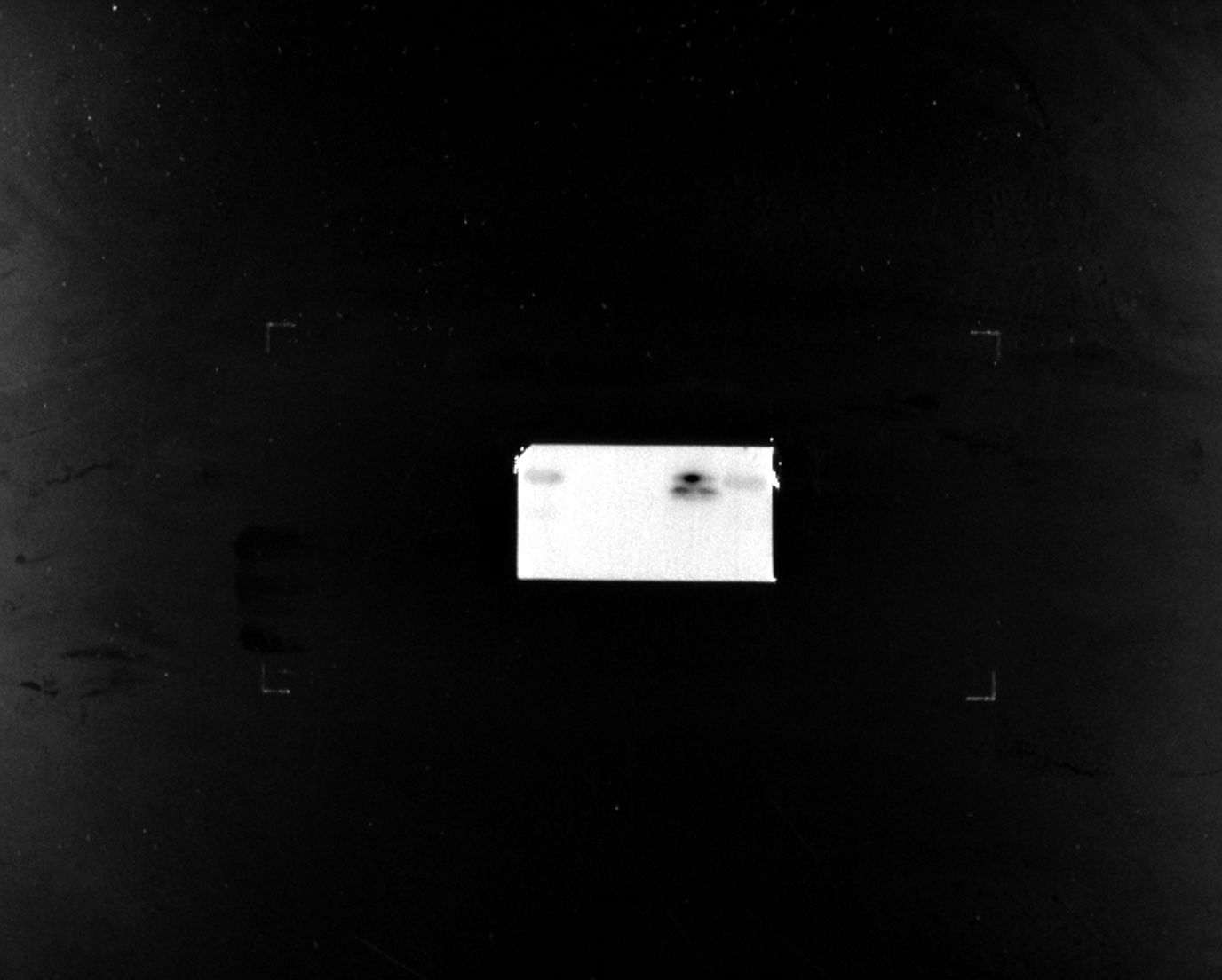

Supplement: Supplementary file 7 — Source data Fig. 2 [file 44318_2024_359_MOESM7_ESM.zip › Figure 2/Fig 2B/4-hSPAR-merge.Tif]

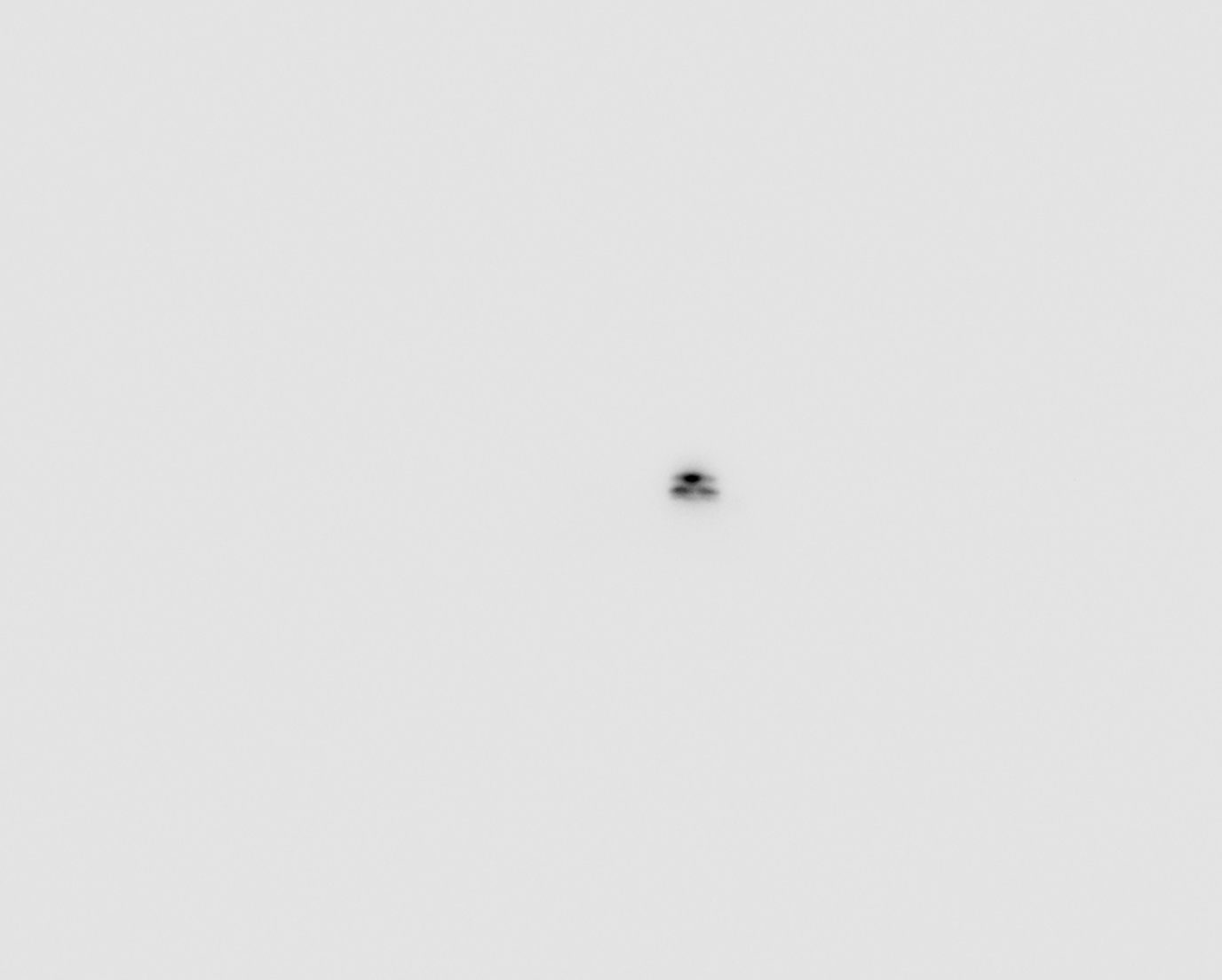

Supplement: Supplementary file 7 — Source data Fig. 2 [file 44318_2024_359_MOESM7_ESM.zip › Figure 2/Fig 2B/4-hSPAR.Tif]

**Fig 2B**

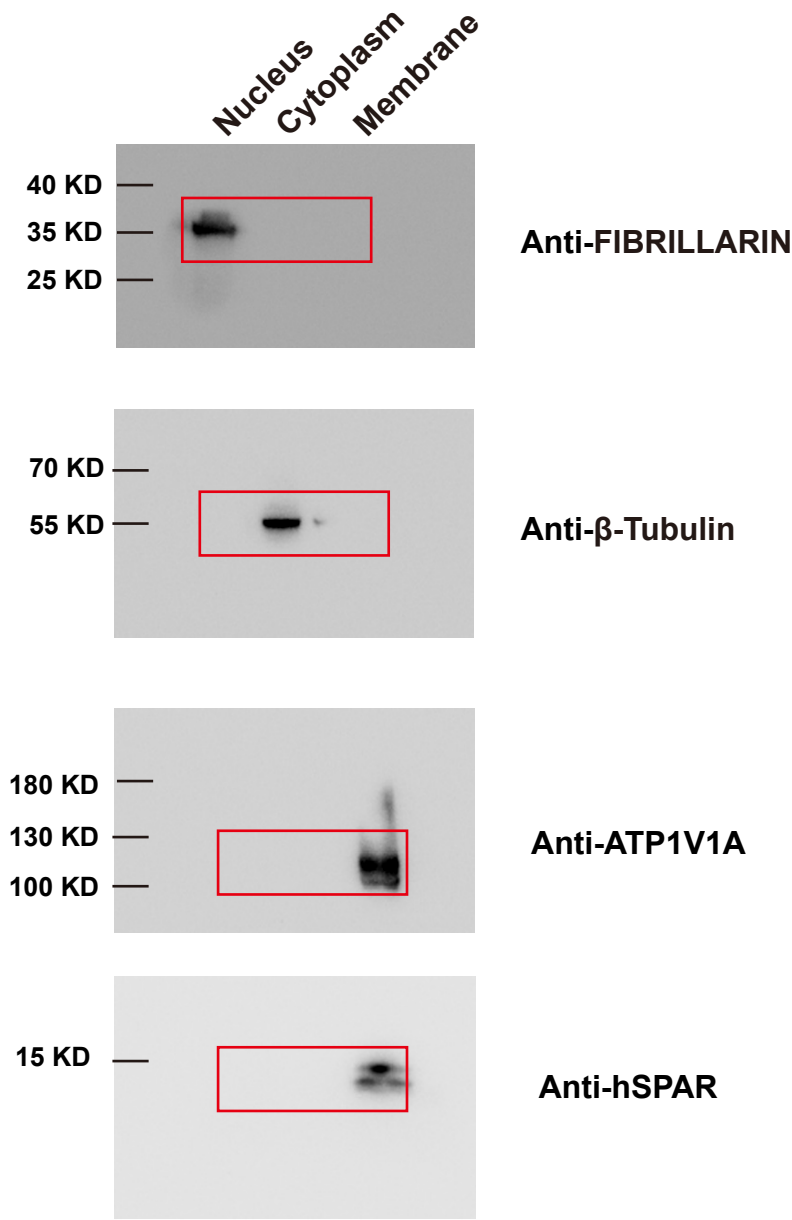

Supplement: Supplementary file 7 — Source data Fig. 2 [file 44318_2024_359_MOESM7_ESM.zip › Figure 2/Fig 2B/Fig 2B.pdf]

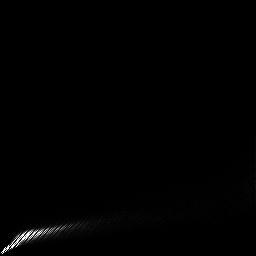

Supplement: Supplementary file 7 — Source data Fig. 2 [file 44318_2024_359_MOESM7_ESM.zip › Figure 2/Fig 2D/Fig 2D Pearson correlation.jpg]

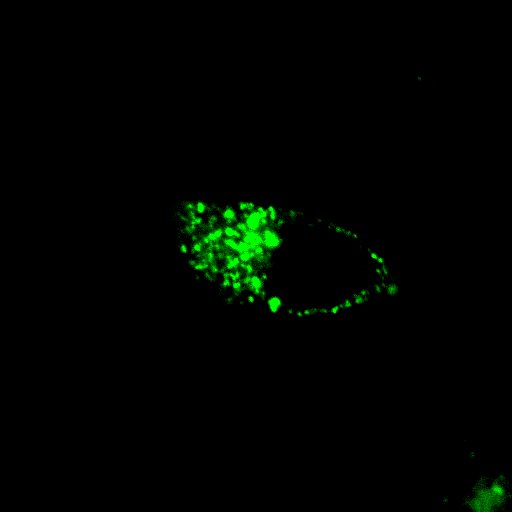

Supplement: Supplementary file 7 — Source data Fig. 2 [file 44318_2024_359_MOESM7_ESM.zip › Figure 2/Fig 2D/GFP-hSPAR.jpg]

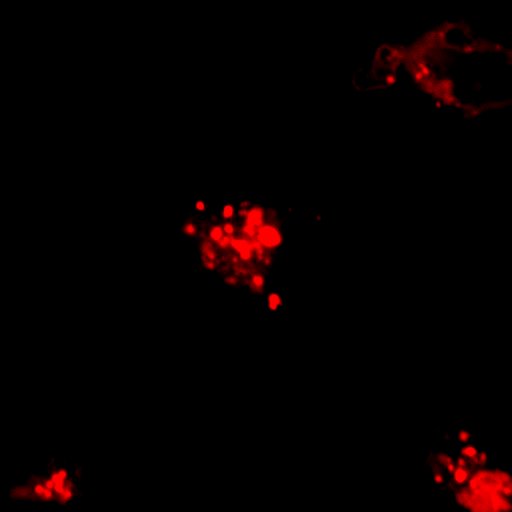

Supplement: Supplementary file 7 — Source data Fig. 2 [file 44318_2024_359_MOESM7_ESM.zip › Figure 2/Fig 2D/lysotracker.jpg]

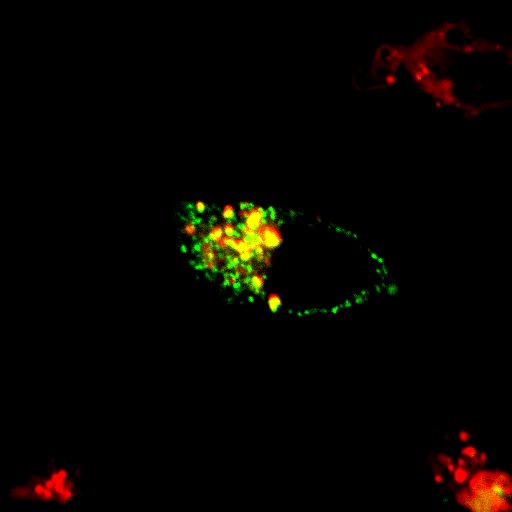

Supplement: Supplementary file 7 — Source data Fig. 2 [file 44318_2024_359_MOESM7_ESM.zip › Figure 2/Fig 2D/merge.jpg]

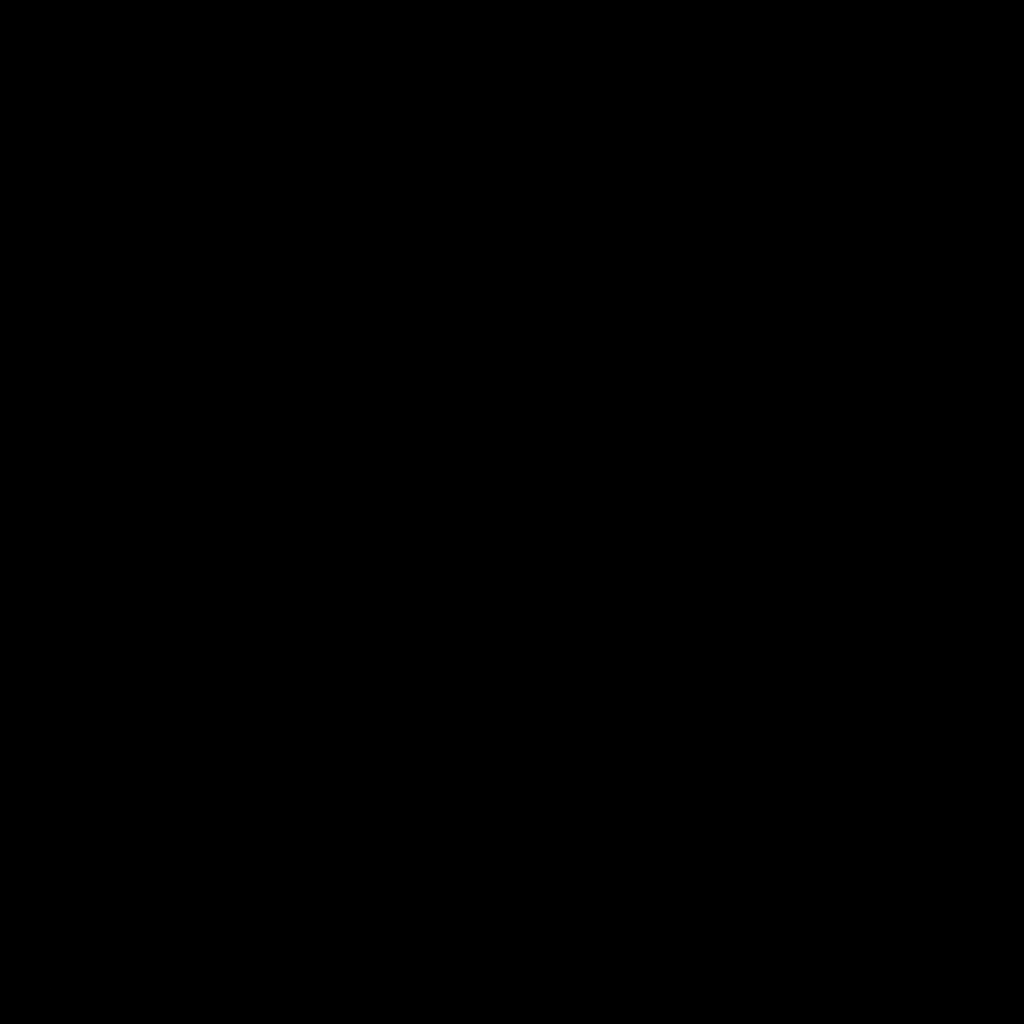

Supplement: Supplementary file 7 — Source data Fig. 2 [file 44318_2024_359_MOESM7_ESM.zip › Figure 2/Fig 2E/ATG/ATG-Flag-1.tif]

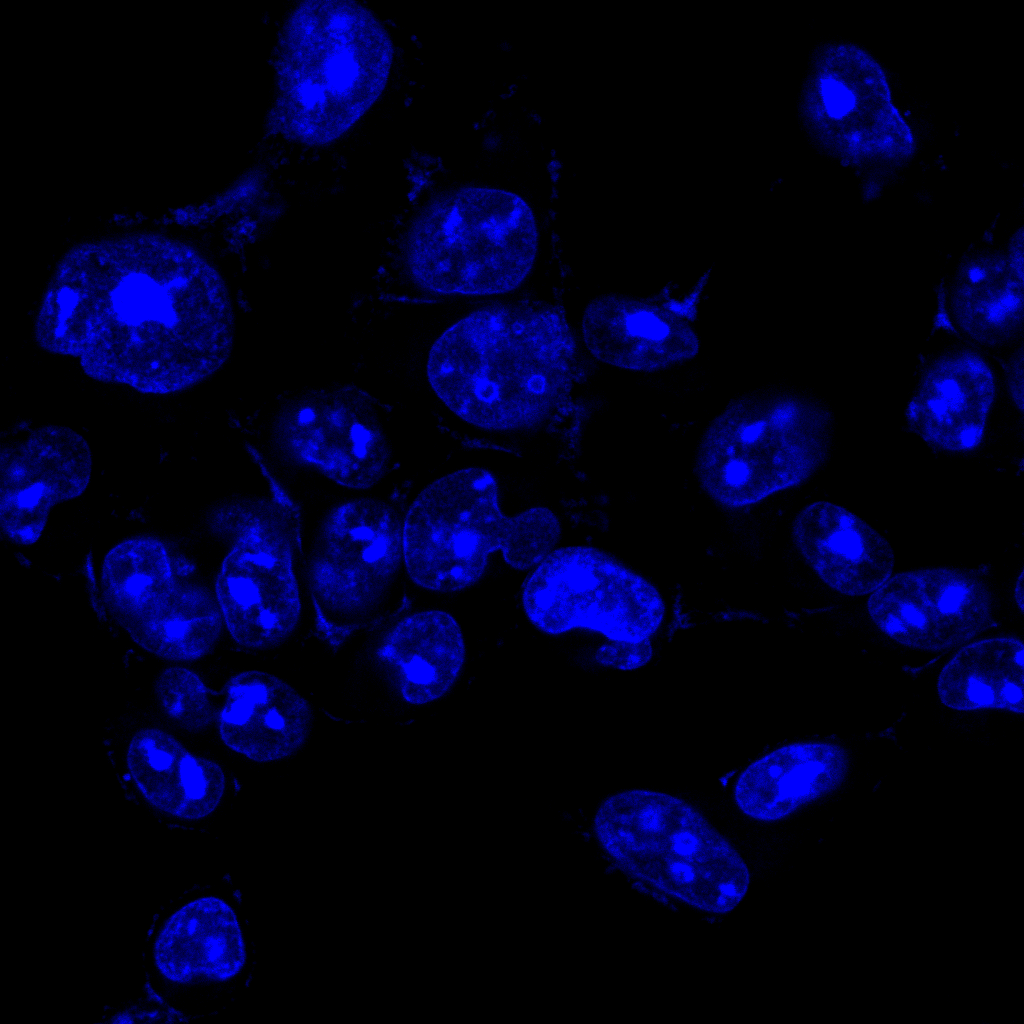

Supplement: Supplementary file 7 — Source data Fig. 2 [file 44318_2024_359_MOESM7_ESM.zip › Figure 2/Fig 2E/ATG/ATG-Hoechst.tif]

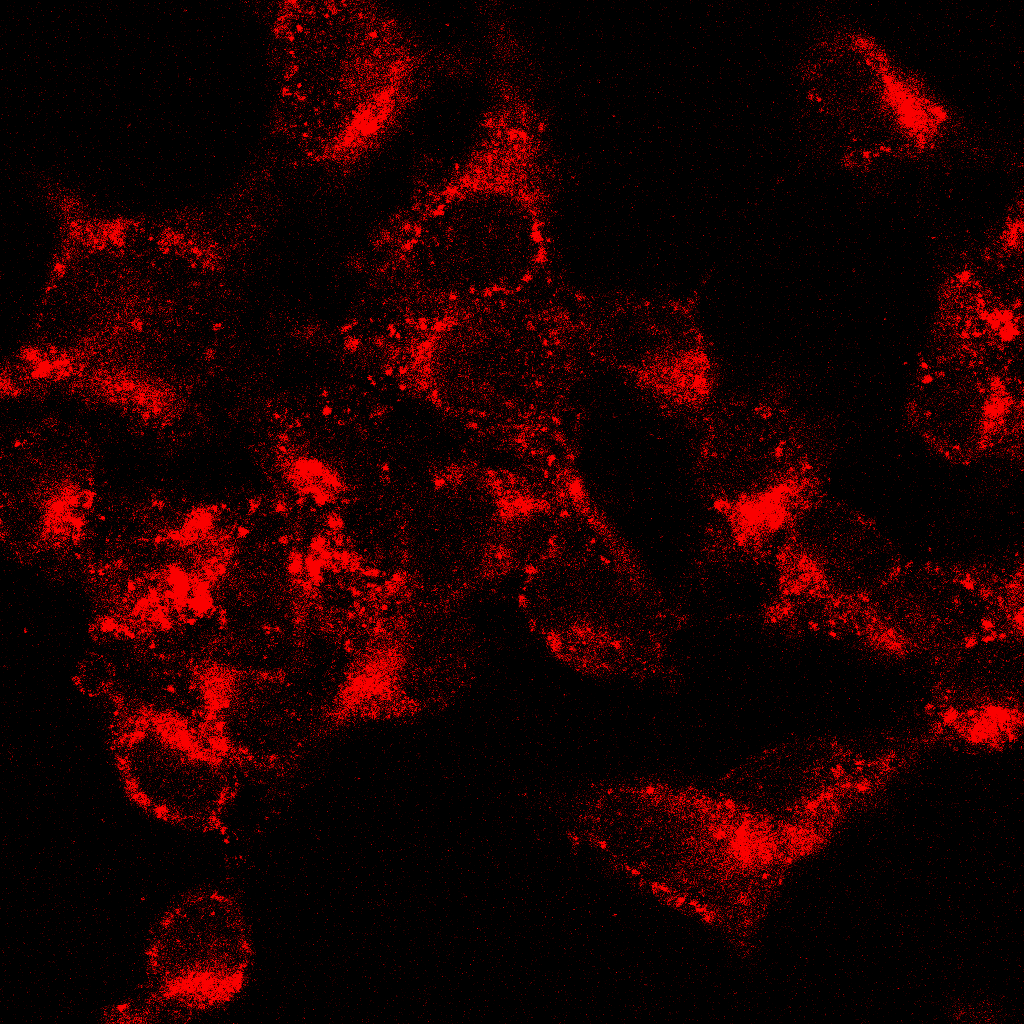

Supplement: Supplementary file 7 — Source data Fig. 2 [file 44318_2024_359_MOESM7_ESM.zip › Figure 2/Fig 2E/ATG/ATG-LAMP1.tif]

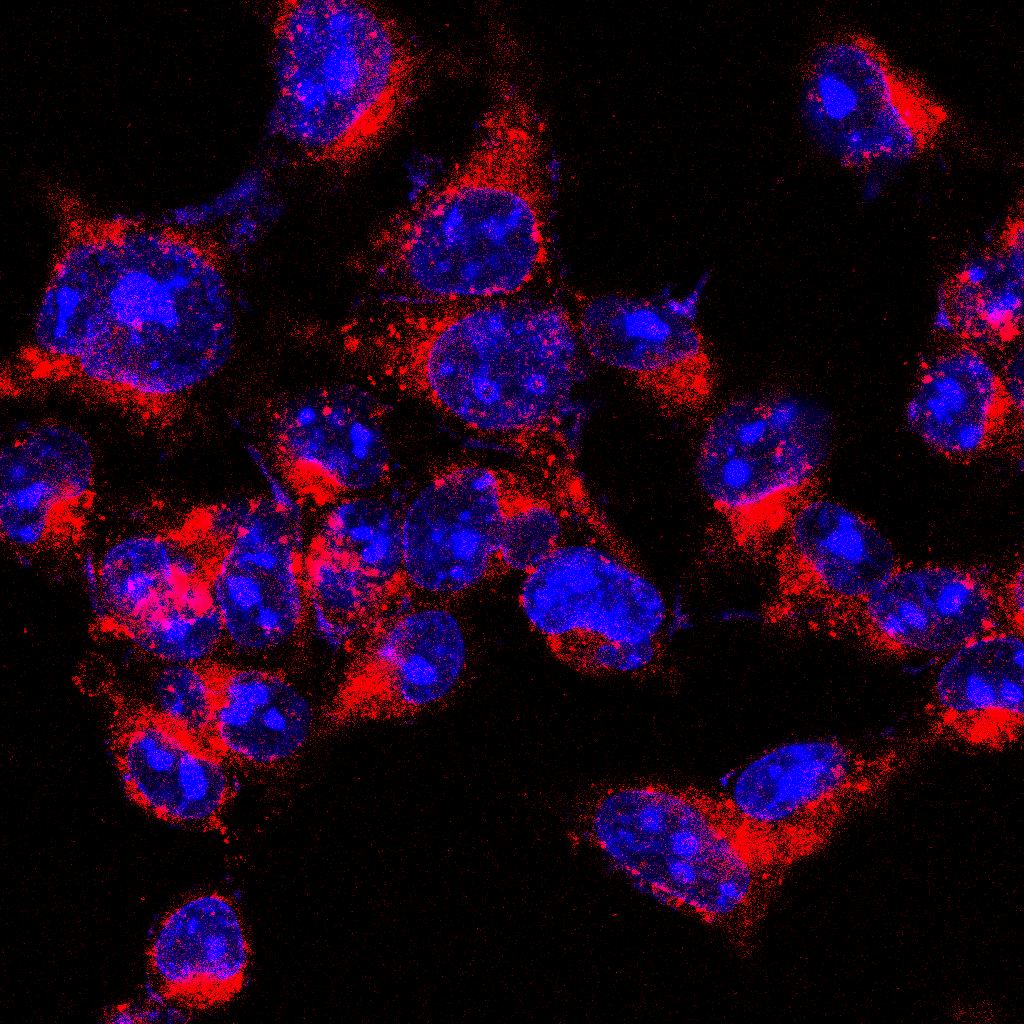

Supplement: Supplementary file 7 — Source data Fig. 2 [file 44318_2024_359_MOESM7_ESM.zip › Figure 2/Fig 2E/ATG/ATG-merge.tif]

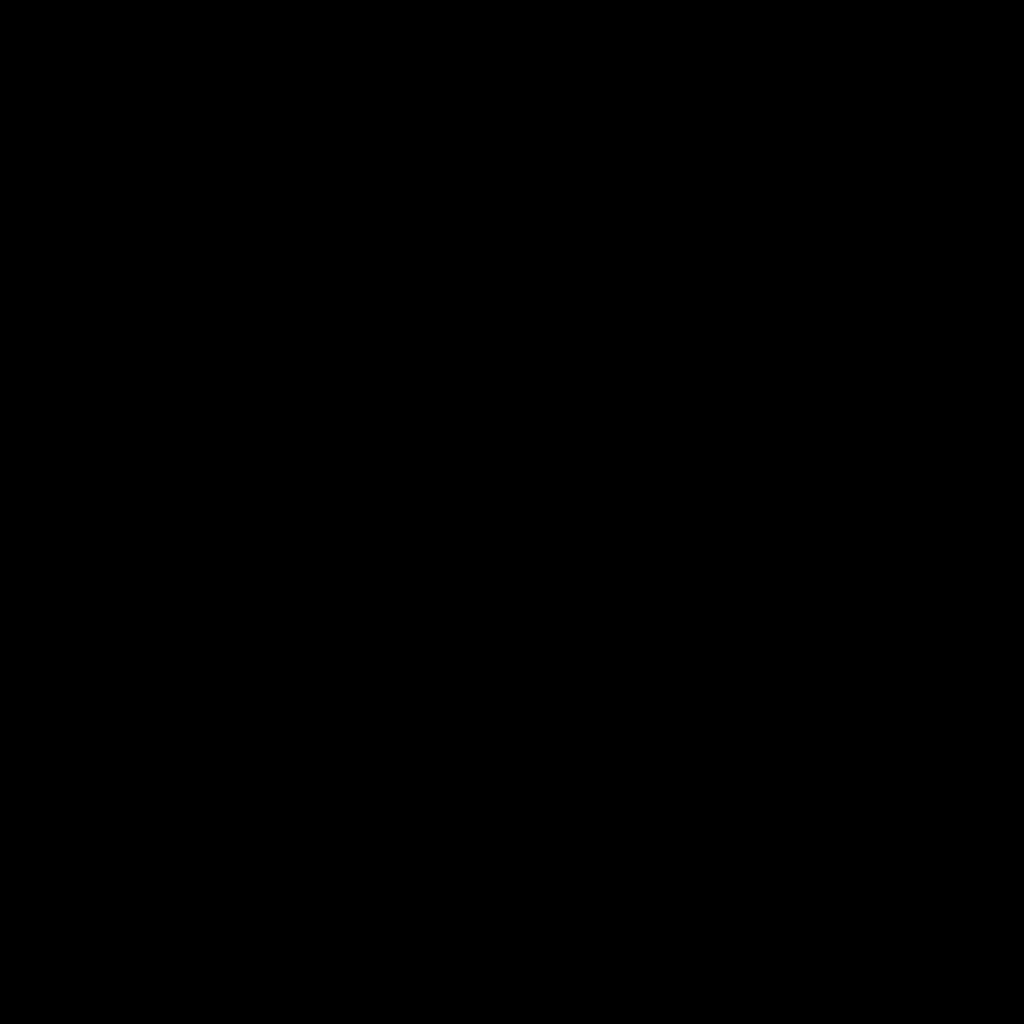

Supplement: Supplementary file 7 — Source data Fig. 2 [file 44318_2024_359_MOESM7_ESM.zip › Figure 2/Fig 2E/Vector Ctrl/Vector Ctrl-Flag.tif]

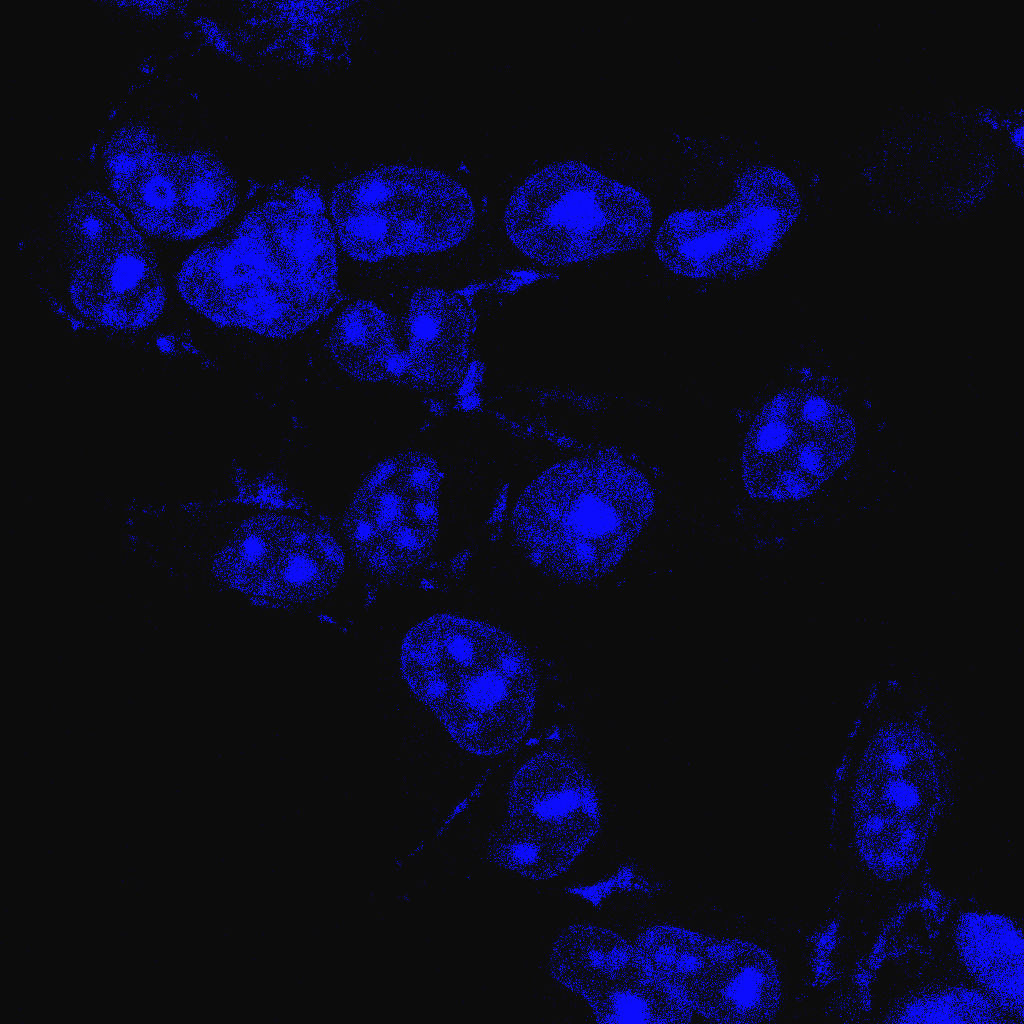

Supplement: Supplementary file 7 — Source data Fig. 2 [file 44318_2024_359_MOESM7_ESM.zip › Figure 2/Fig 2E/Vector Ctrl/Vector Ctrl-Hoechst.tif]

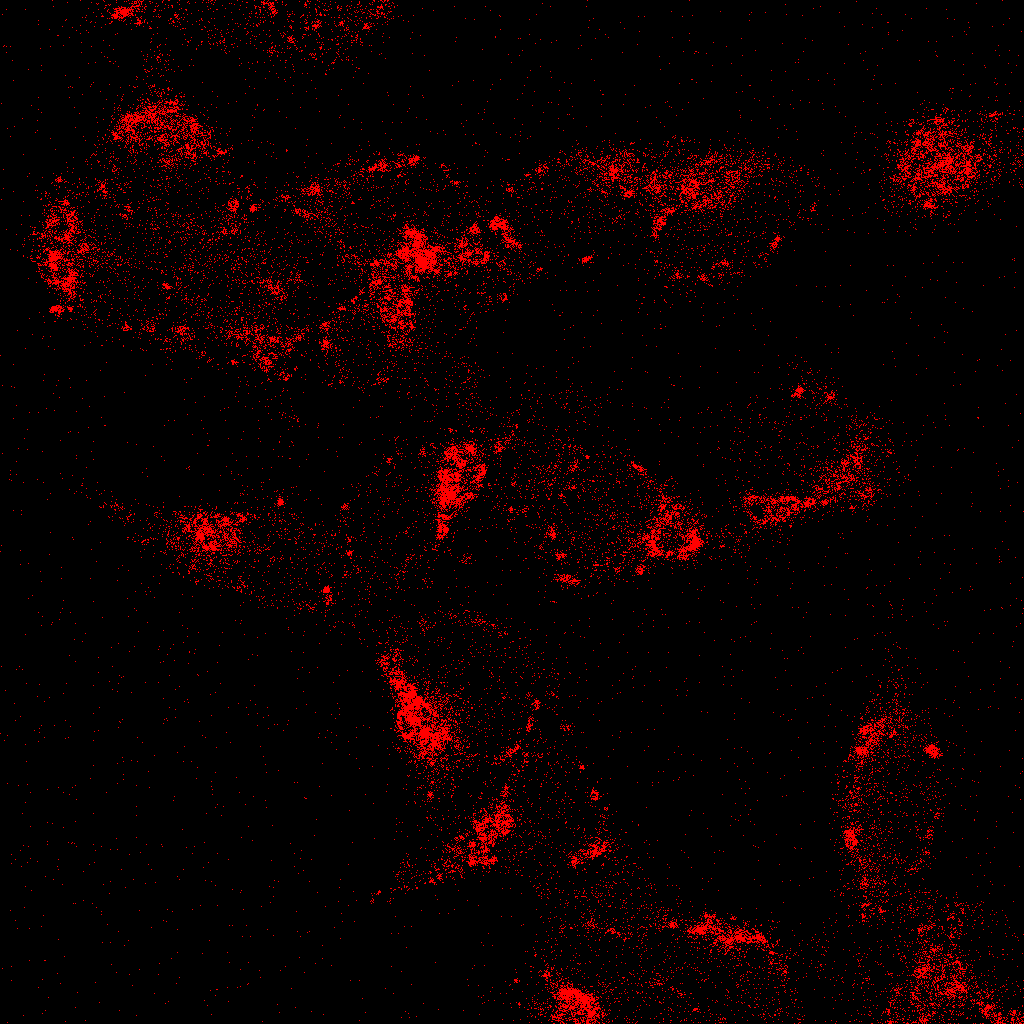

Supplement: Supplementary file 7 — Source data Fig. 2 [file 44318_2024_359_MOESM7_ESM.zip › Figure 2/Fig 2E/Vector Ctrl/Vector Ctrl-LAMP1.tif]

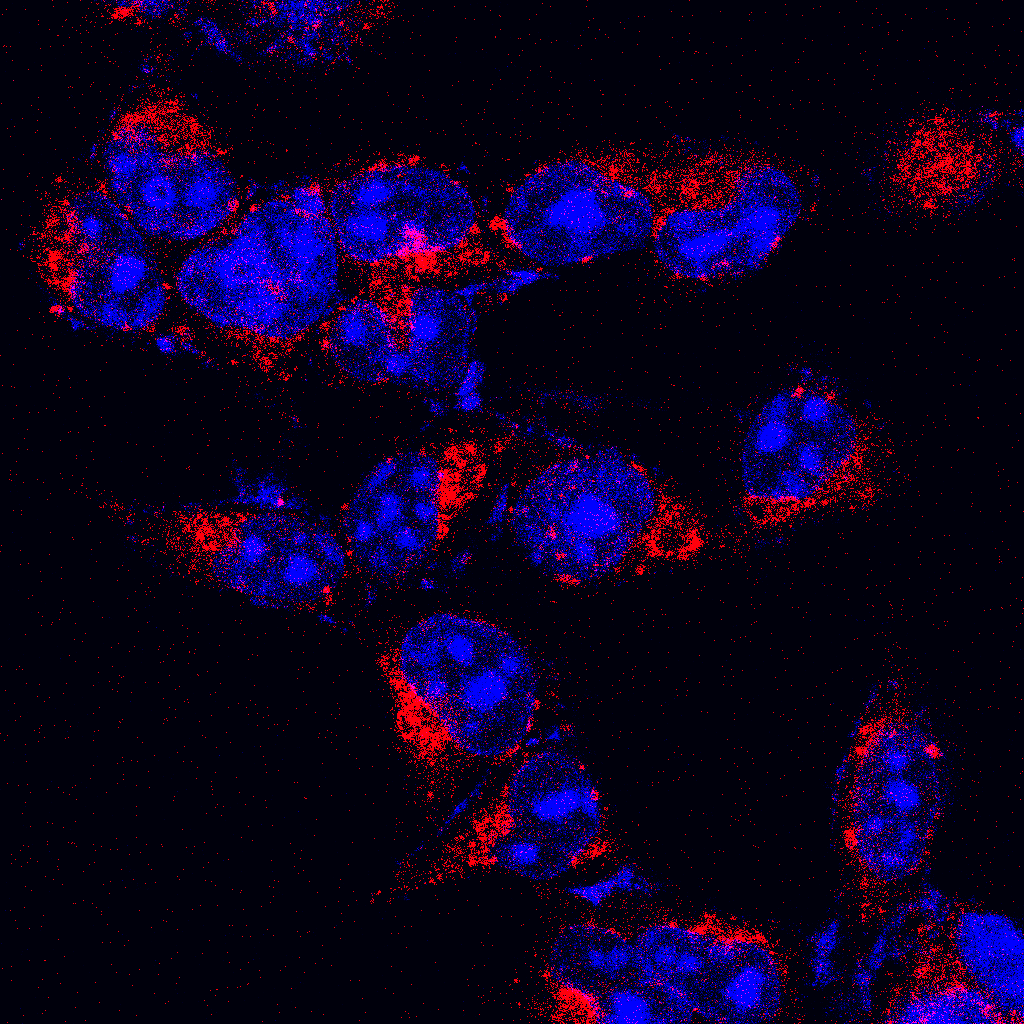

Supplement: Supplementary file 7 — Source data Fig. 2 [file 44318_2024_359_MOESM7_ESM.zip › Figure 2/Fig 2E/Vector Ctrl/Vector Ctrl-merge.tif]

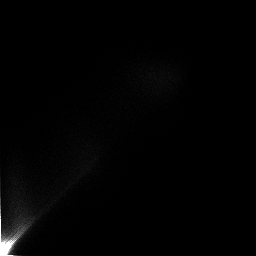

Supplement: Supplementary file 7 — Source data Fig. 2 [file 44318_2024_359_MOESM7_ESM.zip › Figure 2/Fig 2E/hSPAR/Fig 2E Pearson correlation.tif]

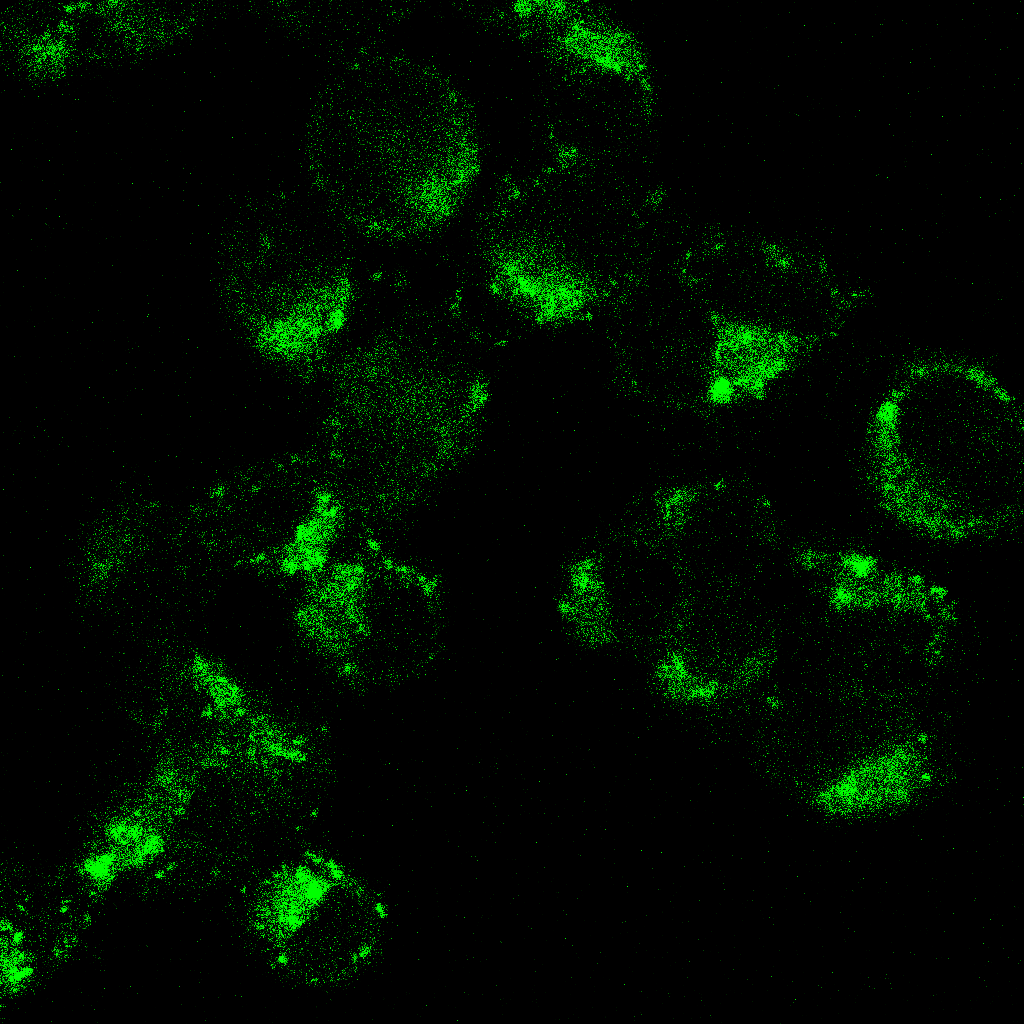

Supplement: Supplementary file 7 — Source data Fig. 2 [file 44318_2024_359_MOESM7_ESM.zip › Figure 2/Fig 2E/hSPAR/hSPAR-Flag.tif]

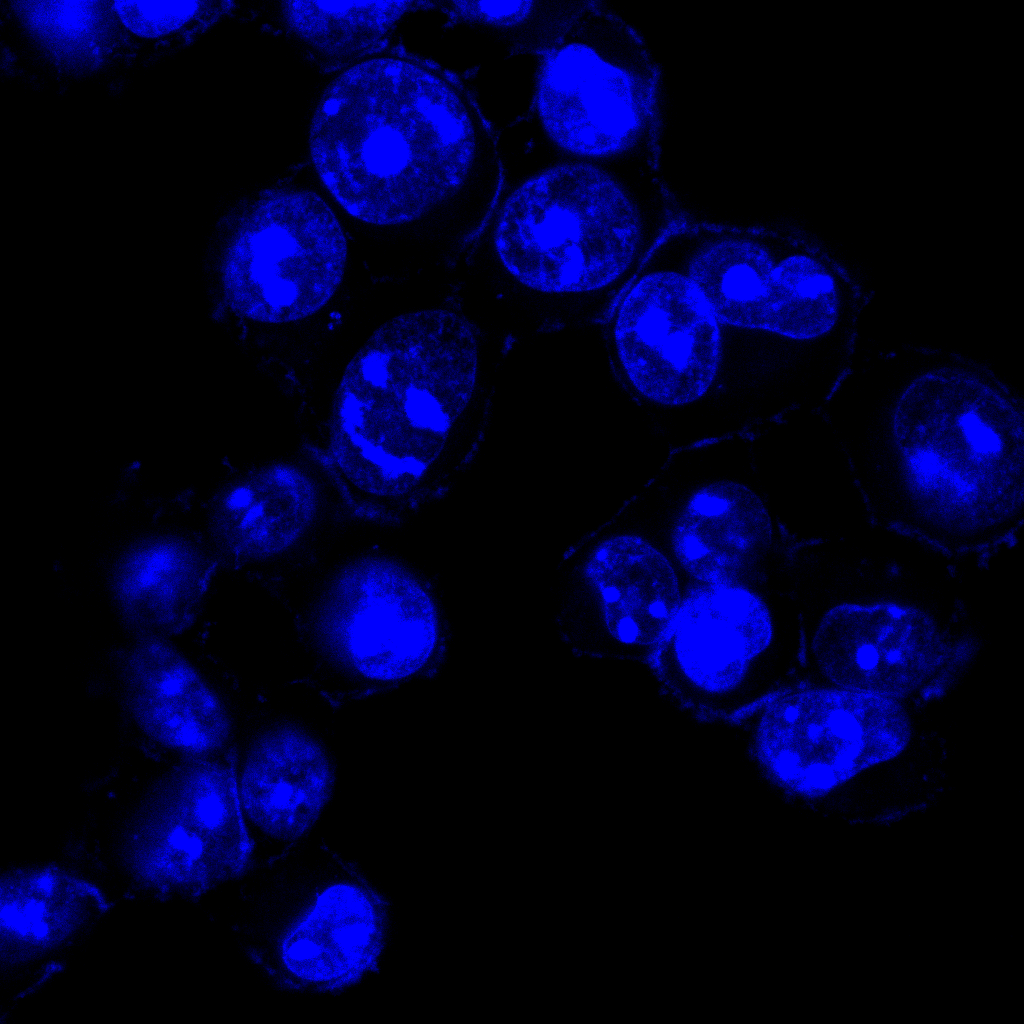

Supplement: Supplementary file 7 — Source data Fig. 2 [file 44318_2024_359_MOESM7_ESM.zip › Figure 2/Fig 2E/hSPAR/hSPAR-Hoechst.tif]

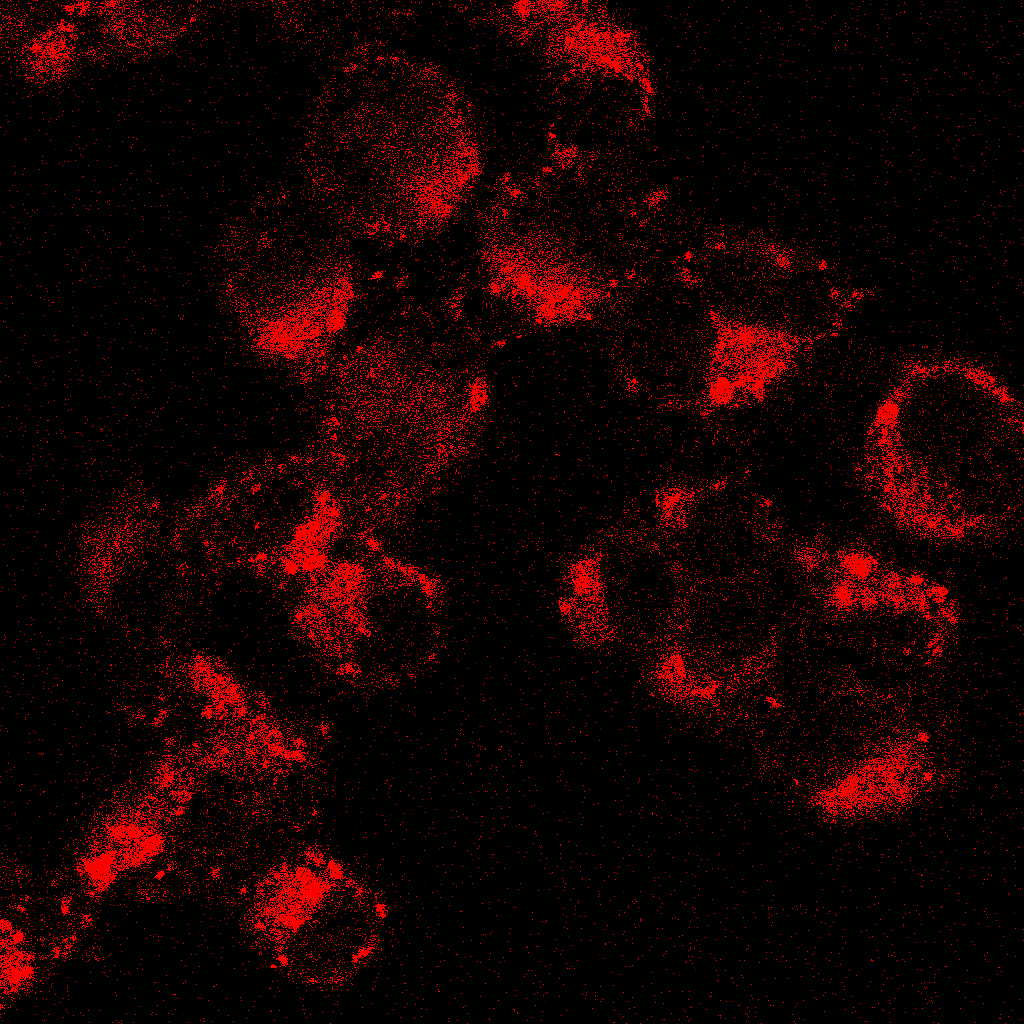

Supplement: Supplementary file 7 — Source data Fig. 2 [file 44318_2024_359_MOESM7_ESM.zip › Figure 2/Fig 2E/hSPAR/hSPAR-LAMP1.tif]

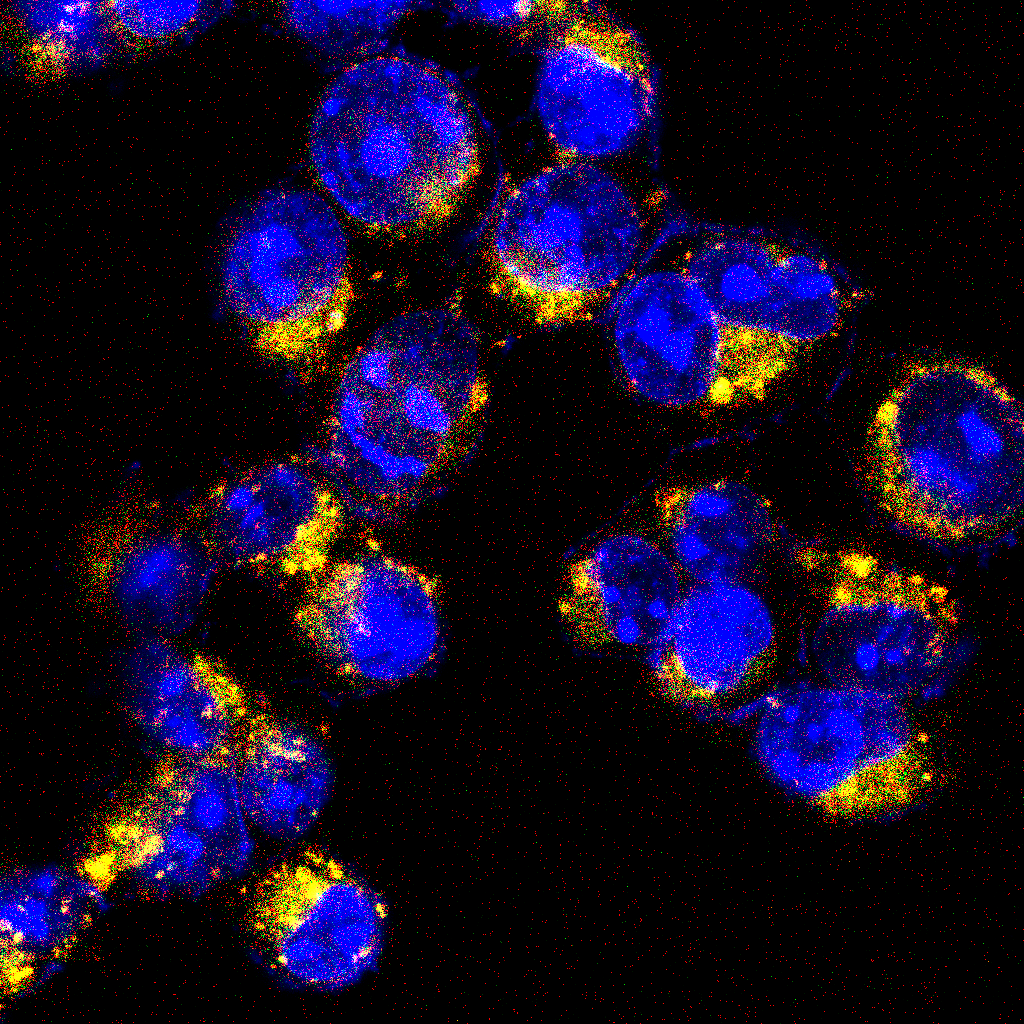

Supplement: Supplementary file 7 — Source data Fig. 2 [file 44318_2024_359_MOESM7_ESM.zip › Figure 2/Fig 2E/hSPAR/hSPAR-merge.tif]

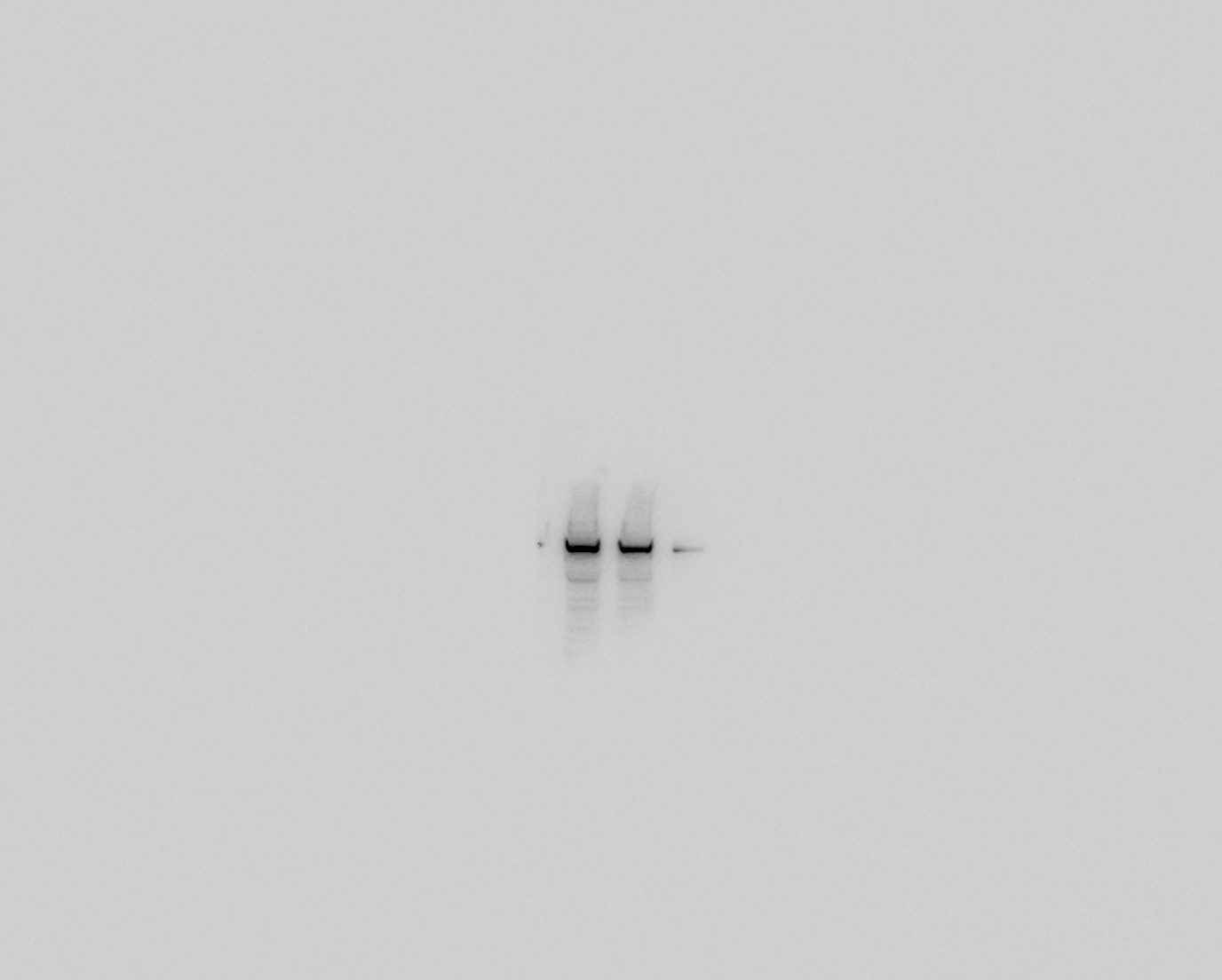

Supplement: Supplementary file 7 — Source data Fig. 2 [file 44318_2024_359_MOESM7_ESM.zip › Figure 2/Fig 2F and 2G/Fig 2F/1-p-mTOR.Tif]

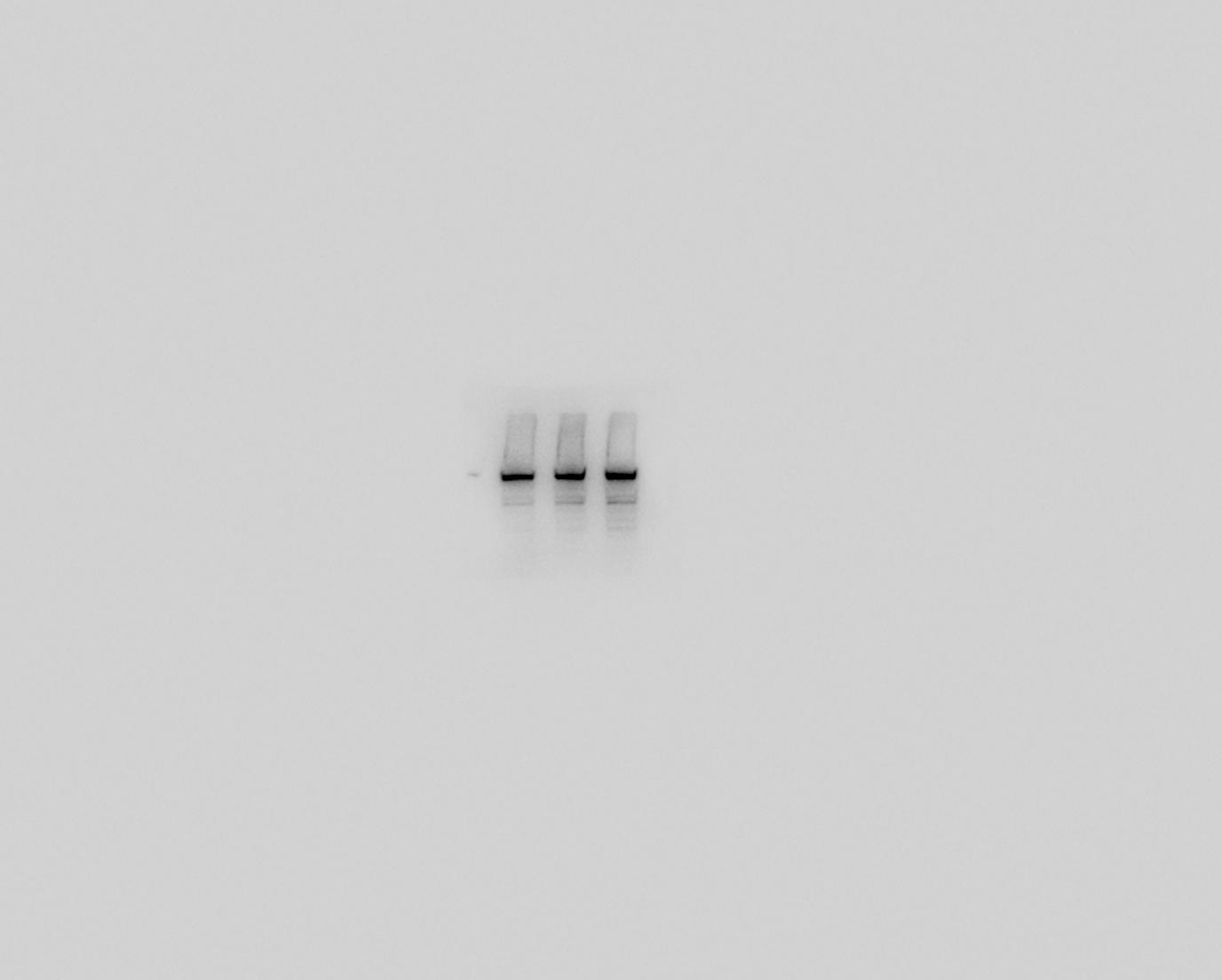

Supplement: Supplementary file 7 — Source data Fig. 2 [file 44318_2024_359_MOESM7_ESM.zip › Figure 2/Fig 2F and 2G/Fig 2F/2-mtor.Tif]

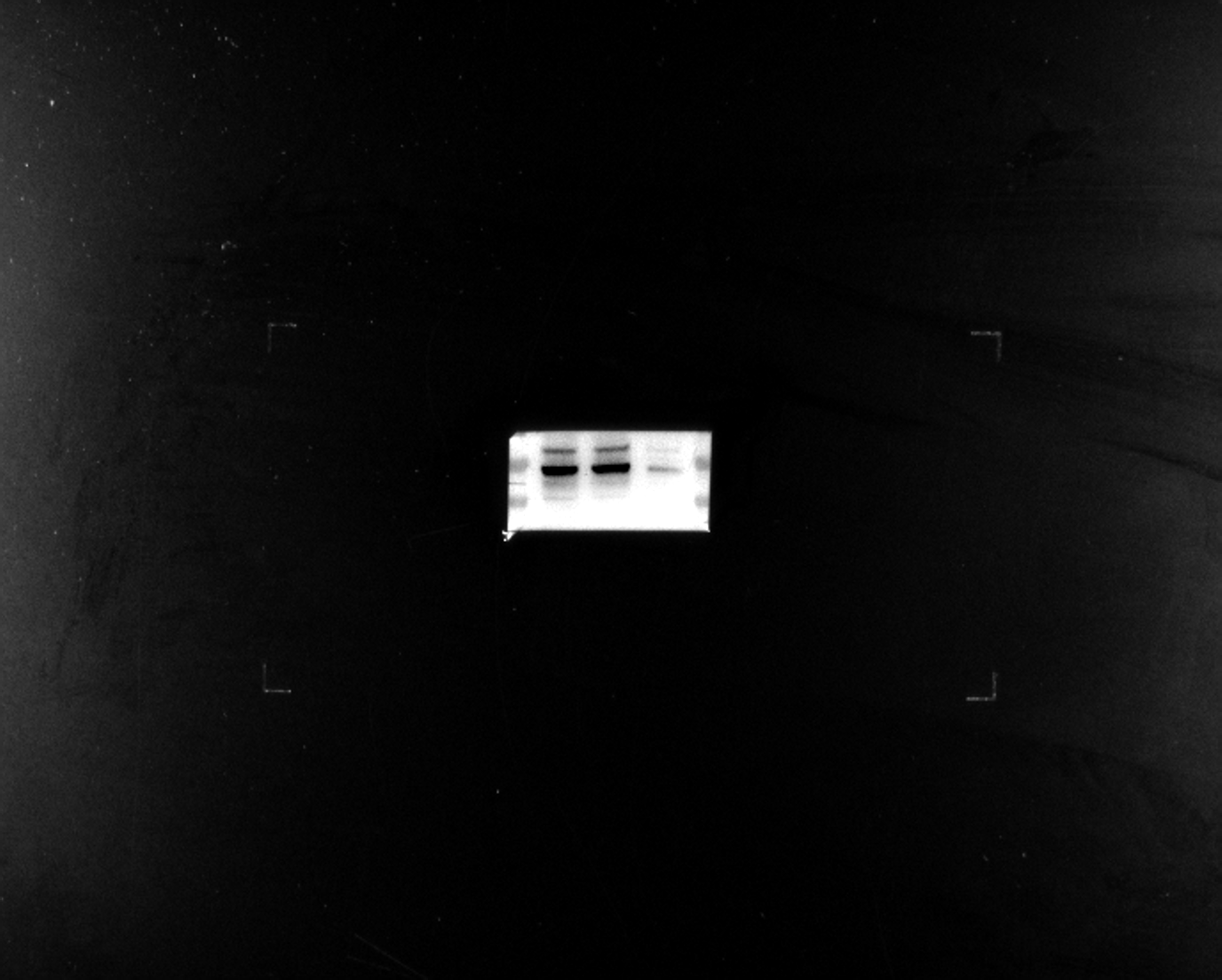

Supplement: Supplementary file 7 — Source data Fig. 2 [file 44318_2024_359_MOESM7_ESM.zip › Figure 2/Fig 2F and 2G/Fig 2F/3-p-S6K-merge.Tif]

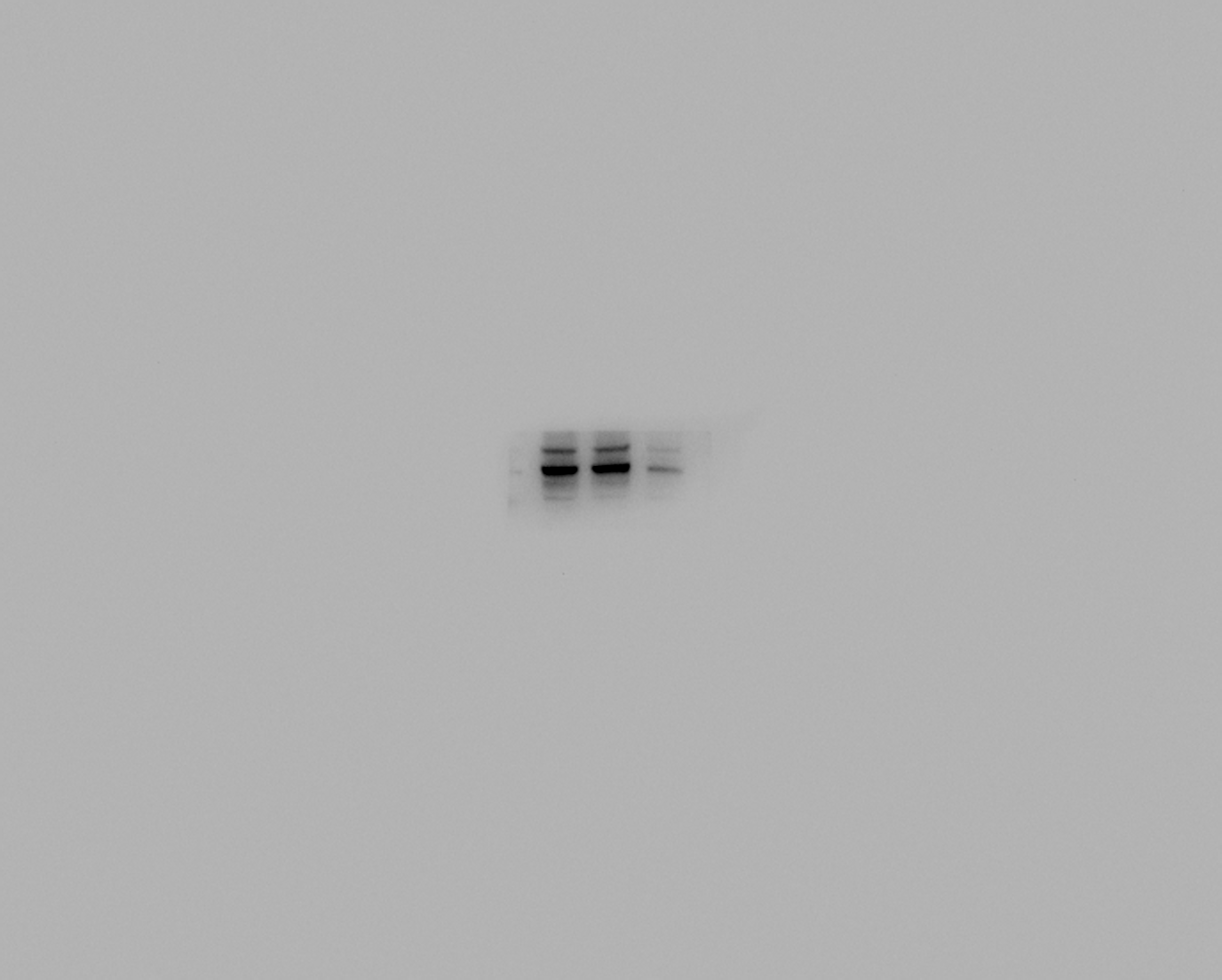

Supplement: Supplementary file 7 — Source data Fig. 2 [file 44318_2024_359_MOESM7_ESM.zip › Figure 2/Fig 2F and 2G/Fig 2F/3-p-s6k.Tif]

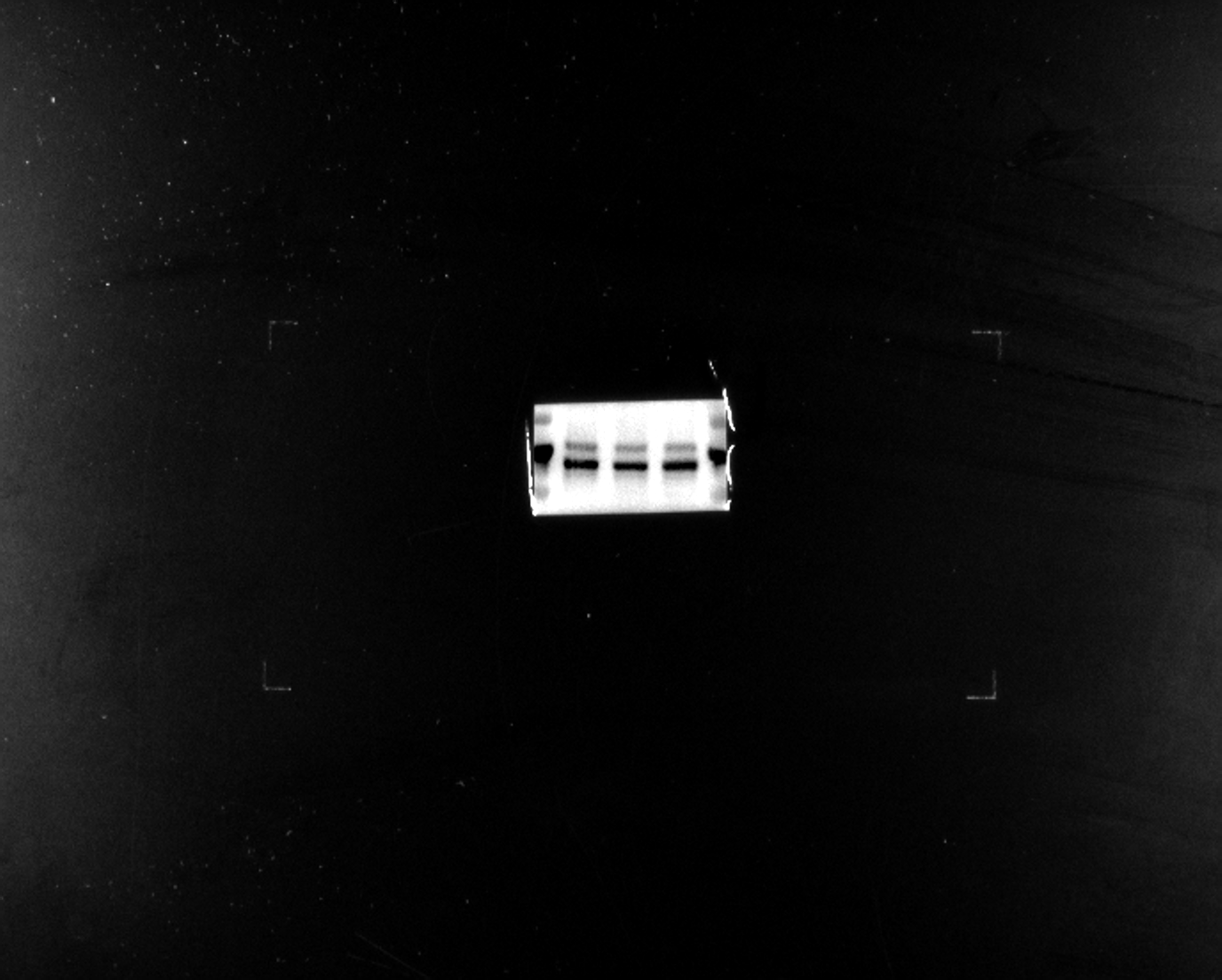

Supplement: Supplementary file 7 — Source data Fig. 2 [file 44318_2024_359_MOESM7_ESM.zip › Figure 2/Fig 2F and 2G/Fig 2F/4-S6K-merge.Tif]

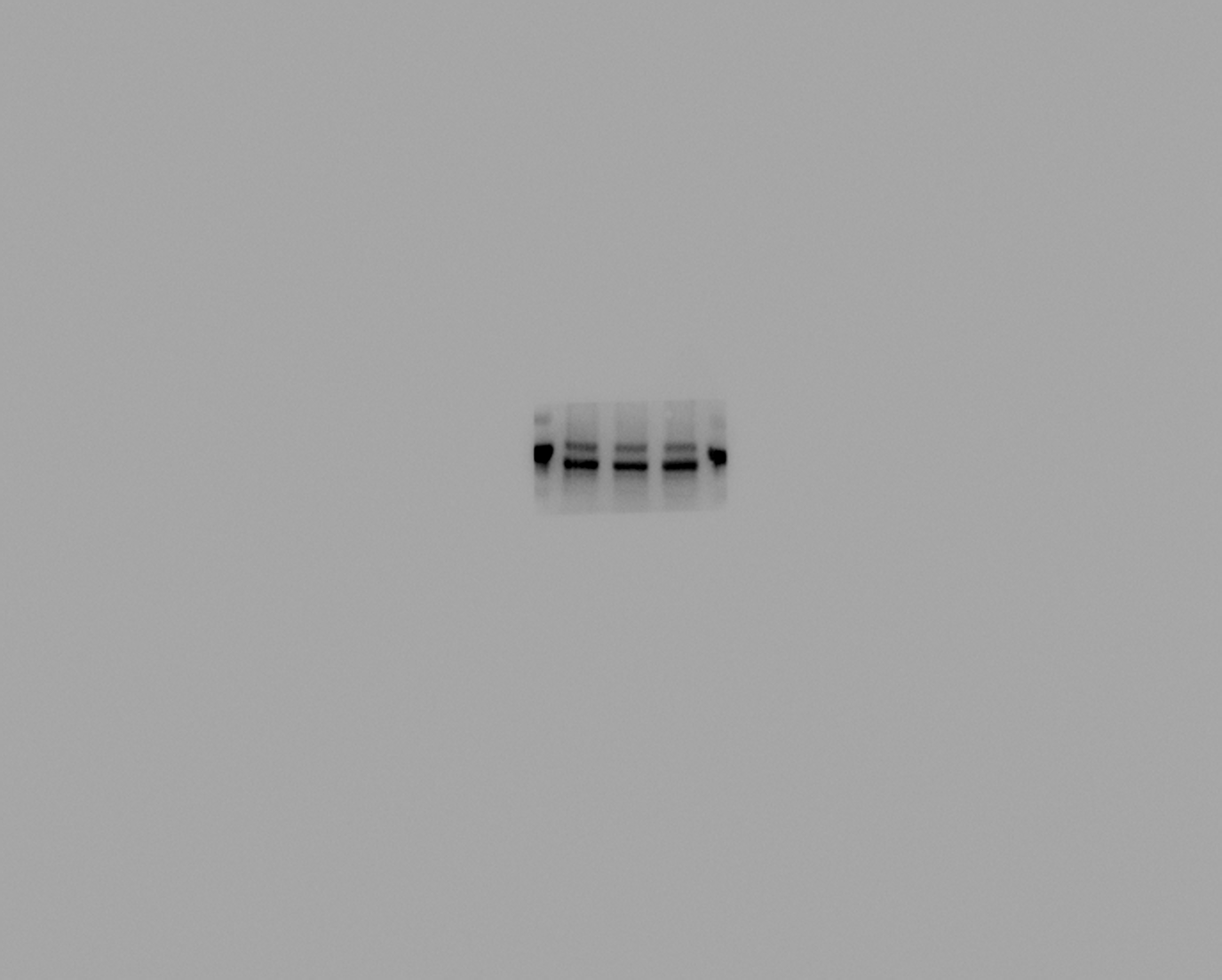

Supplement: Supplementary file 7 — Source data Fig. 2 [file 44318_2024_359_MOESM7_ESM.zip › Figure 2/Fig 2F and 2G/Fig 2F/4-S6K.Tif]

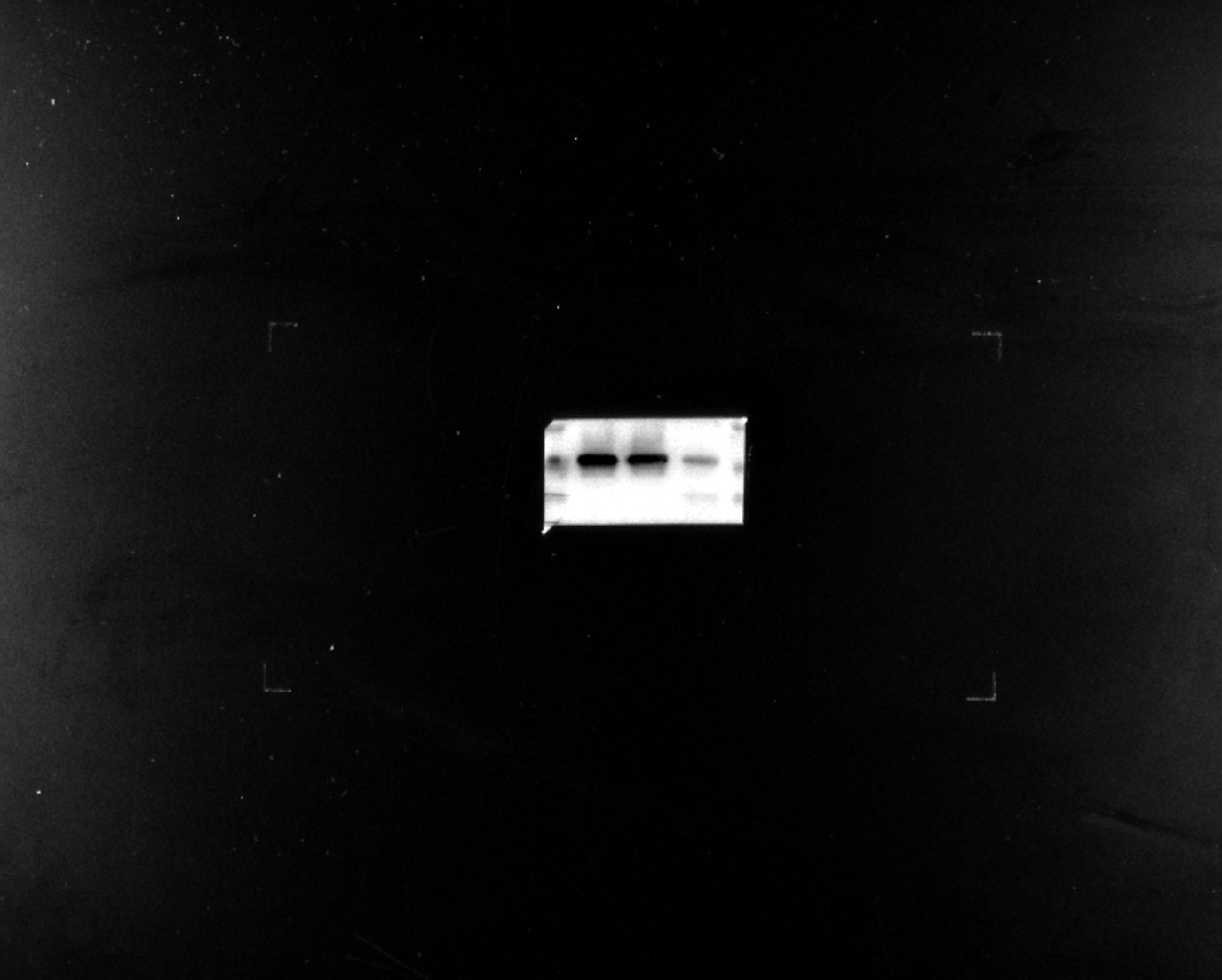

Supplement: Supplementary file 7 — Source data Fig. 2 [file 44318_2024_359_MOESM7_ESM.zip › Figure 2/Fig 2F and 2G/Fig 2F/5-p-S6-merge.Tif]

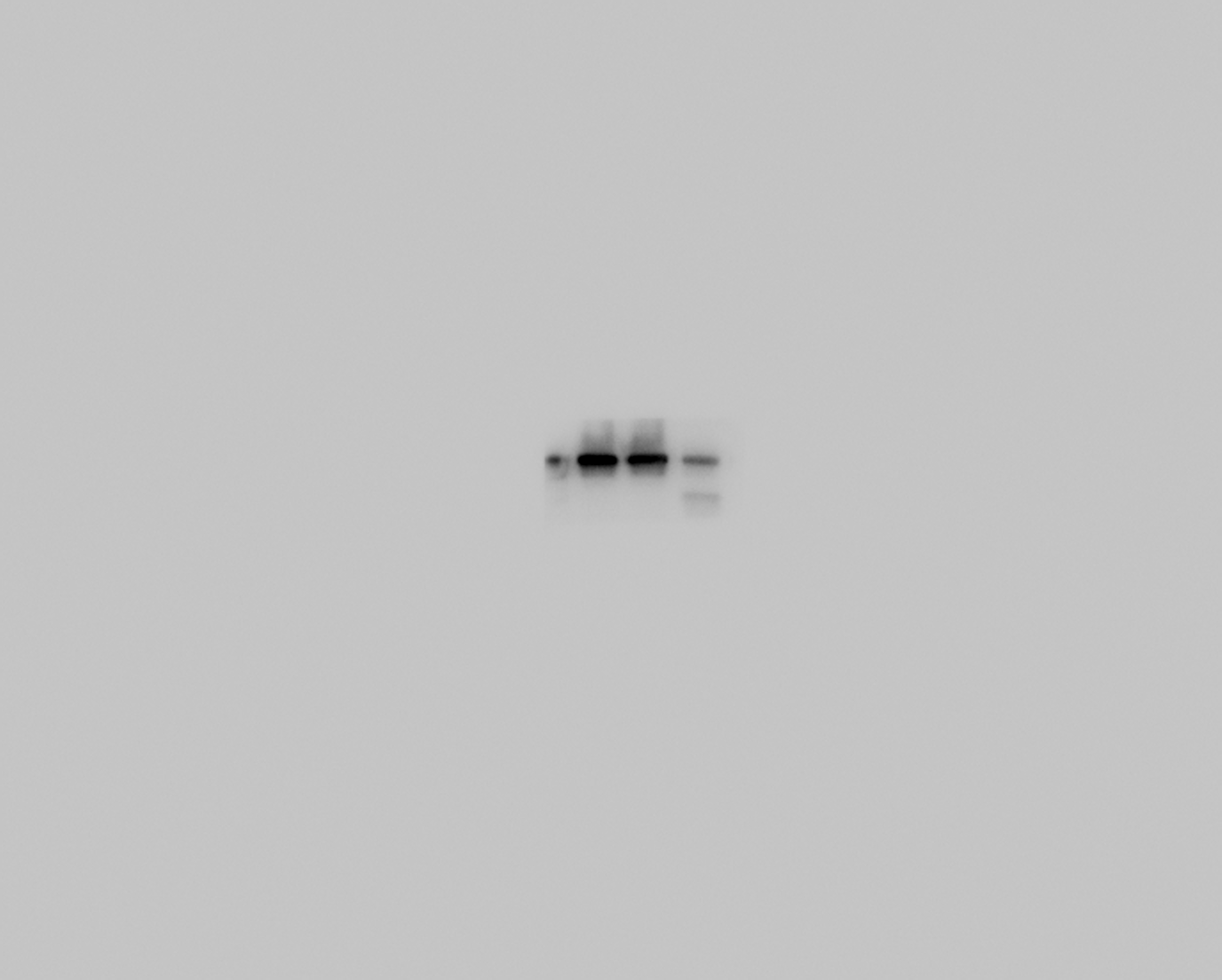

Supplement: Supplementary file 7 — Source data Fig. 2 [file 44318_2024_359_MOESM7_ESM.zip › Figure 2/Fig 2F and 2G/Fig 2F/5-p-S6.Tif]

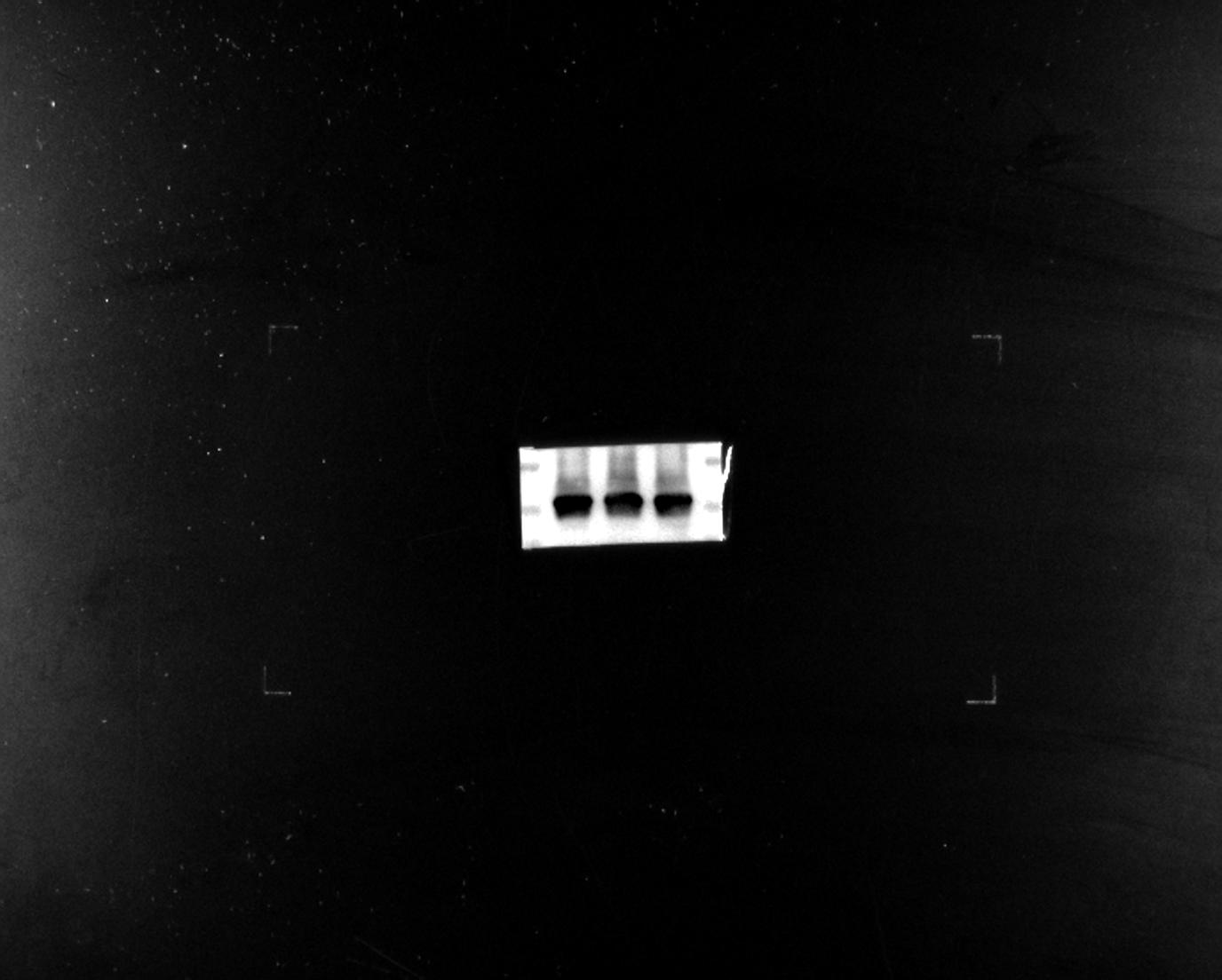

Supplement: Supplementary file 7 — Source data Fig. 2 [file 44318_2024_359_MOESM7_ESM.zip › Figure 2/Fig 2F and 2G/Fig 2F/6-S6-merge.Tif]

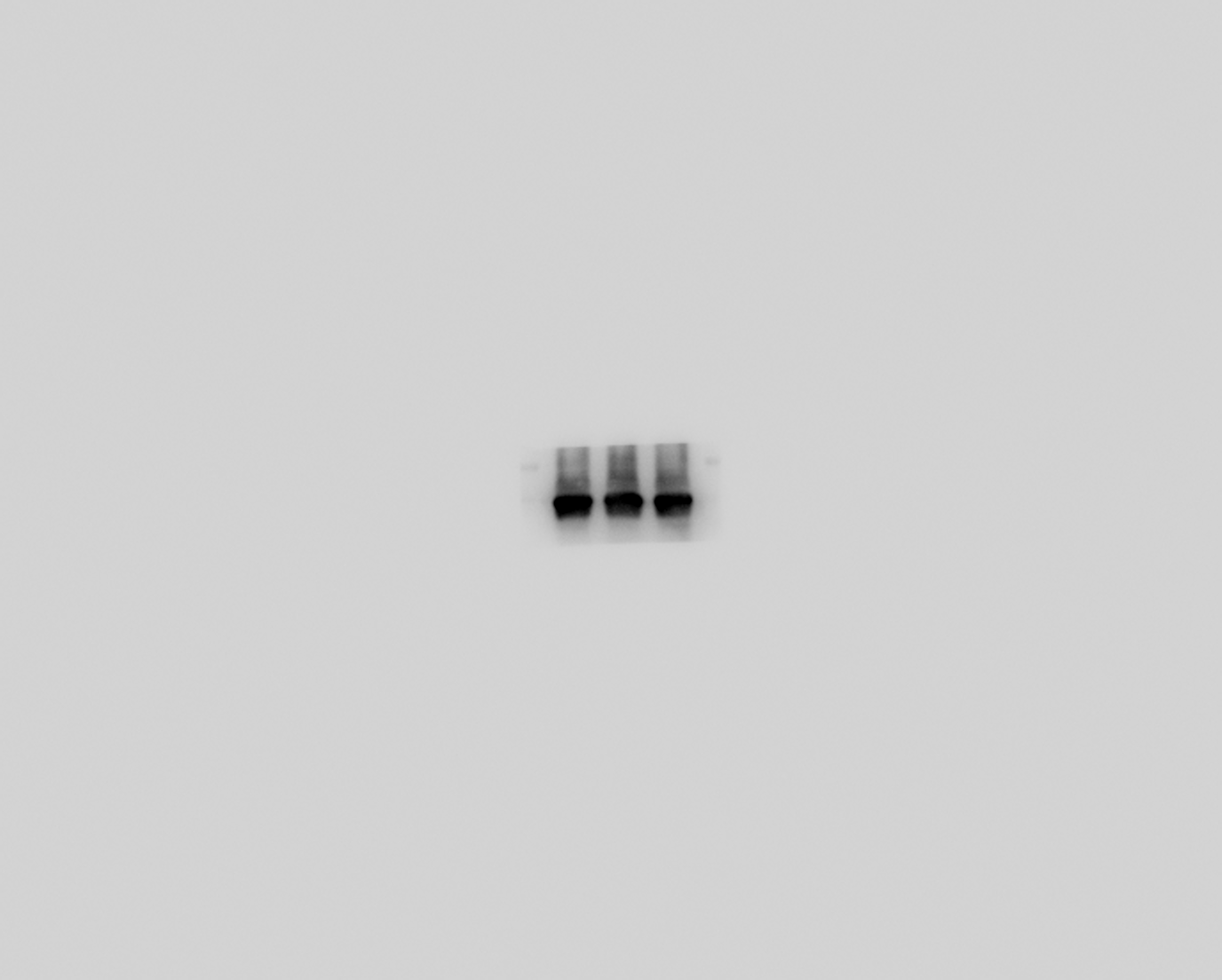

Supplement: Supplementary file 7 — Source data Fig. 2 [file 44318_2024_359_MOESM7_ESM.zip › Figure 2/Fig 2F and 2G/Fig 2F/6-S6.Tif]

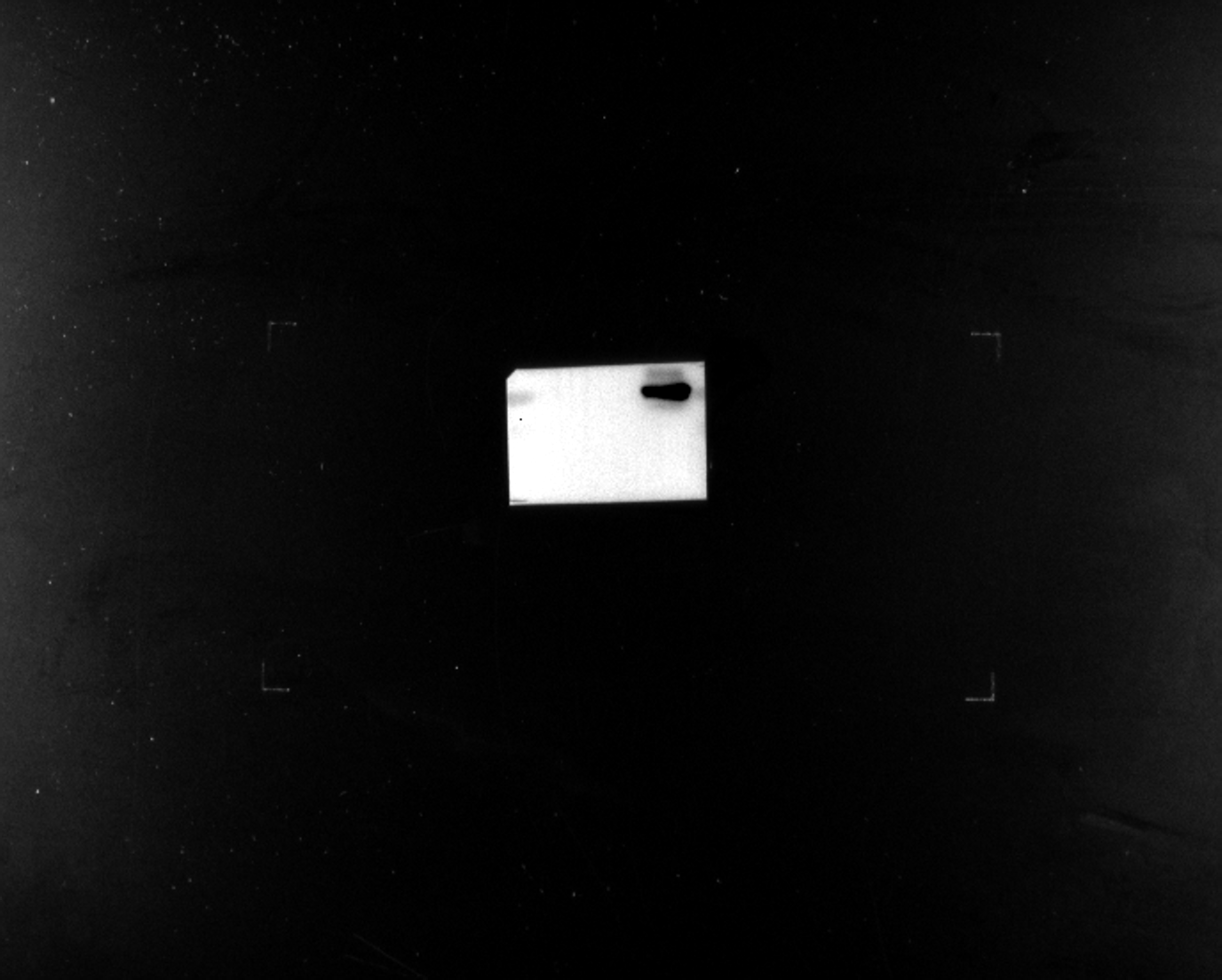

Supplement: Supplementary file 7 — Source data Fig. 2 [file 44318_2024_359_MOESM7_ESM.zip › Figure 2/Fig 2F and 2G/Fig 2F/7-Flag-merge.Tif]

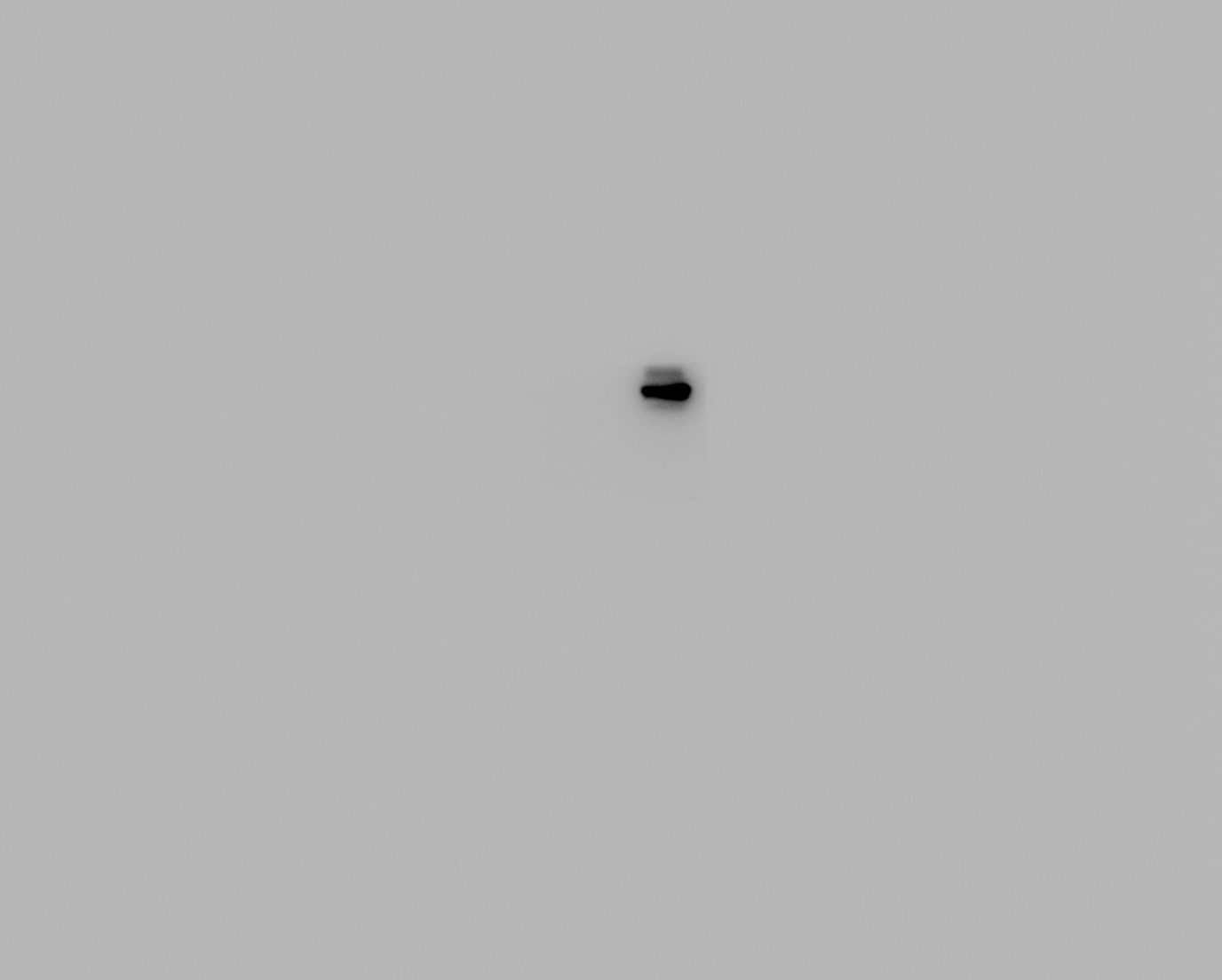

Supplement: Supplementary file 7 — Source data Fig. 2 [file 44318_2024_359_MOESM7_ESM.zip › Figure 2/Fig 2F and 2G/Fig 2F/7-Flag.Tif]

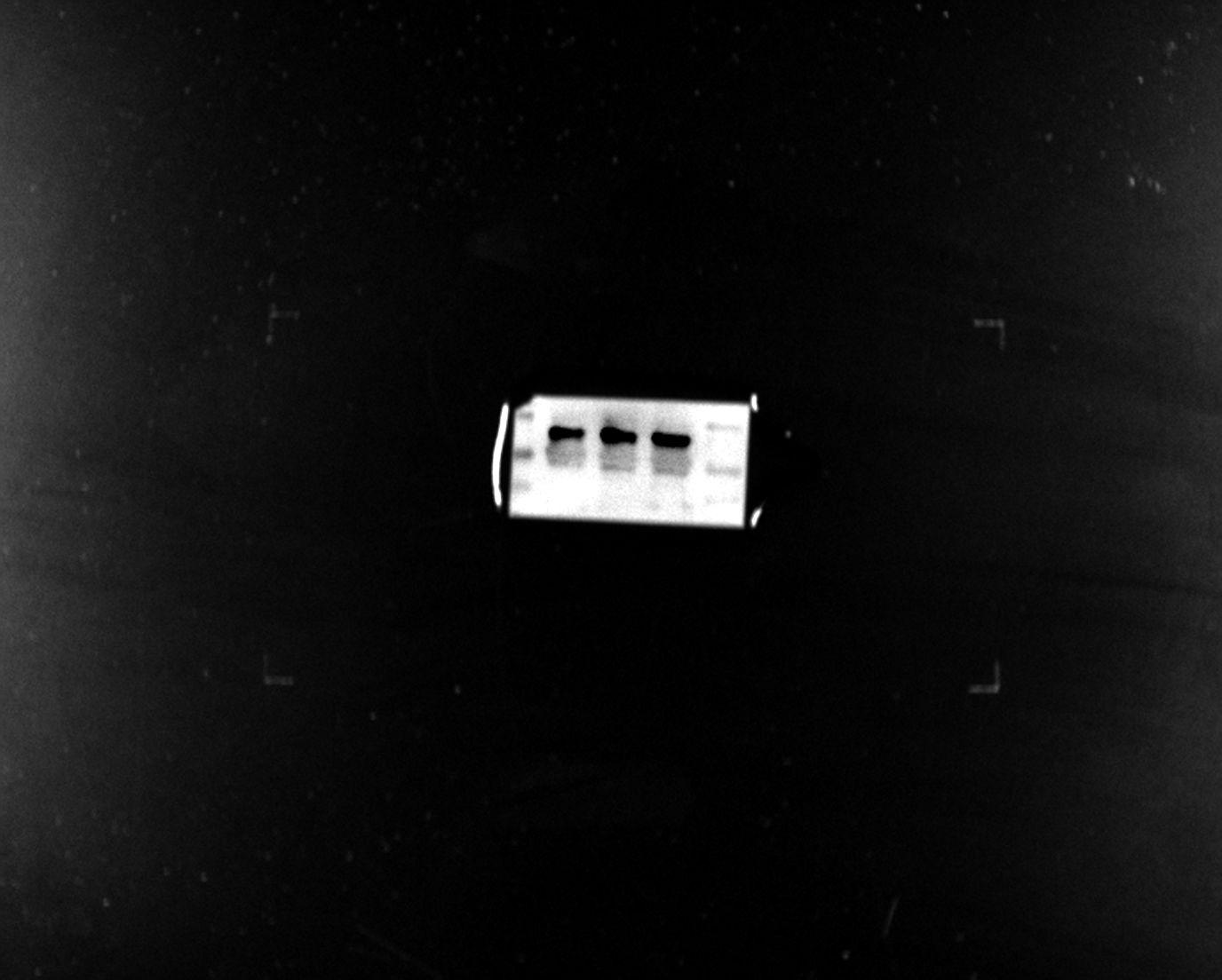

Supplement: Supplementary file 7 — Source data Fig. 2 [file 44318_2024_359_MOESM7_ESM.zip › Figure 2/Fig 2F and 2G/Fig 2F/8-GAPDH-merge.Tif]

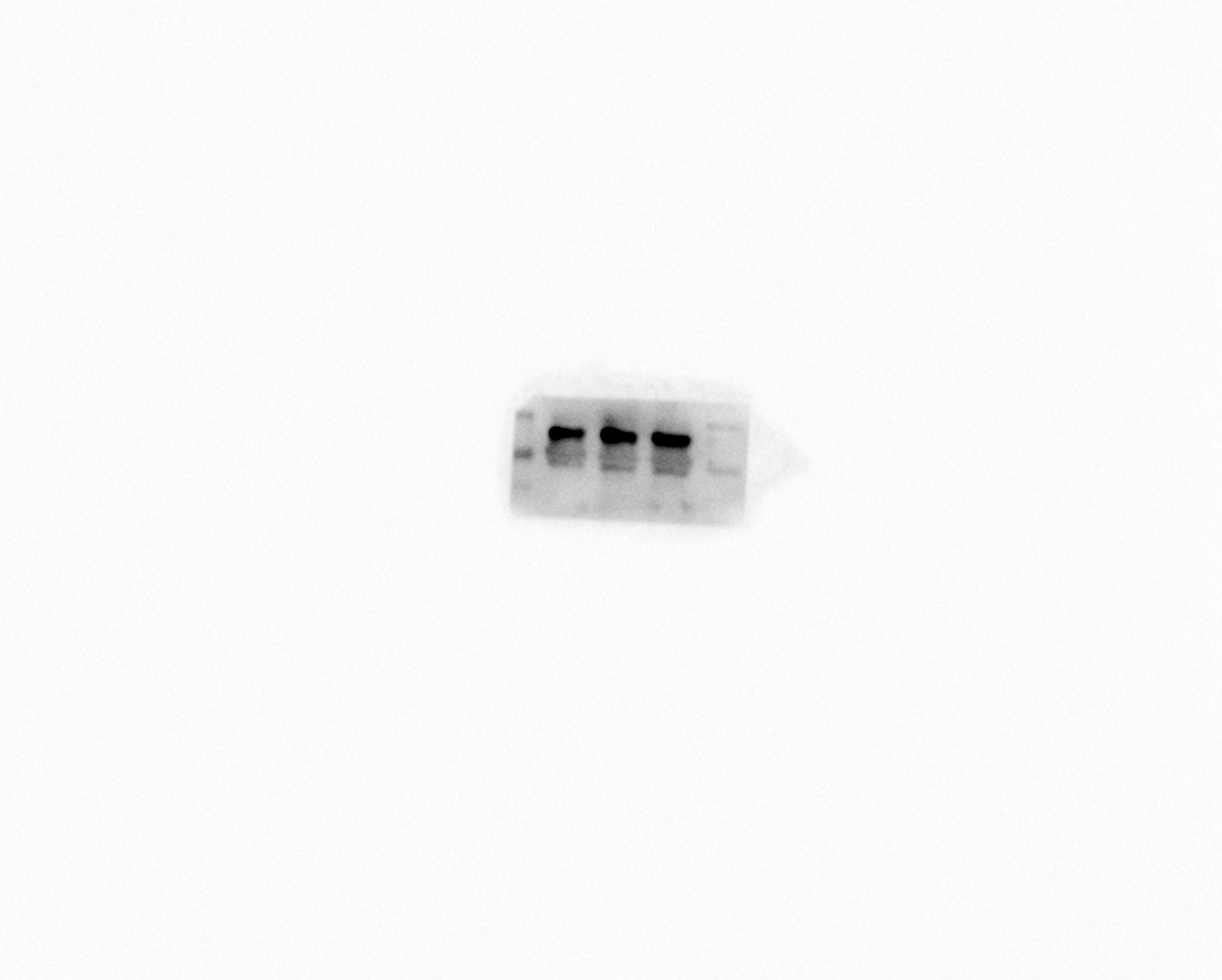

Supplement: Supplementary file 7 — Source data Fig. 2 [file 44318_2024_359_MOESM7_ESM.zip › Figure 2/Fig 2F and 2G/Fig 2F/8-GAPDH.Tif]

**Fig 2F**

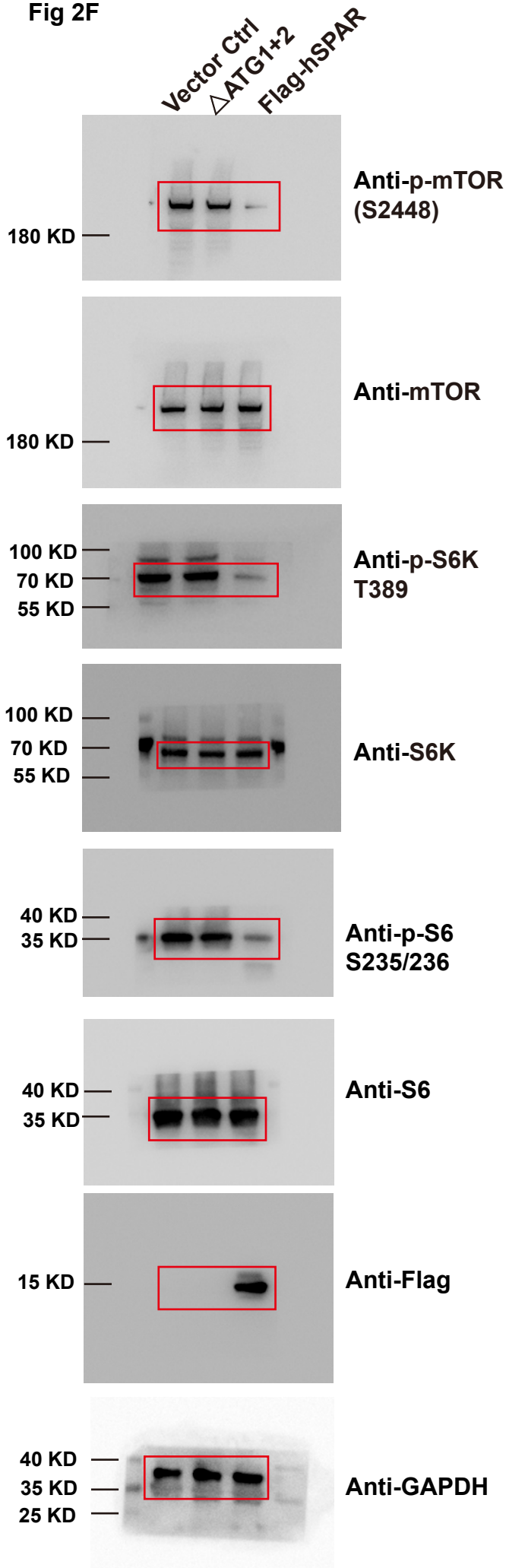

Supplement: Supplementary file 7 — Source data Fig. 2 [file 44318_2024_359_MOESM7_ESM.zip › Figure 2/Fig 2F and 2G/Fig 2F/Fig 2F.pdf]

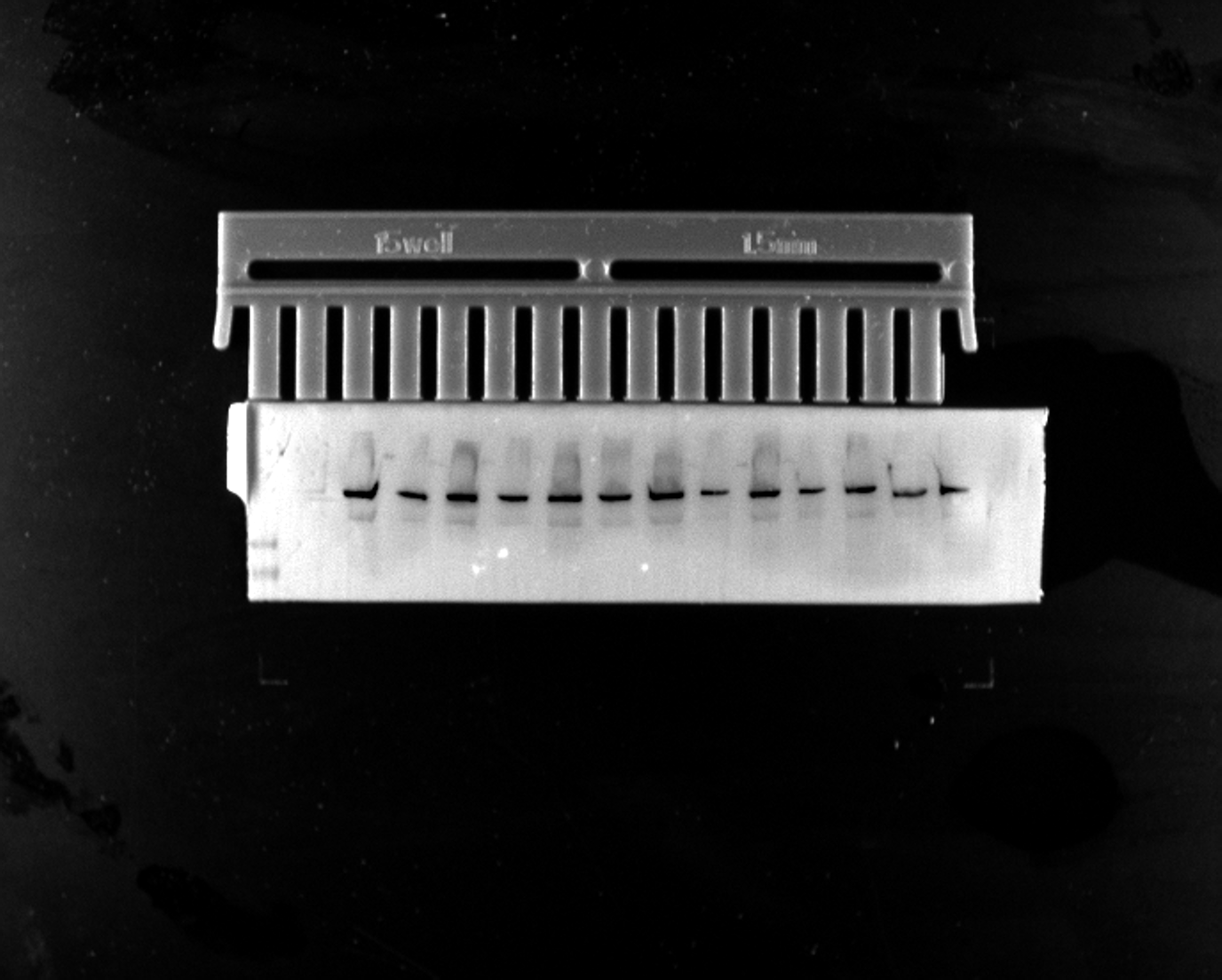

Supplement: Supplementary file 7 — Source data Fig. 2 [file 44318_2024_359_MOESM7_ESM.zip › Figure 2/Fig 2H and 2I/Fig 2H/(#1-#7) Triple-negative/1-p-mTOR-merge.Tif]

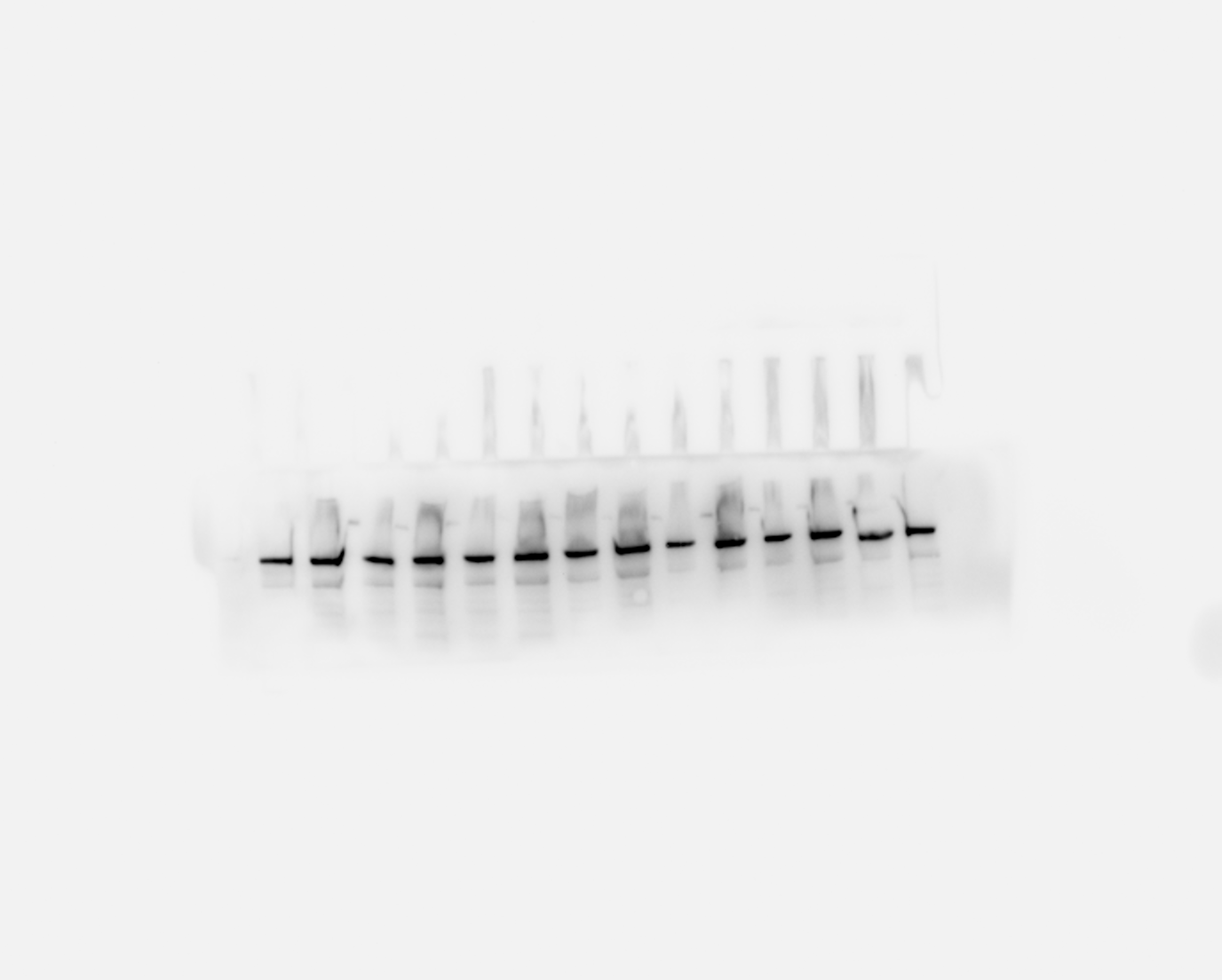

Supplement: Supplementary file 7 — Source data Fig. 2 [file 44318_2024_359_MOESM7_ESM.zip › Figure 2/Fig 2H and 2I/Fig 2H/(#1-#7) Triple-negative/1-p-mTOR.Tif]
