# Supplementary figures and images for: Micropeptide hSPAR regulates glutamine levels and suppresses mammary tumor growth via a TRIM21-P27KIP1-mTOR axis (part 2 of 7)
Source: EMBO J. 2025 Jan 28;44(5):1414–41. doi: 10.1038/s44318-024-00359-z (PMC11876615; doi:10.1038/s44318-024-00359-z)

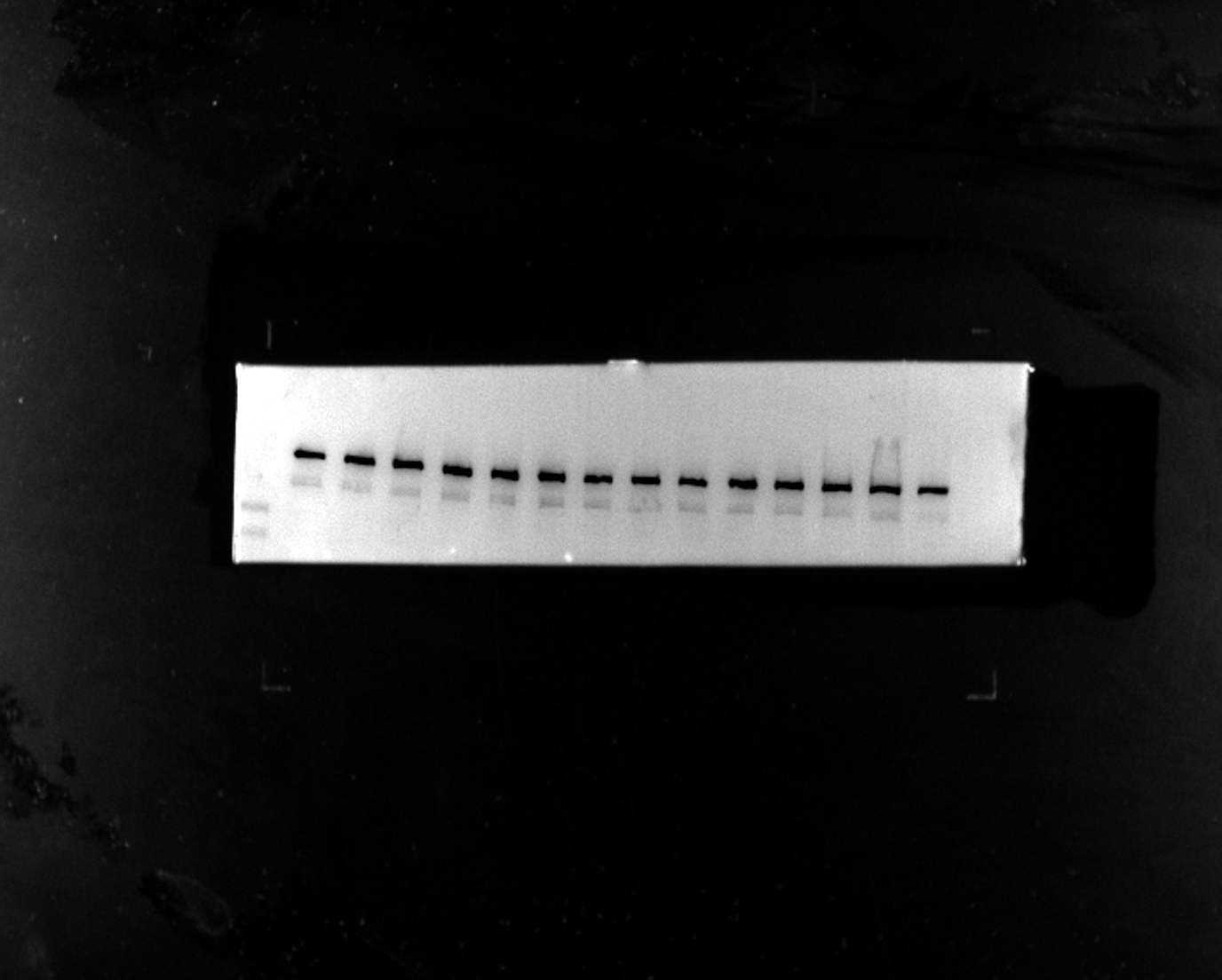

Supplement: Supplementary file 7 — Source data Fig. 2 [file 44318_2024_359_MOESM7_ESM.zip › Figure 2/Fig 2H and 2I/Fig 2H/(#1-#7) Triple-negative/2-mTOR-merge.Tif]

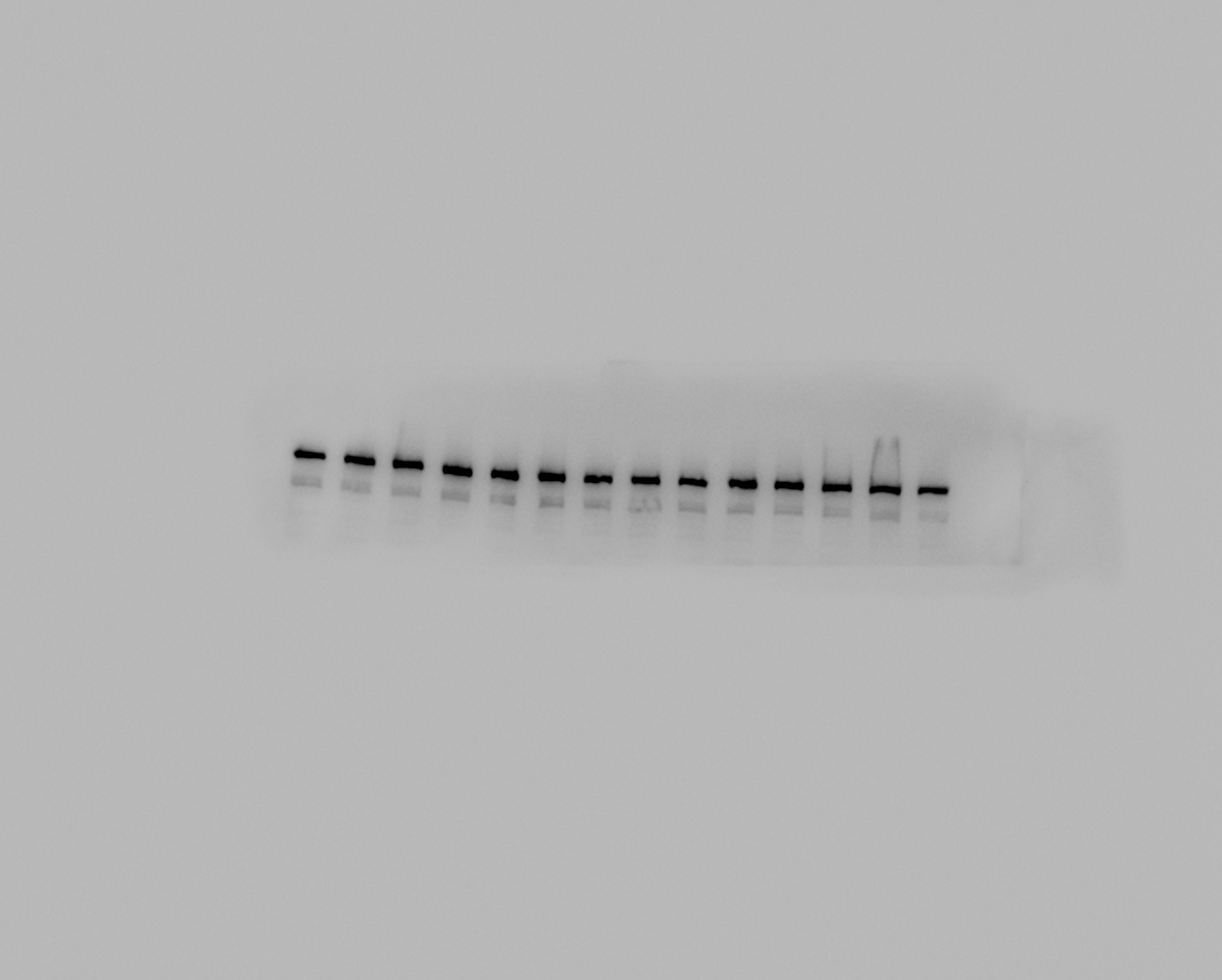

Supplement: Supplementary file 7 — Source data Fig. 2 [file 44318_2024_359_MOESM7_ESM.zip › Figure 2/Fig 2H and 2I/Fig 2H/(#1-#7) Triple-negative/2-mTOR.Tif]

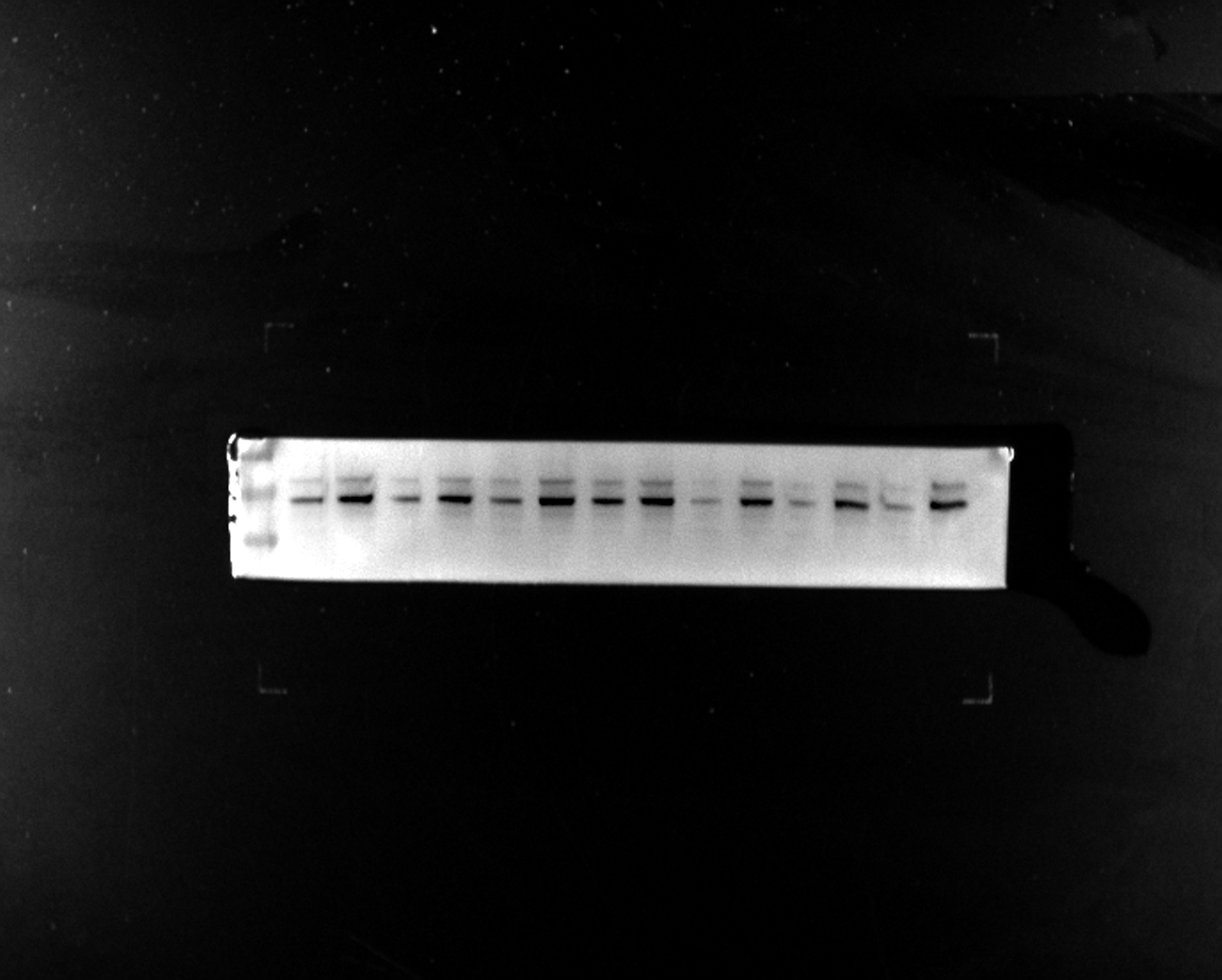

Supplement: Supplementary file 7 — Source data Fig. 2 [file 44318_2024_359_MOESM7_ESM.zip › Figure 2/Fig 2H and 2I/Fig 2H/(#1-#7) Triple-negative/3-p-S6K-merge.Tif]

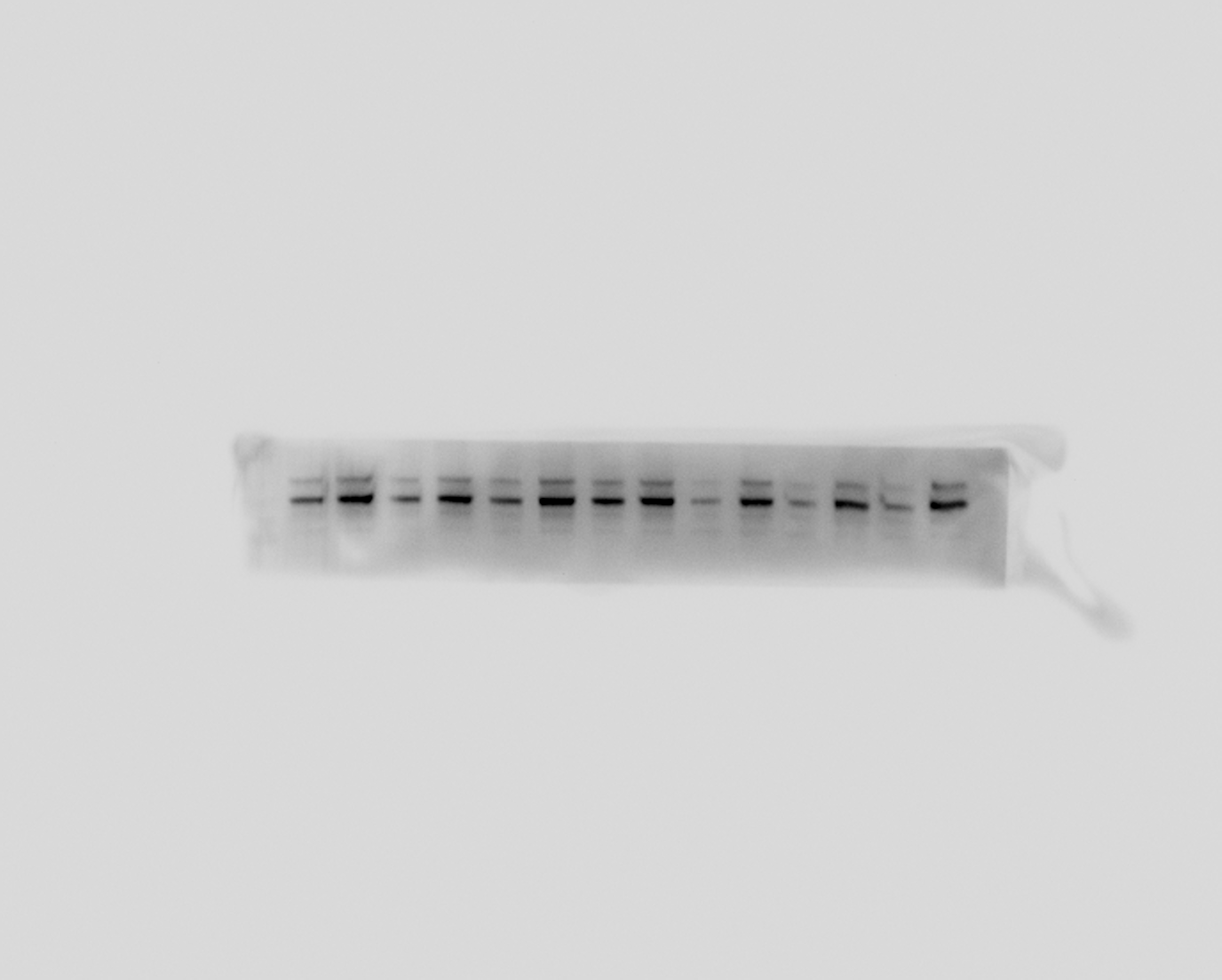

Supplement: Supplementary file 7 — Source data Fig. 2 [file 44318_2024_359_MOESM7_ESM.zip › Figure 2/Fig 2H and 2I/Fig 2H/(#1-#7) Triple-negative/3-p-S6K.Tif]

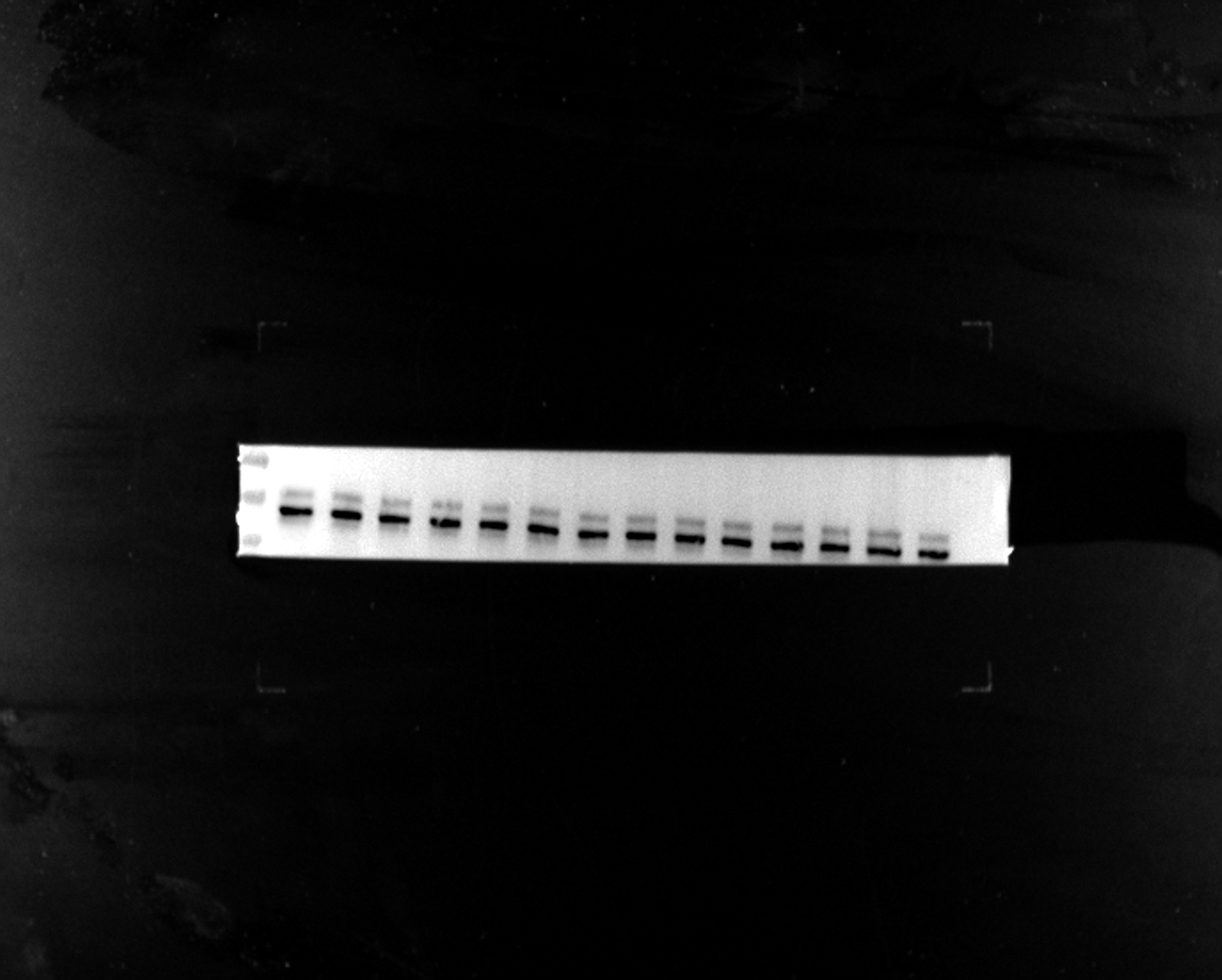

Supplement: Supplementary file 7 — Source data Fig. 2 [file 44318_2024_359_MOESM7_ESM.zip › Figure 2/Fig 2H and 2I/Fig 2H/(#1-#7) Triple-negative/4-S6K-merge.Tif]

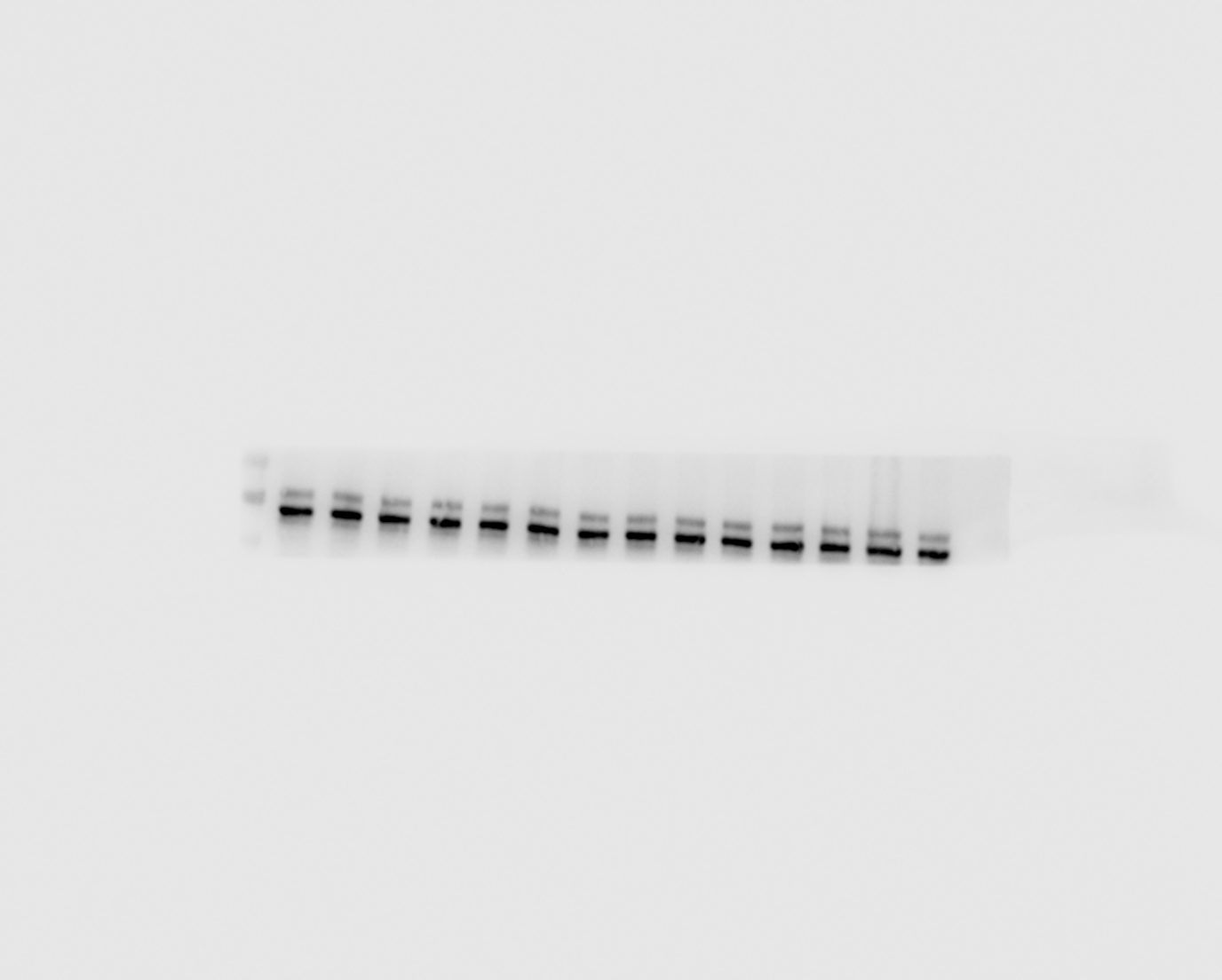

Supplement: Supplementary file 7 — Source data Fig. 2 [file 44318_2024_359_MOESM7_ESM.zip › Figure 2/Fig 2H and 2I/Fig 2H/(#1-#7) Triple-negative/4-s6k.Tif]

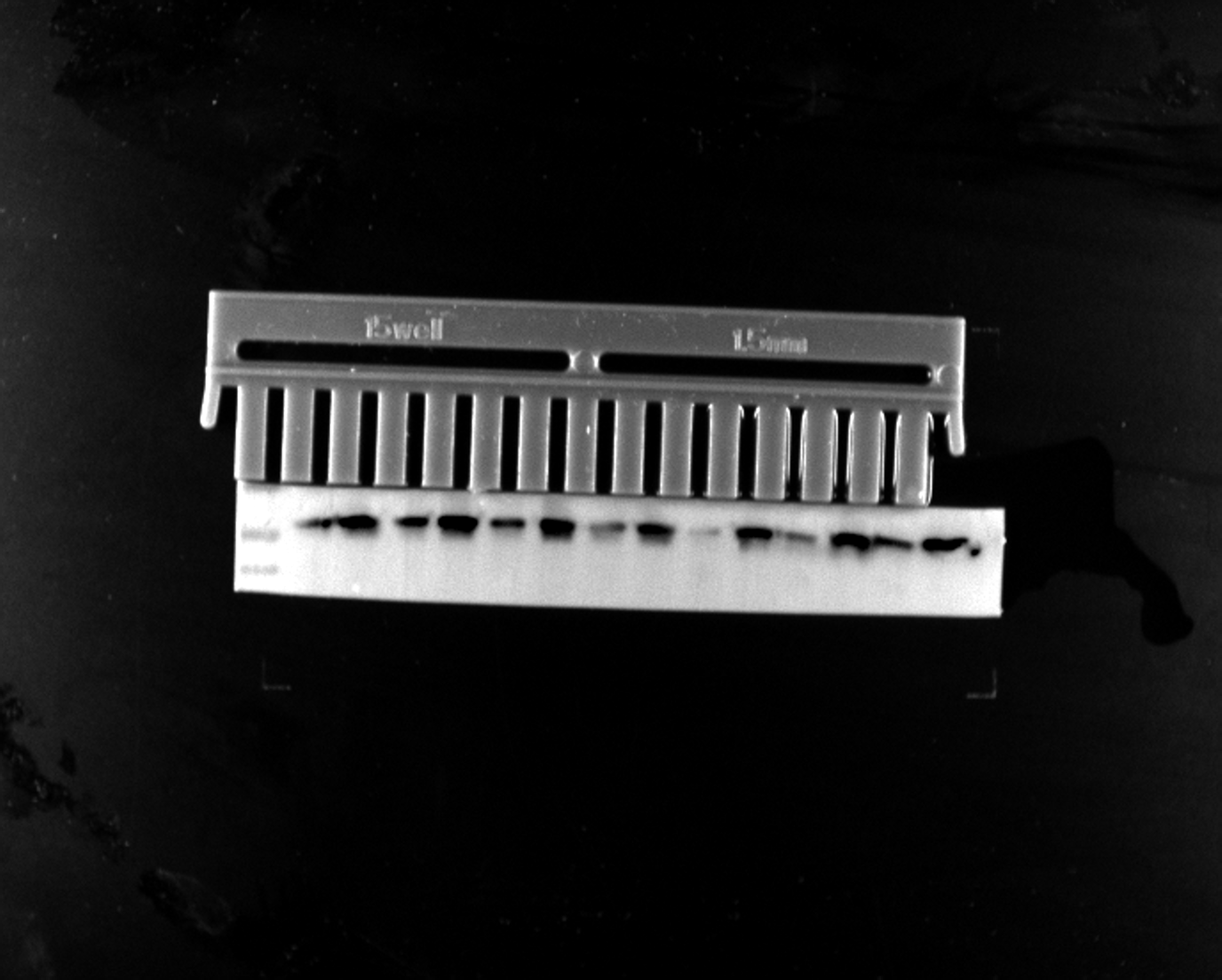

Supplement: Supplementary file 7 — Source data Fig. 2 [file 44318_2024_359_MOESM7_ESM.zip › Figure 2/Fig 2H and 2I/Fig 2H/(#1-#7) Triple-negative/5-p-S6-merge.Tif]

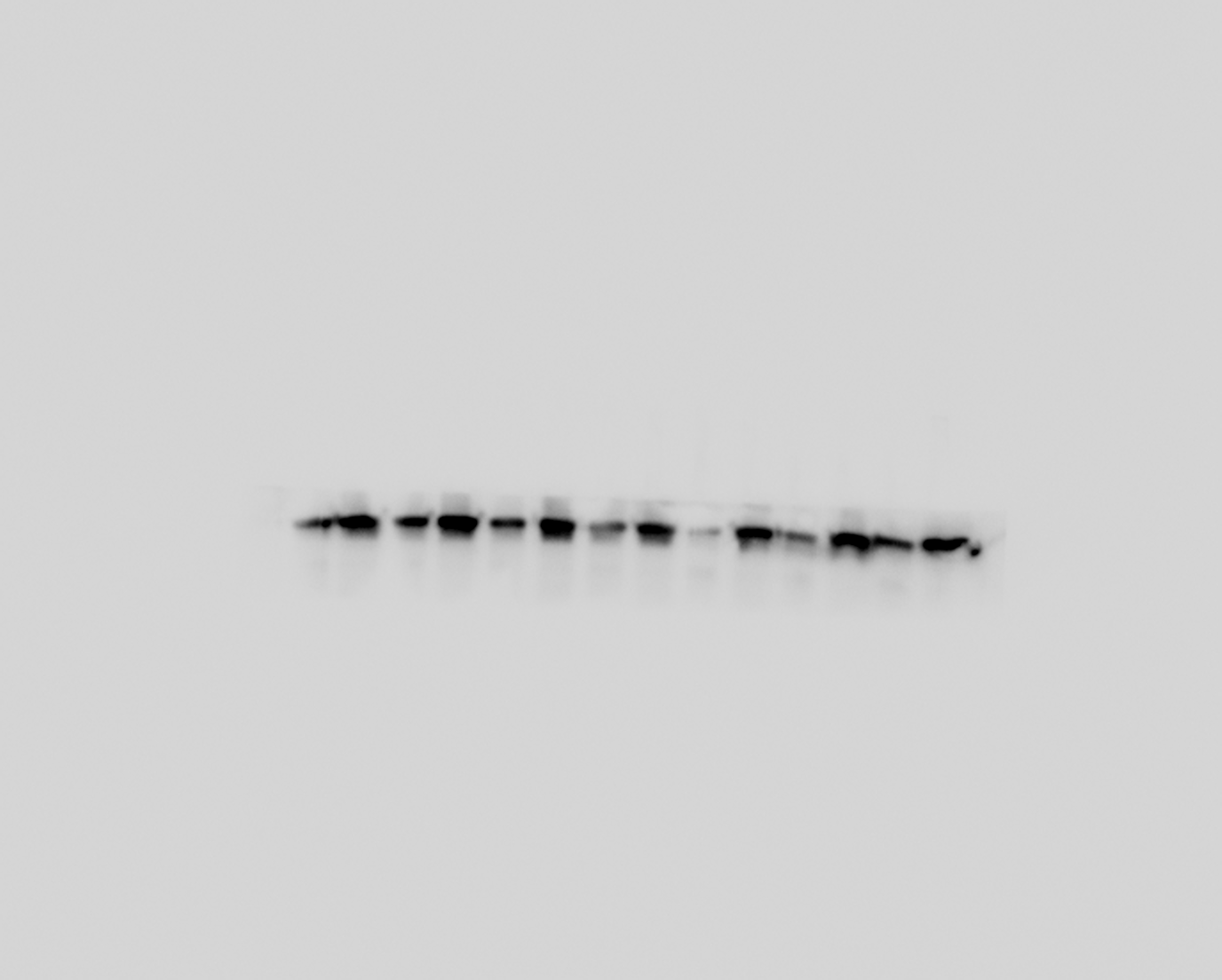

Supplement: Supplementary file 7 — Source data Fig. 2 [file 44318_2024_359_MOESM7_ESM.zip › Figure 2/Fig 2H and 2I/Fig 2H/(#1-#7) Triple-negative/5-p-S6.Tif]

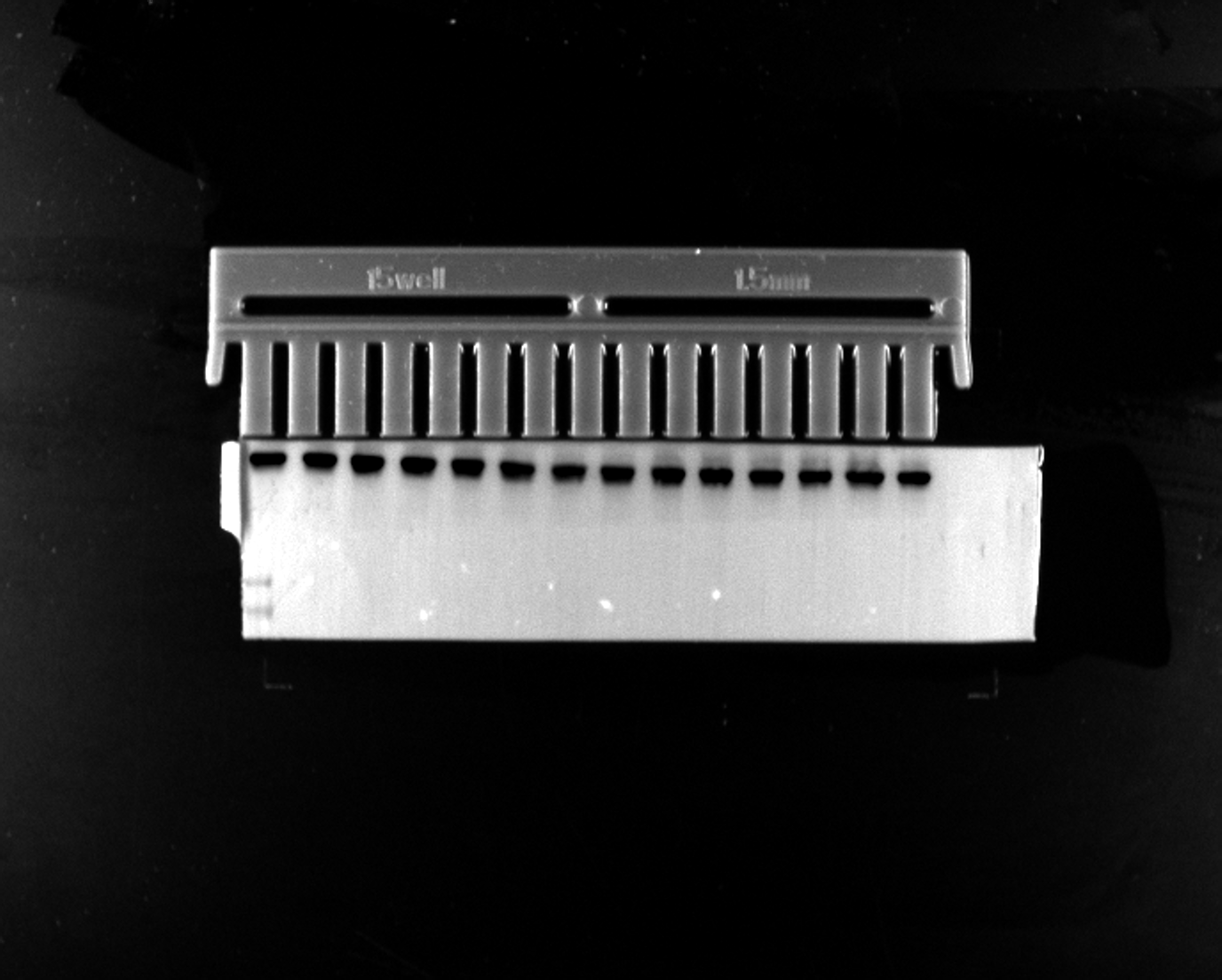

Supplement: Supplementary file 7 — Source data Fig. 2 [file 44318_2024_359_MOESM7_ESM.zip › Figure 2/Fig 2H and 2I/Fig 2H/(#1-#7) Triple-negative/6-S6-merge.Tif]

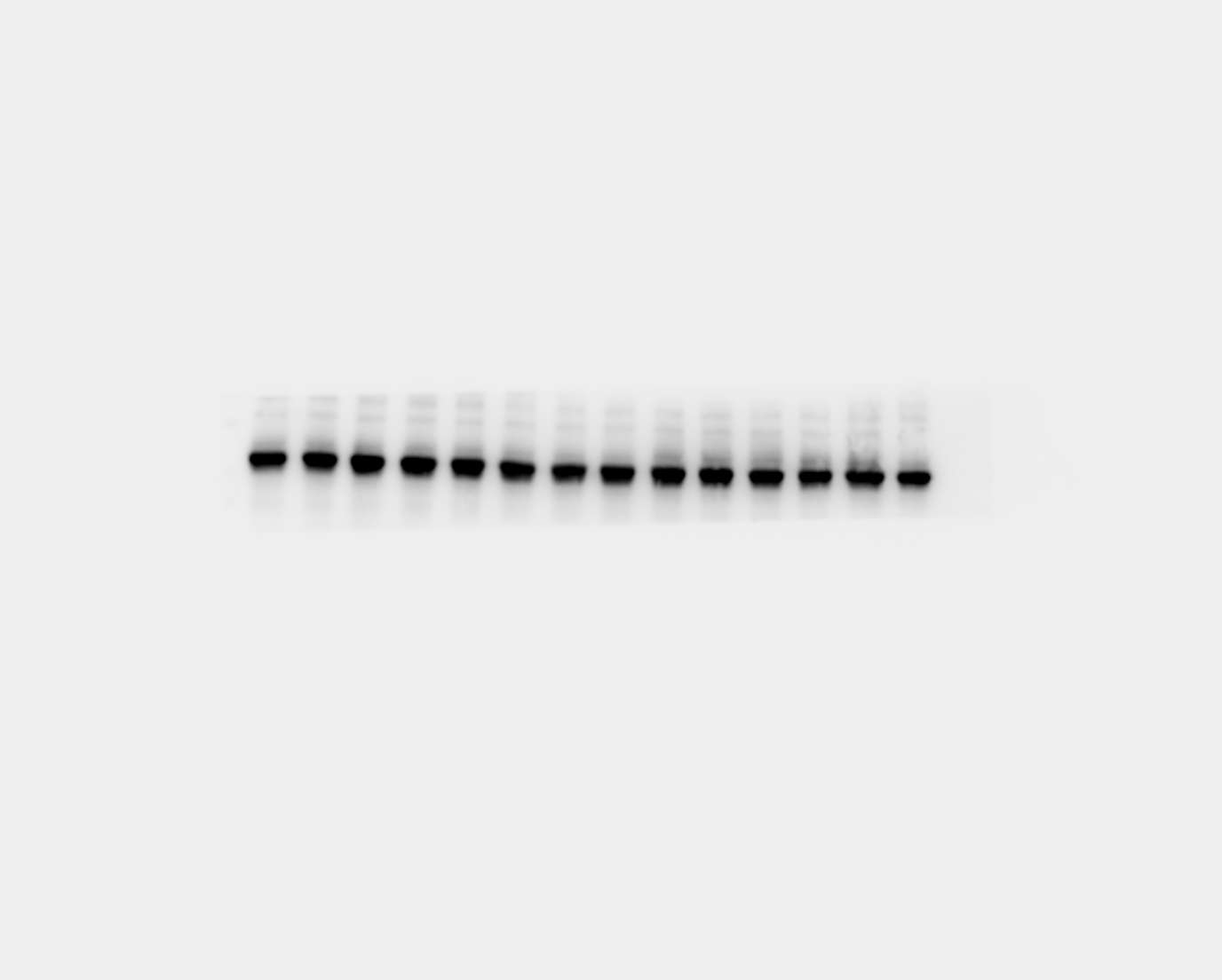

Supplement: Supplementary file 7 — Source data Fig. 2 [file 44318_2024_359_MOESM7_ESM.zip › Figure 2/Fig 2H and 2I/Fig 2H/(#1-#7) Triple-negative/6-S6.Tif]

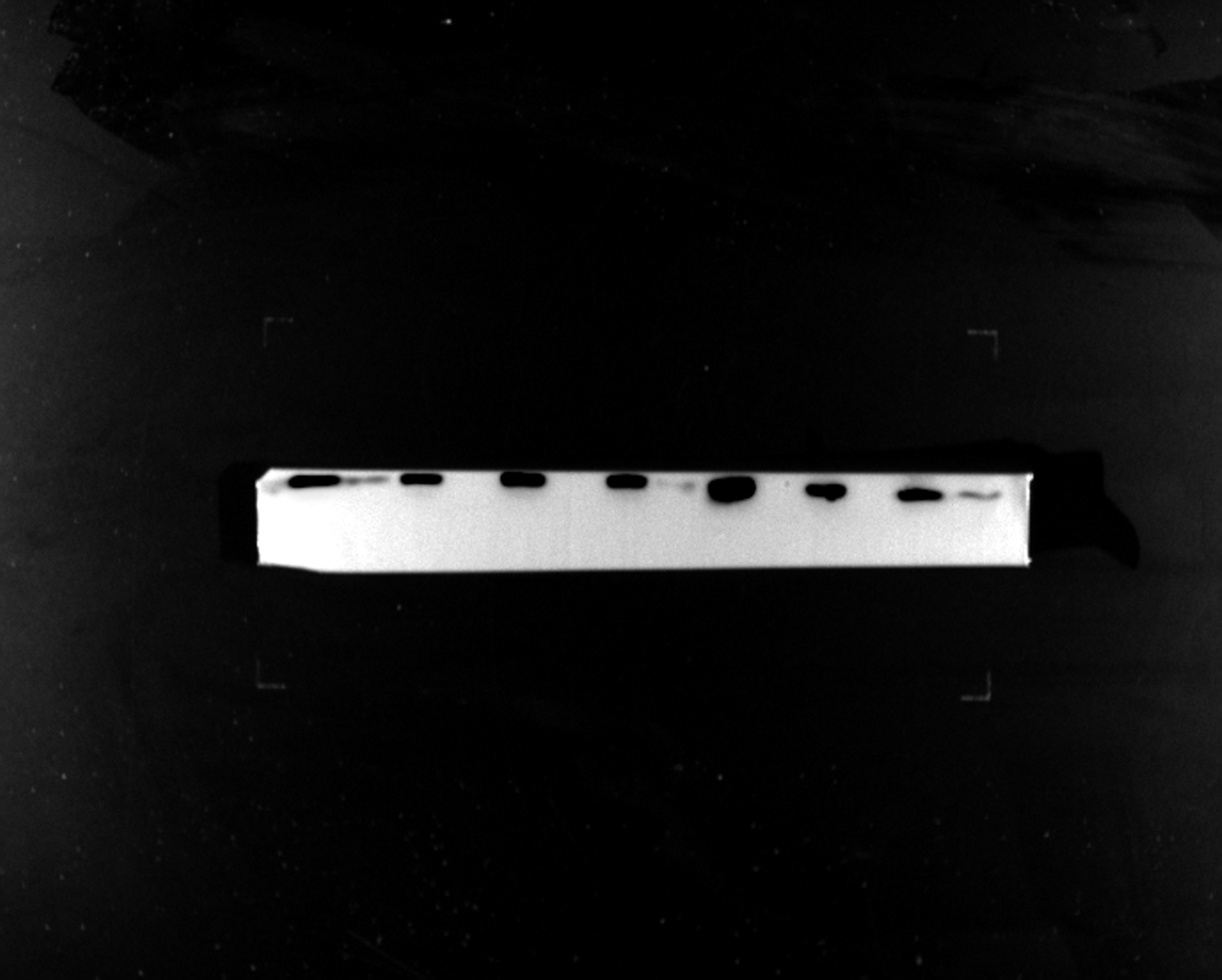

Supplement: Supplementary file 7 — Source data Fig. 2 [file 44318_2024_359_MOESM7_ESM.zip › Figure 2/Fig 2H and 2I/Fig 2H/(#1-#7) Triple-negative/7-hSPAR-merge.Tif]

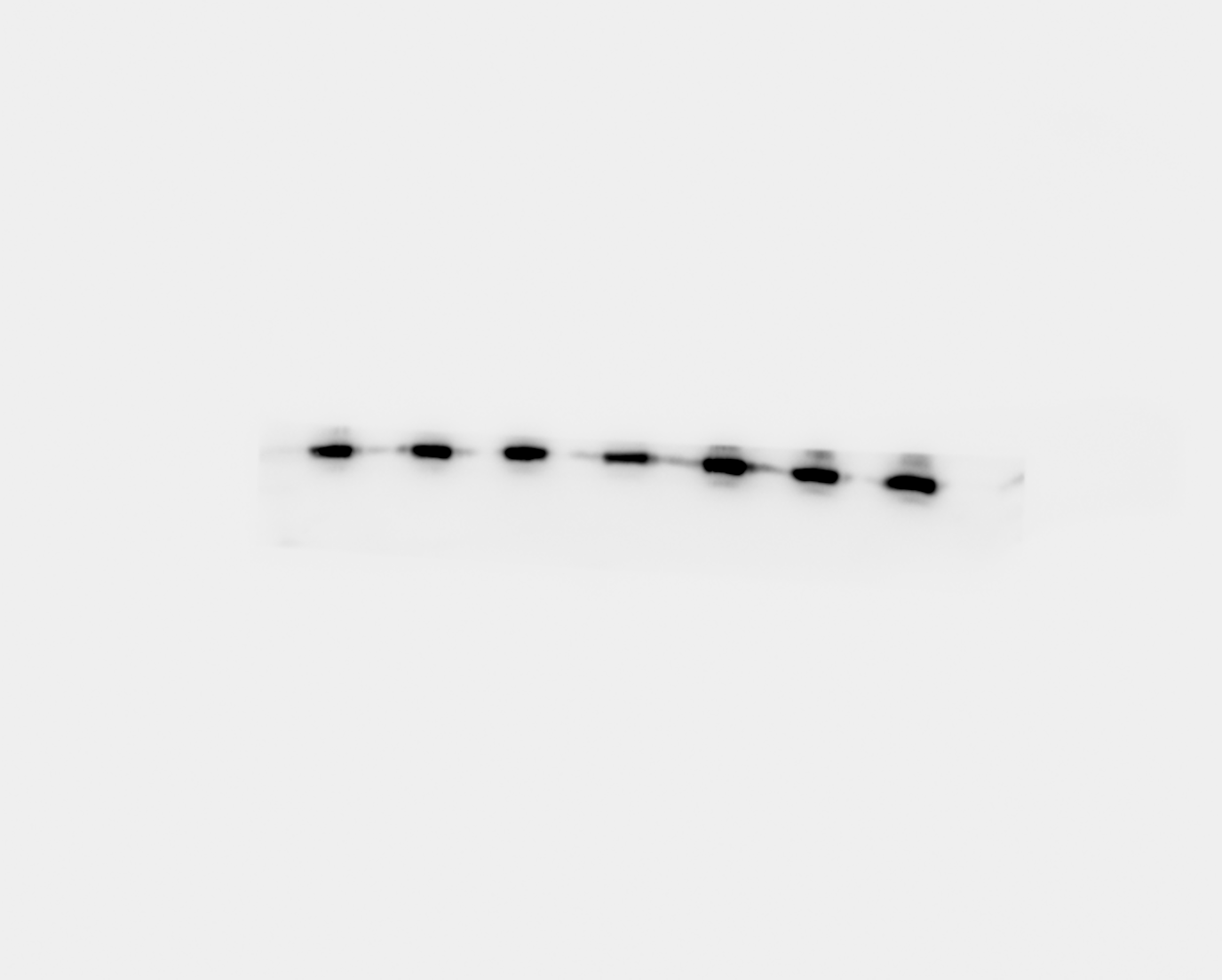

Supplement: Supplementary file 7 — Source data Fig. 2 [file 44318_2024_359_MOESM7_ESM.zip › Figure 2/Fig 2H and 2I/Fig 2H/(#1-#7) Triple-negative/7-hSPAR.Tif]

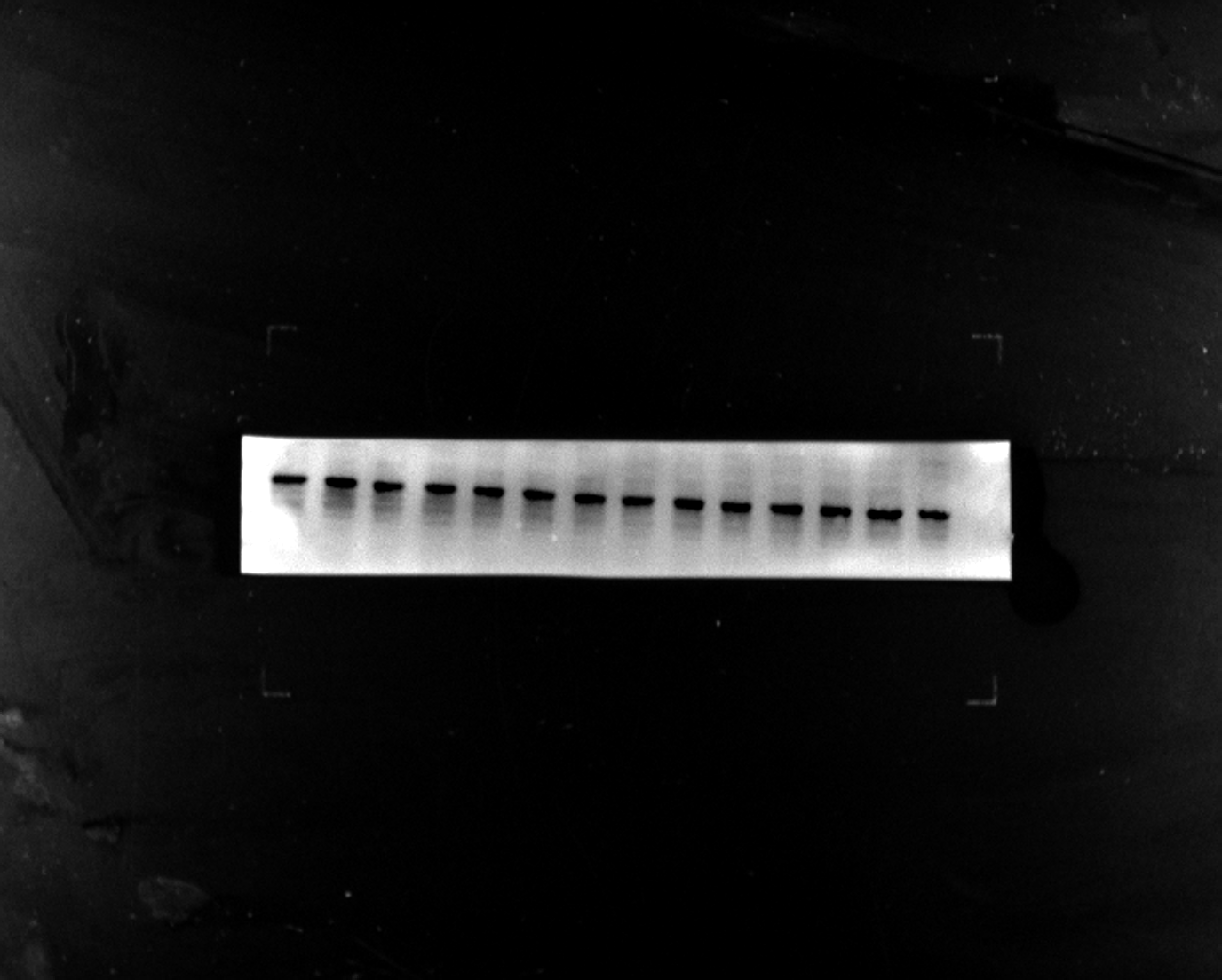

Supplement: Supplementary file 7 — Source data Fig. 2 [file 44318_2024_359_MOESM7_ESM.zip › Figure 2/Fig 2H and 2I/Fig 2H/(#1-#7) Triple-negative/8-GAPDH-merge.Tif]

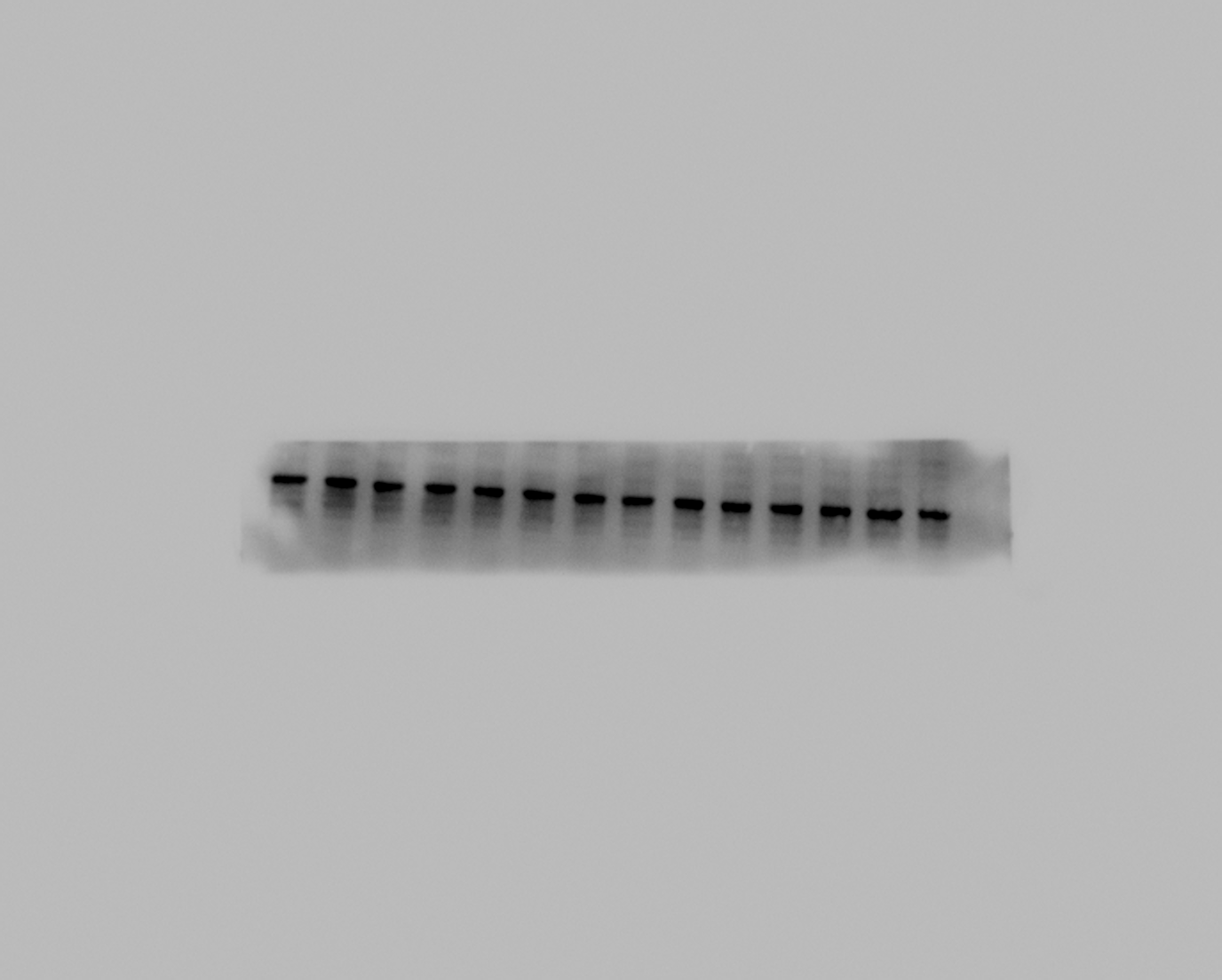

Supplement: Supplementary file 7 — Source data Fig. 2 [file 44318_2024_359_MOESM7_ESM.zip › Figure 2/Fig 2H and 2I/Fig 2H/(#1-#7) Triple-negative/8-GAPDH.Tif]

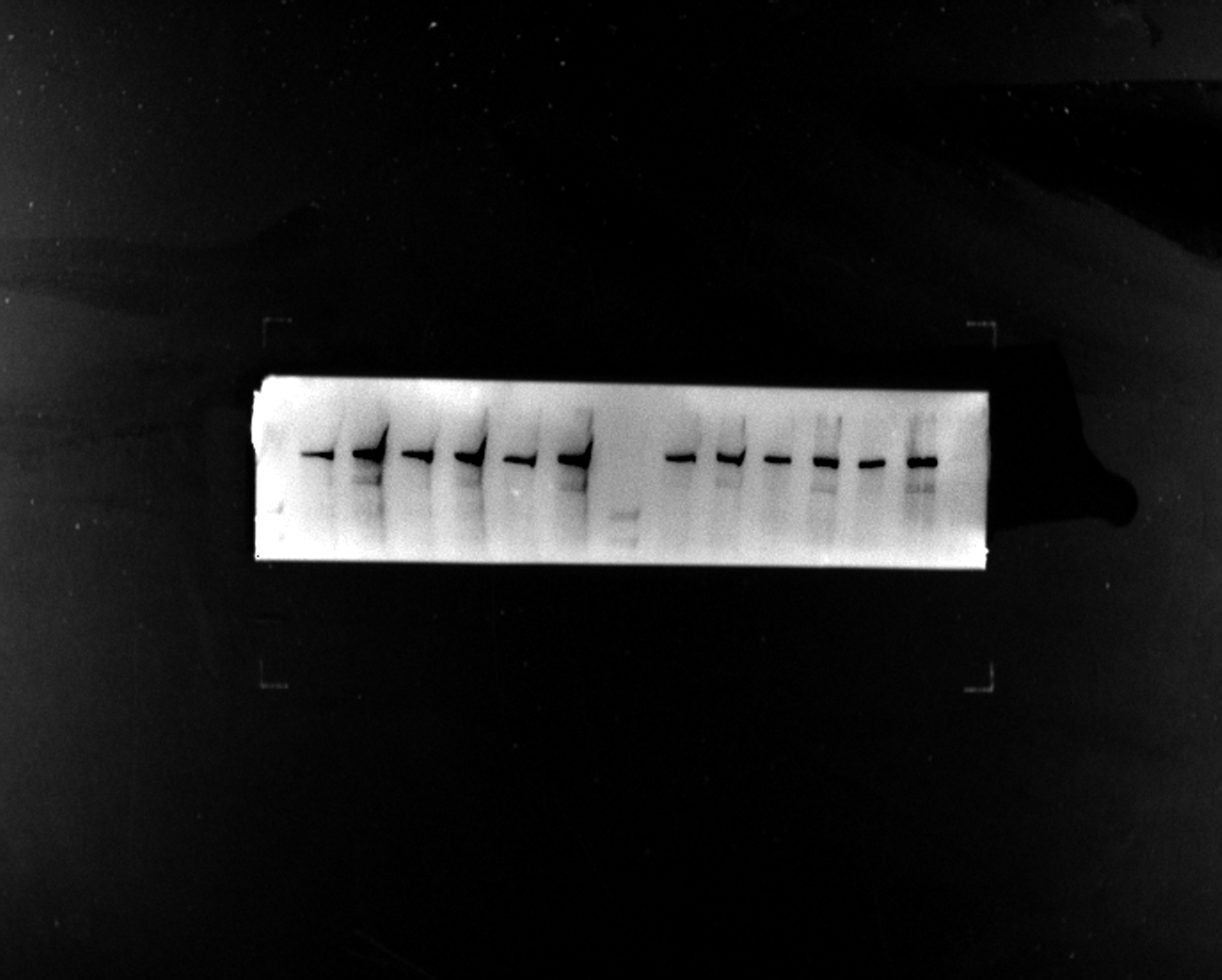

Supplement: Supplementary file 7 — Source data Fig. 2 [file 44318_2024_359_MOESM7_ESM.zip › Figure 2/Fig 2H and 2I/Fig 2H/(#11-#16) Luminal A+Luminal B/1-p-mtor-merge.Tif]

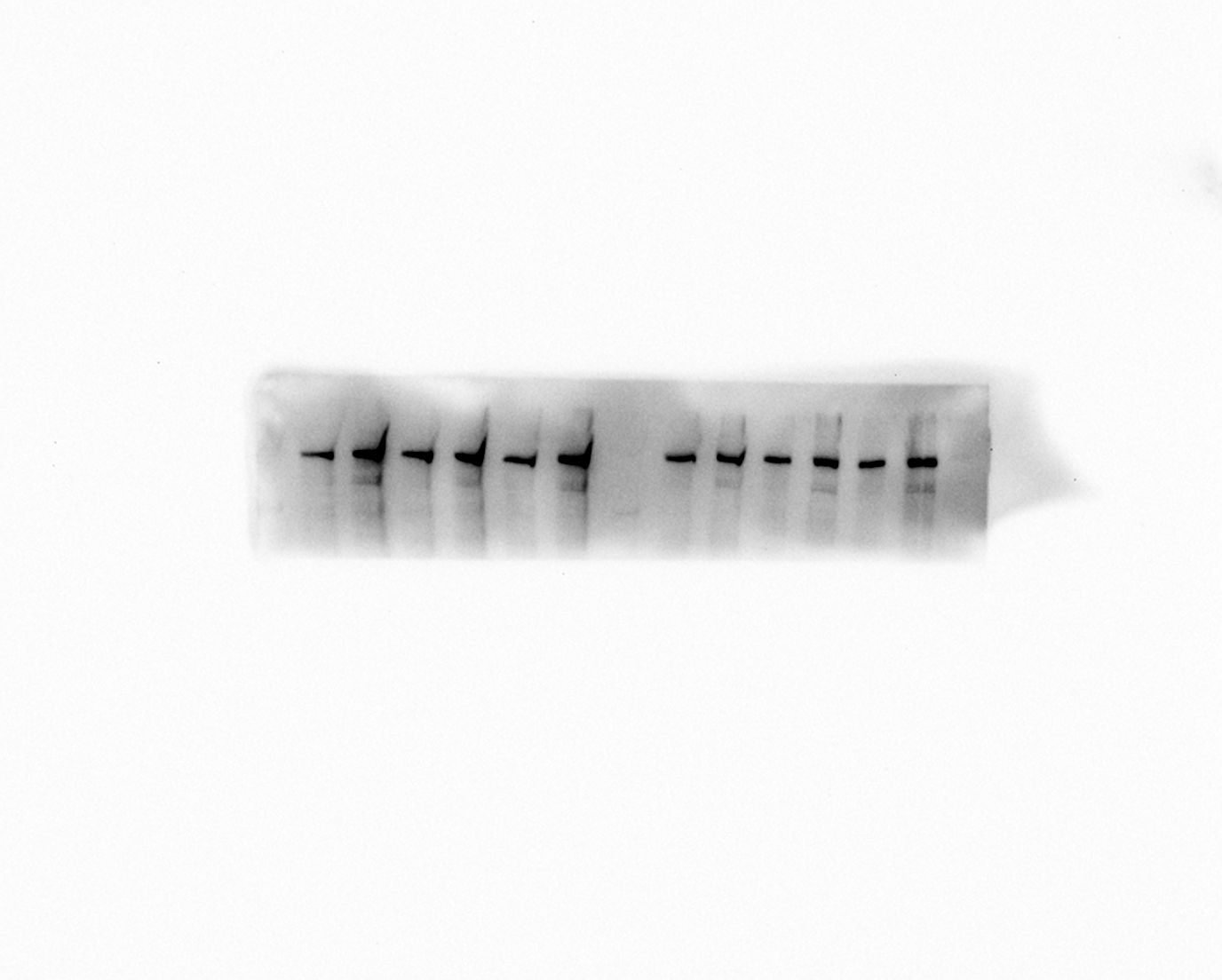

Supplement: Supplementary file 7 — Source data Fig. 2 [file 44318_2024_359_MOESM7_ESM.zip › Figure 2/Fig 2H and 2I/Fig 2H/(#11-#16) Luminal A+Luminal B/1-p-mtor.Tif]

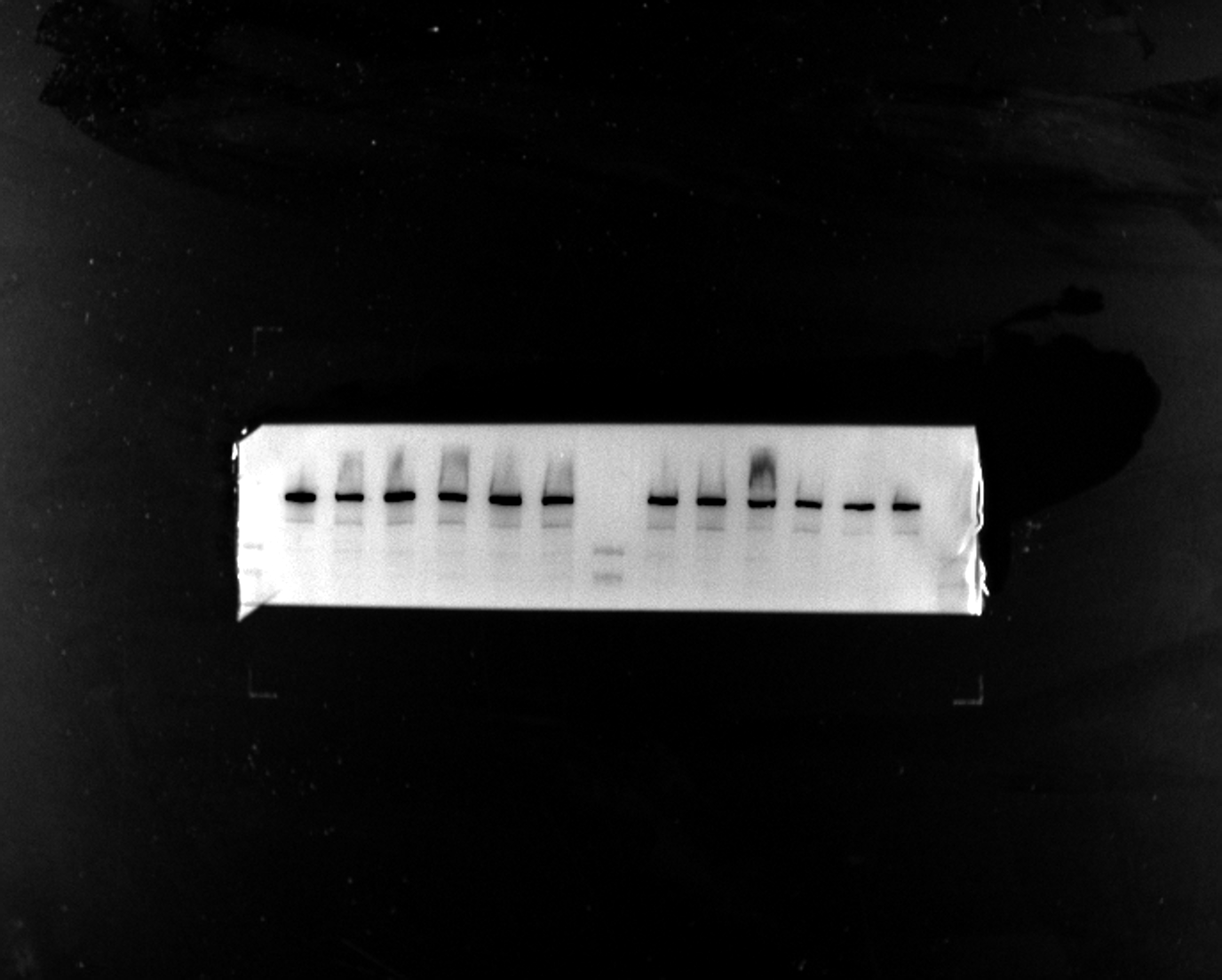

Supplement: Supplementary file 7 — Source data Fig. 2 [file 44318_2024_359_MOESM7_ESM.zip › Figure 2/Fig 2H and 2I/Fig 2H/(#11-#16) Luminal A+Luminal B/2-mTOR-merge.Tif]

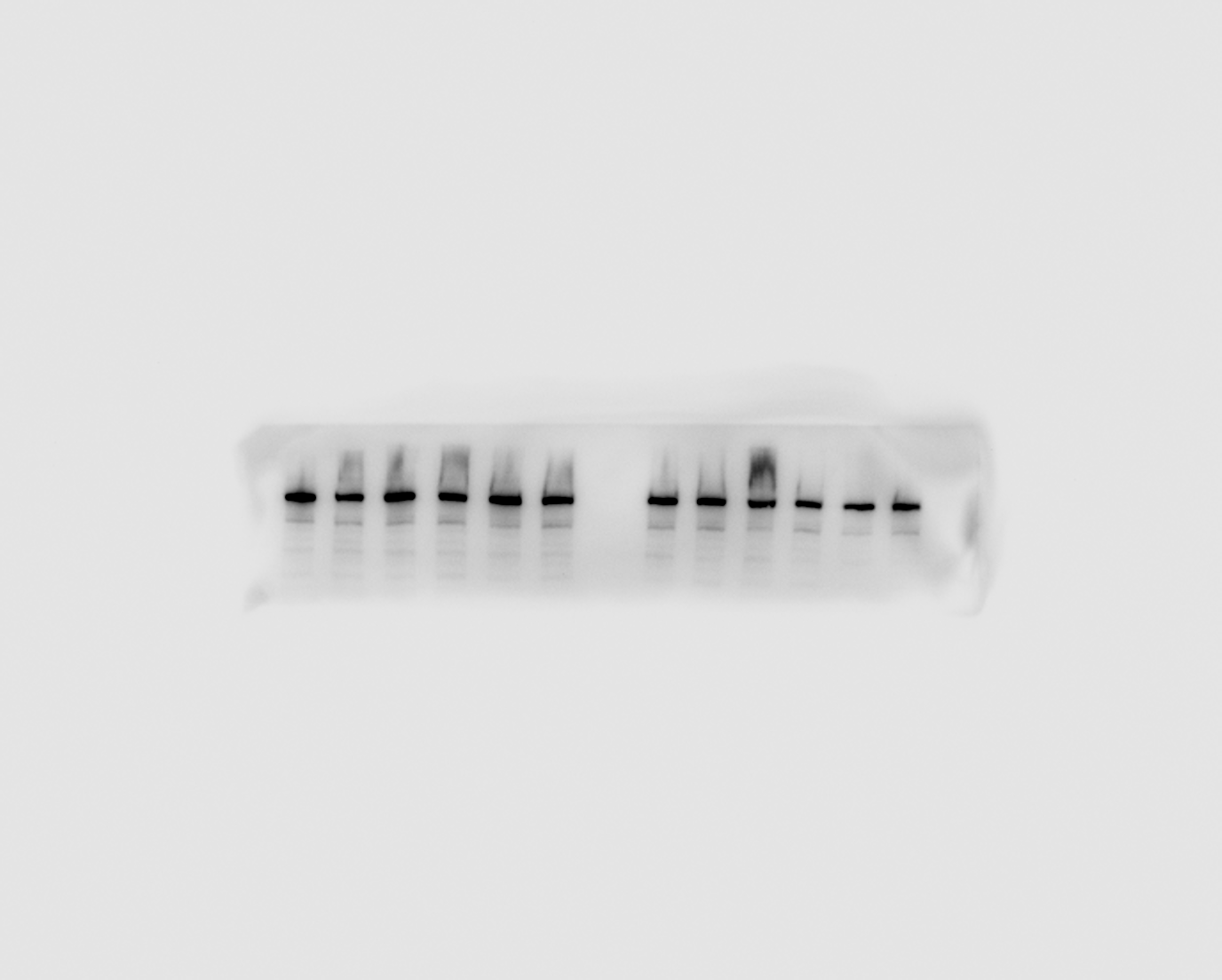

Supplement: Supplementary file 7 — Source data Fig. 2 [file 44318_2024_359_MOESM7_ESM.zip › Figure 2/Fig 2H and 2I/Fig 2H/(#11-#16) Luminal A+Luminal B/2-mTOR.Tif]

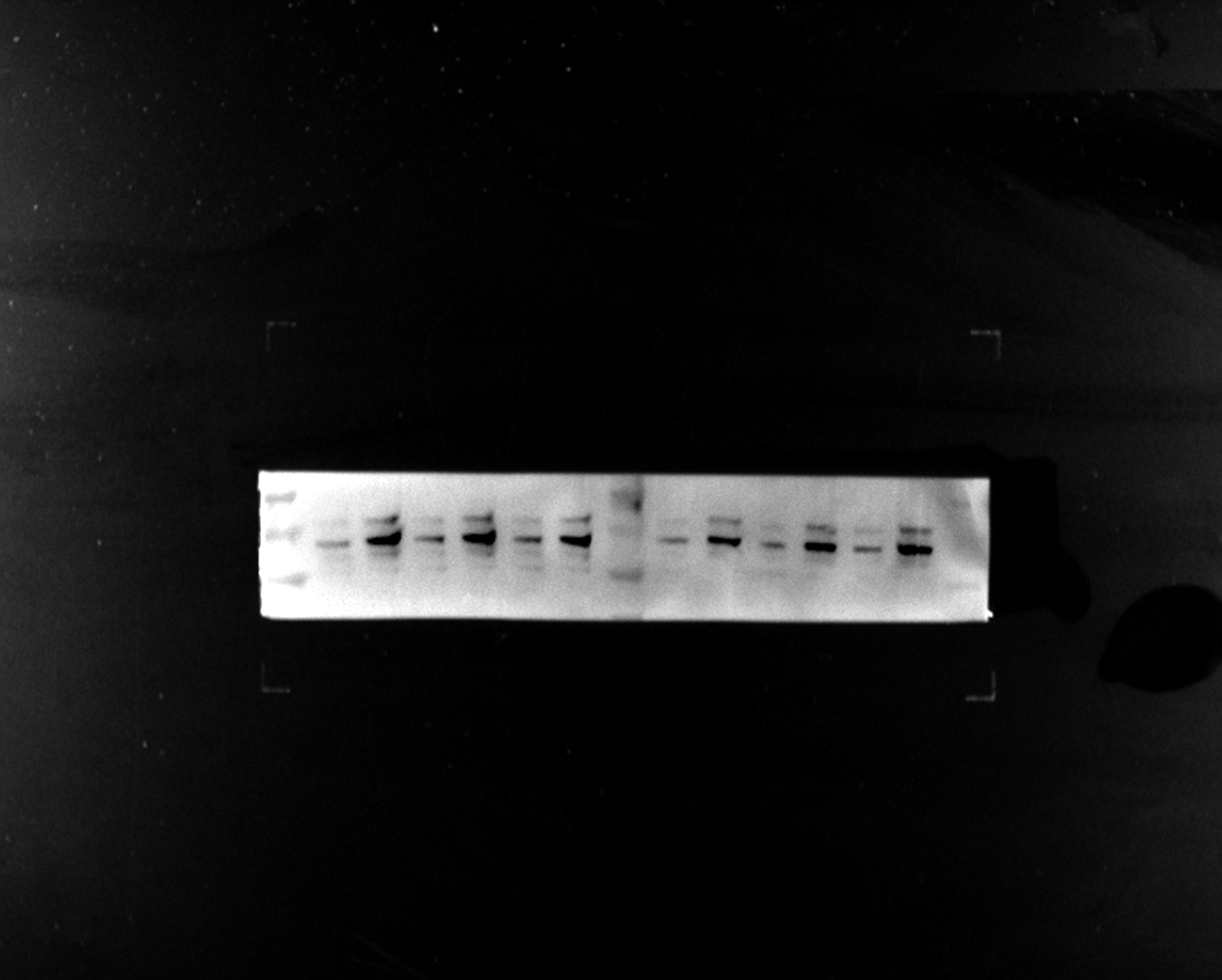

Supplement: Supplementary file 7 — Source data Fig. 2 [file 44318_2024_359_MOESM7_ESM.zip › Figure 2/Fig 2H and 2I/Fig 2H/(#11-#16) Luminal A+Luminal B/3-p-S6K-merge.Tif]

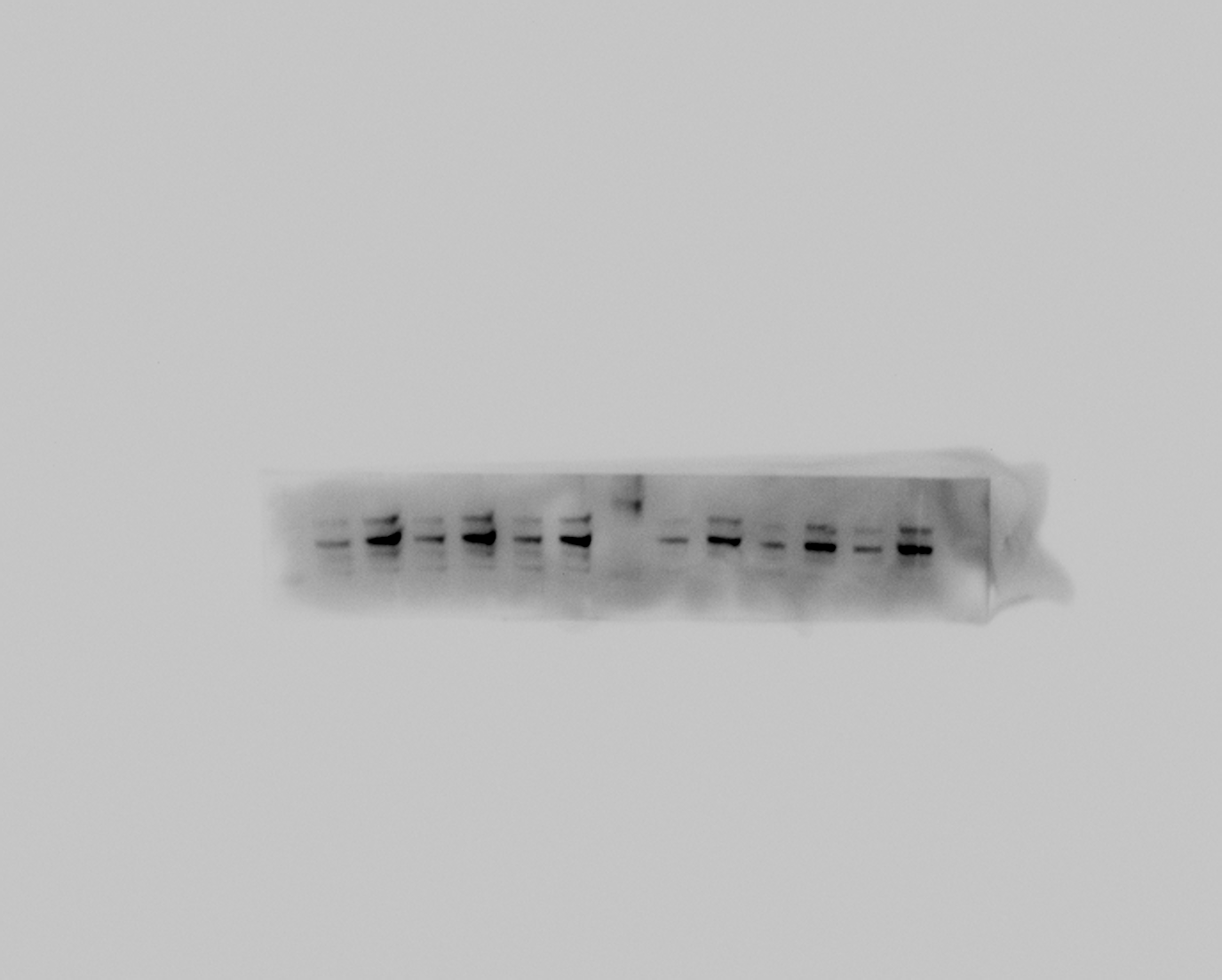

Supplement: Supplementary file 7 — Source data Fig. 2 [file 44318_2024_359_MOESM7_ESM.zip › Figure 2/Fig 2H and 2I/Fig 2H/(#11-#16) Luminal A+Luminal B/3-p-S6K.Tif]

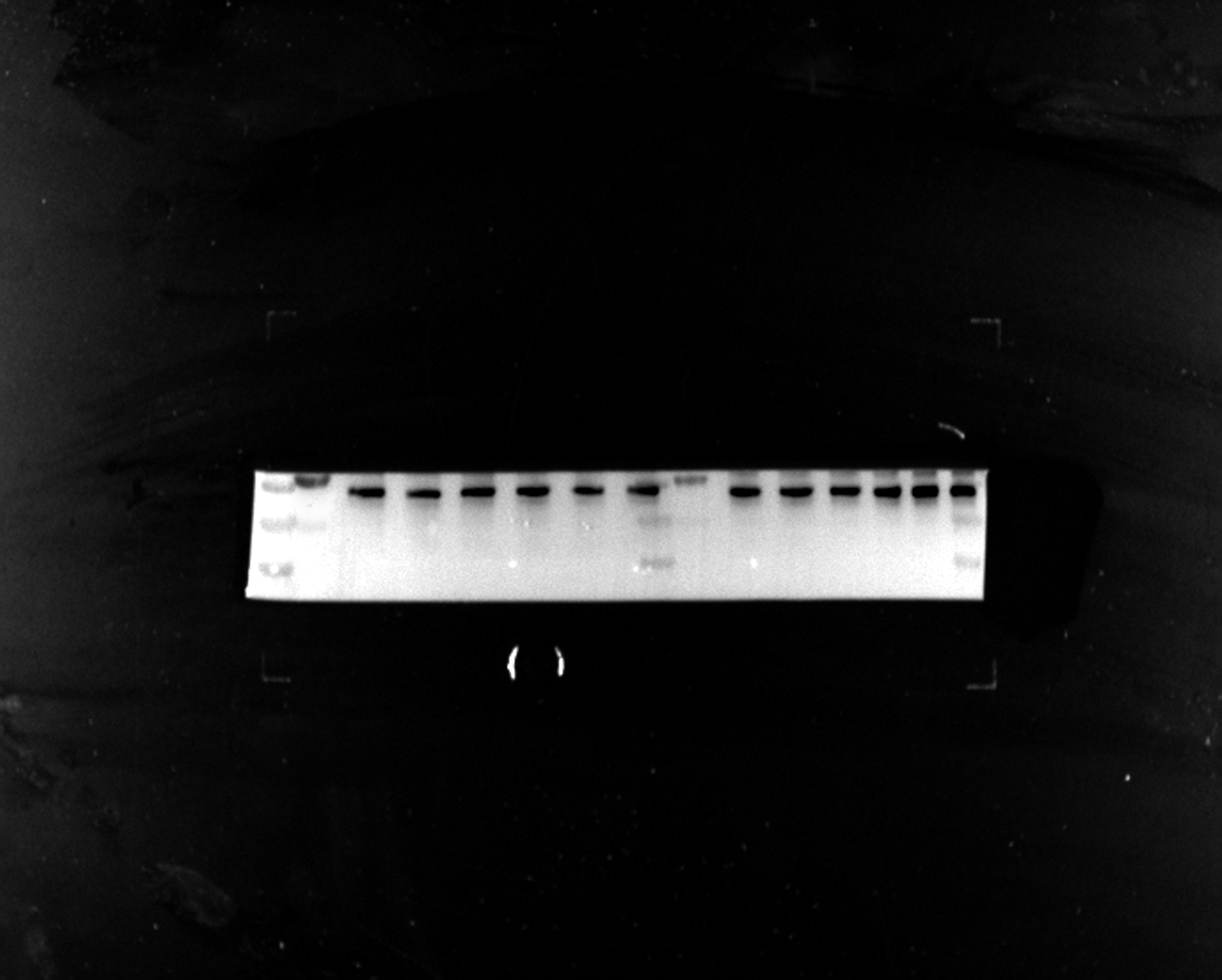

Supplement: Supplementary file 7 — Source data Fig. 2 [file 44318_2024_359_MOESM7_ESM.zip › Figure 2/Fig 2H and 2I/Fig 2H/(#11-#16) Luminal A+Luminal B/4-S6K-merge.Tif]

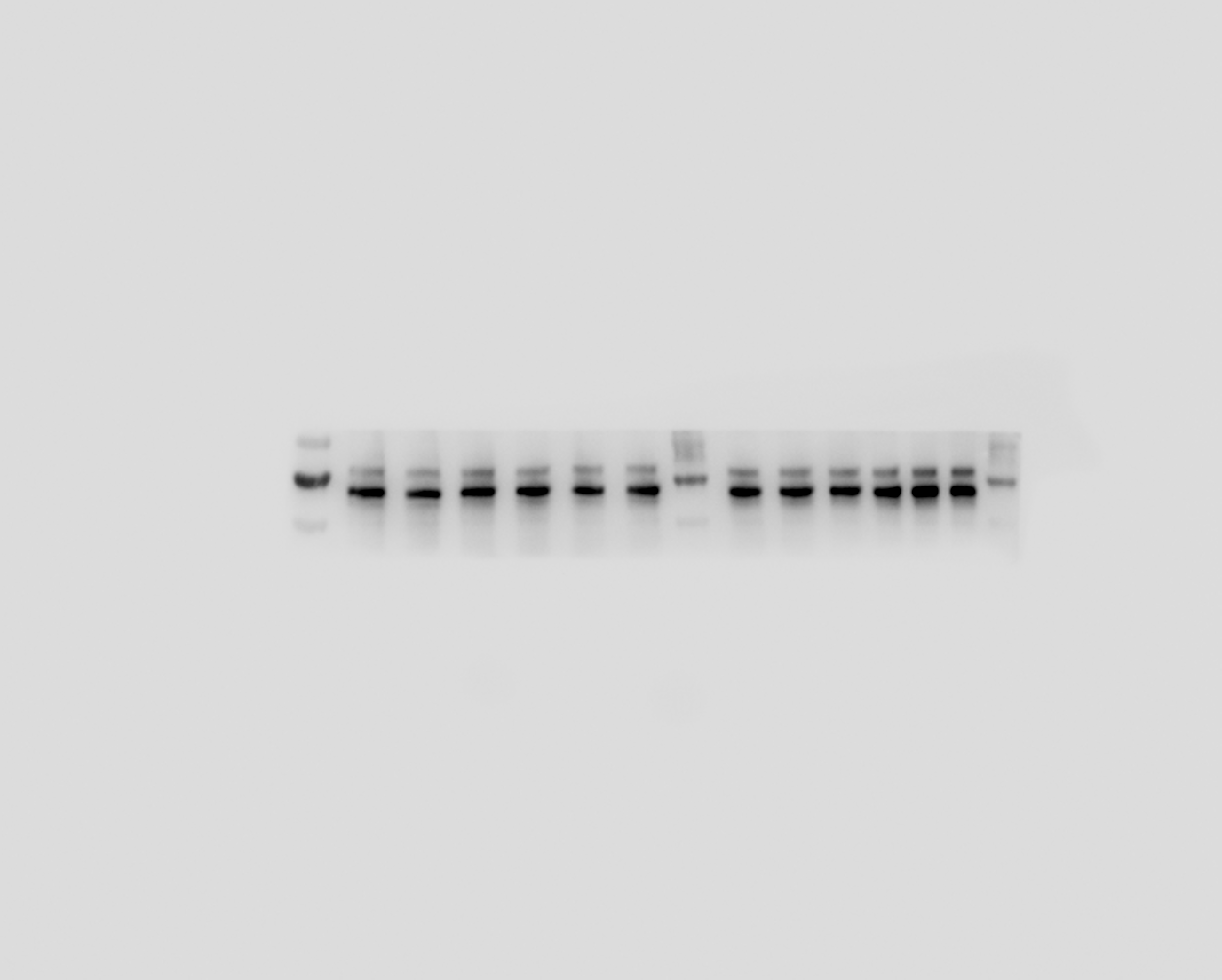

Supplement: Supplementary file 7 — Source data Fig. 2 [file 44318_2024_359_MOESM7_ESM.zip › Figure 2/Fig 2H and 2I/Fig 2H/(#11-#16) Luminal A+Luminal B/4-S6K.Tif]

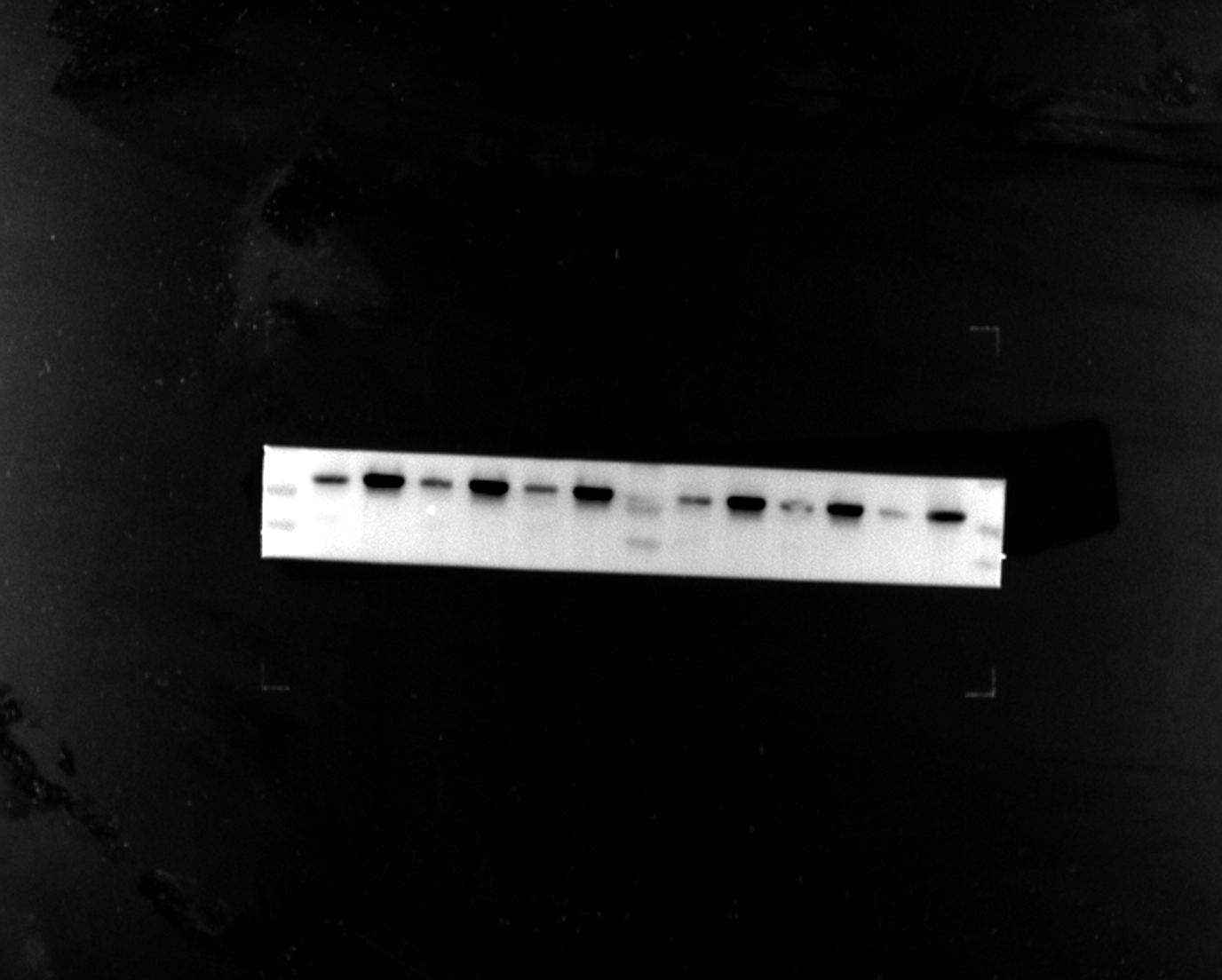

Supplement: Supplementary file 7 — Source data Fig. 2 [file 44318_2024_359_MOESM7_ESM.zip › Figure 2/Fig 2H and 2I/Fig 2H/(#11-#16) Luminal A+Luminal B/5-p-S6-merge.Tif]

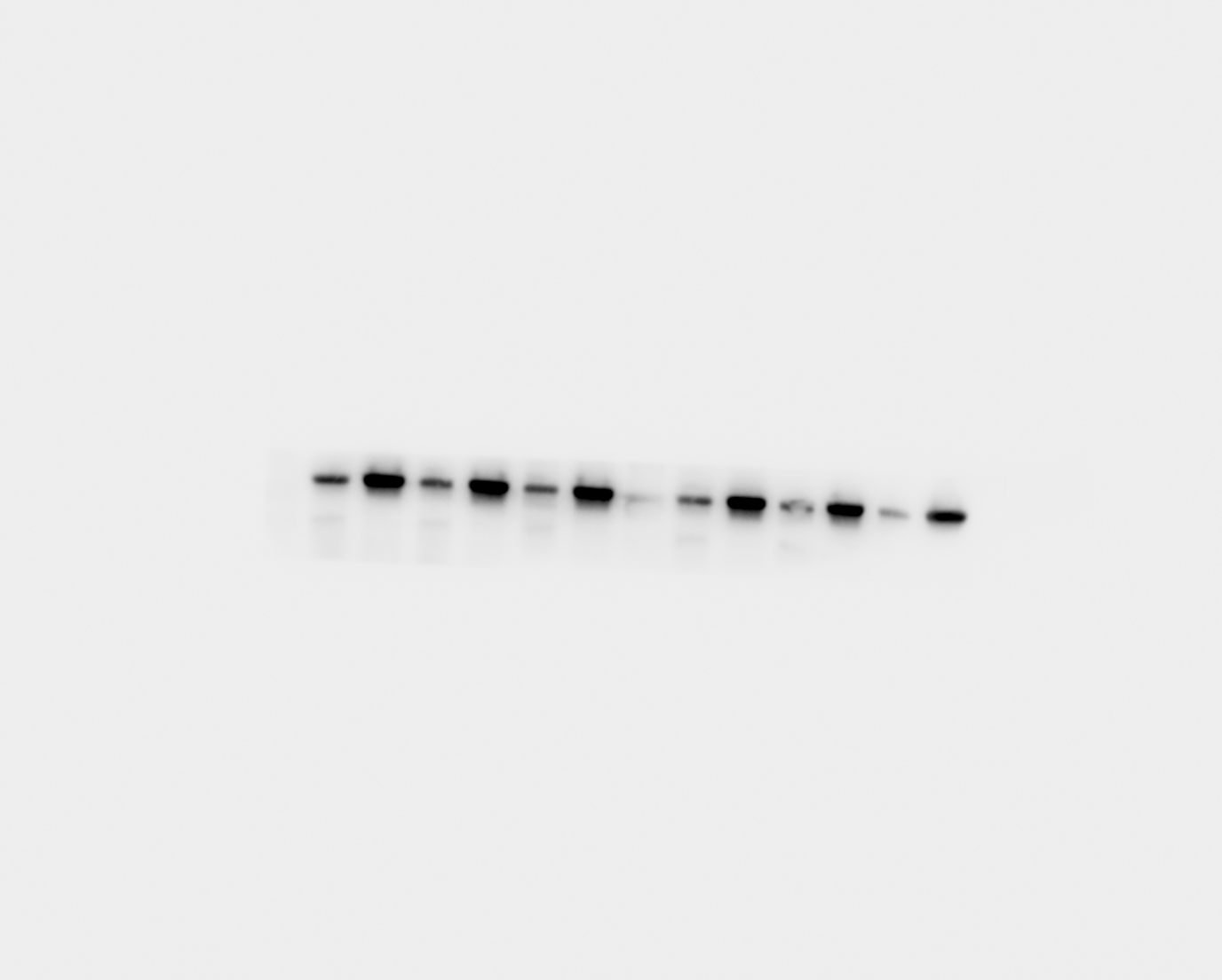

Supplement: Supplementary file 7 — Source data Fig. 2 [file 44318_2024_359_MOESM7_ESM.zip › Figure 2/Fig 2H and 2I/Fig 2H/(#11-#16) Luminal A+Luminal B/5-p-S6.Tif]

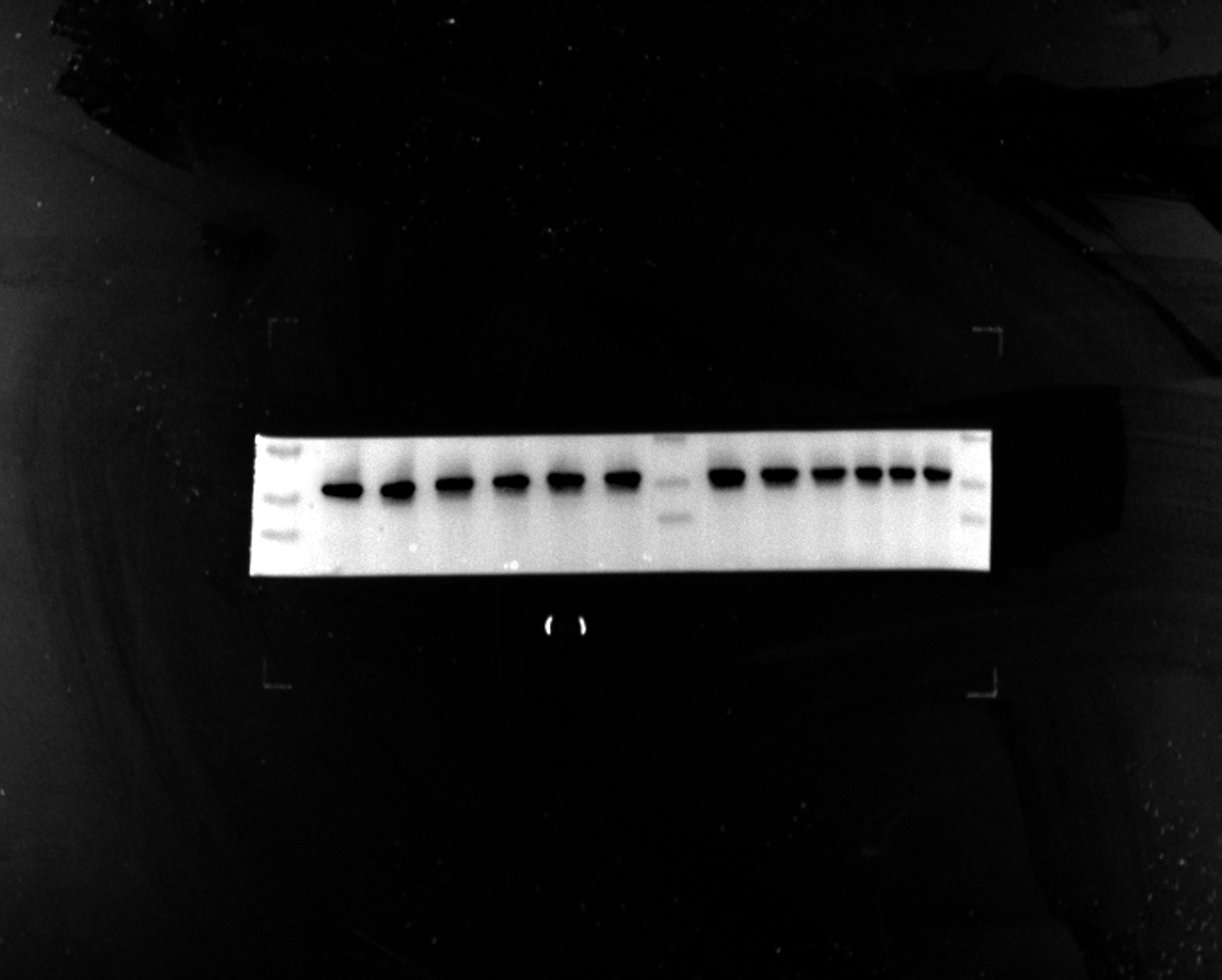

Supplement: Supplementary file 7 — Source data Fig. 2 [file 44318_2024_359_MOESM7_ESM.zip › Figure 2/Fig 2H and 2I/Fig 2H/(#11-#16) Luminal A+Luminal B/6-S6-merge.Tif]

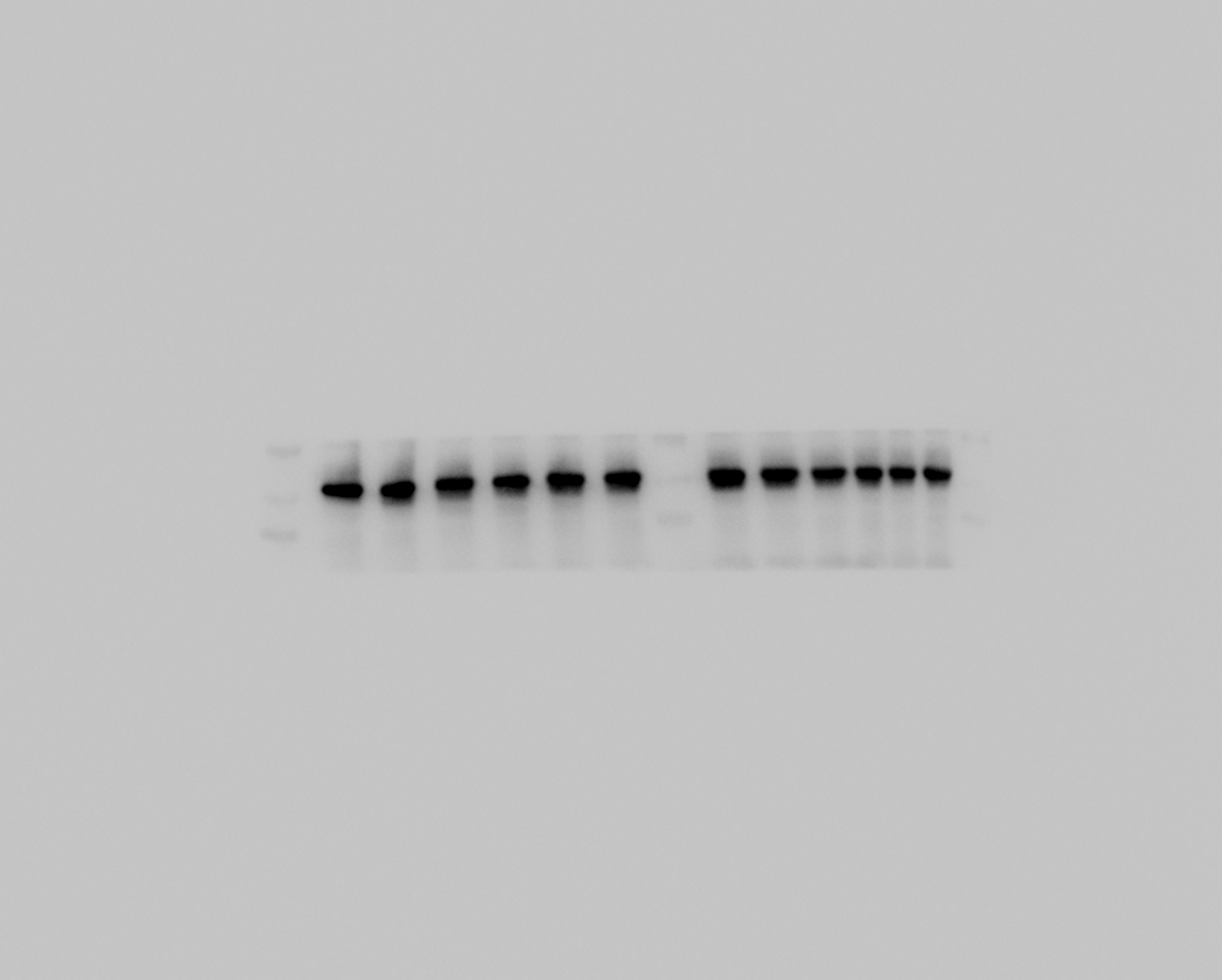

Supplement: Supplementary file 7 — Source data Fig. 2 [file 44318_2024_359_MOESM7_ESM.zip › Figure 2/Fig 2H and 2I/Fig 2H/(#11-#16) Luminal A+Luminal B/6-S6.Tif]

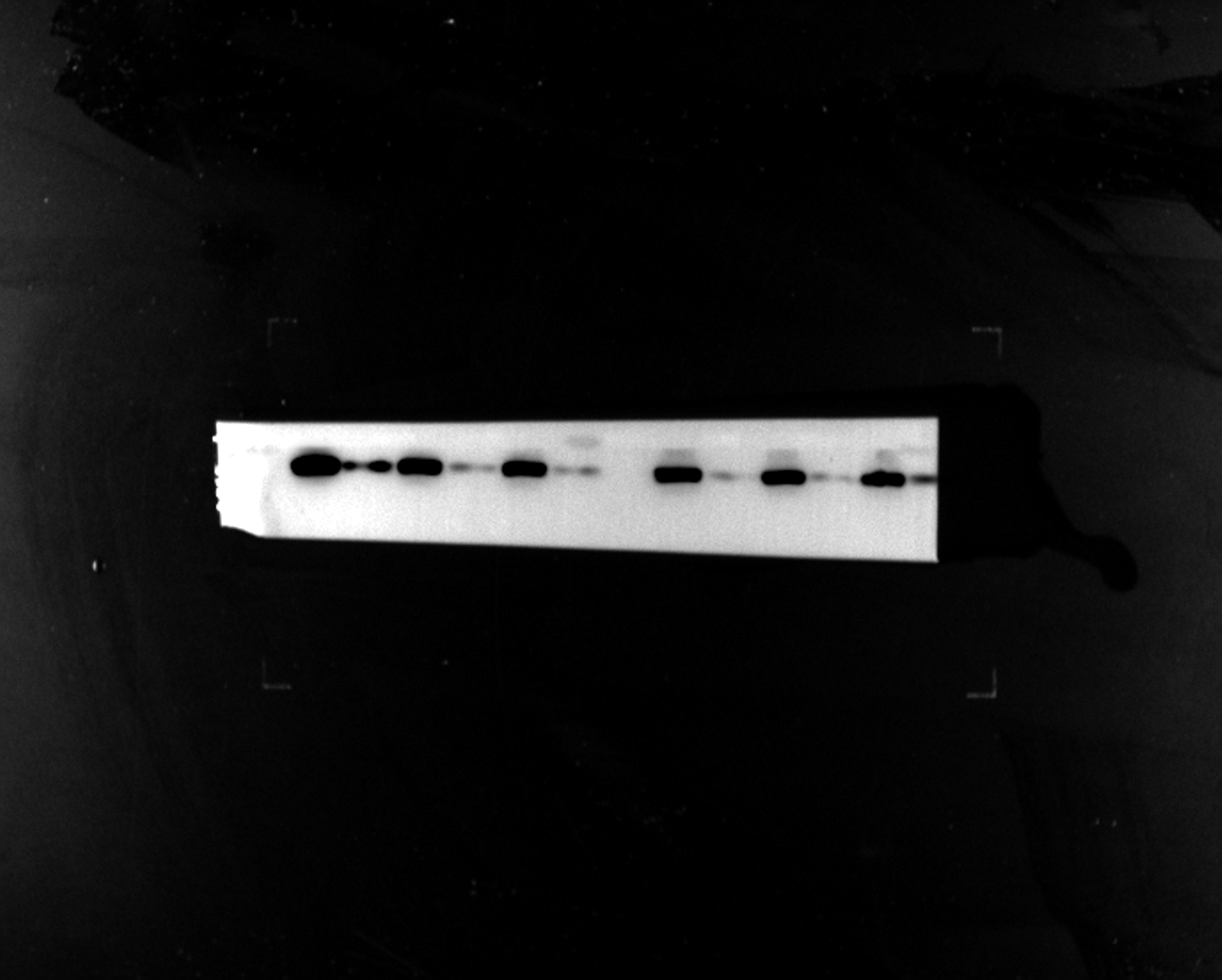

Supplement: Supplementary file 7 — Source data Fig. 2 [file 44318_2024_359_MOESM7_ESM.zip › Figure 2/Fig 2H and 2I/Fig 2H/(#11-#16) Luminal A+Luminal B/7-hSPAR-merge.Tif]

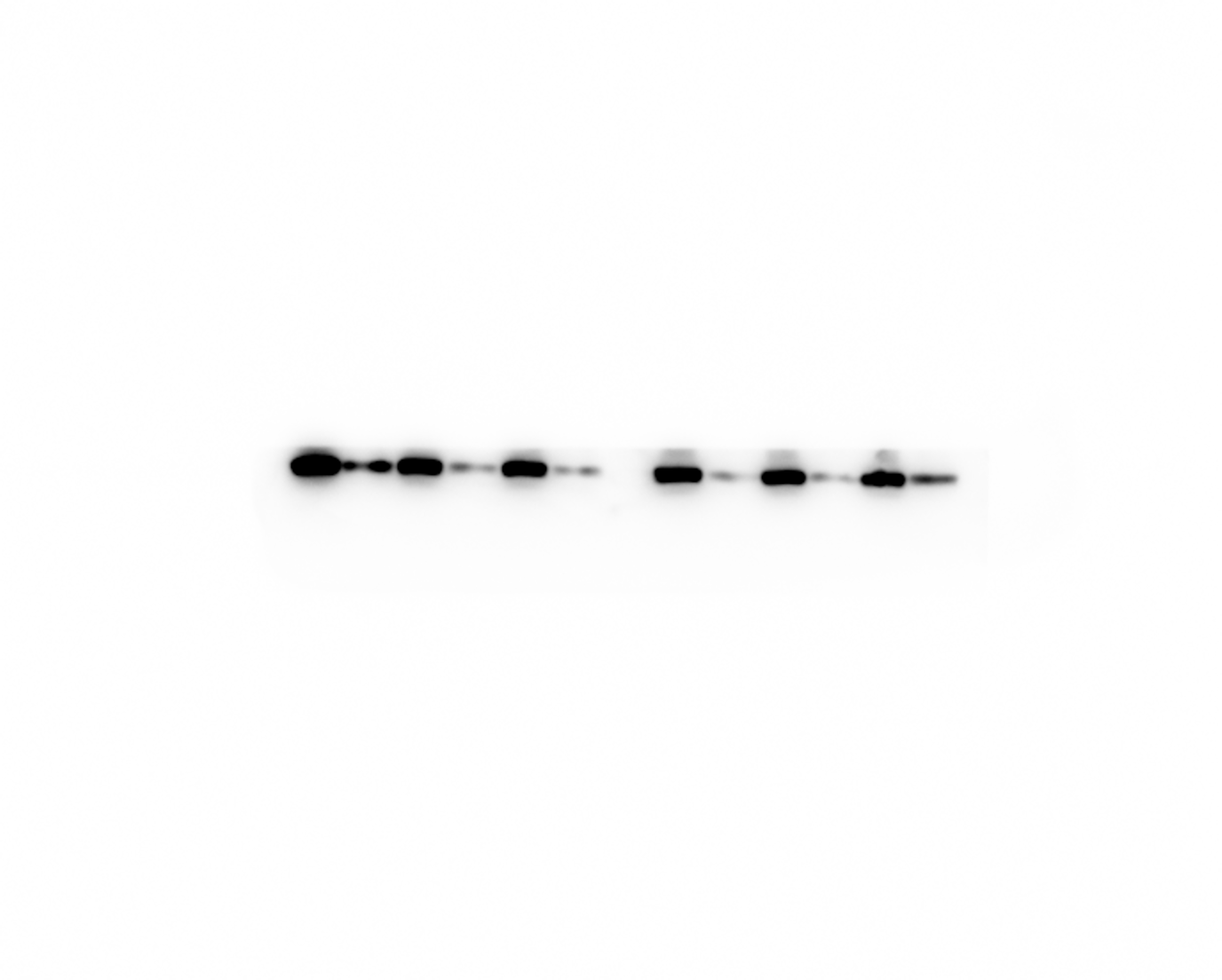

Supplement: Supplementary file 7 — Source data Fig. 2 [file 44318_2024_359_MOESM7_ESM.zip › Figure 2/Fig 2H and 2I/Fig 2H/(#11-#16) Luminal A+Luminal B/7-hSPAR.Tif]

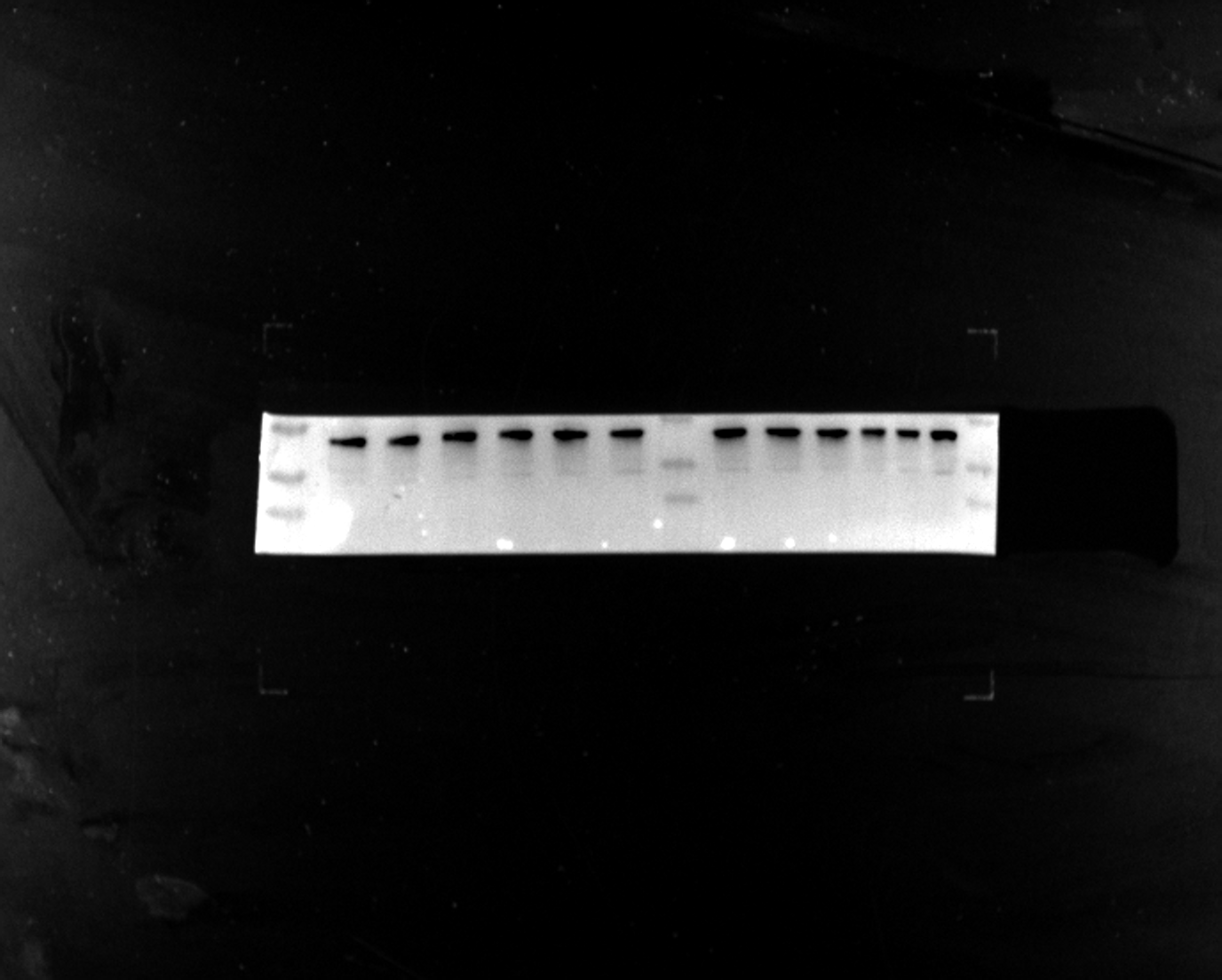

Supplement: Supplementary file 7 — Source data Fig. 2 [file 44318_2024_359_MOESM7_ESM.zip › Figure 2/Fig 2H and 2I/Fig 2H/(#11-#16) Luminal A+Luminal B/8-GAPDH-merge.Tif]

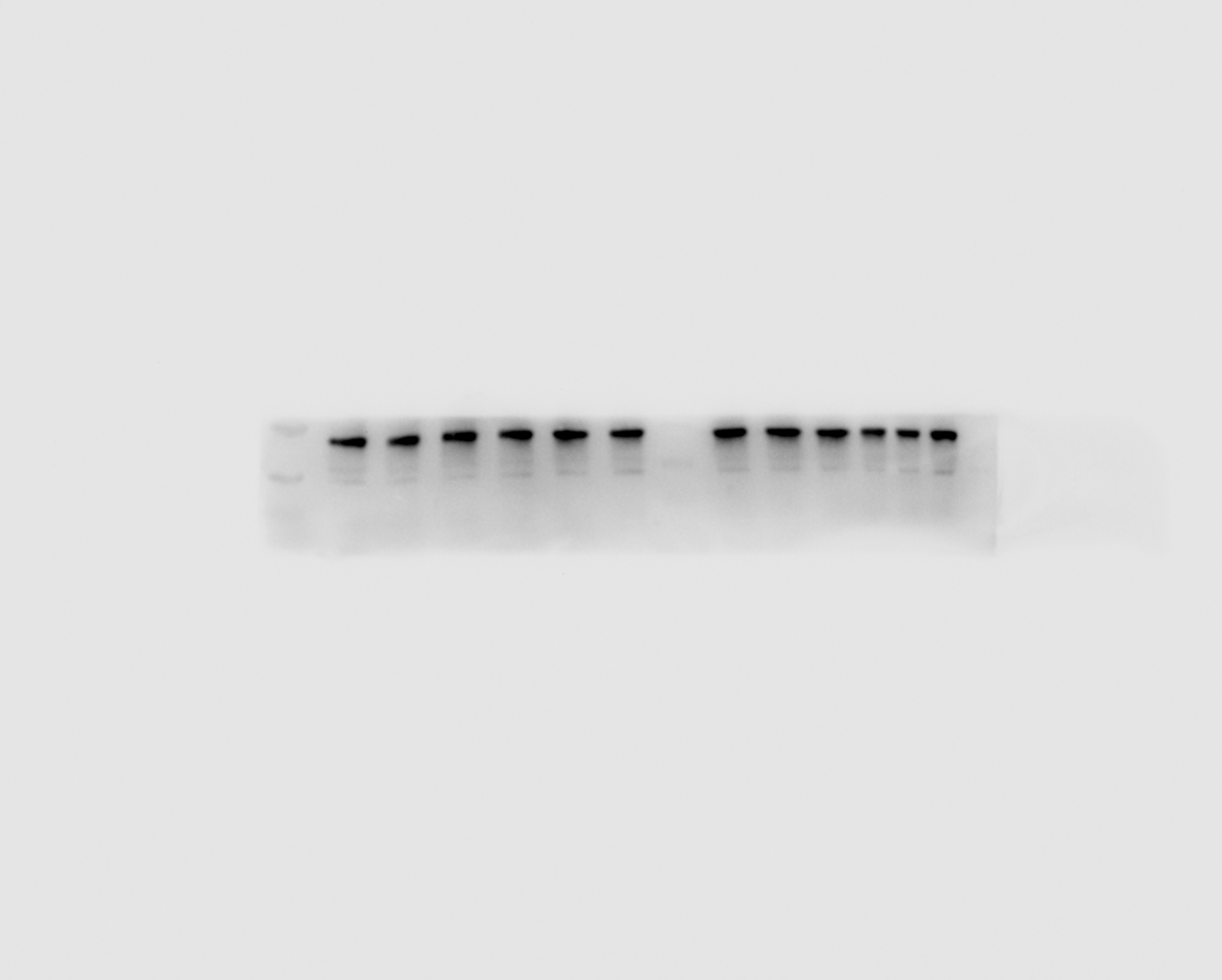

Supplement: Supplementary file 7 — Source data Fig. 2 [file 44318_2024_359_MOESM7_ESM.zip › Figure 2/Fig 2H and 2I/Fig 2H/(#11-#16) Luminal A+Luminal B/8-GAPDH.Tif]

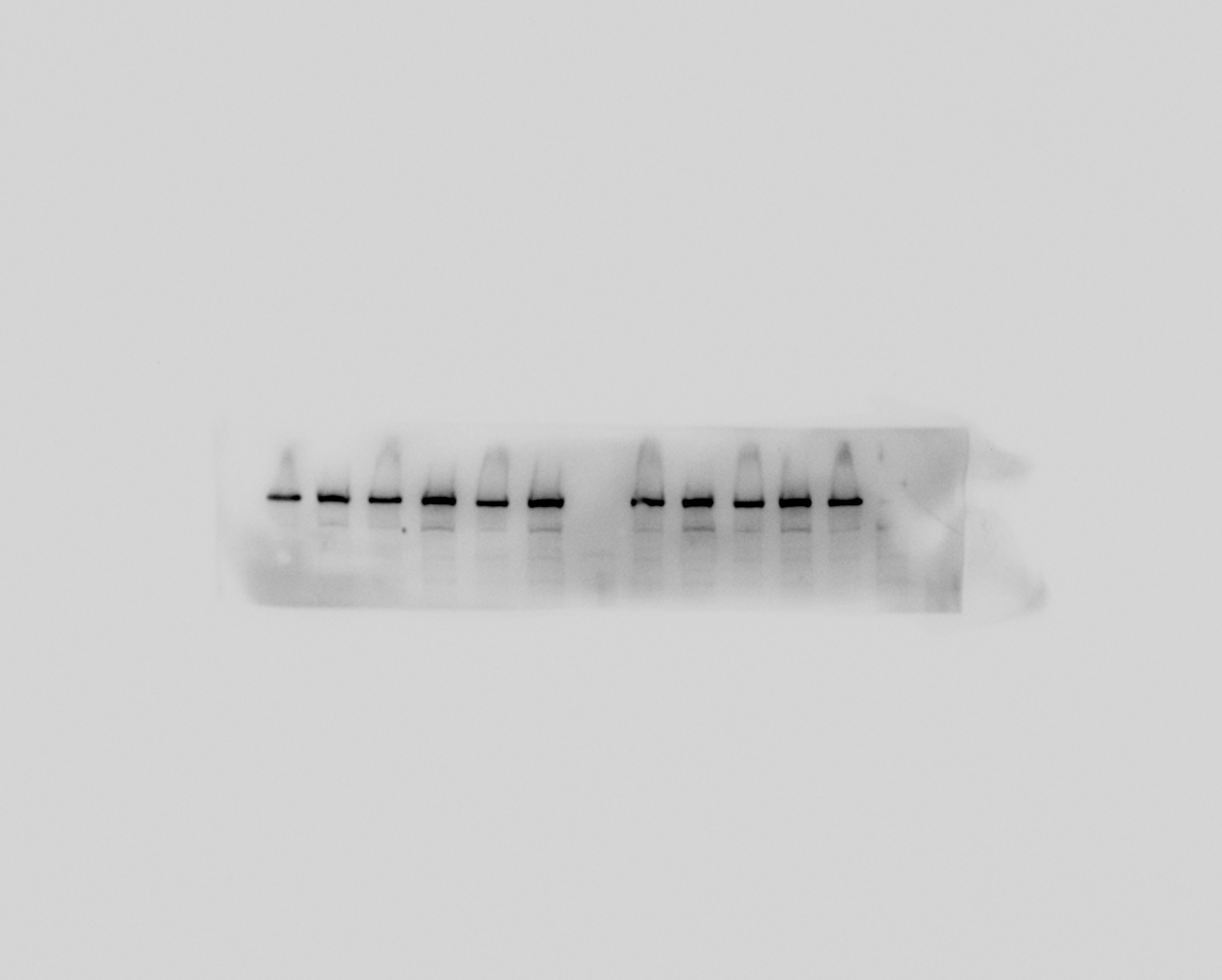

Supplement: Supplementary file 7 — Source data Fig. 2 [file 44318_2024_359_MOESM7_ESM.zip › Figure 2/Fig 2H and 2I/Fig 2H/(#8-#10 and #17-#18) Triple-negative+HER2+/1-p-mtor.Tif]

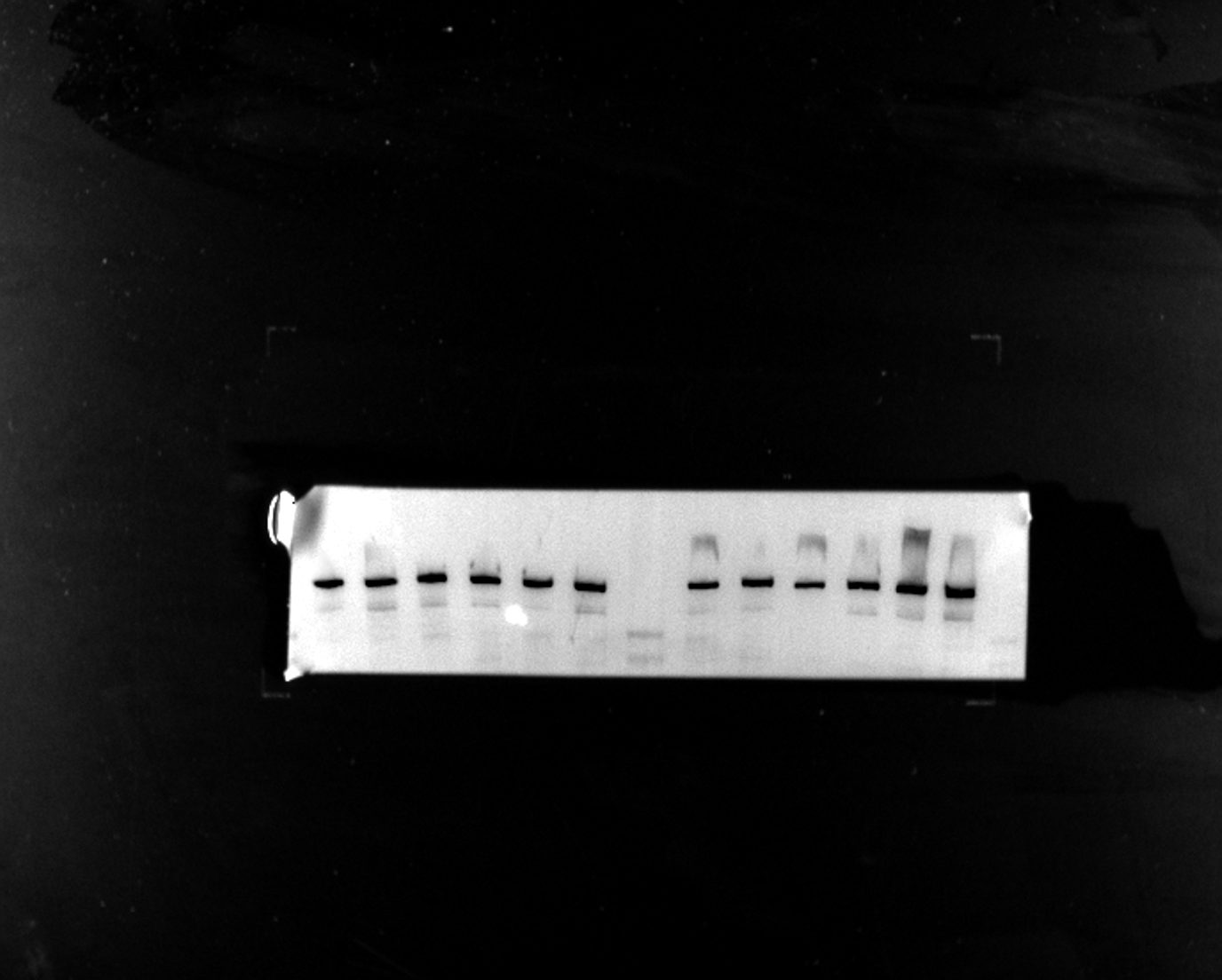

Supplement: Supplementary file 7 — Source data Fig. 2 [file 44318_2024_359_MOESM7_ESM.zip › Figure 2/Fig 2H and 2I/Fig 2H/(#8-#10 and #17-#18) Triple-negative+HER2+/2-mTOR-merge.Tif]

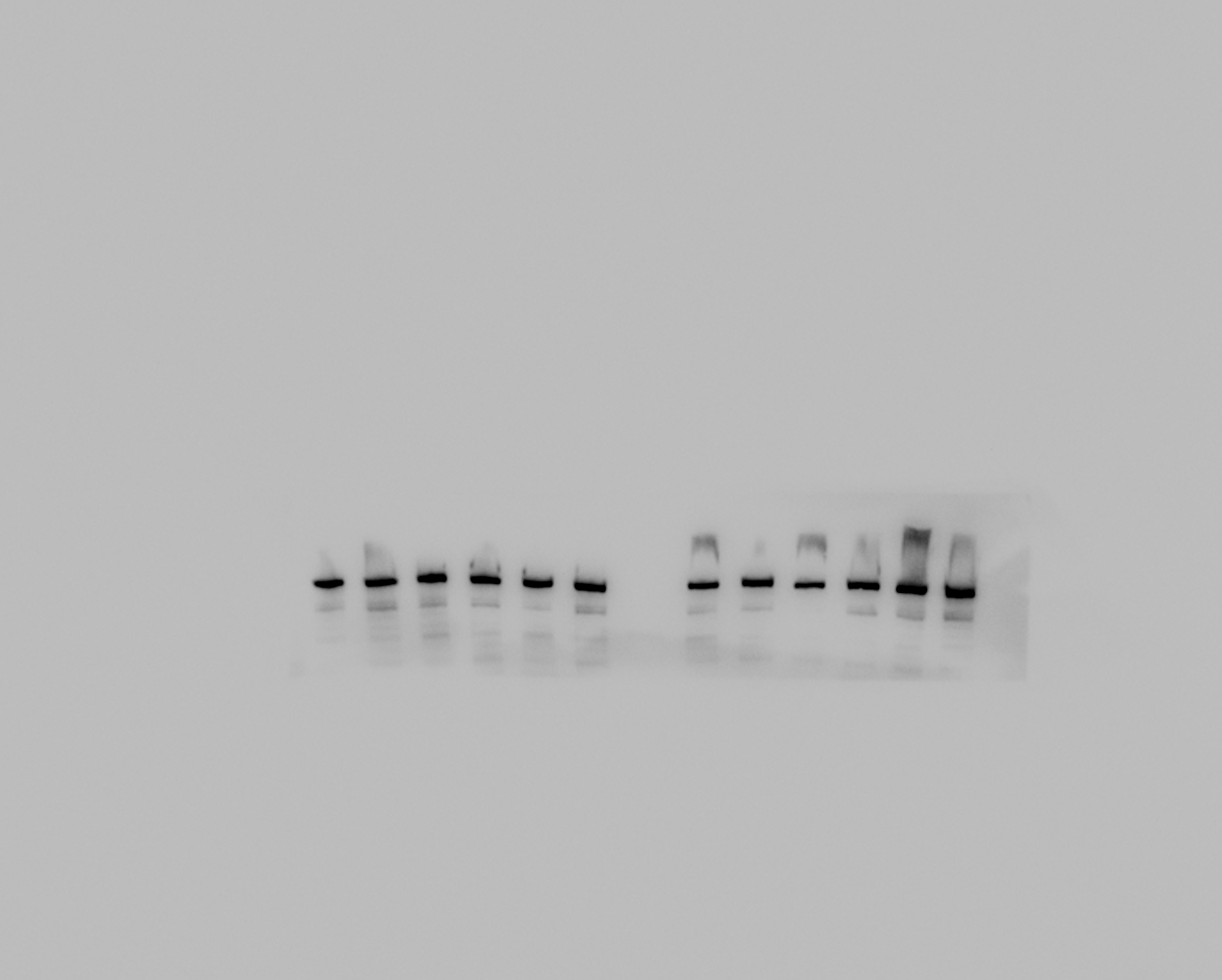

Supplement: Supplementary file 7 — Source data Fig. 2 [file 44318_2024_359_MOESM7_ESM.zip › Figure 2/Fig 2H and 2I/Fig 2H/(#8-#10 and #17-#18) Triple-negative+HER2+/2-mTOR.Tif]

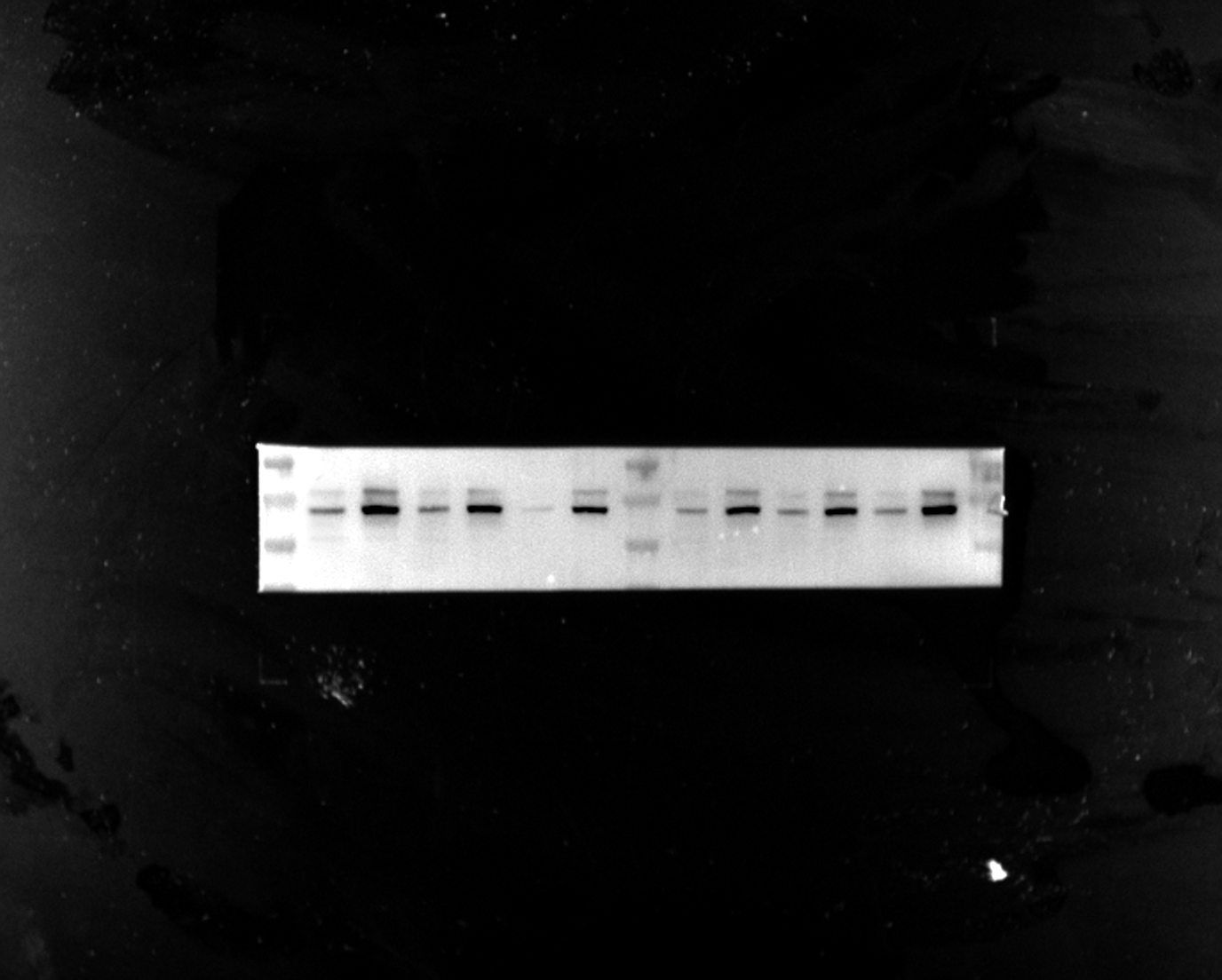

Supplement: Supplementary file 7 — Source data Fig. 2 [file 44318_2024_359_MOESM7_ESM.zip › Figure 2/Fig 2H and 2I/Fig 2H/(#8-#10 and #17-#18) Triple-negative+HER2+/3-p-S6K-merge.Tif]

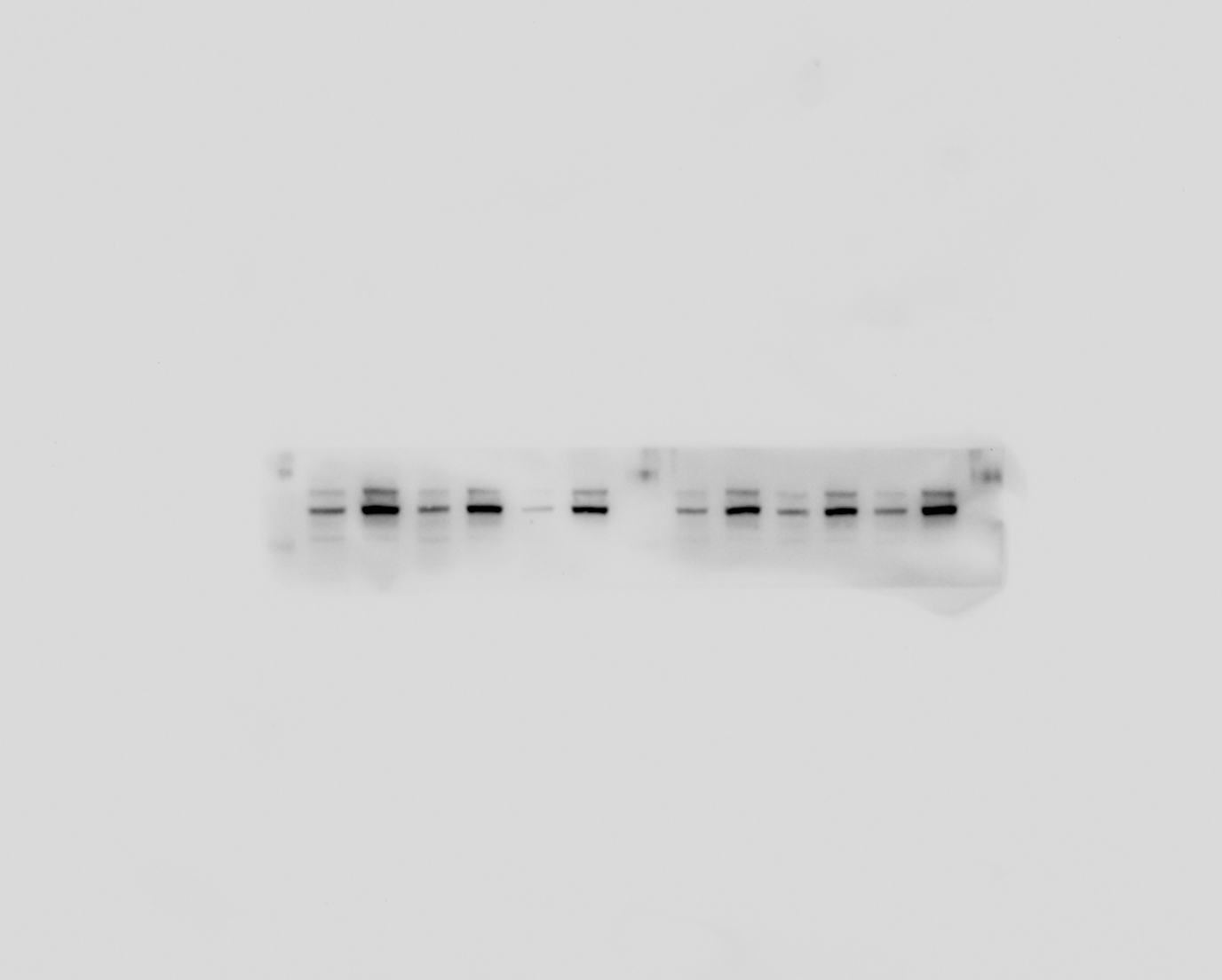

Supplement: Supplementary file 7 — Source data Fig. 2 [file 44318_2024_359_MOESM7_ESM.zip › Figure 2/Fig 2H and 2I/Fig 2H/(#8-#10 and #17-#18) Triple-negative+HER2+/3-p-S6K.Tif]

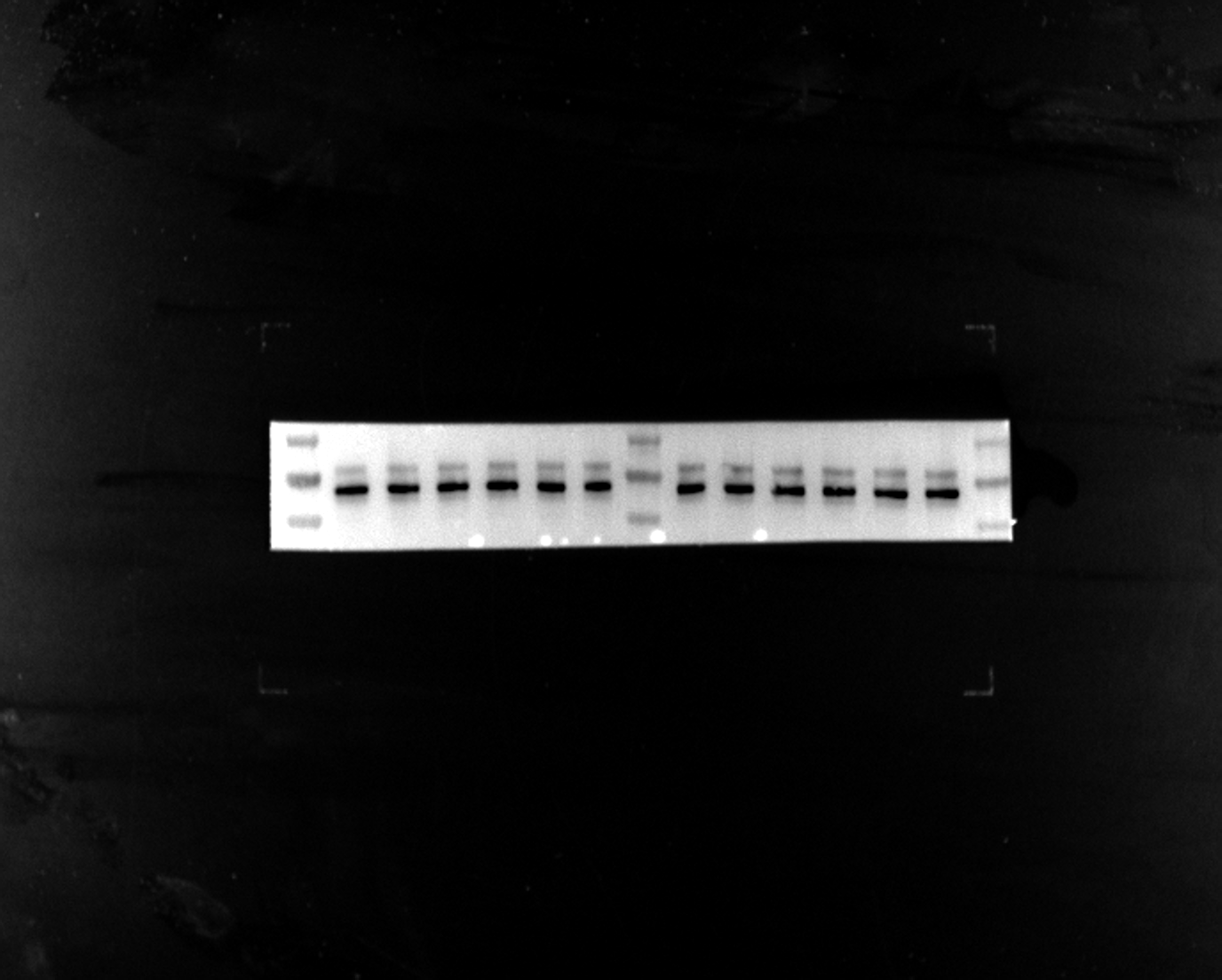

Supplement: Supplementary file 7 — Source data Fig. 2 [file 44318_2024_359_MOESM7_ESM.zip › Figure 2/Fig 2H and 2I/Fig 2H/(#8-#10 and #17-#18) Triple-negative+HER2+/4-S6K-merge.Tif]

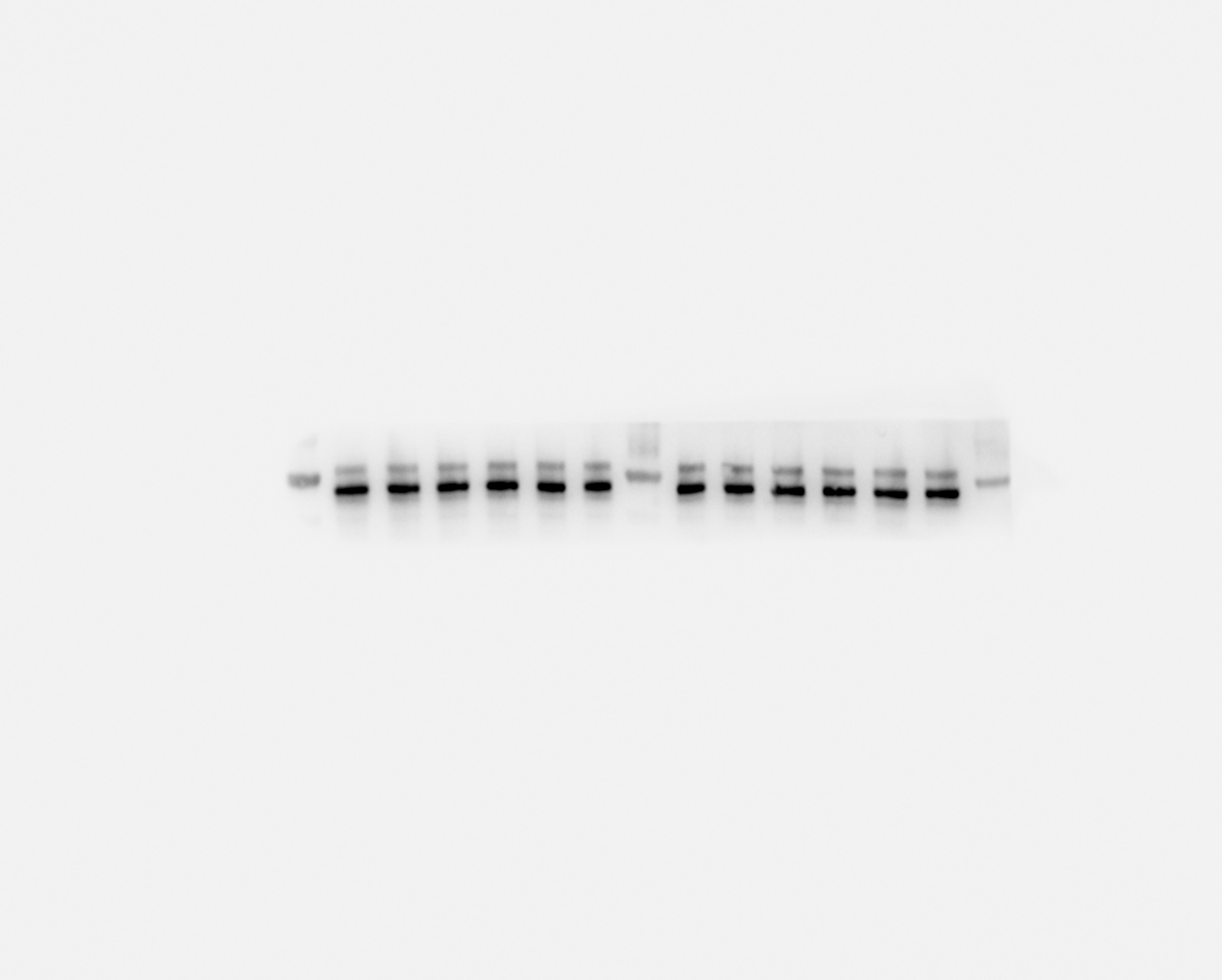

Supplement: Supplementary file 7 — Source data Fig. 2 [file 44318_2024_359_MOESM7_ESM.zip › Figure 2/Fig 2H and 2I/Fig 2H/(#8-#10 and #17-#18) Triple-negative+HER2+/4-S6K.Tif]

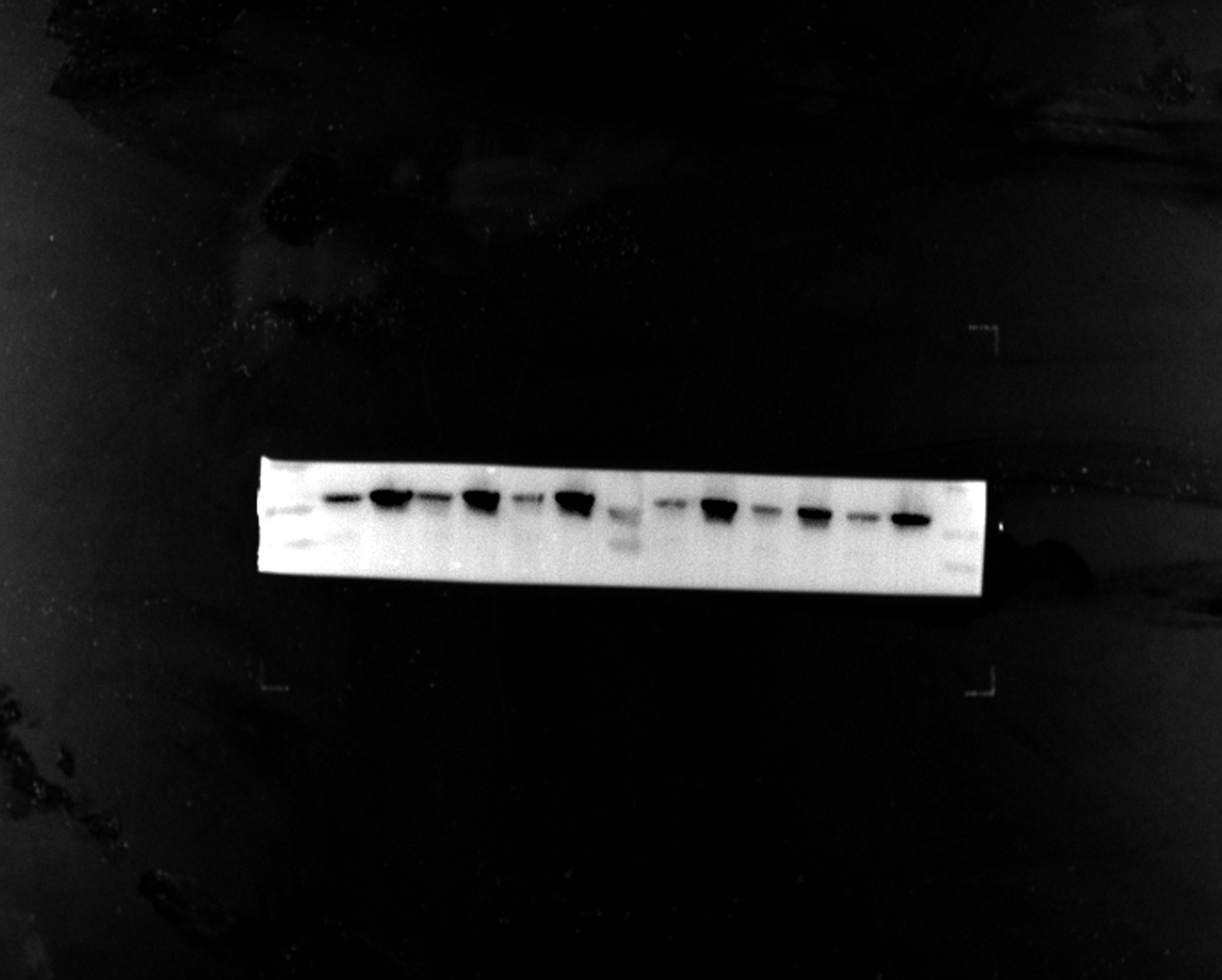

Supplement: Supplementary file 7 — Source data Fig. 2 [file 44318_2024_359_MOESM7_ESM.zip › Figure 2/Fig 2H and 2I/Fig 2H/(#8-#10 and #17-#18) Triple-negative+HER2+/5-p-S6-merge.Tif]

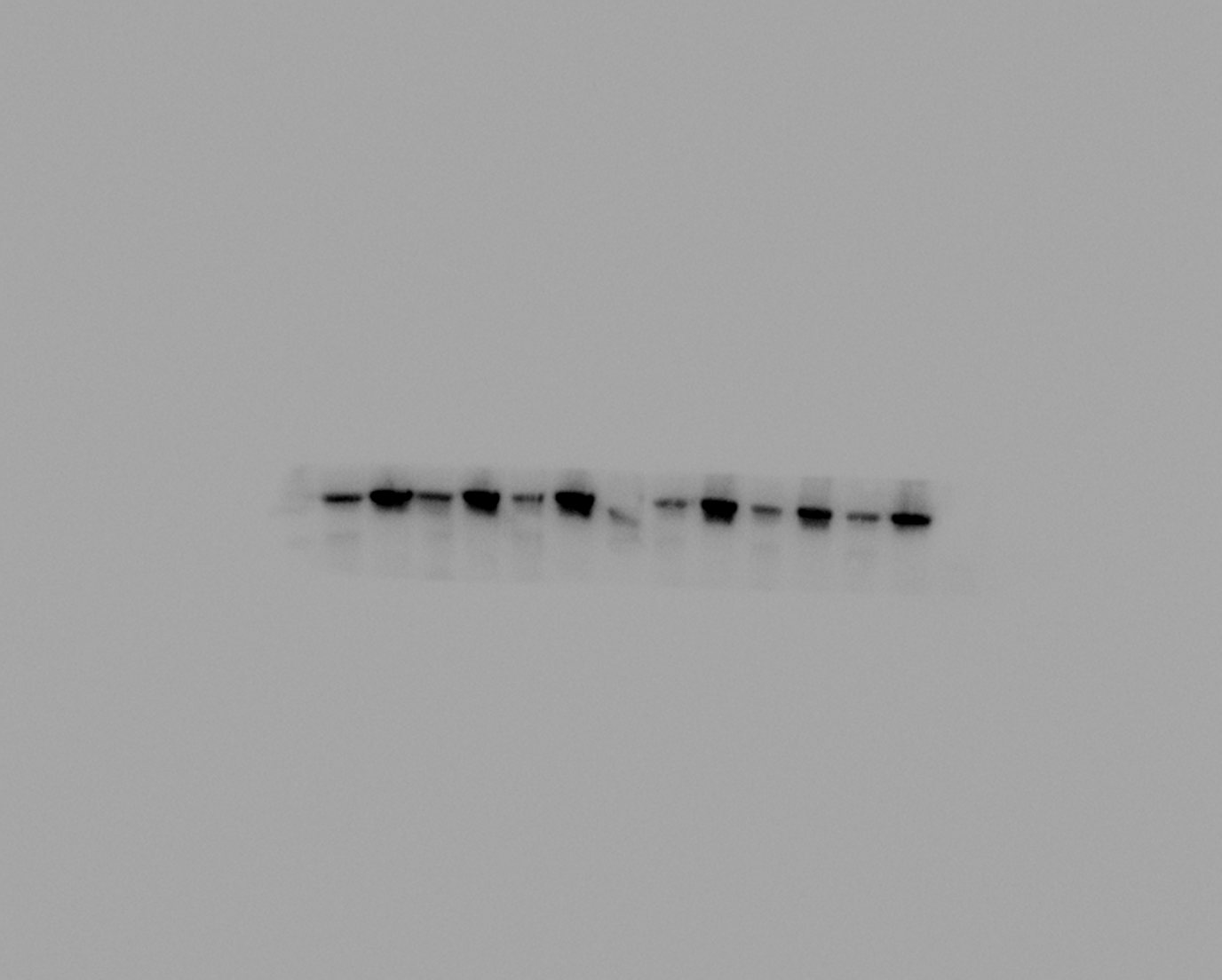

Supplement: Supplementary file 7 — Source data Fig. 2 [file 44318_2024_359_MOESM7_ESM.zip › Figure 2/Fig 2H and 2I/Fig 2H/(#8-#10 and #17-#18) Triple-negative+HER2+/5-p-S6.Tif]

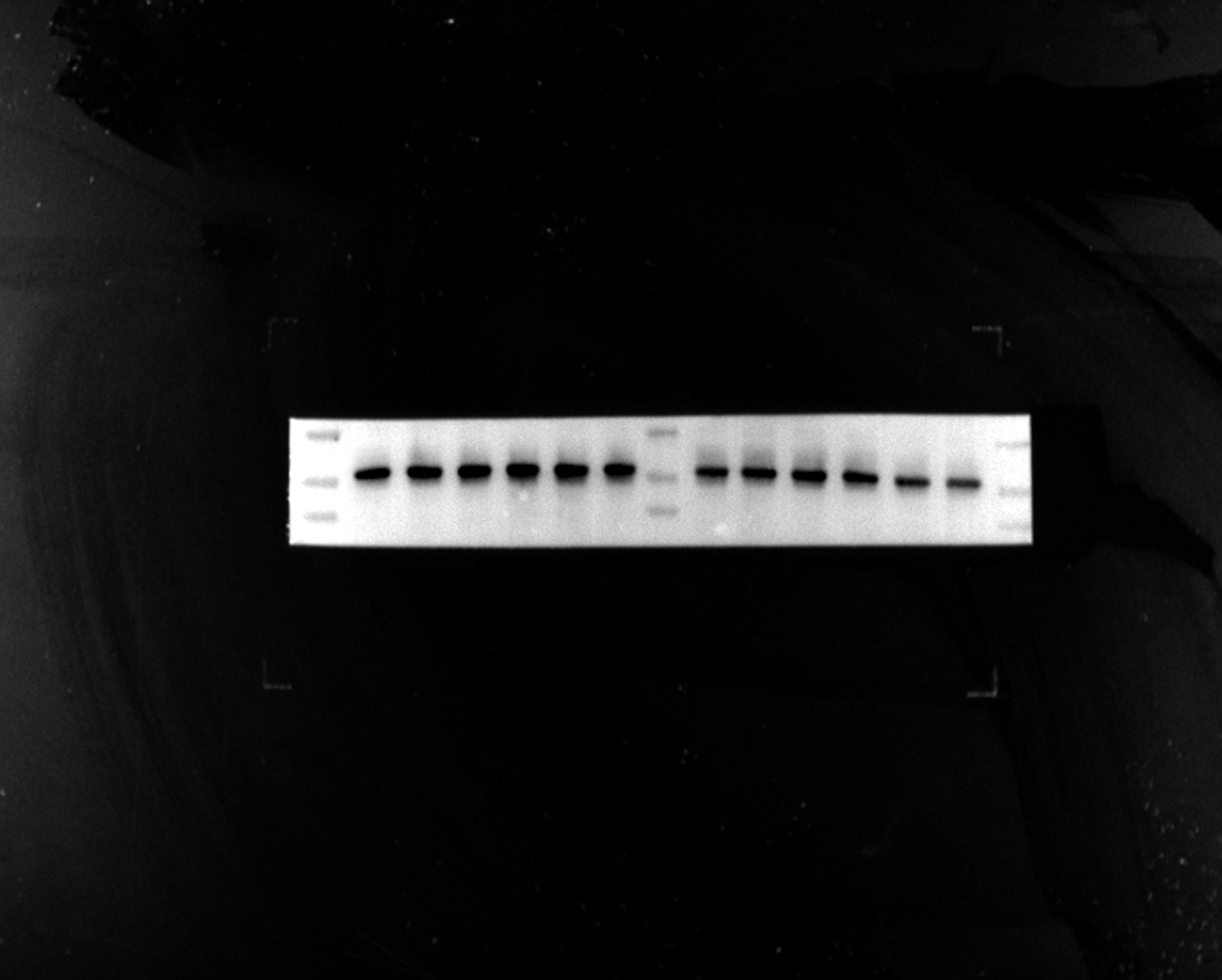

Supplement: Supplementary file 7 — Source data Fig. 2 [file 44318_2024_359_MOESM7_ESM.zip › Figure 2/Fig 2H and 2I/Fig 2H/(#8-#10 and #17-#18) Triple-negative+HER2+/6-S6-merge.Tif]

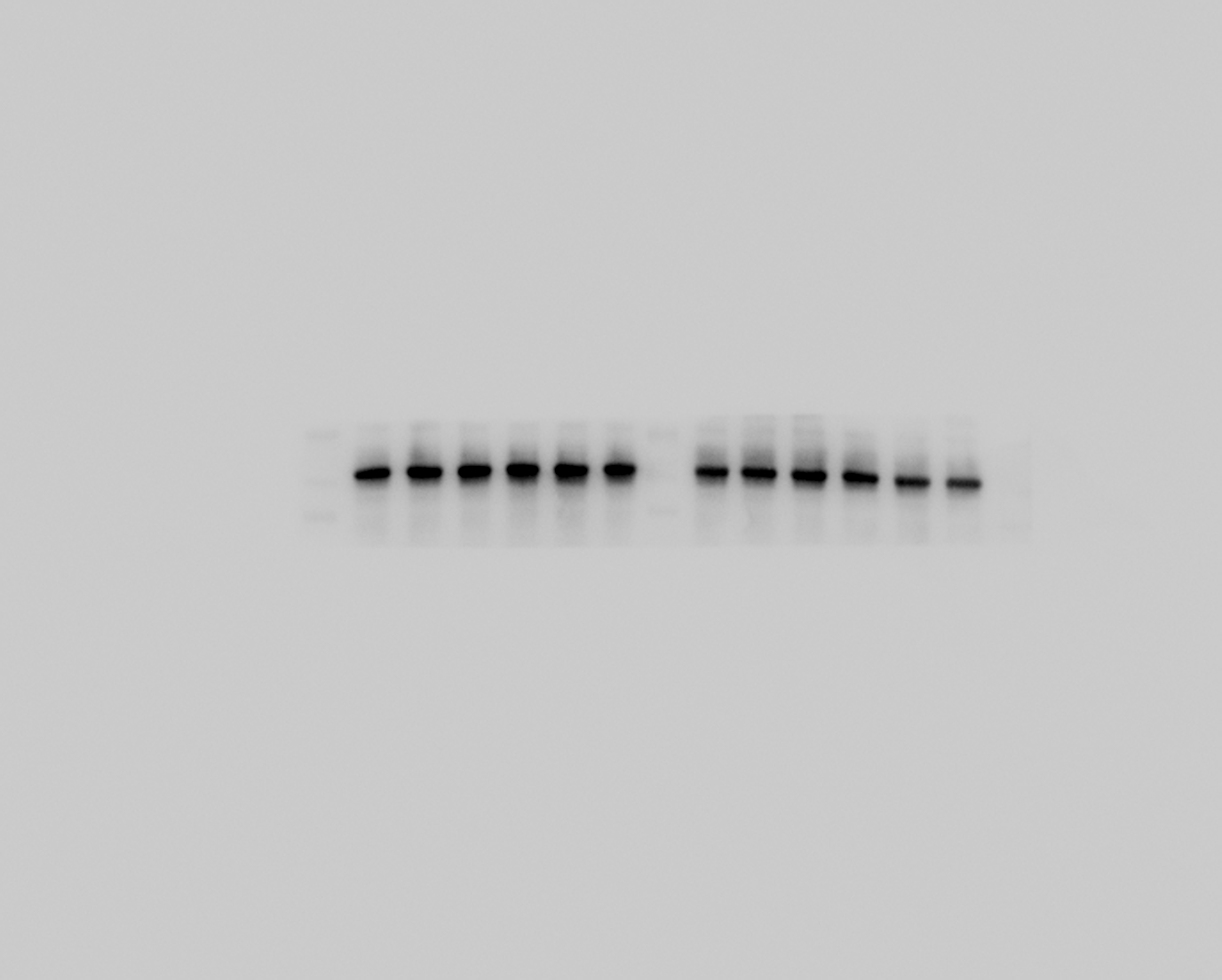

Supplement: Supplementary file 7 — Source data Fig. 2 [file 44318_2024_359_MOESM7_ESM.zip › Figure 2/Fig 2H and 2I/Fig 2H/(#8-#10 and #17-#18) Triple-negative+HER2+/6-S6.Tif]

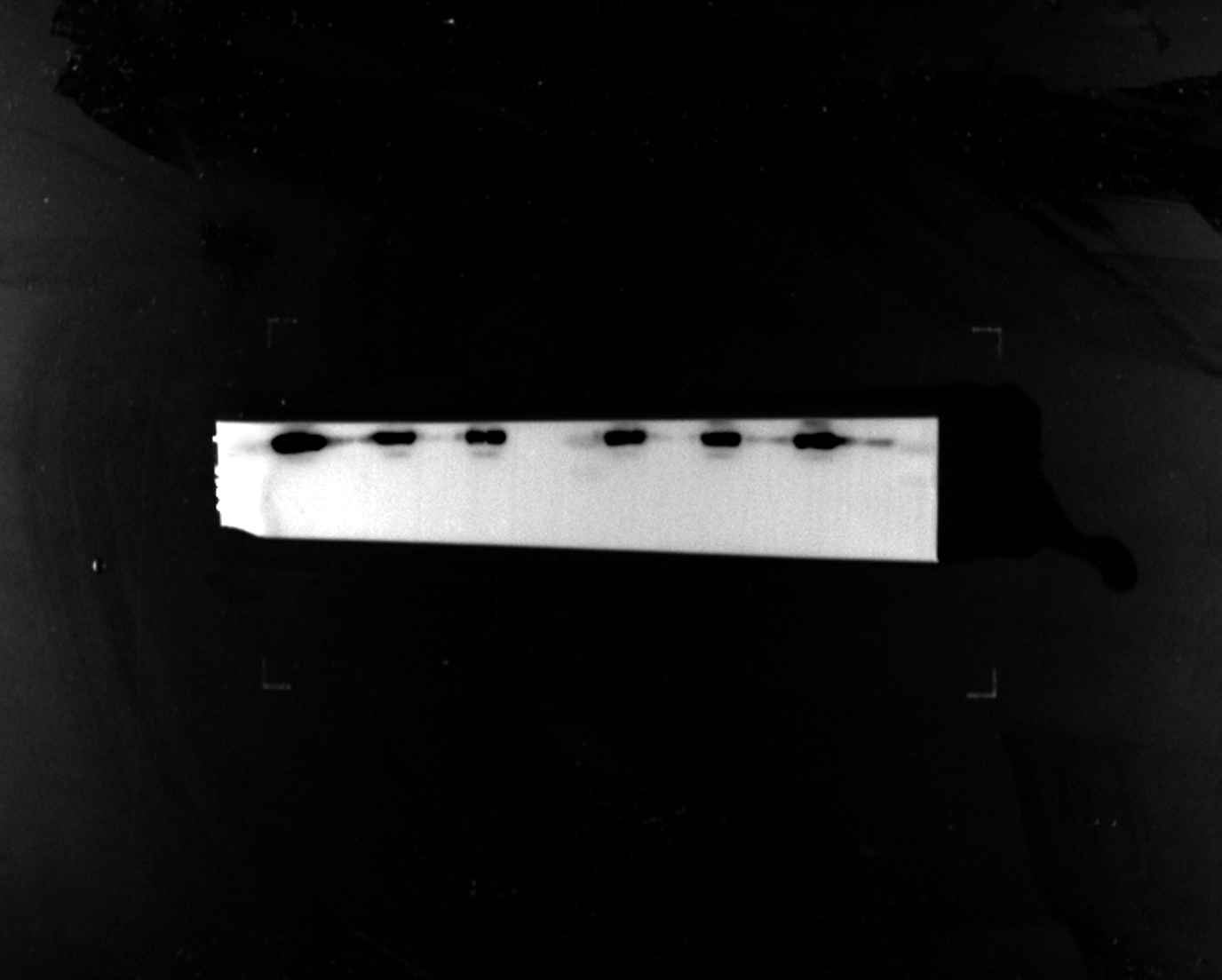

Supplement: Supplementary file 7 — Source data Fig. 2 [file 44318_2024_359_MOESM7_ESM.zip › Figure 2/Fig 2H and 2I/Fig 2H/(#8-#10 and #17-#18) Triple-negative+HER2+/7-hSPAR-merge.Tif]

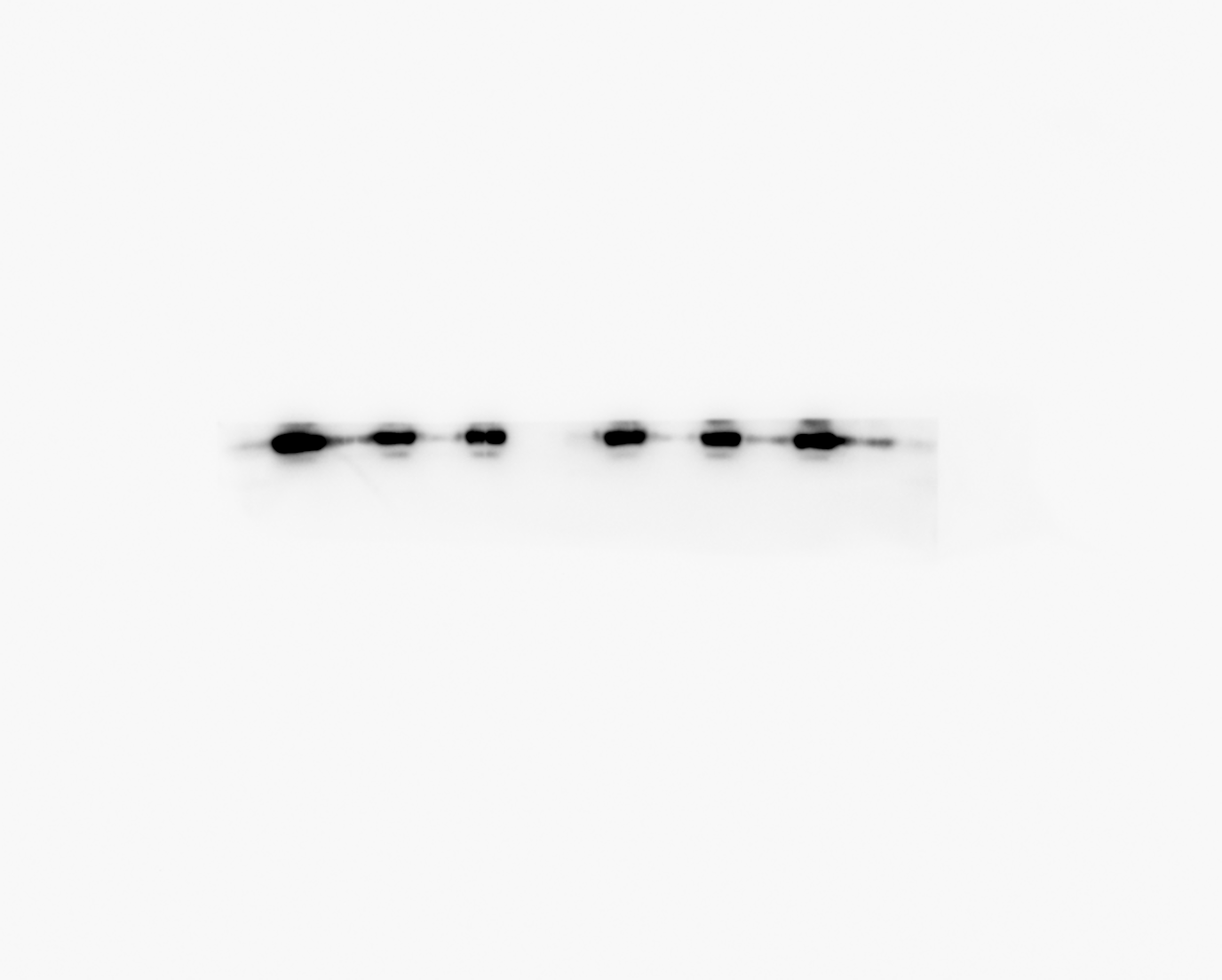

Supplement: Supplementary file 7 — Source data Fig. 2 [file 44318_2024_359_MOESM7_ESM.zip › Figure 2/Fig 2H and 2I/Fig 2H/(#8-#10 and #17-#18) Triple-negative+HER2+/7-hSPAR.Tif]

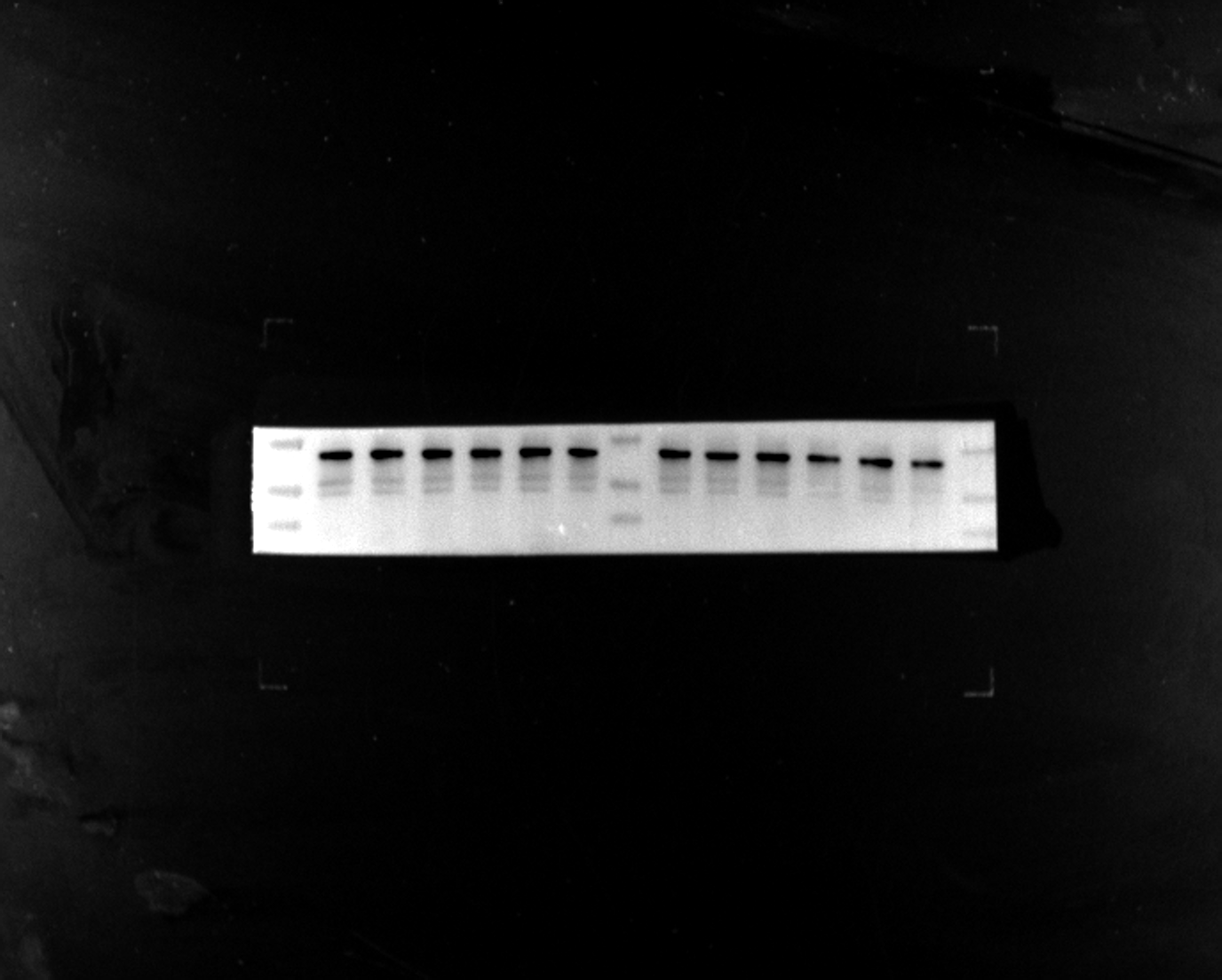

Supplement: Supplementary file 7 — Source data Fig. 2 [file 44318_2024_359_MOESM7_ESM.zip › Figure 2/Fig 2H and 2I/Fig 2H/(#8-#10 and #17-#18) Triple-negative+HER2+/8-GAPDH-merge.Tif]

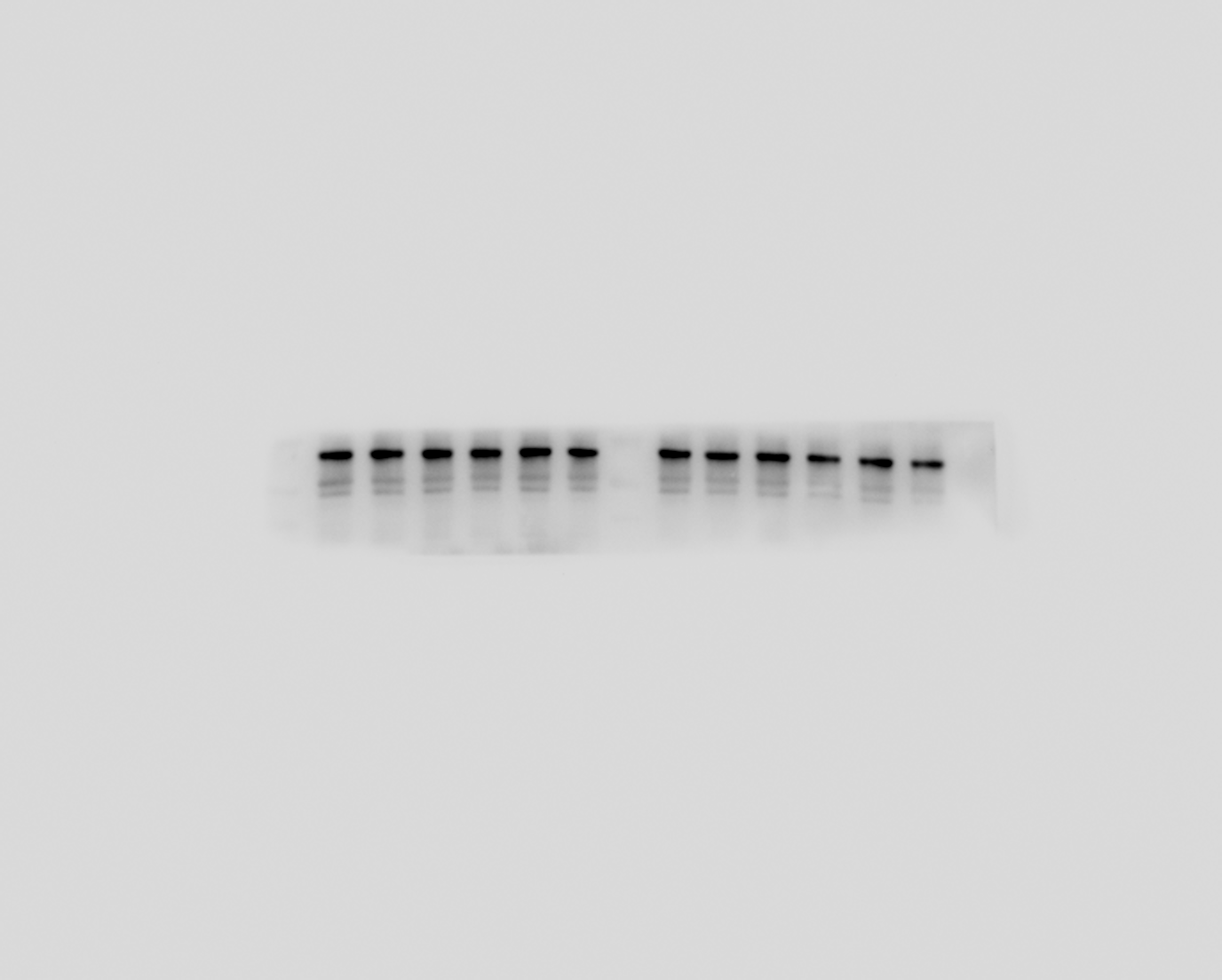

Supplement: Supplementary file 7 — Source data Fig. 2 [file 44318_2024_359_MOESM7_ESM.zip › Figure 2/Fig 2H and 2I/Fig 2H/(#8-#10 and #17-#18) Triple-negative+HER2+/8-GAPDH.Tif]

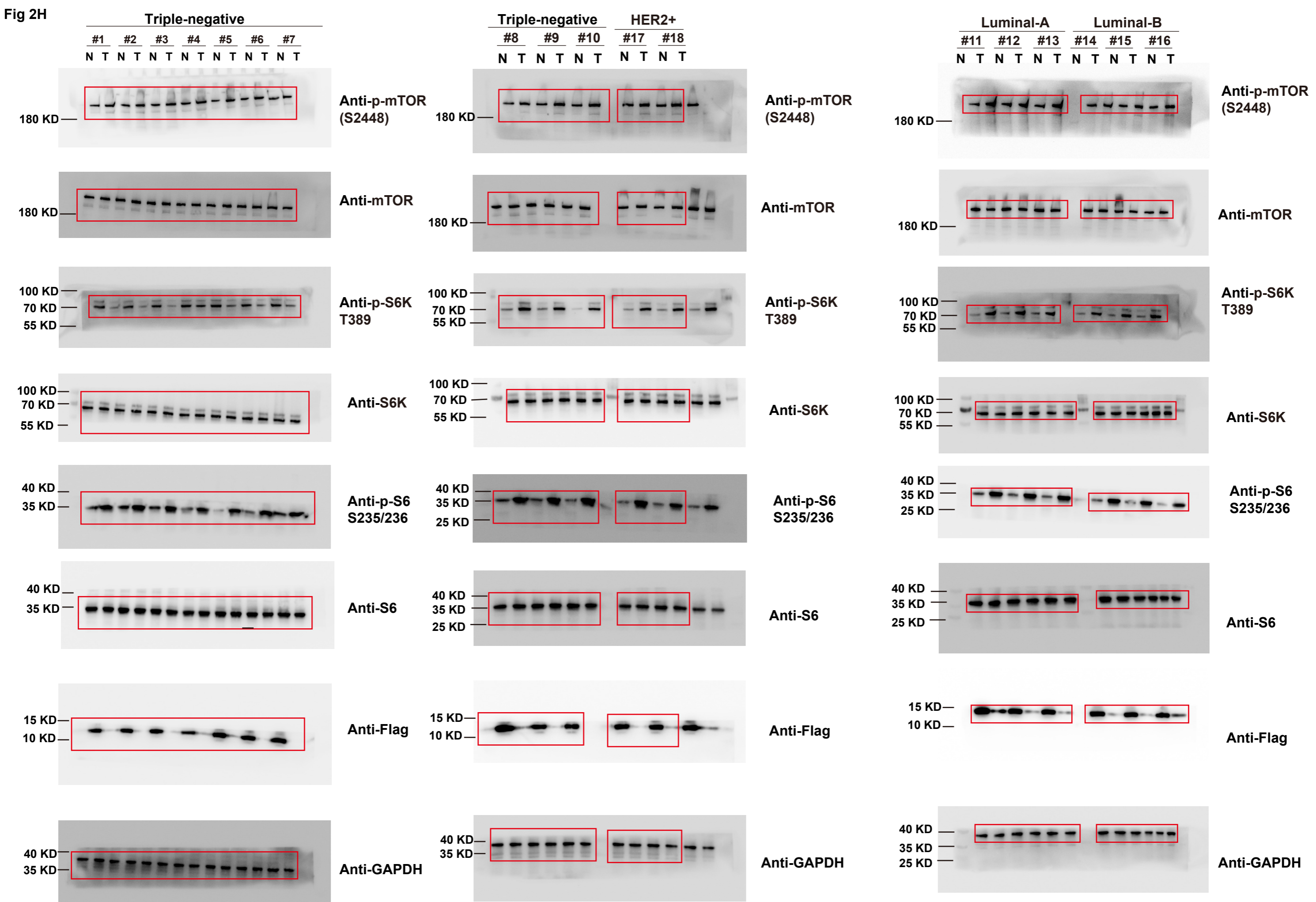

Supplement: Supplementary file 7 — Source data Fig. 2 [file 44318_2024_359_MOESM7_ESM.zip › Figure 2/Fig 2H and 2I/Fig 2H/Fig 2H.pdf]

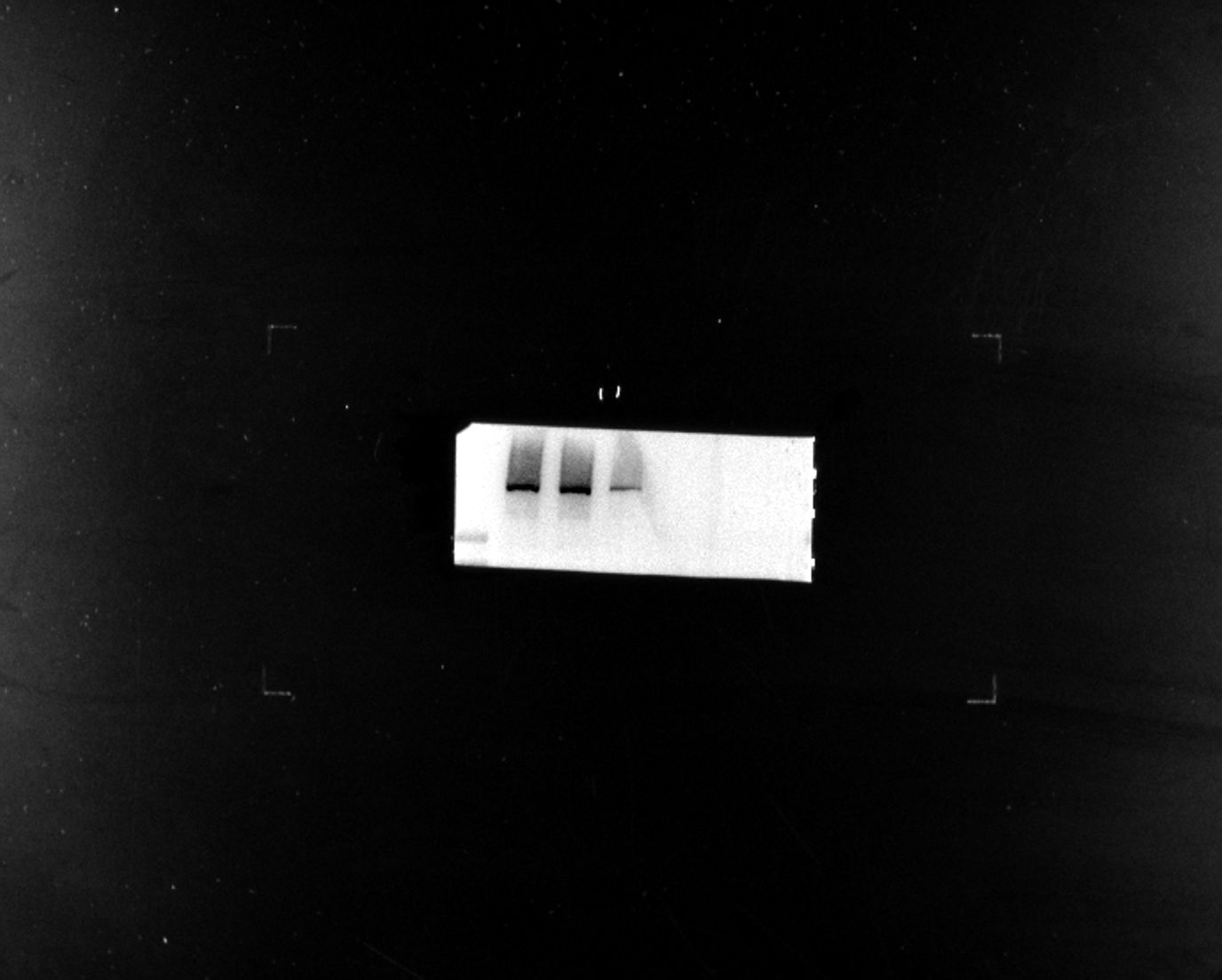

Supplement: Supplementary file 7 — Source data Fig. 2 [file 44318_2024_359_MOESM7_ESM.zip › Figure 2/Fig 2J/1-p-mTOR-merge.Tif]

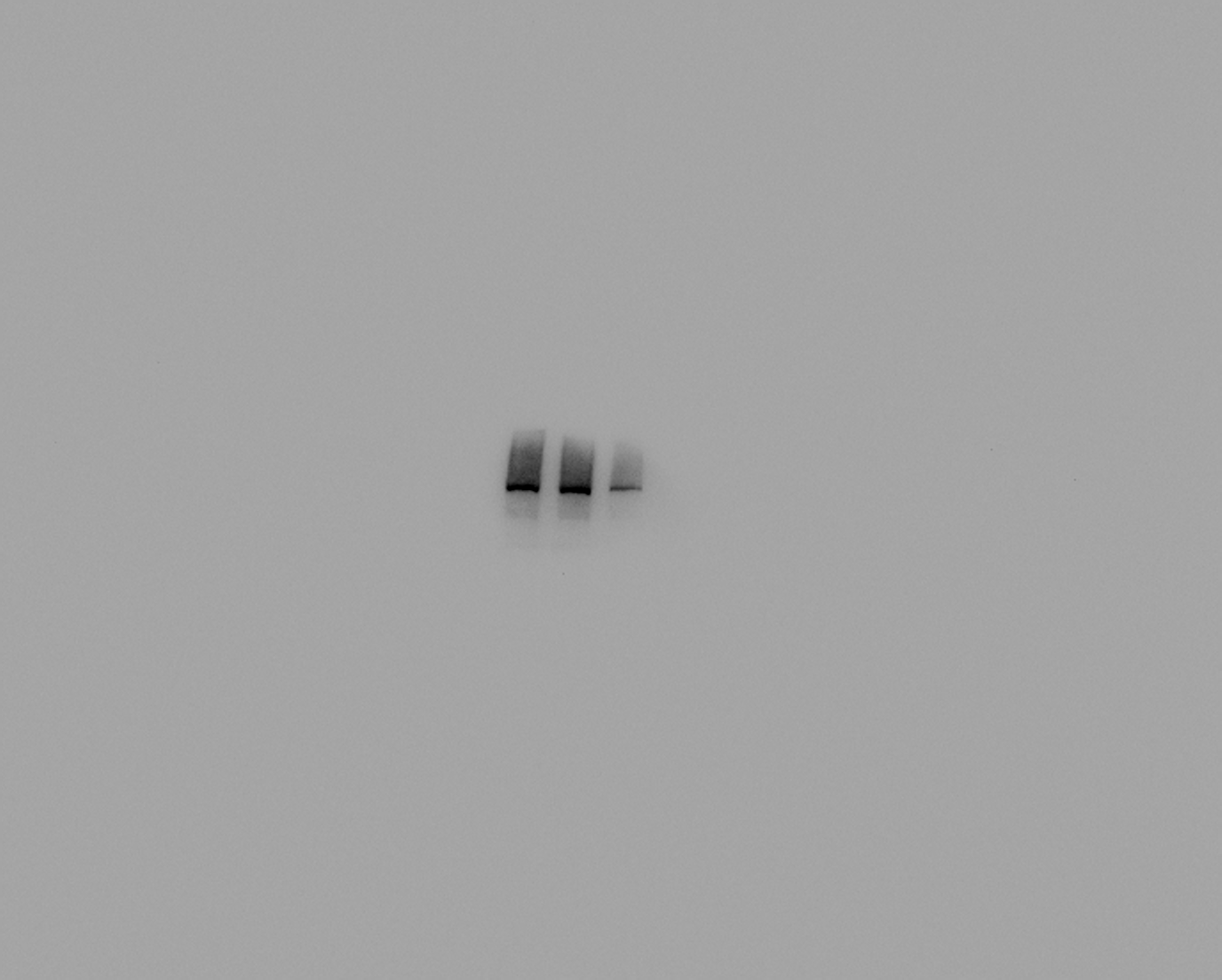

Supplement: Supplementary file 7 — Source data Fig. 2 [file 44318_2024_359_MOESM7_ESM.zip › Figure 2/Fig 2J/1-p-mTOR.Tif]

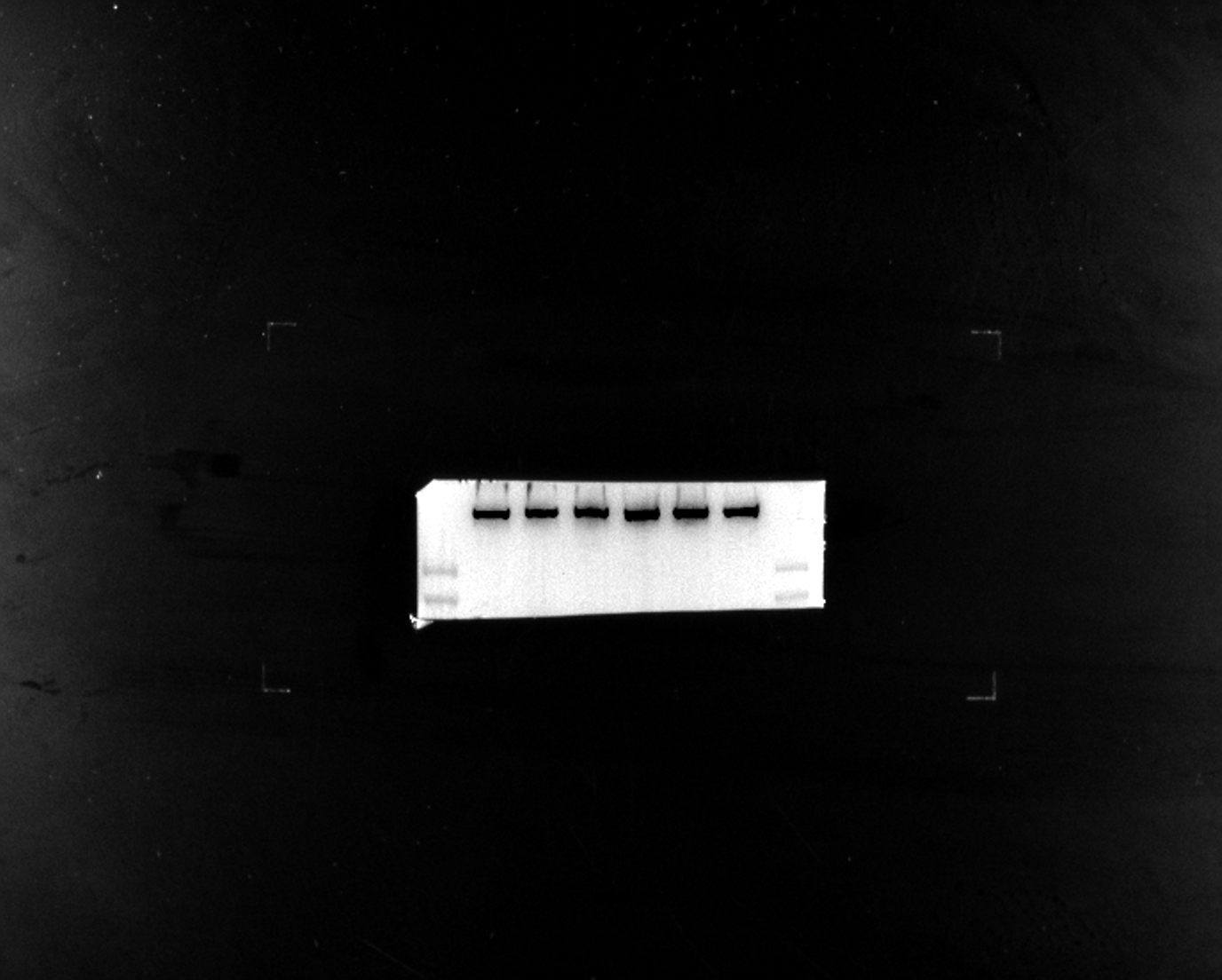

Supplement: Supplementary file 7 — Source data Fig. 2 [file 44318_2024_359_MOESM7_ESM.zip › Figure 2/Fig 2J/2-mTOR-merge.Tif]

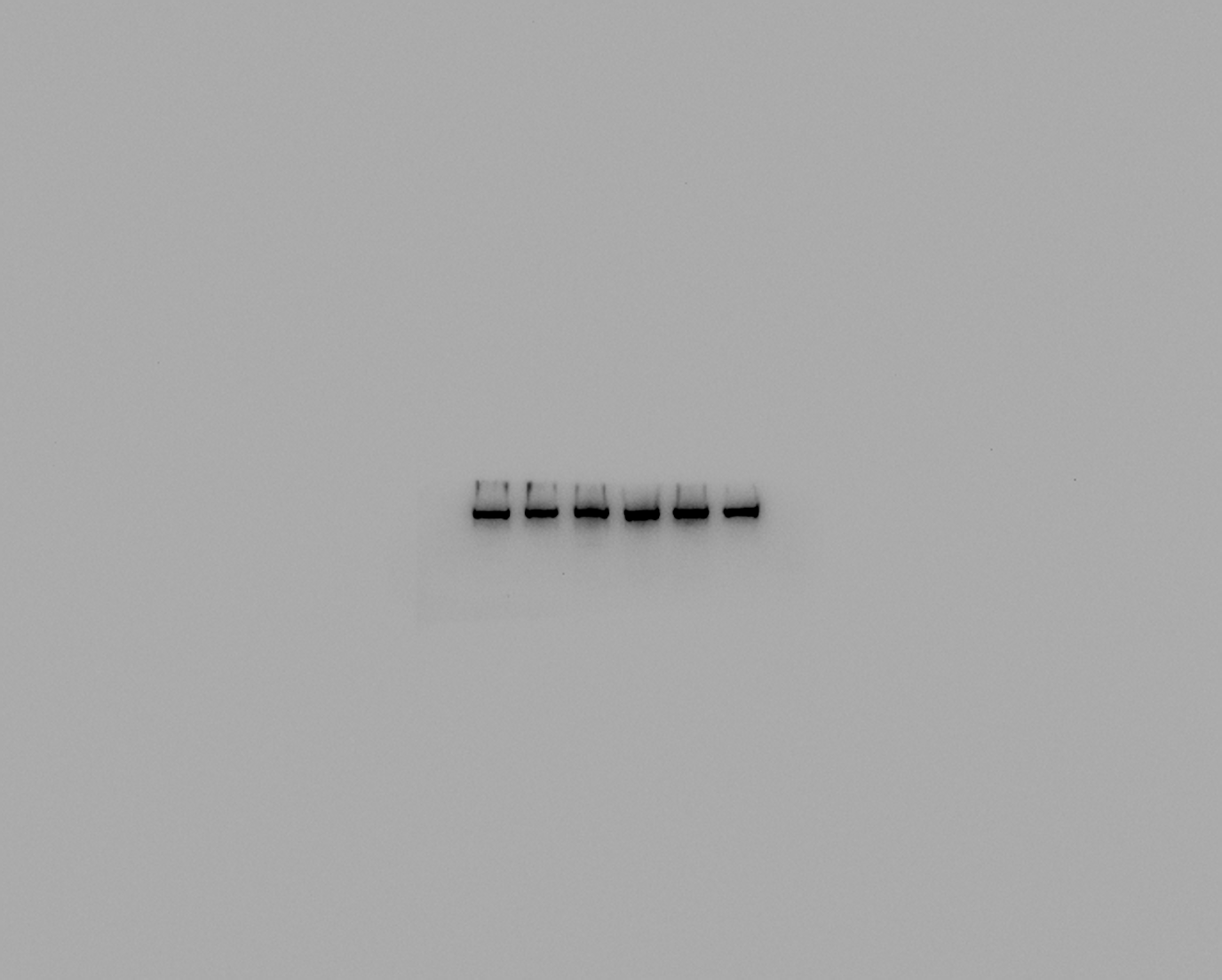

Supplement: Supplementary file 7 — Source data Fig. 2 [file 44318_2024_359_MOESM7_ESM.zip › Figure 2/Fig 2J/2-mTOR.Tif]

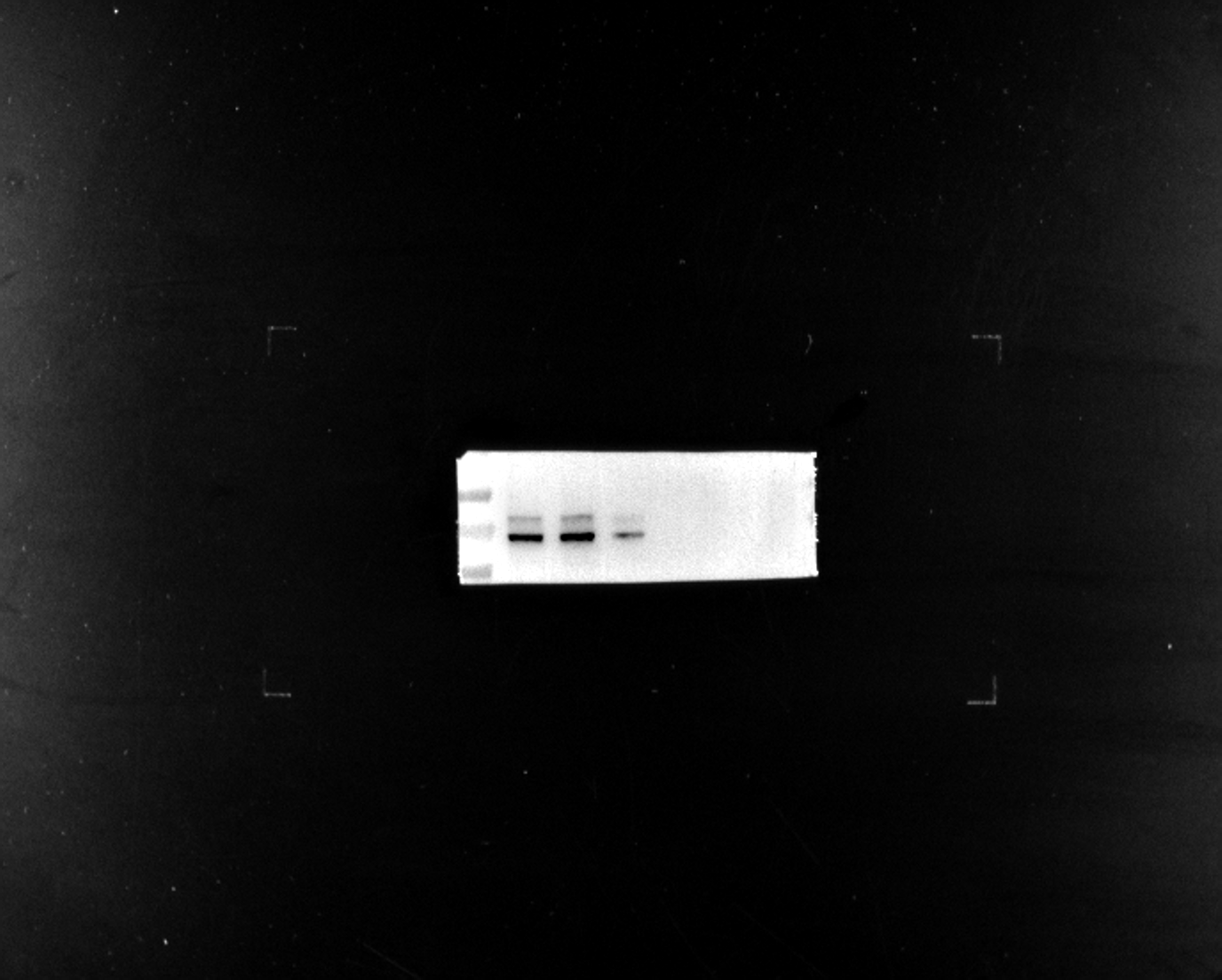

Supplement: Supplementary file 7 — Source data Fig. 2 [file 44318_2024_359_MOESM7_ESM.zip › Figure 2/Fig 2J/3-p-S6K-merge.Tif]

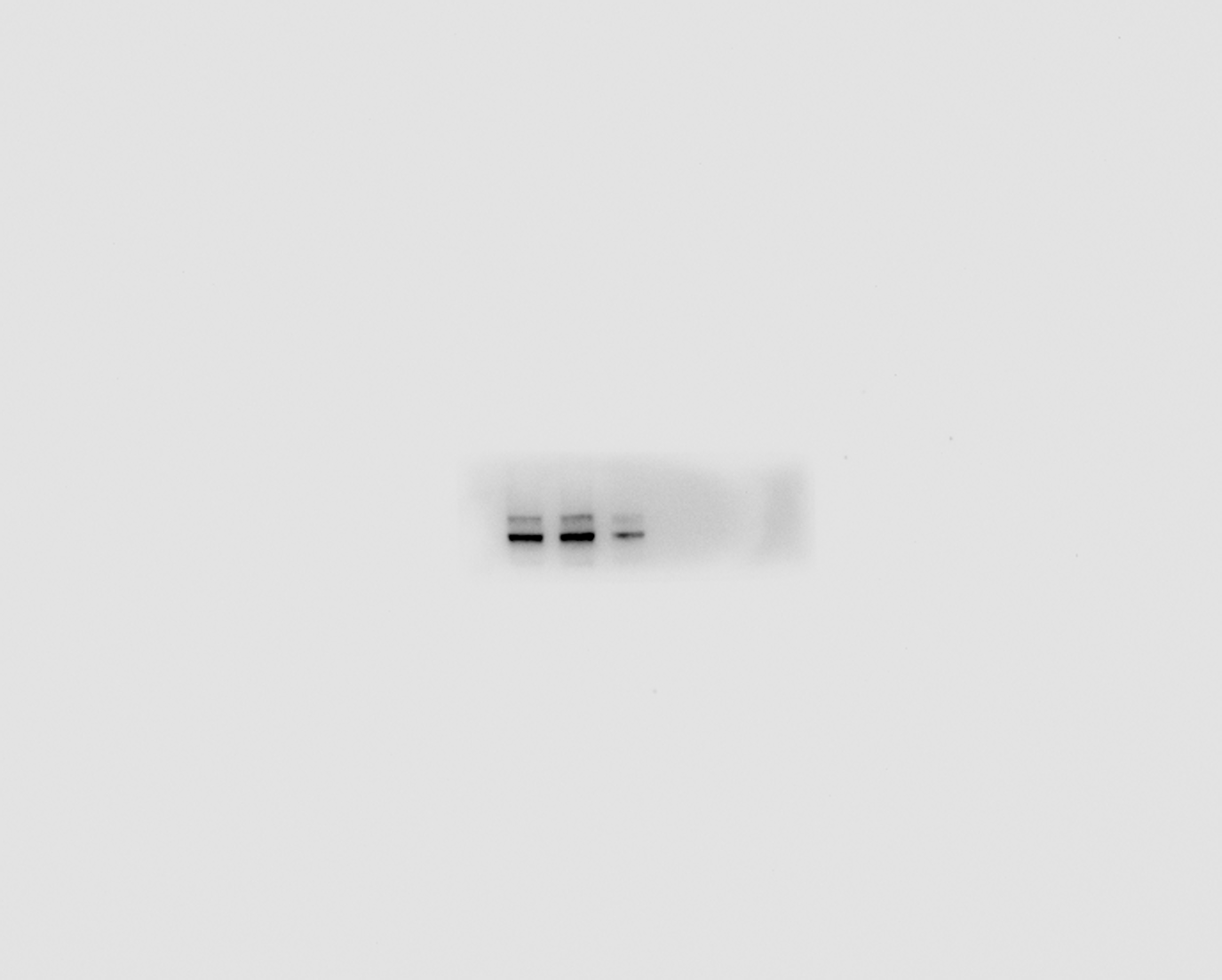

Supplement: Supplementary file 7 — Source data Fig. 2 [file 44318_2024_359_MOESM7_ESM.zip › Figure 2/Fig 2J/3-p-S6K.Tif]

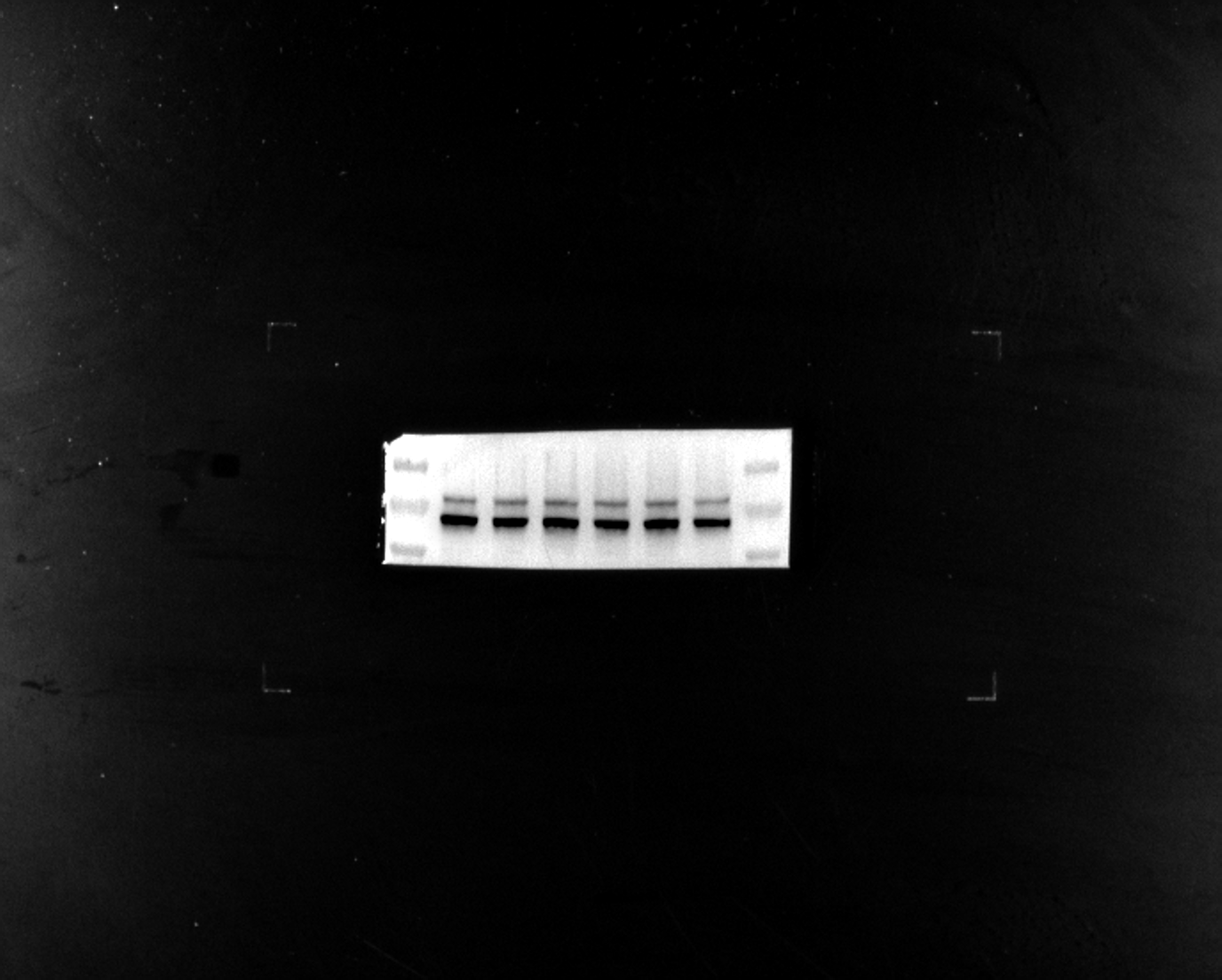

Supplement: Supplementary file 7 — Source data Fig. 2 [file 44318_2024_359_MOESM7_ESM.zip › Figure 2/Fig 2J/4-S6K-merge.Tif]

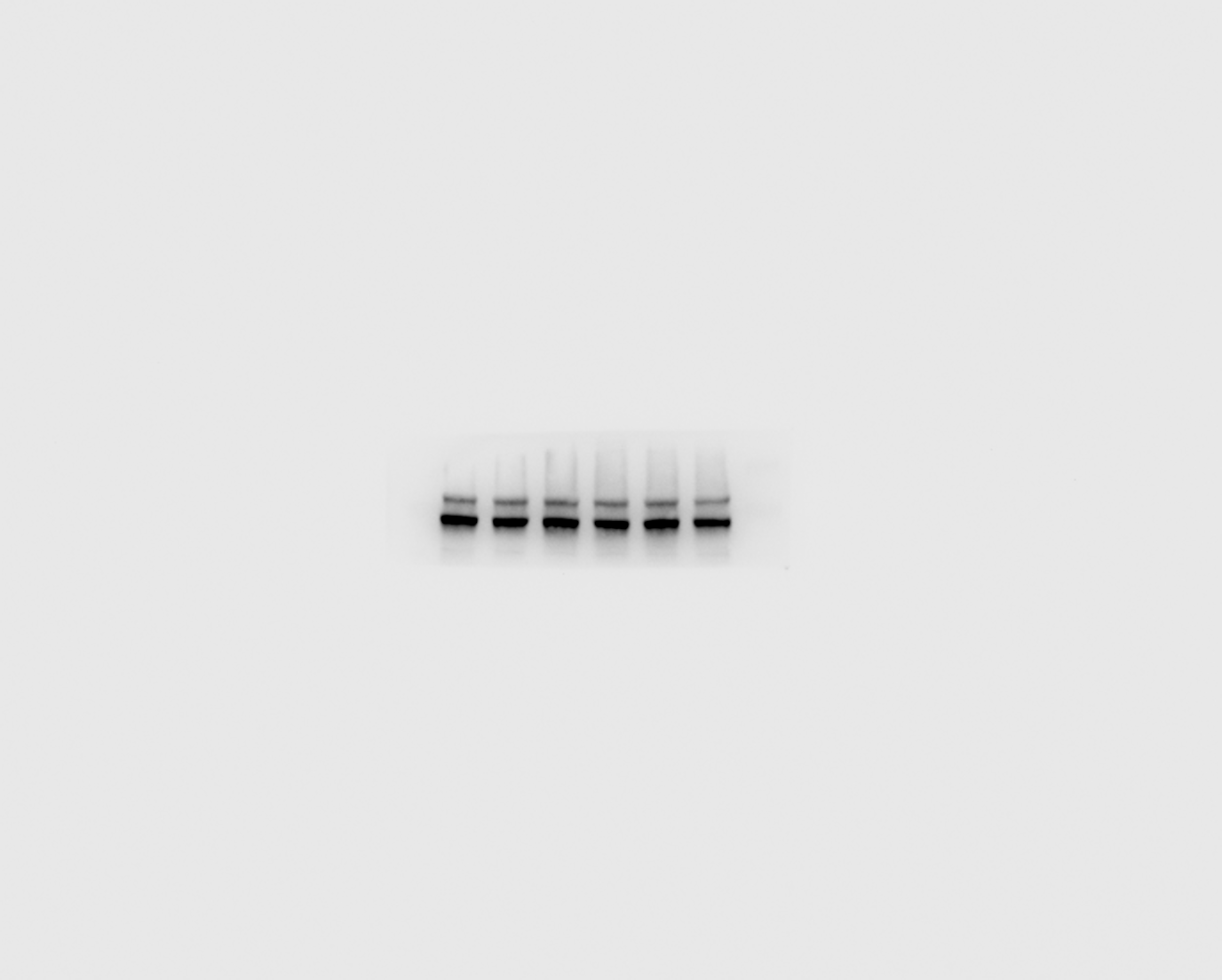

Supplement: Supplementary file 7 — Source data Fig. 2 [file 44318_2024_359_MOESM7_ESM.zip › Figure 2/Fig 2J/4-S6K.Tif]

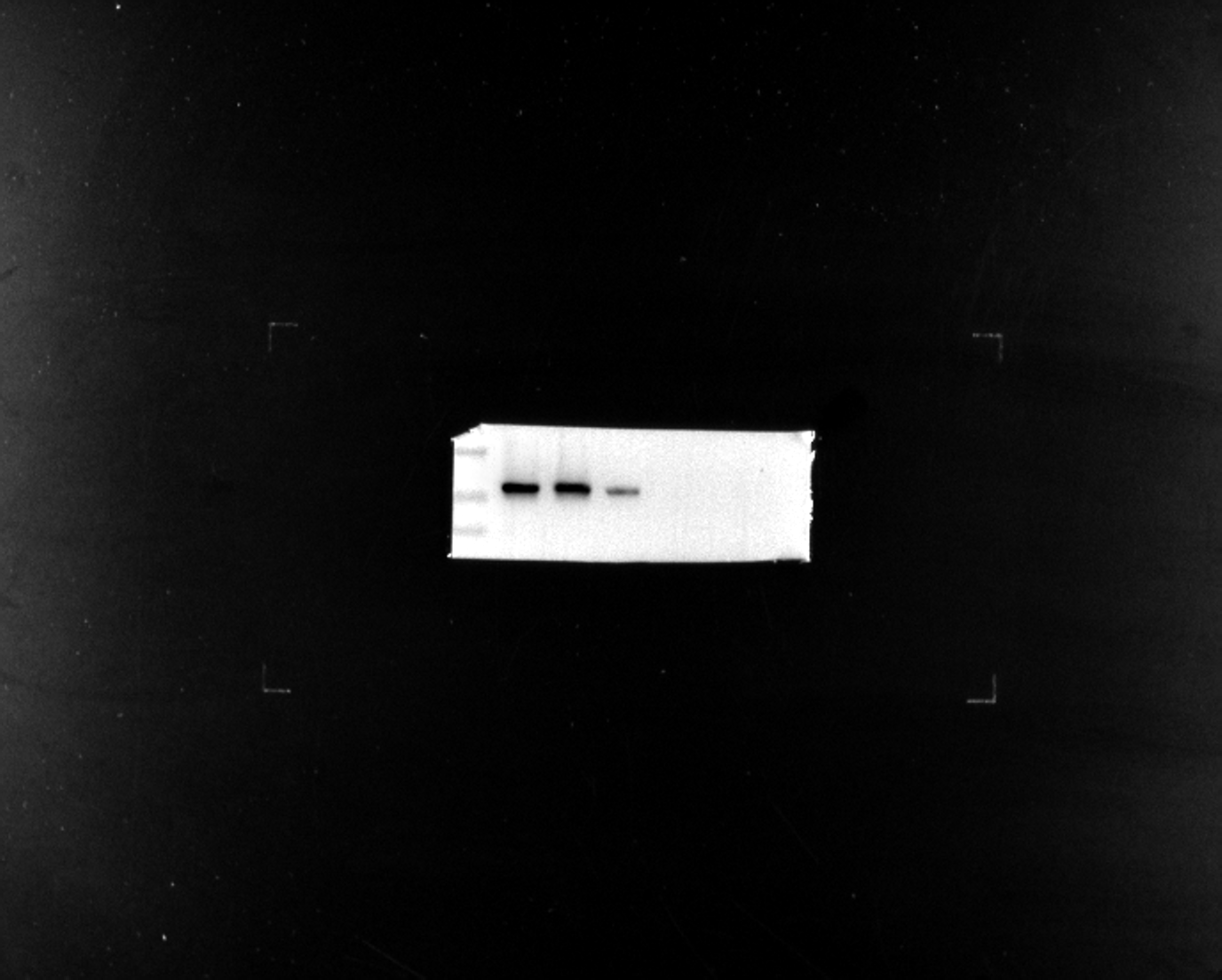

Supplement: Supplementary file 7 — Source data Fig. 2 [file 44318_2024_359_MOESM7_ESM.zip › Figure 2/Fig 2J/5-p-S6-merge.Tif]

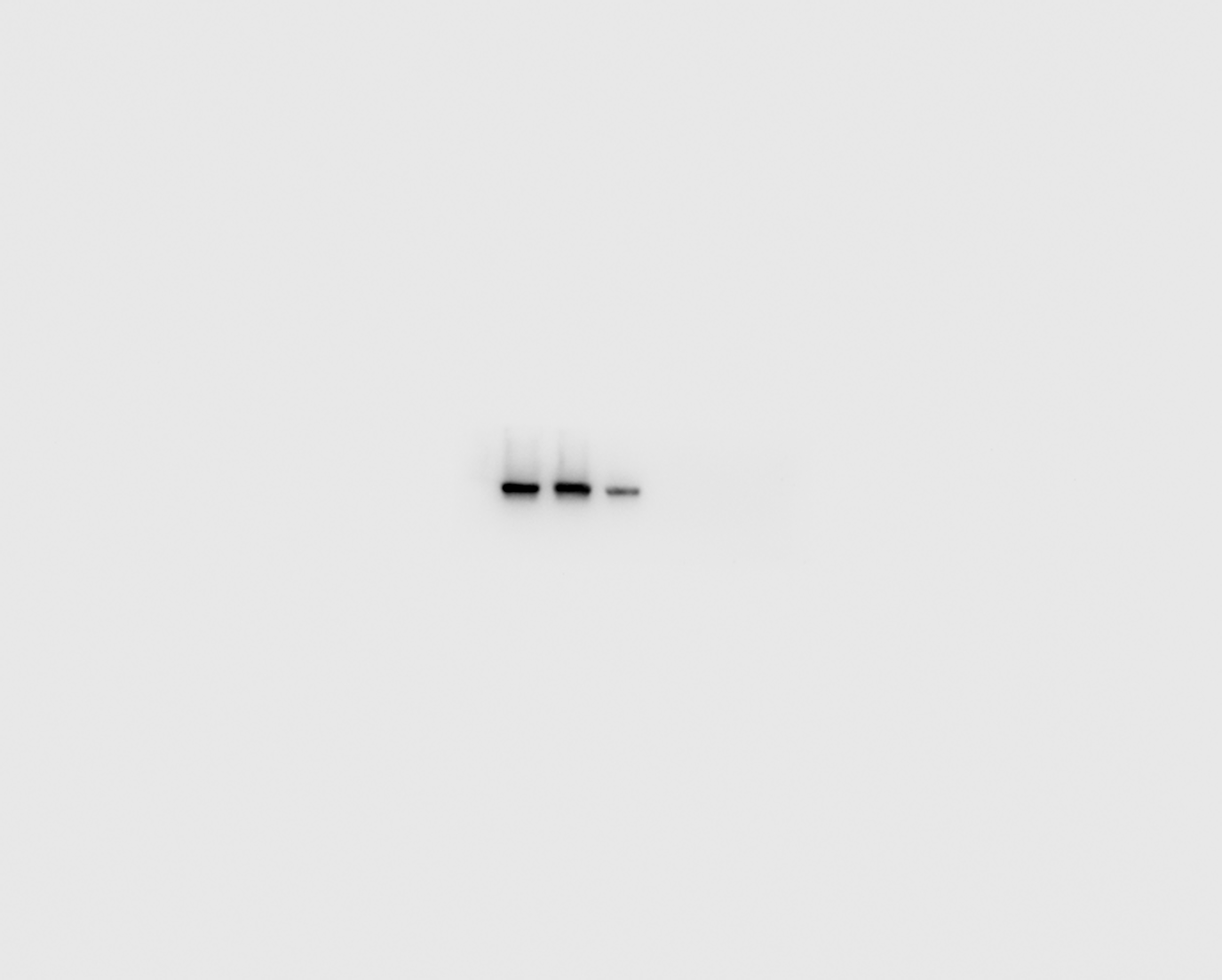

Supplement: Supplementary file 7 — Source data Fig. 2 [file 44318_2024_359_MOESM7_ESM.zip › Figure 2/Fig 2J/5-p-S6.Tif]

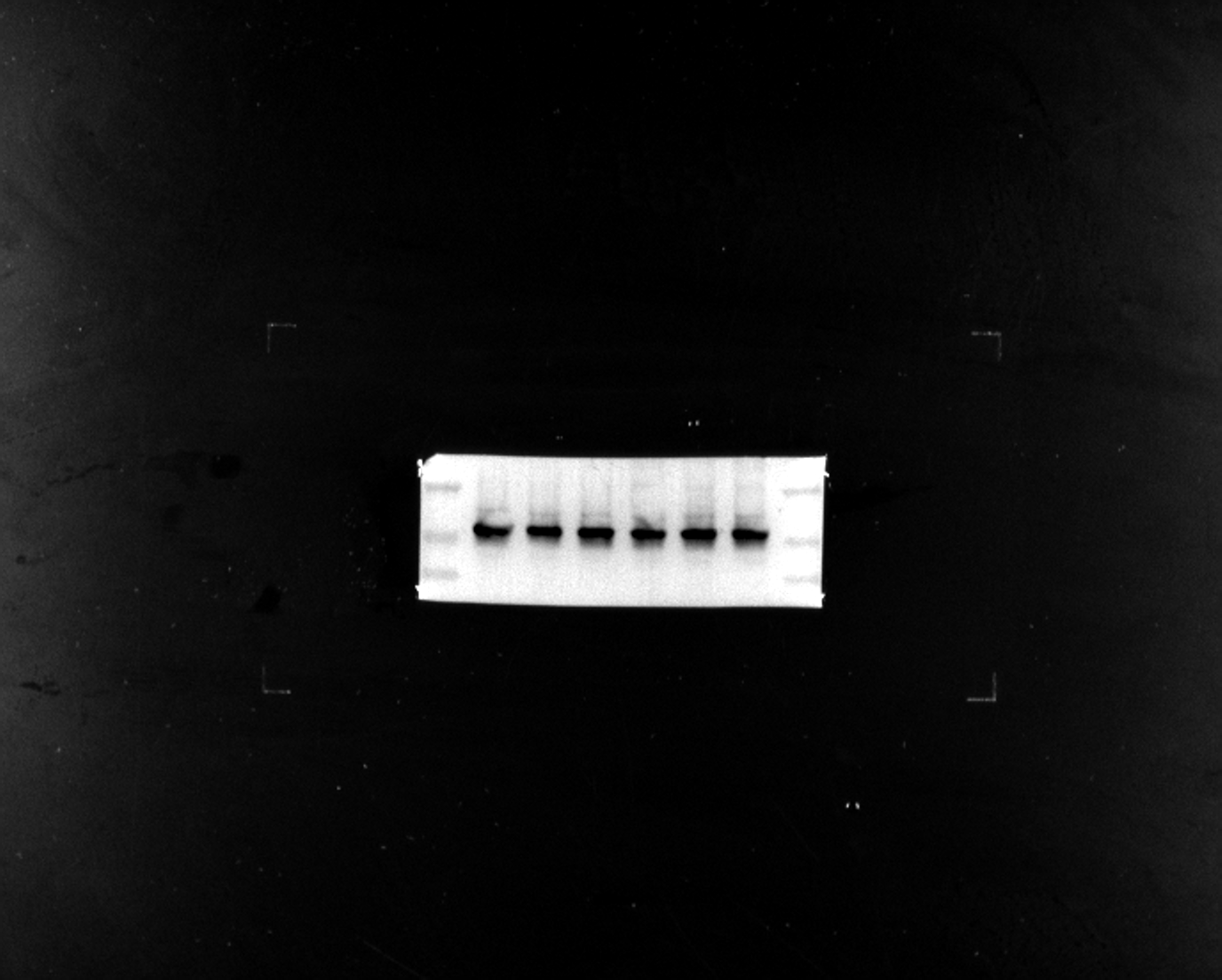

Supplement: Supplementary file 7 — Source data Fig. 2 [file 44318_2024_359_MOESM7_ESM.zip › Figure 2/Fig 2J/6-S6-merge.Tif]

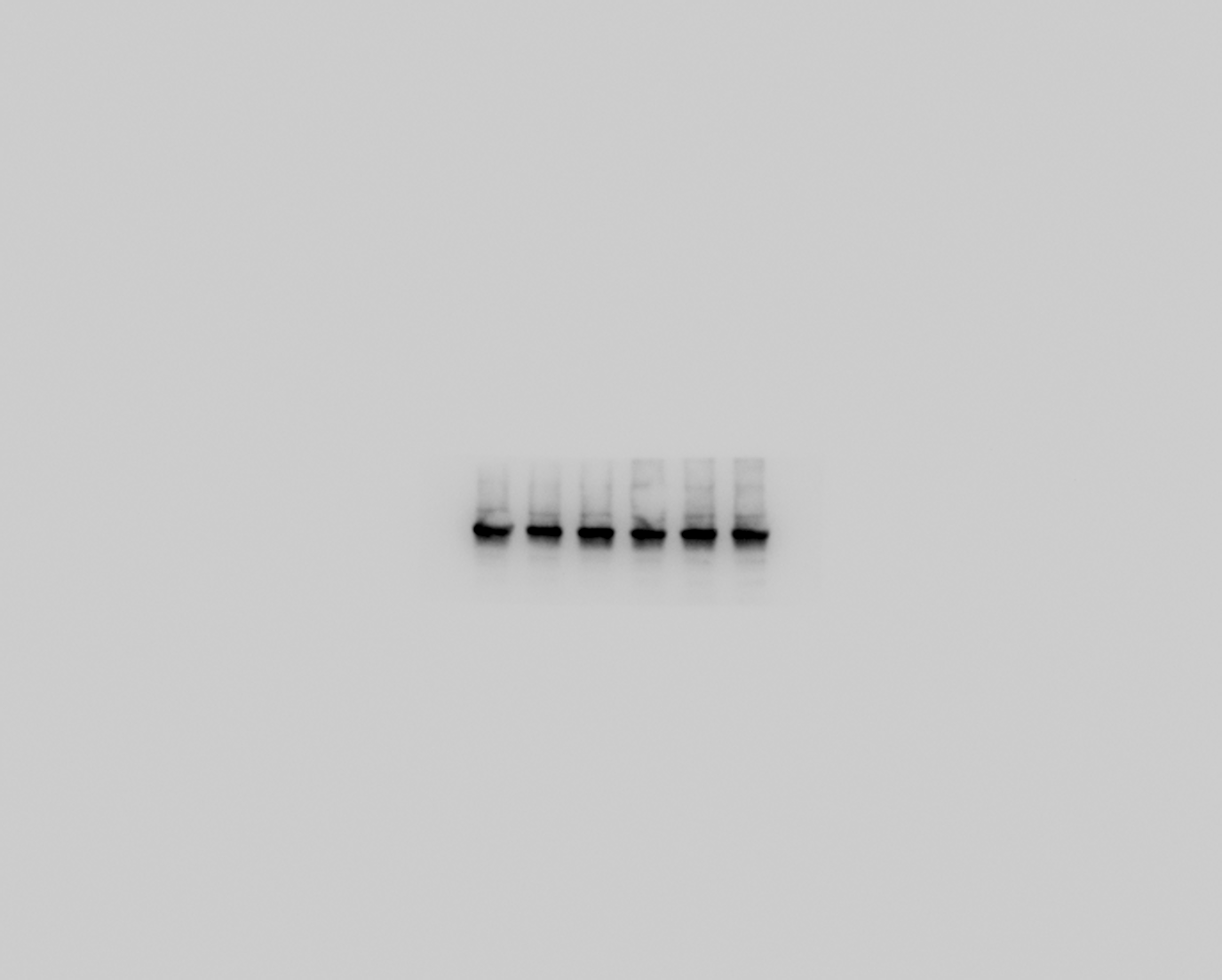

Supplement: Supplementary file 7 — Source data Fig. 2 [file 44318_2024_359_MOESM7_ESM.zip › Figure 2/Fig 2J/6-S6.Tif]

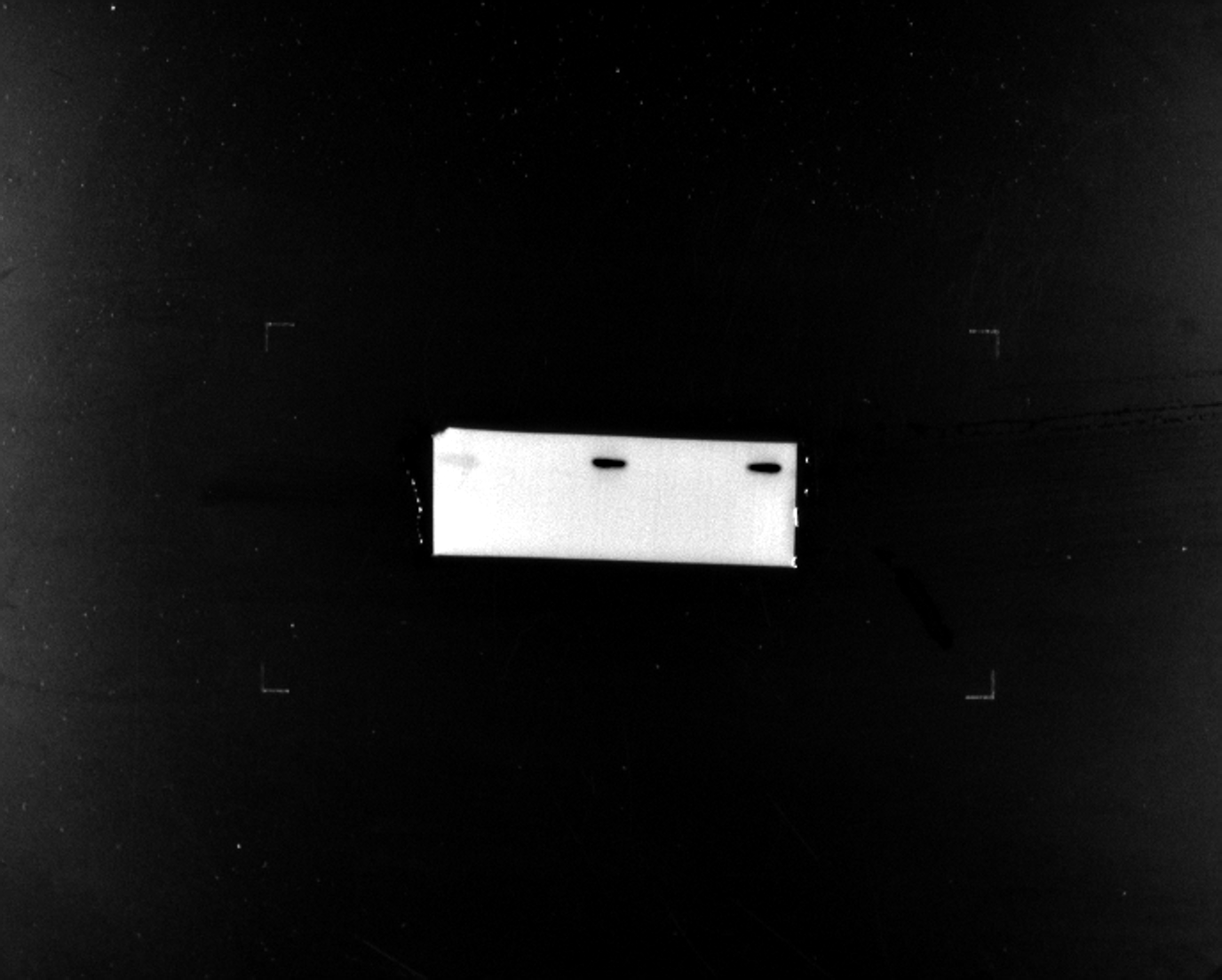

Supplement: Supplementary file 7 — Source data Fig. 2 [file 44318_2024_359_MOESM7_ESM.zip › Figure 2/Fig 2J/7-Flag-mege.Tif]

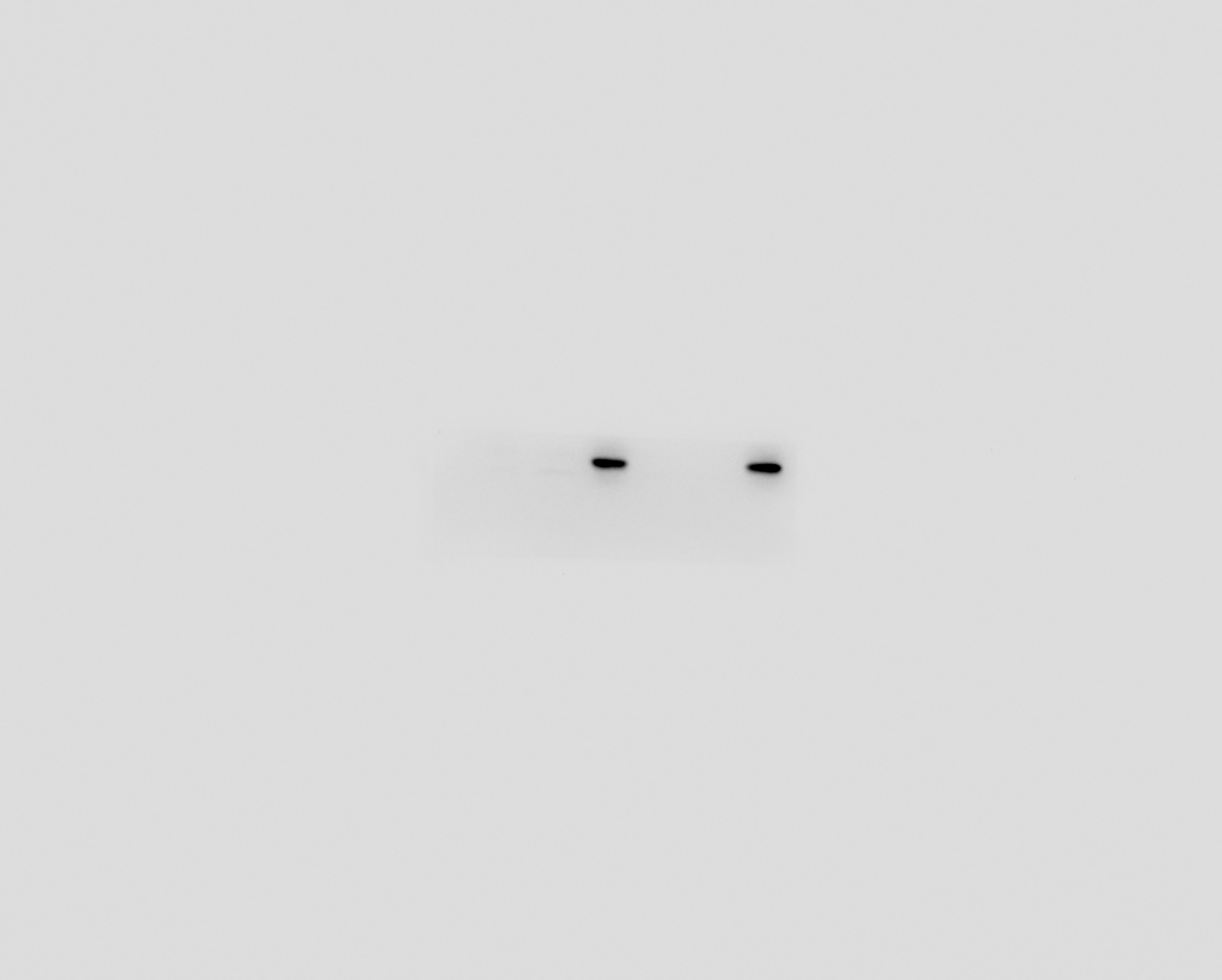

Supplement: Supplementary file 7 — Source data Fig. 2 [file 44318_2024_359_MOESM7_ESM.zip › Figure 2/Fig 2J/7-Flag.Tif]

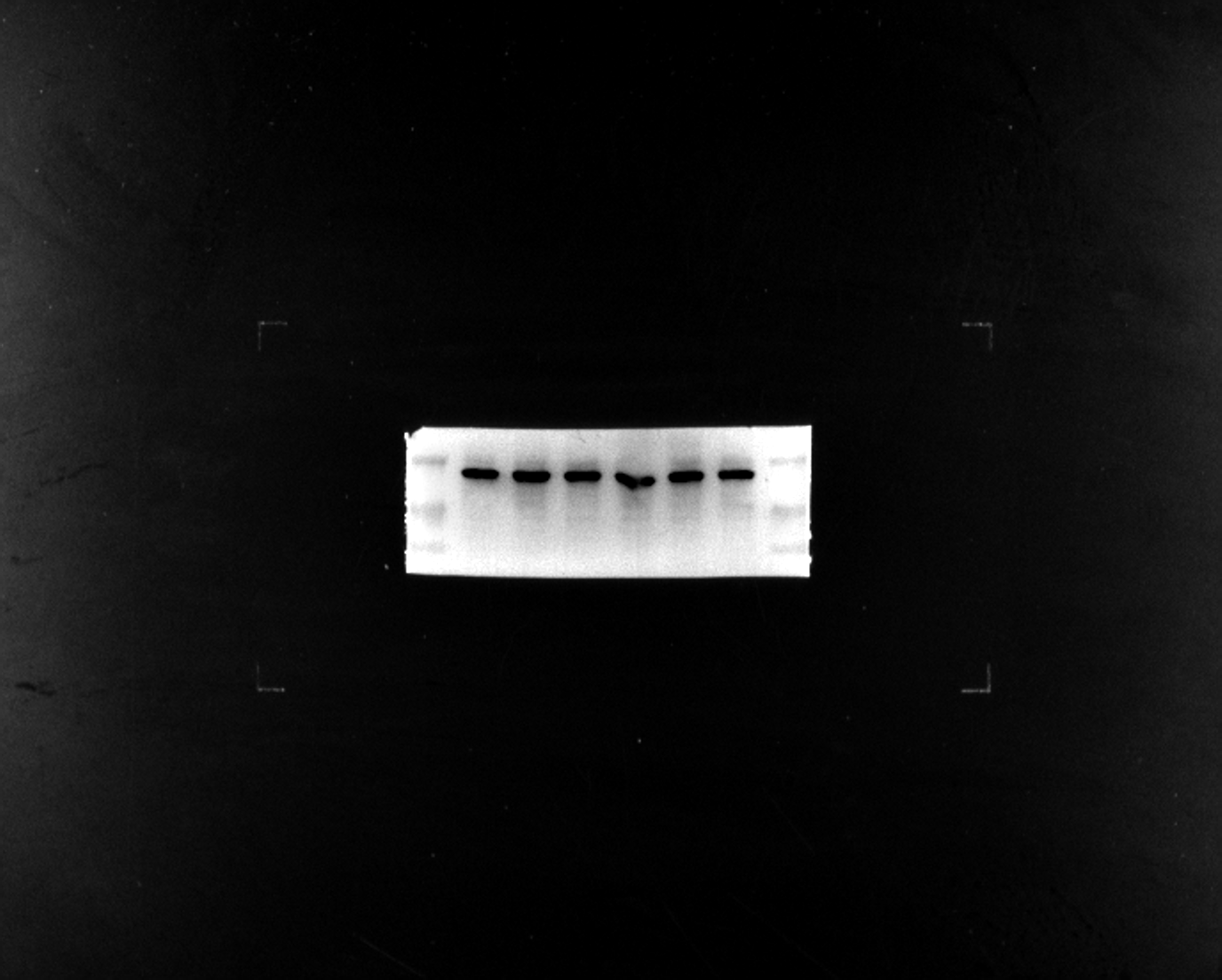

Supplement: Supplementary file 7 — Source data Fig. 2 [file 44318_2024_359_MOESM7_ESM.zip › Figure 2/Fig 2J/8-GAPDH-merge.Tif]

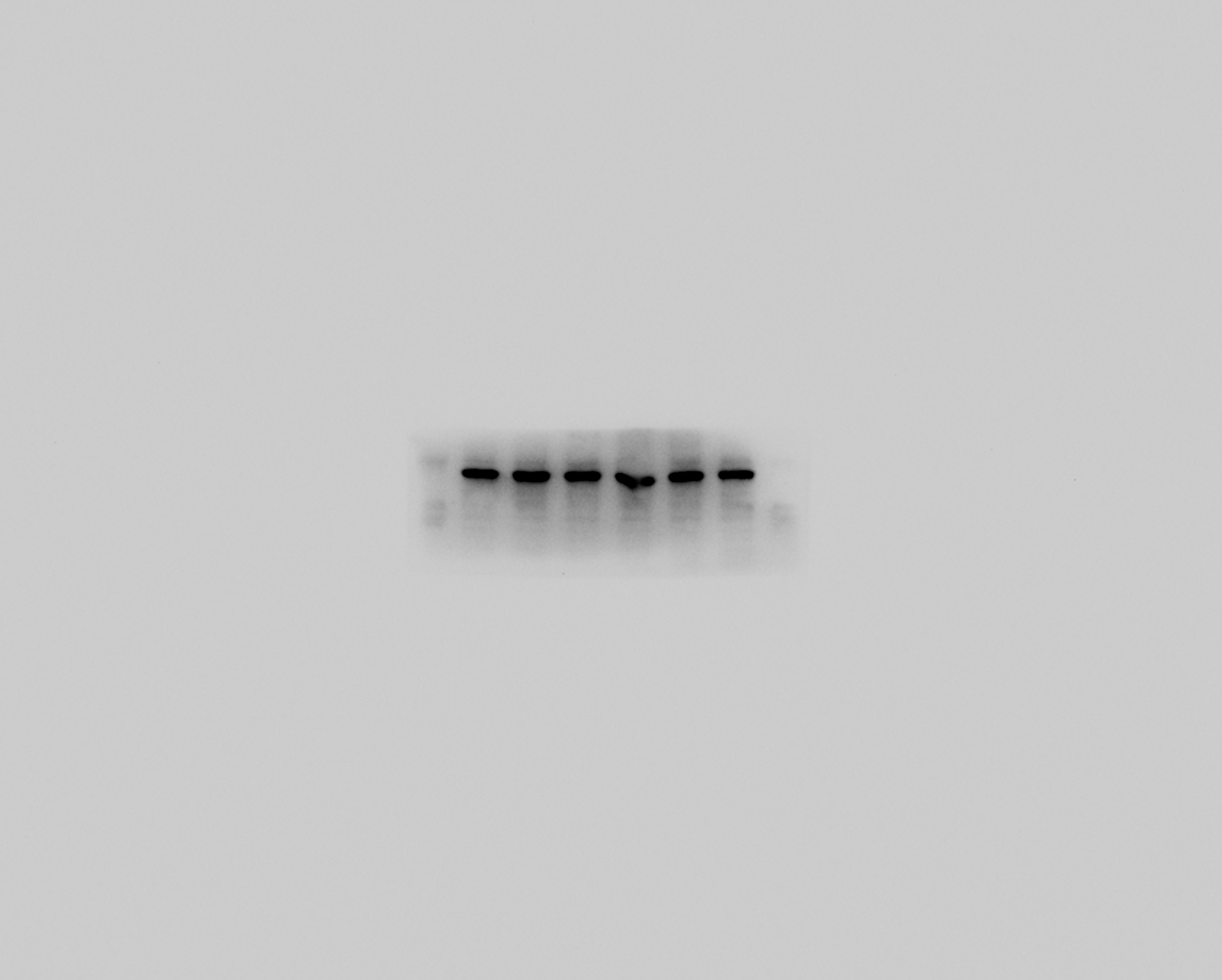

Supplement: Supplementary file 7 — Source data Fig. 2 [file 44318_2024_359_MOESM7_ESM.zip › Figure 2/Fig 2J/8-GAPDH.Tif]

Fig 2J

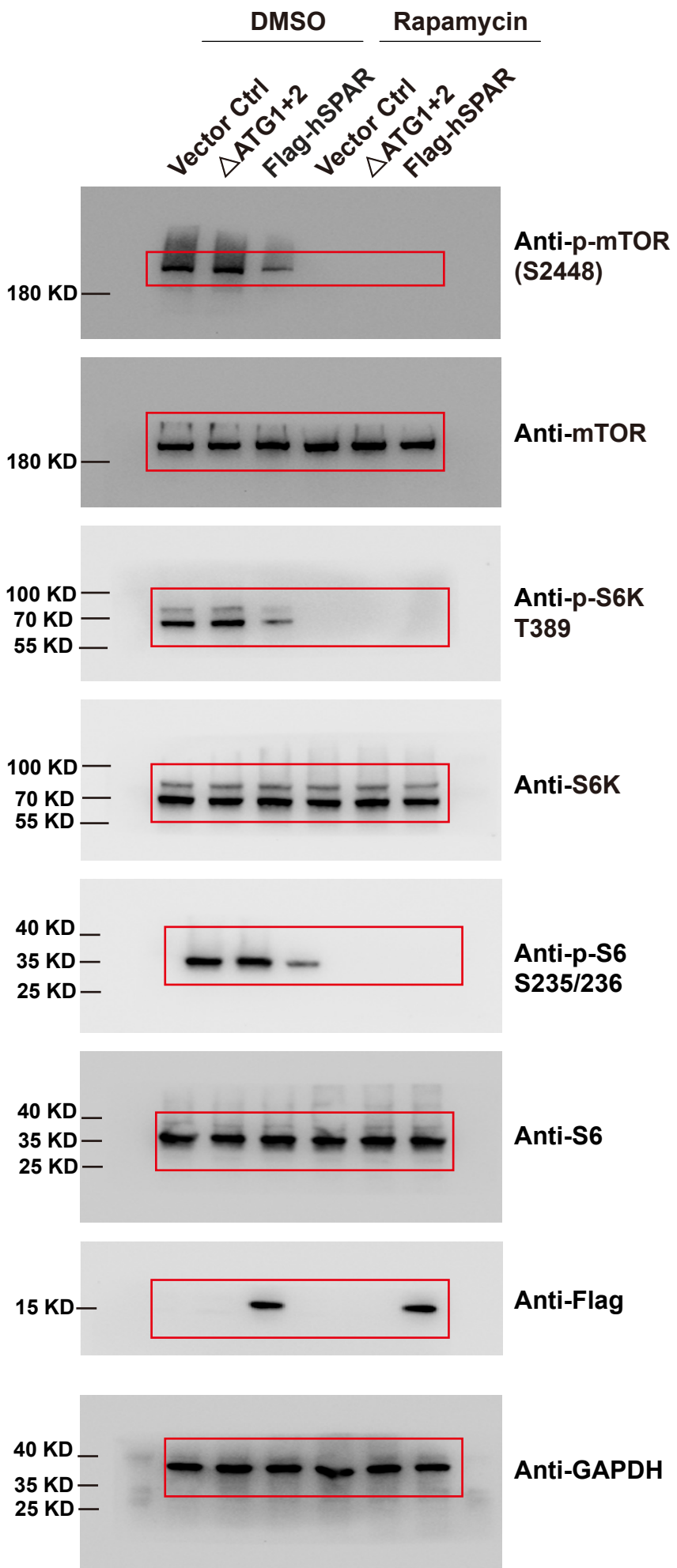

Supplement: Supplementary file 7 — Source data Fig. 2 [file 44318_2024_359_MOESM7_ESM.zip › Figure 2/Fig 2J/Fig 2J.pdf]

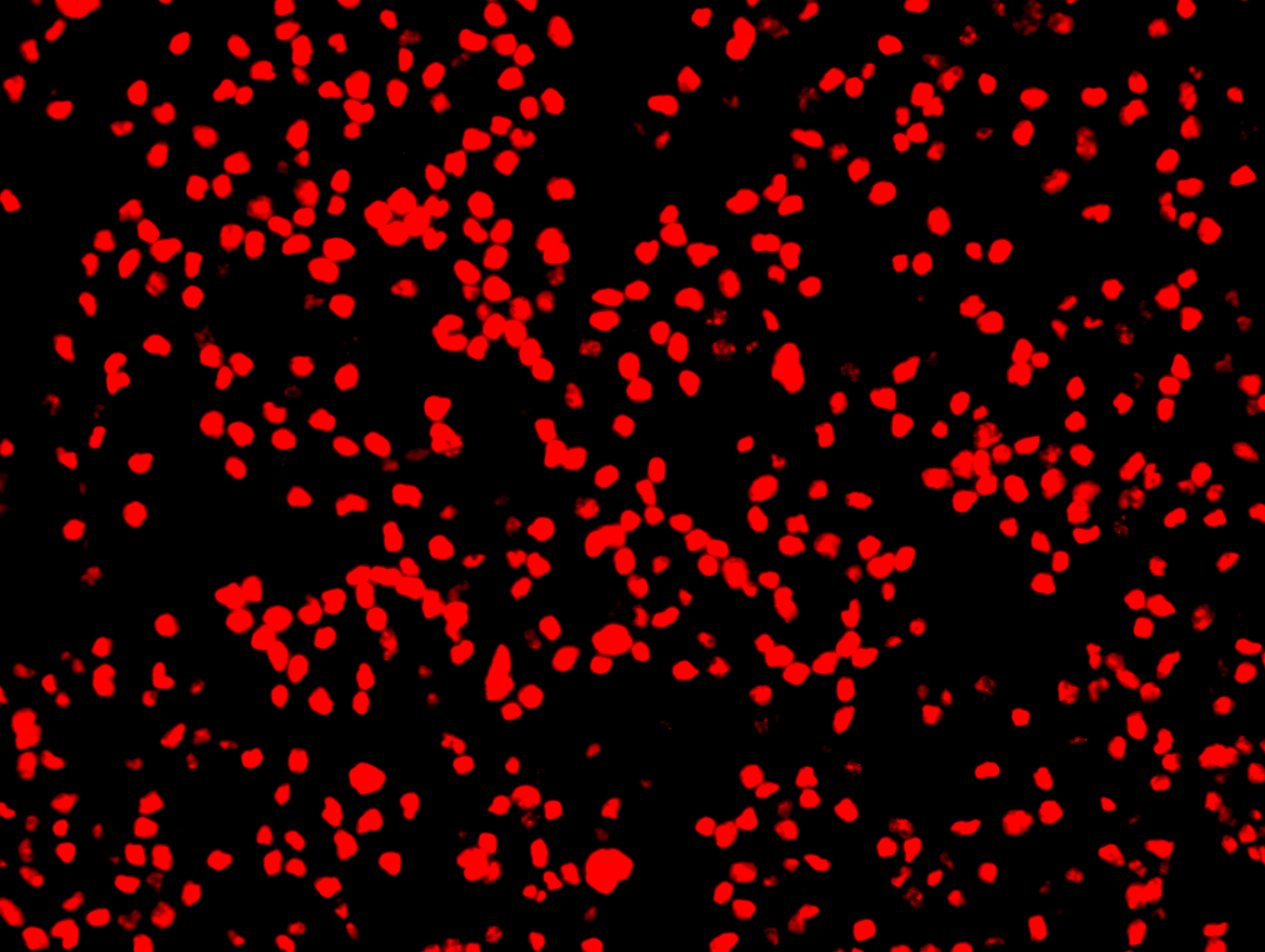

Supplement: Supplementary file 7 — Source data Fig. 2 [file 44318_2024_359_MOESM7_ESM.zip › Figure 2/Fig 2K and 2L/Fig 2K/ATG 1+2/1-edu in manu .tif]

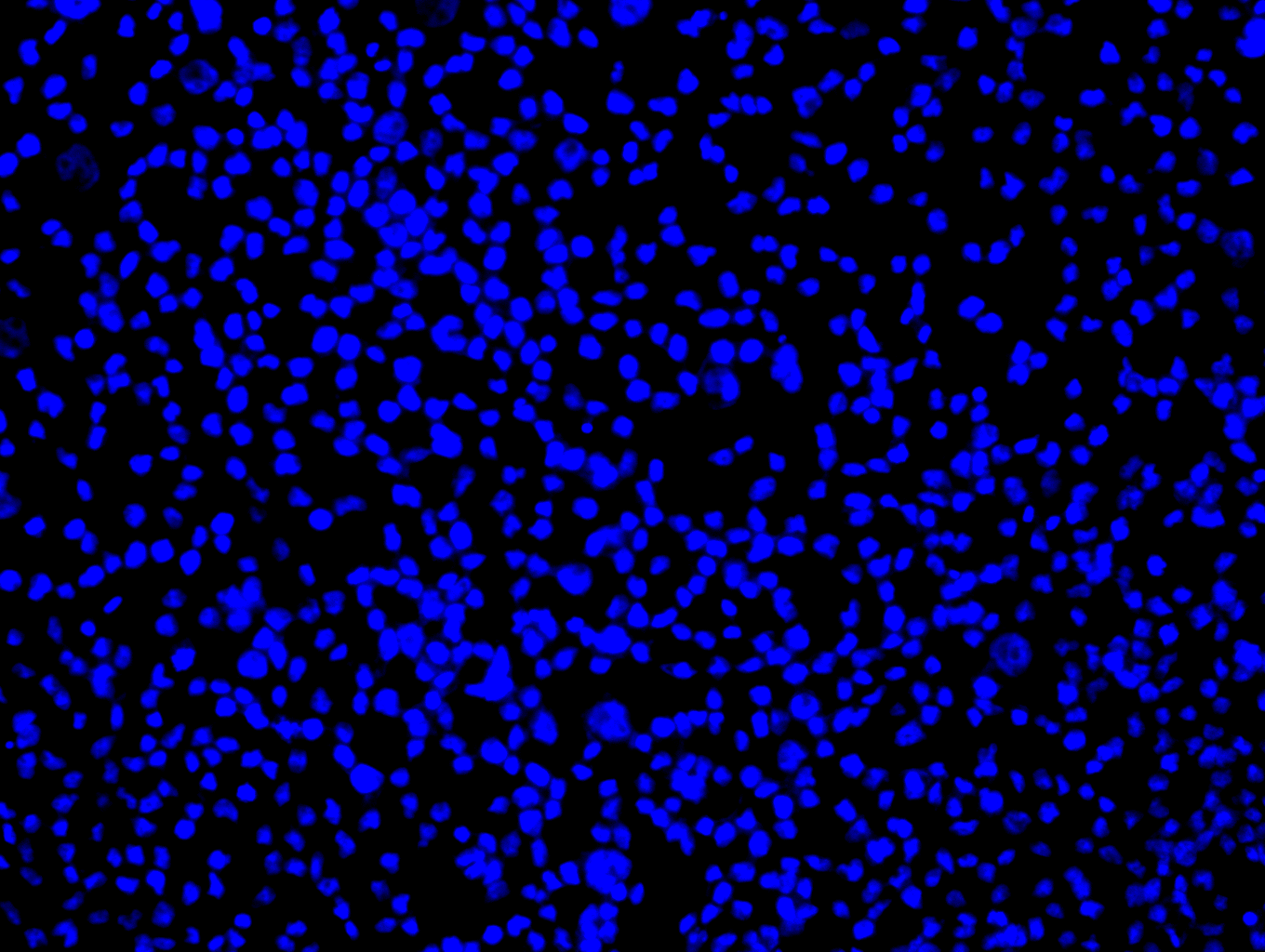

Supplement: Supplementary file 7 — Source data Fig. 2 [file 44318_2024_359_MOESM7_ESM.zip › Figure 2/Fig 2K and 2L/Fig 2K/ATG 1+2/1-hoechst in manu.tif]

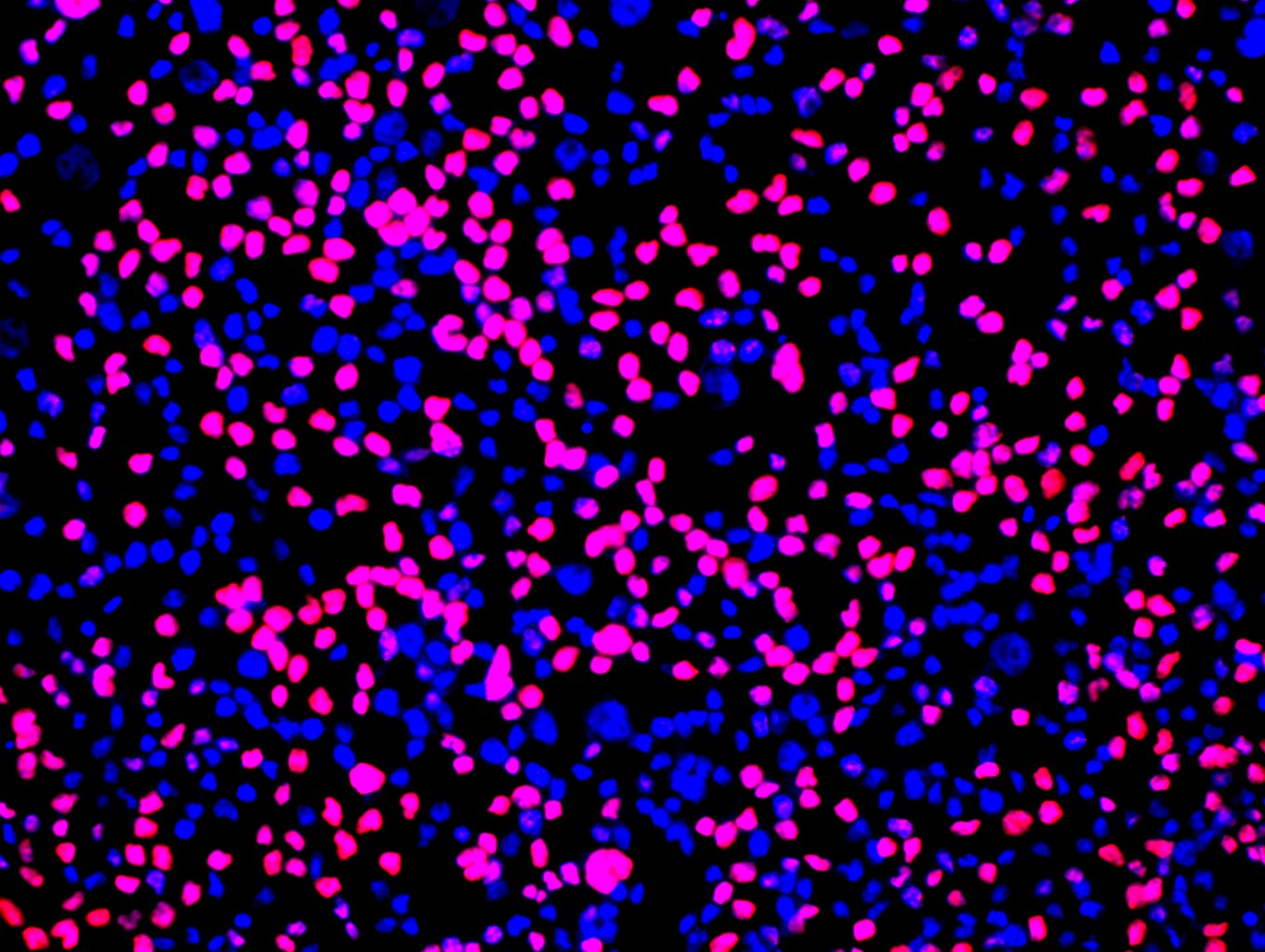

Supplement: Supplementary file 7 — Source data Fig. 2 [file 44318_2024_359_MOESM7_ESM.zip › Figure 2/Fig 2K and 2L/Fig 2K/ATG 1+2/1-merge in manu.jpg]

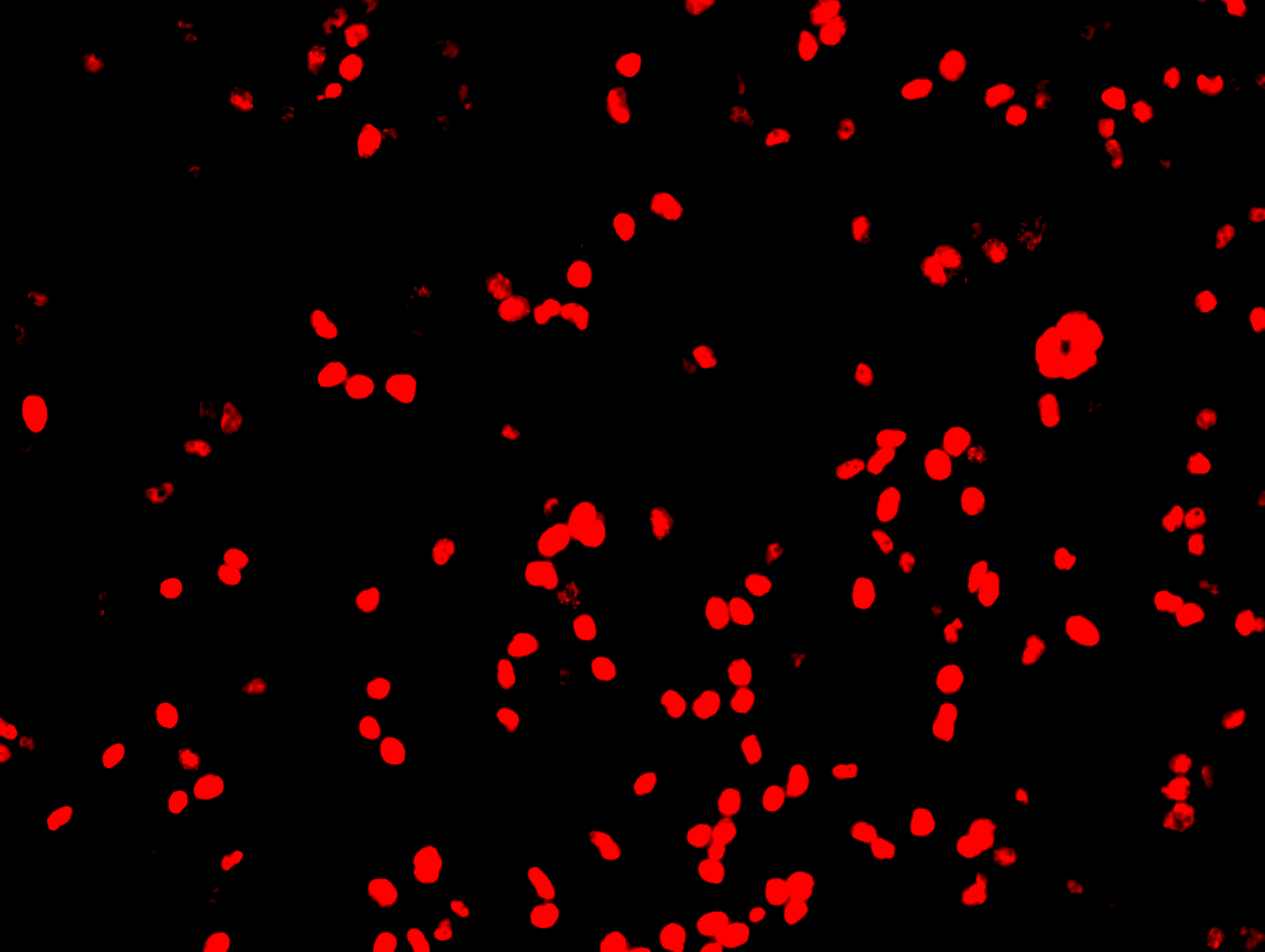

Supplement: Supplementary file 7 — Source data Fig. 2 [file 44318_2024_359_MOESM7_ESM.zip › Figure 2/Fig 2K and 2L/Fig 2K/ATG 1+2 +rapamycin/1-edu in manu.tif]

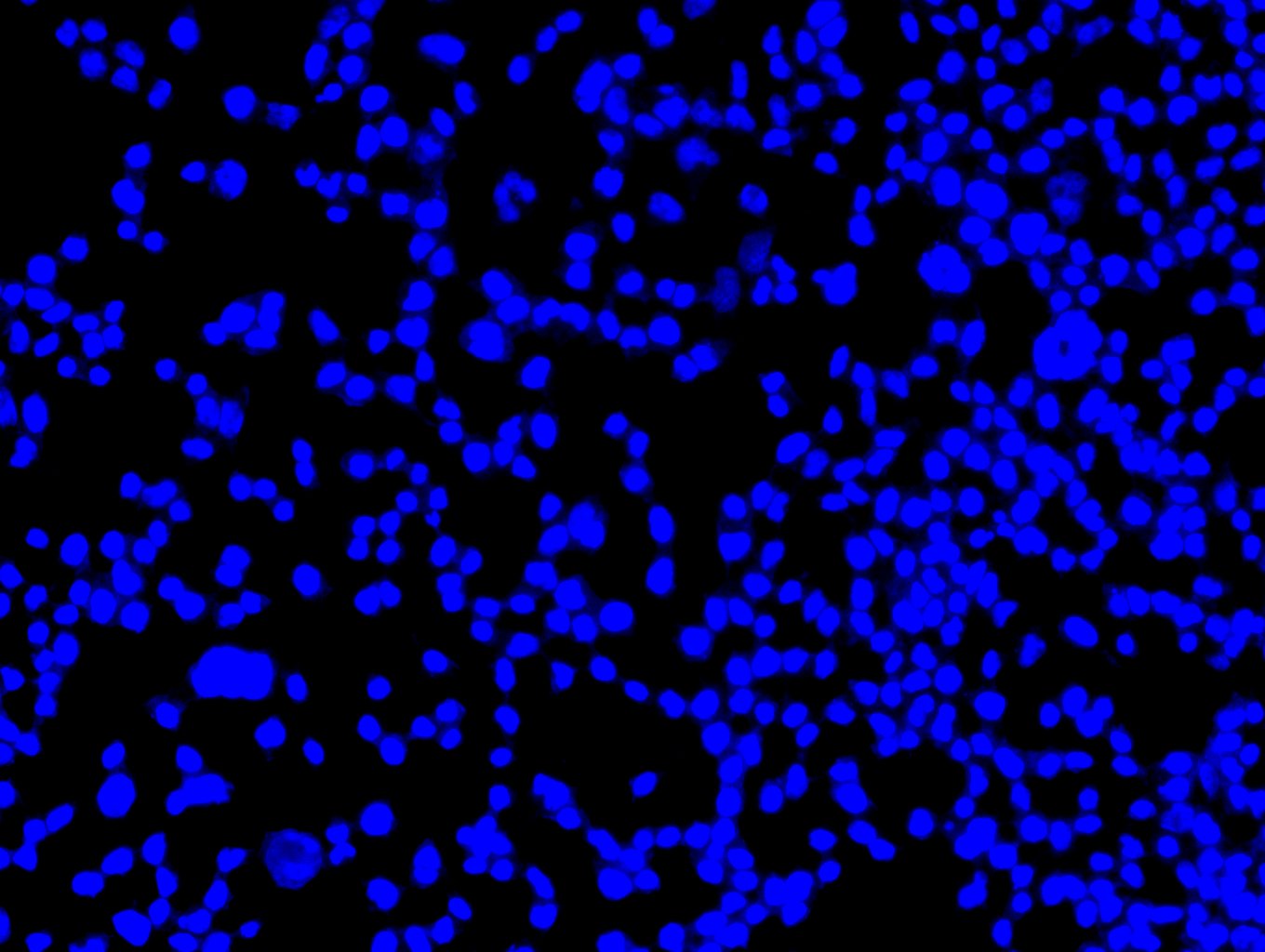

Supplement: Supplementary file 7 — Source data Fig. 2 [file 44318_2024_359_MOESM7_ESM.zip › Figure 2/Fig 2K and 2L/Fig 2K/ATG 1+2 +rapamycin/1-hoechst in manu.jpg]

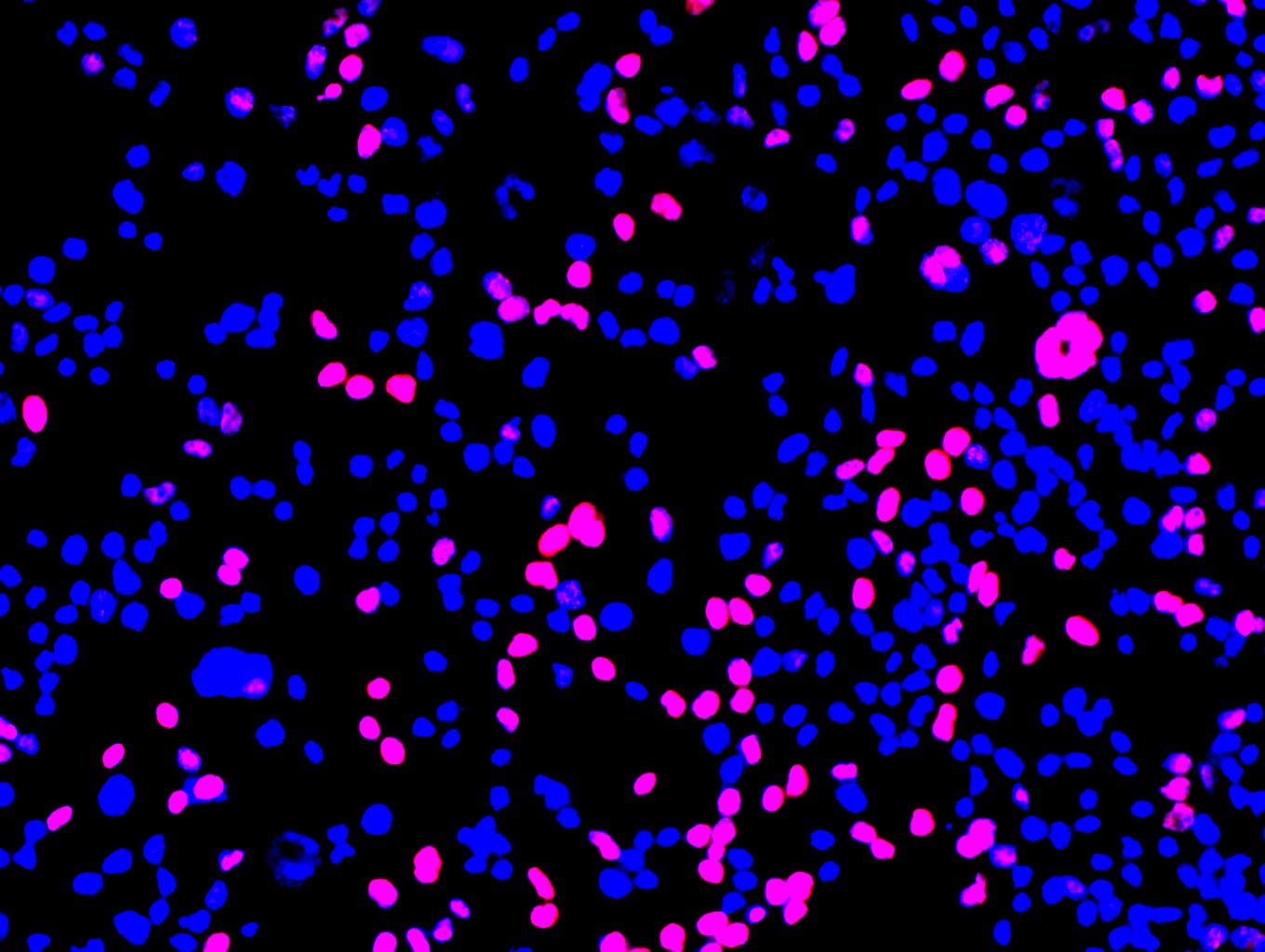

Supplement: Supplementary file 7 — Source data Fig. 2 [file 44318_2024_359_MOESM7_ESM.zip › Figure 2/Fig 2K and 2L/Fig 2K/ATG 1+2 +rapamycin/1-merge in manu.jpg]

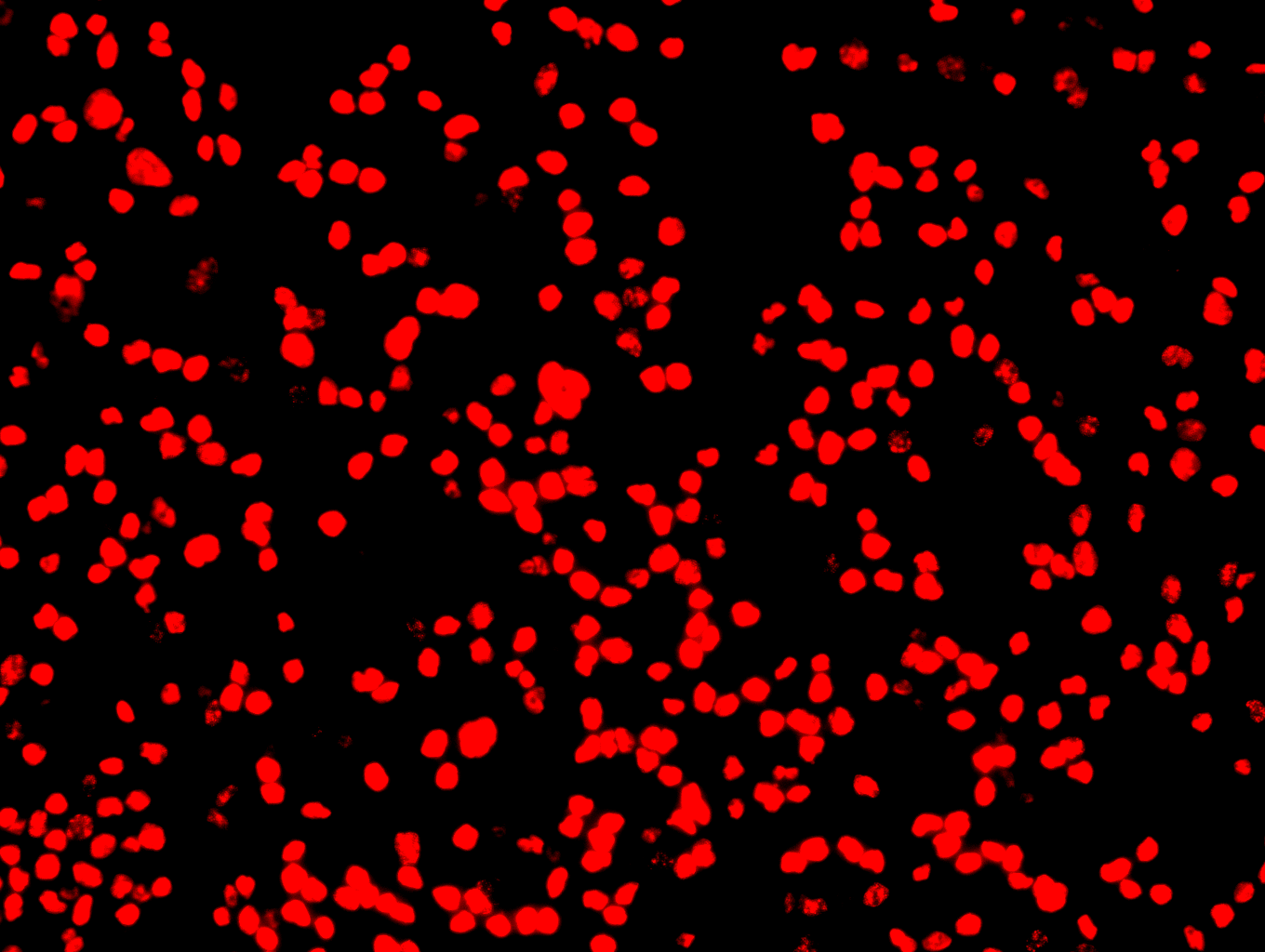

Supplement: Supplementary file 7 — Source data Fig. 2 [file 44318_2024_359_MOESM7_ESM.zip › Figure 2/Fig 2K and 2L/Fig 2K/Vector Ctrl/1-edu in manu .tif]

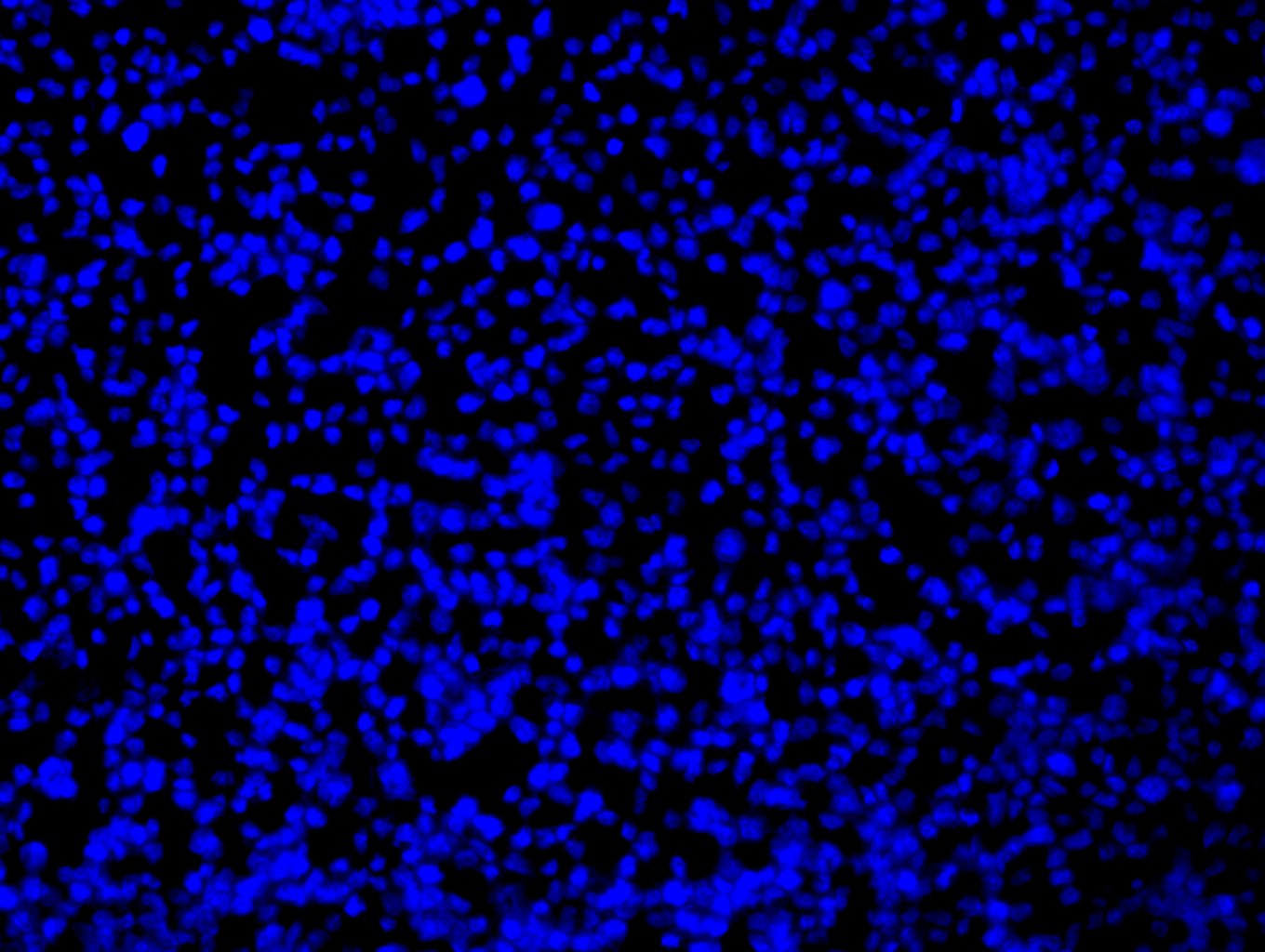

Supplement: Supplementary file 7 — Source data Fig. 2 [file 44318_2024_359_MOESM7_ESM.zip › Figure 2/Fig 2K and 2L/Fig 2K/Vector Ctrl/1-hoechst in manu .jpg]

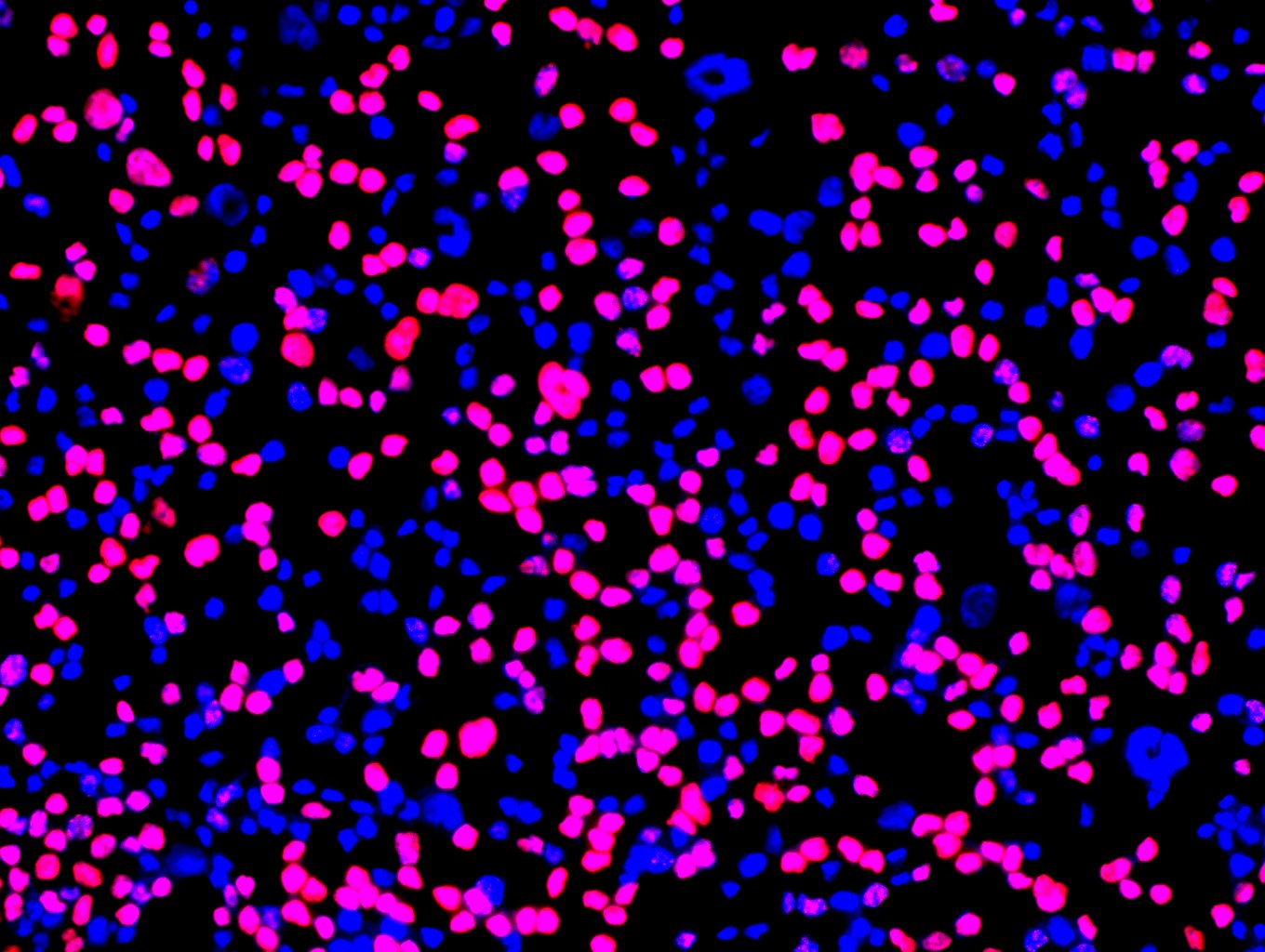

Supplement: Supplementary file 7 — Source data Fig. 2 [file 44318_2024_359_MOESM7_ESM.zip › Figure 2/Fig 2K and 2L/Fig 2K/Vector Ctrl/1-merge in manu .jpg]

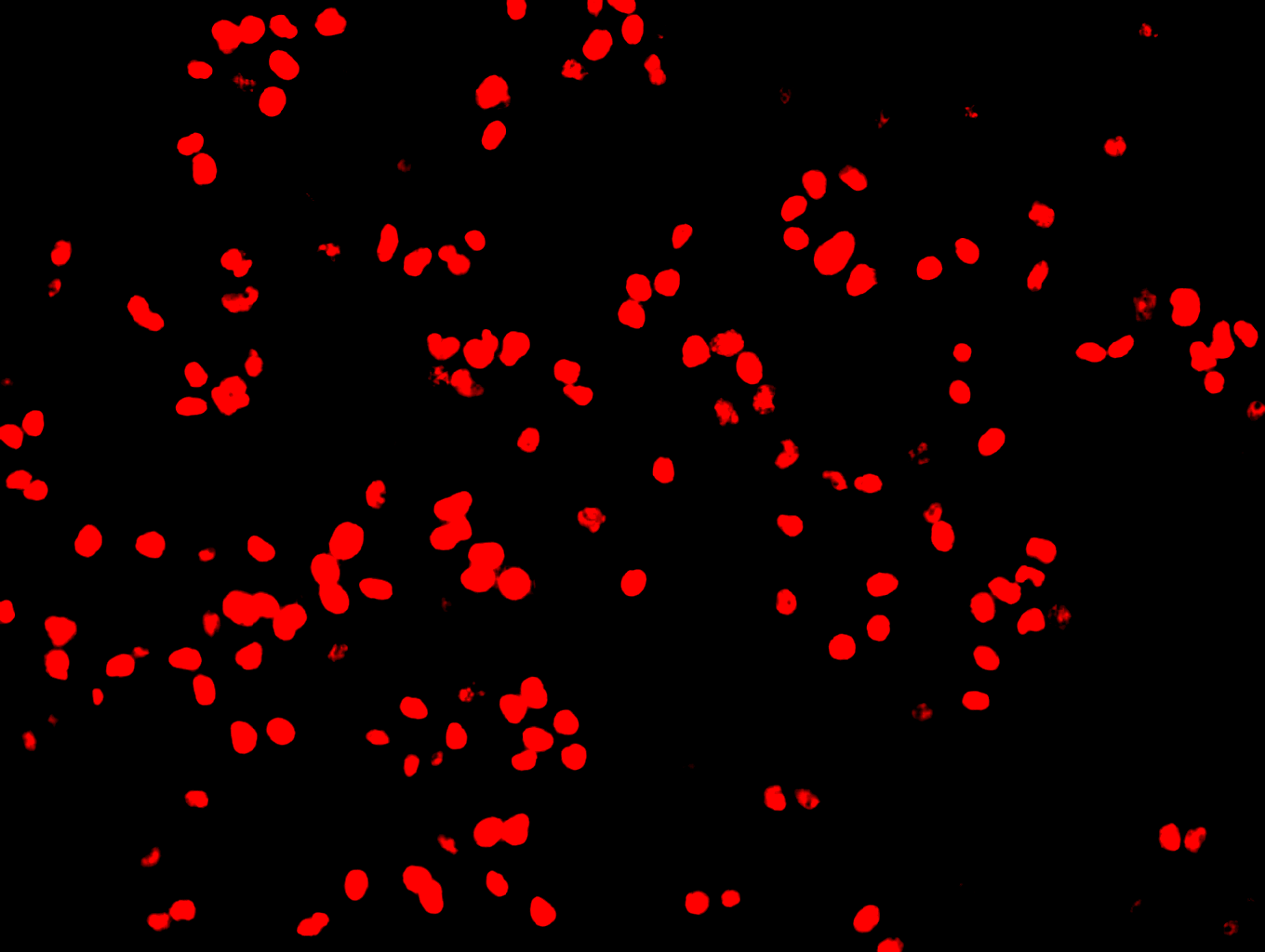

Supplement: Supplementary file 7 — Source data Fig. 2 [file 44318_2024_359_MOESM7_ESM.zip › Figure 2/Fig 2K and 2L/Fig 2K/Vector Ctrl +rapamycin/1-edu in manu.tif]

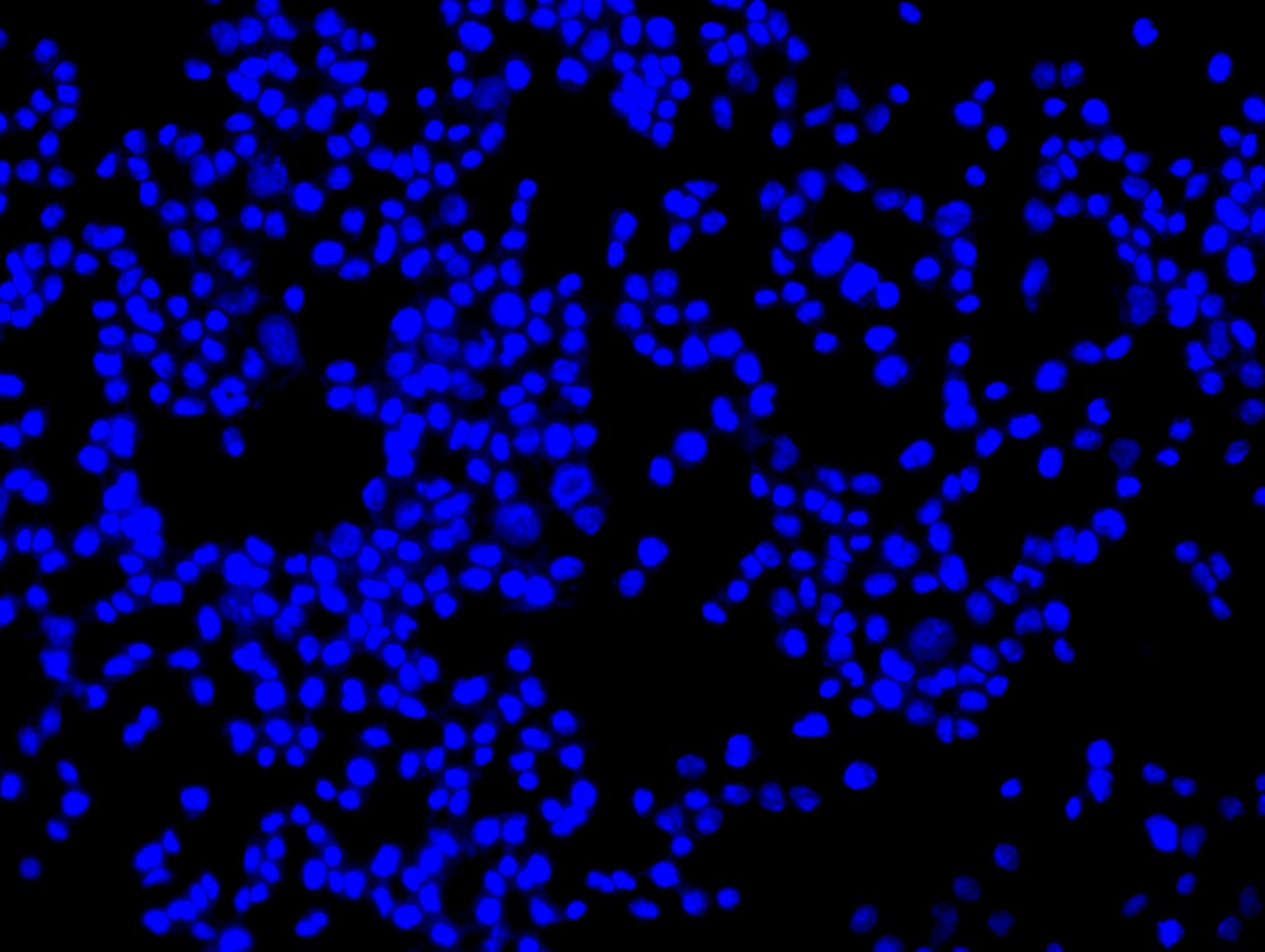

Supplement: Supplementary file 7 — Source data Fig. 2 [file 44318_2024_359_MOESM7_ESM.zip › Figure 2/Fig 2K and 2L/Fig 2K/Vector Ctrl +rapamycin/1-hoechst in manu.jpg]

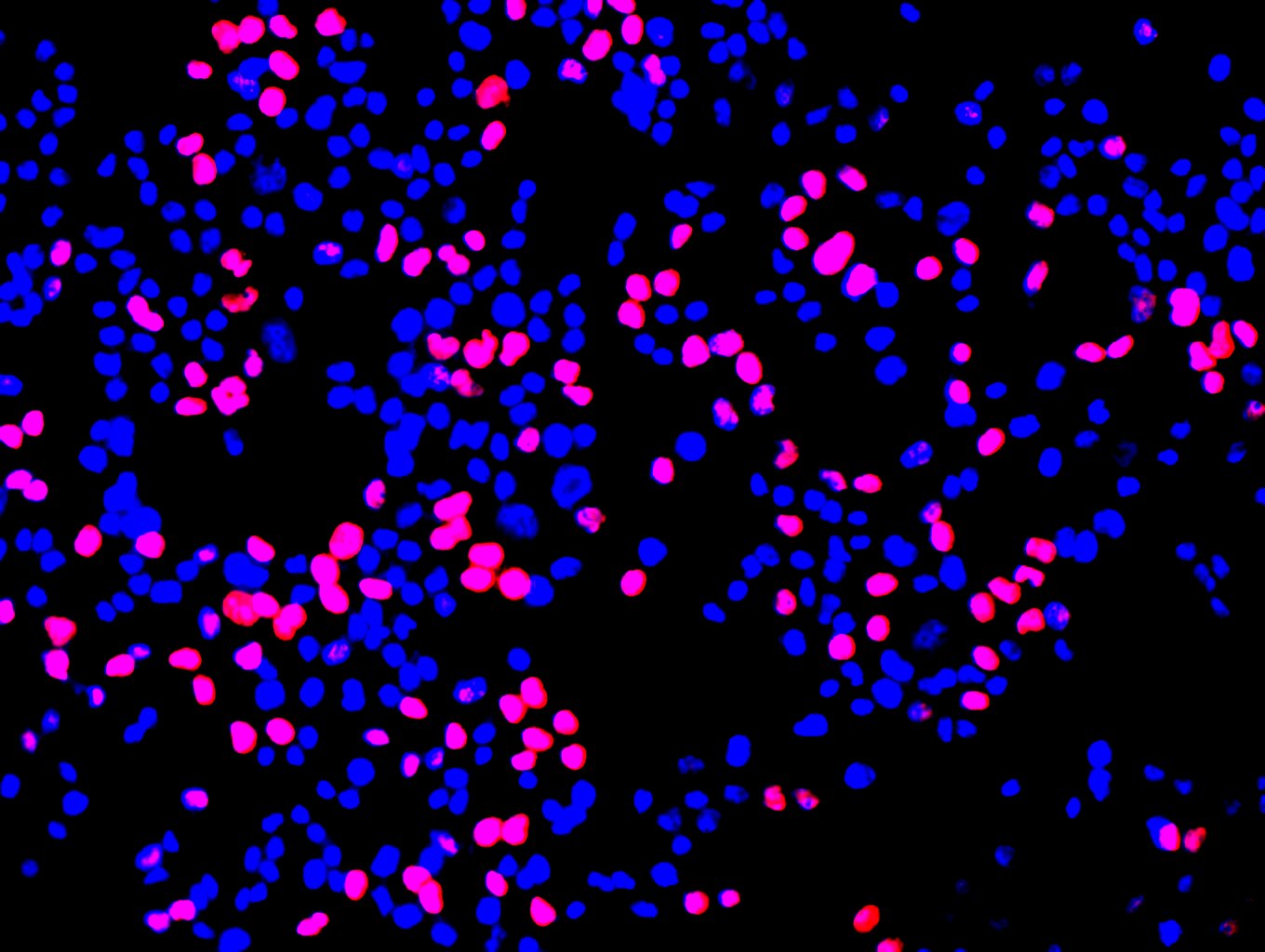

Supplement: Supplementary file 7 — Source data Fig. 2 [file 44318_2024_359_MOESM7_ESM.zip › Figure 2/Fig 2K and 2L/Fig 2K/Vector Ctrl +rapamycin/1-merge in manu.jpg]

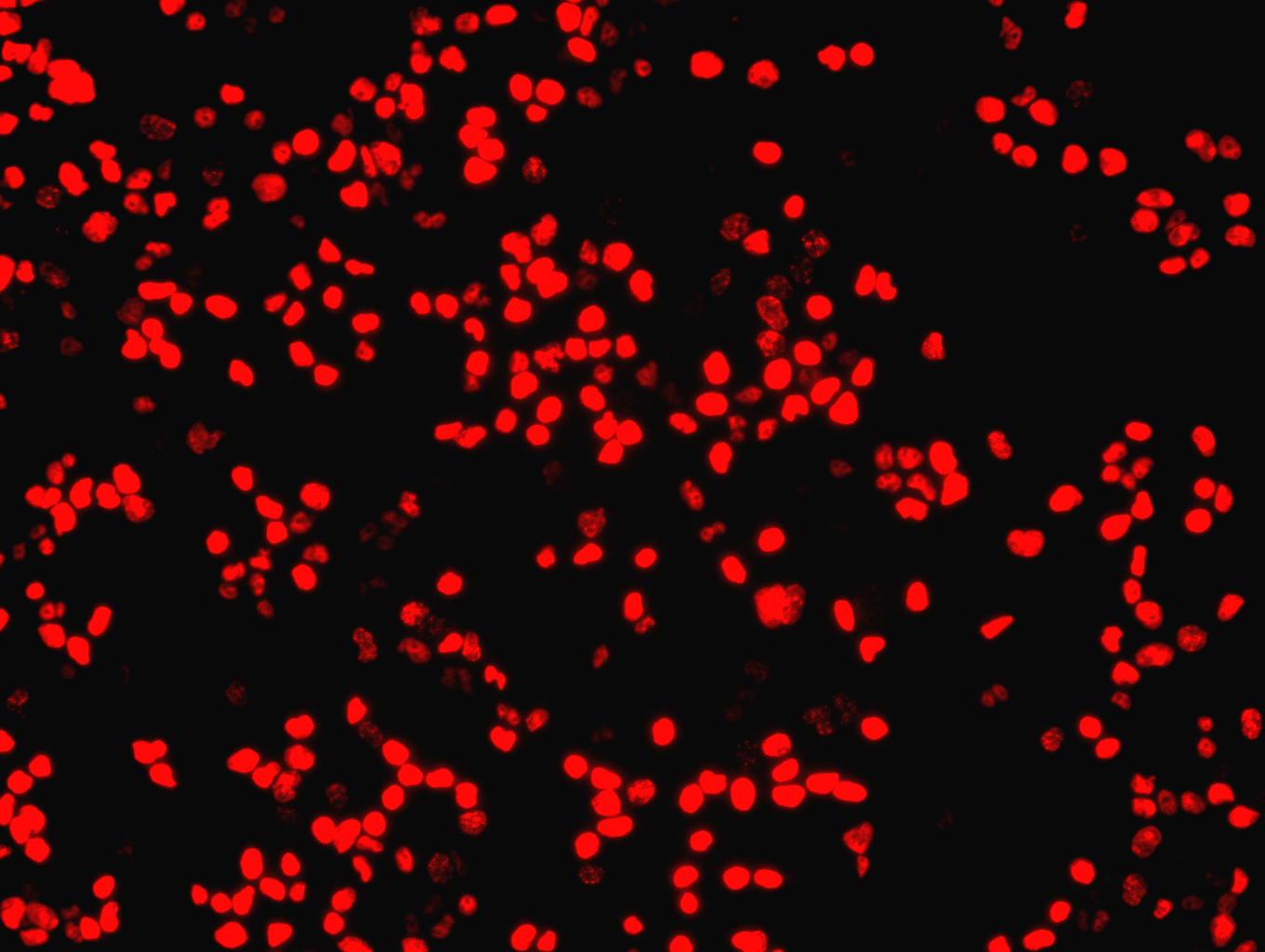

Supplement: Supplementary file 7 — Source data Fig. 2 [file 44318_2024_359_MOESM7_ESM.zip › Figure 2/Fig 2K and 2L/Fig 2K/hSPAR/1-edu in manu.tif]

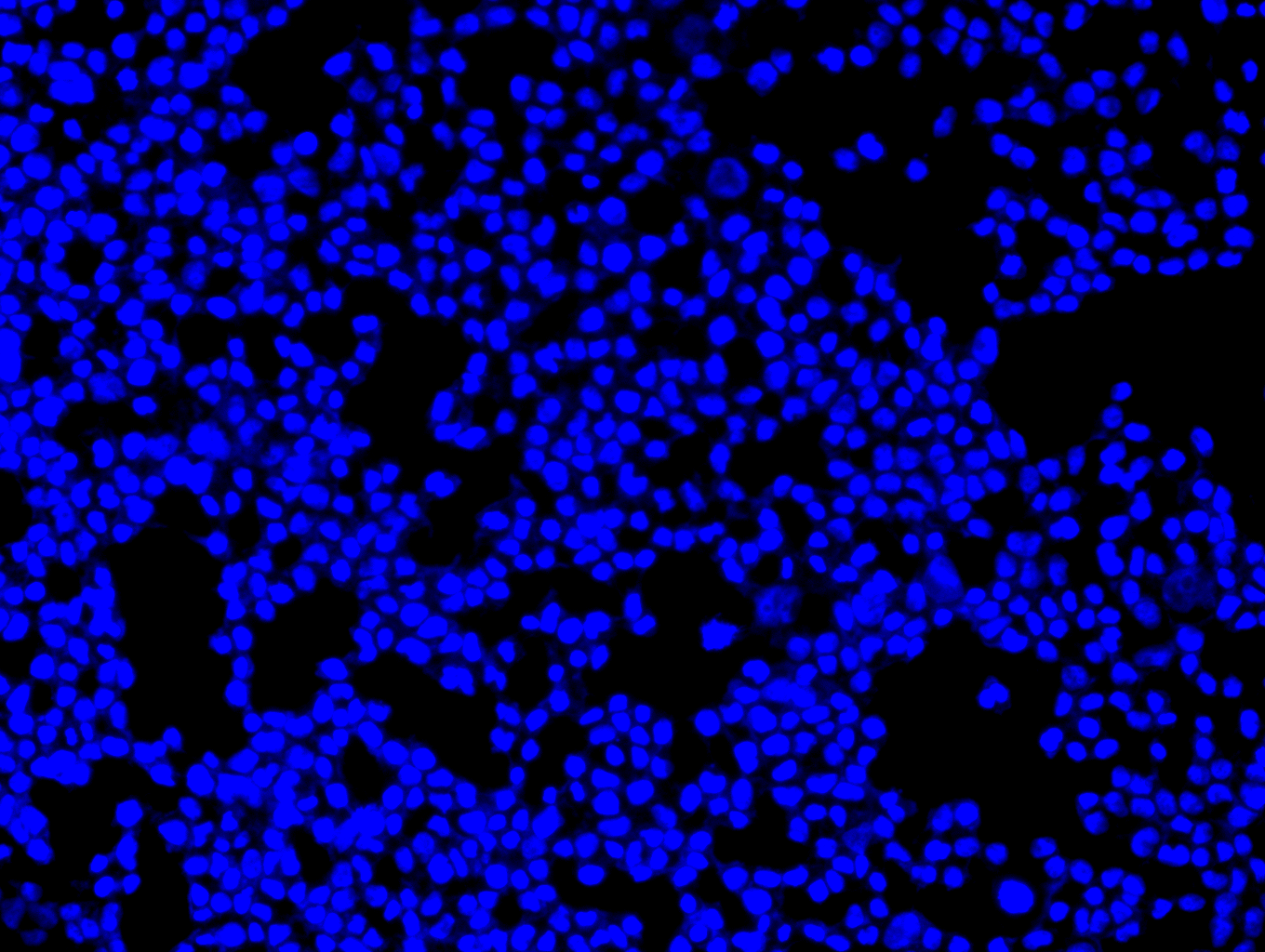

Supplement: Supplementary file 7 — Source data Fig. 2 [file 44318_2024_359_MOESM7_ESM.zip › Figure 2/Fig 2K and 2L/Fig 2K/hSPAR/1-hoechst in manu.tif]

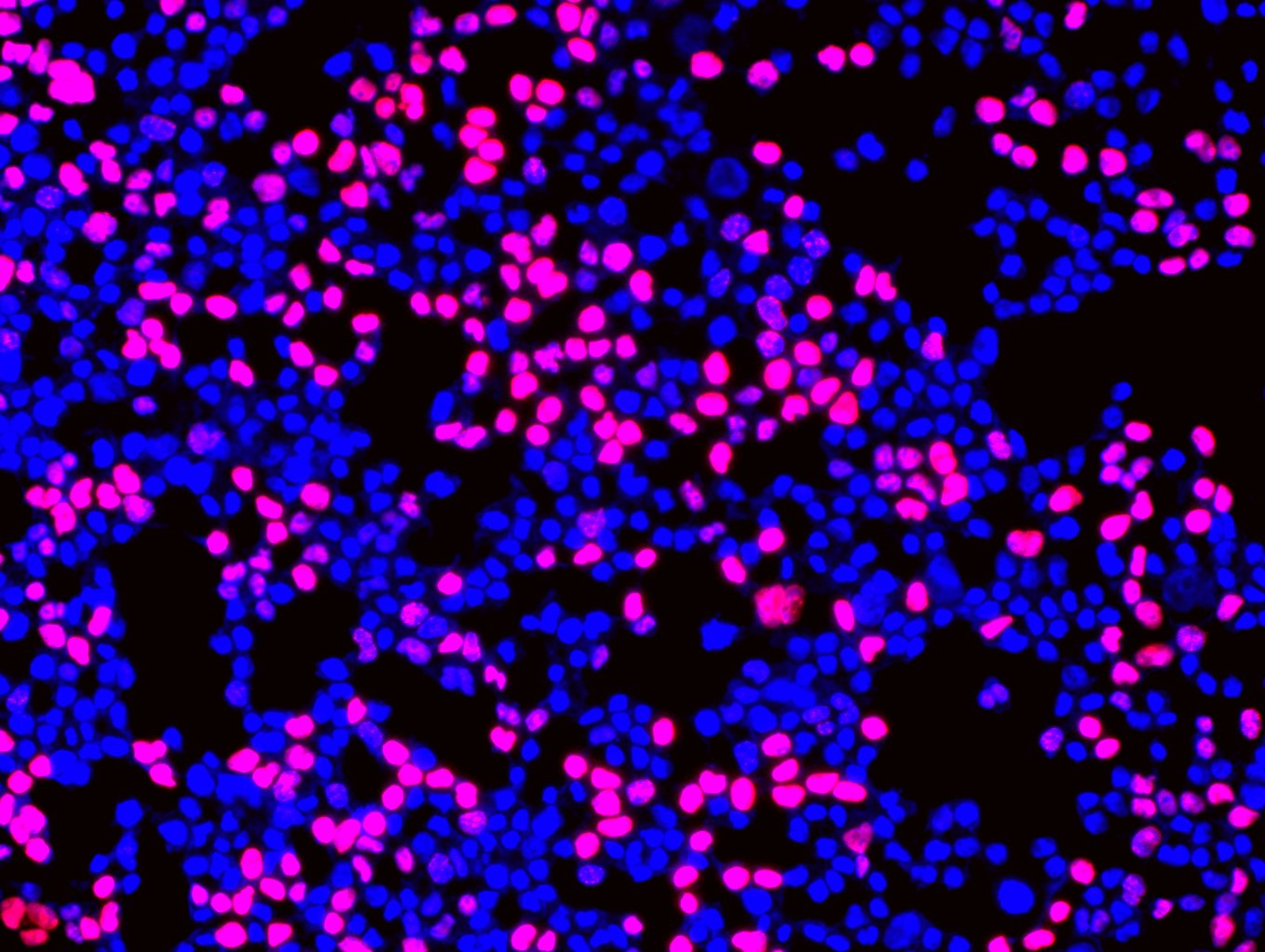

Supplement: Supplementary file 7 — Source data Fig. 2 [file 44318_2024_359_MOESM7_ESM.zip › Figure 2/Fig 2K and 2L/Fig 2K/hSPAR/1-merge in manu.jpg]

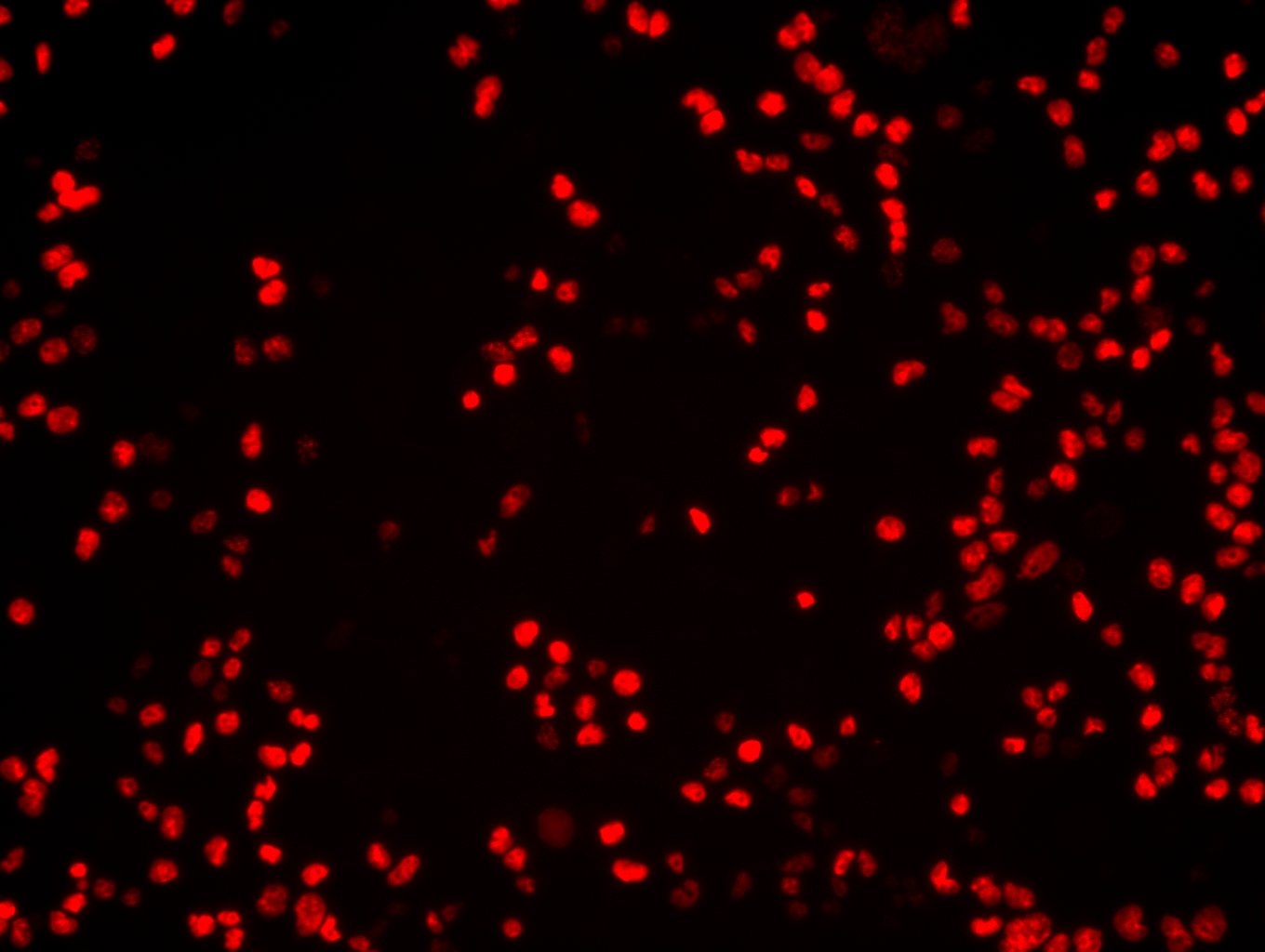

Supplement: Supplementary file 7 — Source data Fig. 2 [file 44318_2024_359_MOESM7_ESM.zip › Figure 2/Fig 2K and 2L/Fig 2K/hSPAR+rapamycin/1-edu in manu.jpg]

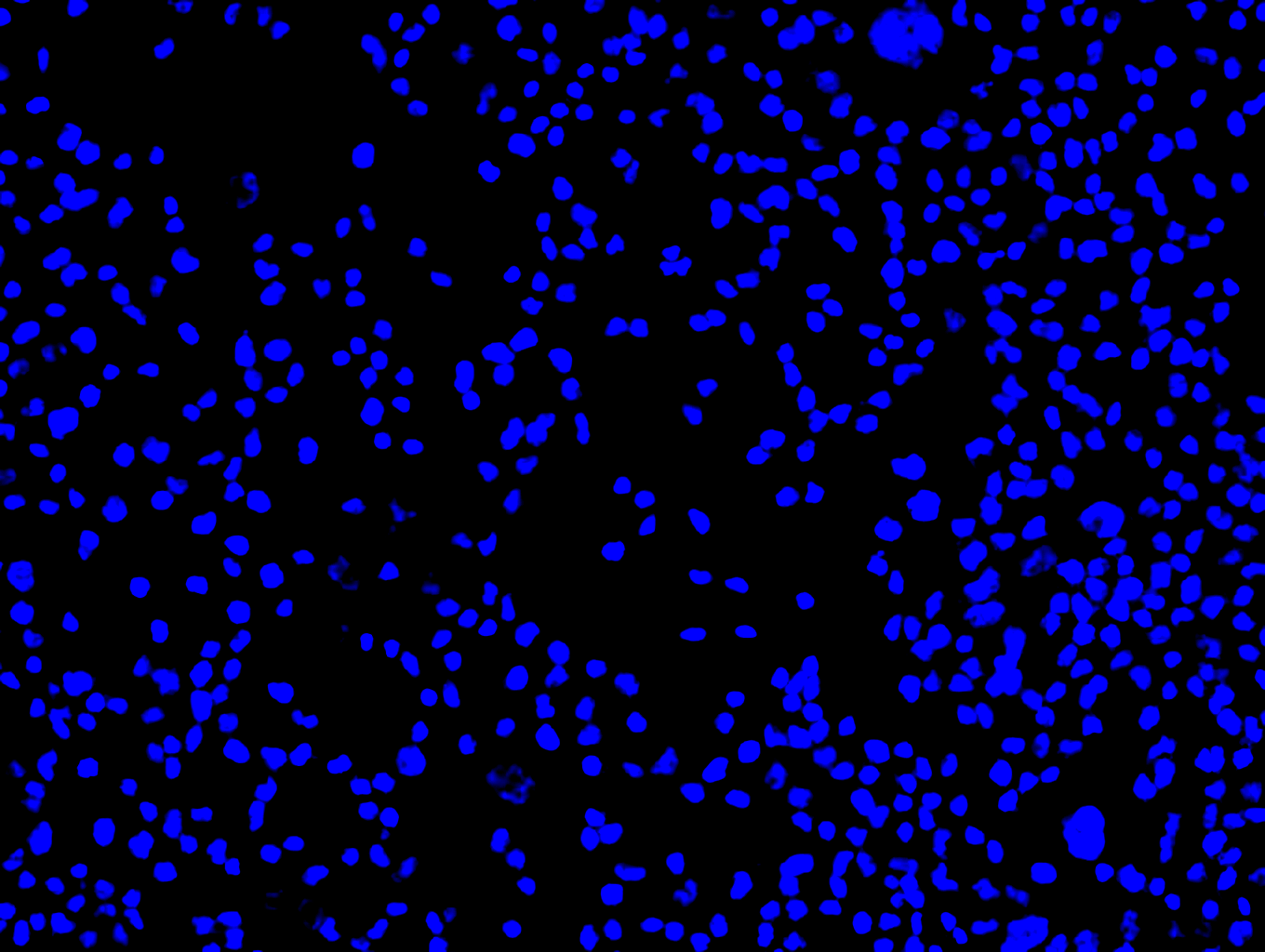

Supplement: Supplementary file 7 — Source data Fig. 2 [file 44318_2024_359_MOESM7_ESM.zip › Figure 2/Fig 2K and 2L/Fig 2K/hSPAR+rapamycin/1-hoechst in manu.tif]

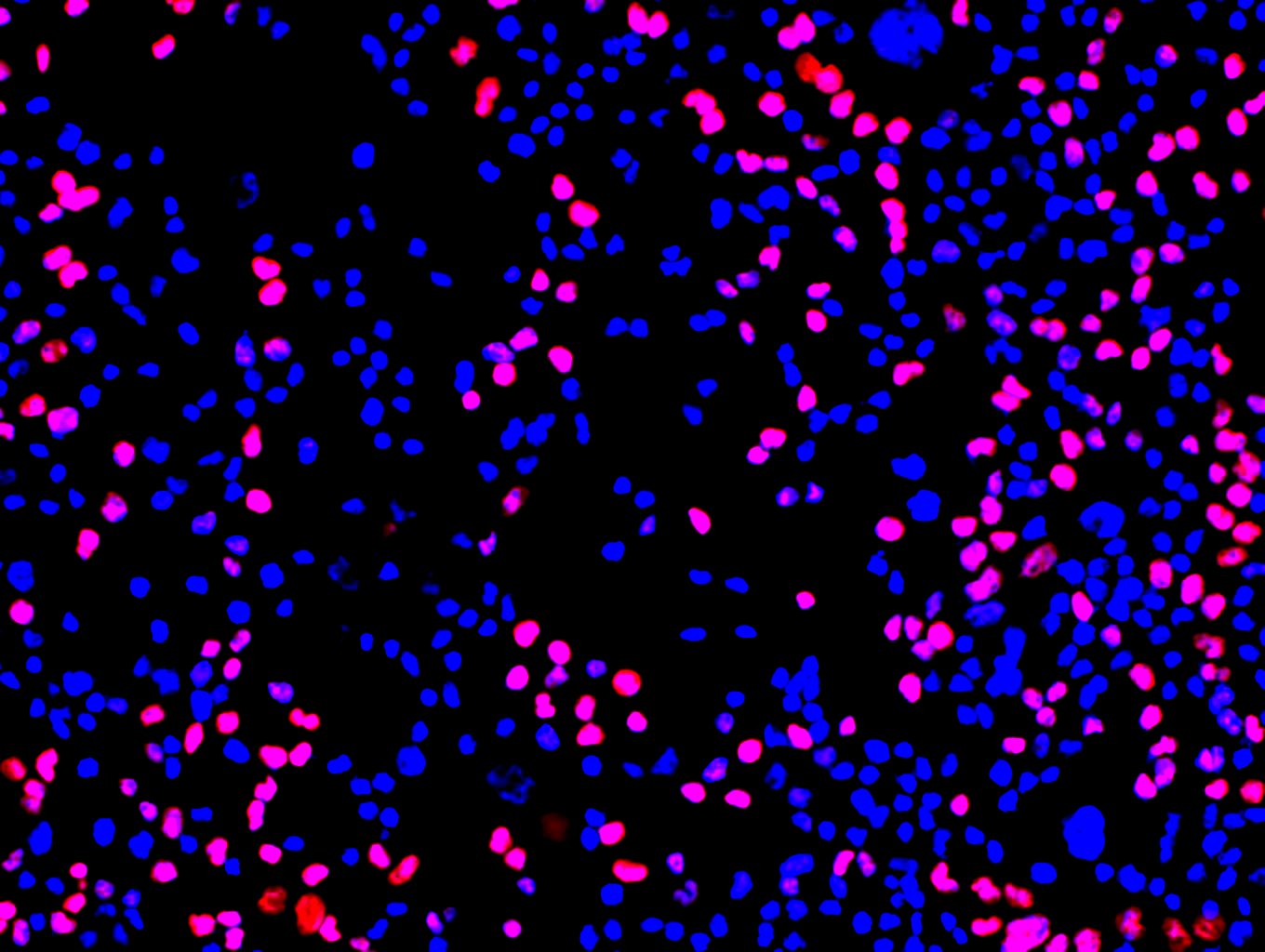

Supplement: Supplementary file 7 — Source data Fig. 2 [file 44318_2024_359_MOESM7_ESM.zip › Figure 2/Fig 2K and 2L/Fig 2K/hSPAR+rapamycin/1-merge in manu.jpg]

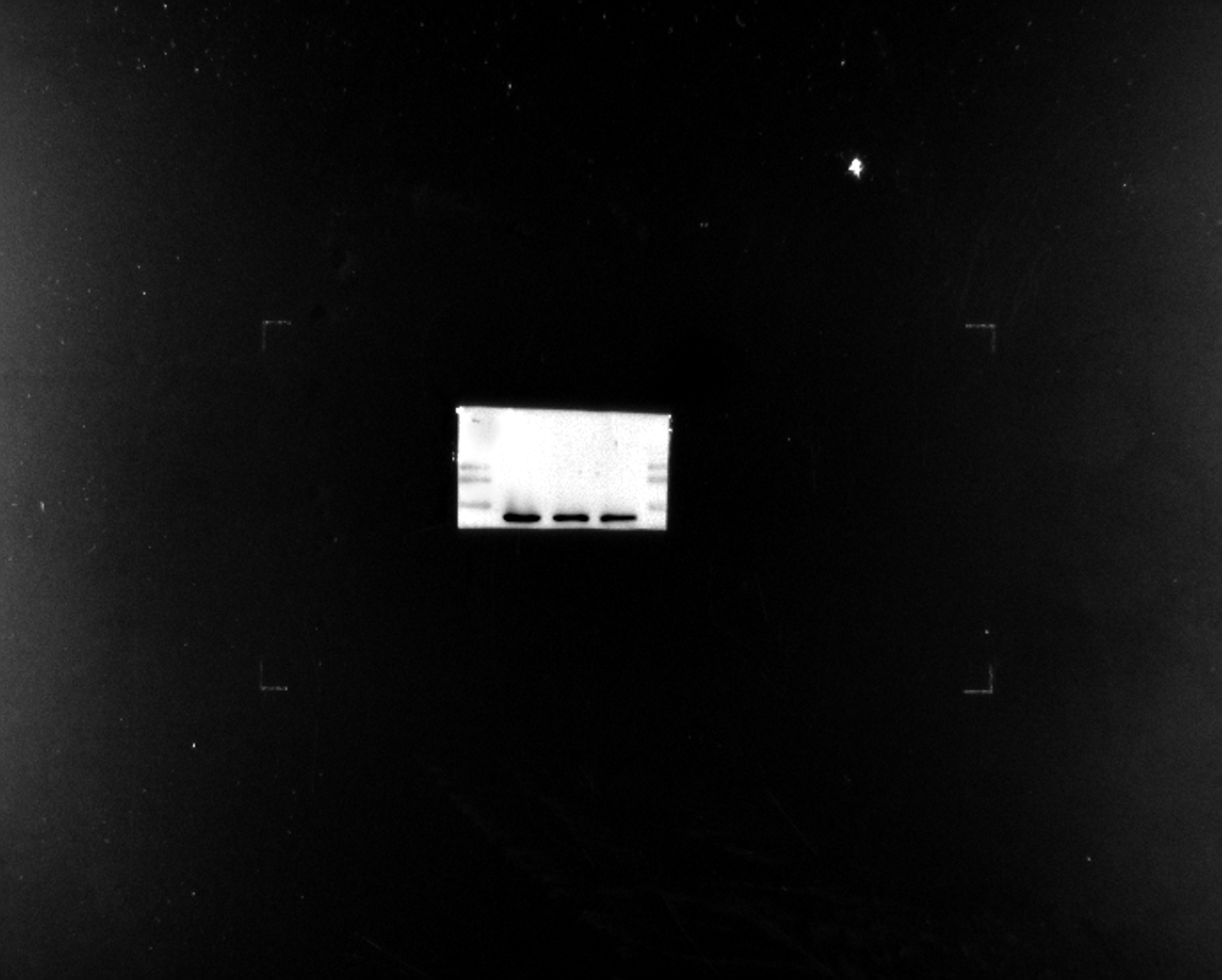

Supplement: Supplementary file 8 — Source data Fig. 3 [file 44318_2024_359_MOESM8_ESM.zip › Figure 3/Fig 3B/1-input-TRIM21-merge.Tif]

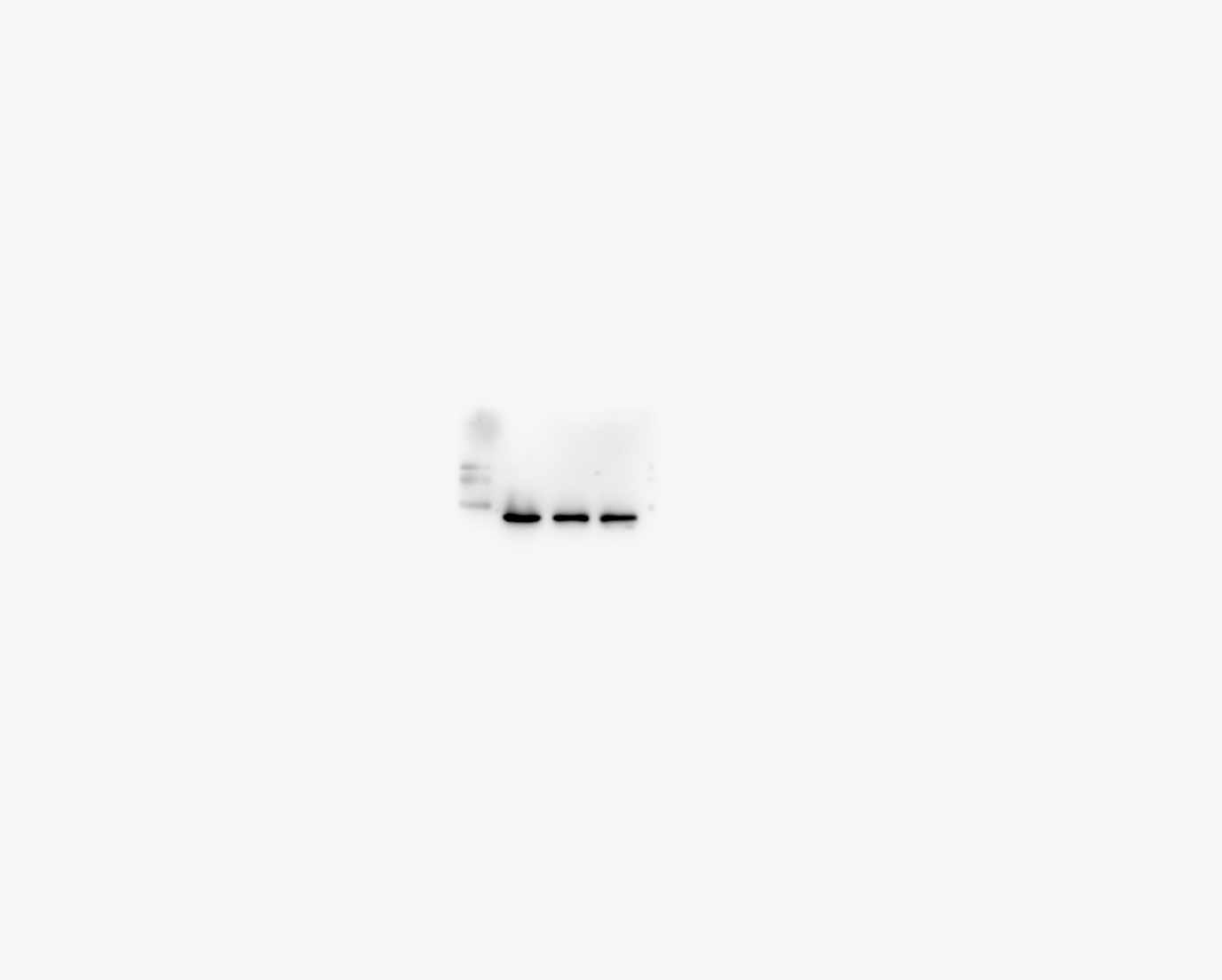

Supplement: Supplementary file 8 — Source data Fig. 3 [file 44318_2024_359_MOESM8_ESM.zip › Figure 3/Fig 3B/1-input-TRIM21.Tif]

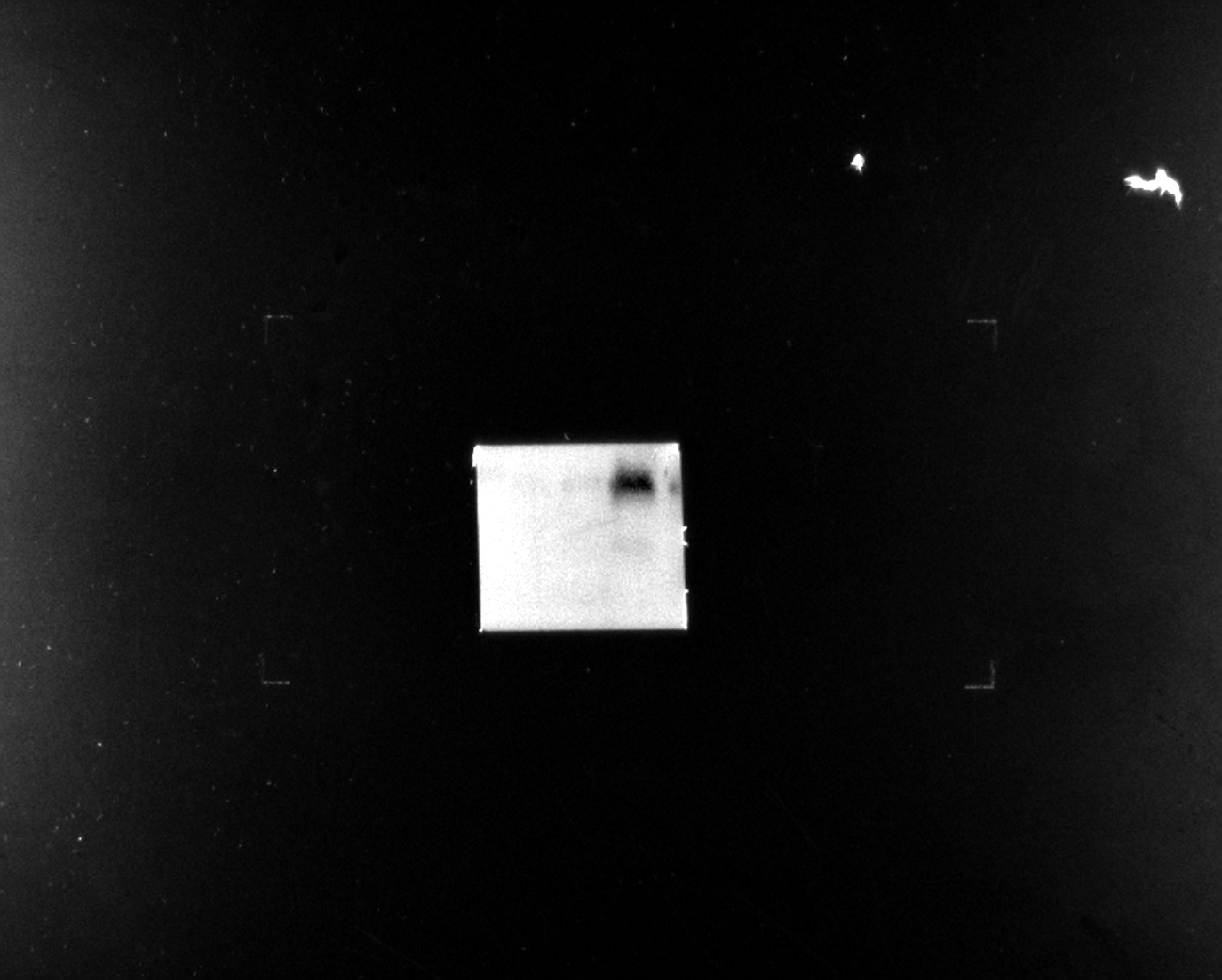

Supplement: Supplementary file 8 — Source data Fig. 3 [file 44318_2024_359_MOESM8_ESM.zip › Figure 3/Fig 3B/2-input-Flag-merge.Tif]

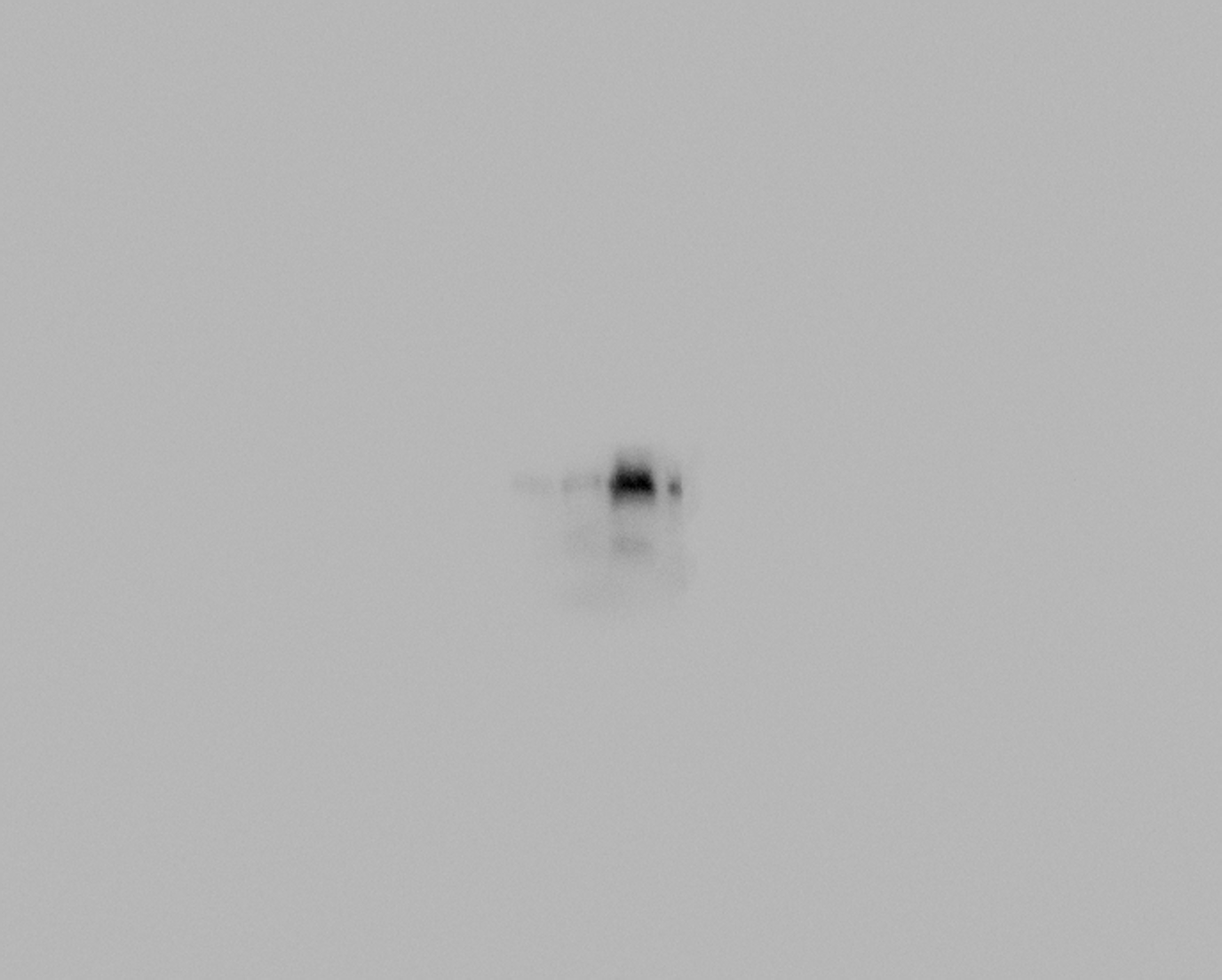

Supplement: Supplementary file 8 — Source data Fig. 3 [file 44318_2024_359_MOESM8_ESM.zip › Figure 3/Fig 3B/2-input-Flag.Tif]

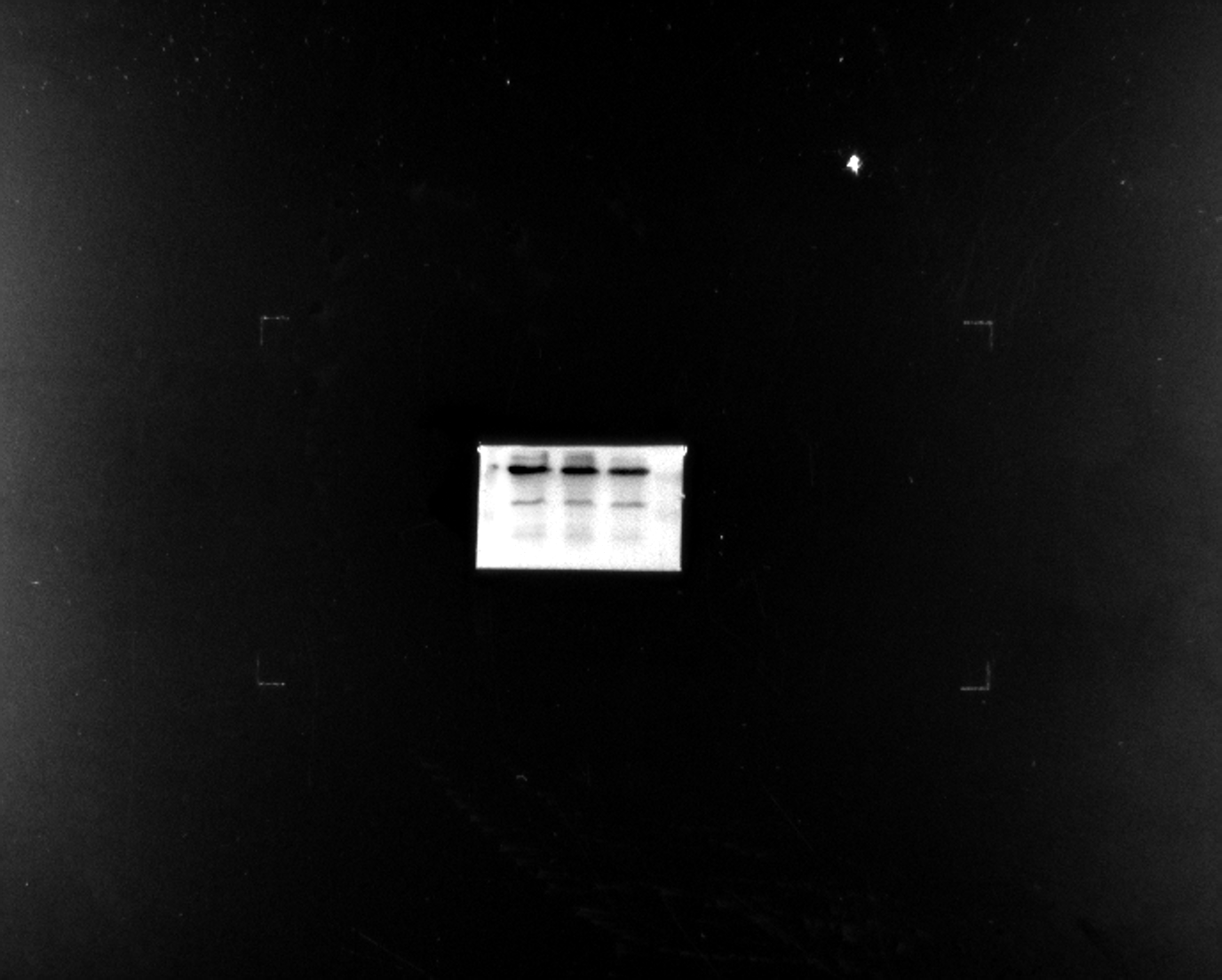

Supplement: Supplementary file 8 — Source data Fig. 3 [file 44318_2024_359_MOESM8_ESM.zip › Figure 3/Fig 3B/3-input-GAPDH-merge.Tif]

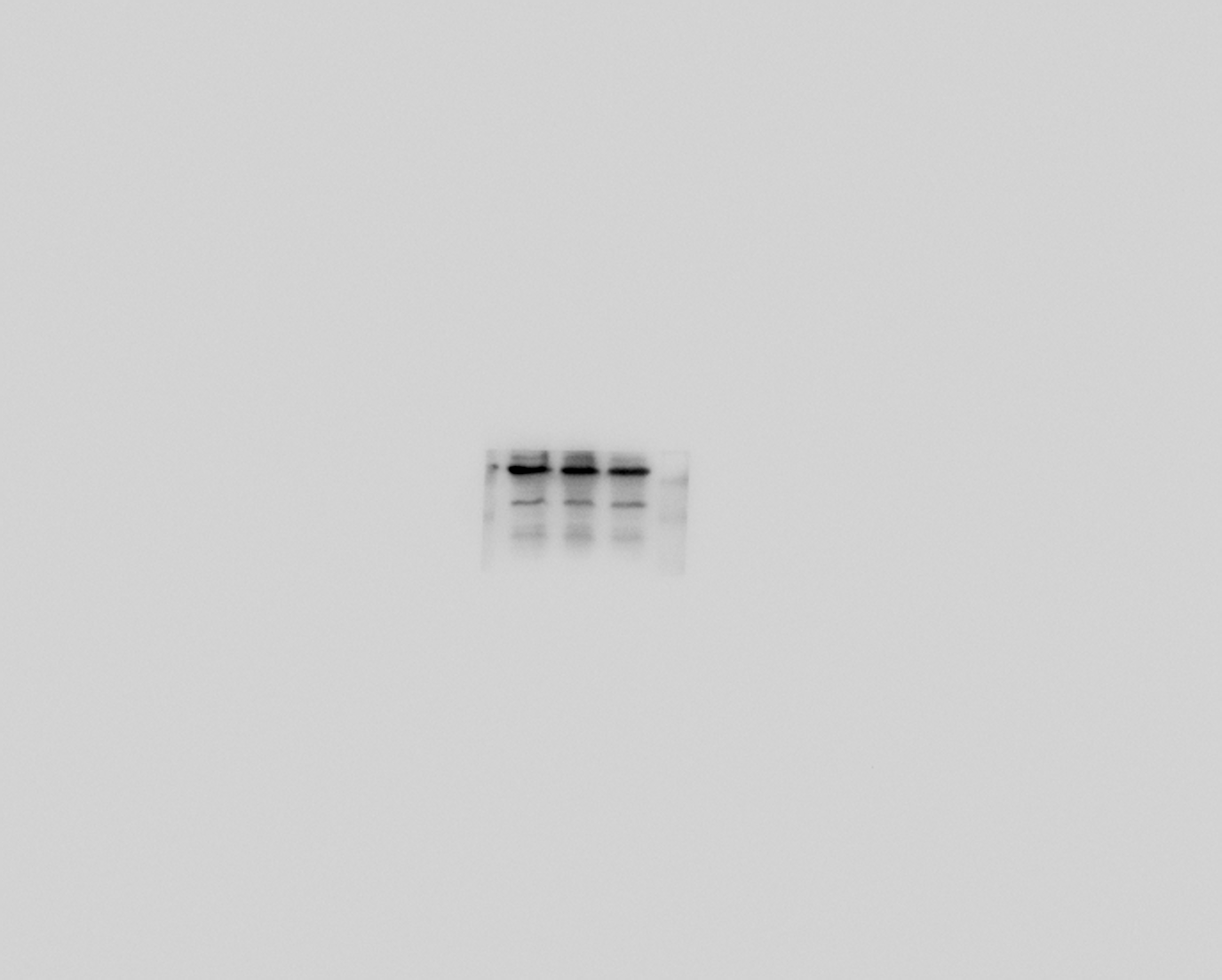

Supplement: Supplementary file 8 — Source data Fig. 3 [file 44318_2024_359_MOESM8_ESM.zip › Figure 3/Fig 3B/3-input-GAPDH.Tif]

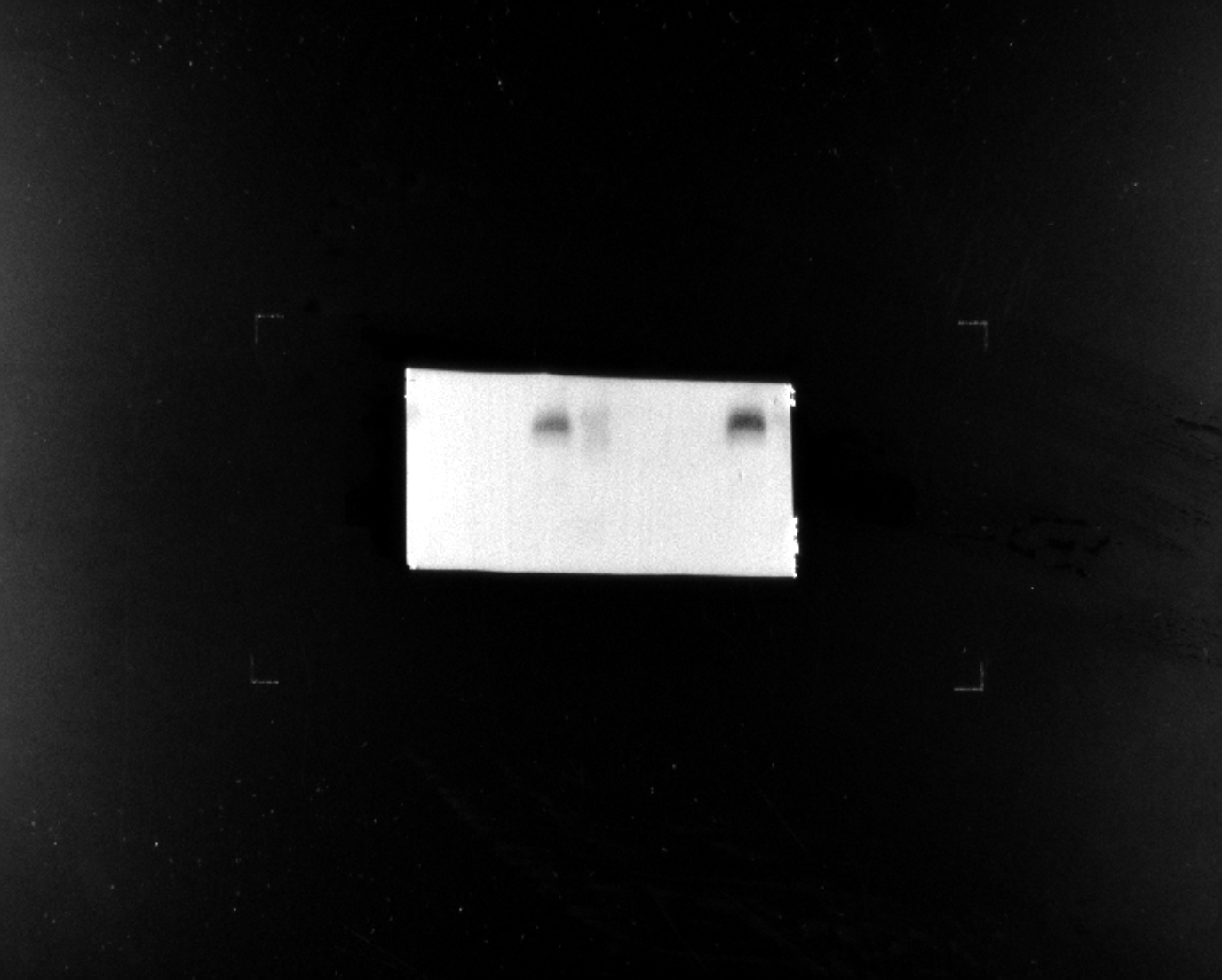

Supplement: Supplementary file 8 — Source data Fig. 3 [file 44318_2024_359_MOESM8_ESM.zip › Figure 3/Fig 3B/4-ip-TRIM21 wb Flag+ip Flag wb Flag-Flag-merge.Tif]

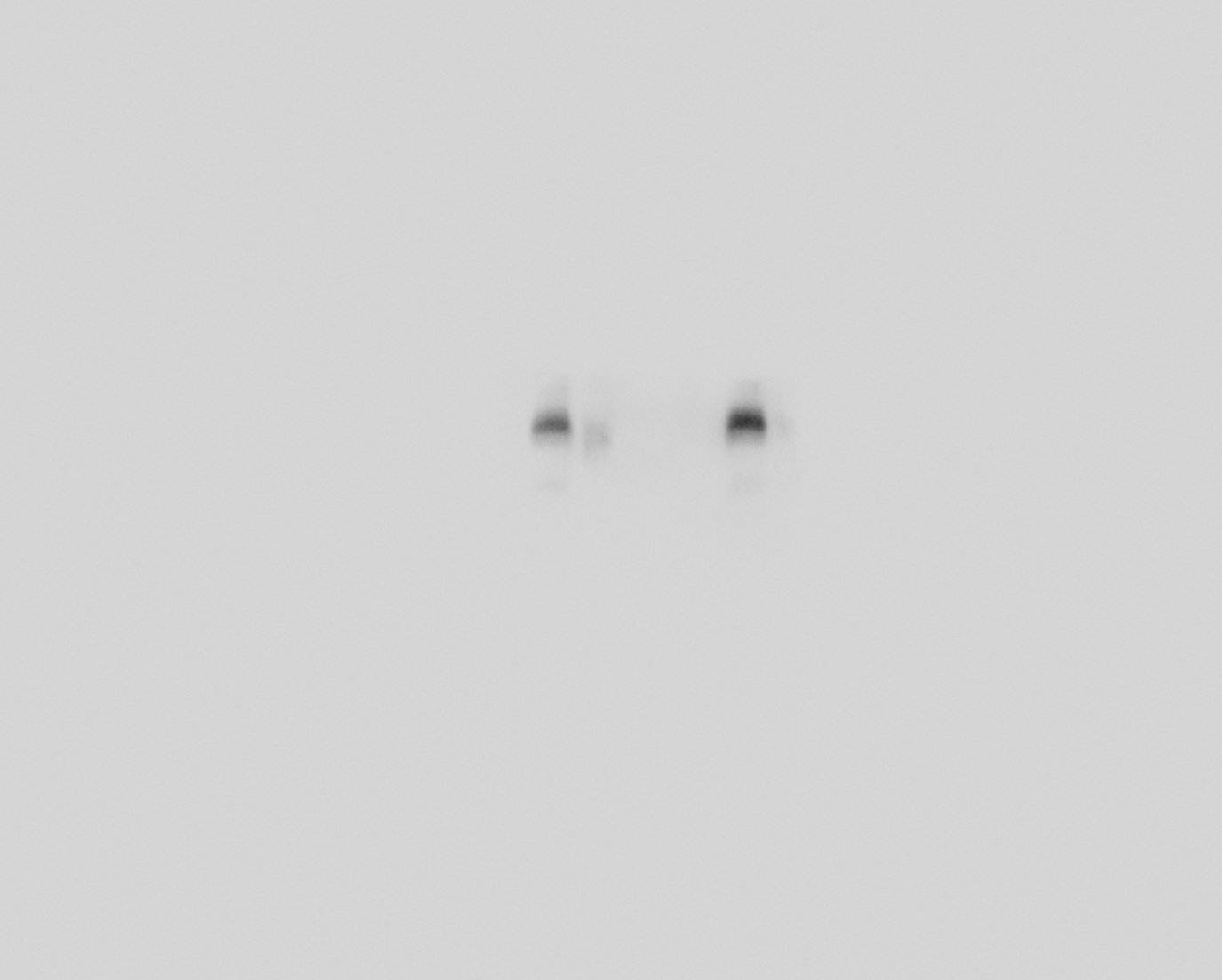

Supplement: Supplementary file 8 — Source data Fig. 3 [file 44318_2024_359_MOESM8_ESM.zip › Figure 3/Fig 3B/4-ip-TRIM21 wb Flag+ip Flag wb Flag-Flag.Tif]

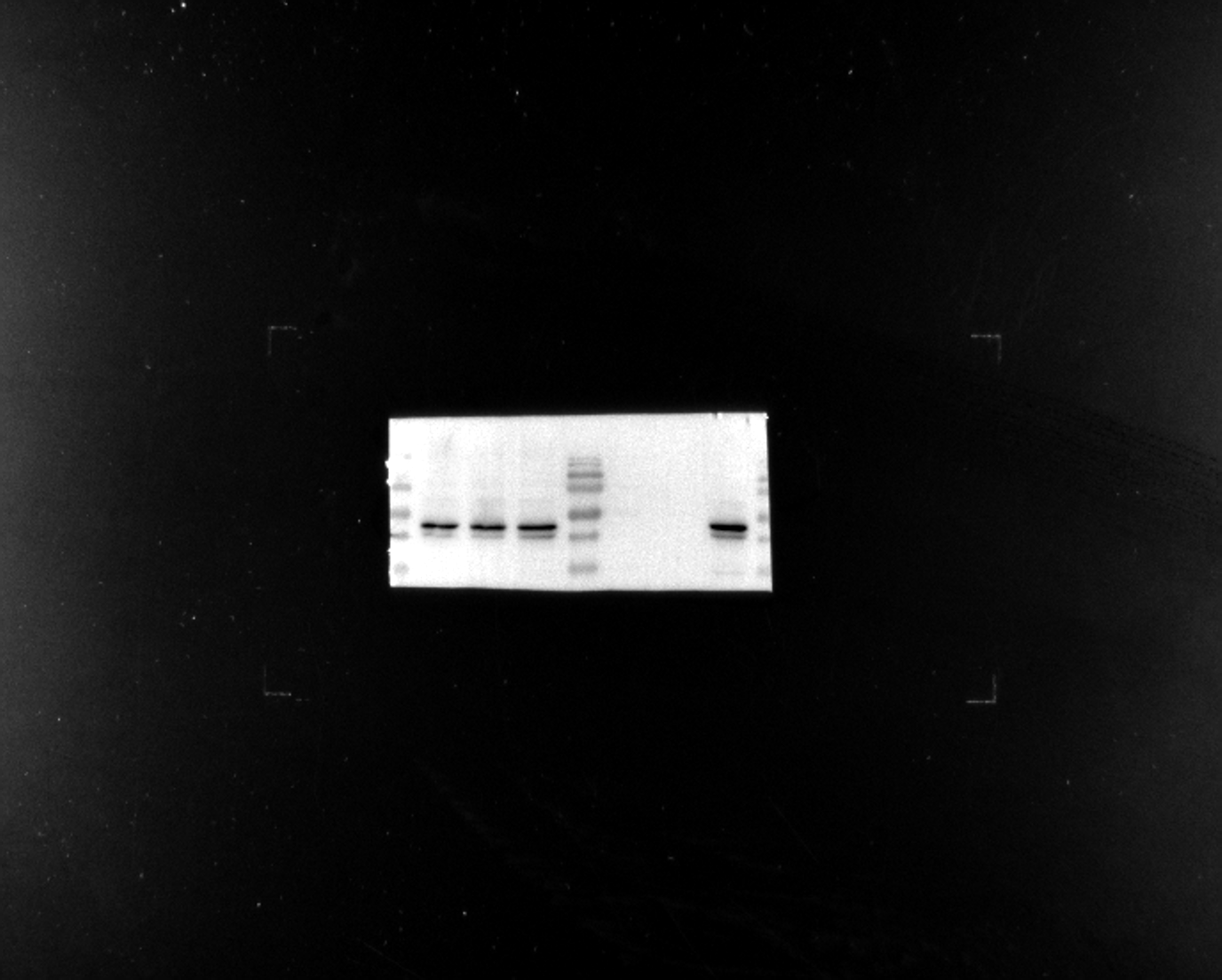

Supplement: Supplementary file 8 — Source data Fig. 3 [file 44318_2024_359_MOESM8_ESM.zip › Figure 3/Fig 3B/5-ip-TRIM21 wb Flag+ip Flag wb Flag-TRIM21-merge.Tif]

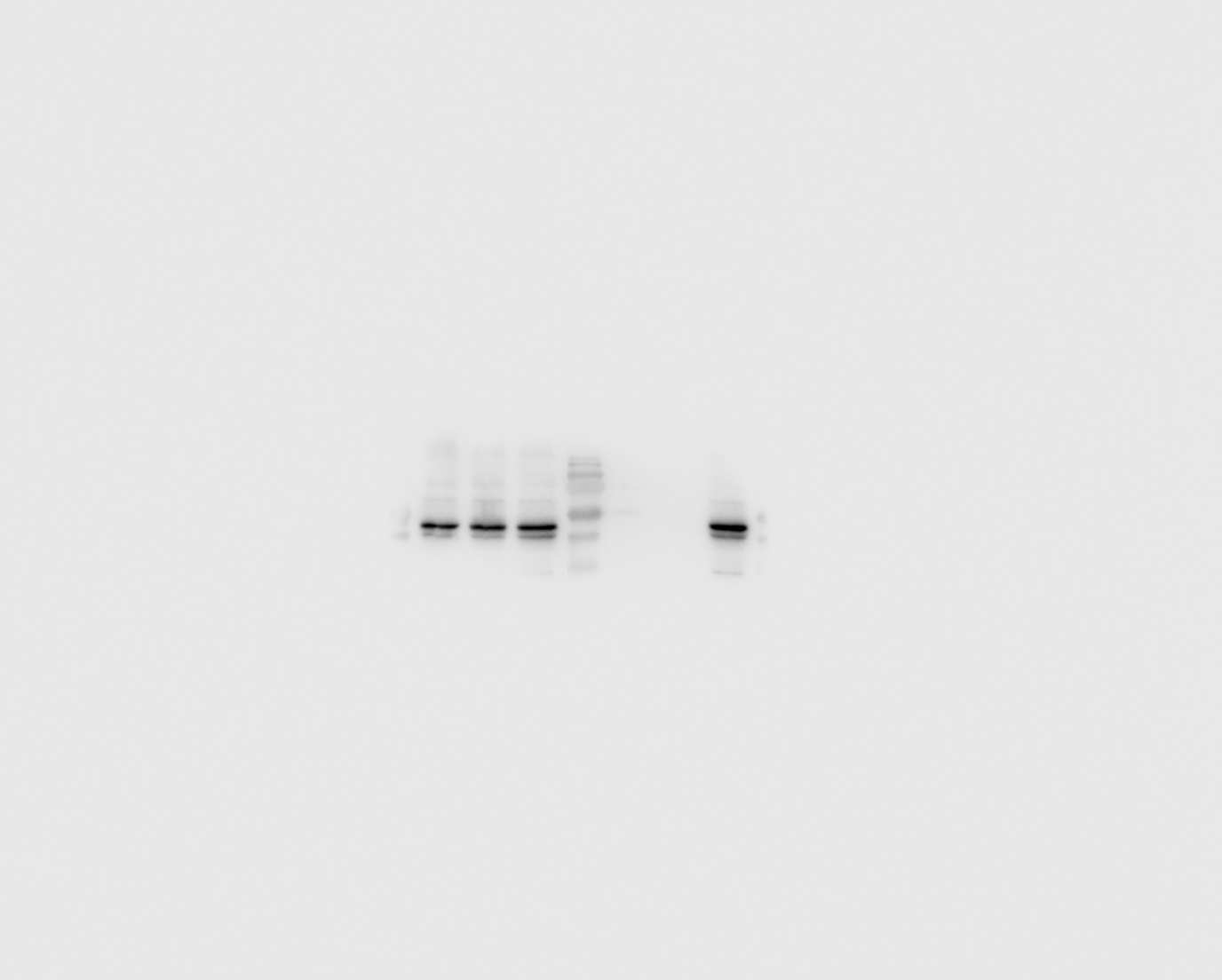

Supplement: Supplementary file 8 — Source data Fig. 3 [file 44318_2024_359_MOESM8_ESM.zip › Figure 3/Fig 3B/5-ip-TRIM21 wb Flag+ip Flag wb Flag-TRIM21.Tif]

**Fig. 3B**

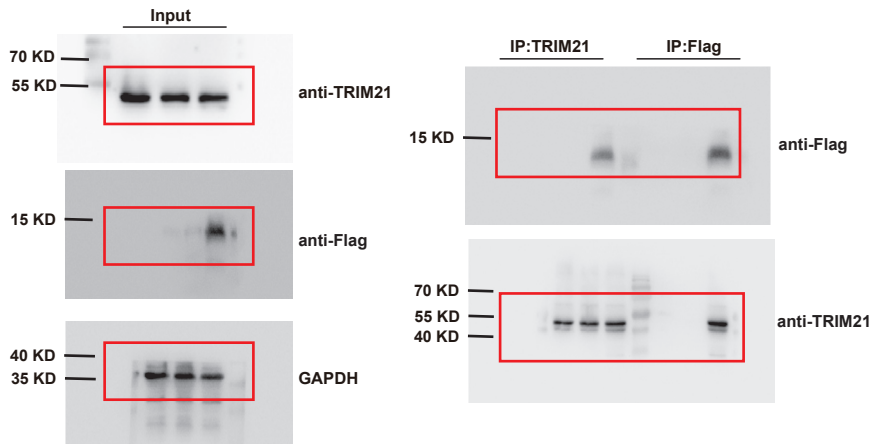

Supplement: Supplementary file 8 — Source data Fig. 3 [file 44318_2024_359_MOESM8_ESM.zip › Figure 3/Fig 3B/Fig 3B.pdf]

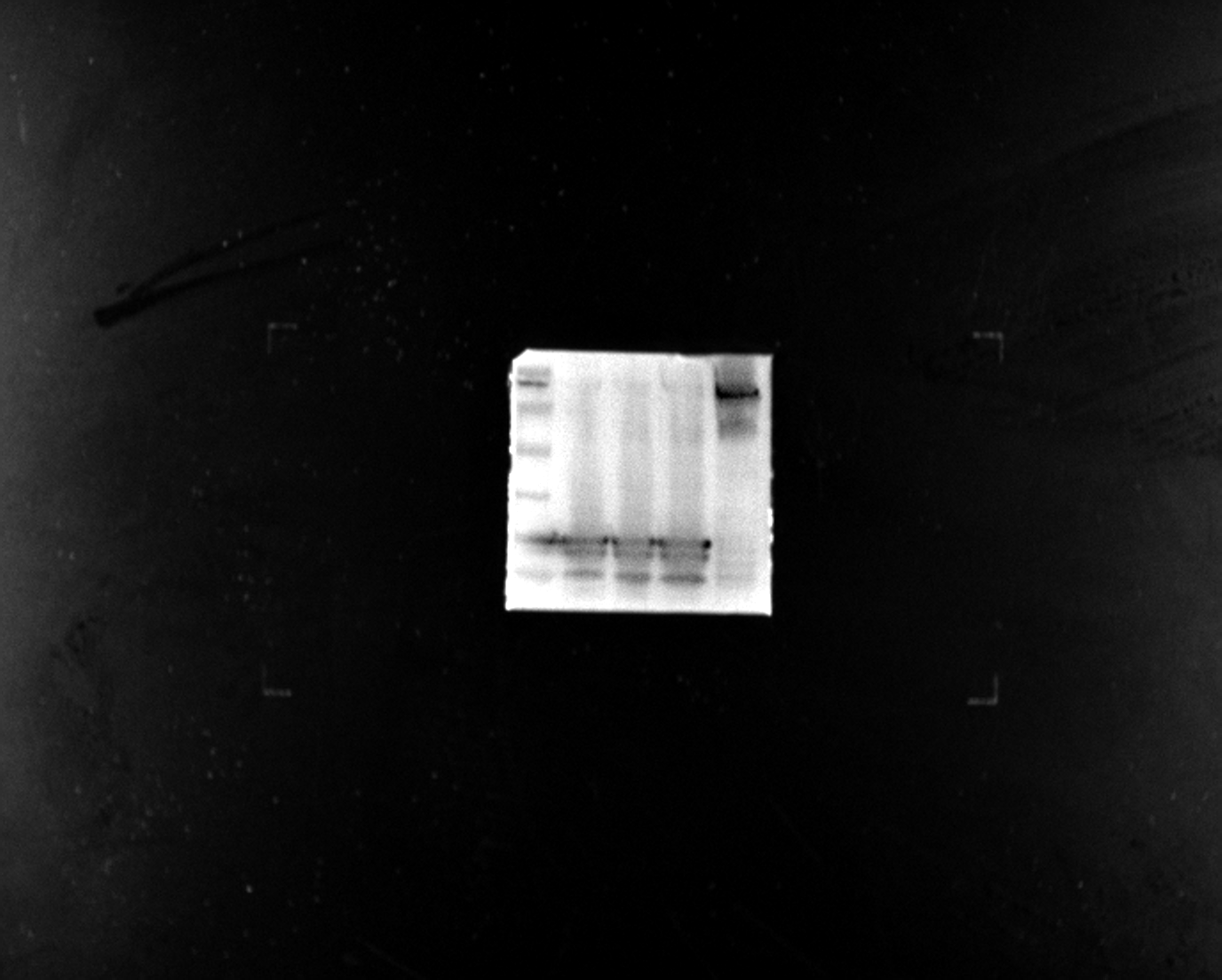

Supplement: Supplementary file 8 — Source data Fig. 3 [file 44318_2024_359_MOESM8_ESM.zip › Figure 3/Fig 3C and 3D/Fig 3C/1-GFP-merge.Tif]

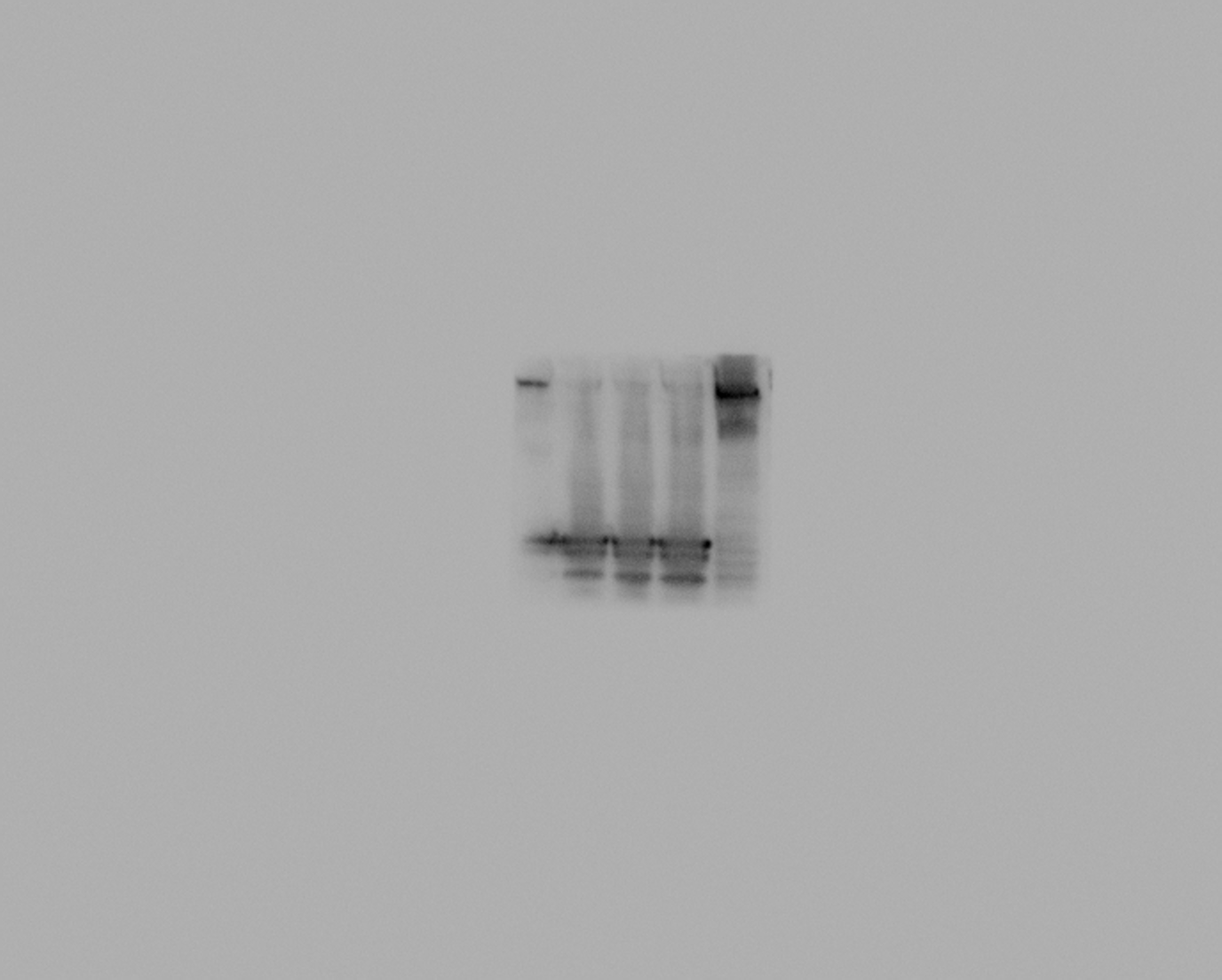

Supplement: Supplementary file 8 — Source data Fig. 3 [file 44318_2024_359_MOESM8_ESM.zip › Figure 3/Fig 3C and 3D/Fig 3C/1-GFP.Tif]

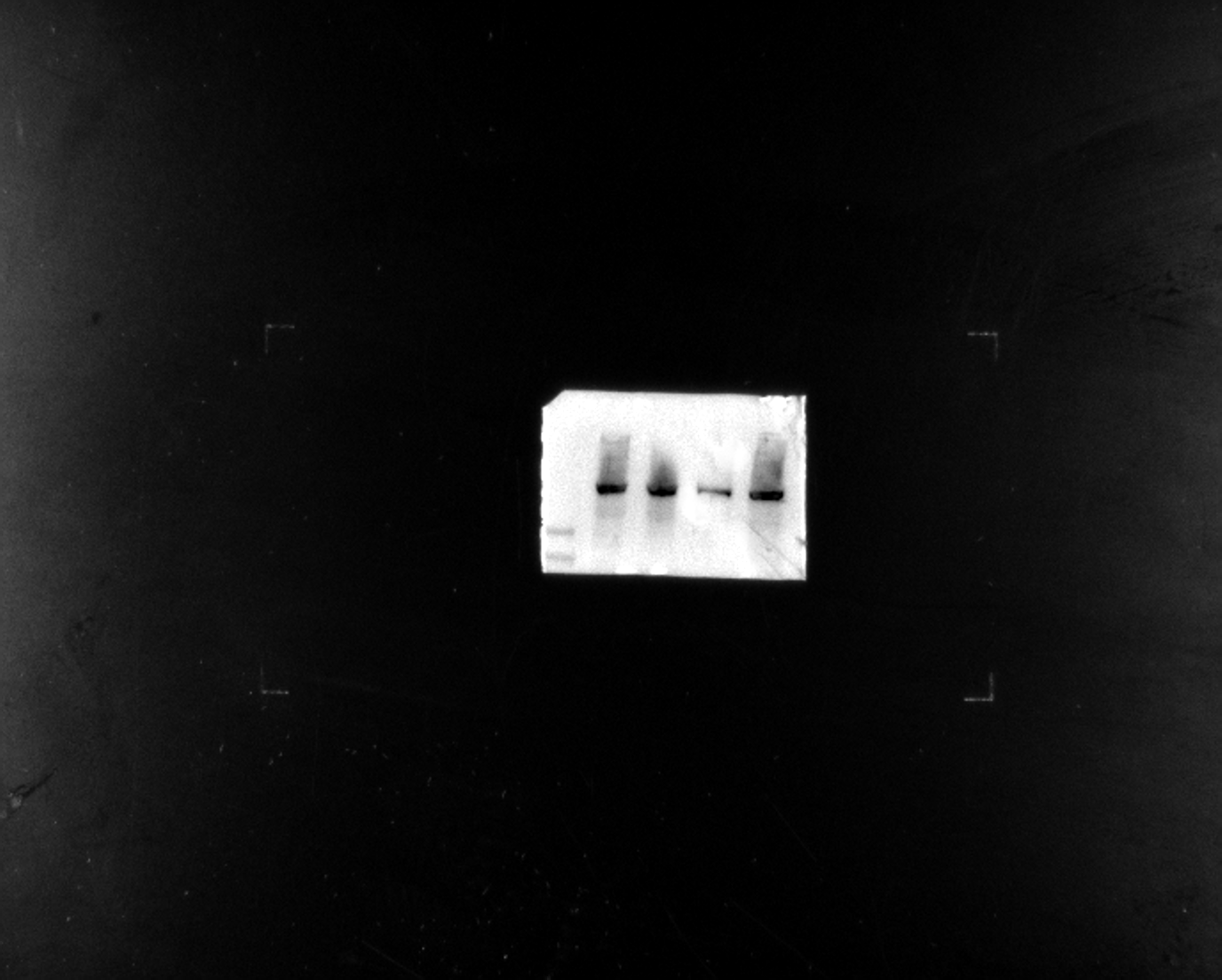

Supplement: Supplementary file 8 — Source data Fig. 3 [file 44318_2024_359_MOESM8_ESM.zip › Figure 3/Fig 3C and 3D/Fig 3C/2-p-mTOR-merge.Tif]

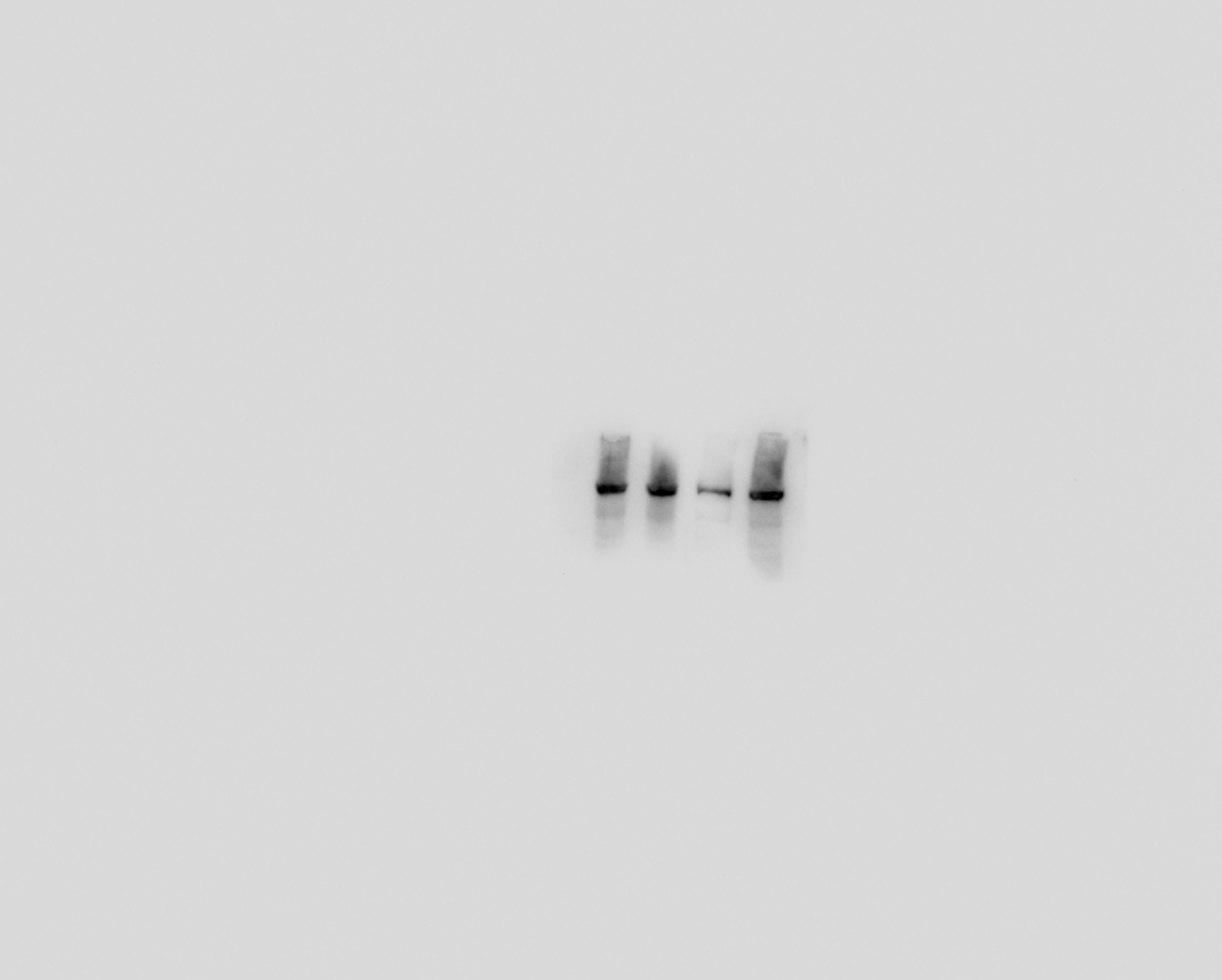

Supplement: Supplementary file 8 — Source data Fig. 3 [file 44318_2024_359_MOESM8_ESM.zip › Figure 3/Fig 3C and 3D/Fig 3C/2-p-mTOR.Tif]

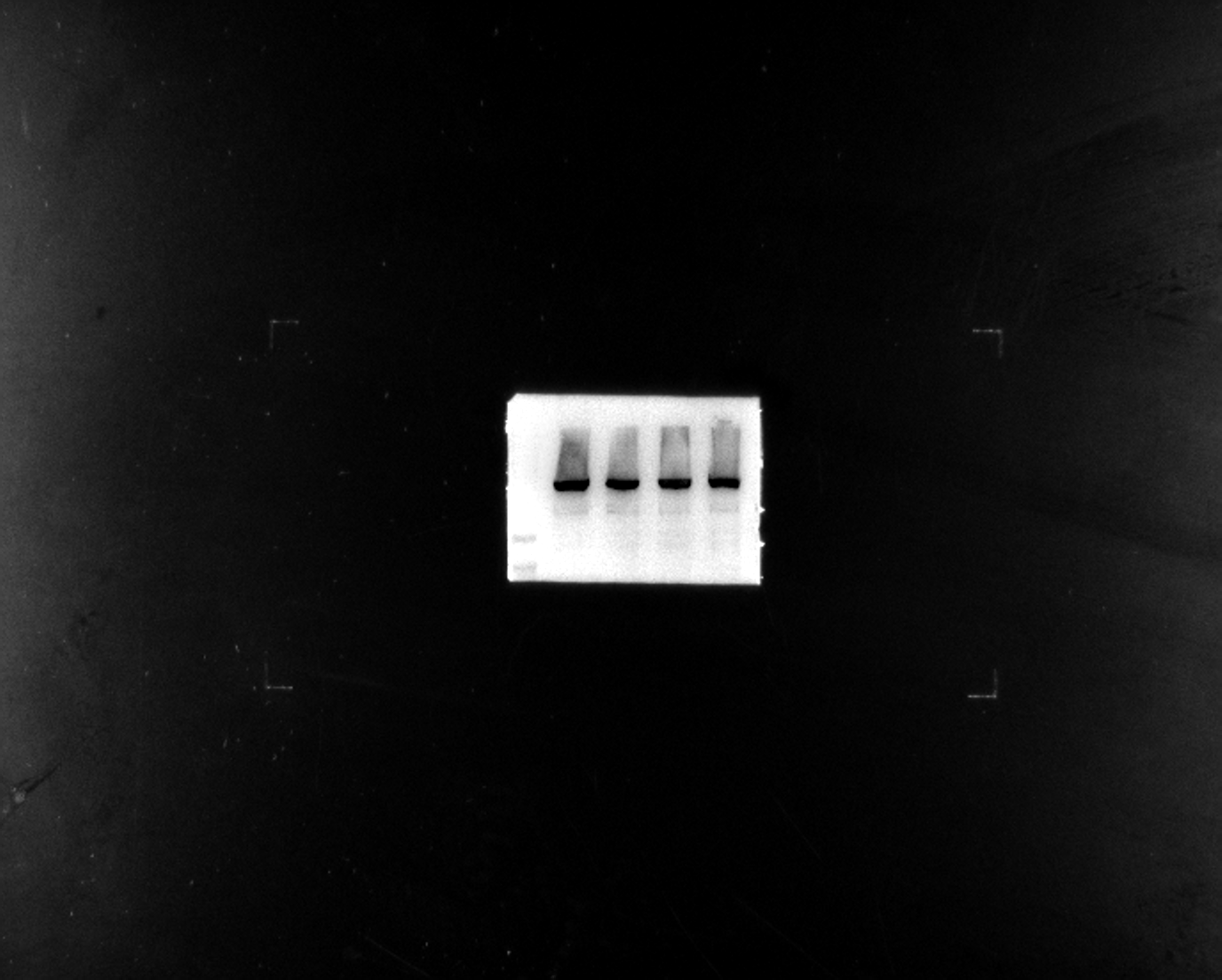

Supplement: Supplementary file 8 — Source data Fig. 3 [file 44318_2024_359_MOESM8_ESM.zip › Figure 3/Fig 3C and 3D/Fig 3C/3-mTOR-merge.Tif]

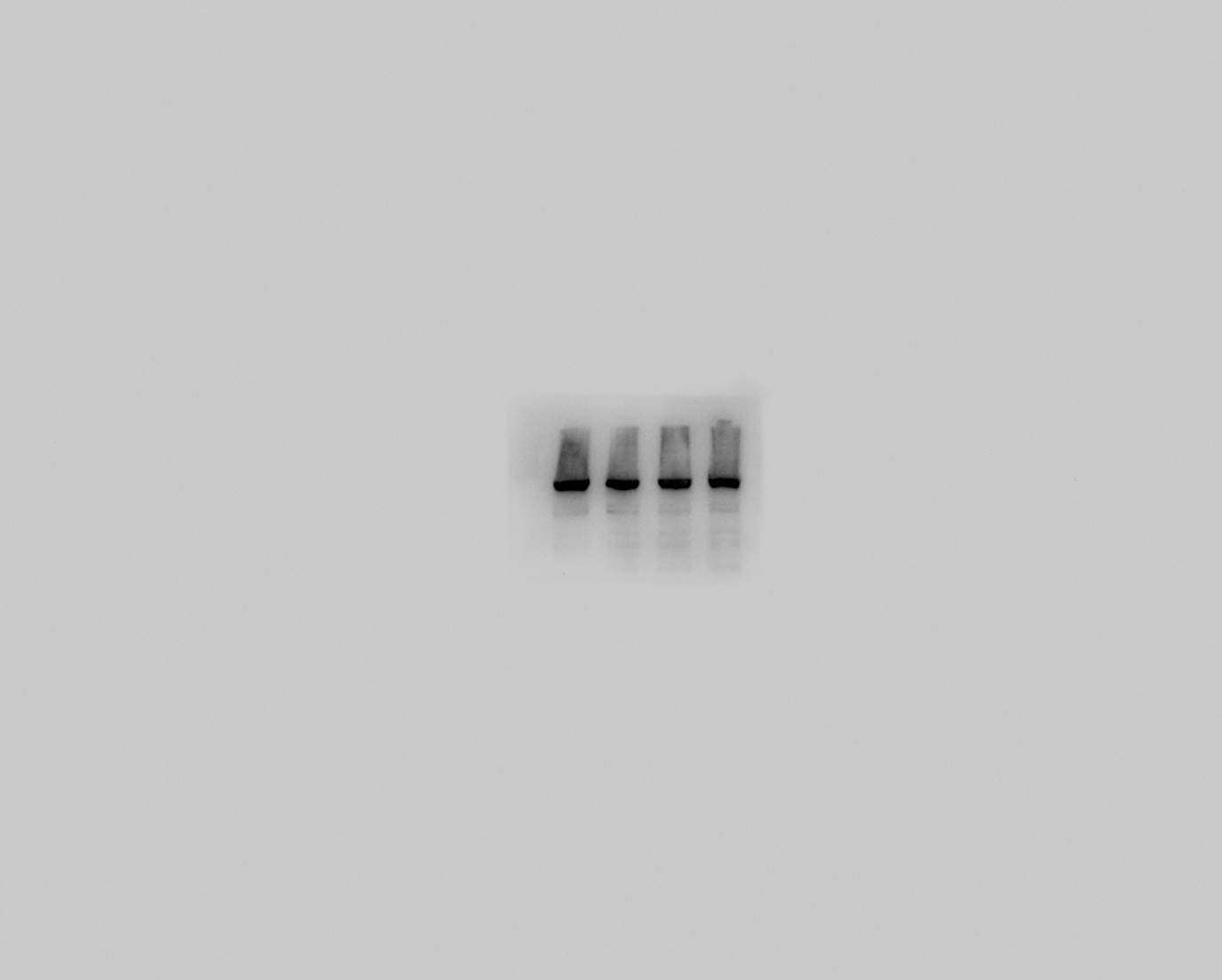

Supplement: Supplementary file 8 — Source data Fig. 3 [file 44318_2024_359_MOESM8_ESM.zip › Figure 3/Fig 3C and 3D/Fig 3C/3-mtor.Tif]

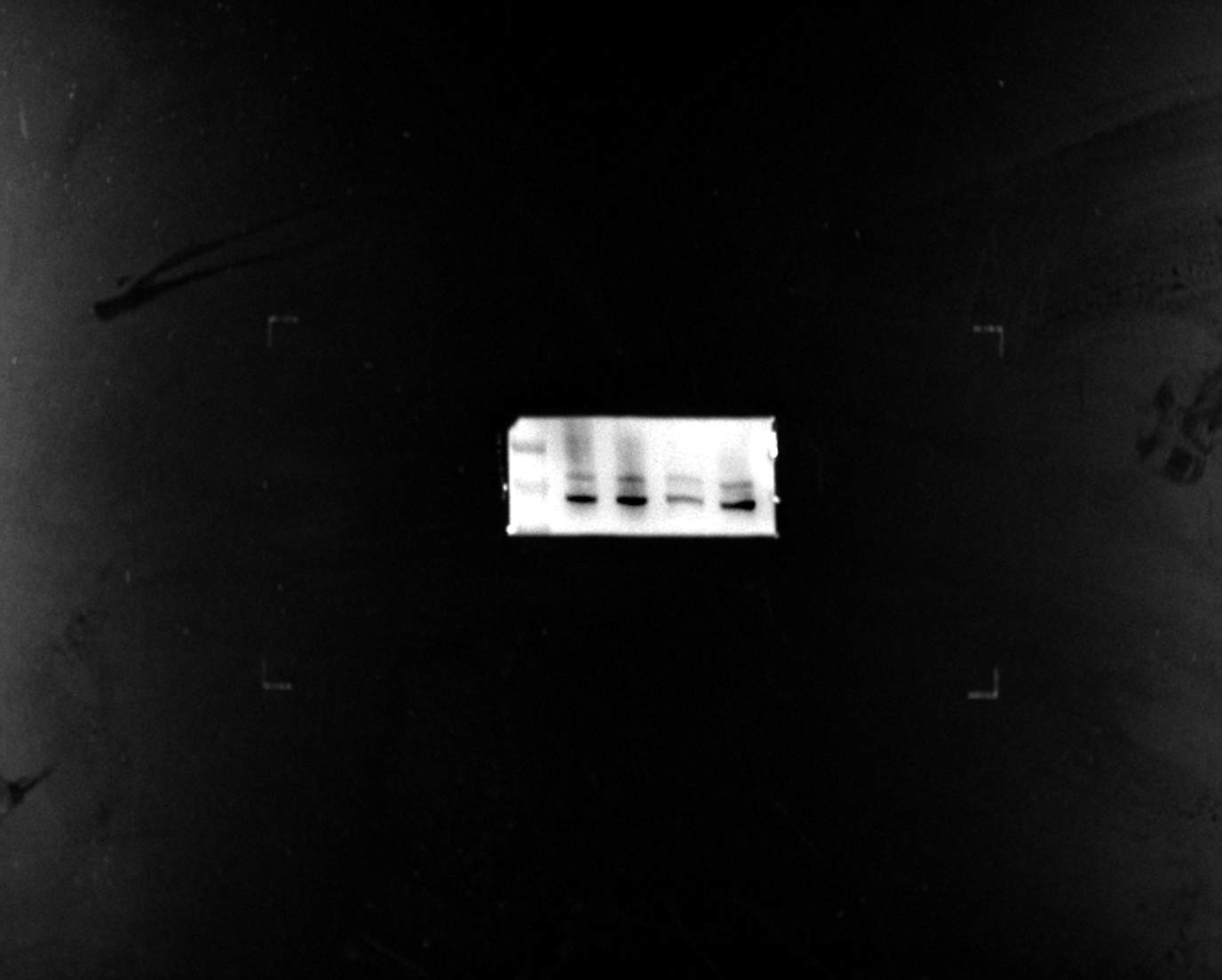

Supplement: Supplementary file 8 — Source data Fig. 3 [file 44318_2024_359_MOESM8_ESM.zip › Figure 3/Fig 3C and 3D/Fig 3C/4-p-S6K-merge.Tif]

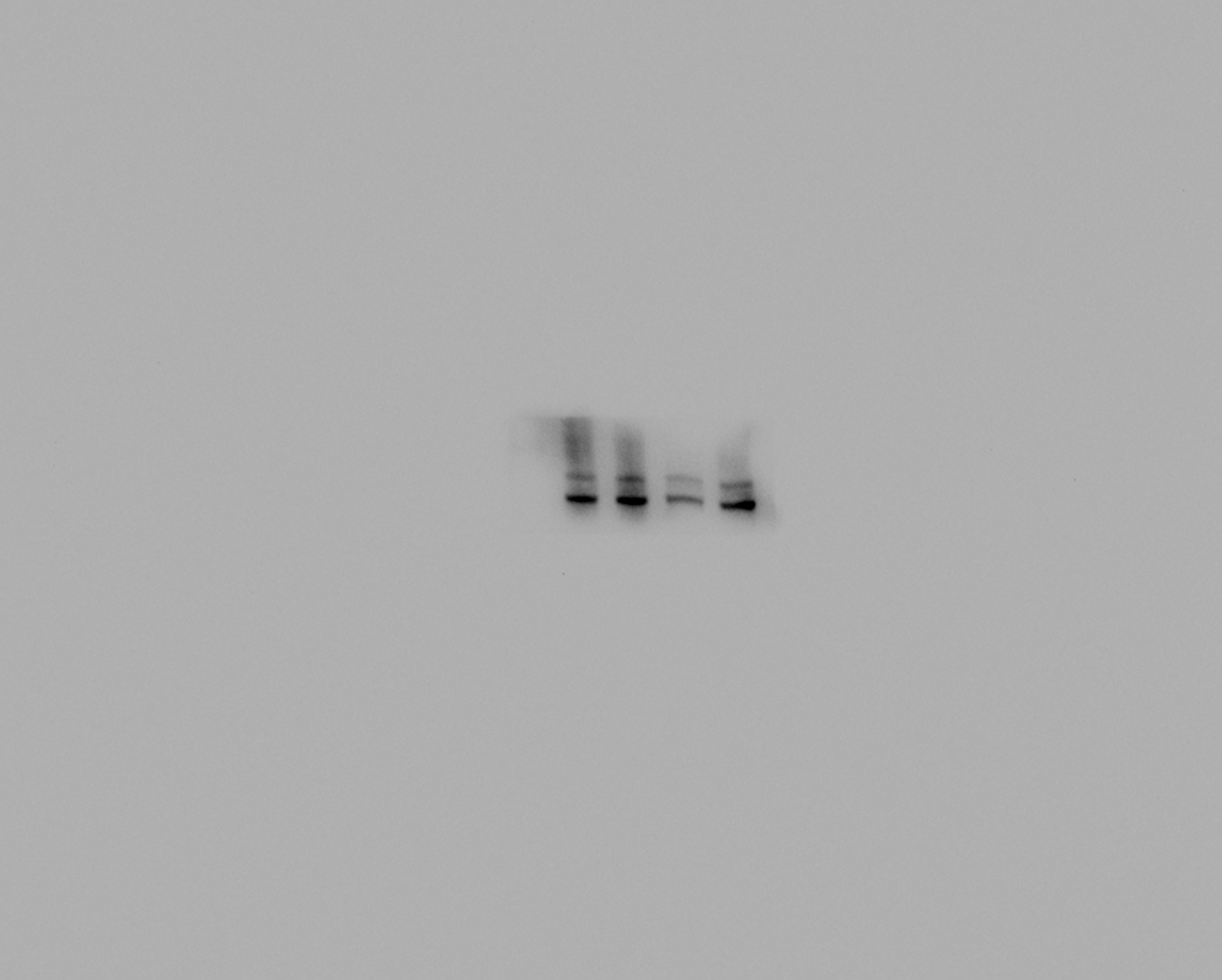

Supplement: Supplementary file 8 — Source data Fig. 3 [file 44318_2024_359_MOESM8_ESM.zip › Figure 3/Fig 3C and 3D/Fig 3C/4-p-S6K.Tif]
