# Supplementary figures and images for: Micropeptide hSPAR regulates glutamine levels and suppresses mammary tumor growth via a TRIM21-P27KIP1-mTOR axis (part 3 of 7)
Source: EMBO J. 2025 Jan 28;44(5):1414–41. doi: 10.1038/s44318-024-00359-z (PMC11876615; doi:10.1038/s44318-024-00359-z)

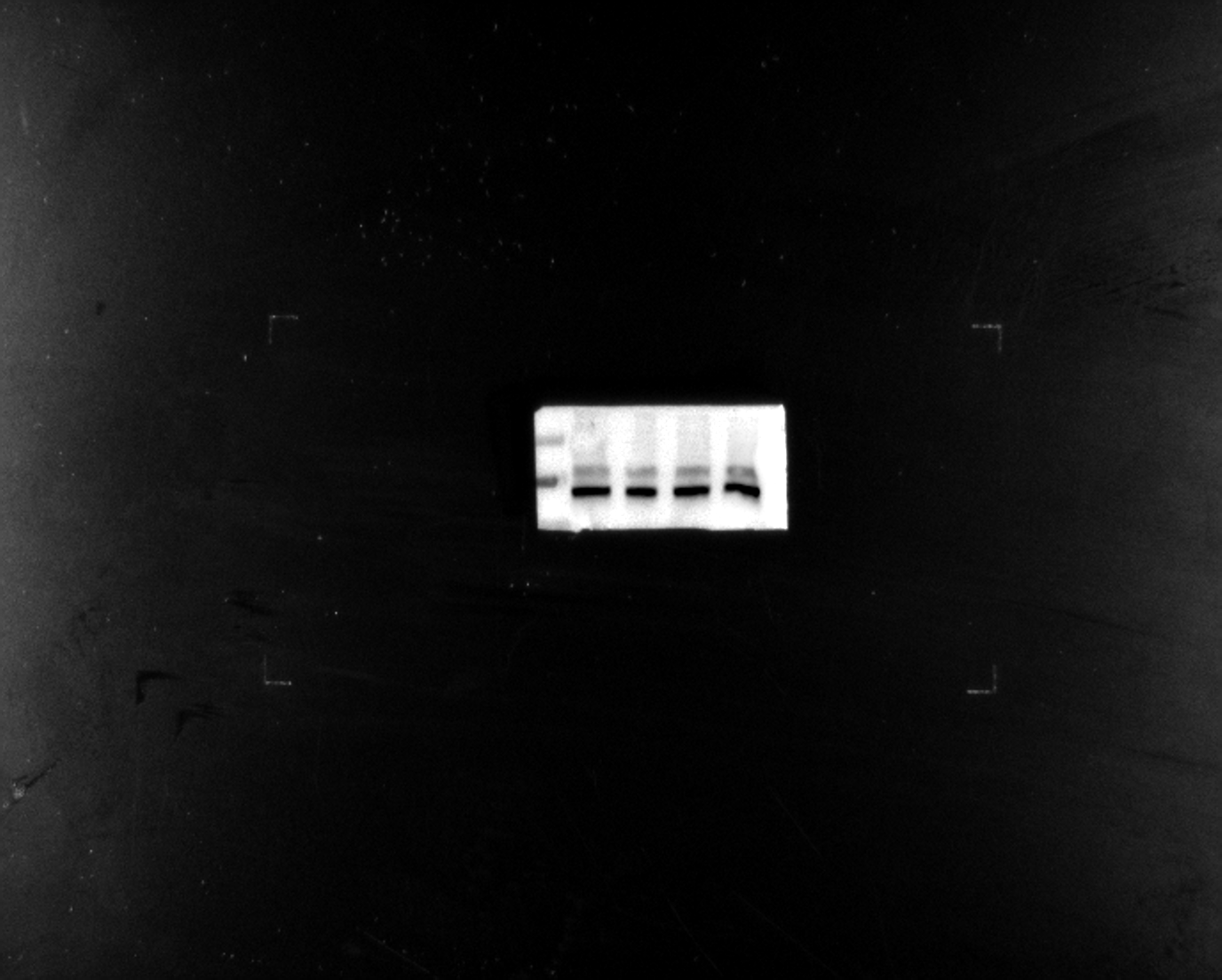

Supplement: Supplementary file 8 — Source data Fig. 3 [file 44318_2024_359_MOESM8_ESM.zip › Figure 3/Fig 3C and 3D/Fig 3C/5-S6K-merge.Tif]

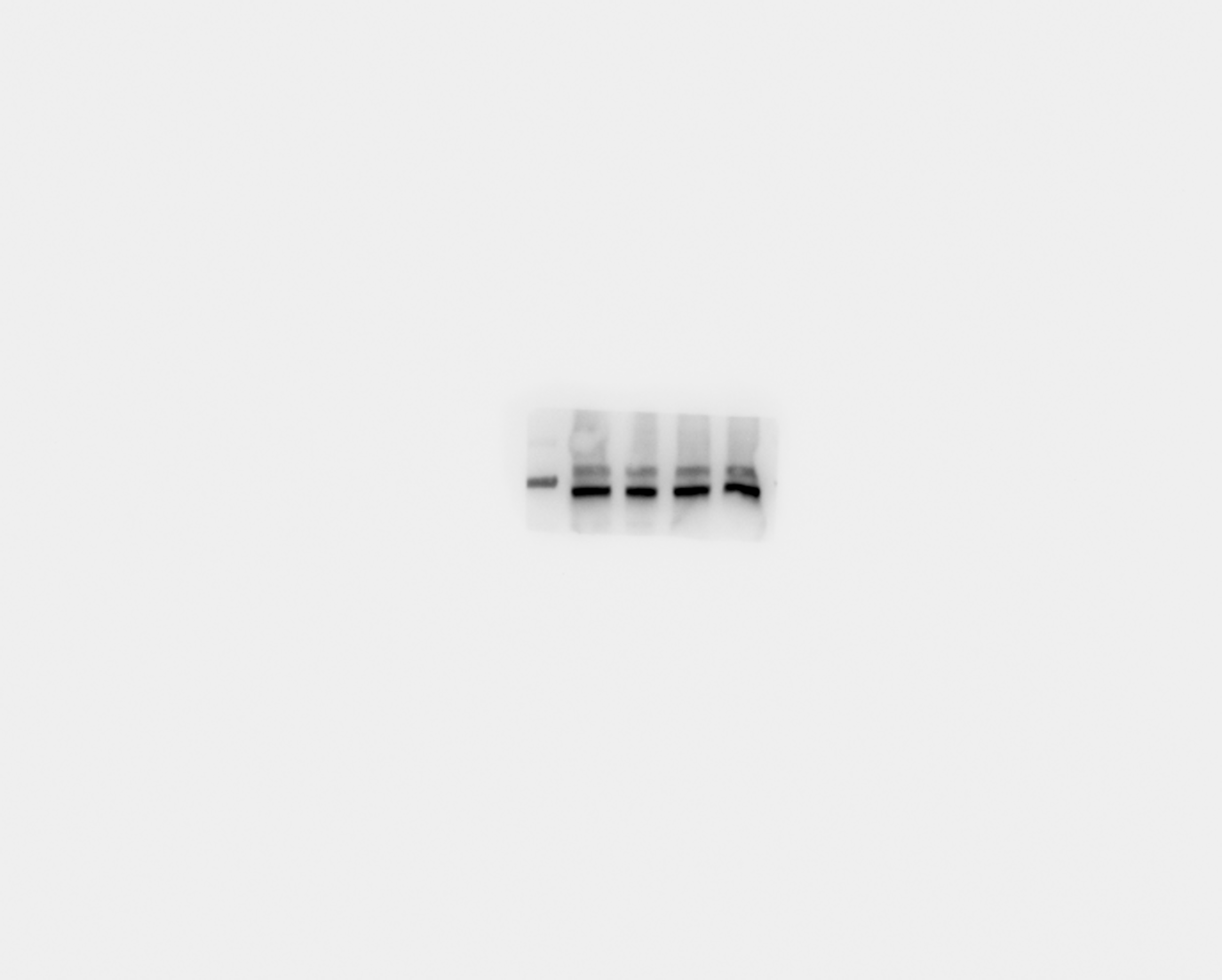

Supplement: Supplementary file 8 — Source data Fig. 3 [file 44318_2024_359_MOESM8_ESM.zip › Figure 3/Fig 3C and 3D/Fig 3C/5-S6K.Tif]

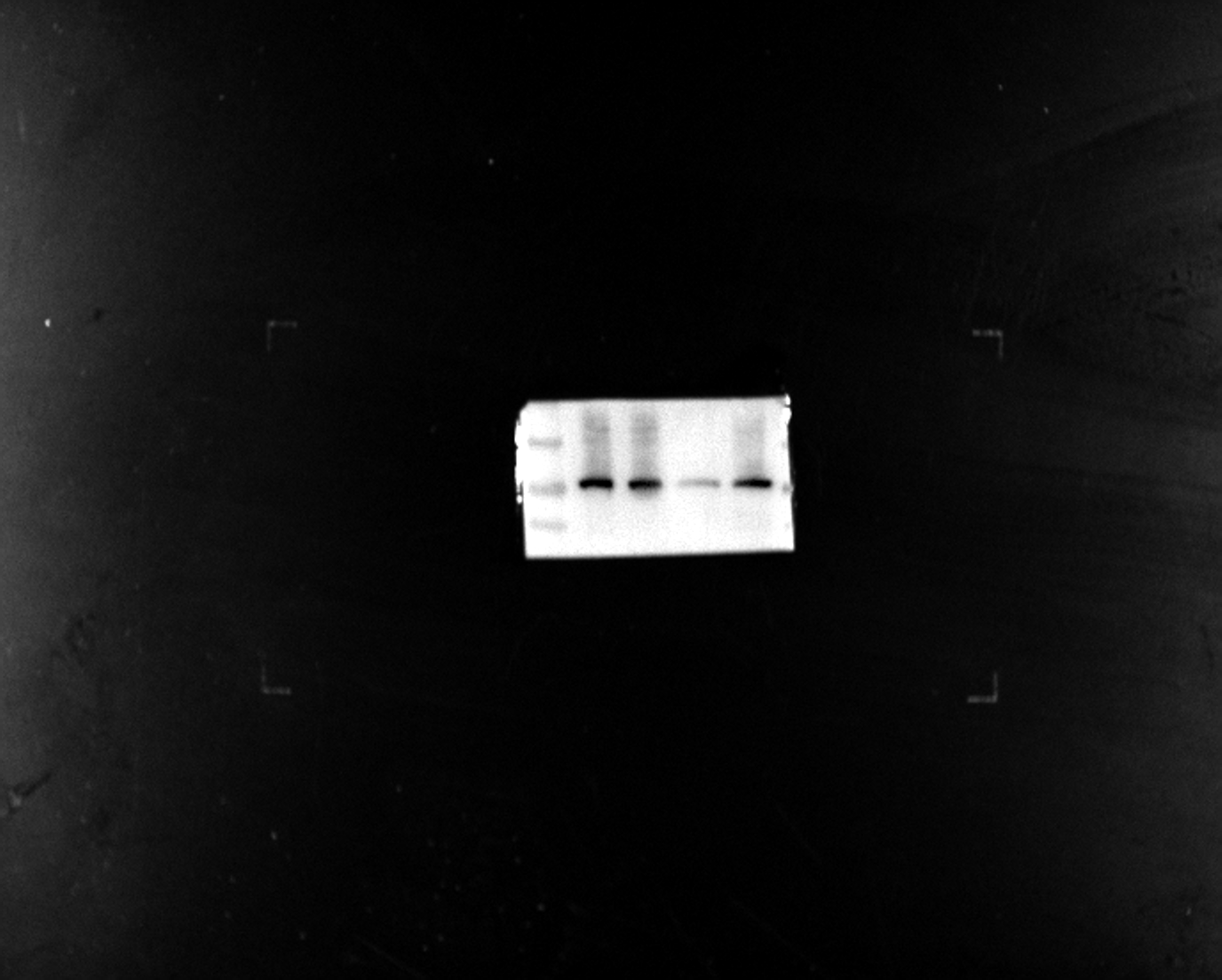

Supplement: Supplementary file 8 — Source data Fig. 3 [file 44318_2024_359_MOESM8_ESM.zip › Figure 3/Fig 3C and 3D/Fig 3C/5-p-S6-merge.Tif]

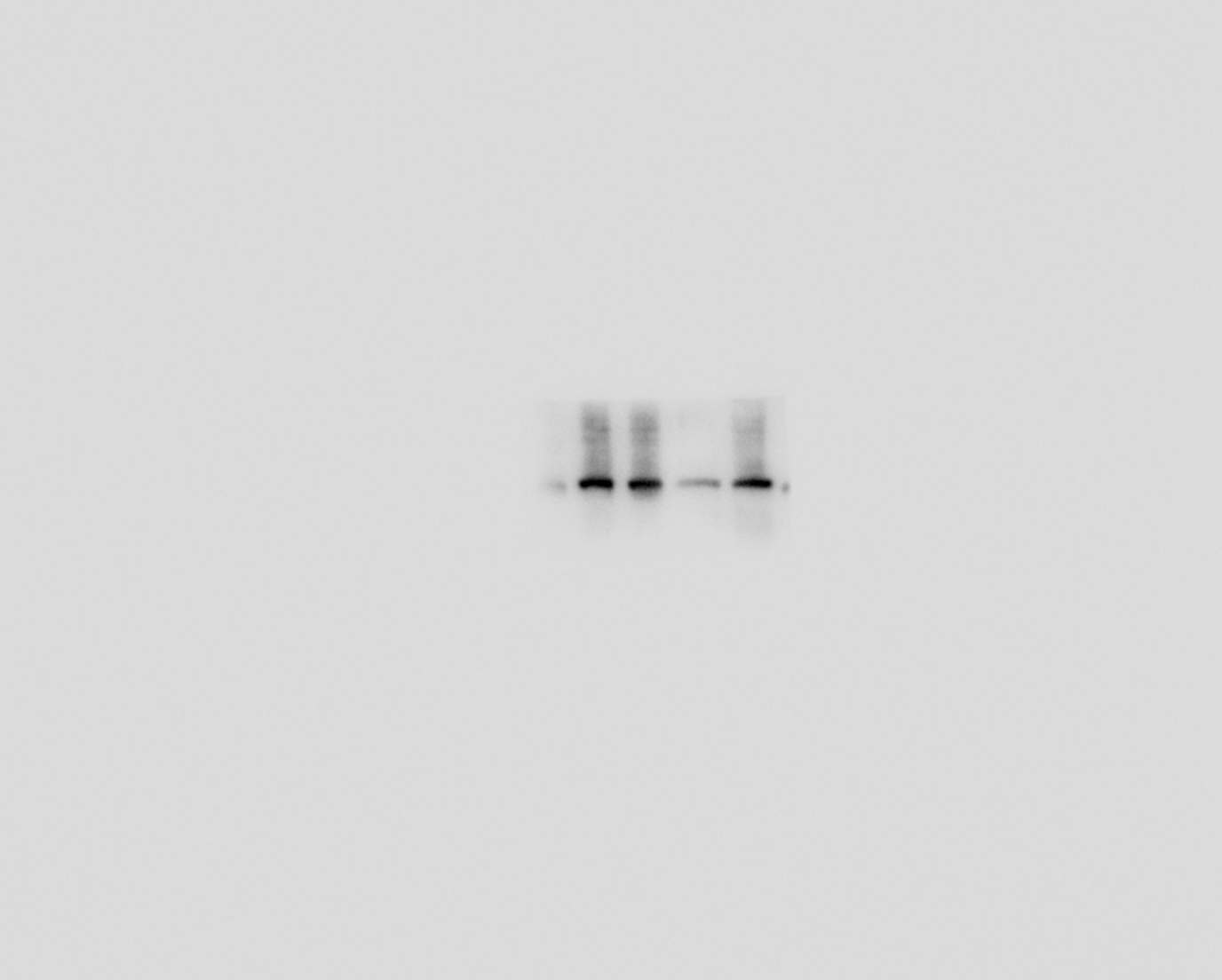

Supplement: Supplementary file 8 — Source data Fig. 3 [file 44318_2024_359_MOESM8_ESM.zip › Figure 3/Fig 3C and 3D/Fig 3C/5-p-S6.Tif]

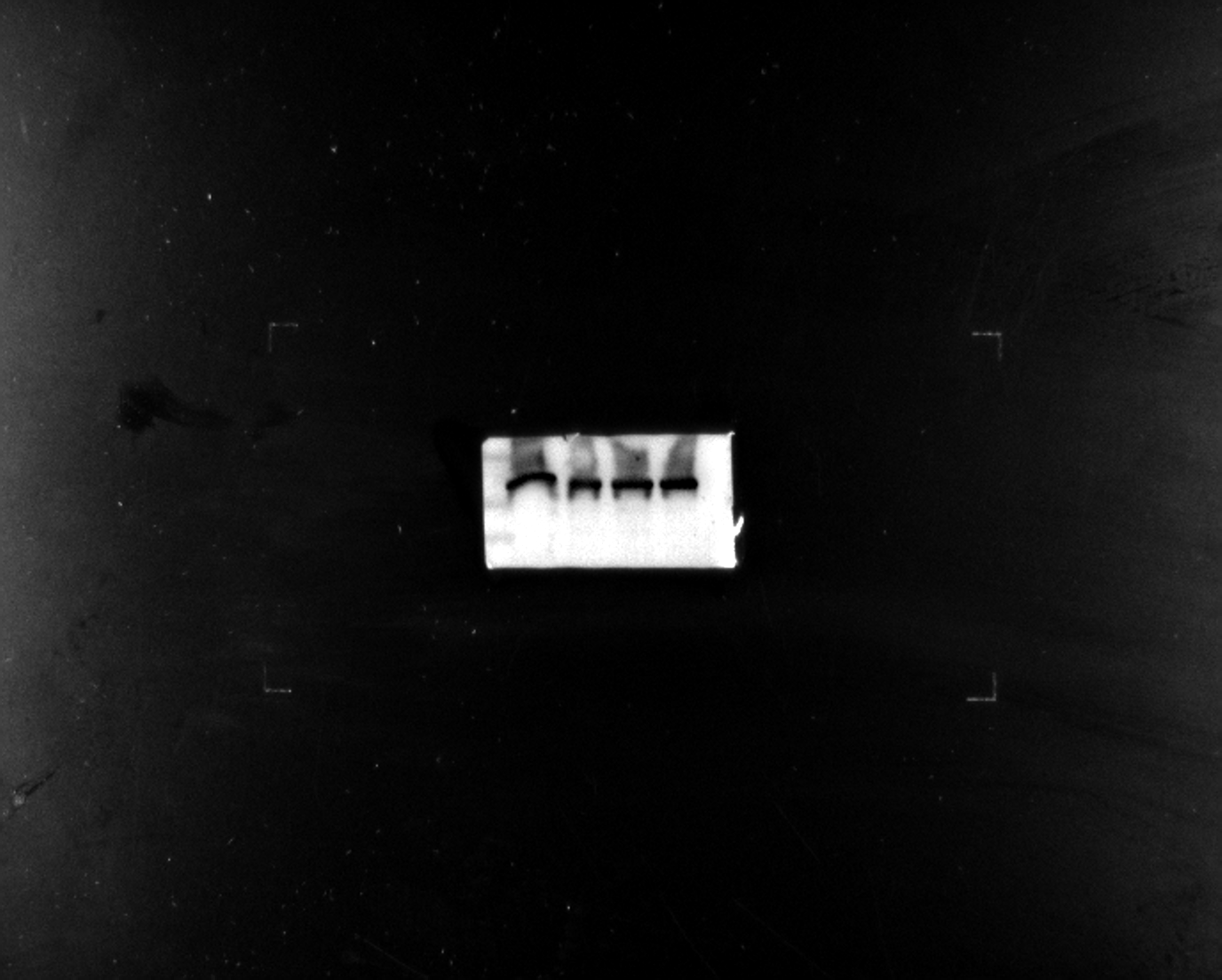

Supplement: Supplementary file 8 — Source data Fig. 3 [file 44318_2024_359_MOESM8_ESM.zip › Figure 3/Fig 3C and 3D/Fig 3C/6-S6-merge.Tif]

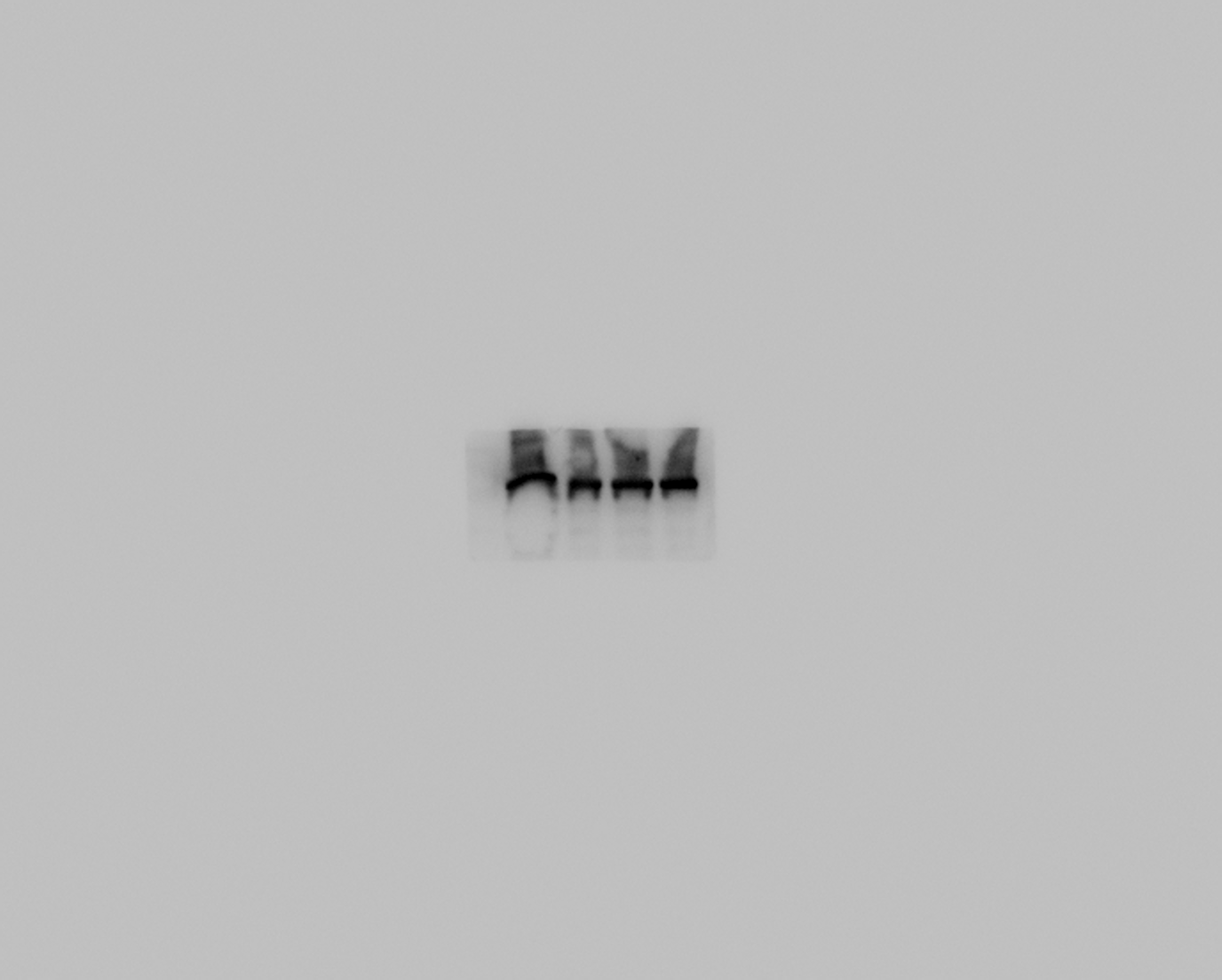

Supplement: Supplementary file 8 — Source data Fig. 3 [file 44318_2024_359_MOESM8_ESM.zip › Figure 3/Fig 3C and 3D/Fig 3C/6-S6.Tif]

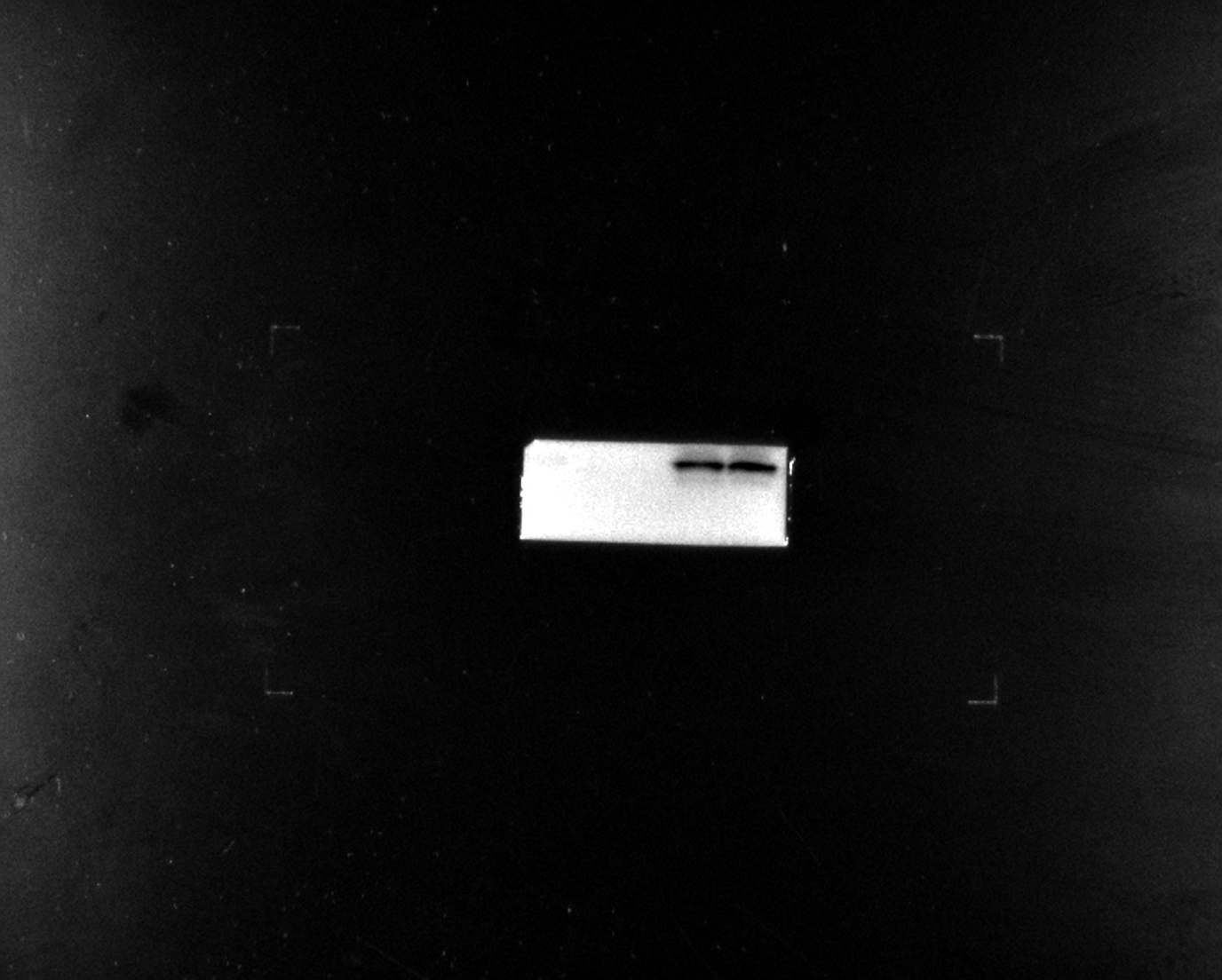

Supplement: Supplementary file 8 — Source data Fig. 3 [file 44318_2024_359_MOESM8_ESM.zip › Figure 3/Fig 3C and 3D/Fig 3C/7-Flag-merge.Tif]

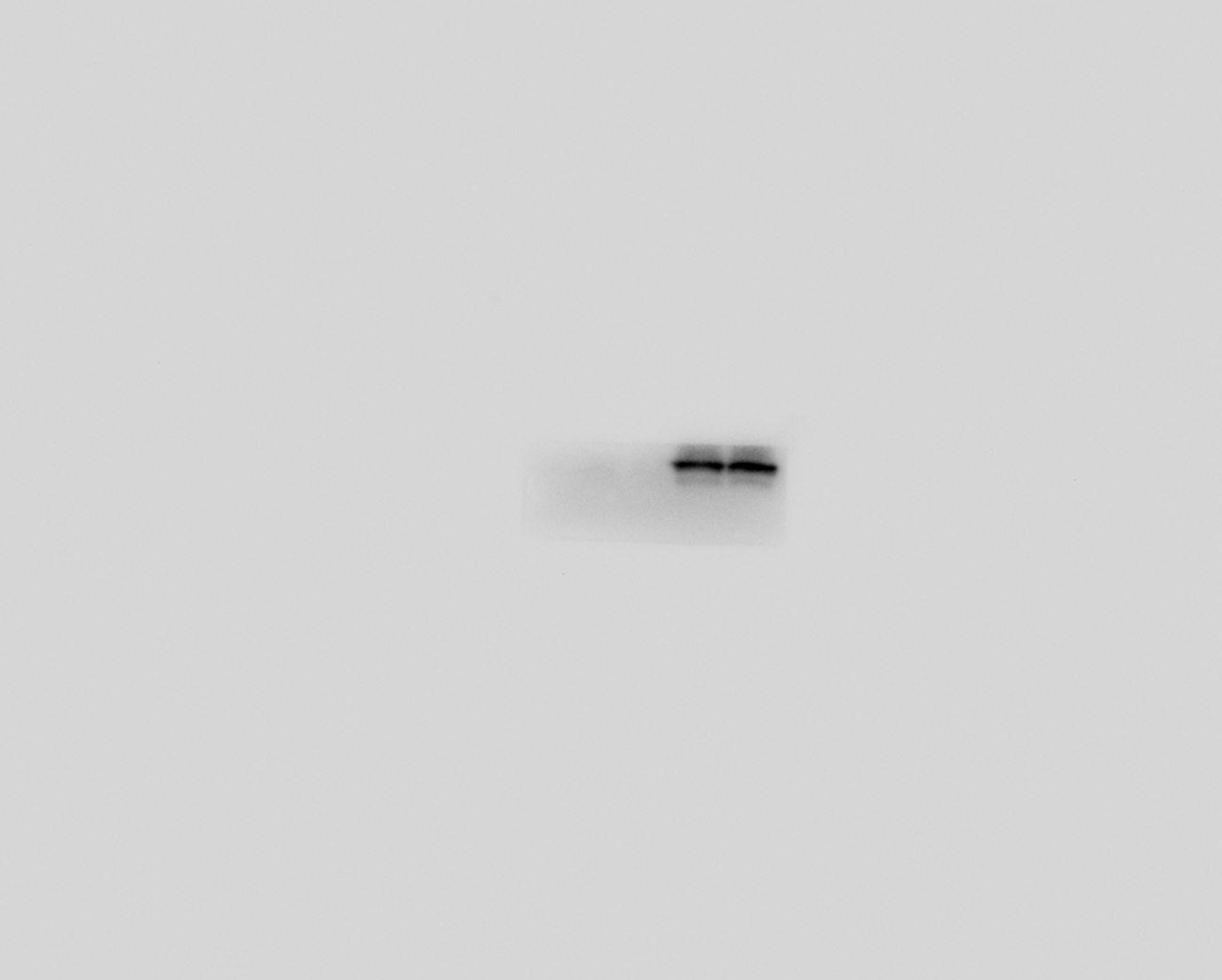

Supplement: Supplementary file 8 — Source data Fig. 3 [file 44318_2024_359_MOESM8_ESM.zip › Figure 3/Fig 3C and 3D/Fig 3C/7-Flag.Tif]

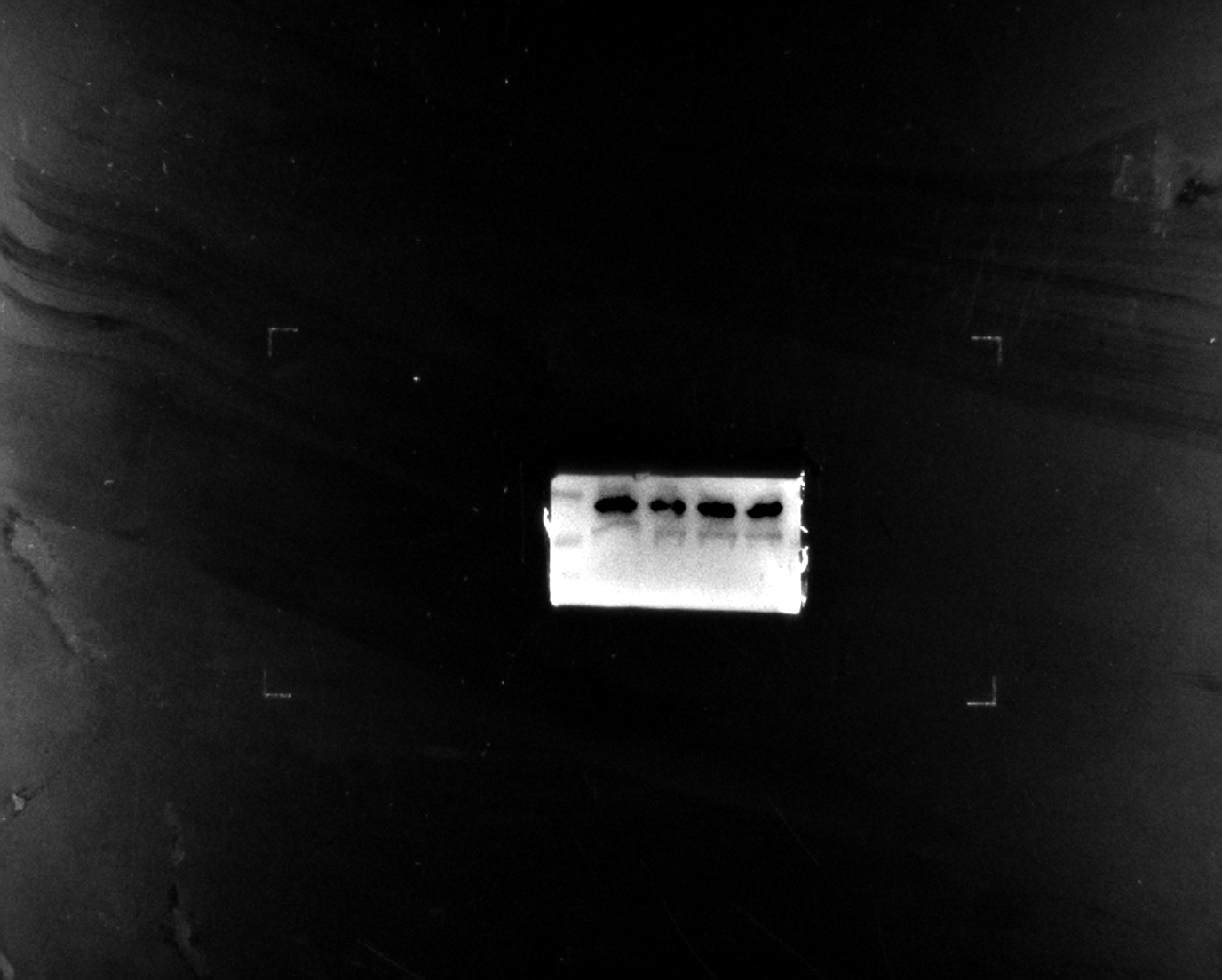

Supplement: Supplementary file 8 — Source data Fig. 3 [file 44318_2024_359_MOESM8_ESM.zip › Figure 3/Fig 3C and 3D/Fig 3C/8-GAPDH-merge.Tif]

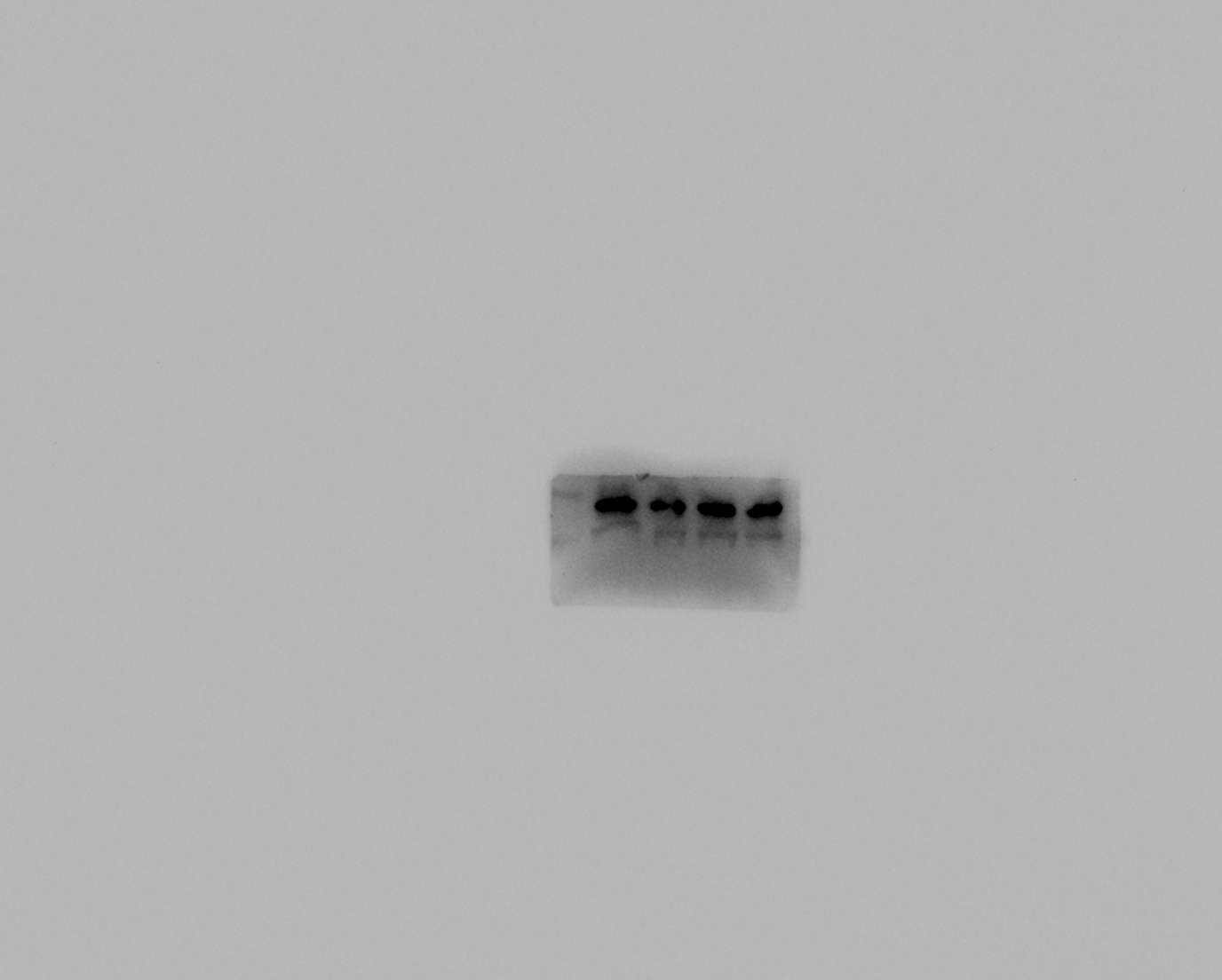

Supplement: Supplementary file 8 — Source data Fig. 3 [file 44318_2024_359_MOESM8_ESM.zip › Figure 3/Fig 3C and 3D/Fig 3C/8-GAPDH.Tif]

Fig. 3C

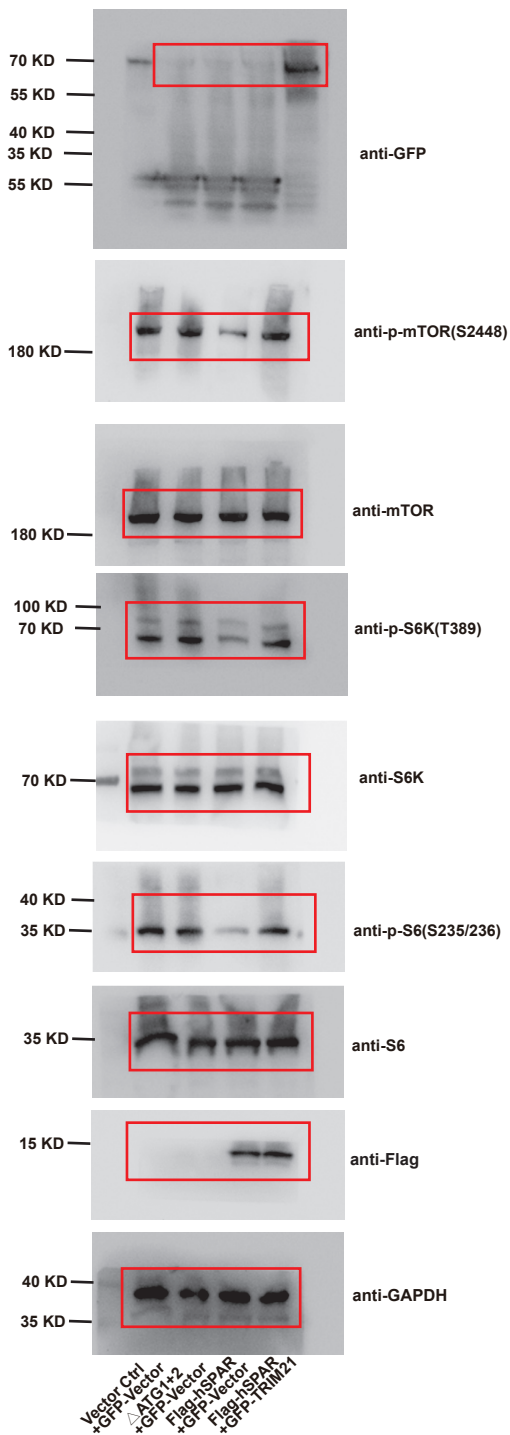

Supplement: Supplementary file 8 — Source data Fig. 3 [file 44318_2024_359_MOESM8_ESM.zip › Figure 3/Fig 3C and 3D/Fig 3C/Fig 3C.pdf]

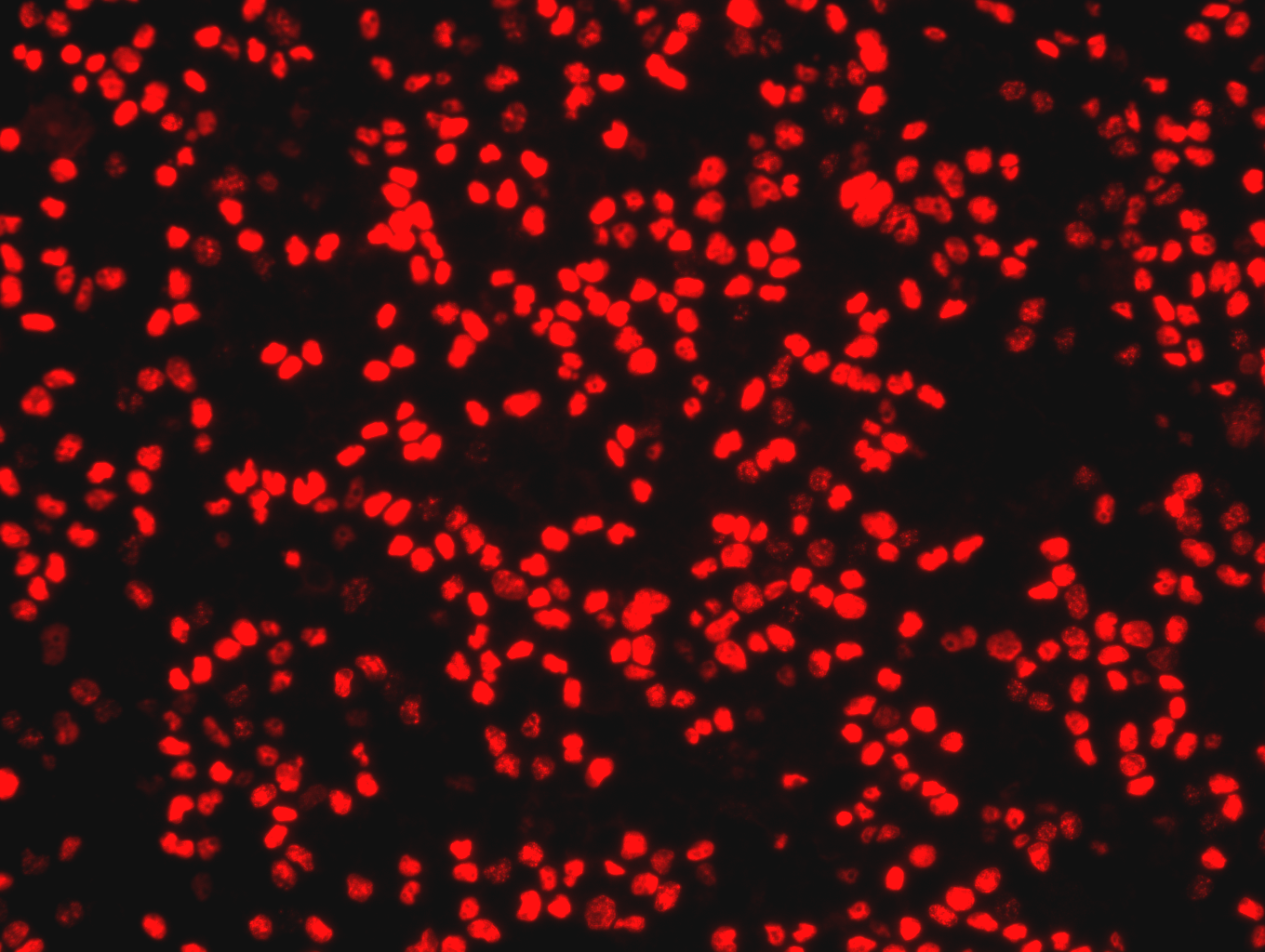

Supplement: Supplementary file 8 — Source data Fig. 3 [file 44318_2024_359_MOESM8_ESM.zip › Figure 3/Fig 3E and 3F/Fig 3E/ATG/1-edu in manu.tif]

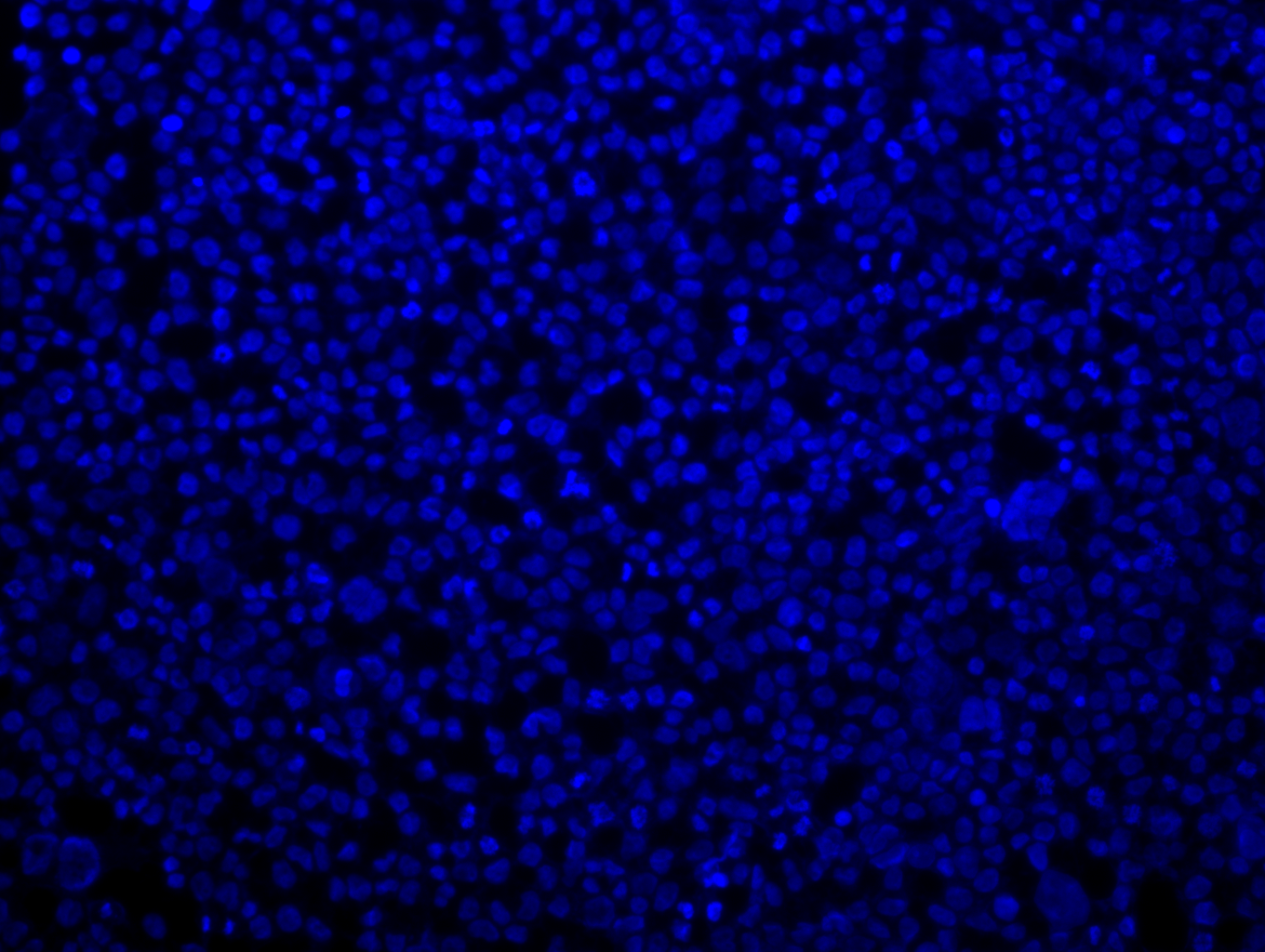

Supplement: Supplementary file 8 — Source data Fig. 3 [file 44318_2024_359_MOESM8_ESM.zip › Figure 3/Fig 3E and 3F/Fig 3E/ATG/1-hoechst in manu.tif]

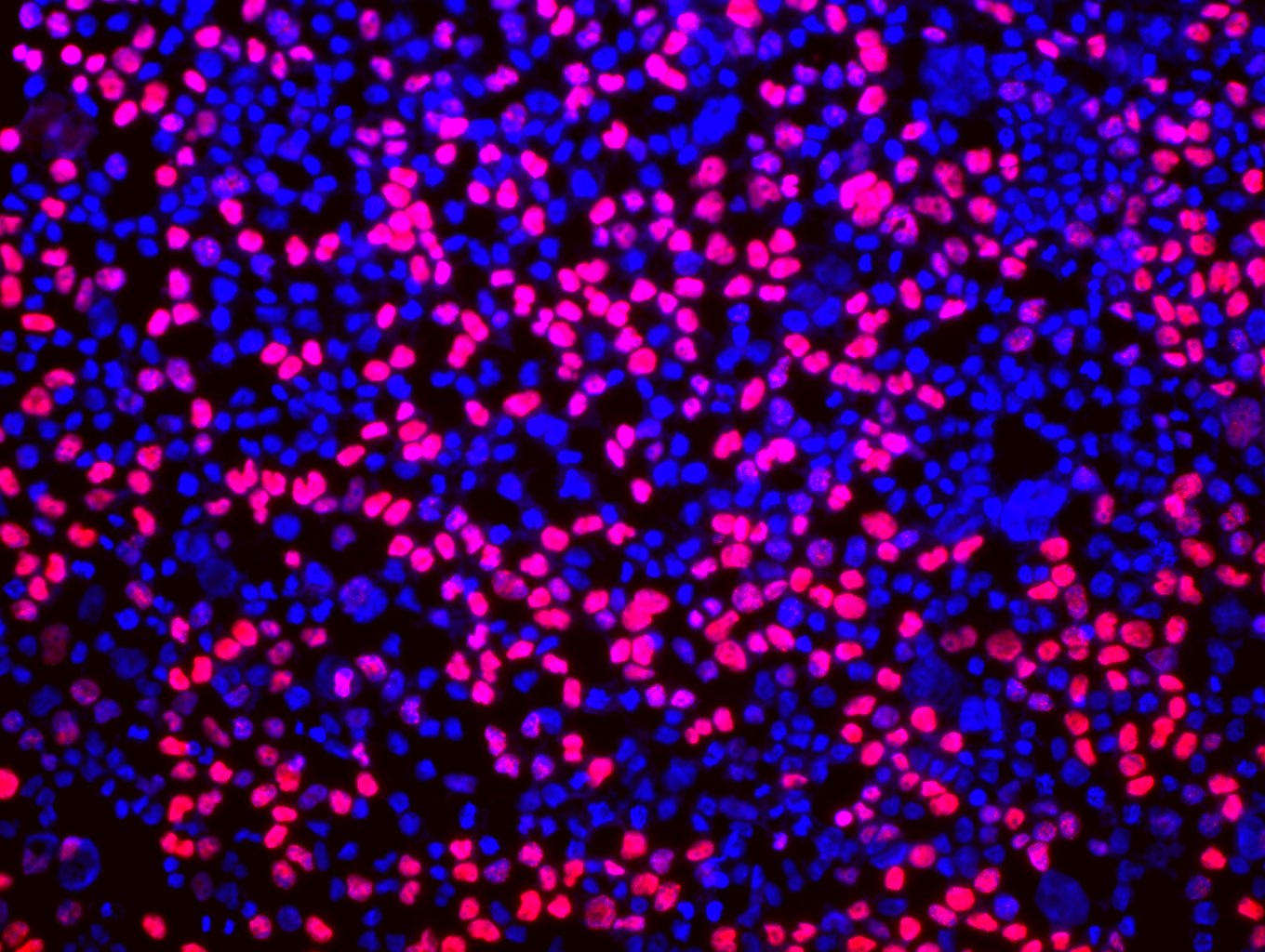

Supplement: Supplementary file 8 — Source data Fig. 3 [file 44318_2024_359_MOESM8_ESM.zip › Figure 3/Fig 3E and 3F/Fig 3E/ATG/1-merge in manu.jpg]

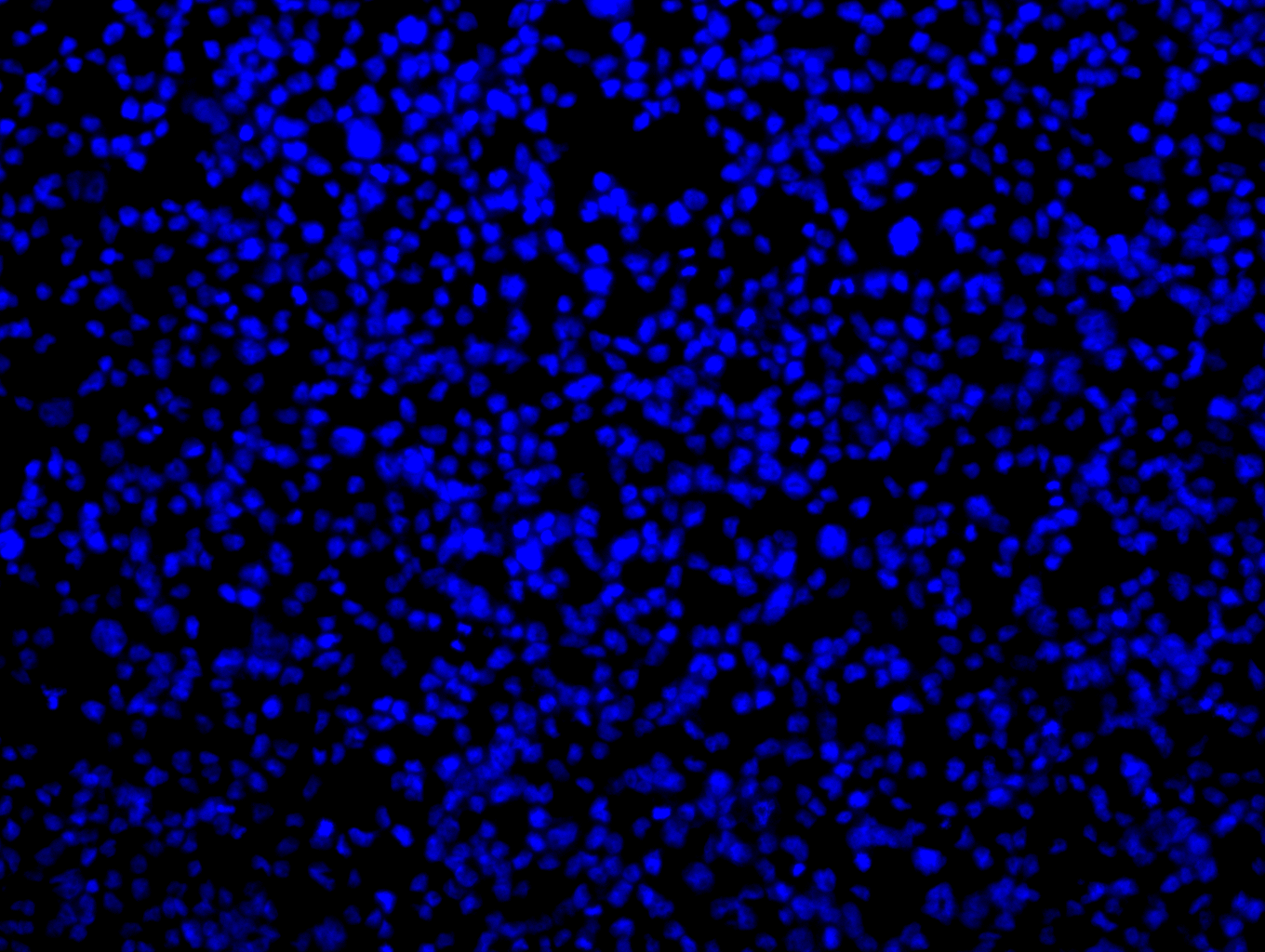

Supplement: Supplementary file 8 — Source data Fig. 3 [file 44318_2024_359_MOESM8_ESM.zip › Figure 3/Fig 3E and 3F/Fig 3E/Vector Ctrl/1-Hoechst in manu.tif]

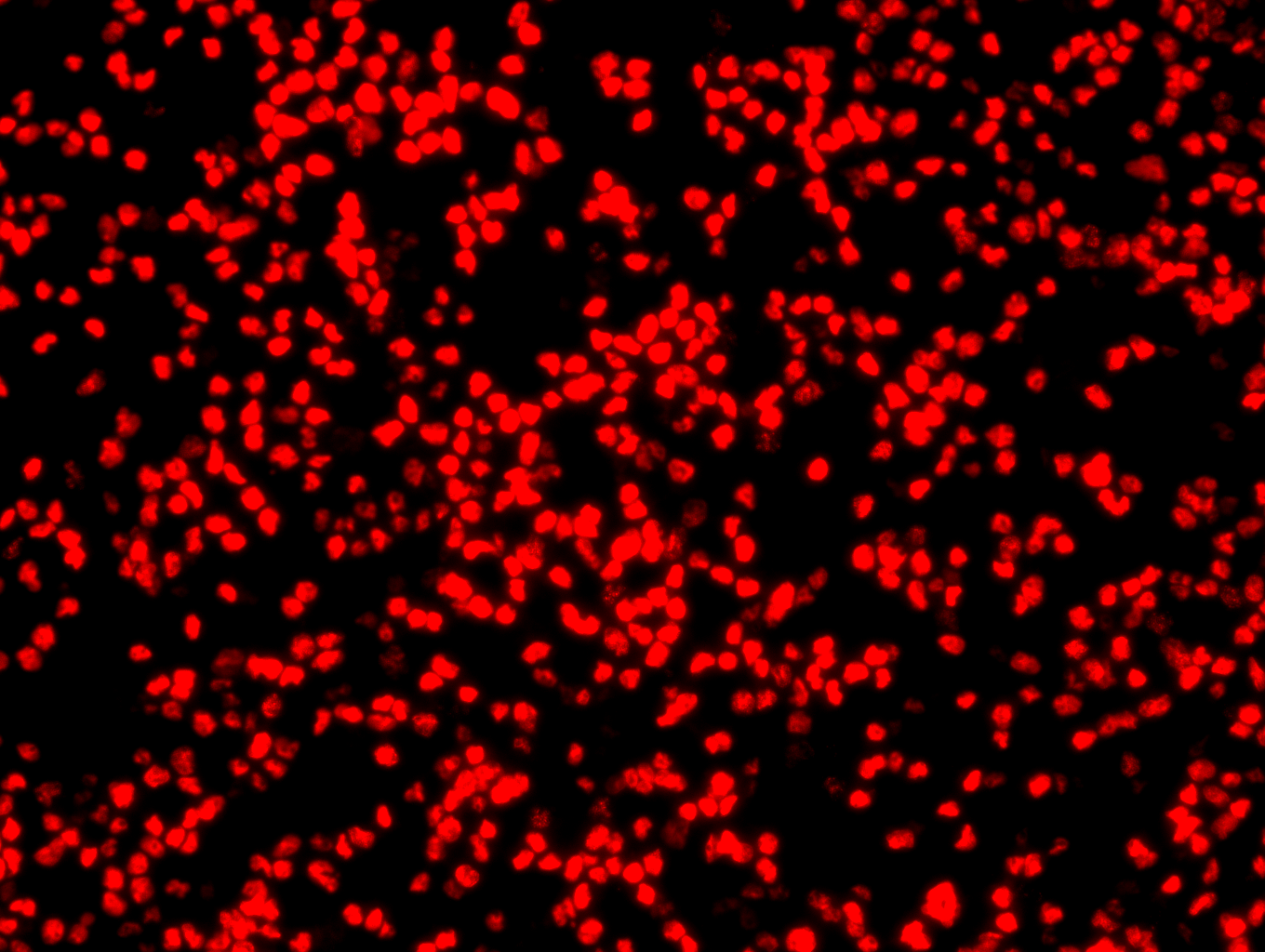

Supplement: Supplementary file 8 — Source data Fig. 3 [file 44318_2024_359_MOESM8_ESM.zip › Figure 3/Fig 3E and 3F/Fig 3E/Vector Ctrl/1-edu in manu.tif]

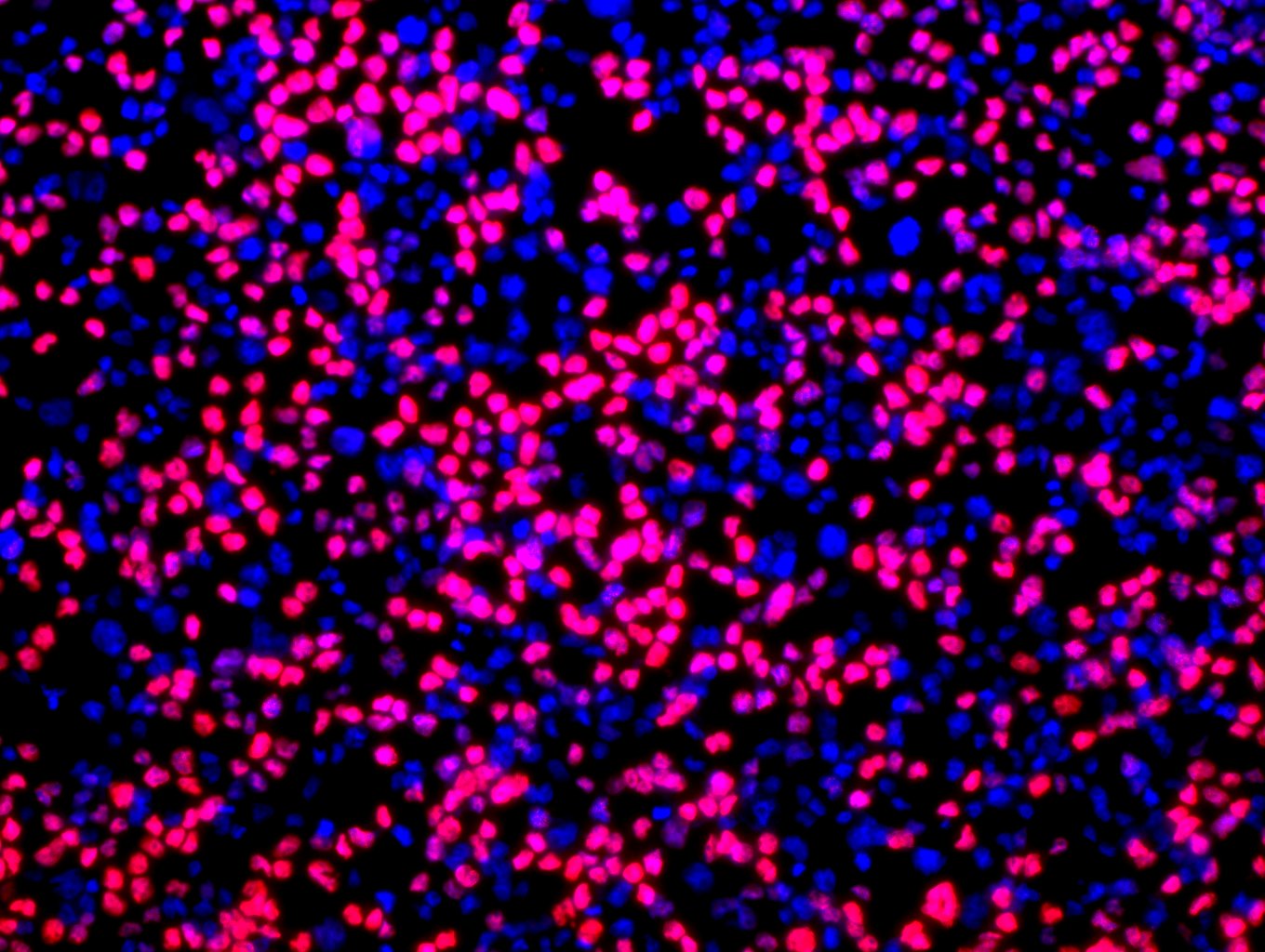

Supplement: Supplementary file 8 — Source data Fig. 3 [file 44318_2024_359_MOESM8_ESM.zip › Figure 3/Fig 3E and 3F/Fig 3E/Vector Ctrl/1-merge in manu.jpg]

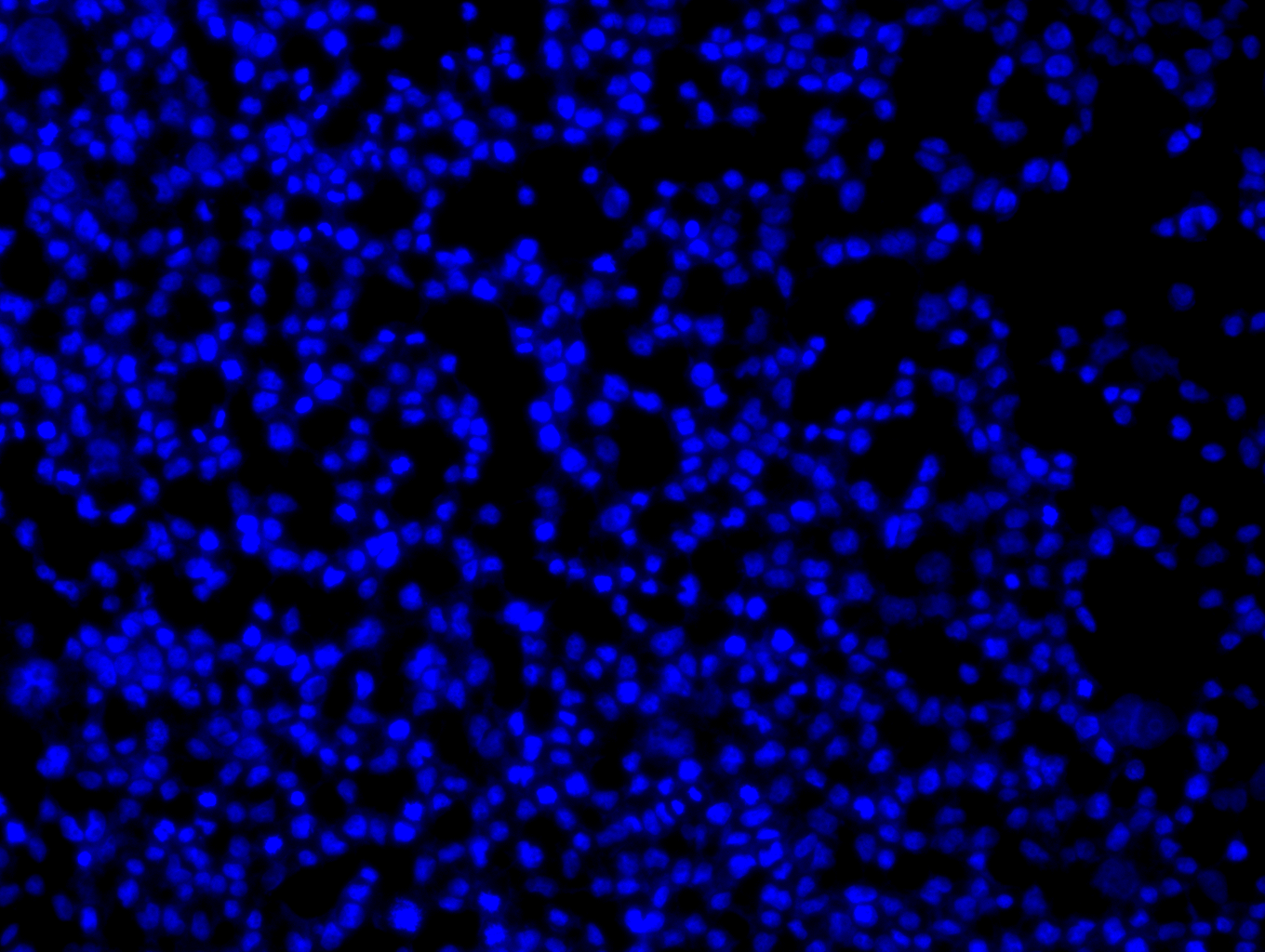

Supplement: Supplementary file 8 — Source data Fig. 3 [file 44318_2024_359_MOESM8_ESM.zip › Figure 3/Fig 3E and 3F/Fig 3E/ovhSPAR+ov GFP-Vector/1-Hoechst in manu.tif]

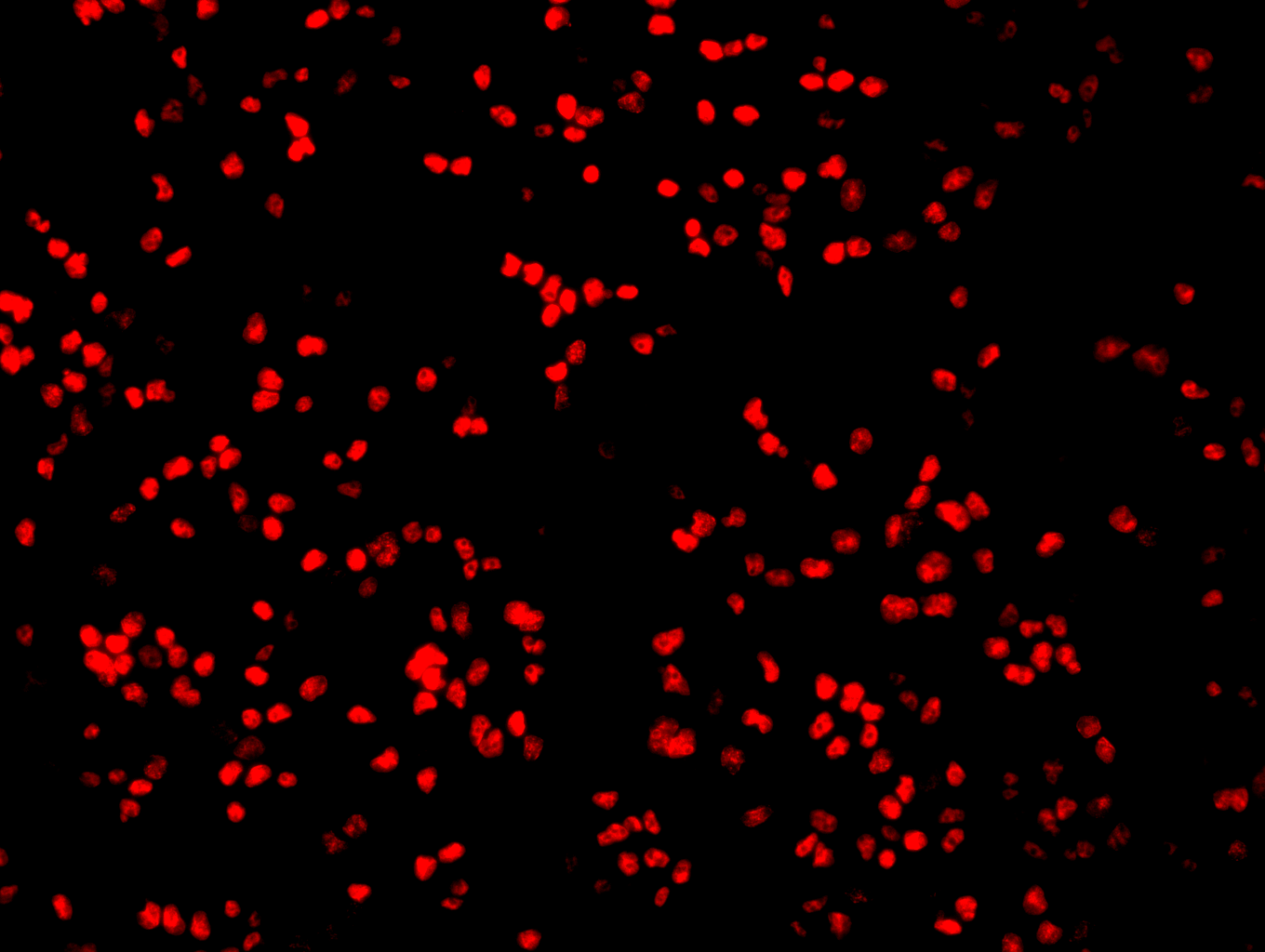

Supplement: Supplementary file 8 — Source data Fig. 3 [file 44318_2024_359_MOESM8_ESM.zip › Figure 3/Fig 3E and 3F/Fig 3E/ovhSPAR+ov GFP-Vector/1-edu in manu.tif]

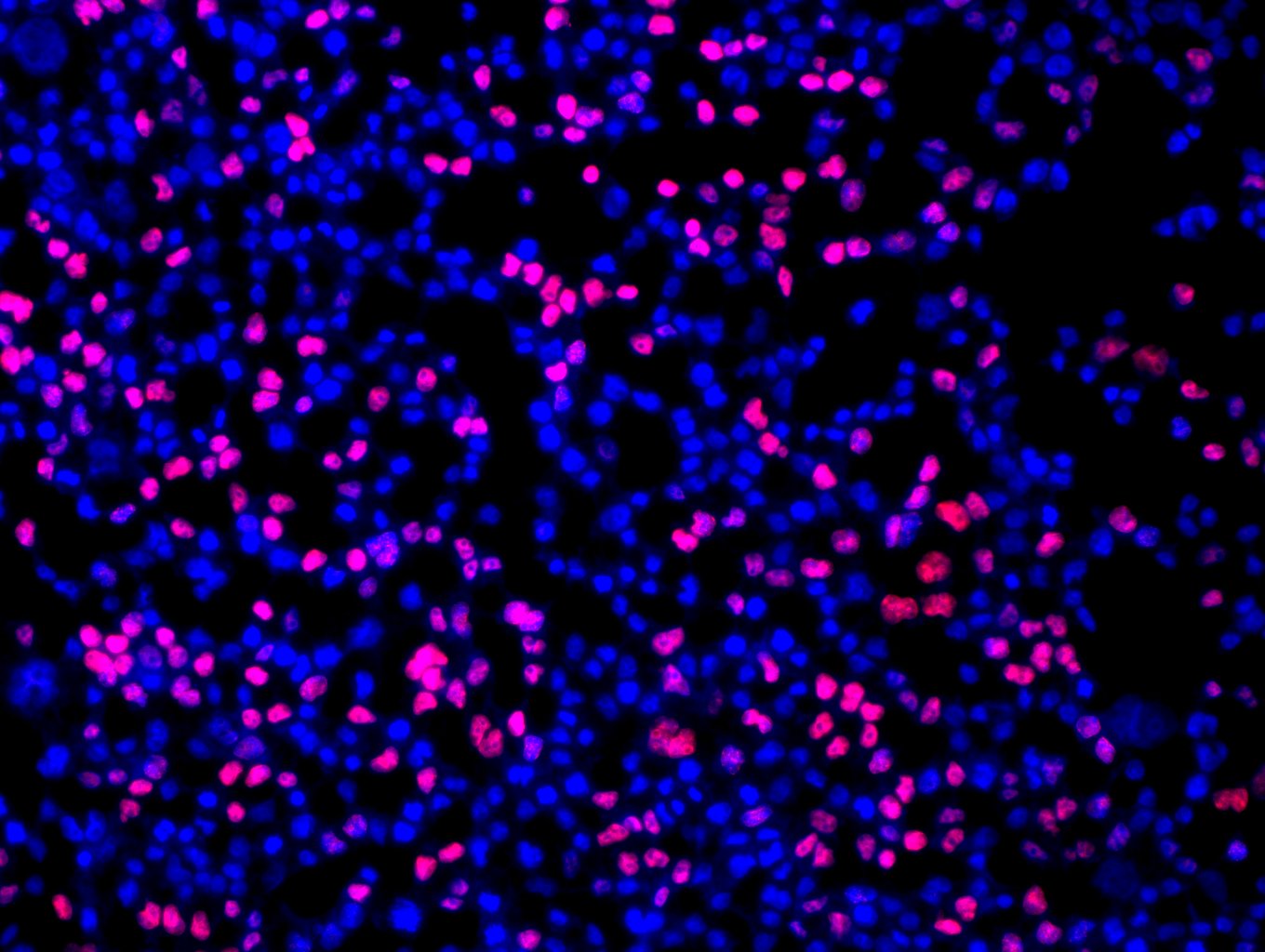

Supplement: Supplementary file 8 — Source data Fig. 3 [file 44318_2024_359_MOESM8_ESM.zip › Figure 3/Fig 3E and 3F/Fig 3E/ovhSPAR+ov GFP-Vector/1-merge in manu.jpg]

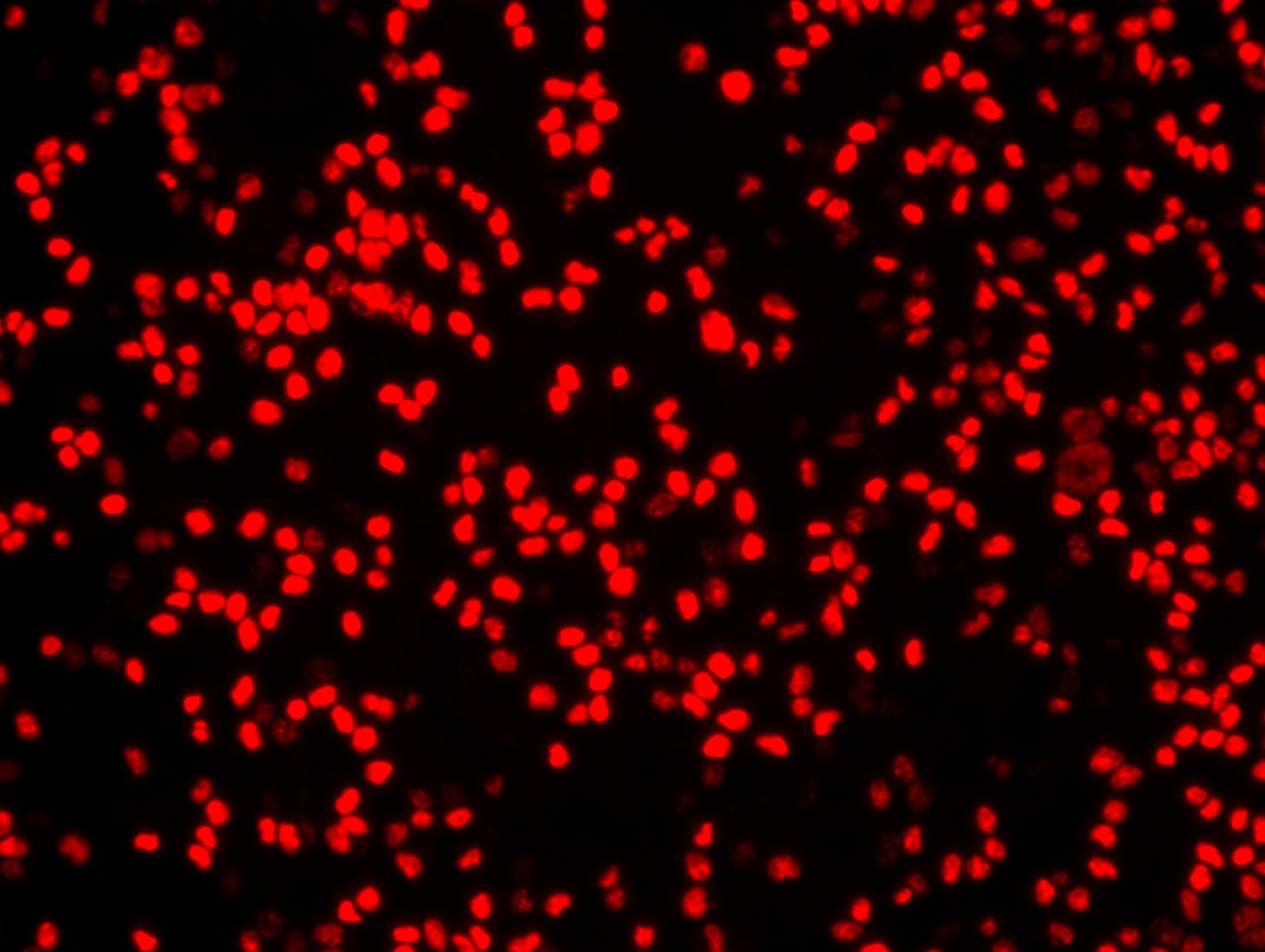

Supplement: Supplementary file 8 — Source data Fig. 3 [file 44318_2024_359_MOESM8_ESM.zip › Figure 3/Fig 3E and 3F/Fig 3E/ovhSPAR+ovGFP-TRIM21/1-edu in manu.jpg]

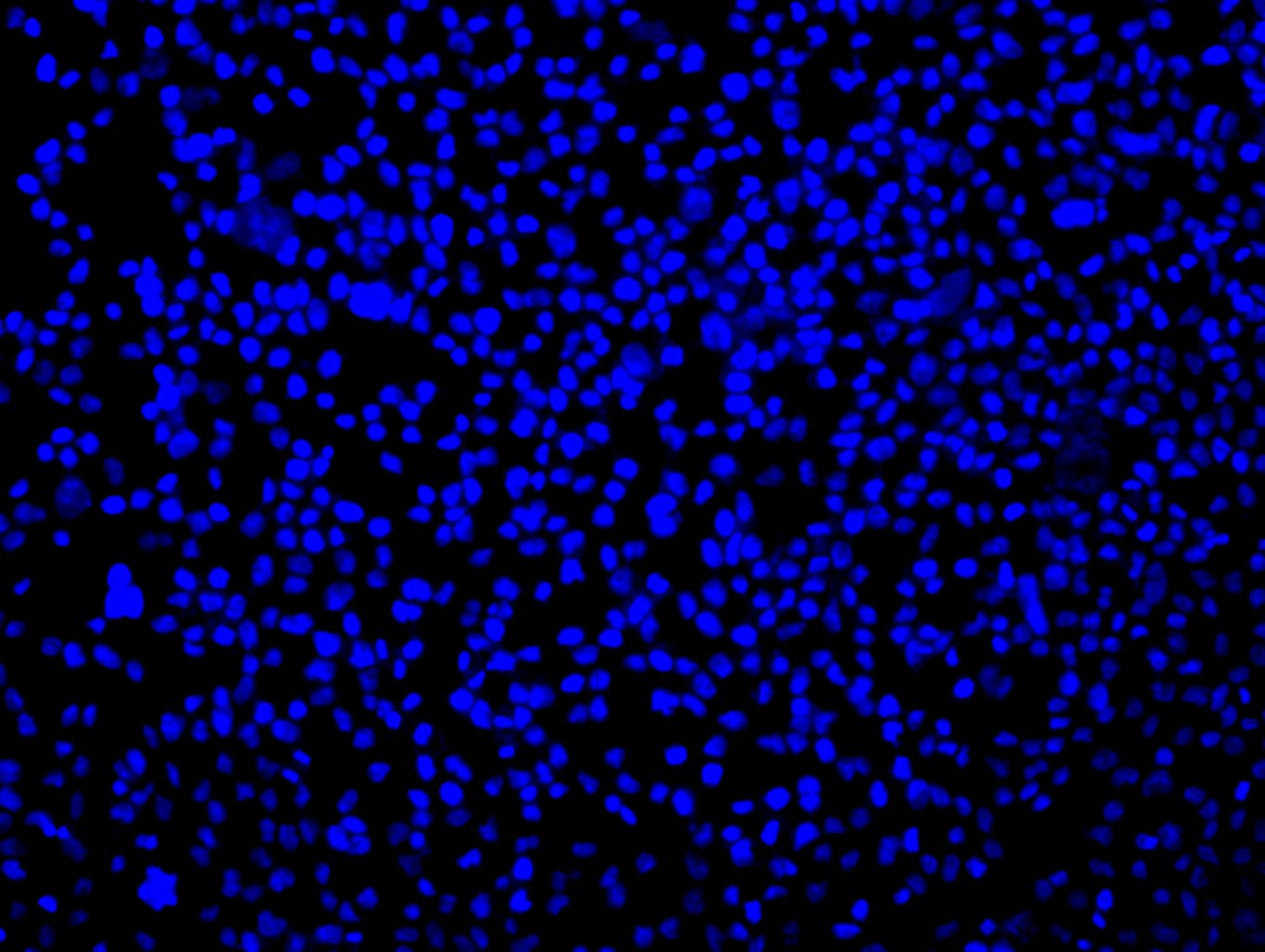

Supplement: Supplementary file 8 — Source data Fig. 3 [file 44318_2024_359_MOESM8_ESM.zip › Figure 3/Fig 3E and 3F/Fig 3E/ovhSPAR+ovGFP-TRIM21/1-hoechst in manu.jpg]

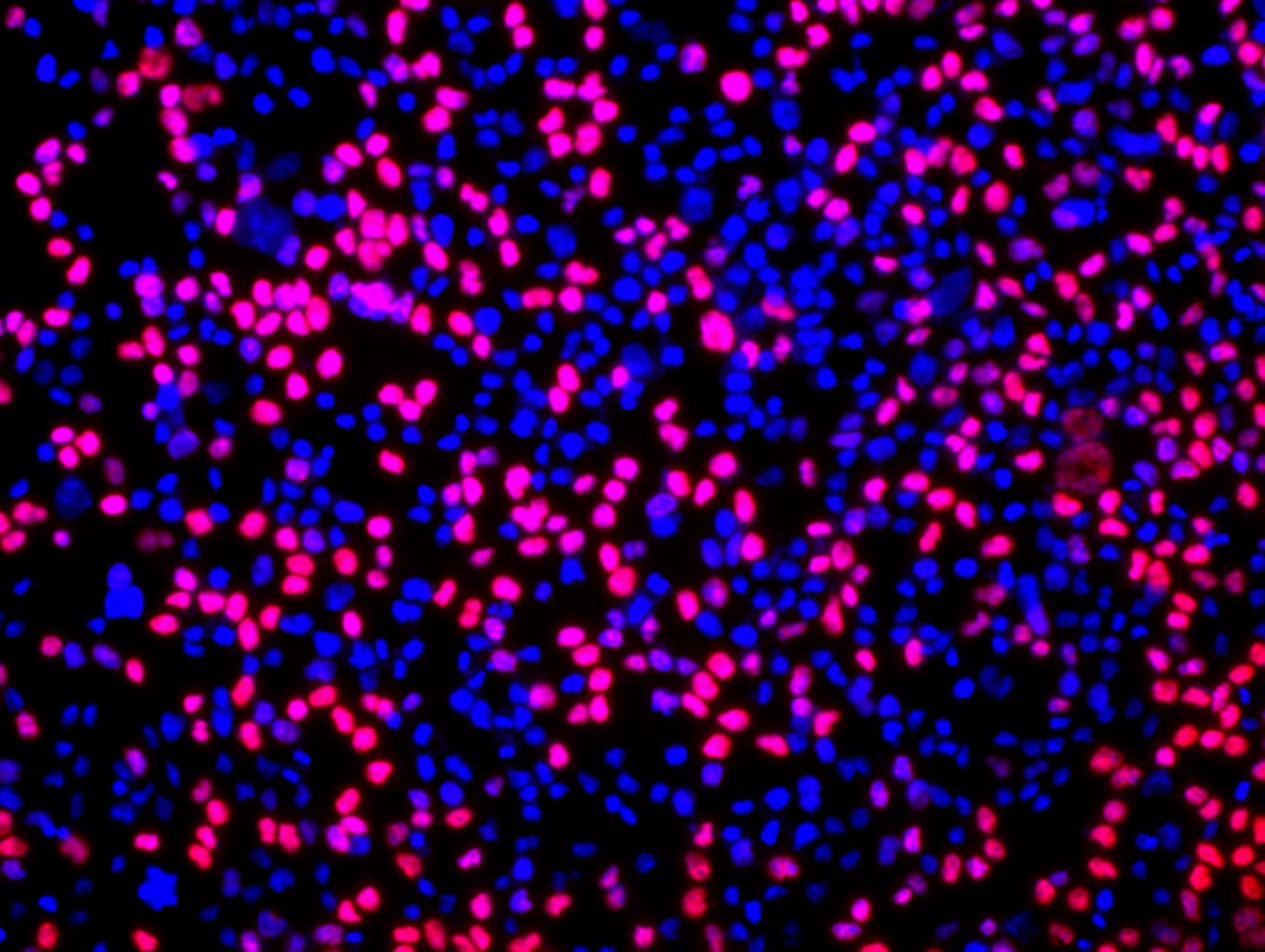

Supplement: Supplementary file 8 — Source data Fig. 3 [file 44318_2024_359_MOESM8_ESM.zip › Figure 3/Fig 3E and 3F/Fig 3E/ovhSPAR+ovGFP-TRIM21/1-merge in manu.jpg]

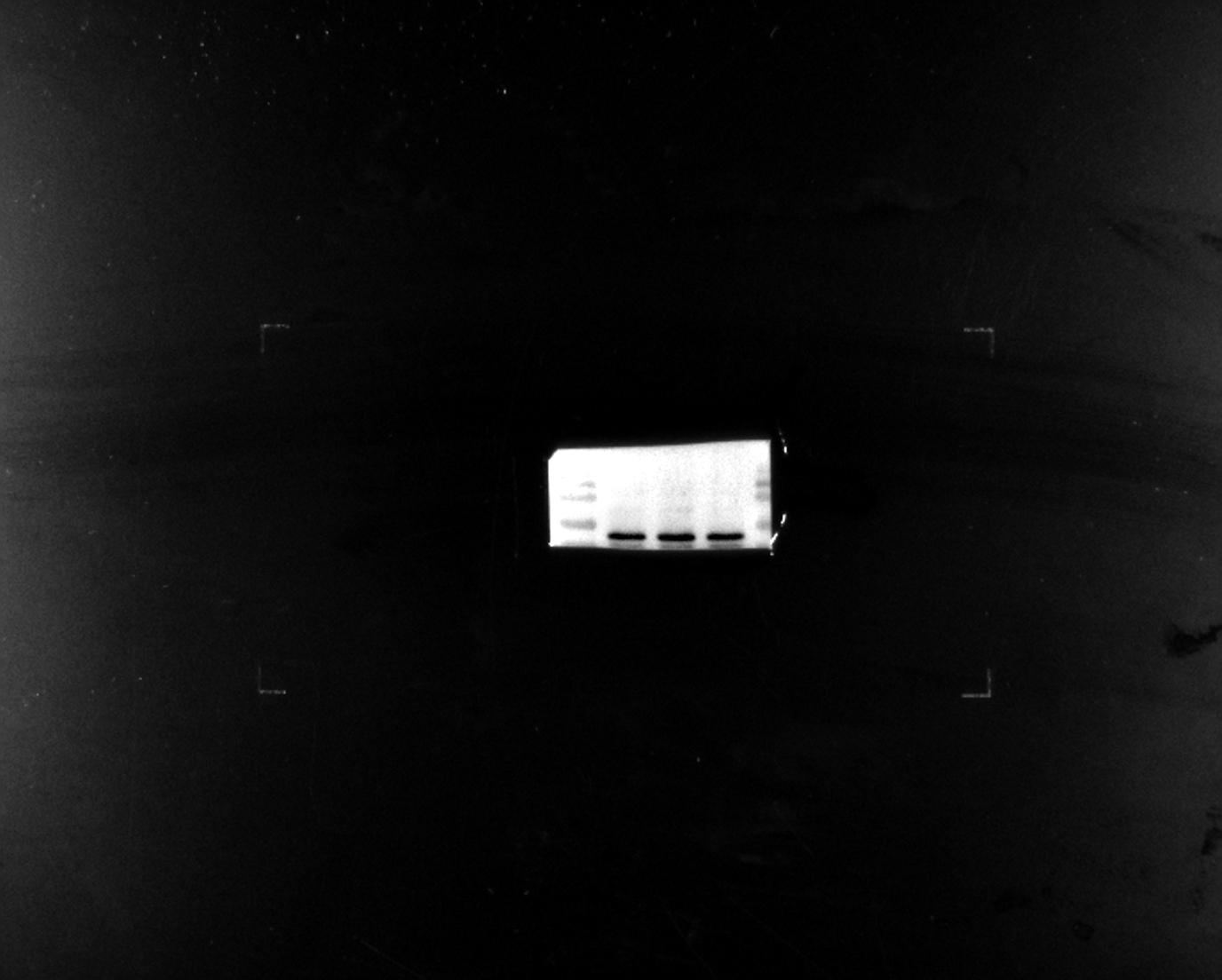

Supplement: Supplementary file 9 — Source data Fig. 4 [file 44318_2024_359_MOESM9_ESM.zip › Figure 4/Fig 4A/1-TRIM21-merge.Tif]

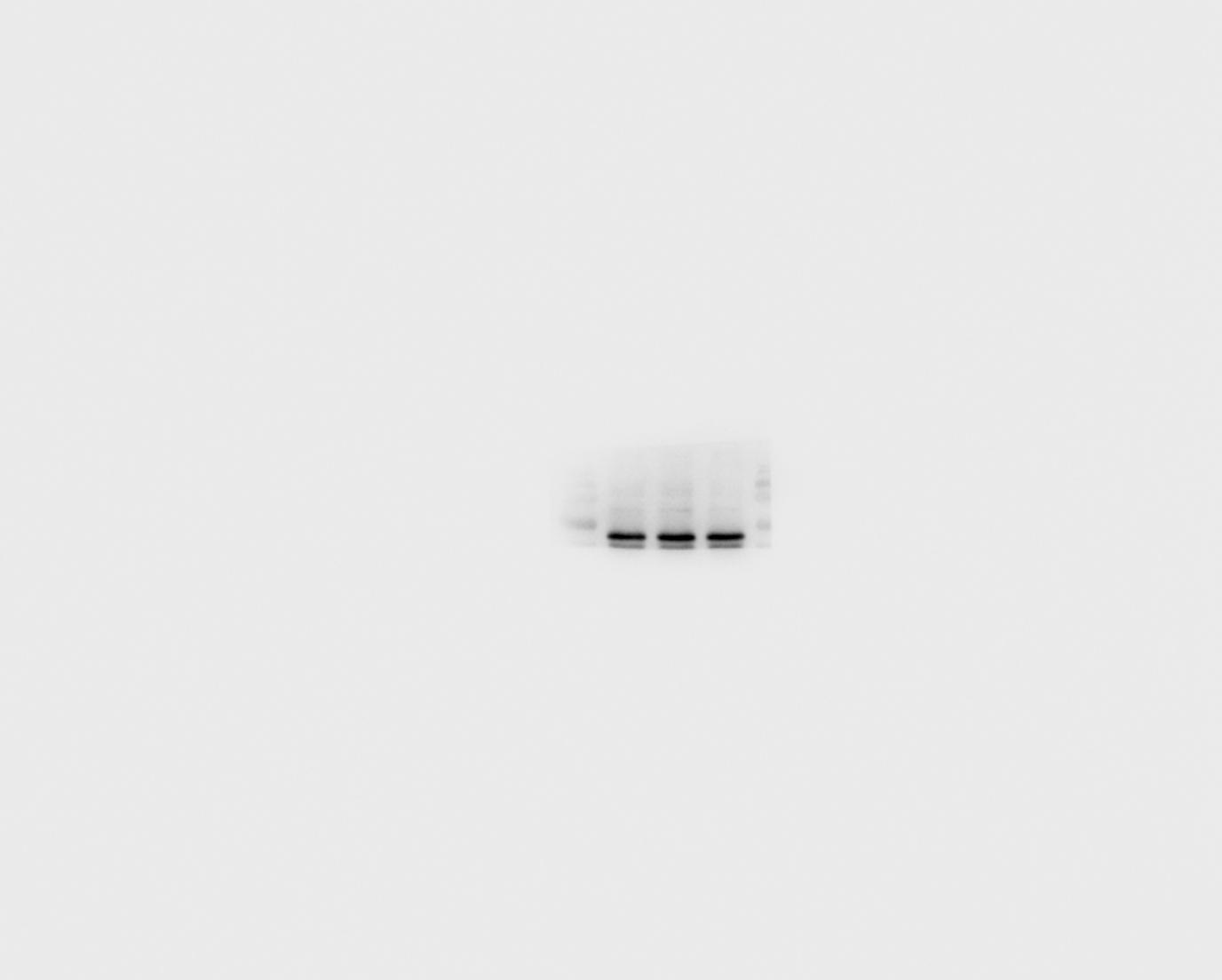

Supplement: Supplementary file 9 — Source data Fig. 4 [file 44318_2024_359_MOESM9_ESM.zip › Figure 4/Fig 4A/1-input-TRIM21.Tif]

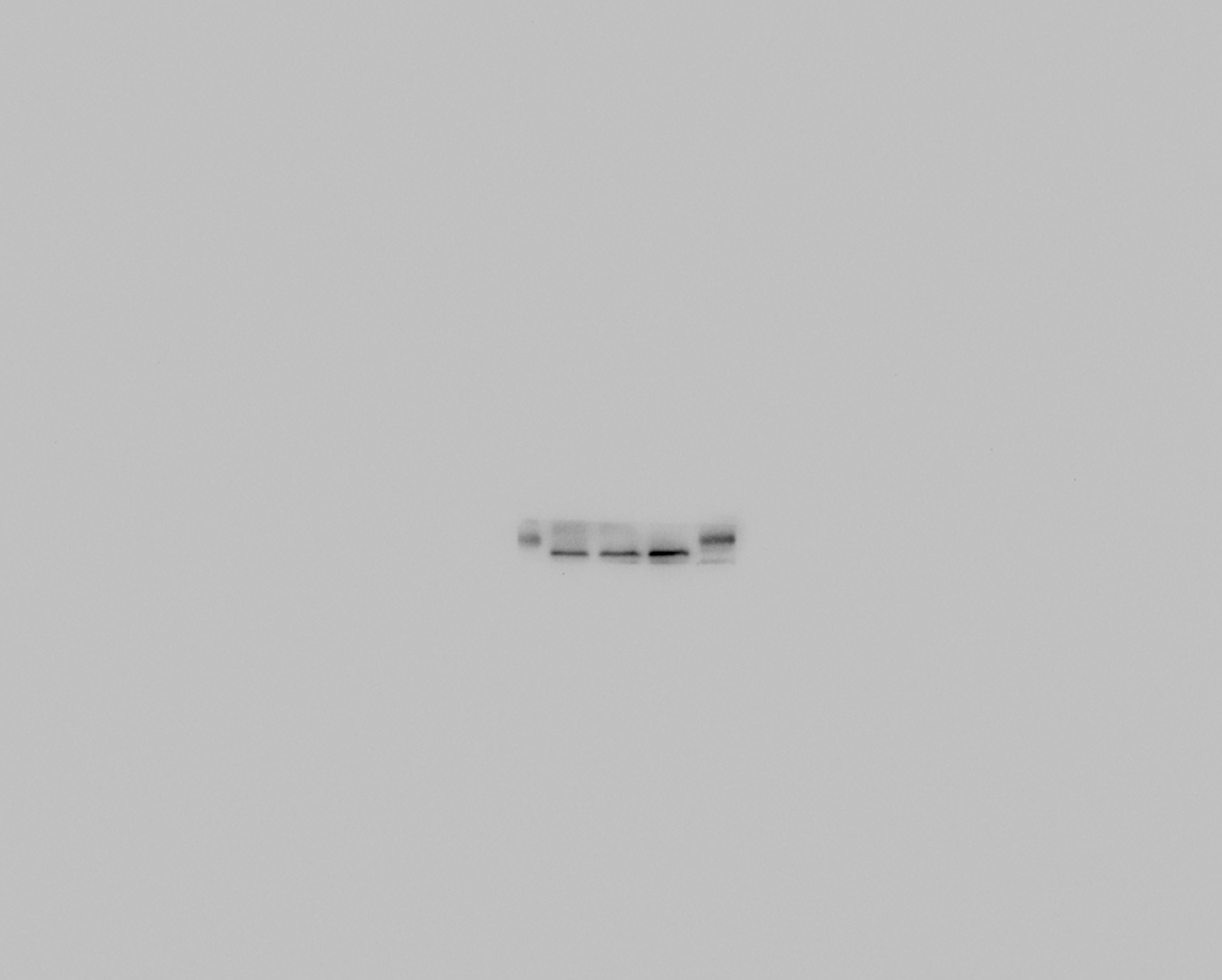

Supplement: Supplementary file 9 — Source data Fig. 4 [file 44318_2024_359_MOESM9_ESM.zip › Figure 4/Fig 4A/2-input-p27.Tif]

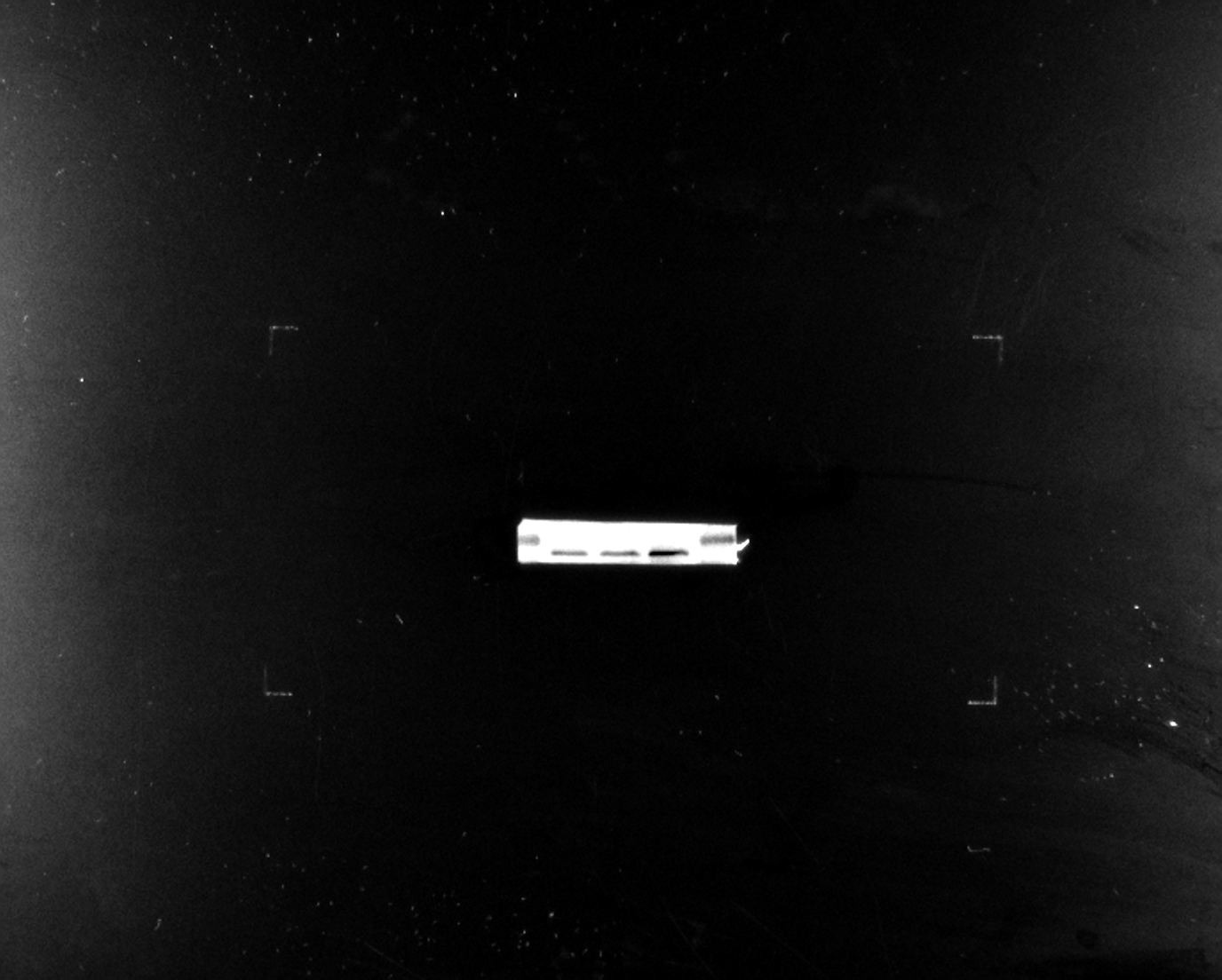

Supplement: Supplementary file 9 — Source data Fig. 4 [file 44318_2024_359_MOESM9_ESM.zip › Figure 4/Fig 4A/2-p27-merge.Tif]

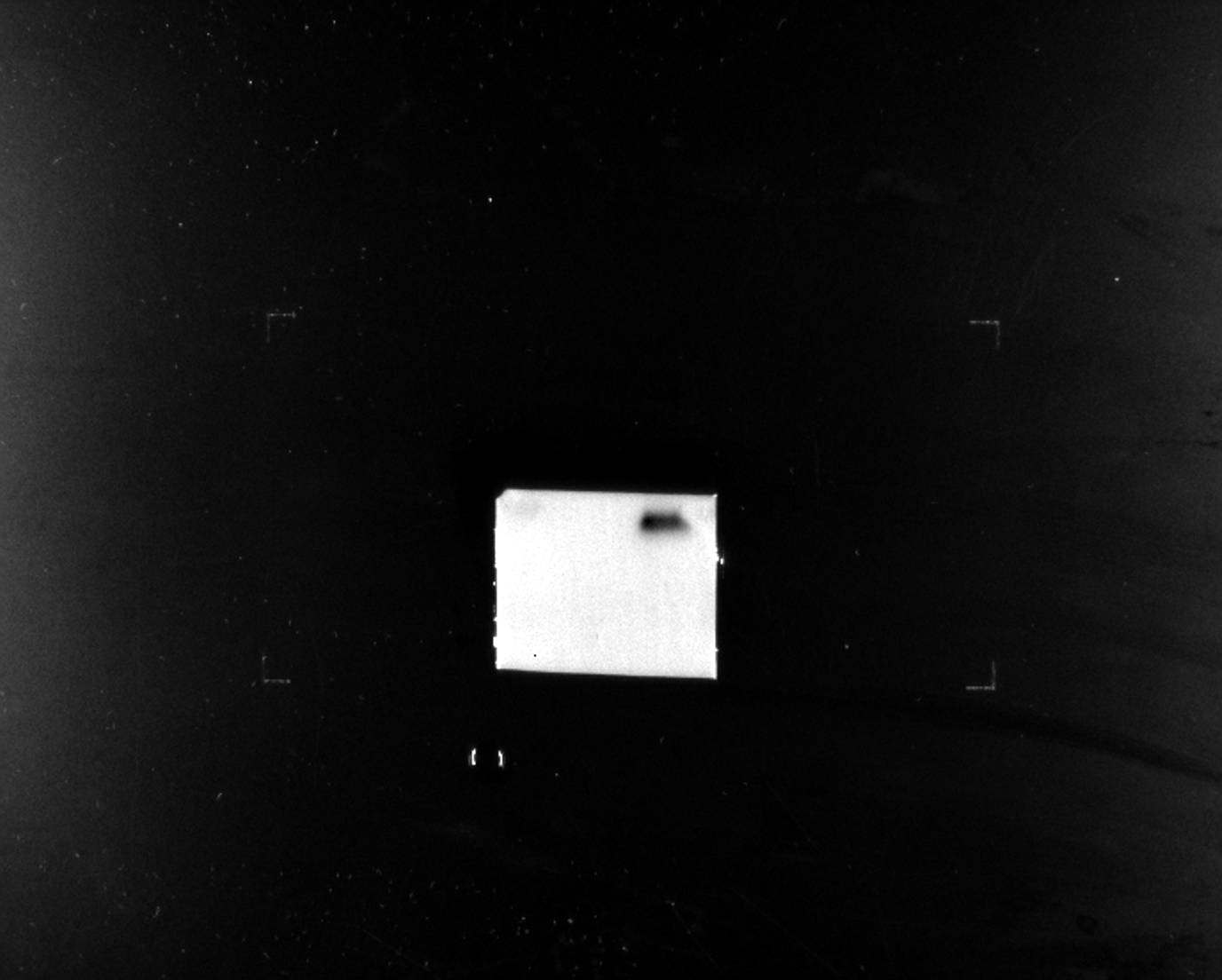

Supplement: Supplementary file 9 — Source data Fig. 4 [file 44318_2024_359_MOESM9_ESM.zip › Figure 4/Fig 4A/3-input-Flag-merge.Tif]

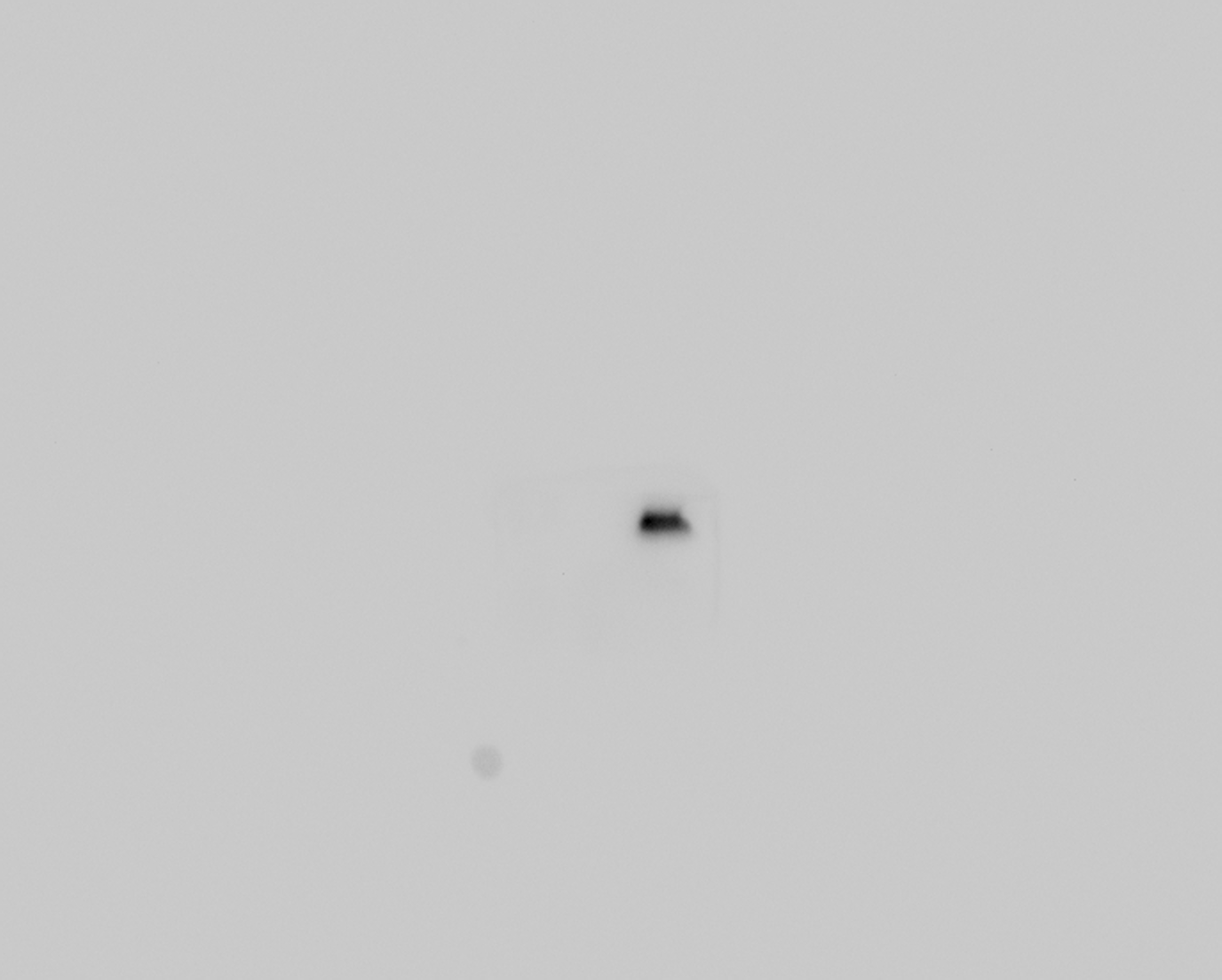

Supplement: Supplementary file 9 — Source data Fig. 4 [file 44318_2024_359_MOESM9_ESM.zip › Figure 4/Fig 4A/3-input-Flag.Tif]

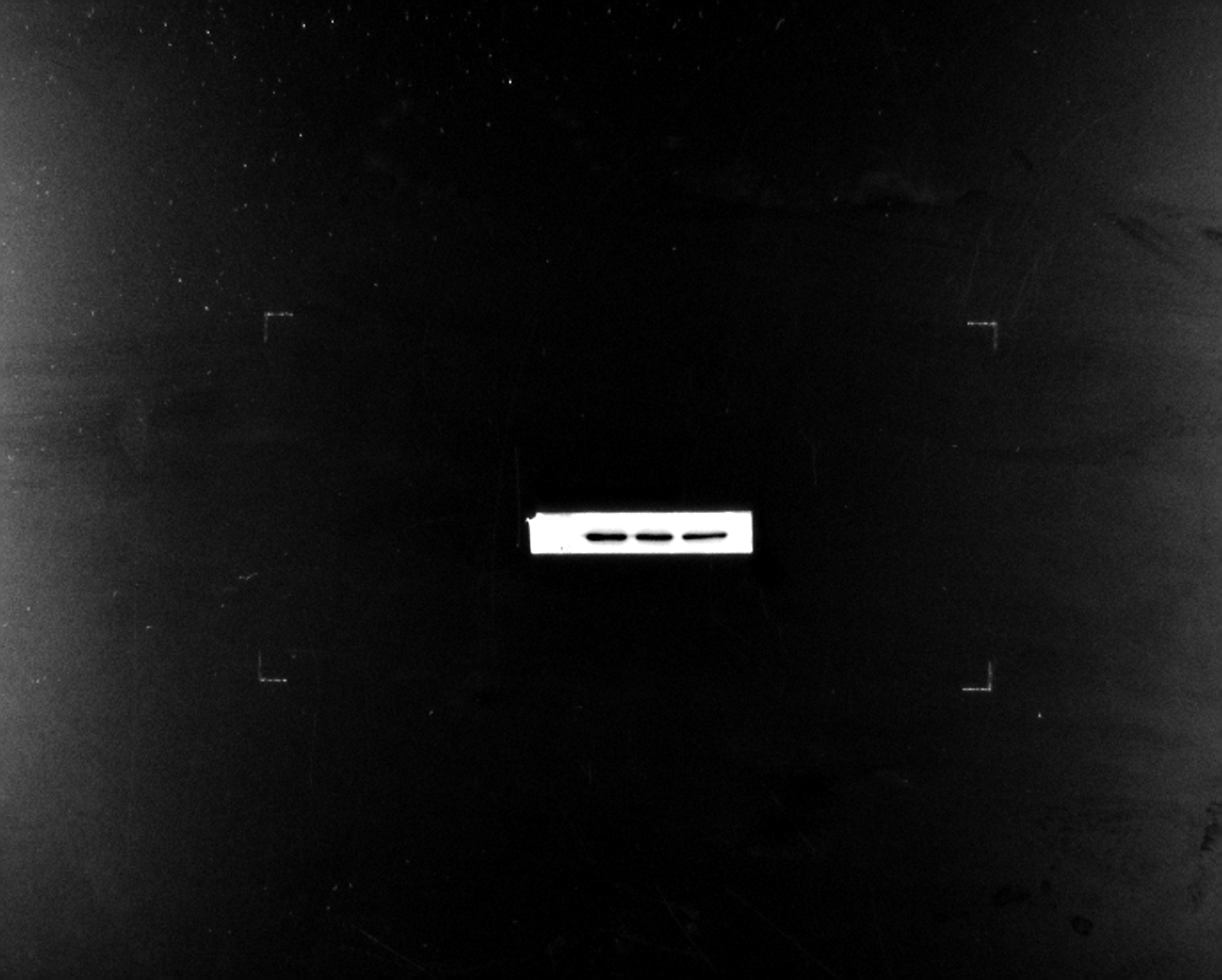

Supplement: Supplementary file 9 — Source data Fig. 4 [file 44318_2024_359_MOESM9_ESM.zip › Figure 4/Fig 4A/4-input-GAPDH-merge.Tif]

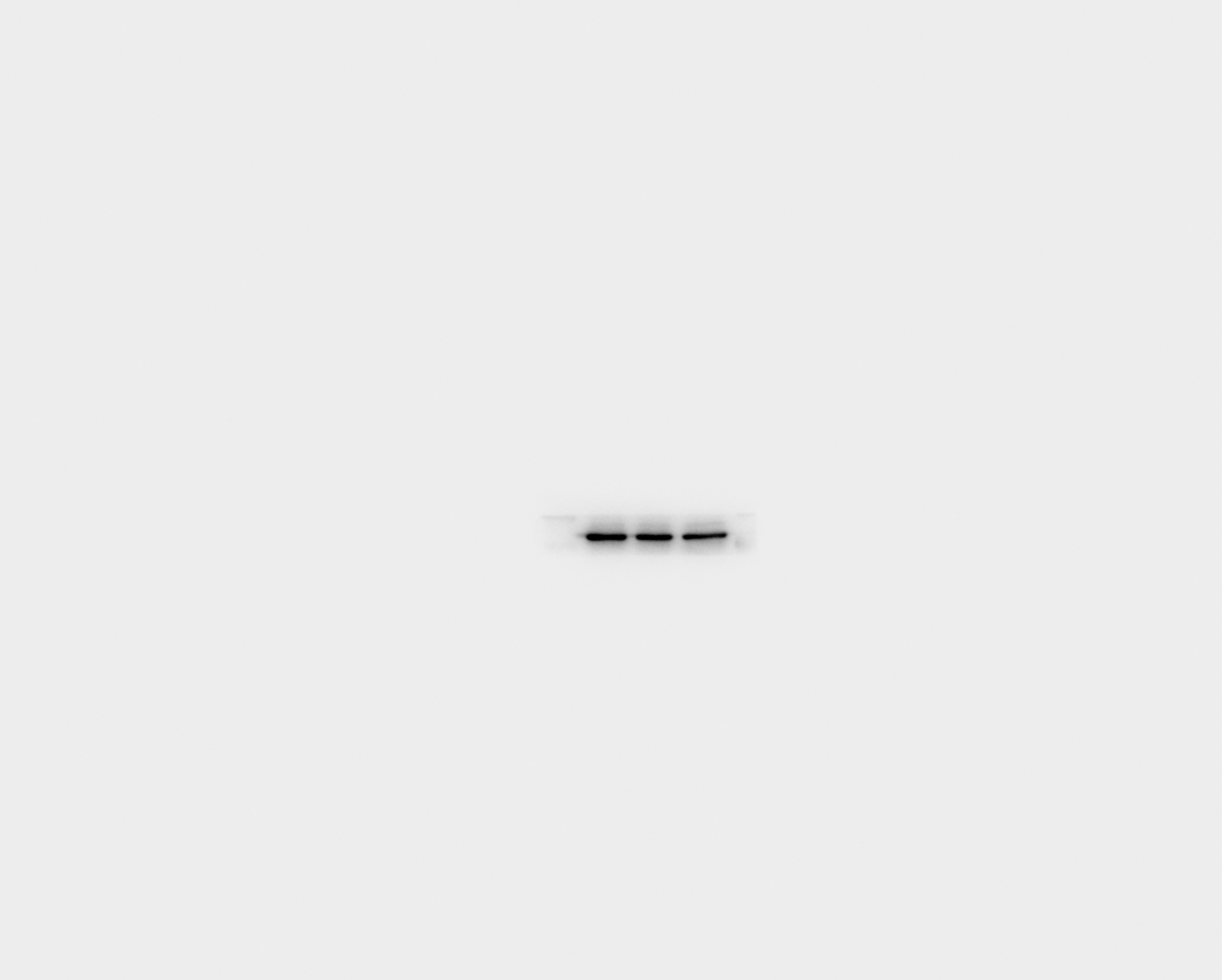

Supplement: Supplementary file 9 — Source data Fig. 4 [file 44318_2024_359_MOESM9_ESM.zip › Figure 4/Fig 4A/4-input-GAPDH.Tif]

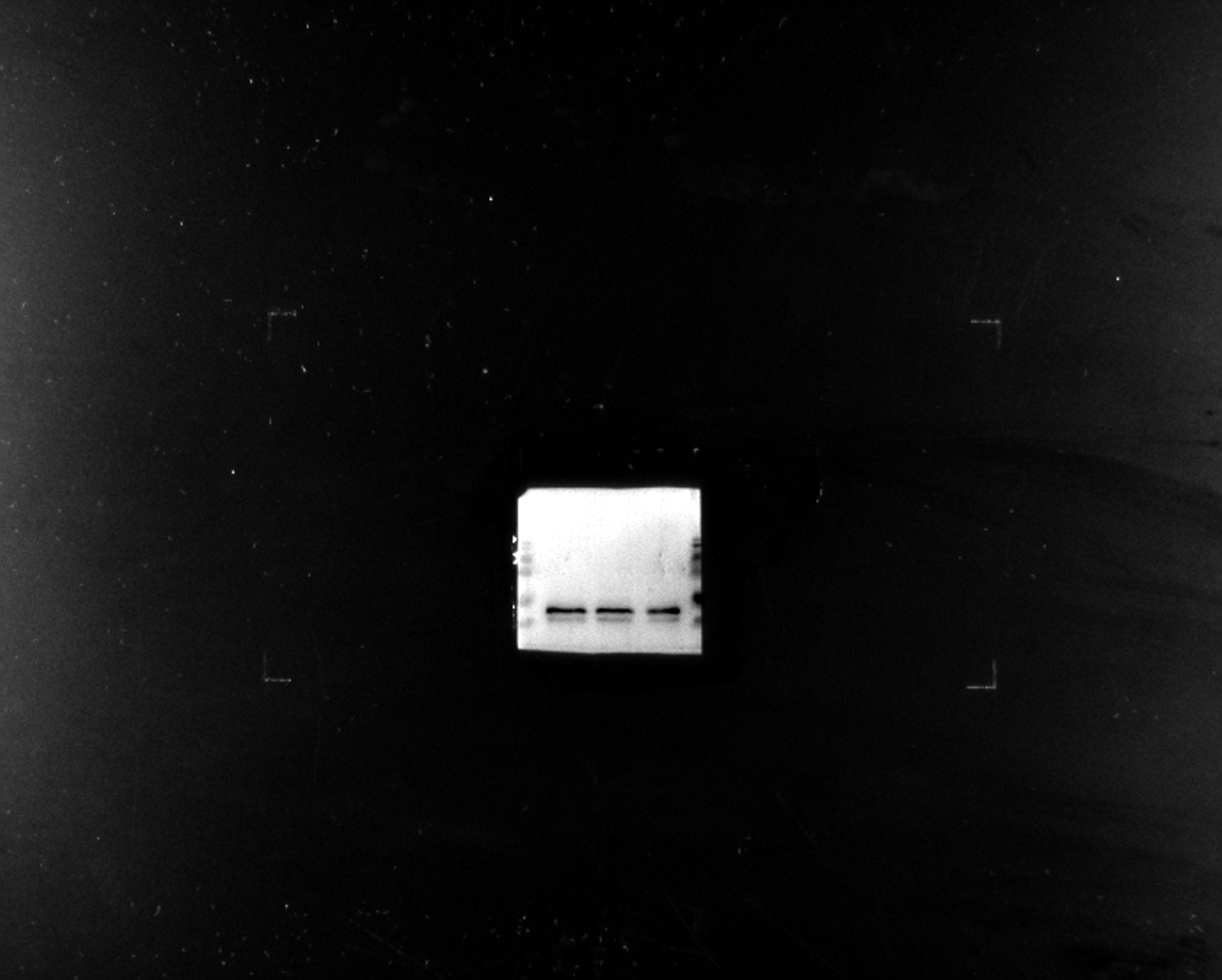

Supplement: Supplementary file 9 — Source data Fig. 4 [file 44318_2024_359_MOESM9_ESM.zip › Figure 4/Fig 4A/5-ip(TRIM21)-TRIM21-merge.Tif]

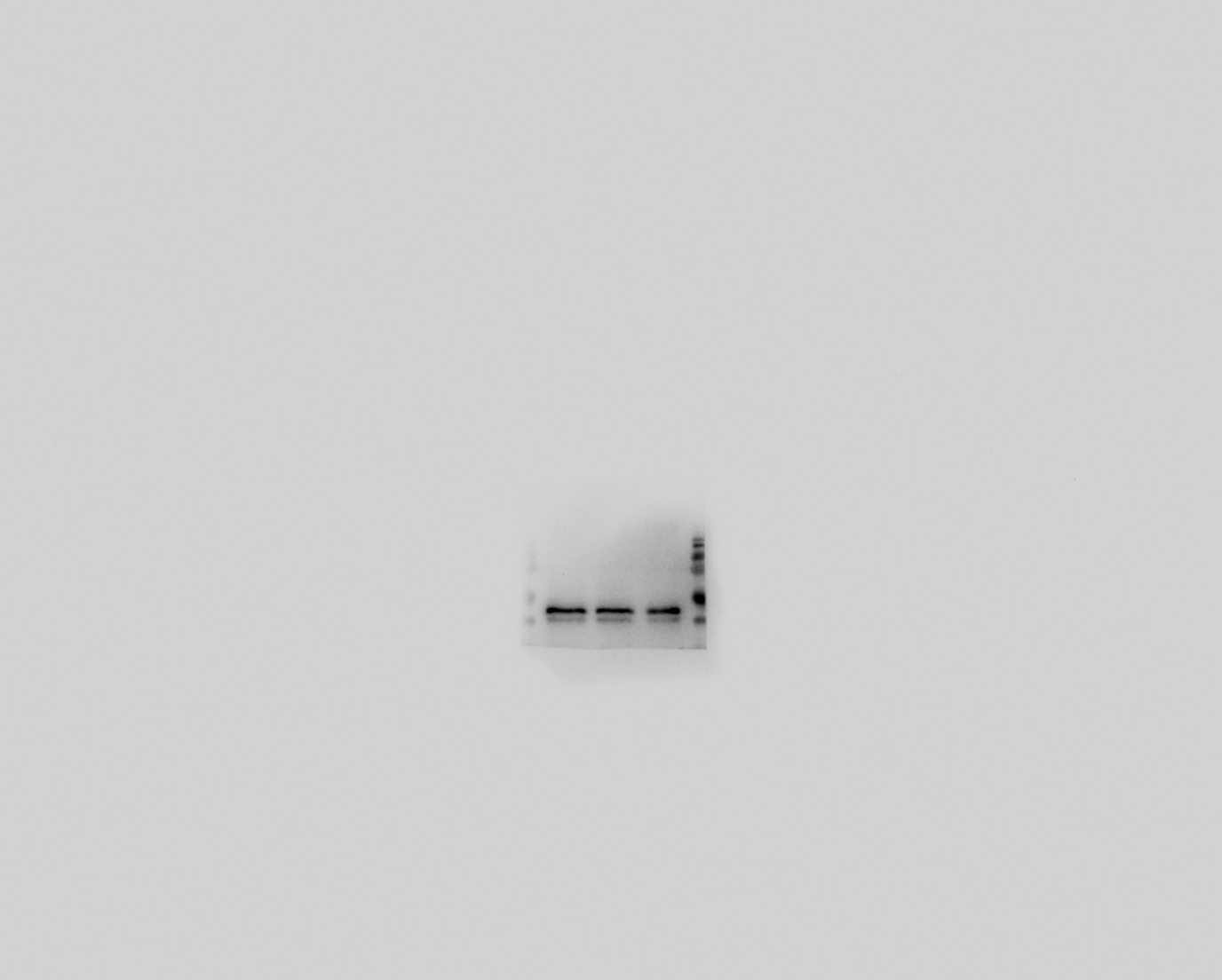

Supplement: Supplementary file 9 — Source data Fig. 4 [file 44318_2024_359_MOESM9_ESM.zip › Figure 4/Fig 4A/5-ip(TRIM21)-TRIM21.Tif]

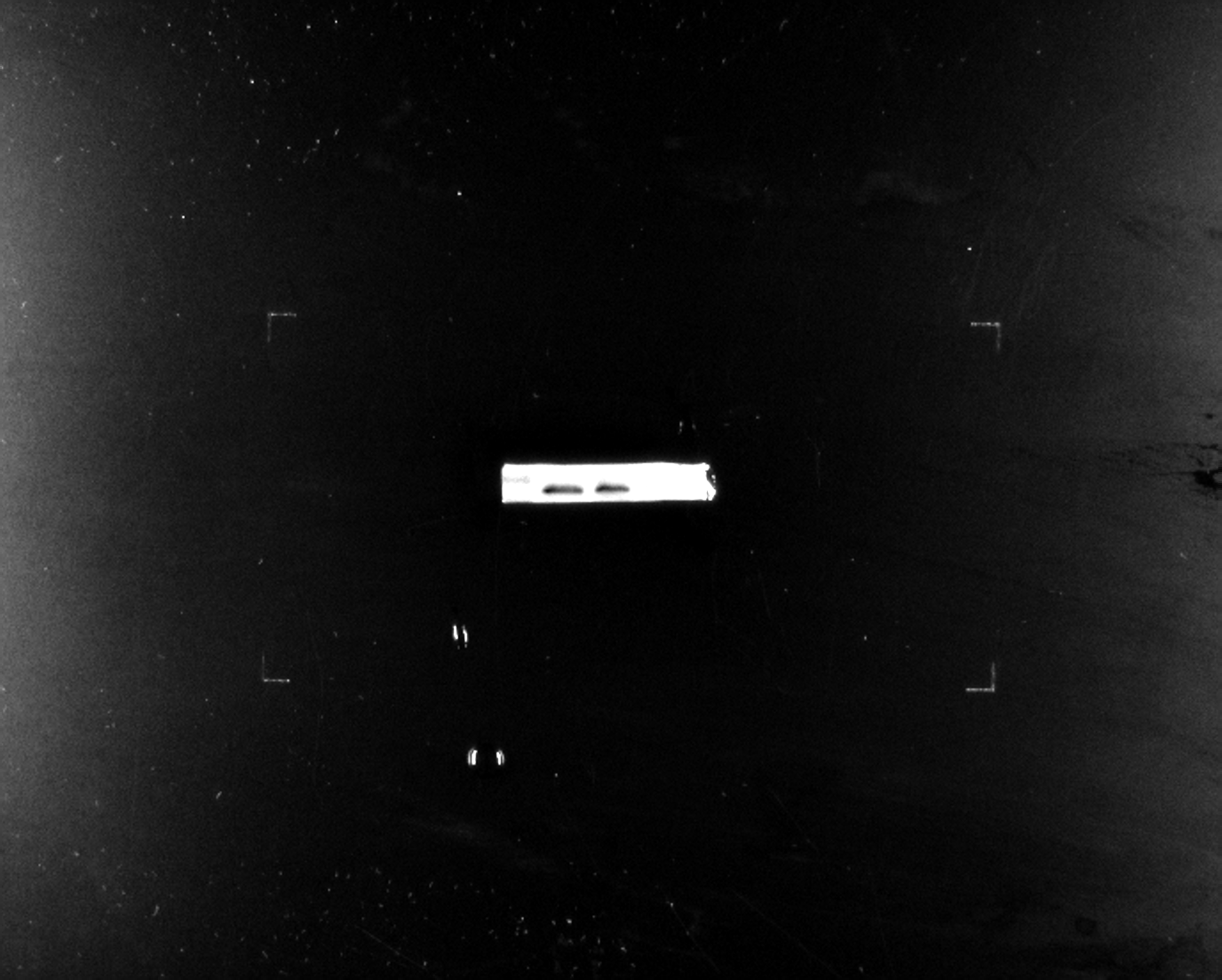

Supplement: Supplementary file 9 — Source data Fig. 4 [file 44318_2024_359_MOESM9_ESM.zip › Figure 4/Fig 4A/6-ip(TRIM21)-P27-merge.Tif]

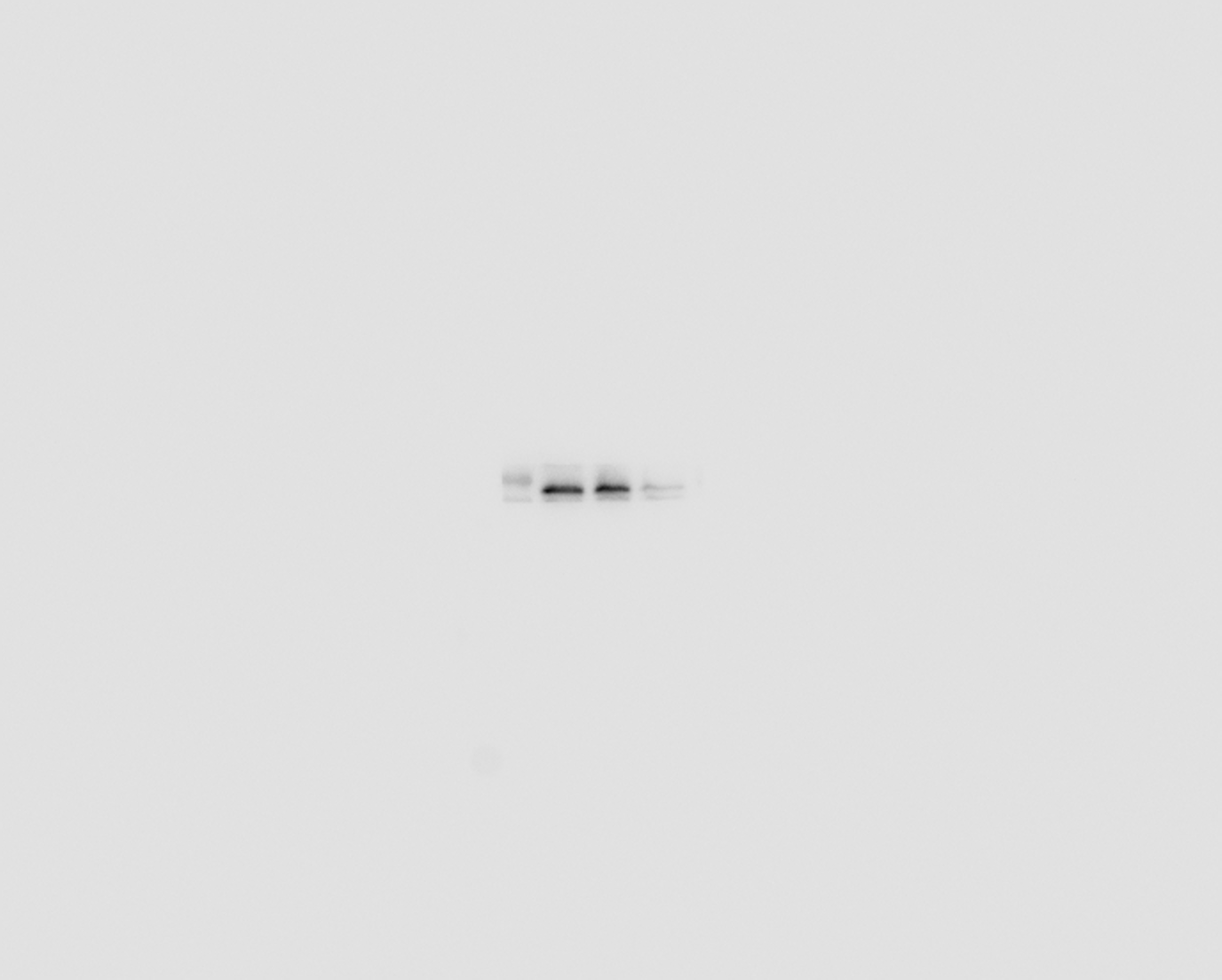

Supplement: Supplementary file 9 — Source data Fig. 4 [file 44318_2024_359_MOESM9_ESM.zip › Figure 4/Fig 4A/6-ip(TRIM21)-P27.Tif]

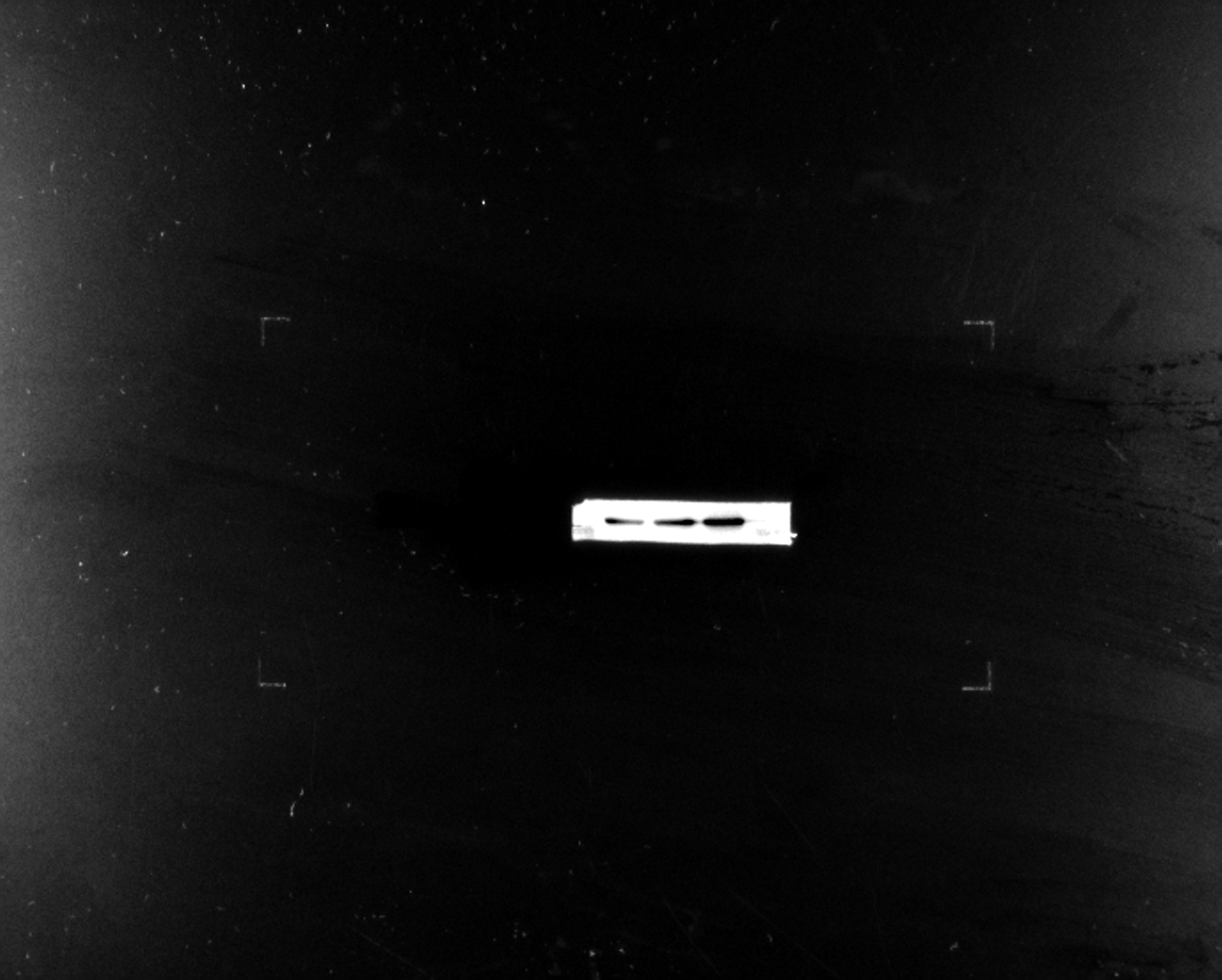

Supplement: Supplementary file 9 — Source data Fig. 4 [file 44318_2024_359_MOESM9_ESM.zip › Figure 4/Fig 4A/7-ip(p27)-p27-merge.Tif]

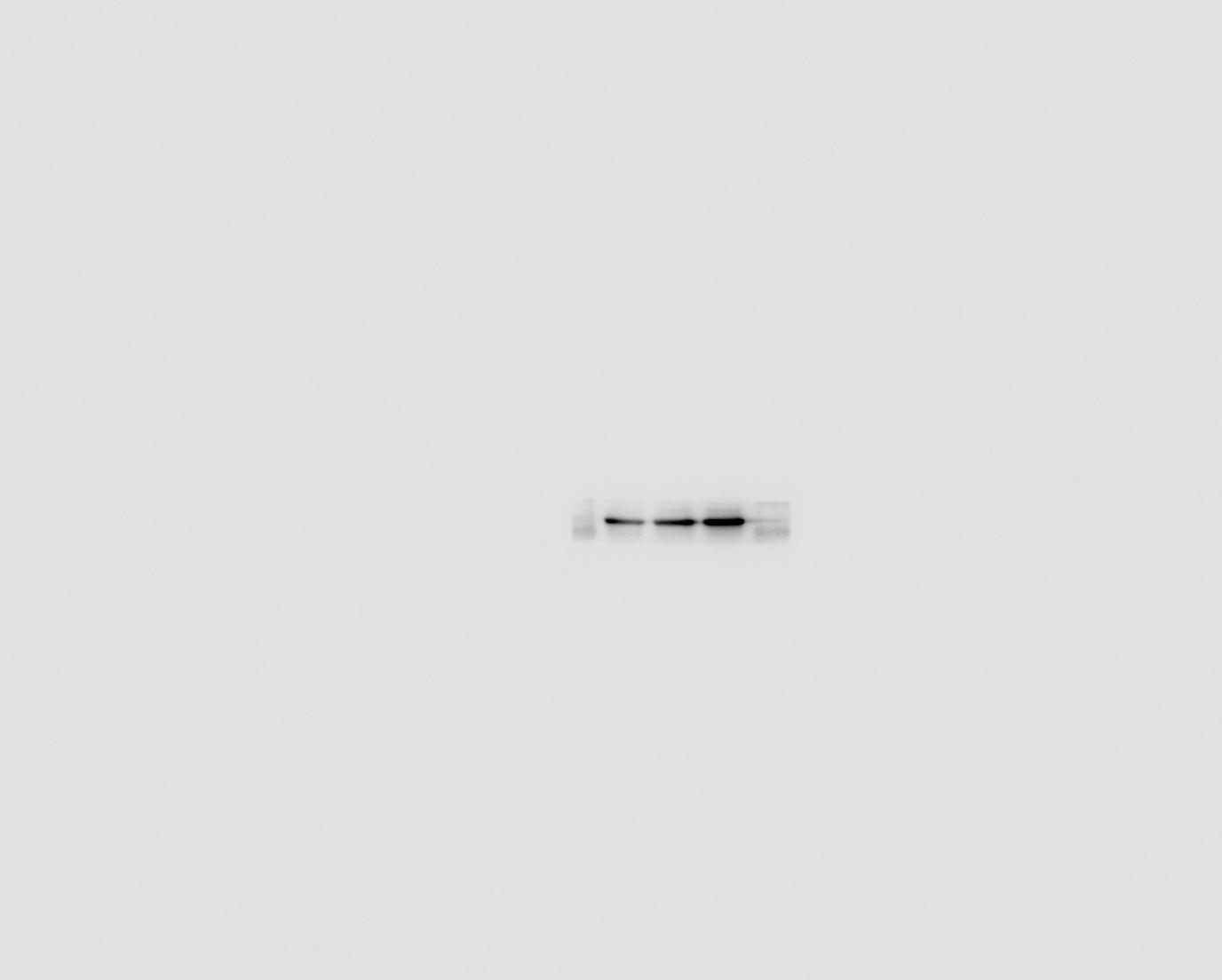

Supplement: Supplementary file 9 — Source data Fig. 4 [file 44318_2024_359_MOESM9_ESM.zip › Figure 4/Fig 4A/7-ip(p27)-p27.Tif]

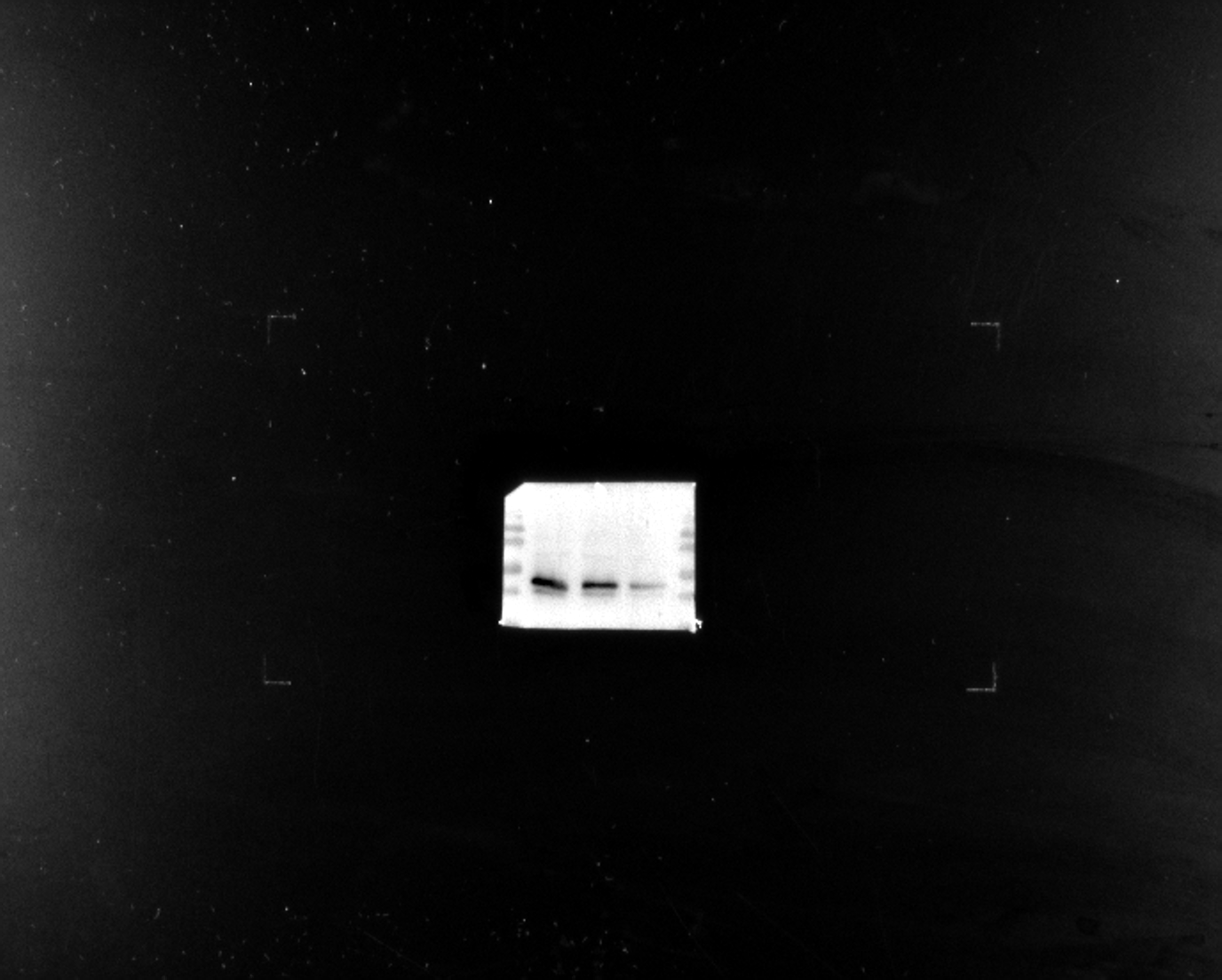

Supplement: Supplementary file 9 — Source data Fig. 4 [file 44318_2024_359_MOESM9_ESM.zip › Figure 4/Fig 4A/8-ip(p27)-TRIM21-merge.Tif]

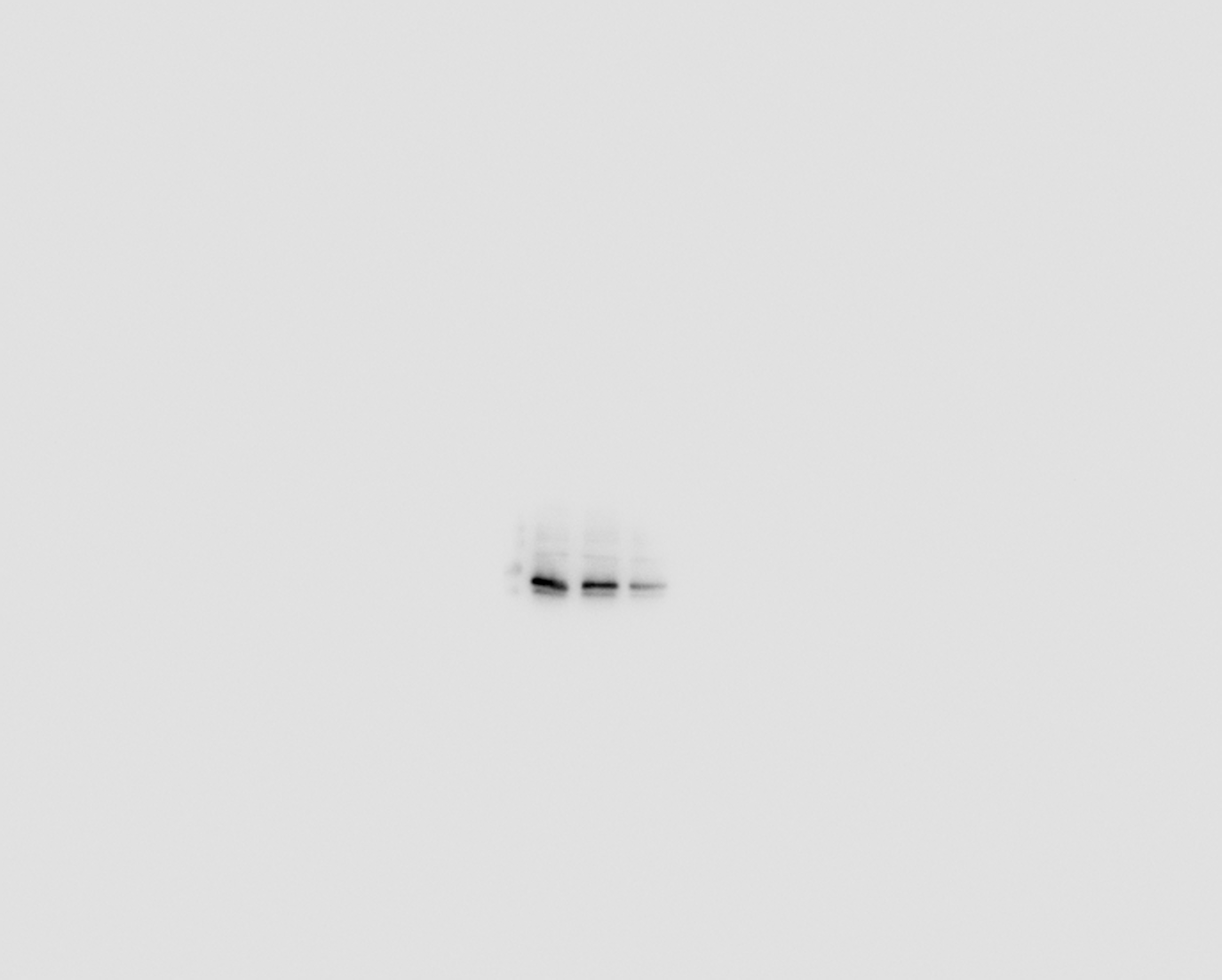

Supplement: Supplementary file 9 — Source data Fig. 4 [file 44318_2024_359_MOESM9_ESM.zip › Figure 4/Fig 4A/8-ip(p27)-TRIM21.Tif]

Fig. 4A

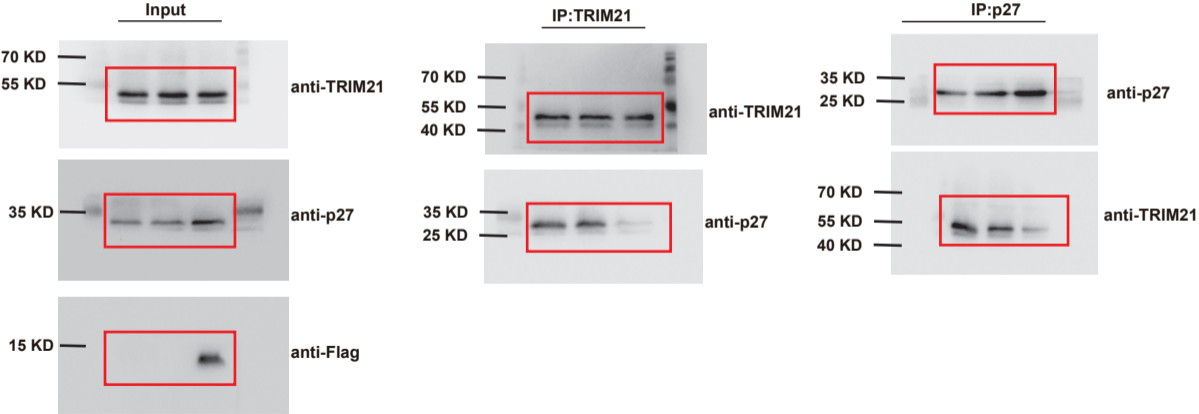

Supplement: Supplementary file 9 — Source data Fig. 4 [file 44318_2024_359_MOESM9_ESM.zip › Figure 4/Fig 4A/Fig 4A.pdf]

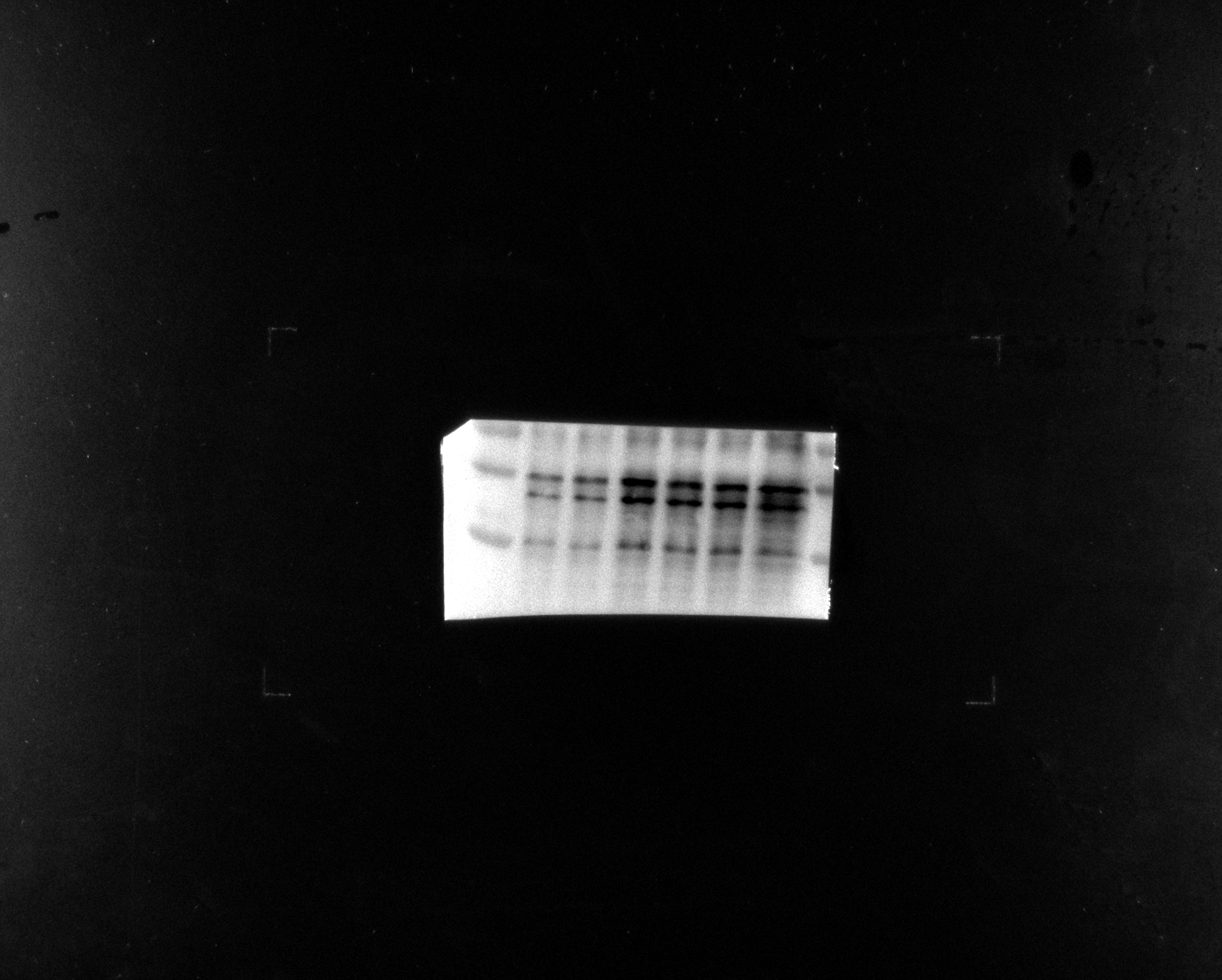

Supplement: Supplementary file 9 — Source data Fig. 4 [file 44318_2024_359_MOESM9_ESM.zip › Figure 4/Fig 4B/1-input-p27-merge.Tif]

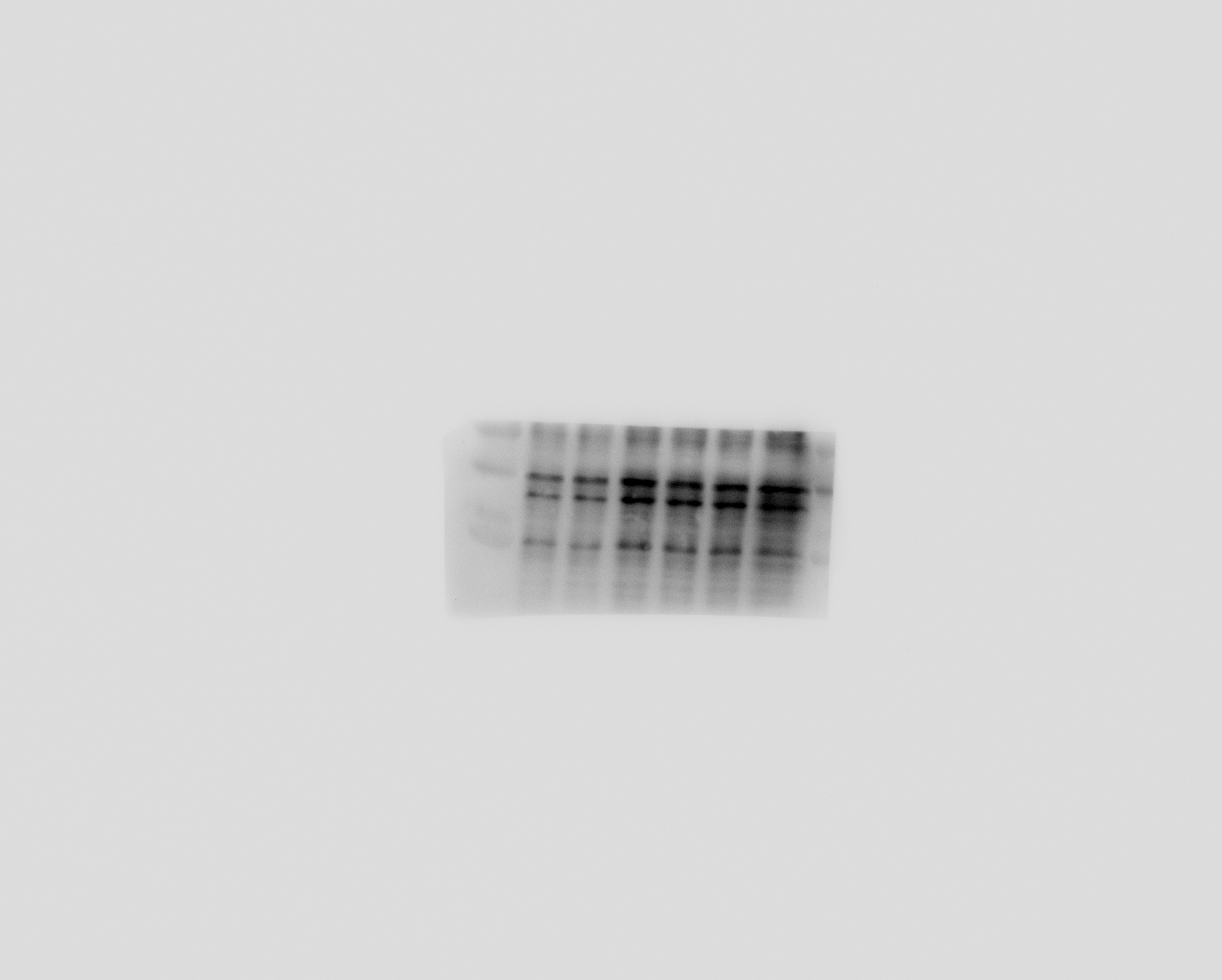

Supplement: Supplementary file 9 — Source data Fig. 4 [file 44318_2024_359_MOESM9_ESM.zip › Figure 4/Fig 4B/1-input-p27.Tif]

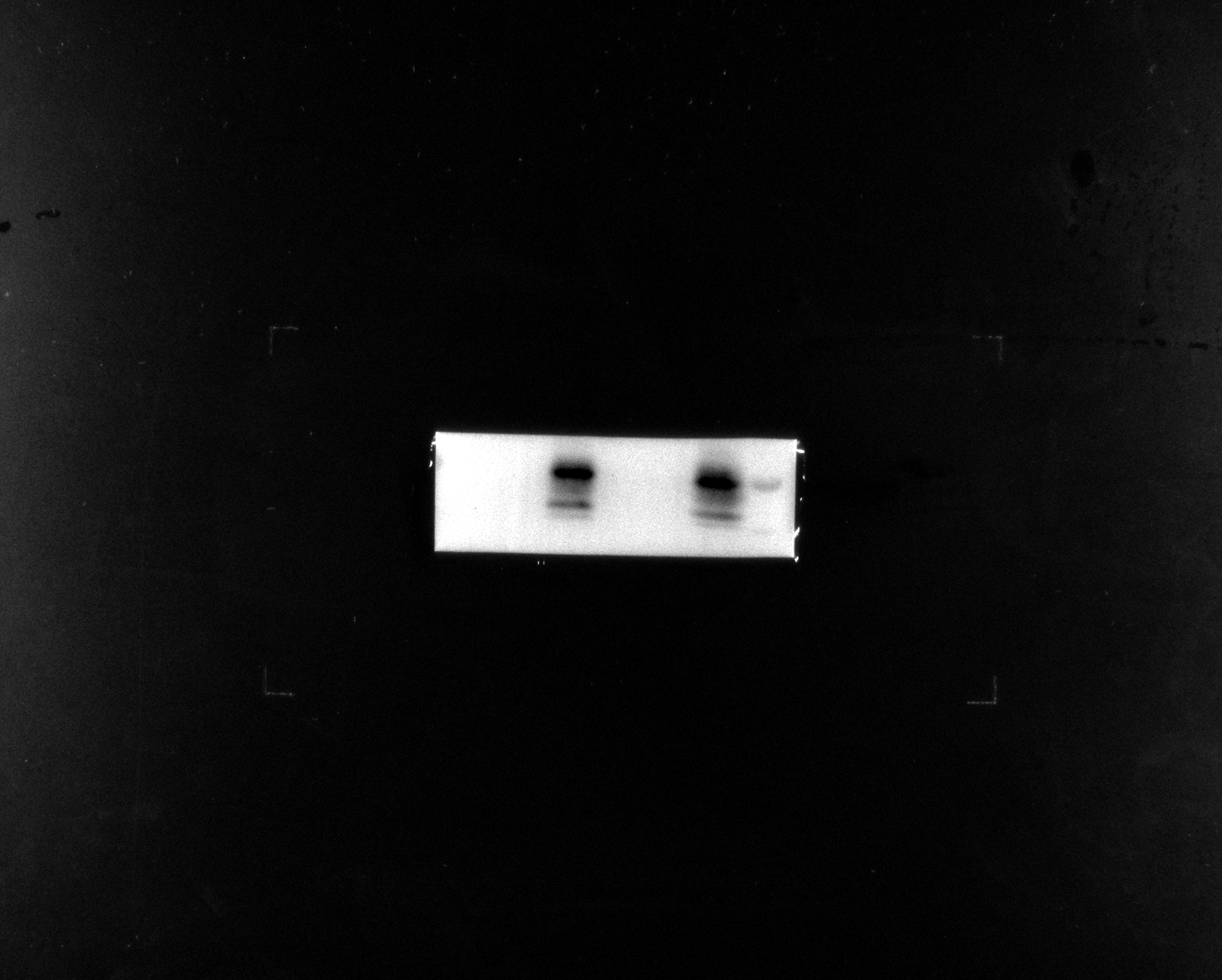

Supplement: Supplementary file 9 — Source data Fig. 4 [file 44318_2024_359_MOESM9_ESM.zip › Figure 4/Fig 4B/2-input-Flag-merge.Tif]

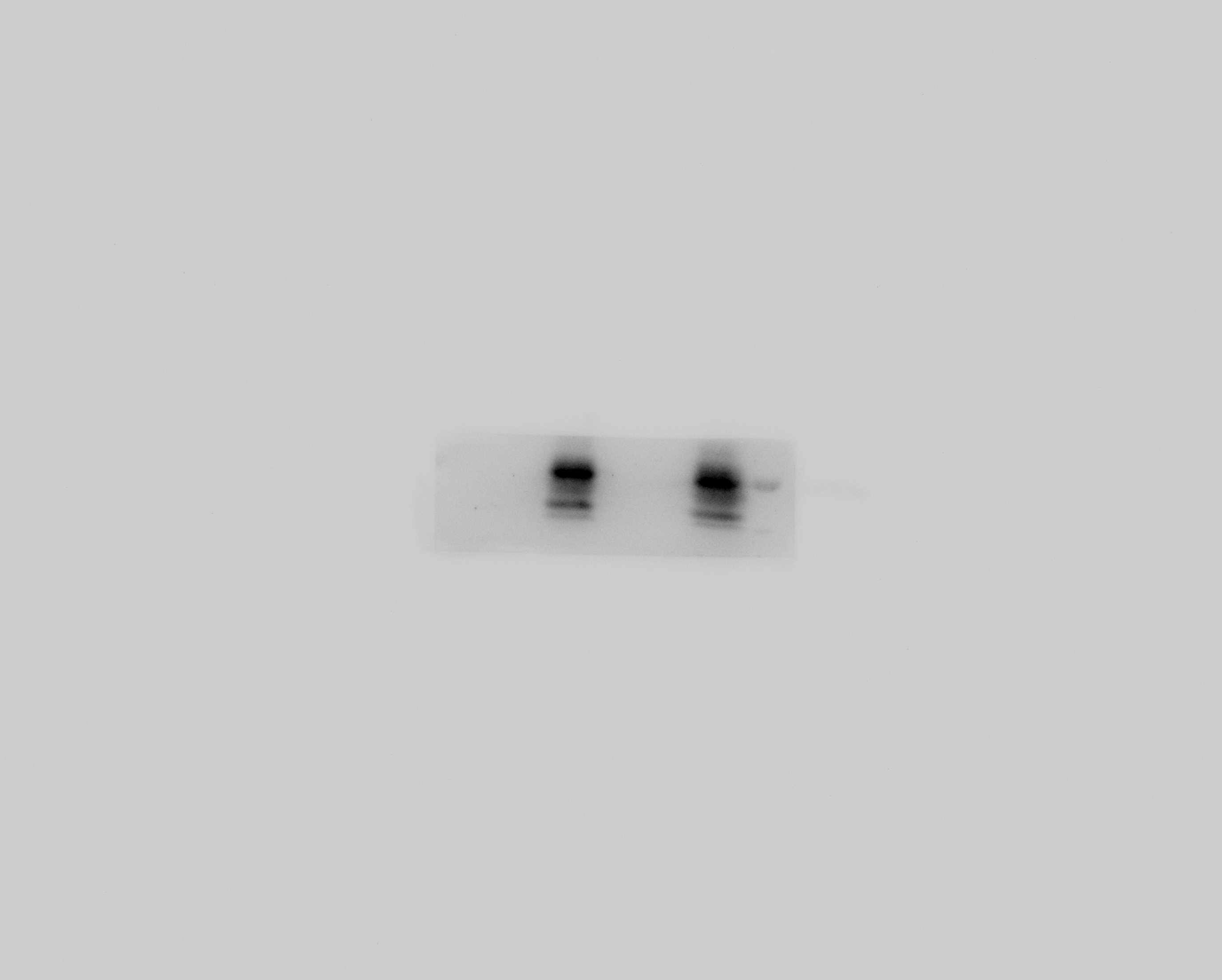

Supplement: Supplementary file 9 — Source data Fig. 4 [file 44318_2024_359_MOESM9_ESM.zip › Figure 4/Fig 4B/2-input-Flag.Tif]

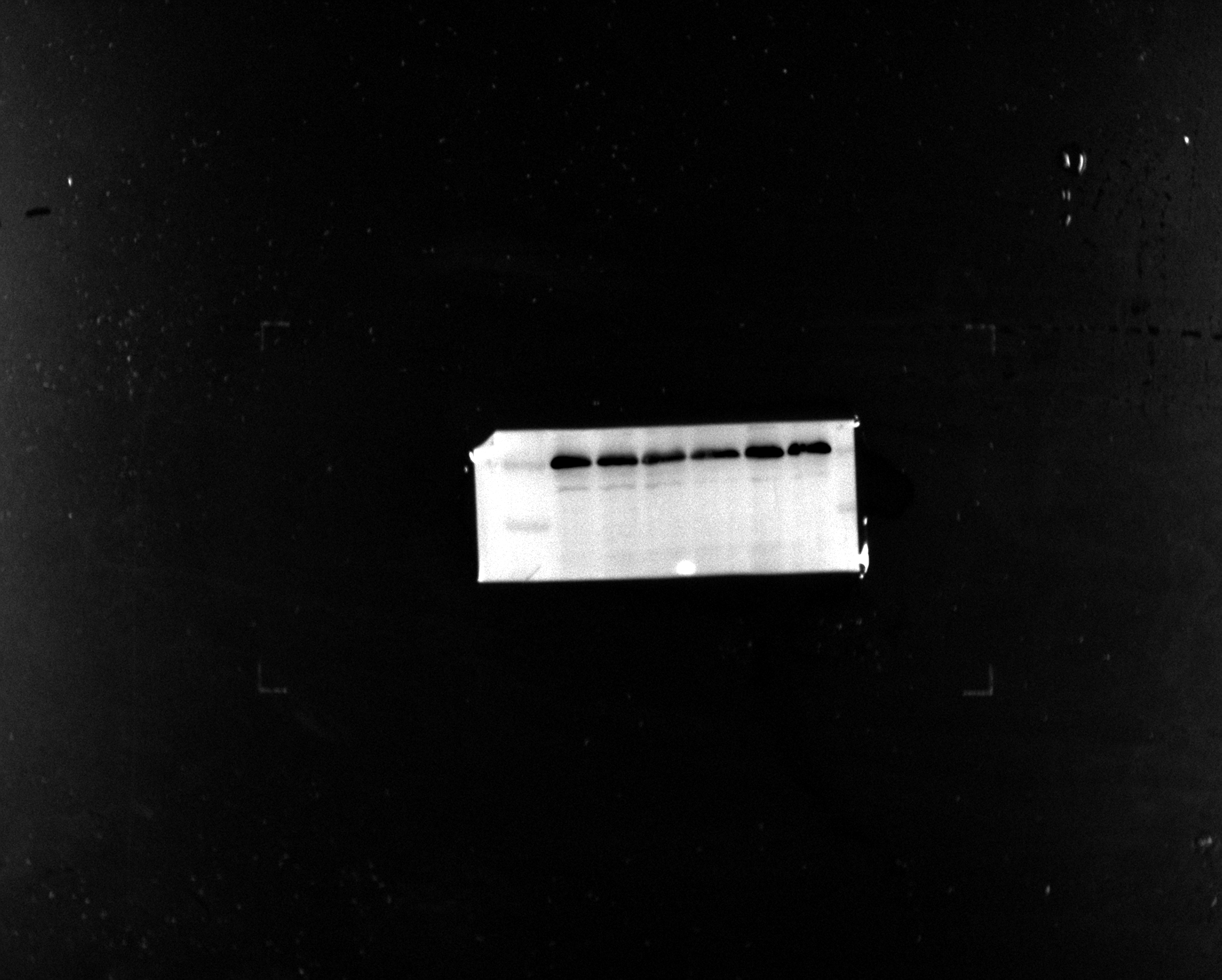

Supplement: Supplementary file 9 — Source data Fig. 4 [file 44318_2024_359_MOESM9_ESM.zip › Figure 4/Fig 4B/3-input-GAPDH-merge.Tif]

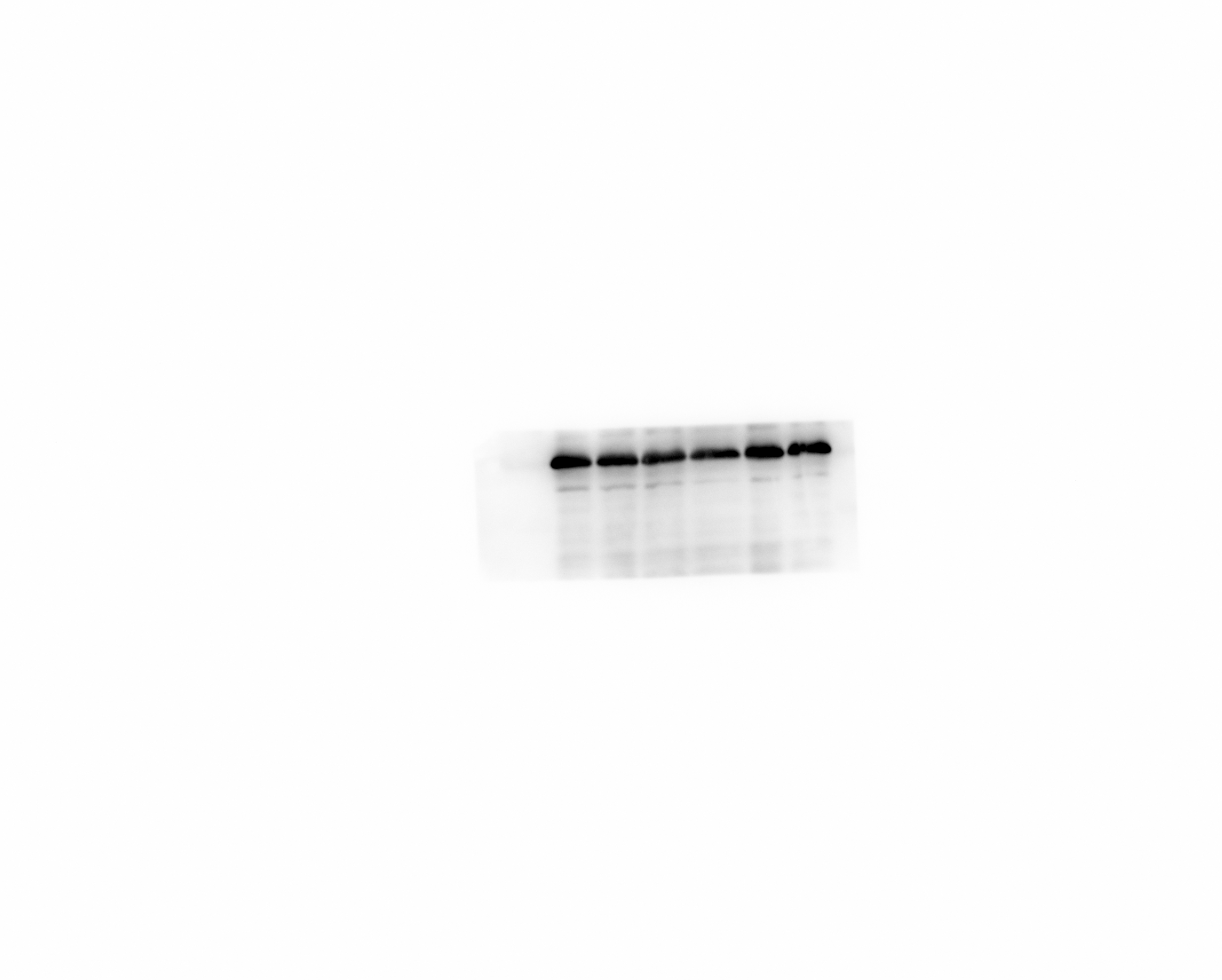

Supplement: Supplementary file 9 — Source data Fig. 4 [file 44318_2024_359_MOESM9_ESM.zip › Figure 4/Fig 4B/3-input-GAPDH.Tif]

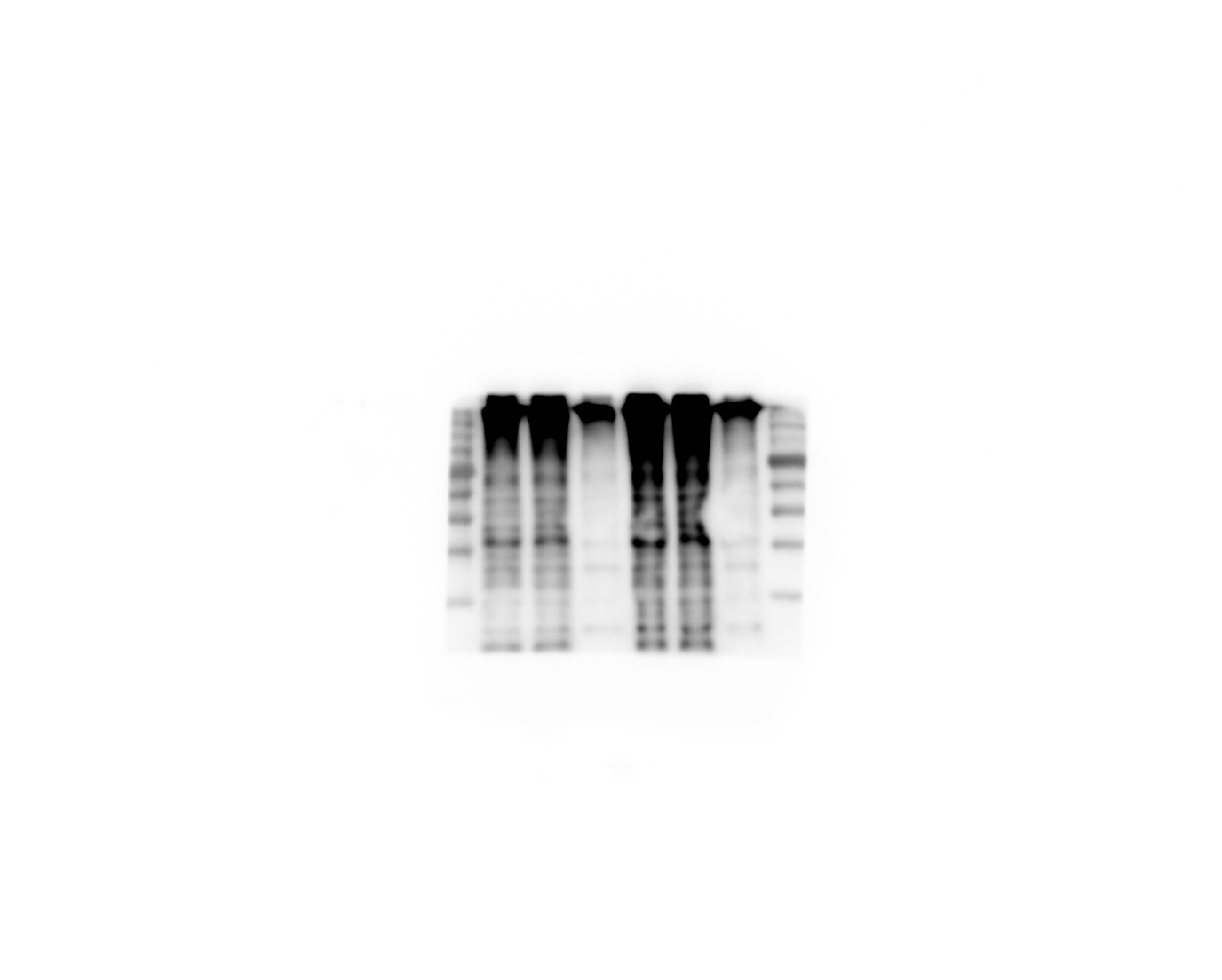

Supplement: Supplementary file 9 — Source data Fig. 4 [file 44318_2024_359_MOESM9_ESM.zip › Figure 4/Fig 4B/4-ip(p27) wb ub .Tif]

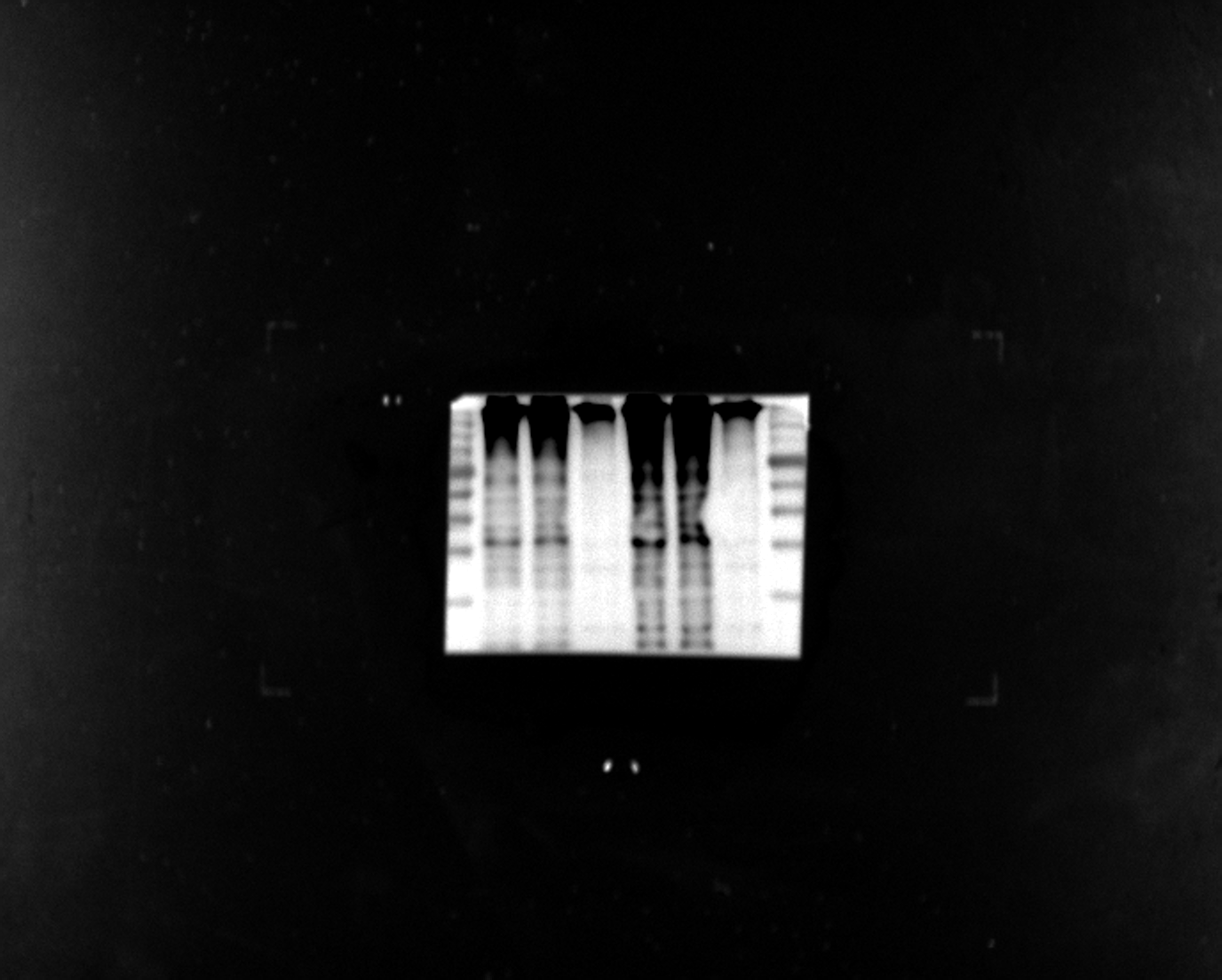

Supplement: Supplementary file 9 — Source data Fig. 4 [file 44318_2024_359_MOESM9_ESM.zip › Figure 4/Fig 4B/4-ip(p27) wb ub-merge.Tif]

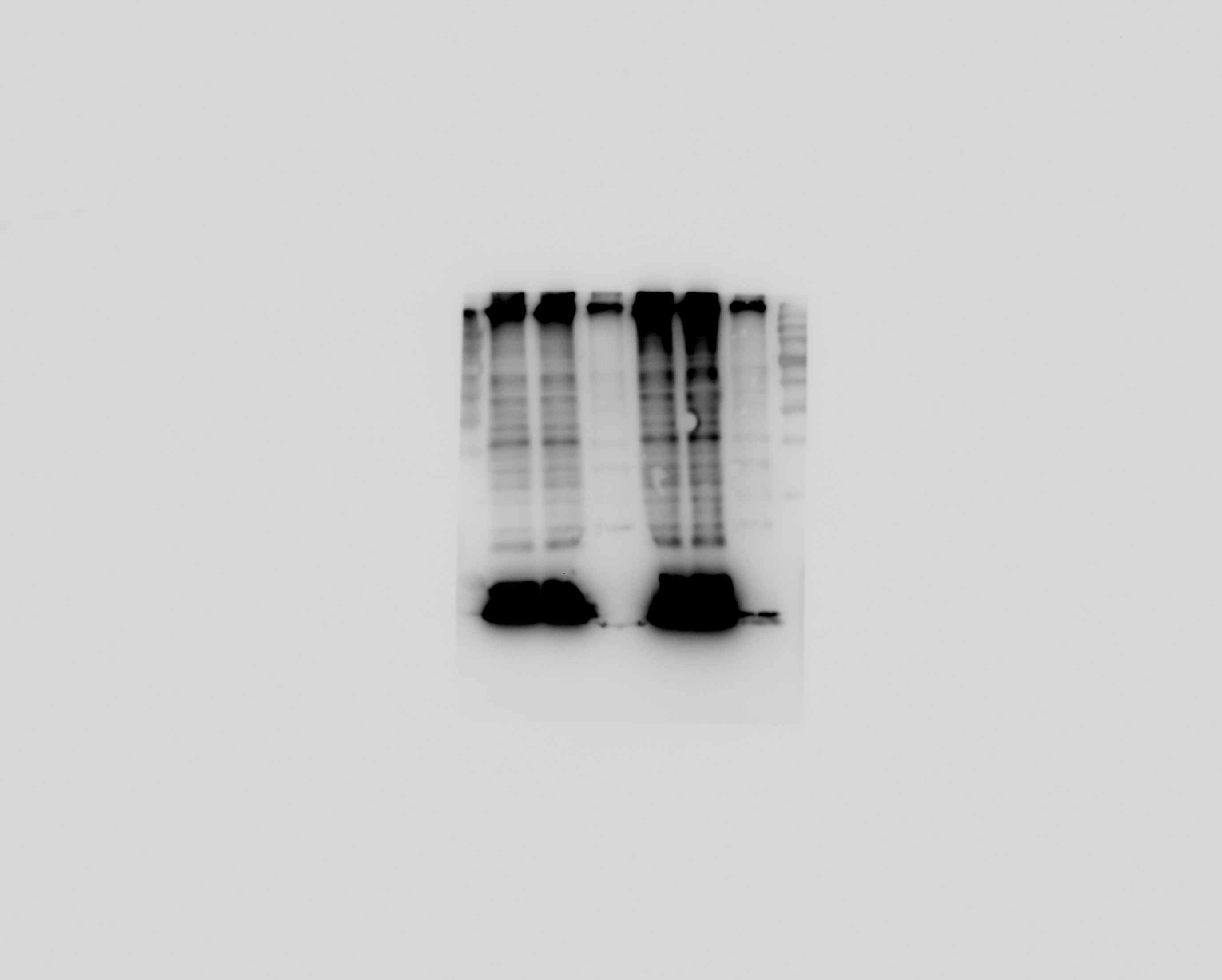

Supplement: Supplementary file 9 — Source data Fig. 4 [file 44318_2024_359_MOESM9_ESM.zip › Figure 4/Fig 4B/5-ip(HA) wb P27KIP1 .Tif]

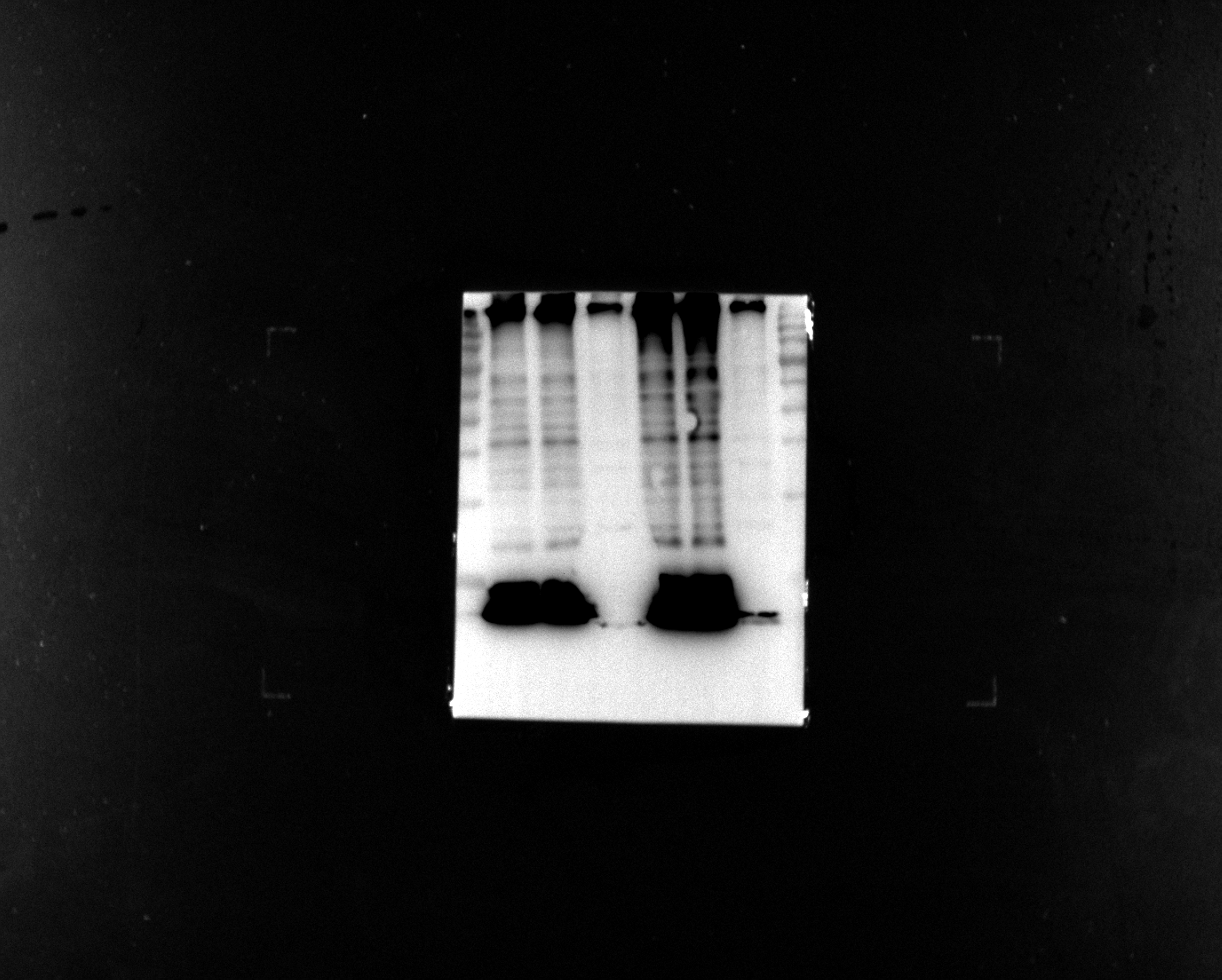

Supplement: Supplementary file 9 — Source data Fig. 4 [file 44318_2024_359_MOESM9_ESM.zip › Figure 4/Fig 4B/5-ip(HA) wb P27KIP1-merge.Tif]

Fig 4B

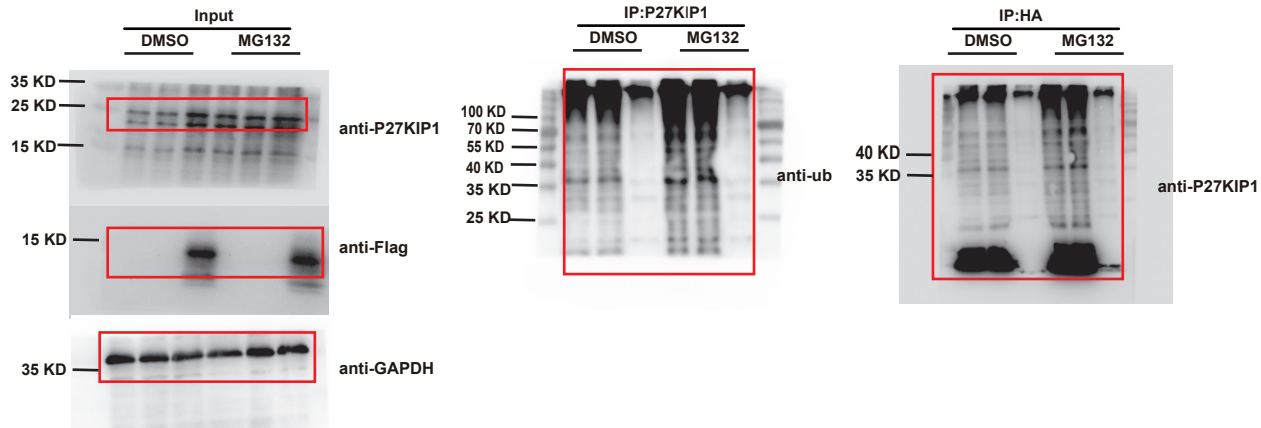

Supplement: Supplementary file 9 — Source data Fig. 4 [file 44318_2024_359_MOESM9_ESM.zip › Figure 4/Fig 4B/Fig 4B.pdf]

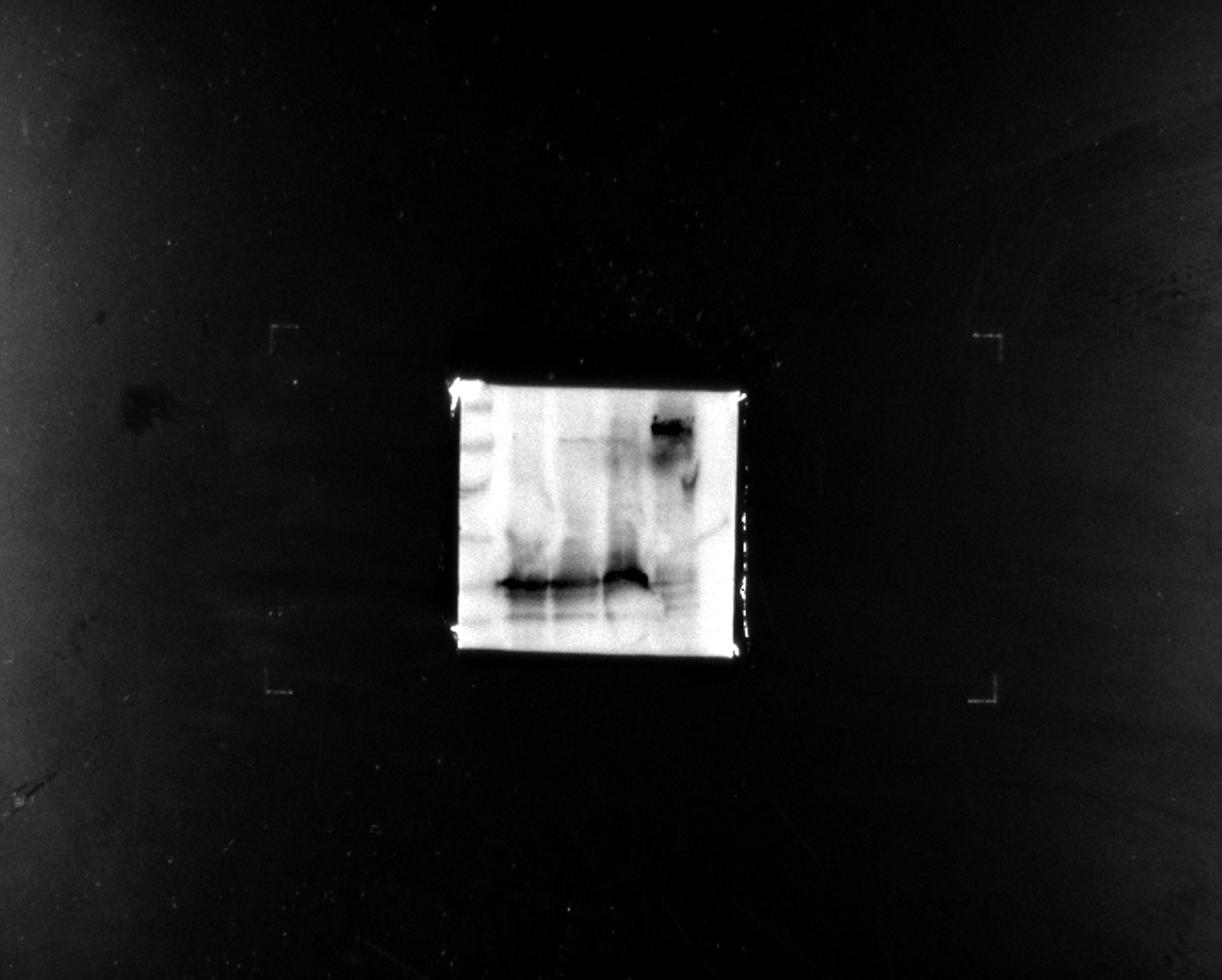

Supplement: Supplementary file 9 — Source data Fig. 4 [file 44318_2024_359_MOESM9_ESM.zip › Figure 4/Fig 4C/1-input-GFP-merge.Tif]

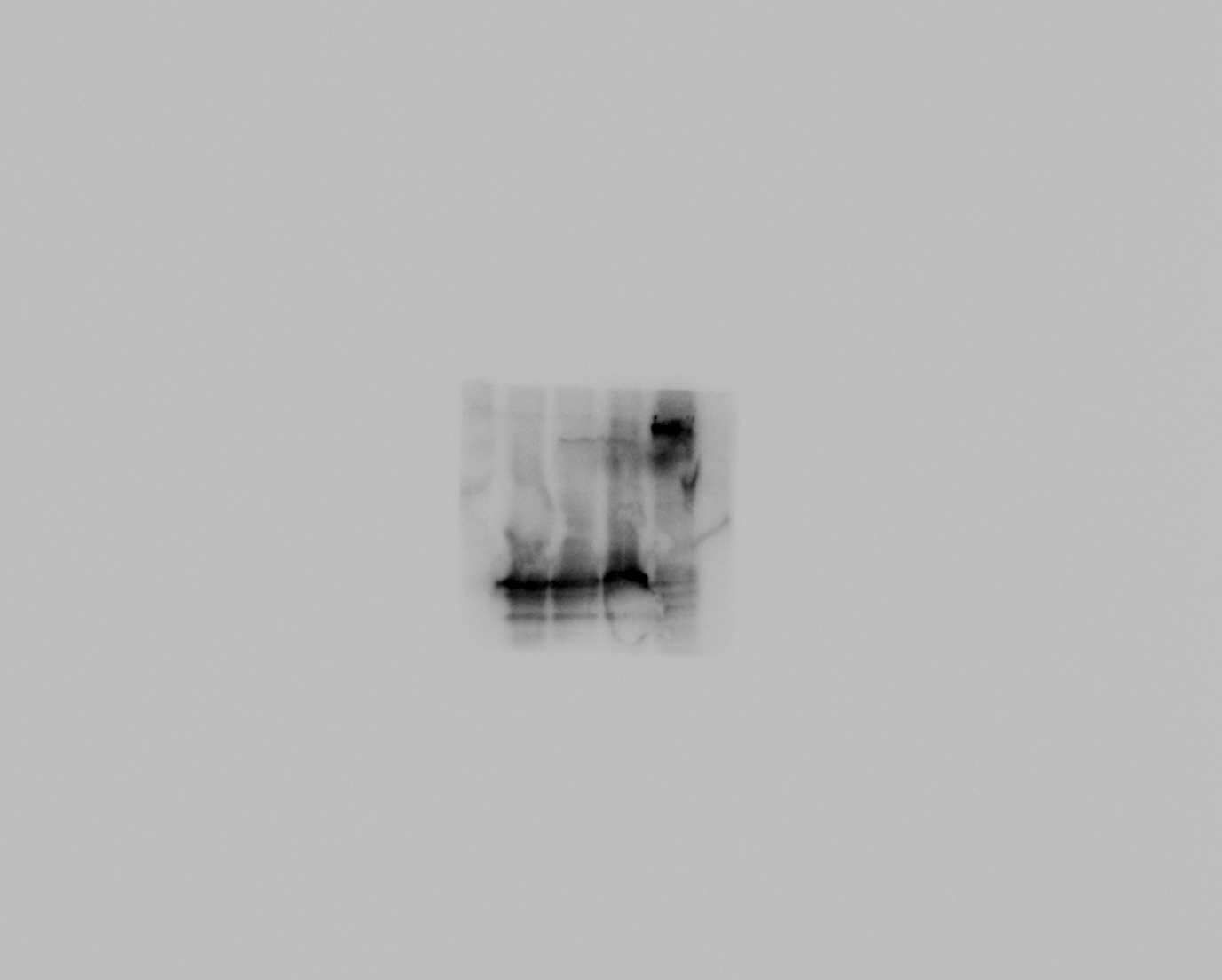

Supplement: Supplementary file 9 — Source data Fig. 4 [file 44318_2024_359_MOESM9_ESM.zip › Figure 4/Fig 4C/1-input-GFP.Tif]

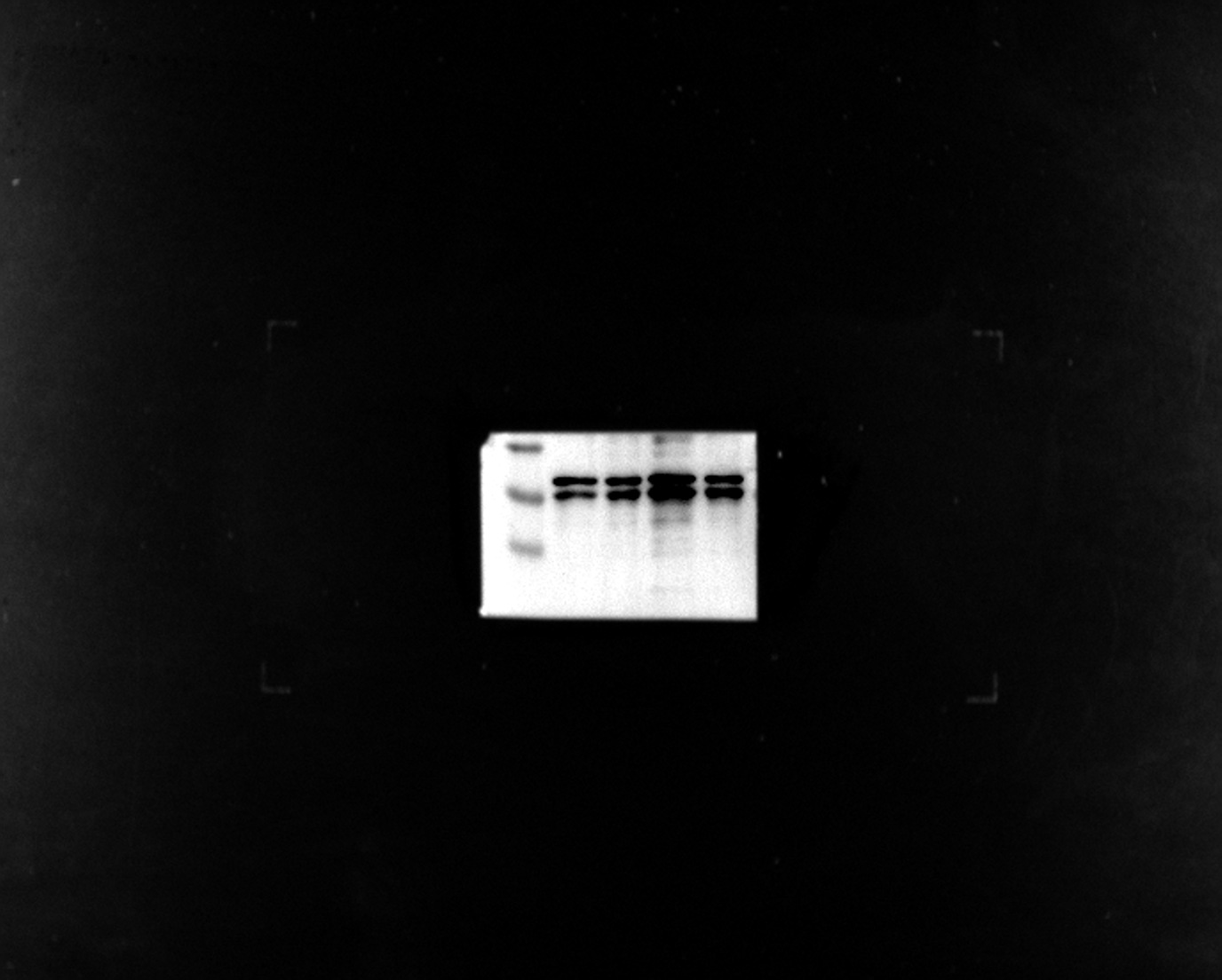

Supplement: Supplementary file 9 — Source data Fig. 4 [file 44318_2024_359_MOESM9_ESM.zip › Figure 4/Fig 4C/3-input-P27KIP1-merge.Tif]

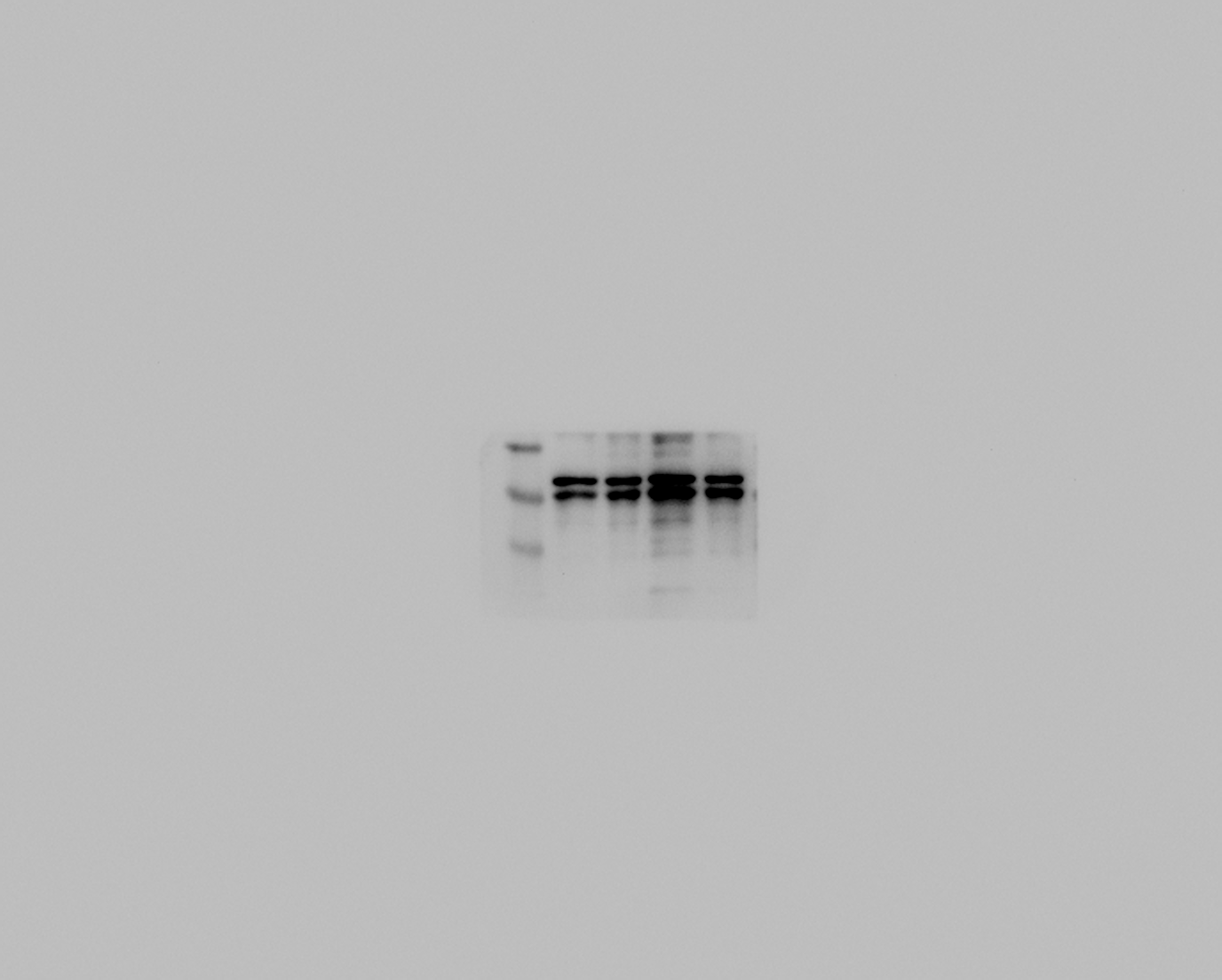

Supplement: Supplementary file 9 — Source data Fig. 4 [file 44318_2024_359_MOESM9_ESM.zip › Figure 4/Fig 4C/3-input-P27KIP1.Tif]

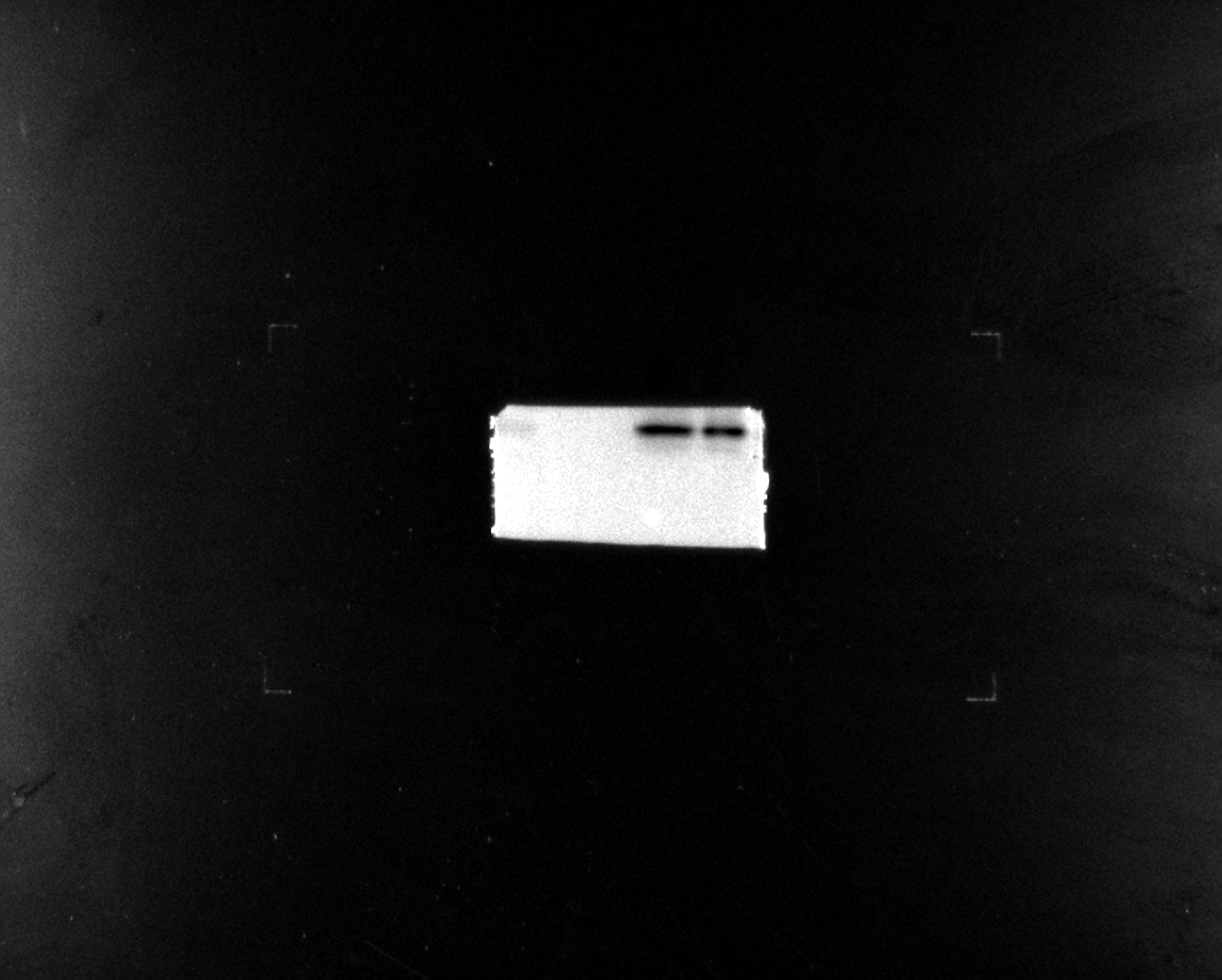

Supplement: Supplementary file 9 — Source data Fig. 4 [file 44318_2024_359_MOESM9_ESM.zip › Figure 4/Fig 4C/4-input-flag-merge.Tif]

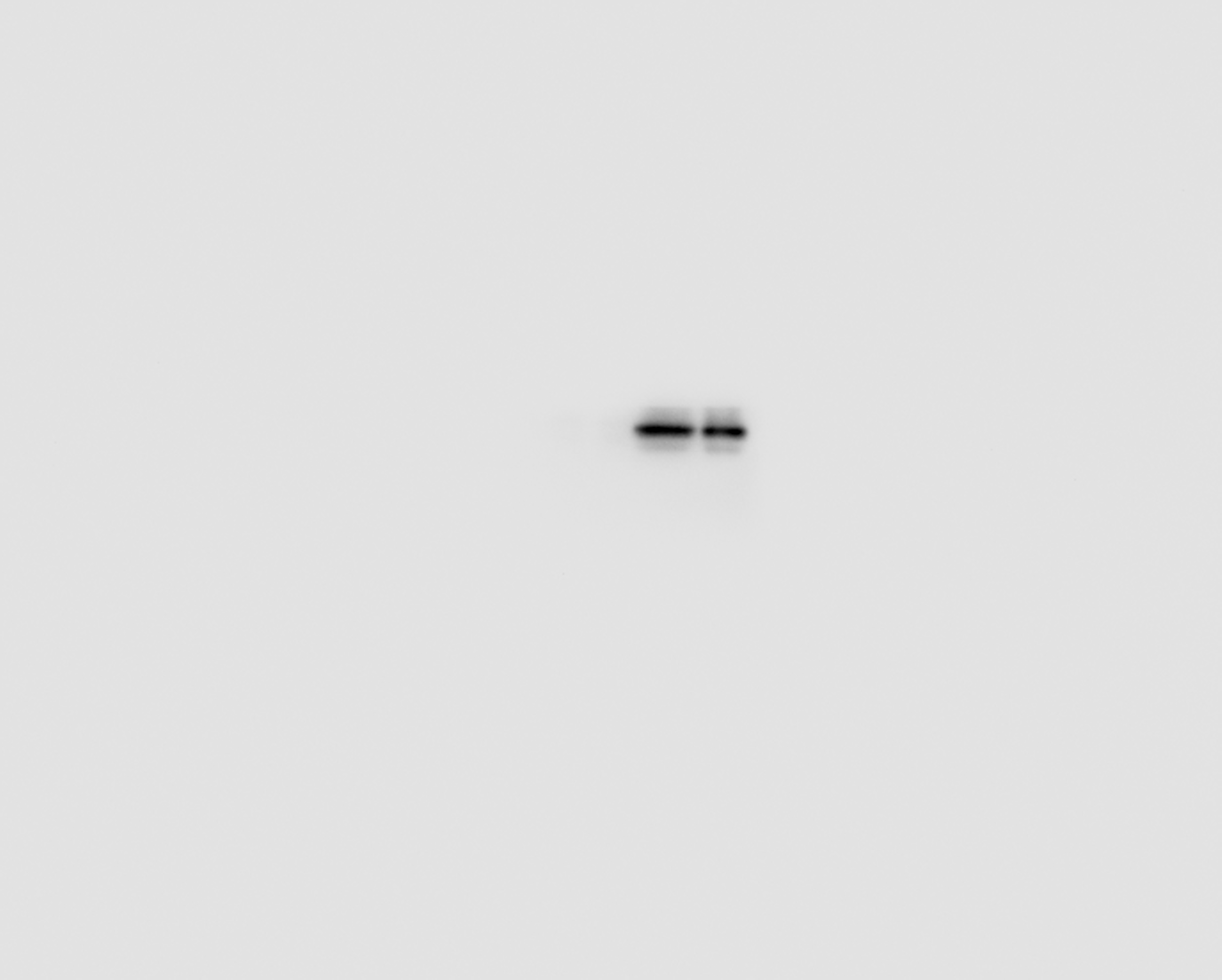

Supplement: Supplementary file 9 — Source data Fig. 4 [file 44318_2024_359_MOESM9_ESM.zip › Figure 4/Fig 4C/4-input-flag.Tif]

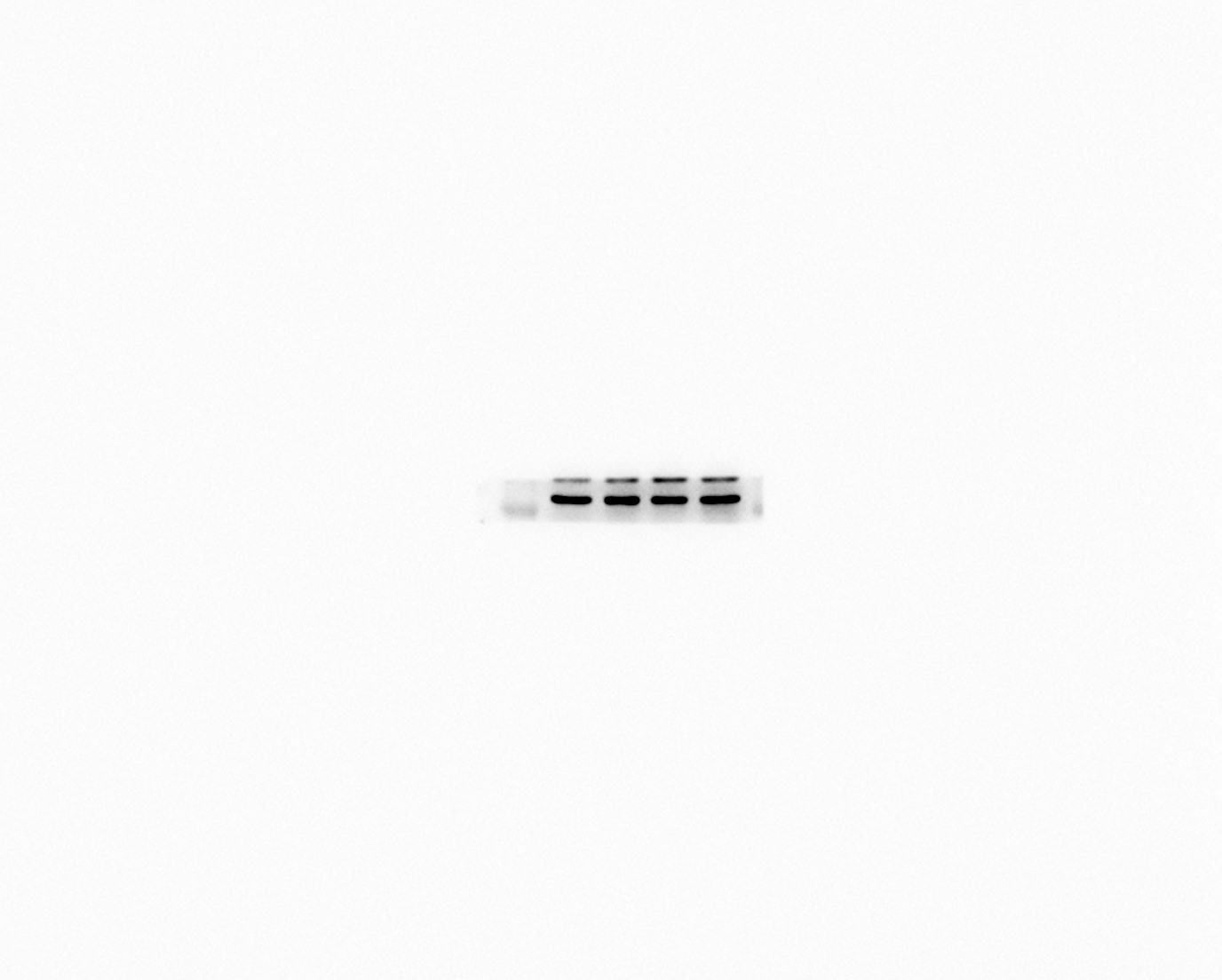

Supplement: Supplementary file 9 — Source data Fig. 4 [file 44318_2024_359_MOESM9_ESM.zip › Figure 4/Fig 4C/5-input-GAPDH .Tif]

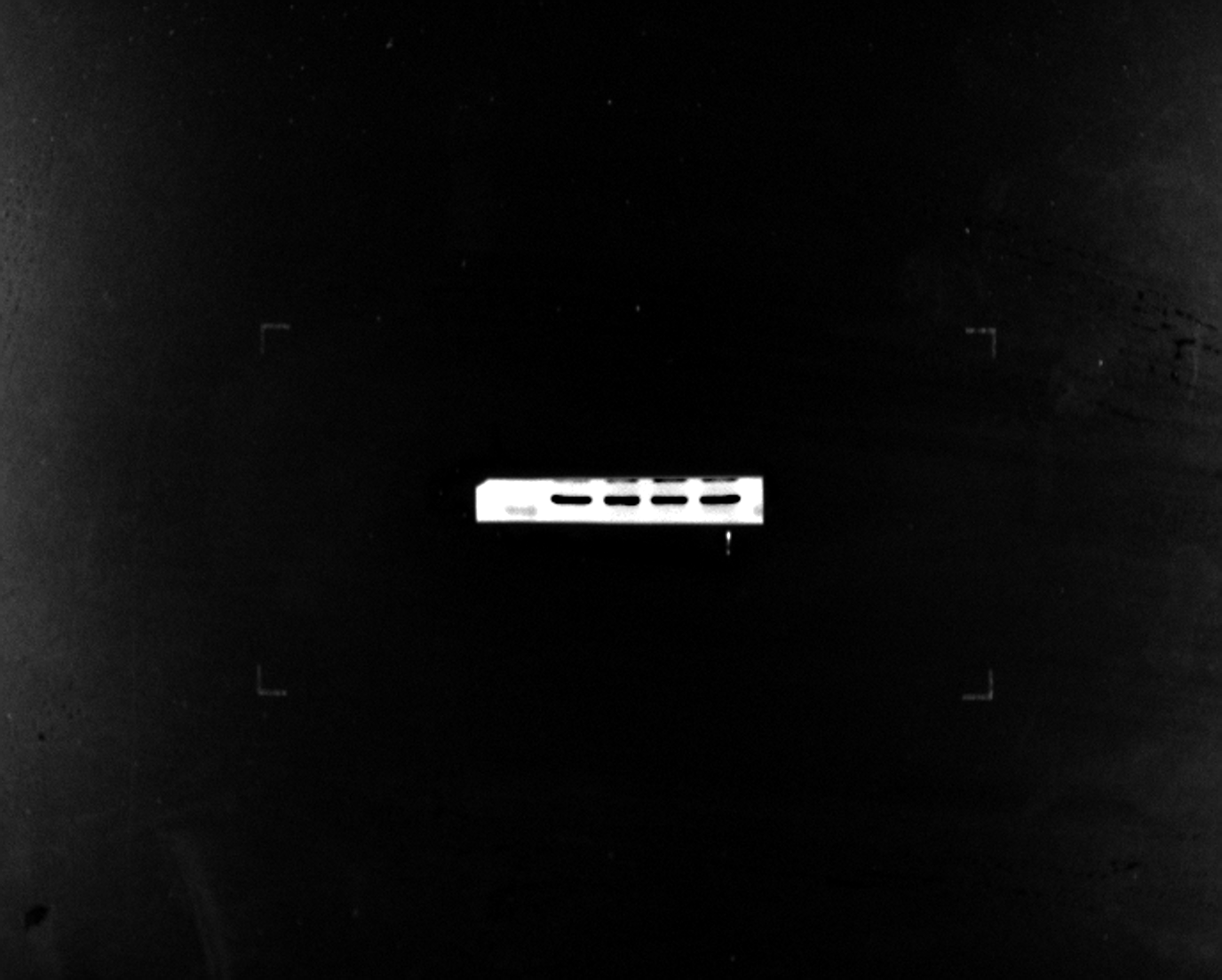

Supplement: Supplementary file 9 — Source data Fig. 4 [file 44318_2024_359_MOESM9_ESM.zip › Figure 4/Fig 4C/5-input-GAPDH-merge.Tif]

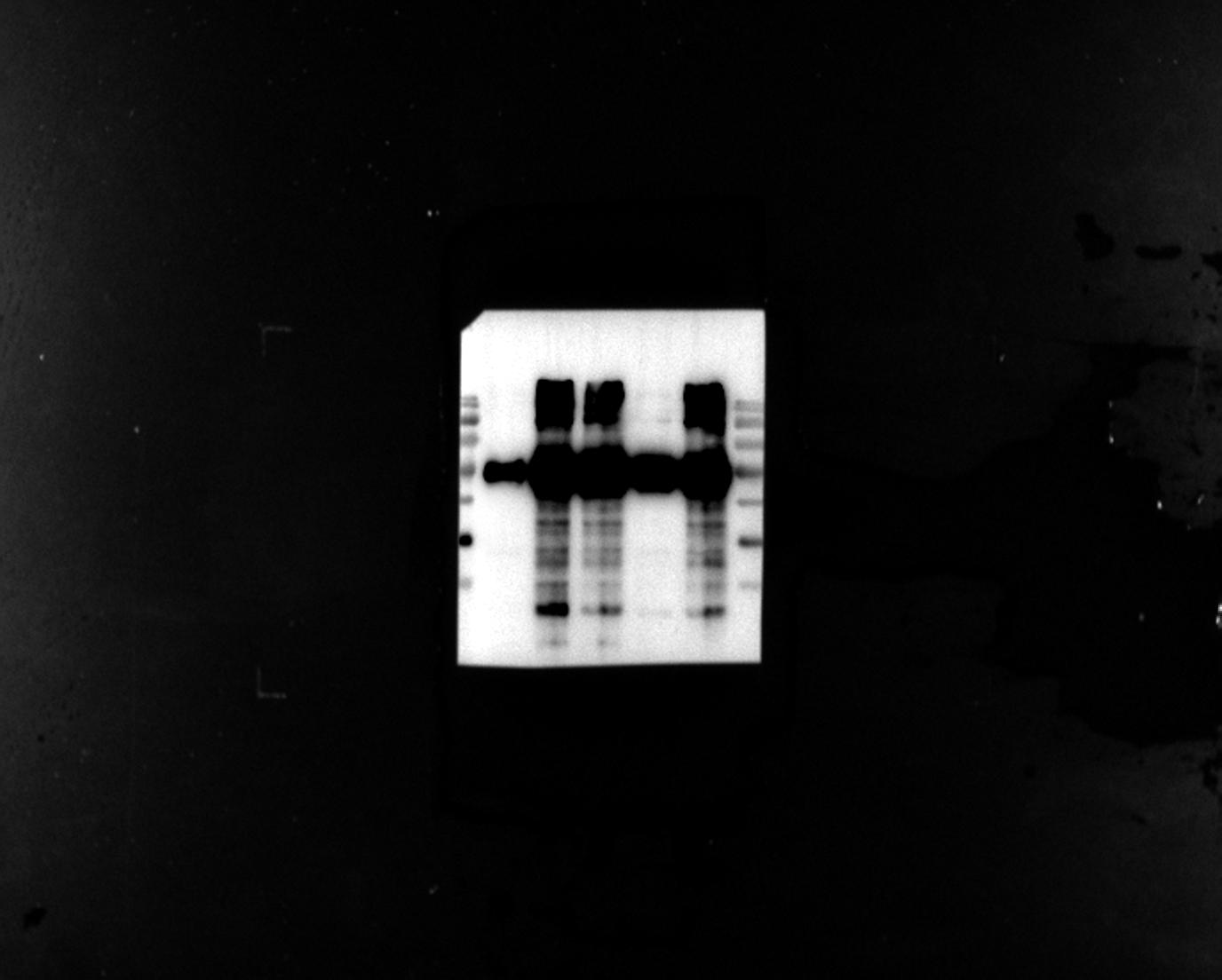

Supplement: Supplementary file 9 — Source data Fig. 4 [file 44318_2024_359_MOESM9_ESM.zip › Figure 4/Fig 4C/6-ip P27KIP1 wb ub-merge.Tif]

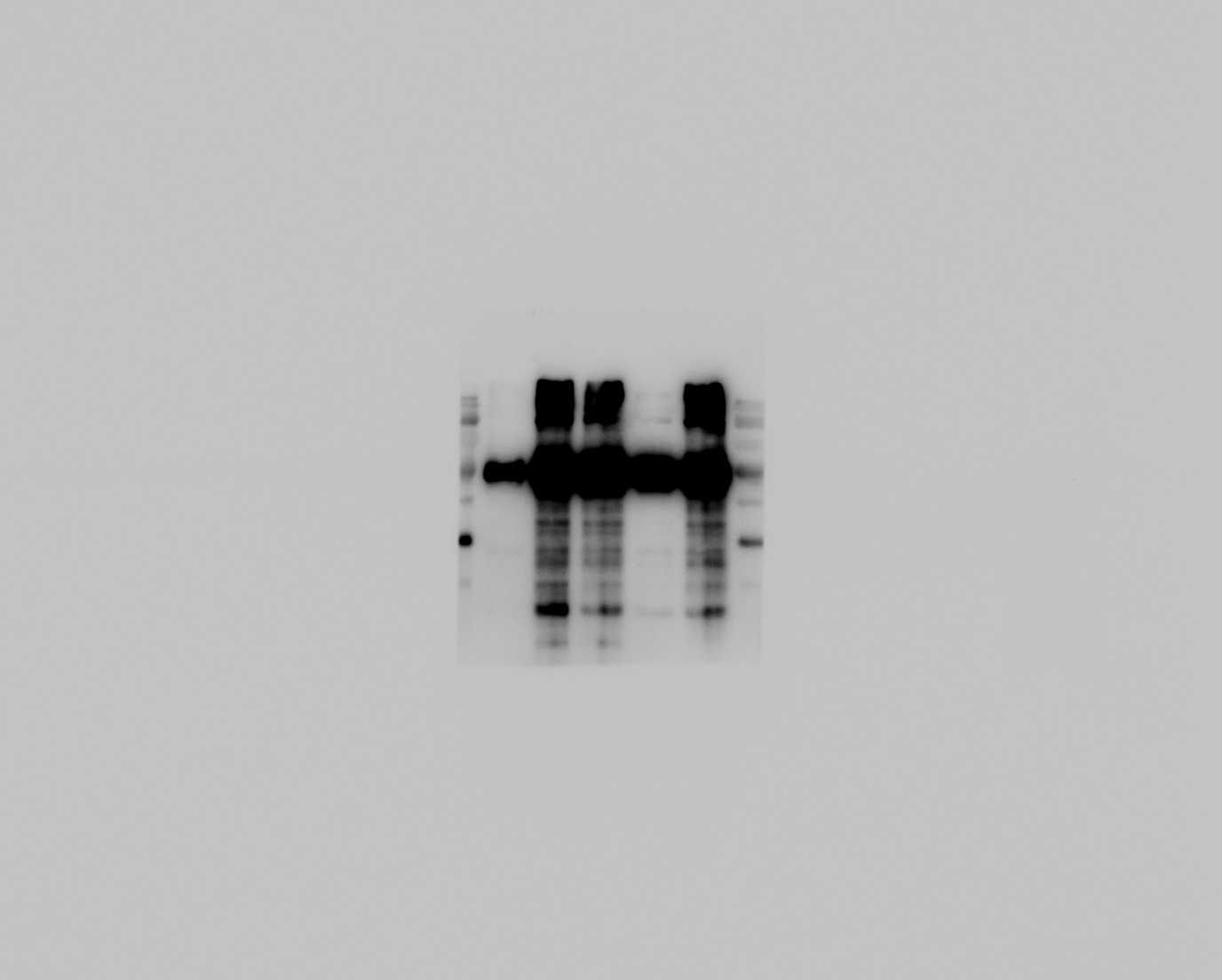

Supplement: Supplementary file 9 — Source data Fig. 4 [file 44318_2024_359_MOESM9_ESM.zip › Figure 4/Fig 4C/6-ip P27KIP1 wb ub.Tif]

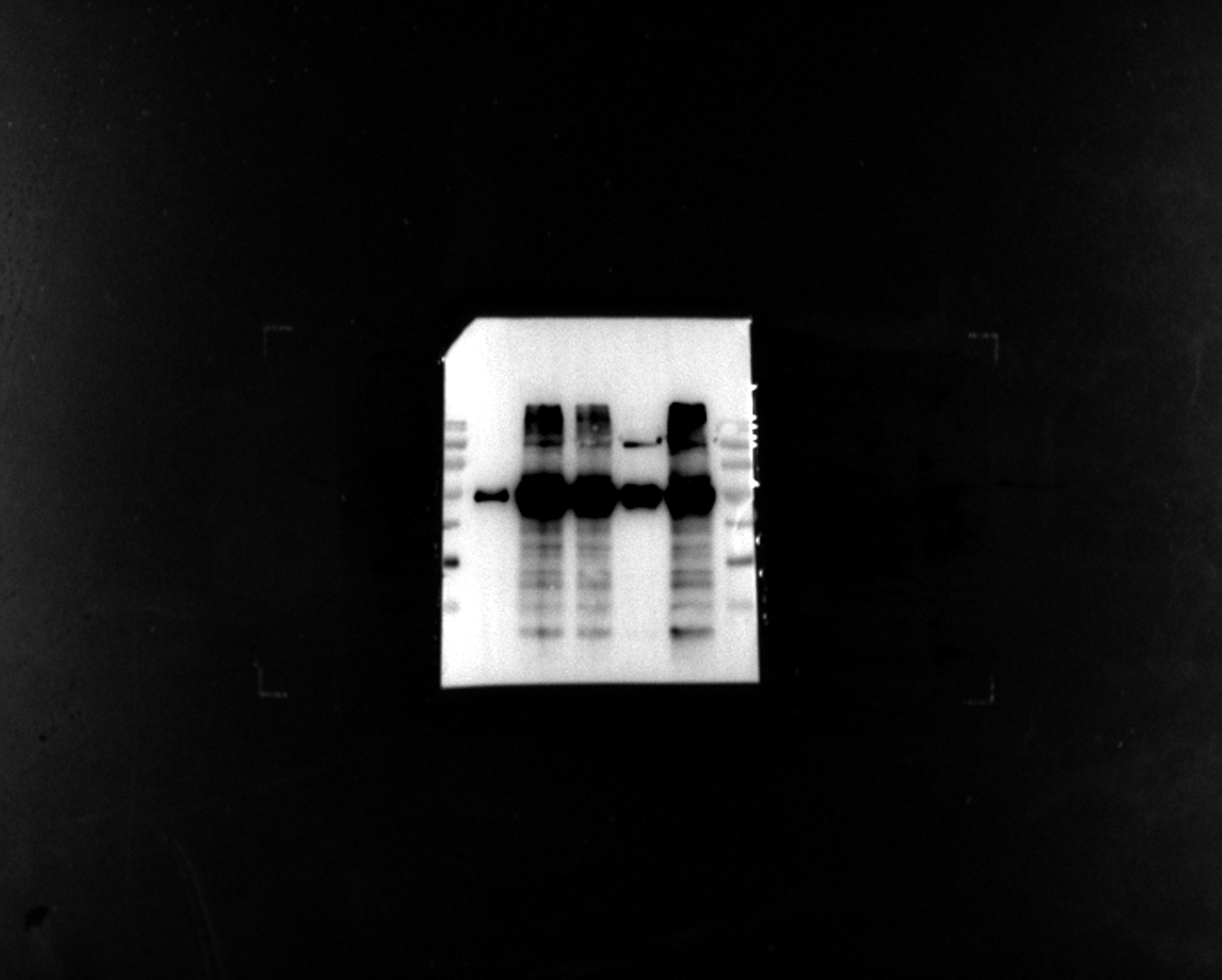

Supplement: Supplementary file 9 — Source data Fig. 4 [file 44318_2024_359_MOESM9_ESM.zip › Figure 4/Fig 4C/7-ip HA wb ub-merge.Tif]

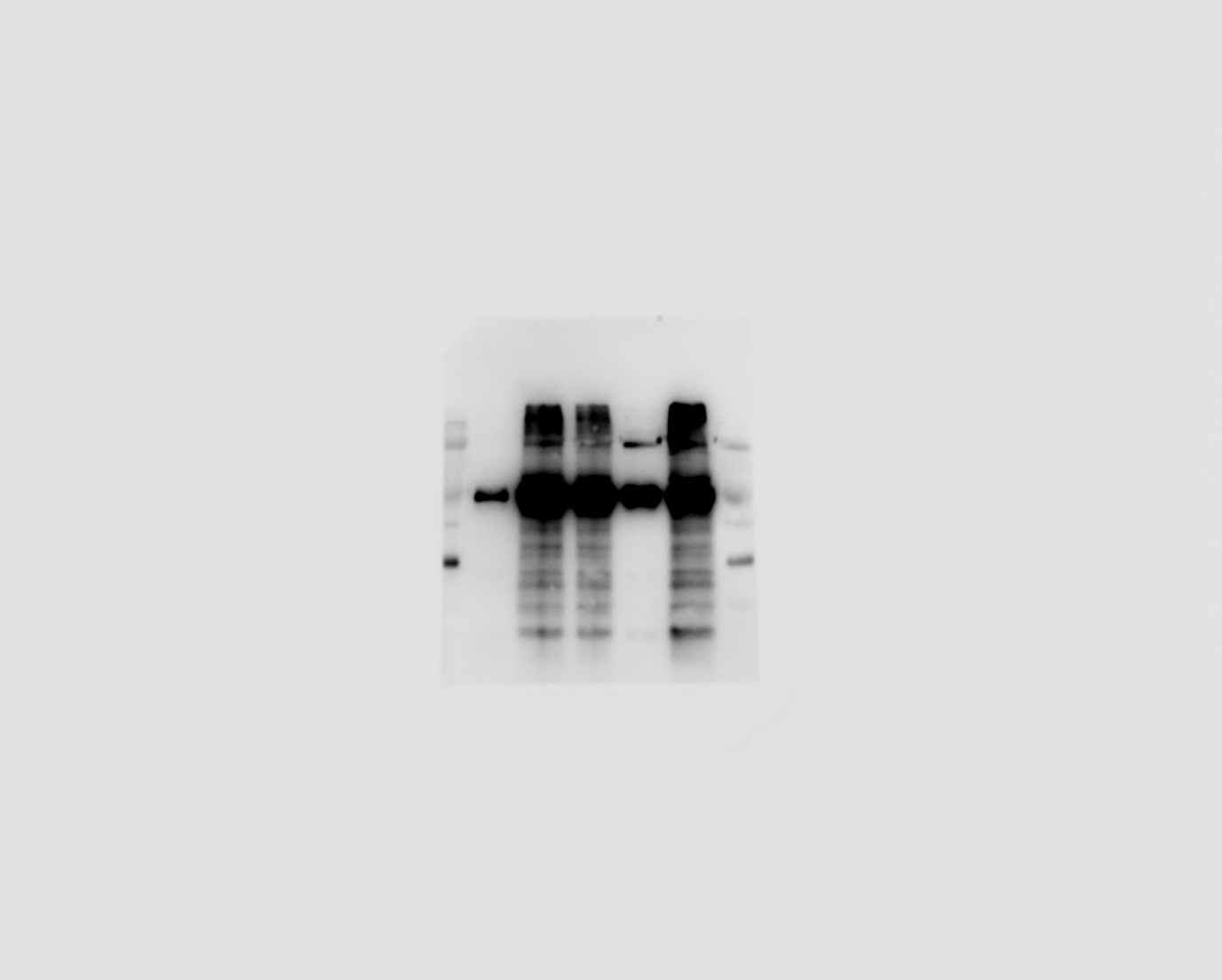

Supplement: Supplementary file 9 — Source data Fig. 4 [file 44318_2024_359_MOESM9_ESM.zip › Figure 4/Fig 4C/7-ip HA wb ub.Tif]

Fig 4C

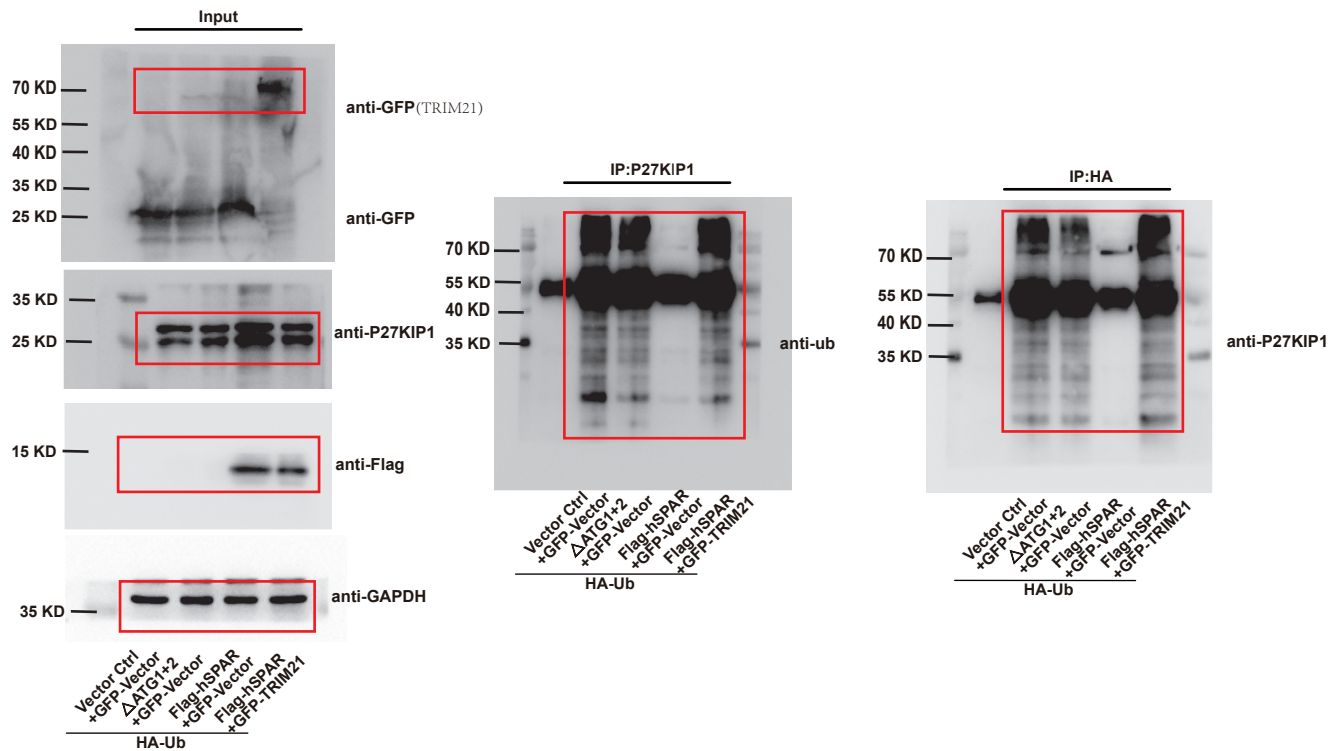

Supplement: Supplementary file 9 — Source data Fig. 4 [file 44318_2024_359_MOESM9_ESM.zip › Figure 4/Fig 4C/Fig 4C.pdf]

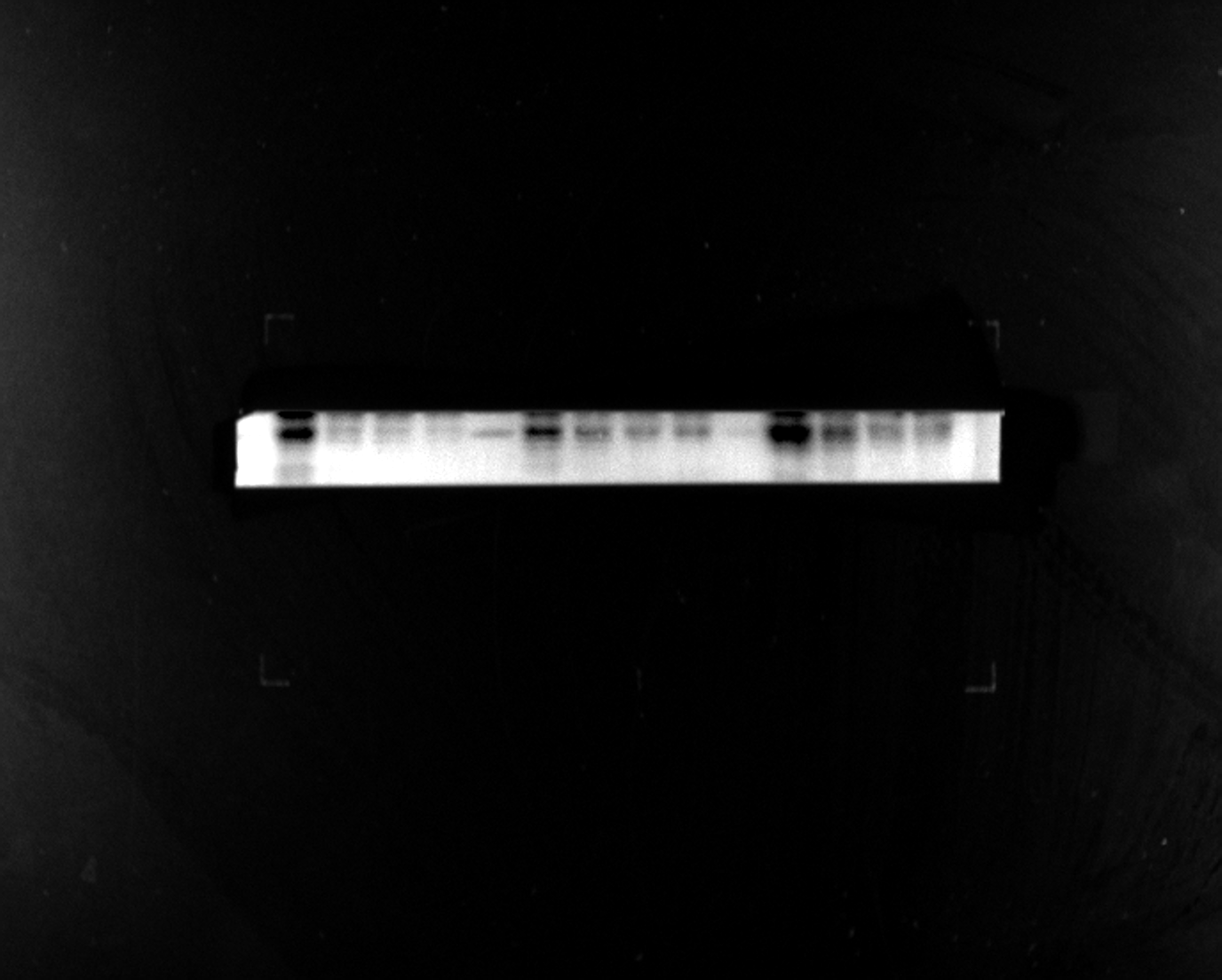

Supplement: Supplementary file 9 — Source data Fig. 4 [file 44318_2024_359_MOESM9_ESM.zip › Figure 4/Fig 4D and 4E/Fig 4D/1-p27-merge.Tif]

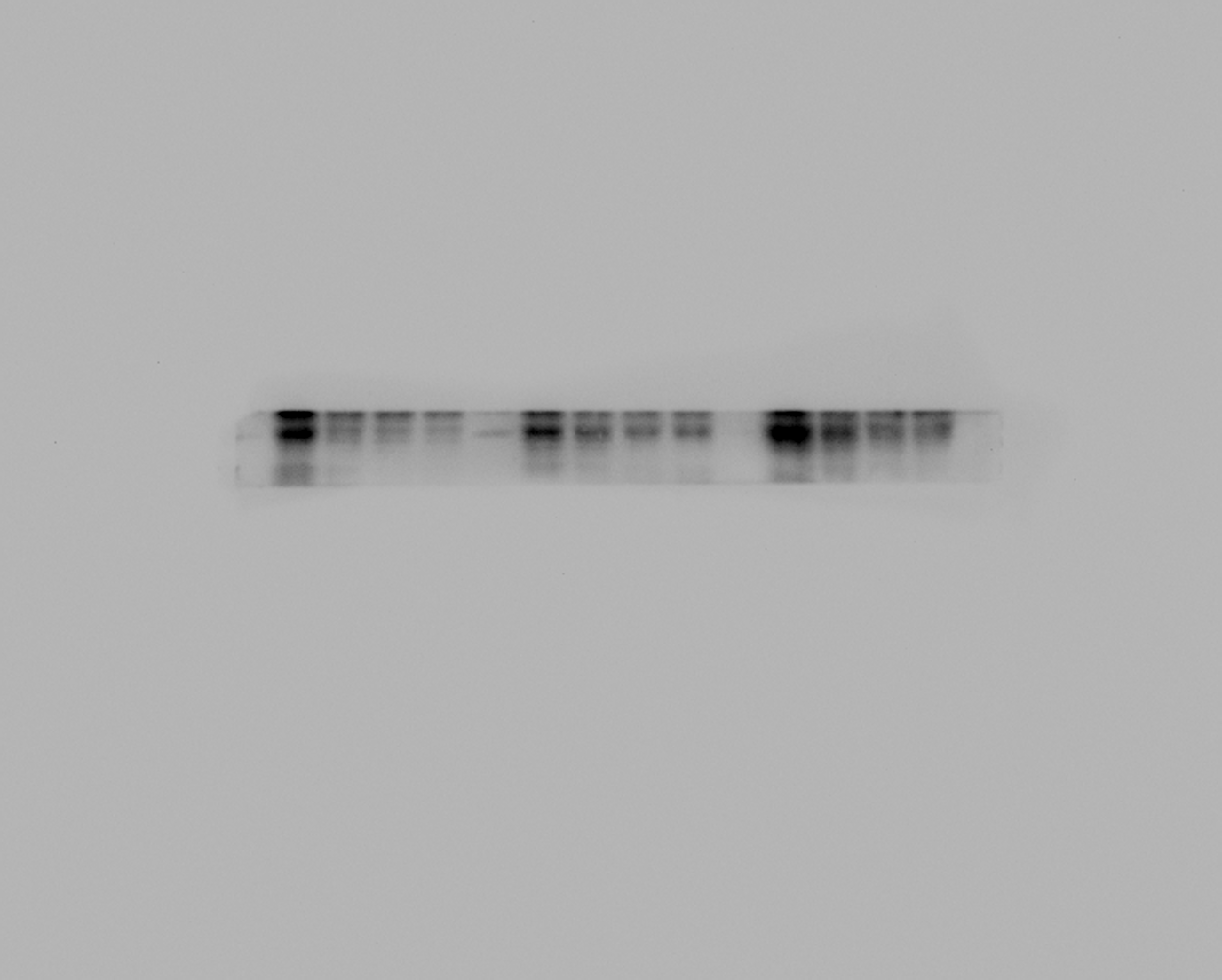

Supplement: Supplementary file 9 — Source data Fig. 4 [file 44318_2024_359_MOESM9_ESM.zip › Figure 4/Fig 4D and 4E/Fig 4D/1-p27.Tif]

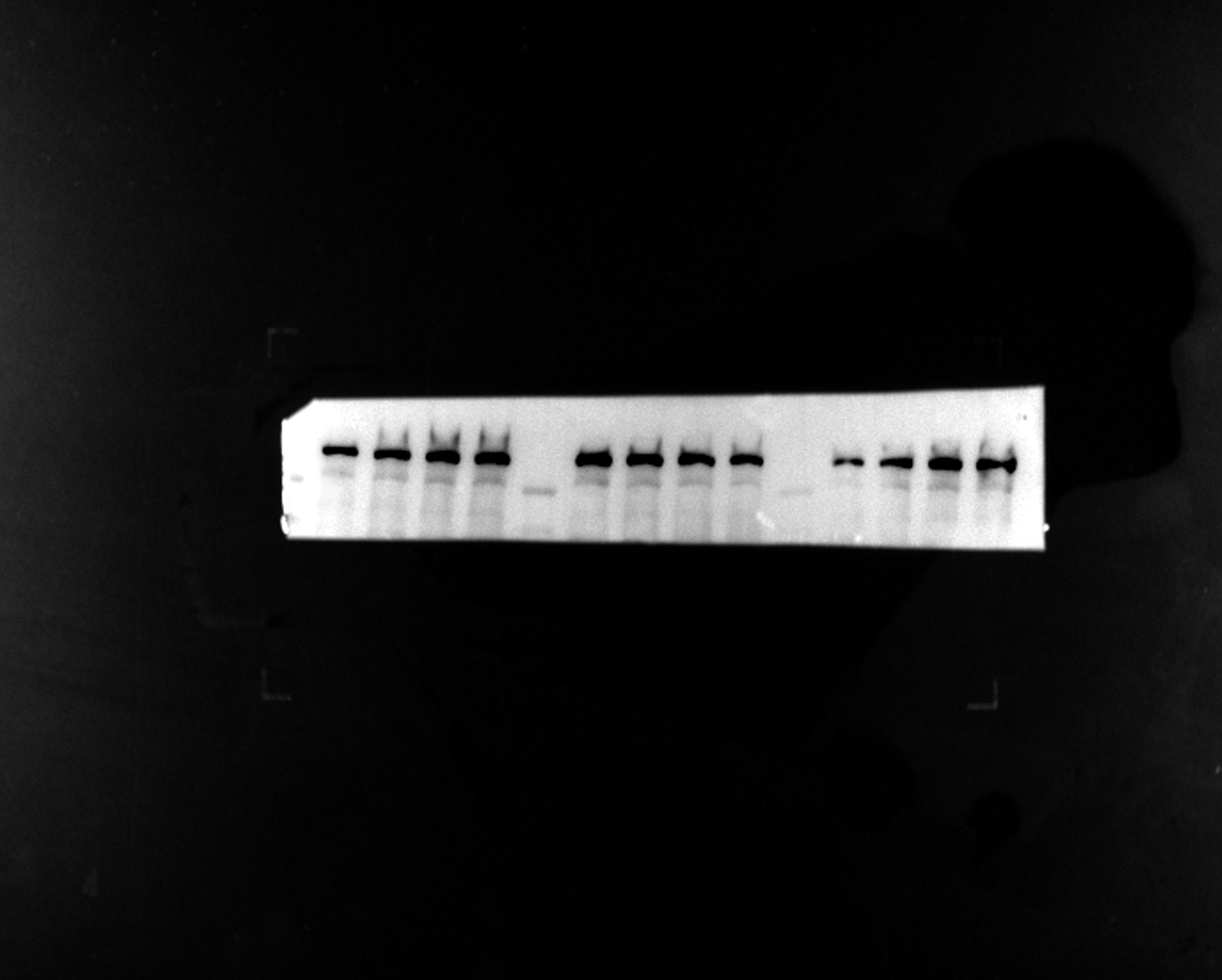

Supplement: Supplementary file 9 — Source data Fig. 4 [file 44318_2024_359_MOESM9_ESM.zip › Figure 4/Fig 4D and 4E/Fig 4D/2-p-mtor-merge.Tif]

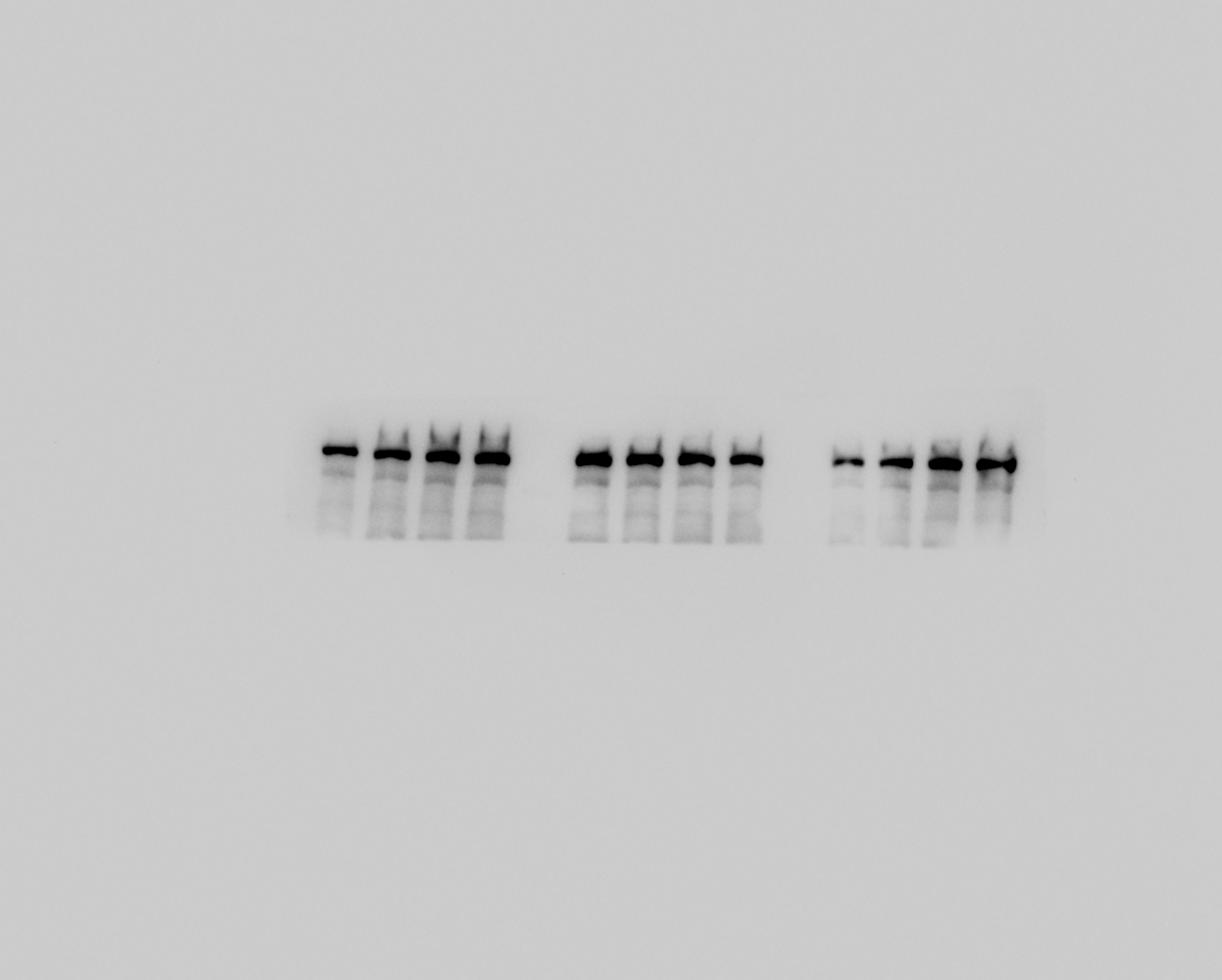

Supplement: Supplementary file 9 — Source data Fig. 4 [file 44318_2024_359_MOESM9_ESM.zip › Figure 4/Fig 4D and 4E/Fig 4D/2-p-mtor.Tif]

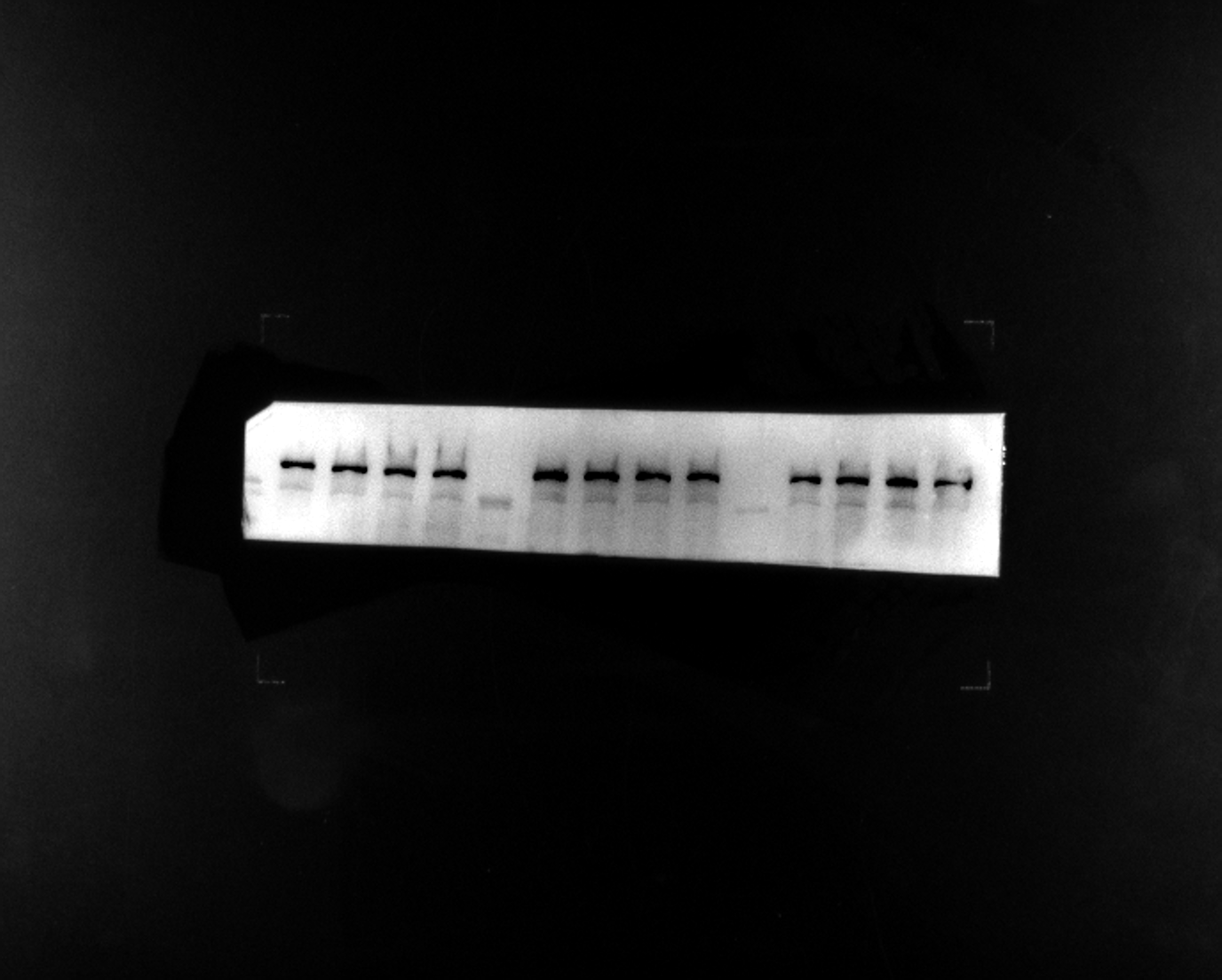

Supplement: Supplementary file 9 — Source data Fig. 4 [file 44318_2024_359_MOESM9_ESM.zip › Figure 4/Fig 4D and 4E/Fig 4D/3-mTOR-merge.Tif]

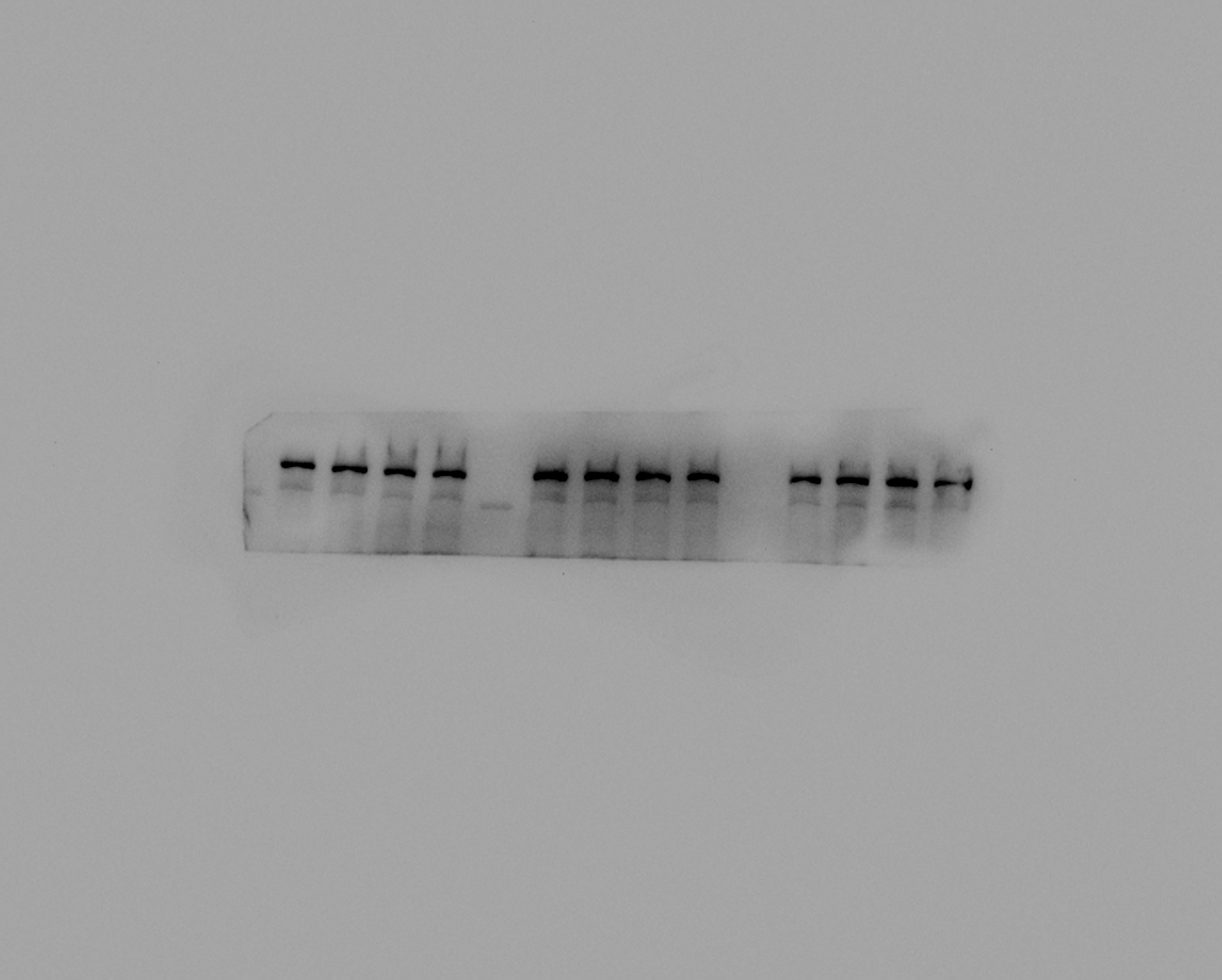

Supplement: Supplementary file 9 — Source data Fig. 4 [file 44318_2024_359_MOESM9_ESM.zip › Figure 4/Fig 4D and 4E/Fig 4D/3-mTOR.Tif]

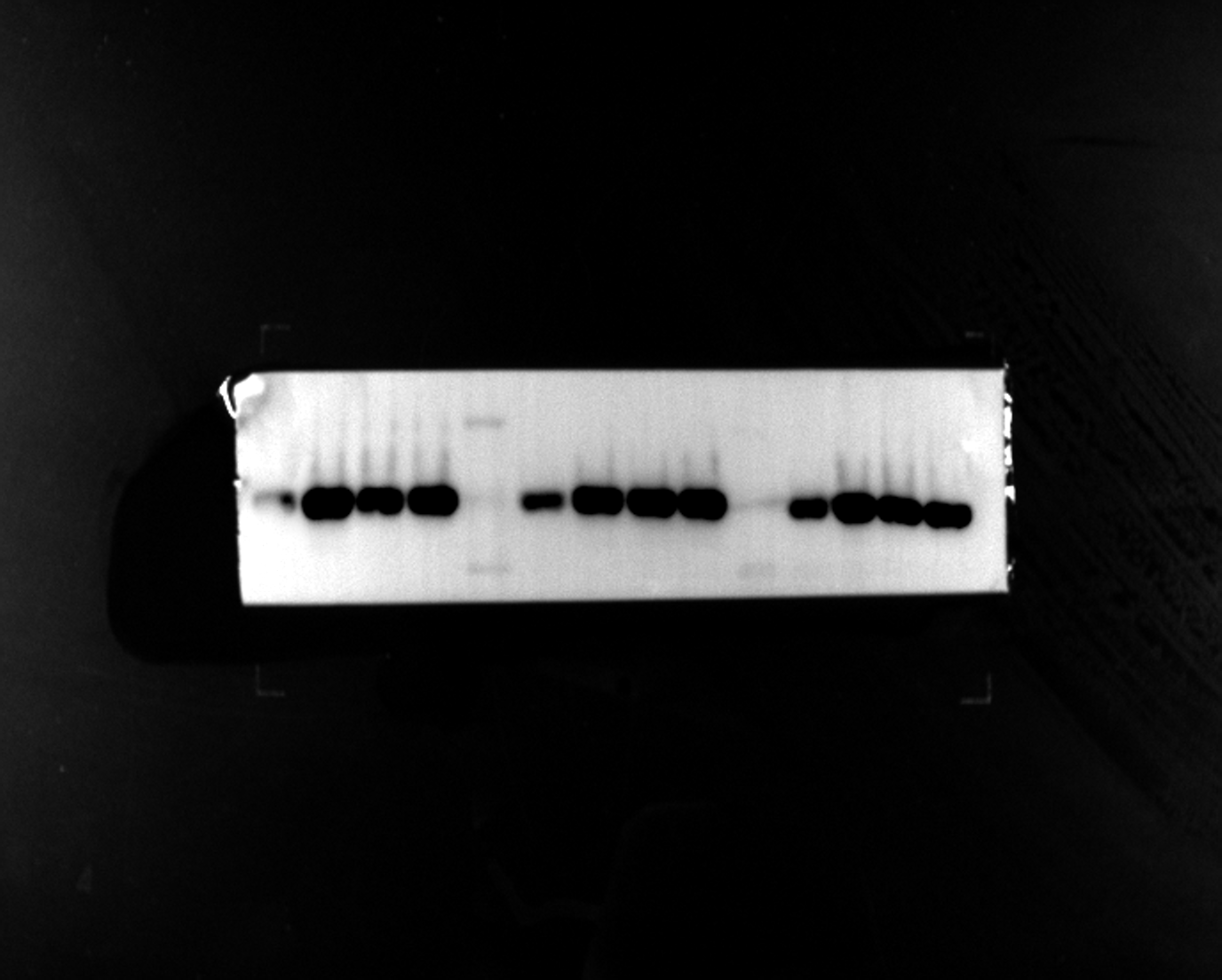

Supplement: Supplementary file 9 — Source data Fig. 4 [file 44318_2024_359_MOESM9_ESM.zip › Figure 4/Fig 4D and 4E/Fig 4D/4-p-S6K-merge.Tif]

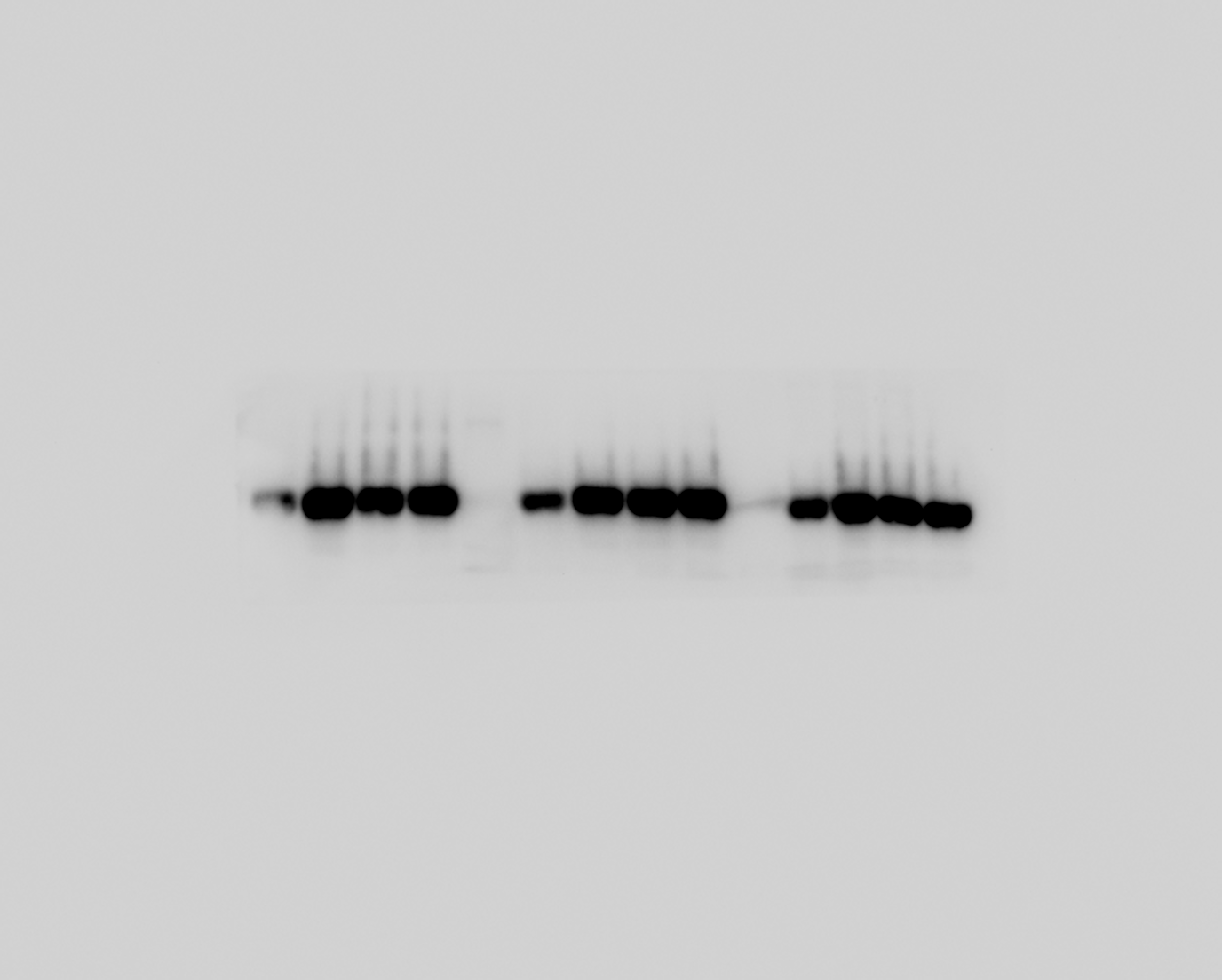

Supplement: Supplementary file 9 — Source data Fig. 4 [file 44318_2024_359_MOESM9_ESM.zip › Figure 4/Fig 4D and 4E/Fig 4D/4-p-S6K.Tif]

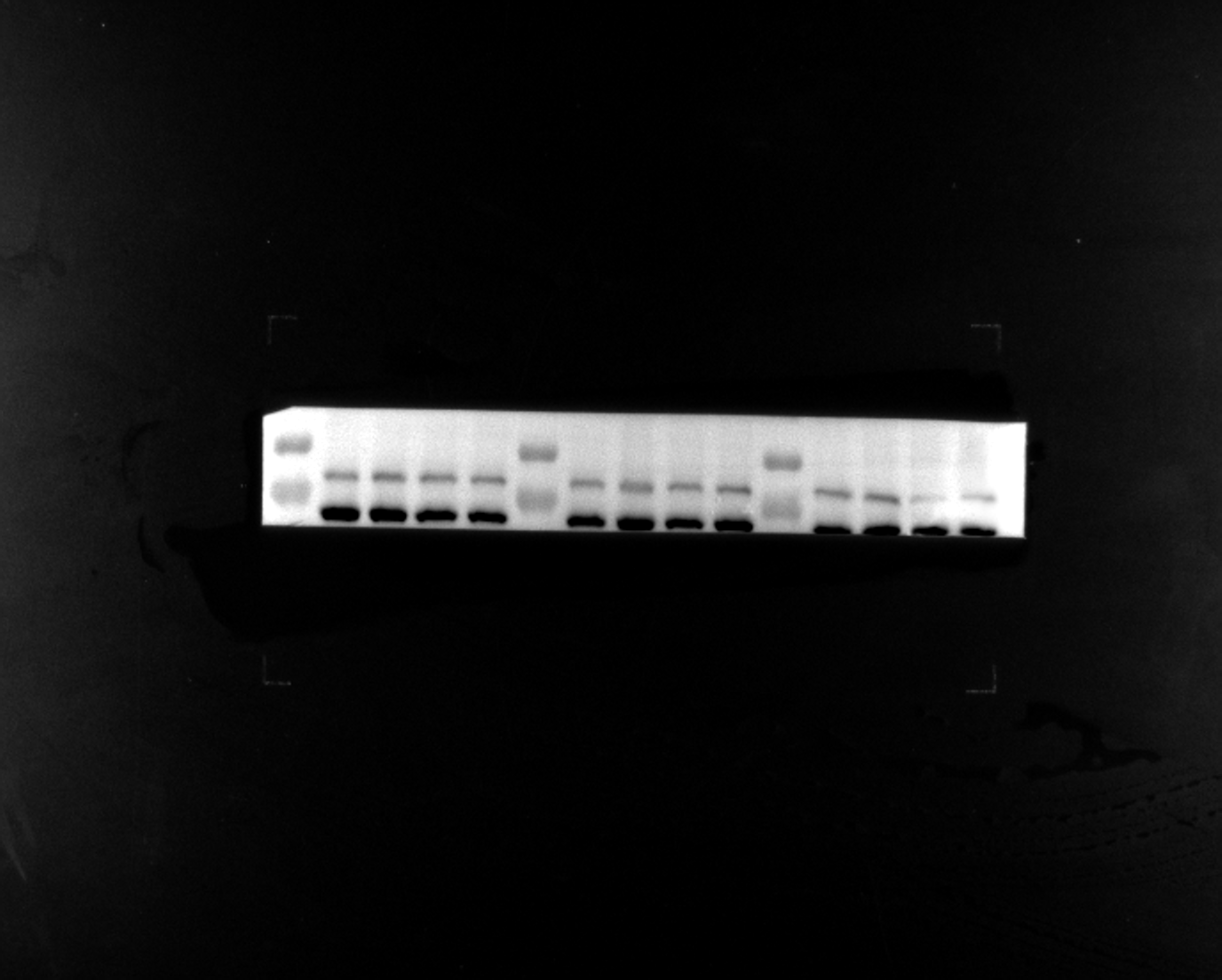

Supplement: Supplementary file 9 — Source data Fig. 4 [file 44318_2024_359_MOESM9_ESM.zip › Figure 4/Fig 4D and 4E/Fig 4D/5-S6K-merge.Tif]

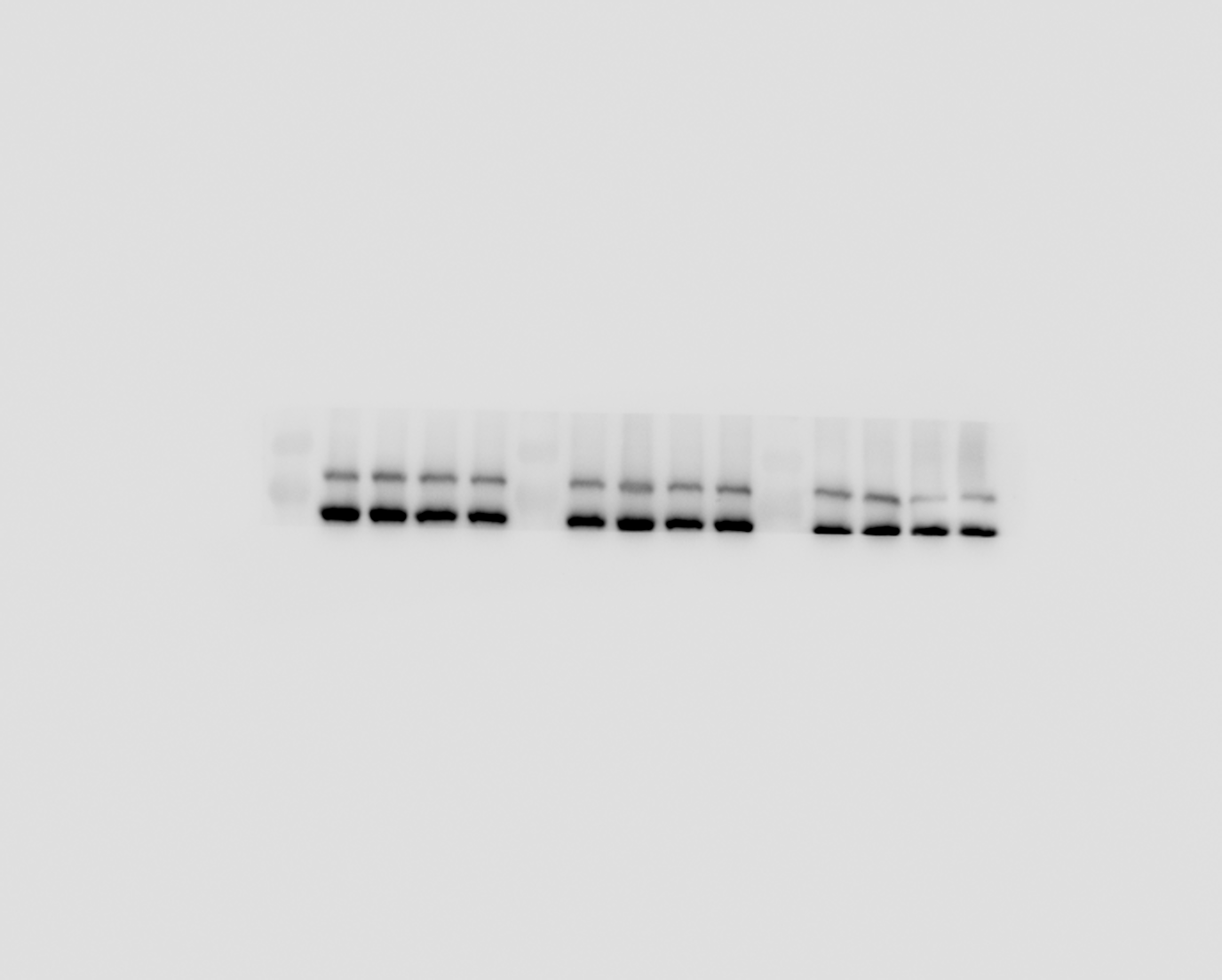

Supplement: Supplementary file 9 — Source data Fig. 4 [file 44318_2024_359_MOESM9_ESM.zip › Figure 4/Fig 4D and 4E/Fig 4D/5-S6K.Tif]

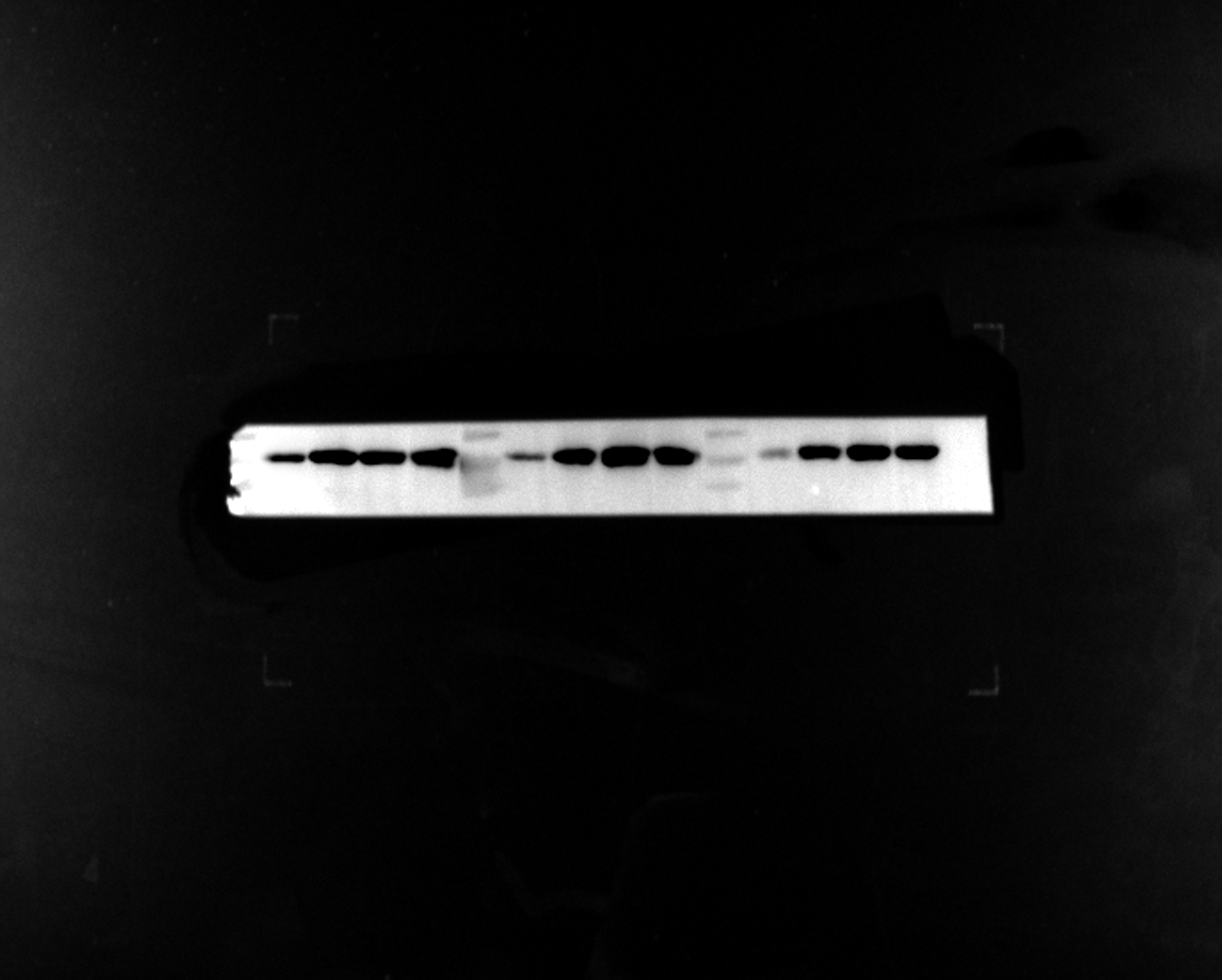

Supplement: Supplementary file 9 — Source data Fig. 4 [file 44318_2024_359_MOESM9_ESM.zip › Figure 4/Fig 4D and 4E/Fig 4D/6-p-S6-merge.Tif]

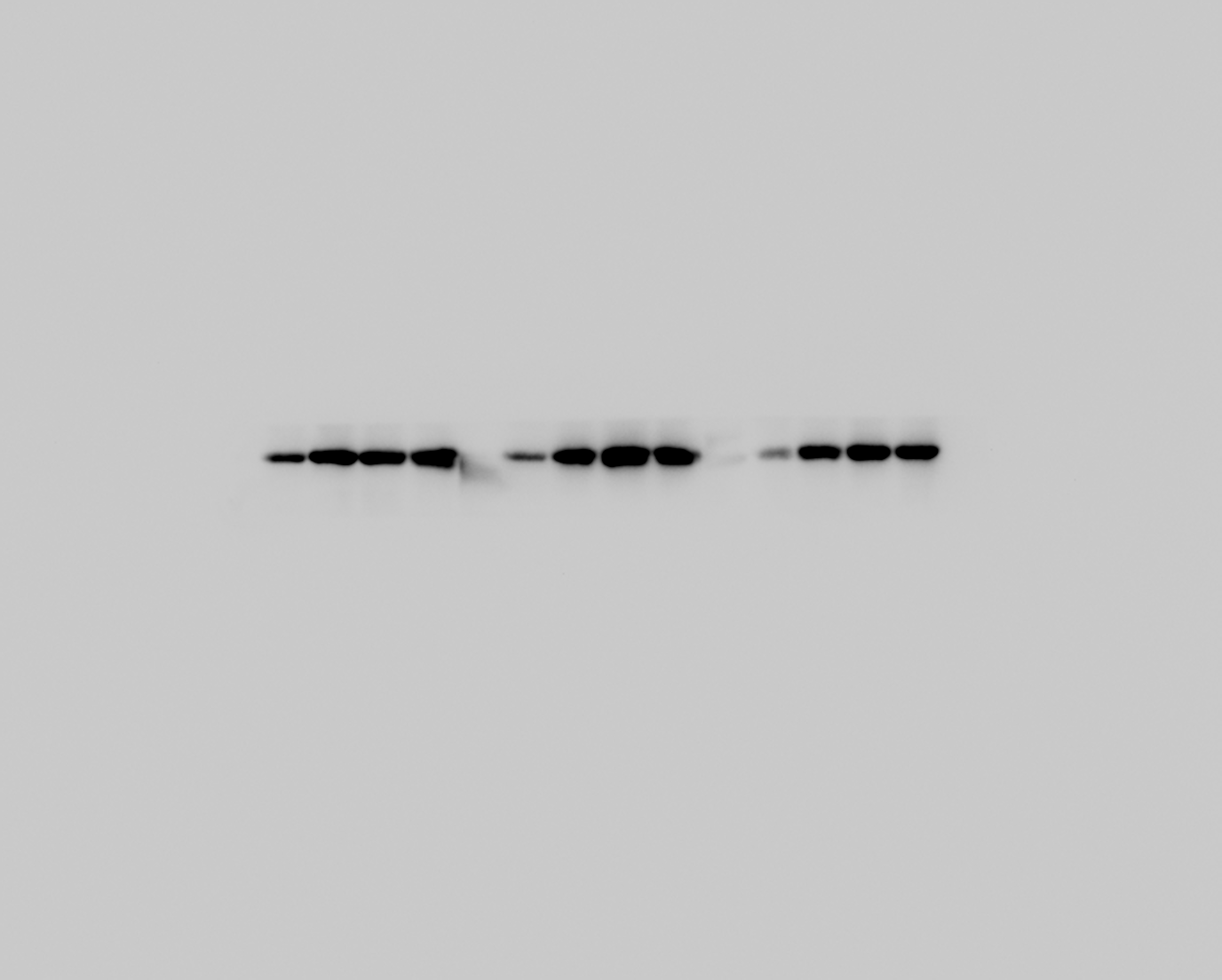

Supplement: Supplementary file 9 — Source data Fig. 4 [file 44318_2024_359_MOESM9_ESM.zip › Figure 4/Fig 4D and 4E/Fig 4D/6-p-S6.Tif]

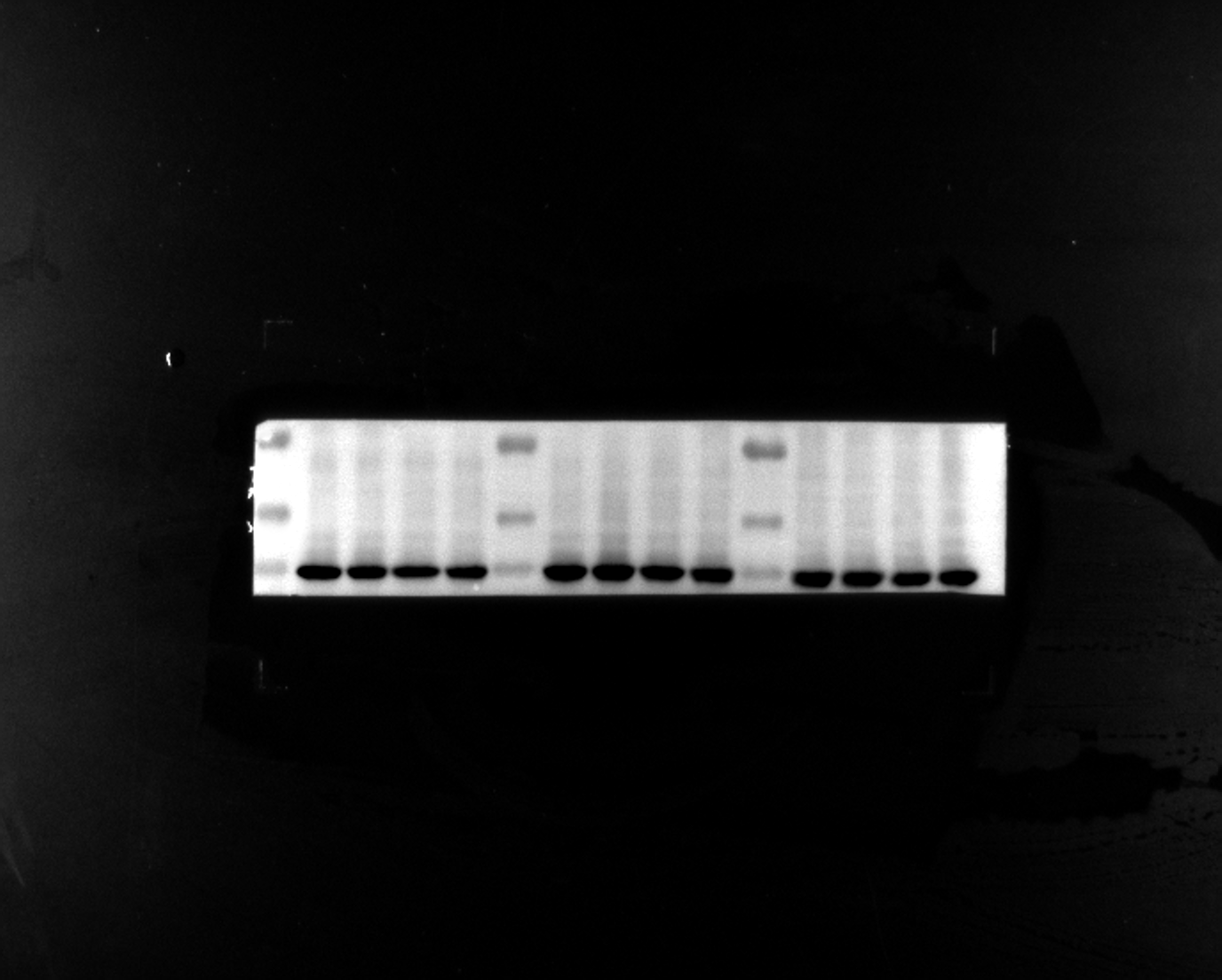

Supplement: Supplementary file 9 — Source data Fig. 4 [file 44318_2024_359_MOESM9_ESM.zip › Figure 4/Fig 4D and 4E/Fig 4D/7-S6-merge.Tif]

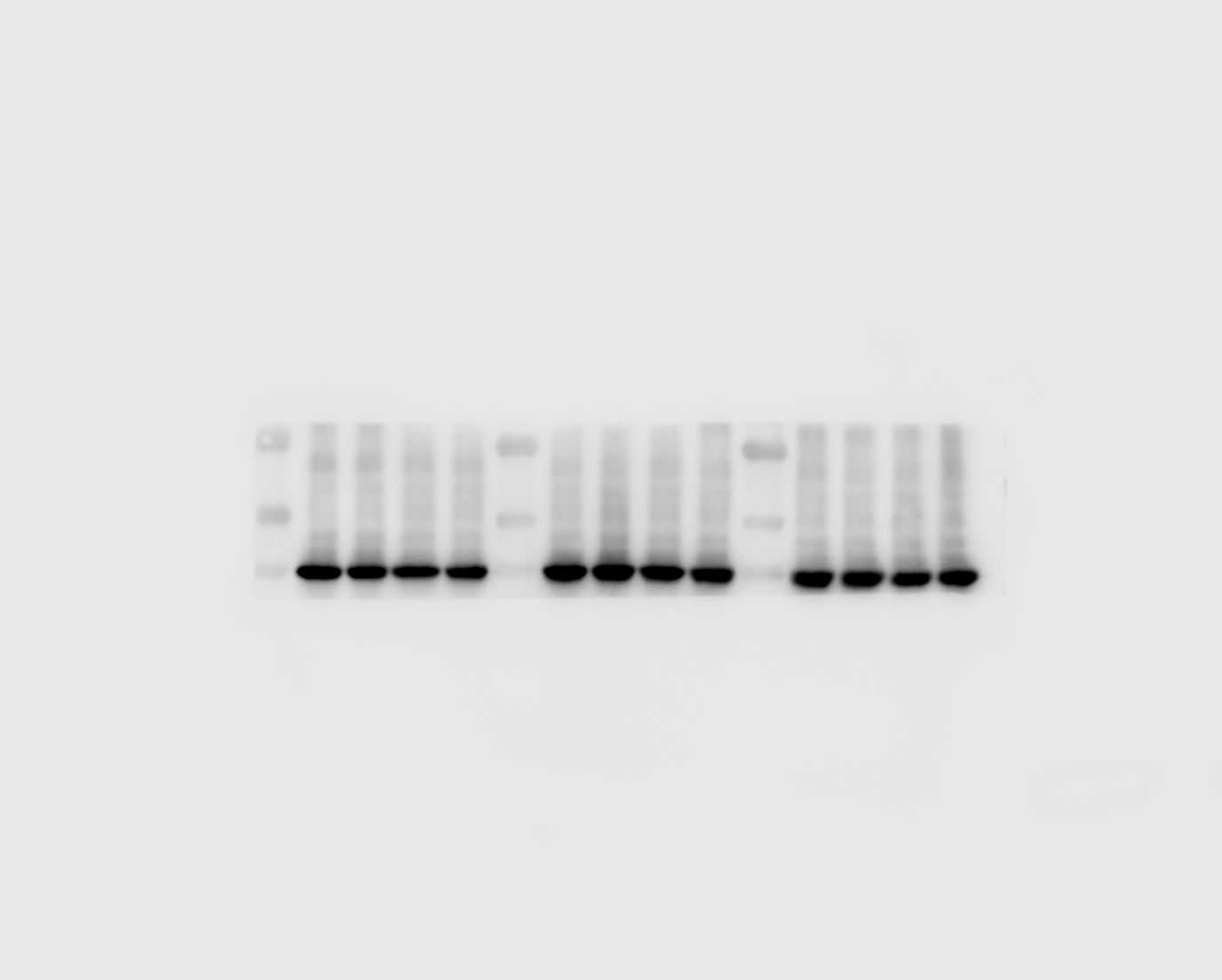

Supplement: Supplementary file 9 — Source data Fig. 4 [file 44318_2024_359_MOESM9_ESM.zip › Figure 4/Fig 4D and 4E/Fig 4D/7-S6.Tif]

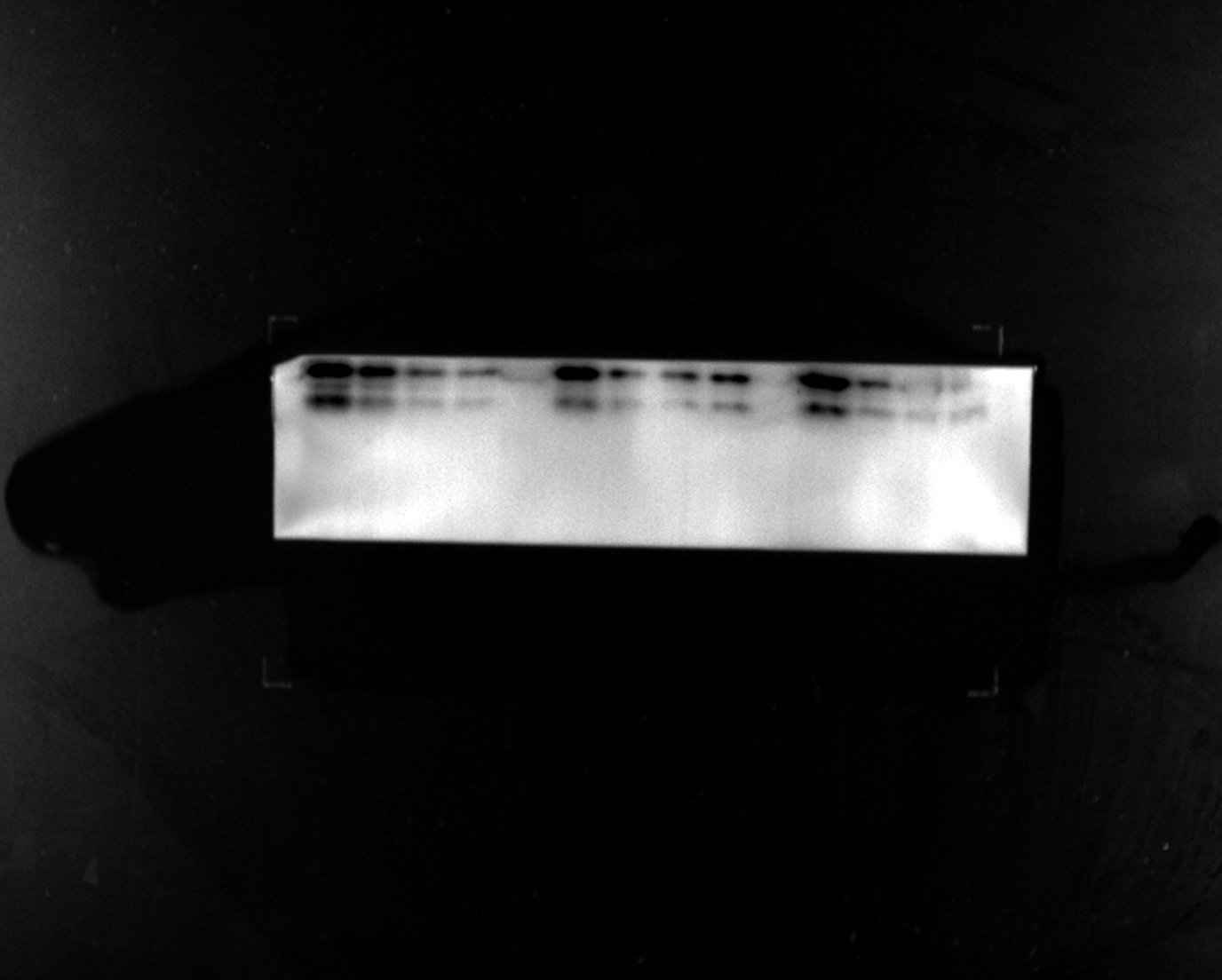

Supplement: Supplementary file 9 — Source data Fig. 4 [file 44318_2024_359_MOESM9_ESM.zip › Figure 4/Fig 4D and 4E/Fig 4D/8-hSPAR-merge .Tif]

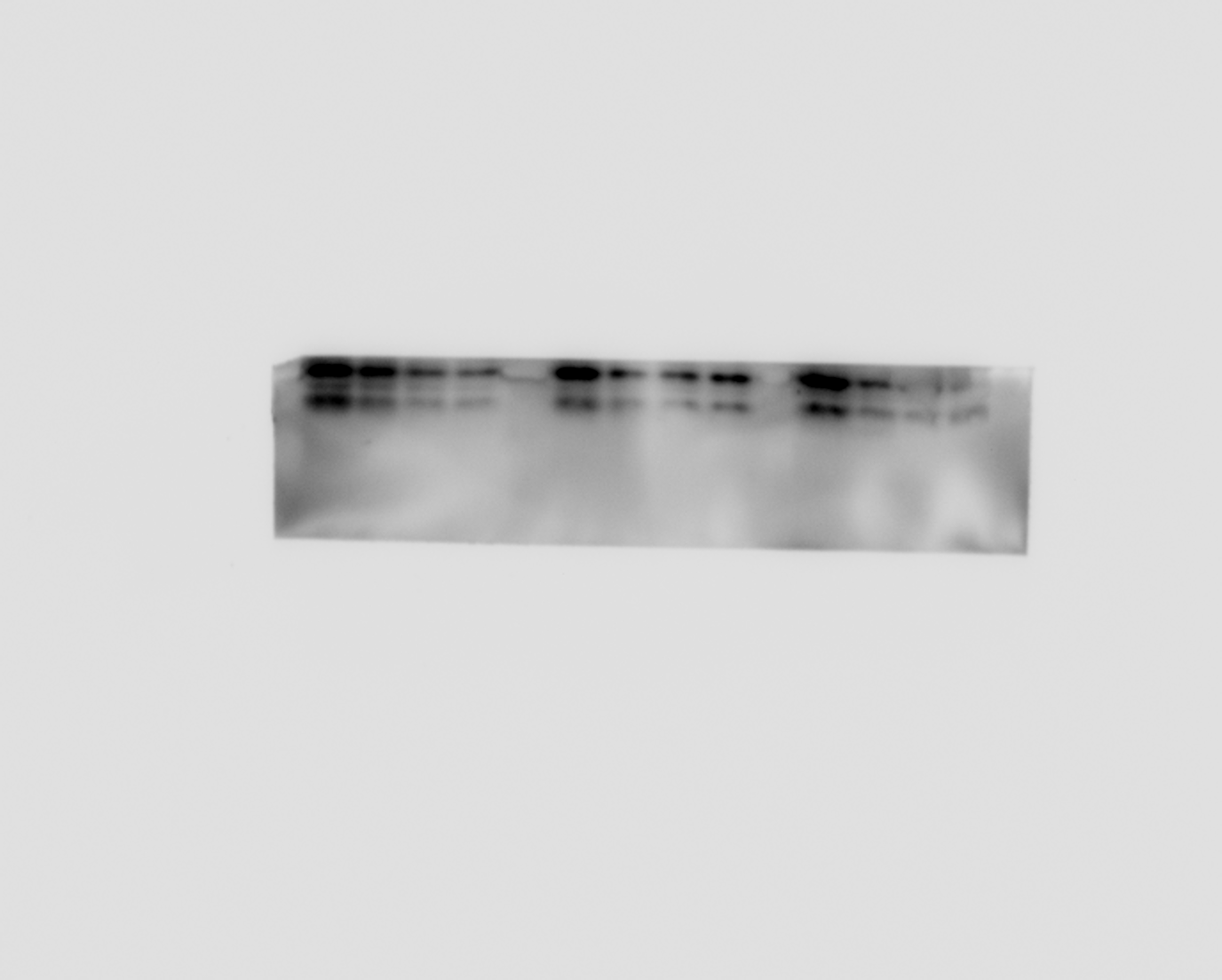

Supplement: Supplementary file 9 — Source data Fig. 4 [file 44318_2024_359_MOESM9_ESM.zip › Figure 4/Fig 4D and 4E/Fig 4D/8-hSPAR.Tif]

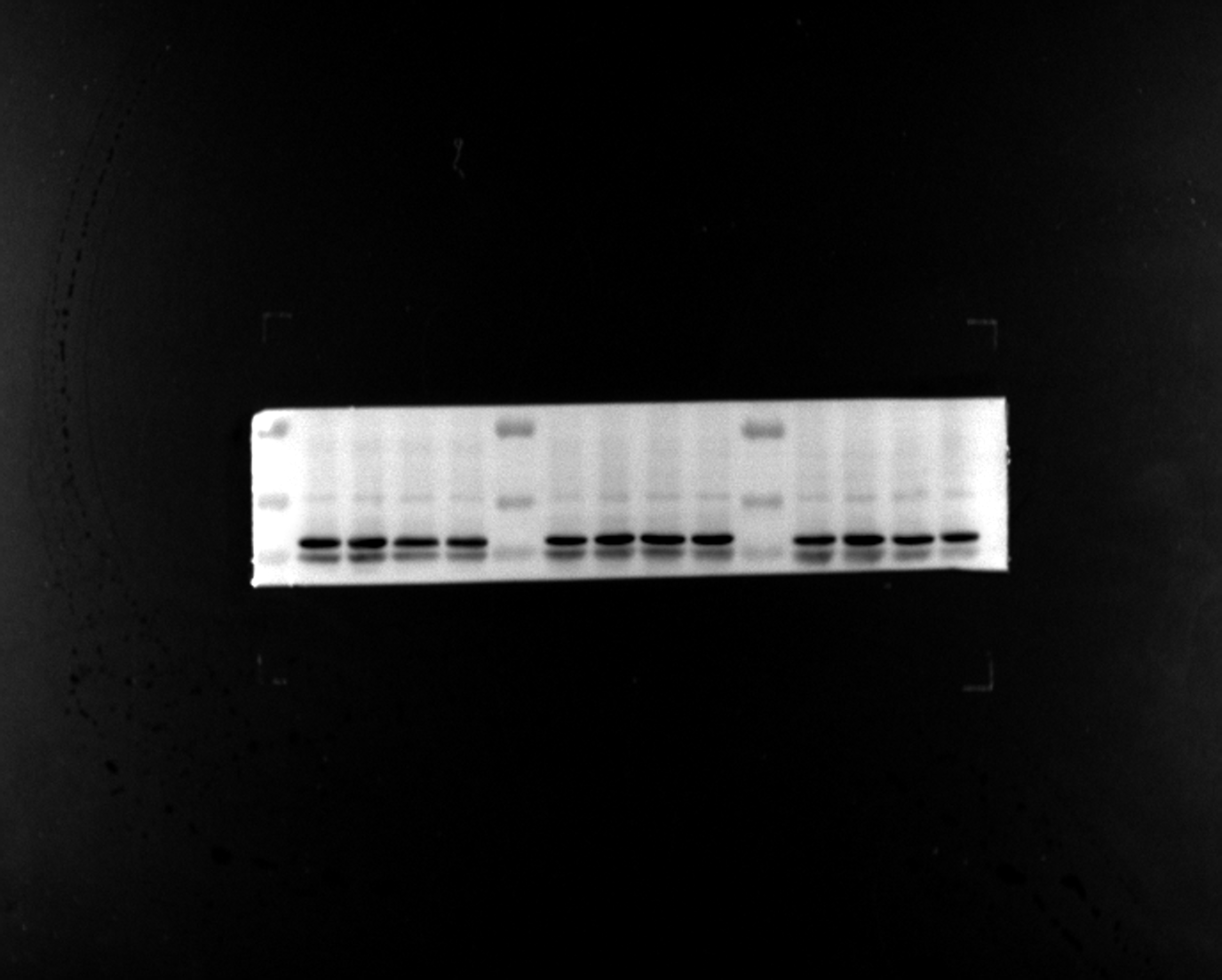

Supplement: Supplementary file 9 — Source data Fig. 4 [file 44318_2024_359_MOESM9_ESM.zip › Figure 4/Fig 4D and 4E/Fig 4D/9-GAPDH-merge.Tif]

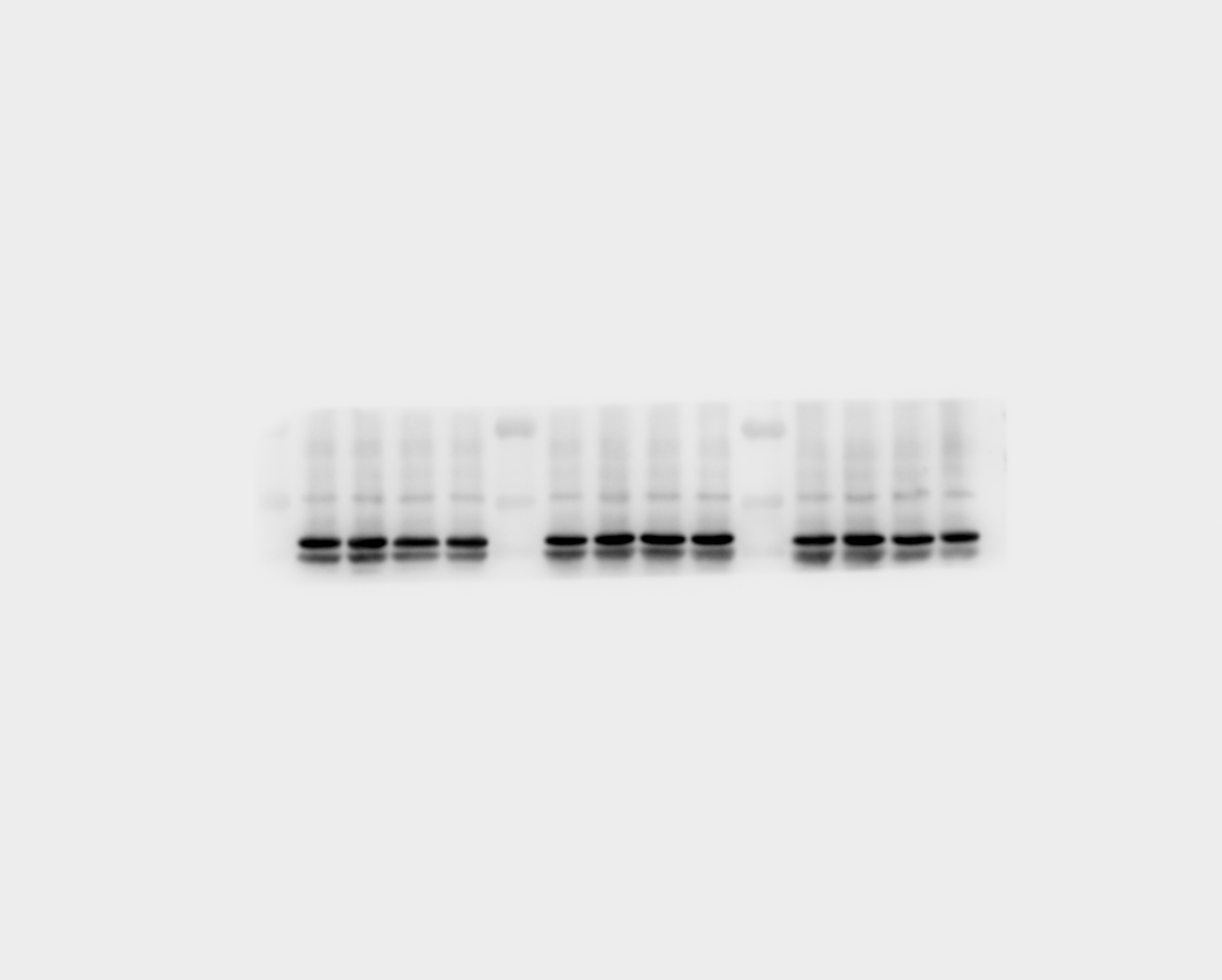

Supplement: Supplementary file 9 — Source data Fig. 4 [file 44318_2024_359_MOESM9_ESM.zip › Figure 4/Fig 4D and 4E/Fig 4D/9-GAPDH.Tif]

Fig 4D

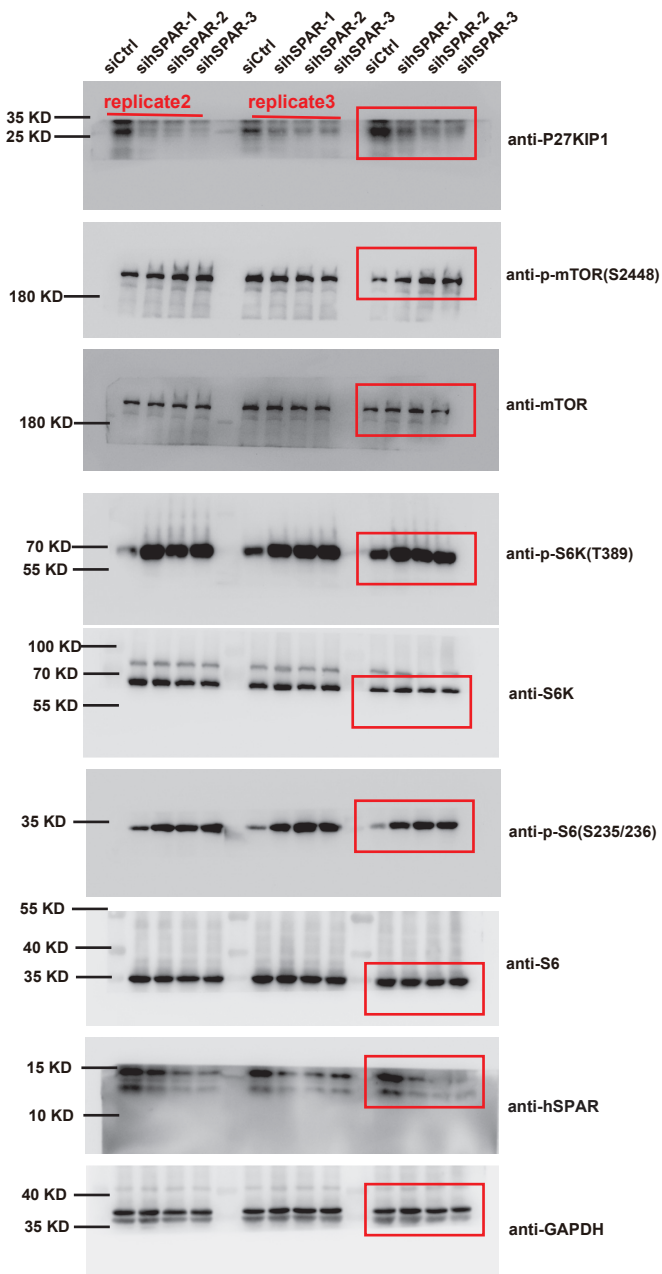

Supplement: Supplementary file 9 — Source data Fig. 4 [file 44318_2024_359_MOESM9_ESM.zip › Figure 4/Fig 4D and 4E/Fig 4D/Fig 4D.pdf]

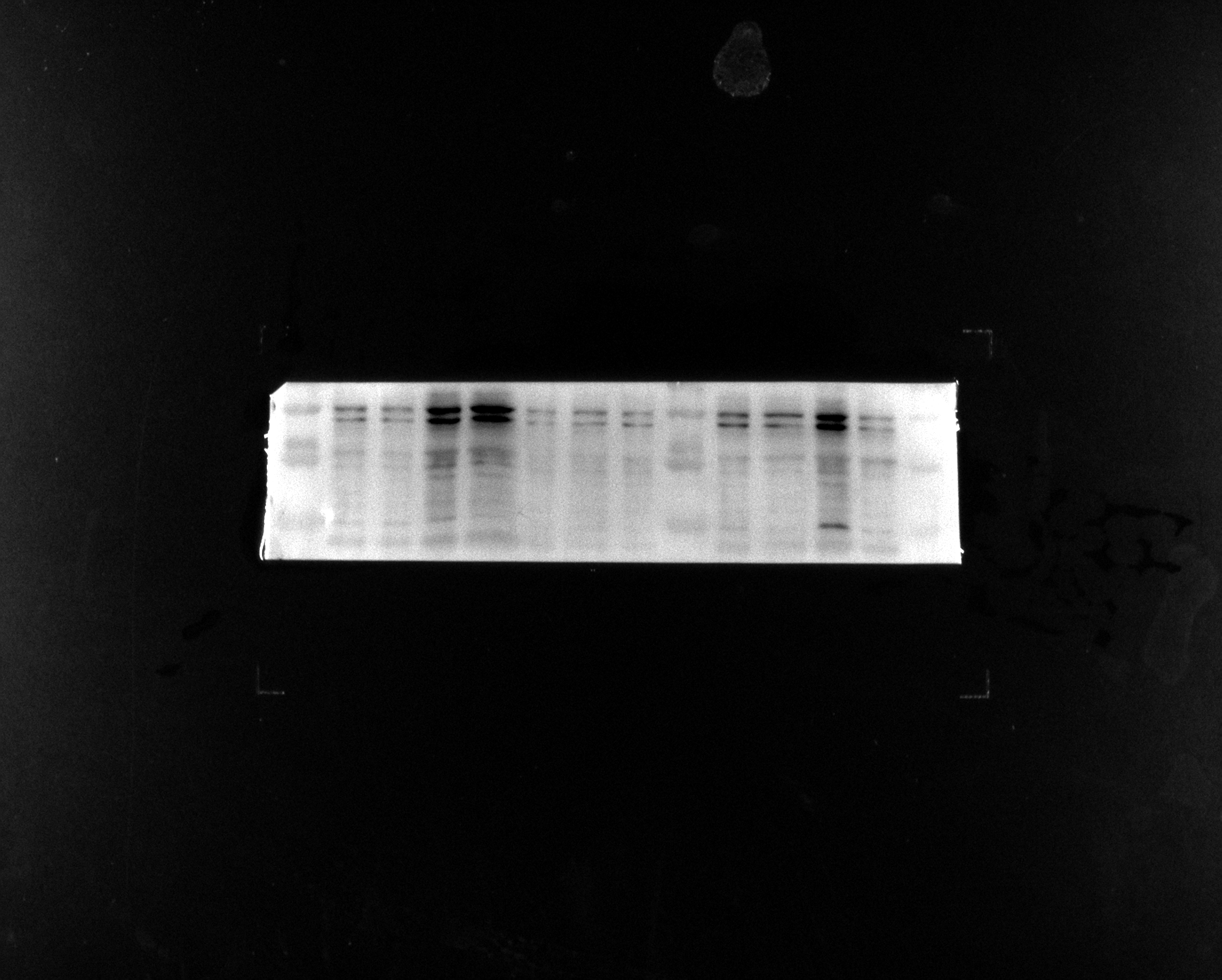

Supplement: Supplementary file 9 — Source data Fig. 4 [file 44318_2024_359_MOESM9_ESM.zip › Figure 4/Fig 4F and 4G/Fig 4F/1-p27-merge.Tif]

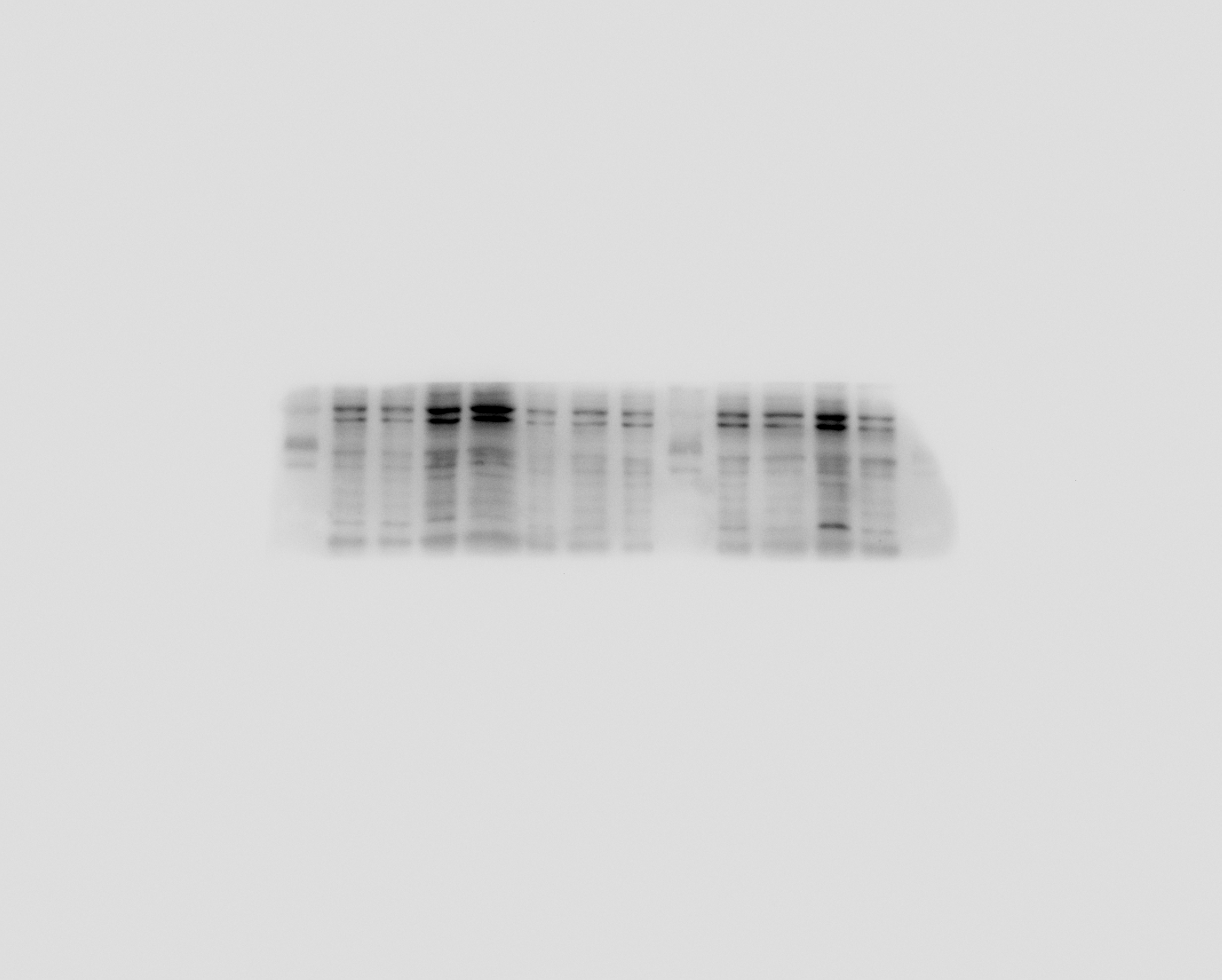

Supplement: Supplementary file 9 — Source data Fig. 4 [file 44318_2024_359_MOESM9_ESM.zip › Figure 4/Fig 4F and 4G/Fig 4F/1-p27.Tif]

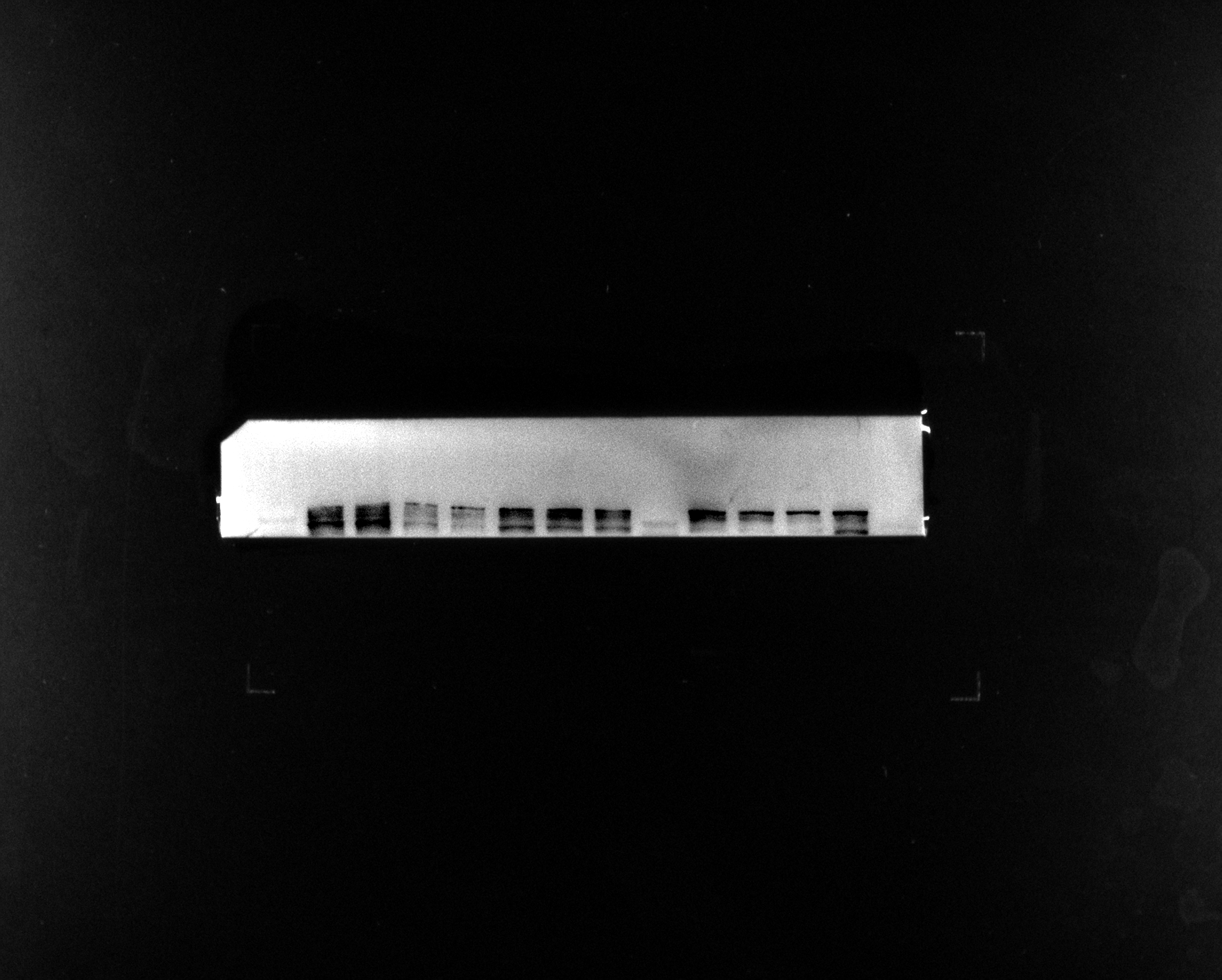

Supplement: Supplementary file 9 — Source data Fig. 4 [file 44318_2024_359_MOESM9_ESM.zip › Figure 4/Fig 4F and 4G/Fig 4F/2-p-mTOR-merge.Tif]

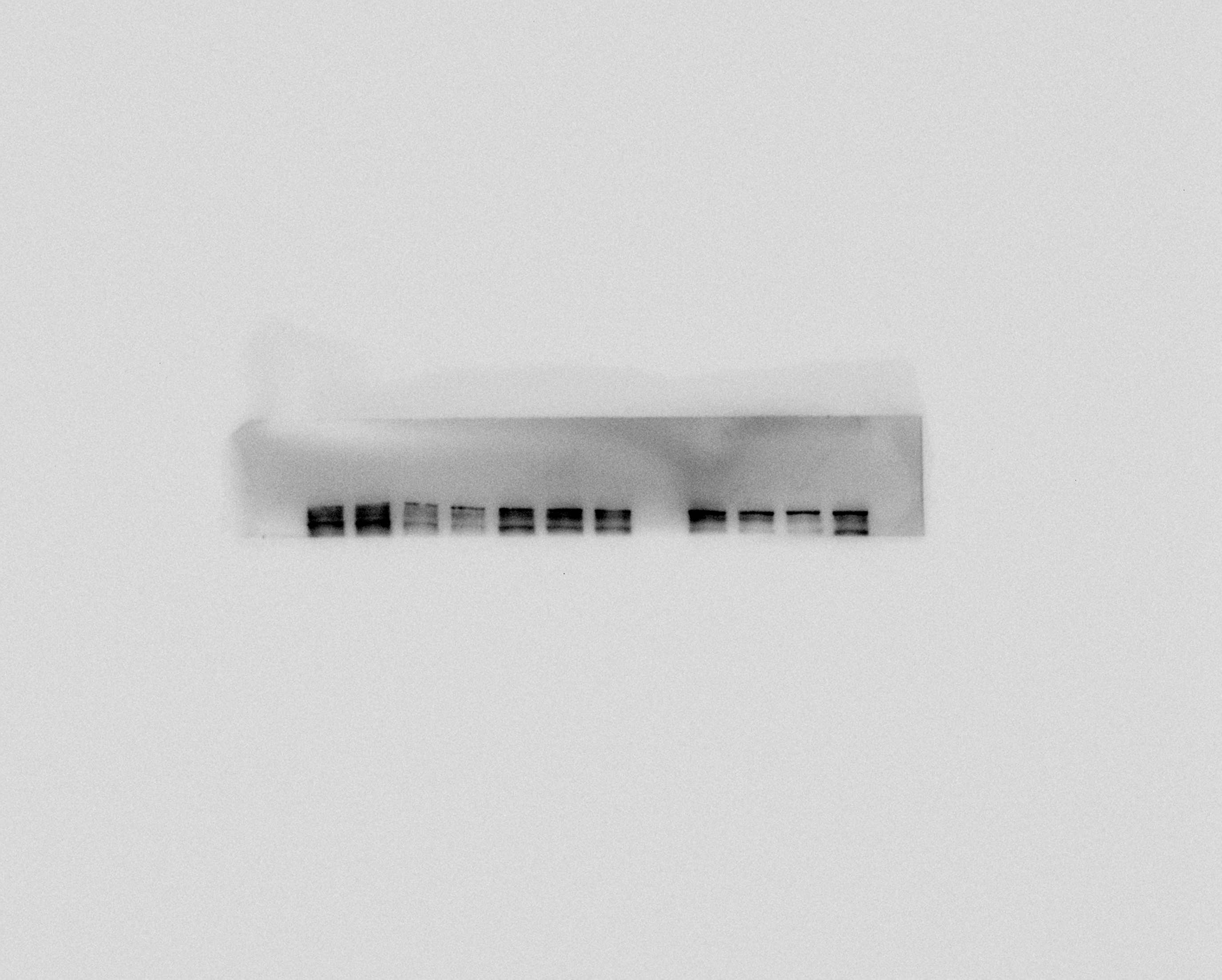

Supplement: Supplementary file 9 — Source data Fig. 4 [file 44318_2024_359_MOESM9_ESM.zip › Figure 4/Fig 4F and 4G/Fig 4F/2-p-mTOR.Tif]

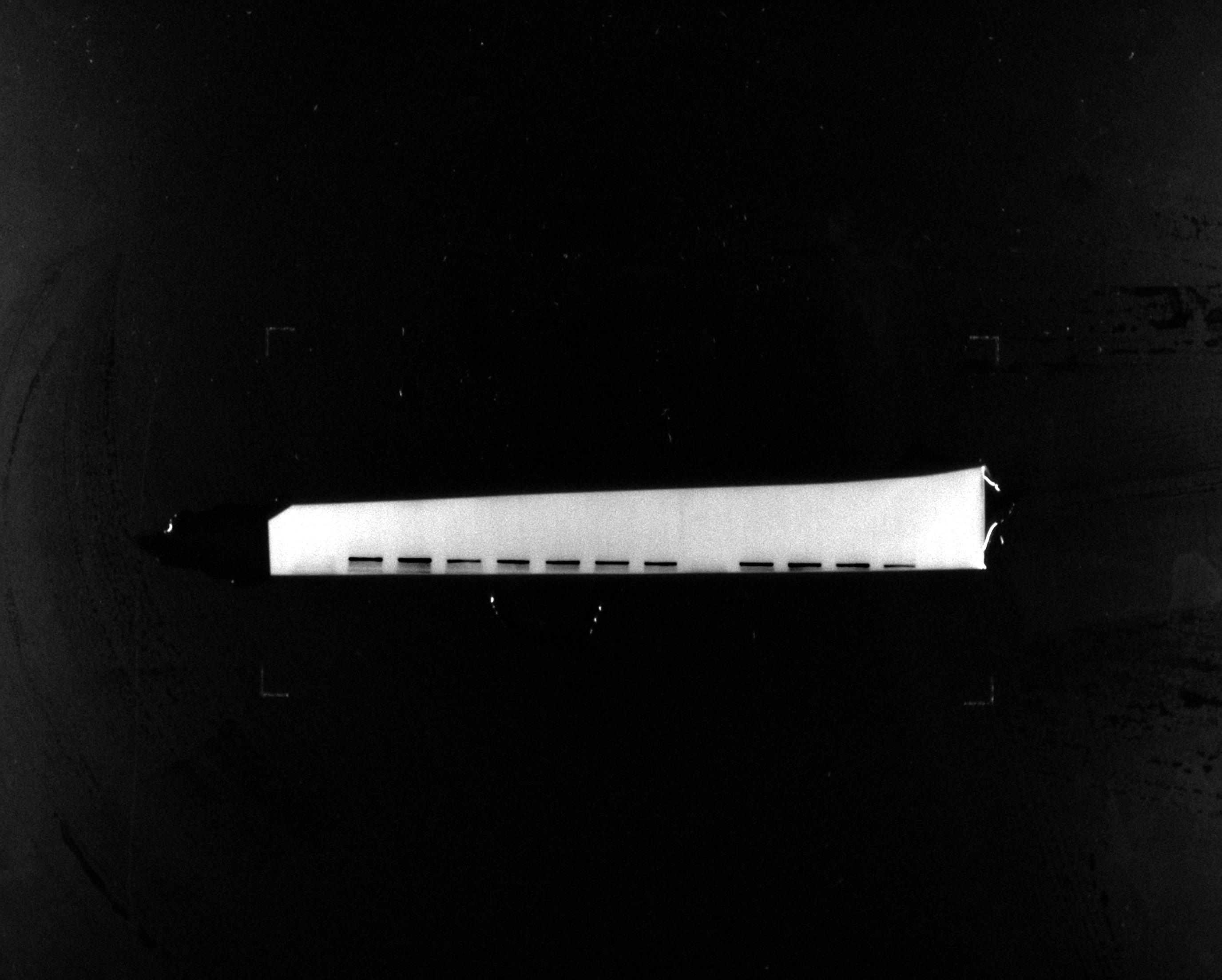

Supplement: Supplementary file 9 — Source data Fig. 4 [file 44318_2024_359_MOESM9_ESM.zip › Figure 4/Fig 4F and 4G/Fig 4F/3-mTOR-merge.Tif]

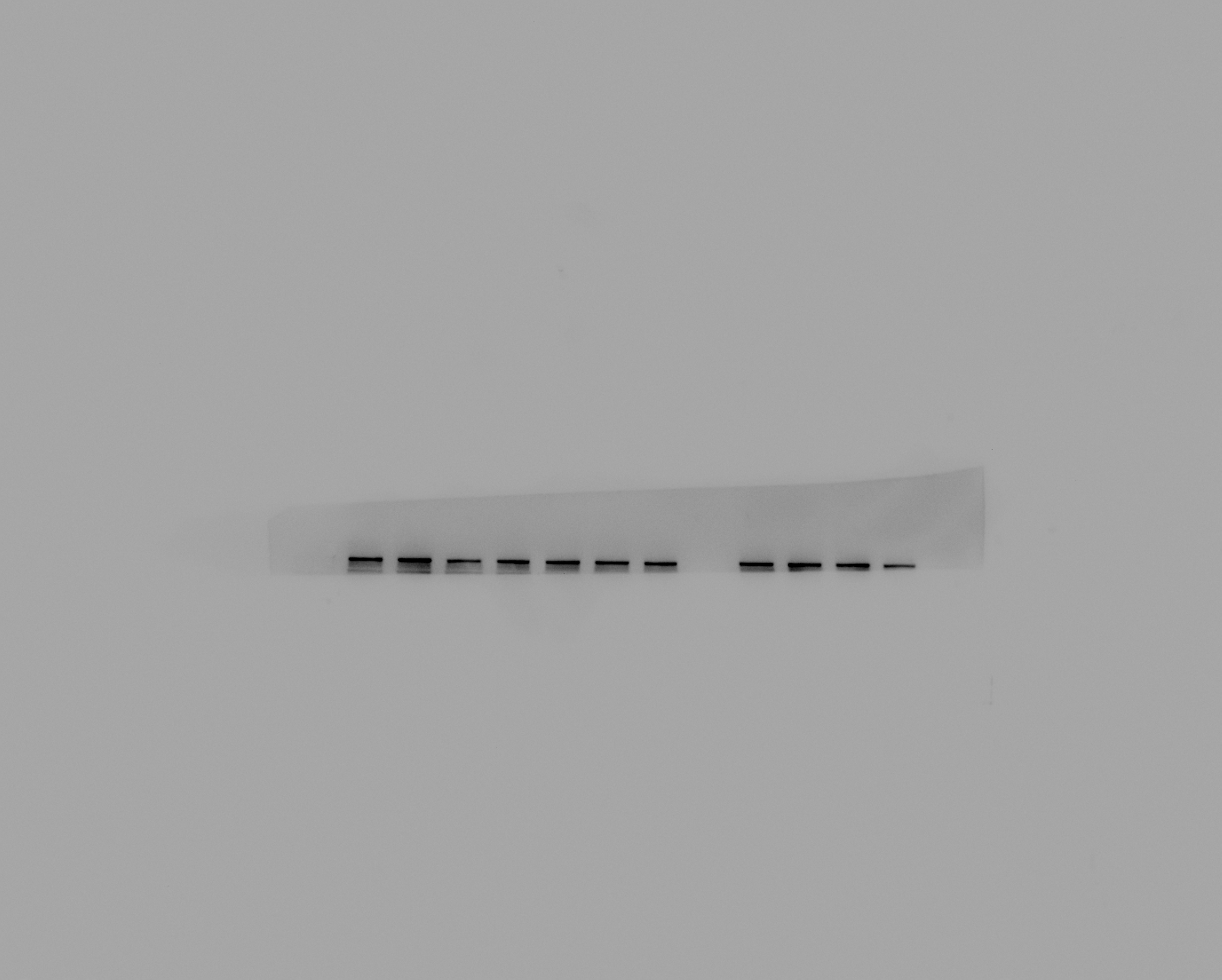

Supplement: Supplementary file 9 — Source data Fig. 4 [file 44318_2024_359_MOESM9_ESM.zip › Figure 4/Fig 4F and 4G/Fig 4F/3-mTOR.Tif]

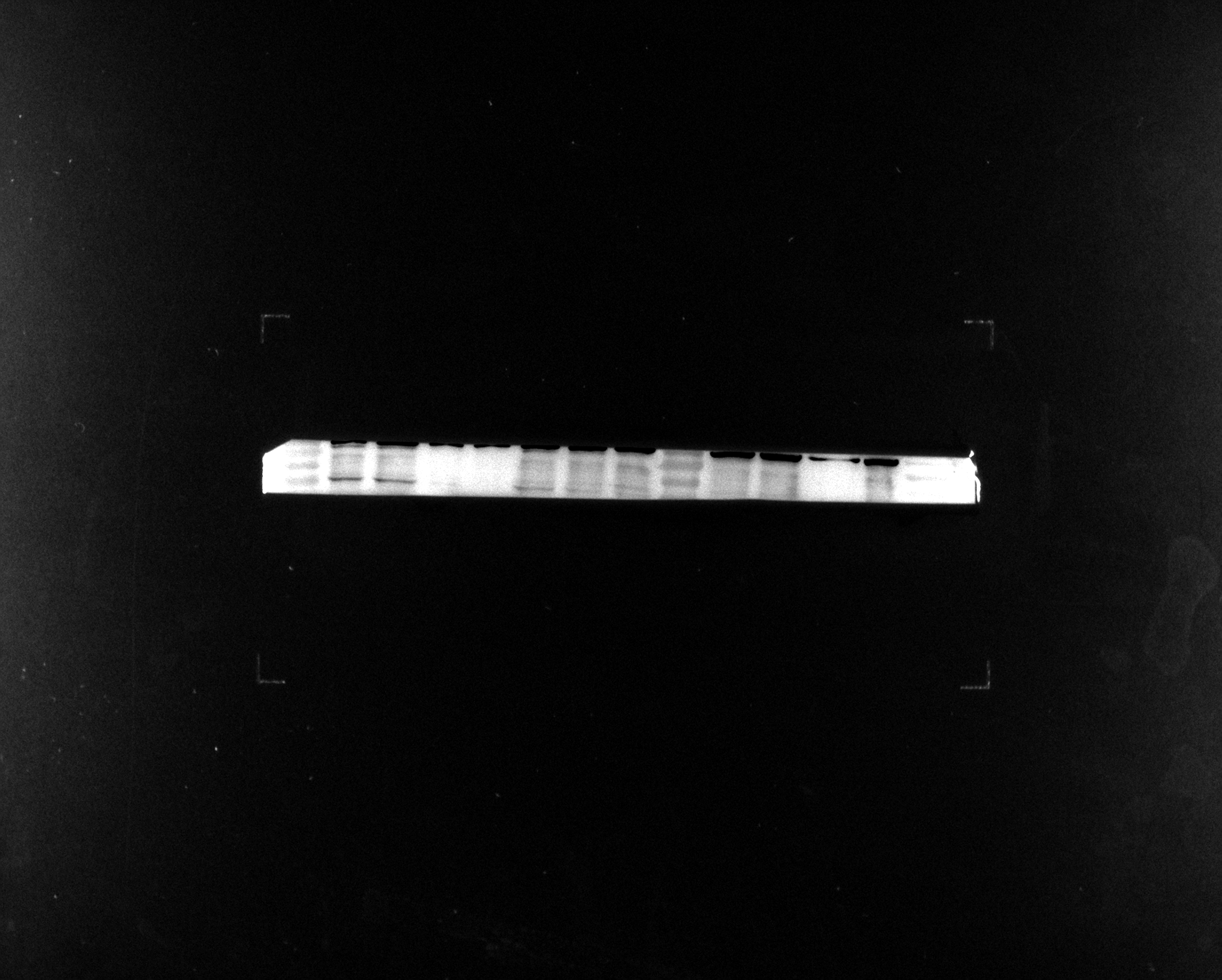

Supplement: Supplementary file 9 — Source data Fig. 4 [file 44318_2024_359_MOESM9_ESM.zip › Figure 4/Fig 4F and 4G/Fig 4F/4-p-S6K-merge.Tif]

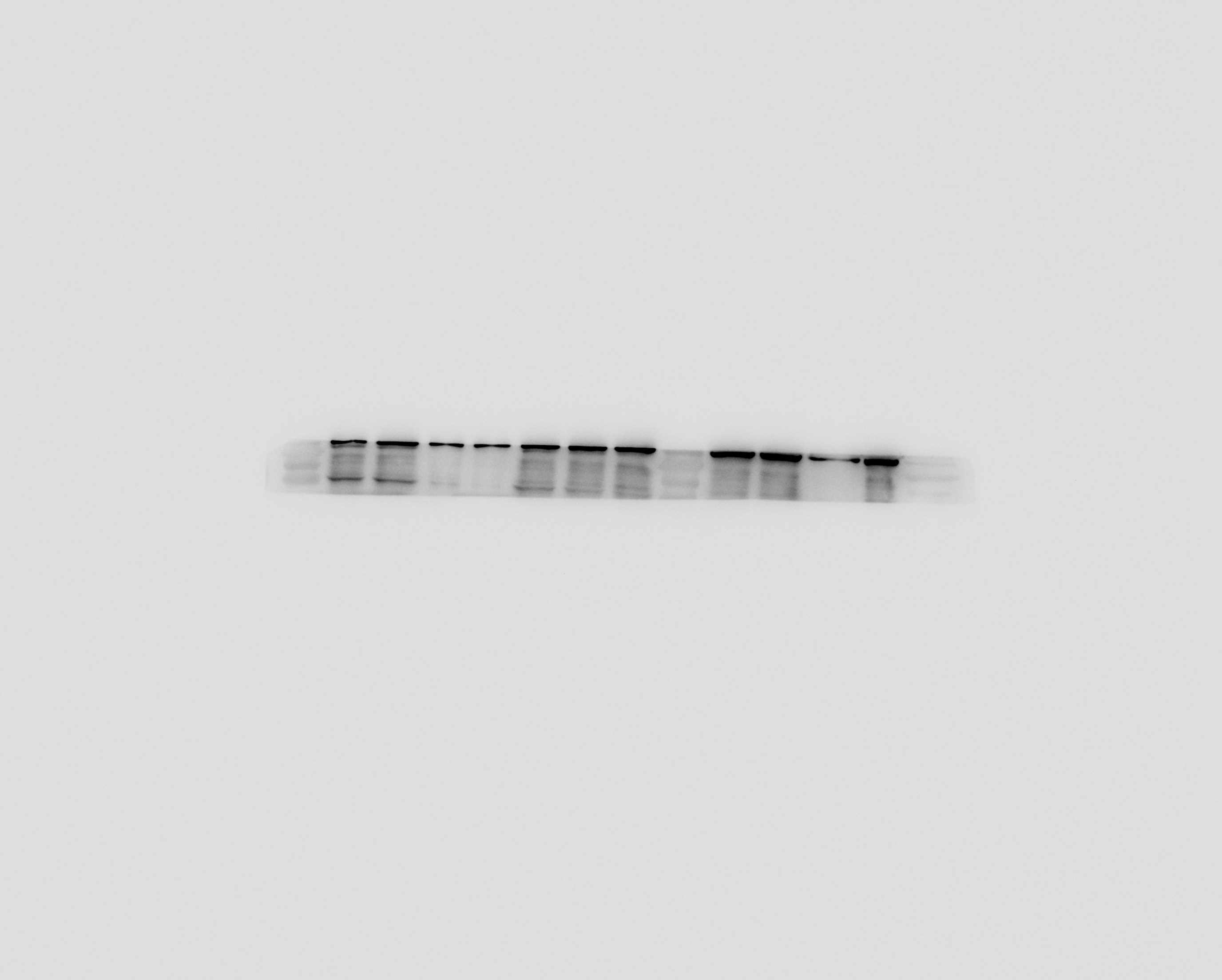

Supplement: Supplementary file 9 — Source data Fig. 4 [file 44318_2024_359_MOESM9_ESM.zip › Figure 4/Fig 4F and 4G/Fig 4F/4-p-S6K.Tif]

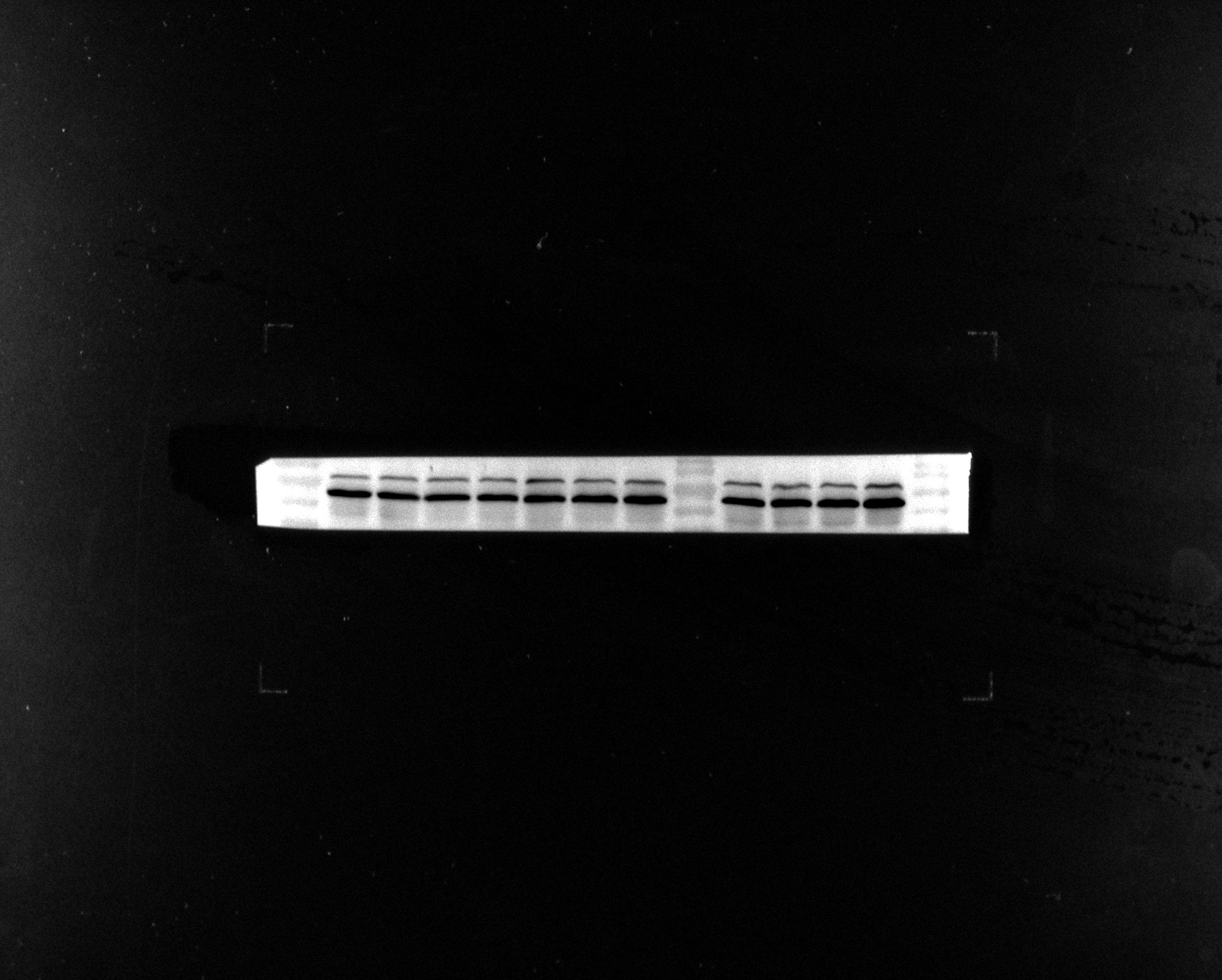

Supplement: Supplementary file 9 — Source data Fig. 4 [file 44318_2024_359_MOESM9_ESM.zip › Figure 4/Fig 4F and 4G/Fig 4F/5-S6K-merge.Tif]

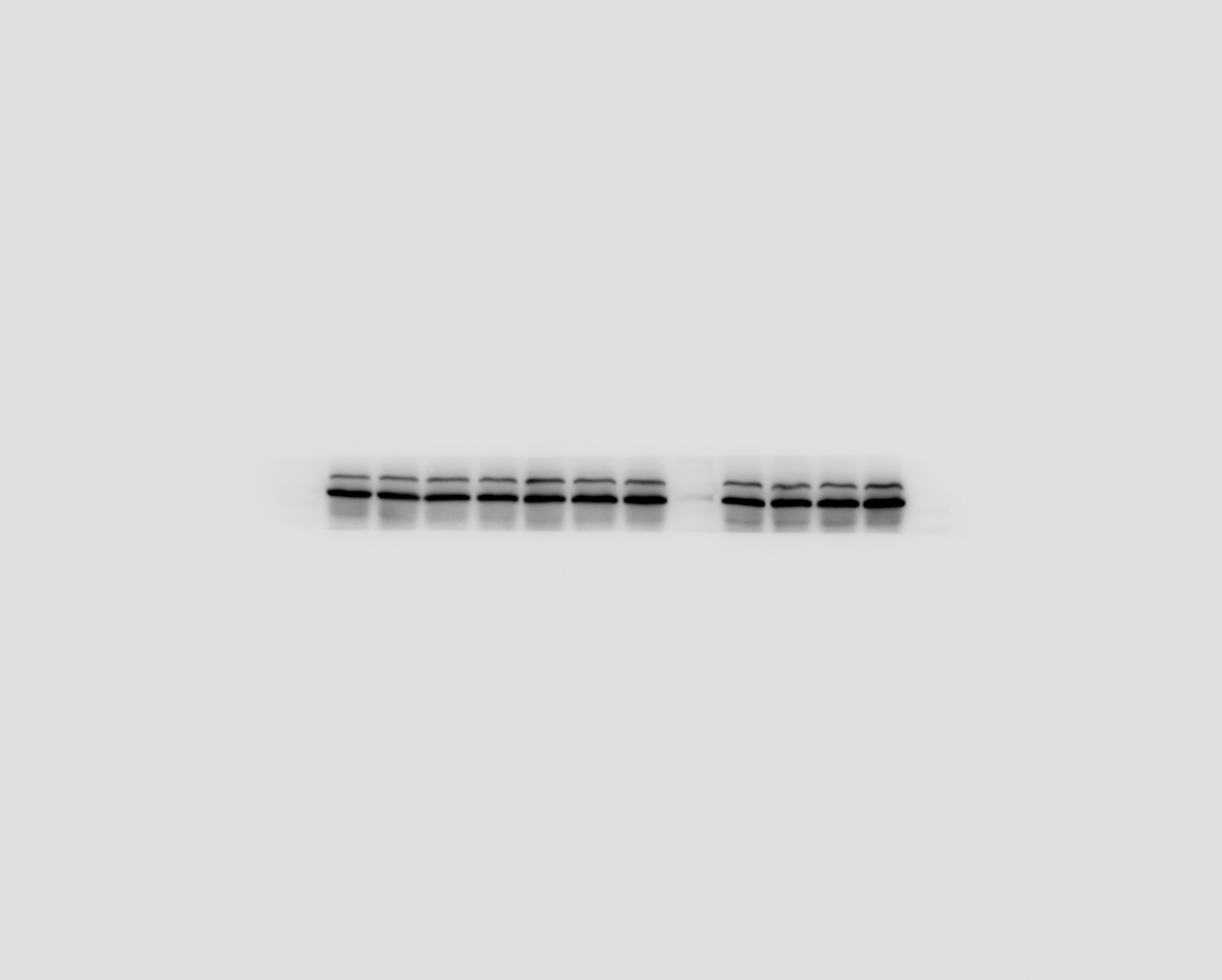

Supplement: Supplementary file 9 — Source data Fig. 4 [file 44318_2024_359_MOESM9_ESM.zip › Figure 4/Fig 4F and 4G/Fig 4F/5-S6K.Tif]

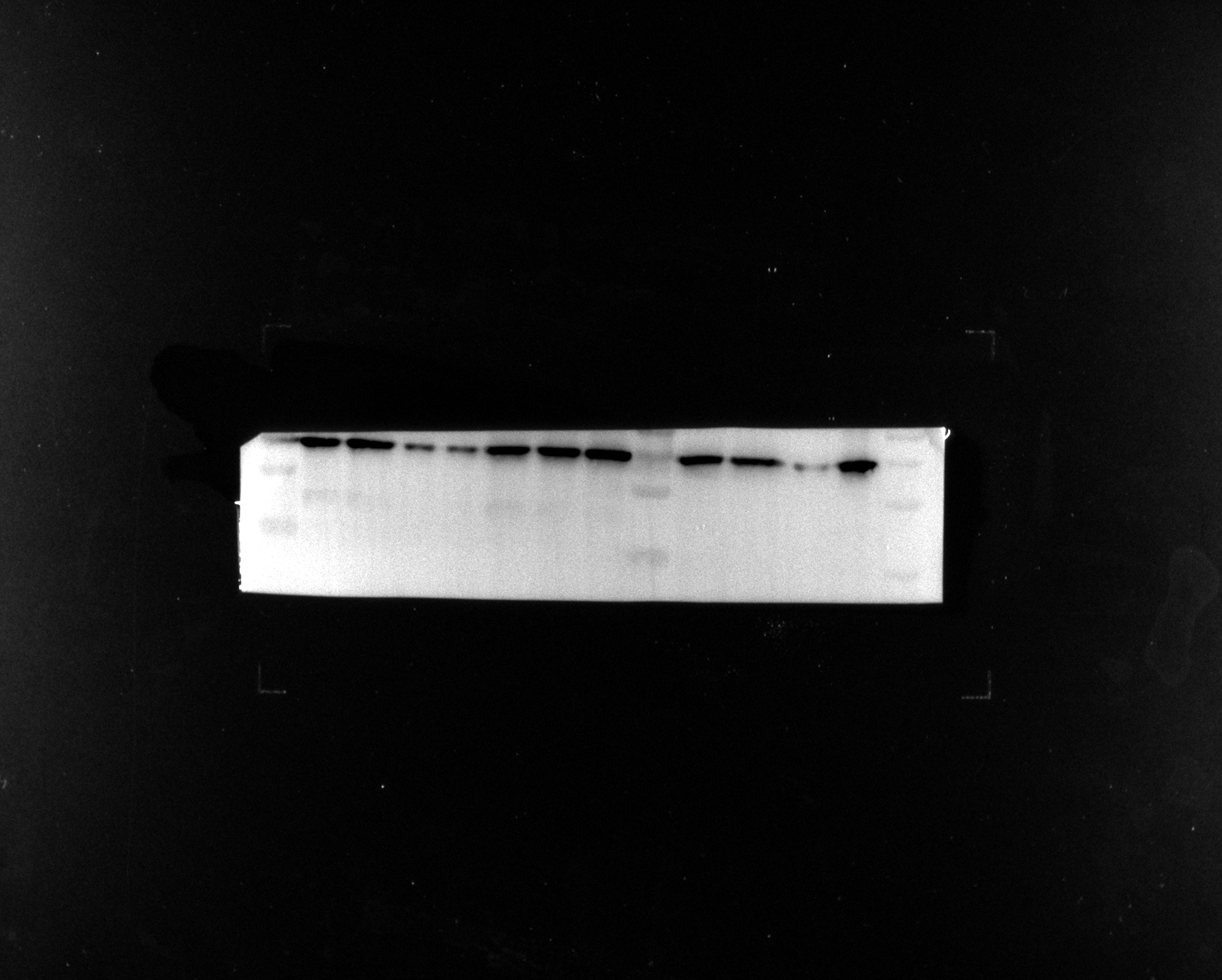

Supplement: Supplementary file 9 — Source data Fig. 4 [file 44318_2024_359_MOESM9_ESM.zip › Figure 4/Fig 4F and 4G/Fig 4F/6-p-S6-merge.Tif]

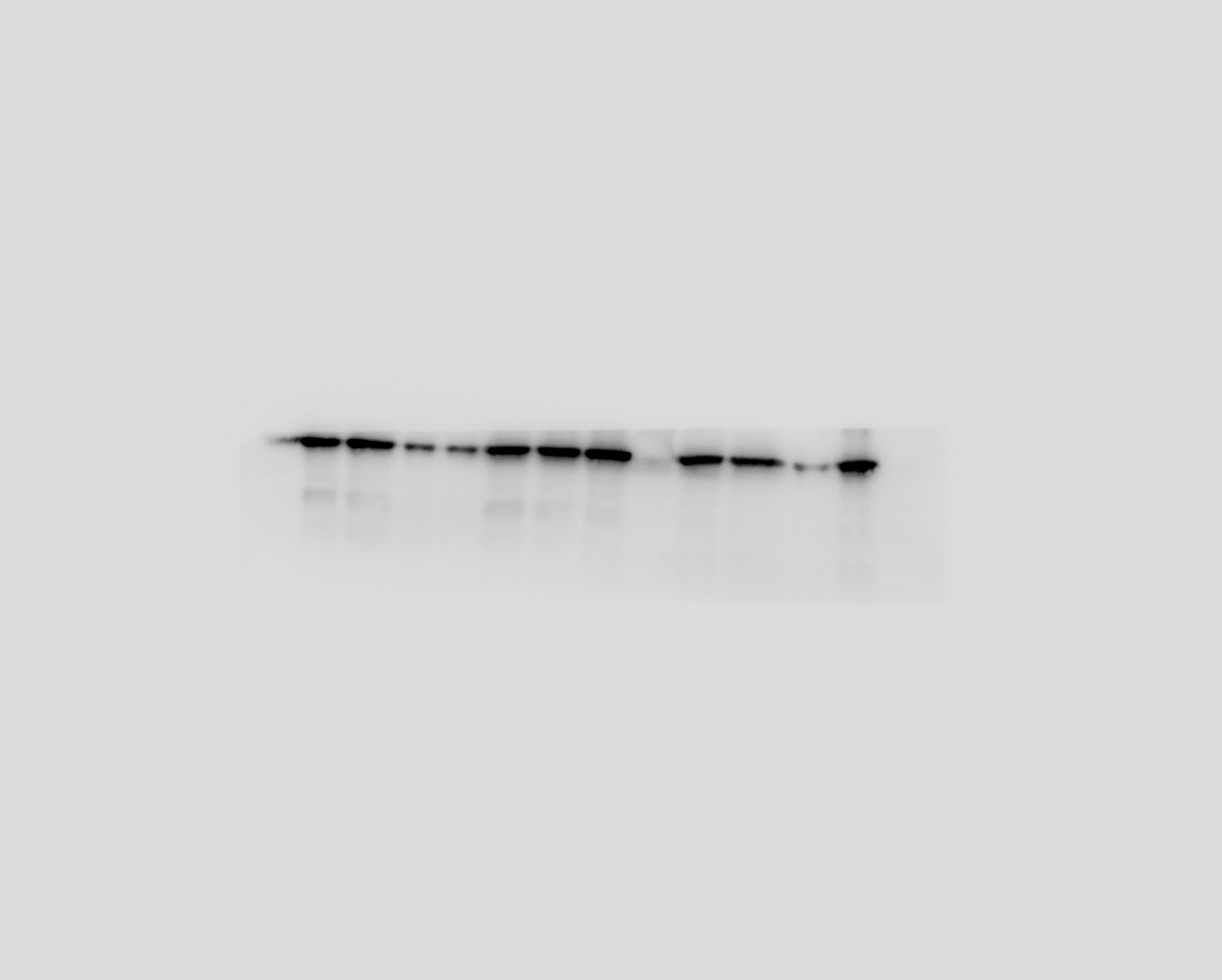

Supplement: Supplementary file 9 — Source data Fig. 4 [file 44318_2024_359_MOESM9_ESM.zip › Figure 4/Fig 4F and 4G/Fig 4F/6-p-S6.Tif]

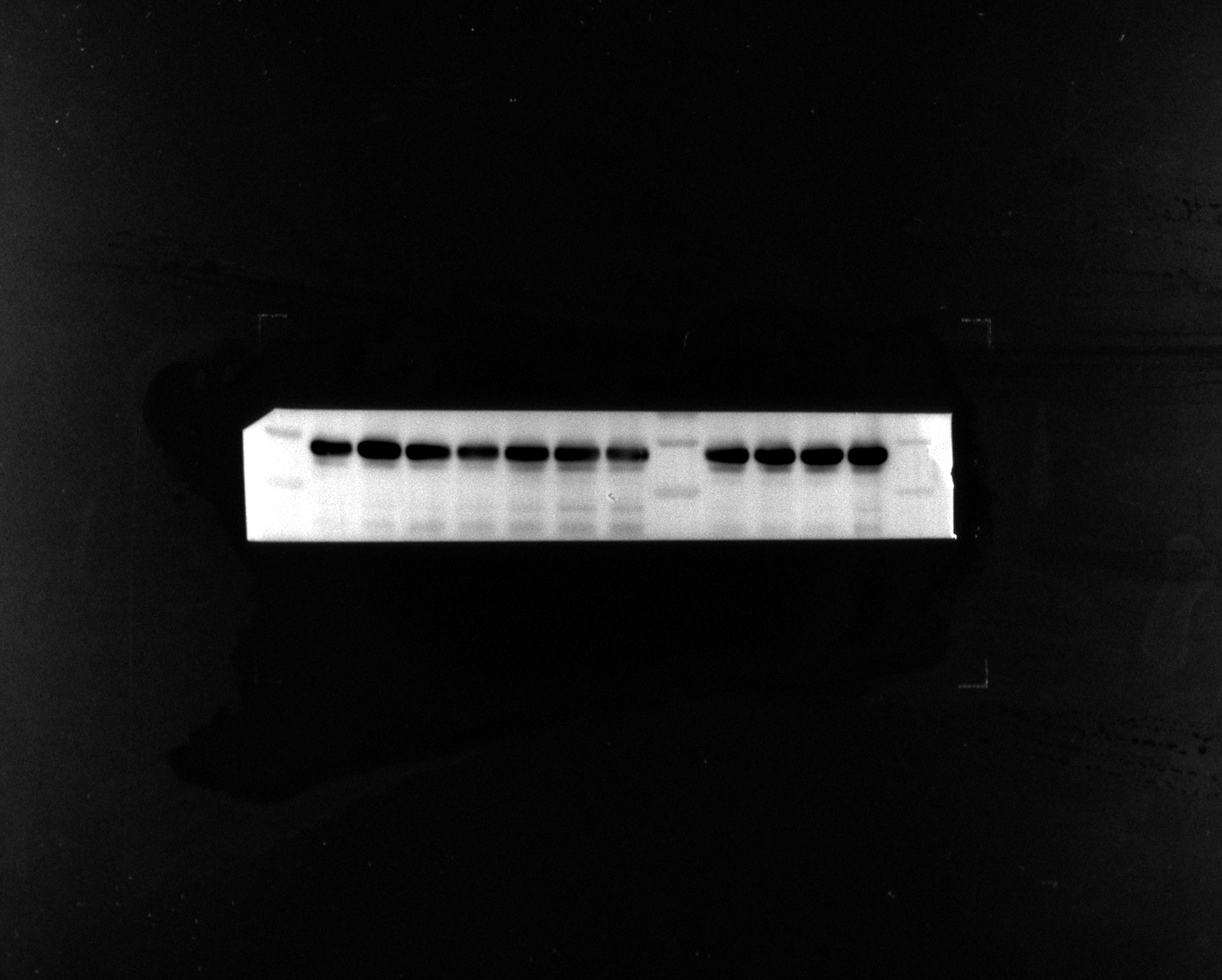

Supplement: Supplementary file 9 — Source data Fig. 4 [file 44318_2024_359_MOESM9_ESM.zip › Figure 4/Fig 4F and 4G/Fig 4F/7-S6-merge.Tif]

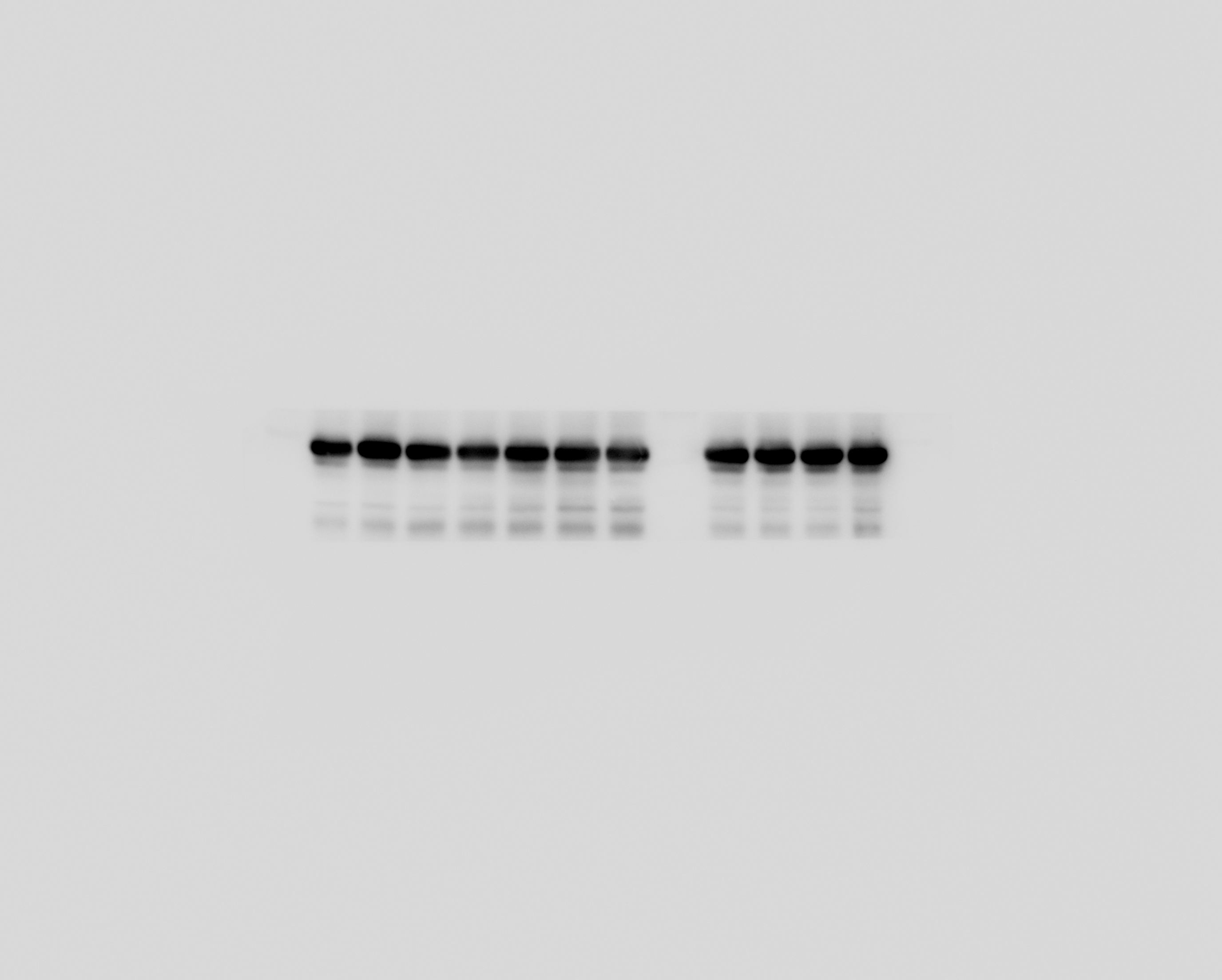

Supplement: Supplementary file 9 — Source data Fig. 4 [file 44318_2024_359_MOESM9_ESM.zip › Figure 4/Fig 4F and 4G/Fig 4F/7-S6.Tif]

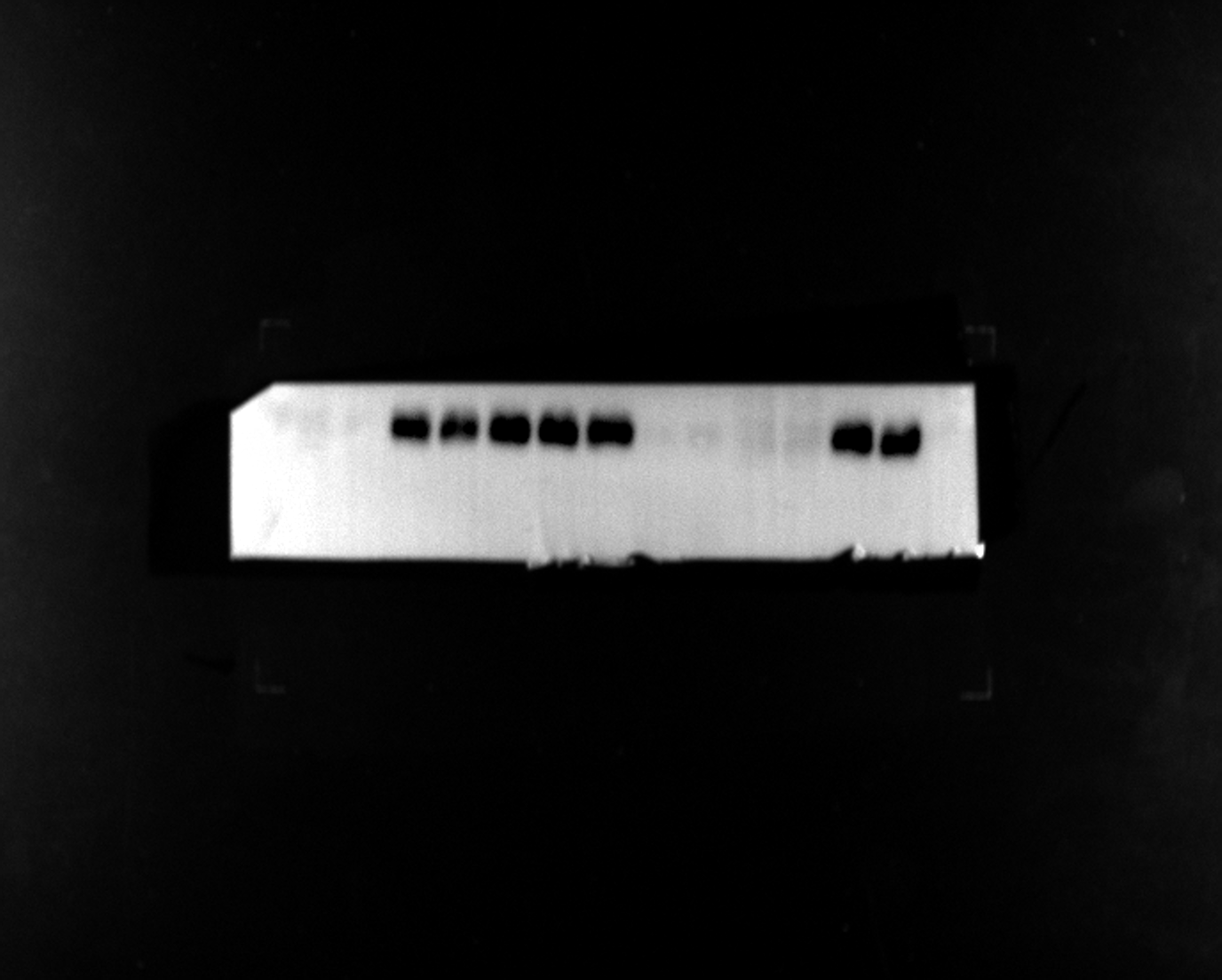

Supplement: Supplementary file 9 — Source data Fig. 4 [file 44318_2024_359_MOESM9_ESM.zip › Figure 4/Fig 4F and 4G/Fig 4F/8-Flag-merge.Tif]

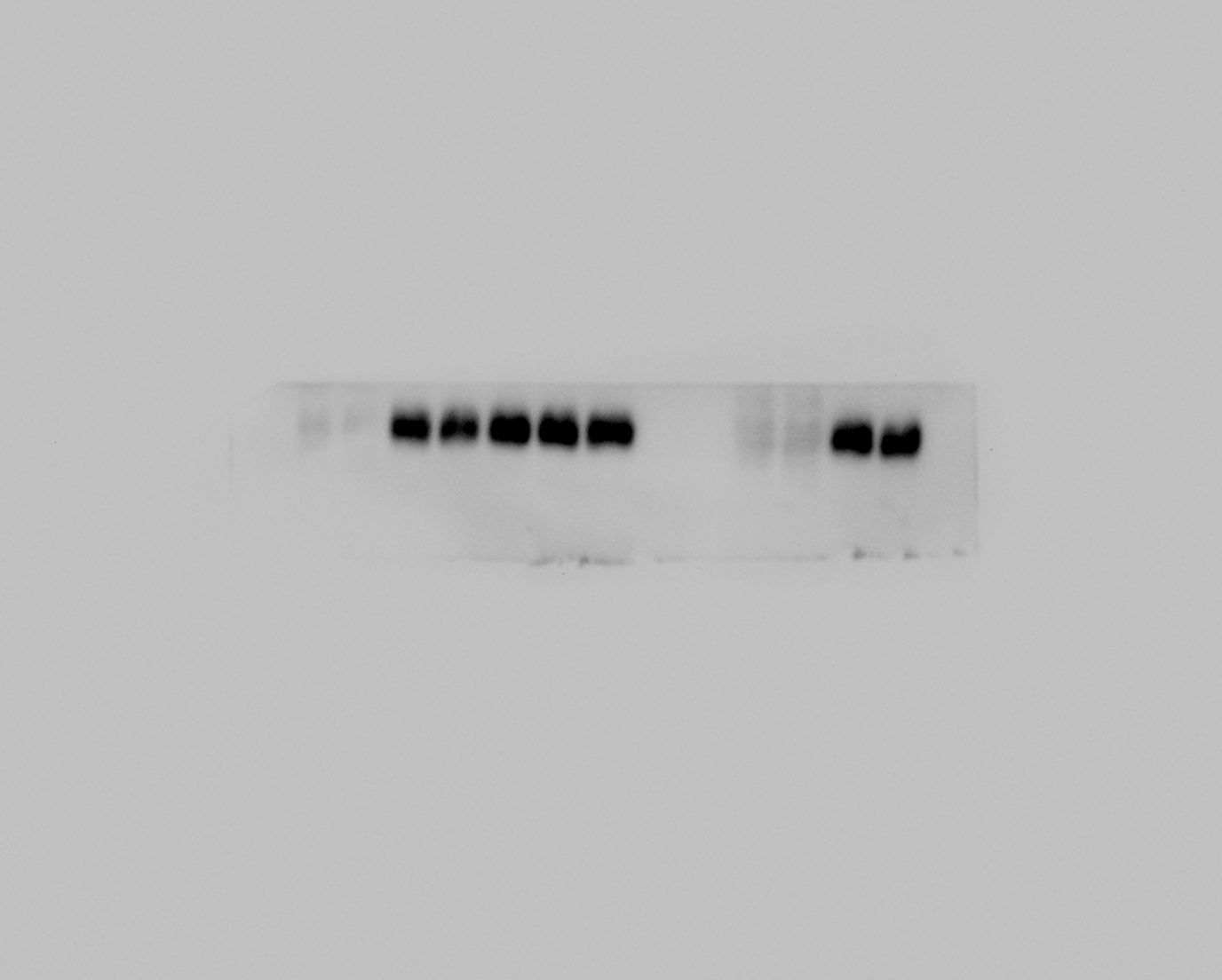

Supplement: Supplementary file 9 — Source data Fig. 4 [file 44318_2024_359_MOESM9_ESM.zip › Figure 4/Fig 4F and 4G/Fig 4F/8-Flag.Tif]

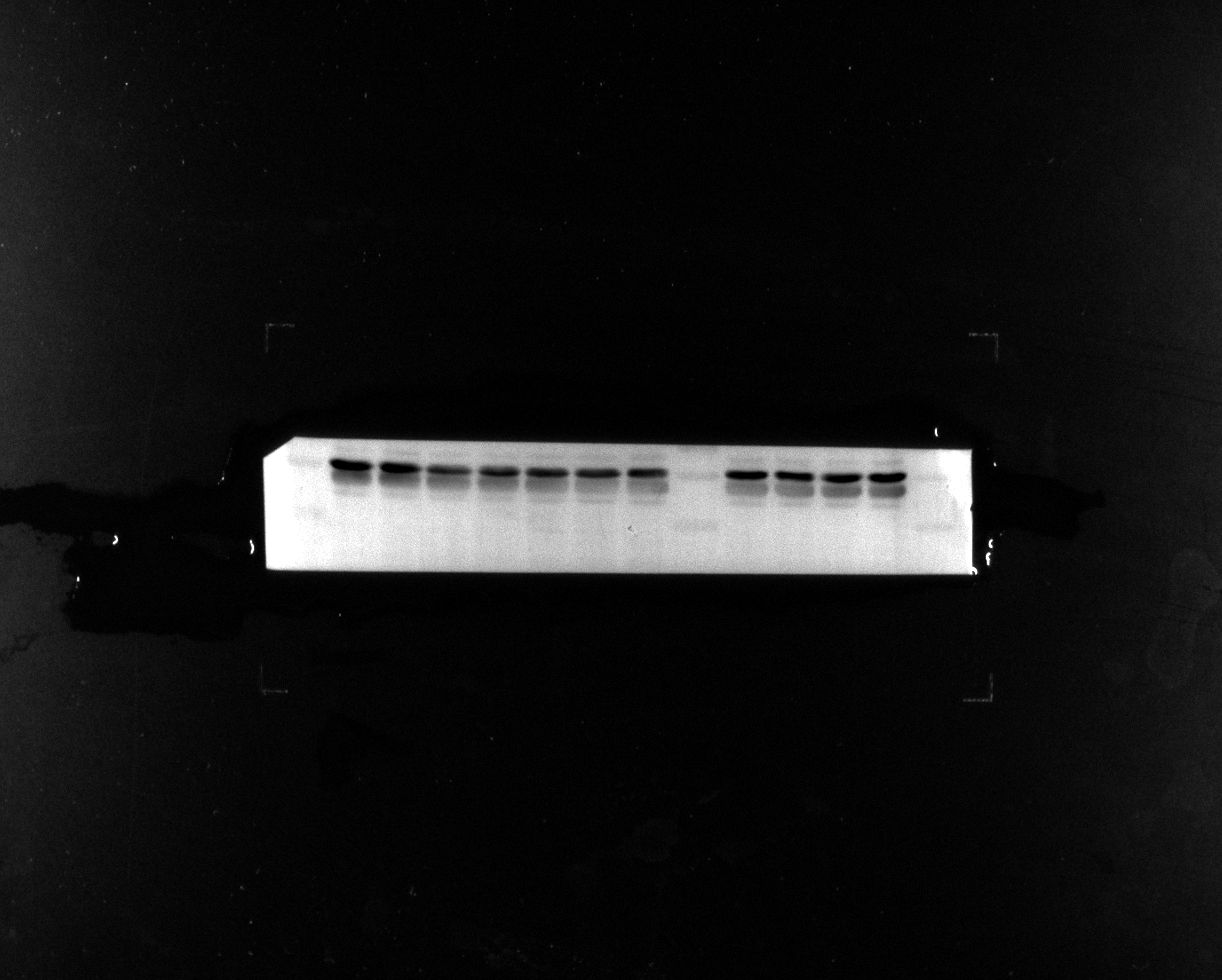

Supplement: Supplementary file 9 — Source data Fig. 4 [file 44318_2024_359_MOESM9_ESM.zip › Figure 4/Fig 4F and 4G/Fig 4F/9-GAPDH-merge.Tif]
