# Supplementary figures and images for: Micropeptide hSPAR regulates glutamine levels and suppresses mammary tumor growth via a TRIM21-P27KIP1-mTOR axis (part 4 of 7)
Source: EMBO J. 2025 Jan 28;44(5):1414–41. doi: 10.1038/s44318-024-00359-z (PMC11876615; doi:10.1038/s44318-024-00359-z)

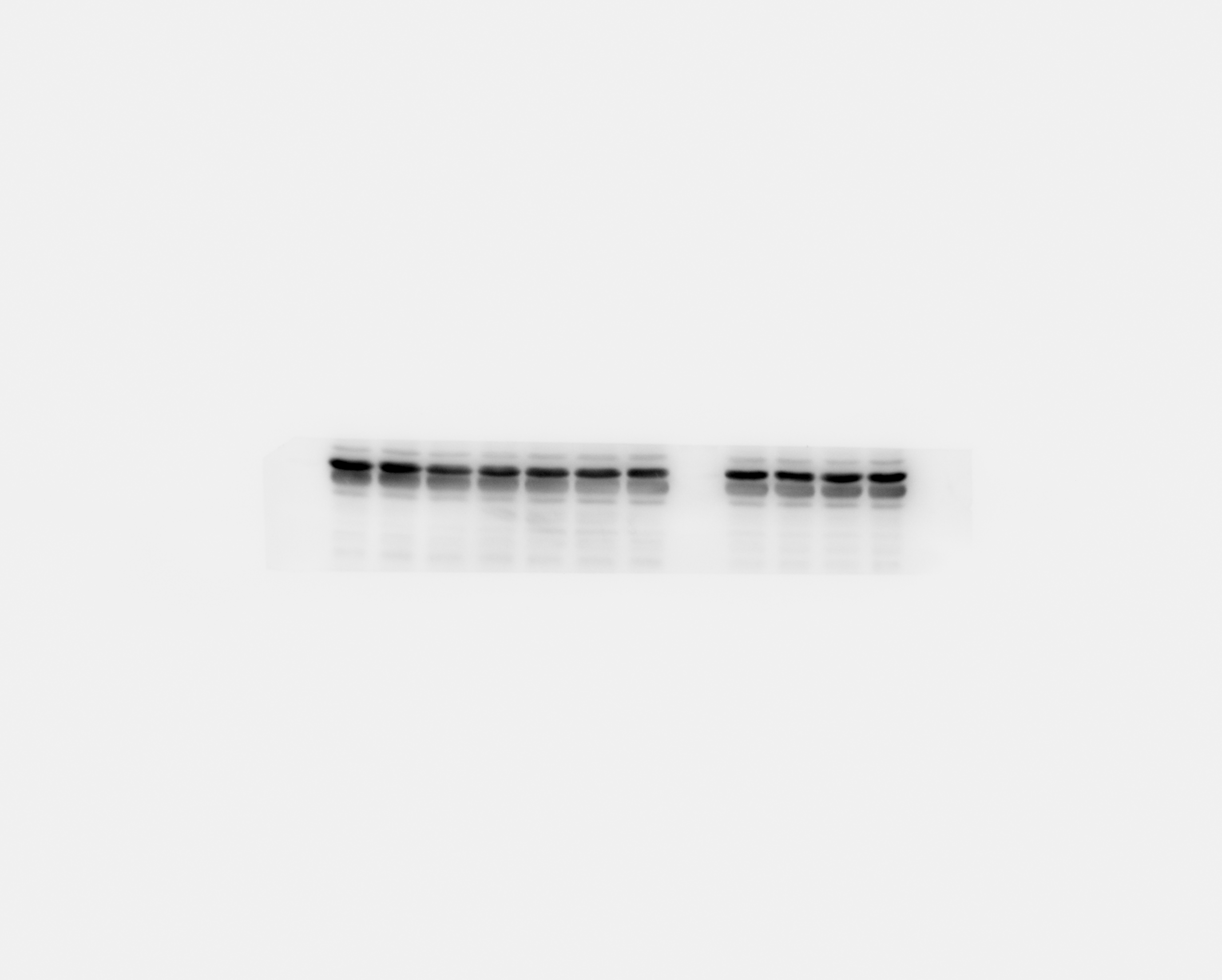

Supplement: Supplementary file 9 — Source data Fig. 4 [file 44318_2024_359_MOESM9_ESM.zip › Figure 4/Fig 4F and 4G/Fig 4F/9-GAPDH.Tif]

Fig 4F

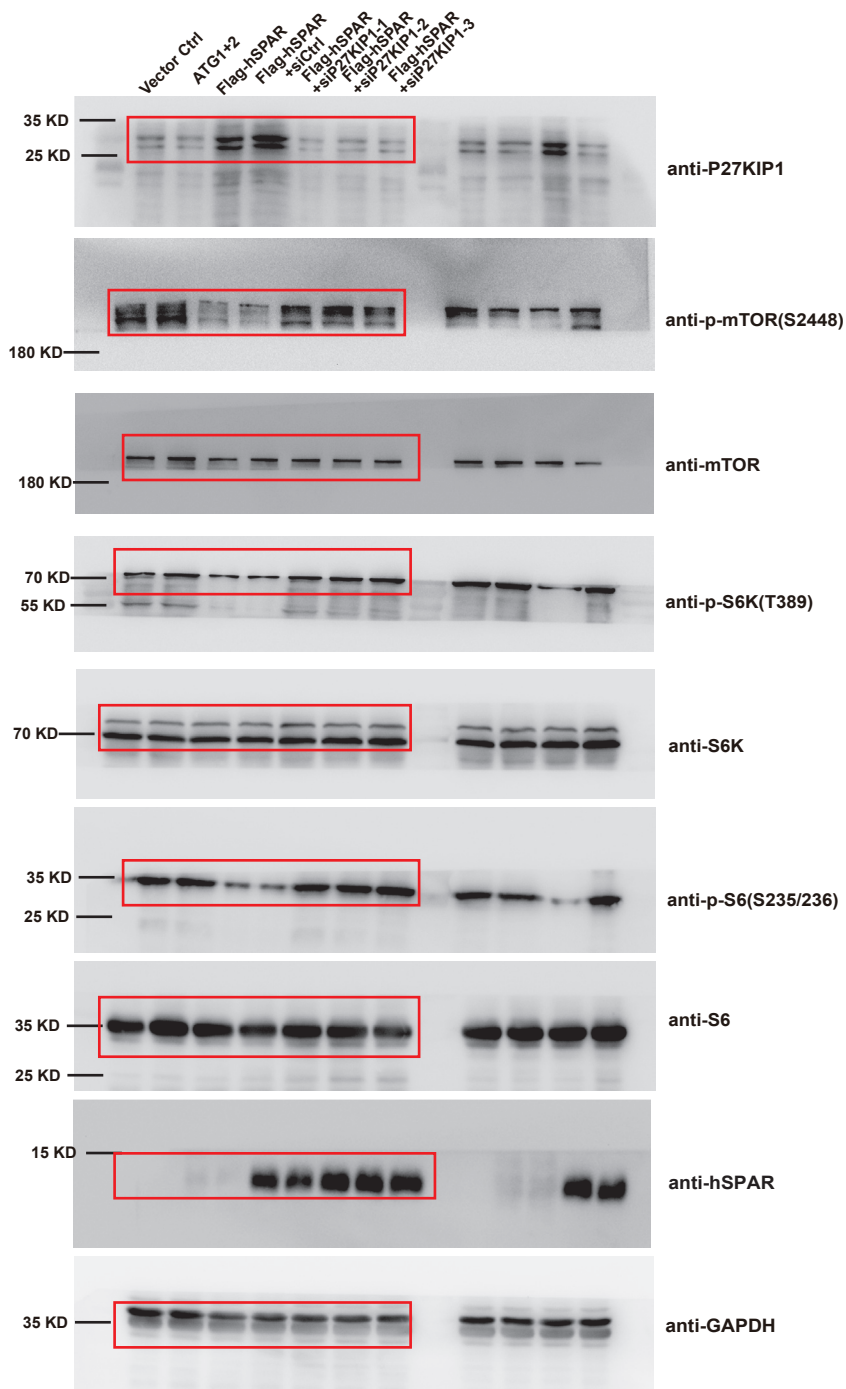

Supplement: Supplementary file 9 — Source data Fig. 4 [file 44318_2024_359_MOESM9_ESM.zip › Figure 4/Fig 4F and 4G/Fig 4F/Fig 4F.pdf]

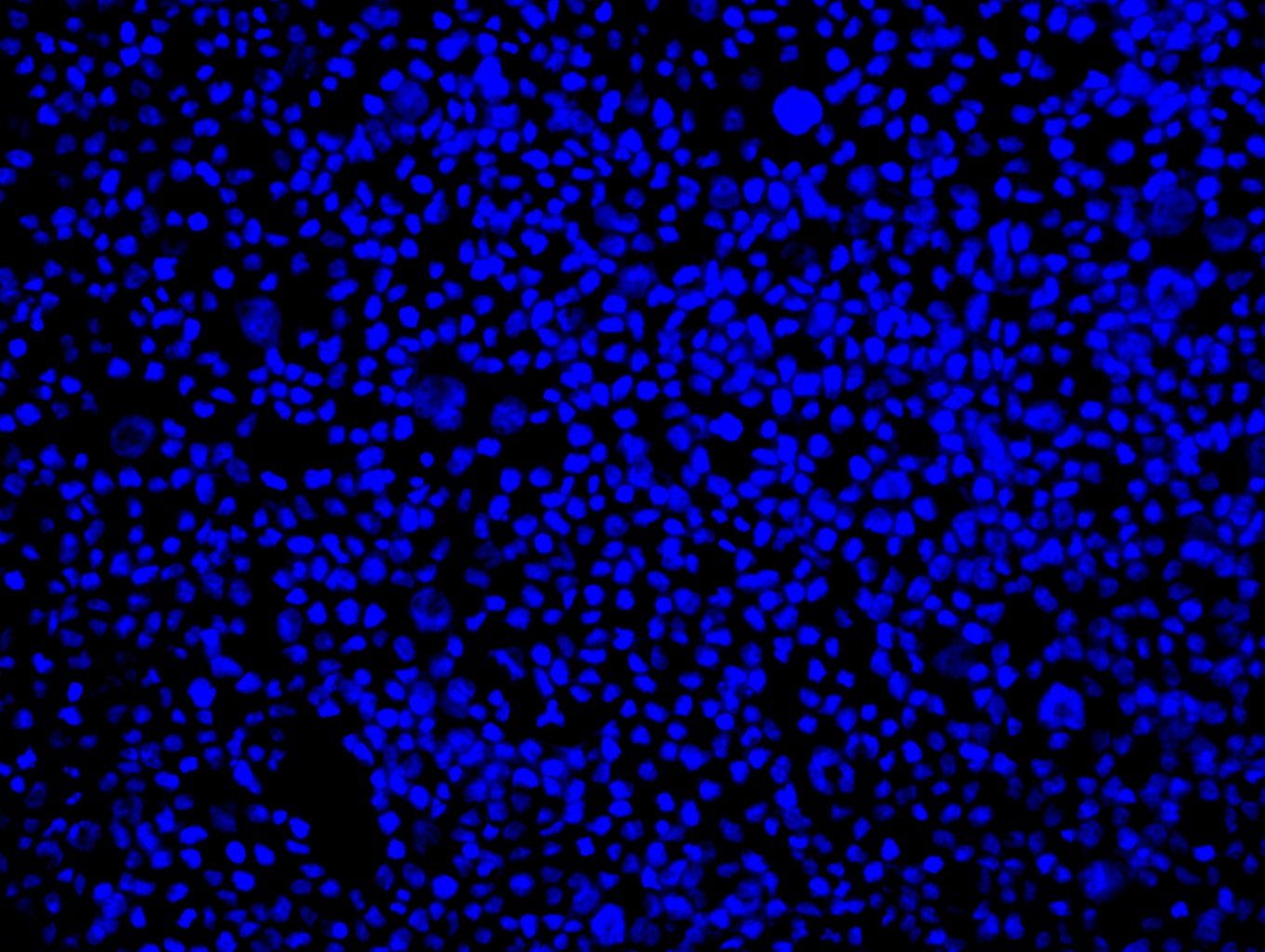

Supplement: Supplementary file 9 — Source data Fig. 4 [file 44318_2024_359_MOESM9_ESM.zip › Figure 4/Fig 4H and 4I/Fig 4H/ATG1+2/1-Hoechst in manu.jpg]

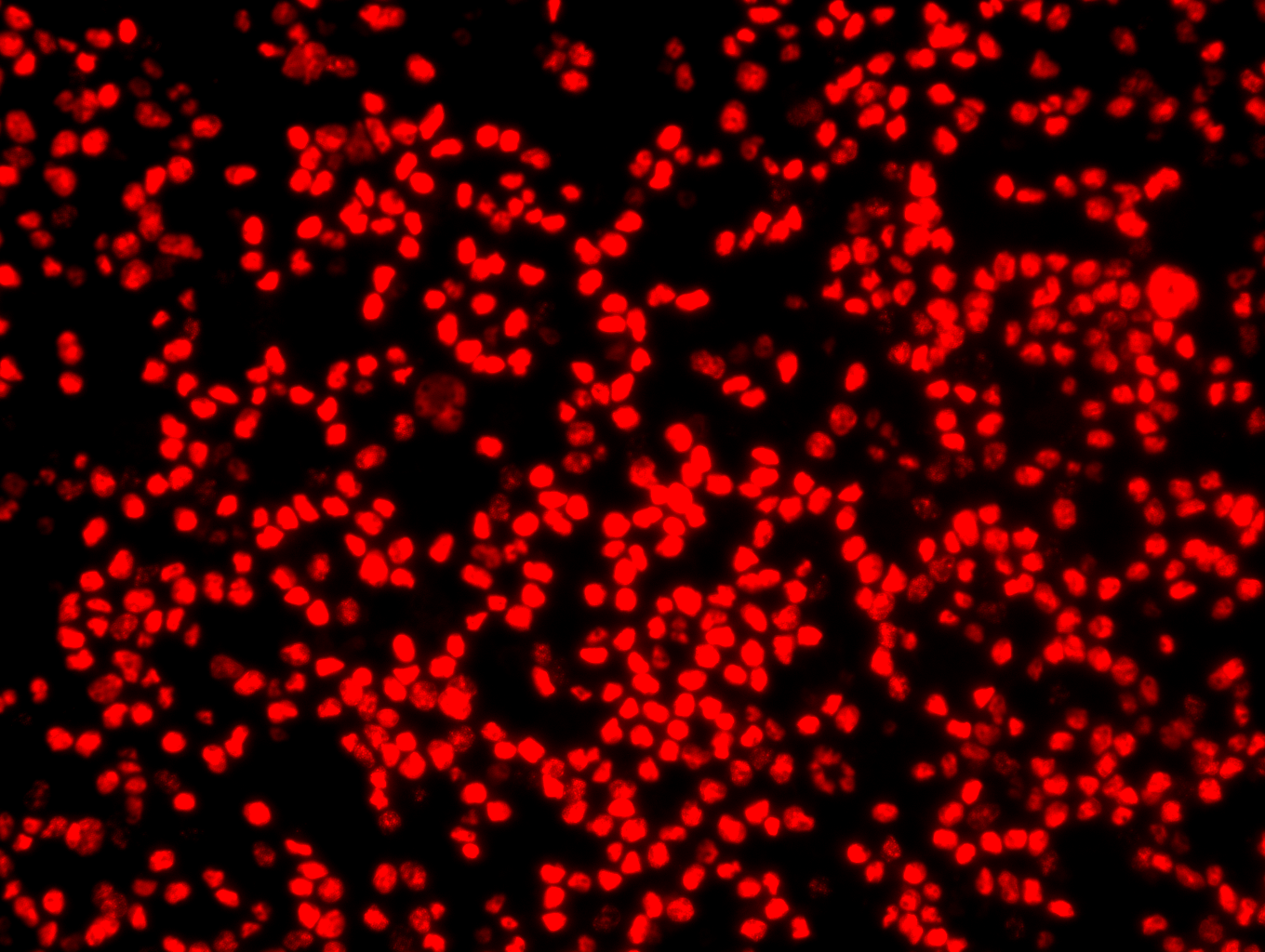

Supplement: Supplementary file 9 — Source data Fig. 4 [file 44318_2024_359_MOESM9_ESM.zip › Figure 4/Fig 4H and 4I/Fig 4H/ATG1+2/1-edu in manu.tif]

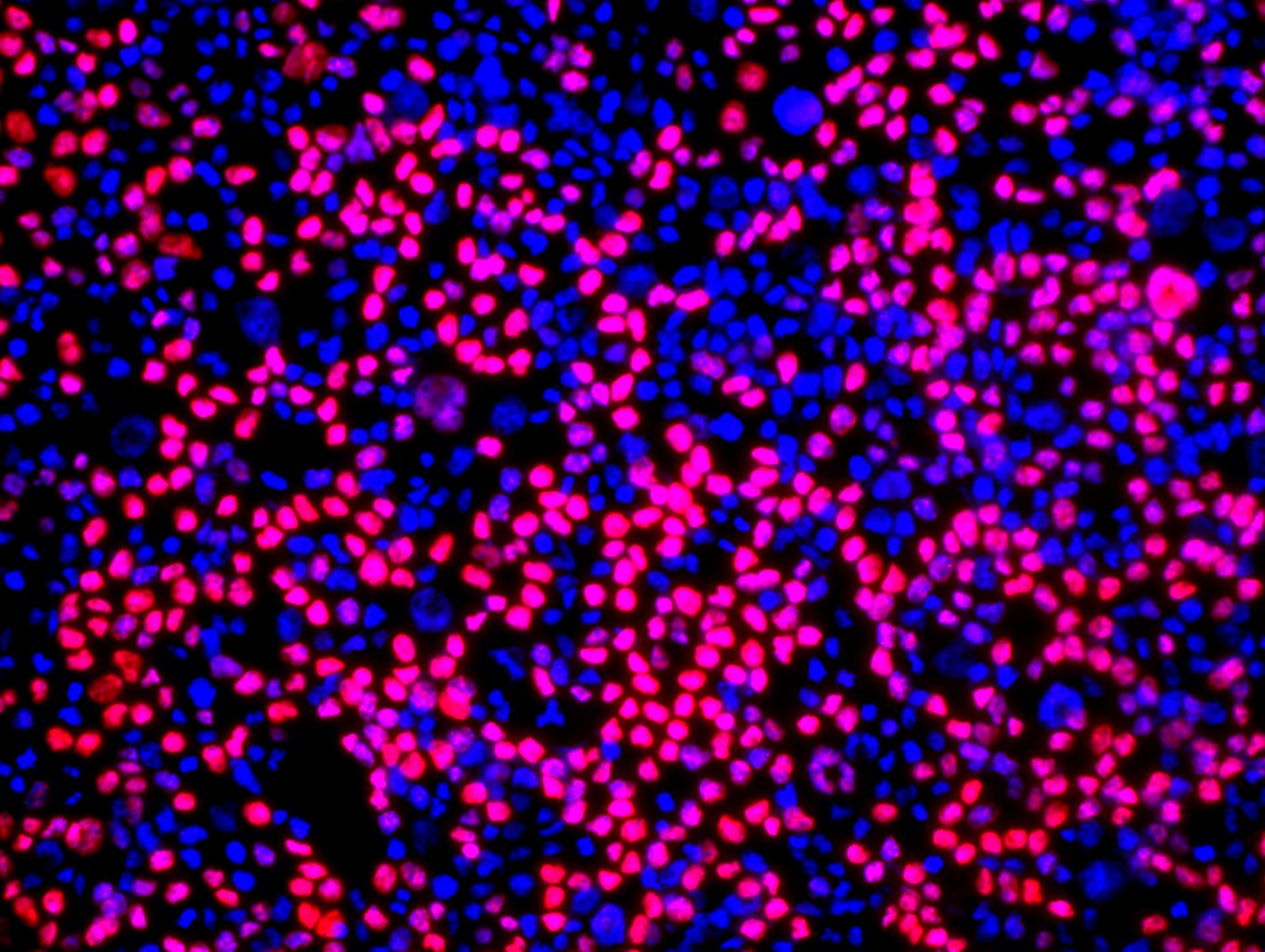

Supplement: Supplementary file 9 — Source data Fig. 4 [file 44318_2024_359_MOESM9_ESM.zip › Figure 4/Fig 4H and 4I/Fig 4H/ATG1+2/1-merge in manu.jpg]

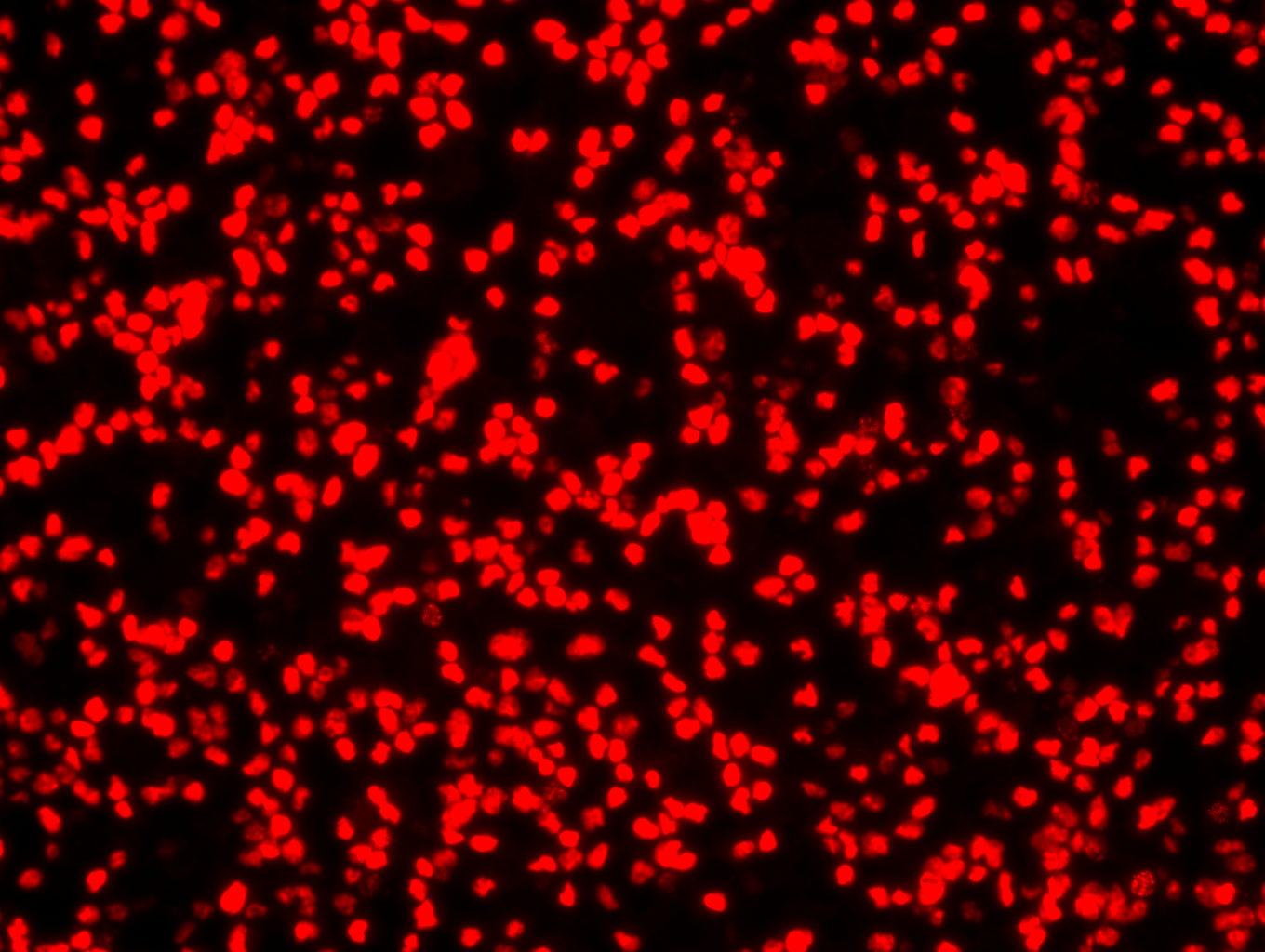

Supplement: Supplementary file 9 — Source data Fig. 4 [file 44318_2024_359_MOESM9_ESM.zip › Figure 4/Fig 4H and 4I/Fig 4H/Vector Ctrl/1-Edu in manu.tif]

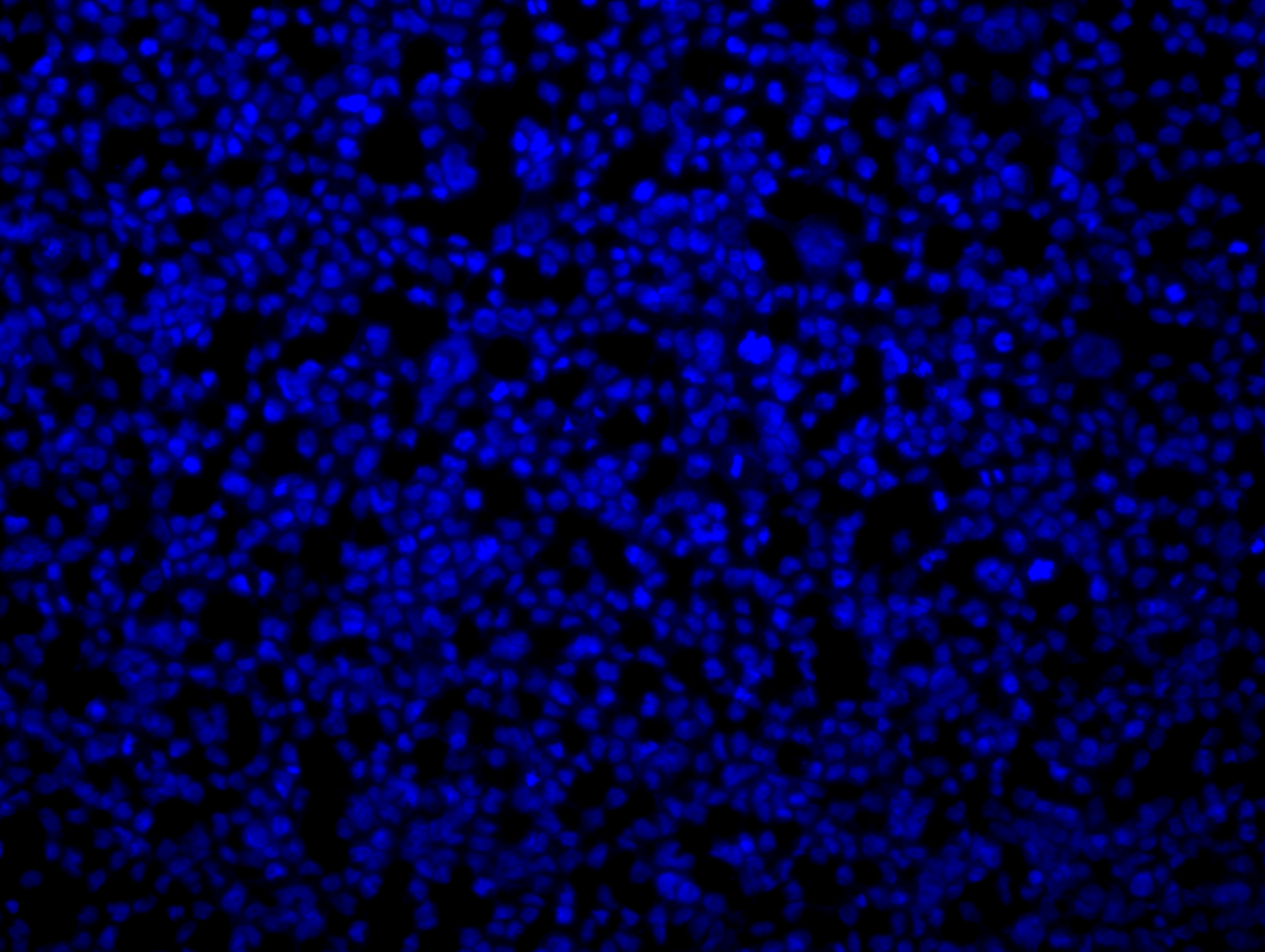

Supplement: Supplementary file 9 — Source data Fig. 4 [file 44318_2024_359_MOESM9_ESM.zip › Figure 4/Fig 4H and 4I/Fig 4H/Vector Ctrl/1-Hoechst in manu.tif]

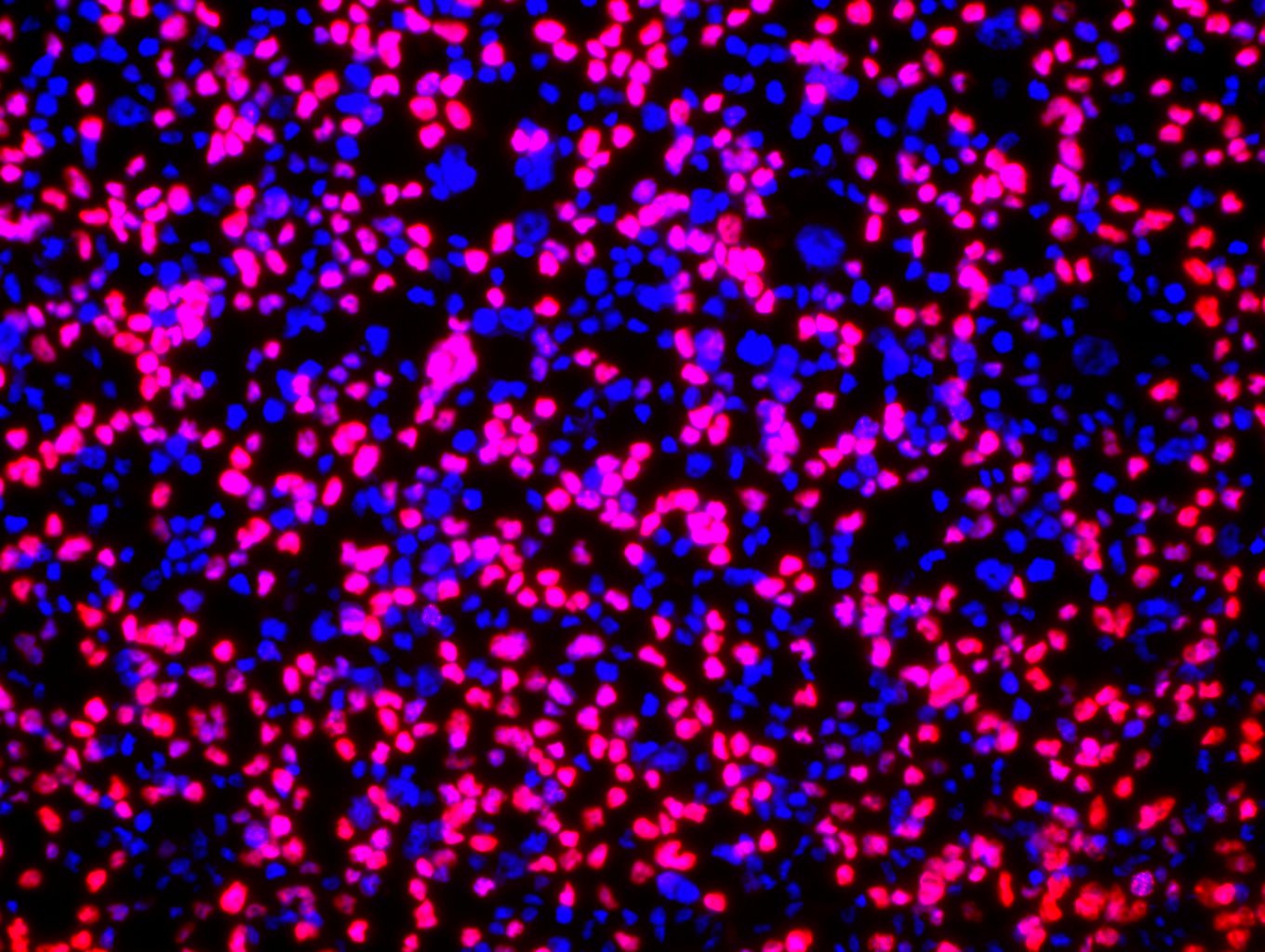

Supplement: Supplementary file 9 — Source data Fig. 4 [file 44318_2024_359_MOESM9_ESM.zip › Figure 4/Fig 4H and 4I/Fig 4H/Vector Ctrl/1-merge in manu.jpg]

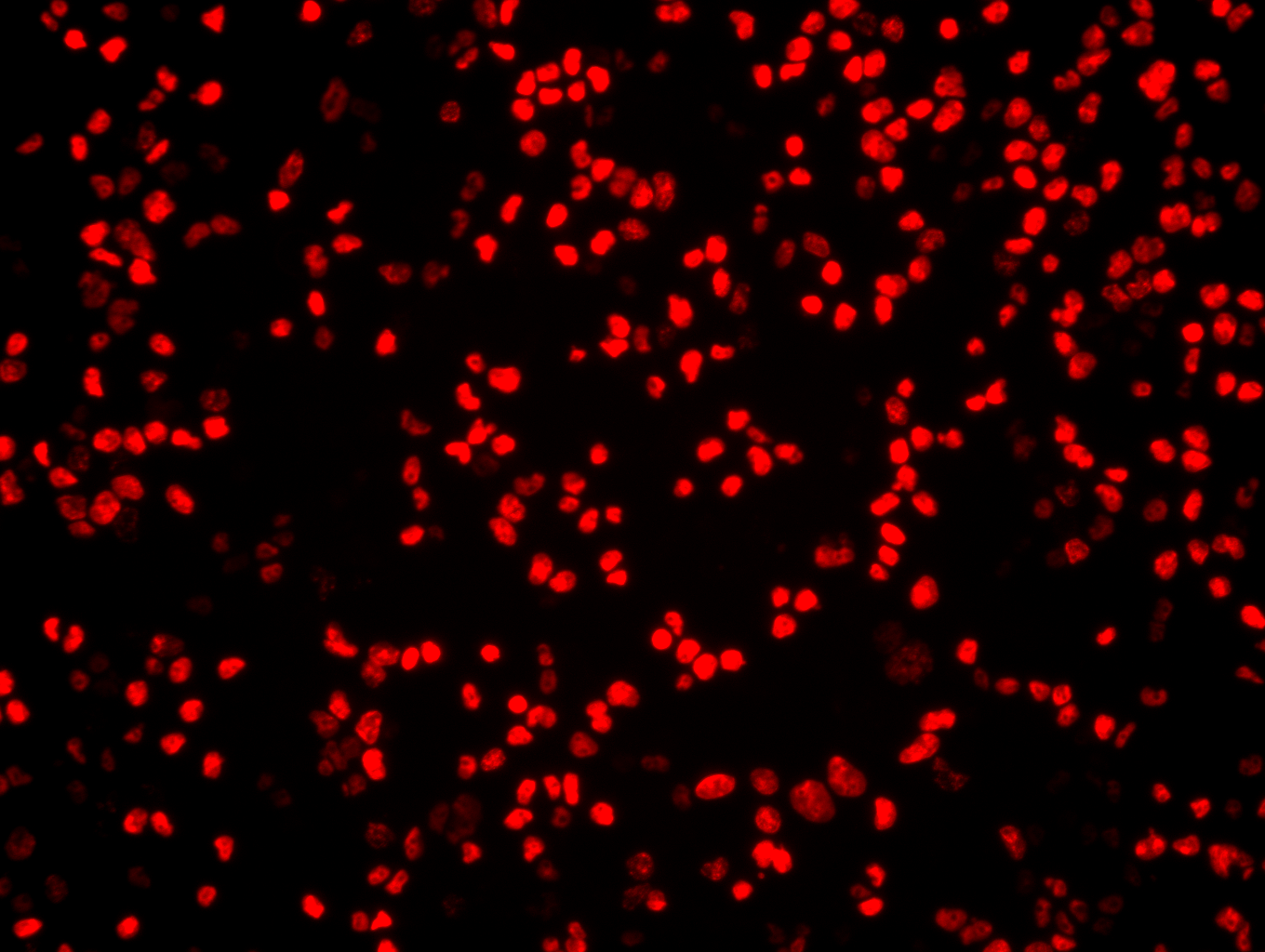

Supplement: Supplementary file 9 — Source data Fig. 4 [file 44318_2024_359_MOESM9_ESM.zip › Figure 4/Fig 4H and 4I/Fig 4H/hSPAR/1-edu in manu.tif]

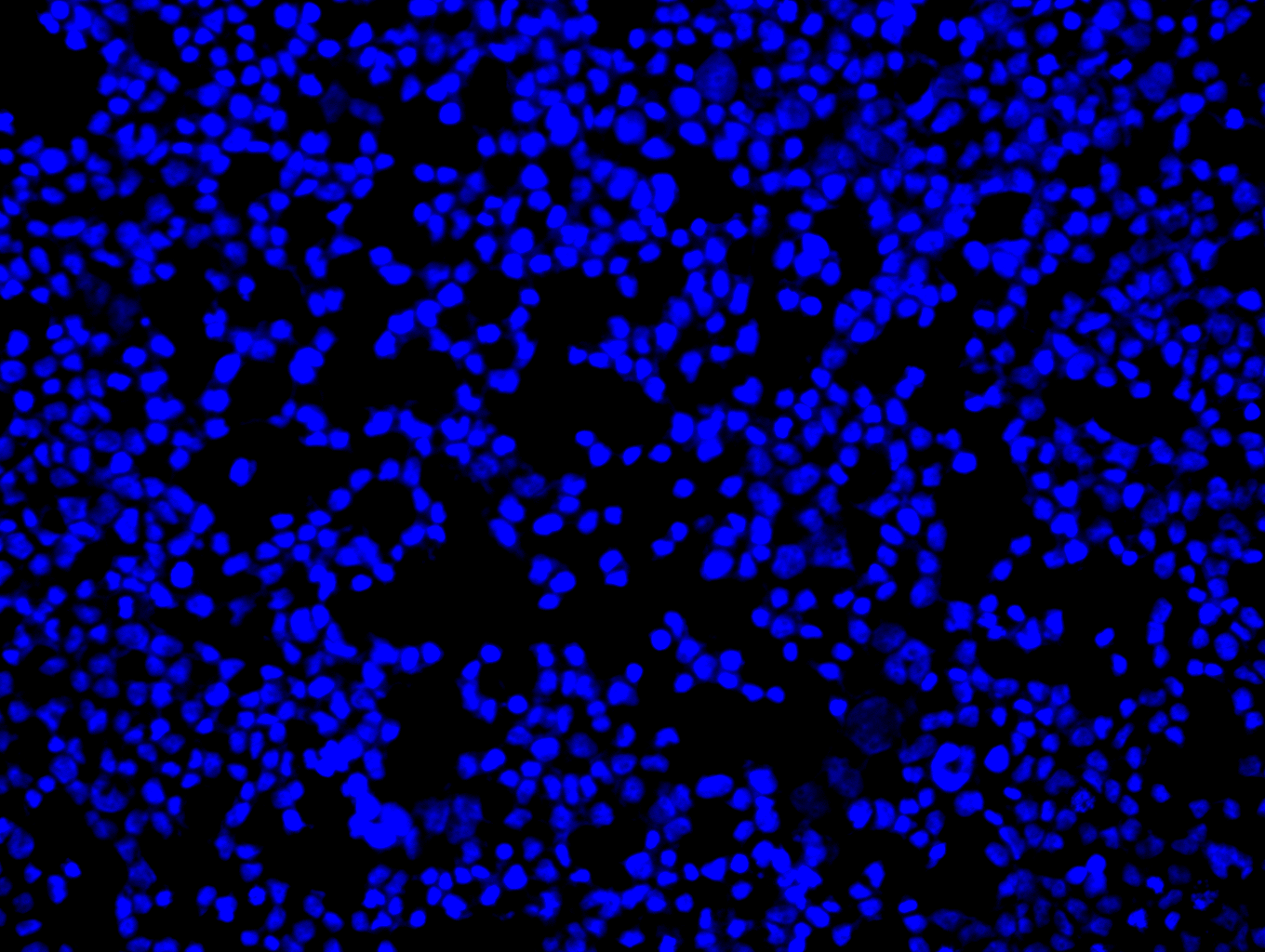

Supplement: Supplementary file 9 — Source data Fig. 4 [file 44318_2024_359_MOESM9_ESM.zip › Figure 4/Fig 4H and 4I/Fig 4H/hSPAR/1-hoechst in manu.tif]

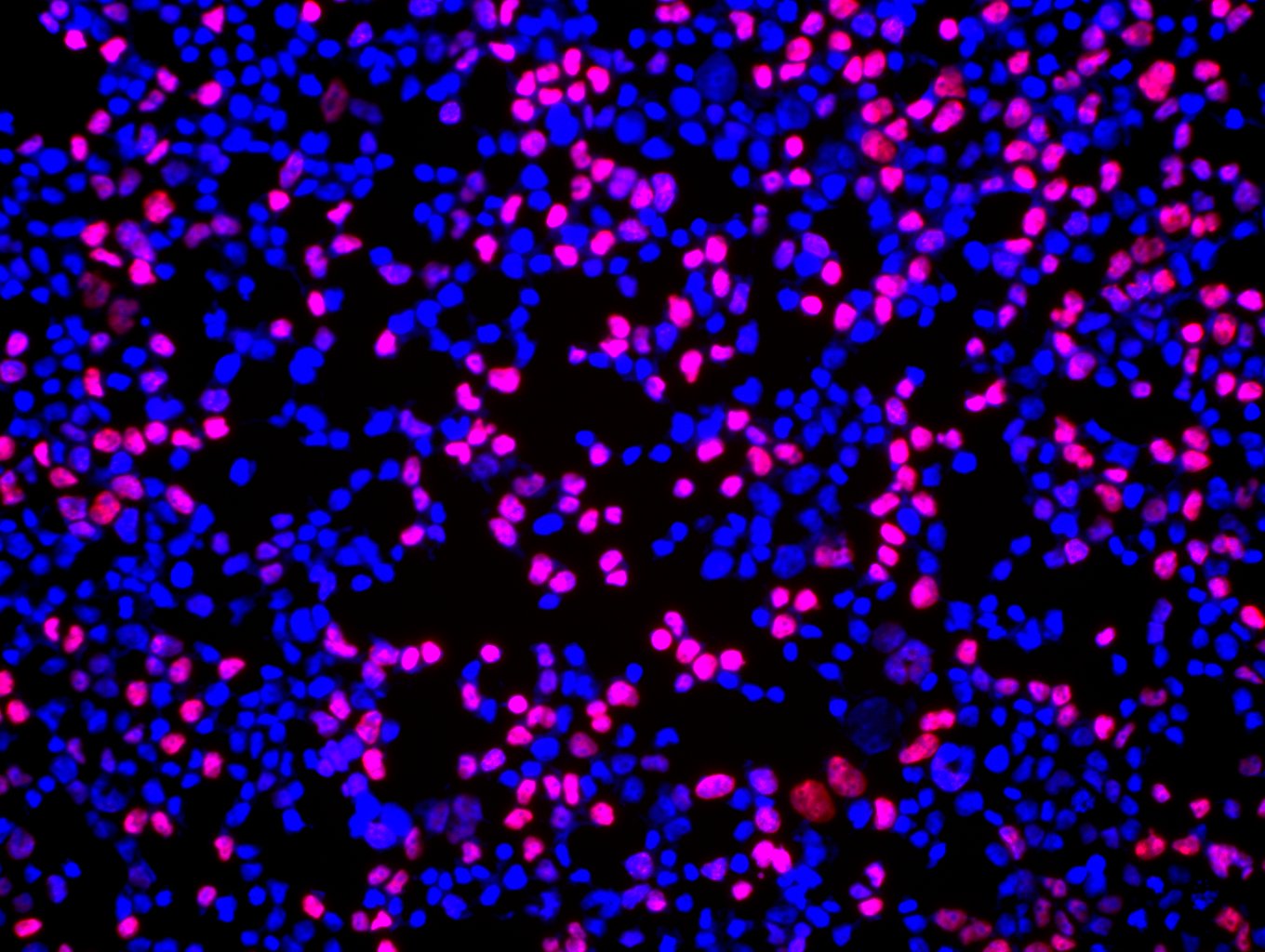

Supplement: Supplementary file 9 — Source data Fig. 4 [file 44318_2024_359_MOESM9_ESM.zip › Figure 4/Fig 4H and 4I/Fig 4H/hSPAR/1-merge in manu.jpg]

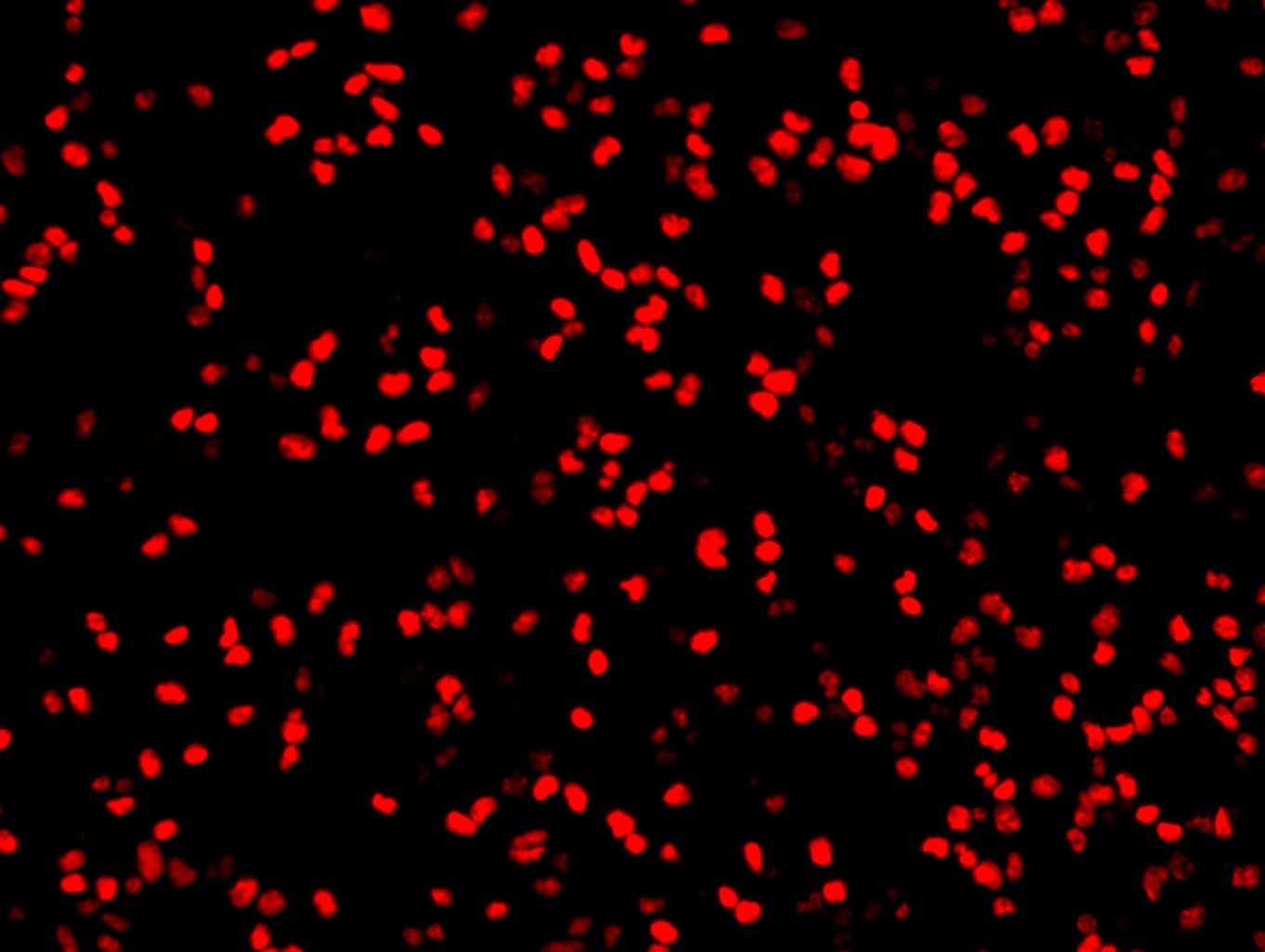

Supplement: Supplementary file 9 — Source data Fig. 4 [file 44318_2024_359_MOESM9_ESM.zip › Figure 4/Fig 4H and 4I/Fig 4H/hSPAR+siCtrl/1-edu(in manu).jpg]

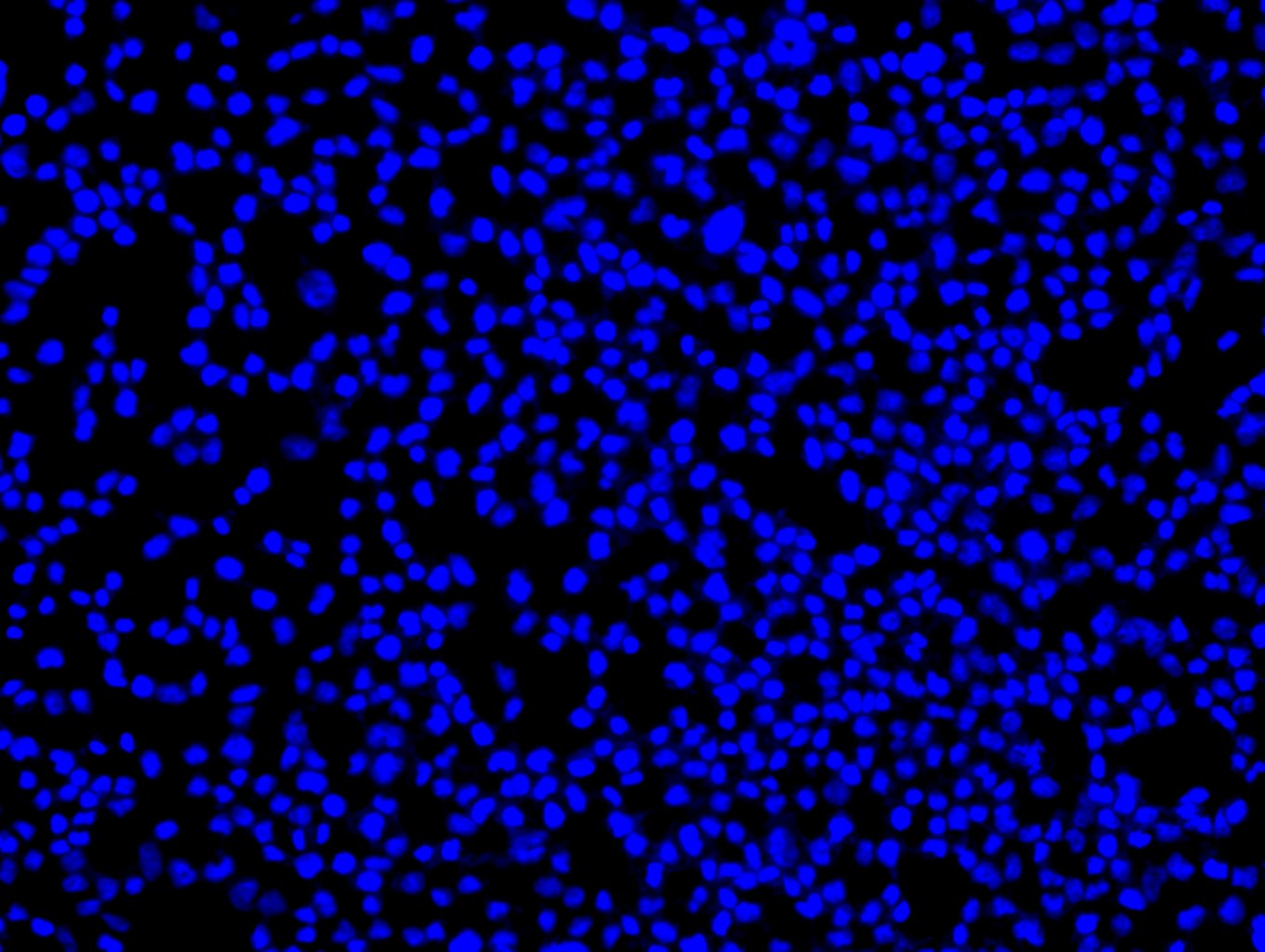

Supplement: Supplementary file 9 — Source data Fig. 4 [file 44318_2024_359_MOESM9_ESM.zip › Figure 4/Fig 4H and 4I/Fig 4H/hSPAR+siCtrl/1-hoechst(in manu).jpg]

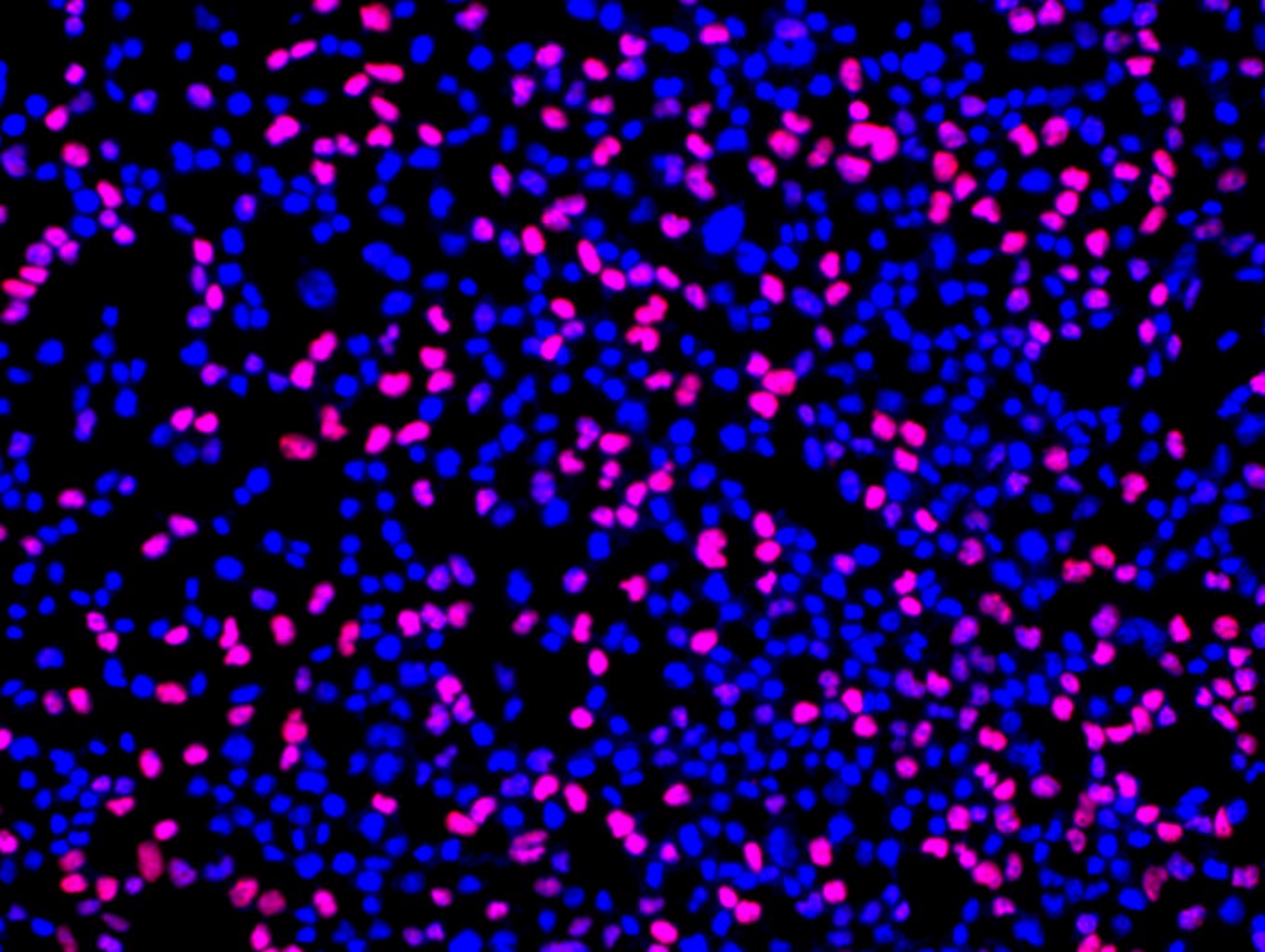

Supplement: Supplementary file 9 — Source data Fig. 4 [file 44318_2024_359_MOESM9_ESM.zip › Figure 4/Fig 4H and 4I/Fig 4H/hSPAR+siCtrl/1-merge(in manu).jpg]

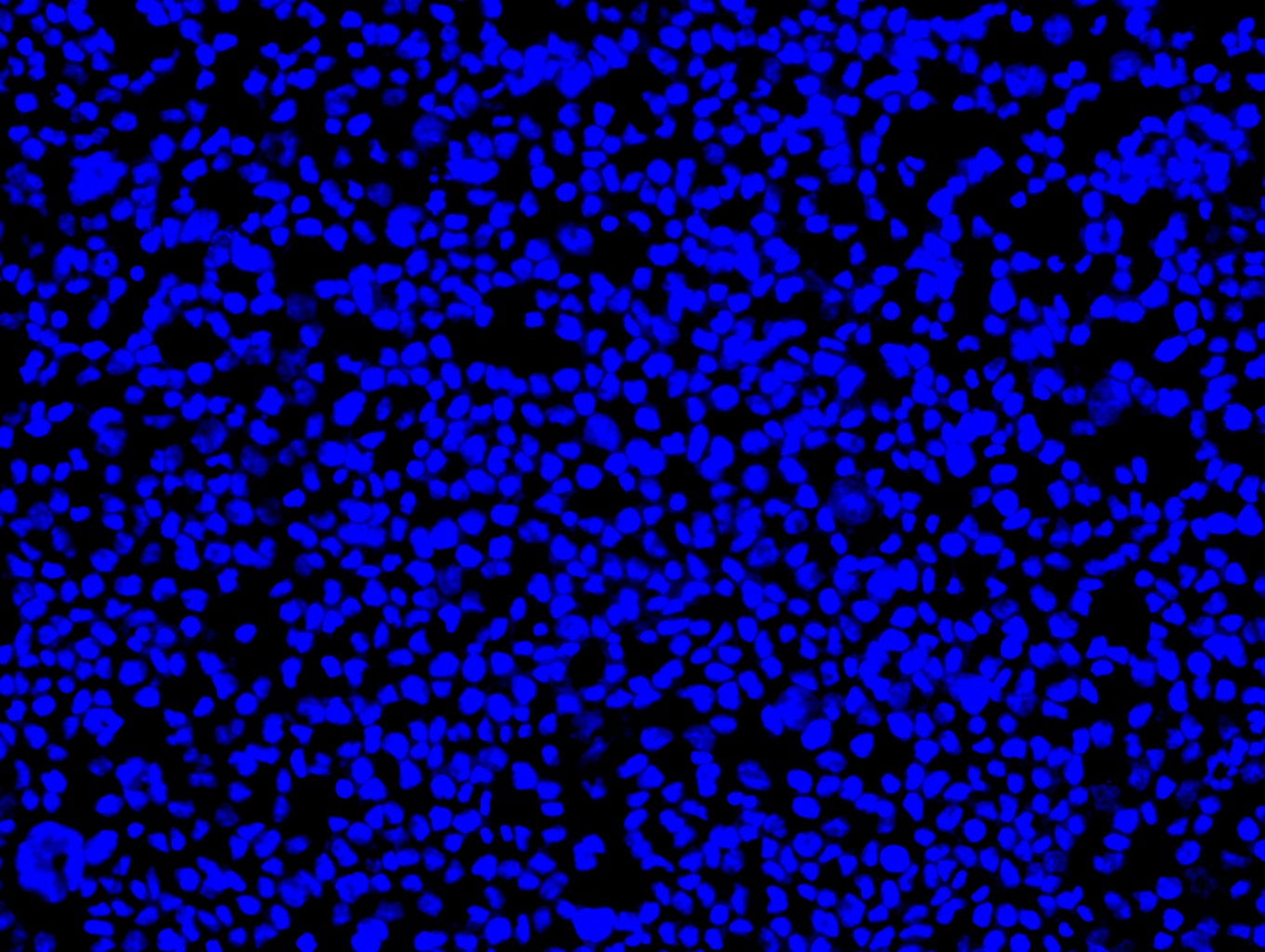

Supplement: Supplementary file 9 — Source data Fig. 4 [file 44318_2024_359_MOESM9_ESM.zip › Figure 4/Fig 4H and 4I/Fig 4H/hSPAR+siP27KIP-1/1-Hoechst(in manu).jpg]

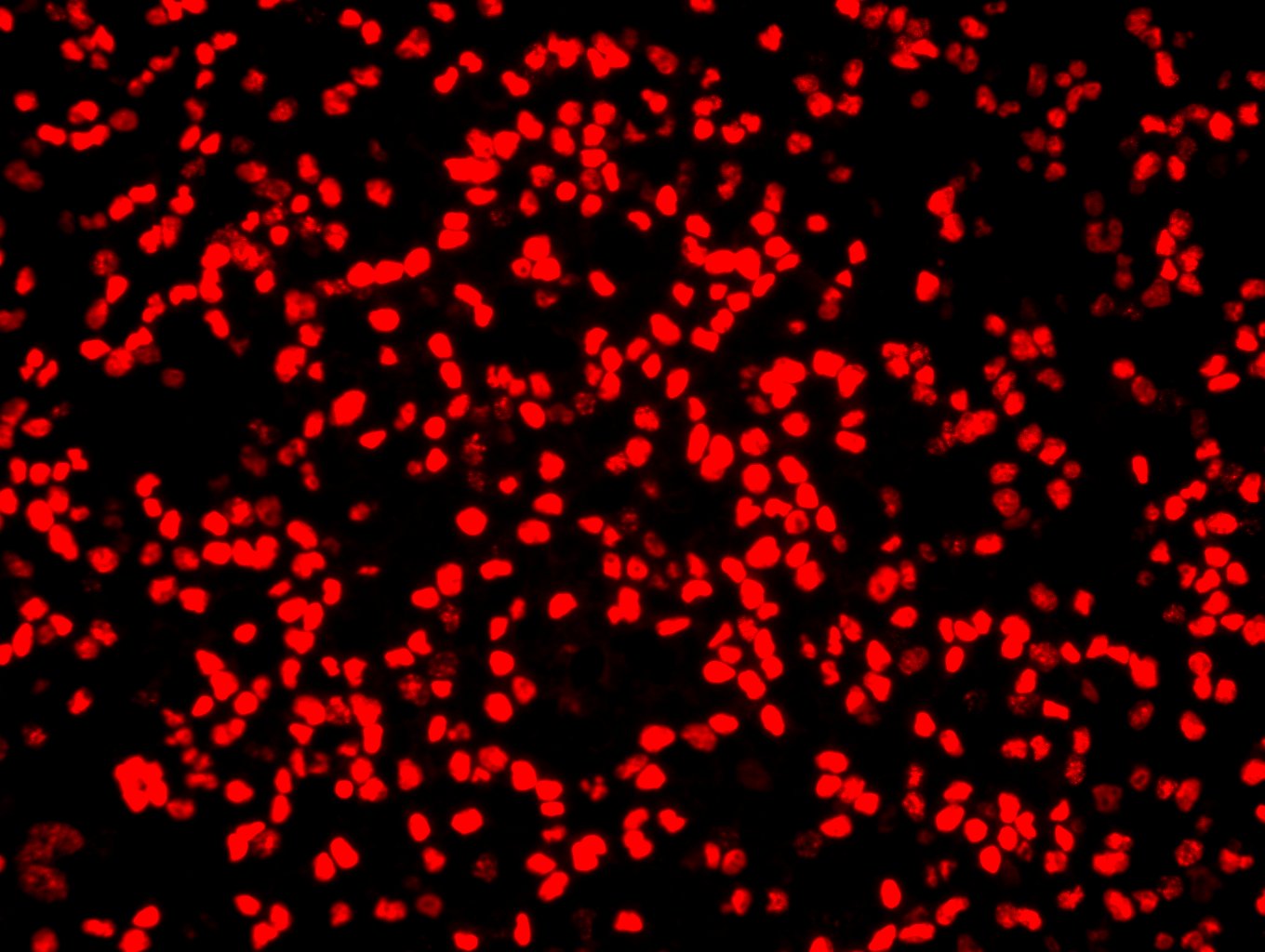

Supplement: Supplementary file 9 — Source data Fig. 4 [file 44318_2024_359_MOESM9_ESM.zip › Figure 4/Fig 4H and 4I/Fig 4H/hSPAR+siP27KIP-1/1-edu(in manu).jpg]

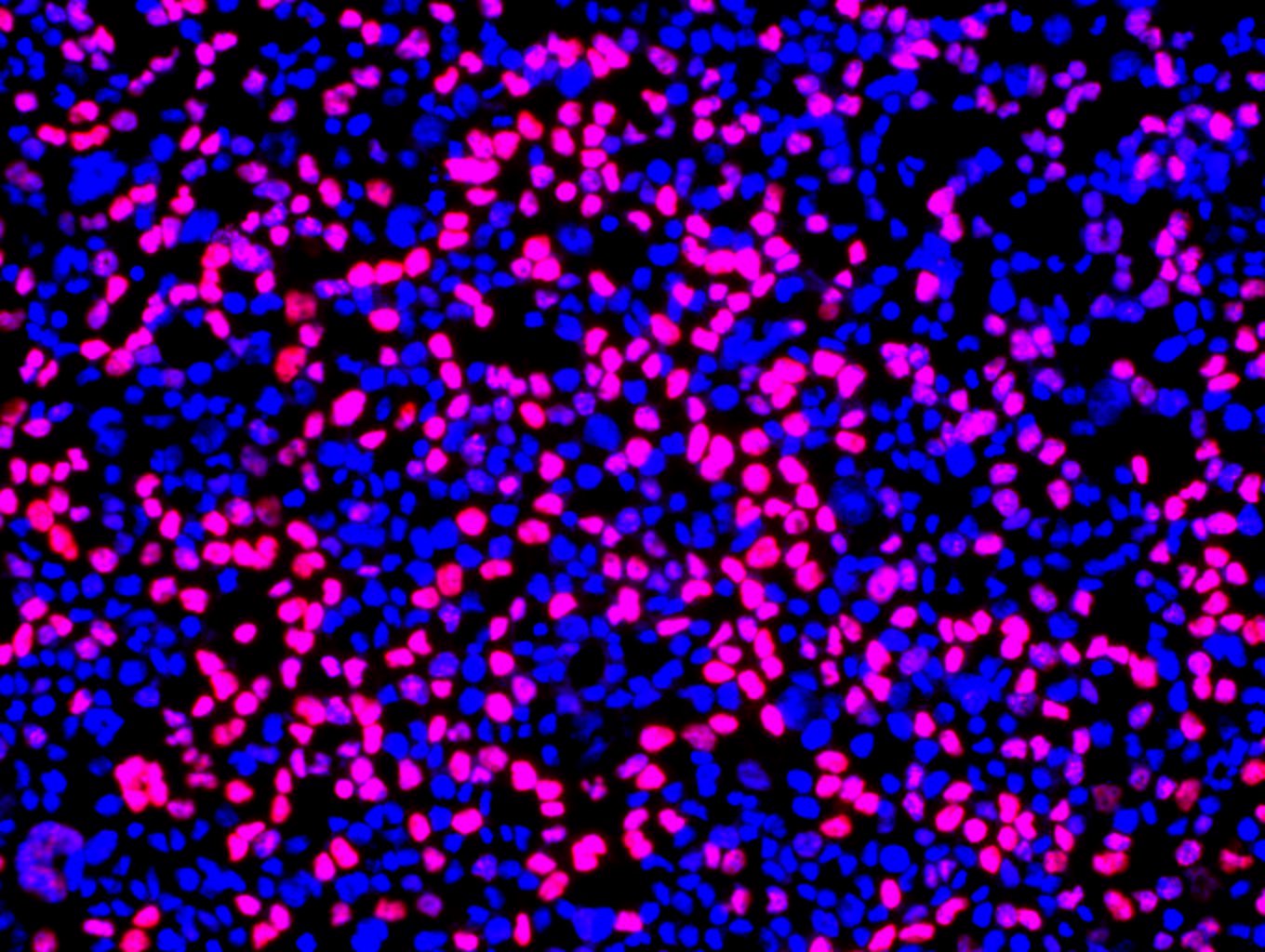

Supplement: Supplementary file 9 — Source data Fig. 4 [file 44318_2024_359_MOESM9_ESM.zip › Figure 4/Fig 4H and 4I/Fig 4H/hSPAR+siP27KIP-1/1-merge(in manu).jpg]

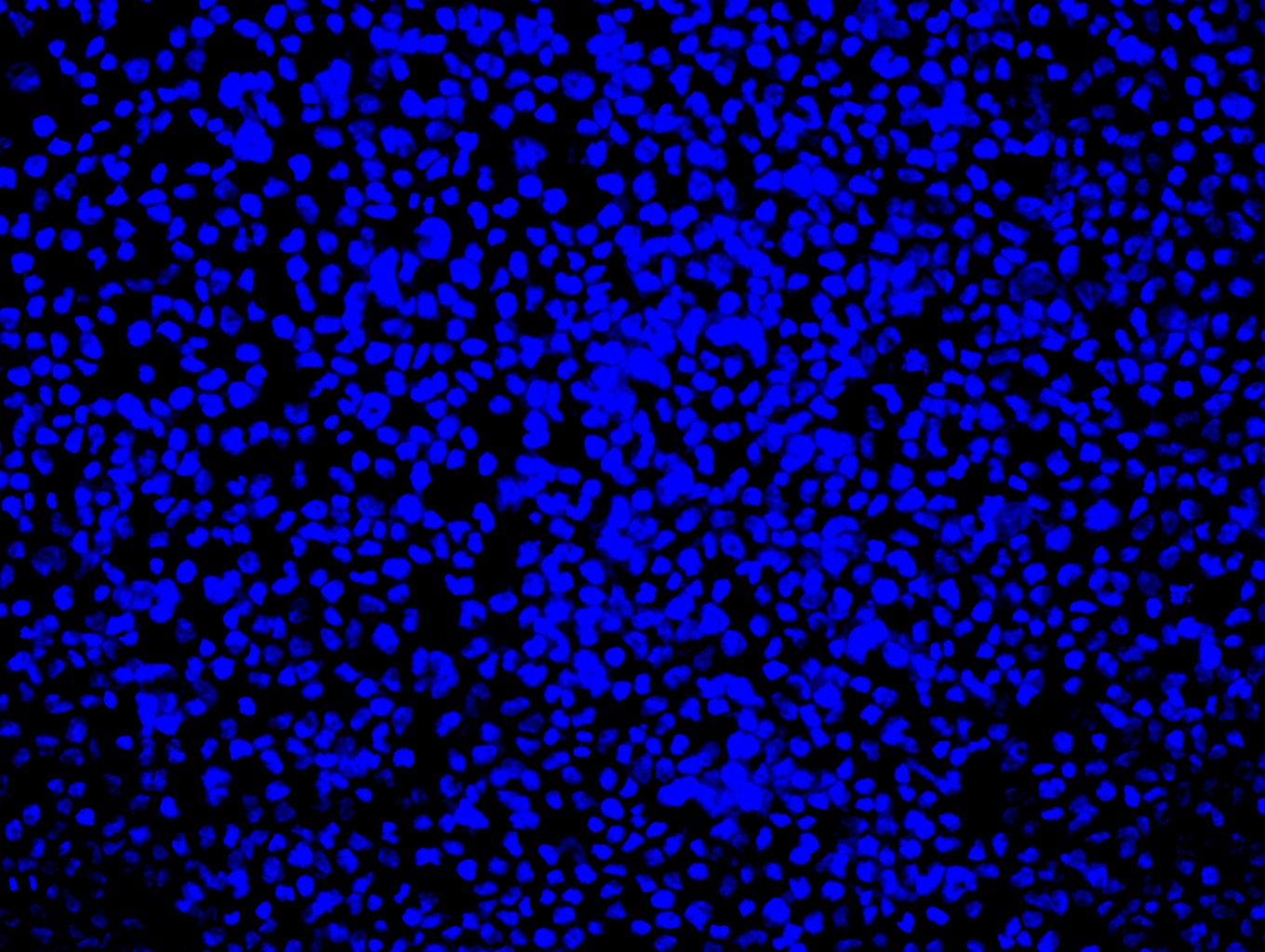

Supplement: Supplementary file 9 — Source data Fig. 4 [file 44318_2024_359_MOESM9_ESM.zip › Figure 4/Fig 4H and 4I/Fig 4H/hSPAR+siP27KIP1-2/1-Hoechst(in manu).jpg]

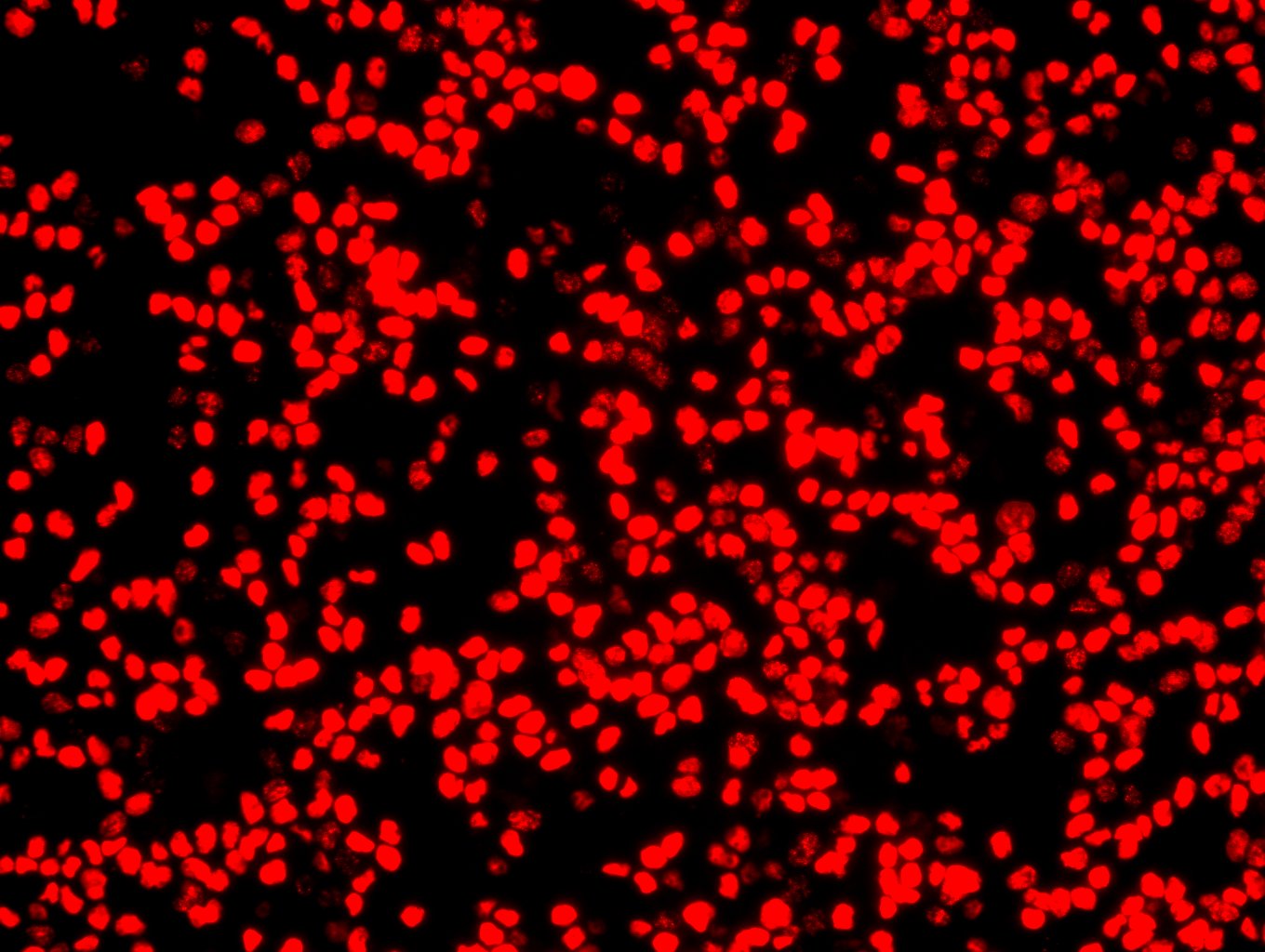

Supplement: Supplementary file 9 — Source data Fig. 4 [file 44318_2024_359_MOESM9_ESM.zip › Figure 4/Fig 4H and 4I/Fig 4H/hSPAR+siP27KIP1-2/1-edu(in manu).jpg]

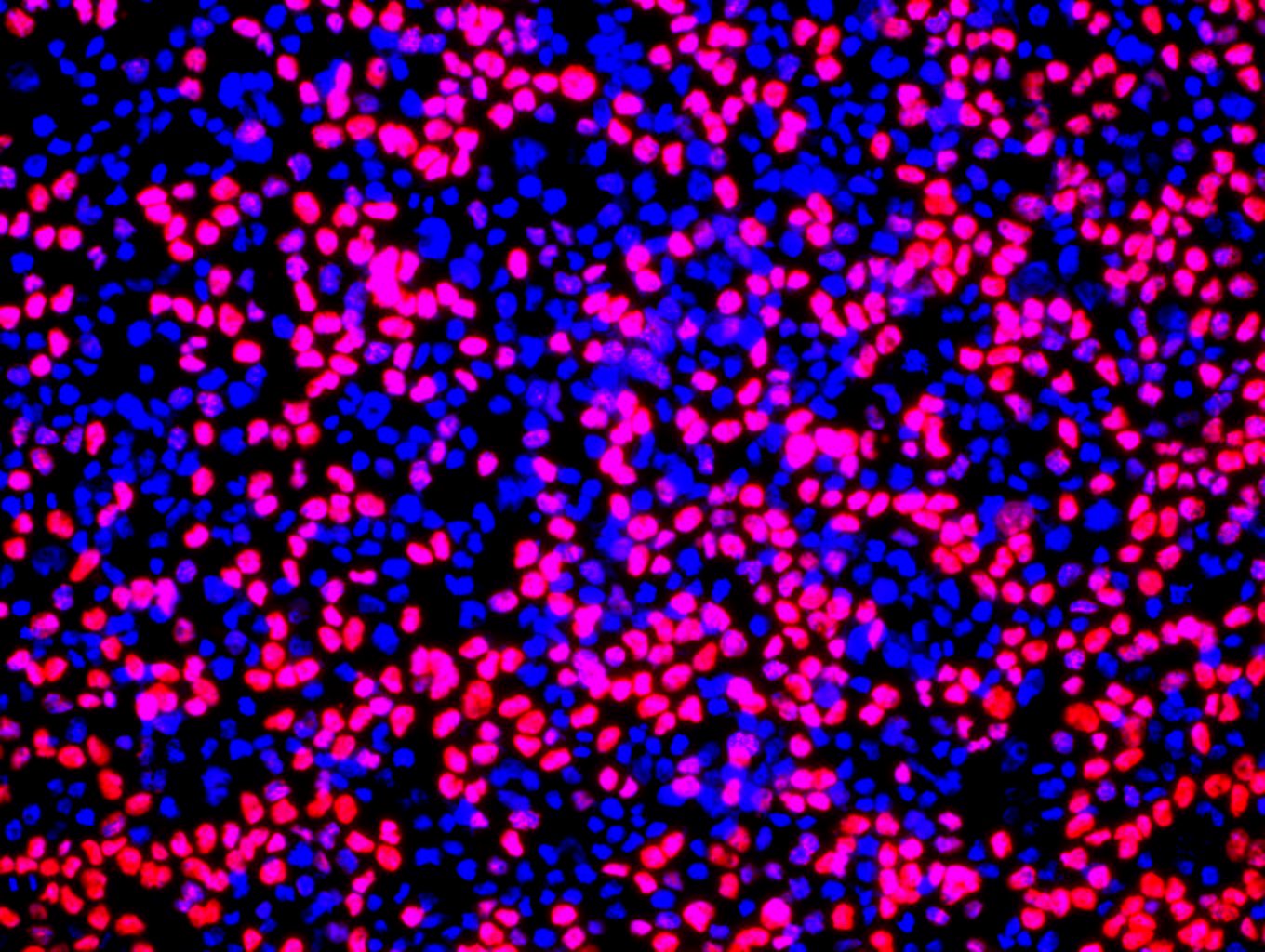

Supplement: Supplementary file 9 — Source data Fig. 4 [file 44318_2024_359_MOESM9_ESM.zip › Figure 4/Fig 4H and 4I/Fig 4H/hSPAR+siP27KIP1-2/1-merge(in manu).jpg]

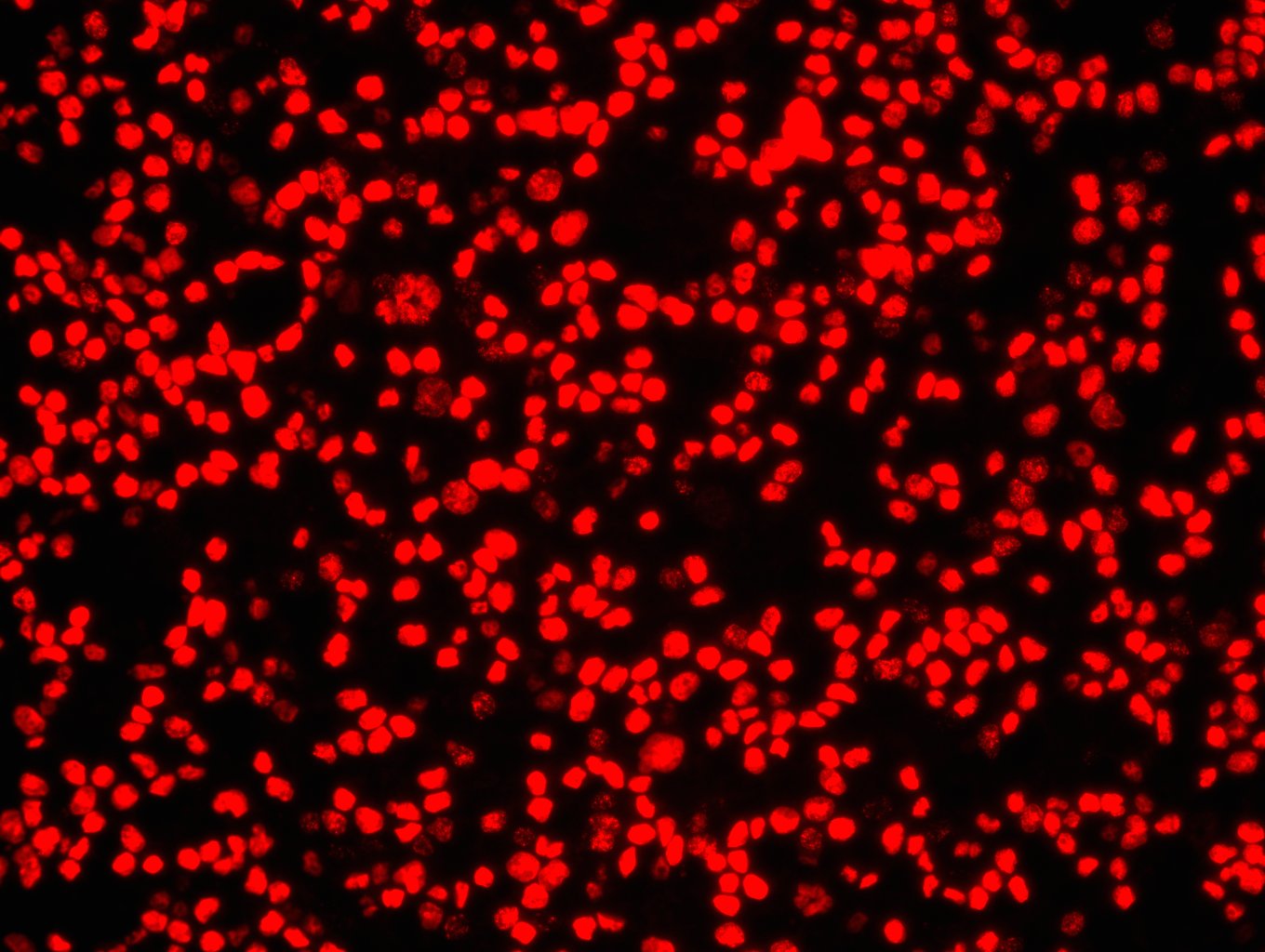

Supplement: Supplementary file 9 — Source data Fig. 4 [file 44318_2024_359_MOESM9_ESM.zip › Figure 4/Fig 4H and 4I/Fig 4H/hSPAR+siP27KIP1-3/1-edu( in manu).jpg]

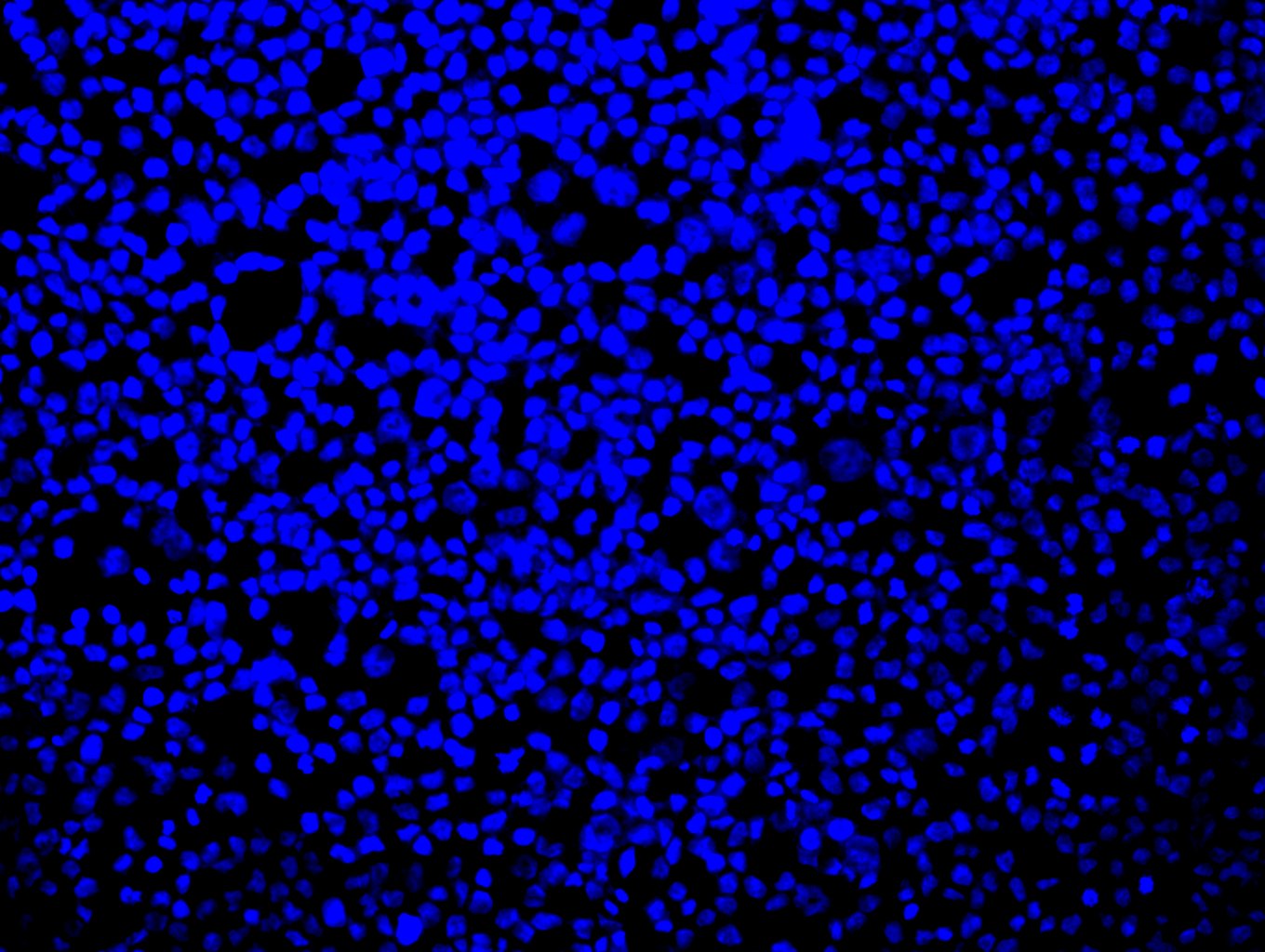

Supplement: Supplementary file 9 — Source data Fig. 4 [file 44318_2024_359_MOESM9_ESM.zip › Figure 4/Fig 4H and 4I/Fig 4H/hSPAR+siP27KIP1-3/1-hoechst(in manu).jpg]

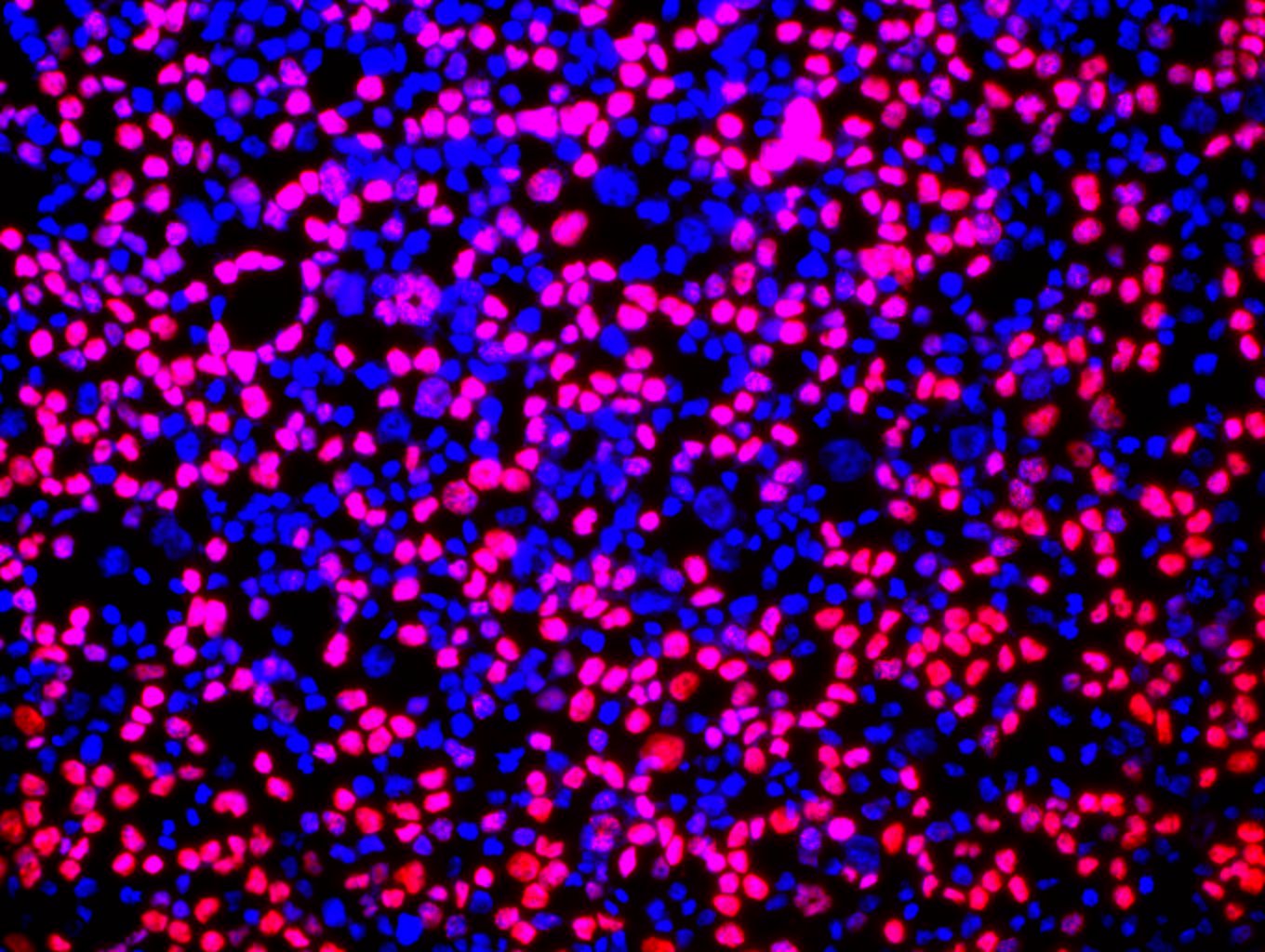

Supplement: Supplementary file 9 — Source data Fig. 4 [file 44318_2024_359_MOESM9_ESM.zip › Figure 4/Fig 4H and 4I/Fig 4H/hSPAR+siP27KIP1-3/1-merge(in manu).jpg]

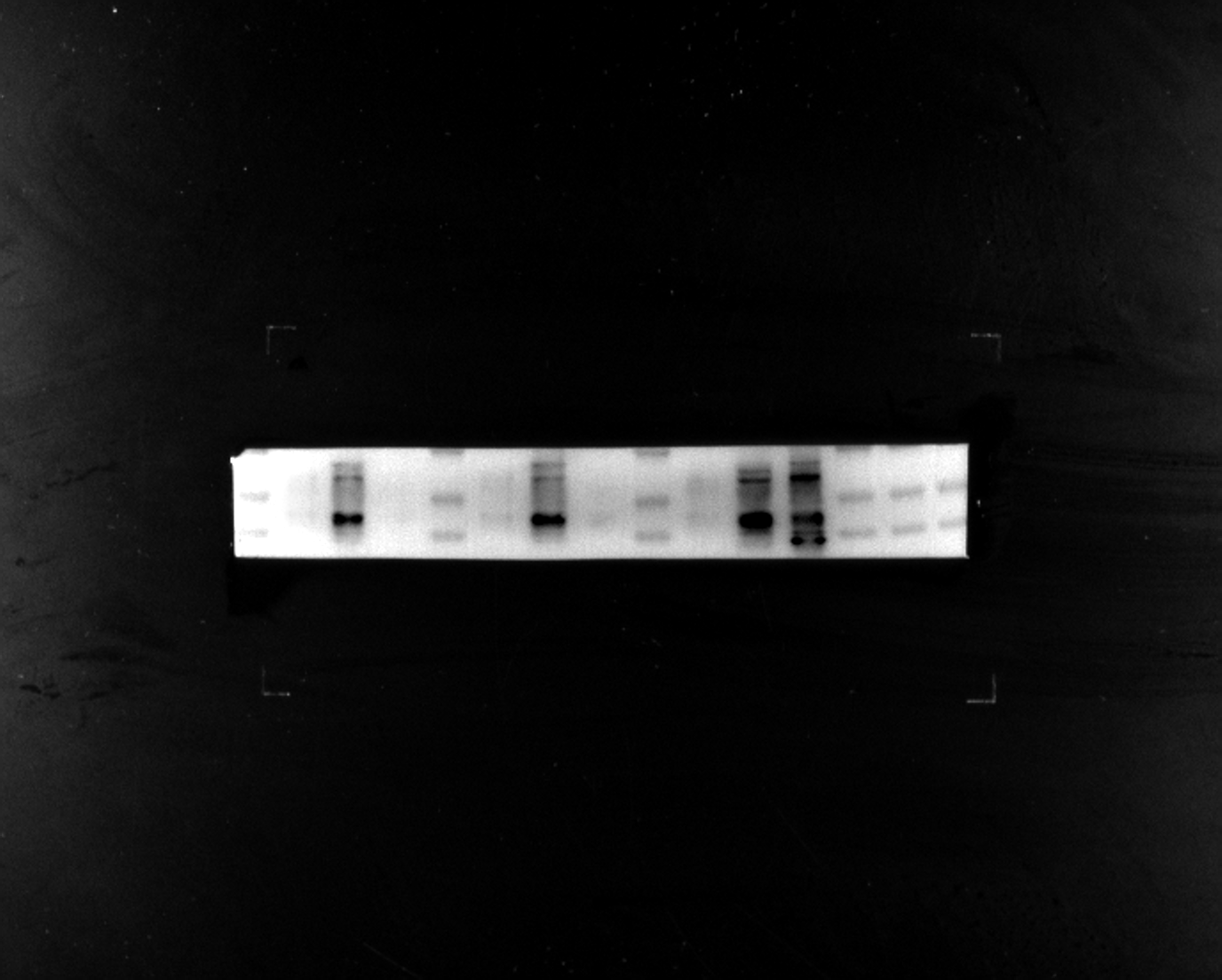

Supplement: Supplementary file 10 — Source data Fig. 5 [file 44318_2024_359_MOESM10_ESM.zip › Figure 5/Fig 5A/1-p27-merge.Tif]

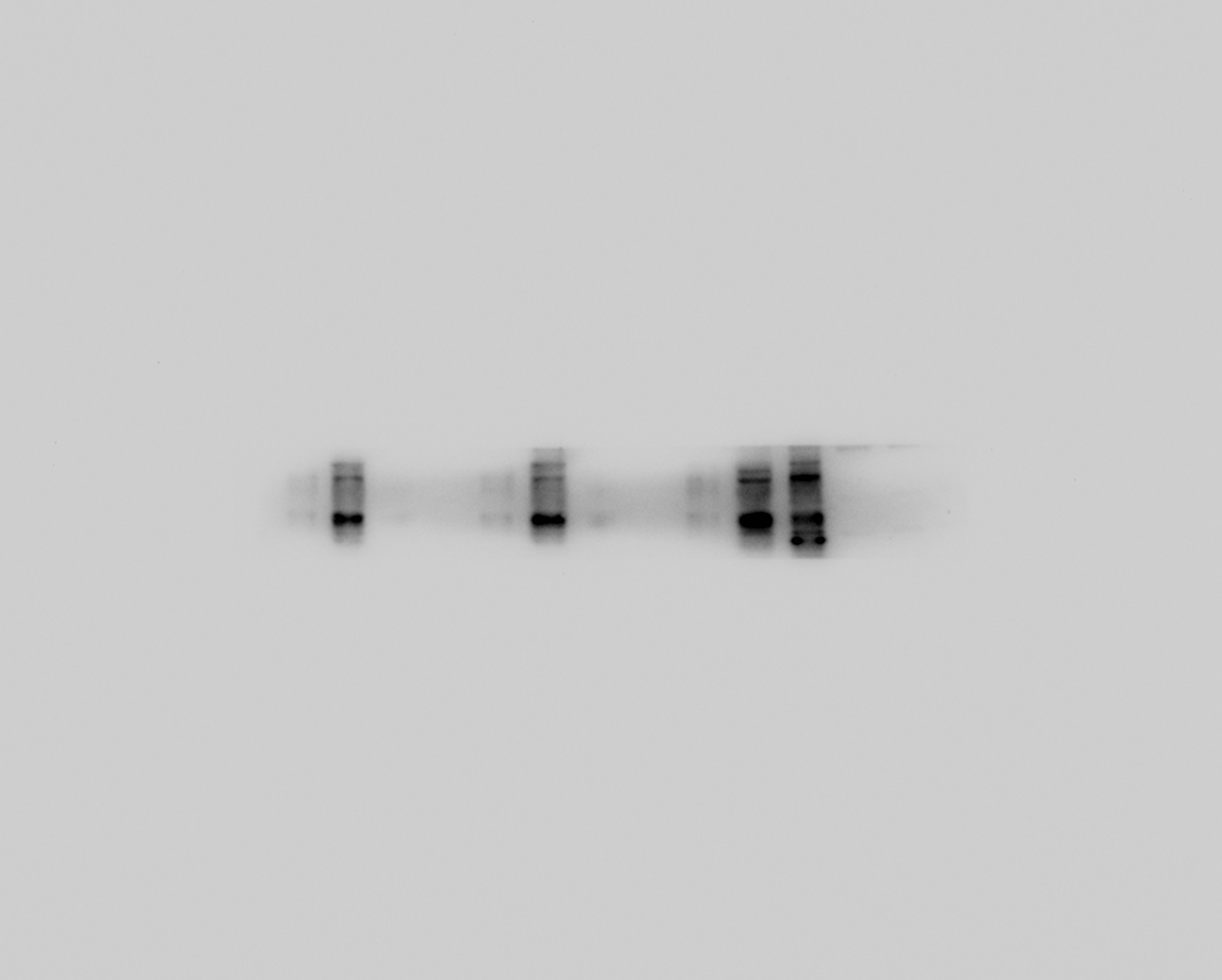

Supplement: Supplementary file 10 — Source data Fig. 5 [file 44318_2024_359_MOESM10_ESM.zip › Figure 5/Fig 5A/1-p27.Tif]

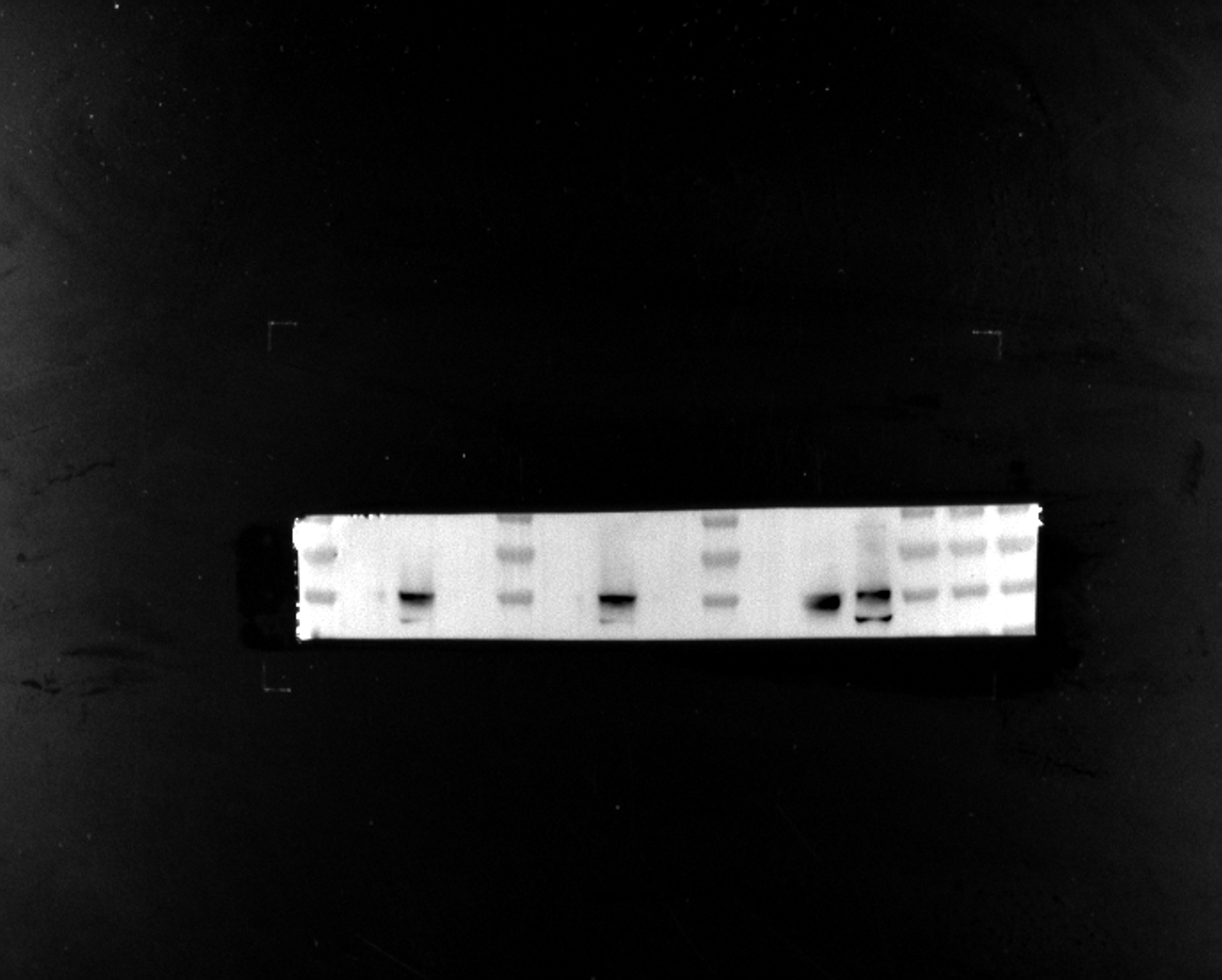

Supplement: Supplementary file 10 — Source data Fig. 5 [file 44318_2024_359_MOESM10_ESM.zip › Figure 5/Fig 5A/2-TRIM21-merge.Tif]

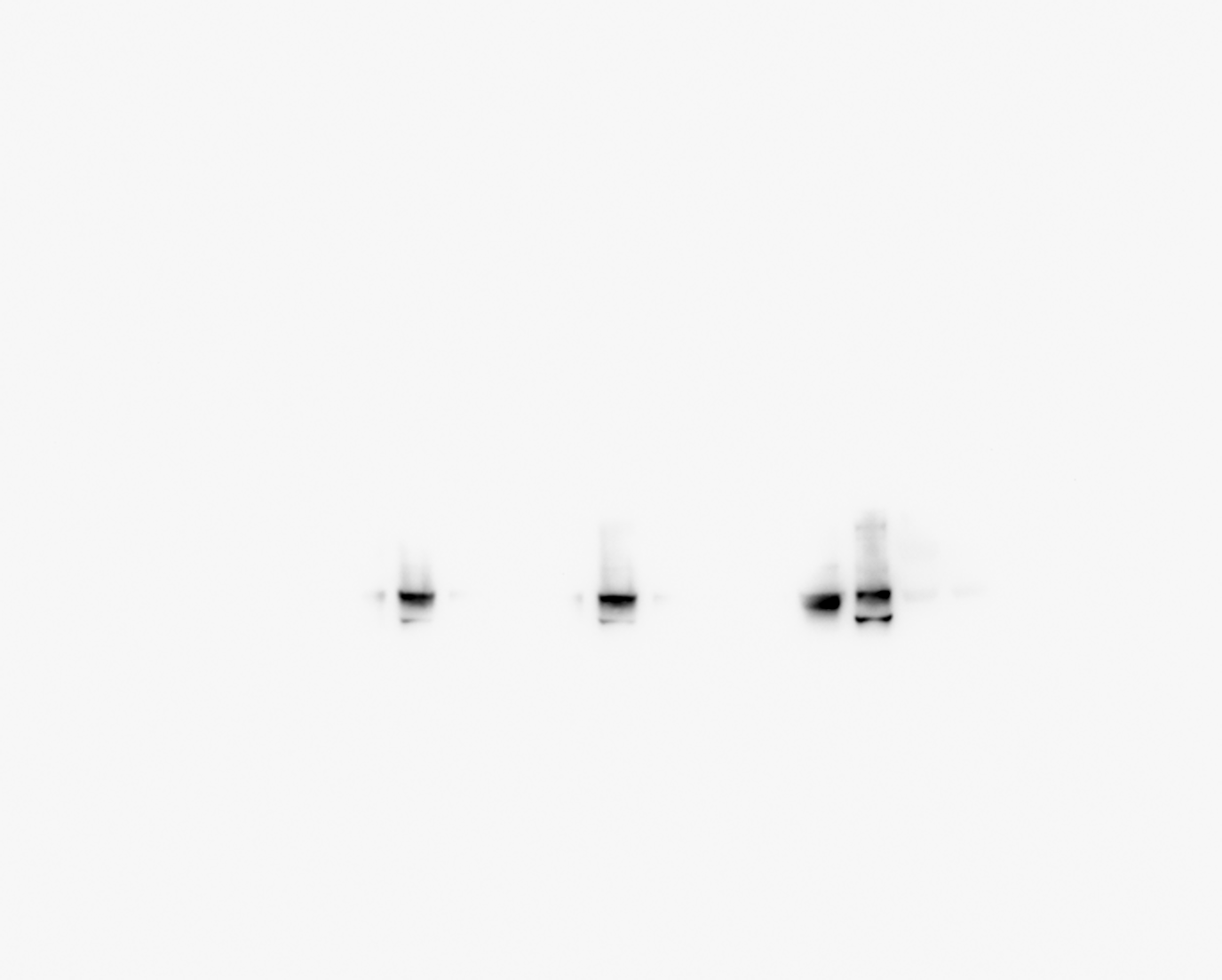

Supplement: Supplementary file 10 — Source data Fig. 5 [file 44318_2024_359_MOESM10_ESM.zip › Figure 5/Fig 5A/2-TRIM21.Tif]

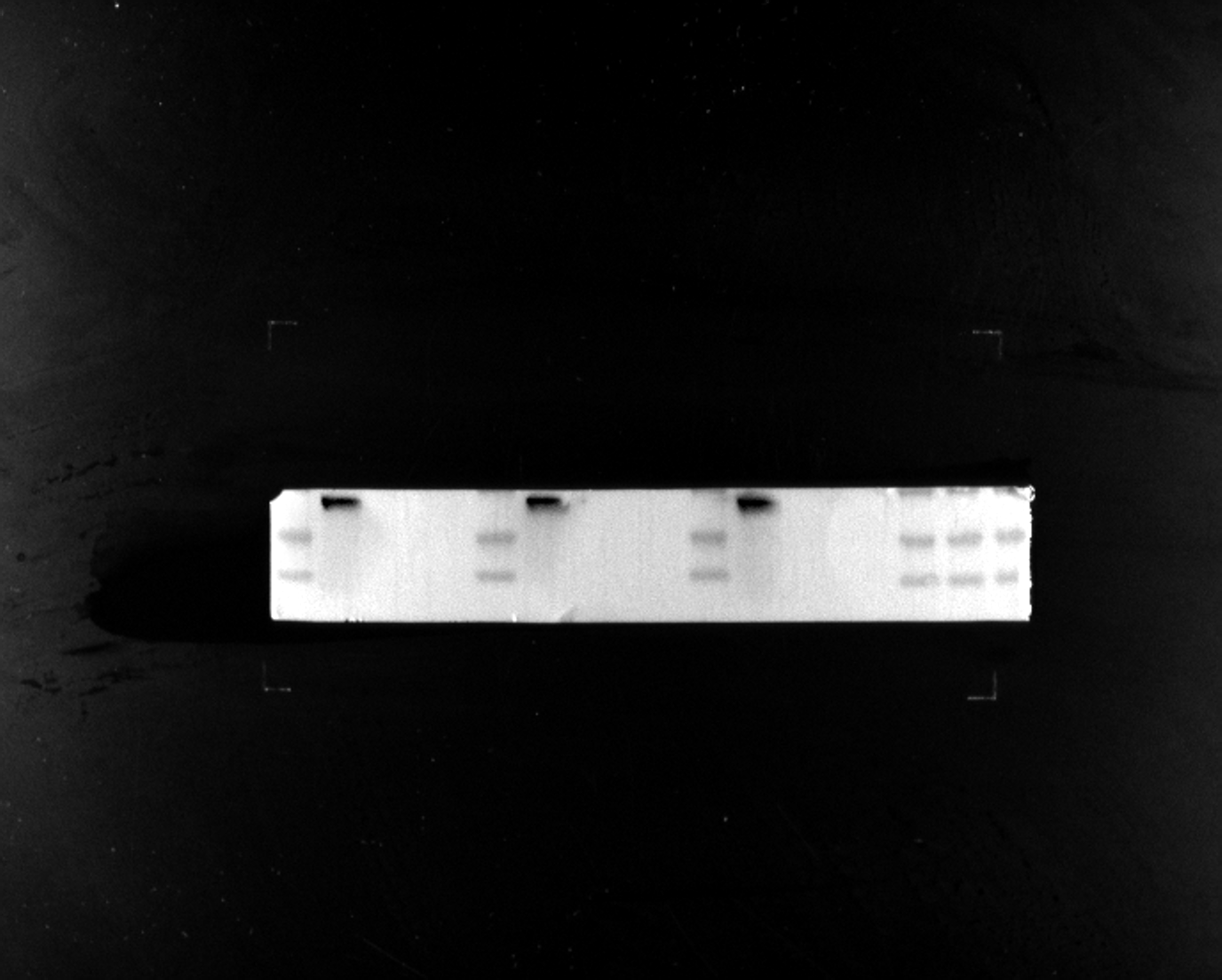

Supplement: Supplementary file 10 — Source data Fig. 5 [file 44318_2024_359_MOESM10_ESM.zip › Figure 5/Fig 5A/3-FBL-merge.Tif]

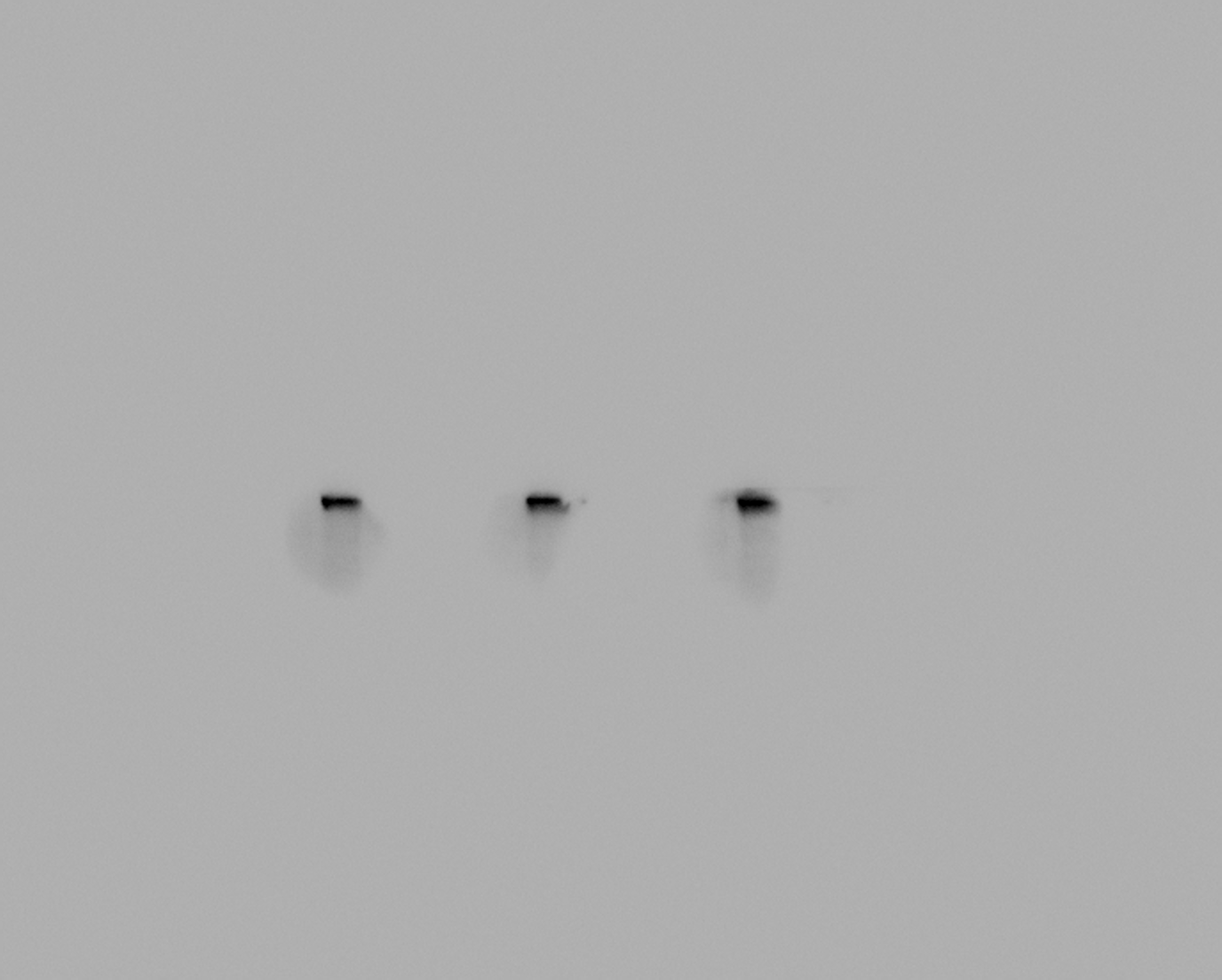

Supplement: Supplementary file 10 — Source data Fig. 5 [file 44318_2024_359_MOESM10_ESM.zip › Figure 5/Fig 5A/3-FBL.Tif]

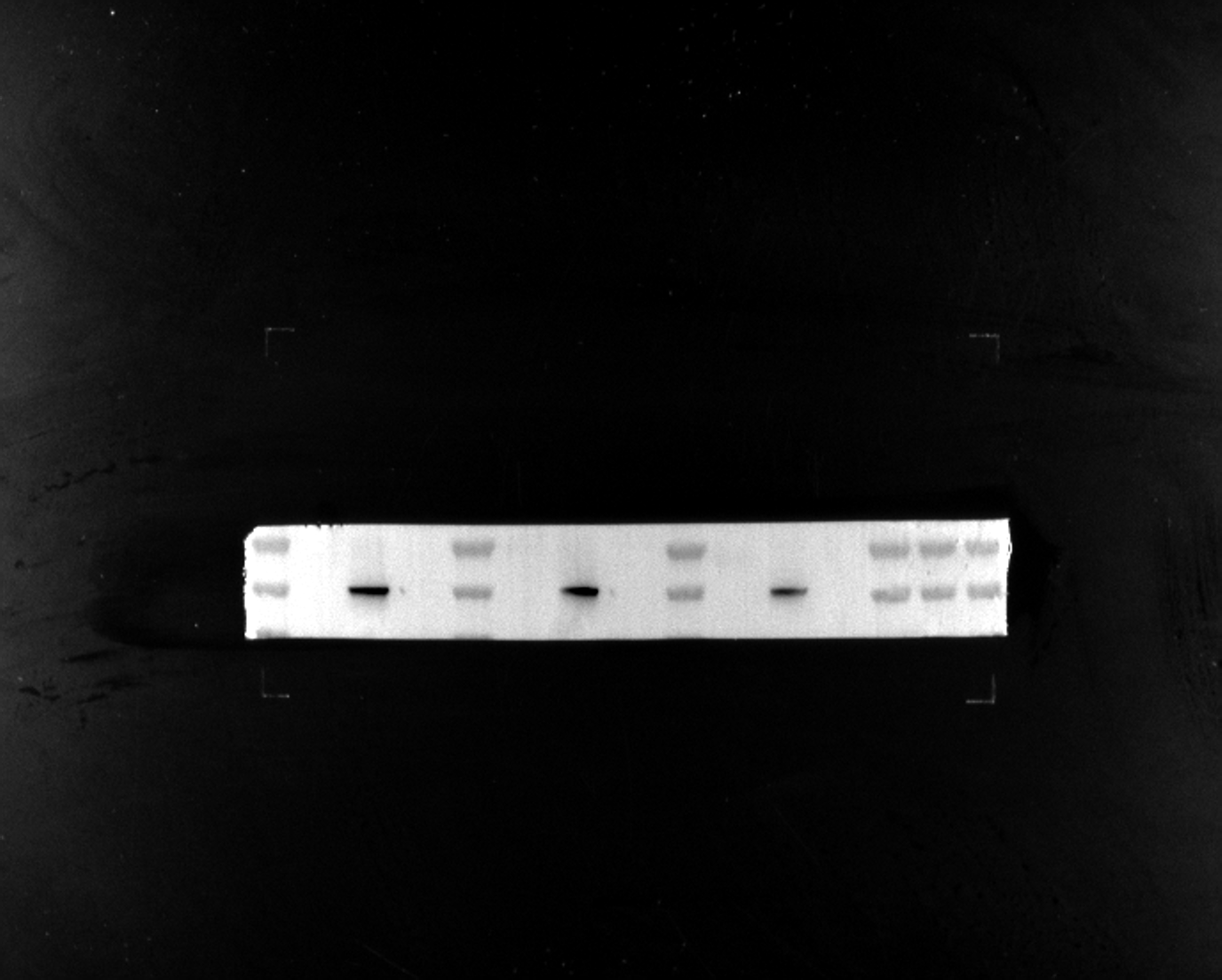

Supplement: Supplementary file 10 — Source data Fig. 5 [file 44318_2024_359_MOESM10_ESM.zip › Figure 5/Fig 5A/4-TUBULIN-merge.Tif]

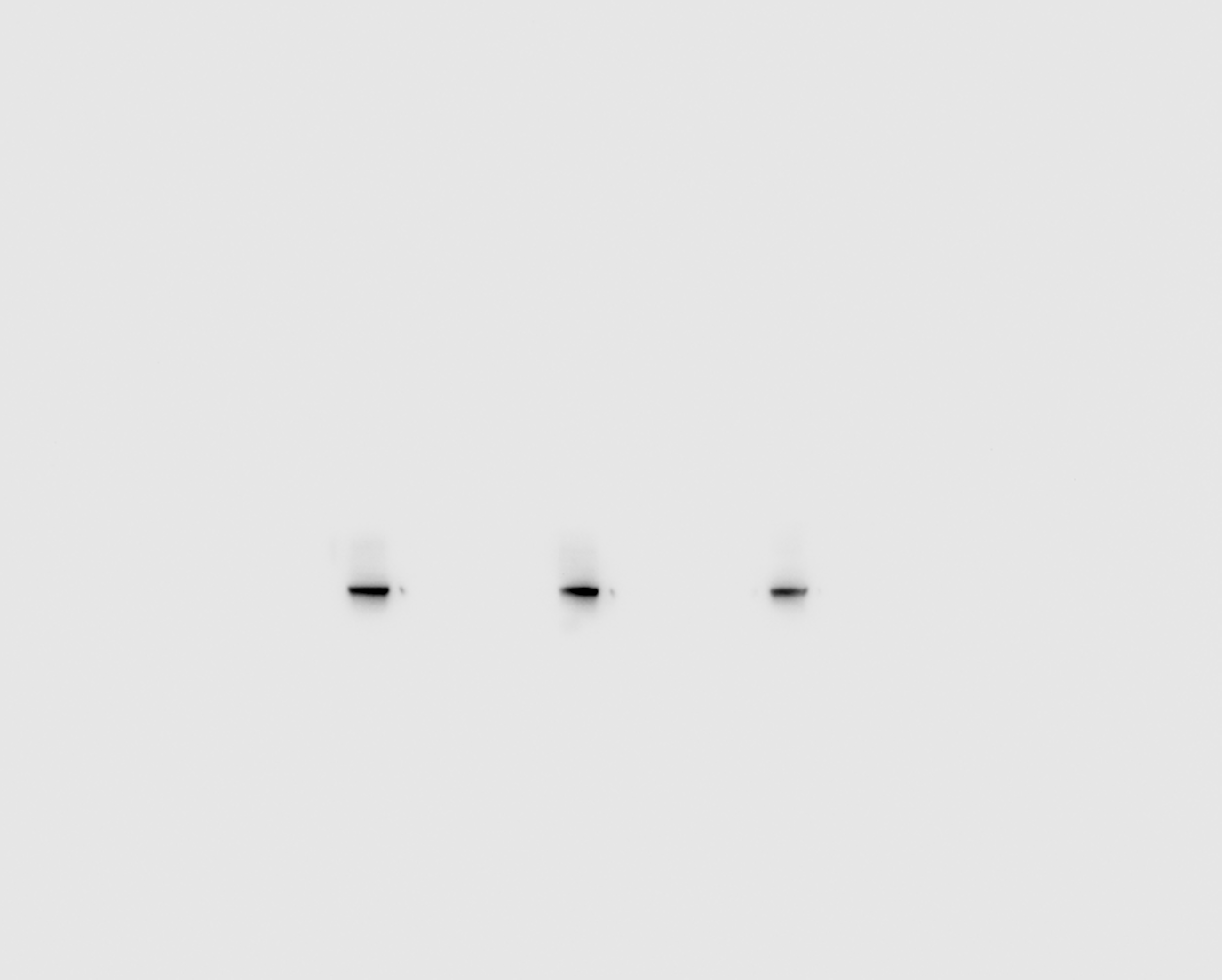

Supplement: Supplementary file 10 — Source data Fig. 5 [file 44318_2024_359_MOESM10_ESM.zip › Figure 5/Fig 5A/4-TUBULIN.Tif]

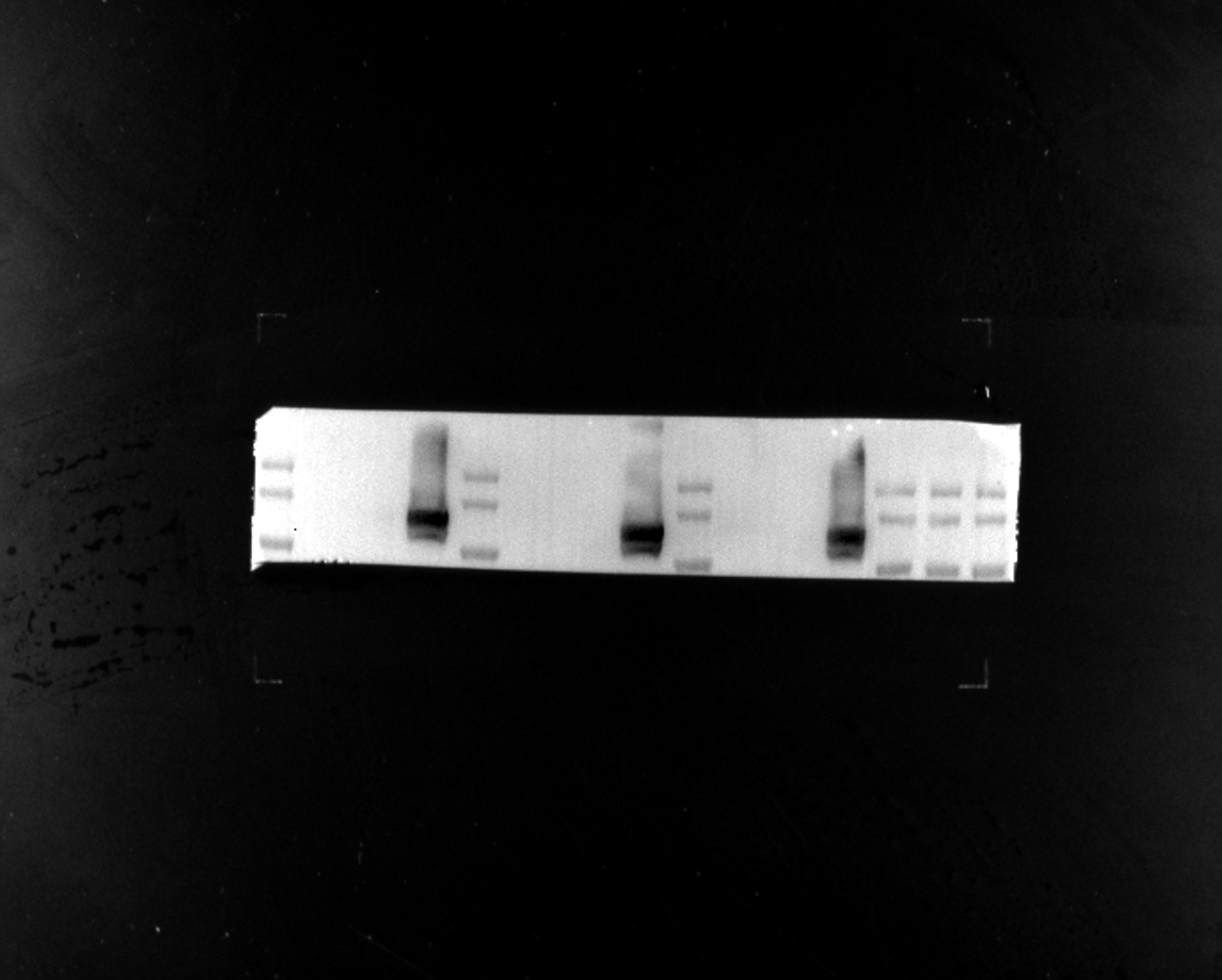

Supplement: Supplementary file 10 — Source data Fig. 5 [file 44318_2024_359_MOESM10_ESM.zip › Figure 5/Fig 5A/5-ATP1V1A-merge.Tif]

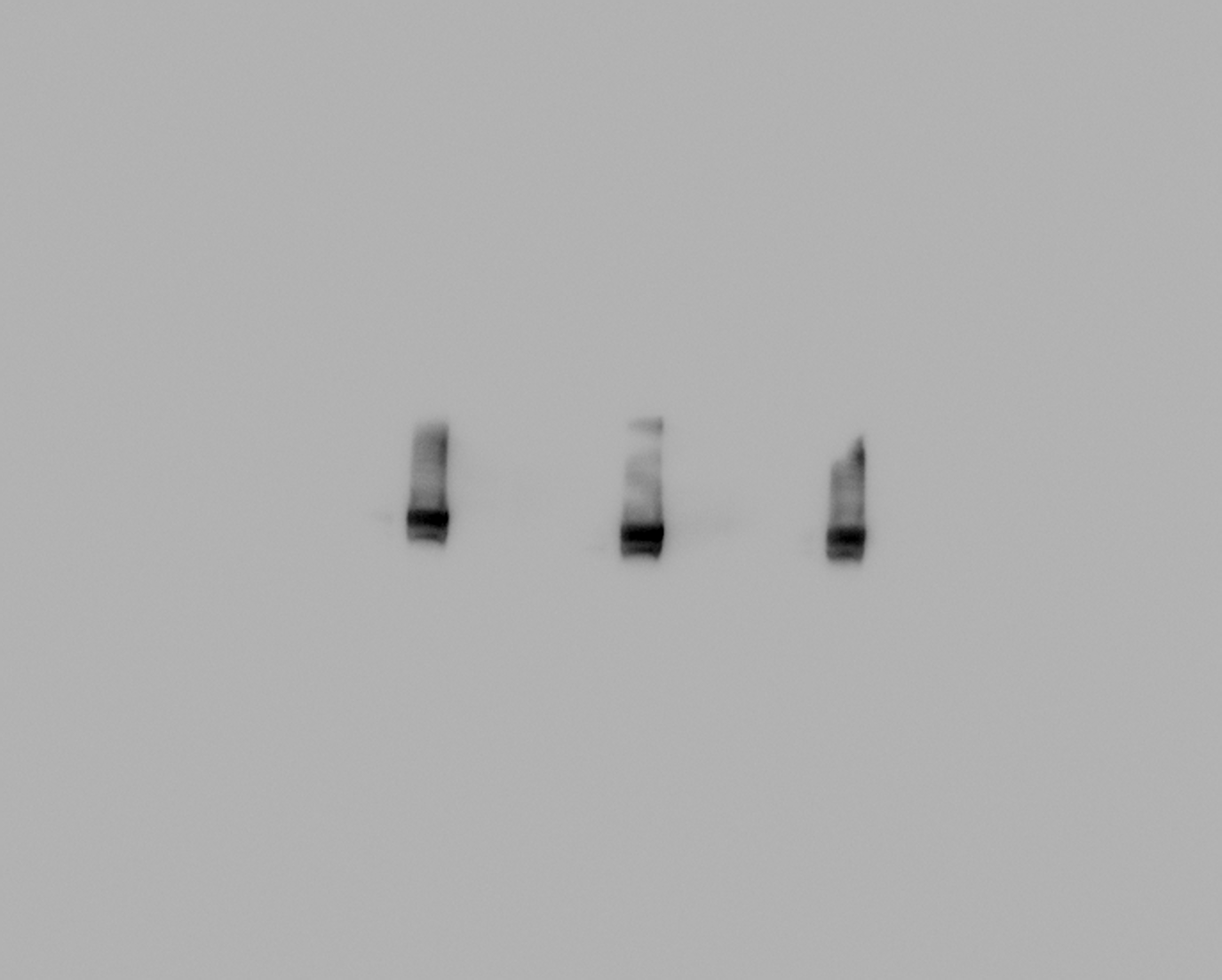

Supplement: Supplementary file 10 — Source data Fig. 5 [file 44318_2024_359_MOESM10_ESM.zip › Figure 5/Fig 5A/5-ATP1V1A.Tif]

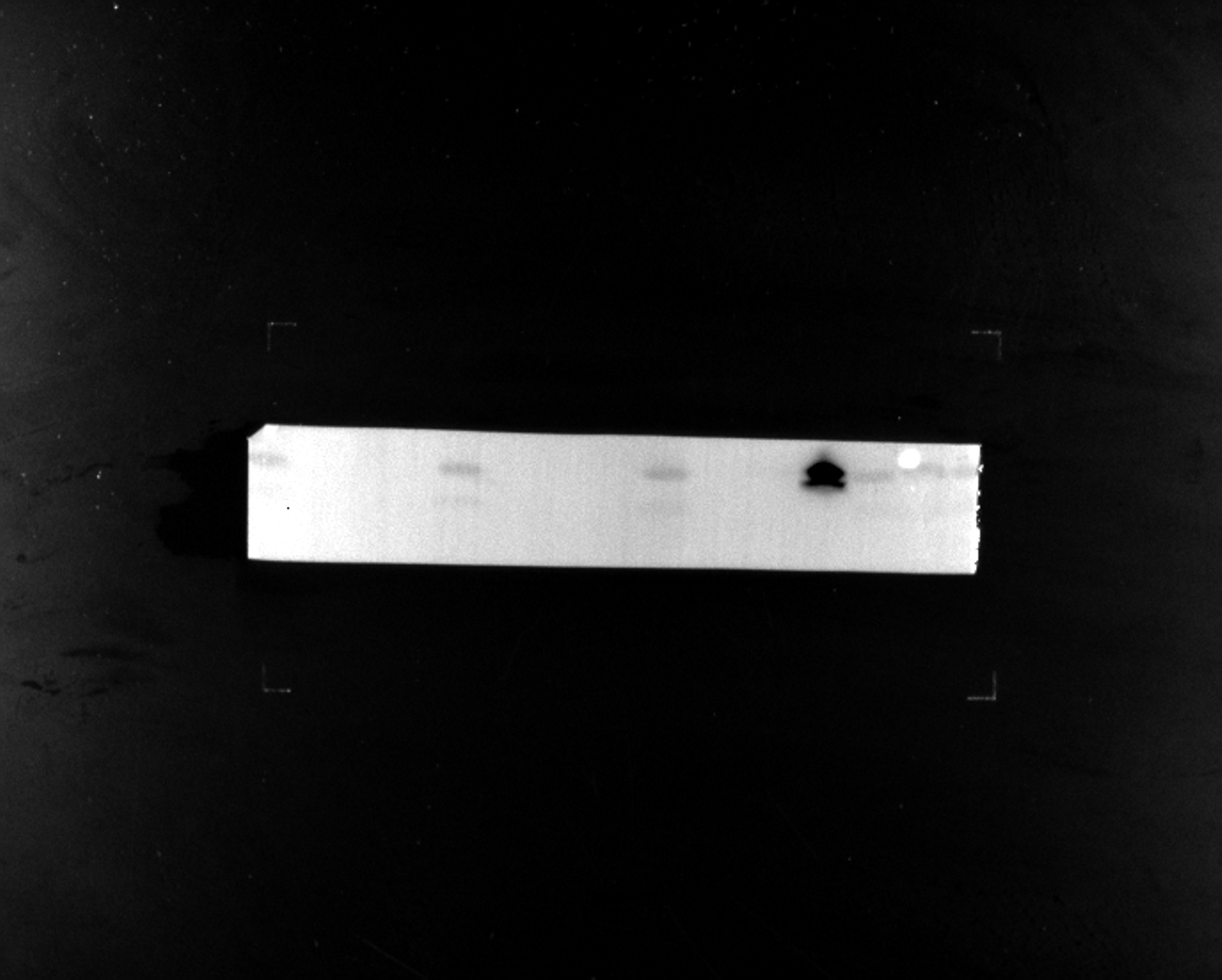

Supplement: Supplementary file 10 — Source data Fig. 5 [file 44318_2024_359_MOESM10_ESM.zip › Figure 5/Fig 5A/6-Flag-merge.Tif]

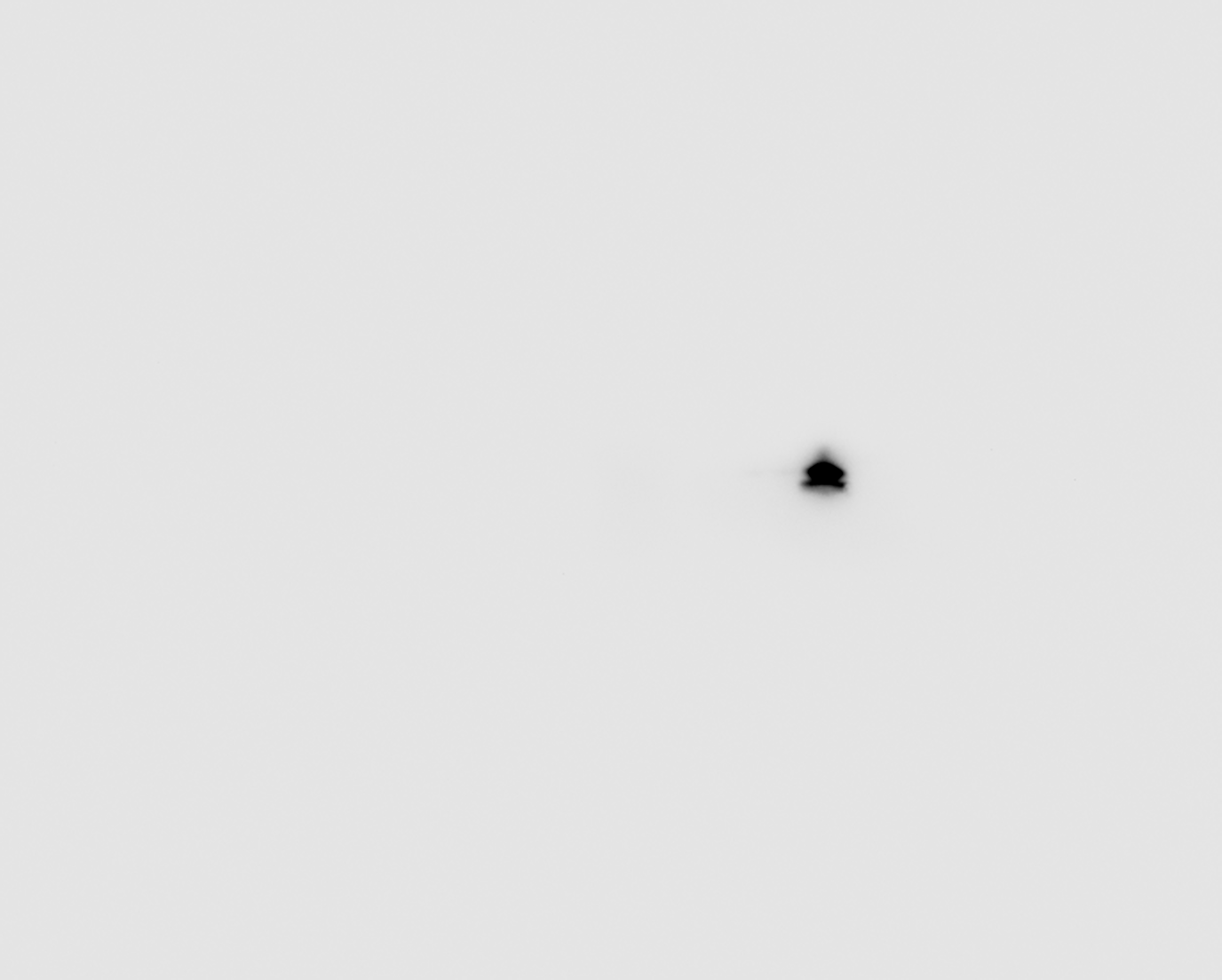

Supplement: Supplementary file 10 — Source data Fig. 5 [file 44318_2024_359_MOESM10_ESM.zip › Figure 5/Fig 5A/6-Flag.Tif]

Fig 5A

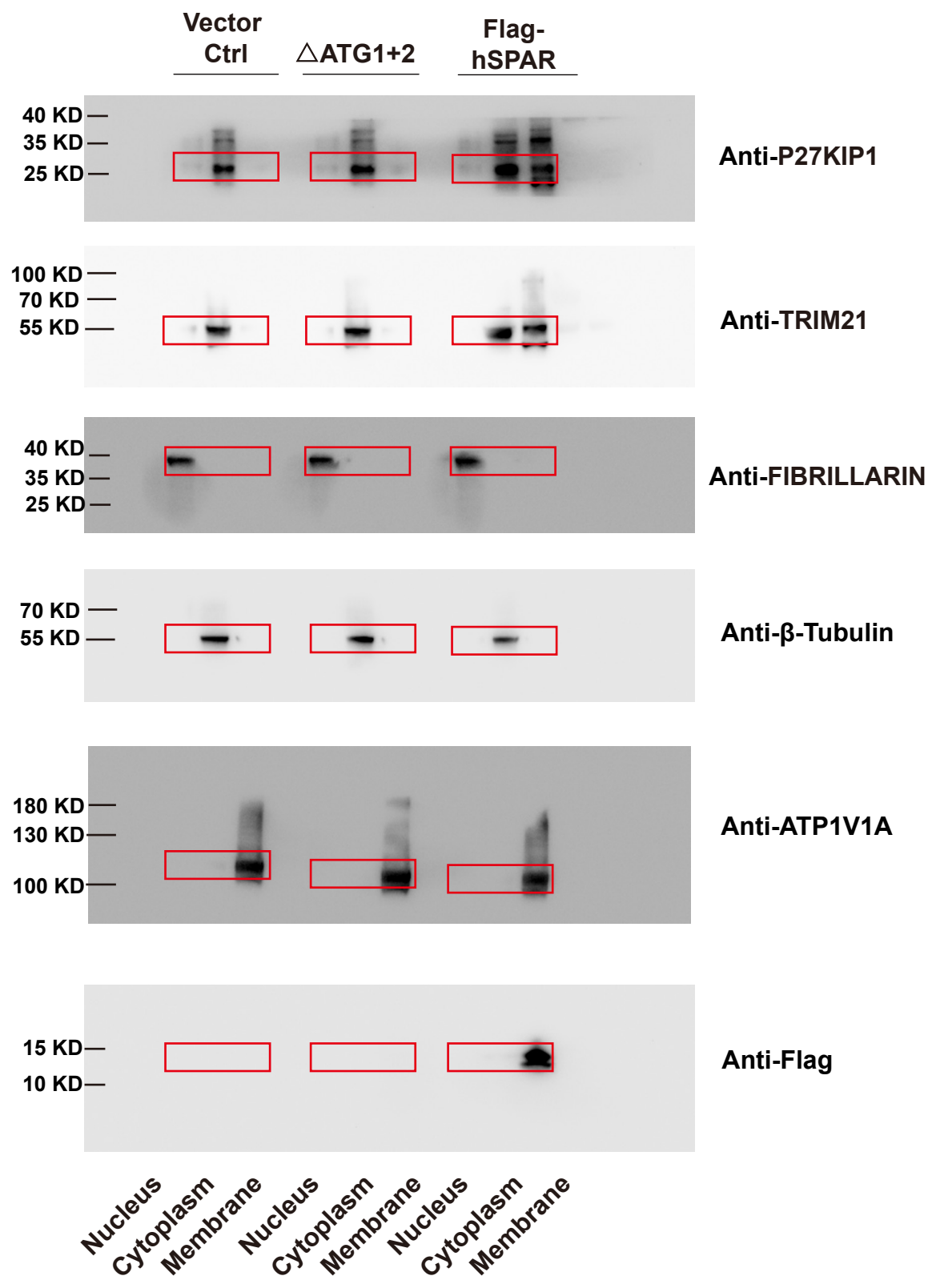

Supplement: Supplementary file 10 — Source data Fig. 5 [file 44318_2024_359_MOESM10_ESM.zip › Figure 5/Fig 5A/Fig 5A.pdf]

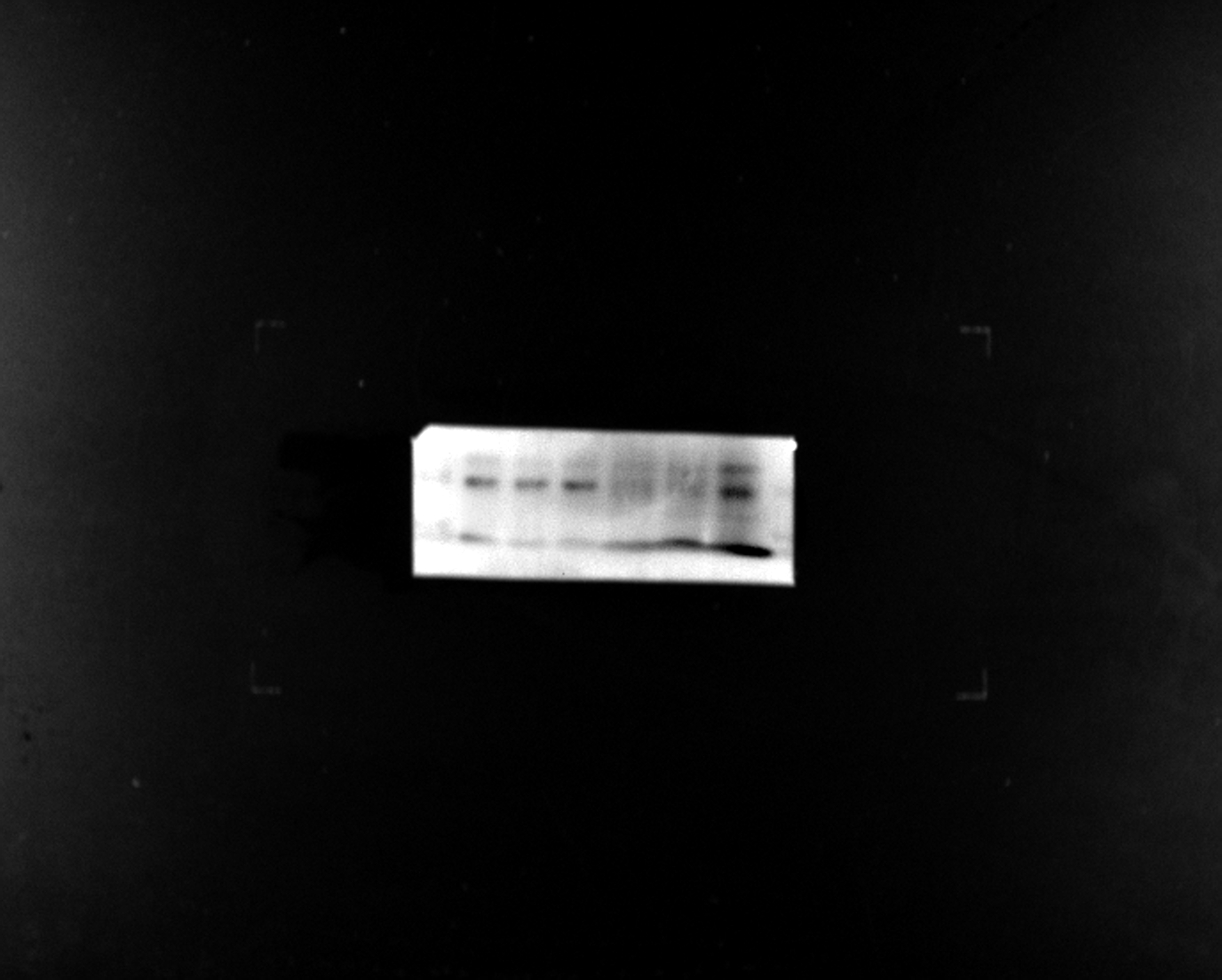

Supplement: Supplementary file 10 — Source data Fig. 5 [file 44318_2024_359_MOESM10_ESM.zip › Figure 5/Fig 5B and 5C/Fig 5B/Fig 5B cytoplasm lysosome extracts/2-p27-merge.Tif]

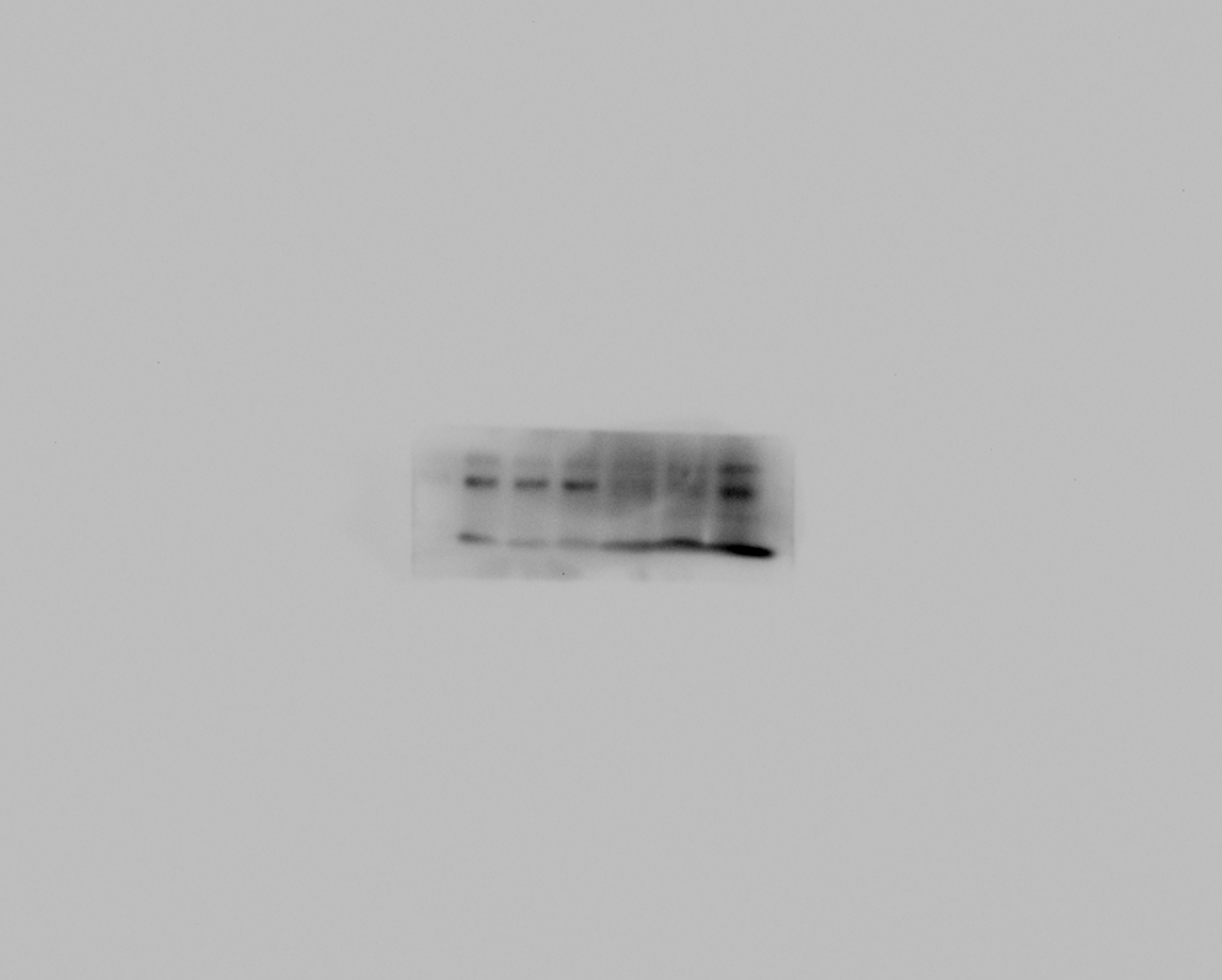

Supplement: Supplementary file 10 — Source data Fig. 5 [file 44318_2024_359_MOESM10_ESM.zip › Figure 5/Fig 5B and 5C/Fig 5B/Fig 5B cytoplasm lysosome extracts/2-p27.Tif]

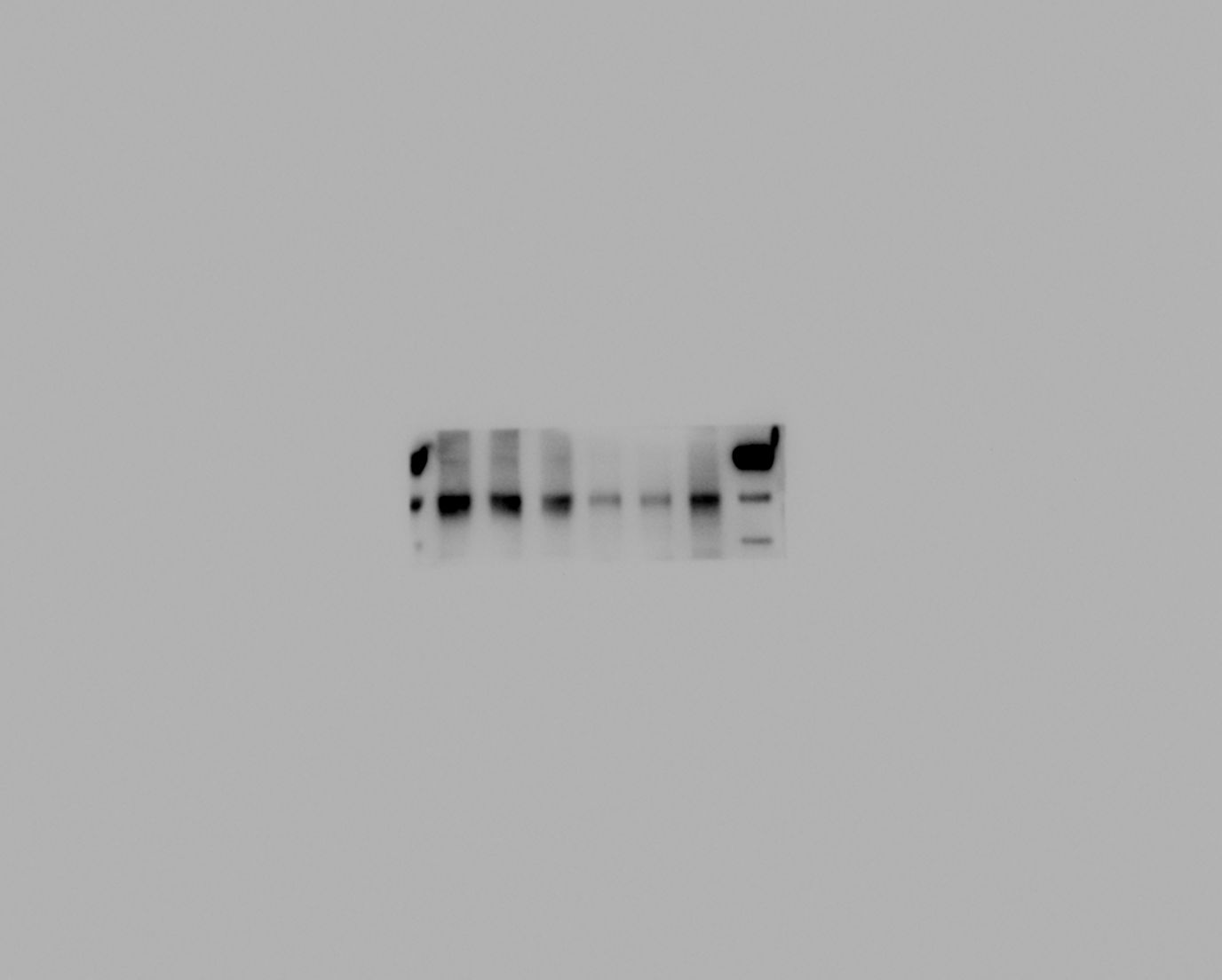

Supplement: Supplementary file 10 — Source data Fig. 5 [file 44318_2024_359_MOESM10_ESM.zip › Figure 5/Fig 5B and 5C/Fig 5B/Fig 5B cytoplasm lysosome extracts/3-TRIM21-1.Tif]

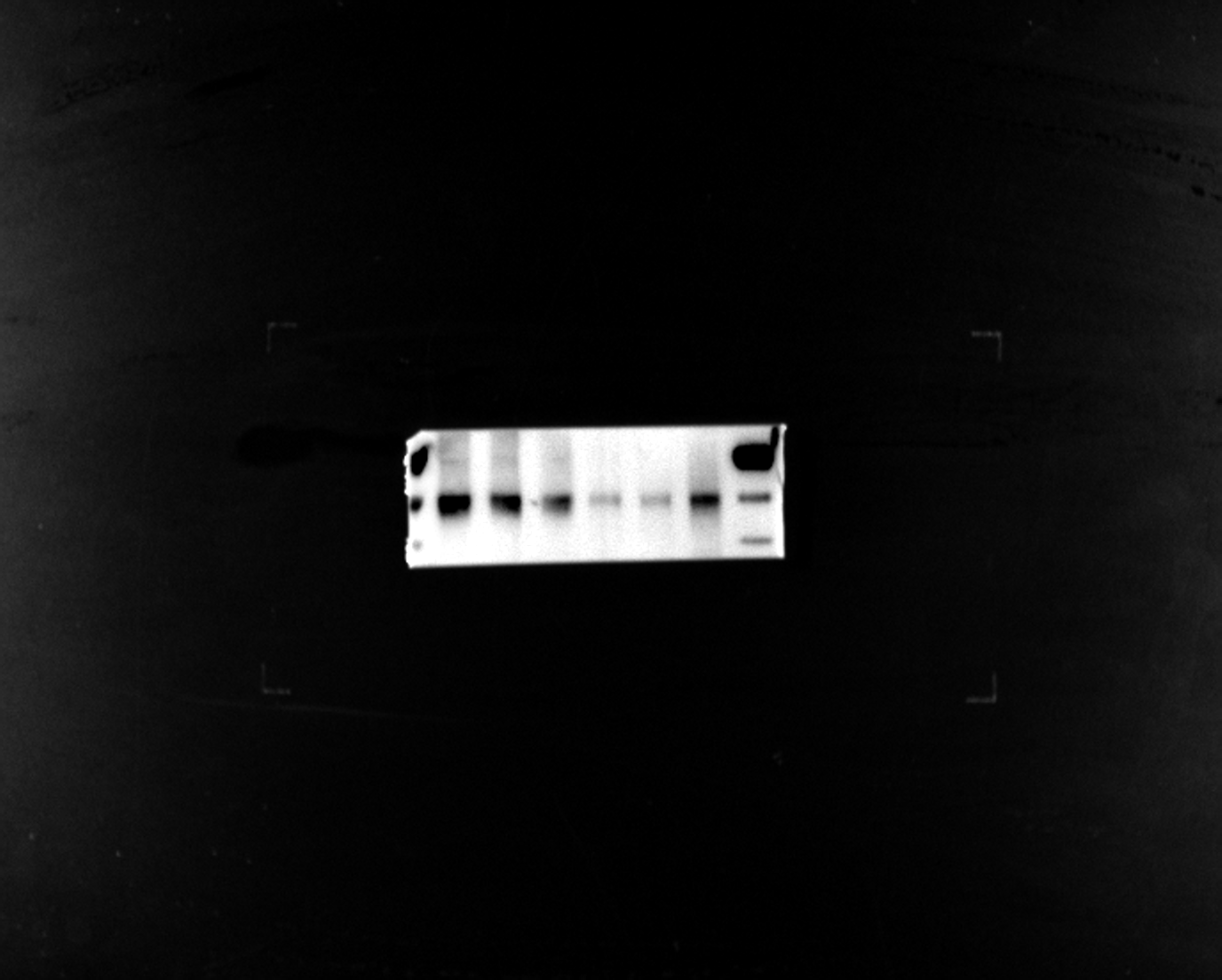

Supplement: Supplementary file 10 — Source data Fig. 5 [file 44318_2024_359_MOESM10_ESM.zip › Figure 5/Fig 5B and 5C/Fig 5B/Fig 5B cytoplasm lysosome extracts/3-TRIM21-2.Tif]

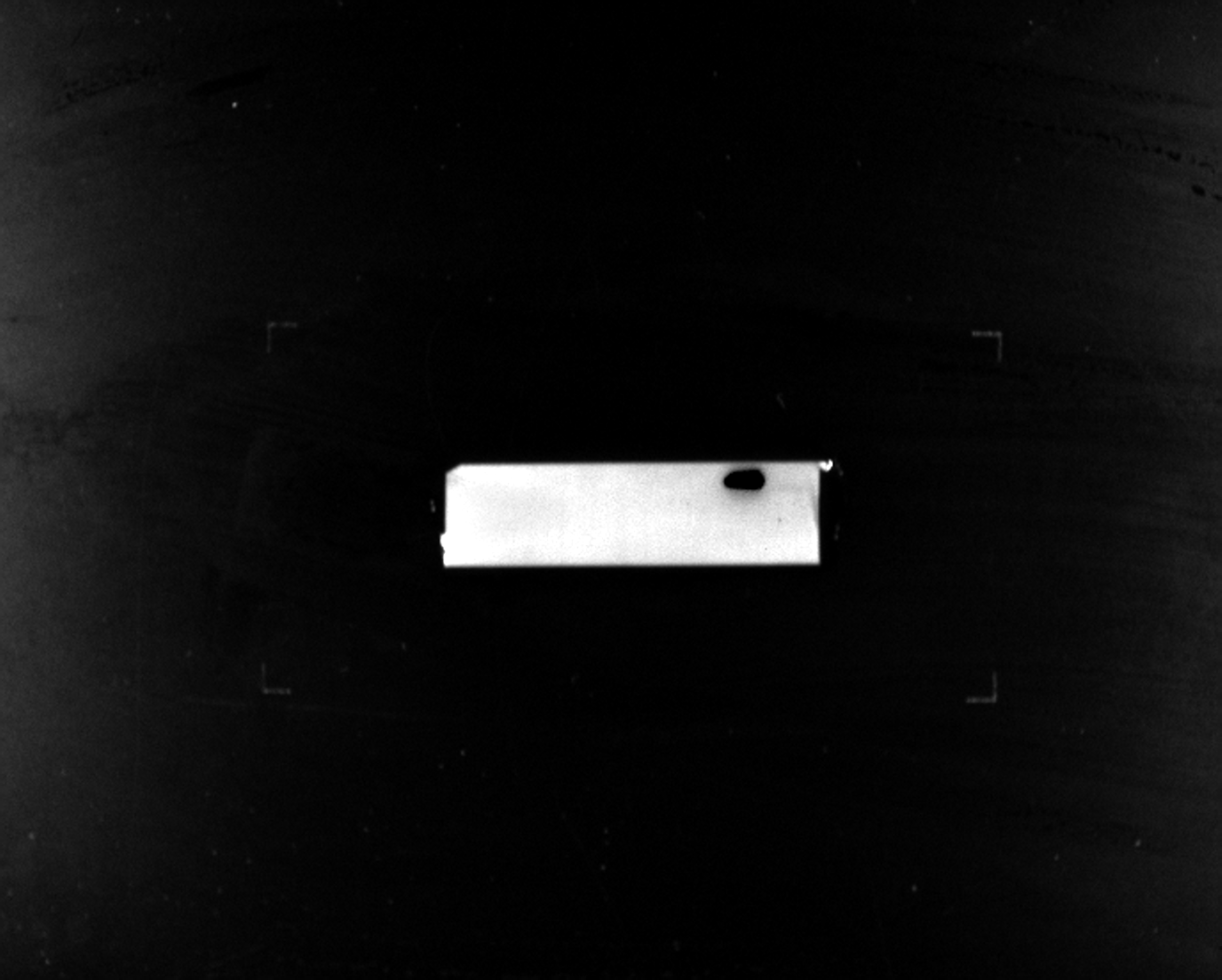

Supplement: Supplementary file 10 — Source data Fig. 5 [file 44318_2024_359_MOESM10_ESM.zip › Figure 5/Fig 5B and 5C/Fig 5B/Fig 5B cytoplasm lysosome extracts/4-Flag-merge.Tif]

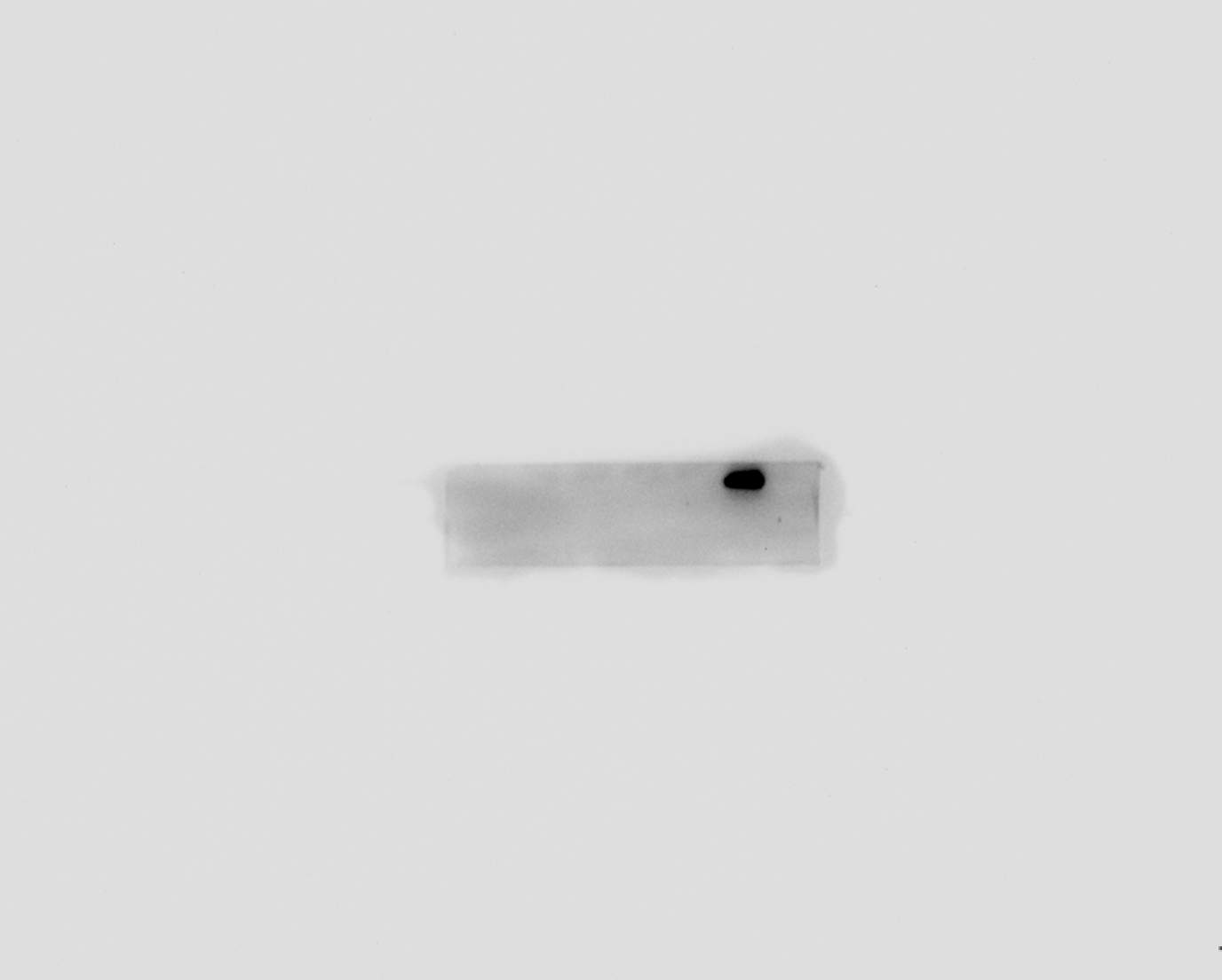

Supplement: Supplementary file 10 — Source data Fig. 5 [file 44318_2024_359_MOESM10_ESM.zip › Figure 5/Fig 5B and 5C/Fig 5B/Fig 5B cytoplasm lysosome extracts/4-Flag.Tif]

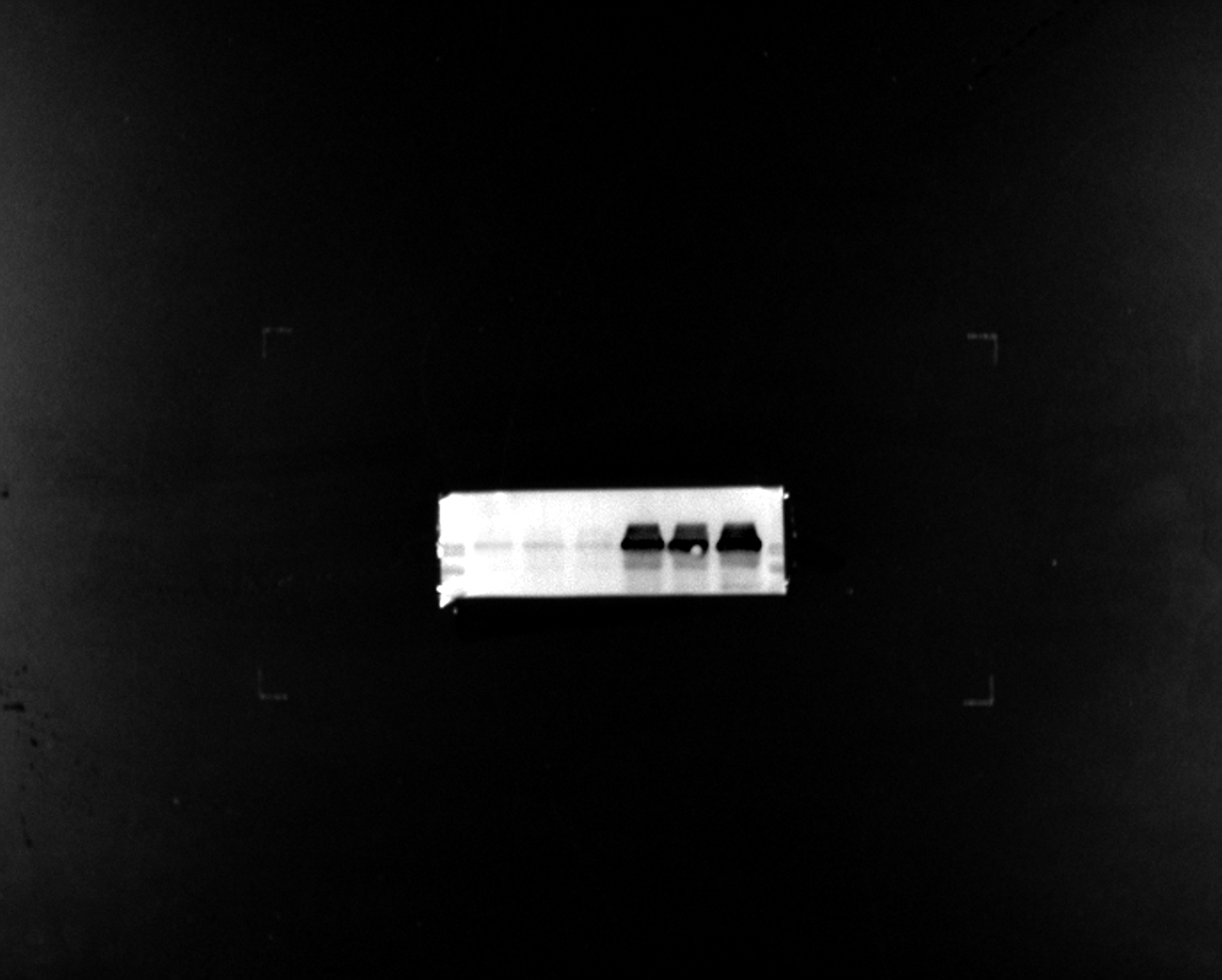

Supplement: Supplementary file 10 — Source data Fig. 5 [file 44318_2024_359_MOESM10_ESM.zip › Figure 5/Fig 5B and 5C/Fig 5B/Fig 5B cytoplasm lysosome extracts/5-LAMP2-merge.Tif]

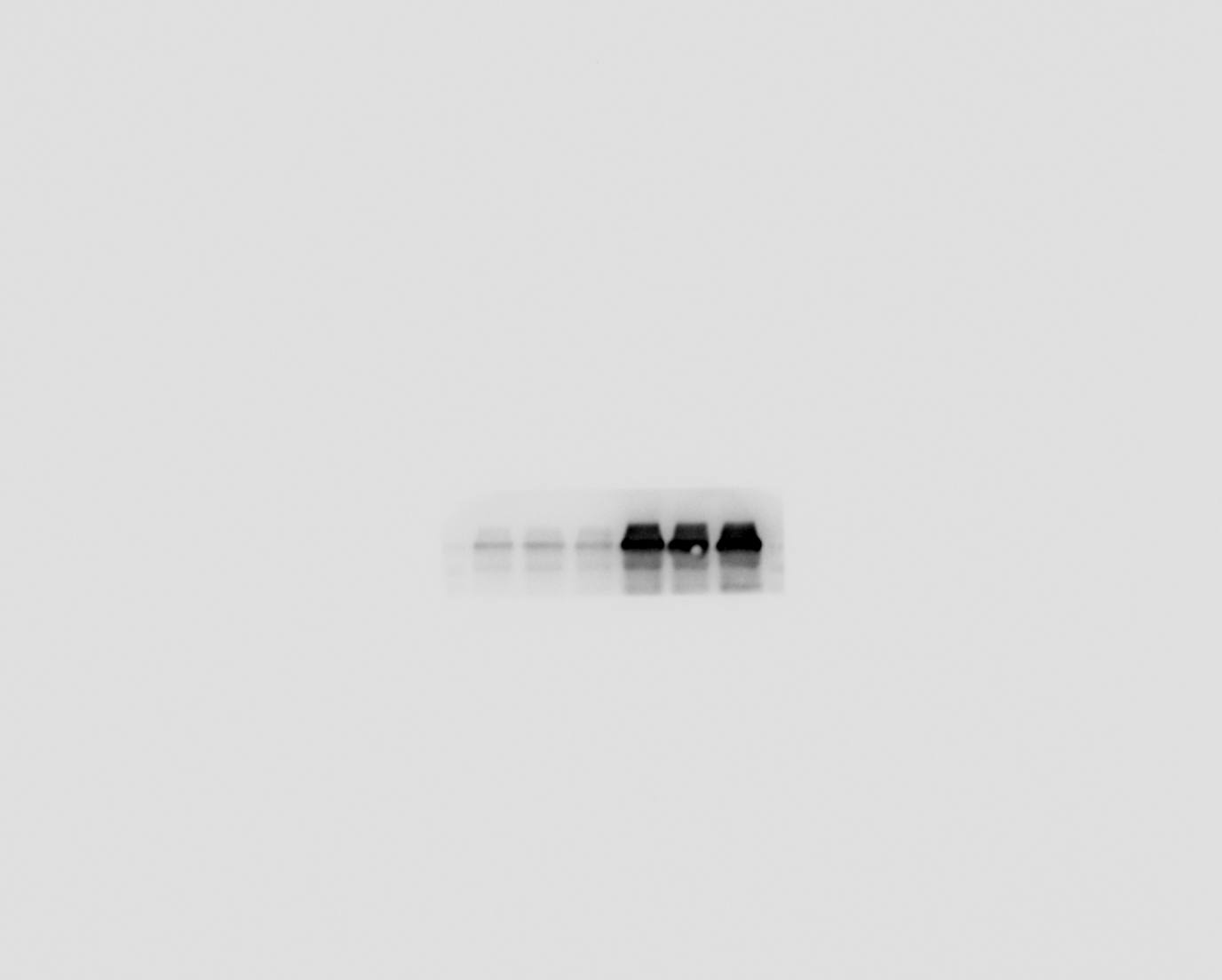

Supplement: Supplementary file 10 — Source data Fig. 5 [file 44318_2024_359_MOESM10_ESM.zip › Figure 5/Fig 5B and 5C/Fig 5B/Fig 5B cytoplasm lysosome extracts/5-LAMP2.Tif]

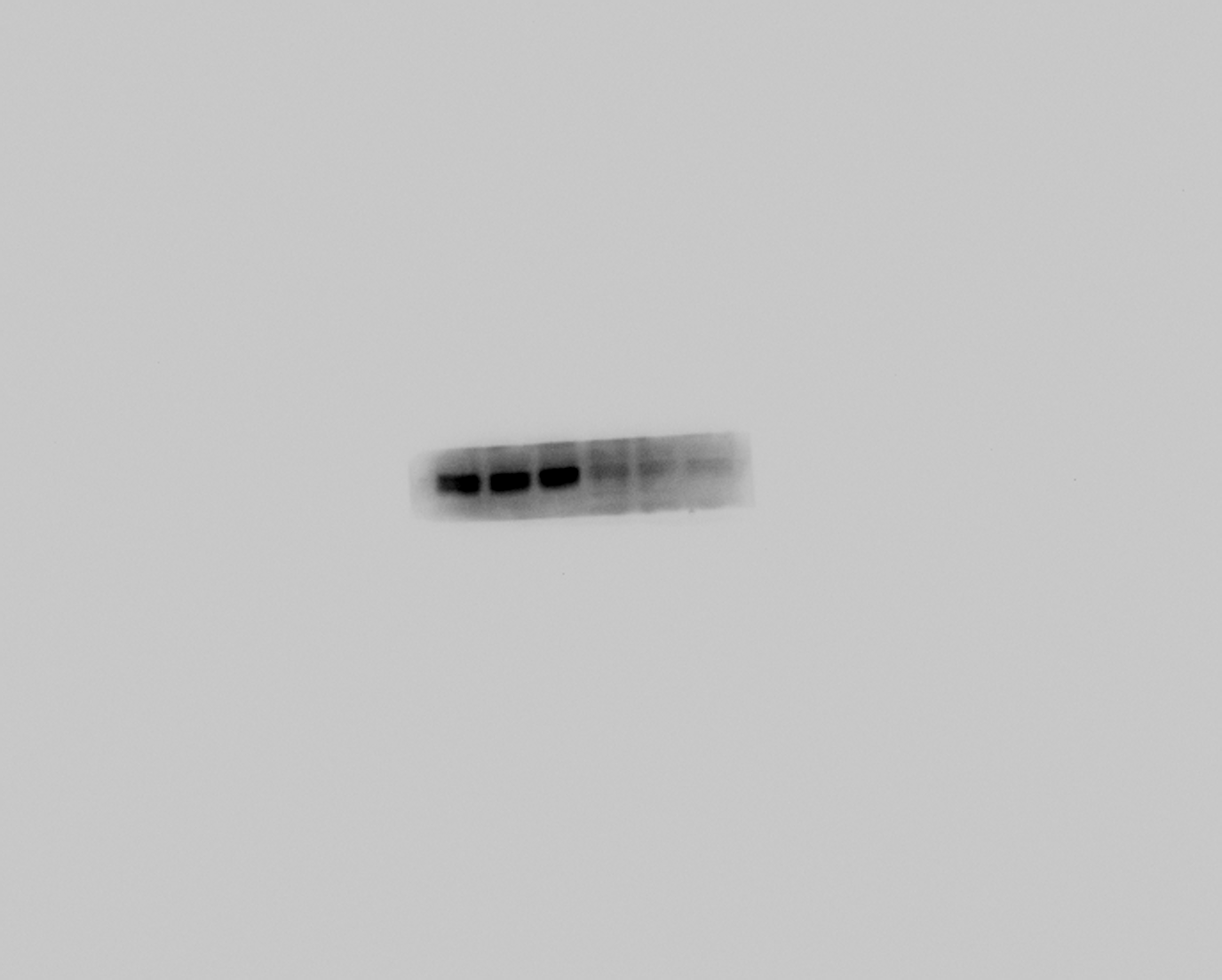

Supplement: Supplementary file 10 — Source data Fig. 5 [file 44318_2024_359_MOESM10_ESM.zip › Figure 5/Fig 5B and 5C/Fig 5B/Fig 5B cytoplasm lysosome extracts/6-TUBULIN.Tif]

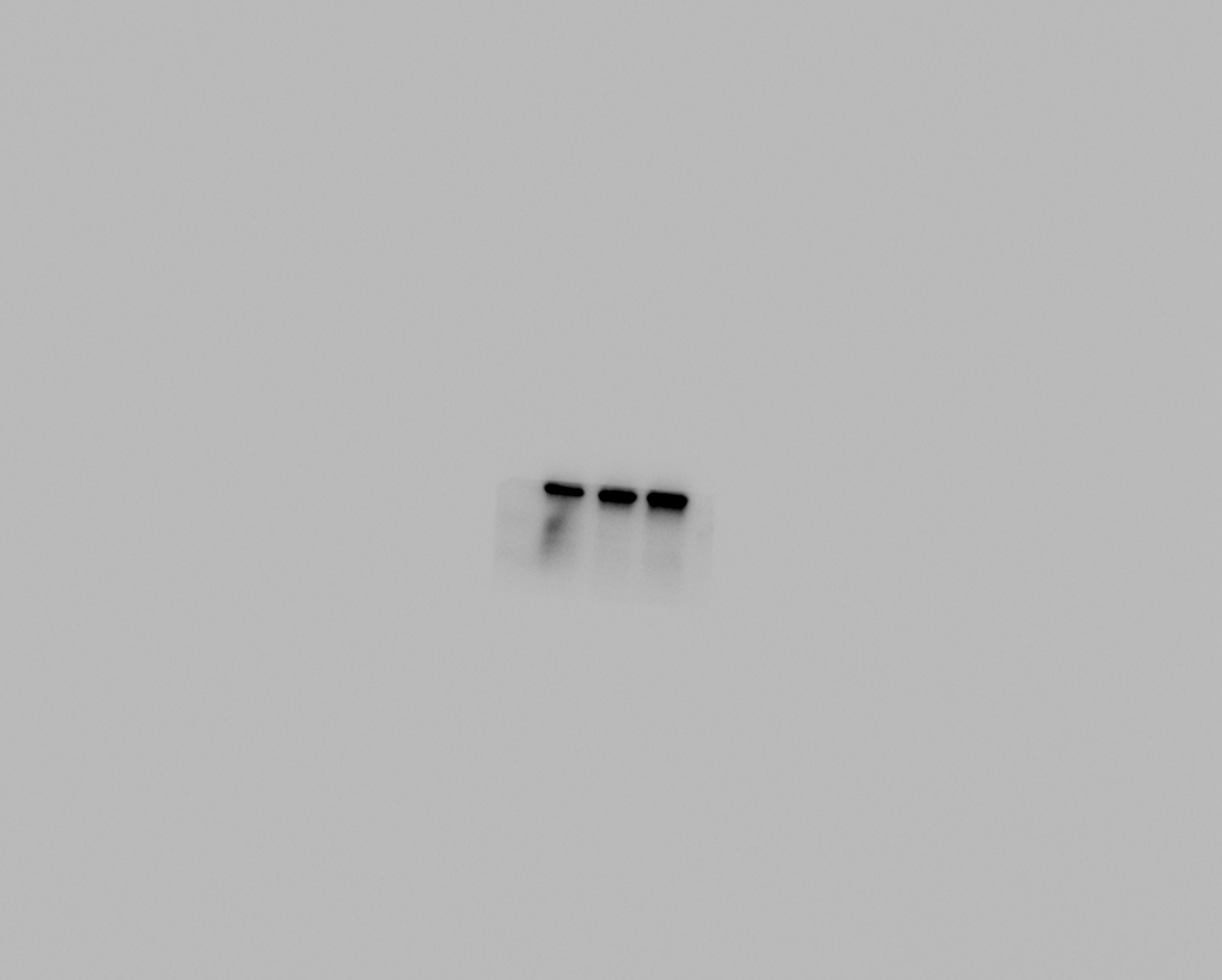

Supplement: Supplementary file 10 — Source data Fig. 5 [file 44318_2024_359_MOESM10_ESM.zip › Figure 5/Fig 5B and 5C/Fig 5B/Fig 5B whole cell extracts/GAPDH.Tif]

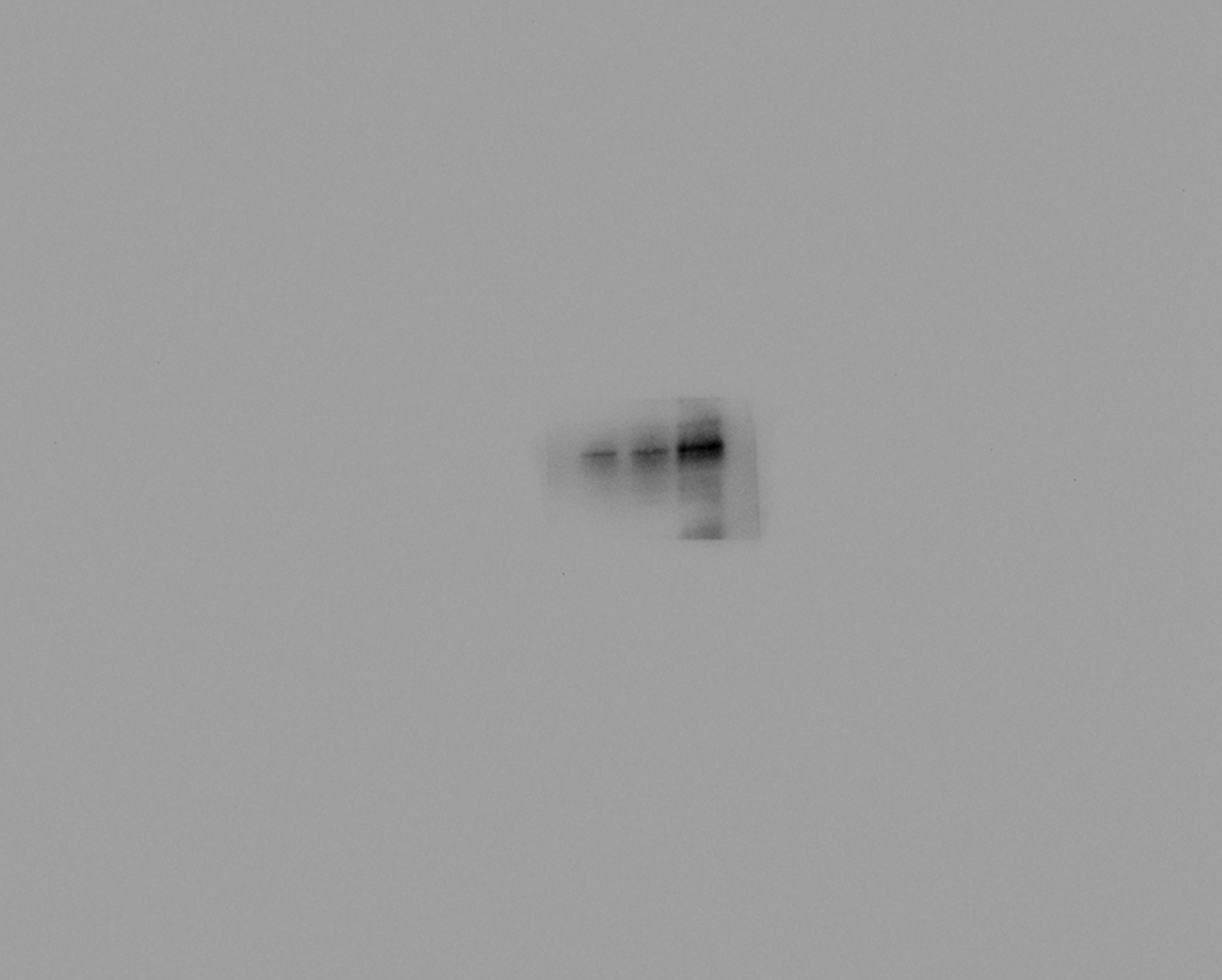

Supplement: Supplementary file 10 — Source data Fig. 5 [file 44318_2024_359_MOESM10_ESM.zip › Figure 5/Fig 5B and 5C/Fig 5B/Fig 5B whole cell extracts/P27KIP1-1.Tif]

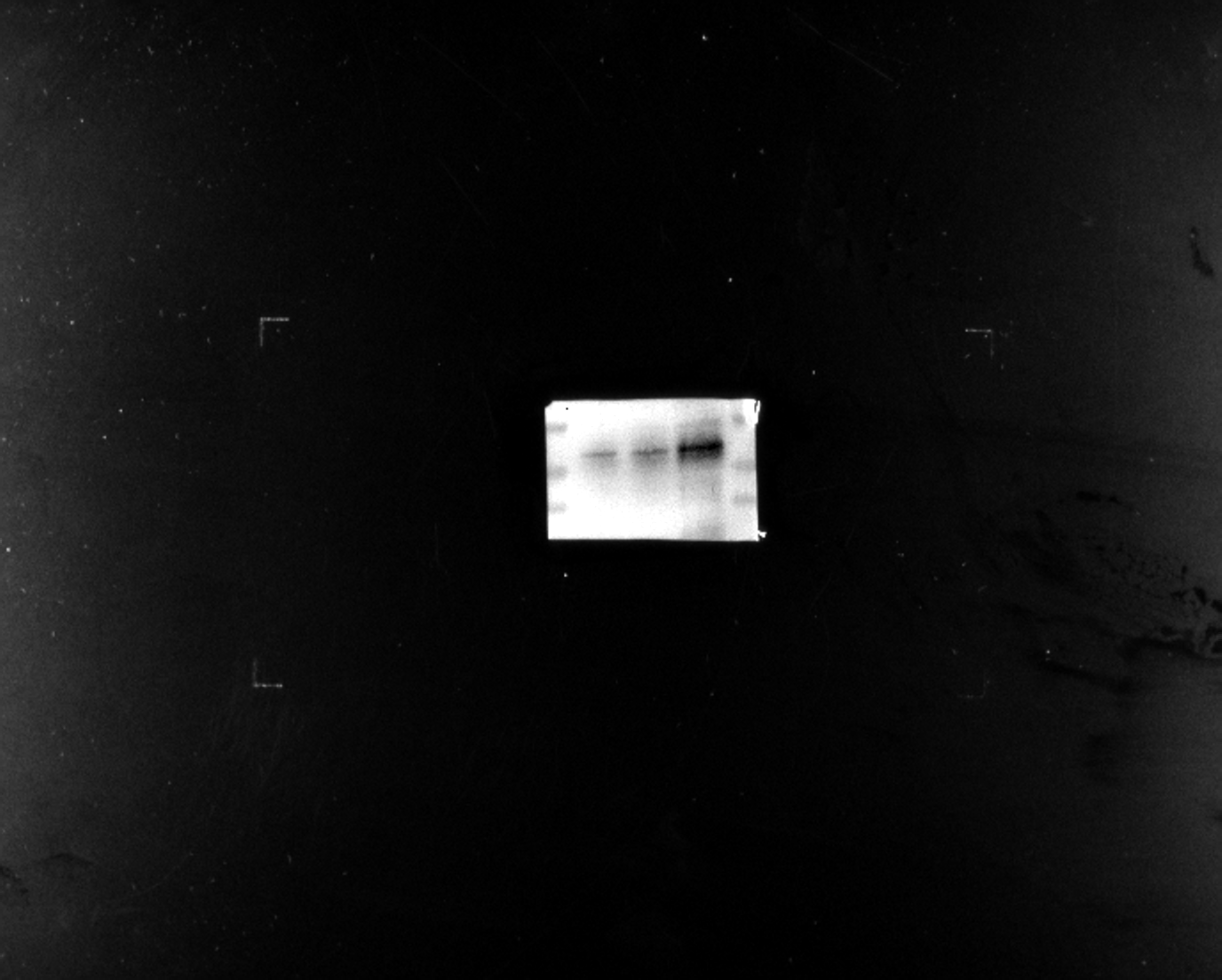

Supplement: Supplementary file 10 — Source data Fig. 5 [file 44318_2024_359_MOESM10_ESM.zip › Figure 5/Fig 5B and 5C/Fig 5B/Fig 5B whole cell extracts/P27KIP1-MERGE.Tif]

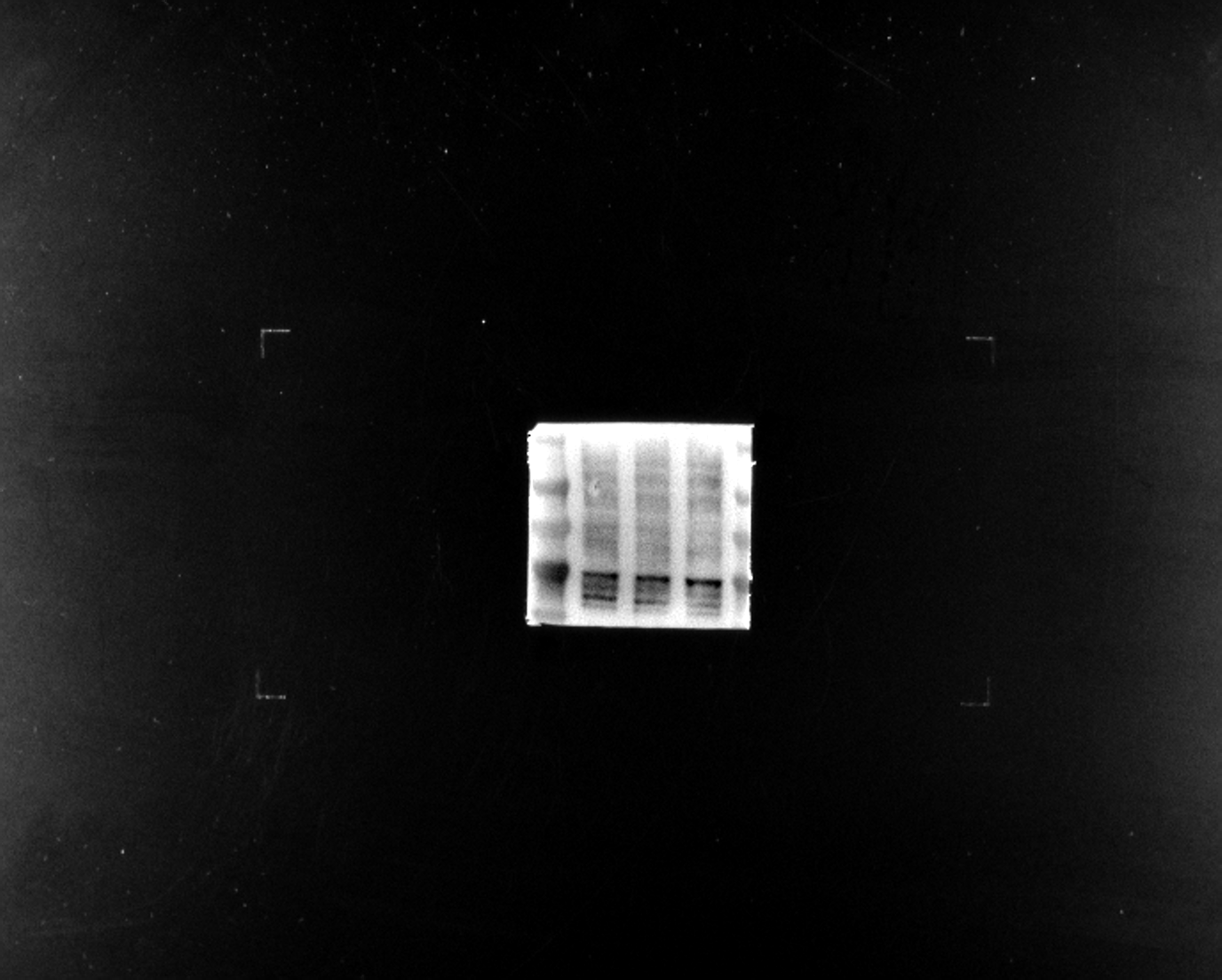

Supplement: Supplementary file 10 — Source data Fig. 5 [file 44318_2024_359_MOESM10_ESM.zip › Figure 5/Fig 5B and 5C/Fig 5B/Fig 5B whole cell extracts/TRIM21-merge.Tif]

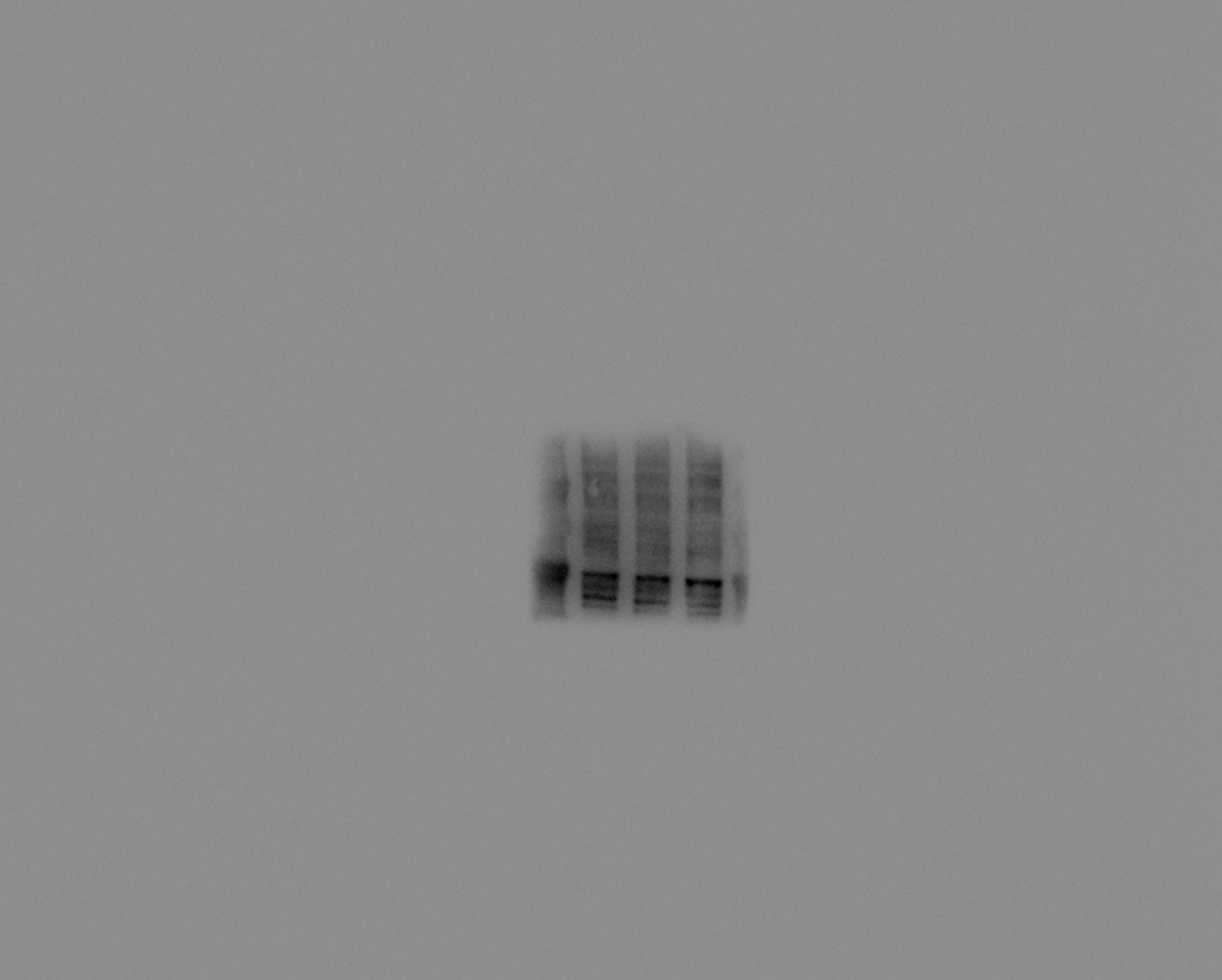

Supplement: Supplementary file 10 — Source data Fig. 5 [file 44318_2024_359_MOESM10_ESM.zip › Figure 5/Fig 5B and 5C/Fig 5B/Fig 5B whole cell extracts/TRIM21.Tif]

**Fig 5B**

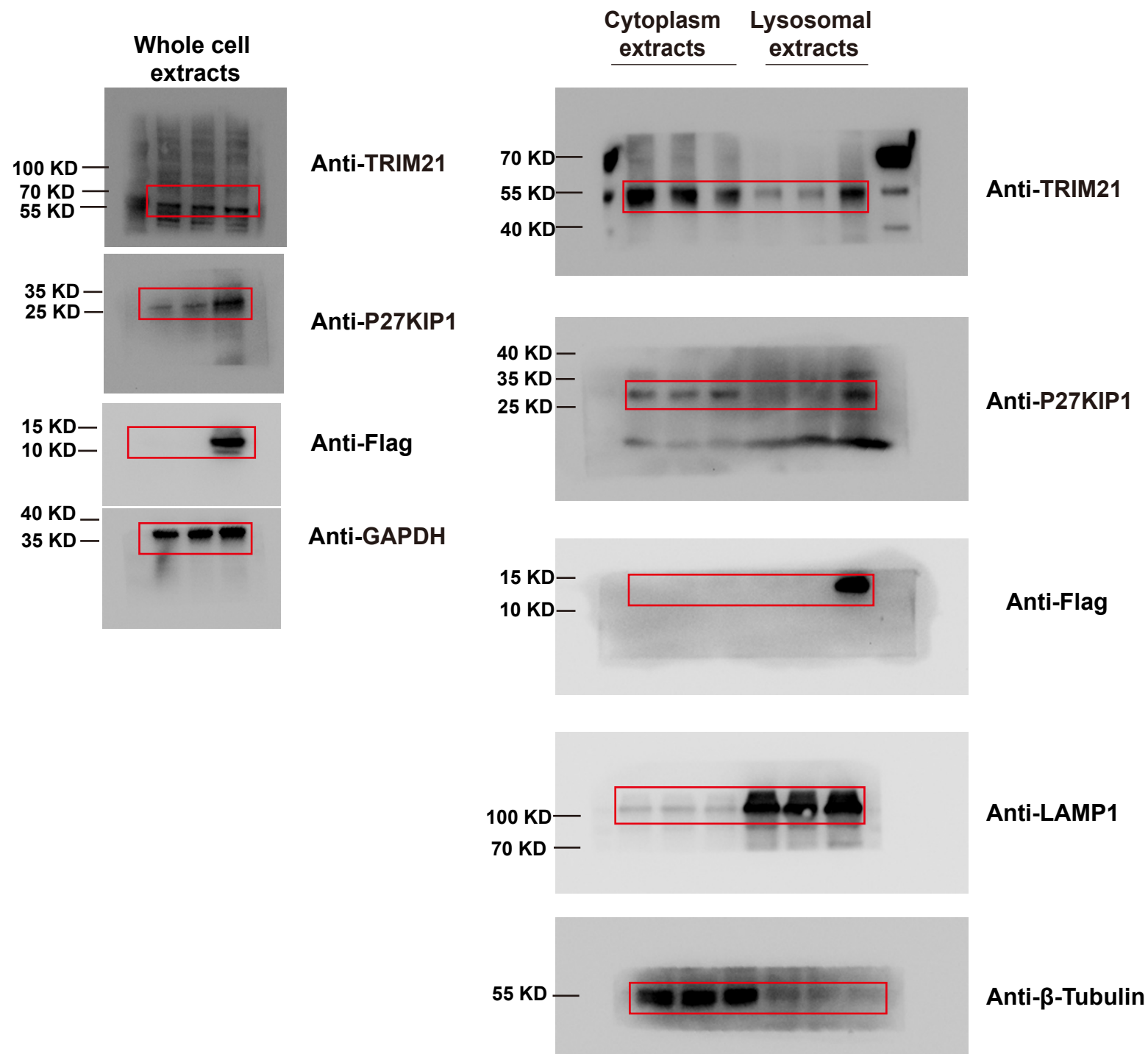

Supplement: Supplementary file 10 — Source data Fig. 5 [file 44318_2024_359_MOESM10_ESM.zip › Figure 5/Fig 5B and 5C/Fig 5B/Fig 5B.pdf]

**Fig 5D**

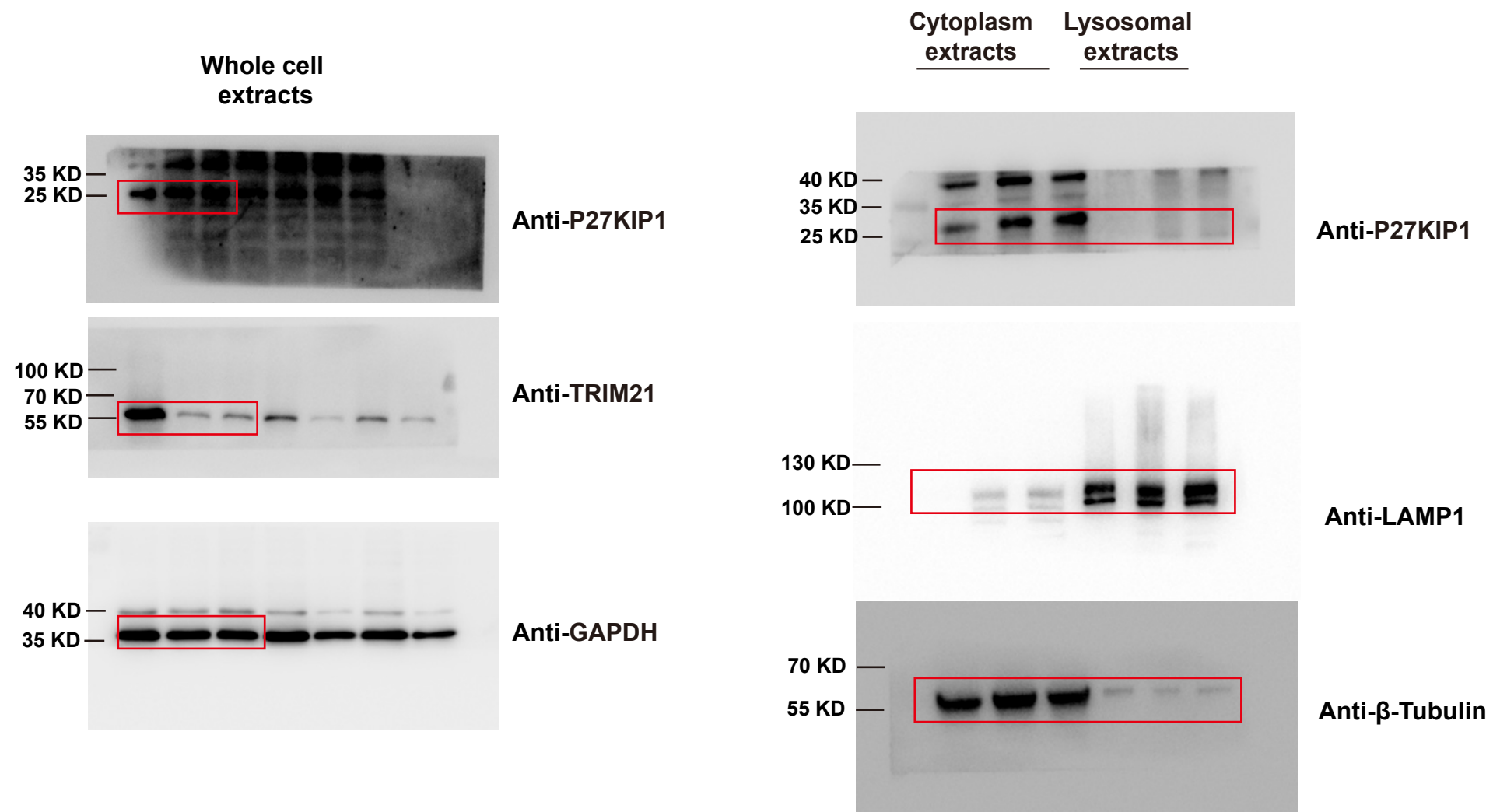

Supplement: Supplementary file 10 — Source data Fig. 5 [file 44318_2024_359_MOESM10_ESM.zip › Figure 5/Fig 5D/Fig 5D.pdf]

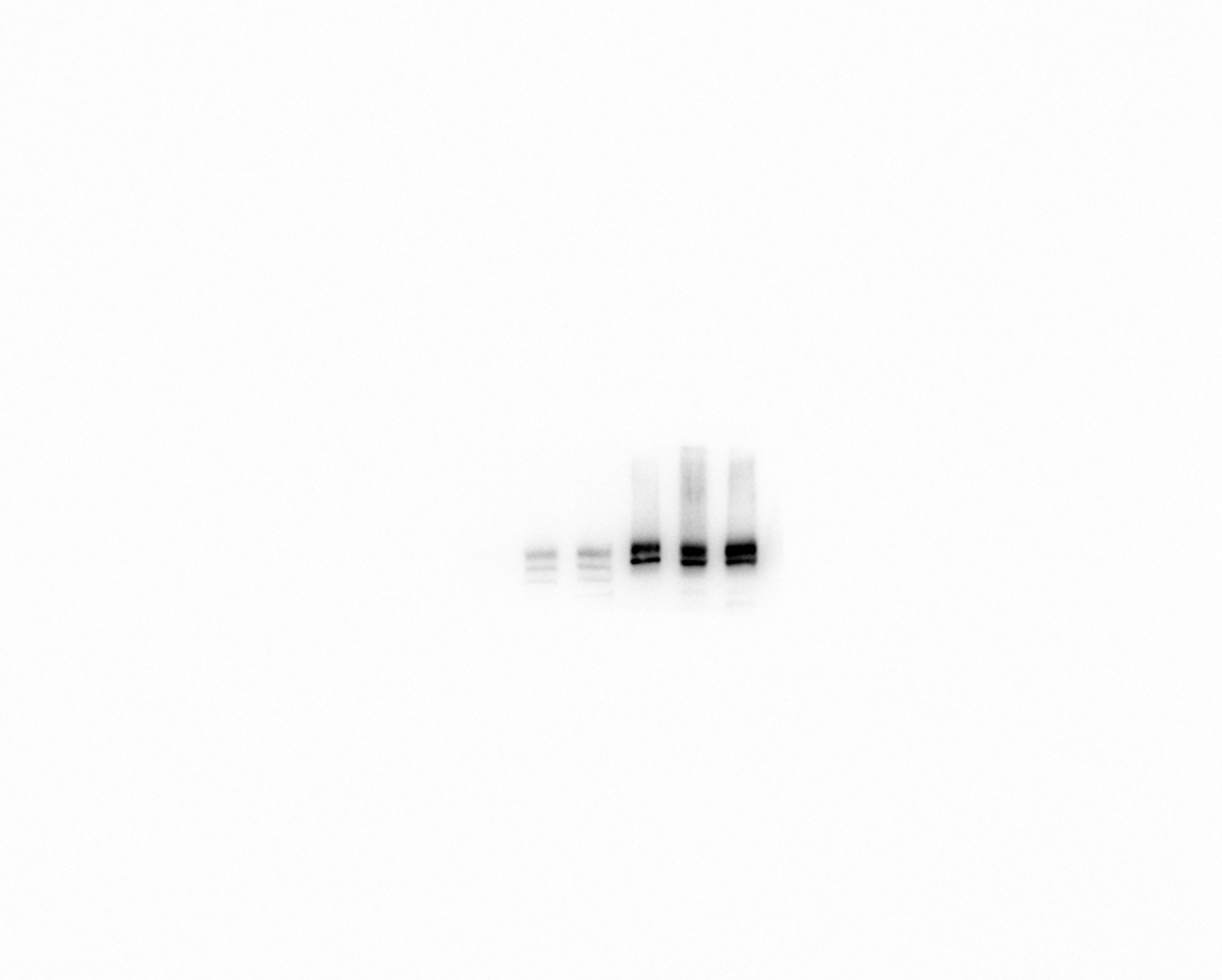

Supplement: Supplementary file 10 — Source data Fig. 5 [file 44318_2024_359_MOESM10_ESM.zip › Figure 5/Fig 5D/cytoplasm-lysosome extracts/2-LAMP2-1.Tif]

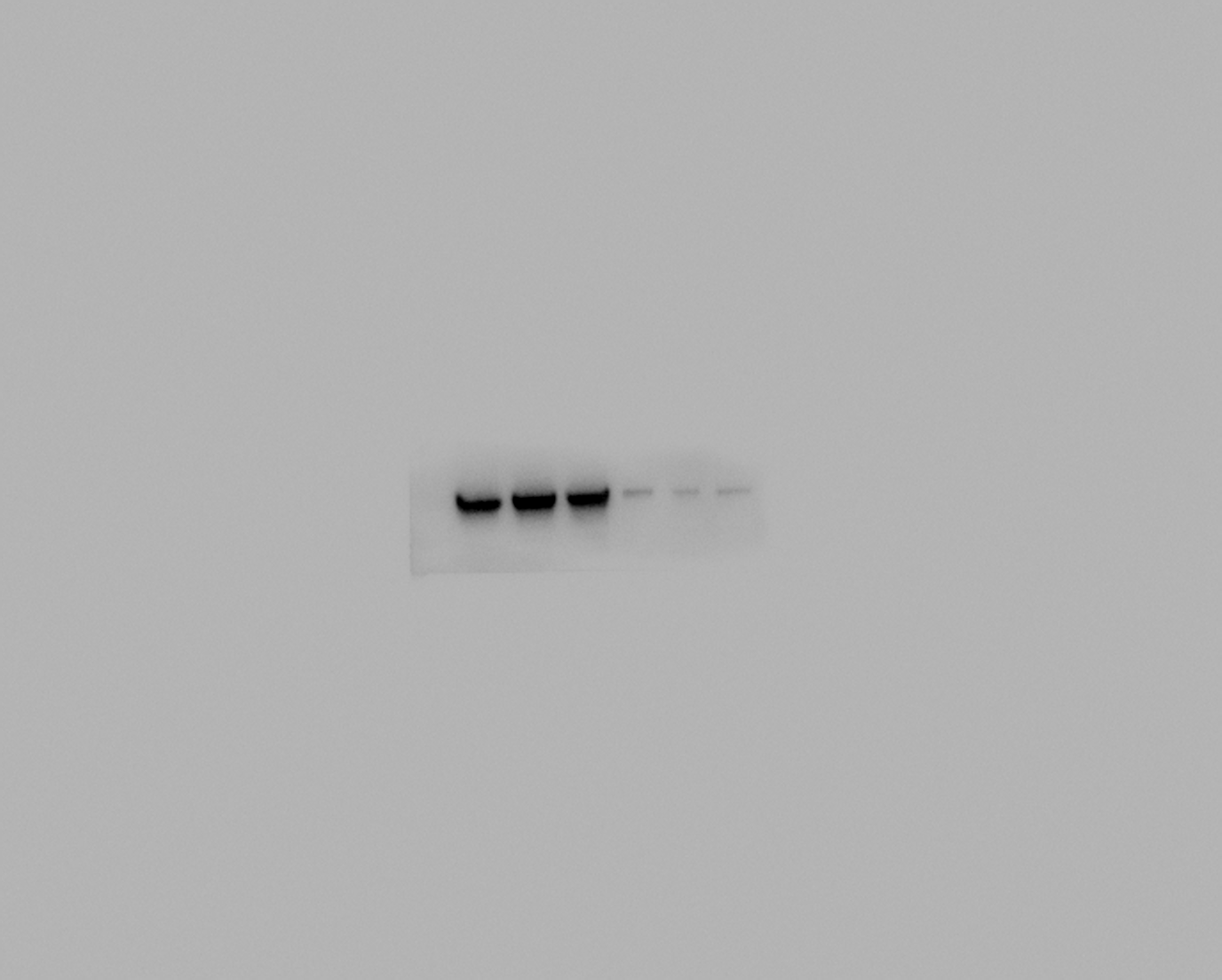

Supplement: Supplementary file 10 — Source data Fig. 5 [file 44318_2024_359_MOESM10_ESM.zip › Figure 5/Fig 5D/cytoplasm-lysosome extracts/2-TUBULIN-1.Tif]

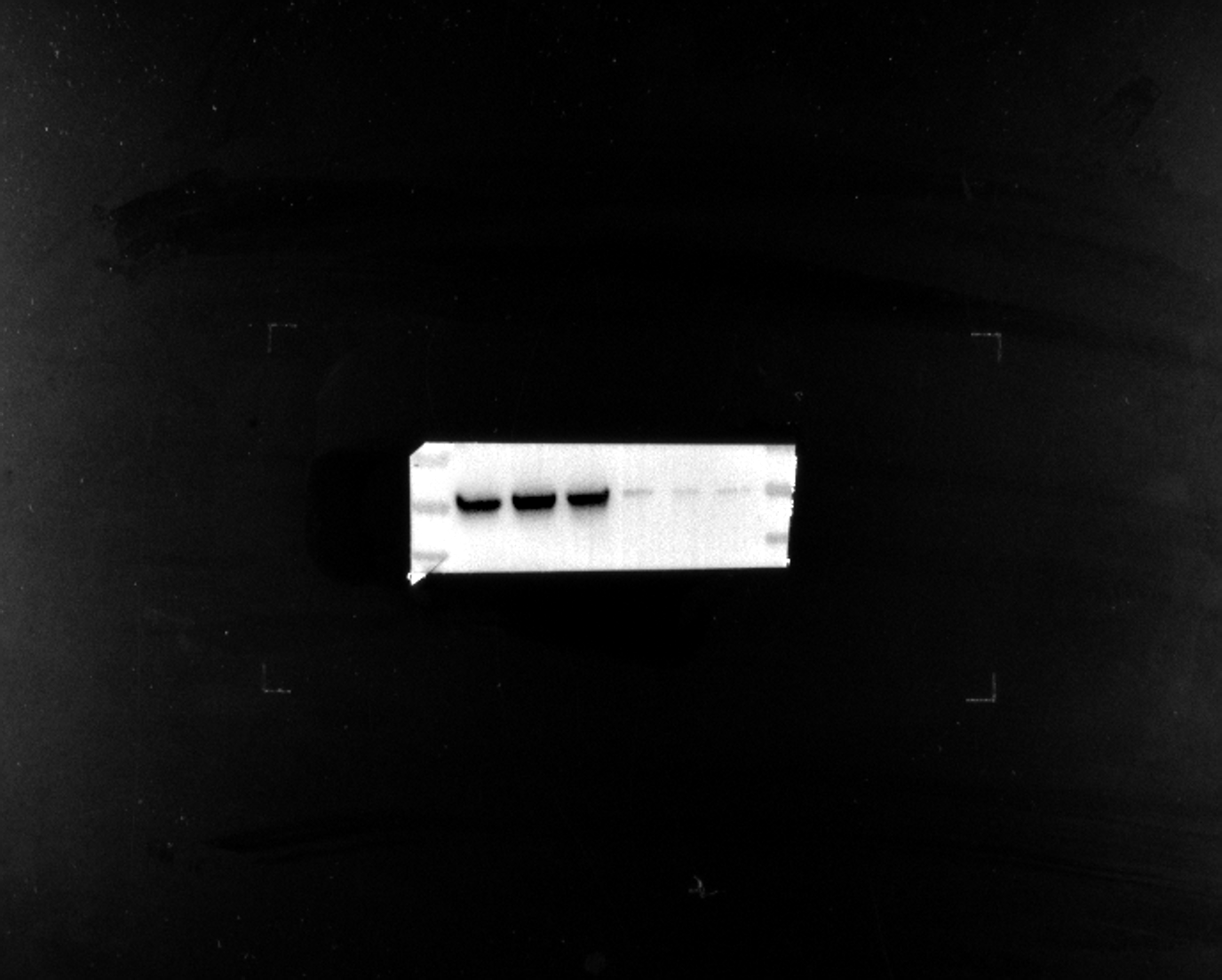

Supplement: Supplementary file 10 — Source data Fig. 5 [file 44318_2024_359_MOESM10_ESM.zip › Figure 5/Fig 5D/cytoplasm-lysosome extracts/2-TUBULIN-2.Tif]

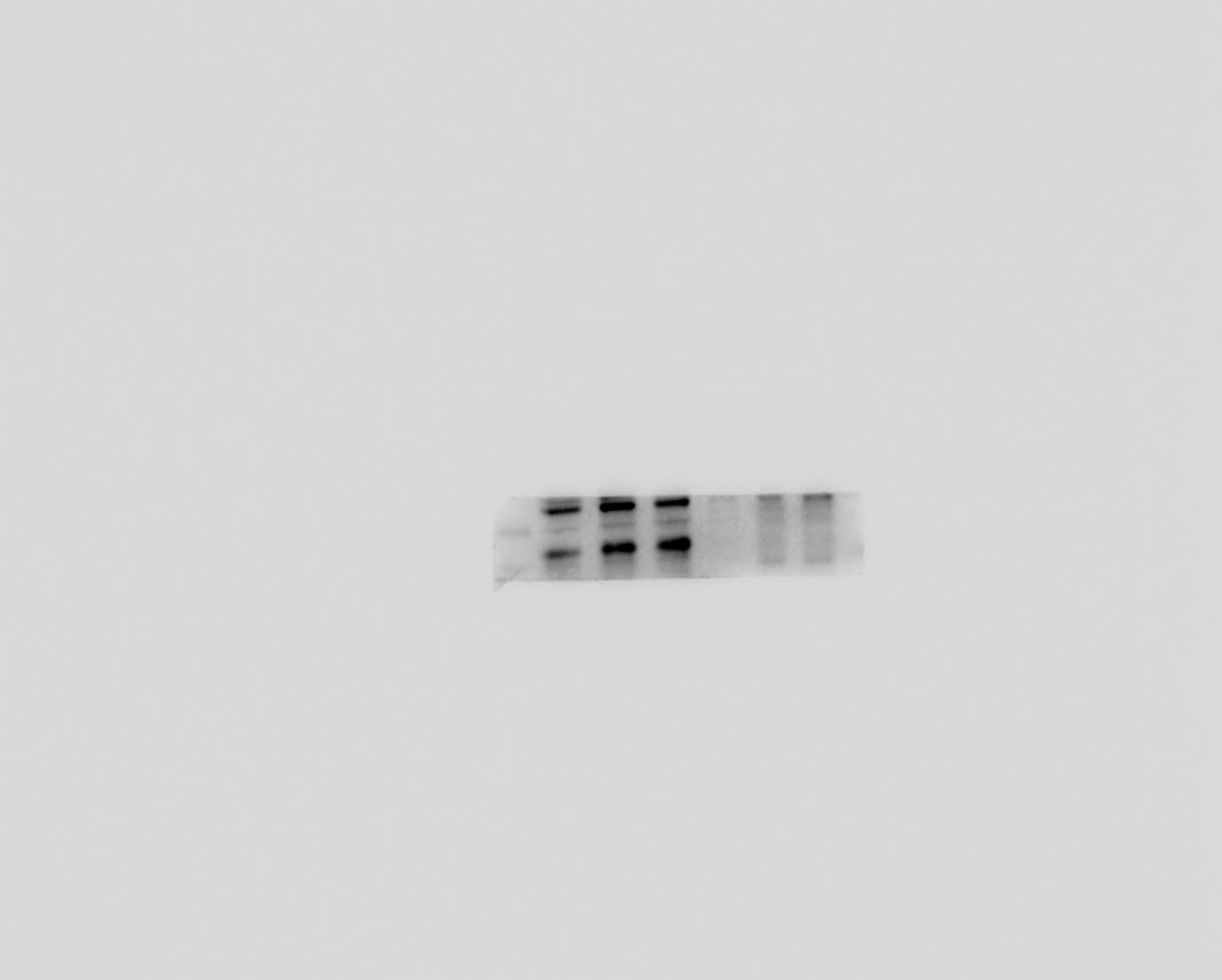

Supplement: Supplementary file 10 — Source data Fig. 5 [file 44318_2024_359_MOESM10_ESM.zip › Figure 5/Fig 5D/cytoplasm-lysosome extracts/2-p27-1.Tif]

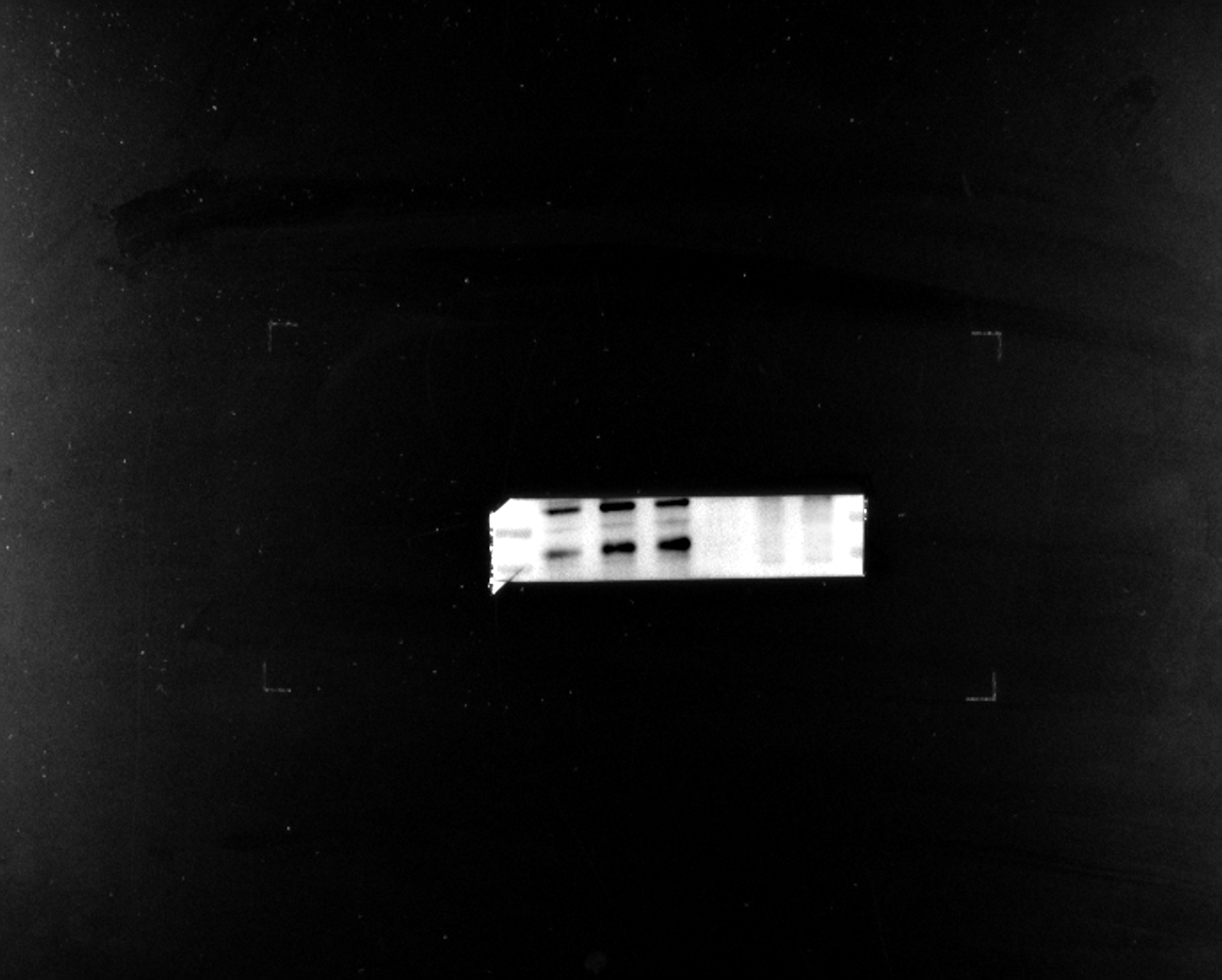

Supplement: Supplementary file 10 — Source data Fig. 5 [file 44318_2024_359_MOESM10_ESM.zip › Figure 5/Fig 5D/cytoplasm-lysosome extracts/2-p27-2.Tif]

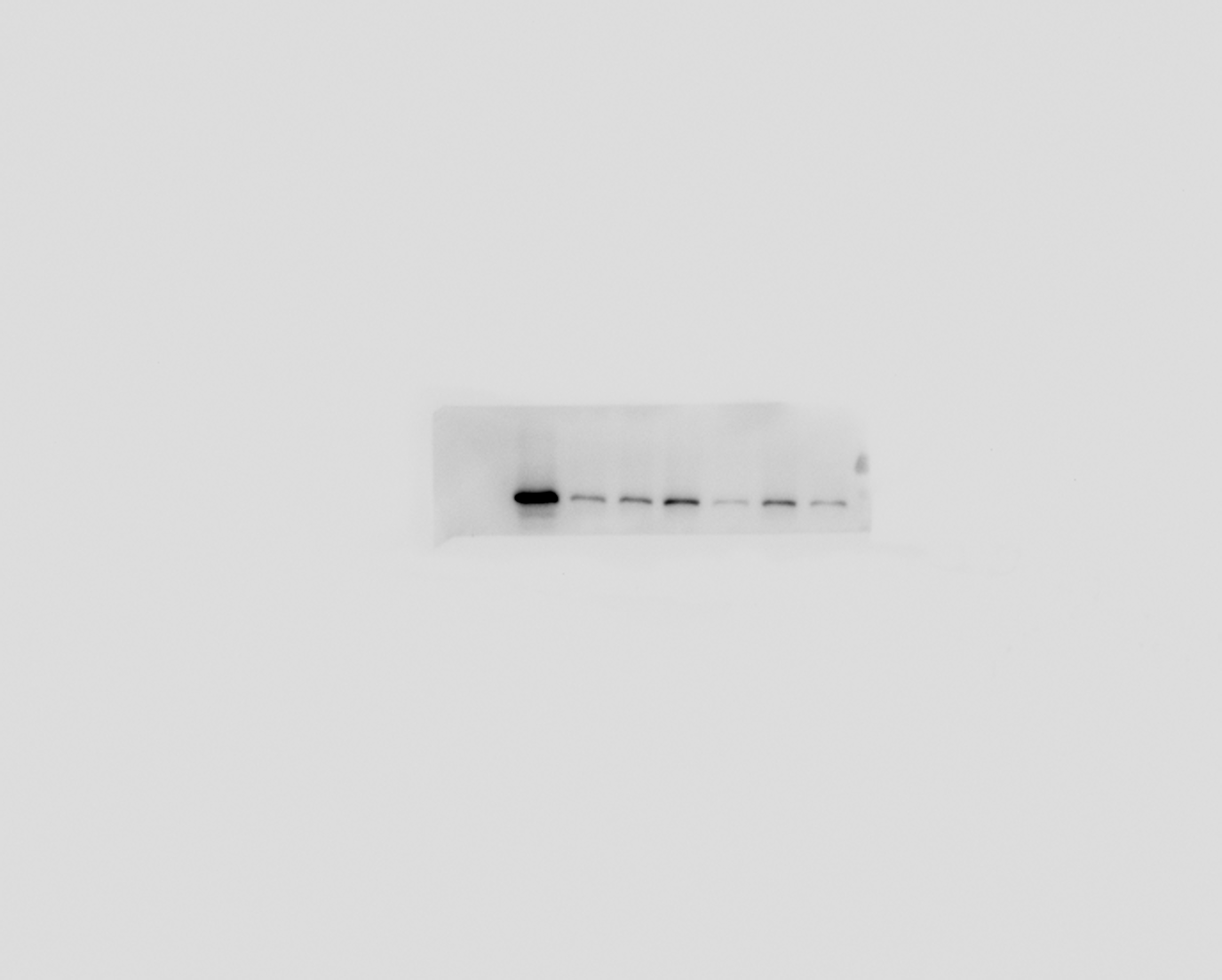

Supplement: Supplementary file 10 — Source data Fig. 5 [file 44318_2024_359_MOESM10_ESM.zip › Figure 5/Fig 5D/whole cell extracts/1-TRIM21-1.Tif]

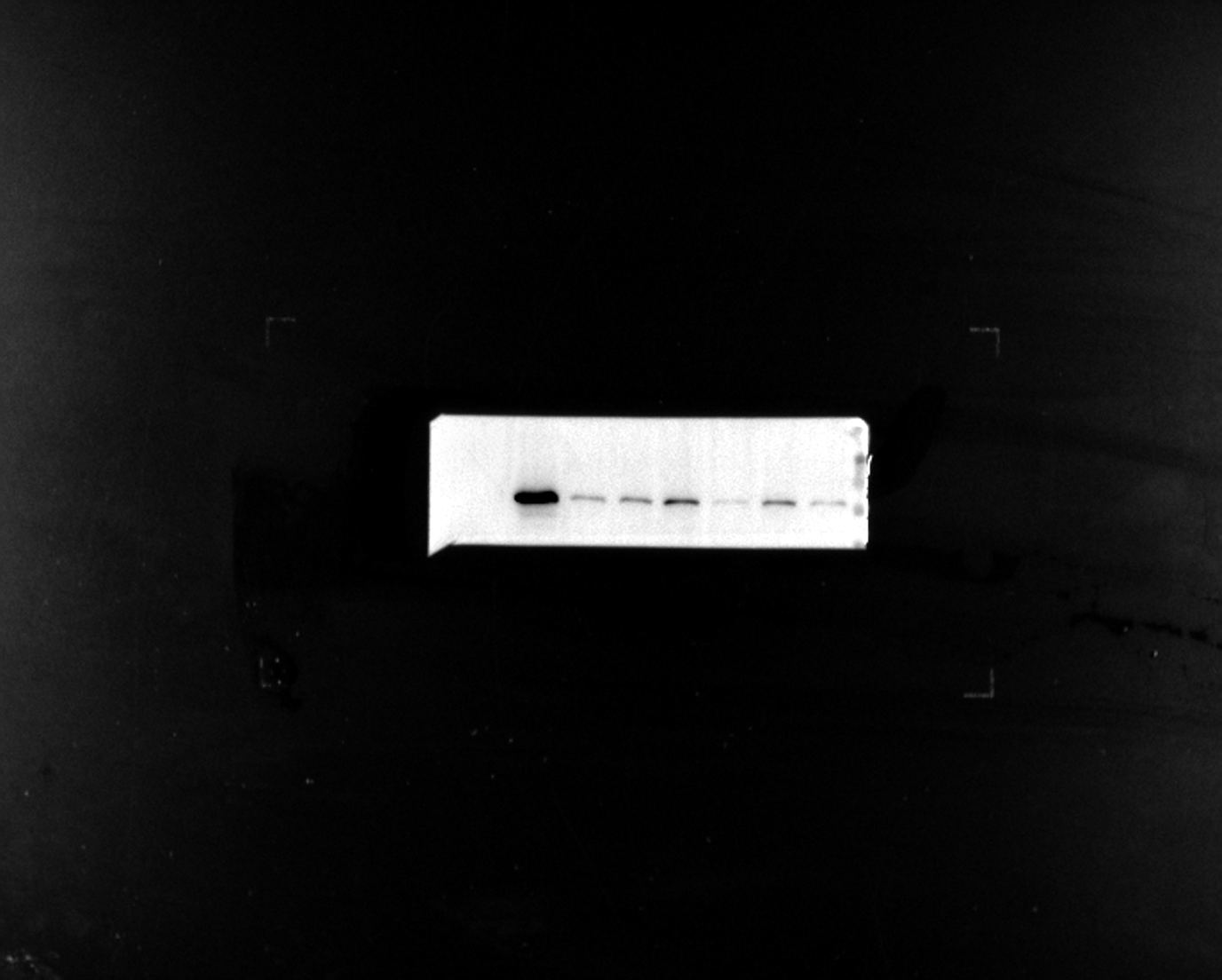

Supplement: Supplementary file 10 — Source data Fig. 5 [file 44318_2024_359_MOESM10_ESM.zip › Figure 5/Fig 5D/whole cell extracts/1-TRIM21-2.Tif]

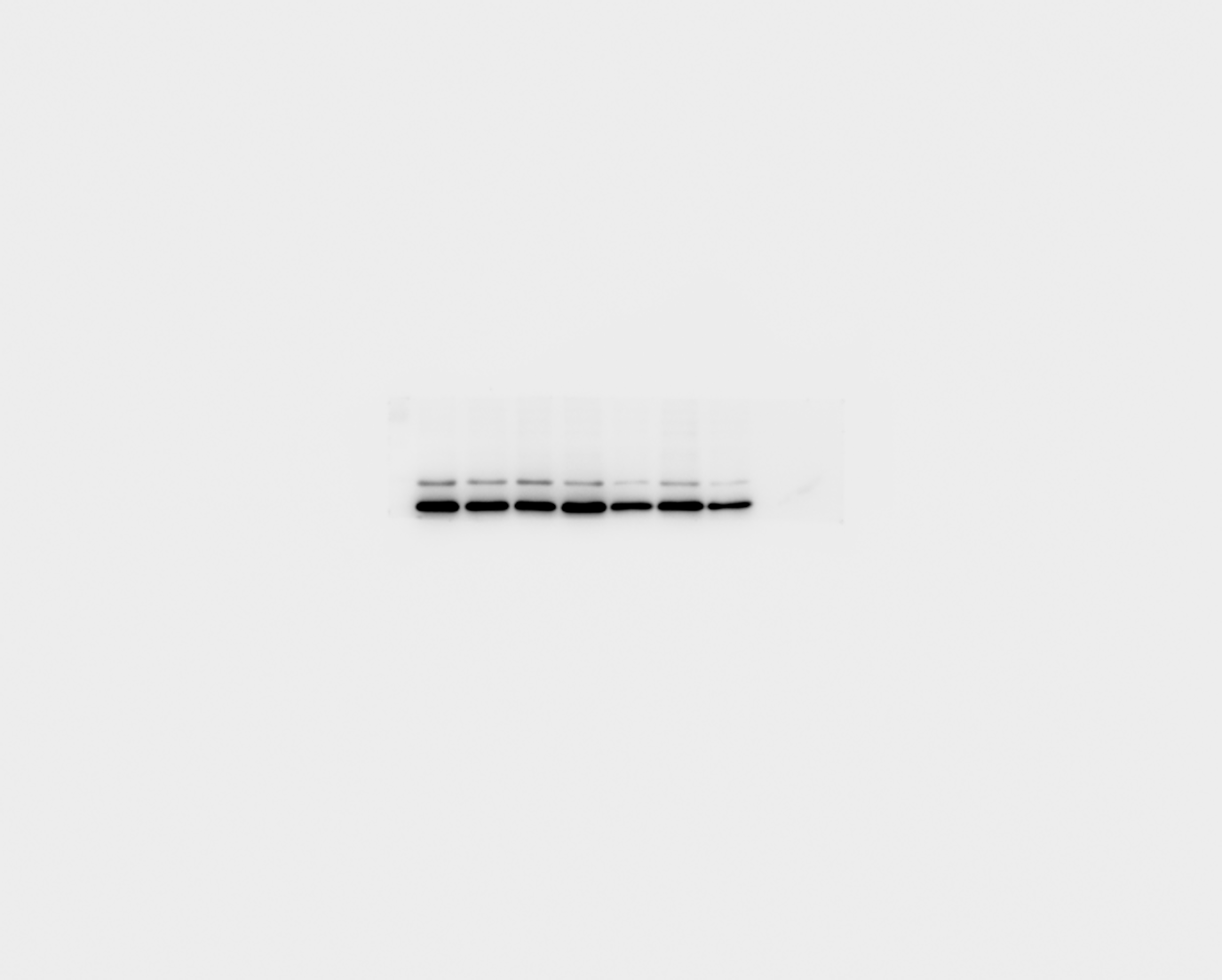

Supplement: Supplementary file 10 — Source data Fig. 5 [file 44318_2024_359_MOESM10_ESM.zip › Figure 5/Fig 5D/whole cell extracts/2-GAPDH-1.Tif]

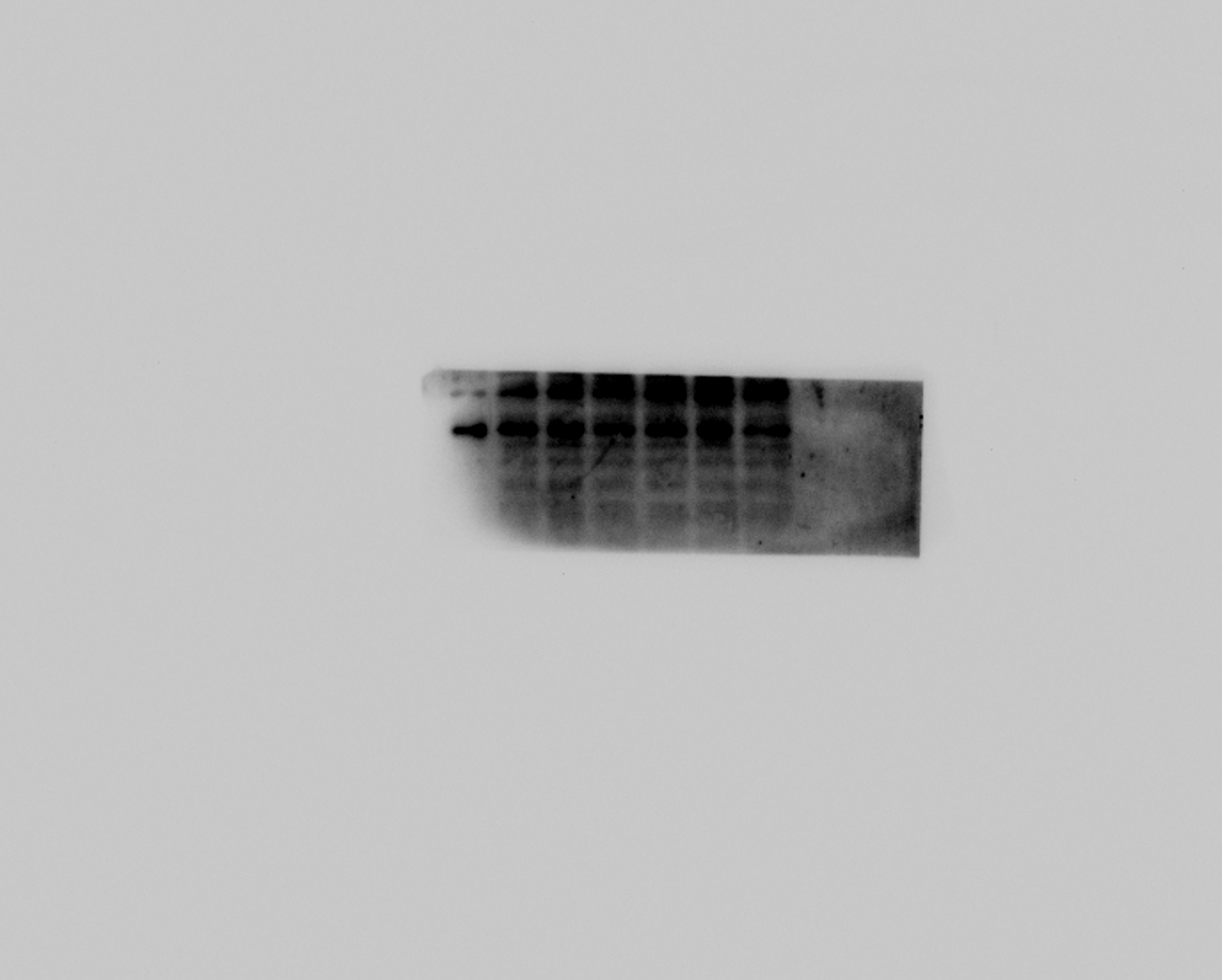

Supplement: Supplementary file 10 — Source data Fig. 5 [file 44318_2024_359_MOESM10_ESM.zip › Figure 5/Fig 5D/whole cell extracts/2-p27-1.Tif]

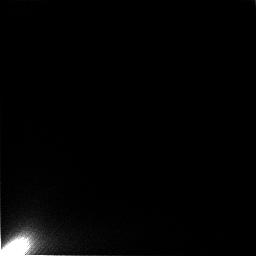

Supplement: Supplementary file 10 — Source data Fig. 5 [file 44318_2024_359_MOESM10_ESM.zip › Figure 5/Fig 5G/Gln+/Fig 5G Gln+ Pearson correlation.tif]

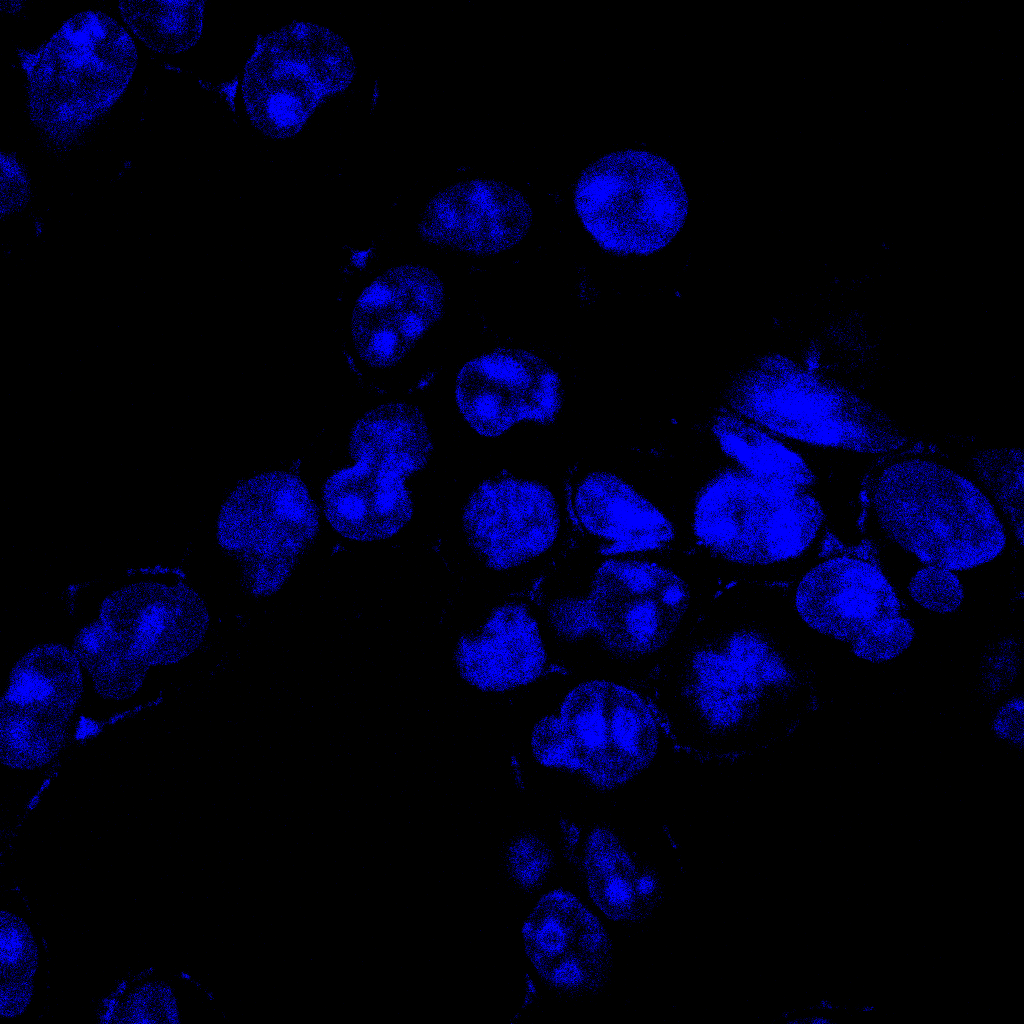

Supplement: Supplementary file 10 — Source data Fig. 5 [file 44318_2024_359_MOESM10_ESM.zip › Figure 5/Fig 5G/Gln+/Gln+ -Hoechst.tif]

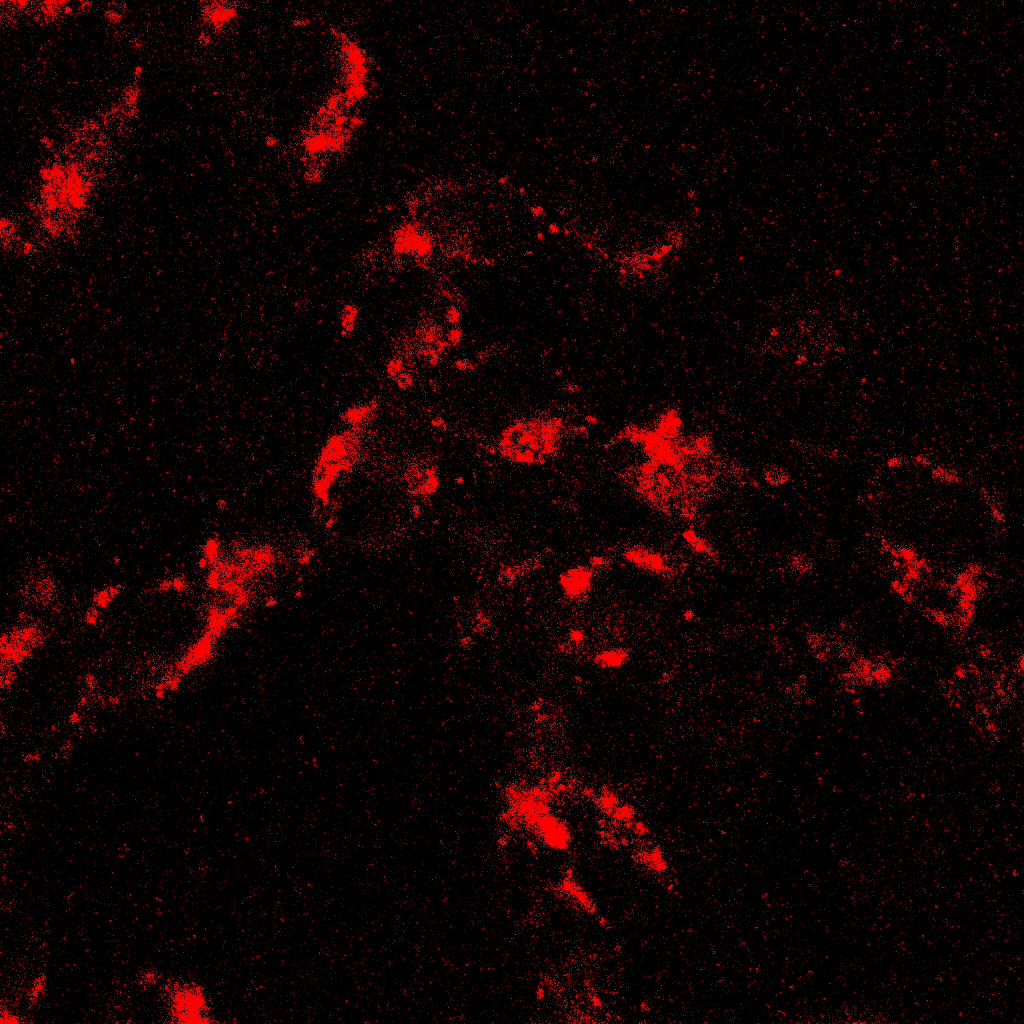

Supplement: Supplementary file 10 — Source data Fig. 5 [file 44318_2024_359_MOESM10_ESM.zip › Figure 5/Fig 5G/Gln+/Gln+ -LAMP1.tif]

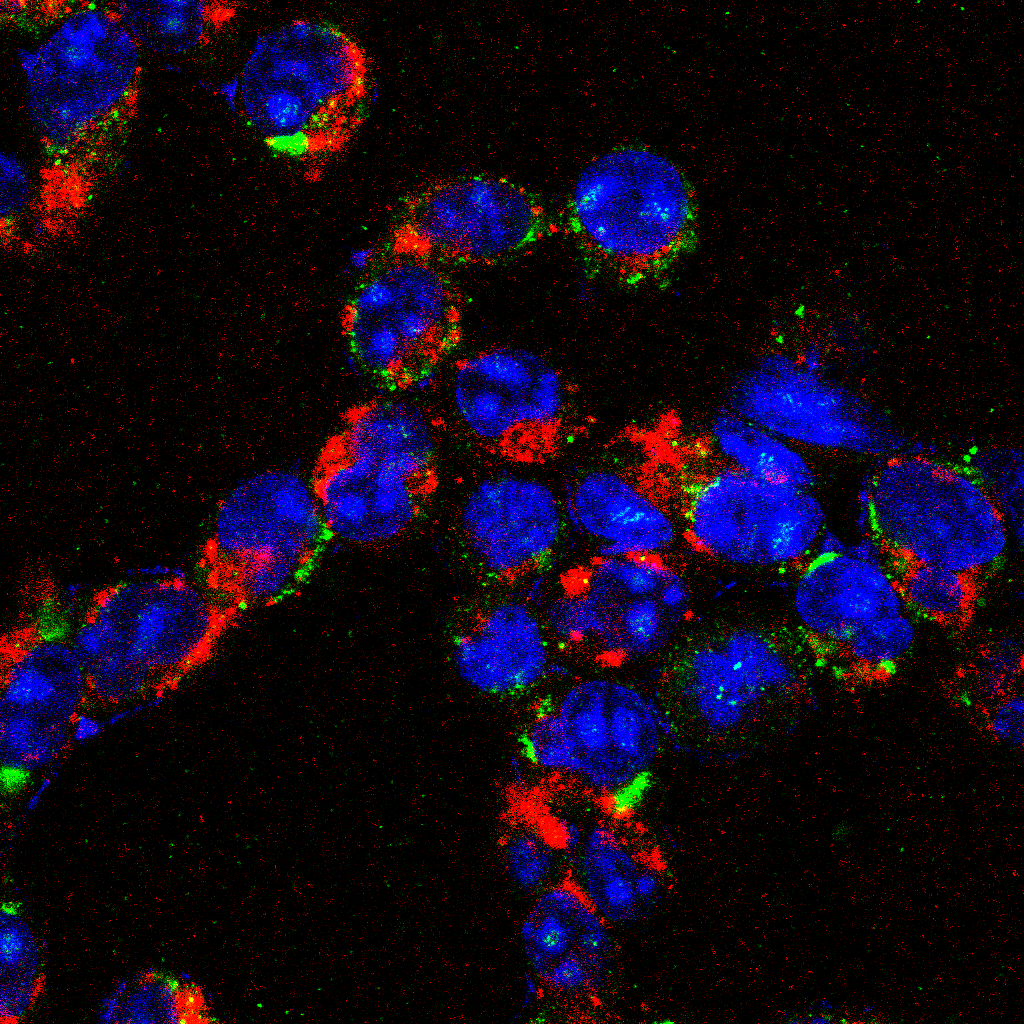

Supplement: Supplementary file 10 — Source data Fig. 5 [file 44318_2024_359_MOESM10_ESM.zip › Figure 5/Fig 5G/Gln+/Gln+ -p27-merge.tif]

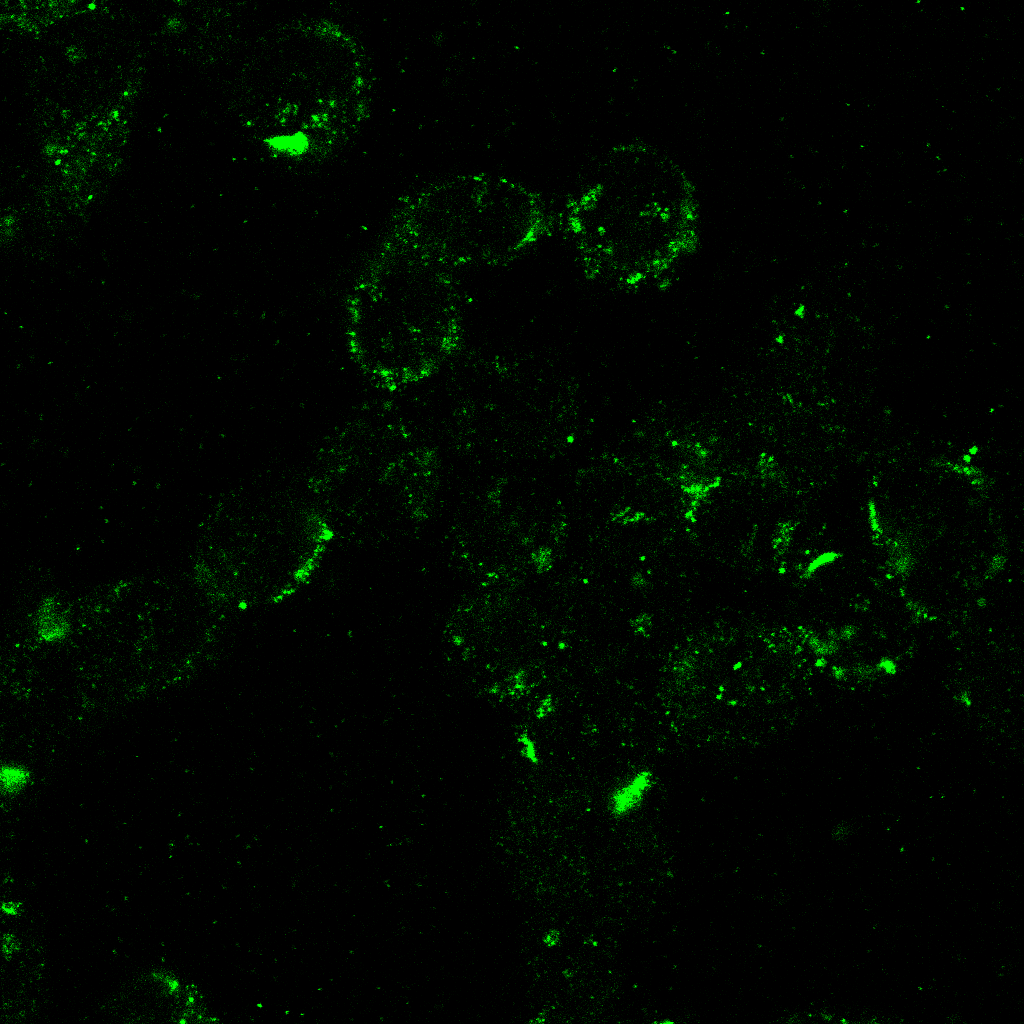

Supplement: Supplementary file 10 — Source data Fig. 5 [file 44318_2024_359_MOESM10_ESM.zip › Figure 5/Fig 5G/Gln+/Gln+ -p27.tif]

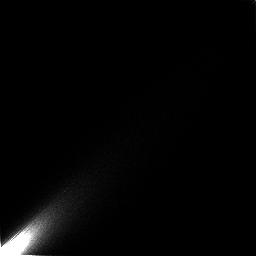

Supplement: Supplementary file 10 — Source data Fig. 5 [file 44318_2024_359_MOESM10_ESM.zip › Figure 5/Fig 5G/Gln-/Fig 5G Gln- Pearson correlation .jpg]

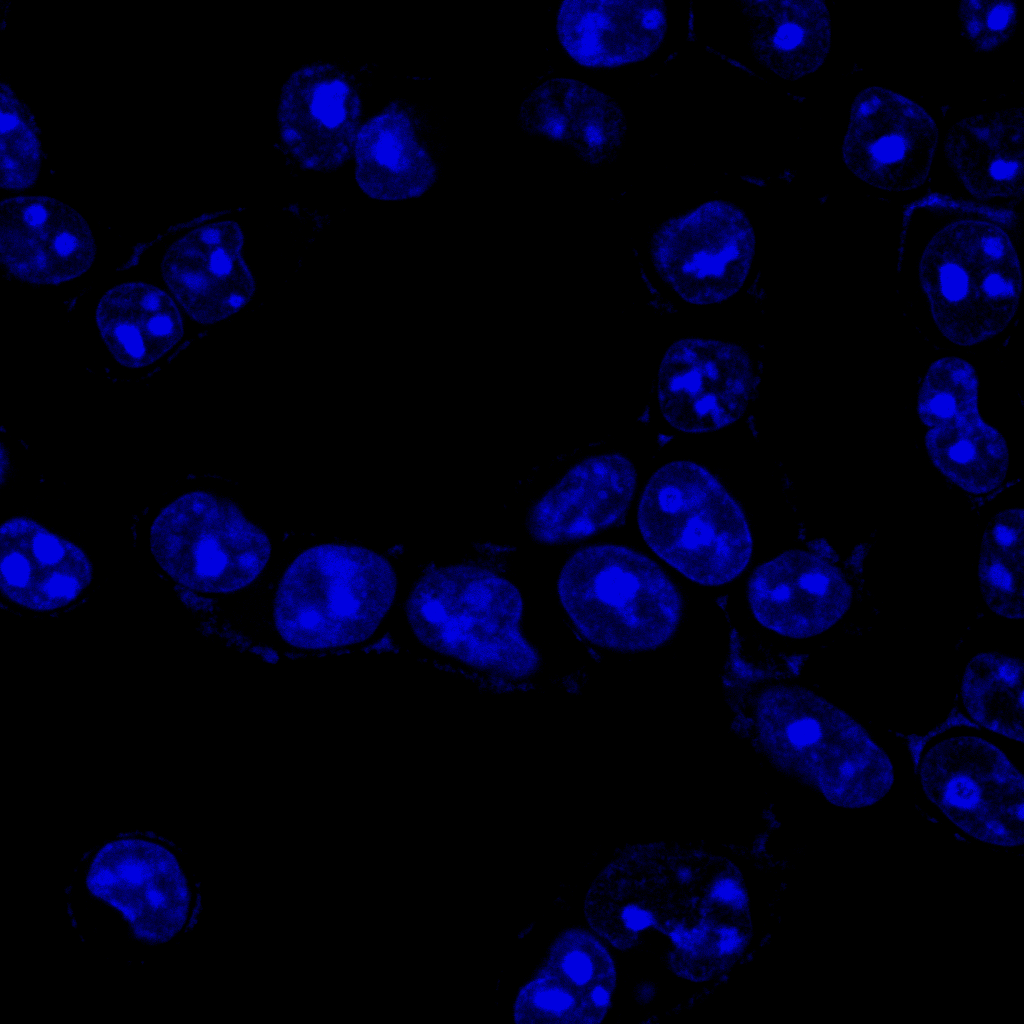

Supplement: Supplementary file 10 — Source data Fig. 5 [file 44318_2024_359_MOESM10_ESM.zip › Figure 5/Fig 5G/Gln-/Gln- -Hoechst.tif]

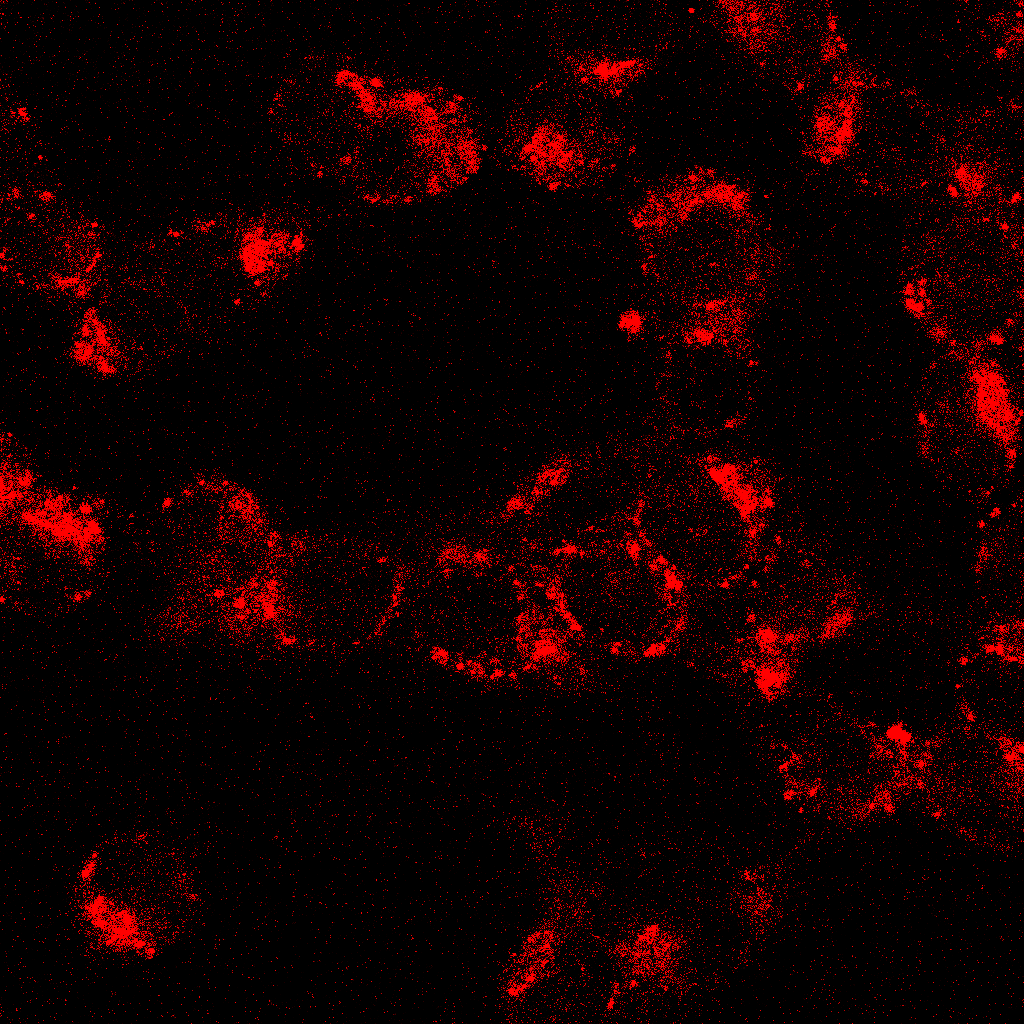

Supplement: Supplementary file 10 — Source data Fig. 5 [file 44318_2024_359_MOESM10_ESM.zip › Figure 5/Fig 5G/Gln-/Gln- -LAMP1.tif]

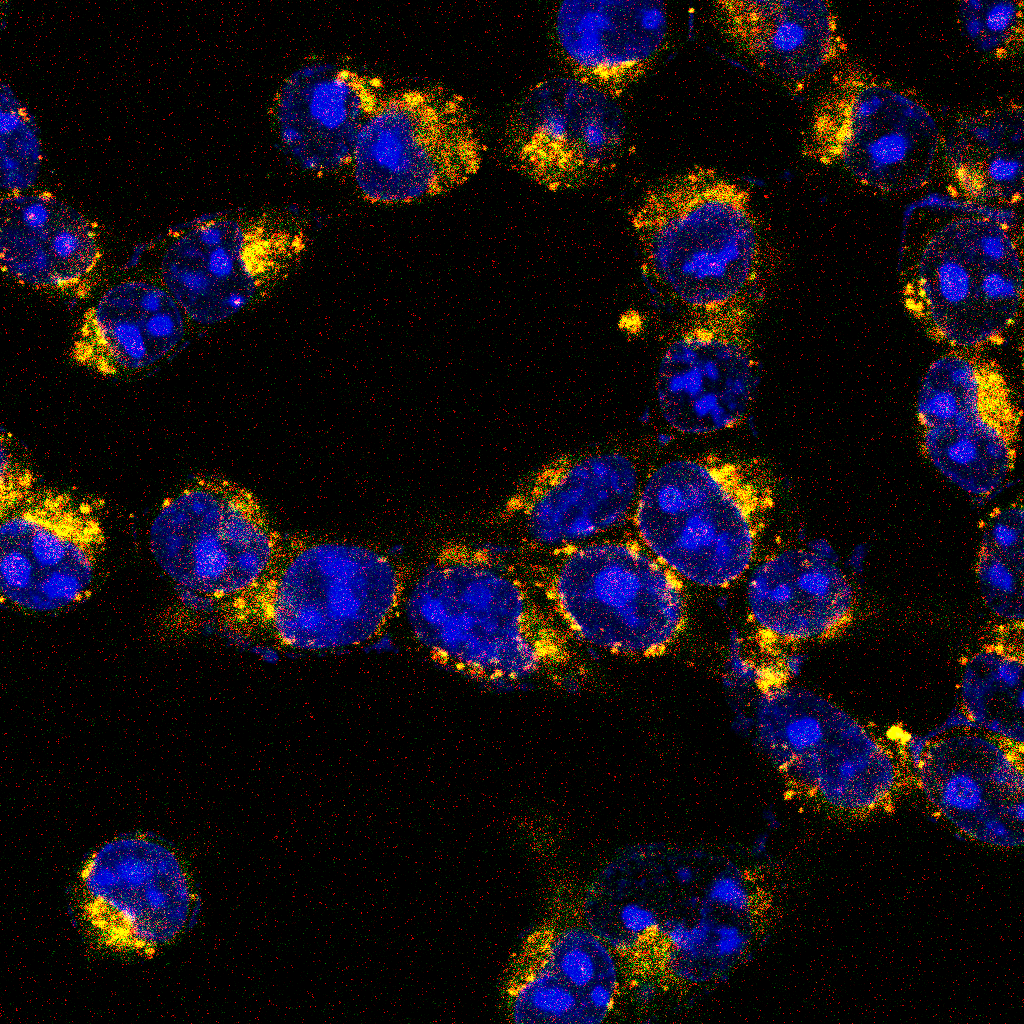

Supplement: Supplementary file 10 — Source data Fig. 5 [file 44318_2024_359_MOESM10_ESM.zip › Figure 5/Fig 5G/Gln-/Gln- -p27-merge.tif]

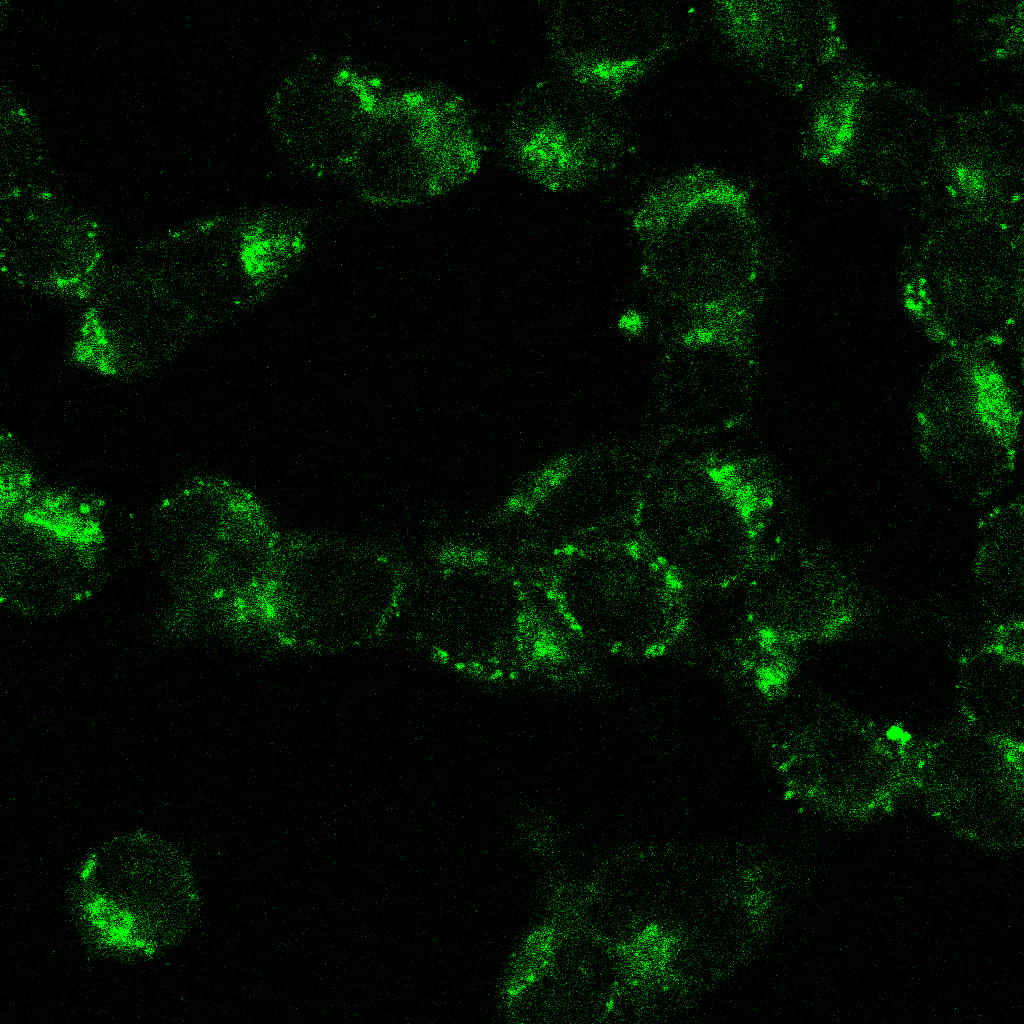

Supplement: Supplementary file 10 — Source data Fig. 5 [file 44318_2024_359_MOESM10_ESM.zip › Figure 5/Fig 5G/Gln-/Gln- -p27.tif]

Fig 5H

MDA-MB-231

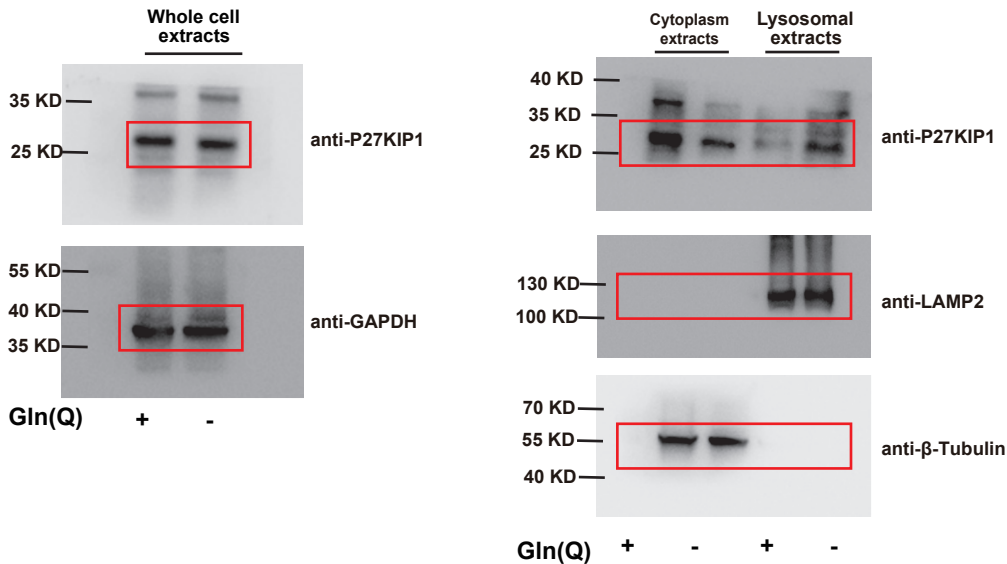

Supplement: Supplementary file 10 — Source data Fig. 5 [file 44318_2024_359_MOESM10_ESM.zip › Figure 5/Fig 5H/Fig 5H.pdf]

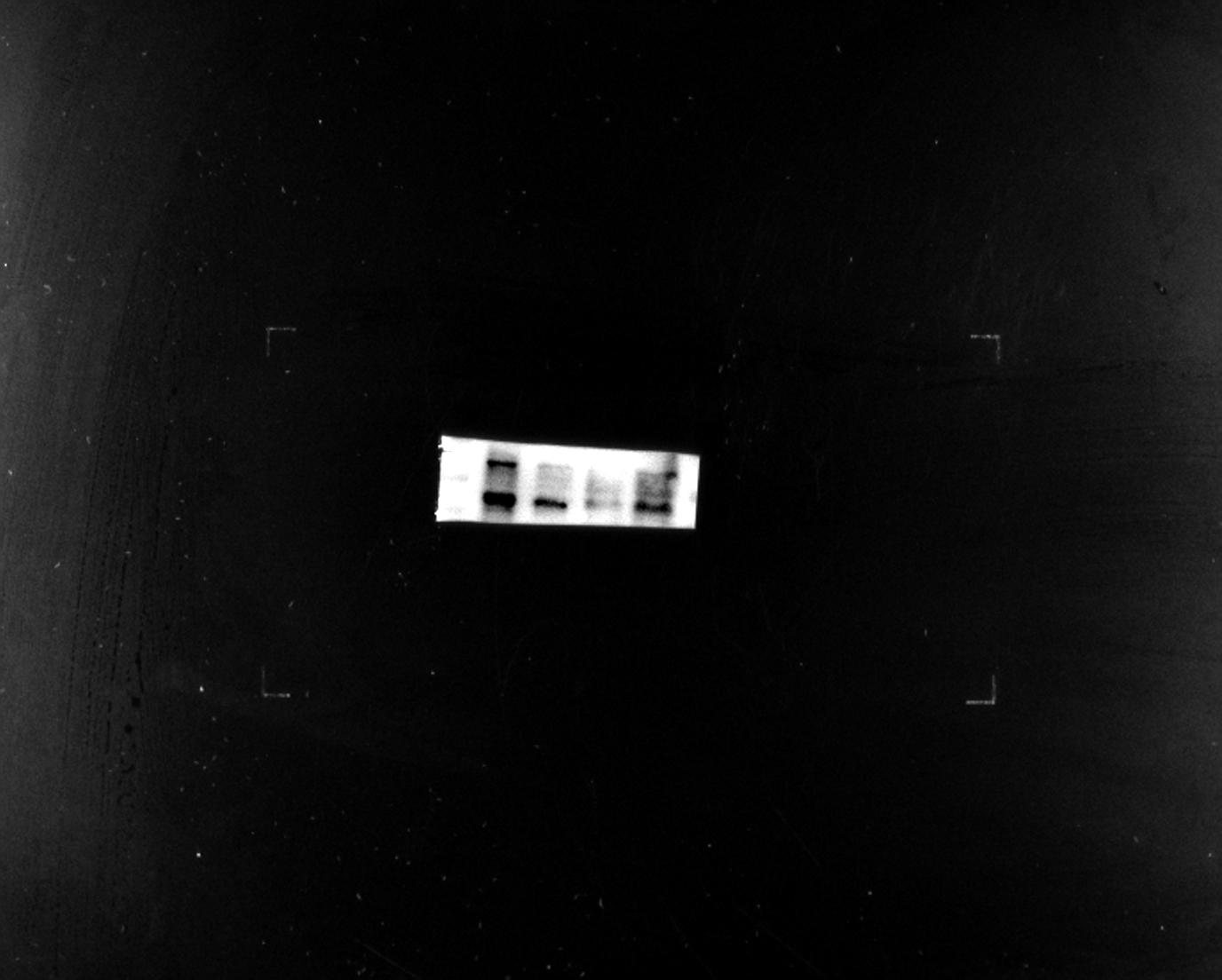

Supplement: Supplementary file 10 — Source data Fig. 5 [file 44318_2024_359_MOESM10_ESM.zip › Figure 5/Fig 5H/cytoplasm-lysosome extraxts/1-P27KIP1-merge.Tif]

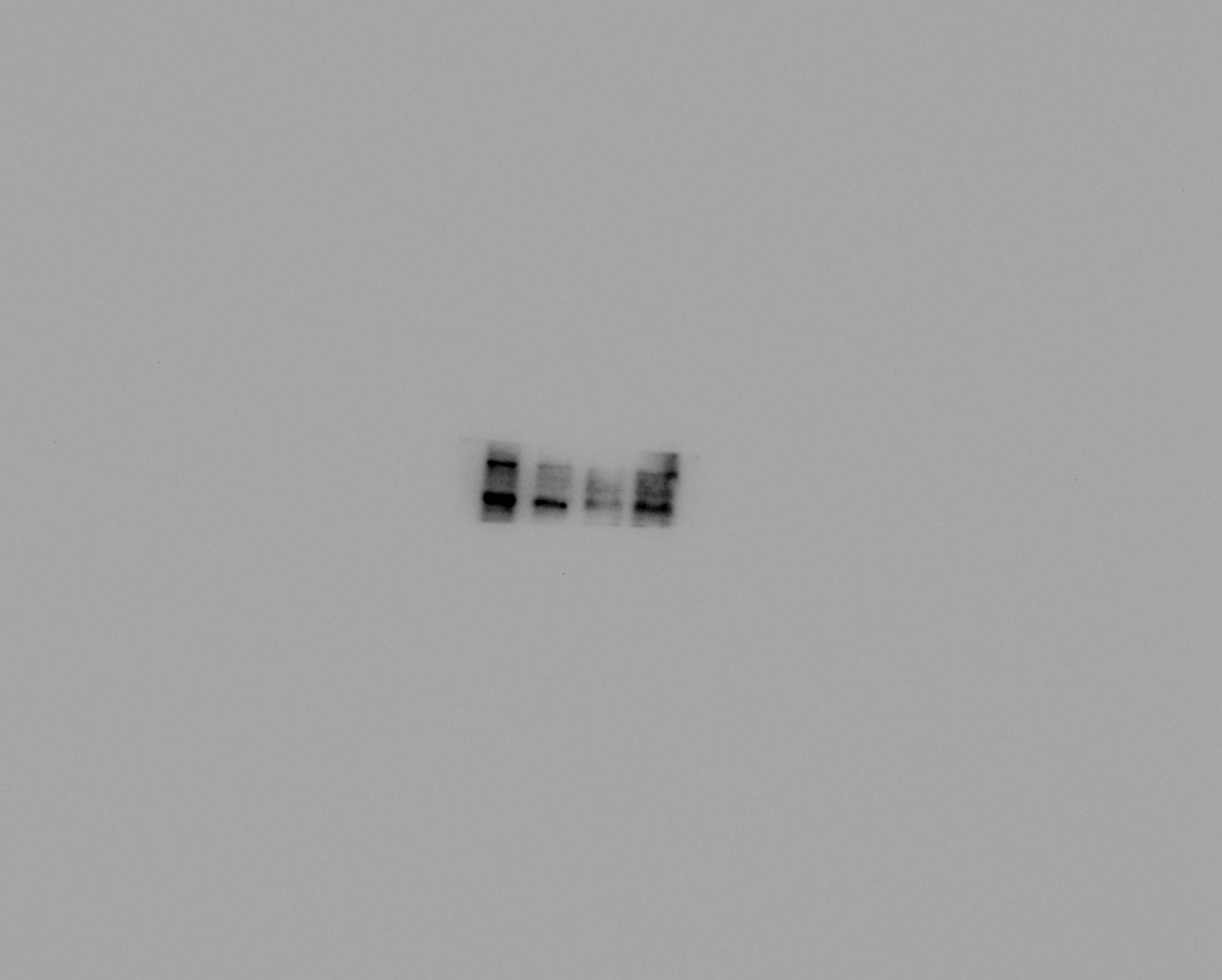

Supplement: Supplementary file 10 — Source data Fig. 5 [file 44318_2024_359_MOESM10_ESM.zip › Figure 5/Fig 5H/cytoplasm-lysosome extraxts/1-P27KIP1.Tif]

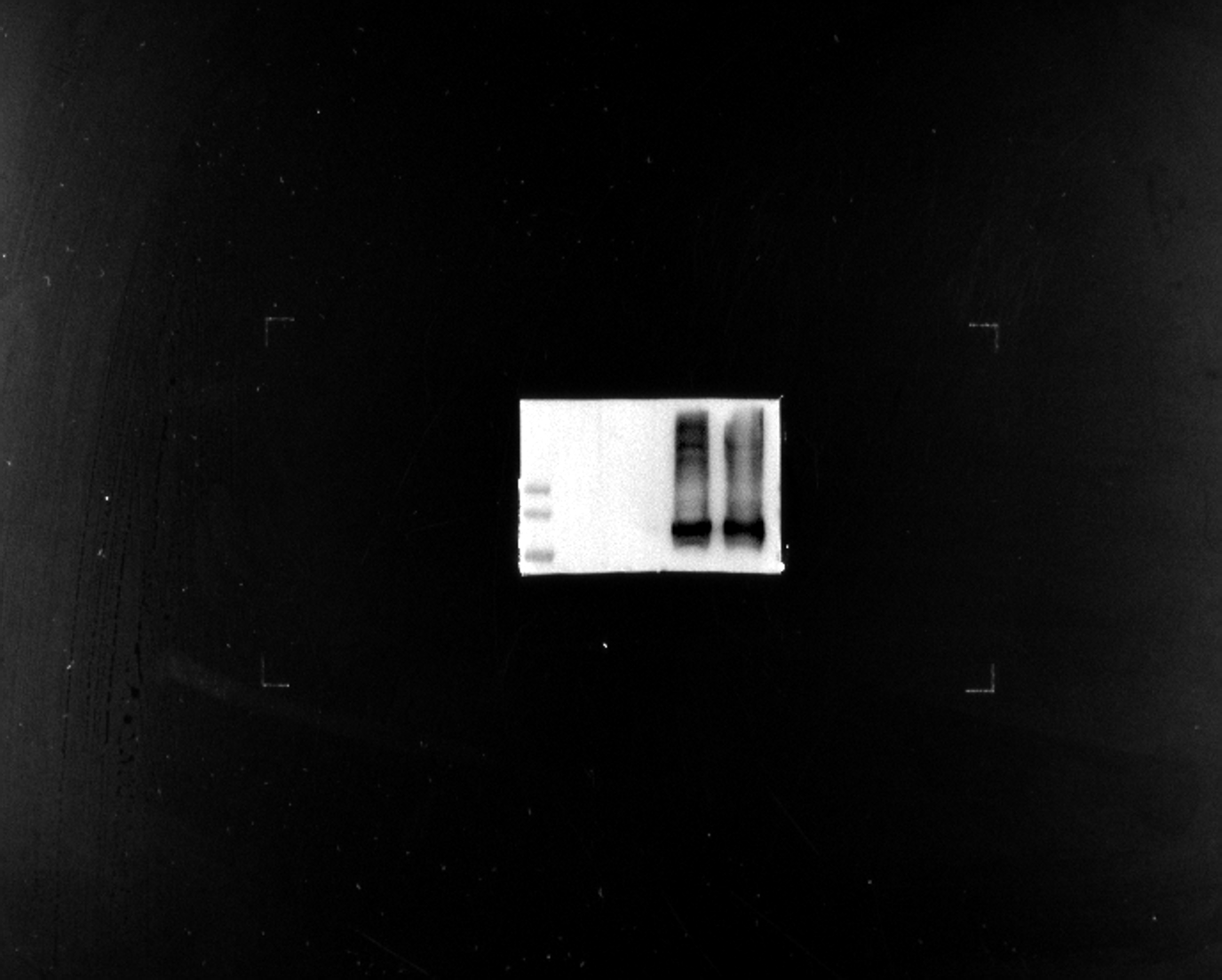

Supplement: Supplementary file 10 — Source data Fig. 5 [file 44318_2024_359_MOESM10_ESM.zip › Figure 5/Fig 5H/cytoplasm-lysosome extraxts/2-LAMP2-merge.Tif]

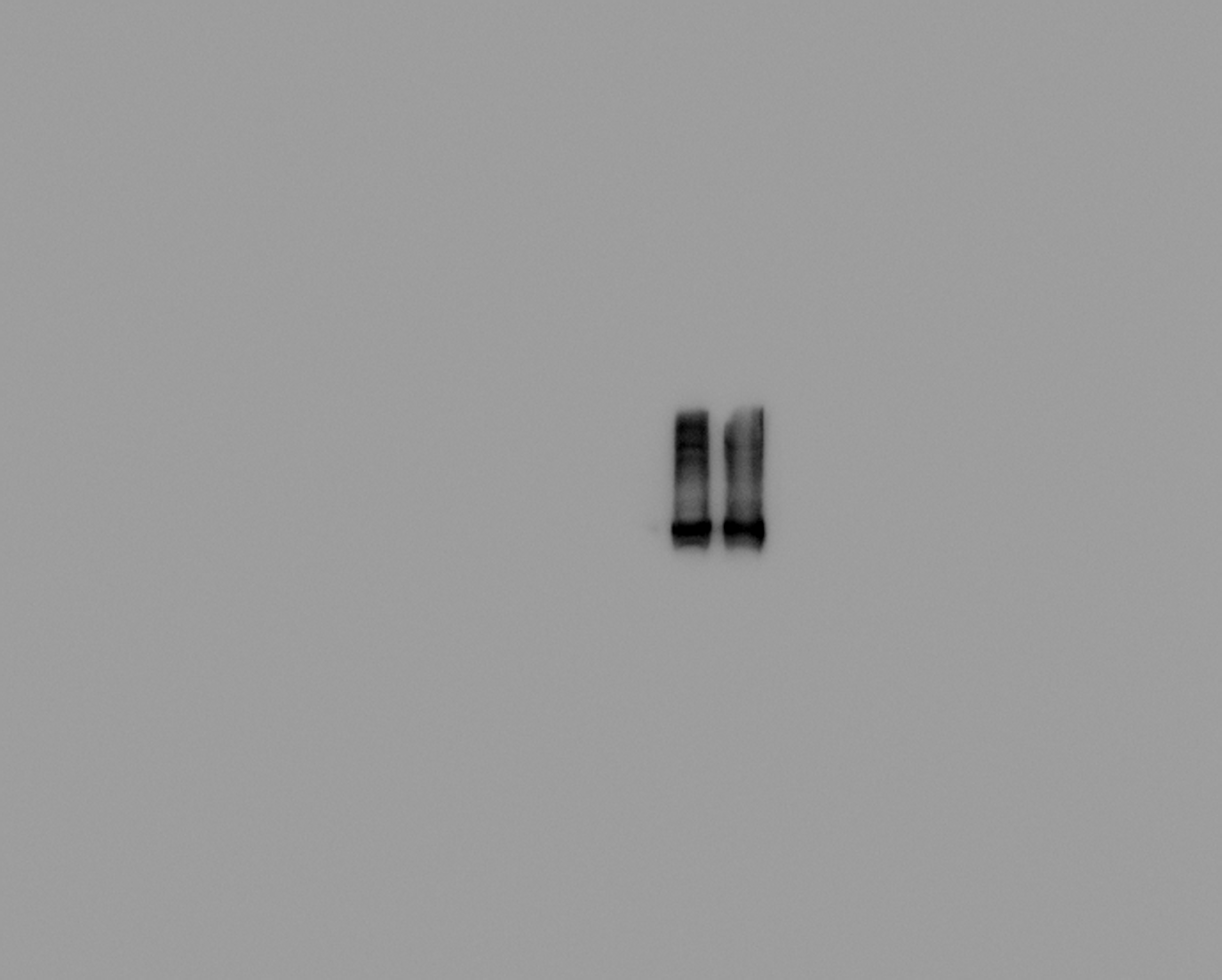

Supplement: Supplementary file 10 — Source data Fig. 5 [file 44318_2024_359_MOESM10_ESM.zip › Figure 5/Fig 5H/cytoplasm-lysosome extraxts/2-LAMP2.Tif]

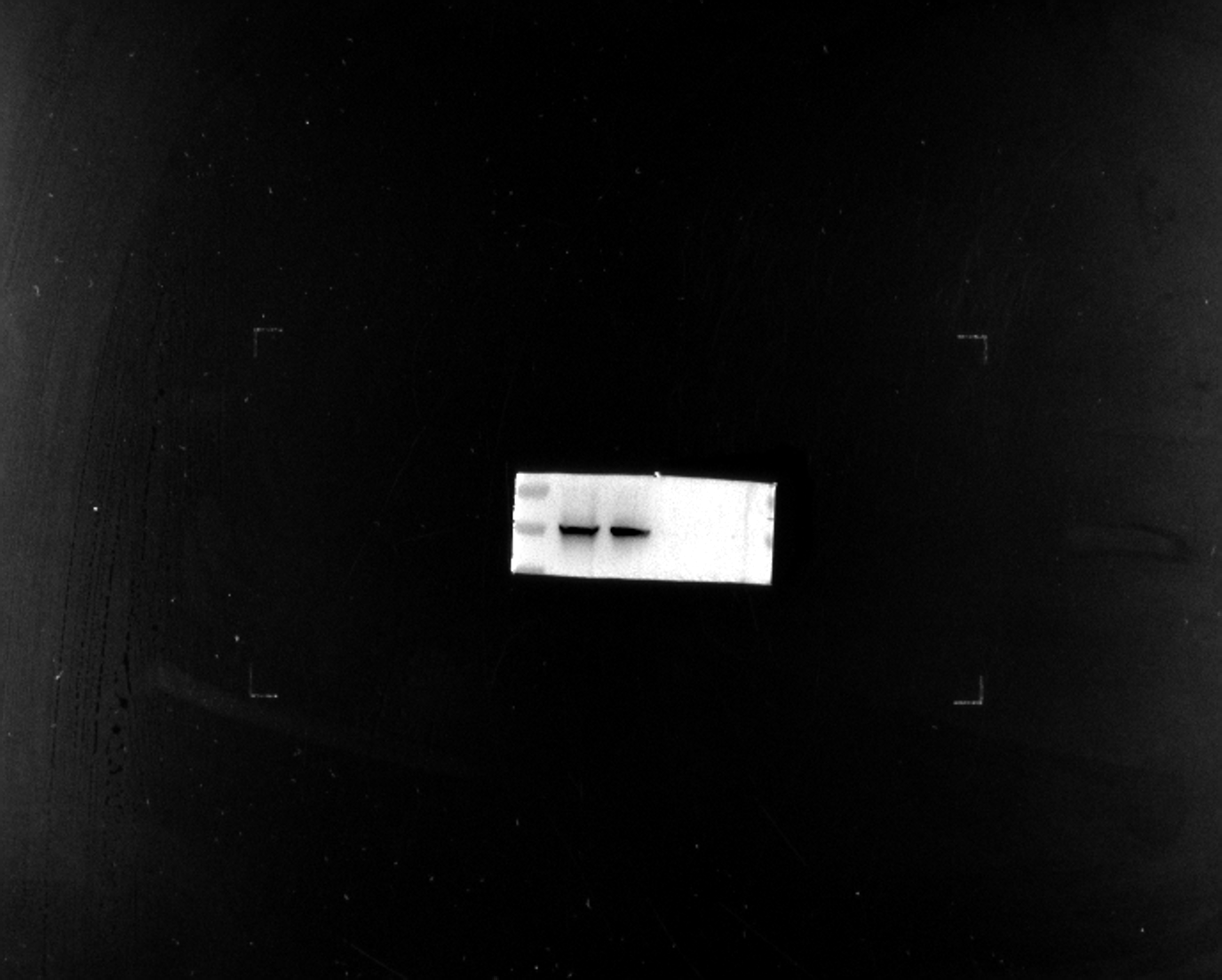

Supplement: Supplementary file 10 — Source data Fig. 5 [file 44318_2024_359_MOESM10_ESM.zip › Figure 5/Fig 5H/cytoplasm-lysosome extraxts/3-TUBULIN-merge.Tif]

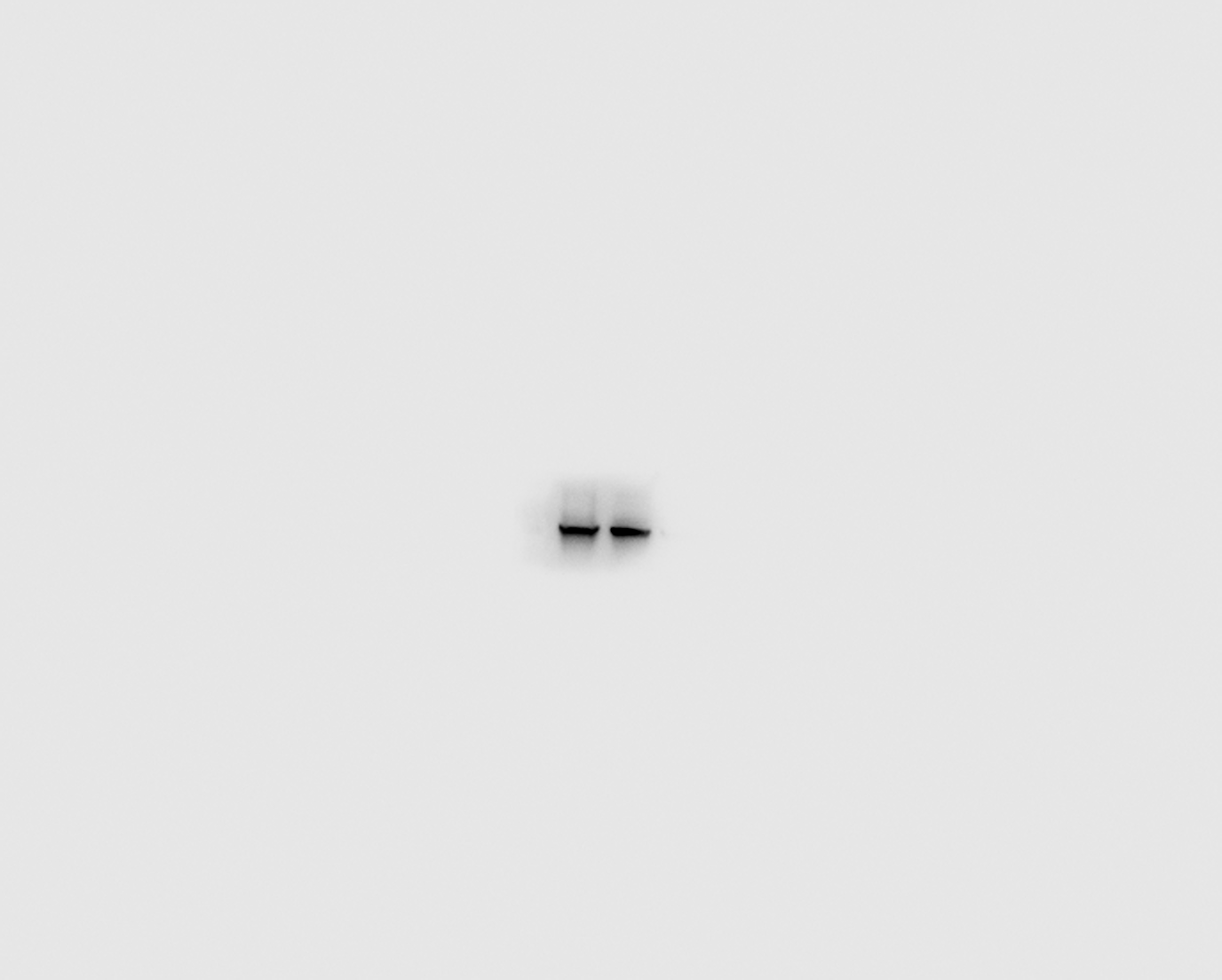

Supplement: Supplementary file 10 — Source data Fig. 5 [file 44318_2024_359_MOESM10_ESM.zip › Figure 5/Fig 5H/cytoplasm-lysosome extraxts/3-TUBULIN.Tif]

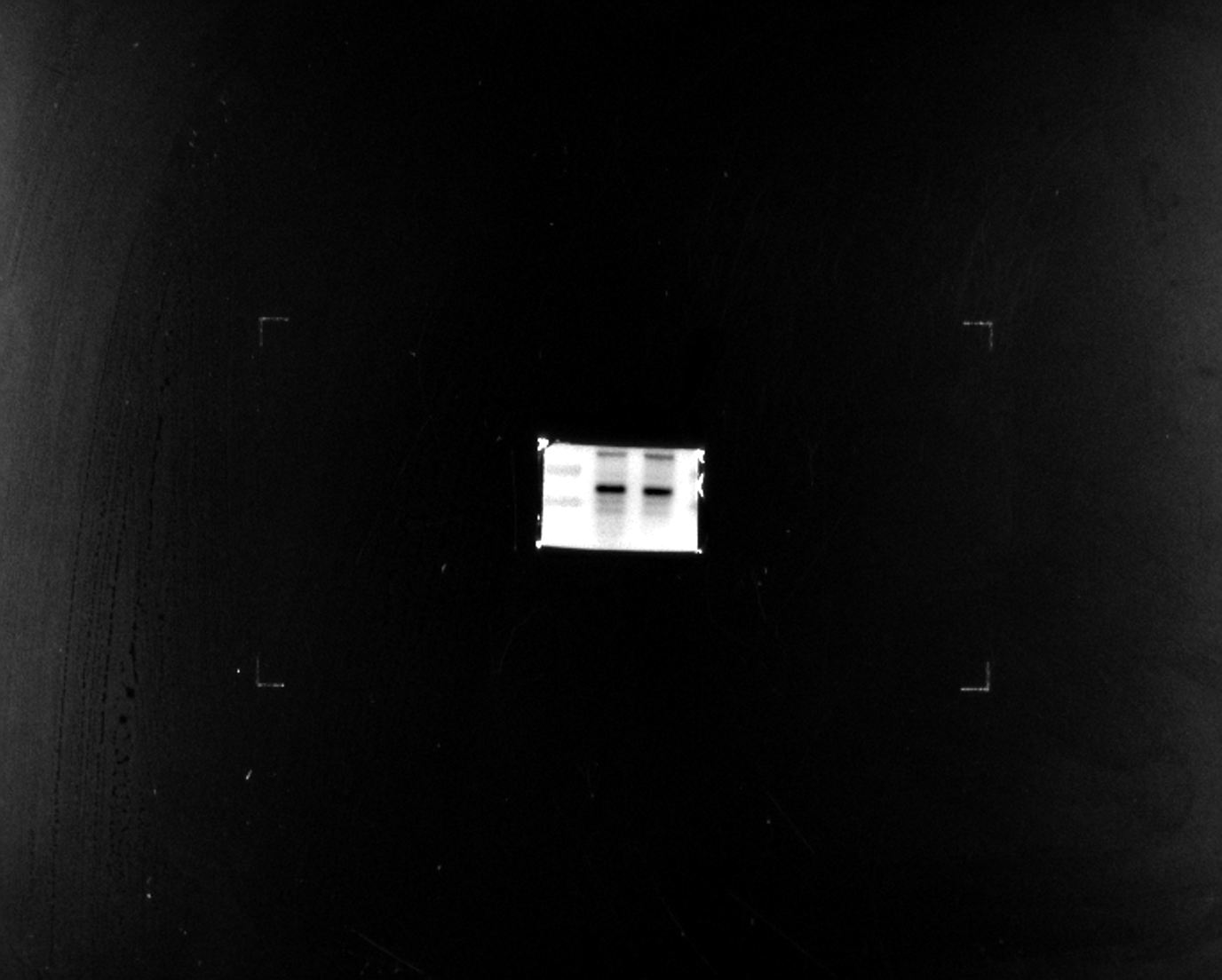

Supplement: Supplementary file 10 — Source data Fig. 5 [file 44318_2024_359_MOESM10_ESM.zip › Figure 5/Fig 5H/whole cell extracts/1-P27KIP1-merge.Tif]

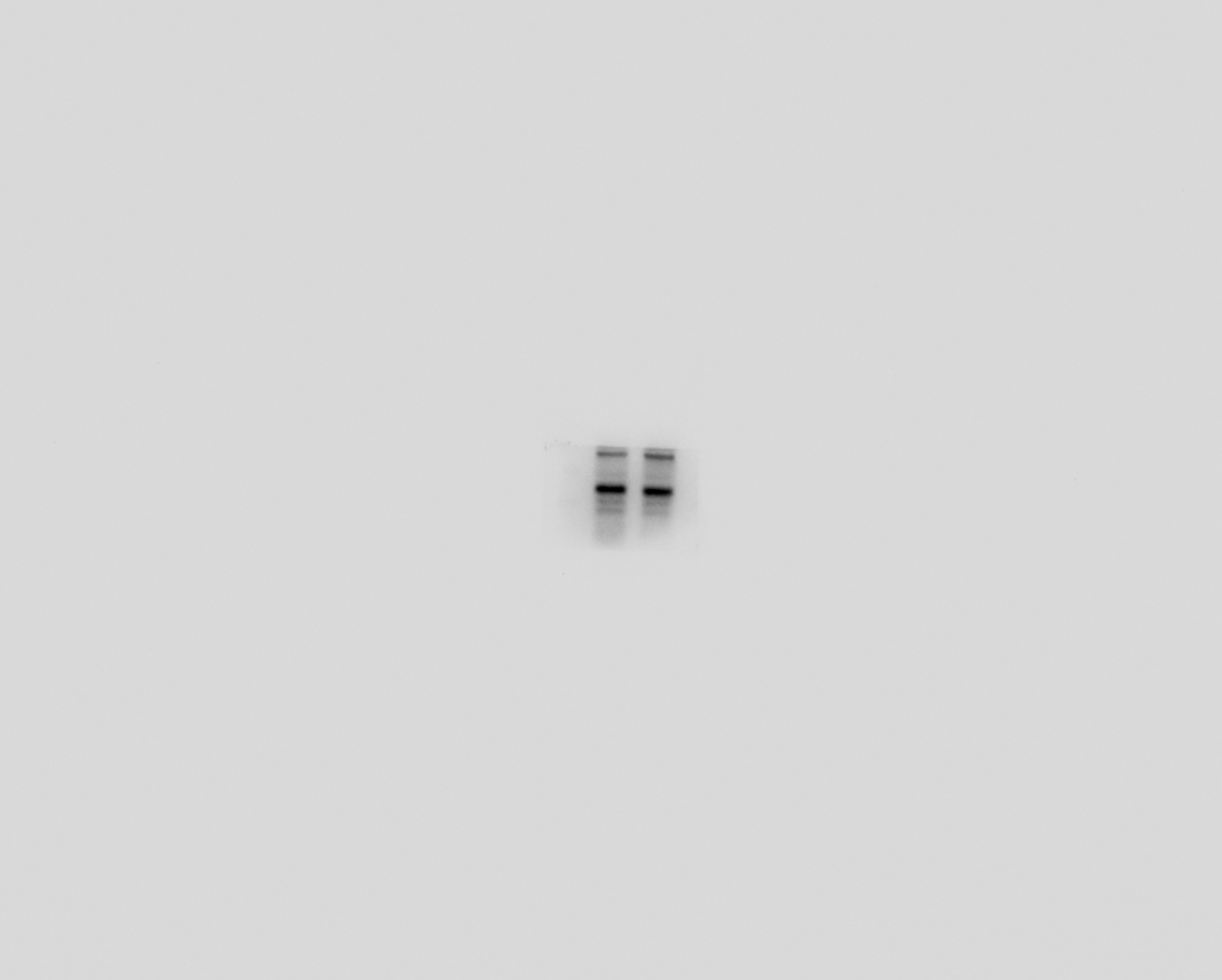

Supplement: Supplementary file 10 — Source data Fig. 5 [file 44318_2024_359_MOESM10_ESM.zip › Figure 5/Fig 5H/whole cell extracts/1-P27KIP1.Tif]

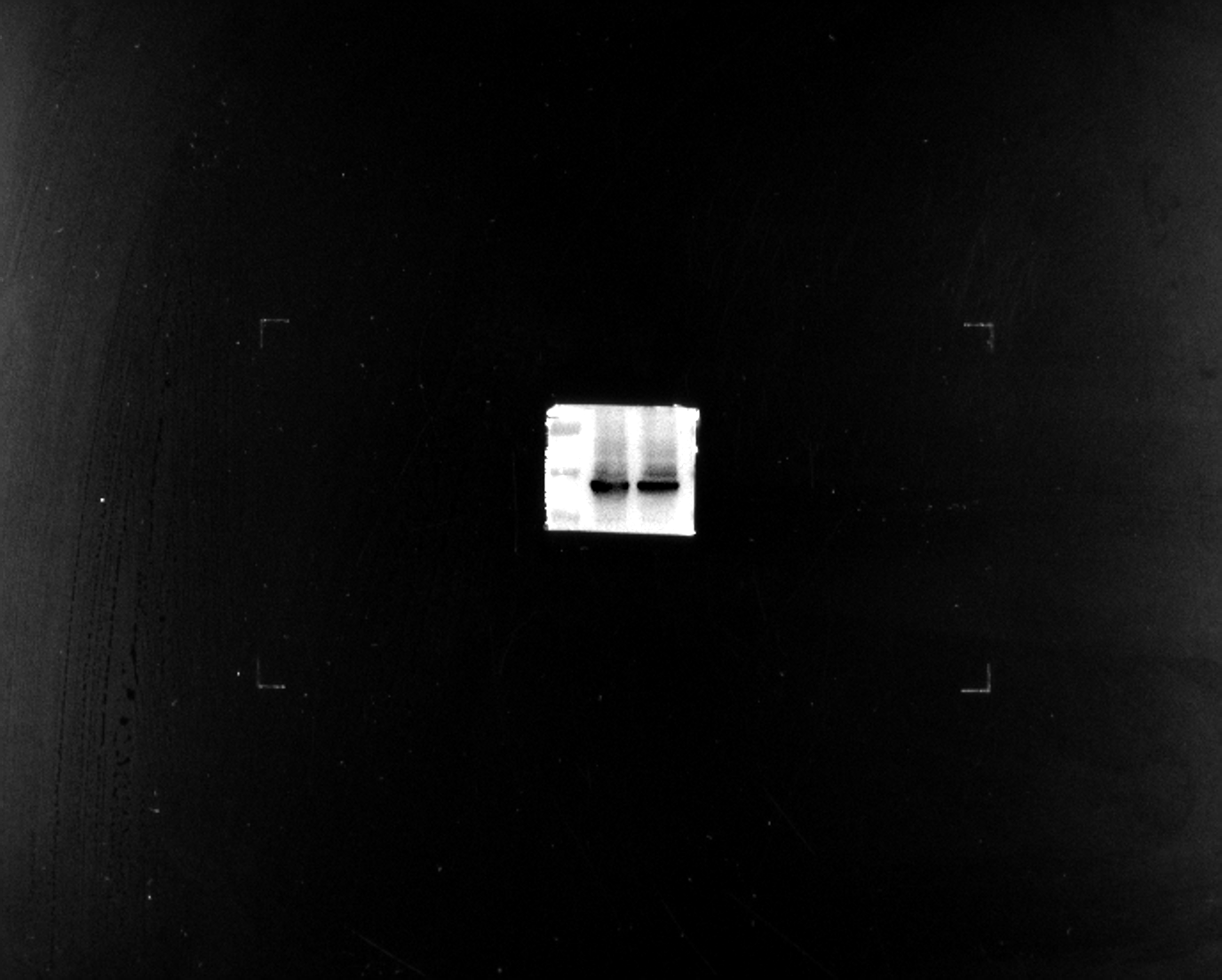

Supplement: Supplementary file 10 — Source data Fig. 5 [file 44318_2024_359_MOESM10_ESM.zip › Figure 5/Fig 5H/whole cell extracts/2-GAPDH-merge.Tif]

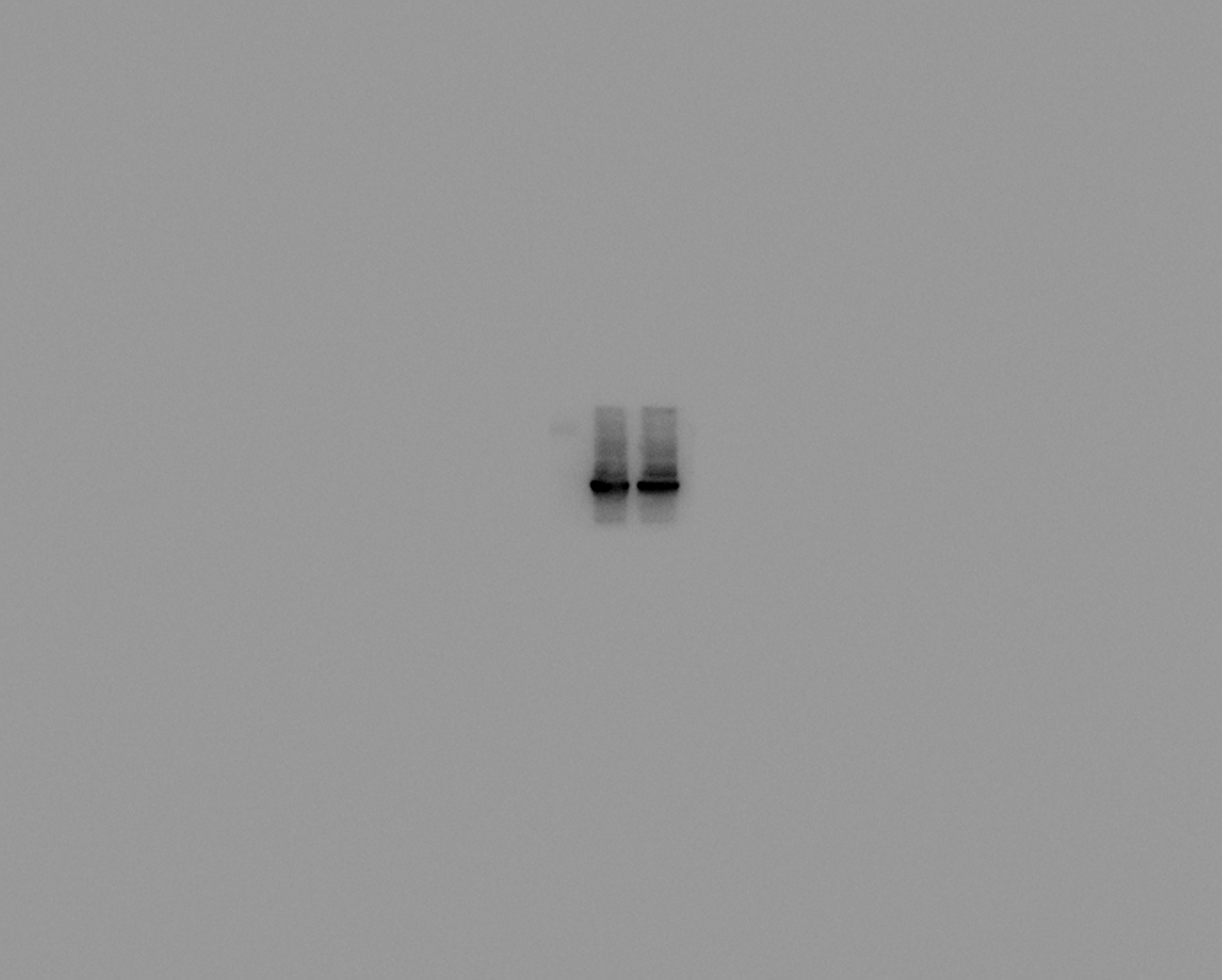

Supplement: Supplementary file 10 — Source data Fig. 5 [file 44318_2024_359_MOESM10_ESM.zip › Figure 5/Fig 5H/whole cell extracts/2-GAPDH.Tif]

Fig 5I

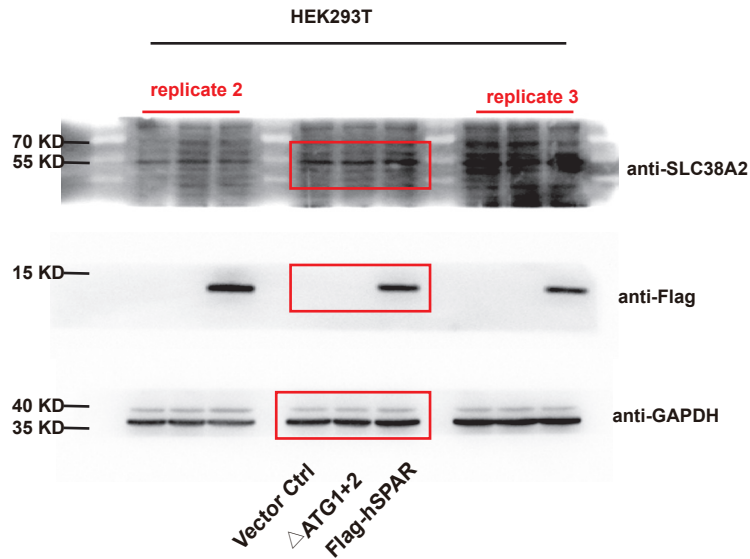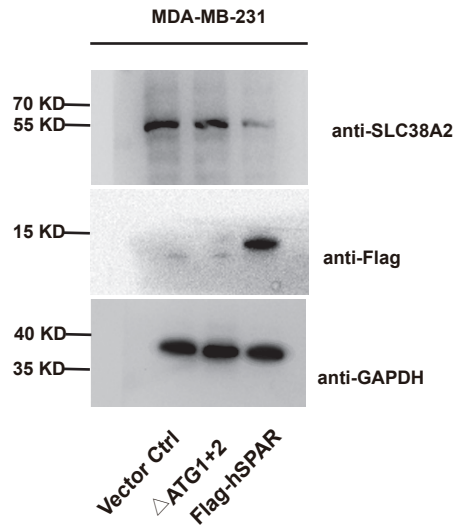

Supplement: Supplementary file 10 — Source data Fig. 5 [file 44318_2024_359_MOESM10_ESM.zip › Figure 5/Fig 5I/Fig 5i.pdf]

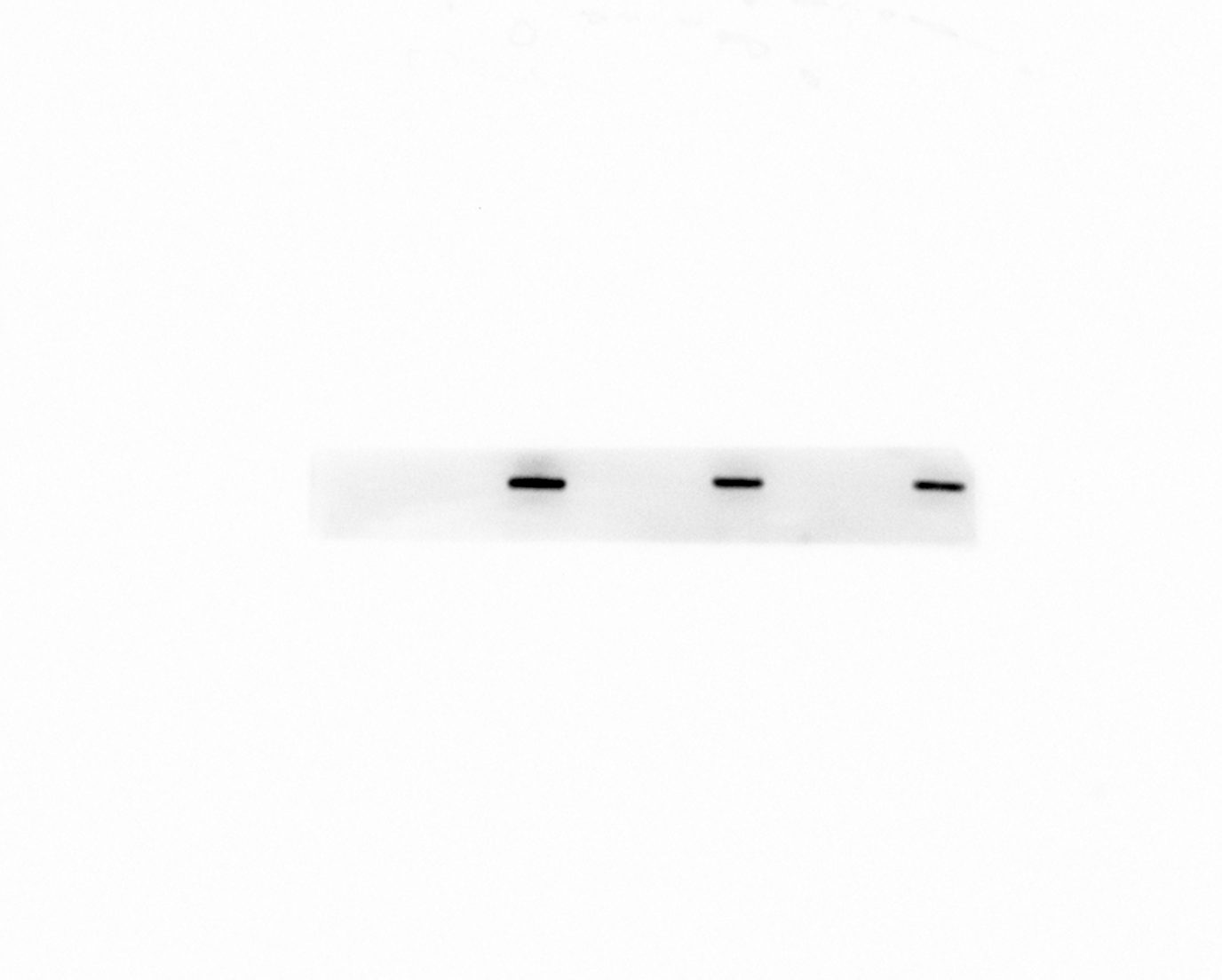

Supplement: Supplementary file 10 — Source data Fig. 5 [file 44318_2024_359_MOESM10_ESM.zip › Figure 5/Fig 5I/HEK293T/Flag-1.Tif]

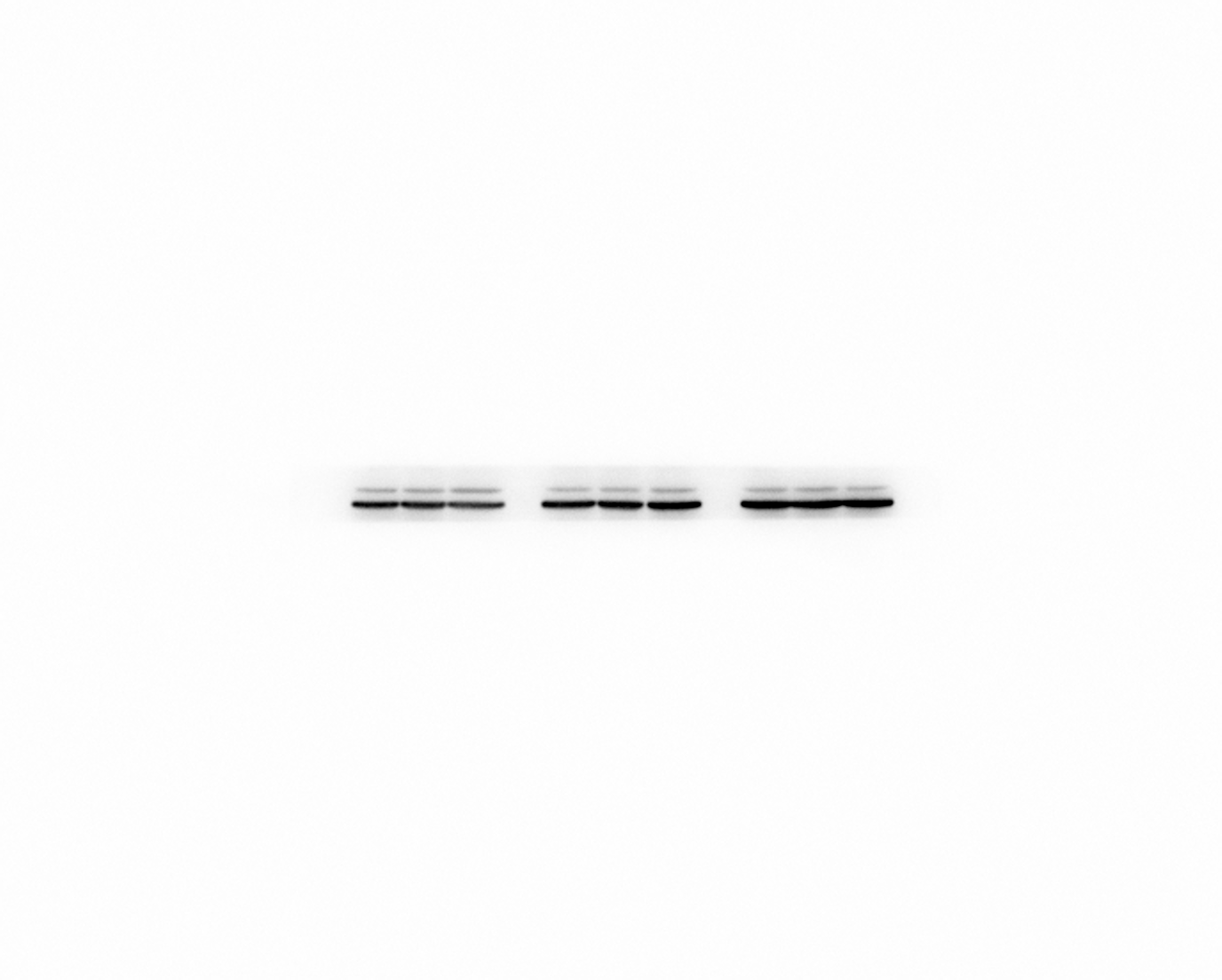

Supplement: Supplementary file 10 — Source data Fig. 5 [file 44318_2024_359_MOESM10_ESM.zip › Figure 5/Fig 5I/HEK293T/GAPDH-1.Tif]

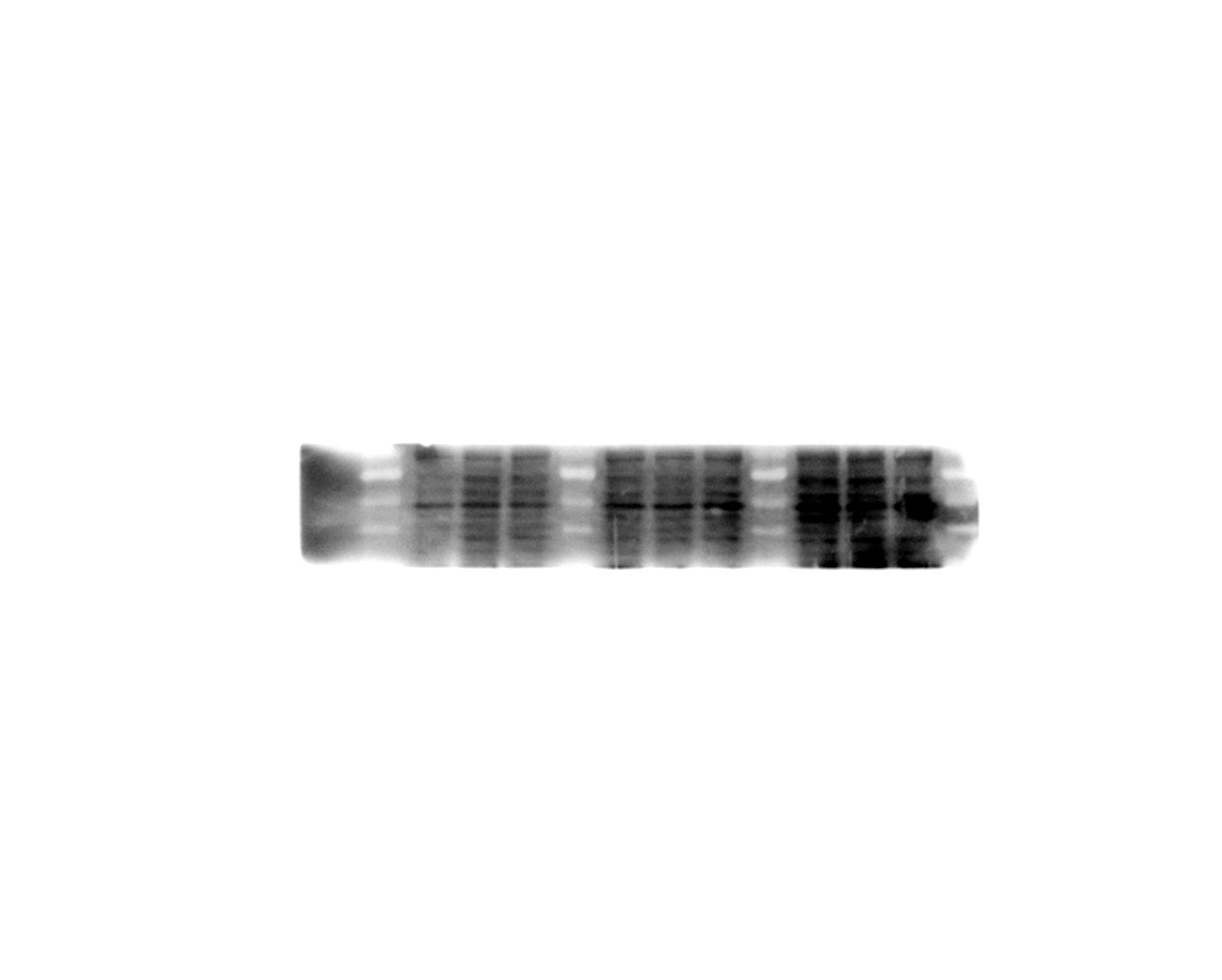

Supplement: Supplementary file 10 — Source data Fig. 5 [file 44318_2024_359_MOESM10_ESM.zip › Figure 5/Fig 5I/HEK293T/SLC38A2-1.Tif]

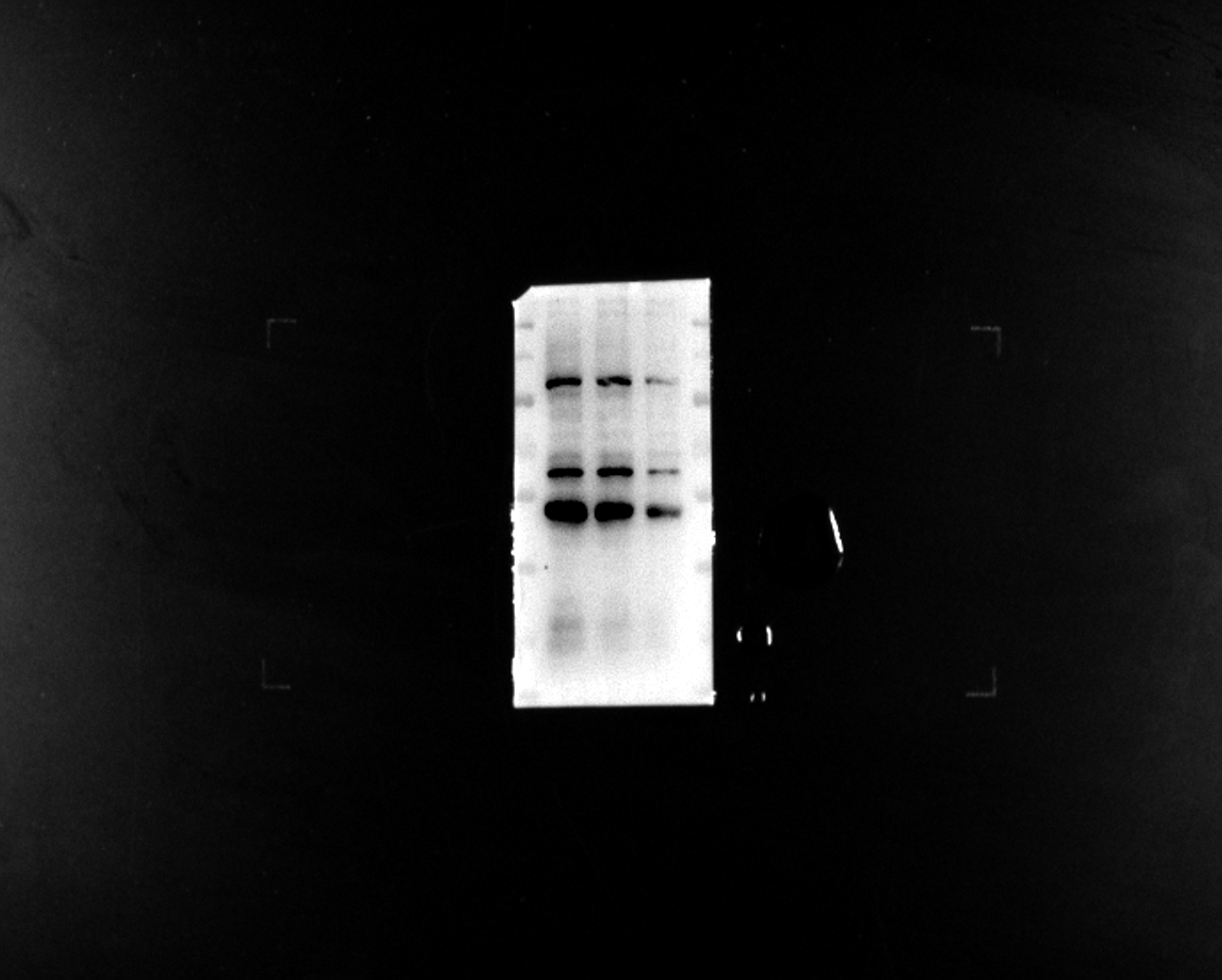

Supplement: Supplementary file 10 — Source data Fig. 5 [file 44318_2024_359_MOESM10_ESM.zip › Figure 5/Fig 5I/MDA-MB-231/1-SLC38A2-merge.Tif]

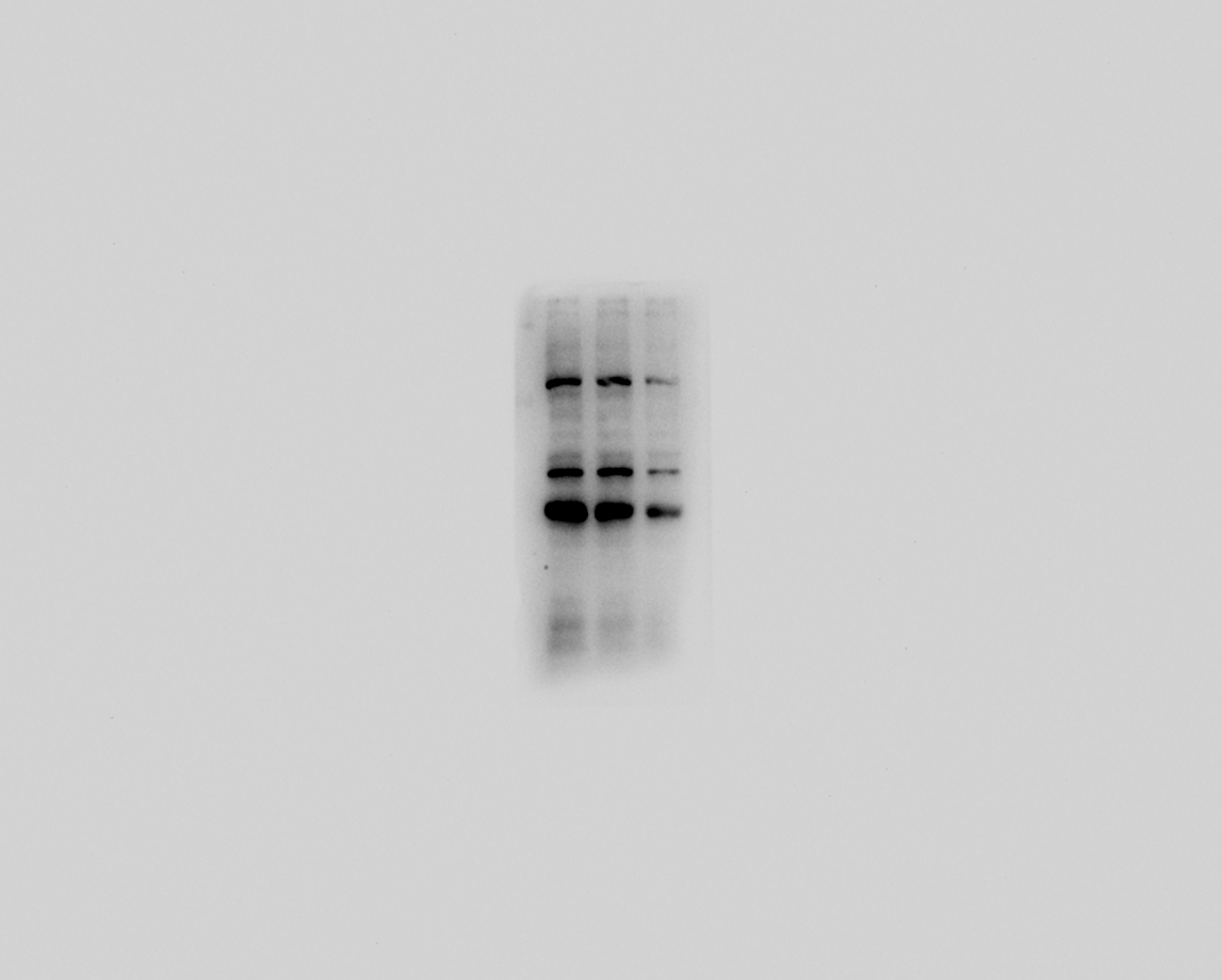

Supplement: Supplementary file 10 — Source data Fig. 5 [file 44318_2024_359_MOESM10_ESM.zip › Figure 5/Fig 5I/MDA-MB-231/1-SLC38A2.Tif]

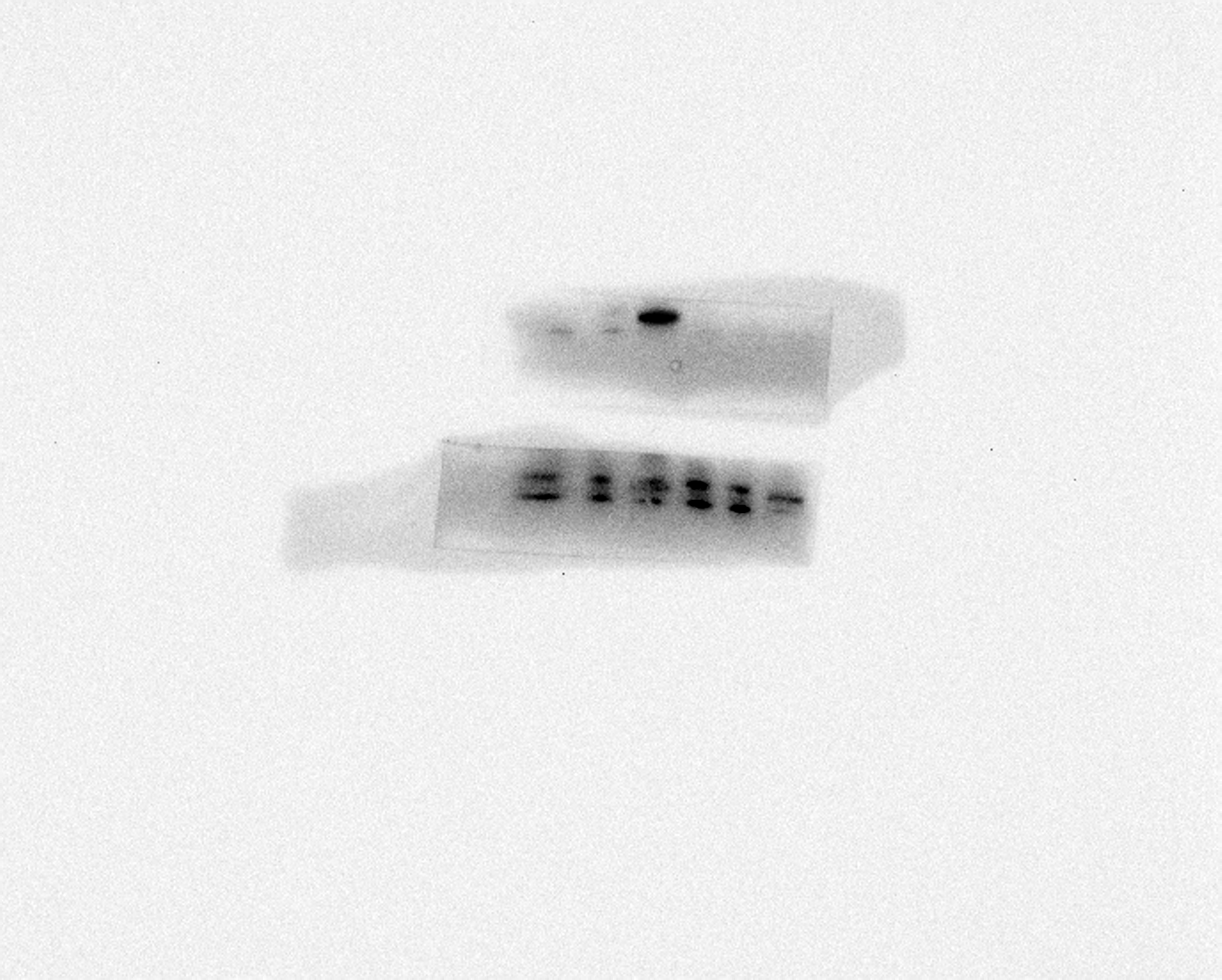

Supplement: Supplementary file 10 — Source data Fig. 5 [file 44318_2024_359_MOESM10_ESM.zip › Figure 5/Fig 5I/MDA-MB-231/2-FLAG.Tif]

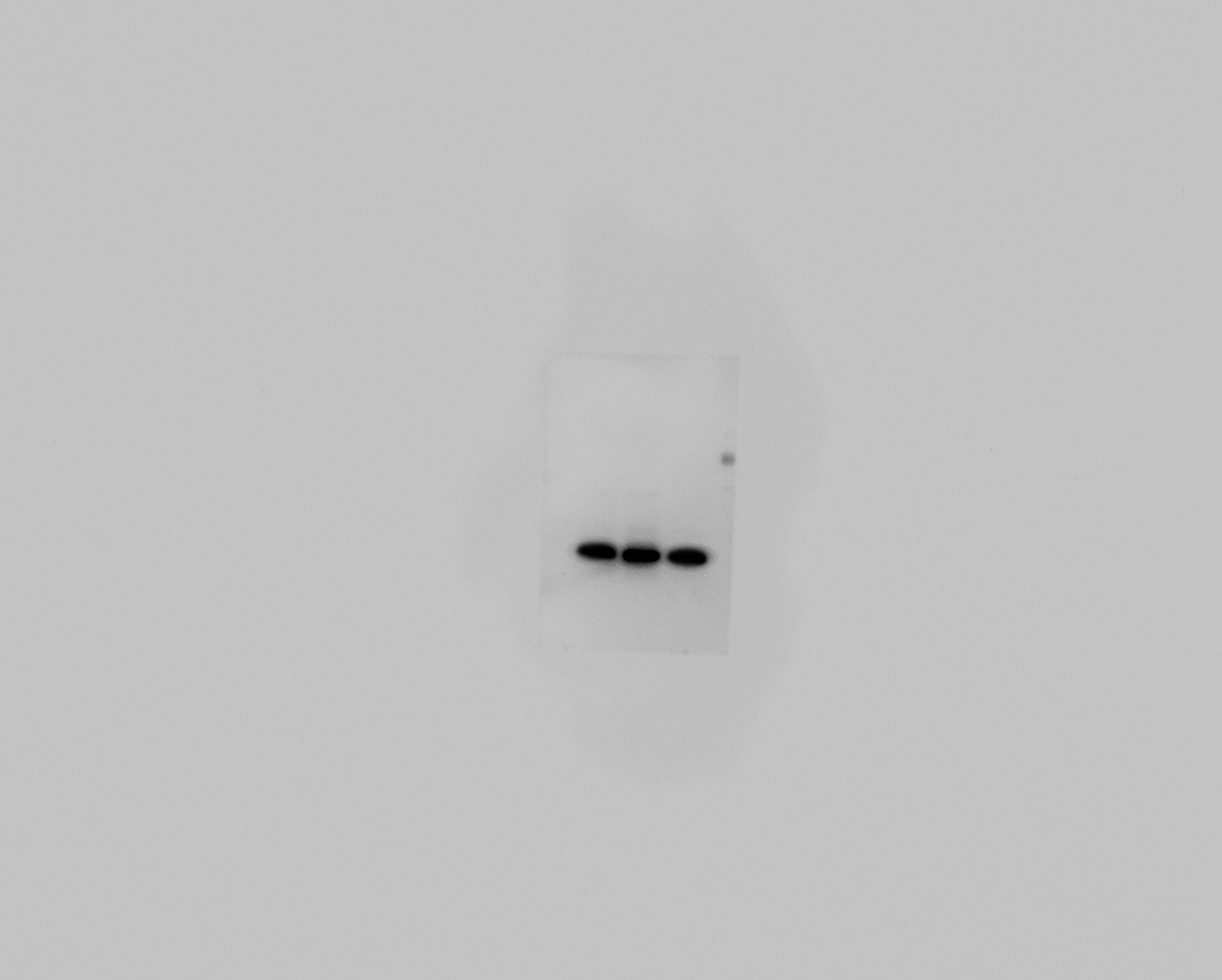

Supplement: Supplementary file 10 — Source data Fig. 5 [file 44318_2024_359_MOESM10_ESM.zip › Figure 5/Fig 5I/MDA-MB-231/3-GAPDH.tif]

Fig 5K

MDA-MB-231

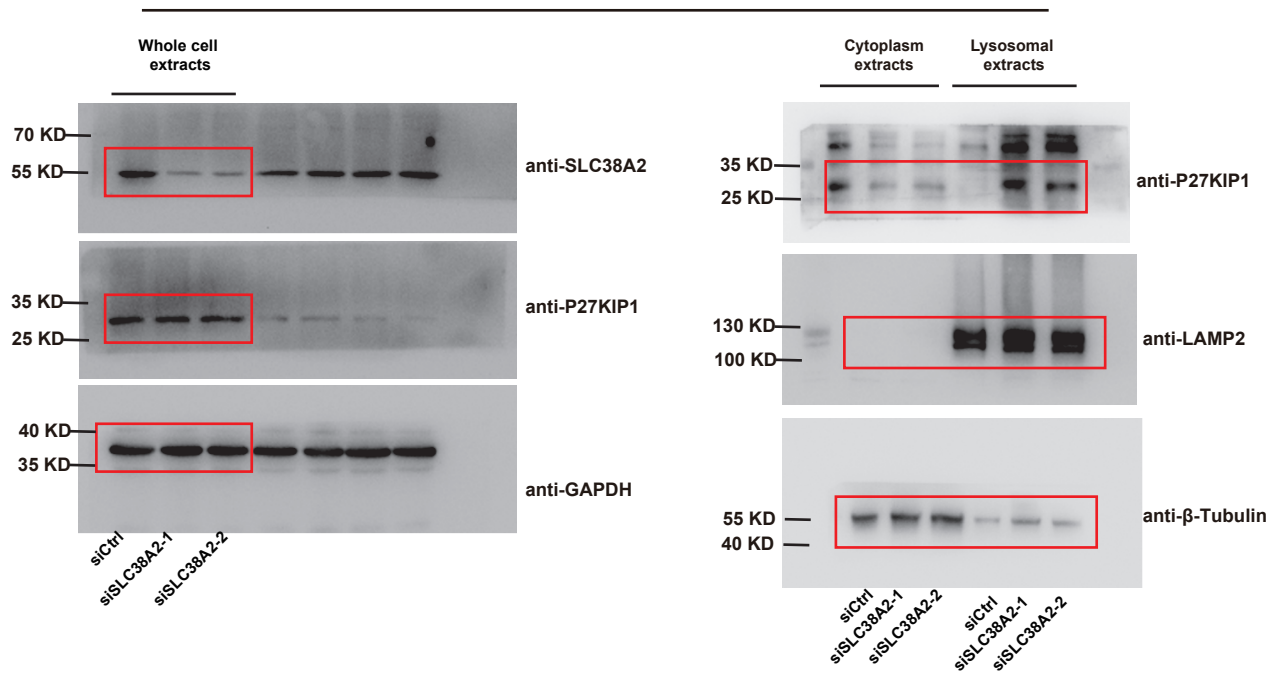

Supplement: Supplementary file 10 — Source data Fig. 5 [file 44318_2024_359_MOESM10_ESM.zip › Figure 5/Fig 5K/Fig 5K.pdf]

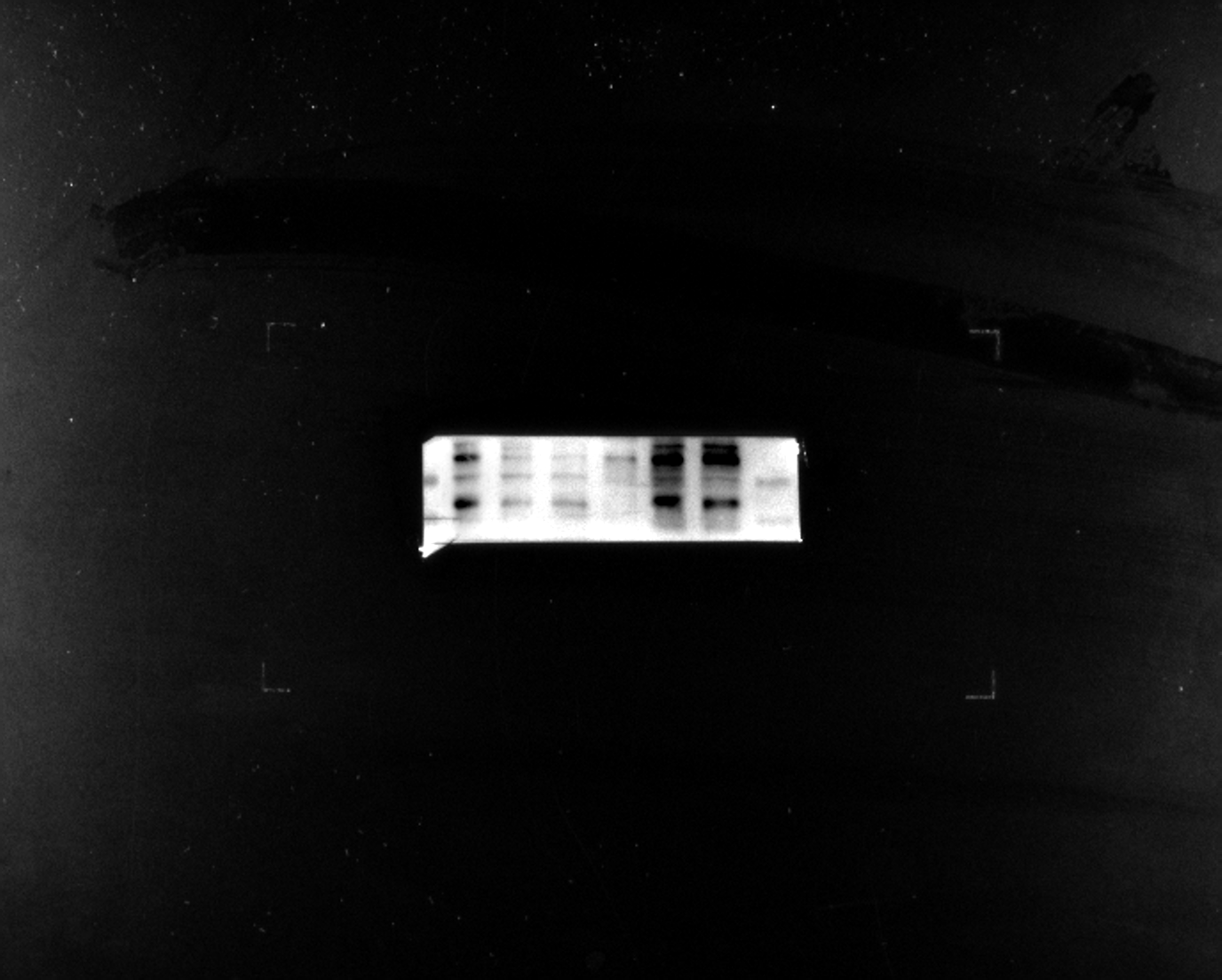

Supplement: Supplementary file 10 — Source data Fig. 5 [file 44318_2024_359_MOESM10_ESM.zip › Figure 5/Fig 5K/cytoplasm lysosome extracts/1-p27-merge.Tif]

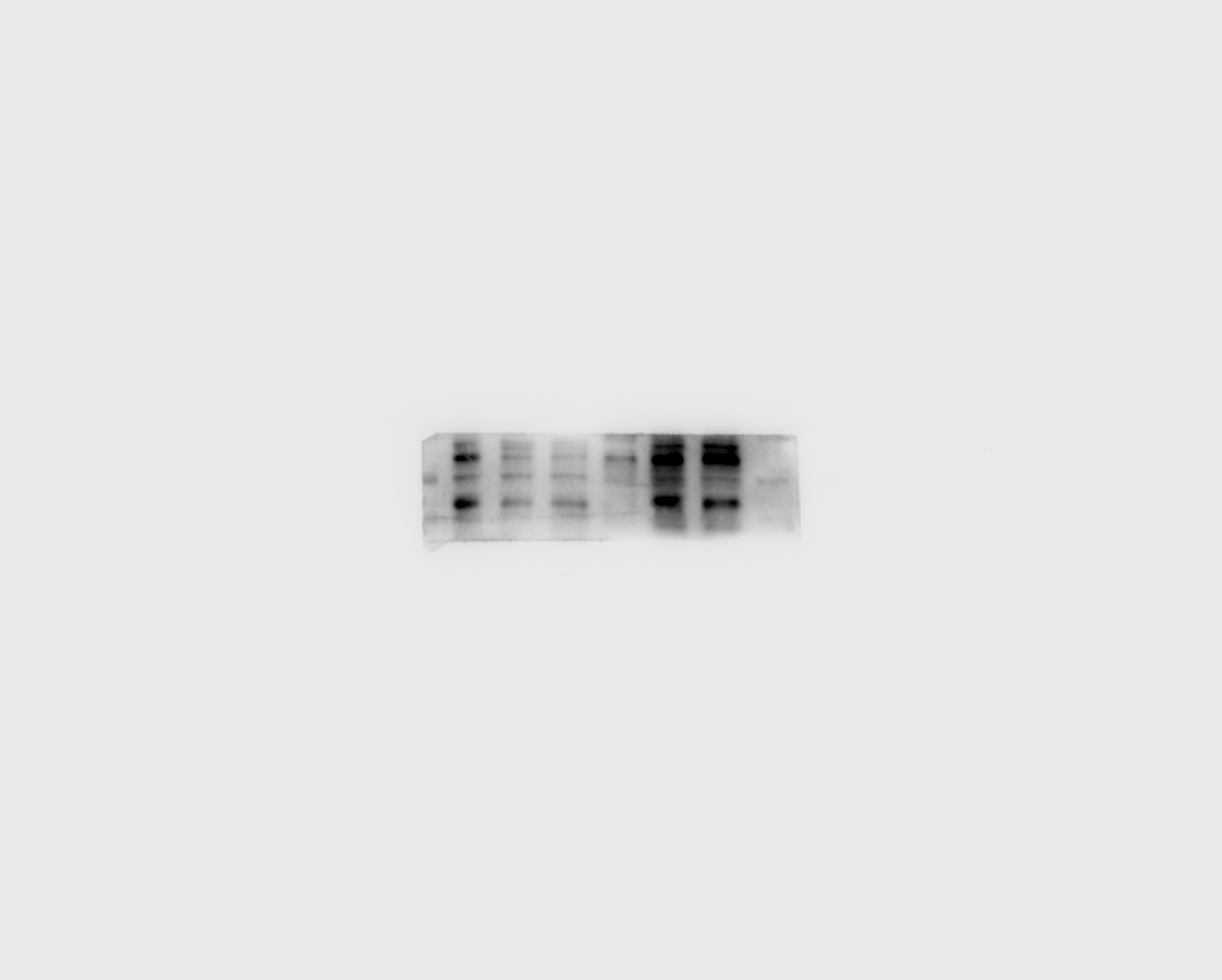

Supplement: Supplementary file 10 — Source data Fig. 5 [file 44318_2024_359_MOESM10_ESM.zip › Figure 5/Fig 5K/cytoplasm lysosome extracts/1-p27.Tif]

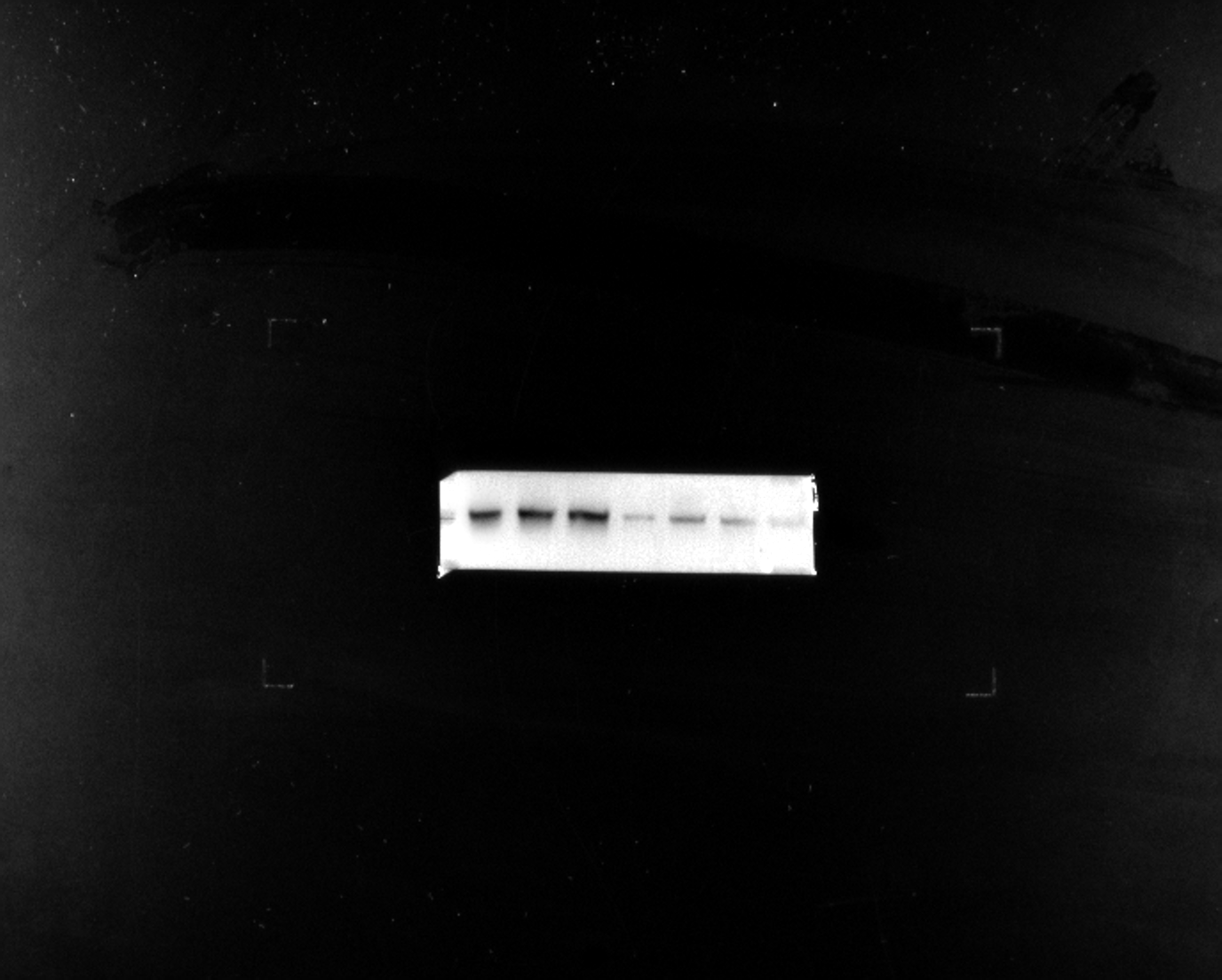

Supplement: Supplementary file 10 — Source data Fig. 5 [file 44318_2024_359_MOESM10_ESM.zip › Figure 5/Fig 5K/cytoplasm lysosome extracts/2-TUBULIN-merge.Tif]

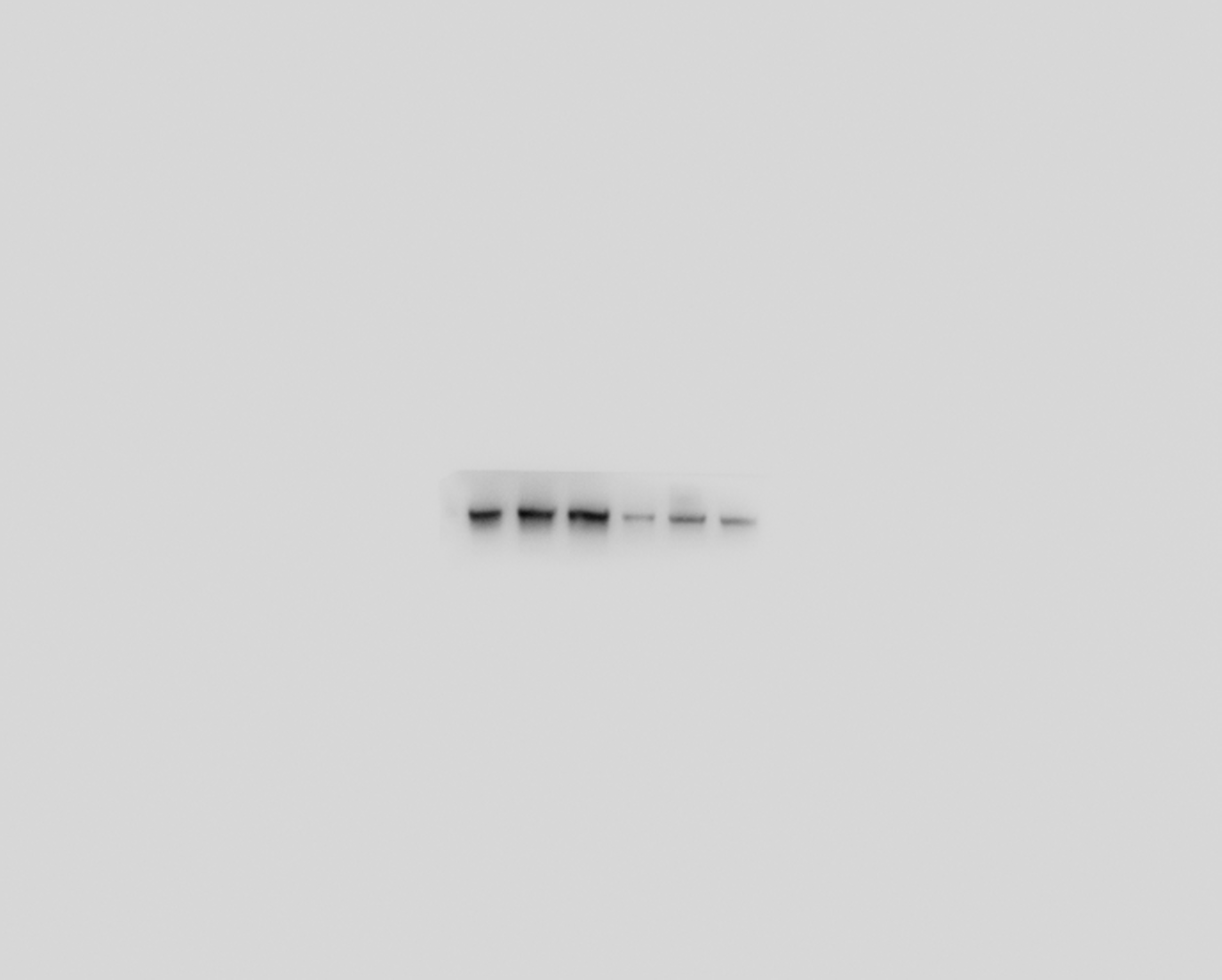

Supplement: Supplementary file 10 — Source data Fig. 5 [file 44318_2024_359_MOESM10_ESM.zip › Figure 5/Fig 5K/cytoplasm lysosome extracts/2-TUBULIN.Tif]

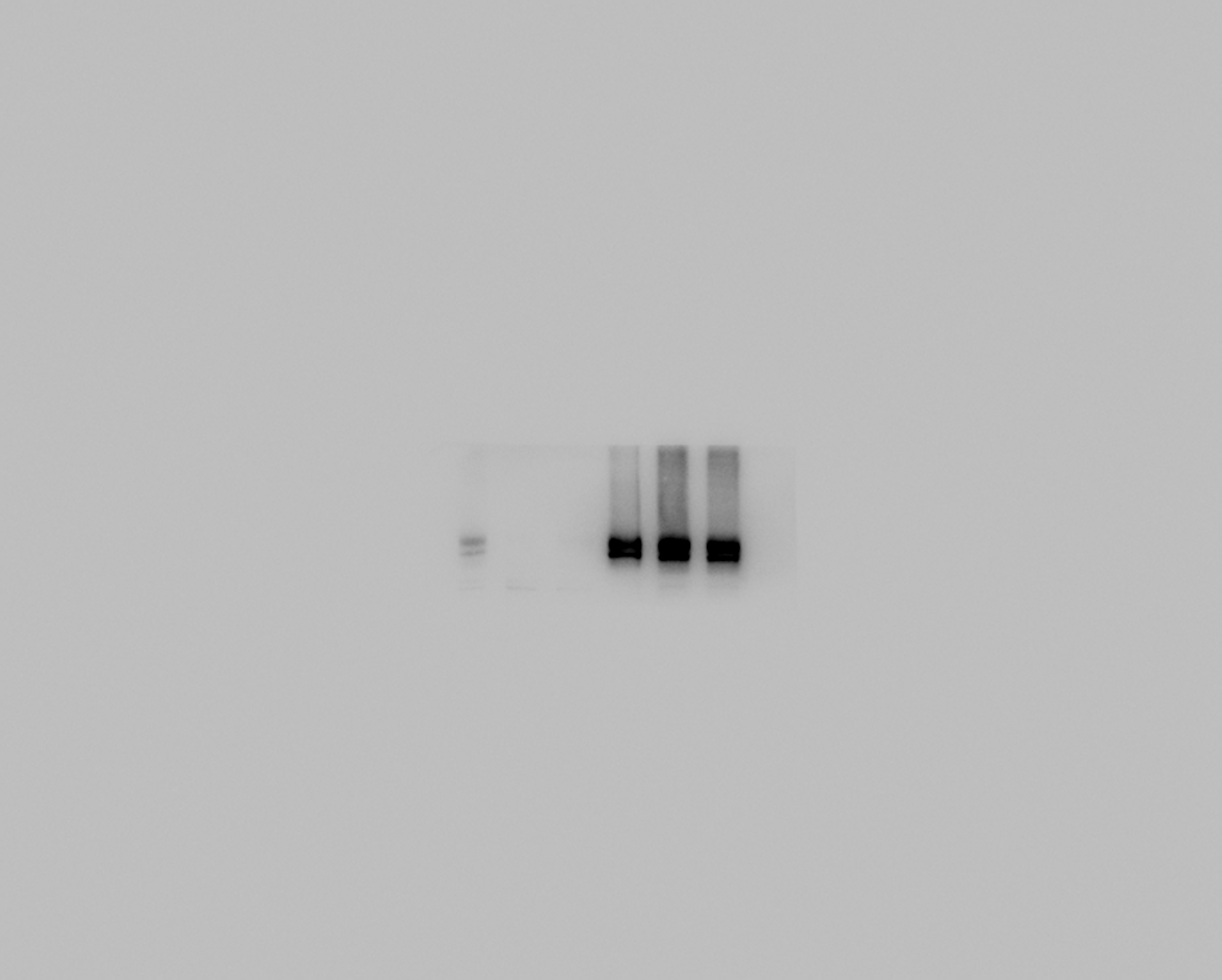

Supplement: Supplementary file 10 — Source data Fig. 5 [file 44318_2024_359_MOESM10_ESM.zip › Figure 5/Fig 5K/cytoplasm lysosome extracts/3-LAMP2.Tif]

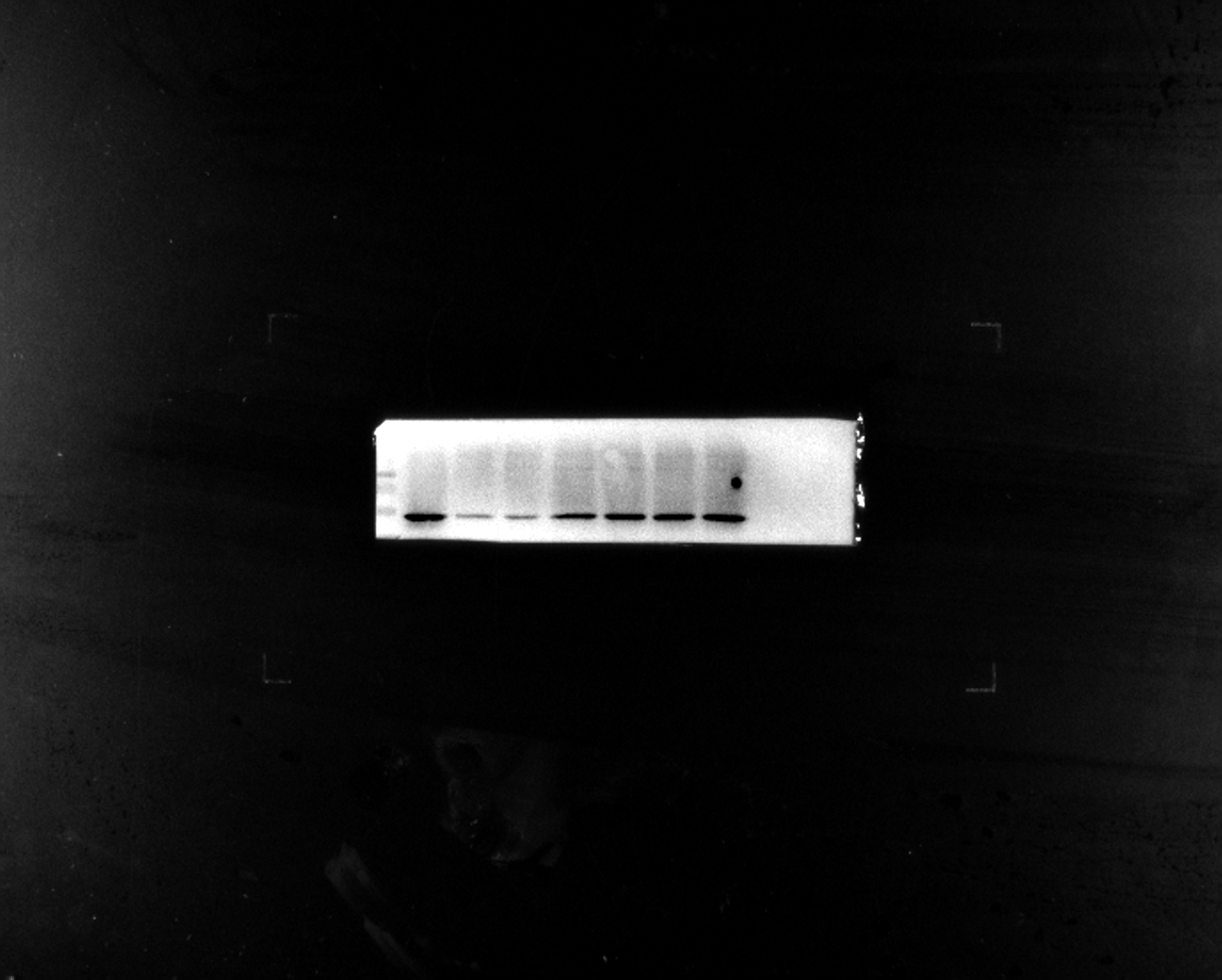

Supplement: Supplementary file 10 — Source data Fig. 5 [file 44318_2024_359_MOESM10_ESM.zip › Figure 5/Fig 5K/whole cell extracts/1-SLC38A2-merge.Tif]

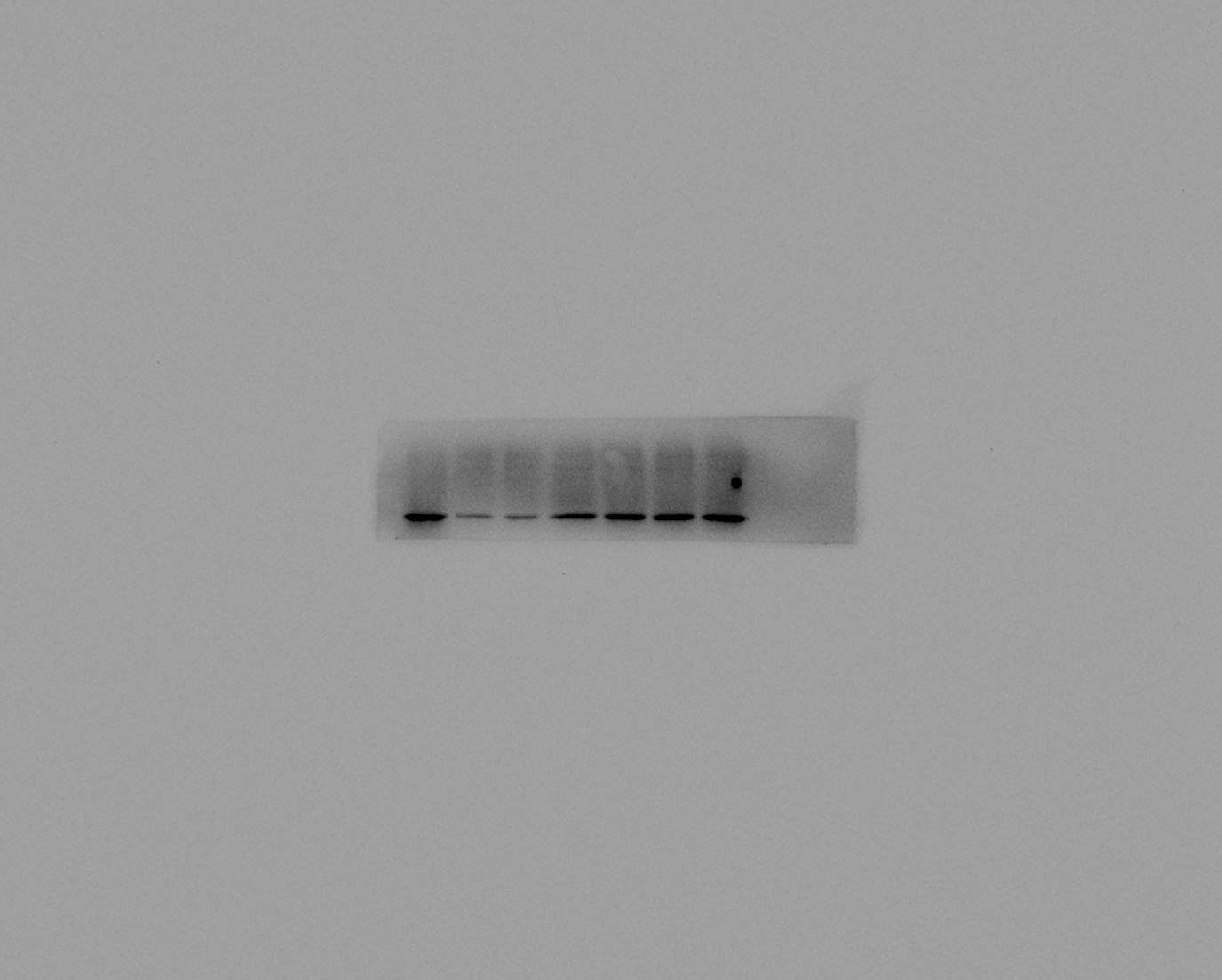

Supplement: Supplementary file 10 — Source data Fig. 5 [file 44318_2024_359_MOESM10_ESM.zip › Figure 5/Fig 5K/whole cell extracts/1-SLC38A2.Tif]

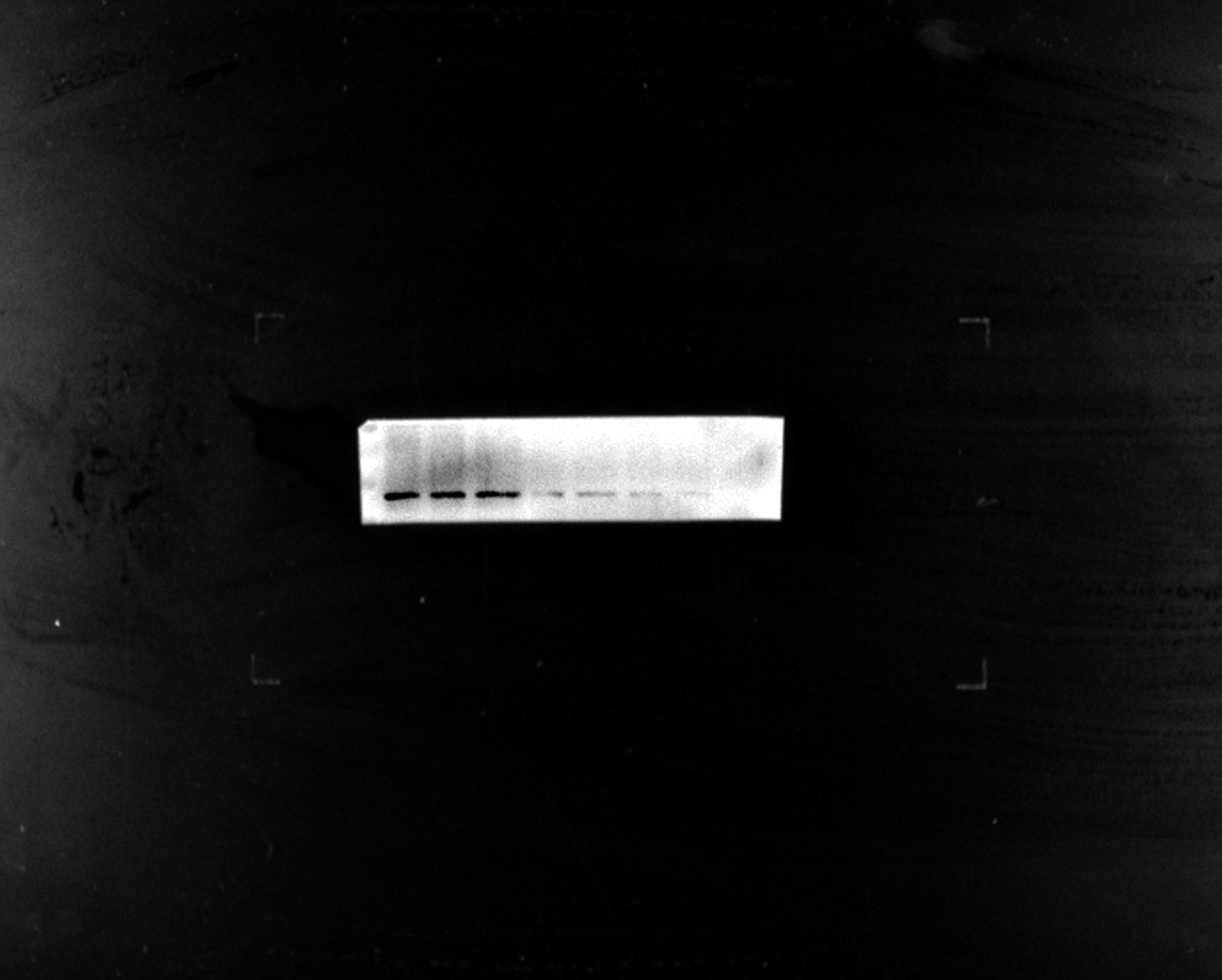

Supplement: Supplementary file 10 — Source data Fig. 5 [file 44318_2024_359_MOESM10_ESM.zip › Figure 5/Fig 5K/whole cell extracts/2-p27-merge.Tif]

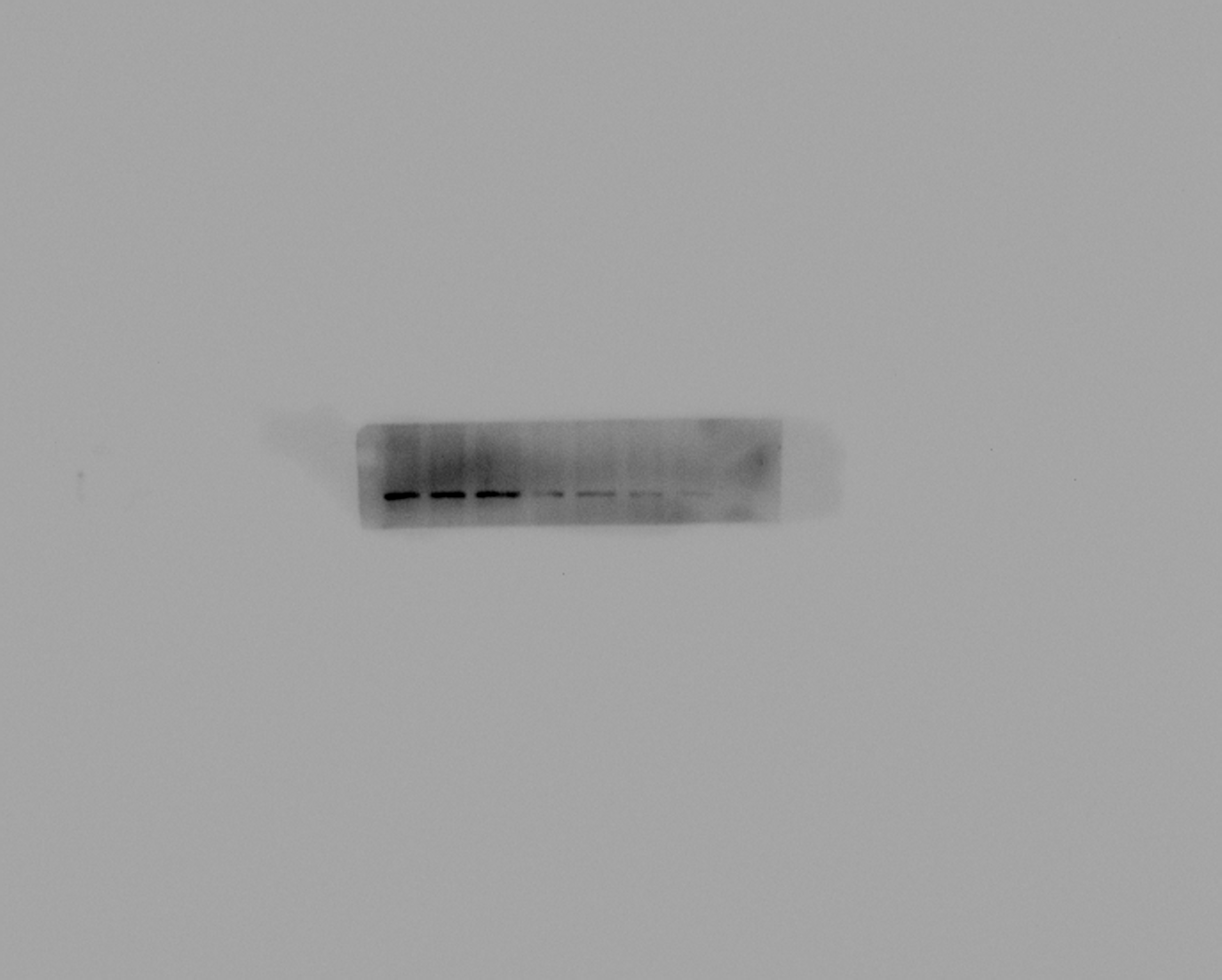

Supplement: Supplementary file 10 — Source data Fig. 5 [file 44318_2024_359_MOESM10_ESM.zip › Figure 5/Fig 5K/whole cell extracts/2-p27.Tif]
