# Supplementary figures and images for: Micropeptide hSPAR regulates glutamine levels and suppresses mammary tumor growth via a TRIM21-P27KIP1-mTOR axis (part 5 of 7)
Source: EMBO J. 2025 Jan 28;44(5):1414–41. doi: 10.1038/s44318-024-00359-z (PMC11876615; doi:10.1038/s44318-024-00359-z)

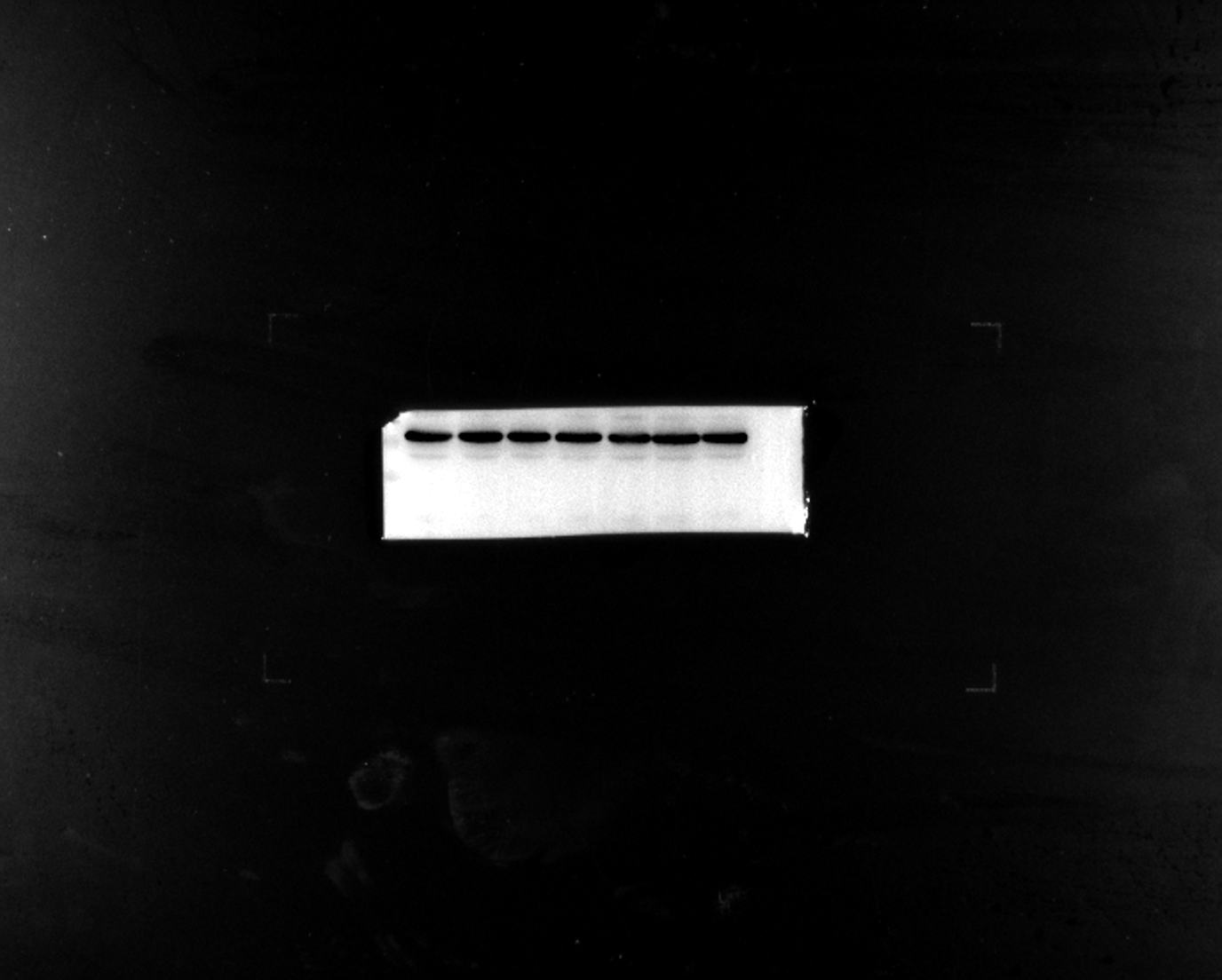

Supplement: Supplementary file 10 — Source data Fig. 5 [file 44318_2024_359_MOESM10_ESM.zip › Figure 5/Fig 5K/whole cell extracts/3-GAPDH-merge.Tif]

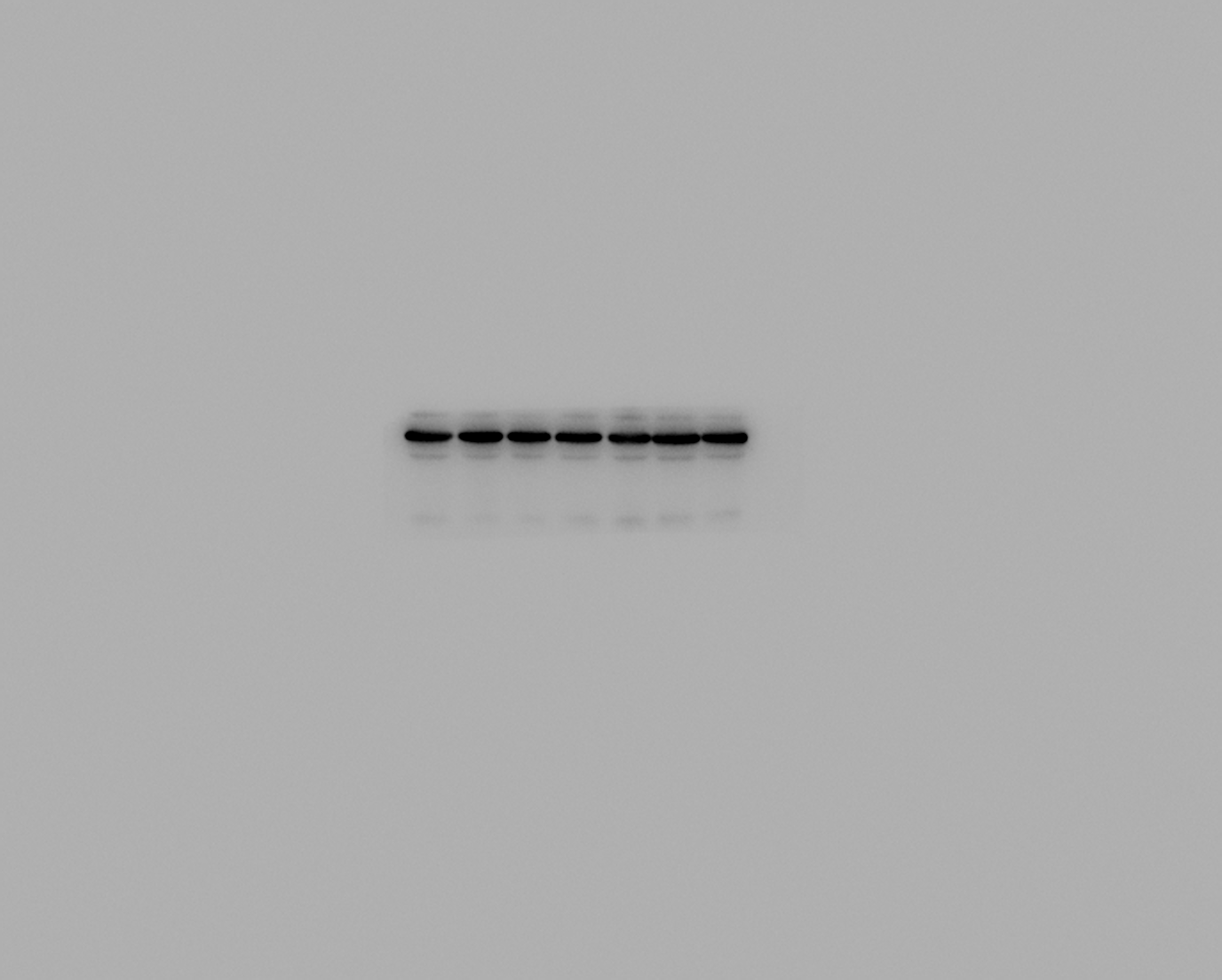

Supplement: Supplementary file 10 — Source data Fig. 5 [file 44318_2024_359_MOESM10_ESM.zip › Figure 5/Fig 5K/whole cell extracts/3-GAPDH.Tif]

Fig 6A

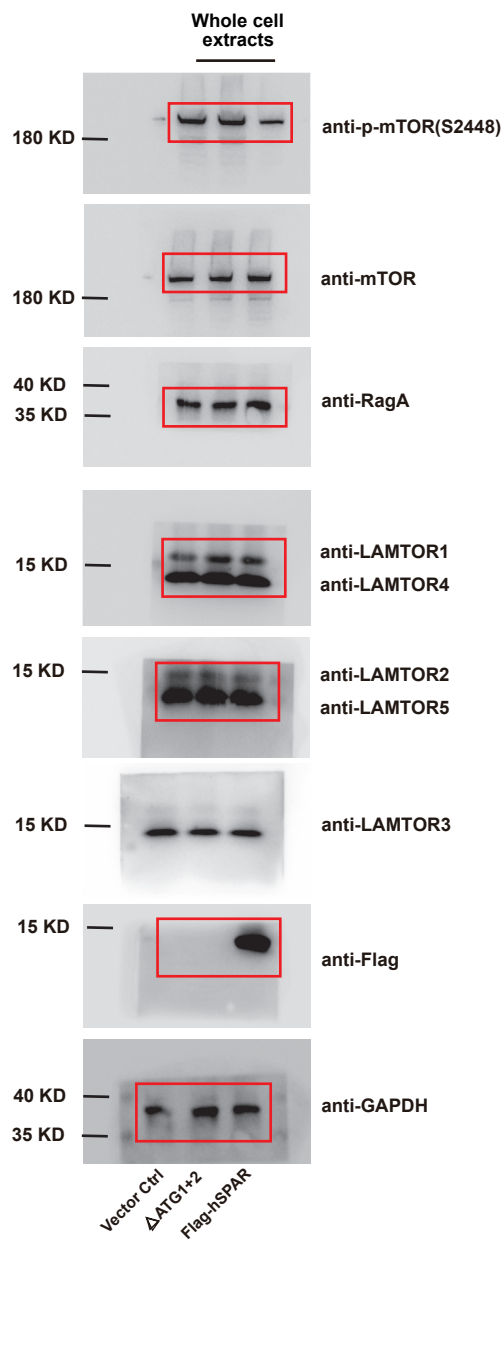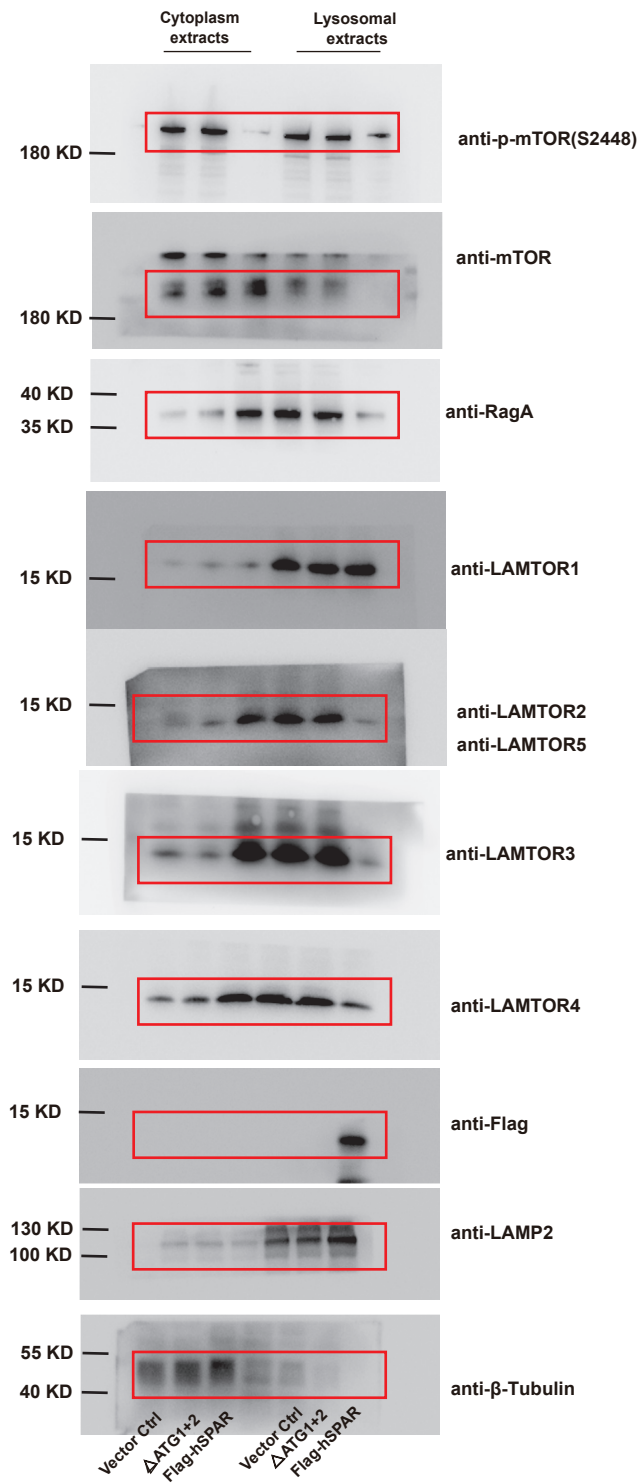

Supplement: Supplementary file 11 — Source data Fig. 6 [file 44318_2024_359_MOESM11_ESM.zip › Figure 6/Fig 6A and 6B/Fig 6A/Fig 6A.pdf]

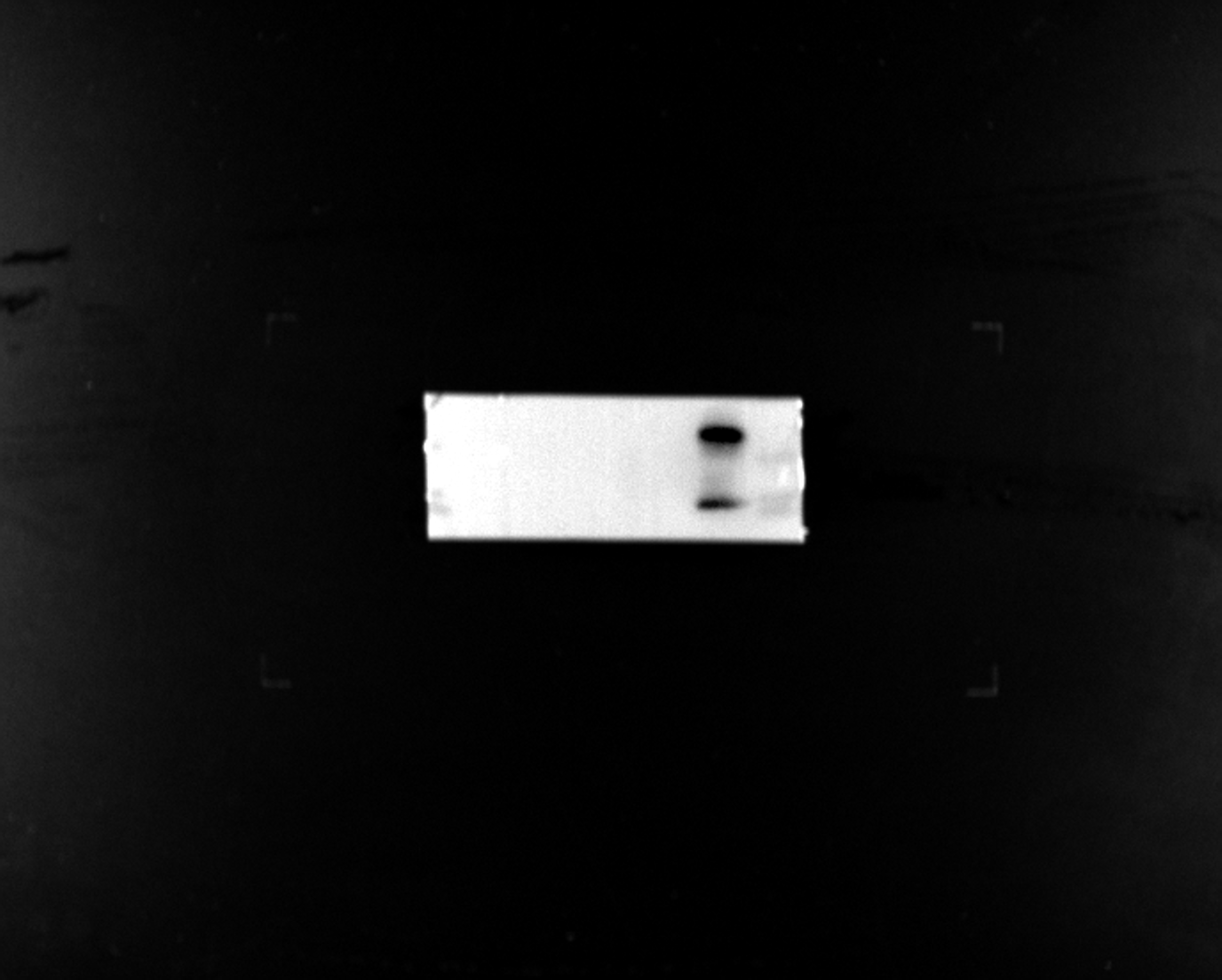

Supplement: Supplementary file 11 — Source data Fig. 6 [file 44318_2024_359_MOESM11_ESM.zip › Figure 6/Fig 6A and 6B/Fig 6A/cyto-lyso/Flag-merge.Tif]

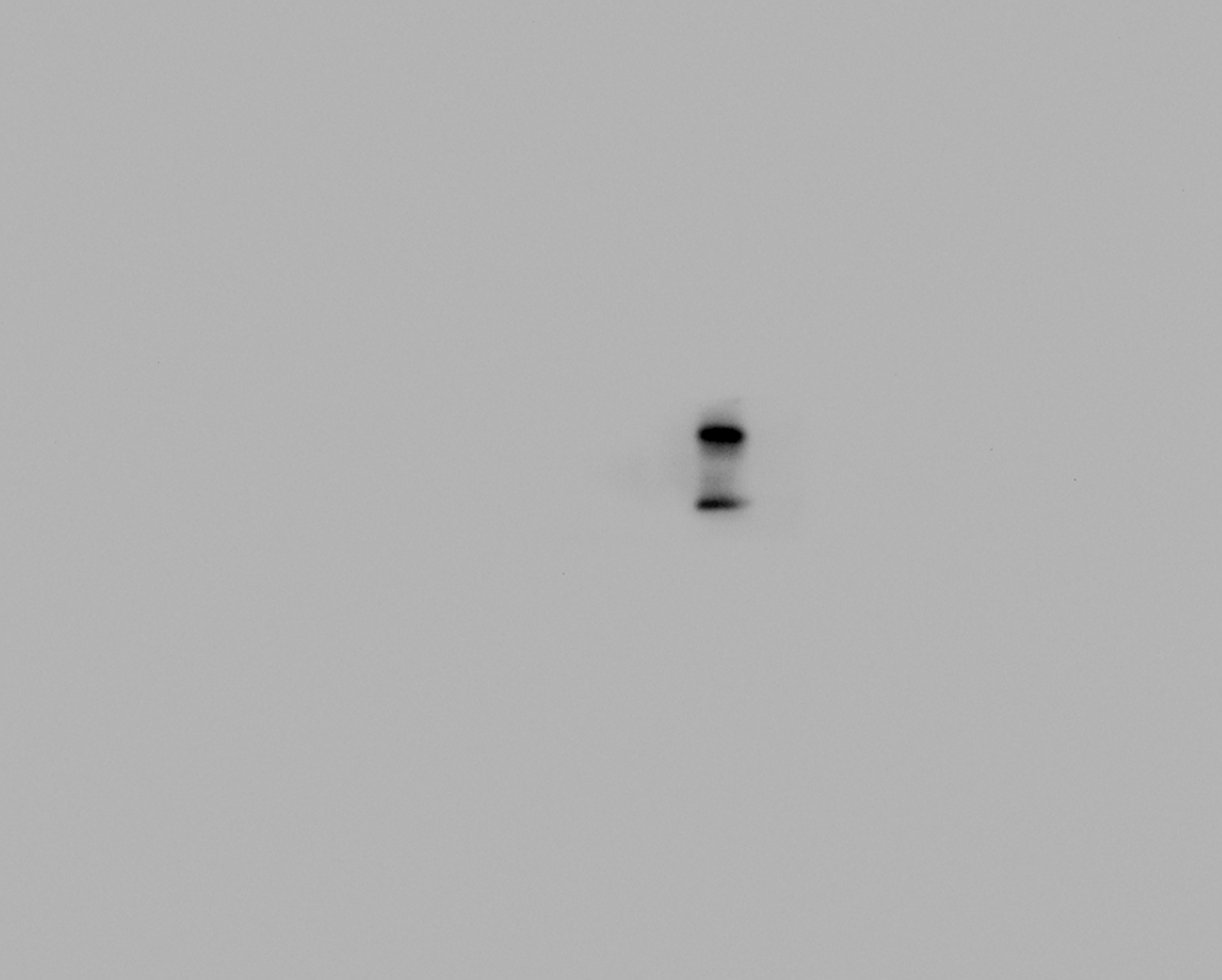

Supplement: Supplementary file 11 — Source data Fig. 6 [file 44318_2024_359_MOESM11_ESM.zip › Figure 6/Fig 6A and 6B/Fig 6A/cyto-lyso/Flag.Tif]

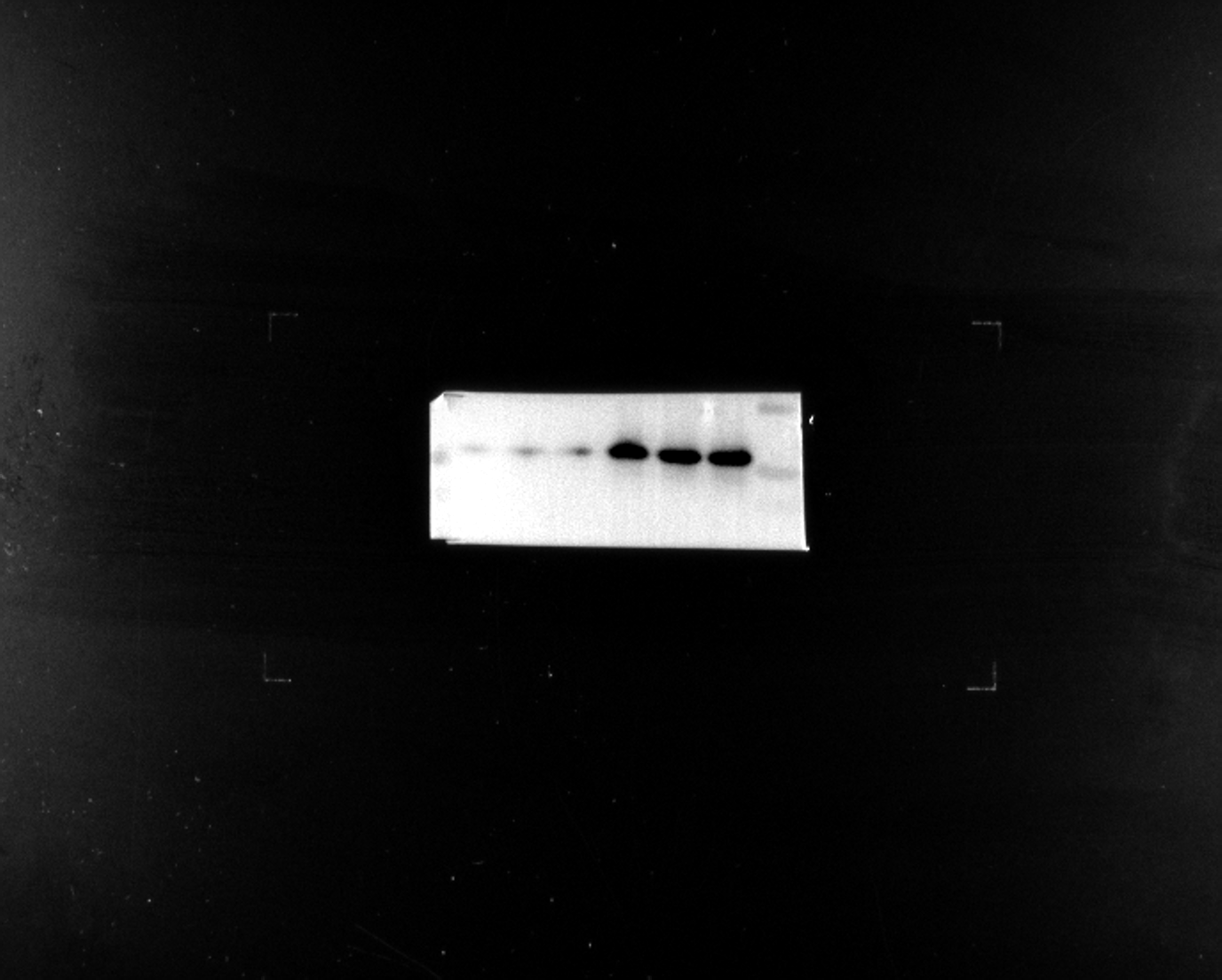

Supplement: Supplementary file 11 — Source data Fig. 6 [file 44318_2024_359_MOESM11_ESM.zip › Figure 6/Fig 6A and 6B/Fig 6A/cyto-lyso/L1-merge.Tif]

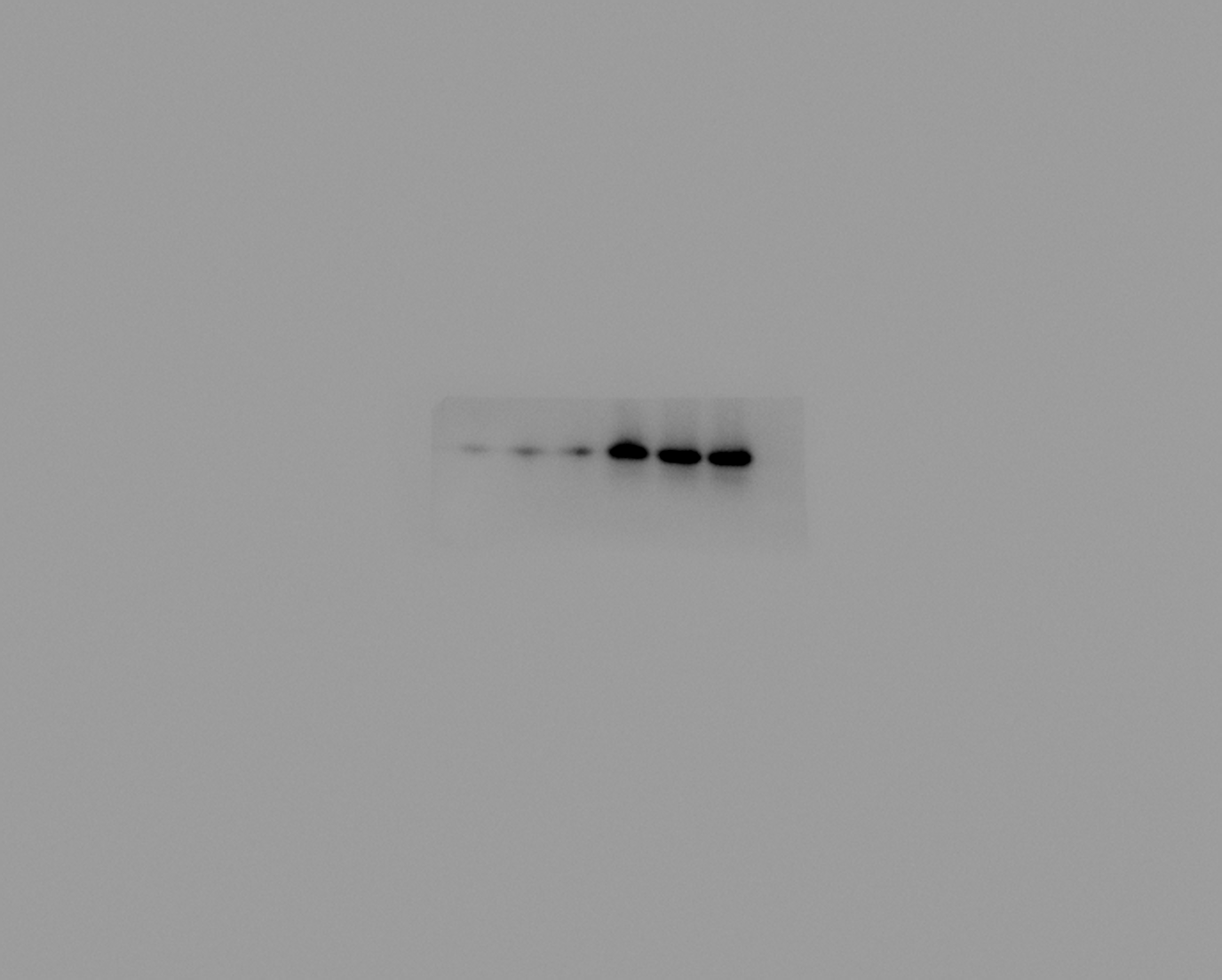

Supplement: Supplementary file 11 — Source data Fig. 6 [file 44318_2024_359_MOESM11_ESM.zip › Figure 6/Fig 6A and 6B/Fig 6A/cyto-lyso/L1.Tif]

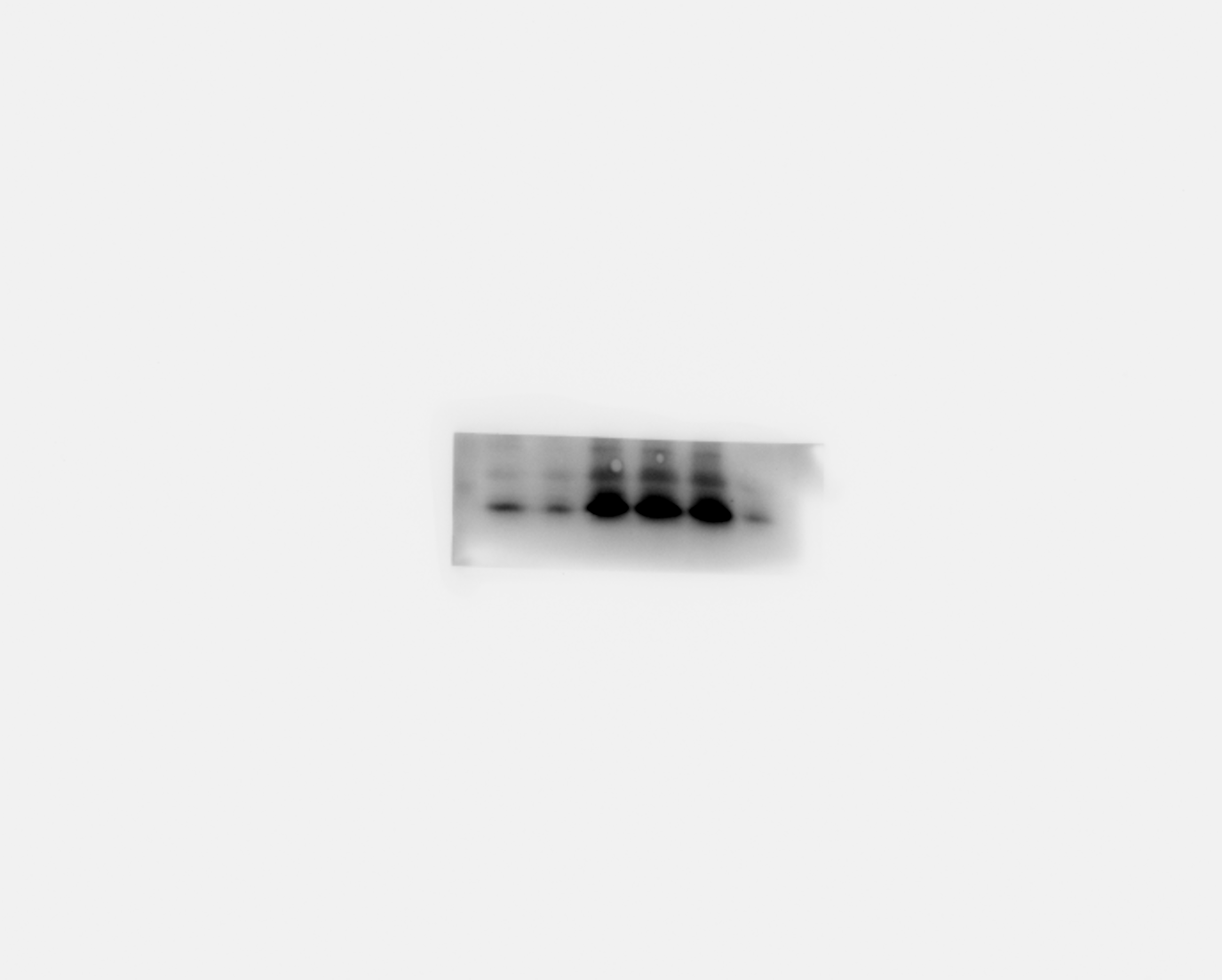

Supplement: Supplementary file 11 — Source data Fig. 6 [file 44318_2024_359_MOESM11_ESM.zip › Figure 6/Fig 6A and 6B/Fig 6A/cyto-lyso/L2+L5 (2).Tif]

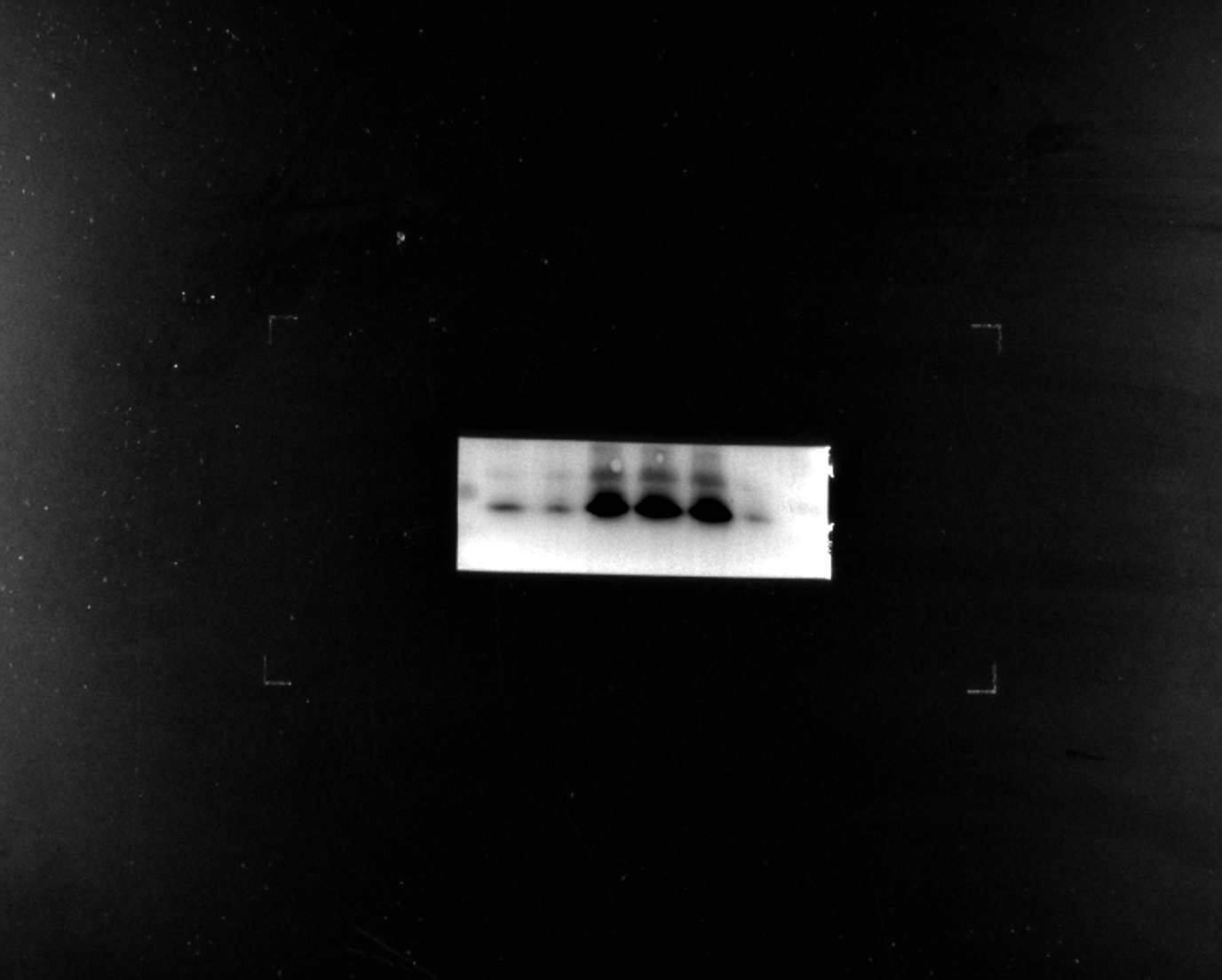

Supplement: Supplementary file 11 — Source data Fig. 6 [file 44318_2024_359_MOESM11_ESM.zip › Figure 6/Fig 6A and 6B/Fig 6A/cyto-lyso/L2+L5-merge (2).Tif]

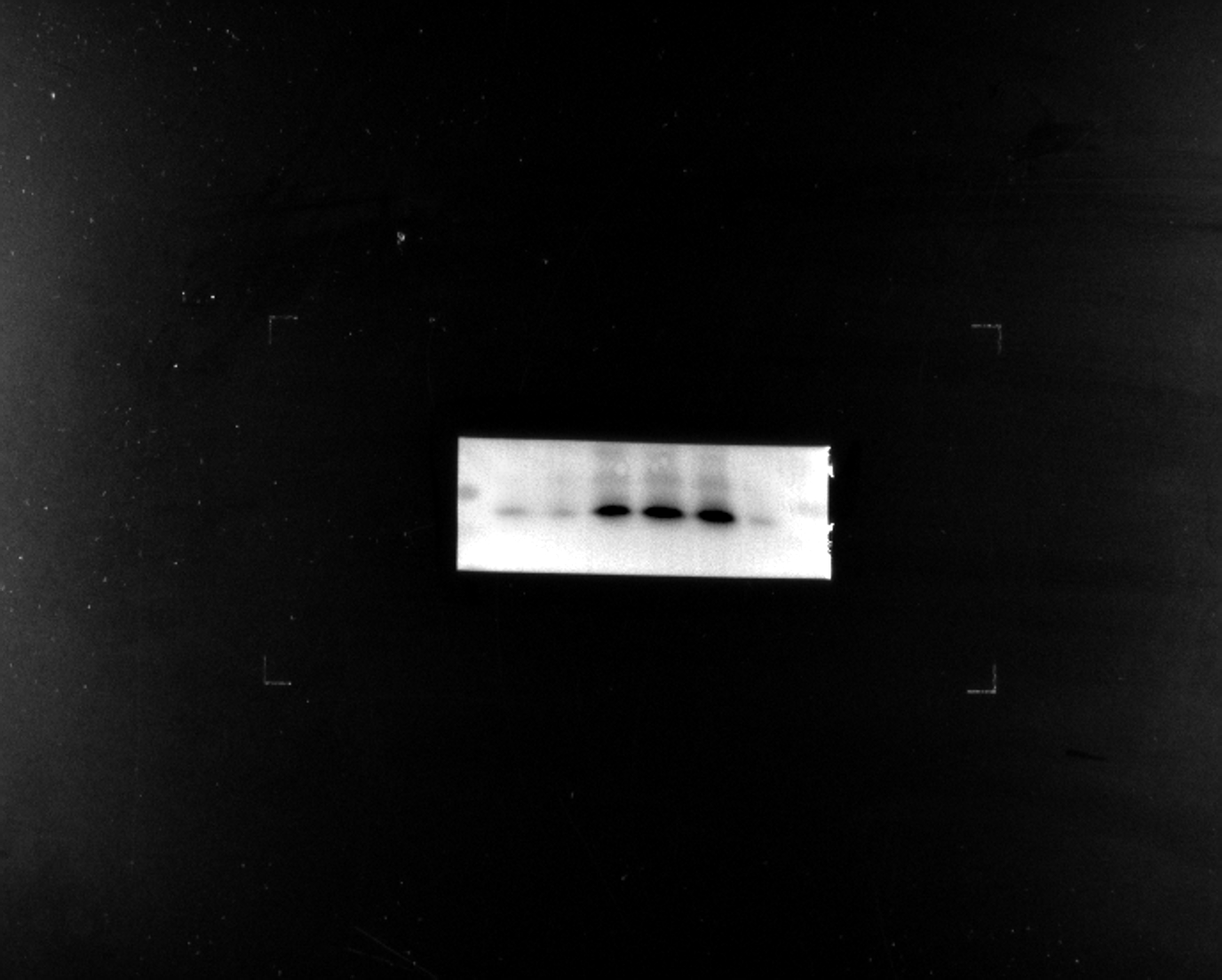

Supplement: Supplementary file 11 — Source data Fig. 6 [file 44318_2024_359_MOESM11_ESM.zip › Figure 6/Fig 6A and 6B/Fig 6A/cyto-lyso/L2+L5-merge.Tif]

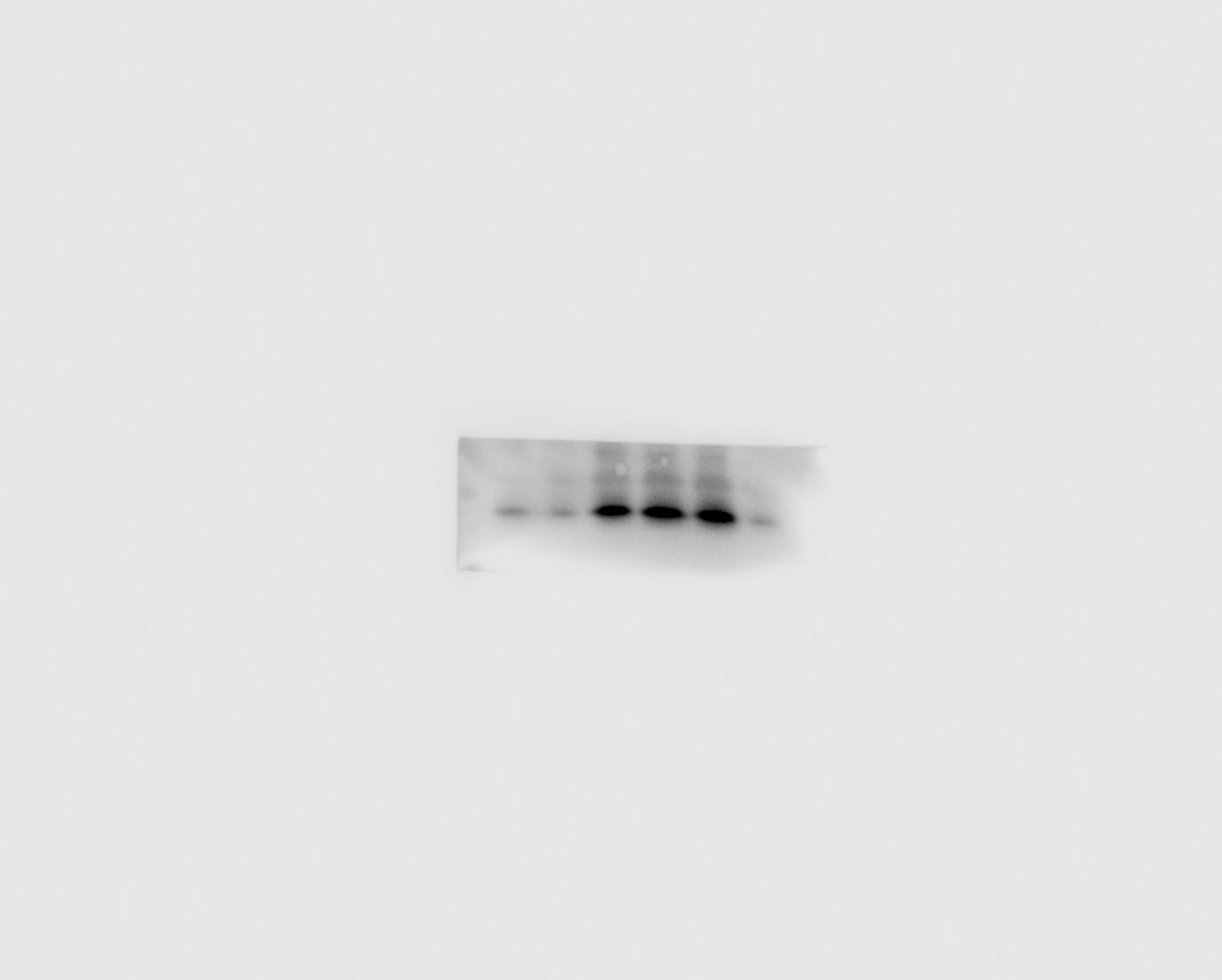

Supplement: Supplementary file 11 — Source data Fig. 6 [file 44318_2024_359_MOESM11_ESM.zip › Figure 6/Fig 6A and 6B/Fig 6A/cyto-lyso/L2+L5.Tif]

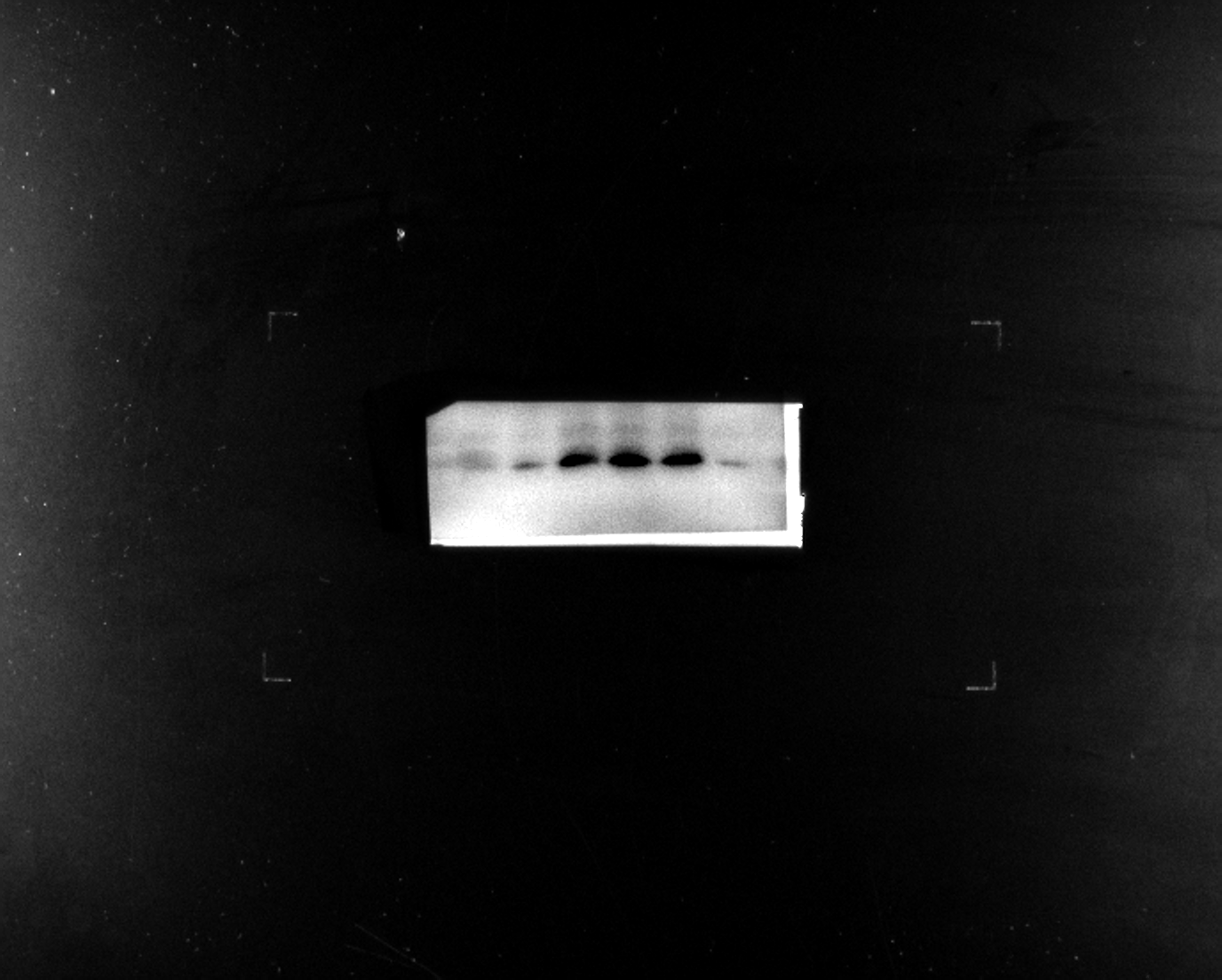

Supplement: Supplementary file 11 — Source data Fig. 6 [file 44318_2024_359_MOESM11_ESM.zip › Figure 6/Fig 6A and 6B/Fig 6A/cyto-lyso/L3-merge.Tif]

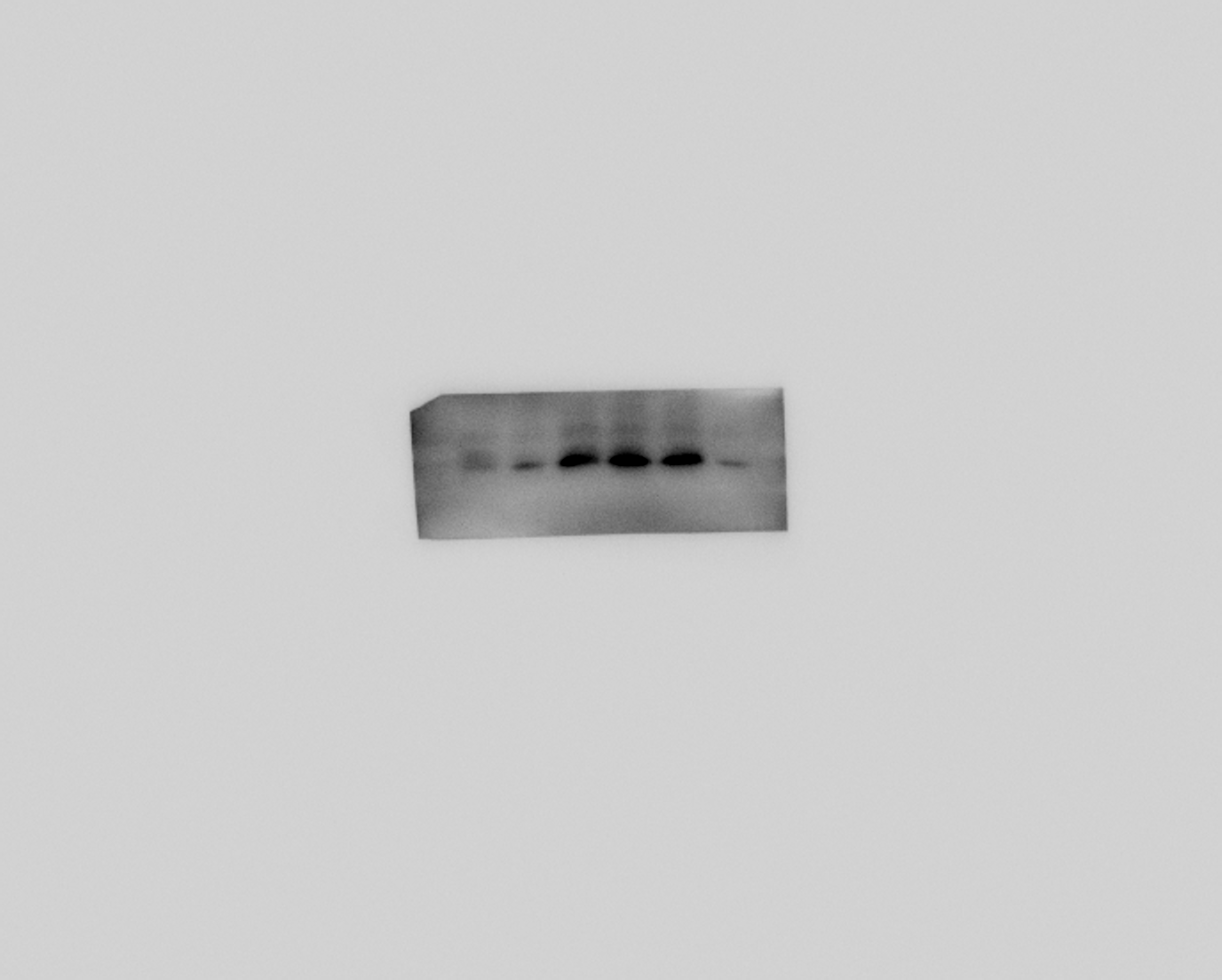

Supplement: Supplementary file 11 — Source data Fig. 6 [file 44318_2024_359_MOESM11_ESM.zip › Figure 6/Fig 6A and 6B/Fig 6A/cyto-lyso/L3.Tif]

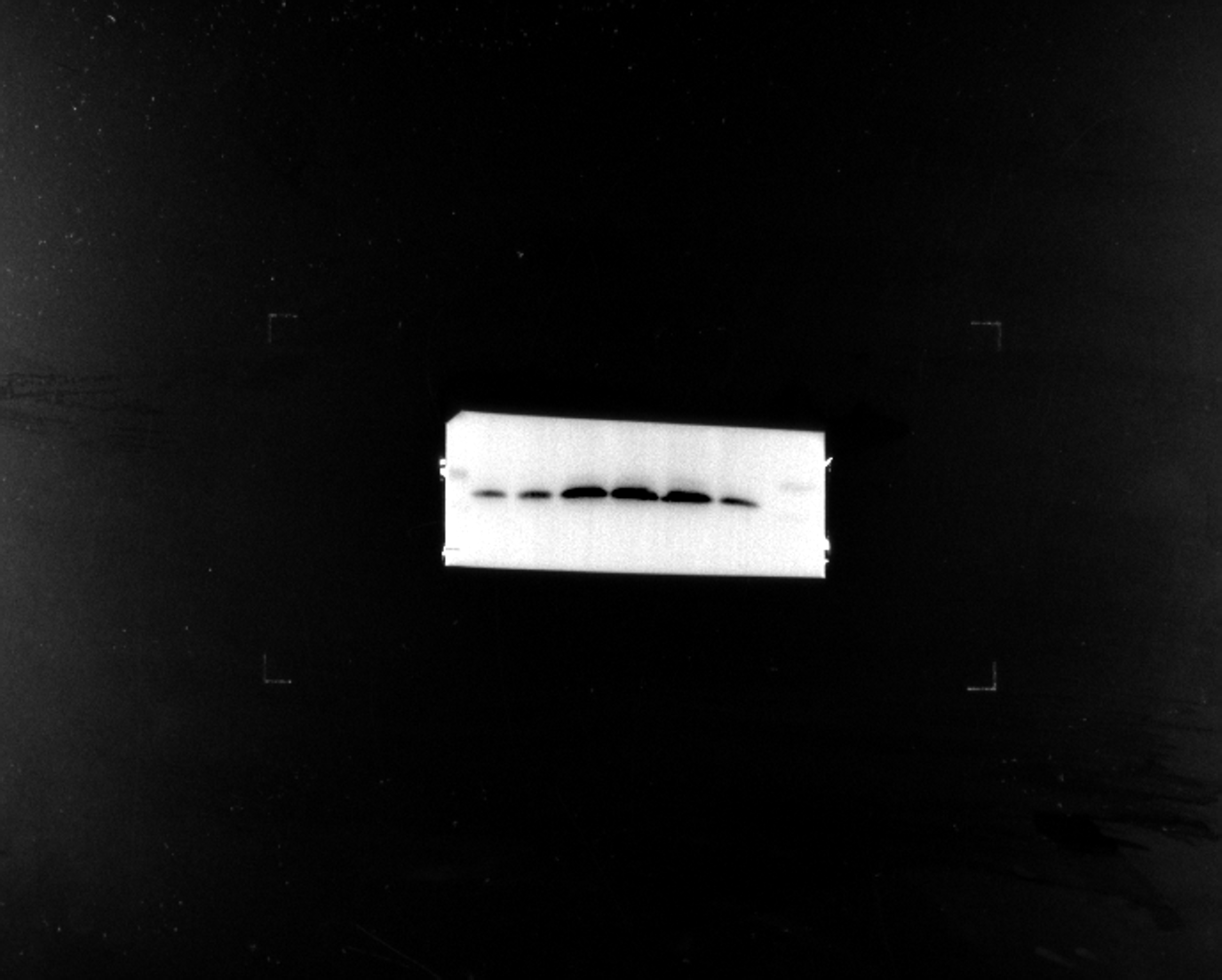

Supplement: Supplementary file 11 — Source data Fig. 6 [file 44318_2024_359_MOESM11_ESM.zip › Figure 6/Fig 6A and 6B/Fig 6A/cyto-lyso/L4-merge.Tif]

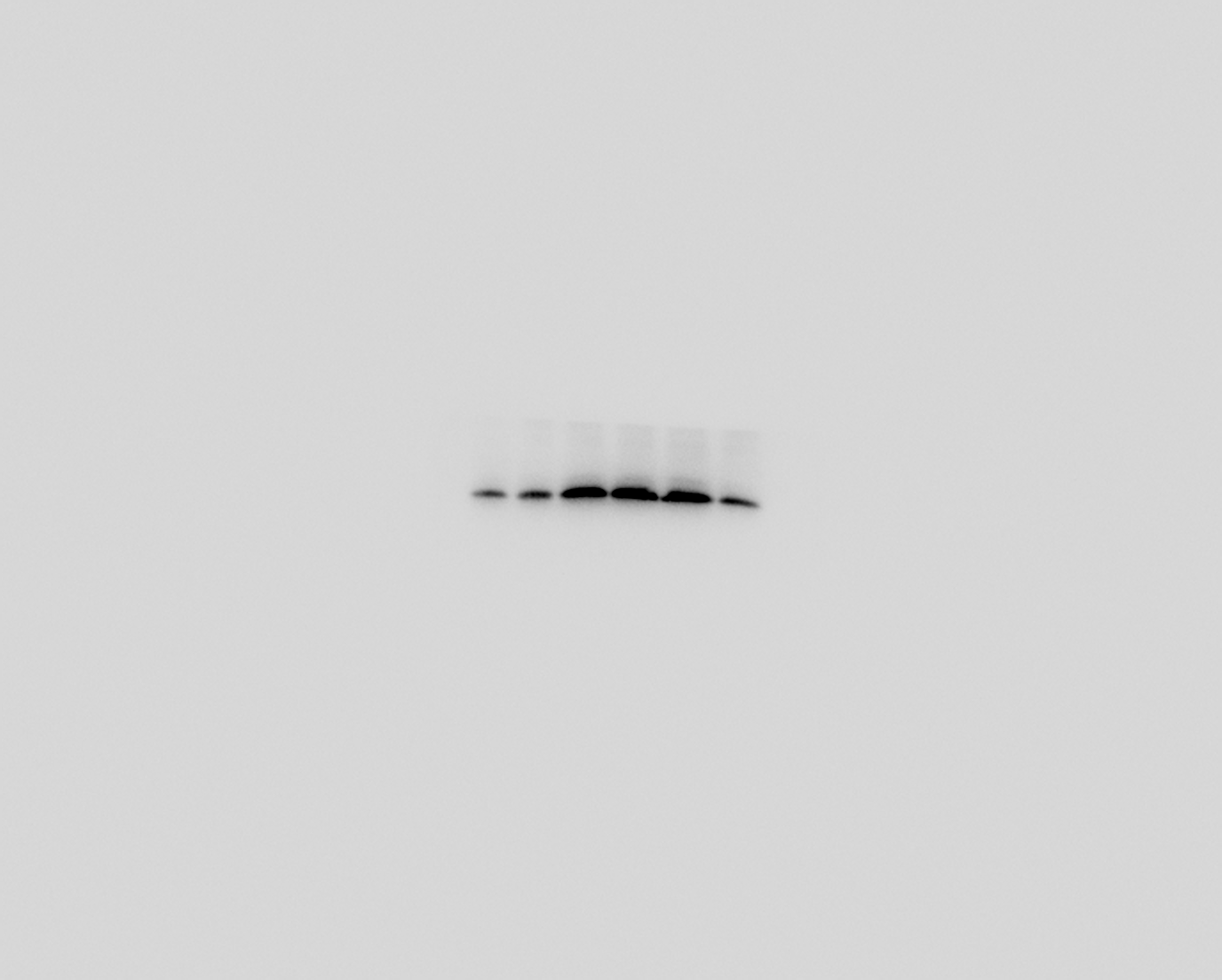

Supplement: Supplementary file 11 — Source data Fig. 6 [file 44318_2024_359_MOESM11_ESM.zip › Figure 6/Fig 6A and 6B/Fig 6A/cyto-lyso/L4.Tif]

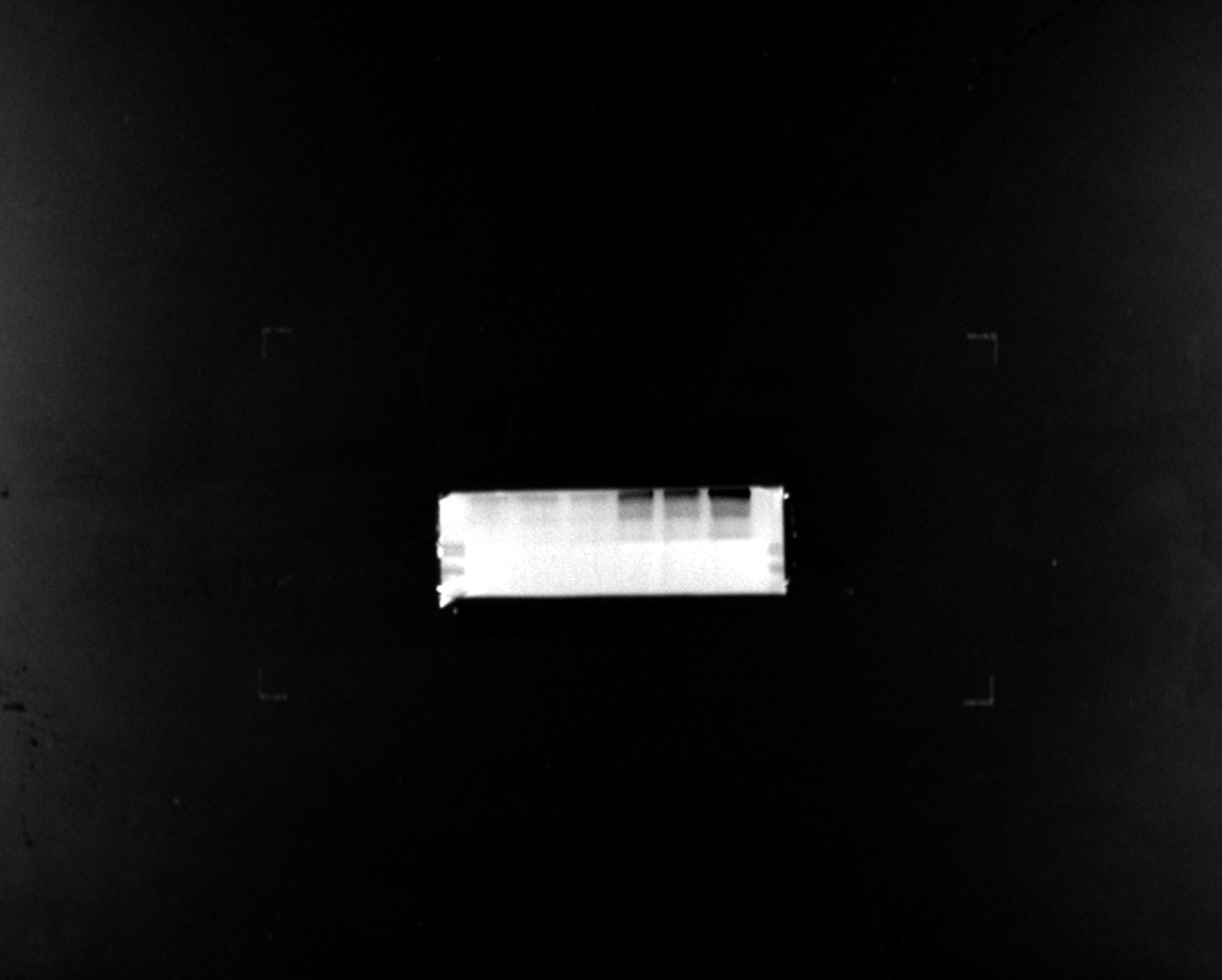

Supplement: Supplementary file 11 — Source data Fig. 6 [file 44318_2024_359_MOESM11_ESM.zip › Figure 6/Fig 6A and 6B/Fig 6A/cyto-lyso/LAMP2-merge.Tif]

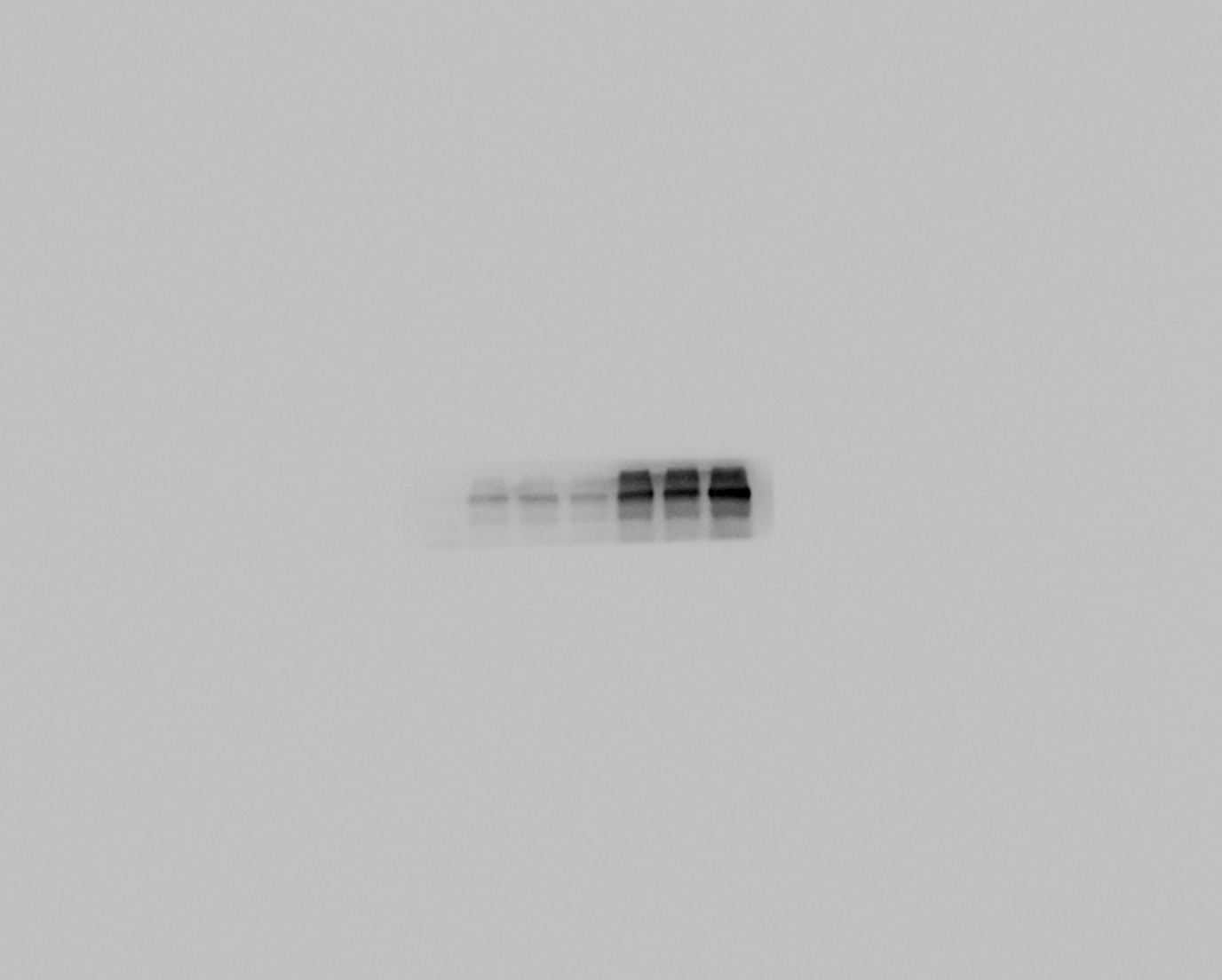

Supplement: Supplementary file 11 — Source data Fig. 6 [file 44318_2024_359_MOESM11_ESM.zip › Figure 6/Fig 6A and 6B/Fig 6A/cyto-lyso/LAMP2.Tif]

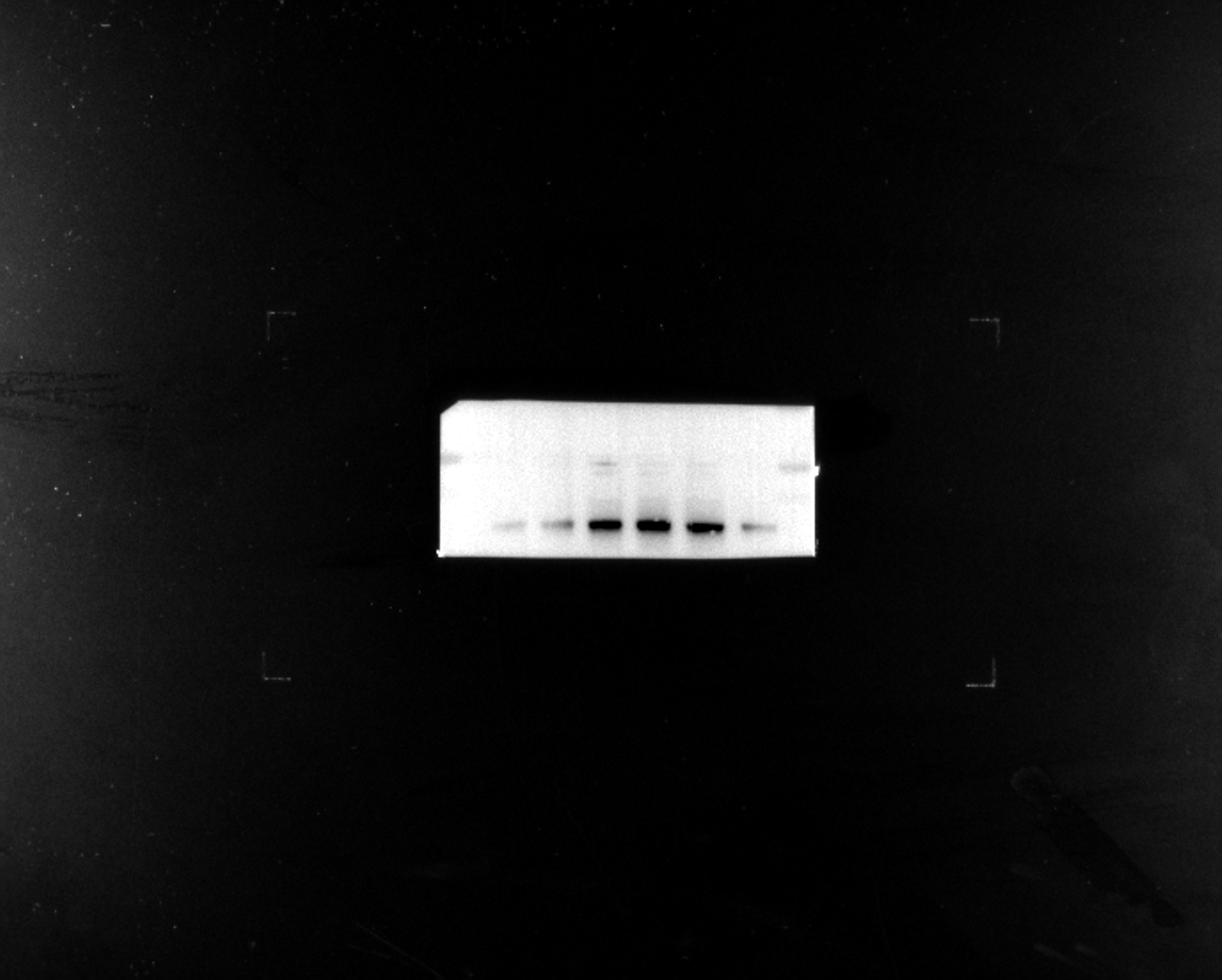

Supplement: Supplementary file 11 — Source data Fig. 6 [file 44318_2024_359_MOESM11_ESM.zip › Figure 6/Fig 6A and 6B/Fig 6A/cyto-lyso/RagA-merge.Tif]

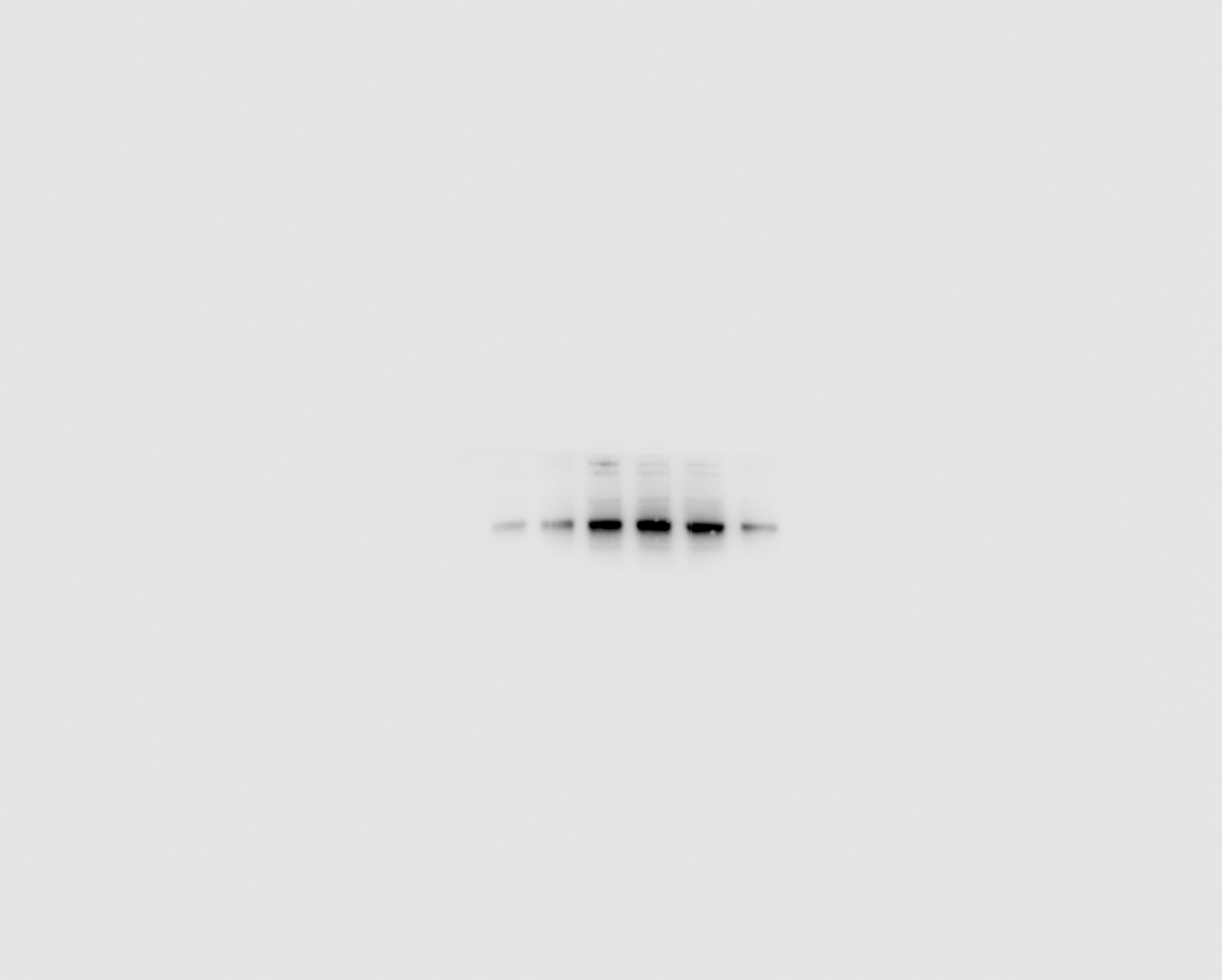

Supplement: Supplementary file 11 — Source data Fig. 6 [file 44318_2024_359_MOESM11_ESM.zip › Figure 6/Fig 6A and 6B/Fig 6A/cyto-lyso/RagA.Tif]

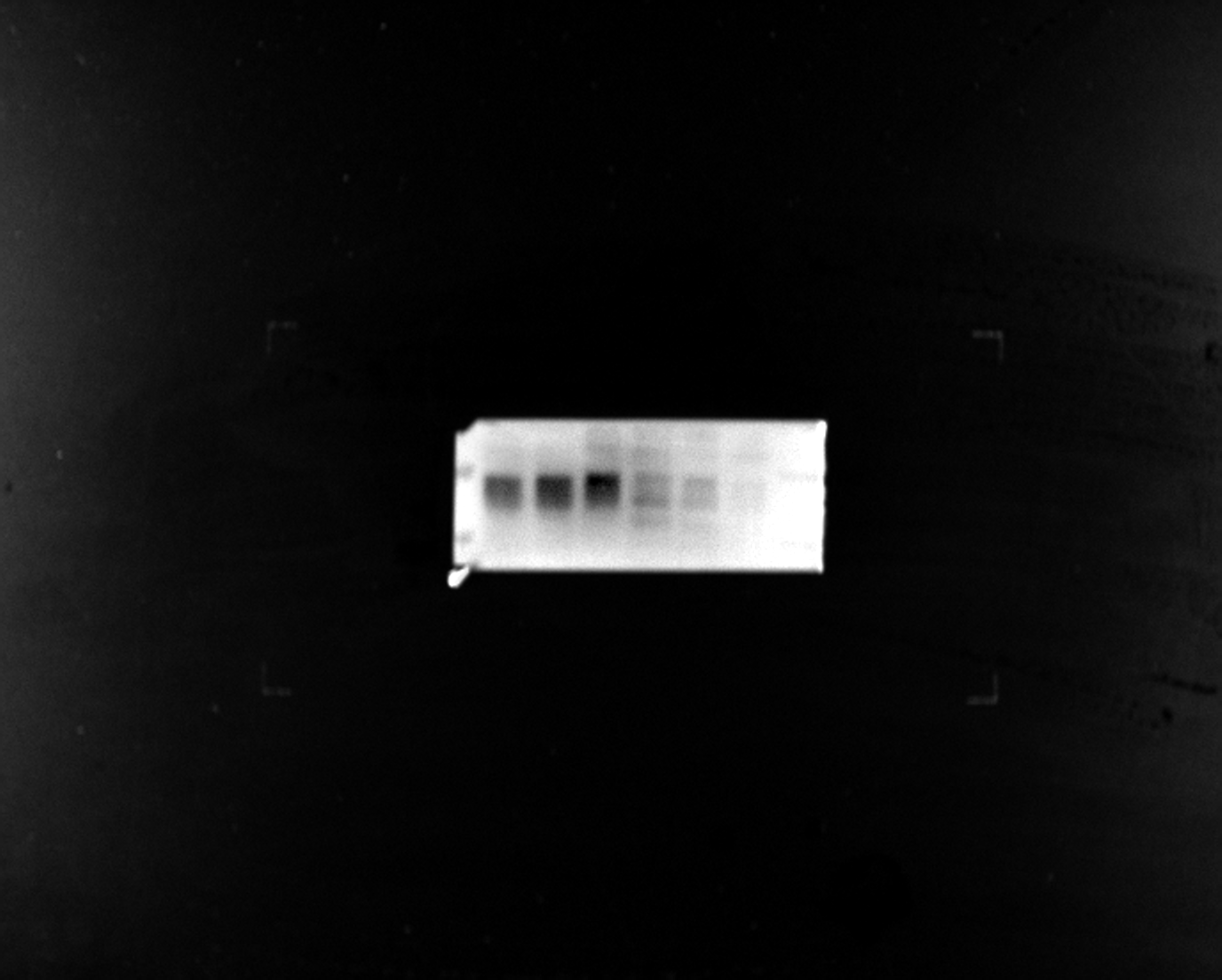

Supplement: Supplementary file 11 — Source data Fig. 6 [file 44318_2024_359_MOESM11_ESM.zip › Figure 6/Fig 6A and 6B/Fig 6A/cyto-lyso/TUBULIN-merge.Tif]

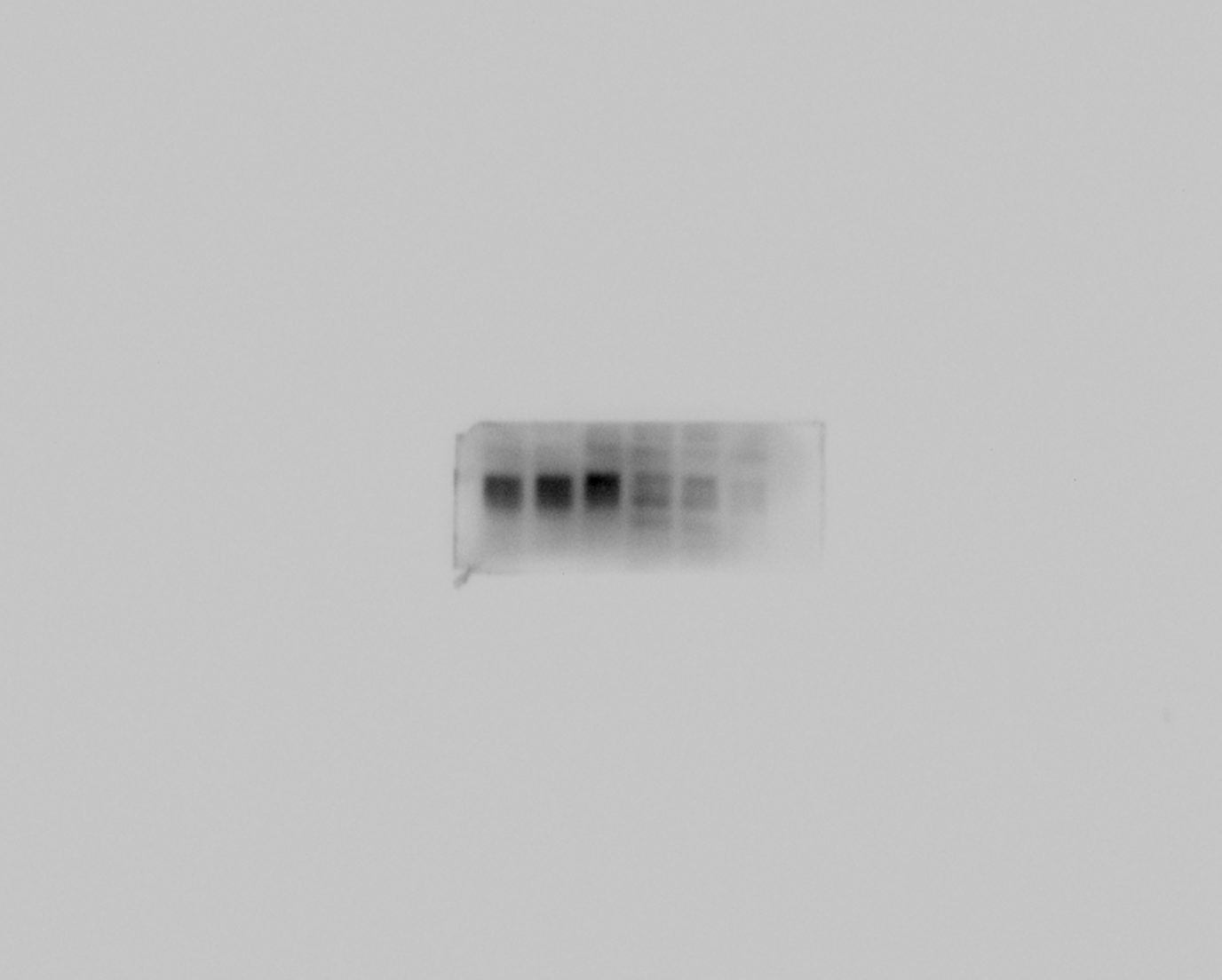

Supplement: Supplementary file 11 — Source data Fig. 6 [file 44318_2024_359_MOESM11_ESM.zip › Figure 6/Fig 6A and 6B/Fig 6A/cyto-lyso/TUBULIN.Tif]

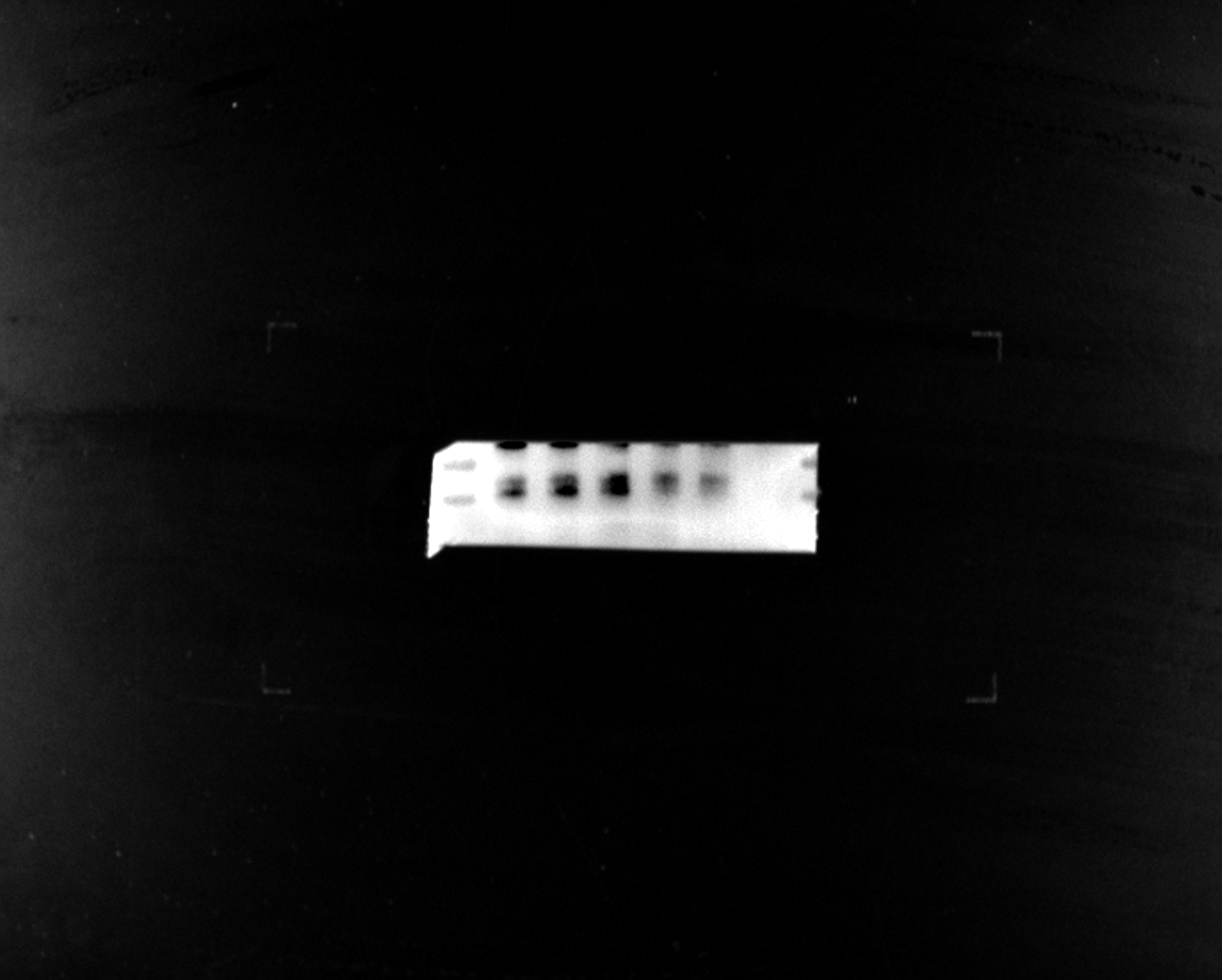

Supplement: Supplementary file 11 — Source data Fig. 6 [file 44318_2024_359_MOESM11_ESM.zip › Figure 6/Fig 6A and 6B/Fig 6A/cyto-lyso/mTOR-merge.Tif]

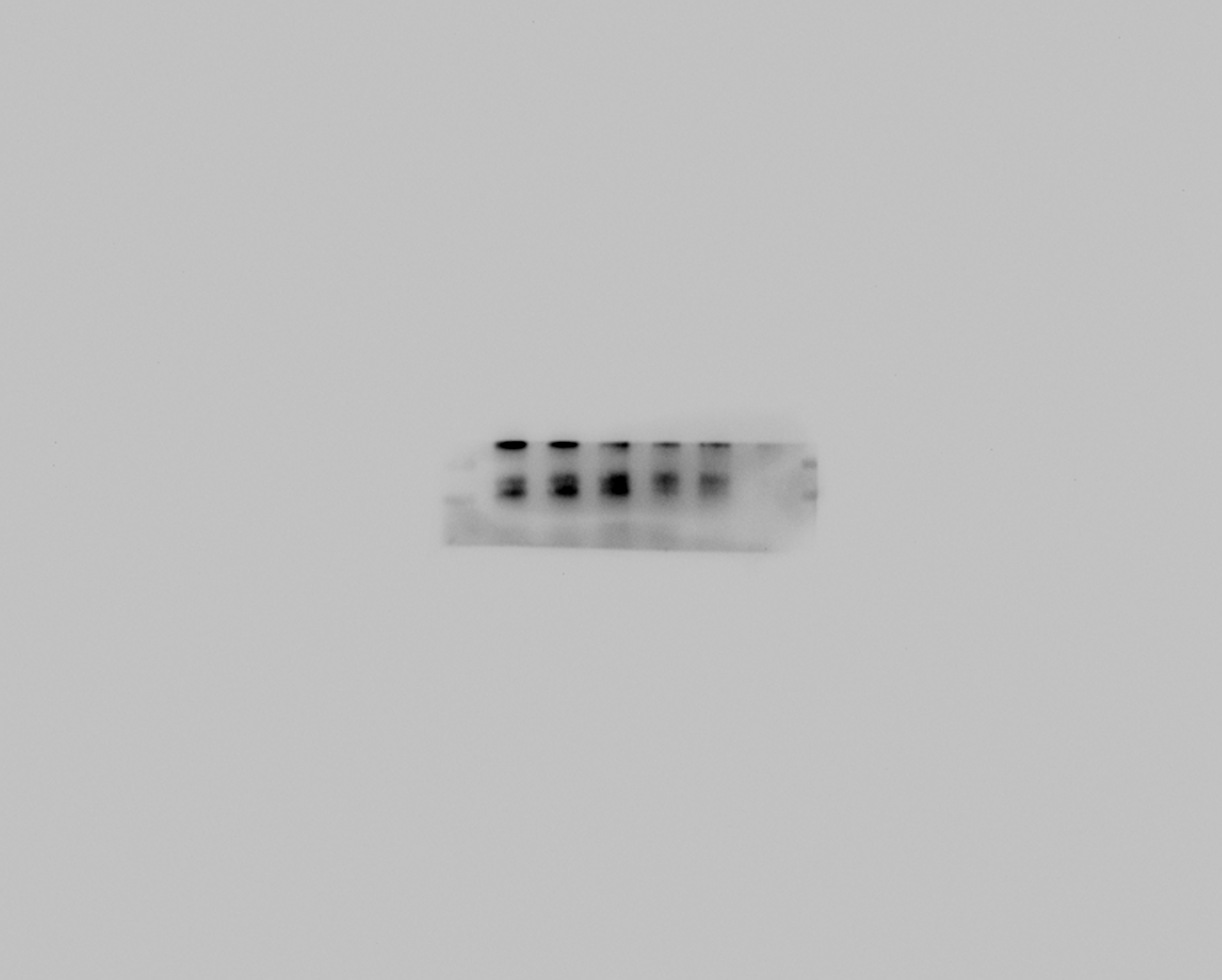

Supplement: Supplementary file 11 — Source data Fig. 6 [file 44318_2024_359_MOESM11_ESM.zip › Figure 6/Fig 6A and 6B/Fig 6A/cyto-lyso/mTOR.Tif]

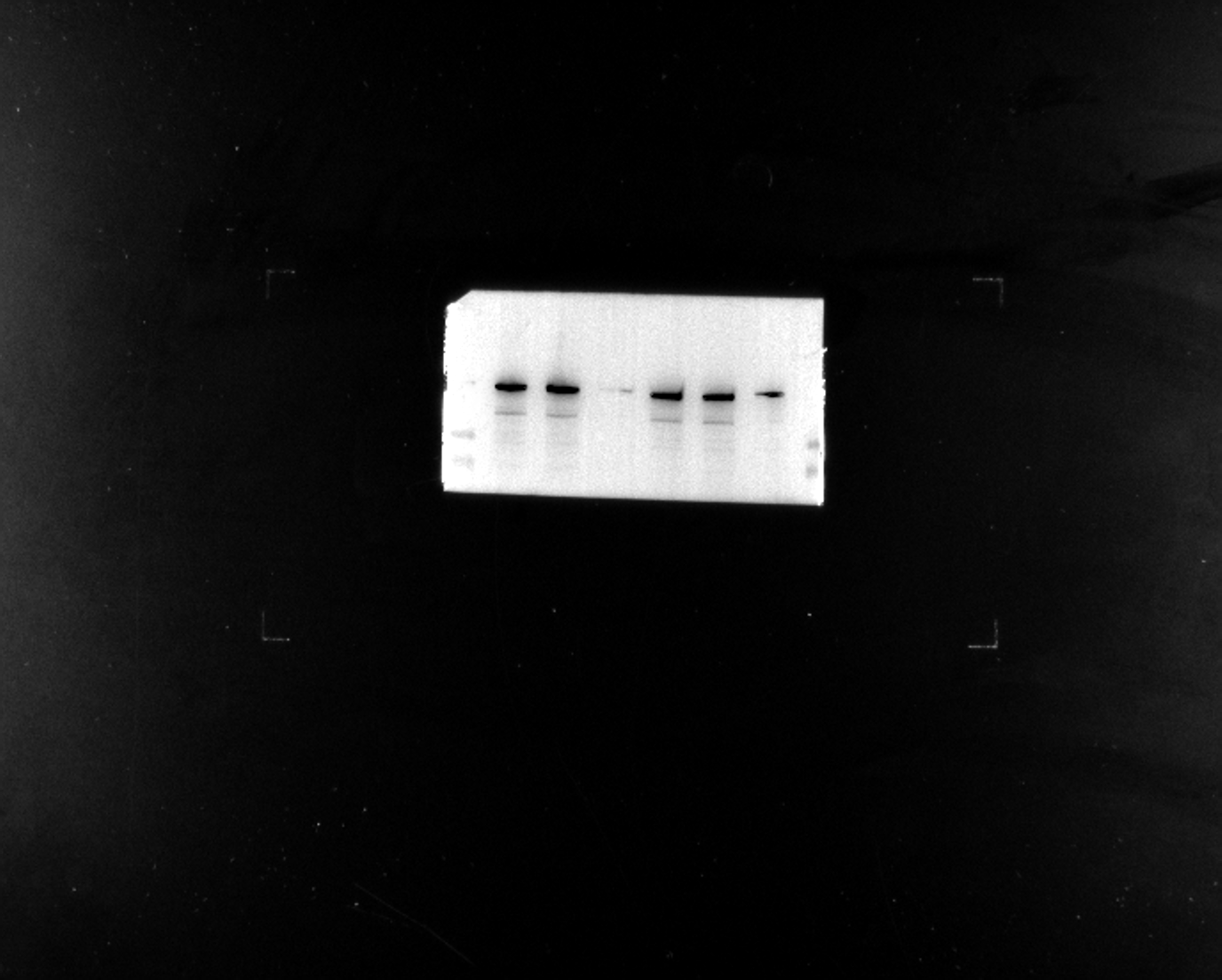

Supplement: Supplementary file 11 — Source data Fig. 6 [file 44318_2024_359_MOESM11_ESM.zip › Figure 6/Fig 6A and 6B/Fig 6A/cyto-lyso/p-mTOR-merge.Tif]

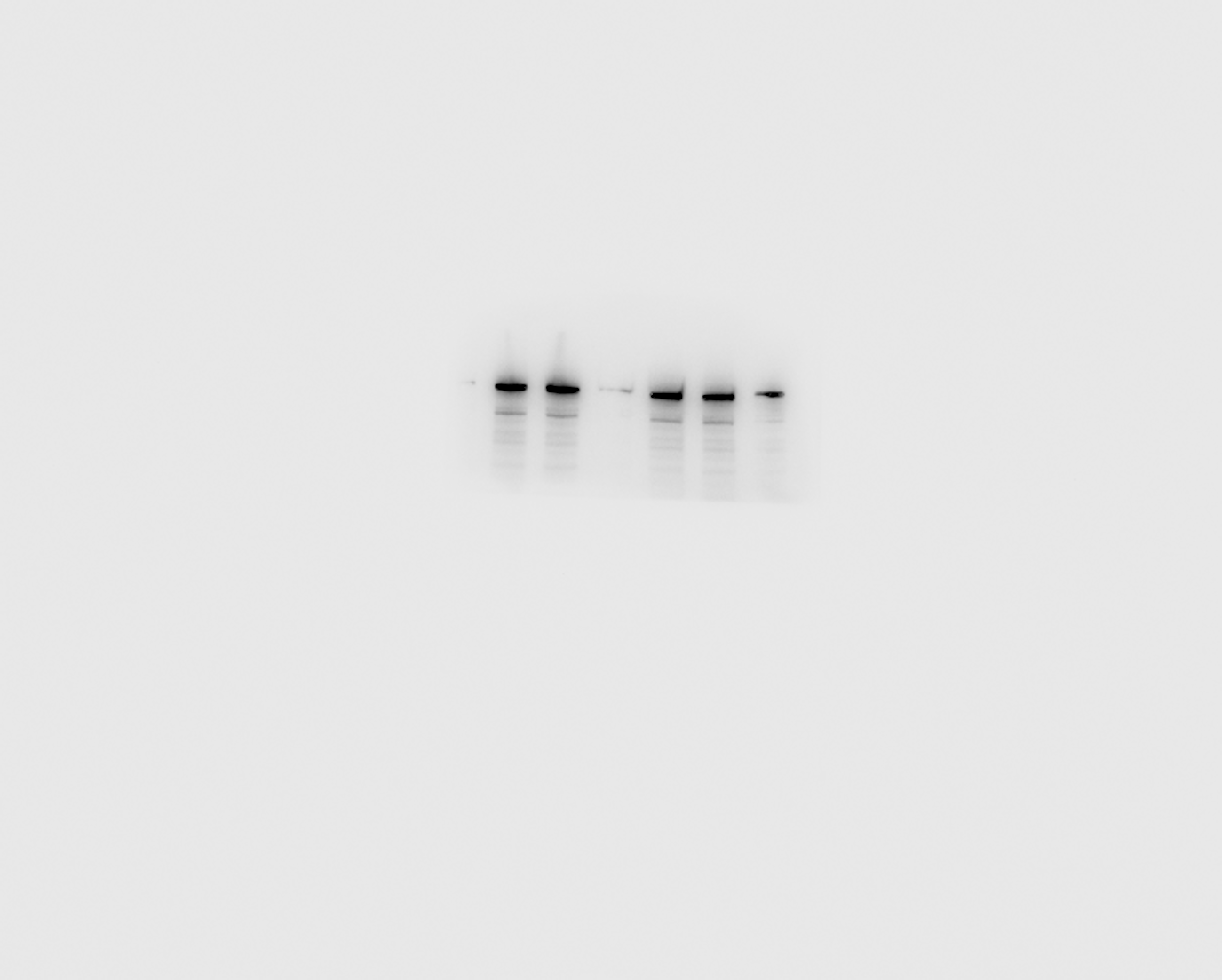

Supplement: Supplementary file 11 — Source data Fig. 6 [file 44318_2024_359_MOESM11_ESM.zip › Figure 6/Fig 6A and 6B/Fig 6A/cyto-lyso/p-mTOR.Tif]

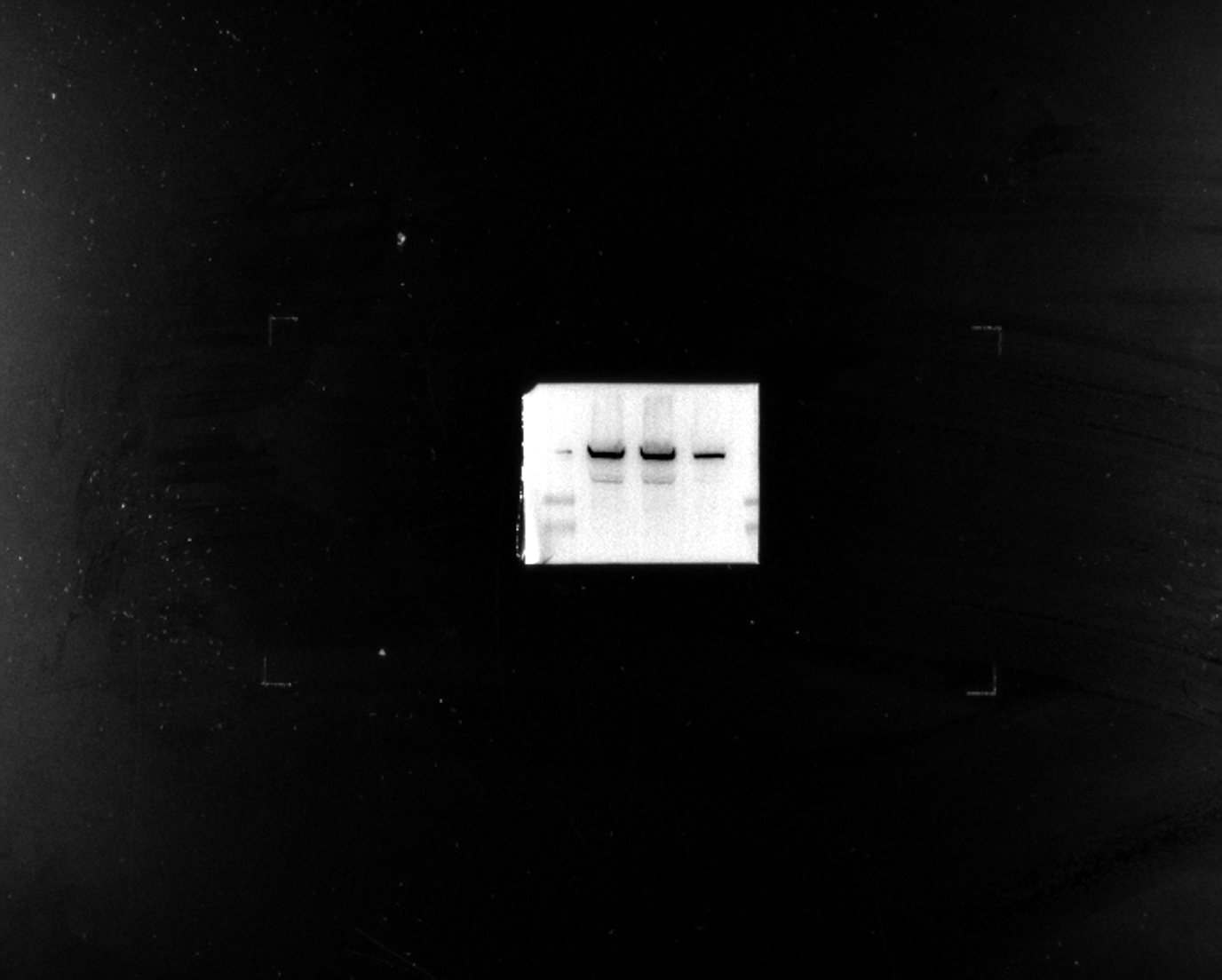

Supplement: Supplementary file 11 — Source data Fig. 6 [file 44318_2024_359_MOESM11_ESM.zip › Figure 6/Fig 6A and 6B/Fig 6A/whole/1-p-mTOR-merge.Tif]

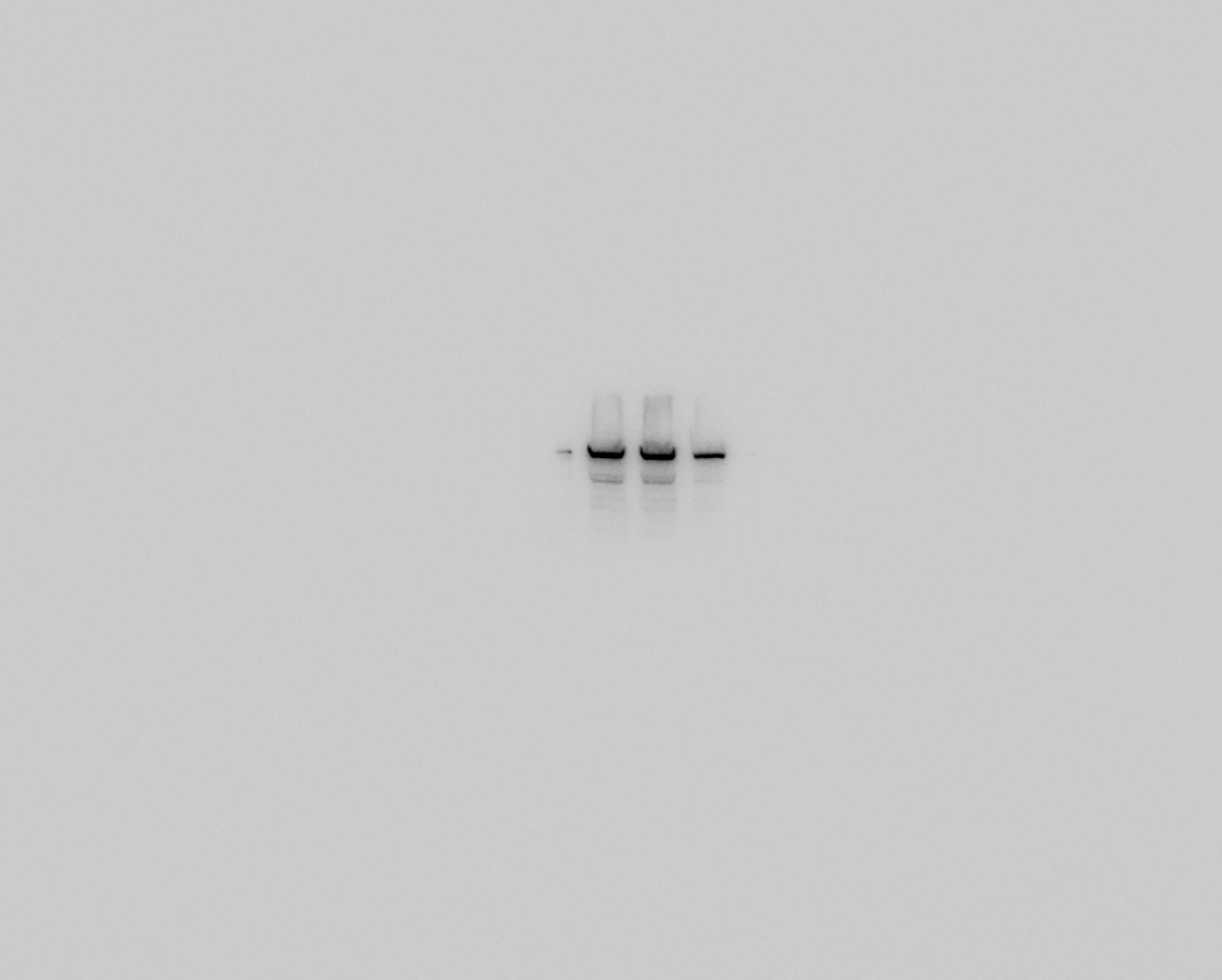

Supplement: Supplementary file 11 — Source data Fig. 6 [file 44318_2024_359_MOESM11_ESM.zip › Figure 6/Fig 6A and 6B/Fig 6A/whole/1-p-mTOR.Tif]

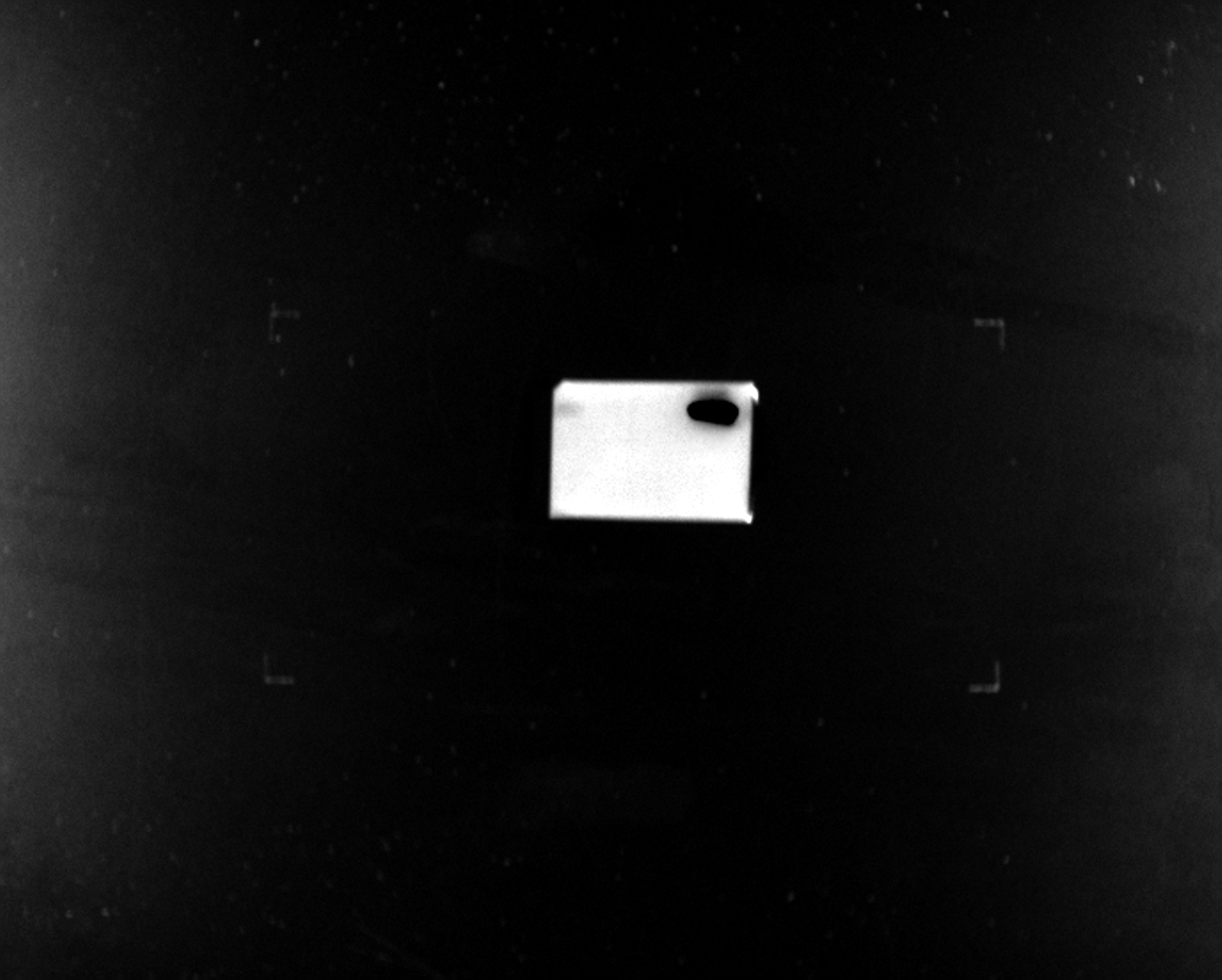

Supplement: Supplementary file 11 — Source data Fig. 6 [file 44318_2024_359_MOESM11_ESM.zip › Figure 6/Fig 6A and 6B/Fig 6A/whole/Flag-merge.Tif]

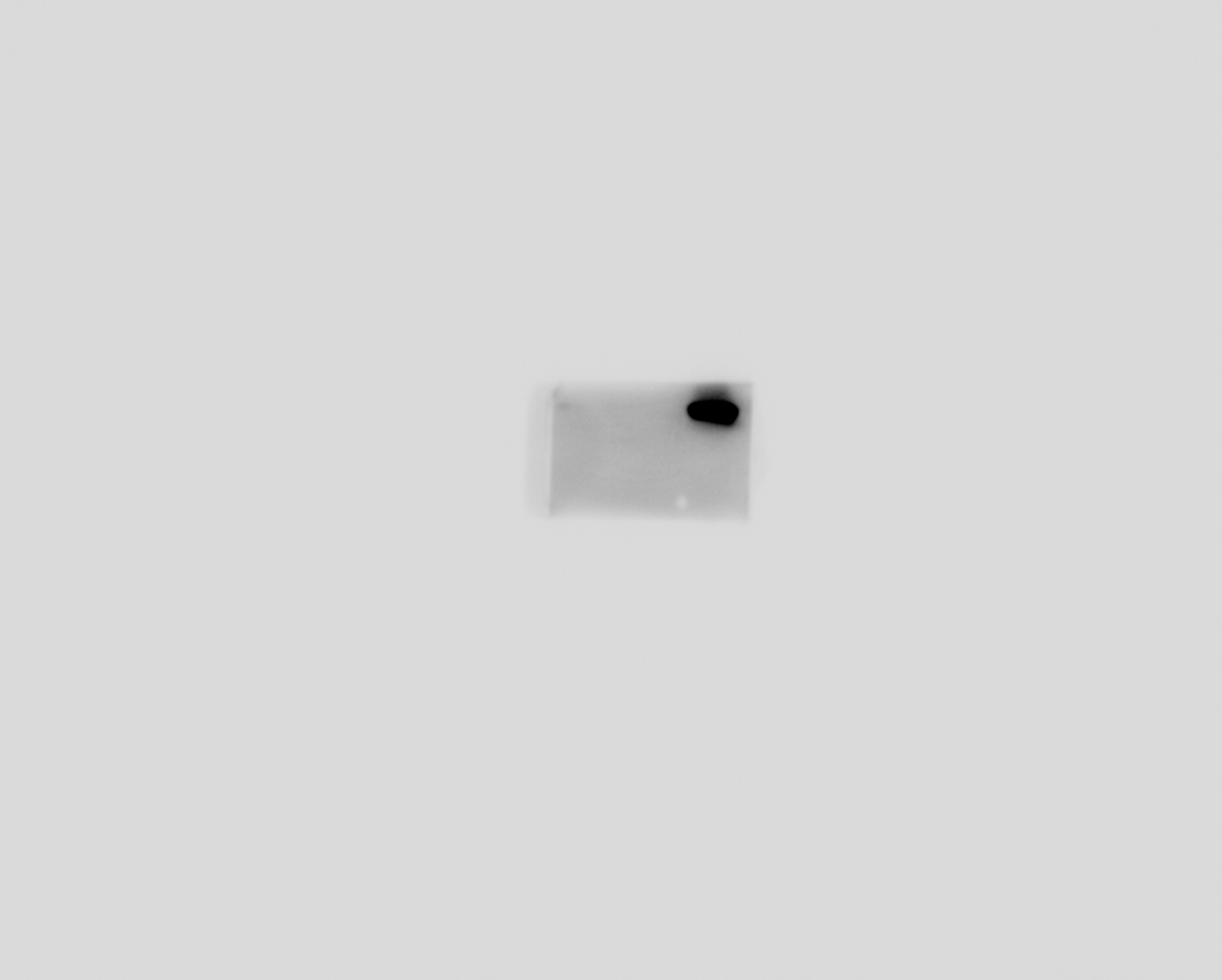

Supplement: Supplementary file 11 — Source data Fig. 6 [file 44318_2024_359_MOESM11_ESM.zip › Figure 6/Fig 6A and 6B/Fig 6A/whole/Flag.Tif]

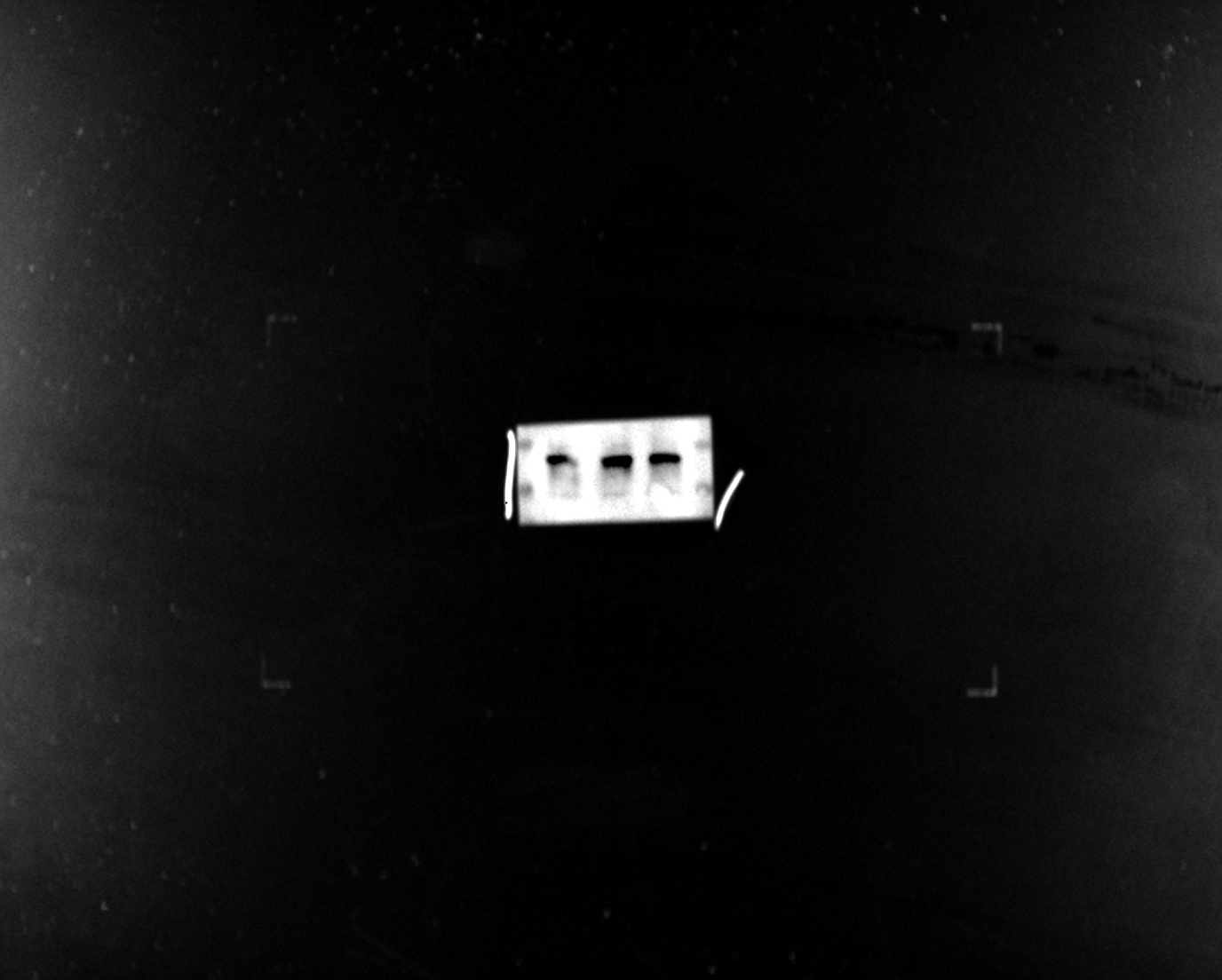

Supplement: Supplementary file 11 — Source data Fig. 6 [file 44318_2024_359_MOESM11_ESM.zip › Figure 6/Fig 6A and 6B/Fig 6A/whole/GAPDH-merge.Tif]

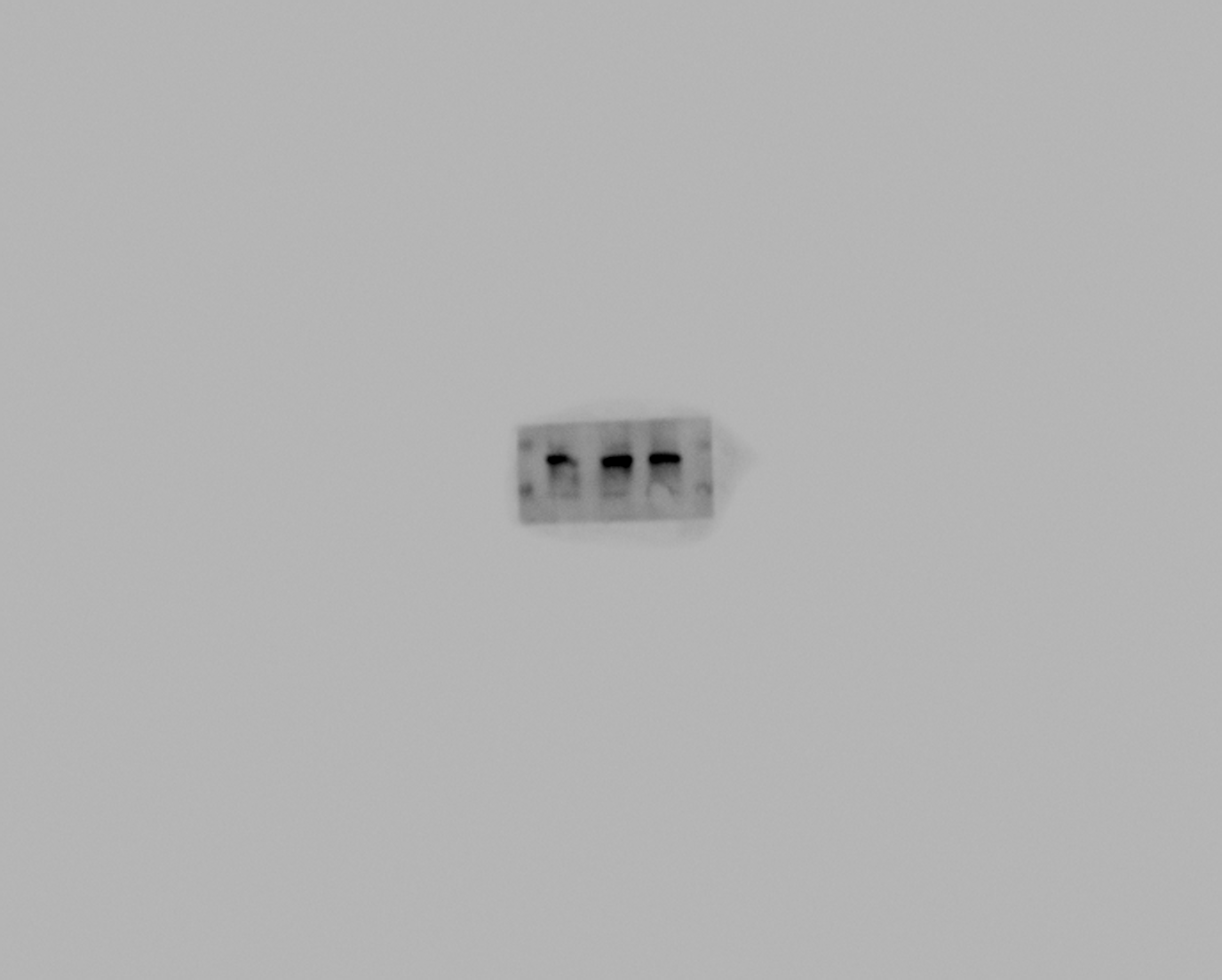

Supplement: Supplementary file 11 — Source data Fig. 6 [file 44318_2024_359_MOESM11_ESM.zip › Figure 6/Fig 6A and 6B/Fig 6A/whole/GAPDH.Tif]

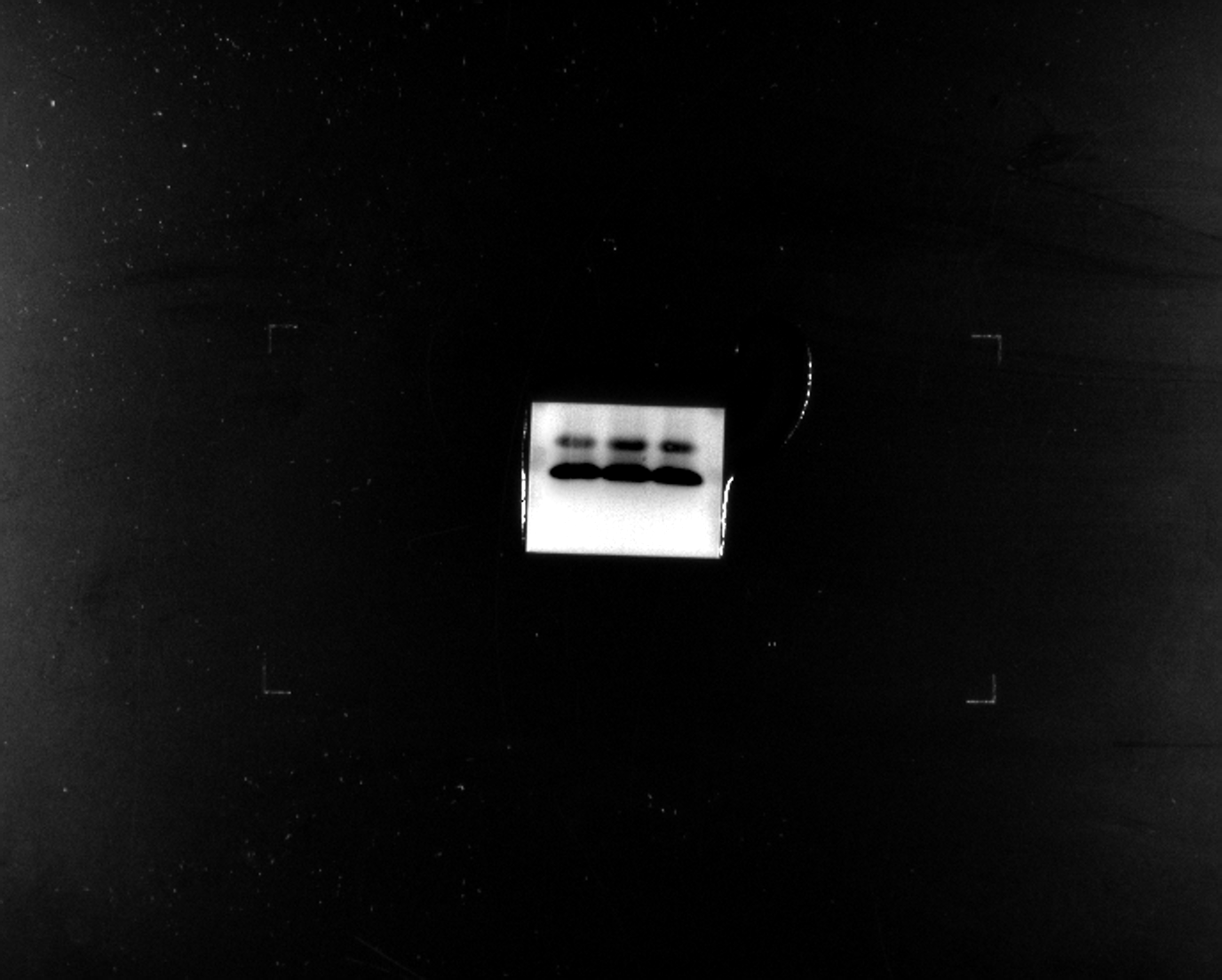

Supplement: Supplementary file 11 — Source data Fig. 6 [file 44318_2024_359_MOESM11_ESM.zip › Figure 6/Fig 6A and 6B/Fig 6A/whole/L1+L4-merge.Tif]

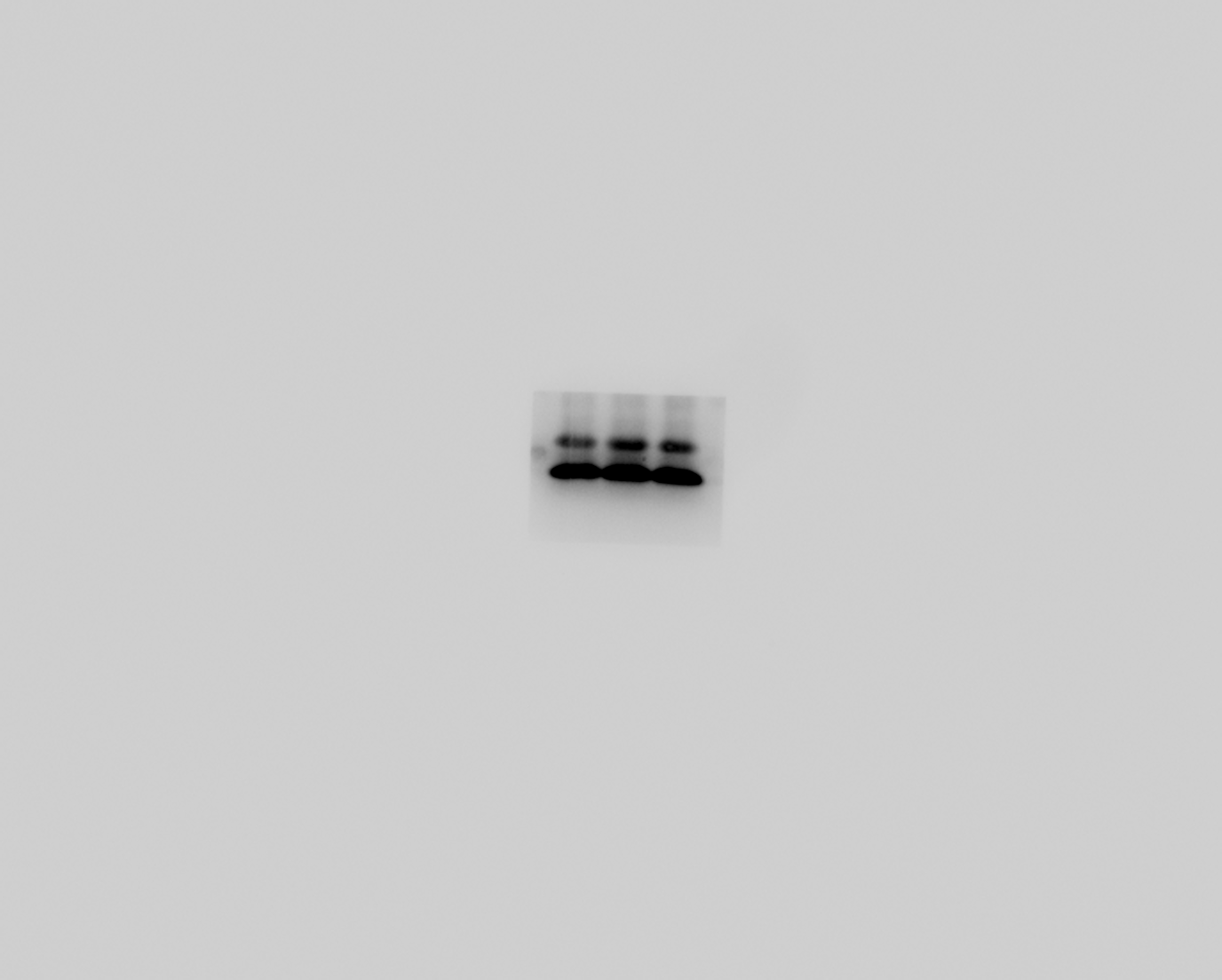

Supplement: Supplementary file 11 — Source data Fig. 6 [file 44318_2024_359_MOESM11_ESM.zip › Figure 6/Fig 6A and 6B/Fig 6A/whole/L1+L4.Tif]

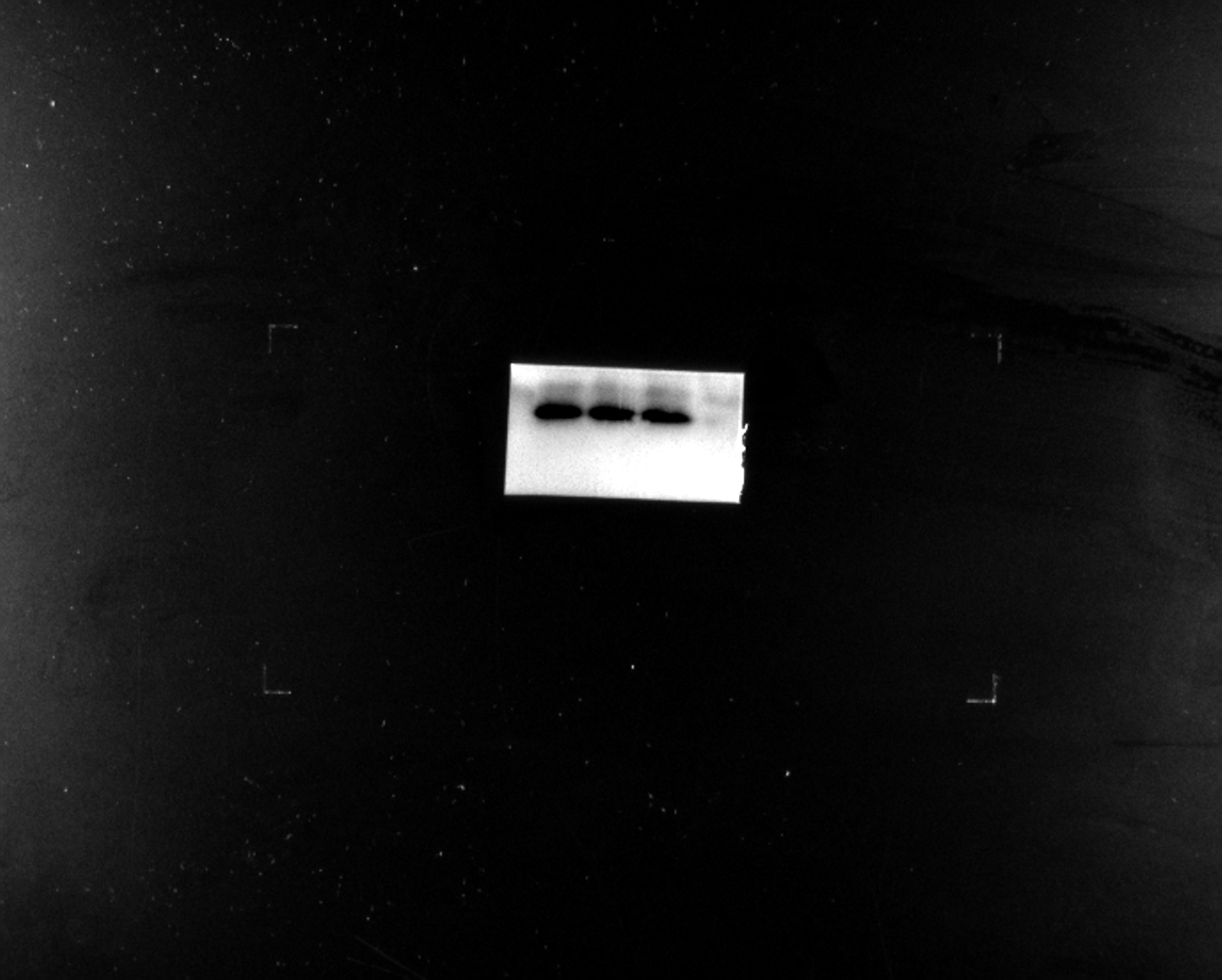

Supplement: Supplementary file 11 — Source data Fig. 6 [file 44318_2024_359_MOESM11_ESM.zip › Figure 6/Fig 6A and 6B/Fig 6A/whole/L2+L5-merge.Tif]

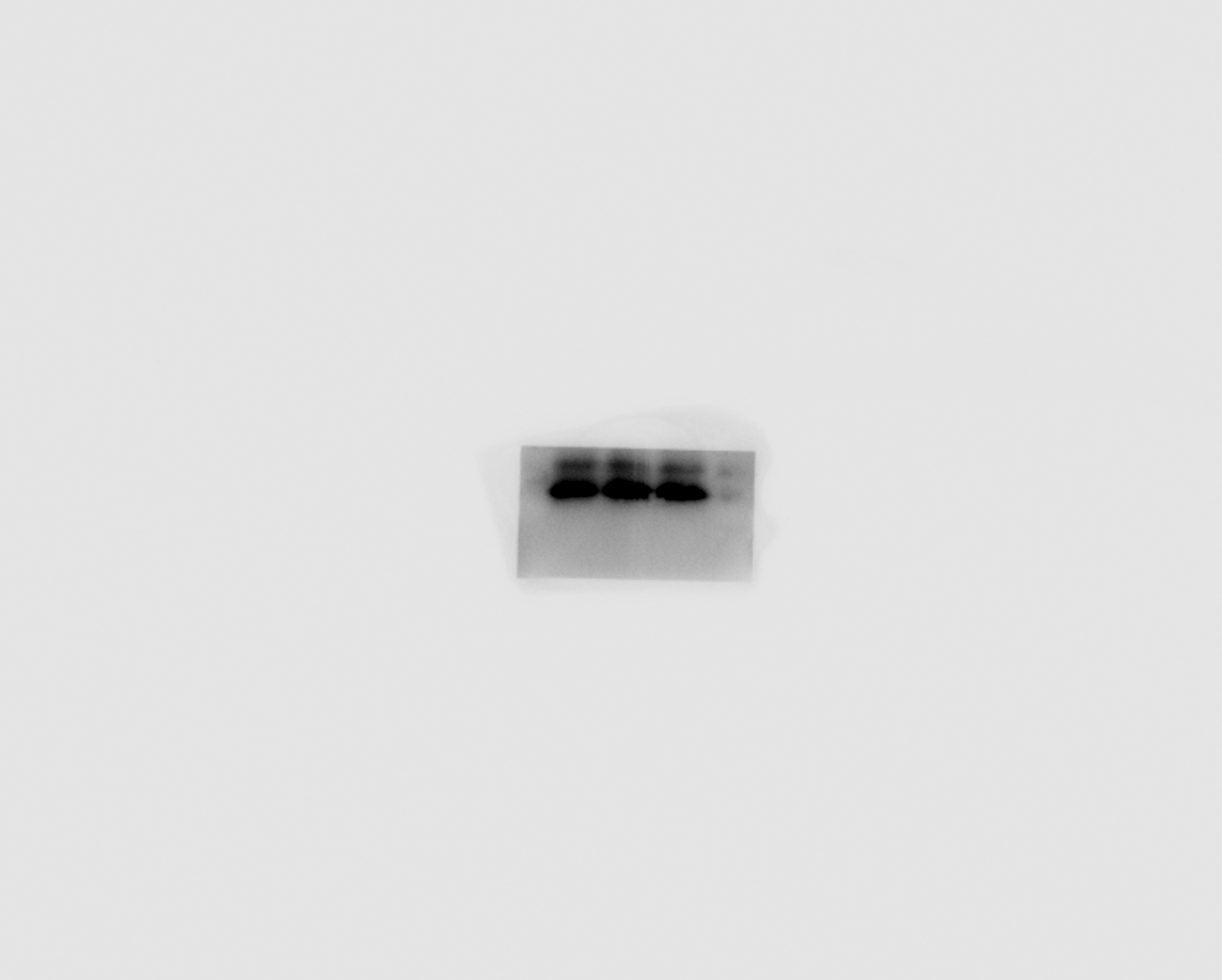

Supplement: Supplementary file 11 — Source data Fig. 6 [file 44318_2024_359_MOESM11_ESM.zip › Figure 6/Fig 6A and 6B/Fig 6A/whole/L2+L5.Tif]

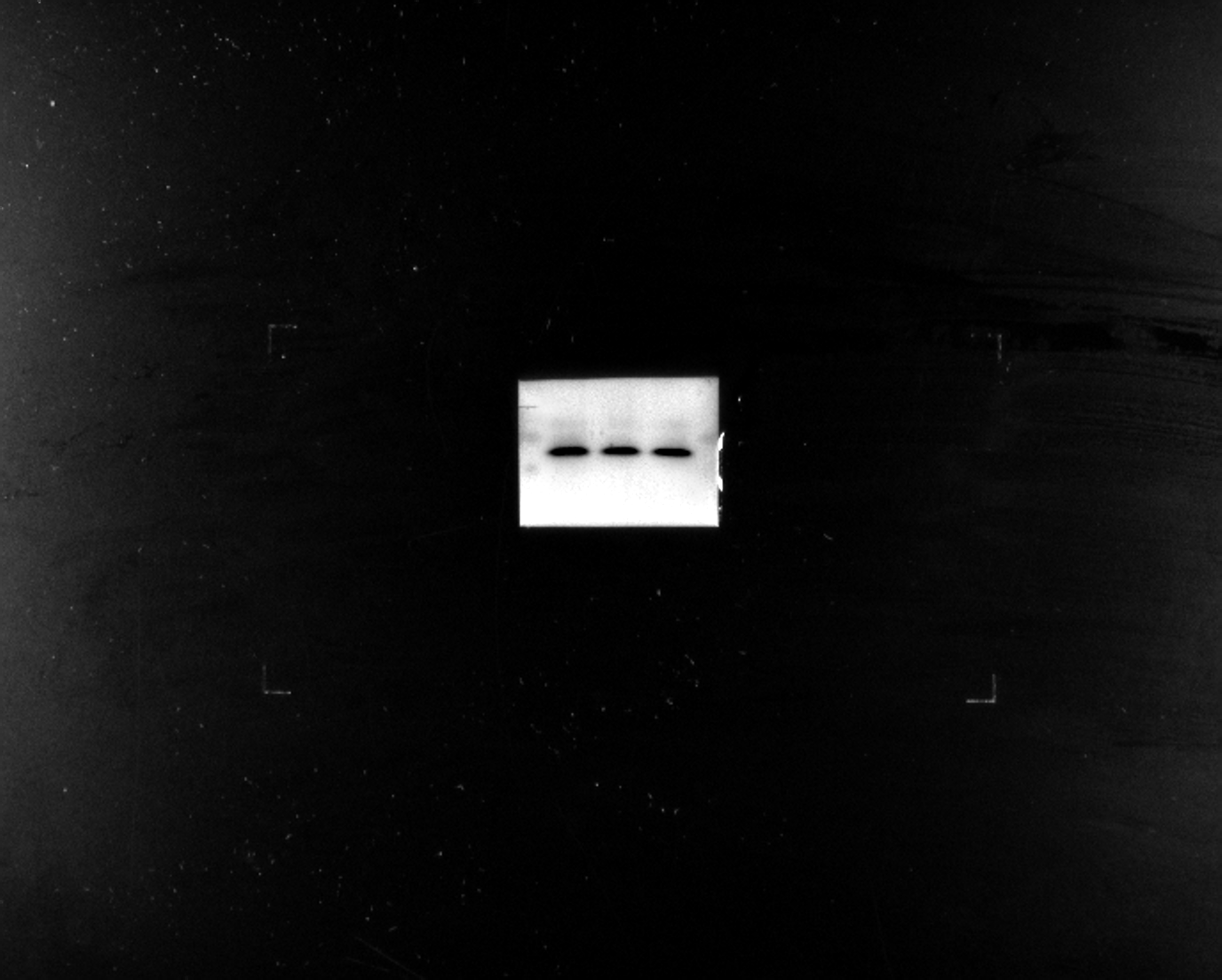

Supplement: Supplementary file 11 — Source data Fig. 6 [file 44318_2024_359_MOESM11_ESM.zip › Figure 6/Fig 6A and 6B/Fig 6A/whole/L3-merge.Tif]

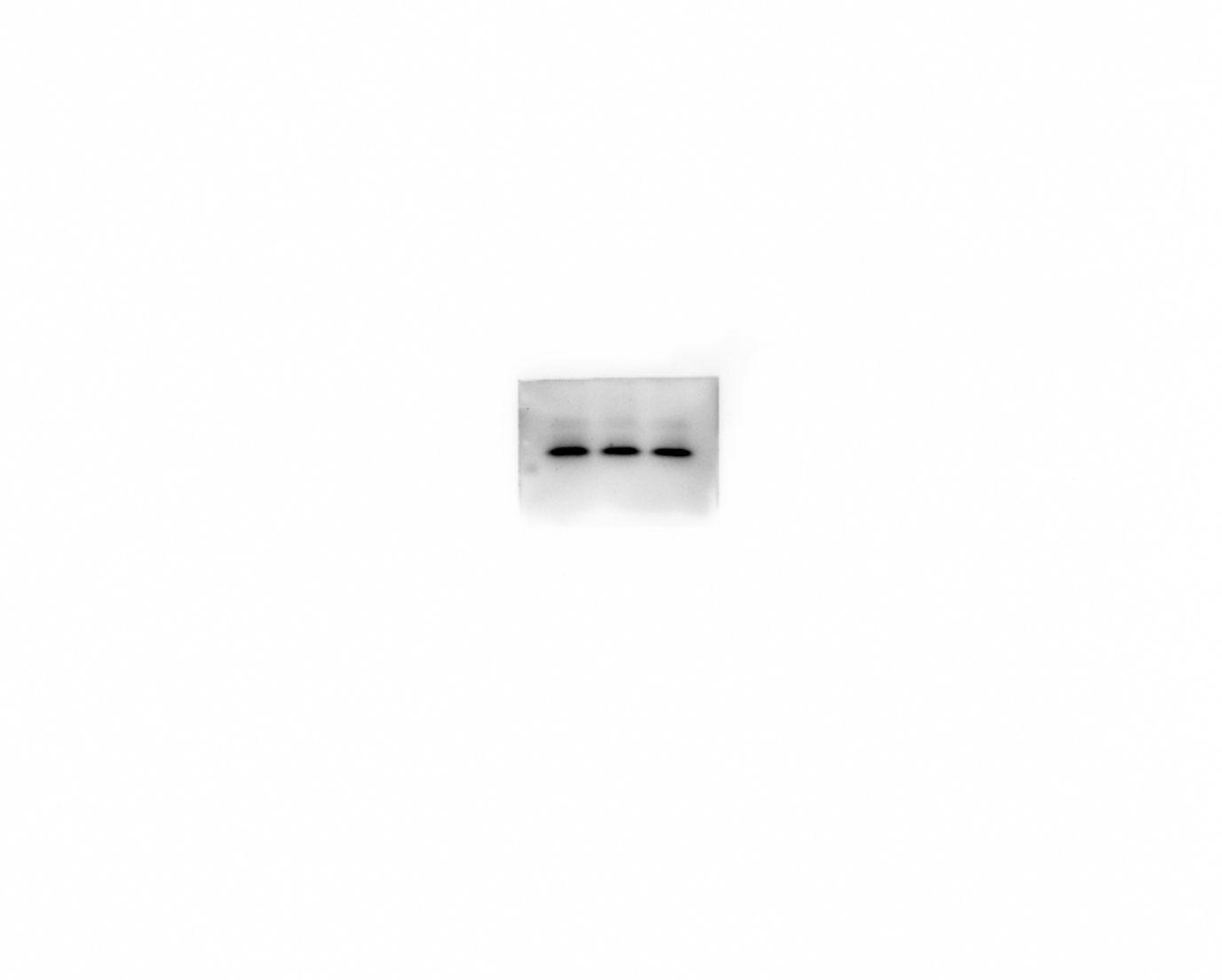

Supplement: Supplementary file 11 — Source data Fig. 6 [file 44318_2024_359_MOESM11_ESM.zip › Figure 6/Fig 6A and 6B/Fig 6A/whole/L3.Tif]

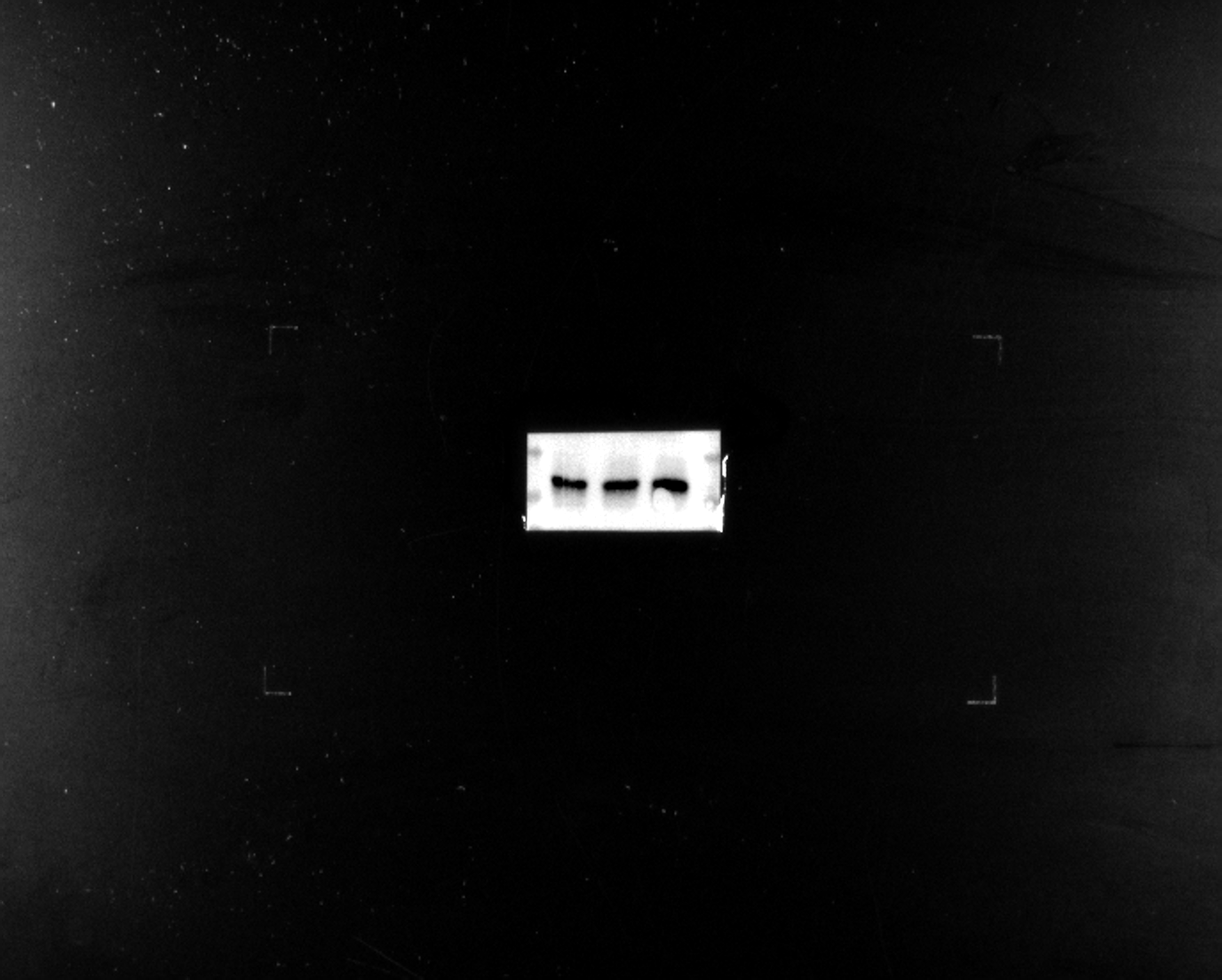

Supplement: Supplementary file 11 — Source data Fig. 6 [file 44318_2024_359_MOESM11_ESM.zip › Figure 6/Fig 6A and 6B/Fig 6A/whole/Rag-merge.Tif]

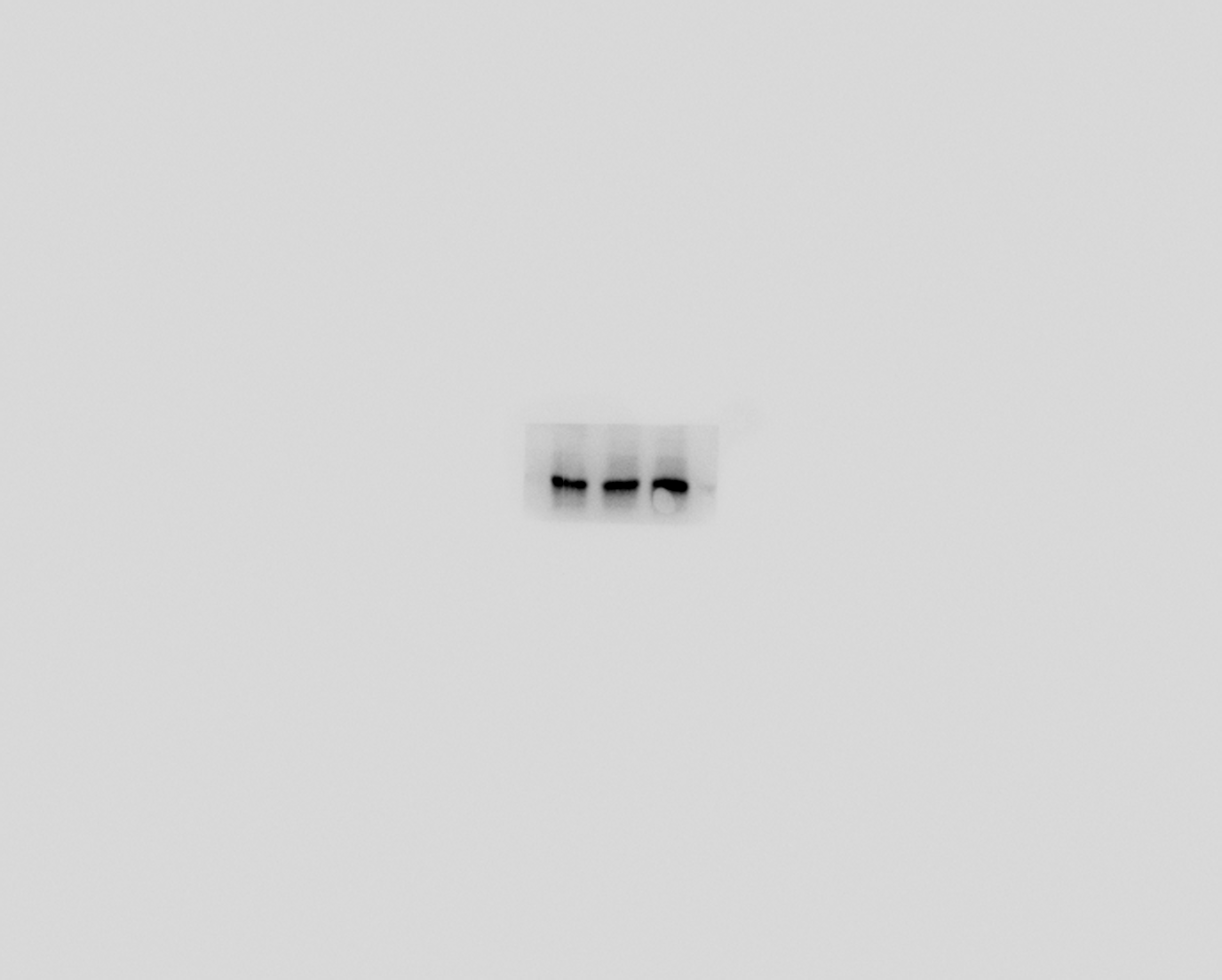

Supplement: Supplementary file 11 — Source data Fig. 6 [file 44318_2024_359_MOESM11_ESM.zip › Figure 6/Fig 6A and 6B/Fig 6A/whole/Rag.Tif]

Fig 6C

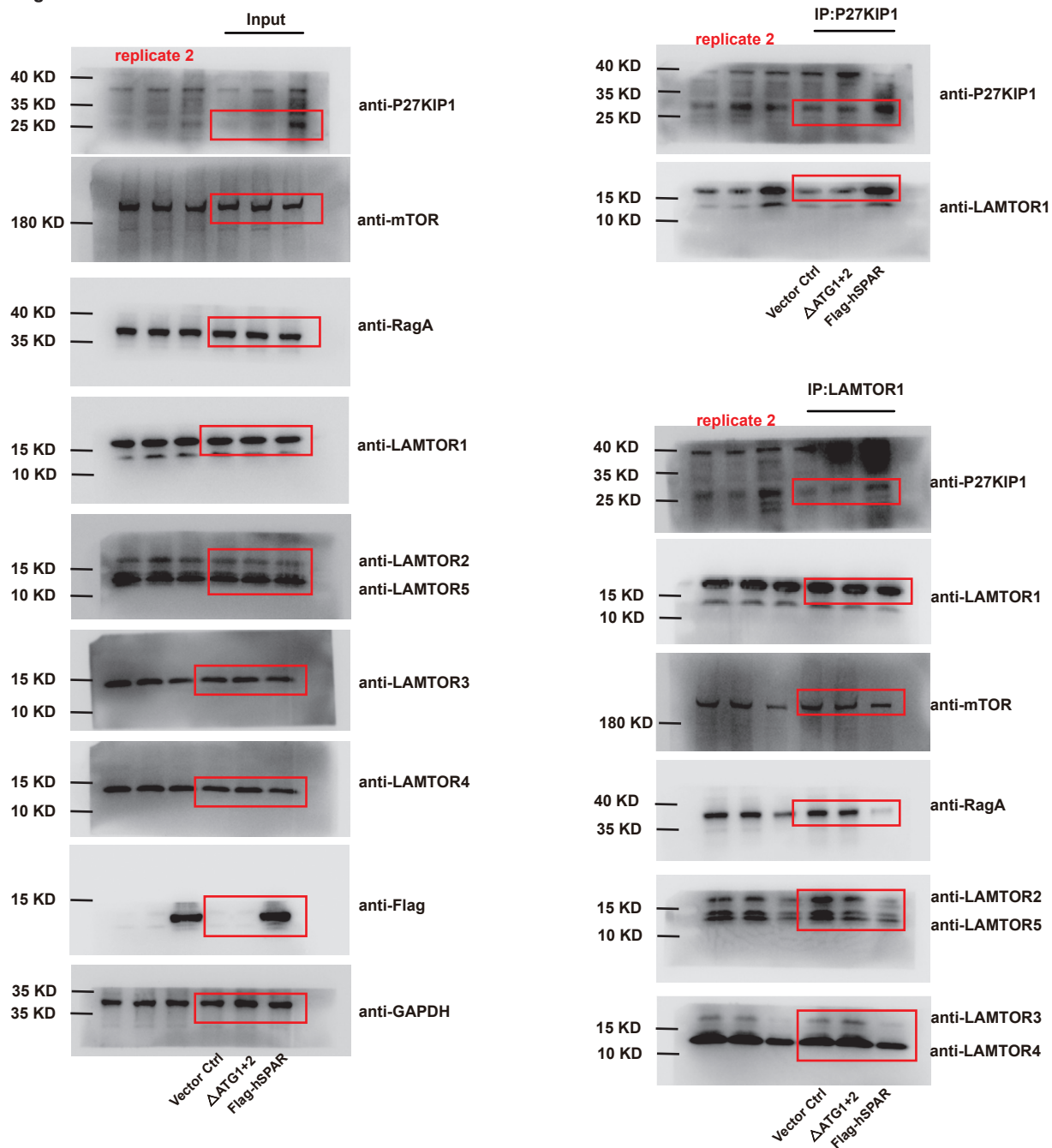

Supplement: Supplementary file 11 — Source data Fig. 6 [file 44318_2024_359_MOESM11_ESM.zip › Figure 6/Fig 6C/Fig 6C.pdf]

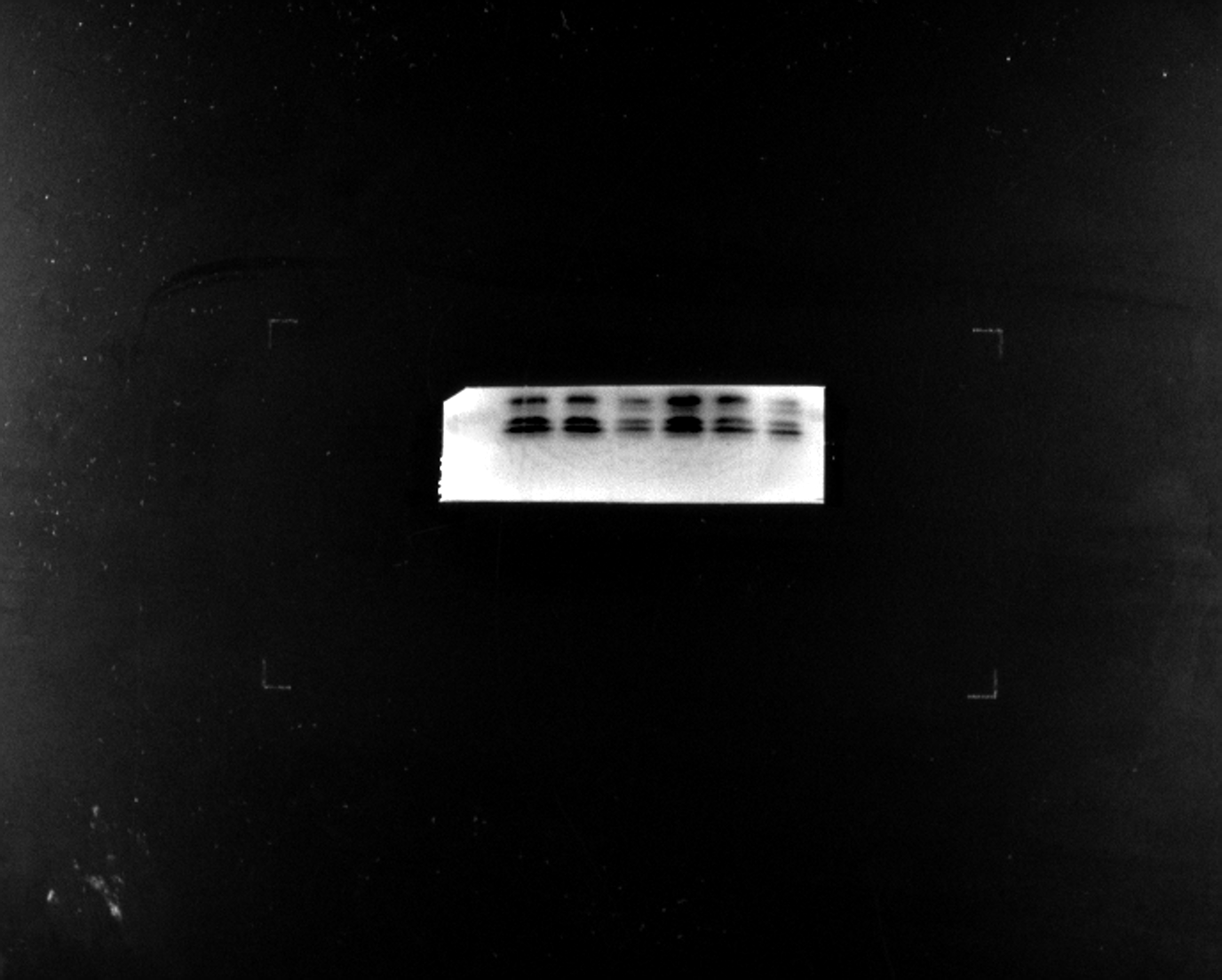

Supplement: Supplementary file 11 — Source data Fig. 6 [file 44318_2024_359_MOESM11_ESM.zip › Figure 6/Fig 6C/IP LAMTOR1/L2+5-merge.Tif]

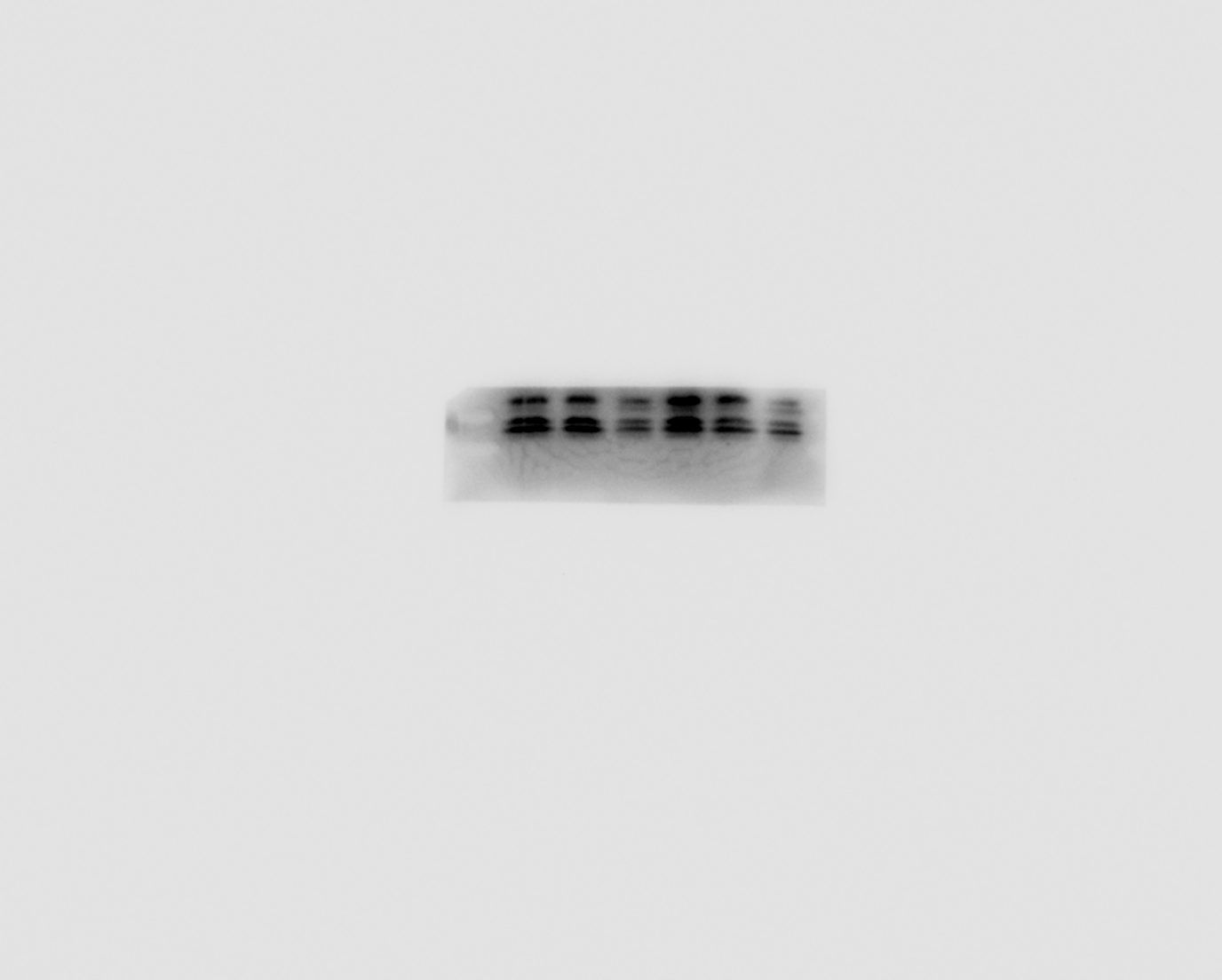

Supplement: Supplementary file 11 — Source data Fig. 6 [file 44318_2024_359_MOESM11_ESM.zip › Figure 6/Fig 6C/IP LAMTOR1/L2+L5.Tif]

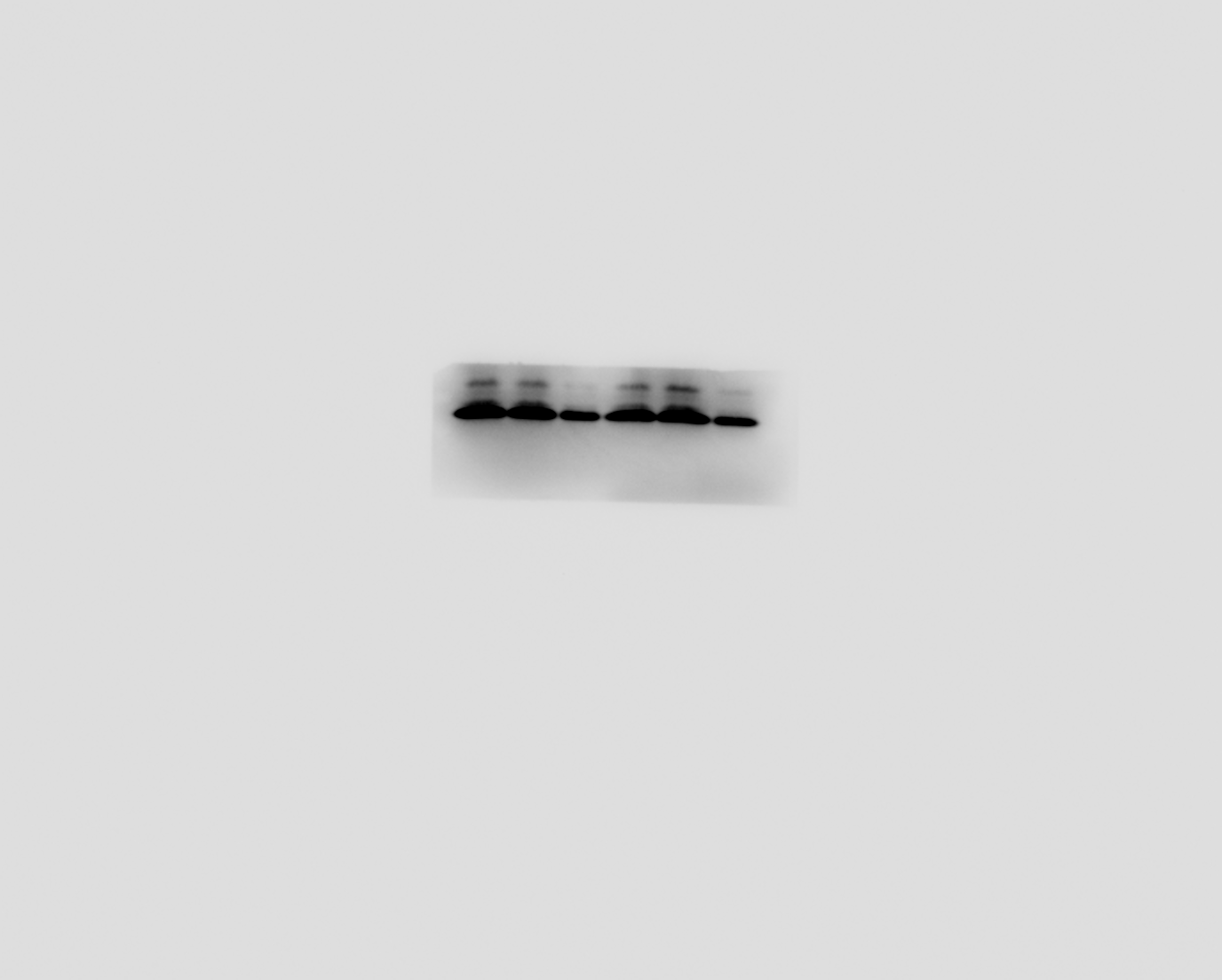

Supplement: Supplementary file 11 — Source data Fig. 6 [file 44318_2024_359_MOESM11_ESM.zip › Figure 6/Fig 6C/IP LAMTOR1/L3+4.Tif]

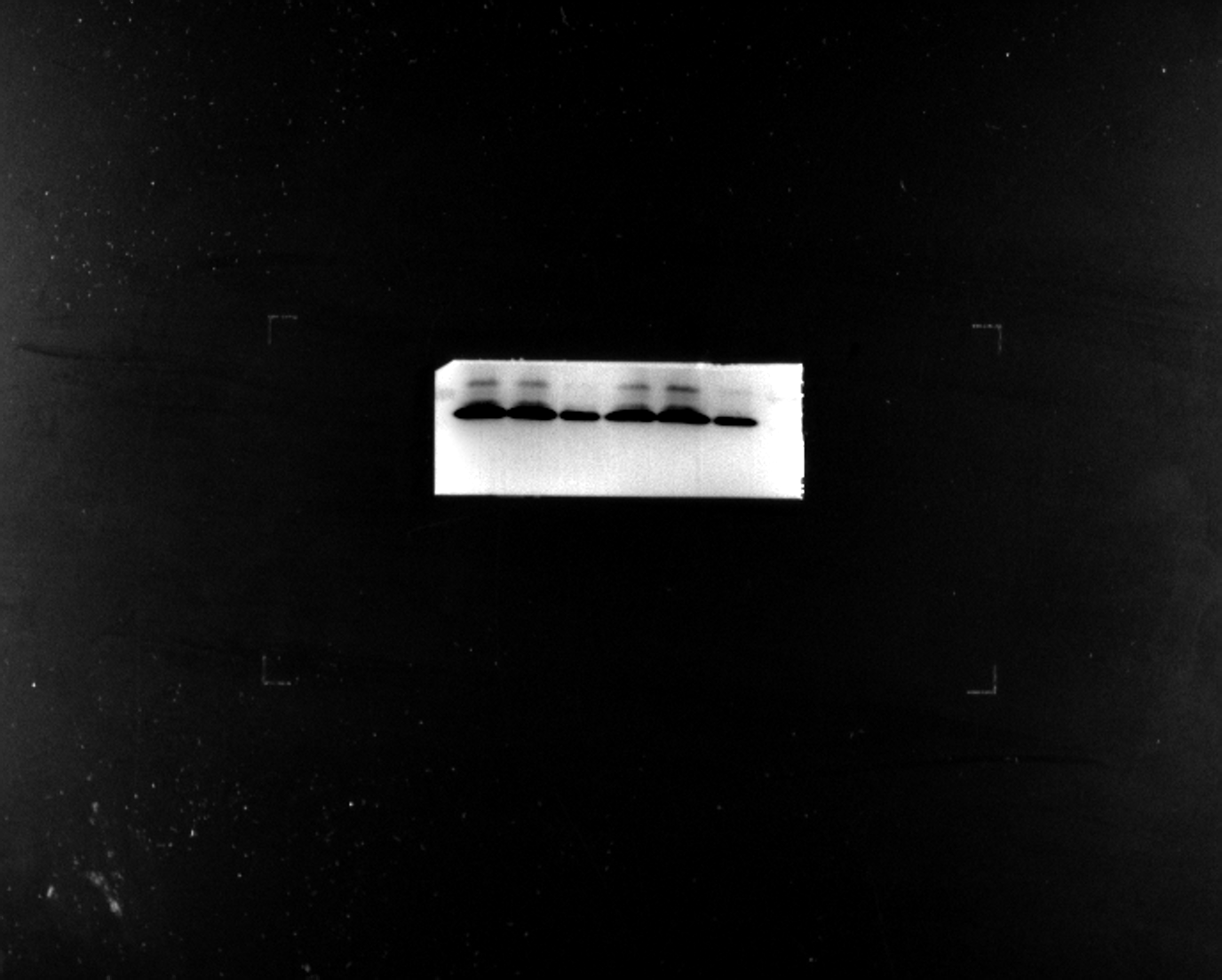

Supplement: Supplementary file 11 — Source data Fig. 6 [file 44318_2024_359_MOESM11_ESM.zip › Figure 6/Fig 6C/IP LAMTOR1/L3+L4-merge.Tif]

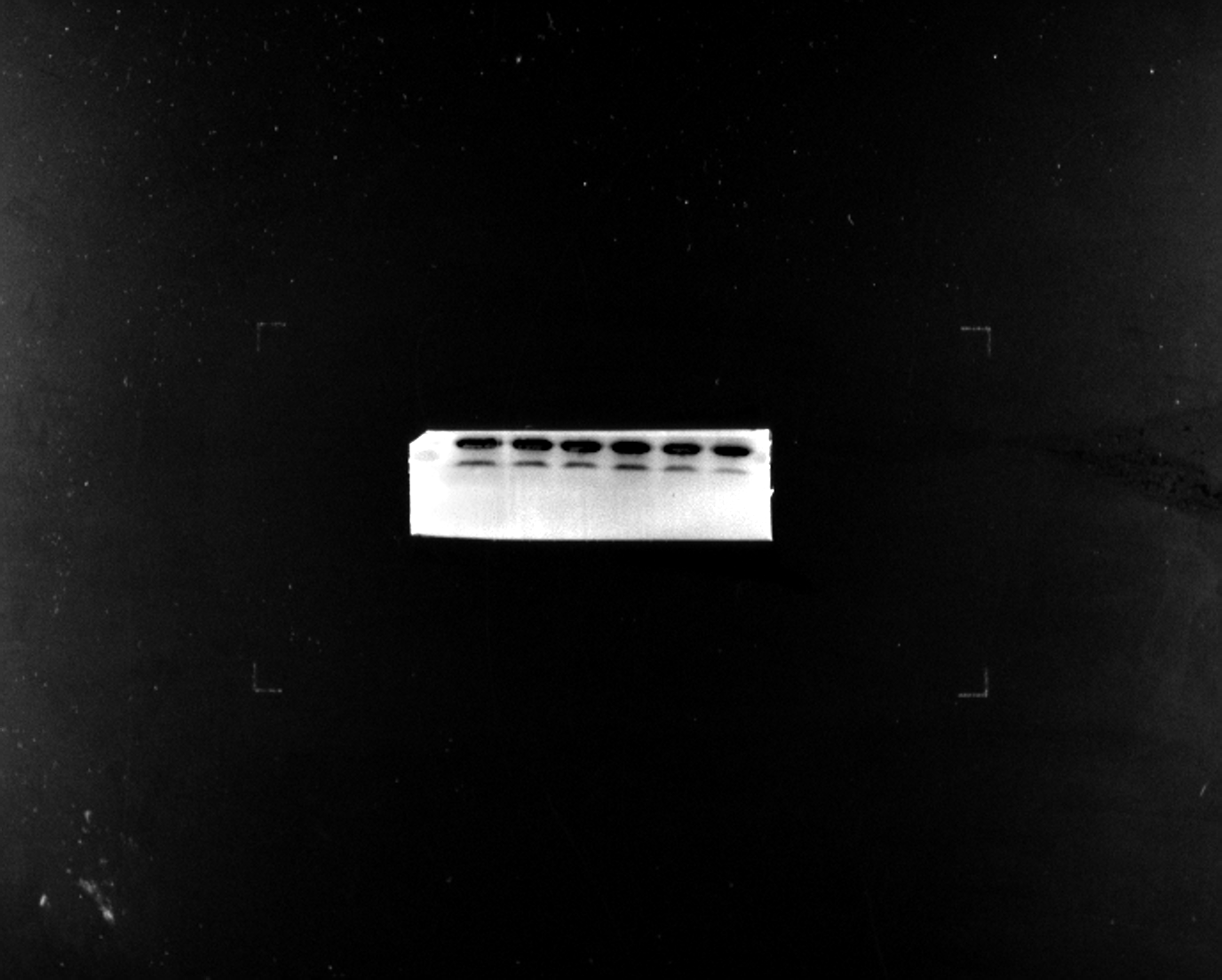

Supplement: Supplementary file 11 — Source data Fig. 6 [file 44318_2024_359_MOESM11_ESM.zip › Figure 6/Fig 6C/IP LAMTOR1/LAMTOR1-merge.Tif]

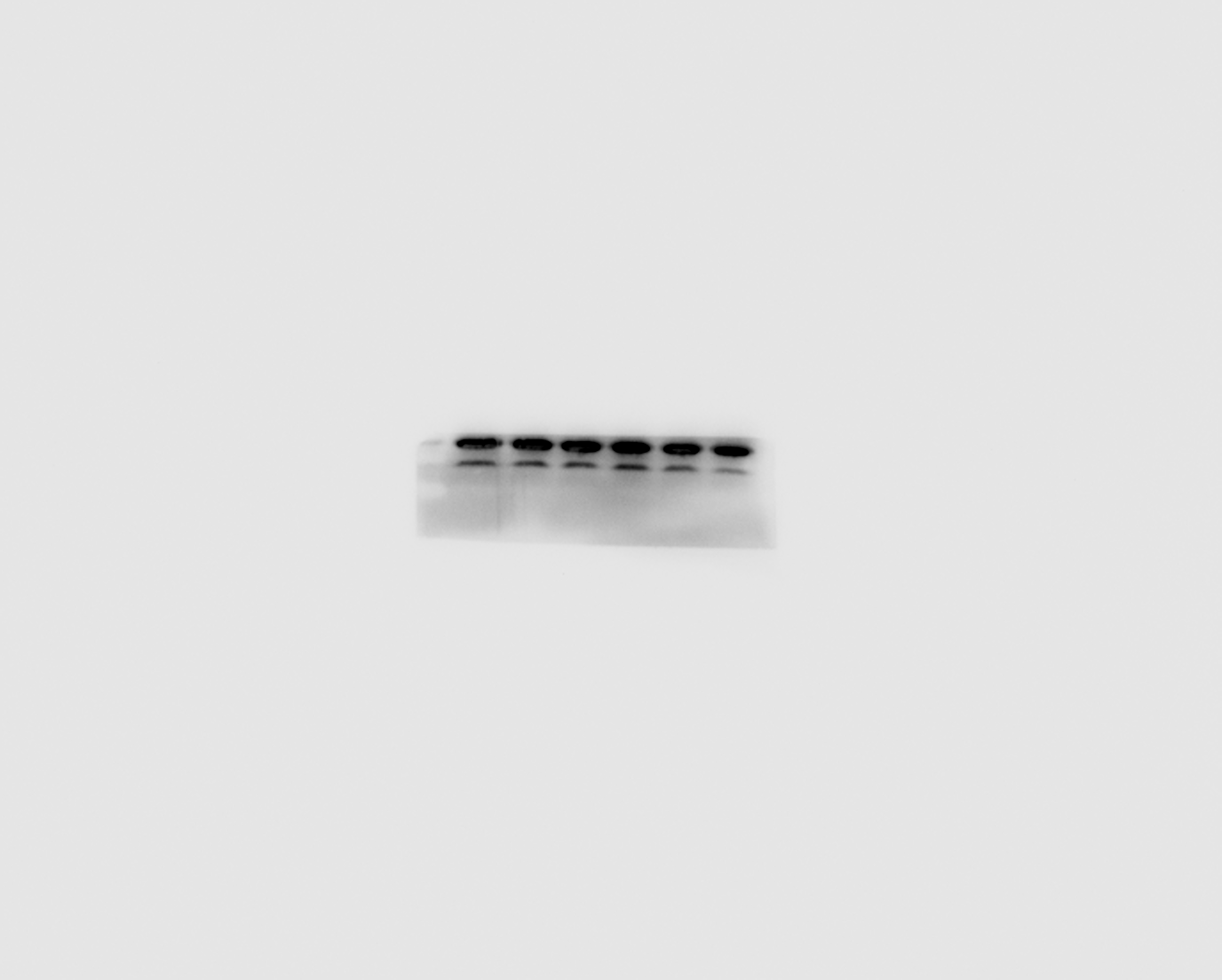

Supplement: Supplementary file 11 — Source data Fig. 6 [file 44318_2024_359_MOESM11_ESM.zip › Figure 6/Fig 6C/IP LAMTOR1/LAMTOR1.Tif]

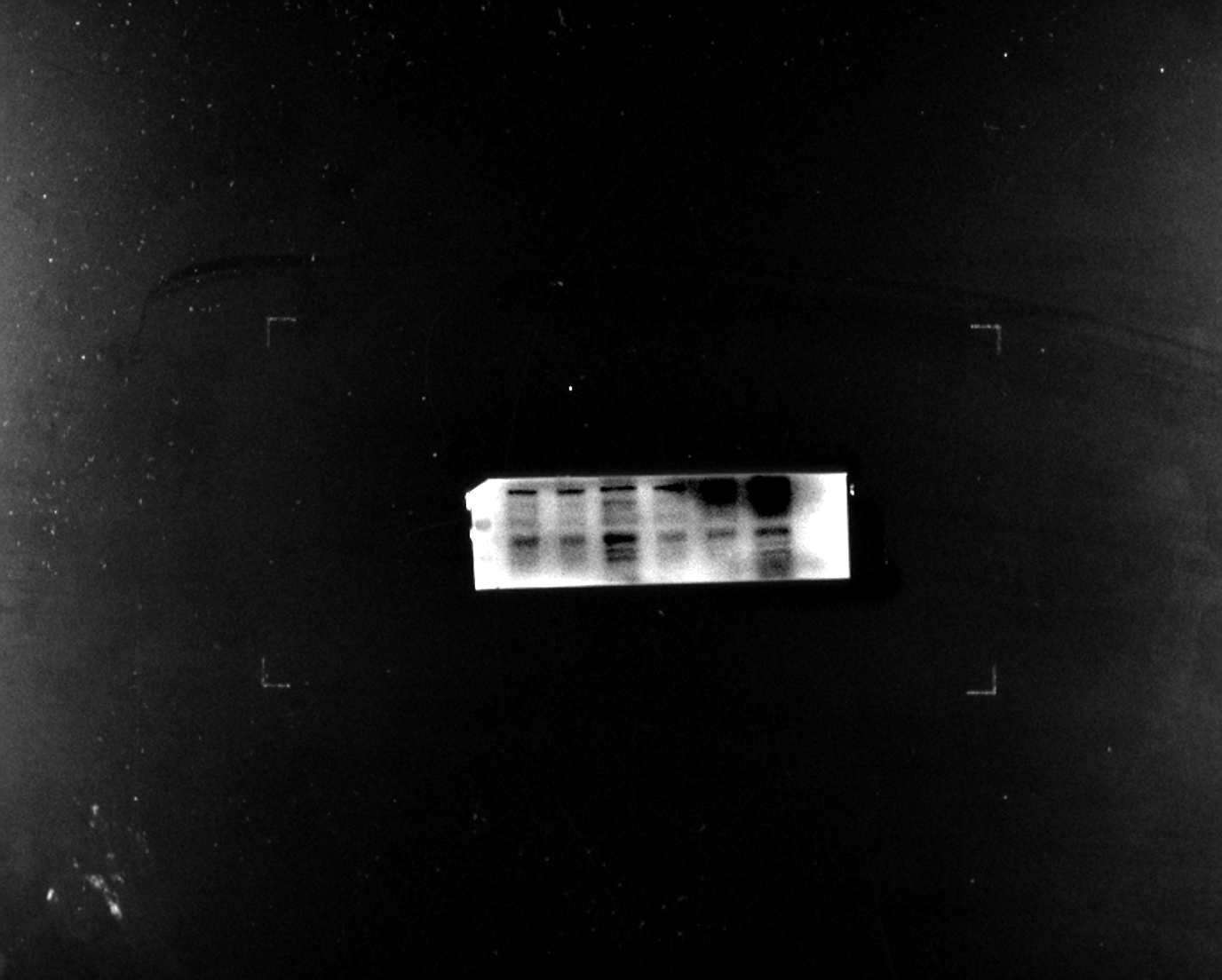

Supplement: Supplementary file 11 — Source data Fig. 6 [file 44318_2024_359_MOESM11_ESM.zip › Figure 6/Fig 6C/IP LAMTOR1/P27KIP1-merge.Tif]

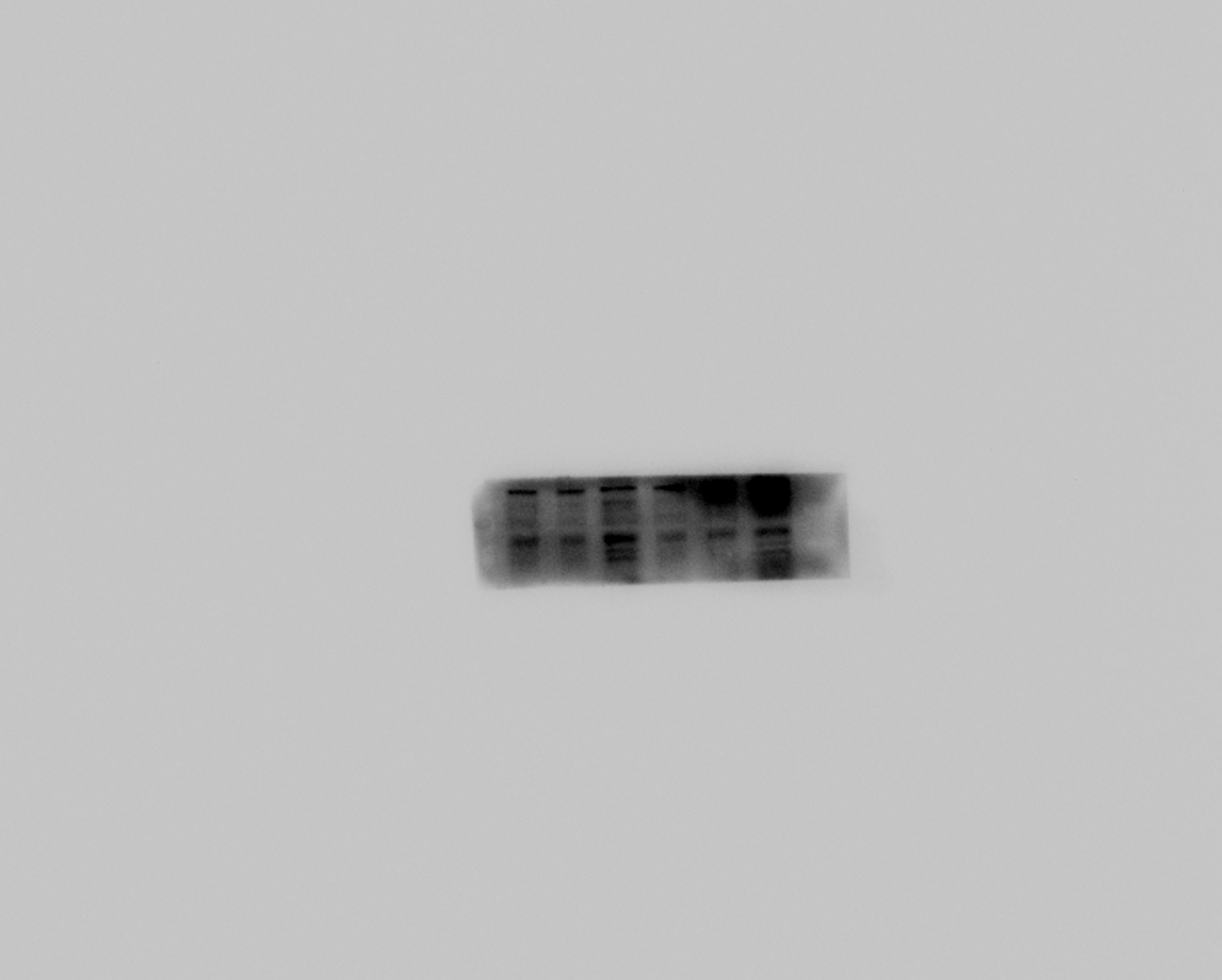

Supplement: Supplementary file 11 — Source data Fig. 6 [file 44318_2024_359_MOESM11_ESM.zip › Figure 6/Fig 6C/IP LAMTOR1/P27KIP1.Tif]

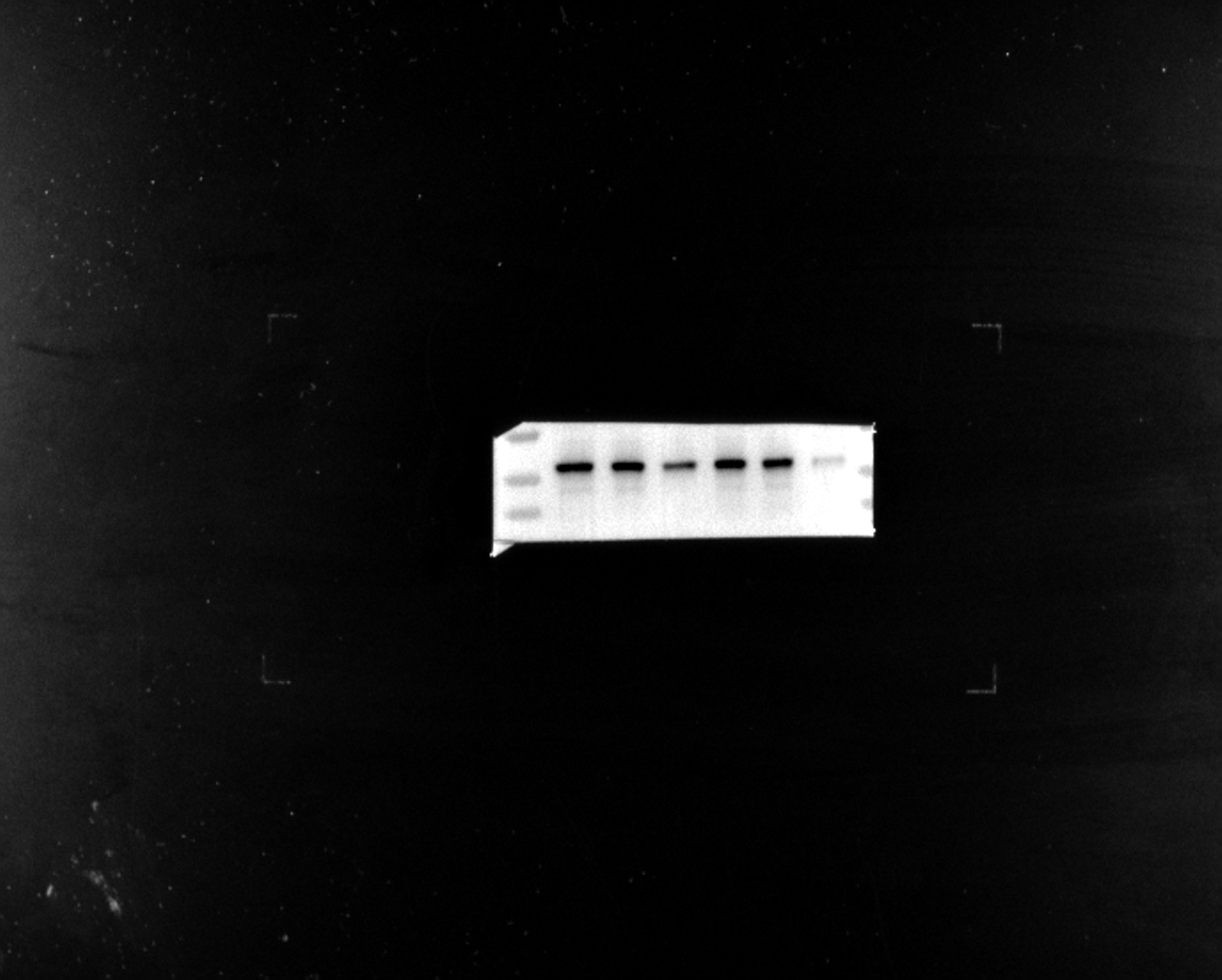

Supplement: Supplementary file 11 — Source data Fig. 6 [file 44318_2024_359_MOESM11_ESM.zip › Figure 6/Fig 6C/IP LAMTOR1/RagA-merge.Tif]

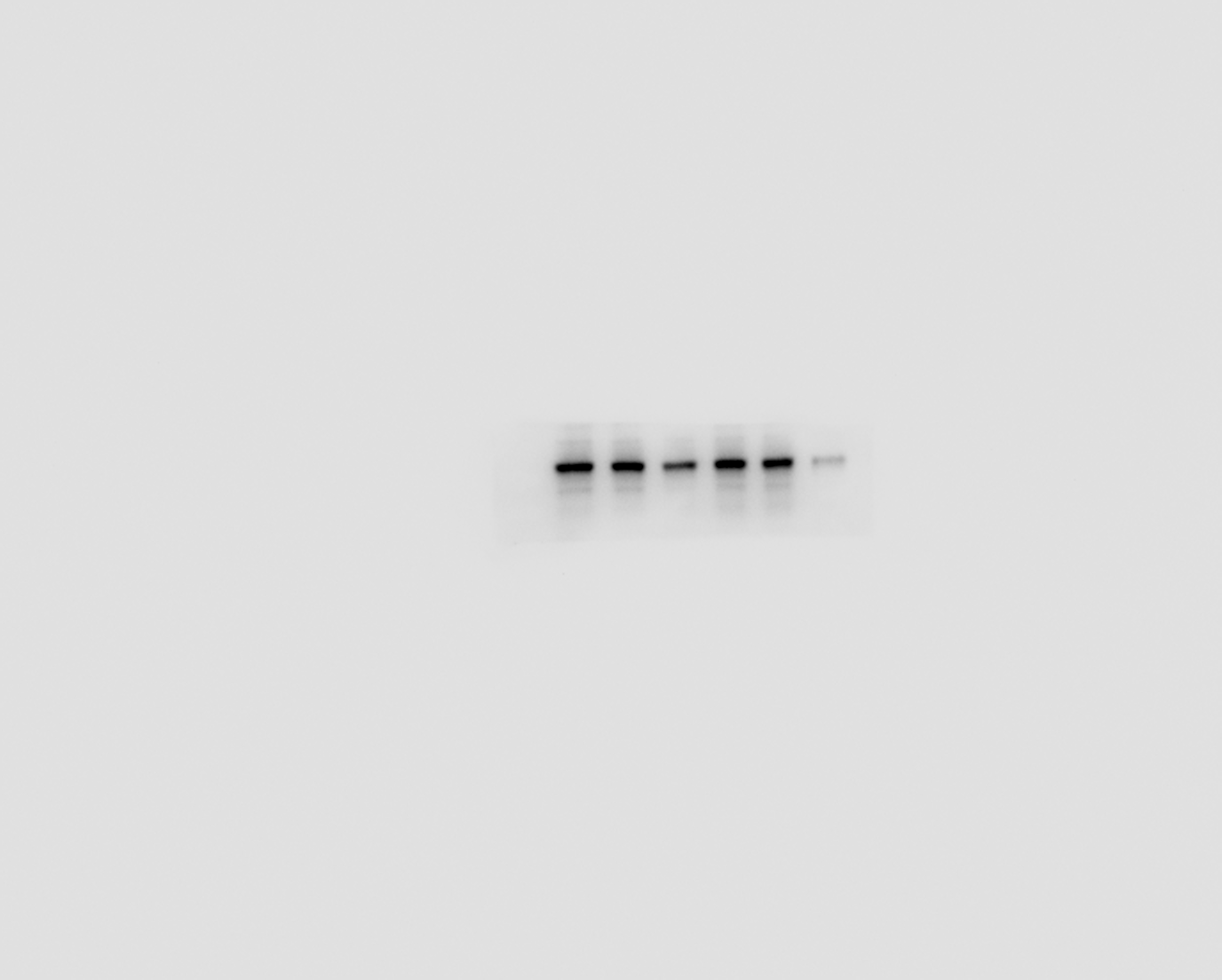

Supplement: Supplementary file 11 — Source data Fig. 6 [file 44318_2024_359_MOESM11_ESM.zip › Figure 6/Fig 6C/IP LAMTOR1/RagA.Tif]

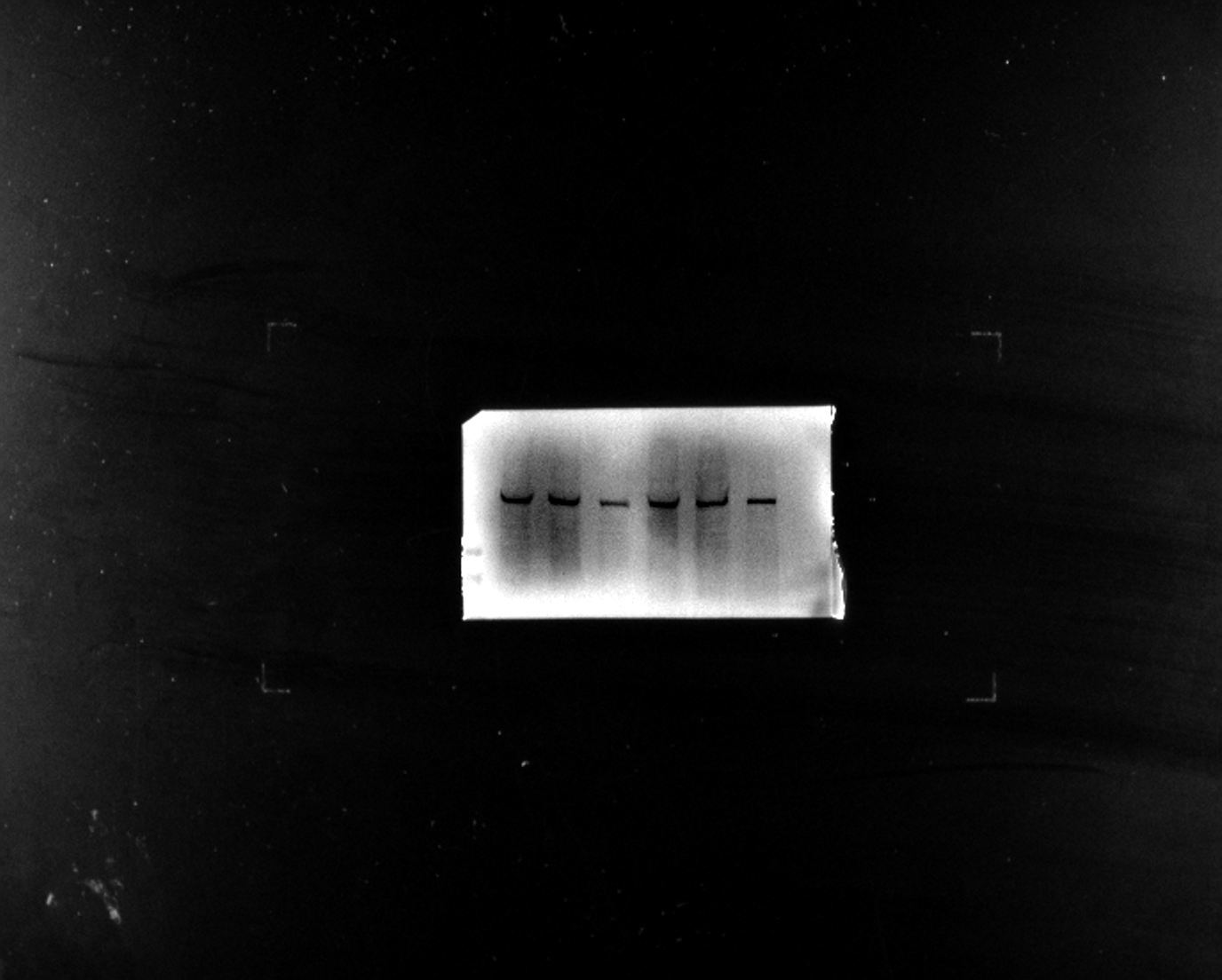

Supplement: Supplementary file 11 — Source data Fig. 6 [file 44318_2024_359_MOESM11_ESM.zip › Figure 6/Fig 6C/IP LAMTOR1/mTOR-merge.Tif]

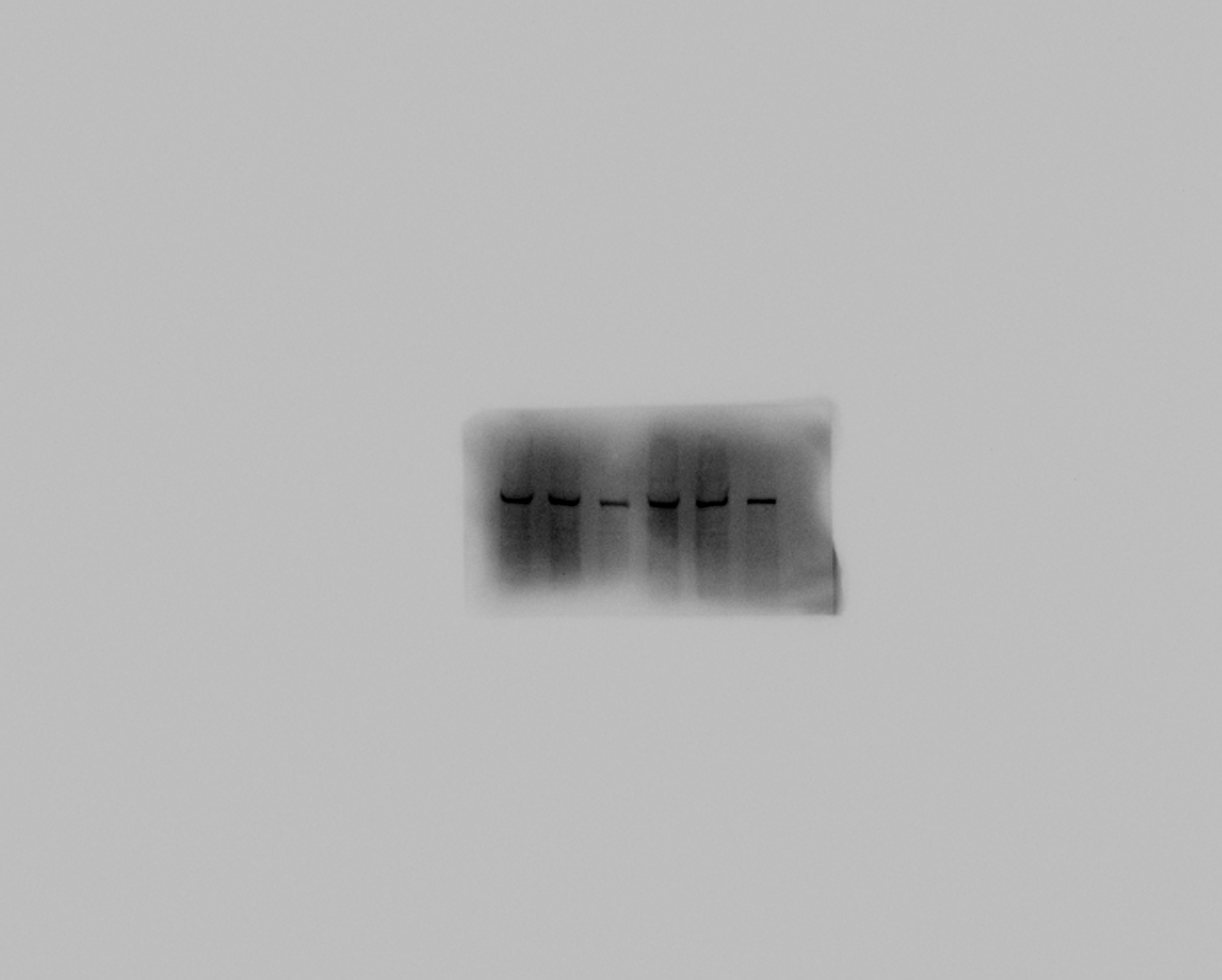

Supplement: Supplementary file 11 — Source data Fig. 6 [file 44318_2024_359_MOESM11_ESM.zip › Figure 6/Fig 6C/IP LAMTOR1/mTOR.Tif]

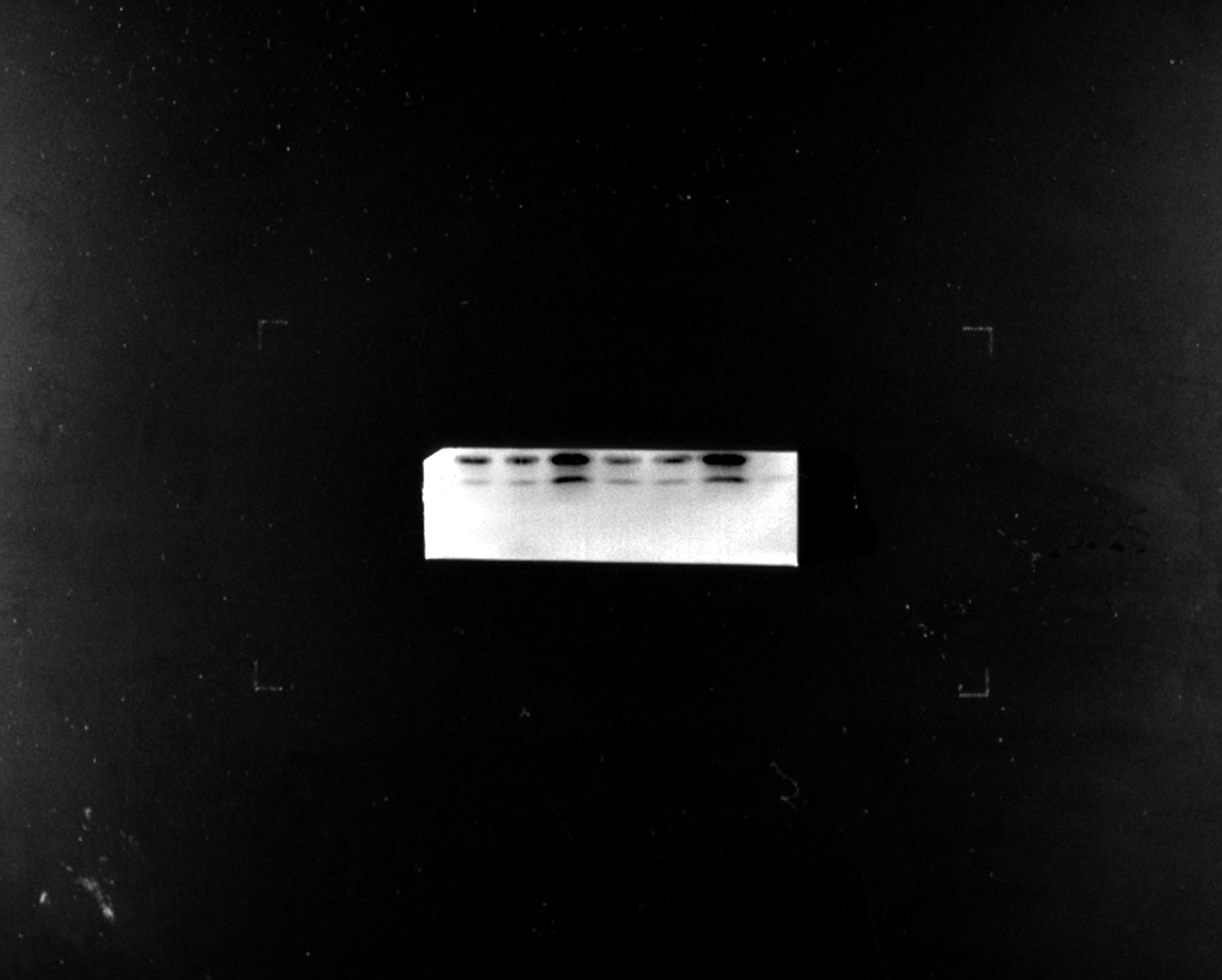

Supplement: Supplementary file 11 — Source data Fig. 6 [file 44318_2024_359_MOESM11_ESM.zip › Figure 6/Fig 6C/IP P27KIP1/LAMTOR1-merge.Tif]

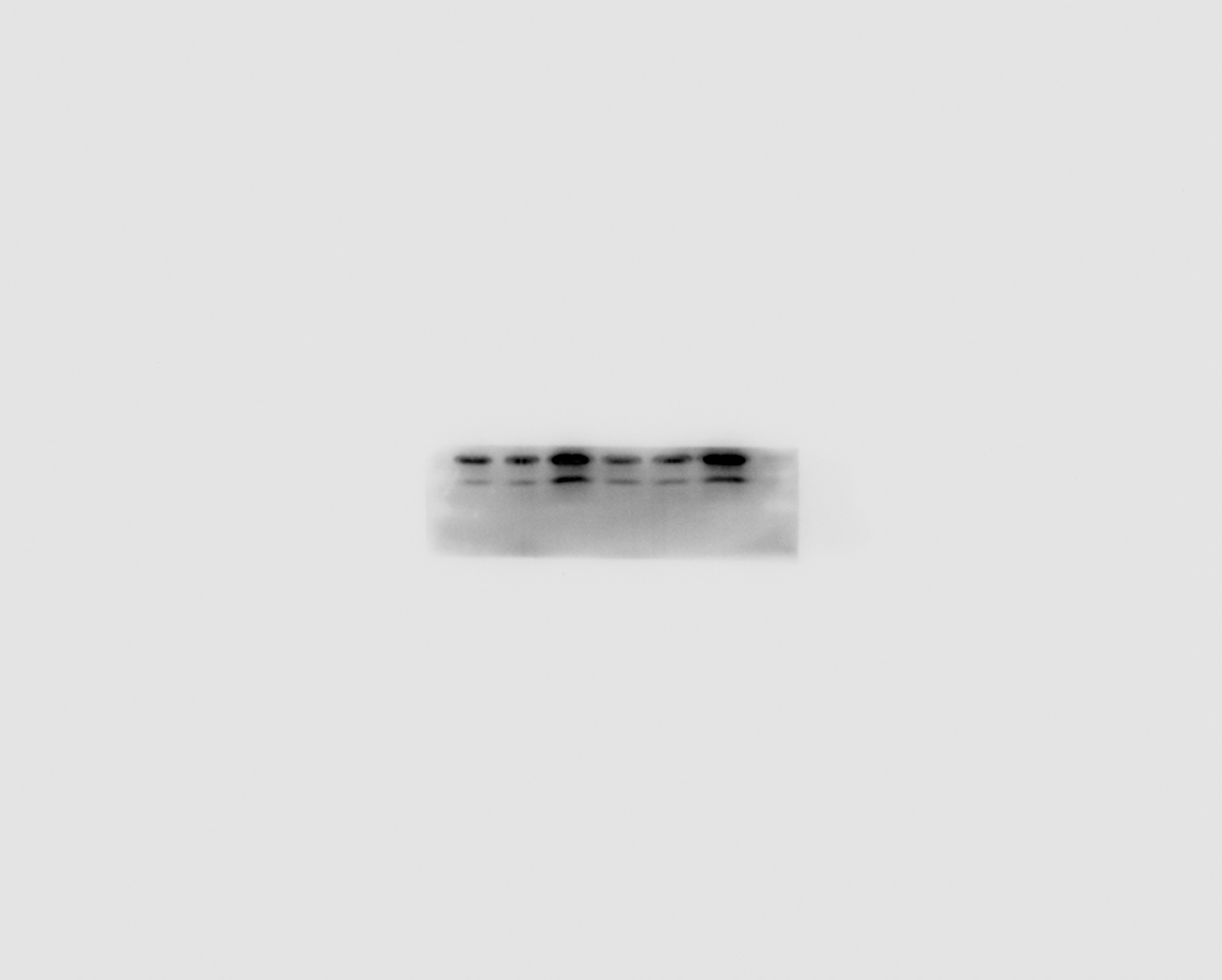

Supplement: Supplementary file 11 — Source data Fig. 6 [file 44318_2024_359_MOESM11_ESM.zip › Figure 6/Fig 6C/IP P27KIP1/LAMTOR1.Tif]

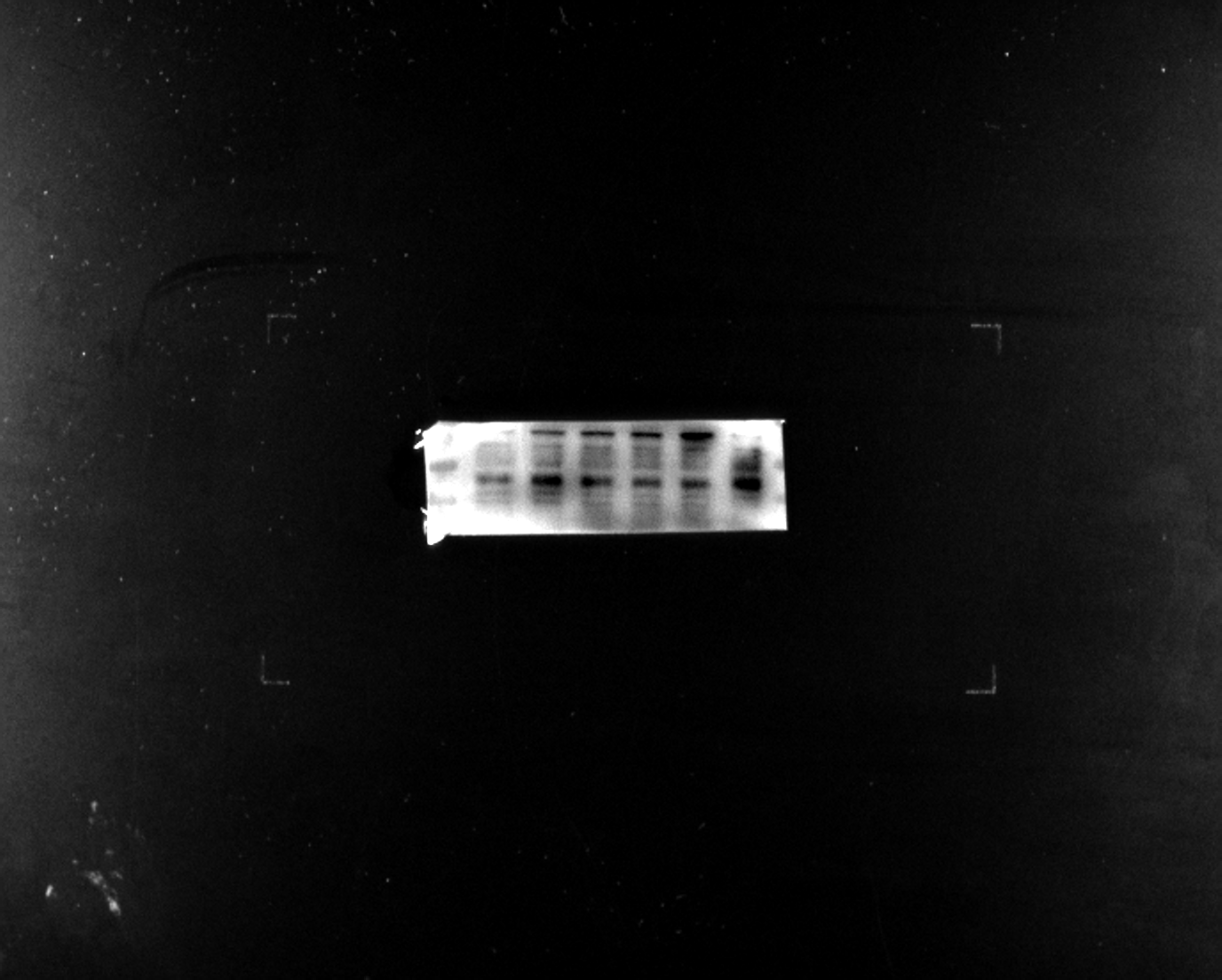

Supplement: Supplementary file 11 — Source data Fig. 6 [file 44318_2024_359_MOESM11_ESM.zip › Figure 6/Fig 6C/IP P27KIP1/P27KIP1-merge.Tif]

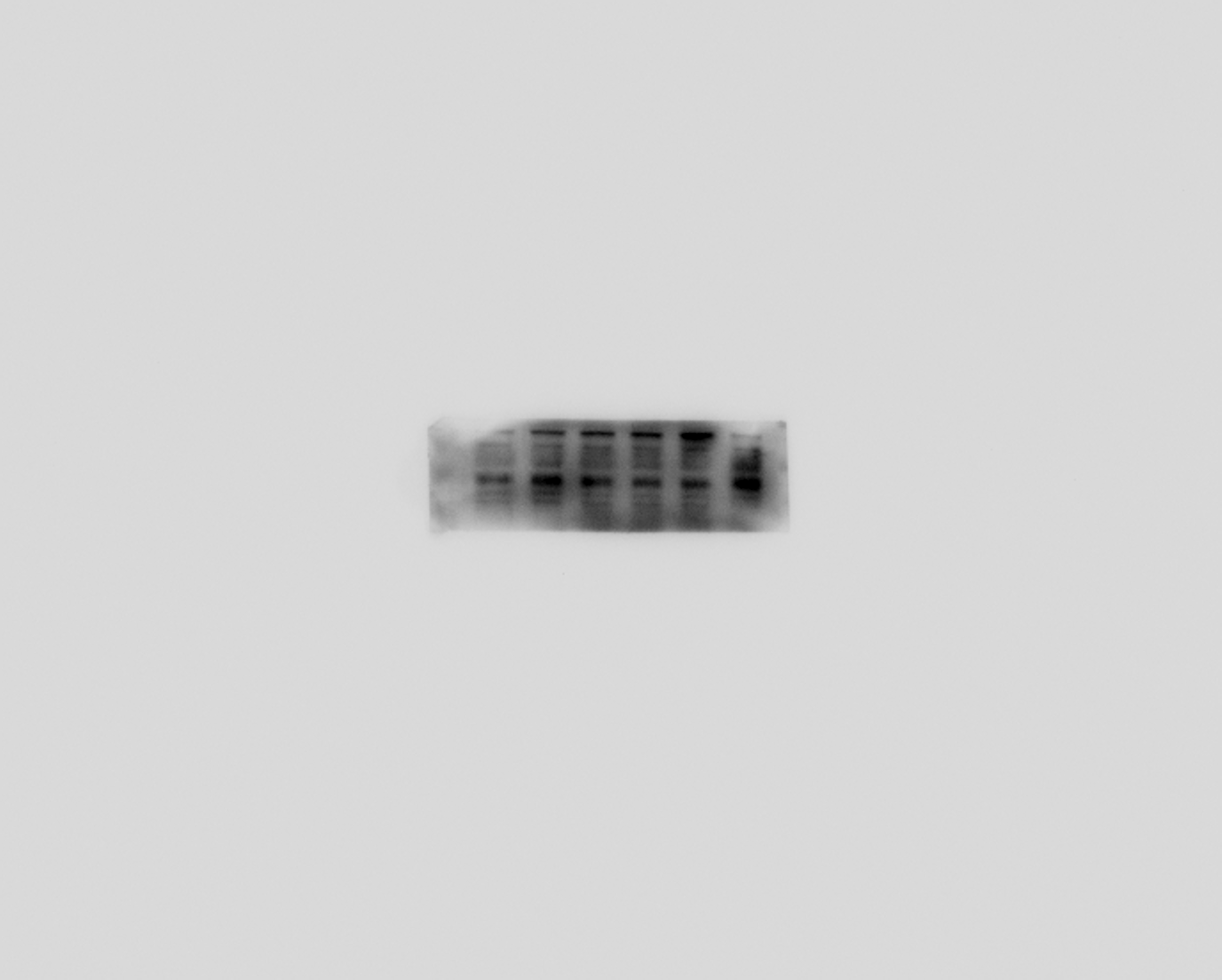

Supplement: Supplementary file 11 — Source data Fig. 6 [file 44318_2024_359_MOESM11_ESM.zip › Figure 6/Fig 6C/IP P27KIP1/P27KIP1.Tif]

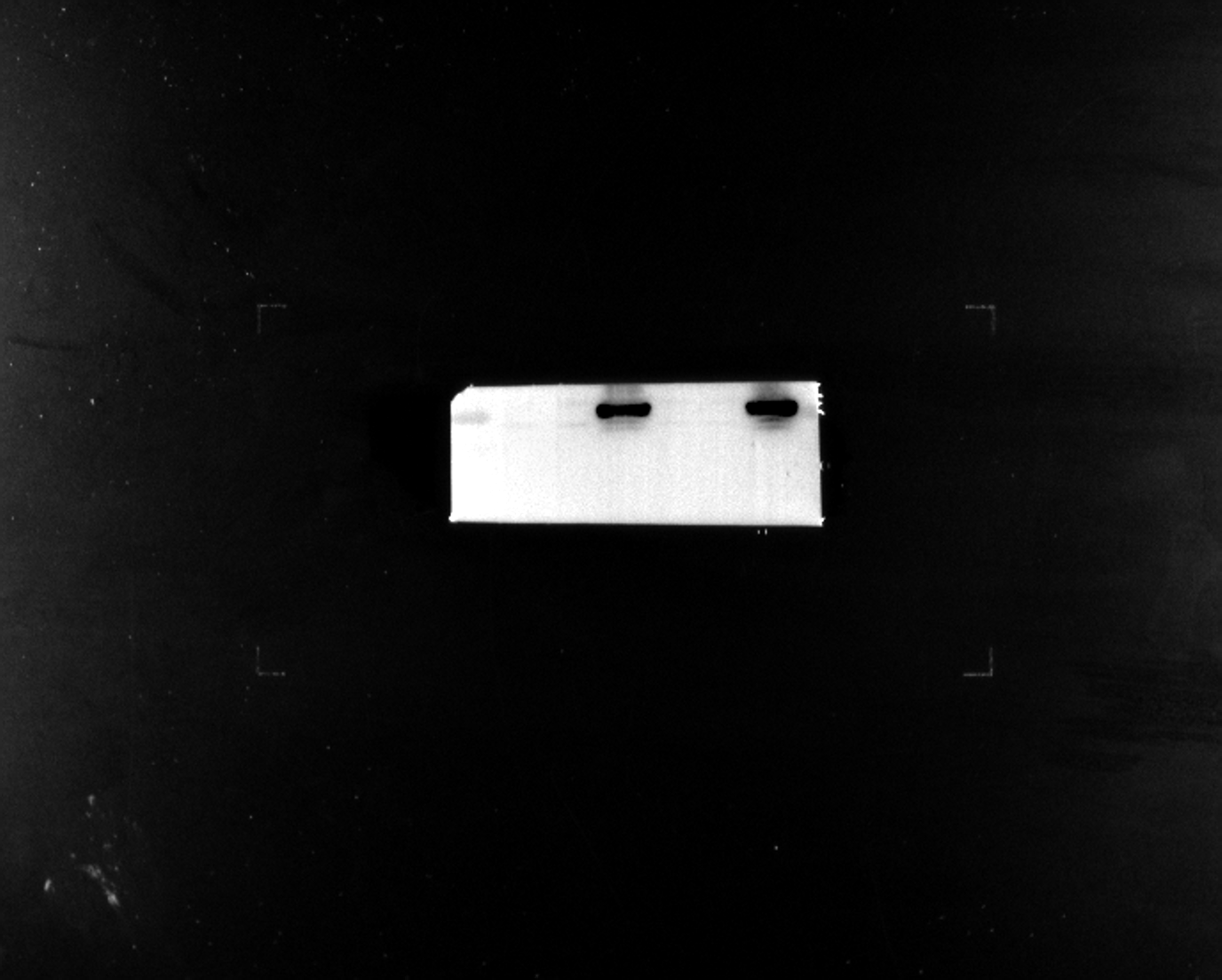

Supplement: Supplementary file 11 — Source data Fig. 6 [file 44318_2024_359_MOESM11_ESM.zip › Figure 6/Fig 6C/Input/Flag-merge.Tif]

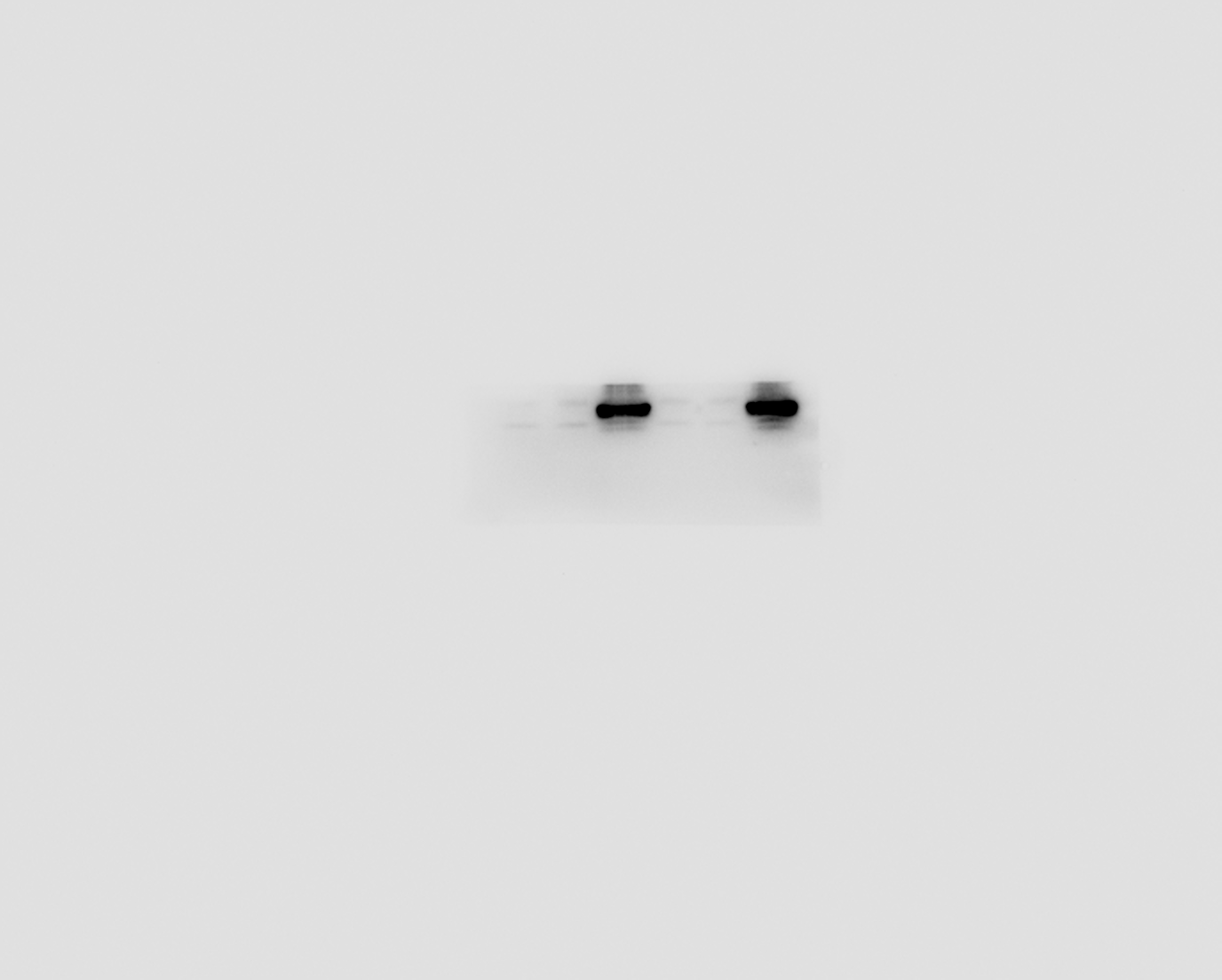

Supplement: Supplementary file 11 — Source data Fig. 6 [file 44318_2024_359_MOESM11_ESM.zip › Figure 6/Fig 6C/Input/Flag.Tif]

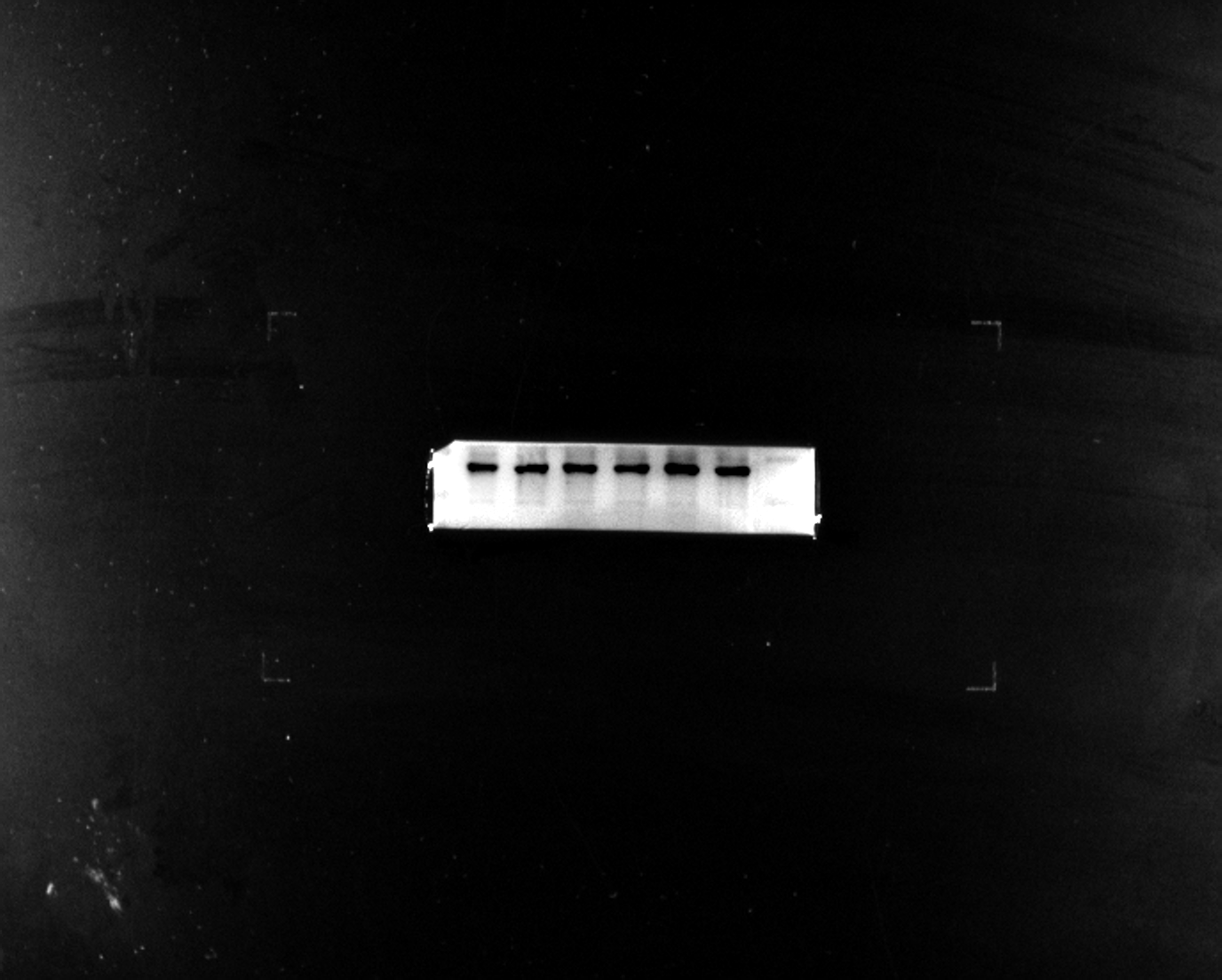

Supplement: Supplementary file 11 — Source data Fig. 6 [file 44318_2024_359_MOESM11_ESM.zip › Figure 6/Fig 6C/Input/GAPDH-merge.Tif]

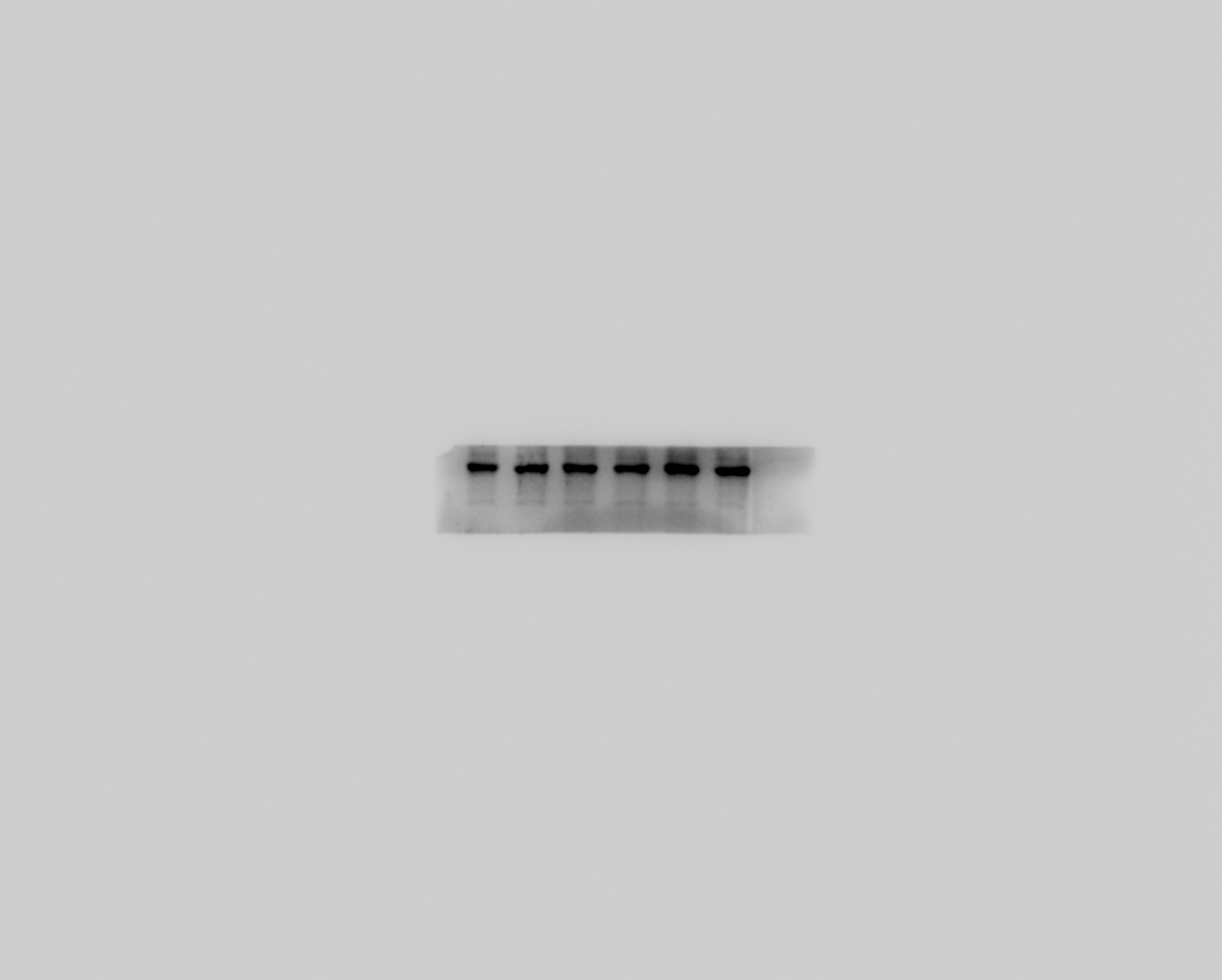

Supplement: Supplementary file 11 — Source data Fig. 6 [file 44318_2024_359_MOESM11_ESM.zip › Figure 6/Fig 6C/Input/GAPDH.Tif]

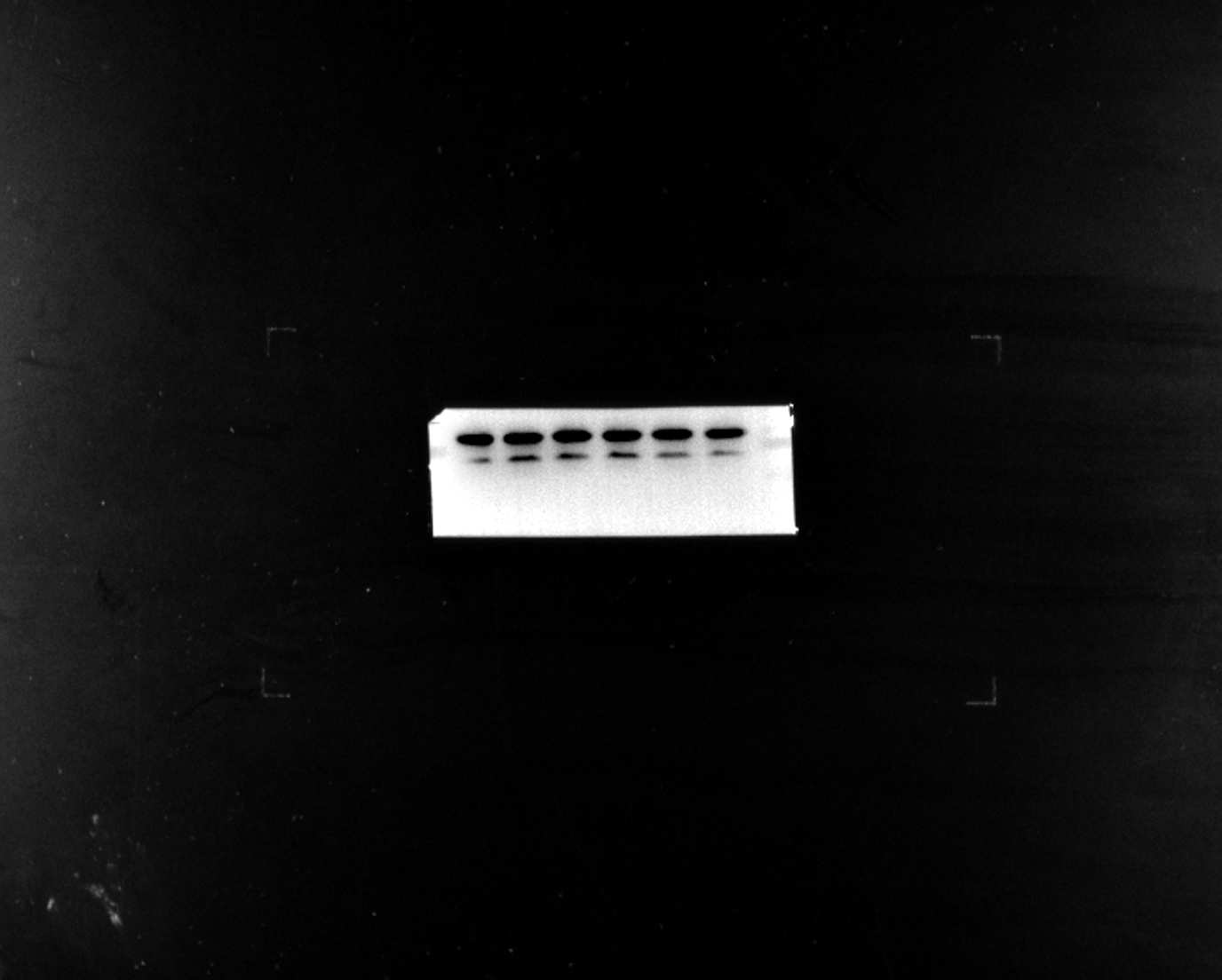

Supplement: Supplementary file 11 — Source data Fig. 6 [file 44318_2024_359_MOESM11_ESM.zip › Figure 6/Fig 6C/Input/L1-merge.Tif]

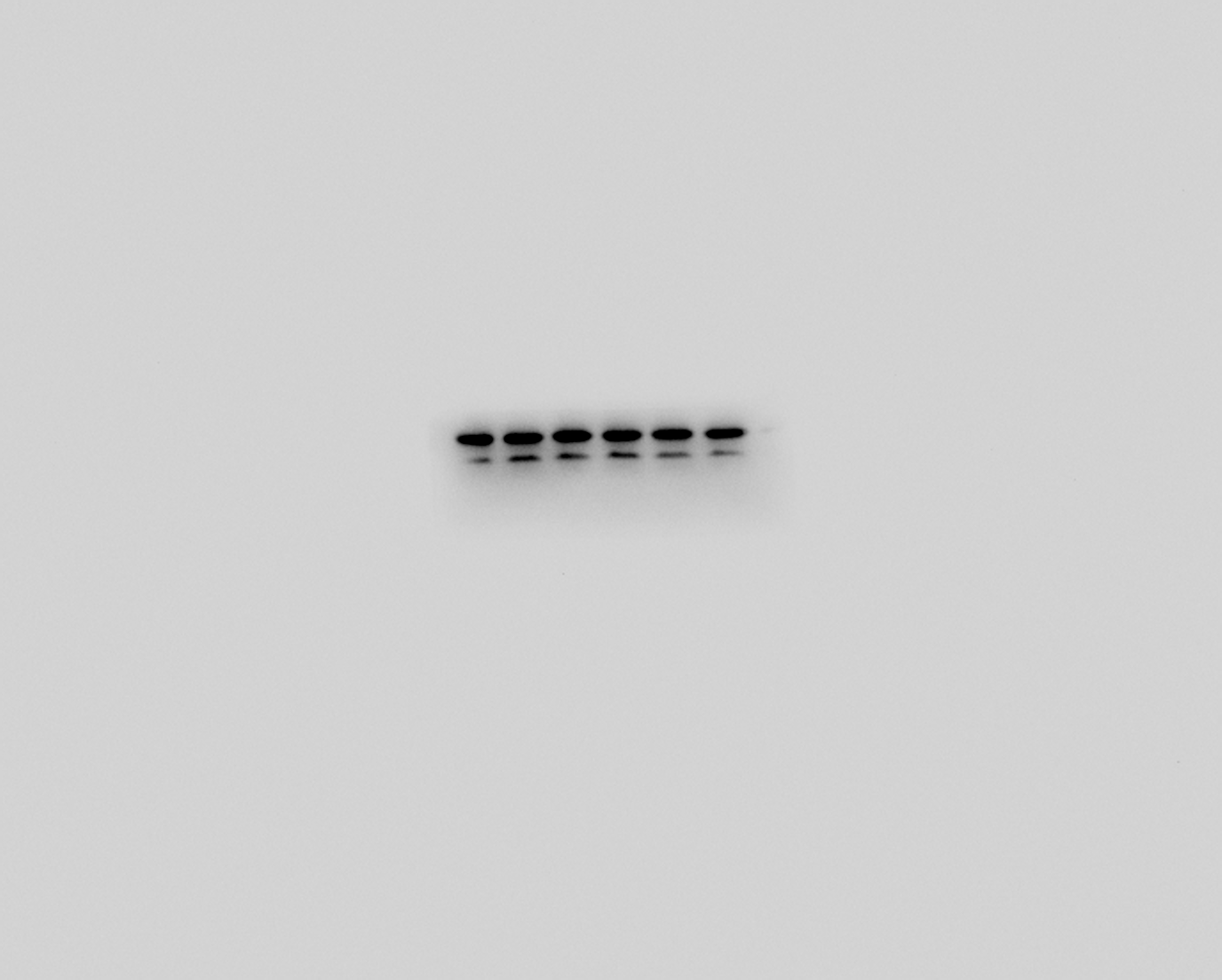

Supplement: Supplementary file 11 — Source data Fig. 6 [file 44318_2024_359_MOESM11_ESM.zip › Figure 6/Fig 6C/Input/L1.Tif]

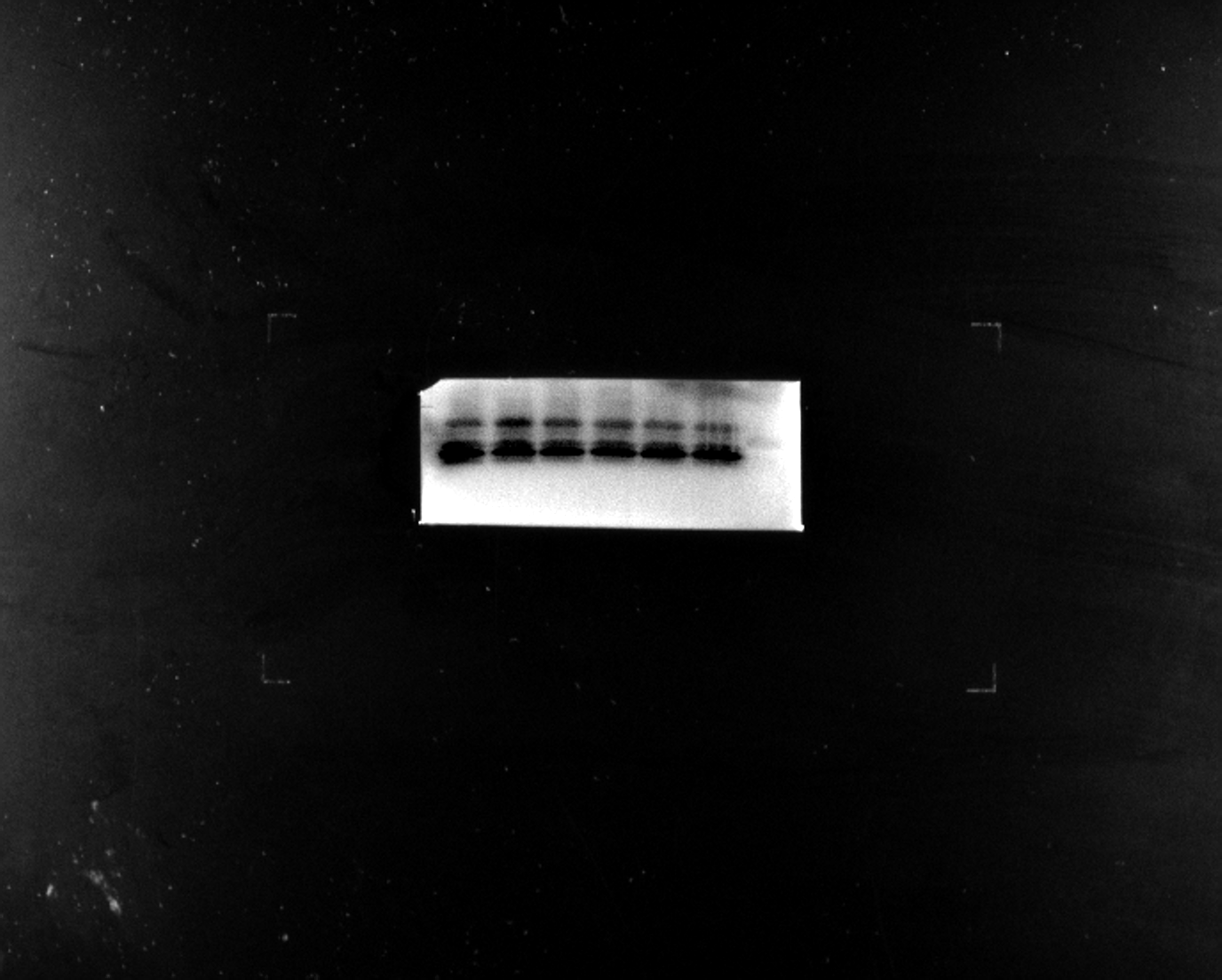

Supplement: Supplementary file 11 — Source data Fig. 6 [file 44318_2024_359_MOESM11_ESM.zip › Figure 6/Fig 6C/Input/L2+L5-merge.Tif]

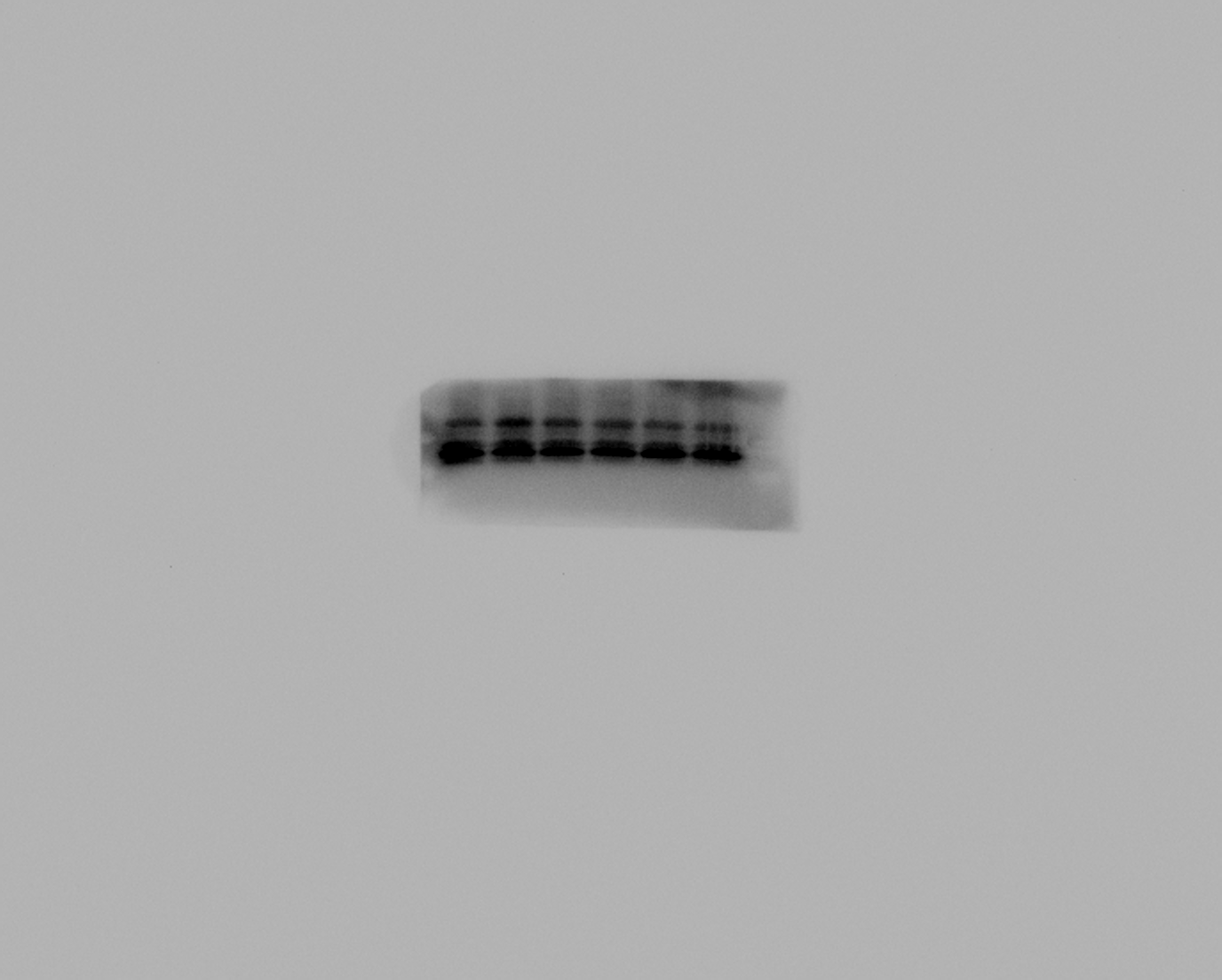

Supplement: Supplementary file 11 — Source data Fig. 6 [file 44318_2024_359_MOESM11_ESM.zip › Figure 6/Fig 6C/Input/L2+L5.Tif]

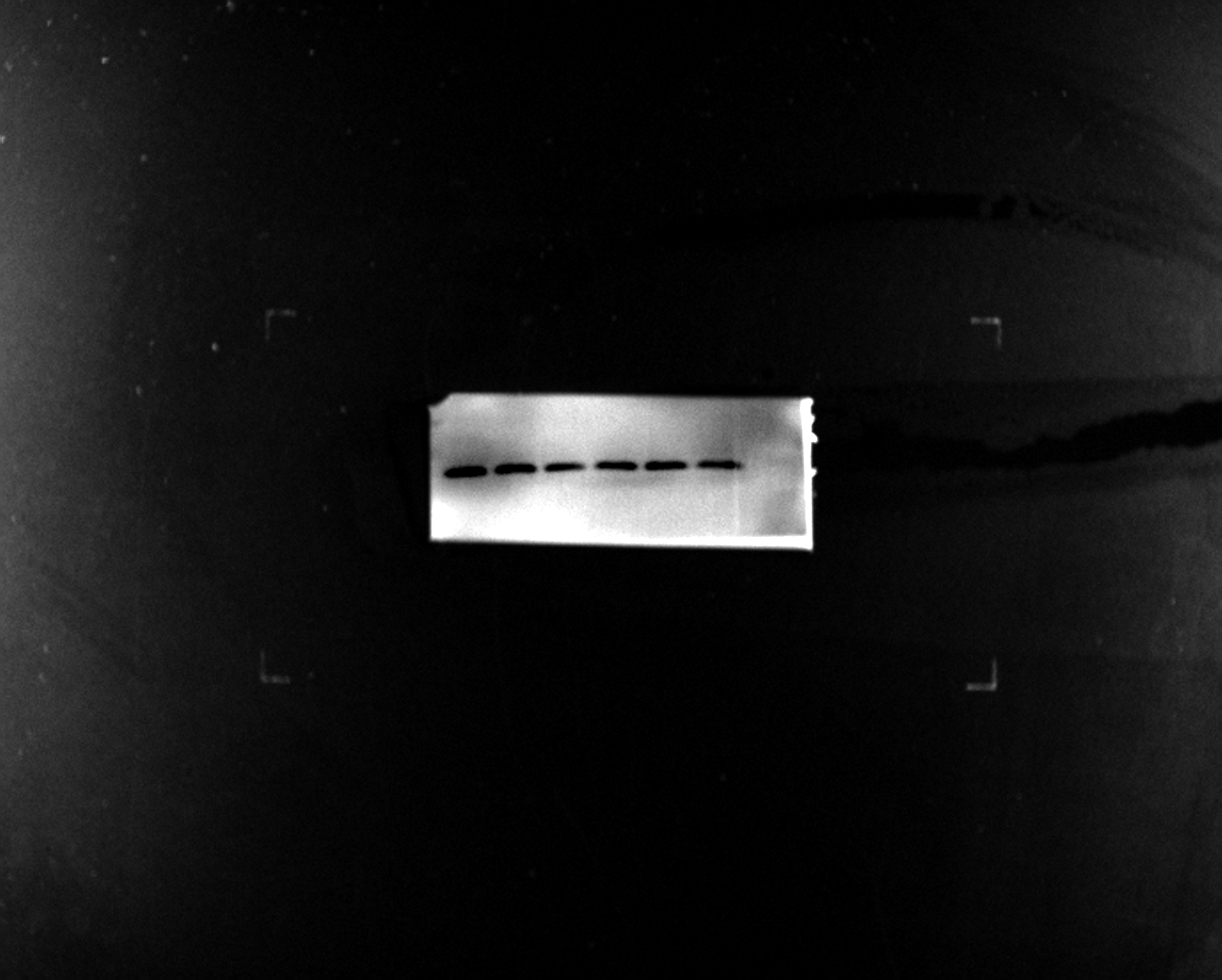

Supplement: Supplementary file 11 — Source data Fig. 6 [file 44318_2024_359_MOESM11_ESM.zip › Figure 6/Fig 6C/Input/L3-merge.Tif]

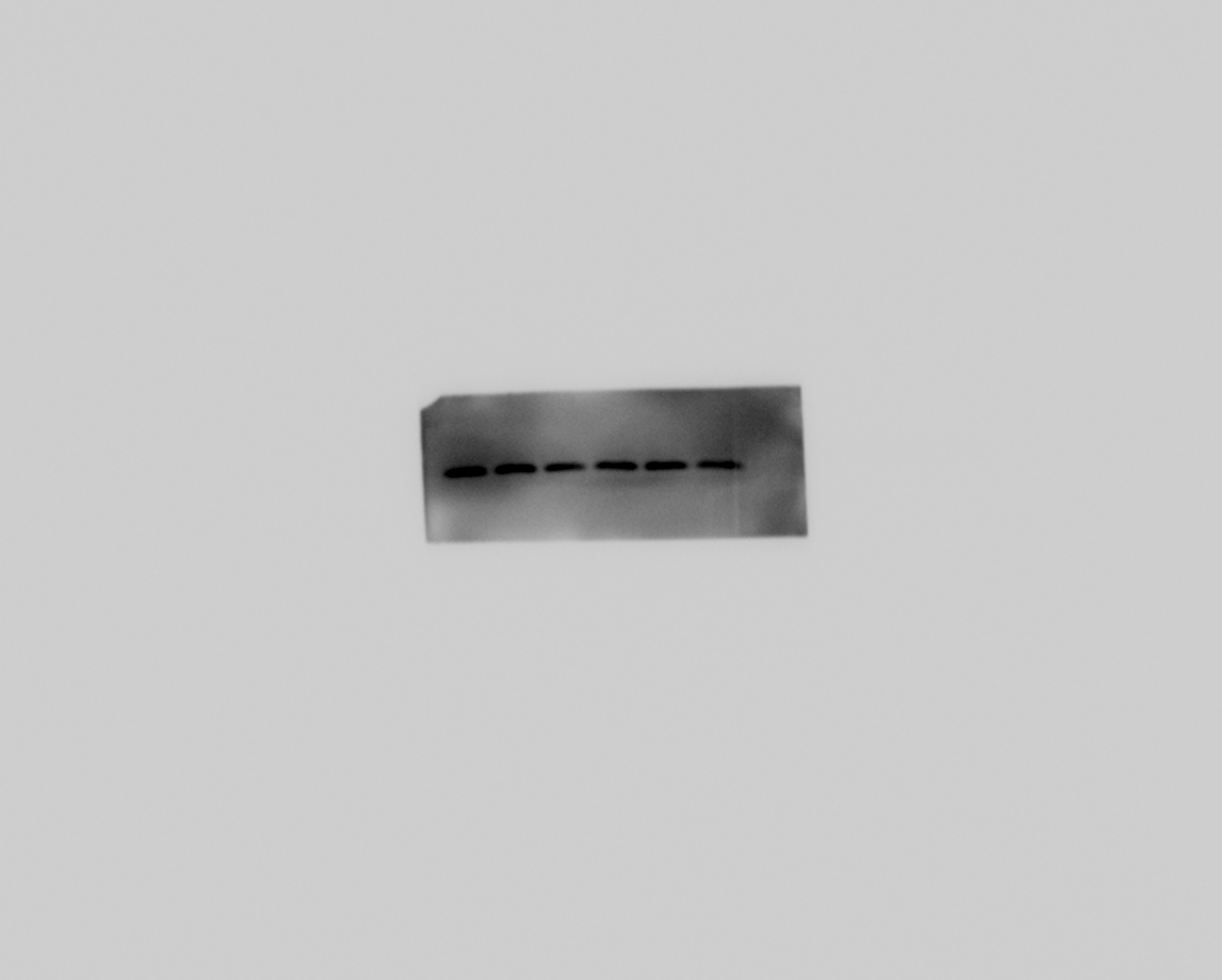

Supplement: Supplementary file 11 — Source data Fig. 6 [file 44318_2024_359_MOESM11_ESM.zip › Figure 6/Fig 6C/Input/L3.Tif]

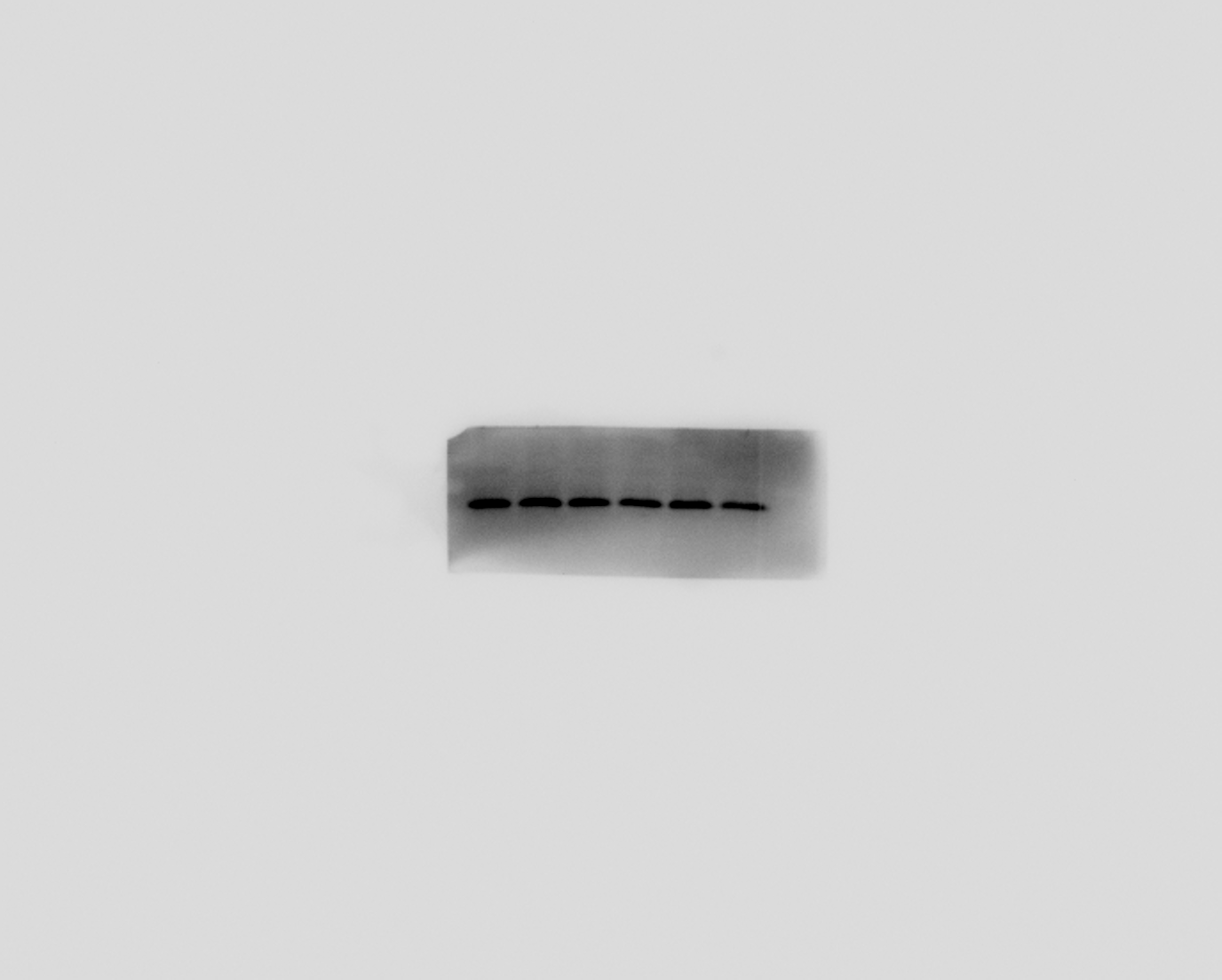

Supplement: Supplementary file 11 — Source data Fig. 6 [file 44318_2024_359_MOESM11_ESM.zip › Figure 6/Fig 6C/Input/L4.Tif]

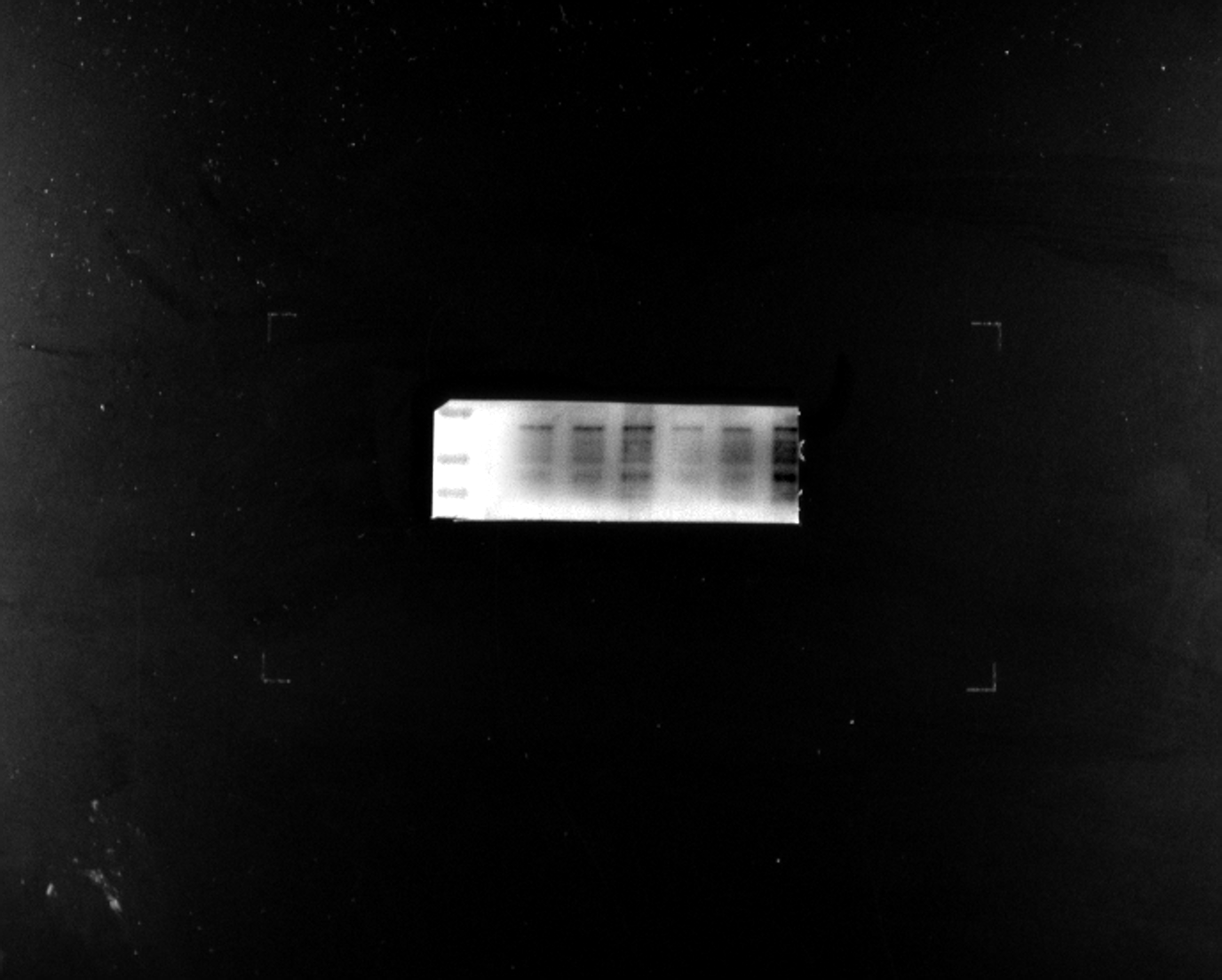

Supplement: Supplementary file 11 — Source data Fig. 6 [file 44318_2024_359_MOESM11_ESM.zip › Figure 6/Fig 6C/Input/P27KIP1-merge.Tif]

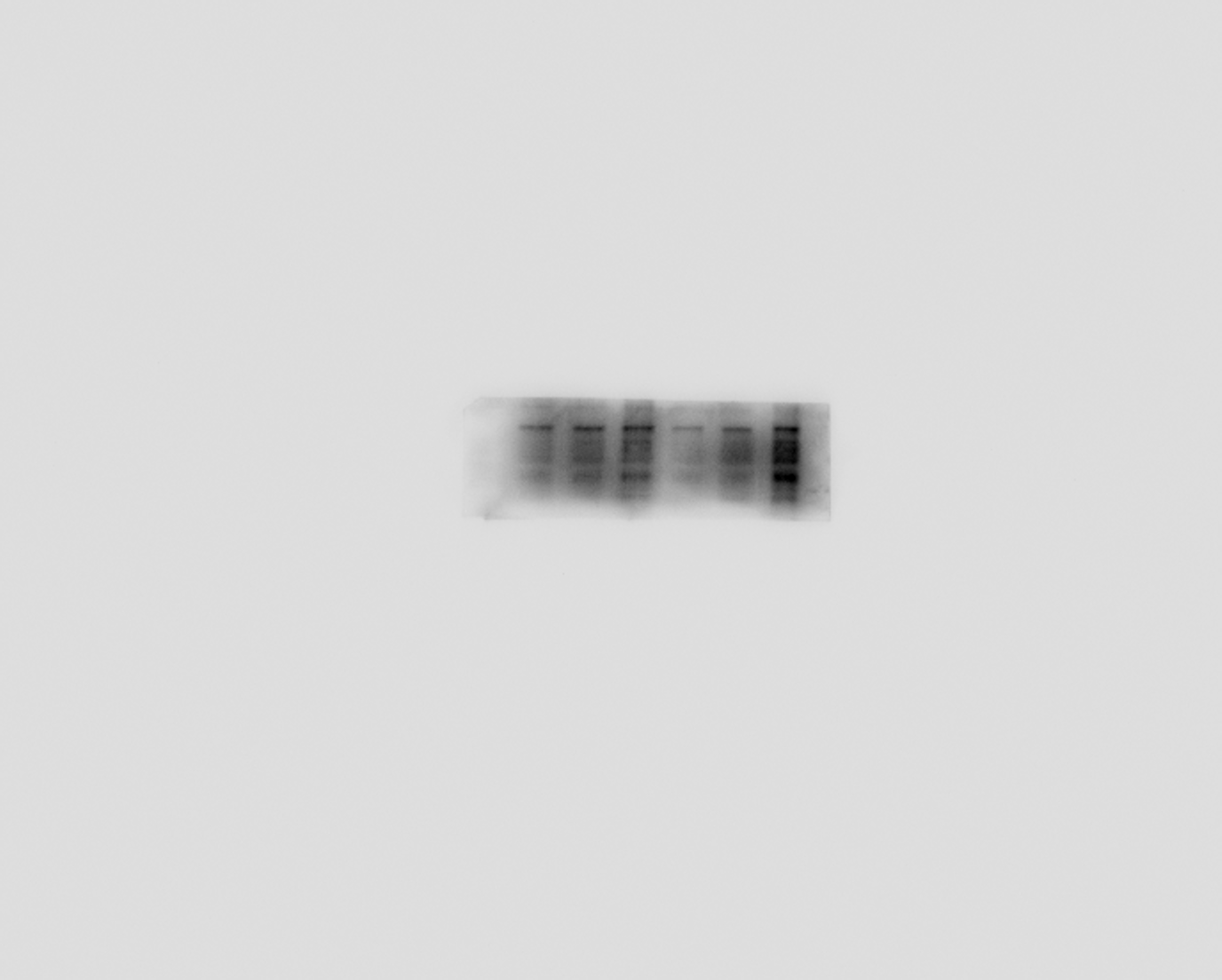

Supplement: Supplementary file 11 — Source data Fig. 6 [file 44318_2024_359_MOESM11_ESM.zip › Figure 6/Fig 6C/Input/P27KIP1.Tif]

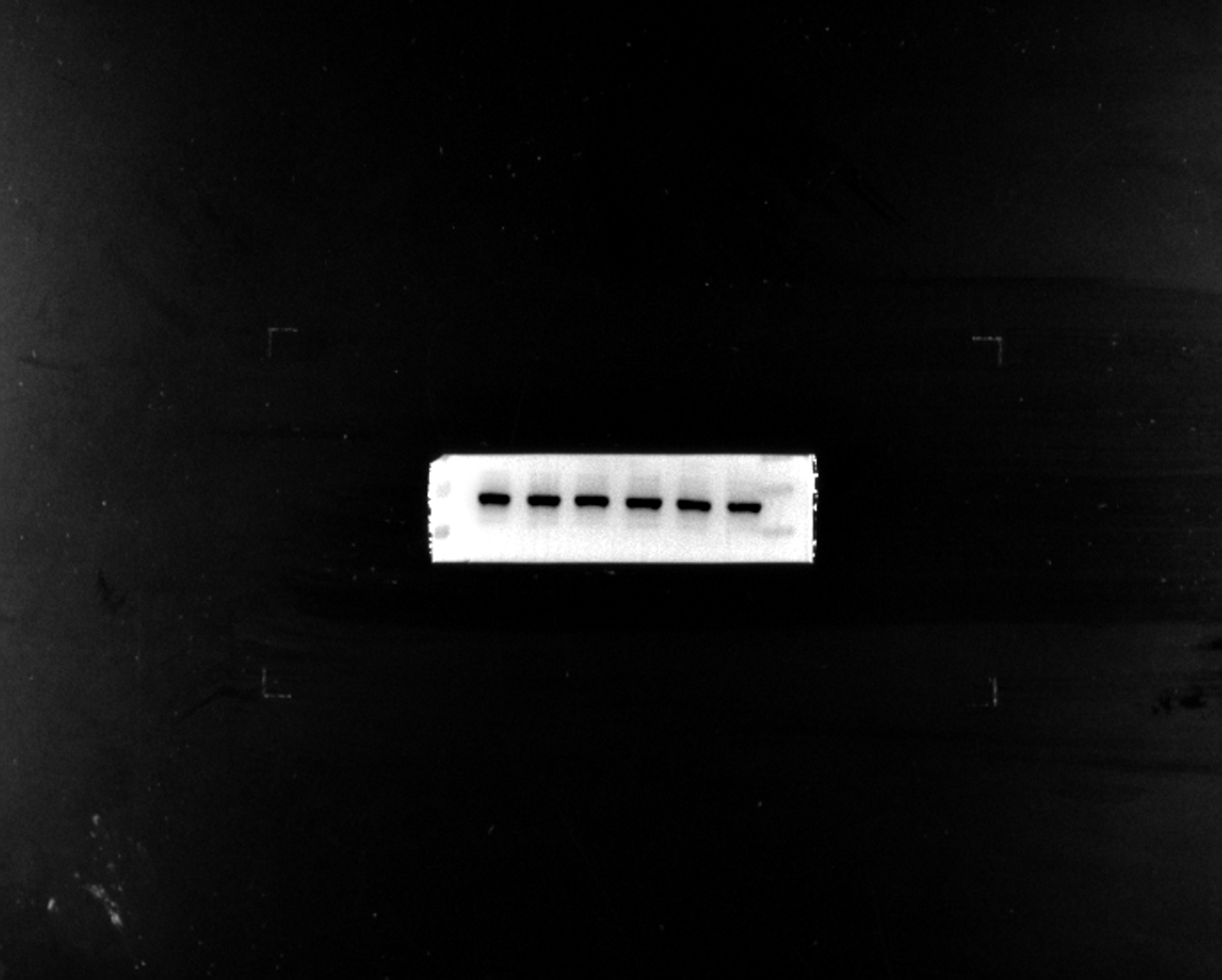

Supplement: Supplementary file 11 — Source data Fig. 6 [file 44318_2024_359_MOESM11_ESM.zip › Figure 6/Fig 6C/Input/RagA-merge.Tif]

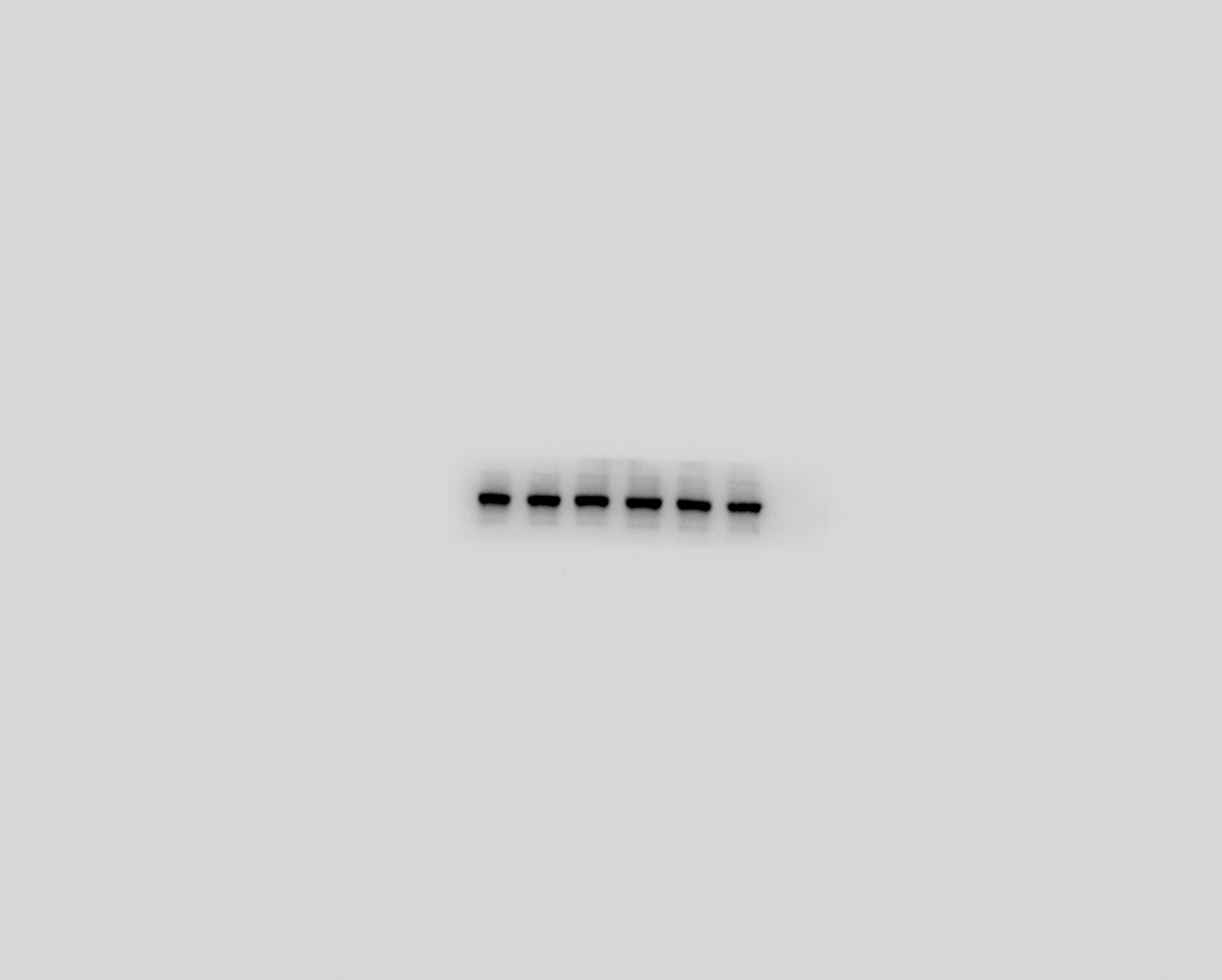

Supplement: Supplementary file 11 — Source data Fig. 6 [file 44318_2024_359_MOESM11_ESM.zip › Figure 6/Fig 6C/Input/RagA.Tif]

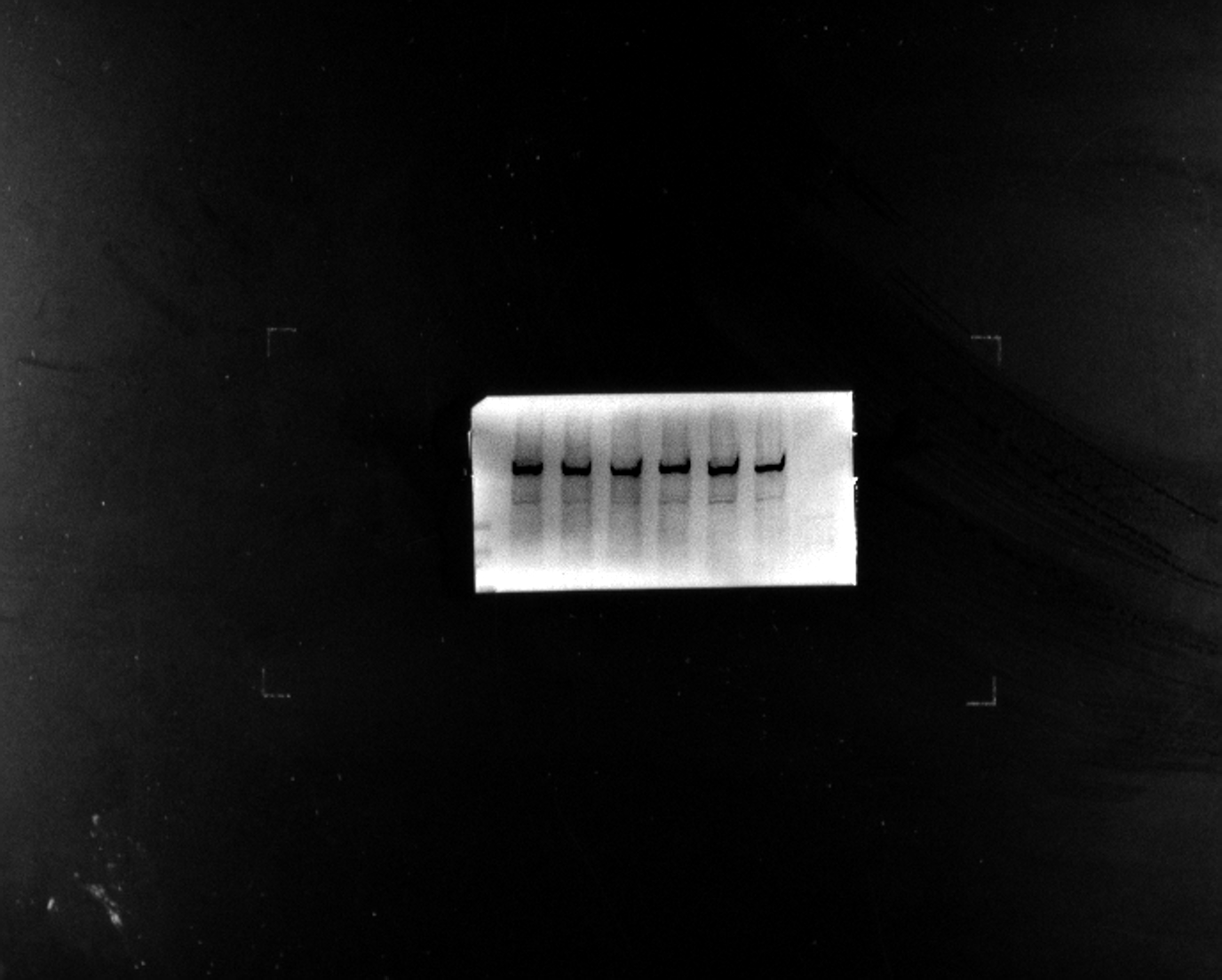

Supplement: Supplementary file 11 — Source data Fig. 6 [file 44318_2024_359_MOESM11_ESM.zip › Figure 6/Fig 6C/Input/mTOR-merge.Tif]

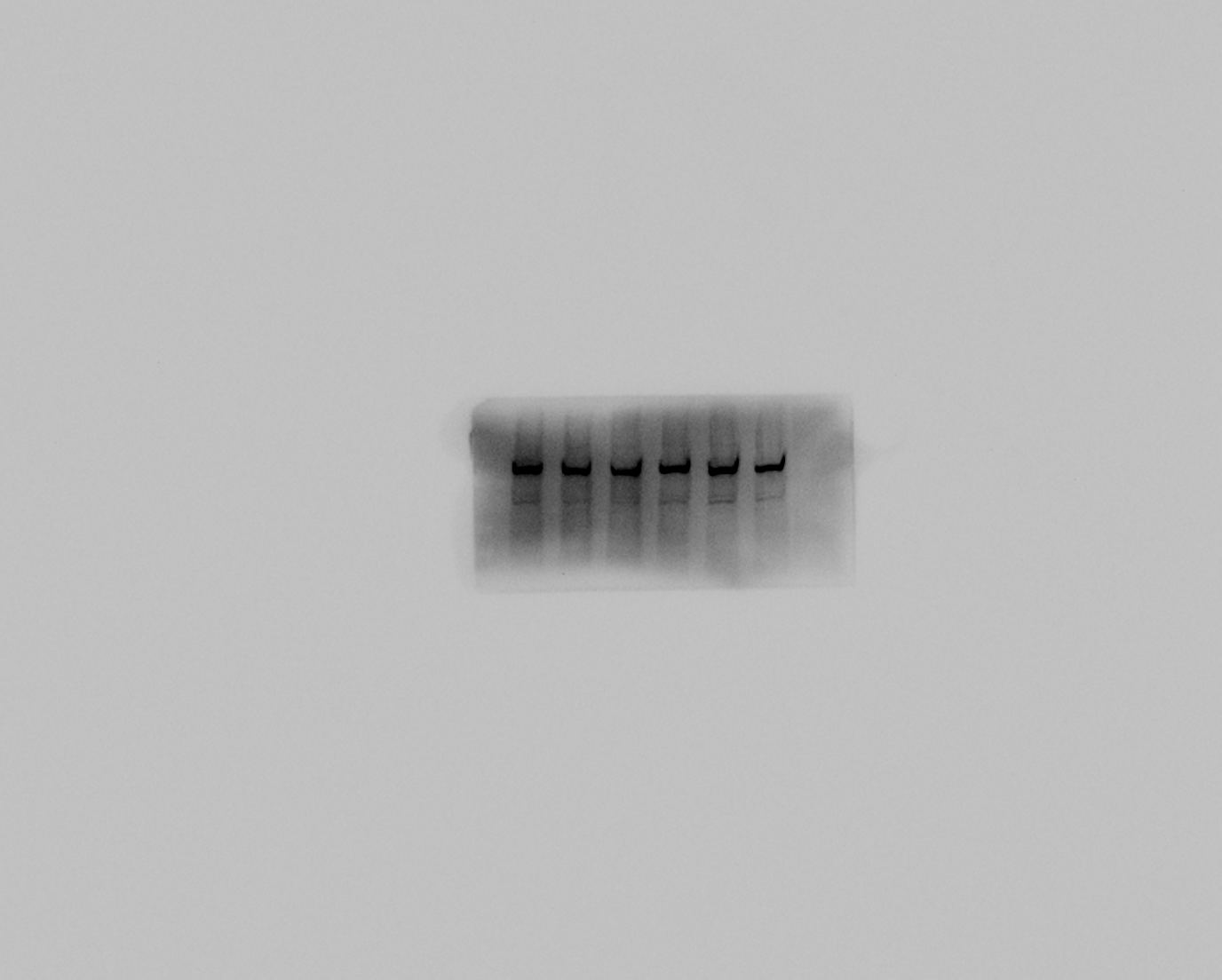

Supplement: Supplementary file 11 — Source data Fig. 6 [file 44318_2024_359_MOESM11_ESM.zip › Figure 6/Fig 6C/Input/mTOR.Tif]

Fig 6D

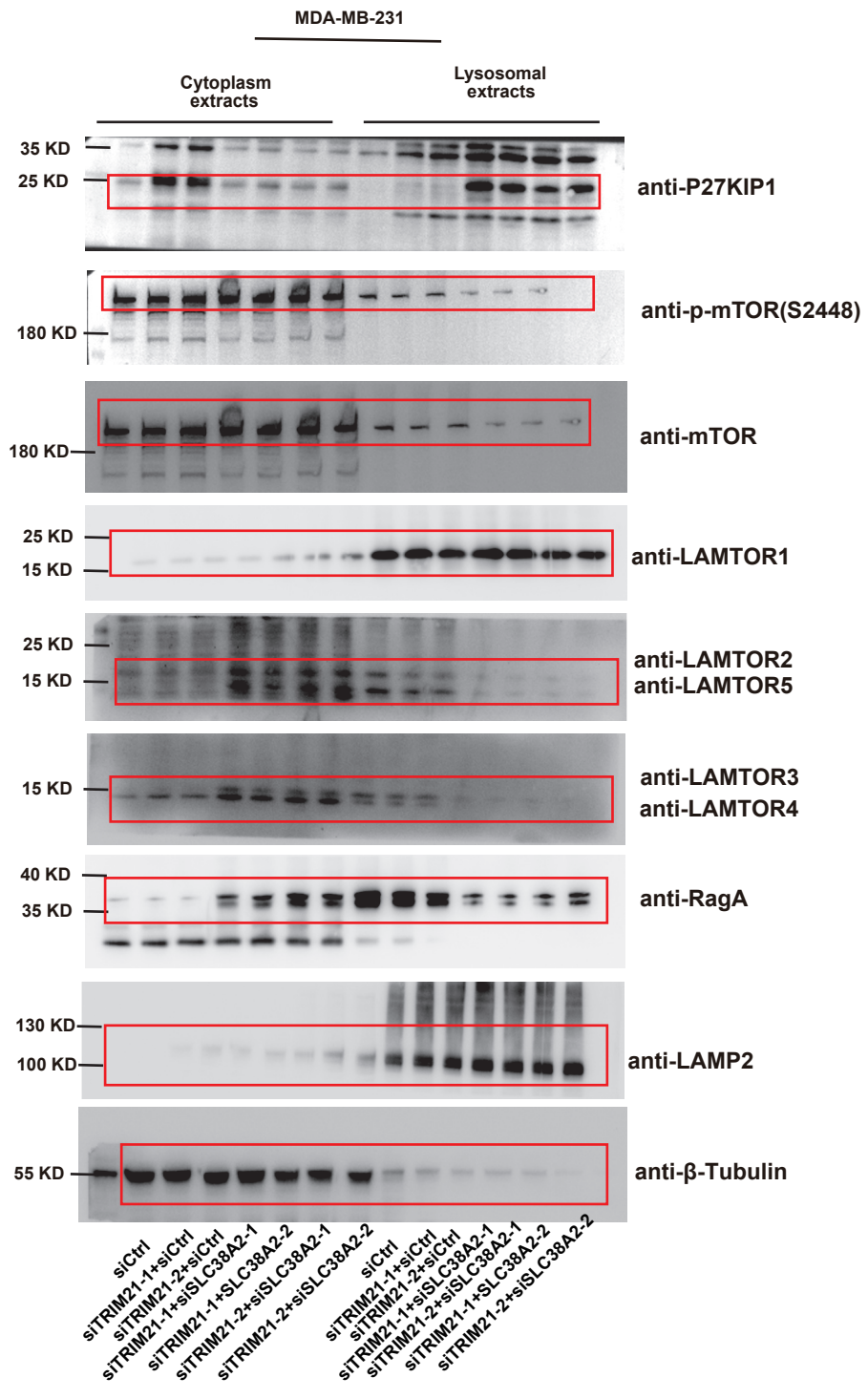

Supplement: Supplementary file 11 — Source data Fig. 6 [file 44318_2024_359_MOESM11_ESM.zip › Figure 6/Fig 6D and 6E/Fig 6D/Fig 6D.pdf]

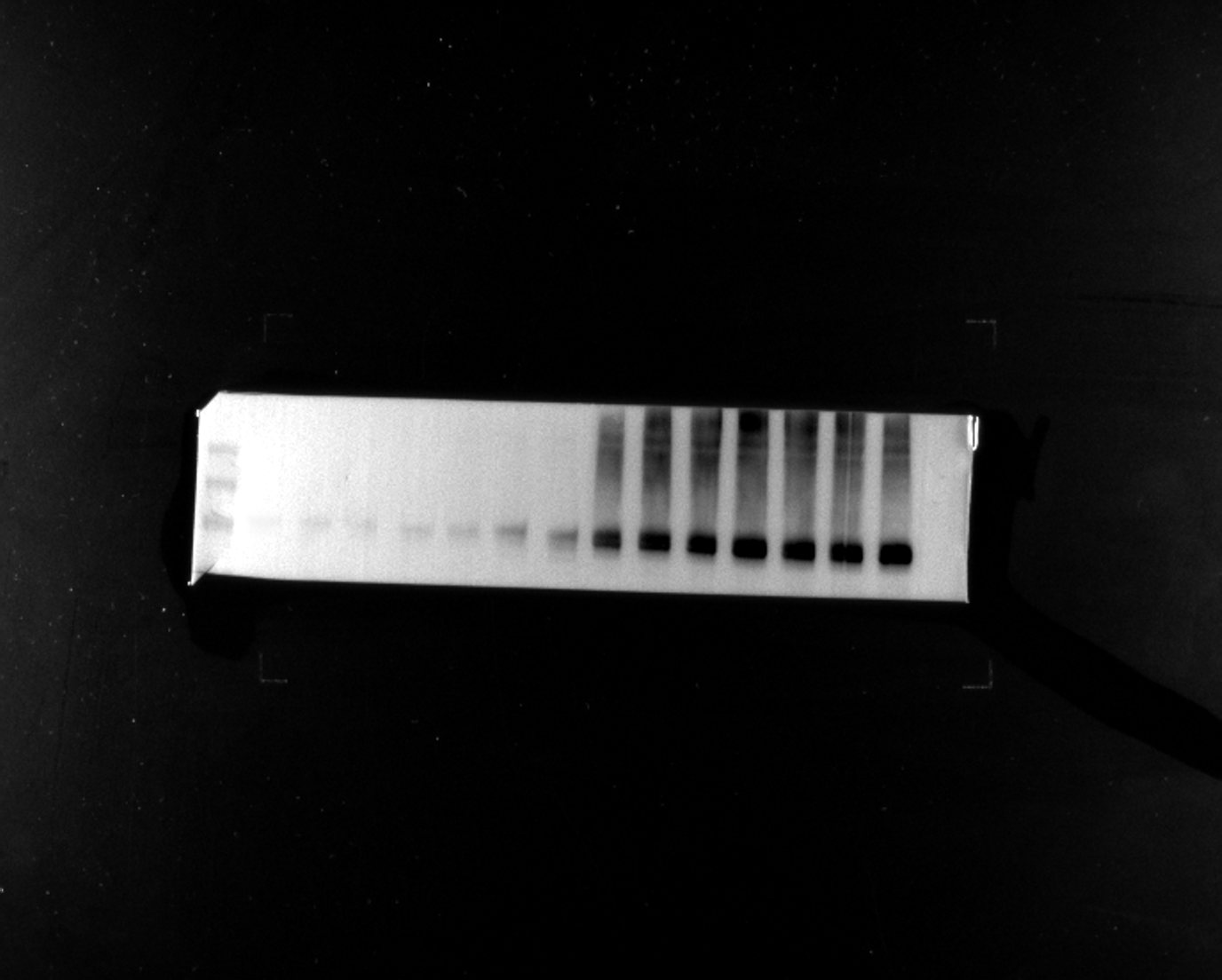

Supplement: Supplementary file 11 — Source data Fig. 6 [file 44318_2024_359_MOESM11_ESM.zip › Figure 6/Fig 6D and 6E/Fig 6D/LAMP2-merge.Tif]

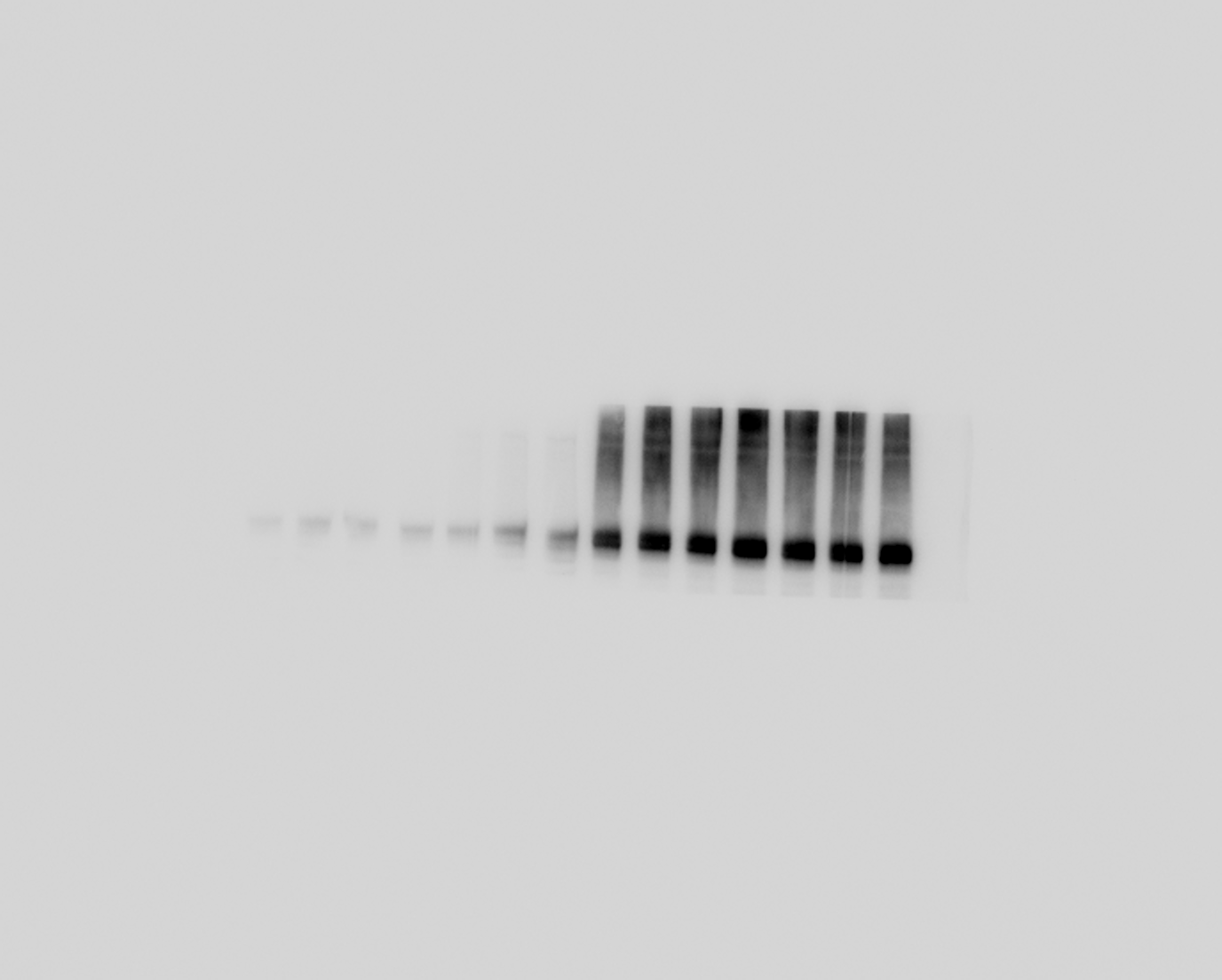

Supplement: Supplementary file 11 — Source data Fig. 6 [file 44318_2024_359_MOESM11_ESM.zip › Figure 6/Fig 6D and 6E/Fig 6D/LAMP2.Tif]

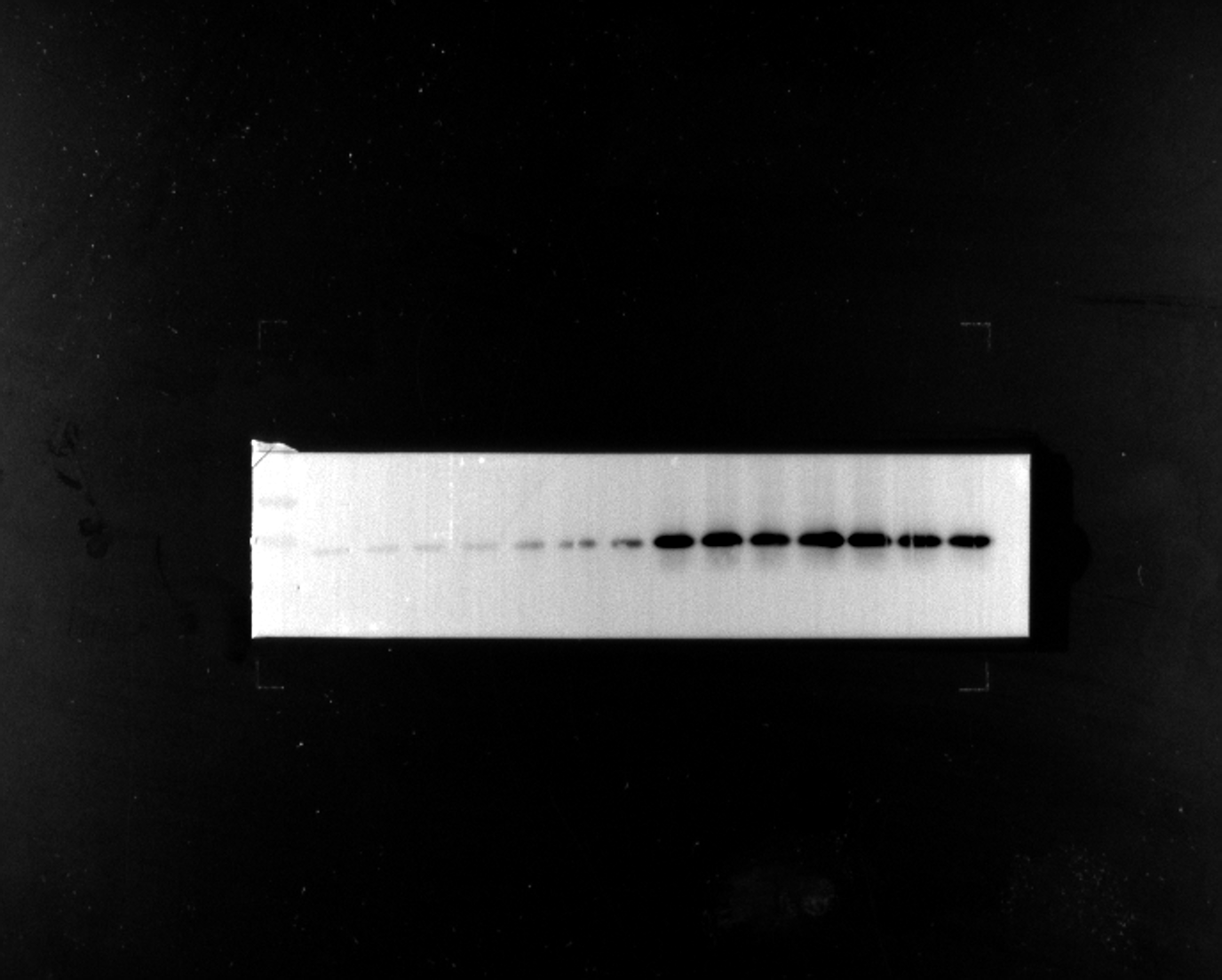

Supplement: Supplementary file 11 — Source data Fig. 6 [file 44318_2024_359_MOESM11_ESM.zip › Figure 6/Fig 6D and 6E/Fig 6D/LAMTOR1-merge.Tif]

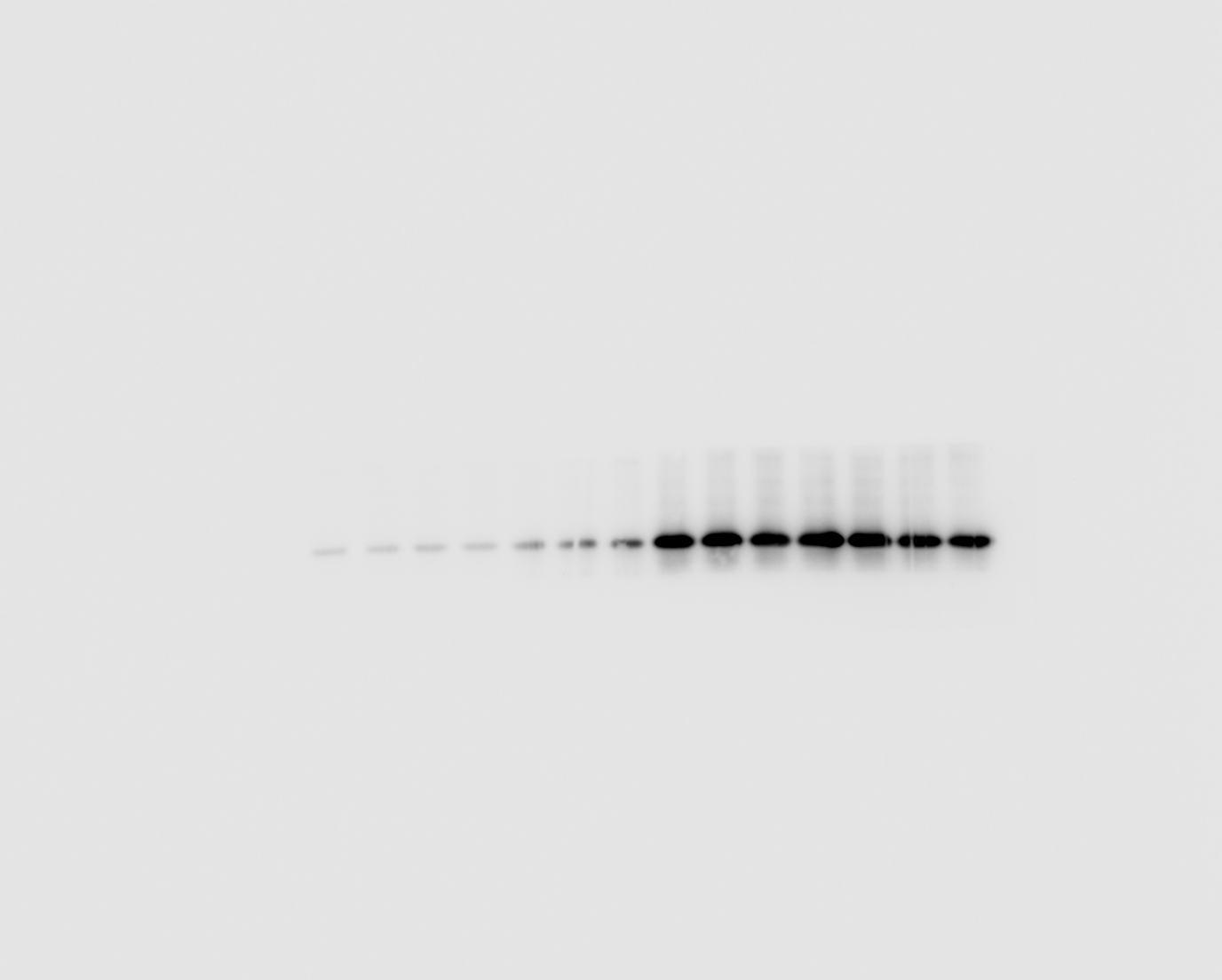

Supplement: Supplementary file 11 — Source data Fig. 6 [file 44318_2024_359_MOESM11_ESM.zip › Figure 6/Fig 6D and 6E/Fig 6D/LAMTOR1.Tif]

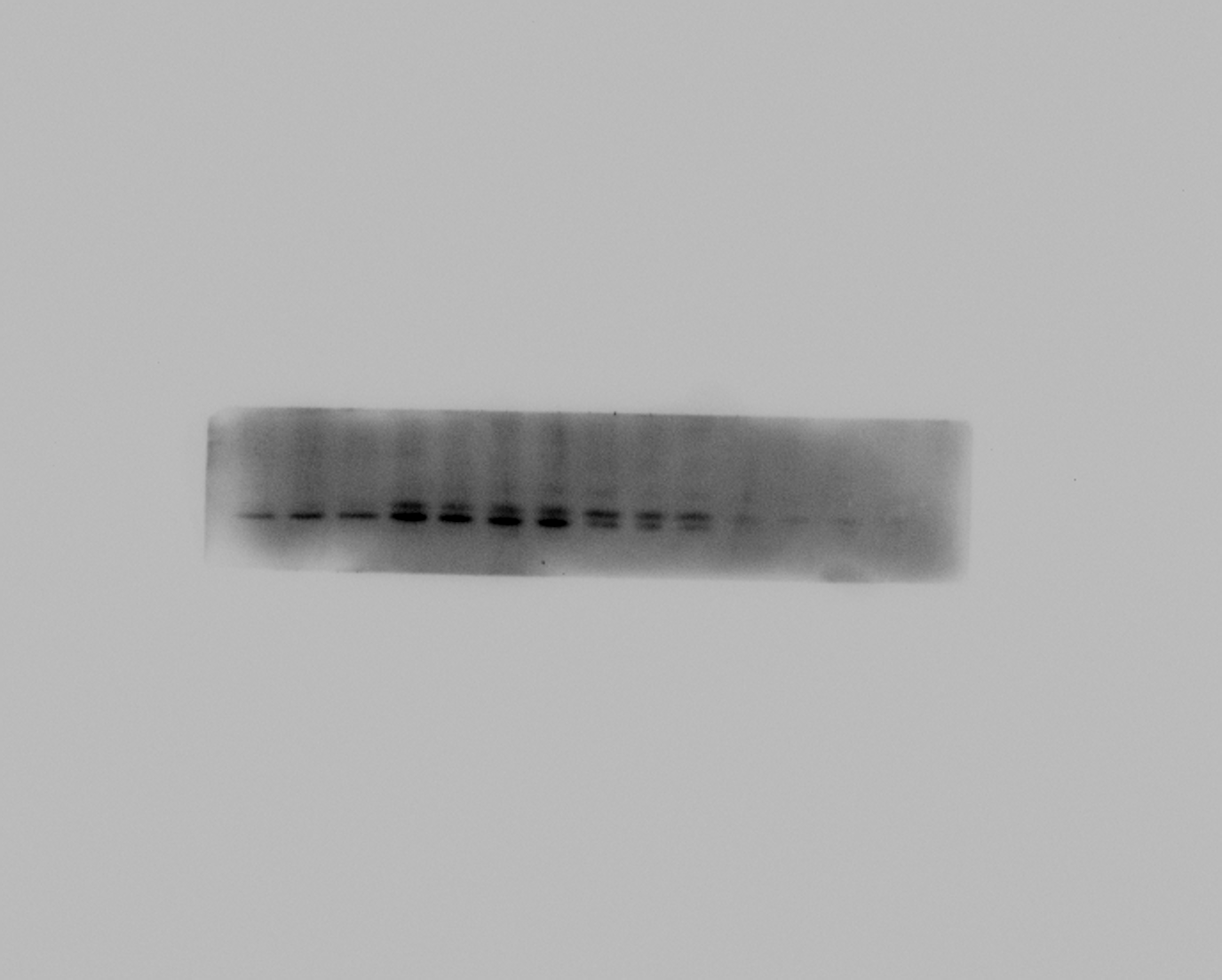

Supplement: Supplementary file 11 — Source data Fig. 6 [file 44318_2024_359_MOESM11_ESM.zip › Figure 6/Fig 6D and 6E/Fig 6D/LAMTOR3+4.Tif]

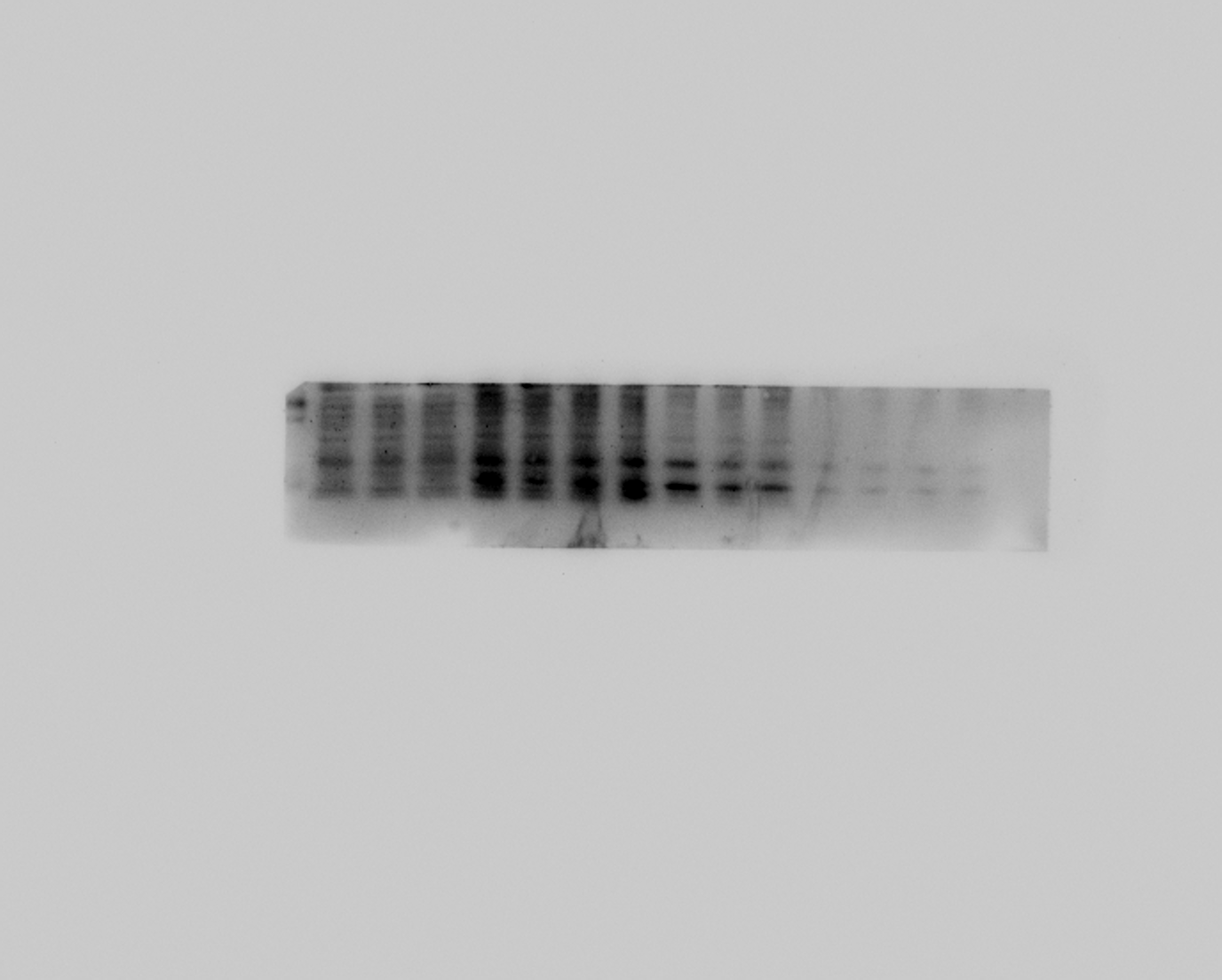

Supplement: Supplementary file 11 — Source data Fig. 6 [file 44318_2024_359_MOESM11_ESM.zip › Figure 6/Fig 6D and 6E/Fig 6D/LAMTOR5+2.Tif]

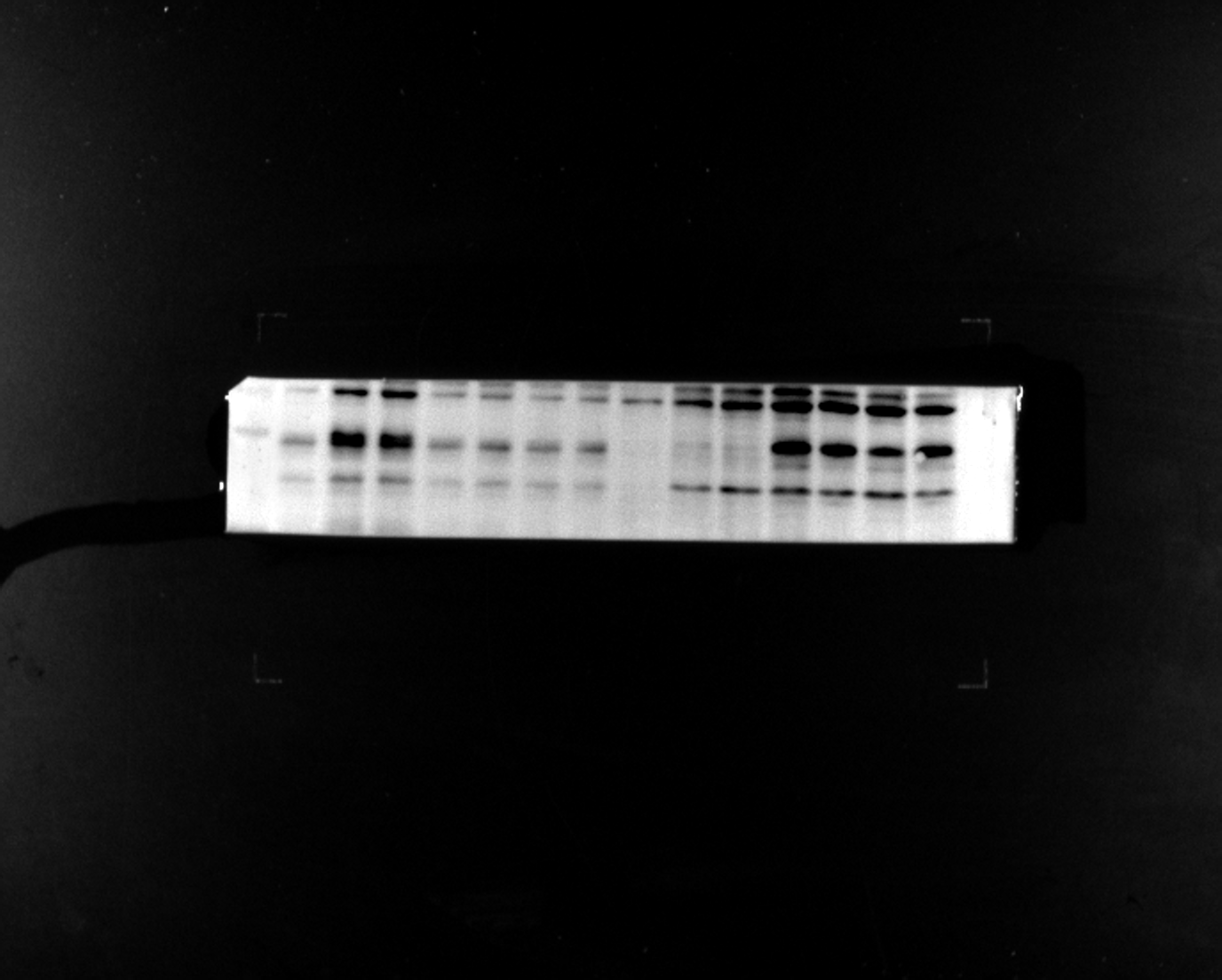

Supplement: Supplementary file 11 — Source data Fig. 6 [file 44318_2024_359_MOESM11_ESM.zip › Figure 6/Fig 6D and 6E/Fig 6D/P27KIP1-merge.Tif]

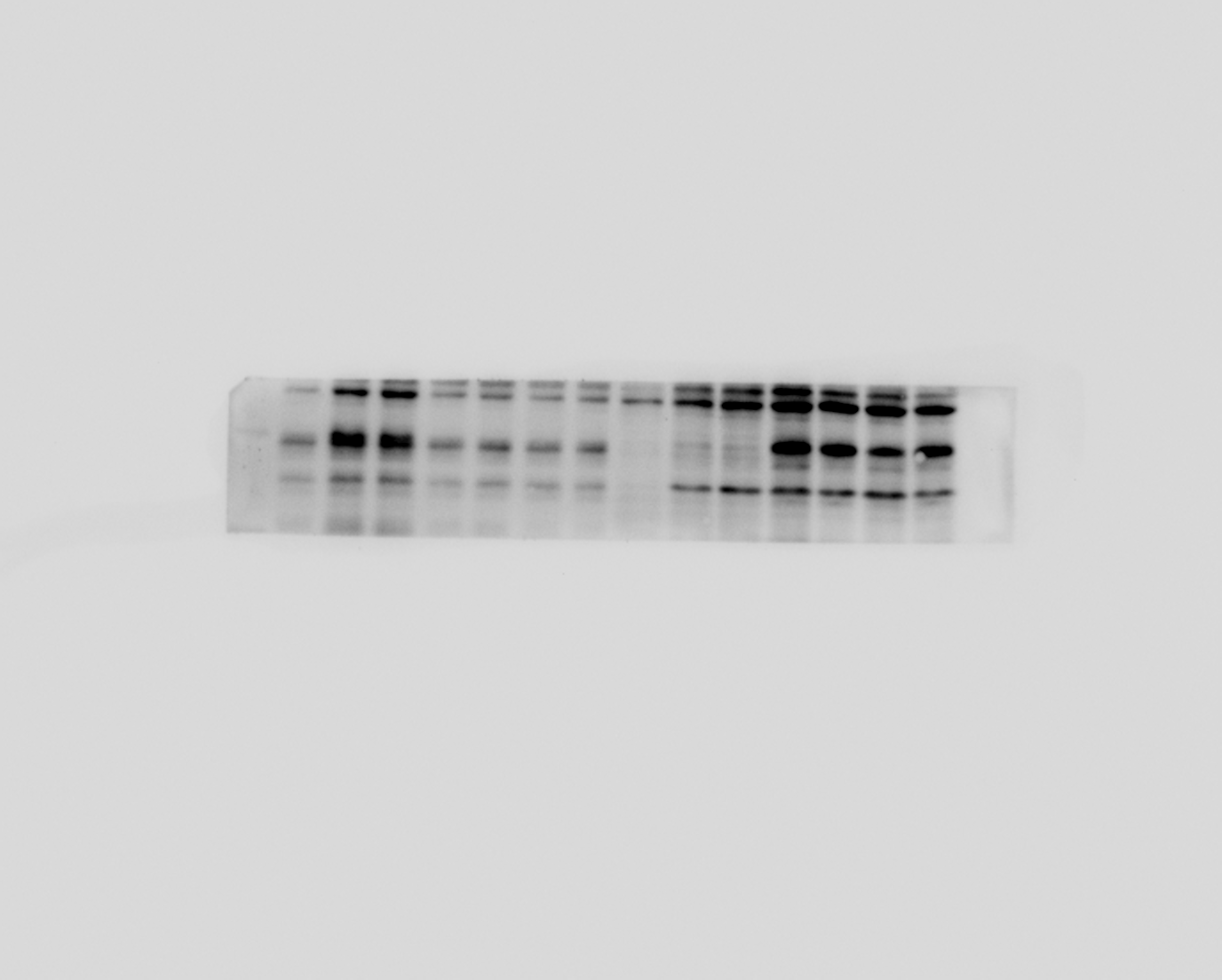

Supplement: Supplementary file 11 — Source data Fig. 6 [file 44318_2024_359_MOESM11_ESM.zip › Figure 6/Fig 6D and 6E/Fig 6D/P27KIP1.Tif]

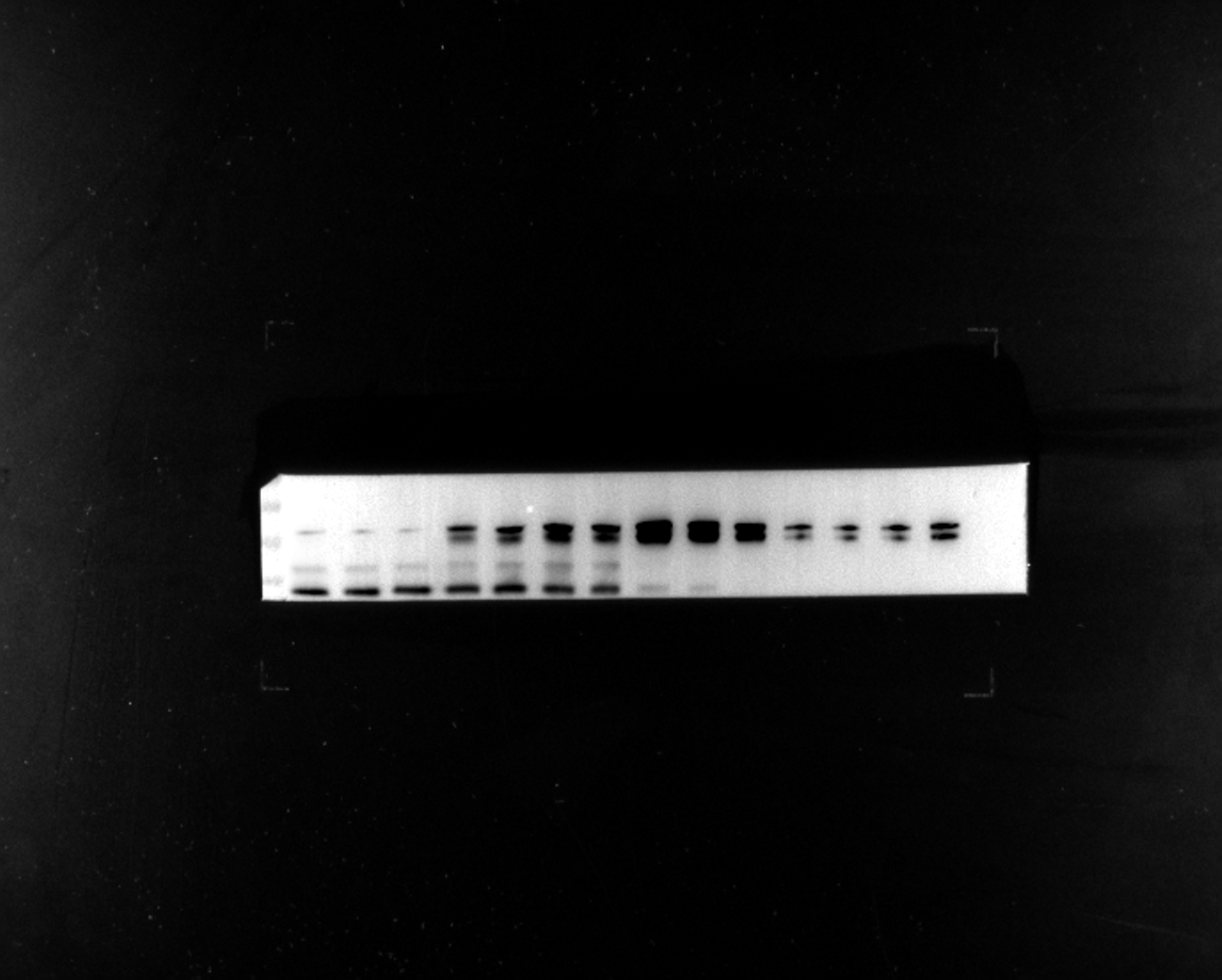

Supplement: Supplementary file 11 — Source data Fig. 6 [file 44318_2024_359_MOESM11_ESM.zip › Figure 6/Fig 6D and 6E/Fig 6D/RagA-merge.Tif]

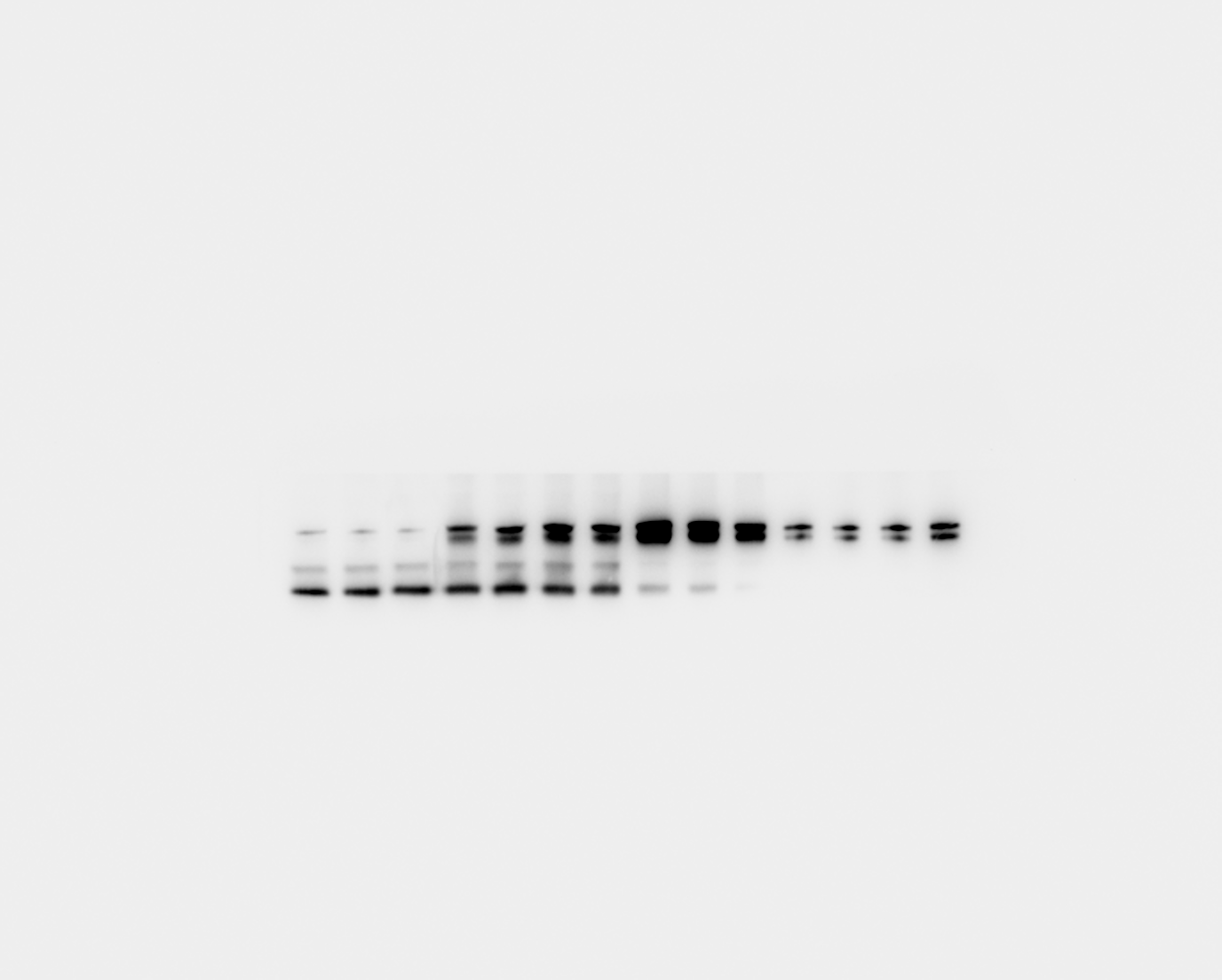

Supplement: Supplementary file 11 — Source data Fig. 6 [file 44318_2024_359_MOESM11_ESM.zip › Figure 6/Fig 6D and 6E/Fig 6D/RagA.Tif]

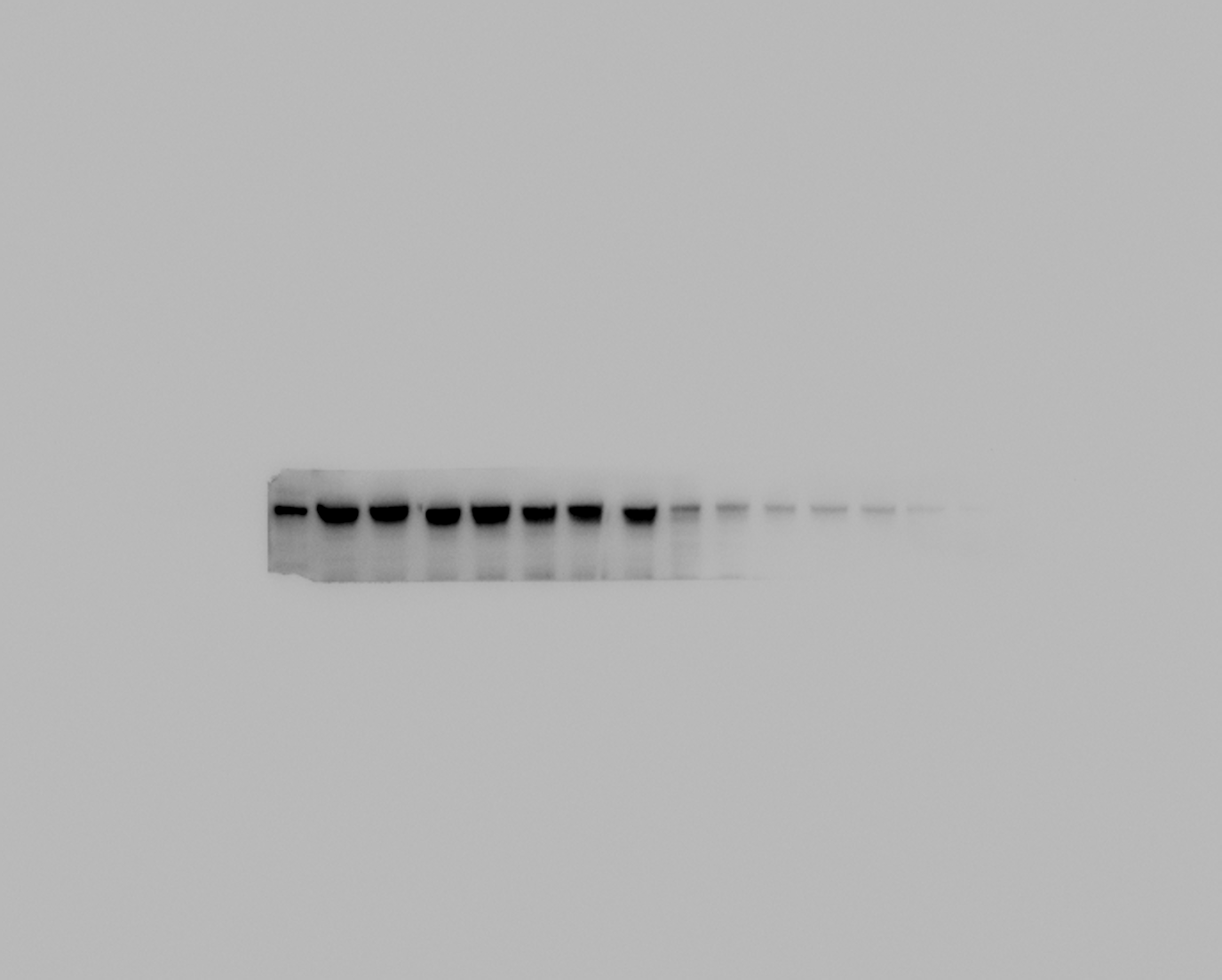

Supplement: Supplementary file 11 — Source data Fig. 6 [file 44318_2024_359_MOESM11_ESM.zip › Figure 6/Fig 6D and 6E/Fig 6D/TUBULIN.Tif]

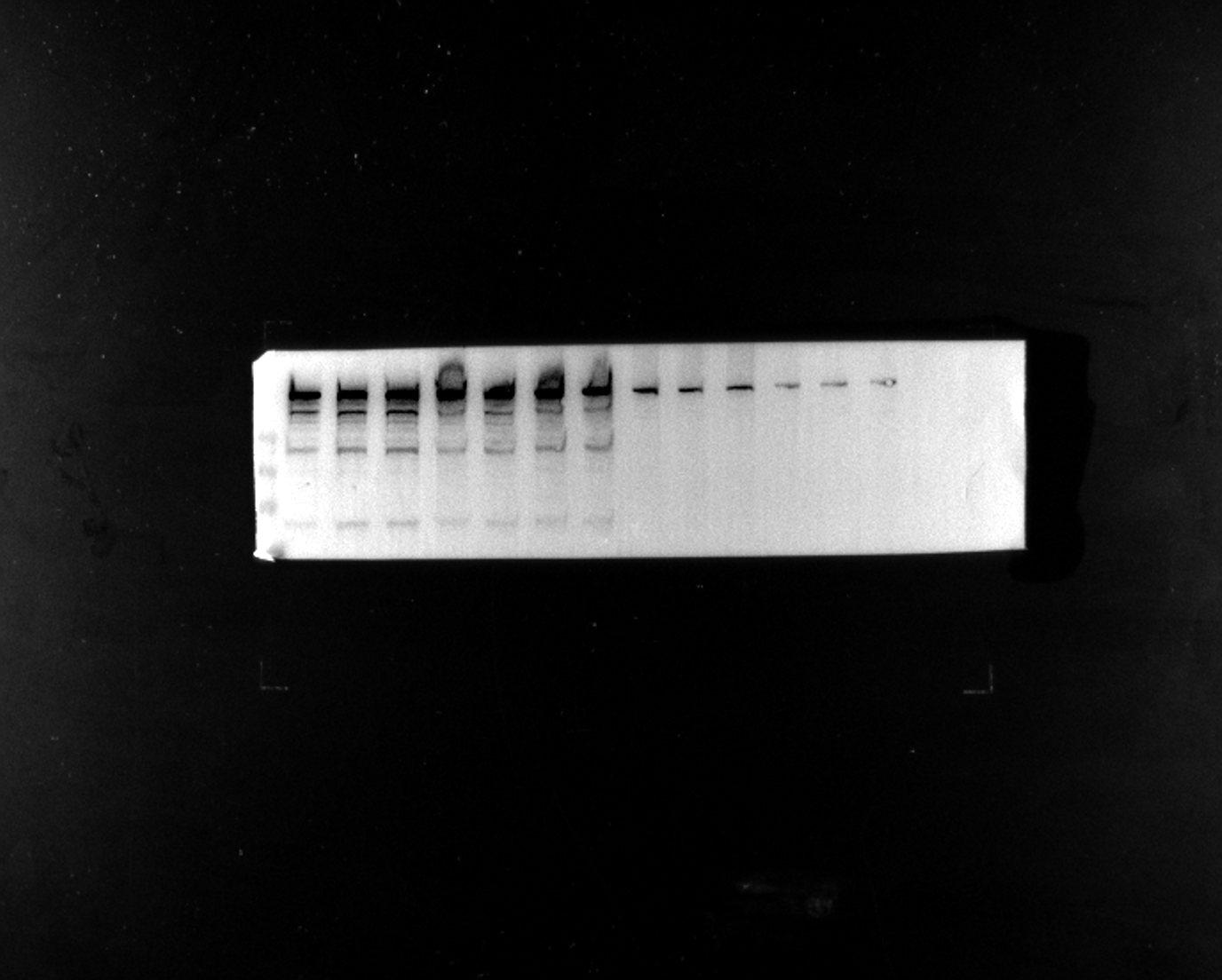

Supplement: Supplementary file 11 — Source data Fig. 6 [file 44318_2024_359_MOESM11_ESM.zip › Figure 6/Fig 6D and 6E/Fig 6D/mTOR-merge.Tif]

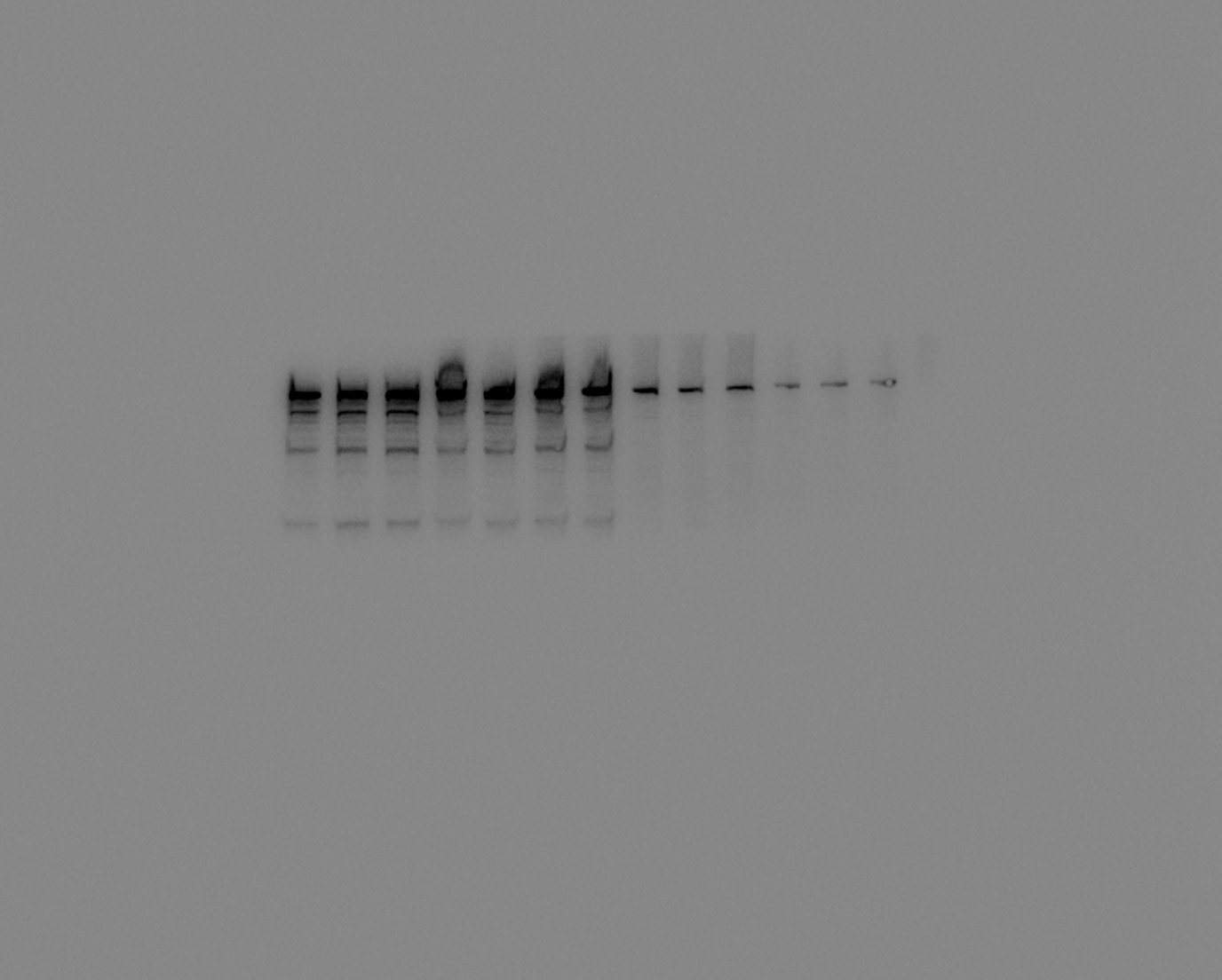

Supplement: Supplementary file 11 — Source data Fig. 6 [file 44318_2024_359_MOESM11_ESM.zip › Figure 6/Fig 6D and 6E/Fig 6D/mTOR.Tif]

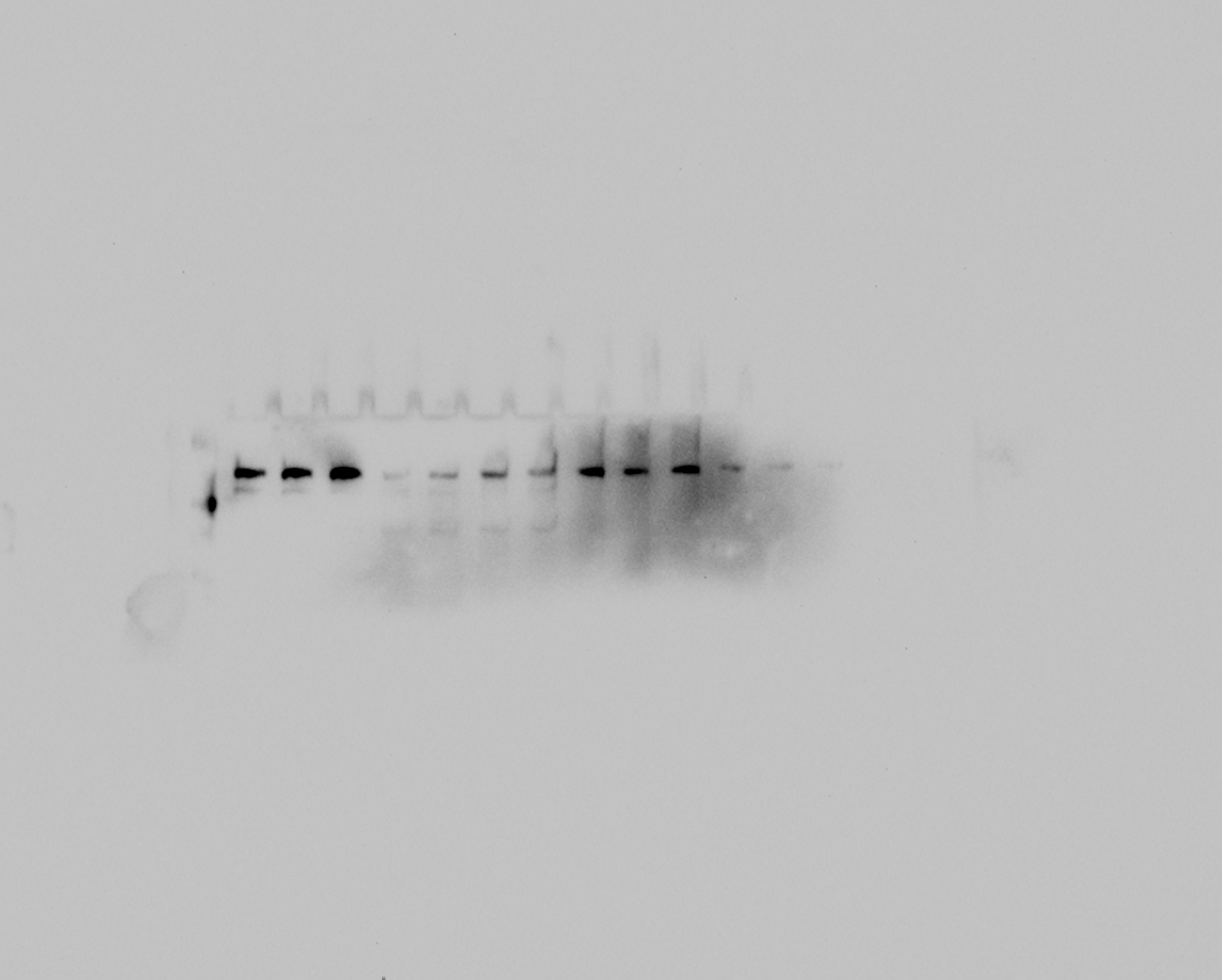

Supplement: Supplementary file 11 — Source data Fig. 6 [file 44318_2024_359_MOESM11_ESM.zip › Figure 6/Fig 6D and 6E/Fig 6D/p-mTOR.Tif]

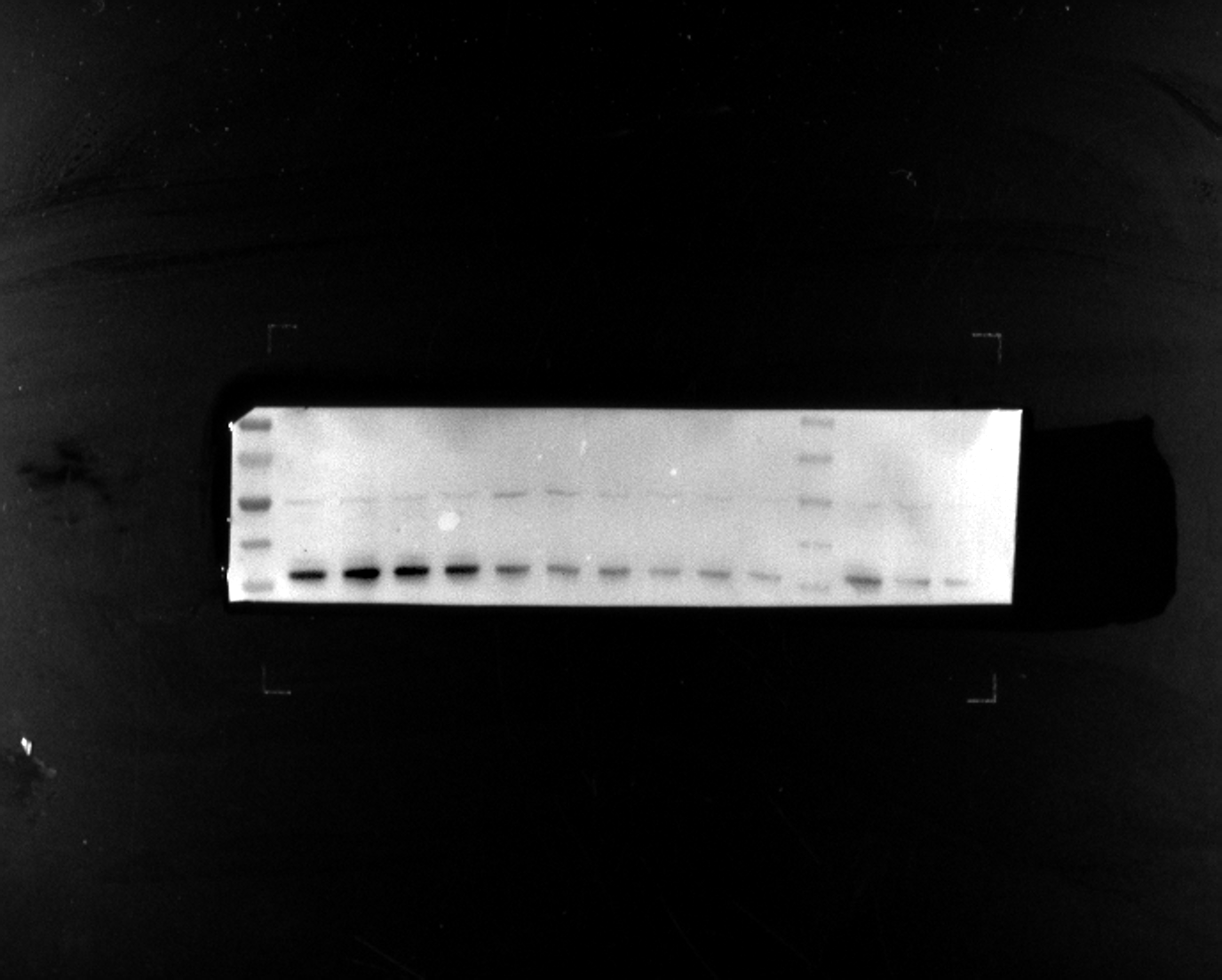

Supplement: Supplementary file 11 — Source data Fig. 6 [file 44318_2024_359_MOESM11_ESM.zip › Figure 6/Fig 6F and 6G/Fig 6F/1-SLC38A2-merge.Tif]

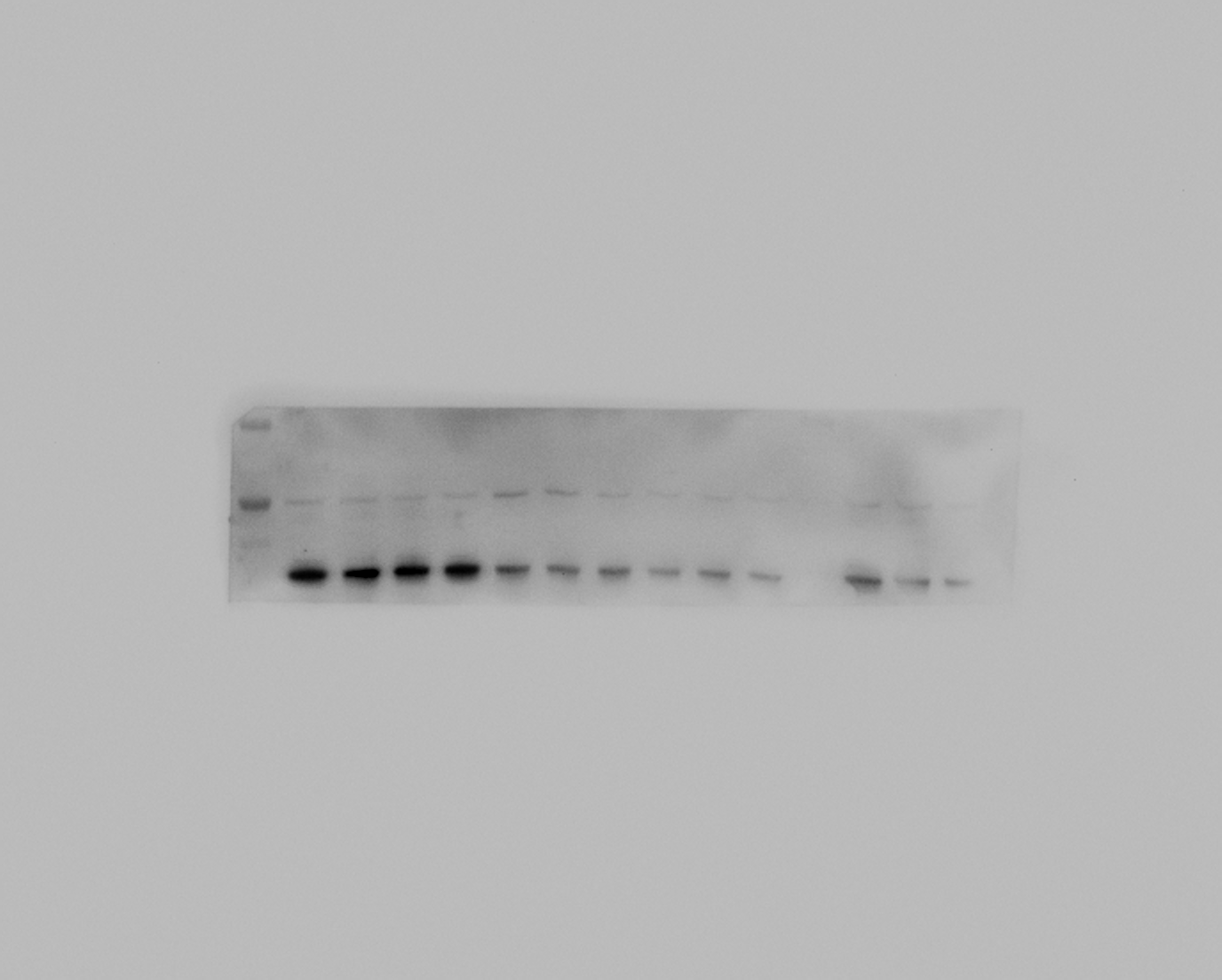

Supplement: Supplementary file 11 — Source data Fig. 6 [file 44318_2024_359_MOESM11_ESM.zip › Figure 6/Fig 6F and 6G/Fig 6F/1-SLC38A2.Tif]

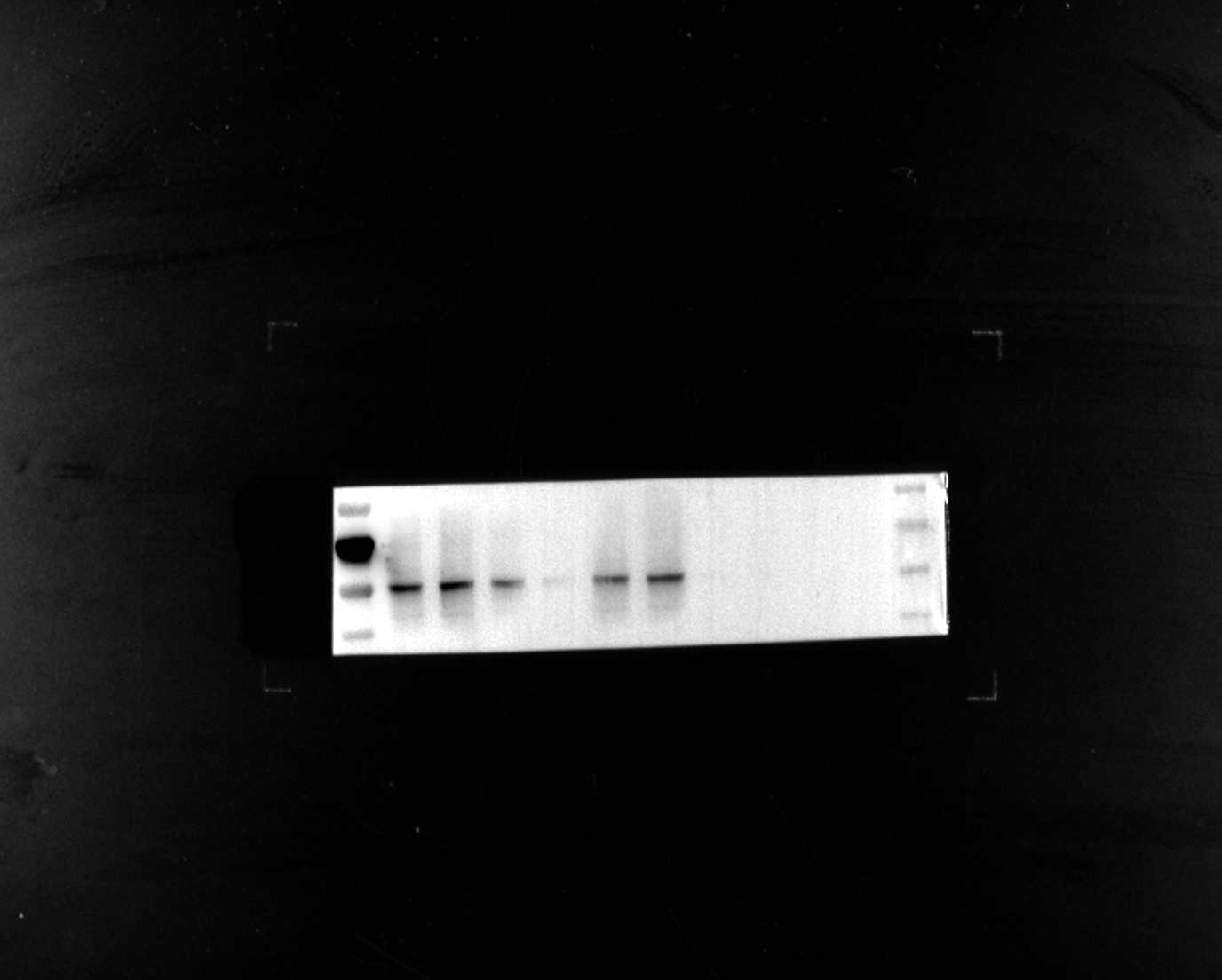

Supplement: Supplementary file 11 — Source data Fig. 6 [file 44318_2024_359_MOESM11_ESM.zip › Figure 6/Fig 6F and 6G/Fig 6F/2-TRIM21-merge.Tif]

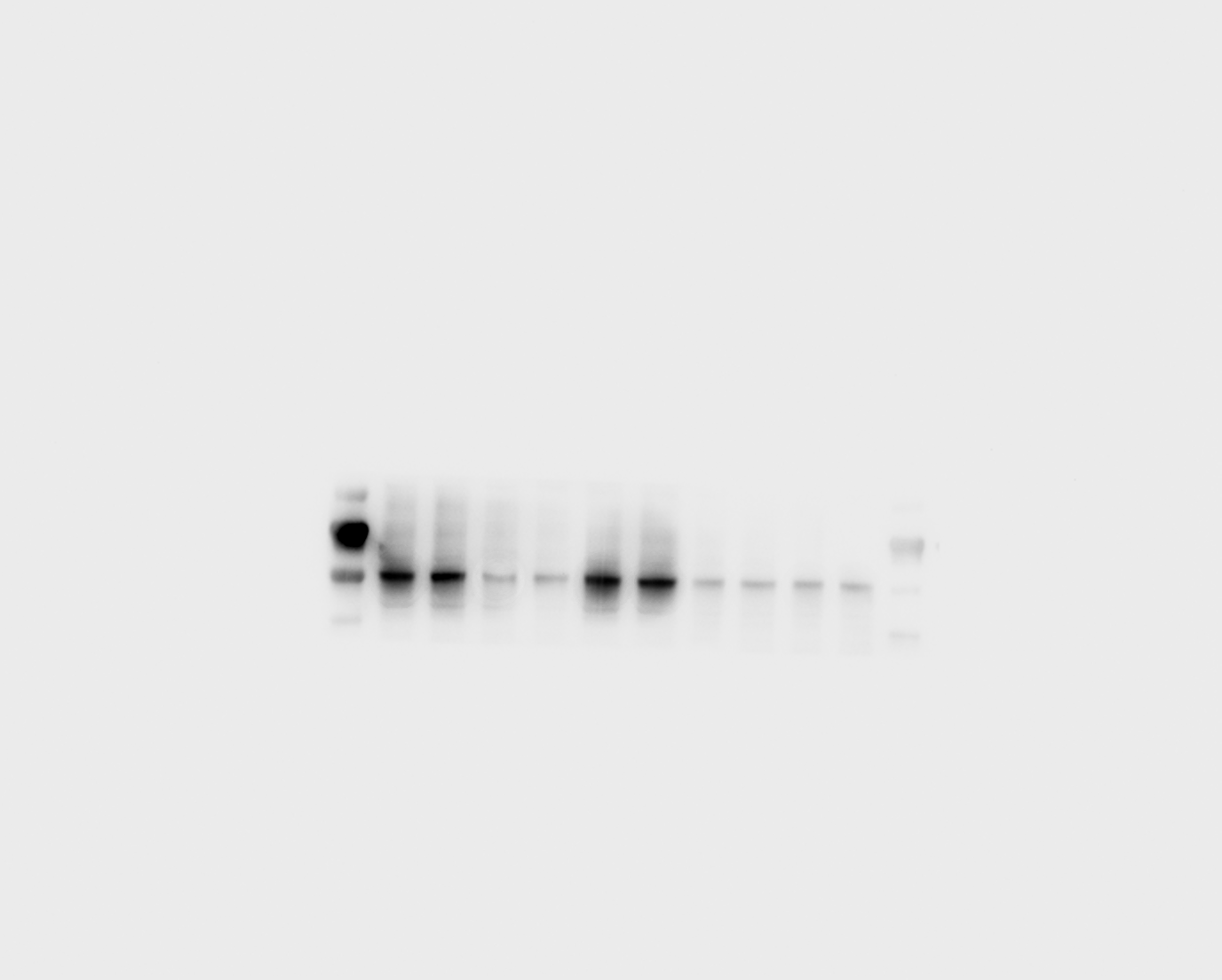

Supplement: Supplementary file 11 — Source data Fig. 6 [file 44318_2024_359_MOESM11_ESM.zip › Figure 6/Fig 6F and 6G/Fig 6F/2-TRIM21.Tif]

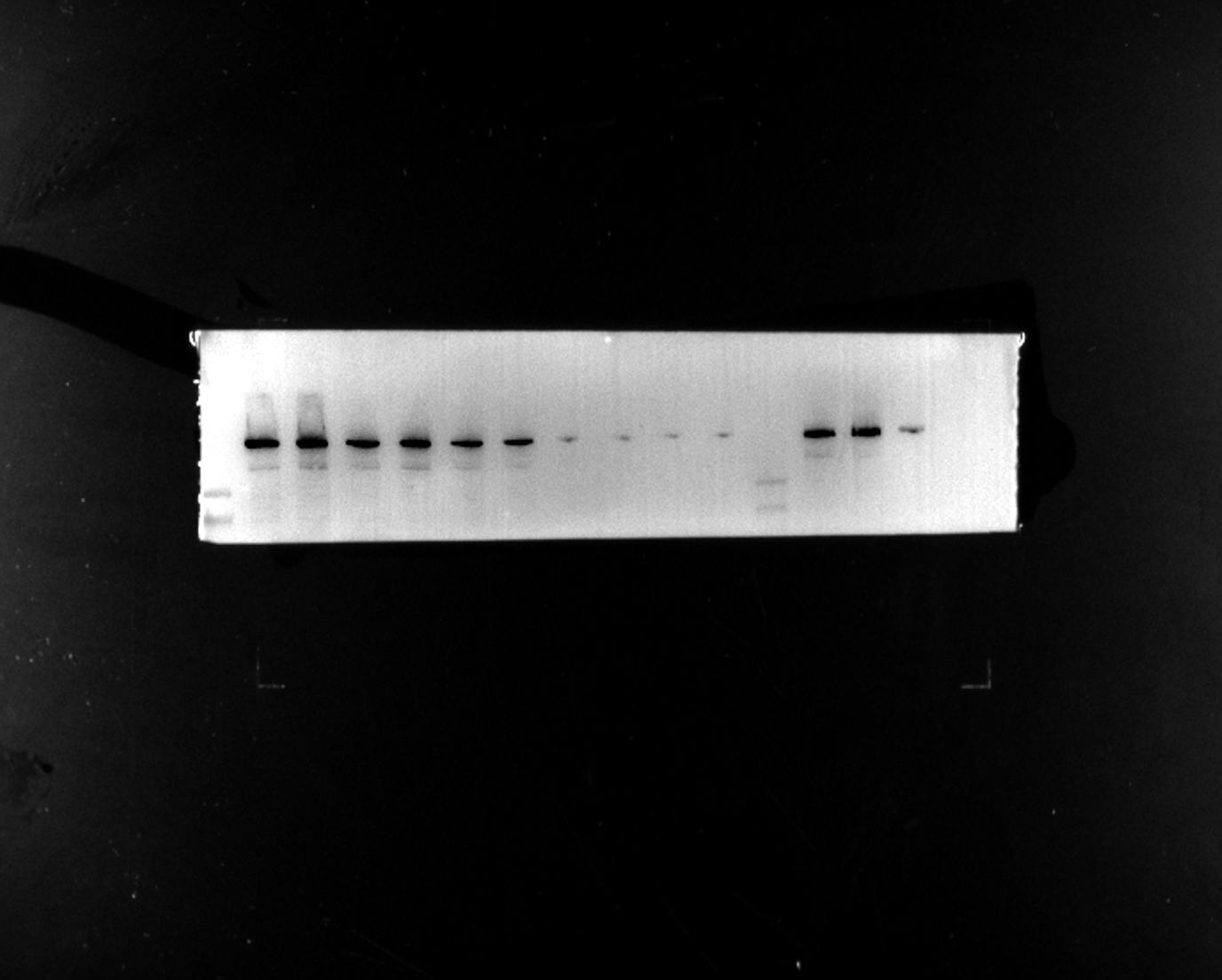

Supplement: Supplementary file 11 — Source data Fig. 6 [file 44318_2024_359_MOESM11_ESM.zip › Figure 6/Fig 6F and 6G/Fig 6F/3-p-mTOR-merge.Tif]

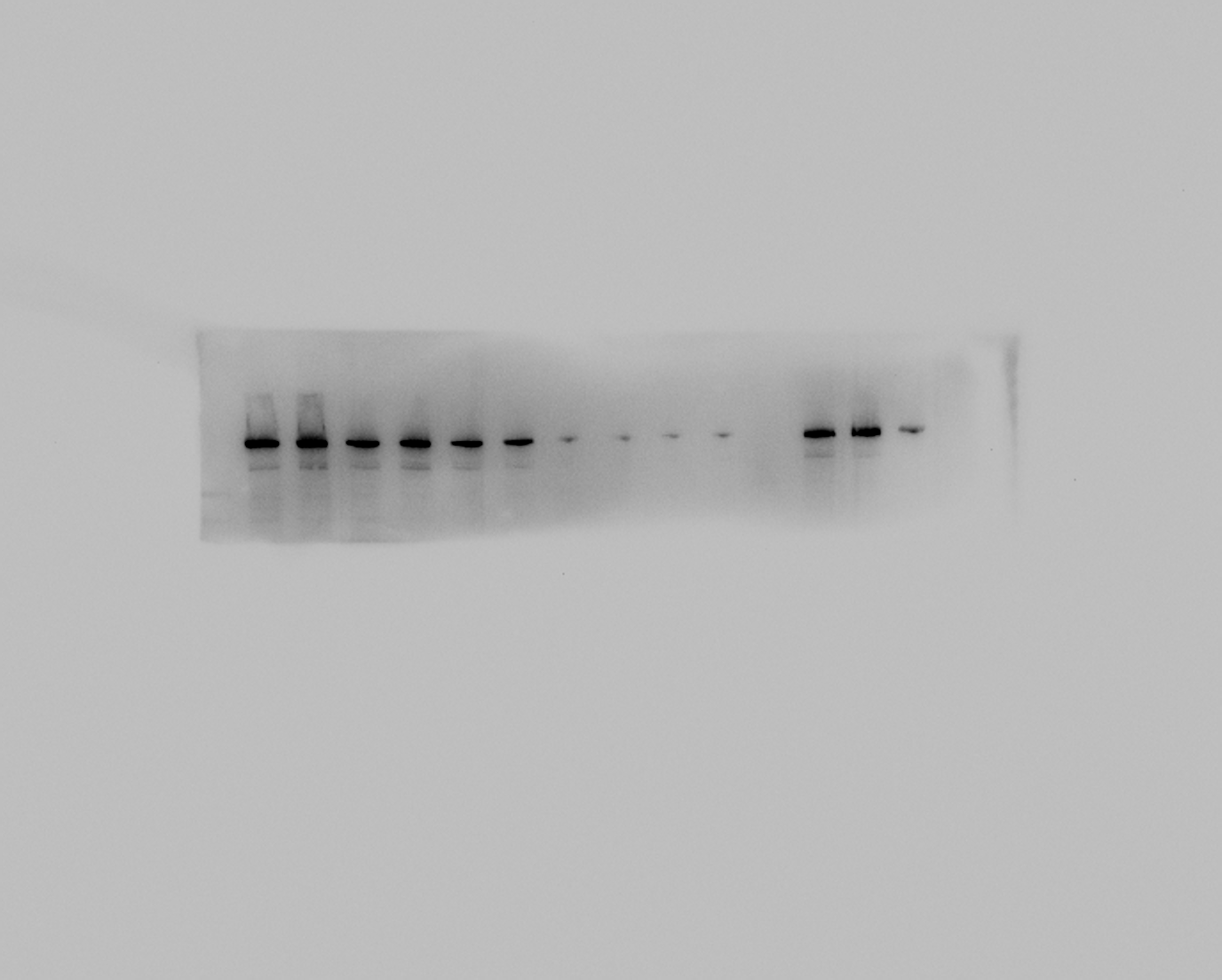

Supplement: Supplementary file 11 — Source data Fig. 6 [file 44318_2024_359_MOESM11_ESM.zip › Figure 6/Fig 6F and 6G/Fig 6F/3-p-mTOR.Tif]

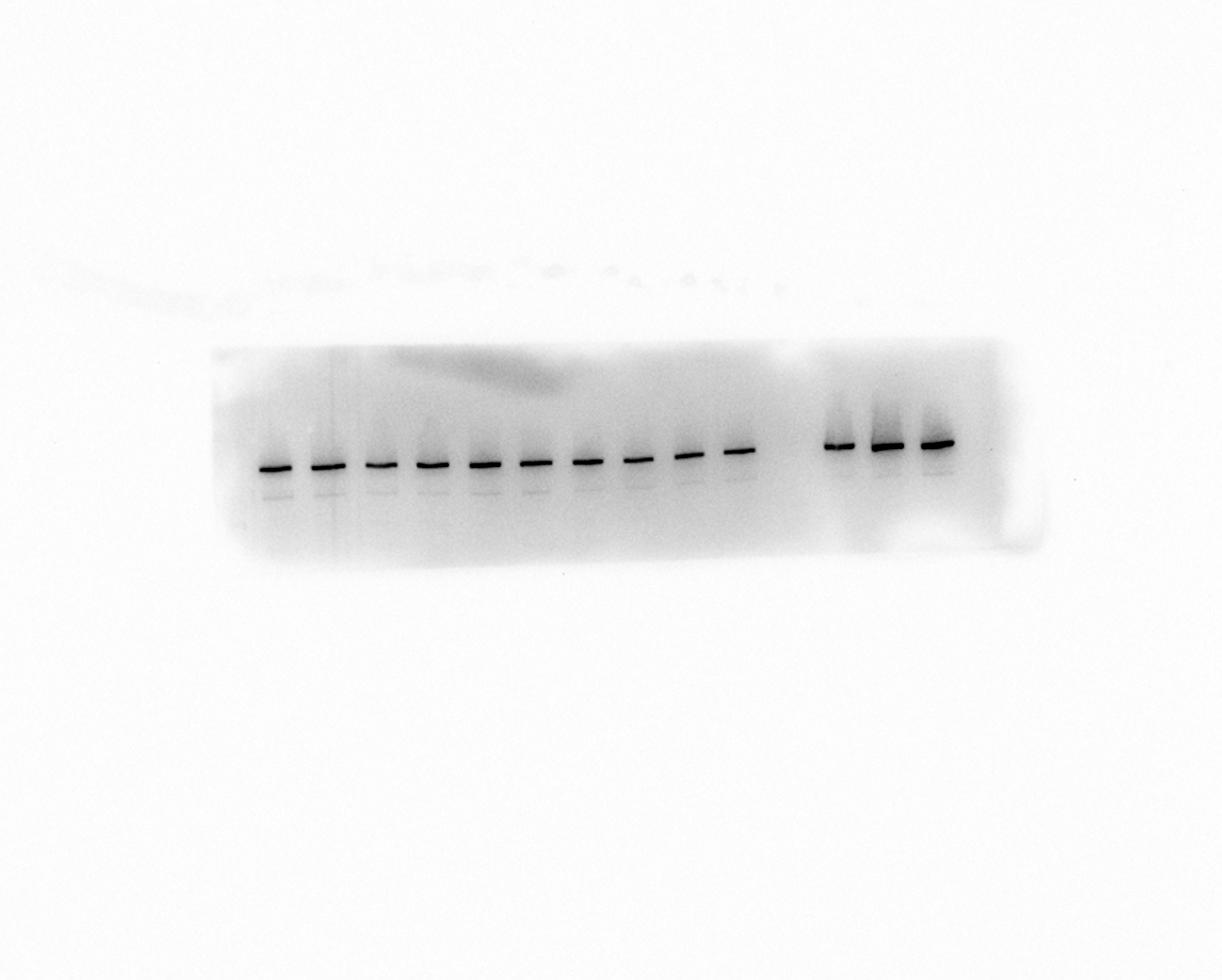

Supplement: Supplementary file 11 — Source data Fig. 6 [file 44318_2024_359_MOESM11_ESM.zip › Figure 6/Fig 6F and 6G/Fig 6F/4-mTOR.Tif]

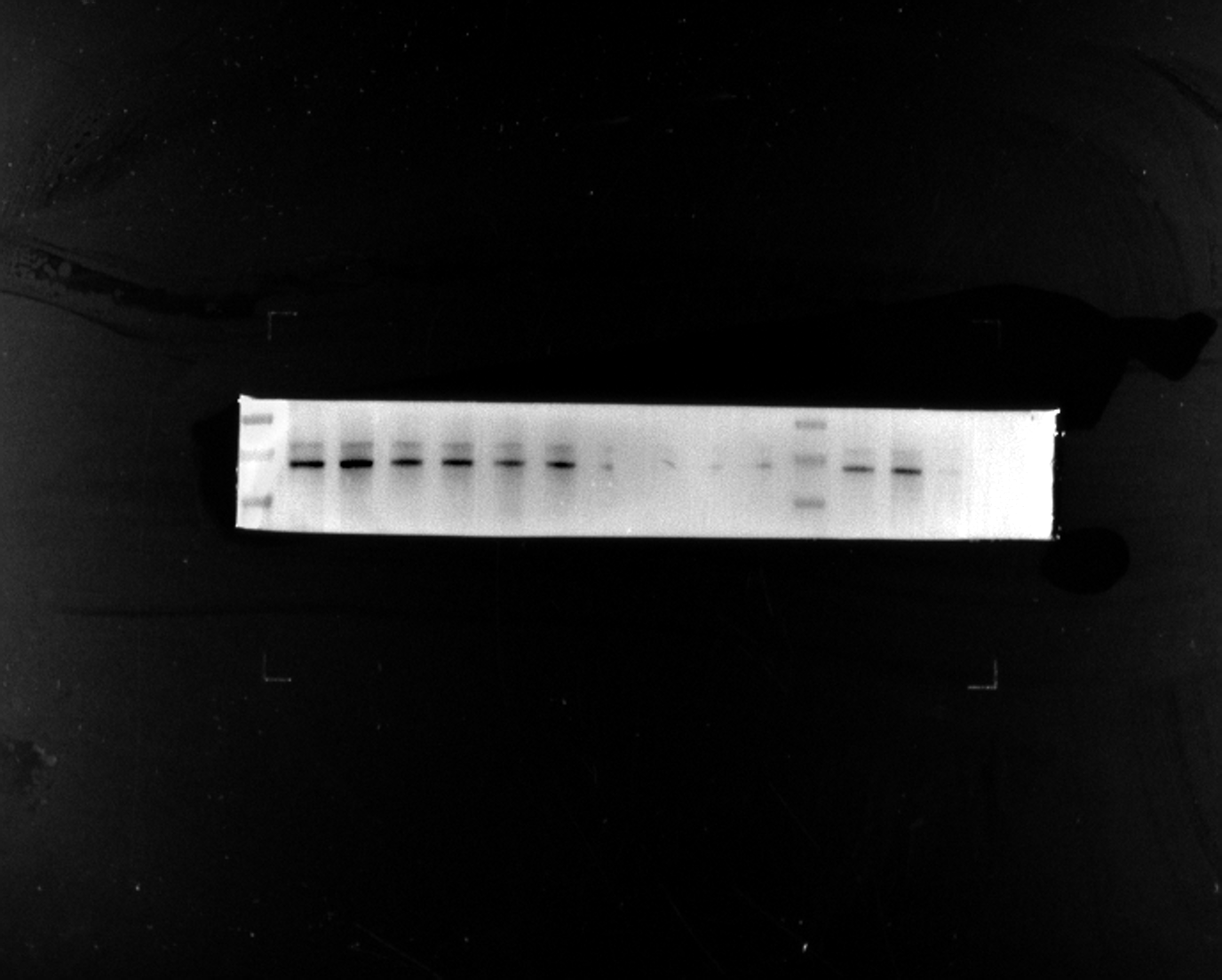

Supplement: Supplementary file 11 — Source data Fig. 6 [file 44318_2024_359_MOESM11_ESM.zip › Figure 6/Fig 6F and 6G/Fig 6F/5-p-S6K-merge.Tif]

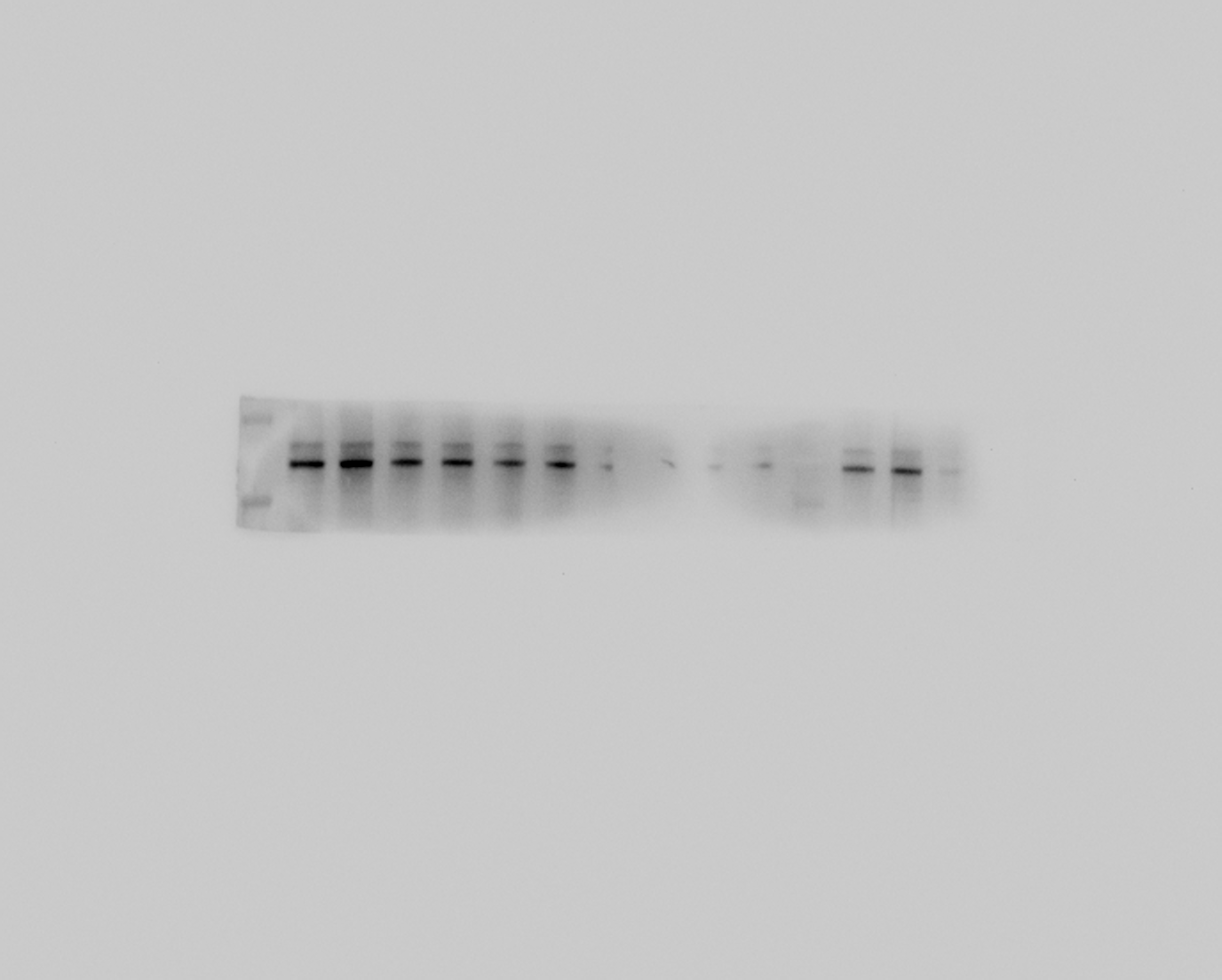

Supplement: Supplementary file 11 — Source data Fig. 6 [file 44318_2024_359_MOESM11_ESM.zip › Figure 6/Fig 6F and 6G/Fig 6F/5-p-S6K.Tif]

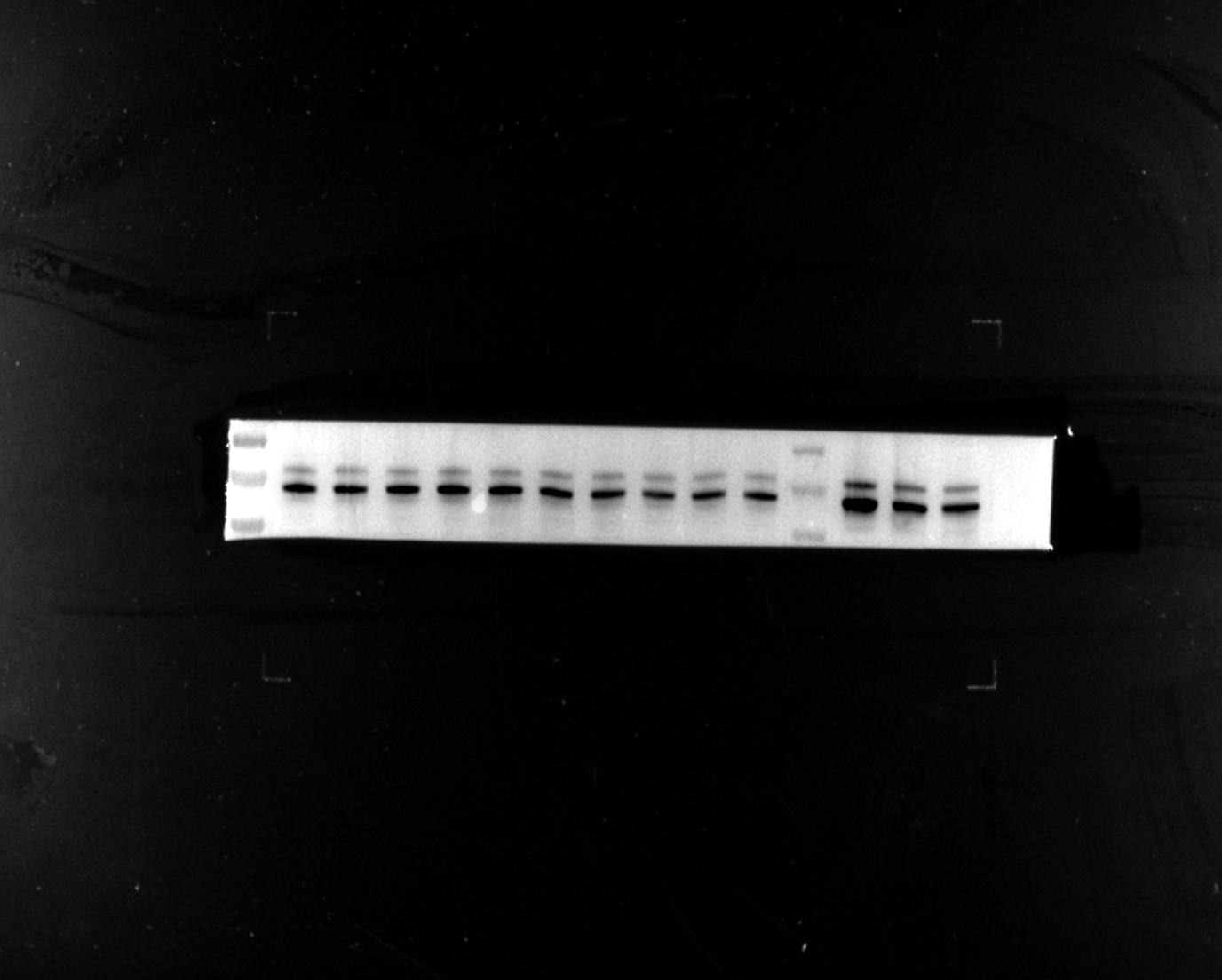

Supplement: Supplementary file 11 — Source data Fig. 6 [file 44318_2024_359_MOESM11_ESM.zip › Figure 6/Fig 6F and 6G/Fig 6F/6-S6K-merge.Tif]

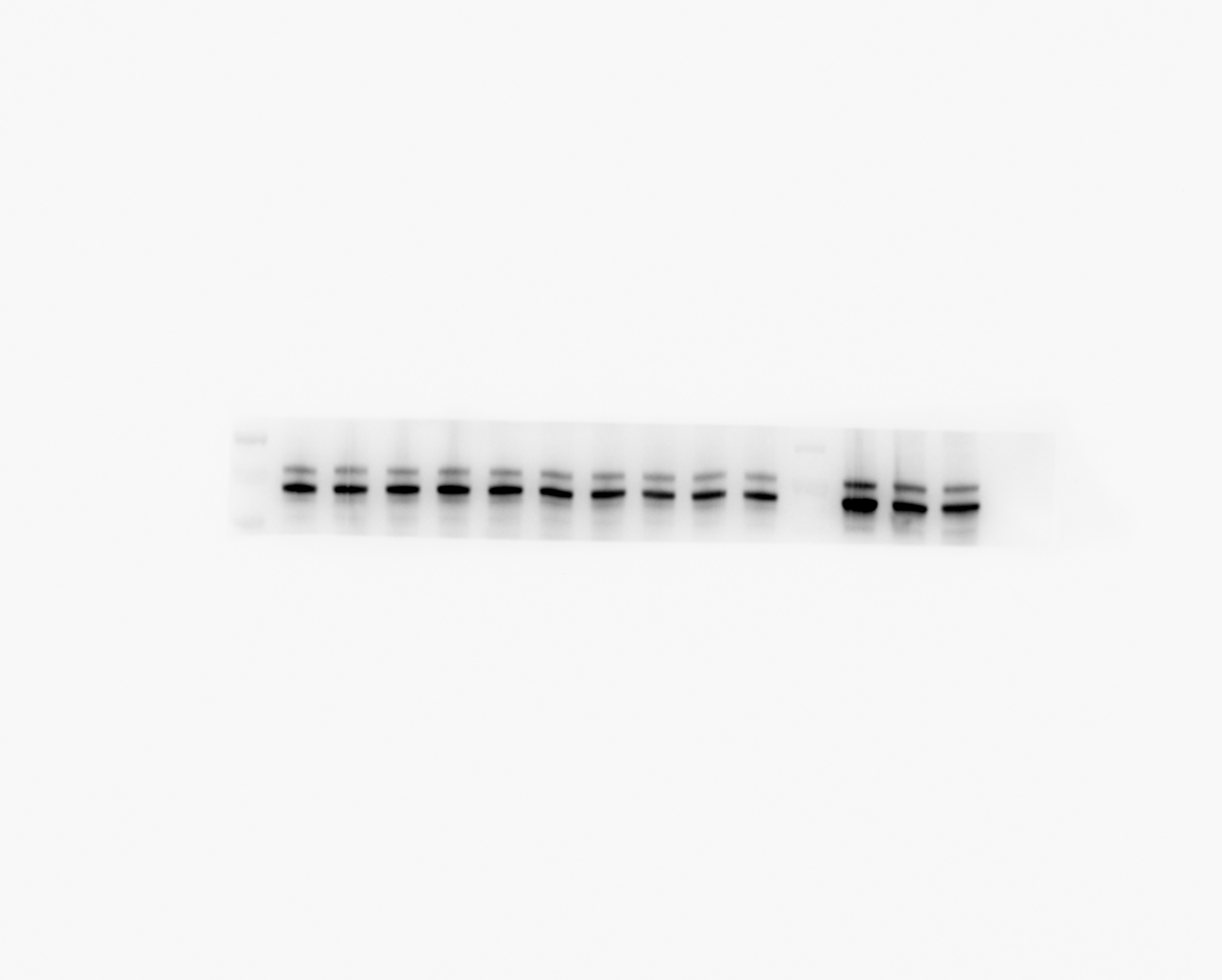

Supplement: Supplementary file 11 — Source data Fig. 6 [file 44318_2024_359_MOESM11_ESM.zip › Figure 6/Fig 6F and 6G/Fig 6F/6-S6K.Tif]

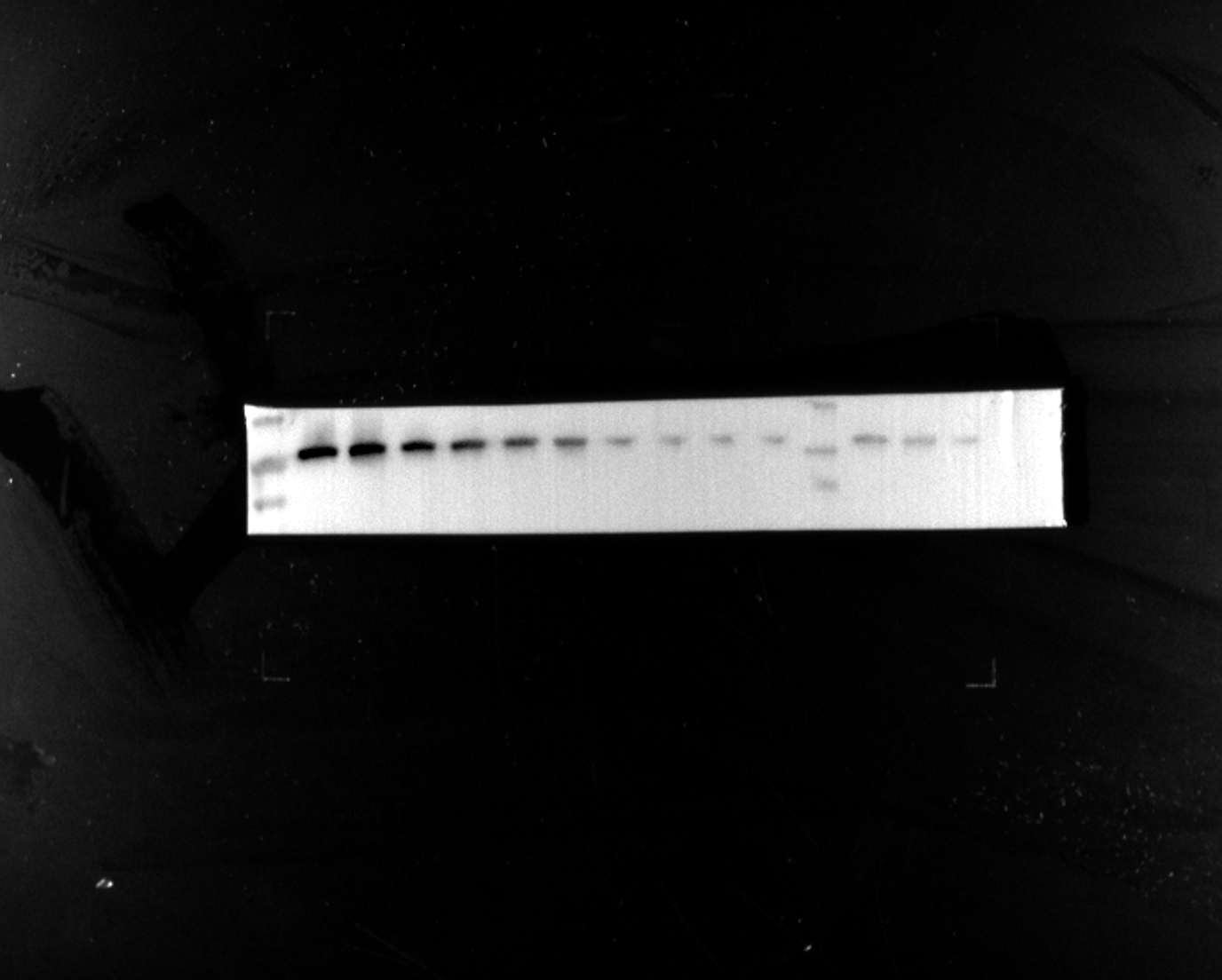

Supplement: Supplementary file 11 — Source data Fig. 6 [file 44318_2024_359_MOESM11_ESM.zip › Figure 6/Fig 6F and 6G/Fig 6F/7-p-S6-merge.Tif]
