# Supplementary figures and images for: Micropeptide hSPAR regulates glutamine levels and suppresses mammary tumor growth via a TRIM21-P27KIP1-mTOR axis (part 6 of 7)
Source: EMBO J. 2025 Jan 28;44(5):1414–41. doi: 10.1038/s44318-024-00359-z (PMC11876615; doi:10.1038/s44318-024-00359-z)

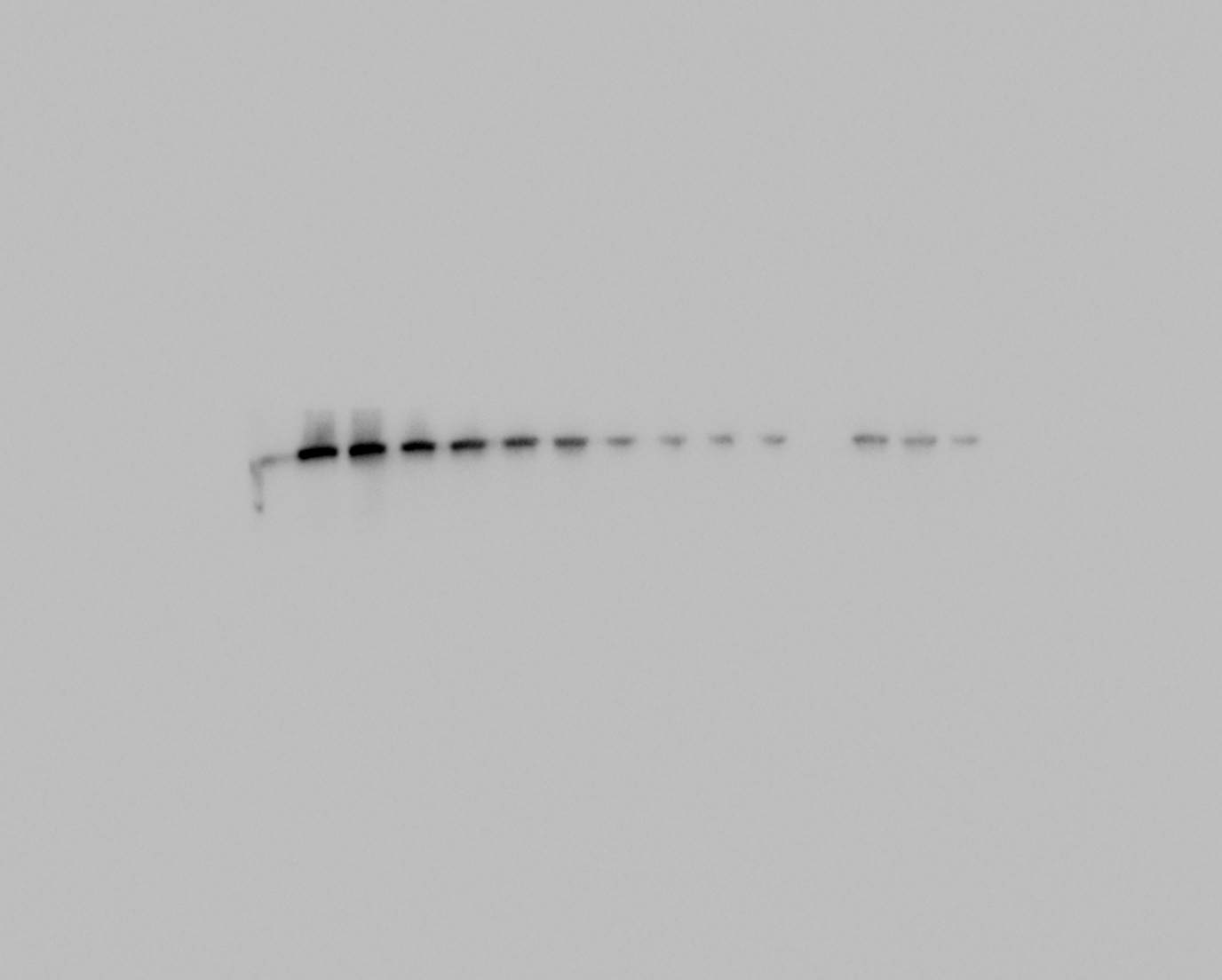

Supplement: Supplementary file 11 — Source data Fig. 6 [file 44318_2024_359_MOESM11_ESM.zip › Figure 6/Fig 6F and 6G/Fig 6F/7-p-S6.Tif]

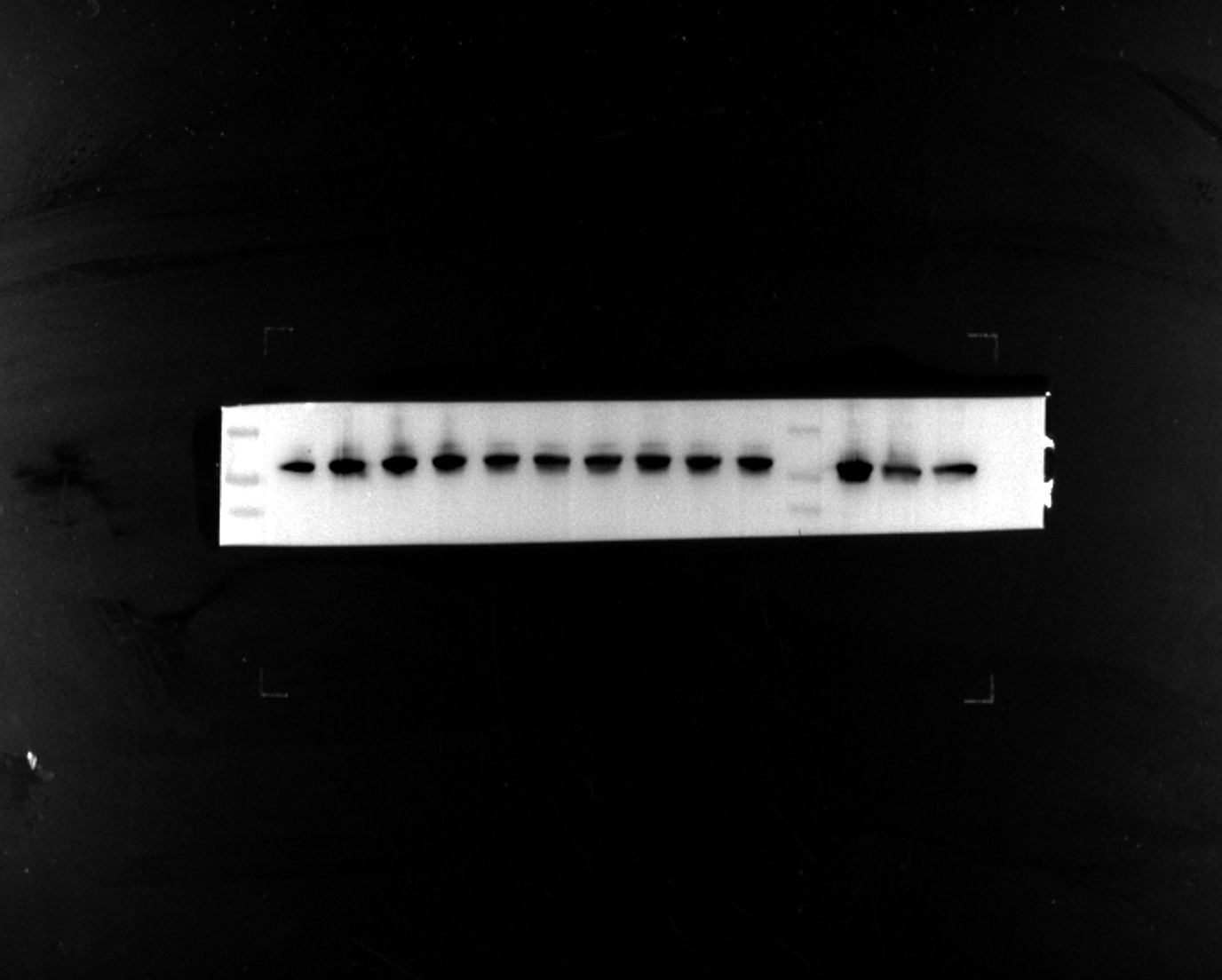

Supplement: Supplementary file 11 — Source data Fig. 6 [file 44318_2024_359_MOESM11_ESM.zip › Figure 6/Fig 6F and 6G/Fig 6F/8-S6-merge.Tif]

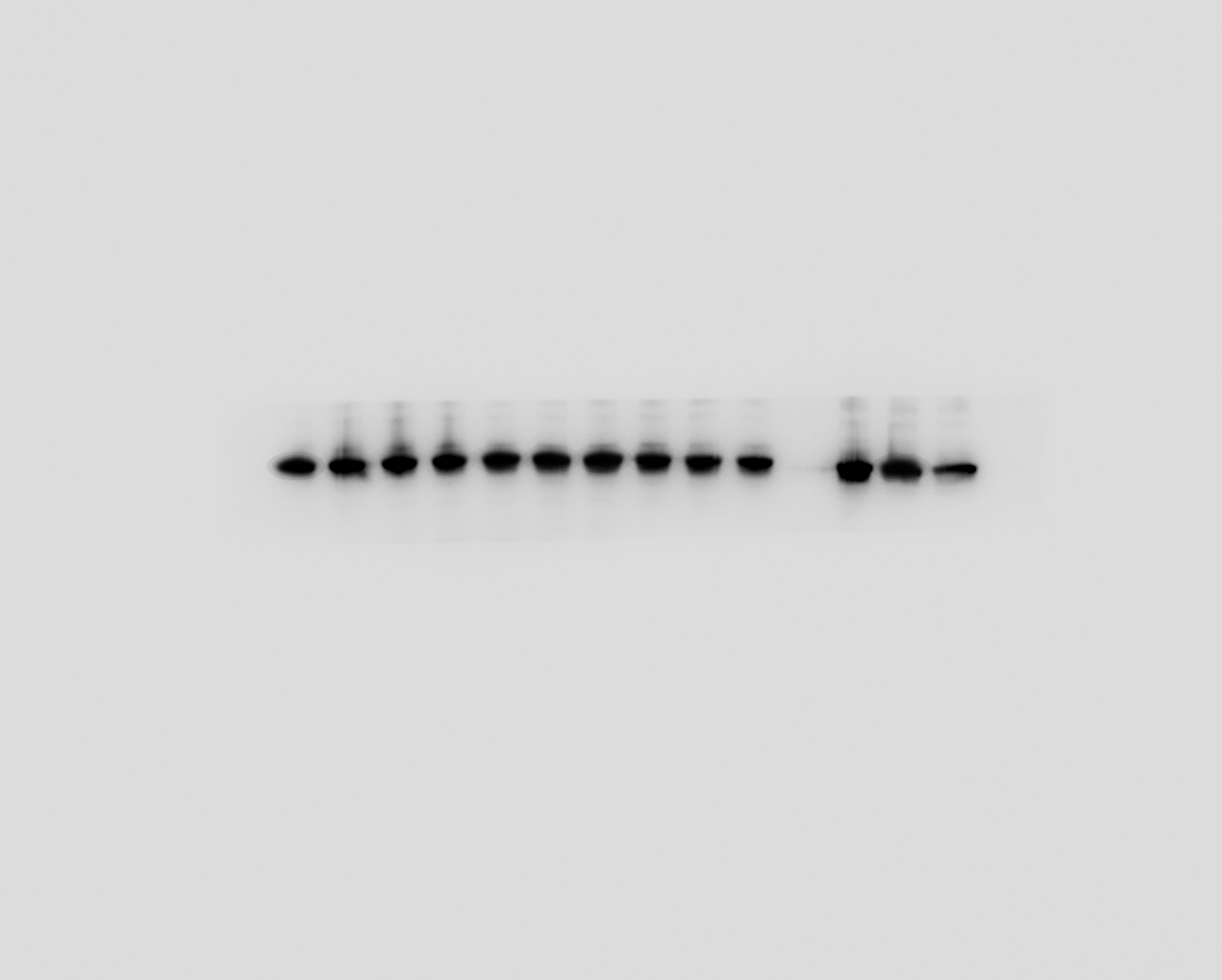

Supplement: Supplementary file 11 — Source data Fig. 6 [file 44318_2024_359_MOESM11_ESM.zip › Figure 6/Fig 6F and 6G/Fig 6F/8-s6.Tif]

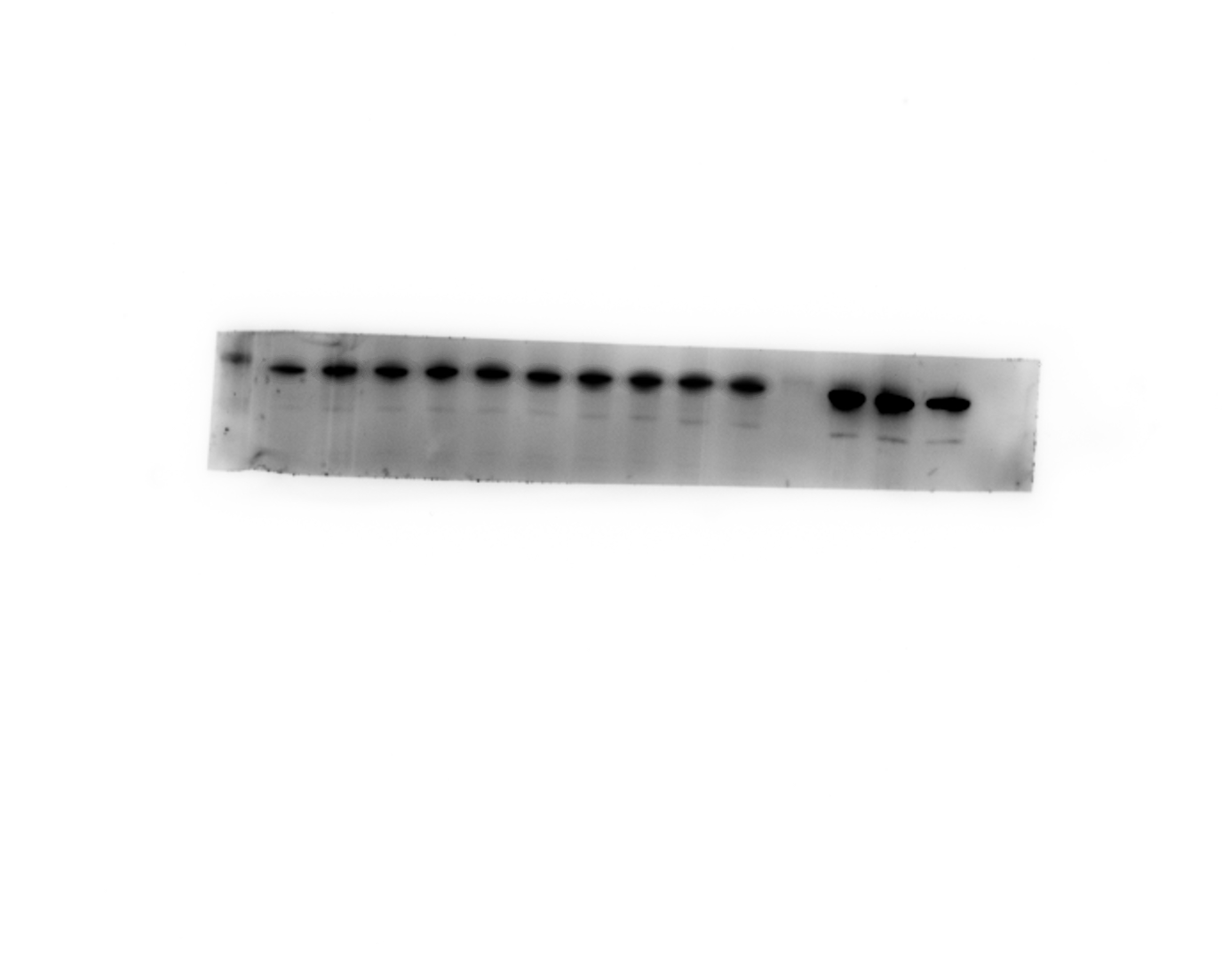

Supplement: Supplementary file 11 — Source data Fig. 6 [file 44318_2024_359_MOESM11_ESM.zip › Figure 6/Fig 6F and 6G/Fig 6F/9-GAPDH.tif]

Fig 6F

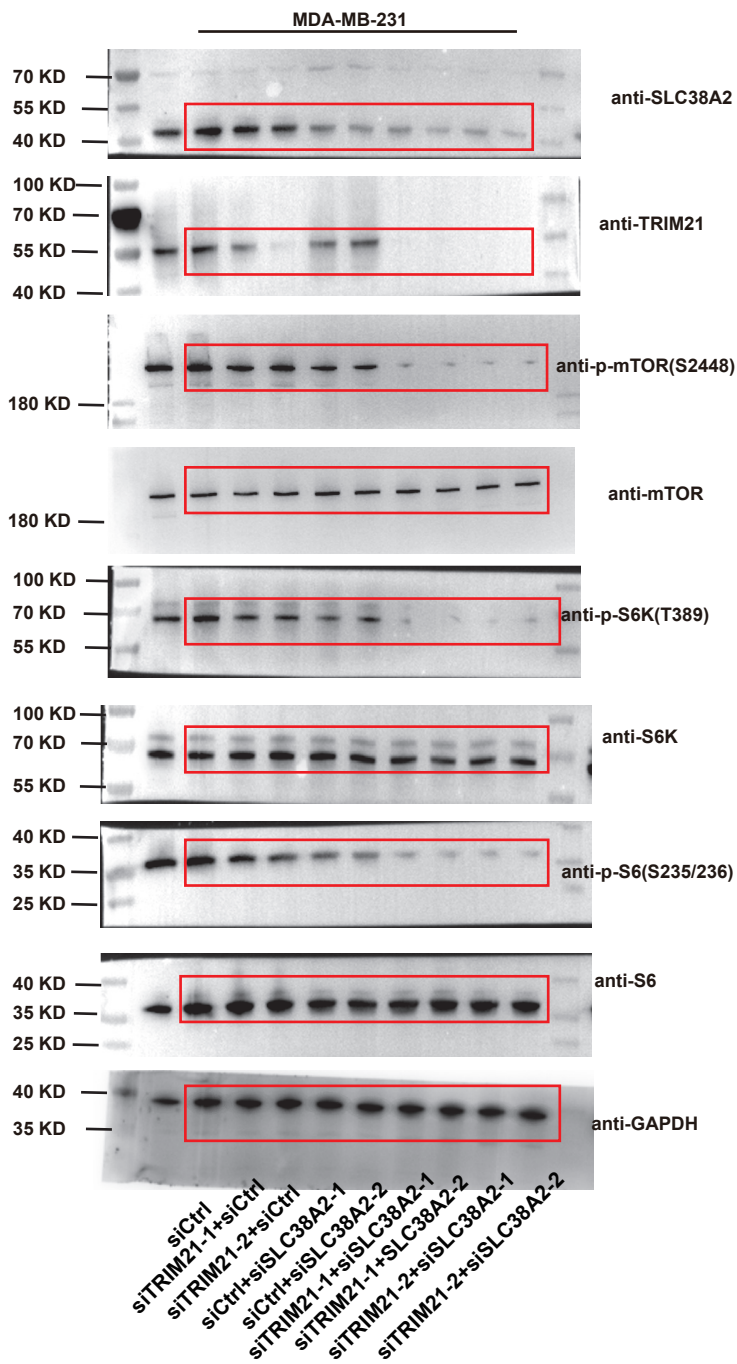

Supplement: Supplementary file 11 — Source data Fig. 6 [file 44318_2024_359_MOESM11_ESM.zip › Figure 6/Fig 6F and 6G/Fig 6F/Fig 6F.pdf]

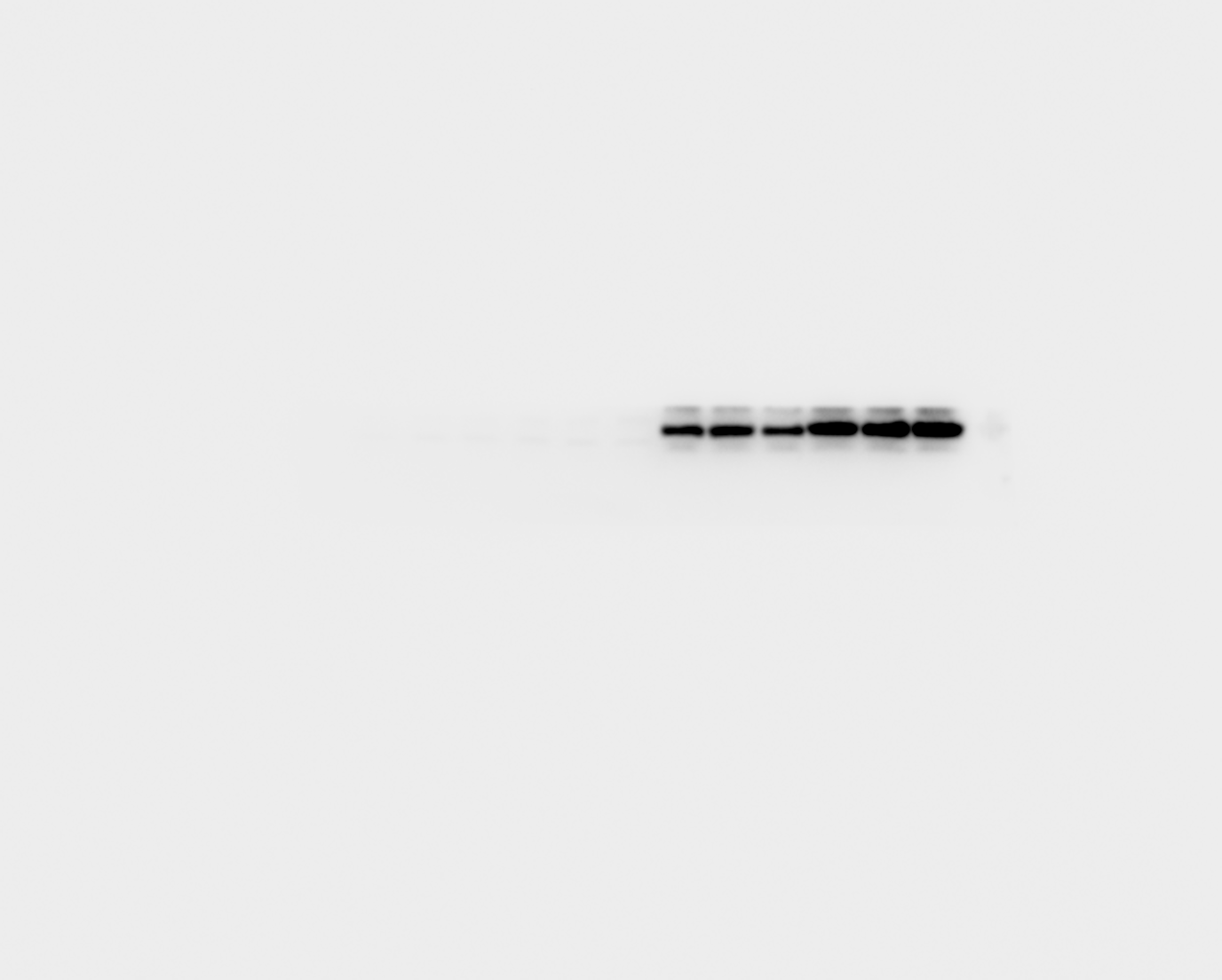

Supplement: Supplementary file 12 — Source data Fig. 7 [file 44318_2024_359_MOESM12_ESM.zip › Figure 7/Fig 7O/9-TAT.Tif]

Fig 7A

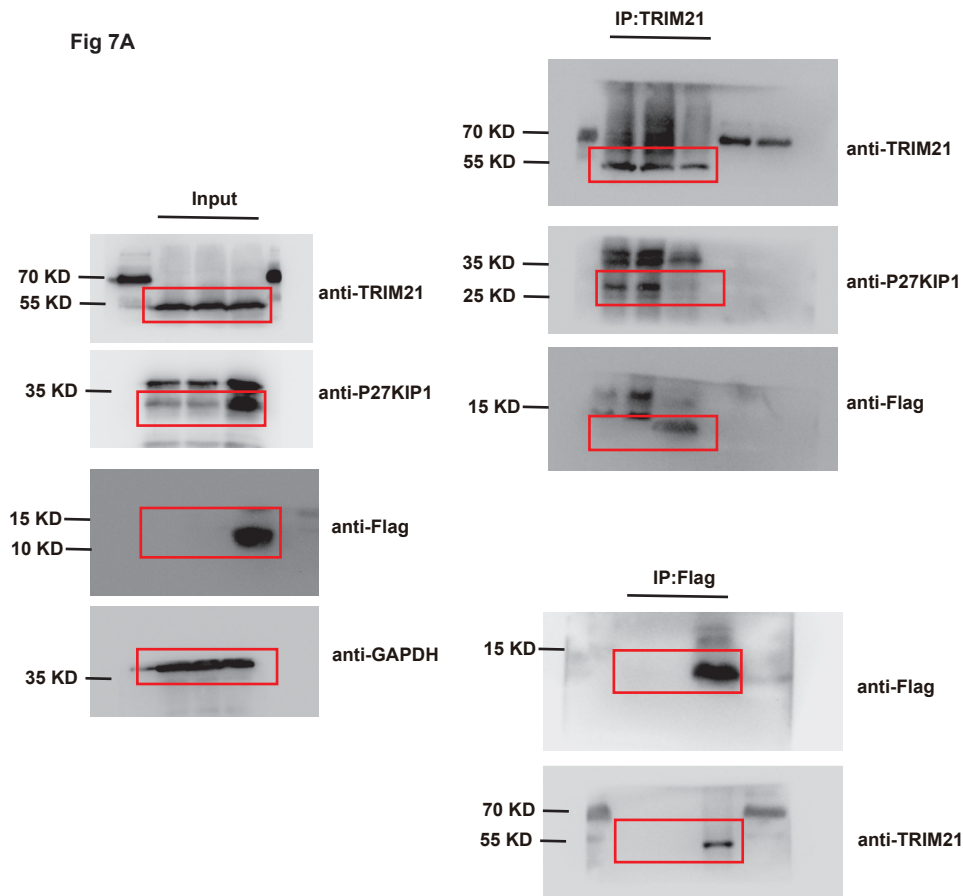

Supplement: Supplementary file 12 — Source data Fig. 7 [file 44318_2024_359_MOESM12_ESM.zip › Figure 7/Fig 7A/Fig. 7A.pdf]

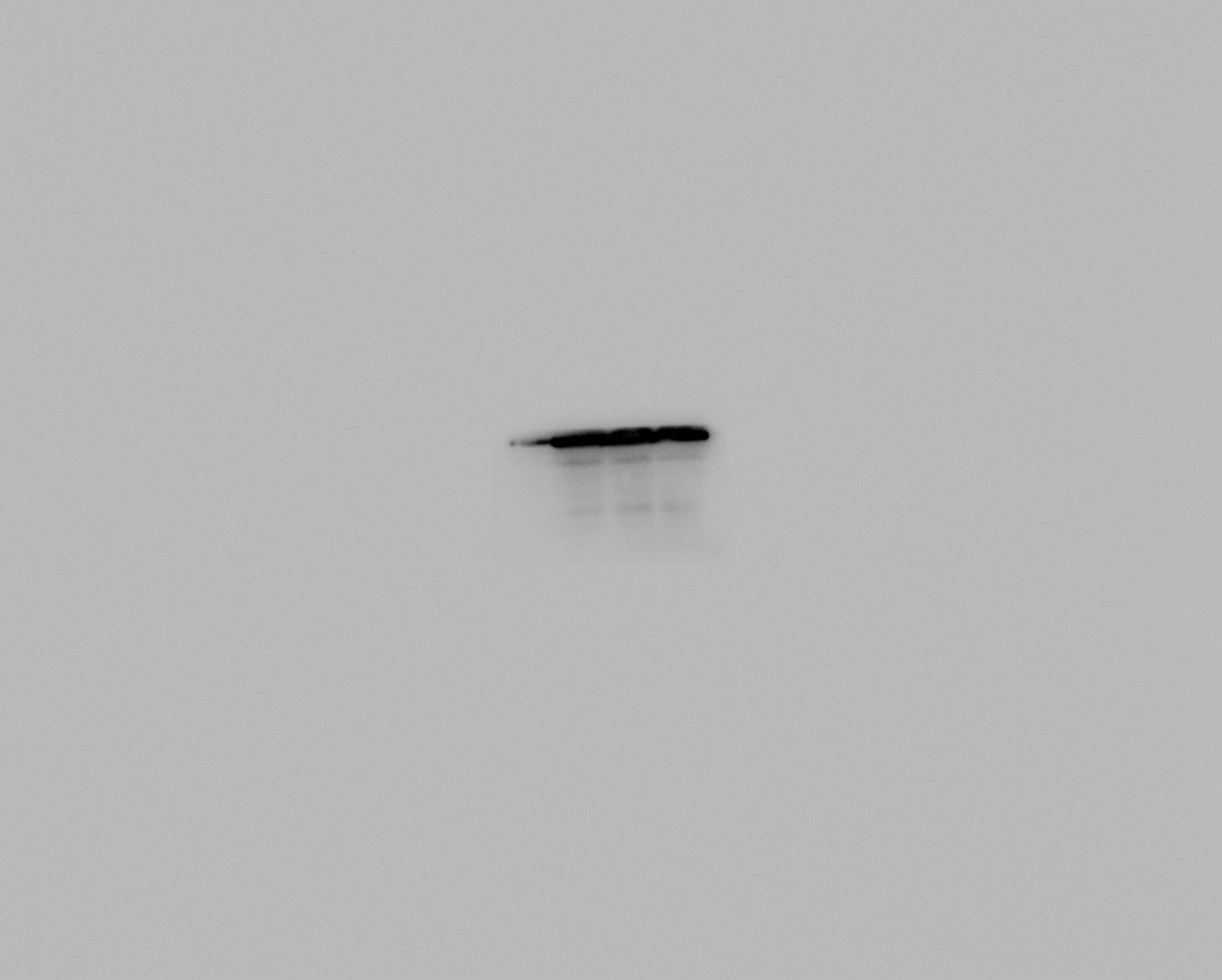

Supplement: Supplementary file 12 — Source data Fig. 7 [file 44318_2024_359_MOESM12_ESM.zip › Figure 7/Fig 7A/input-GAPDH.Tif]

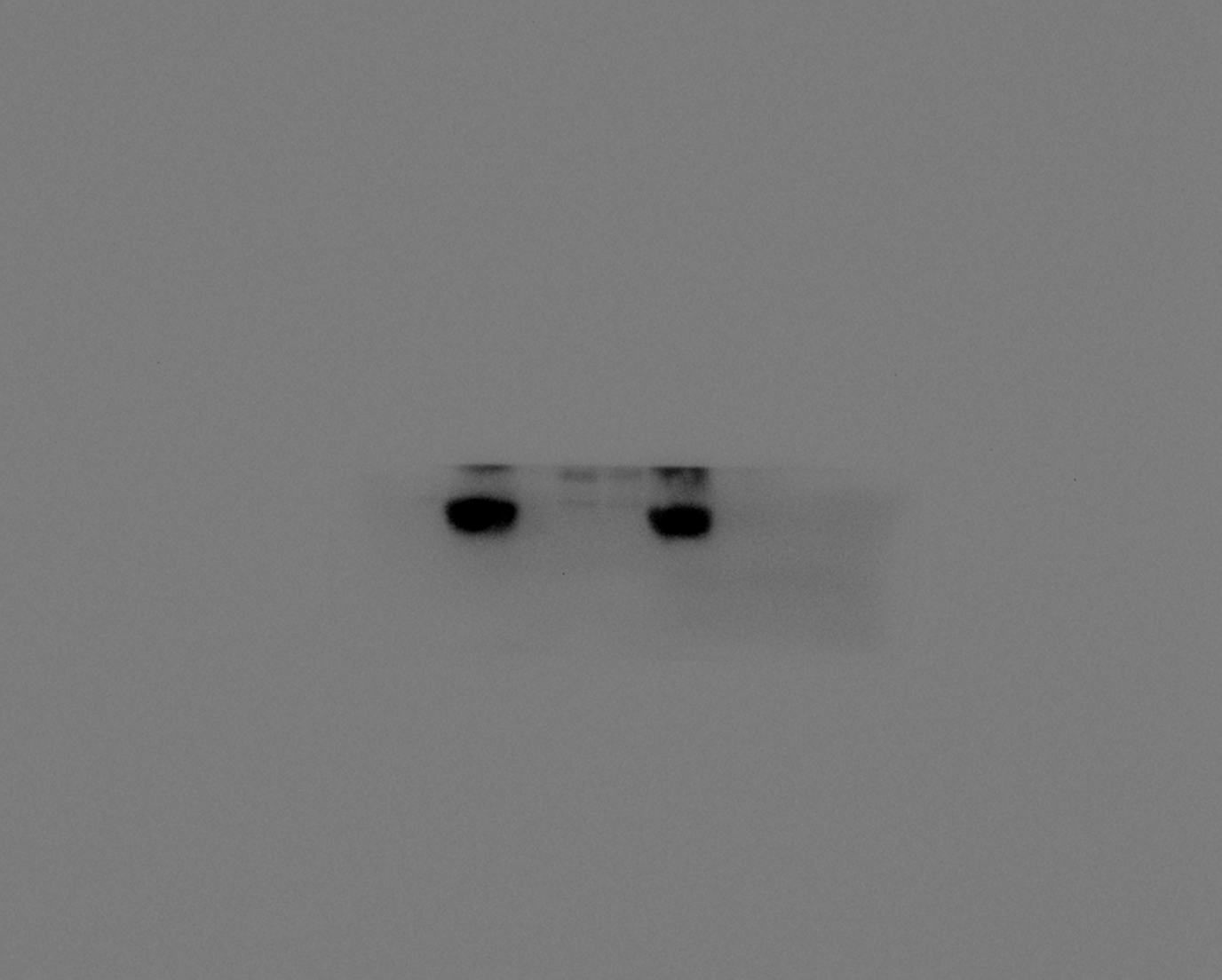

Supplement: Supplementary file 12 — Source data Fig. 7 [file 44318_2024_359_MOESM12_ESM.zip › Figure 7/Fig 7A/input-SPAR.Tif]

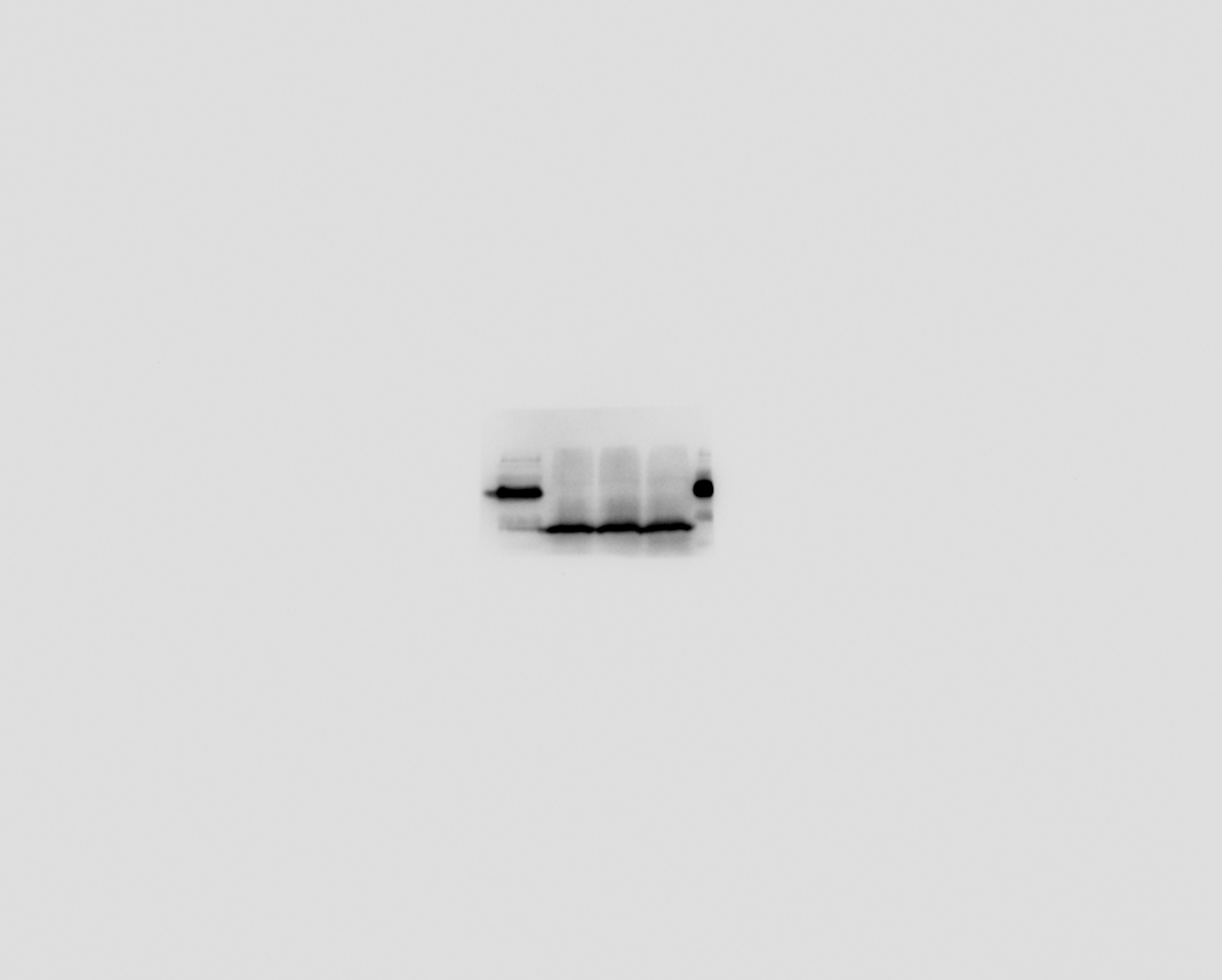

Supplement: Supplementary file 12 — Source data Fig. 7 [file 44318_2024_359_MOESM12_ESM.zip › Figure 7/Fig 7A/input-TRIM21.Tif]

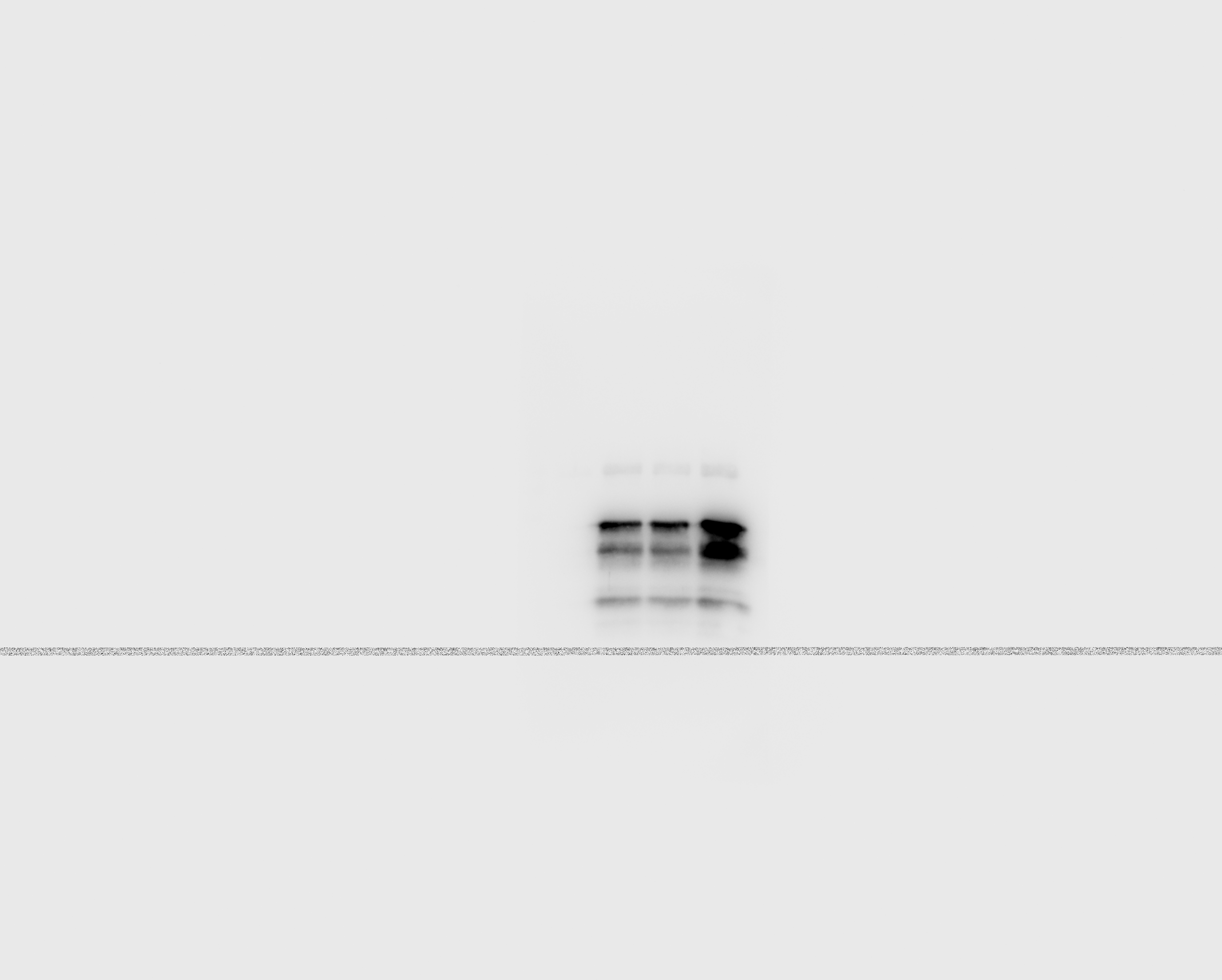

Supplement: Supplementary file 12 — Source data Fig. 7 [file 44318_2024_359_MOESM12_ESM.zip › Figure 7/Fig 7A/input-p27.Tif]

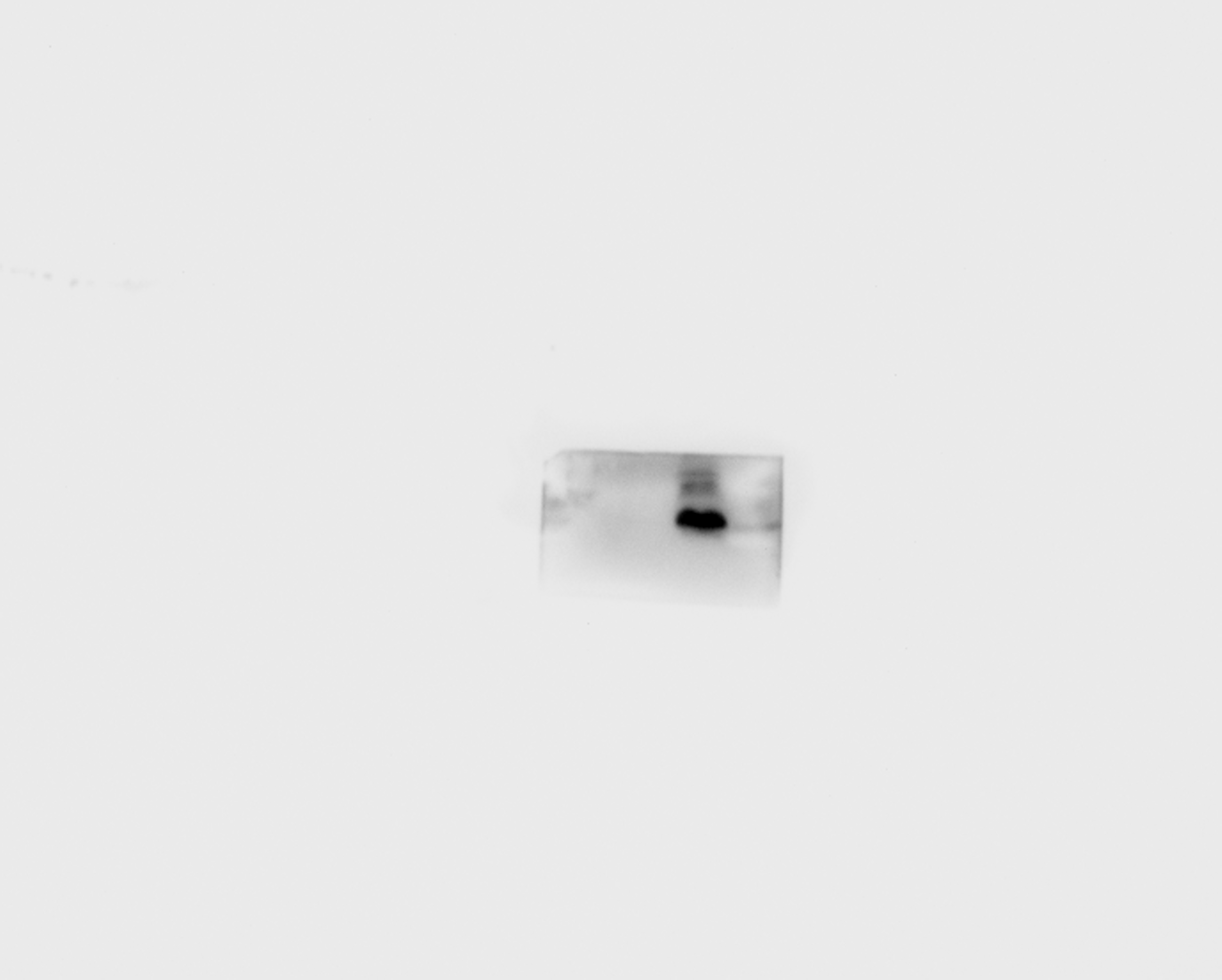

Supplement: Supplementary file 12 — Source data Fig. 7 [file 44318_2024_359_MOESM12_ESM.zip › Figure 7/Fig 7A/ip-SPAR-C wb Flag.Tif]

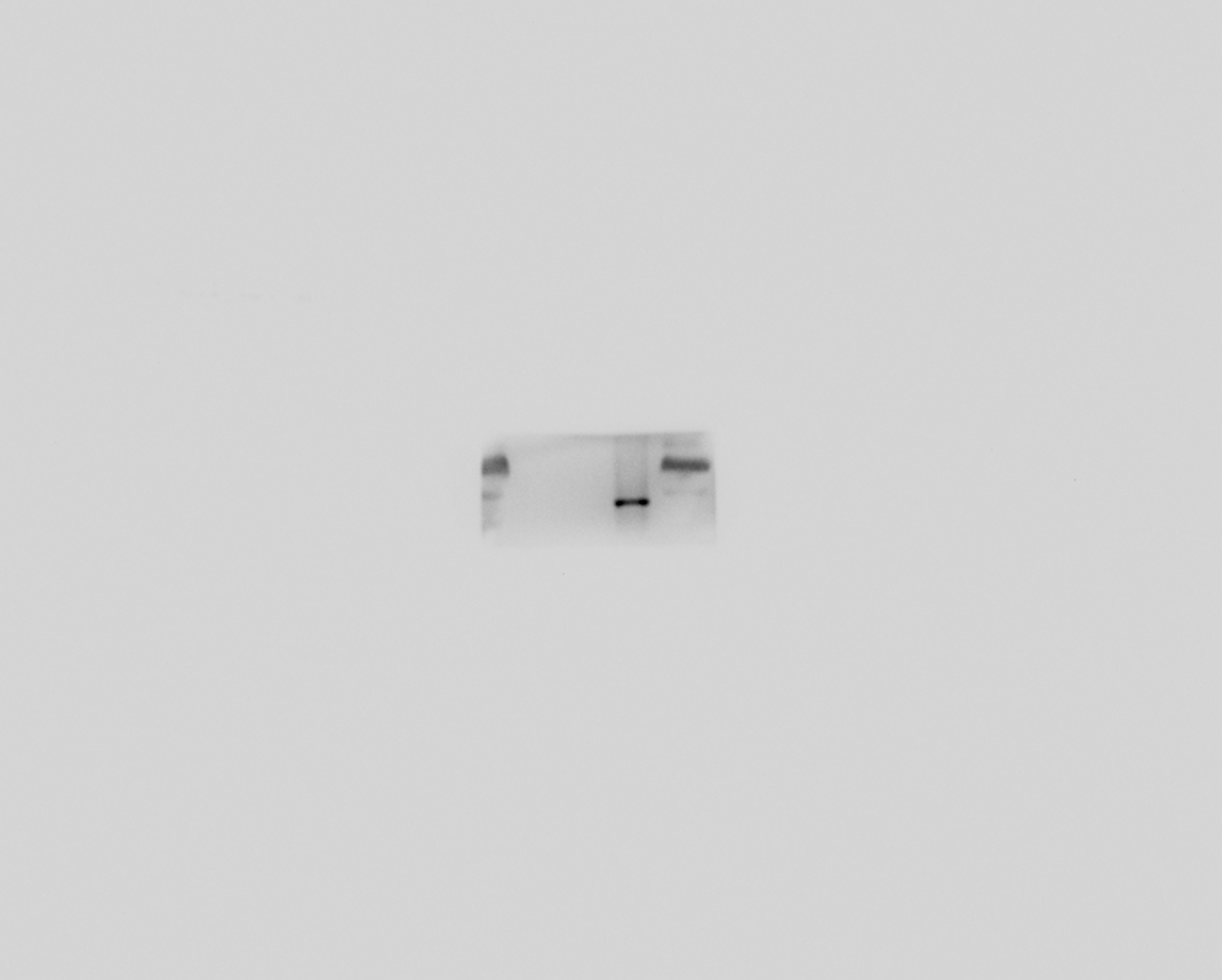

Supplement: Supplementary file 12 — Source data Fig. 7 [file 44318_2024_359_MOESM12_ESM.zip › Figure 7/Fig 7A/ip-SPAR-C wb TRIM21.Tif]

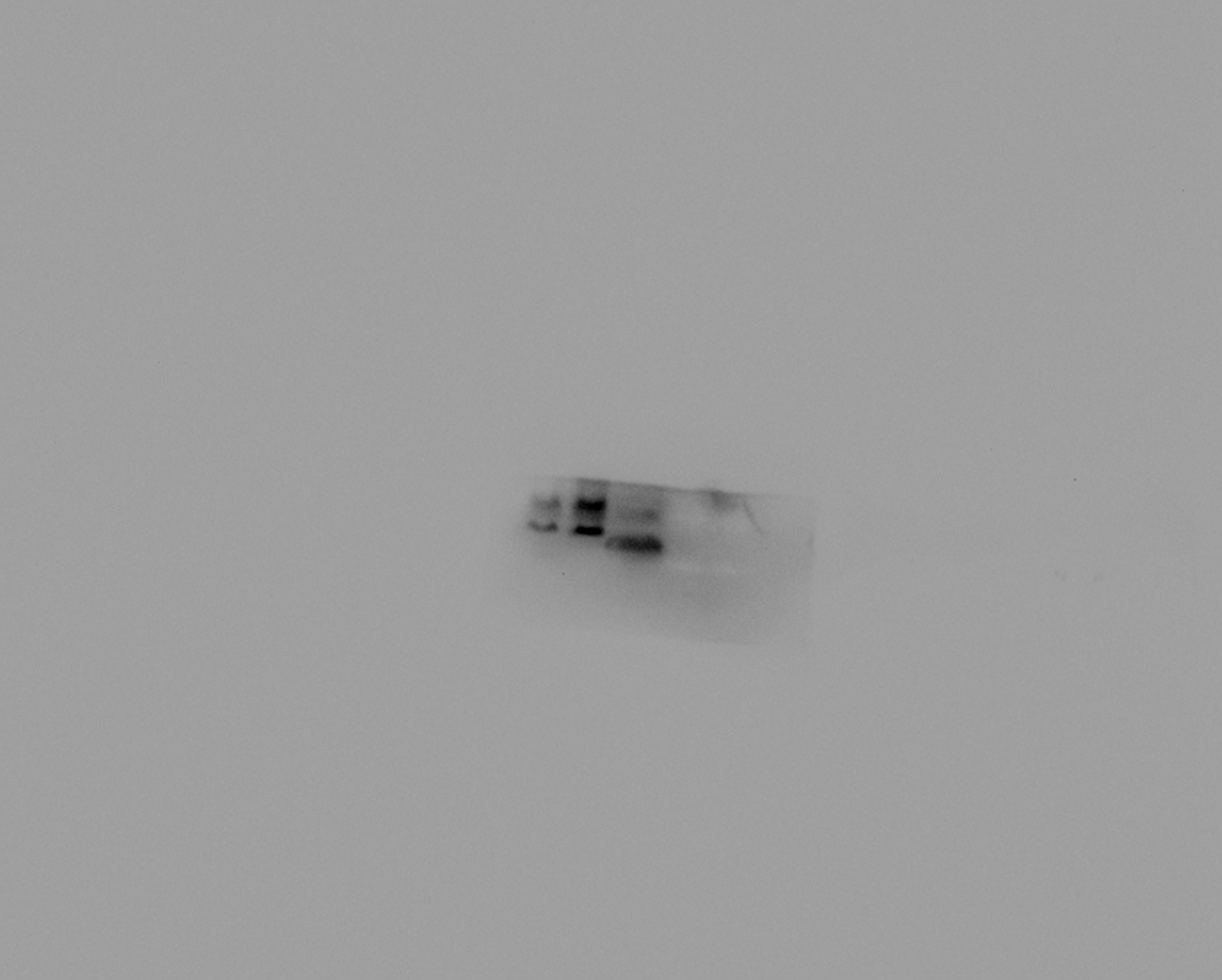

Supplement: Supplementary file 12 — Source data Fig. 7 [file 44318_2024_359_MOESM12_ESM.zip › Figure 7/Fig 7A/ip-TRIM21 wb Flag.Tif]

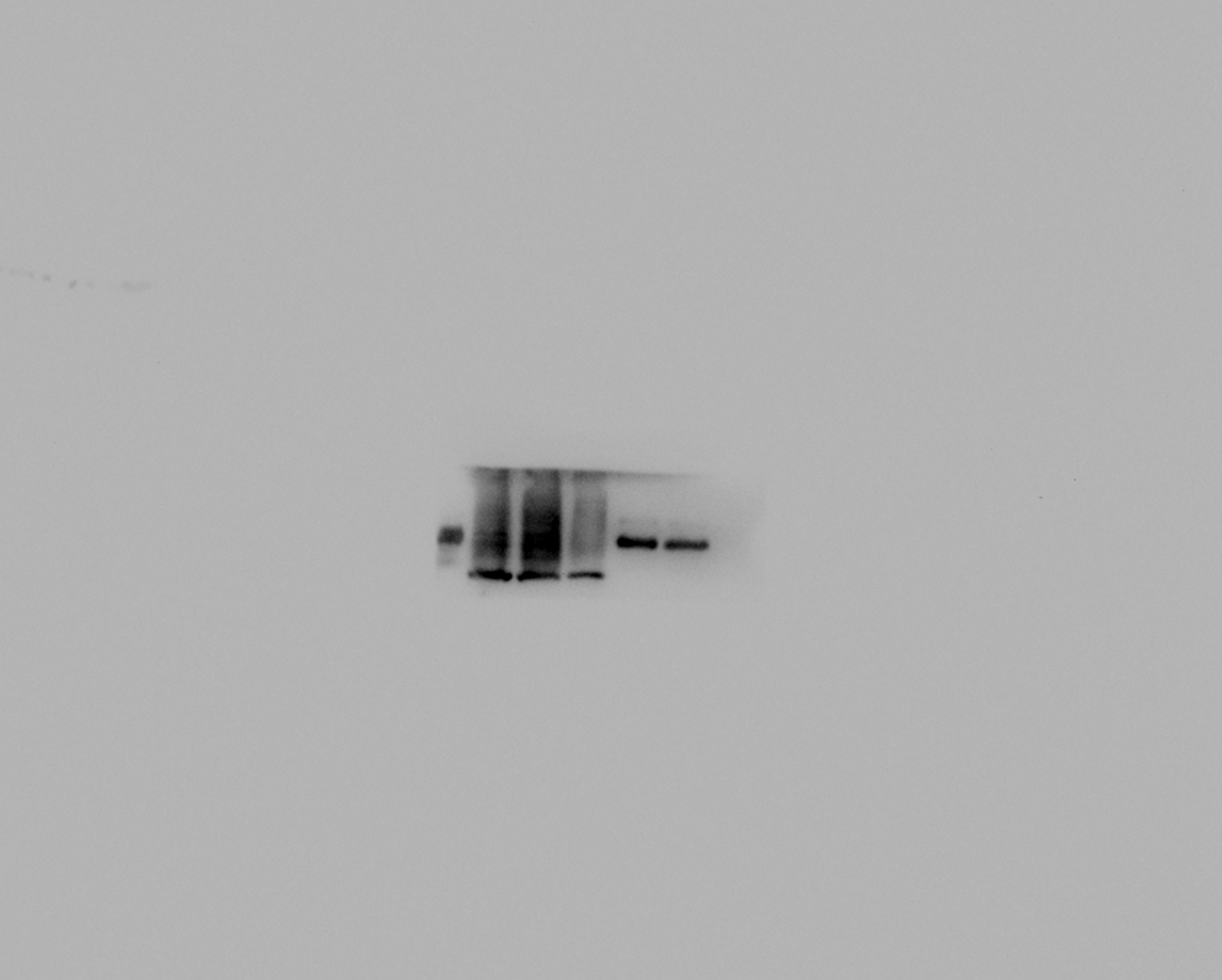

Supplement: Supplementary file 12 — Source data Fig. 7 [file 44318_2024_359_MOESM12_ESM.zip › Figure 7/Fig 7A/ip-TRIM21 wb TRIM21.Tif]

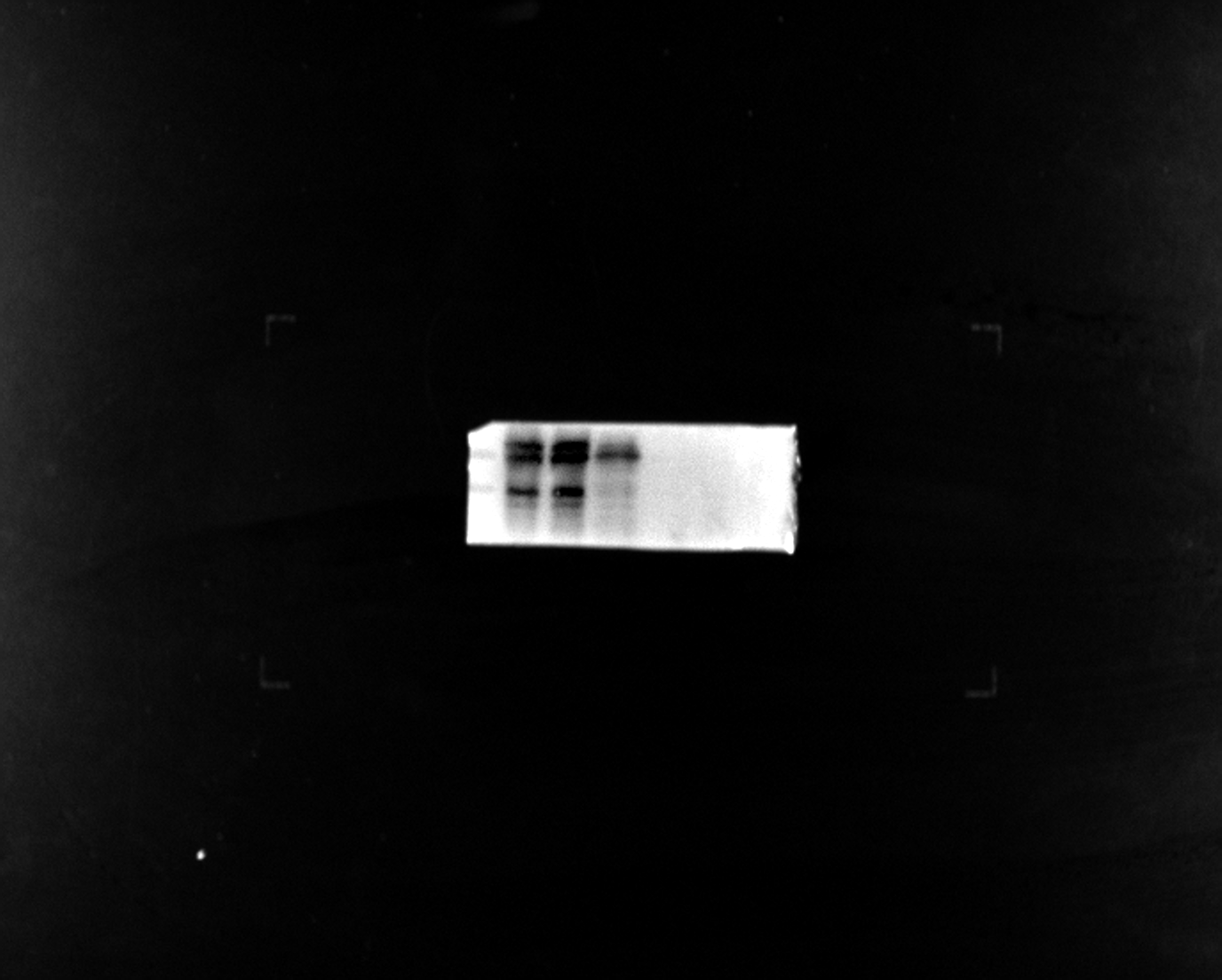

Supplement: Supplementary file 12 — Source data Fig. 7 [file 44318_2024_359_MOESM12_ESM.zip › Figure 7/Fig 7A/ip-TRIM21 wb p27-2.Tif]

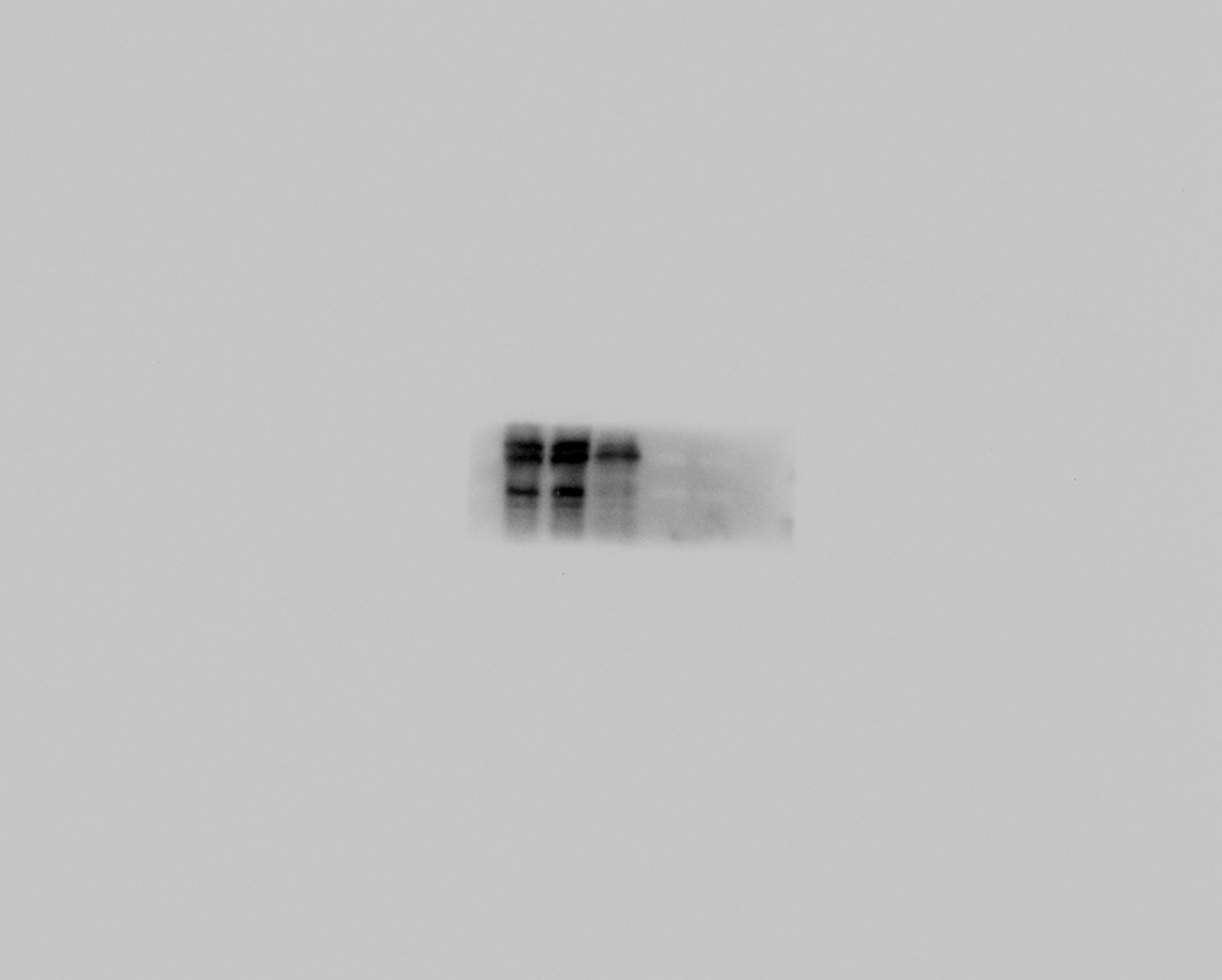

Supplement: Supplementary file 12 — Source data Fig. 7 [file 44318_2024_359_MOESM12_ESM.zip › Figure 7/Fig 7A/ip-TRIM21 wb p27.Tif]

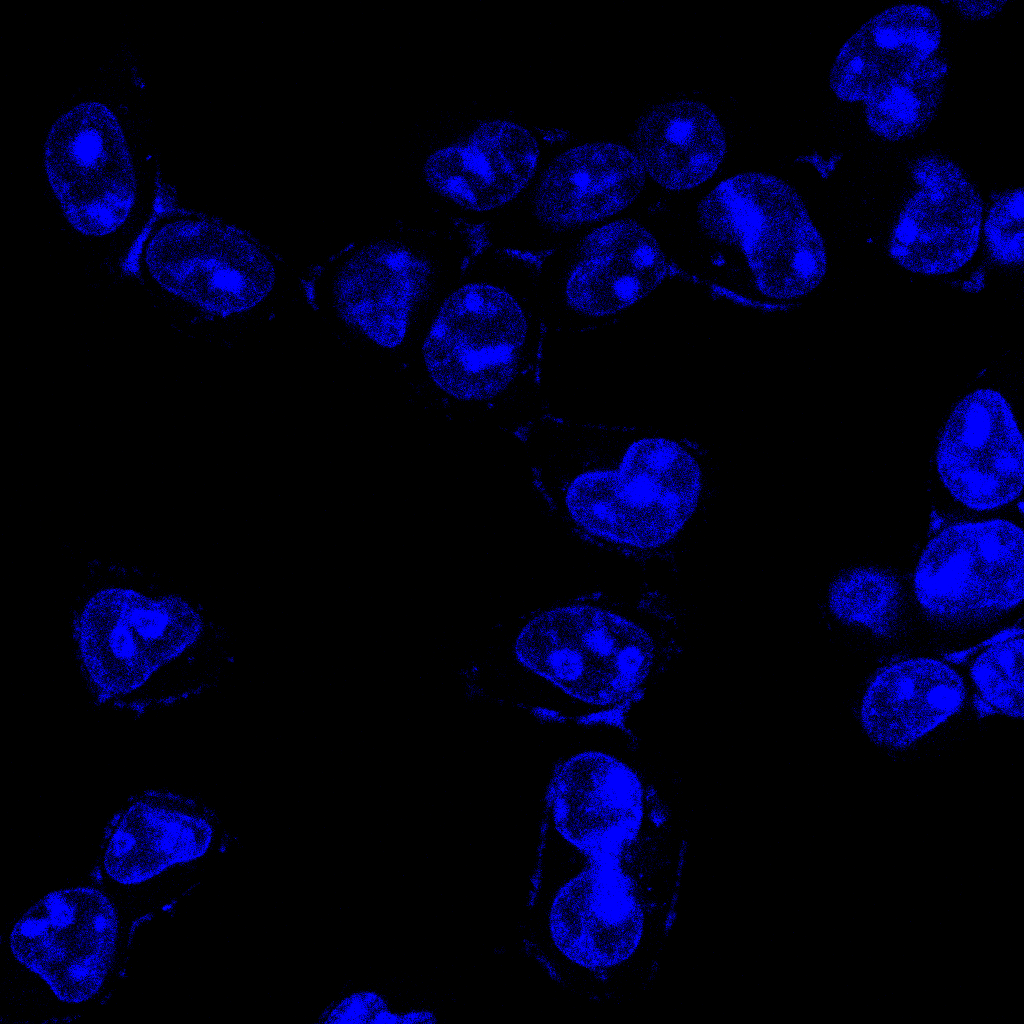

Supplement: Supplementary file 12 — Source data Fig. 7 [file 44318_2024_359_MOESM12_ESM.zip › Figure 7/Fig 7B/ATG/ATG-Hoechst.tif]

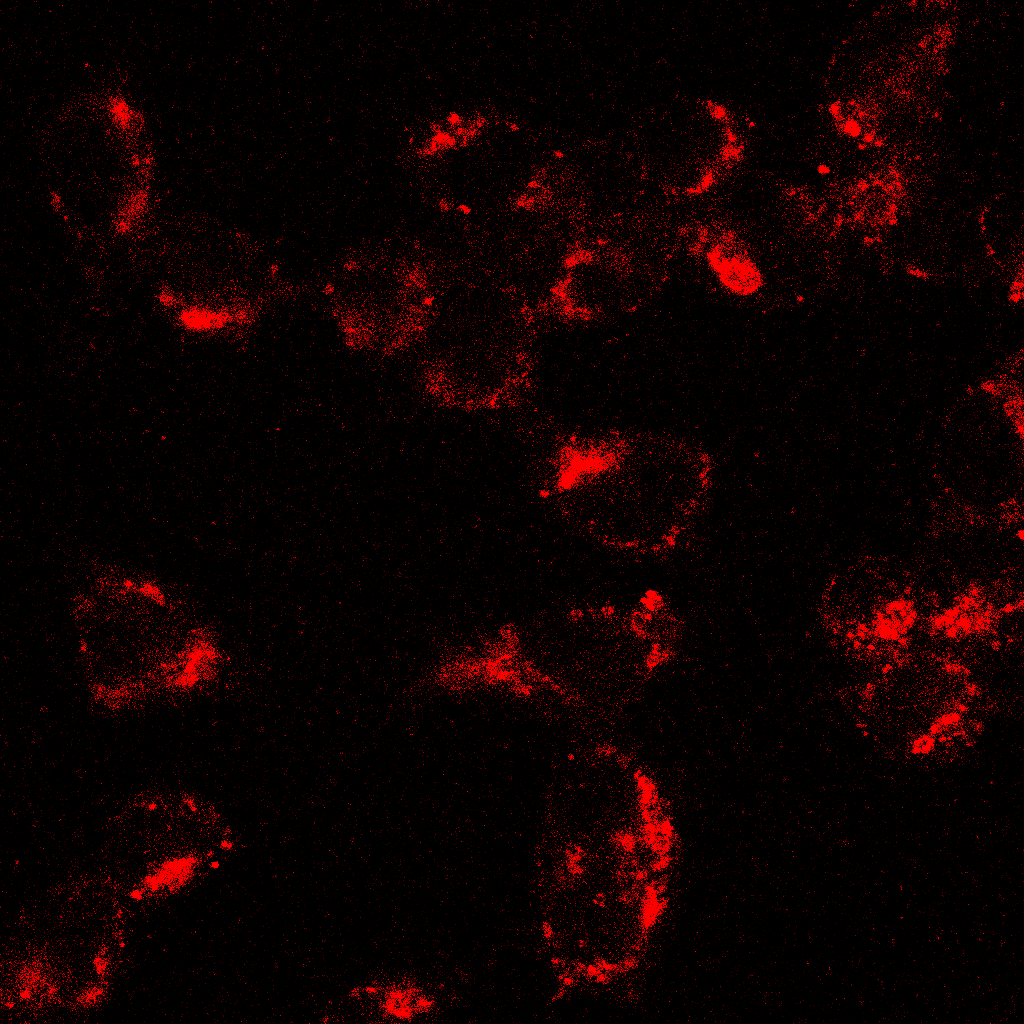

Supplement: Supplementary file 12 — Source data Fig. 7 [file 44318_2024_359_MOESM12_ESM.zip › Figure 7/Fig 7B/ATG/ATG-LAMP1.tif]

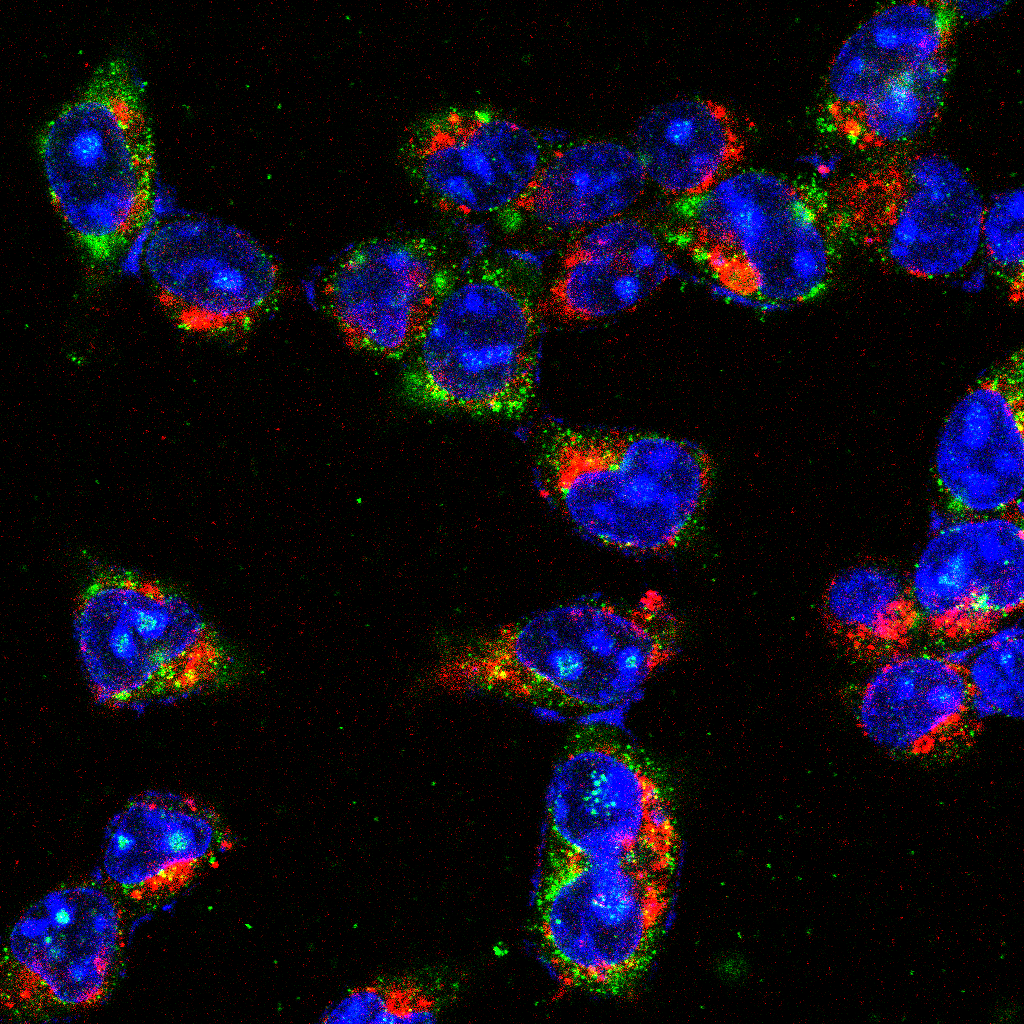

Supplement: Supplementary file 12 — Source data Fig. 7 [file 44318_2024_359_MOESM12_ESM.zip › Figure 7/Fig 7B/ATG/ATG-merge.tif]

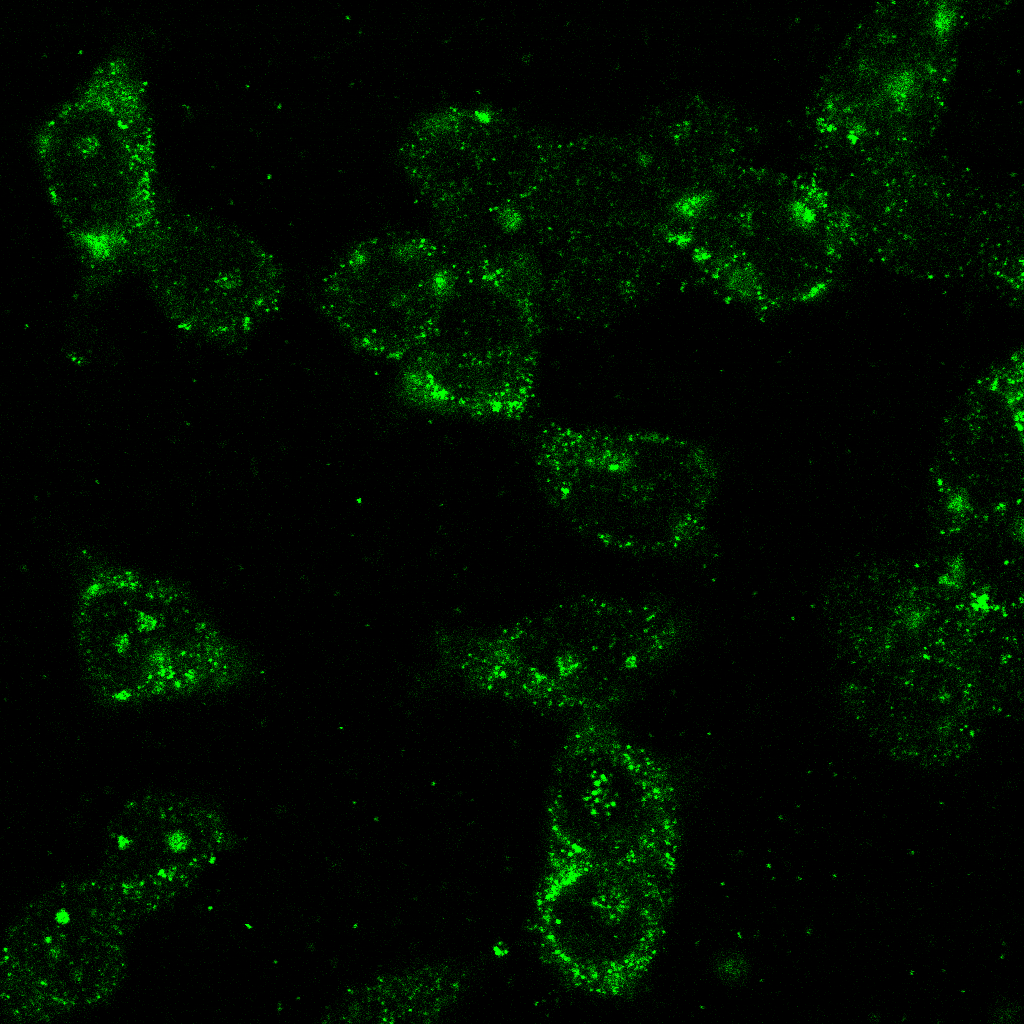

Supplement: Supplementary file 12 — Source data Fig. 7 [file 44318_2024_359_MOESM12_ESM.zip › Figure 7/Fig 7B/ATG/ATG-p27.tif]

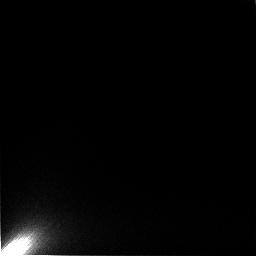

Supplement: Supplementary file 12 — Source data Fig. 7 [file 44318_2024_359_MOESM12_ESM.zip › Figure 7/Fig 7B/ATG/Pearson correlation .tif]

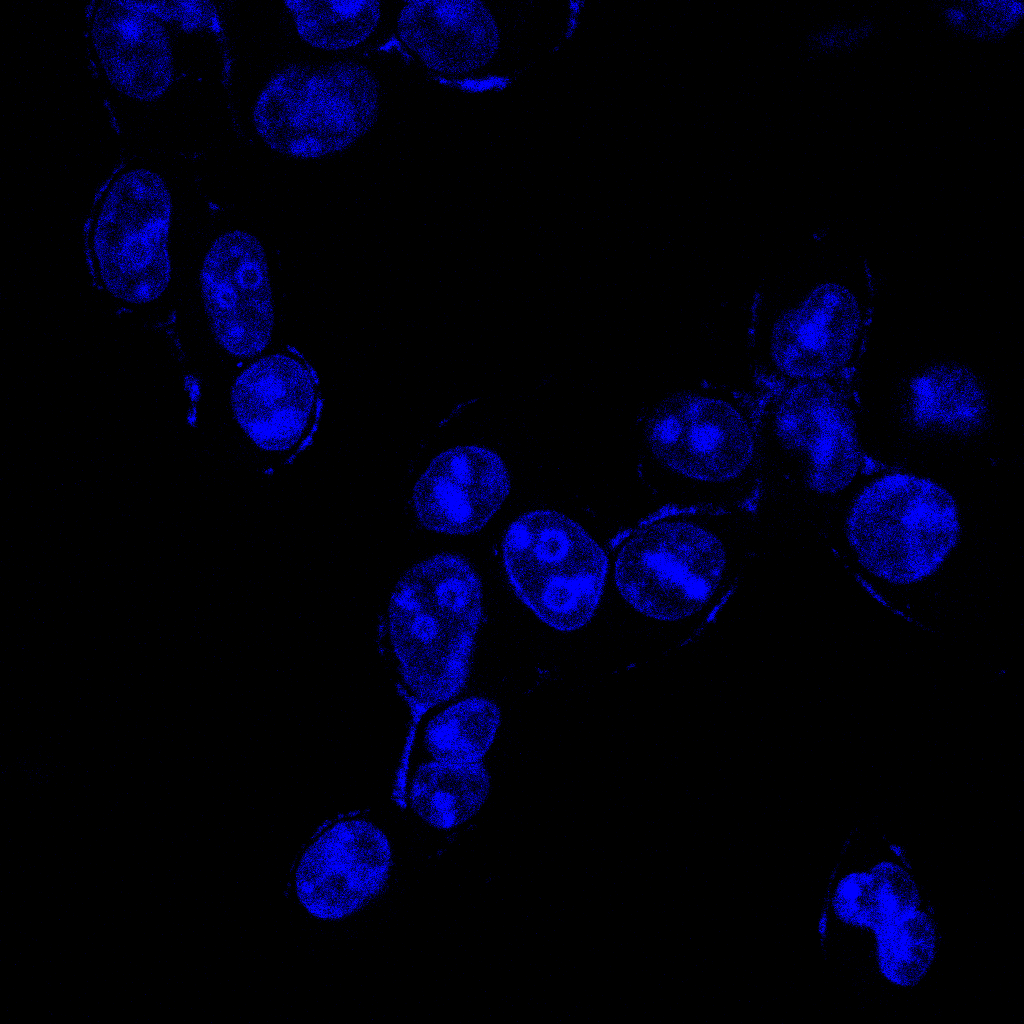

Supplement: Supplementary file 12 — Source data Fig. 7 [file 44318_2024_359_MOESM12_ESM.zip › Figure 7/Fig 7B/C/C-Hoechst.tif]

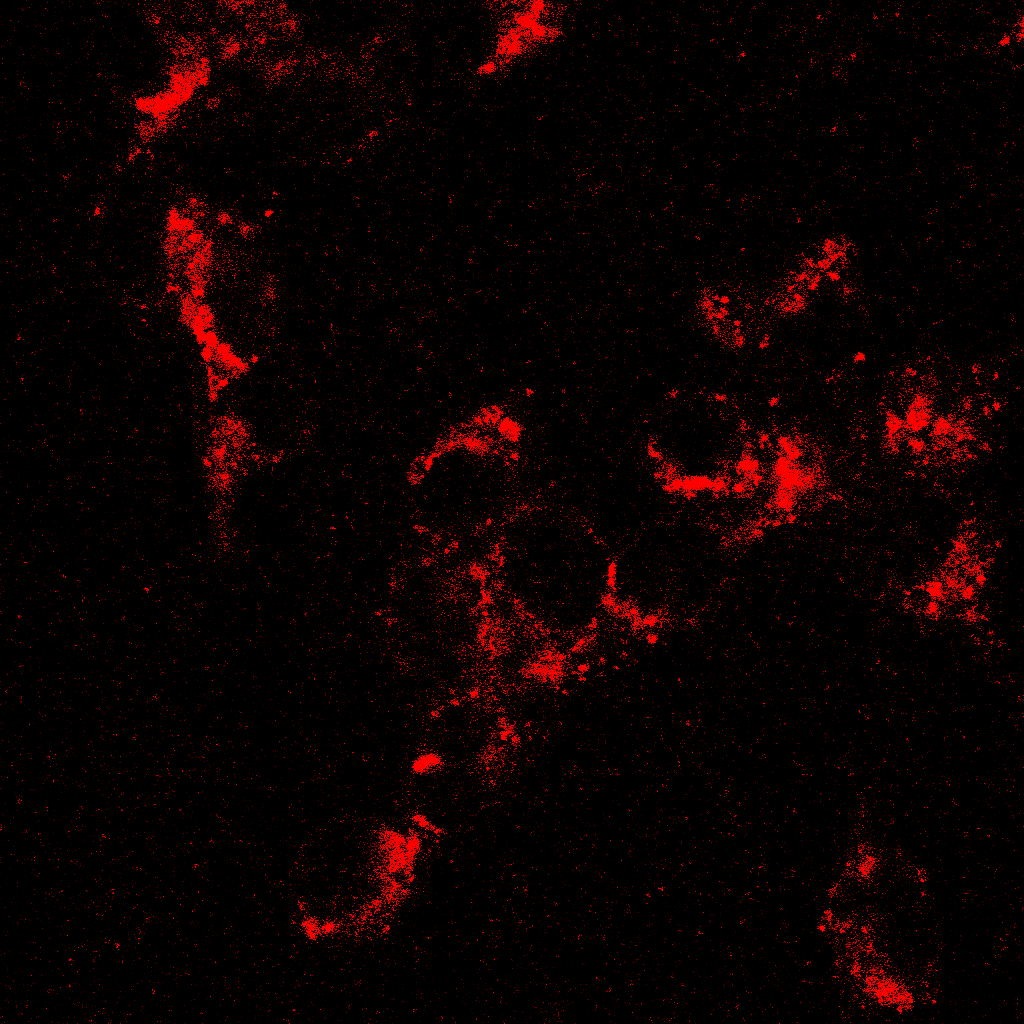

Supplement: Supplementary file 12 — Source data Fig. 7 [file 44318_2024_359_MOESM12_ESM.zip › Figure 7/Fig 7B/C/C-LAMP1.tif]

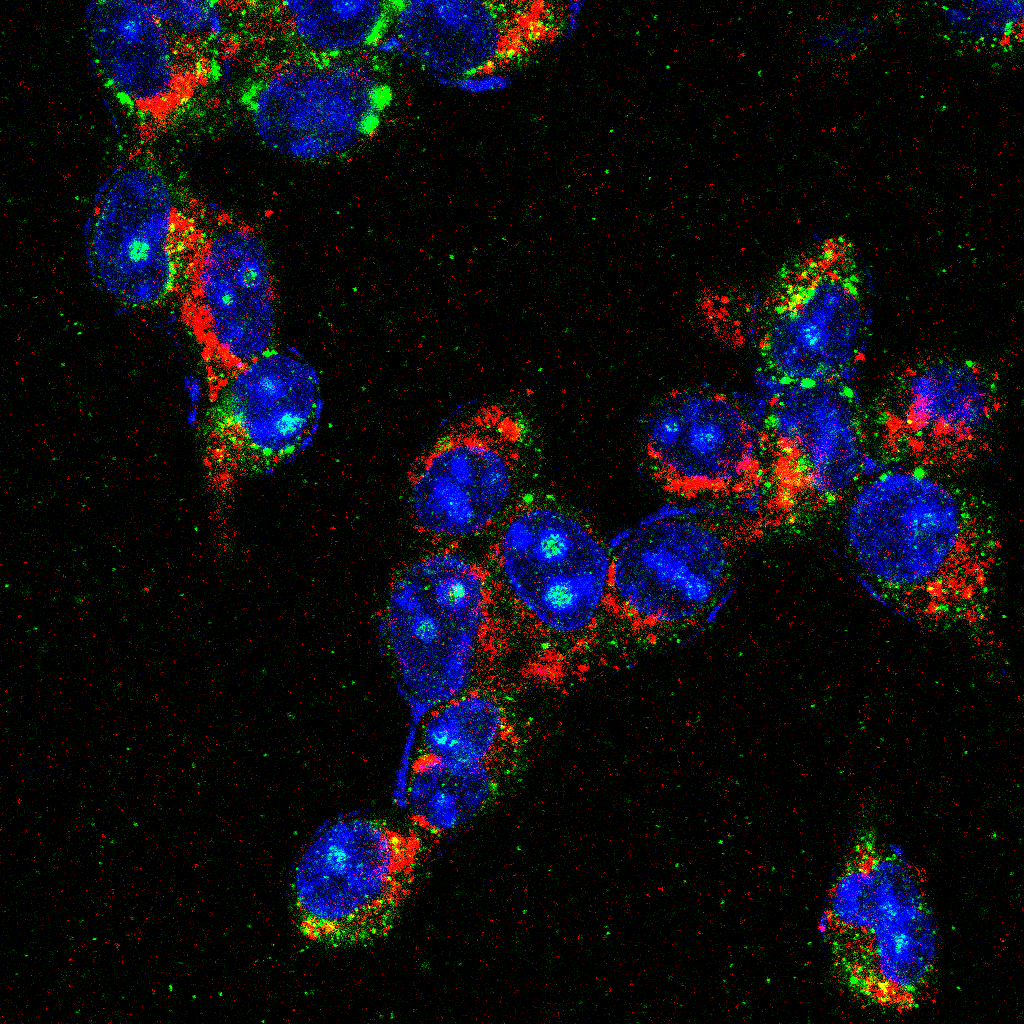

Supplement: Supplementary file 12 — Source data Fig. 7 [file 44318_2024_359_MOESM12_ESM.zip › Figure 7/Fig 7B/C/C-p27-merge.tif]

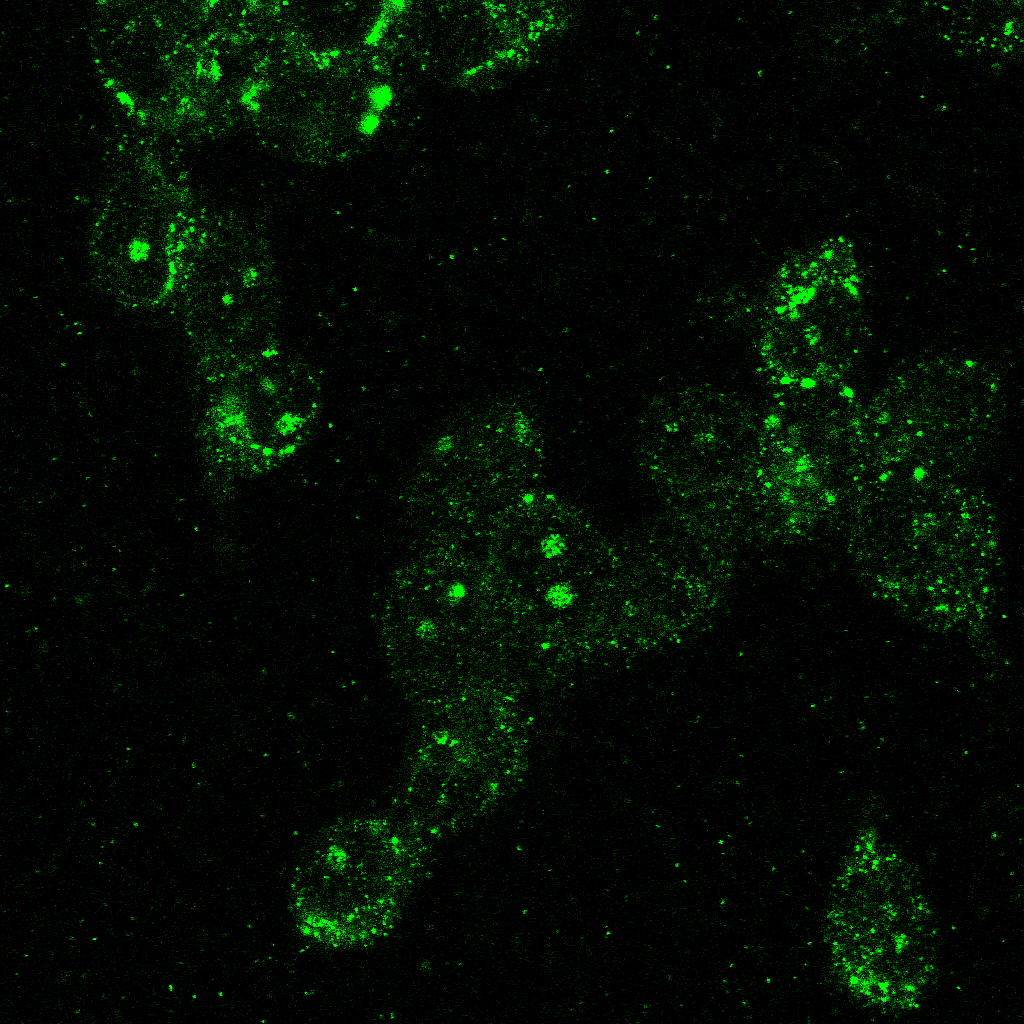

Supplement: Supplementary file 12 — Source data Fig. 7 [file 44318_2024_359_MOESM12_ESM.zip › Figure 7/Fig 7B/C/C-p27.tif]

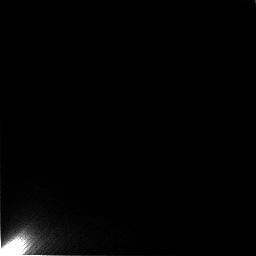

Supplement: Supplementary file 12 — Source data Fig. 7 [file 44318_2024_359_MOESM12_ESM.zip › Figure 7/Fig 7B/C/Pearson correlation.tif]

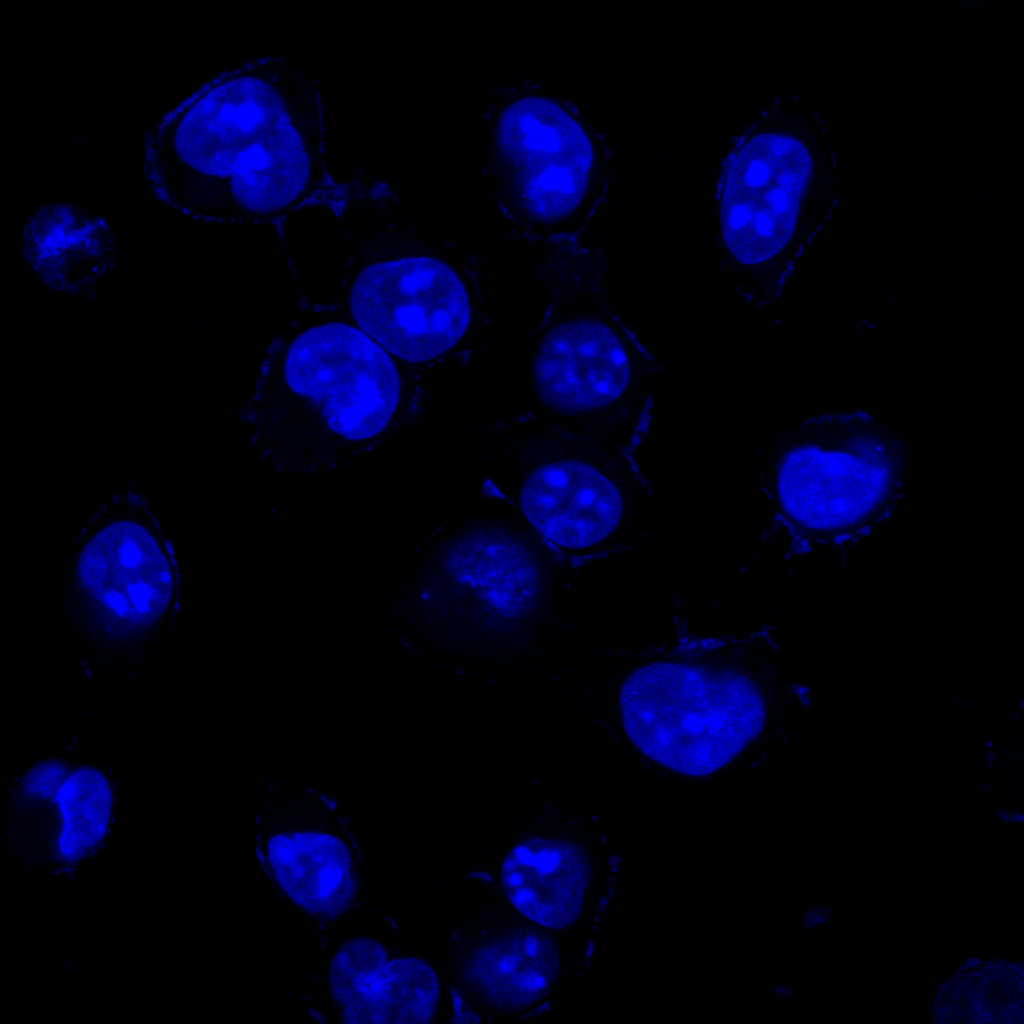

Supplement: Supplementary file 12 — Source data Fig. 7 [file 44318_2024_359_MOESM12_ESM.zip › Figure 7/Fig 7B/N/N-Hoechst.tif]

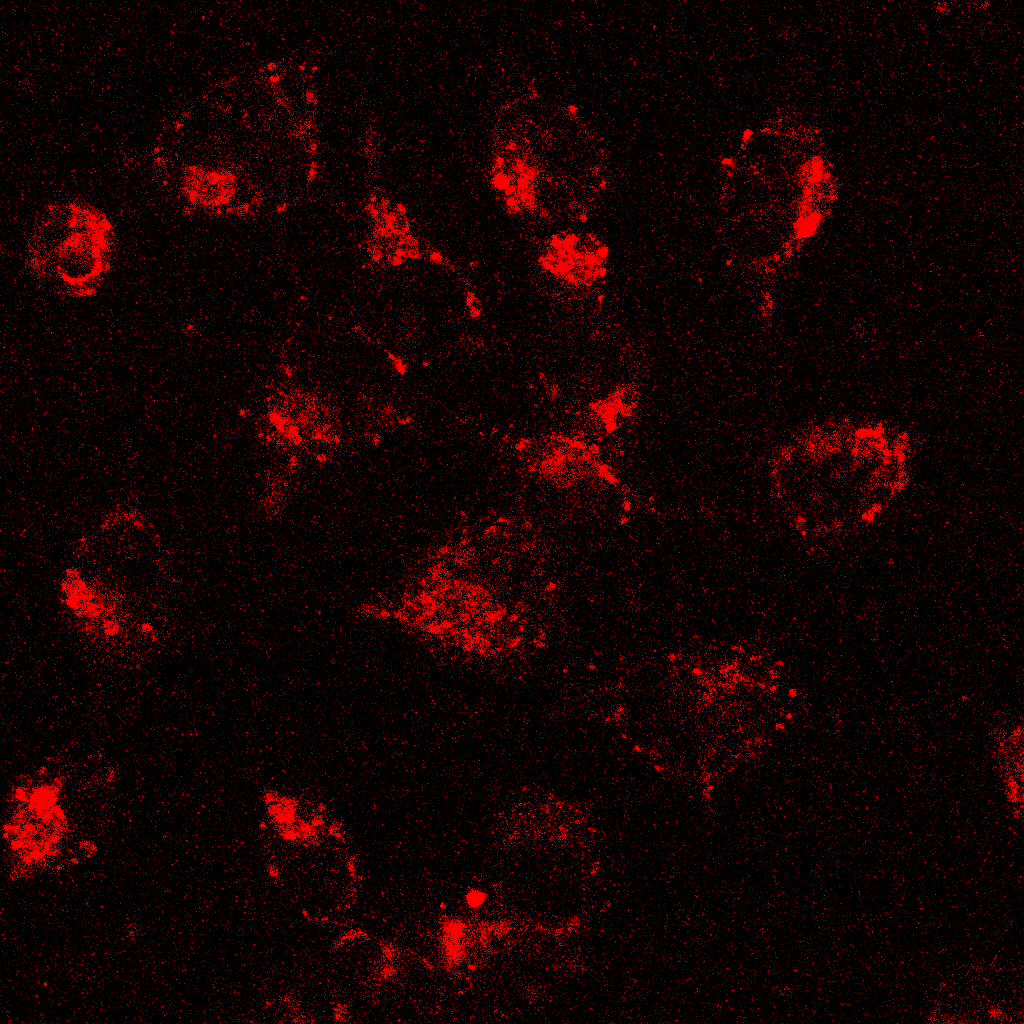

Supplement: Supplementary file 12 — Source data Fig. 7 [file 44318_2024_359_MOESM12_ESM.zip › Figure 7/Fig 7B/N/N-LAMP1.tif]

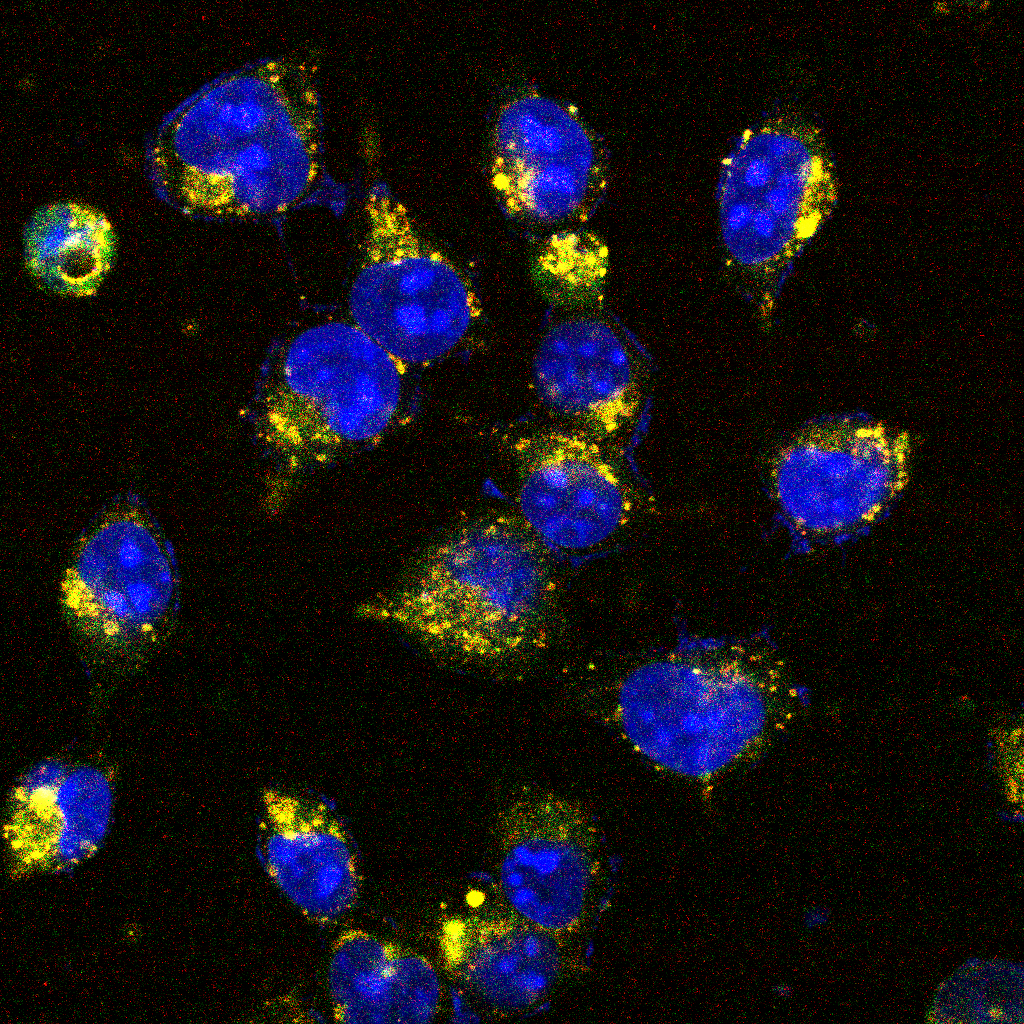

Supplement: Supplementary file 12 — Source data Fig. 7 [file 44318_2024_359_MOESM12_ESM.zip › Figure 7/Fig 7B/N/N-p27-merge.tif]

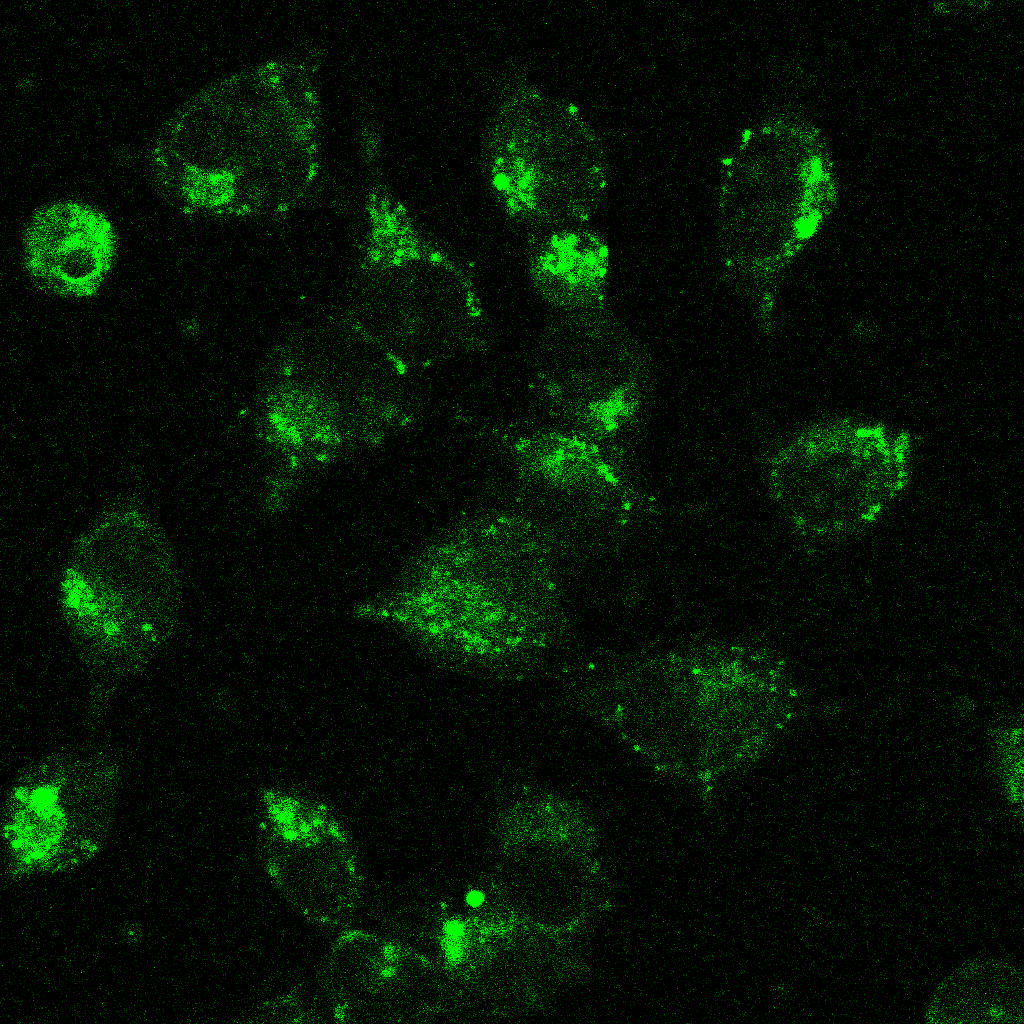

Supplement: Supplementary file 12 — Source data Fig. 7 [file 44318_2024_359_MOESM12_ESM.zip › Figure 7/Fig 7B/N/N-p27.tif]

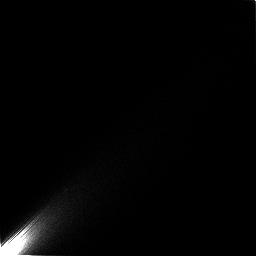

Supplement: Supplementary file 12 — Source data Fig. 7 [file 44318_2024_359_MOESM12_ESM.zip › Figure 7/Fig 7B/N/Pearson correlation.jpg]

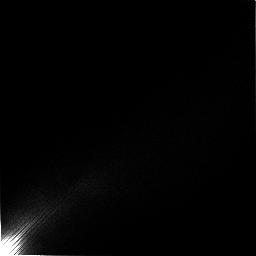

Supplement: Supplementary file 12 — Source data Fig. 7 [file 44318_2024_359_MOESM12_ESM.zip › Figure 7/Fig 7B/TM/Pearson correlation.jpg]

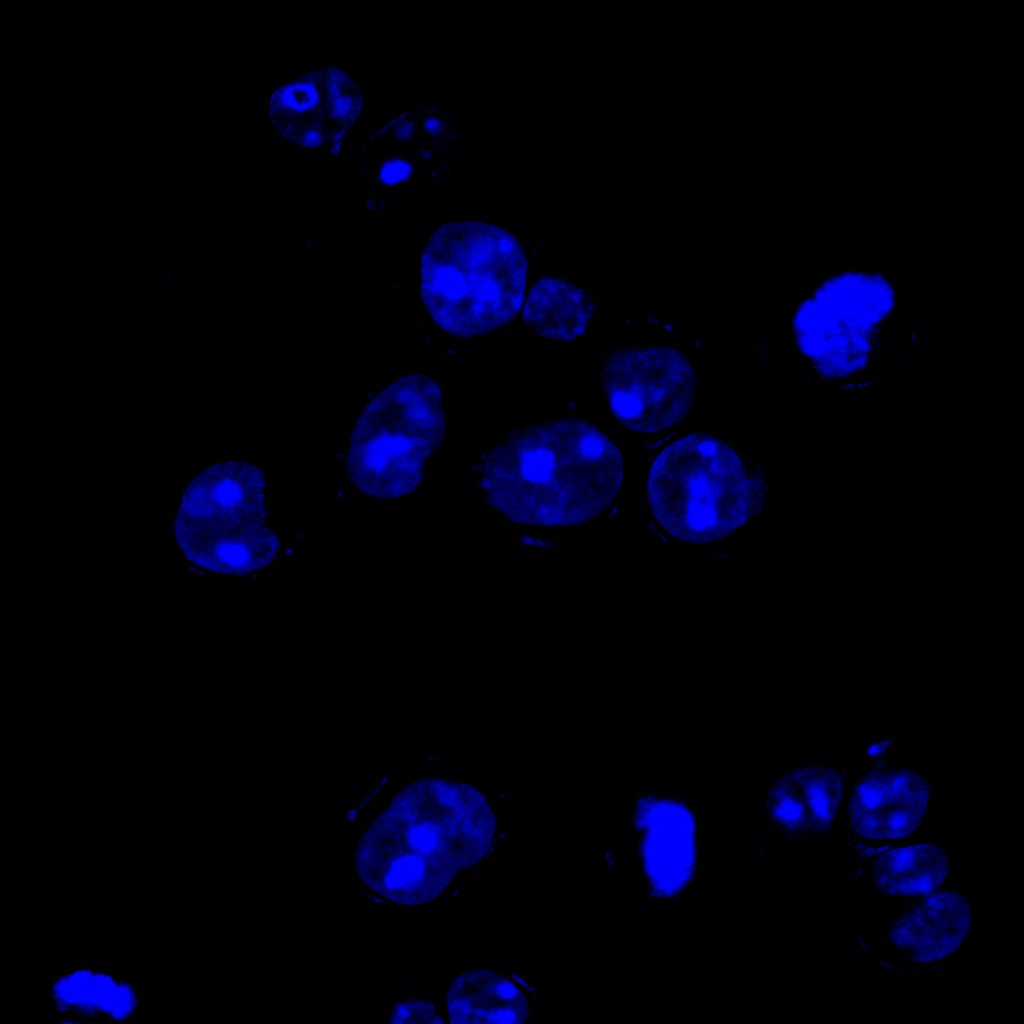

Supplement: Supplementary file 12 — Source data Fig. 7 [file 44318_2024_359_MOESM12_ESM.zip › Figure 7/Fig 7B/TM/TM-Hoechst.tif]

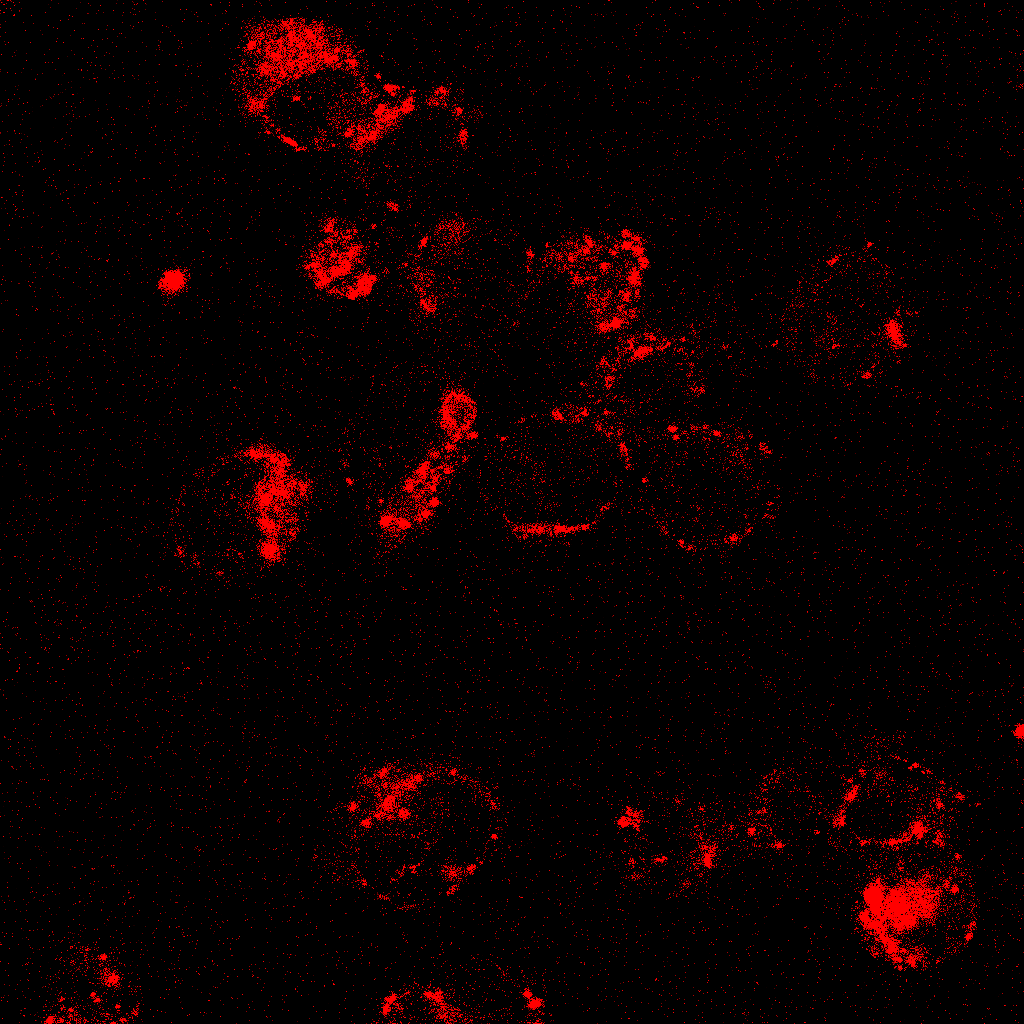

Supplement: Supplementary file 12 — Source data Fig. 7 [file 44318_2024_359_MOESM12_ESM.zip › Figure 7/Fig 7B/TM/TM-LAMP1.tif]

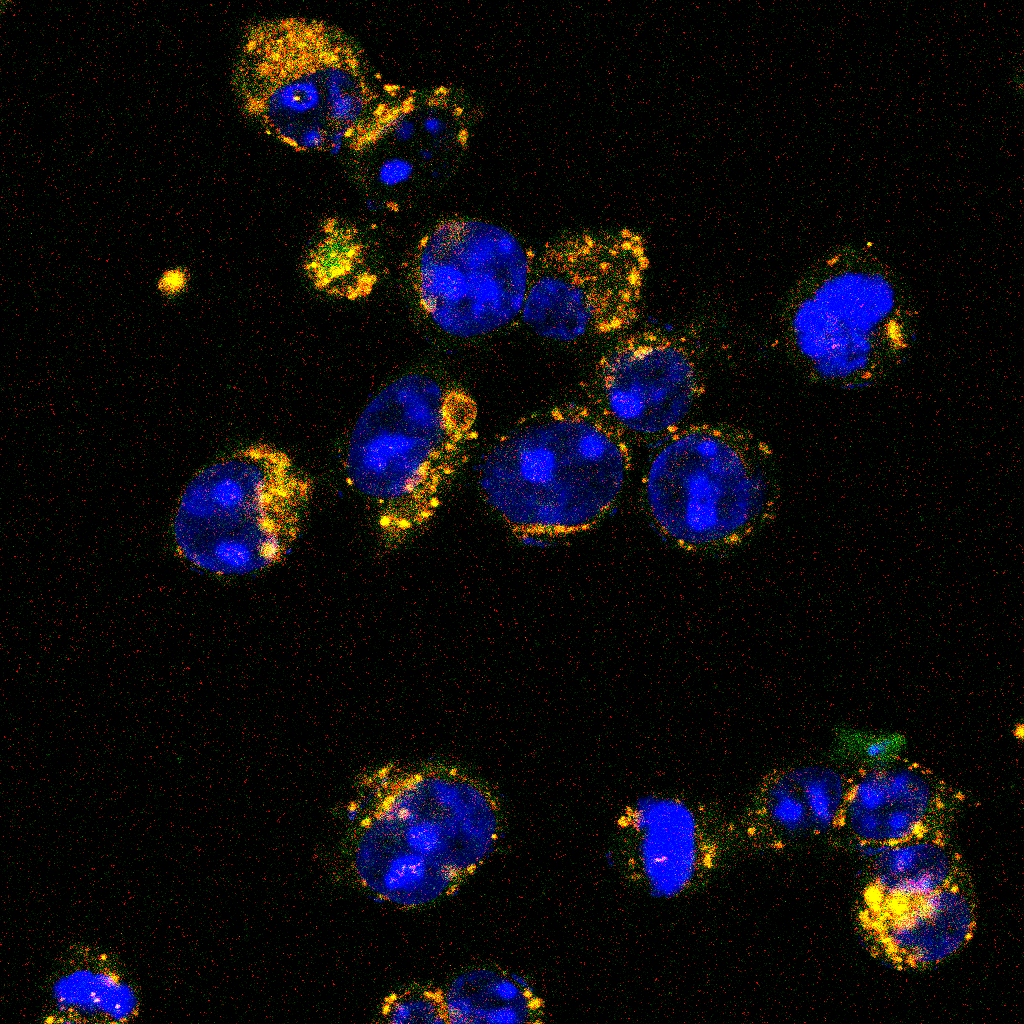

Supplement: Supplementary file 12 — Source data Fig. 7 [file 44318_2024_359_MOESM12_ESM.zip › Figure 7/Fig 7B/TM/TM-p27-merge.tif]

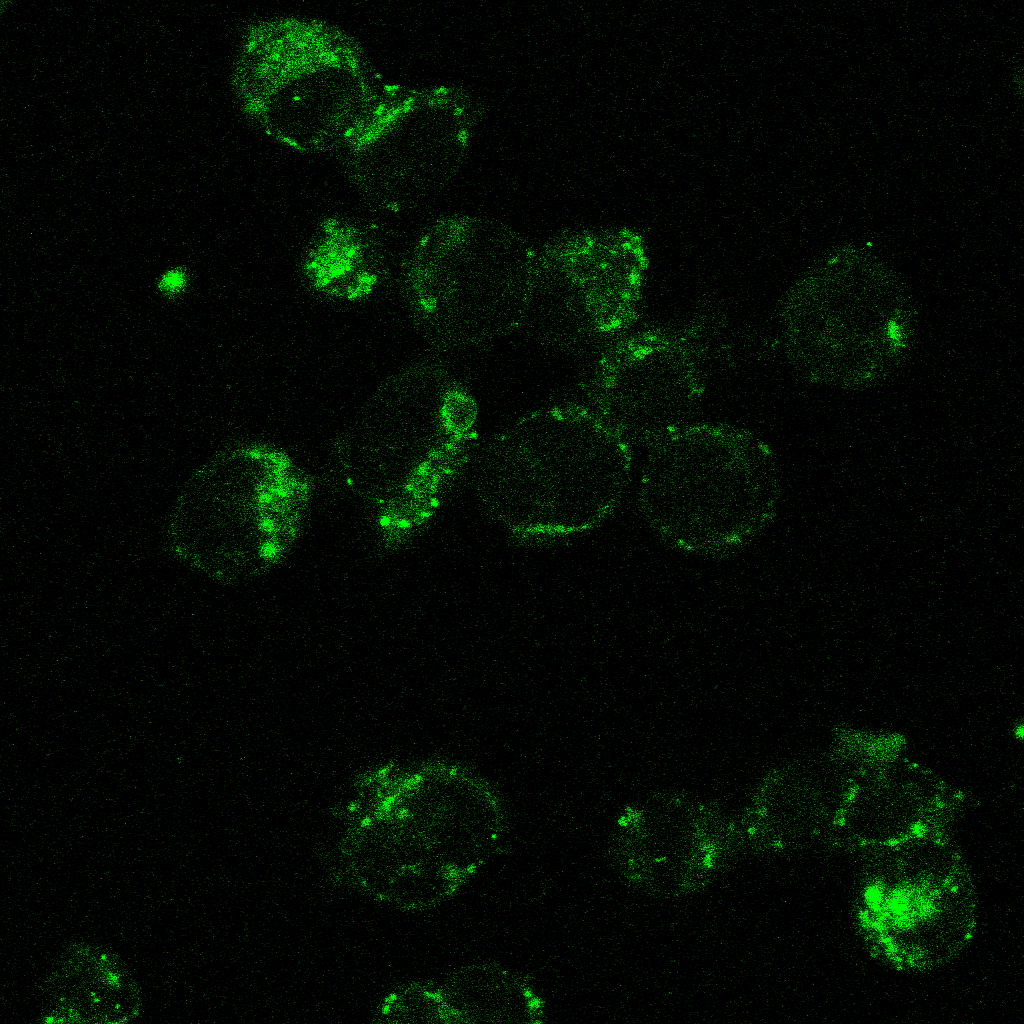

Supplement: Supplementary file 12 — Source data Fig. 7 [file 44318_2024_359_MOESM12_ESM.zip › Figure 7/Fig 7B/TM/TM-p27.tif]

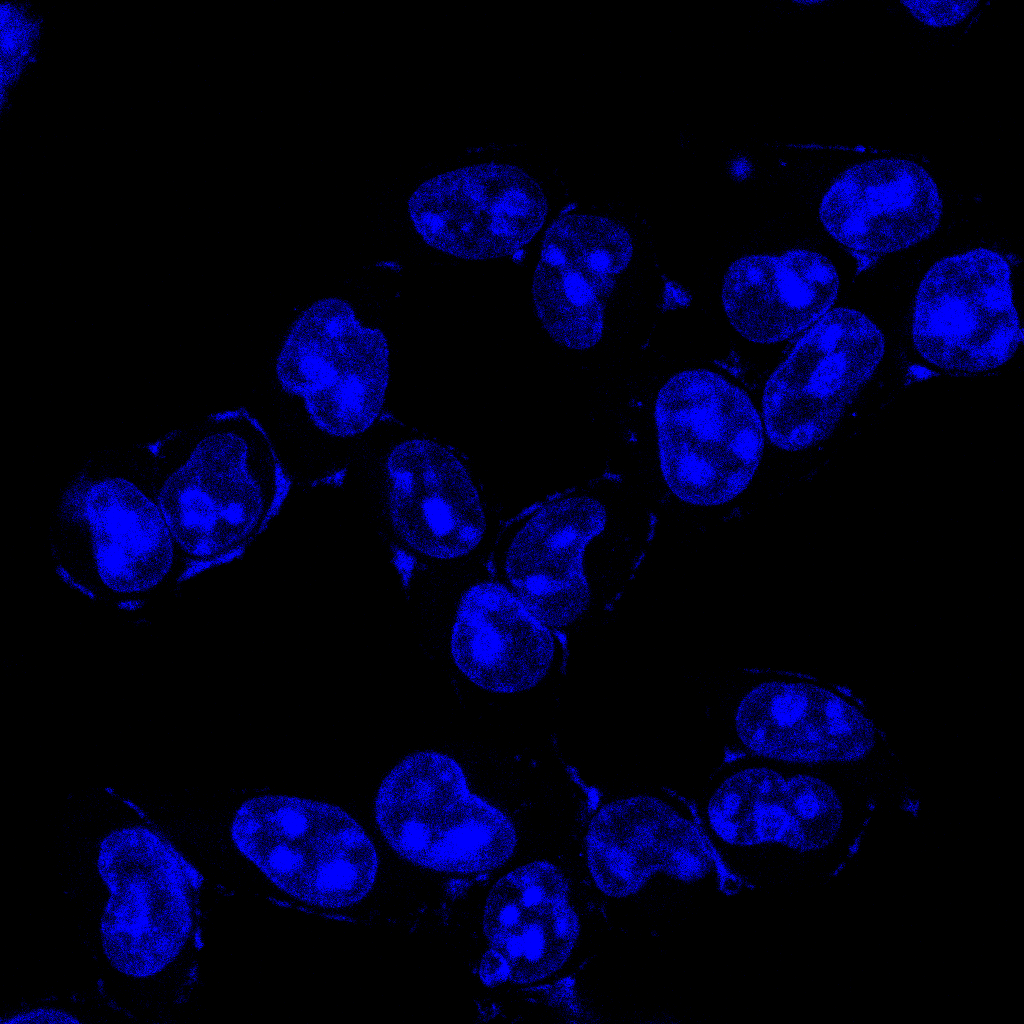

Supplement: Supplementary file 12 — Source data Fig. 7 [file 44318_2024_359_MOESM12_ESM.zip › Figure 7/Fig 7B/Vector Ctrl/Ctrl-Hoechst.tif]

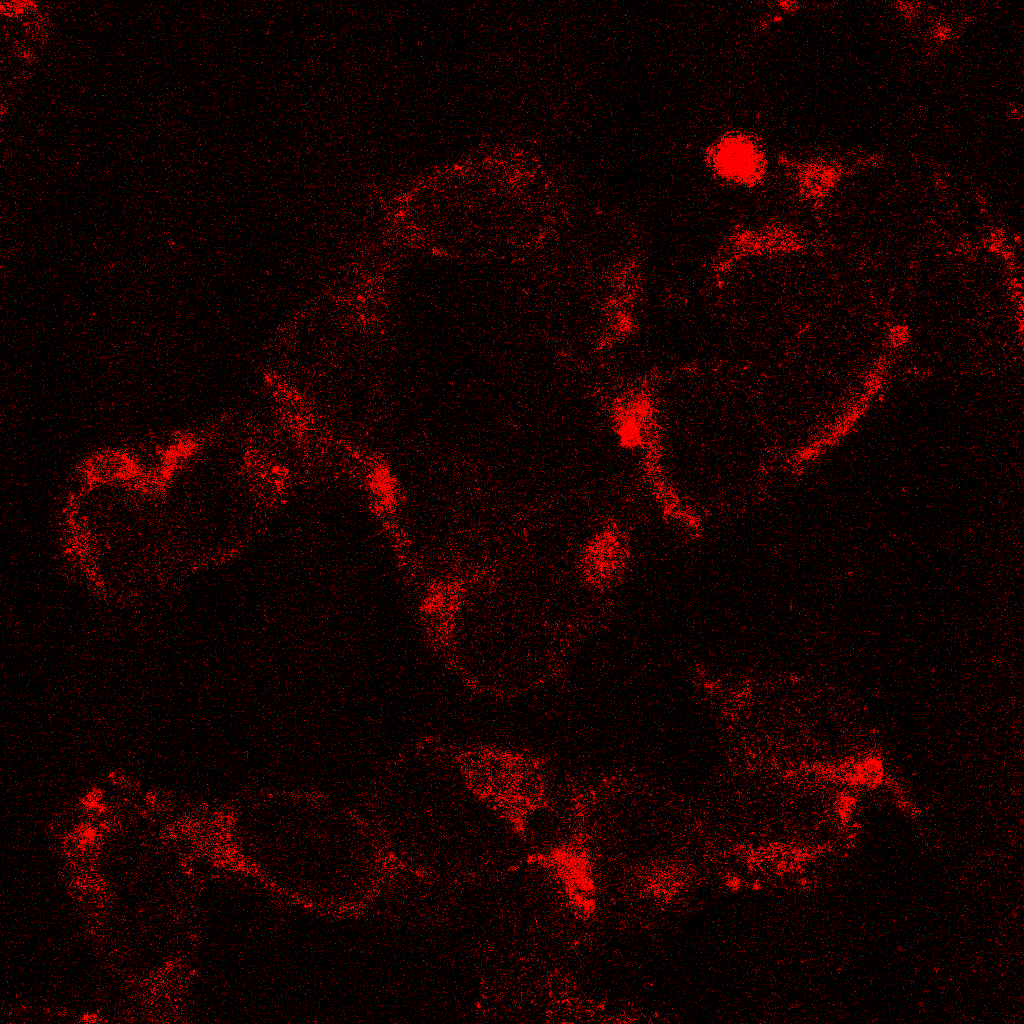

Supplement: Supplementary file 12 — Source data Fig. 7 [file 44318_2024_359_MOESM12_ESM.zip › Figure 7/Fig 7B/Vector Ctrl/Ctrl-LAMP1.tif]

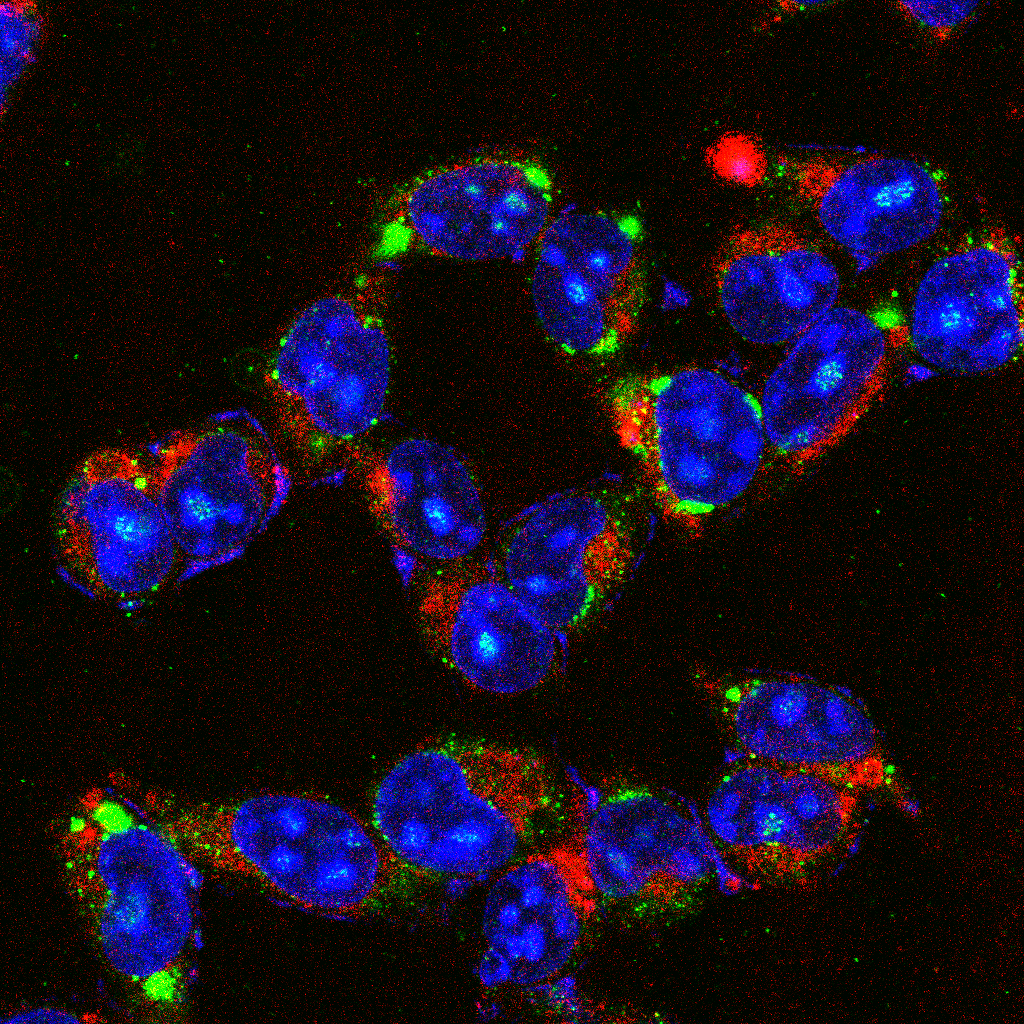

Supplement: Supplementary file 12 — Source data Fig. 7 [file 44318_2024_359_MOESM12_ESM.zip › Figure 7/Fig 7B/Vector Ctrl/Ctrl-merge.tif]

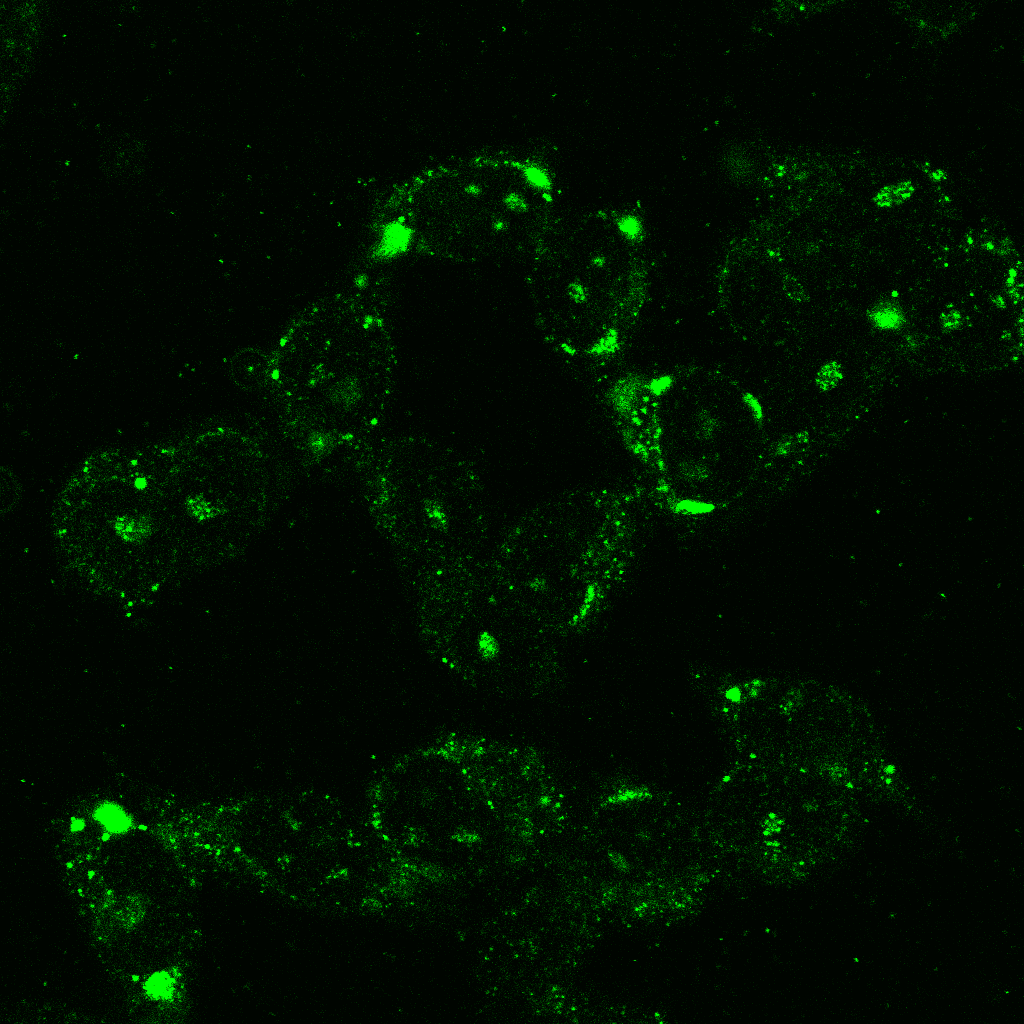

Supplement: Supplementary file 12 — Source data Fig. 7 [file 44318_2024_359_MOESM12_ESM.zip › Figure 7/Fig 7B/Vector Ctrl/Ctrl-p27.tif]

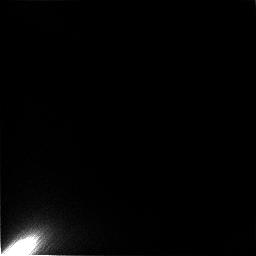

Supplement: Supplementary file 12 — Source data Fig. 7 [file 44318_2024_359_MOESM12_ESM.zip › Figure 7/Fig 7B/Vector Ctrl/Pearson correlation.jpg]

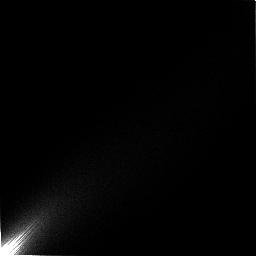

Supplement: Supplementary file 12 — Source data Fig. 7 [file 44318_2024_359_MOESM12_ESM.zip › Figure 7/Fig 7B/hSPAR/Pearson correlation.jpg]

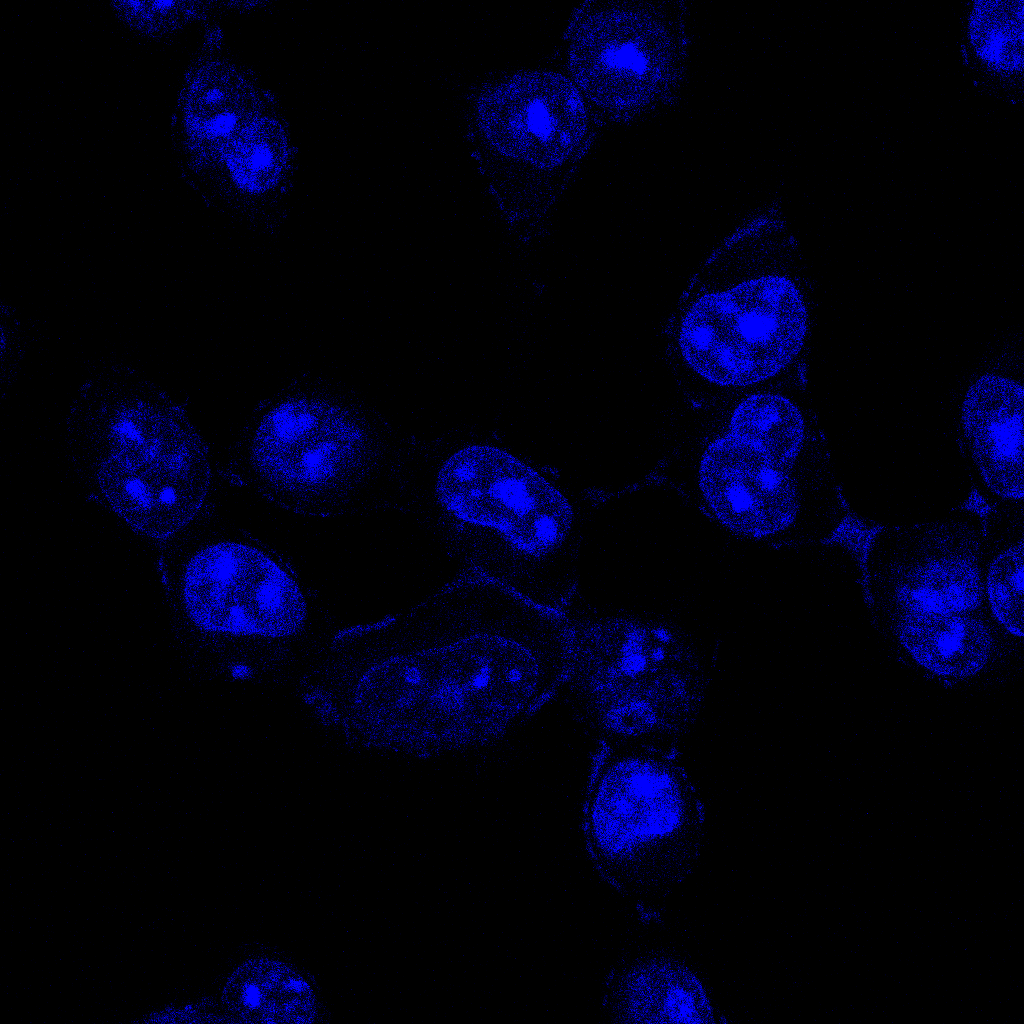

Supplement: Supplementary file 12 — Source data Fig. 7 [file 44318_2024_359_MOESM12_ESM.zip › Figure 7/Fig 7B/hSPAR/hSPAR-Hoechst-1.tif]

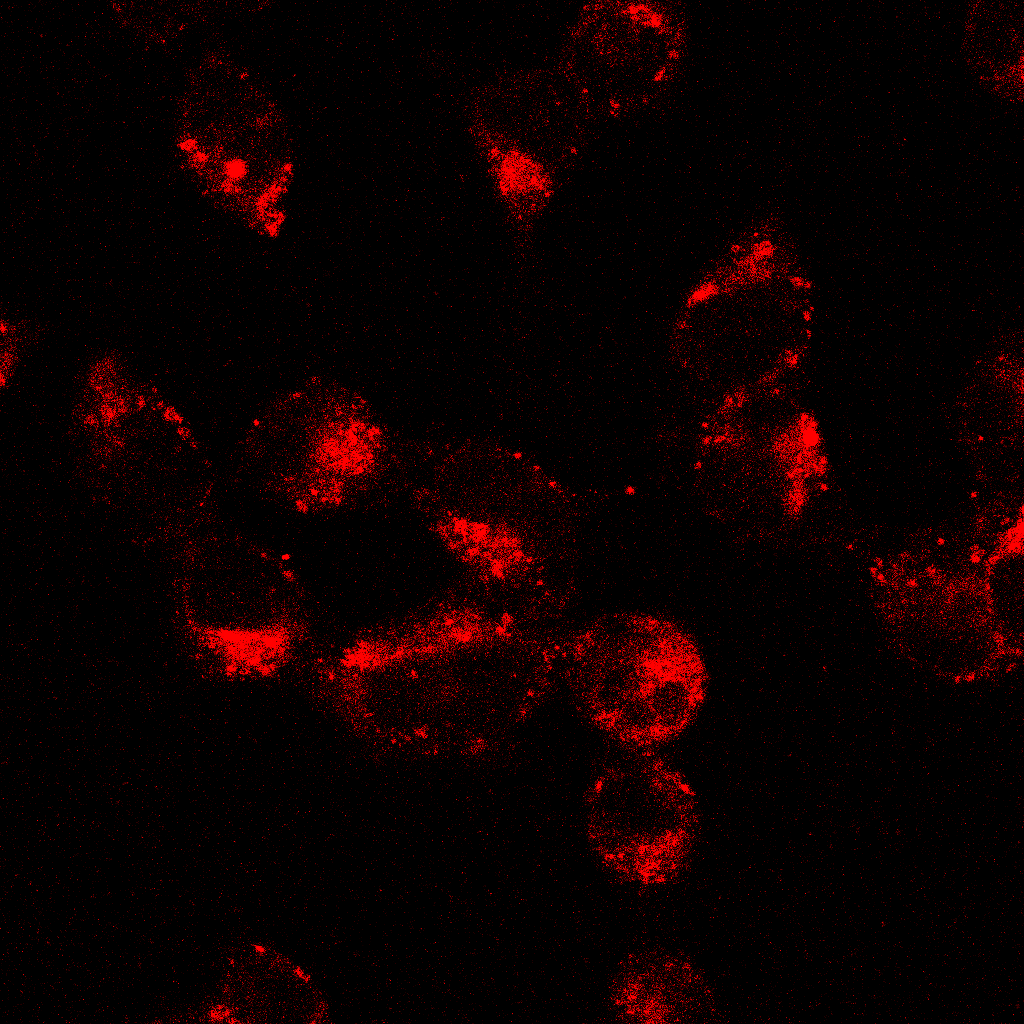

Supplement: Supplementary file 12 — Source data Fig. 7 [file 44318_2024_359_MOESM12_ESM.zip › Figure 7/Fig 7B/hSPAR/hSPAR-LAMP1-1.tif]

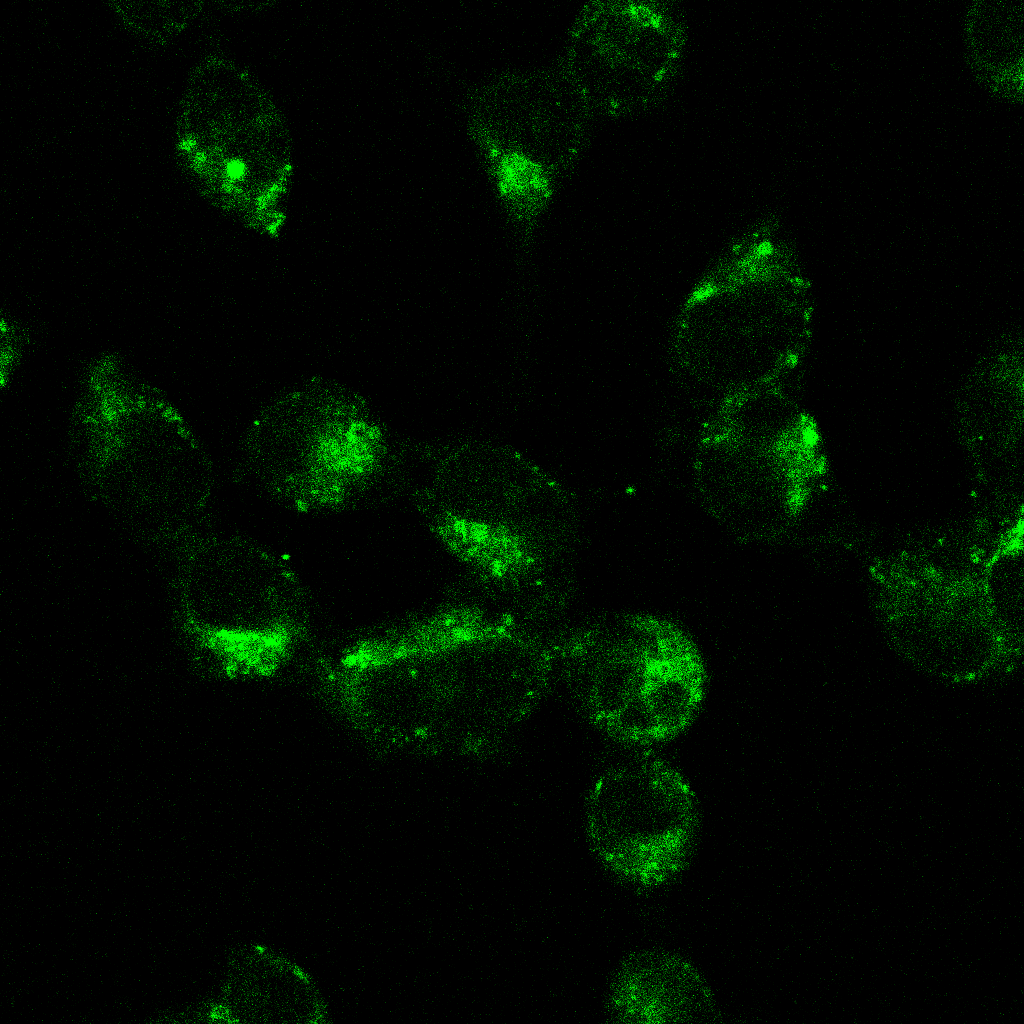

Supplement: Supplementary file 12 — Source data Fig. 7 [file 44318_2024_359_MOESM12_ESM.zip › Figure 7/Fig 7B/hSPAR/hSPAR-p27-1.tif]

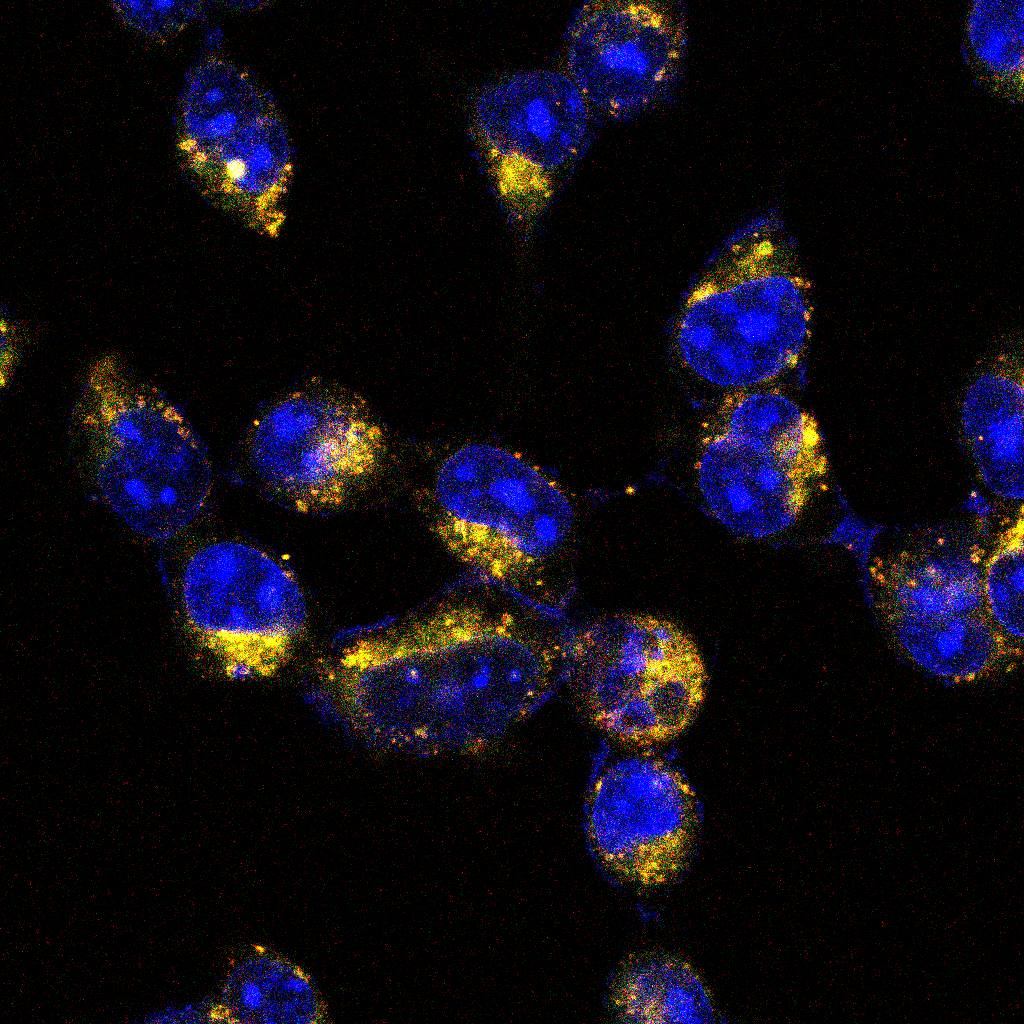

Supplement: Supplementary file 12 — Source data Fig. 7 [file 44318_2024_359_MOESM12_ESM.zip › Figure 7/Fig 7B/hSPAR/hSPAR-p27-merge.tif]

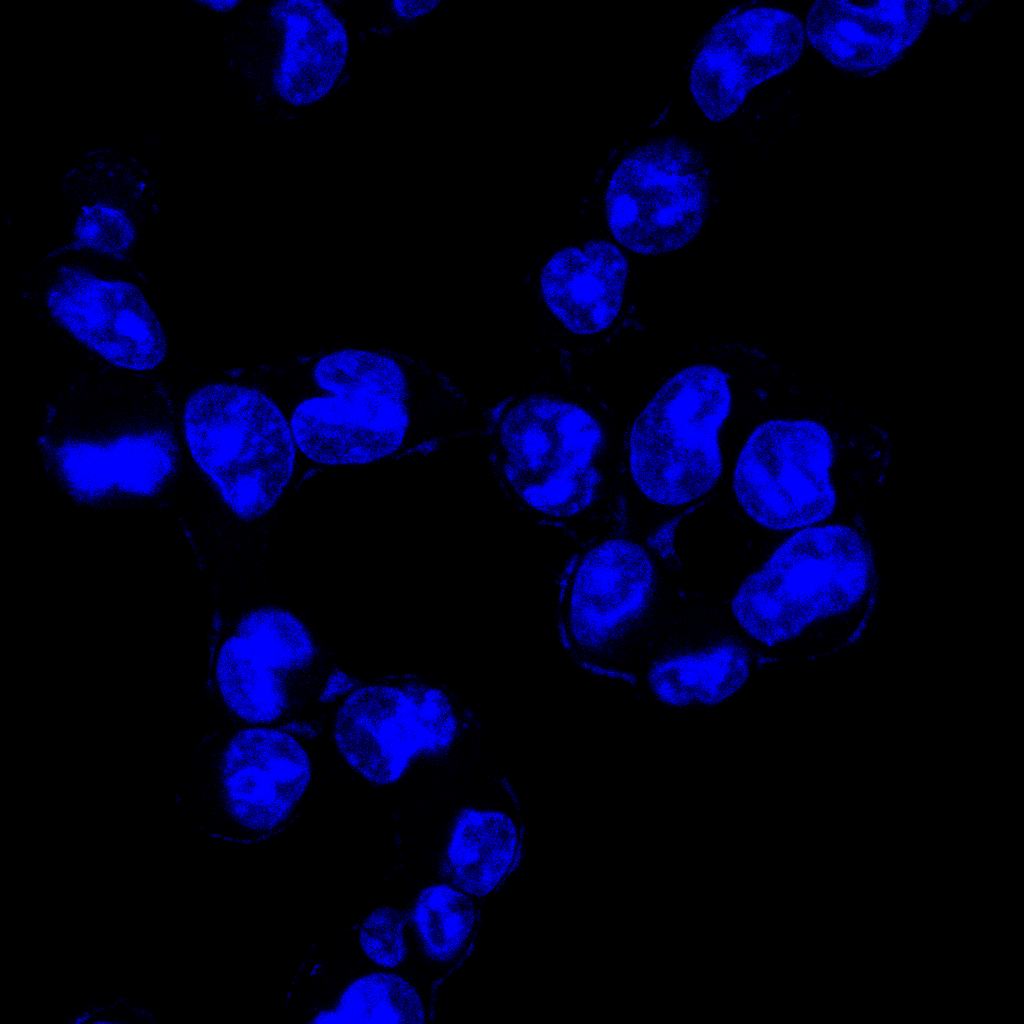

Supplement: Supplementary file 12 — Source data Fig. 7 [file 44318_2024_359_MOESM12_ESM.zip › Figure 7/Fig 7C/ATG/ATG-Hoechst.tif]

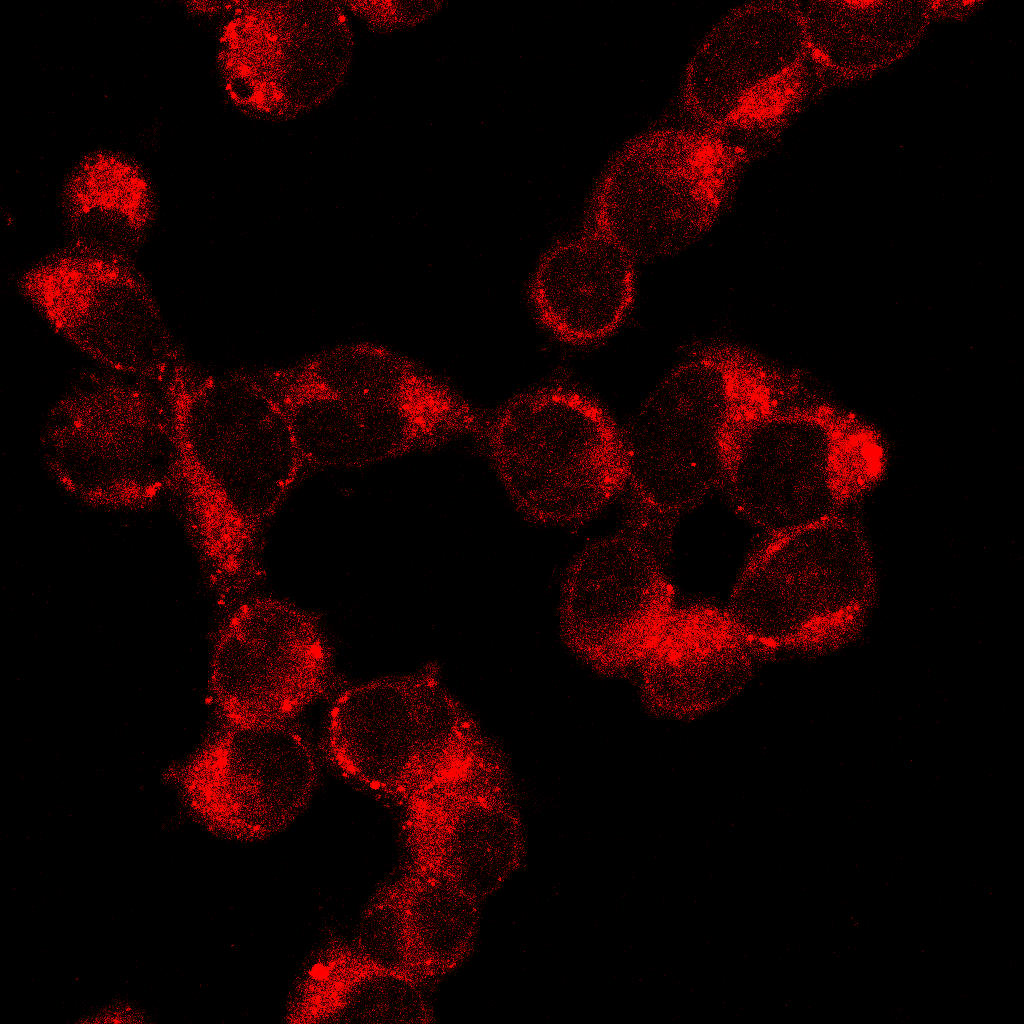

Supplement: Supplementary file 12 — Source data Fig. 7 [file 44318_2024_359_MOESM12_ESM.zip › Figure 7/Fig 7C/ATG/ATG-LAMP1.tif]

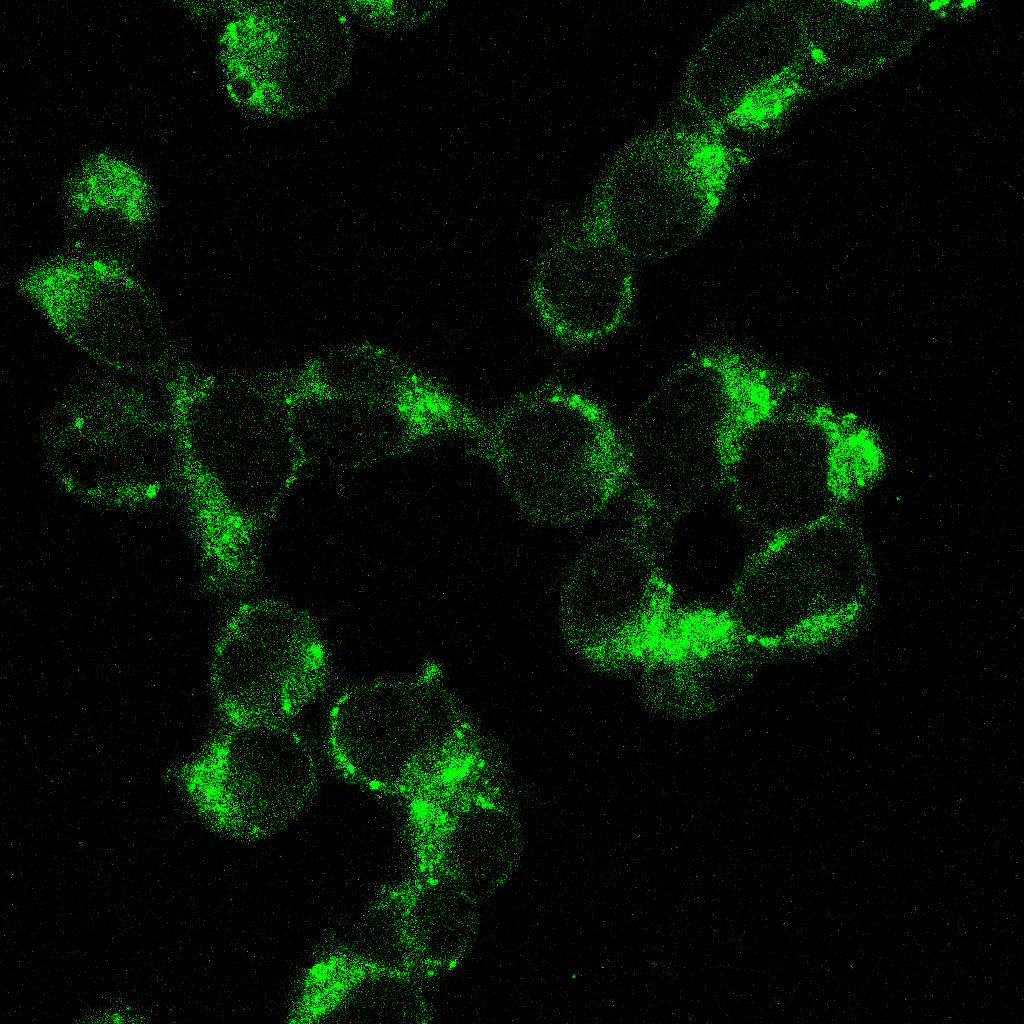

Supplement: Supplementary file 12 — Source data Fig. 7 [file 44318_2024_359_MOESM12_ESM.zip › Figure 7/Fig 7C/ATG/ATG-mTOR.tif]

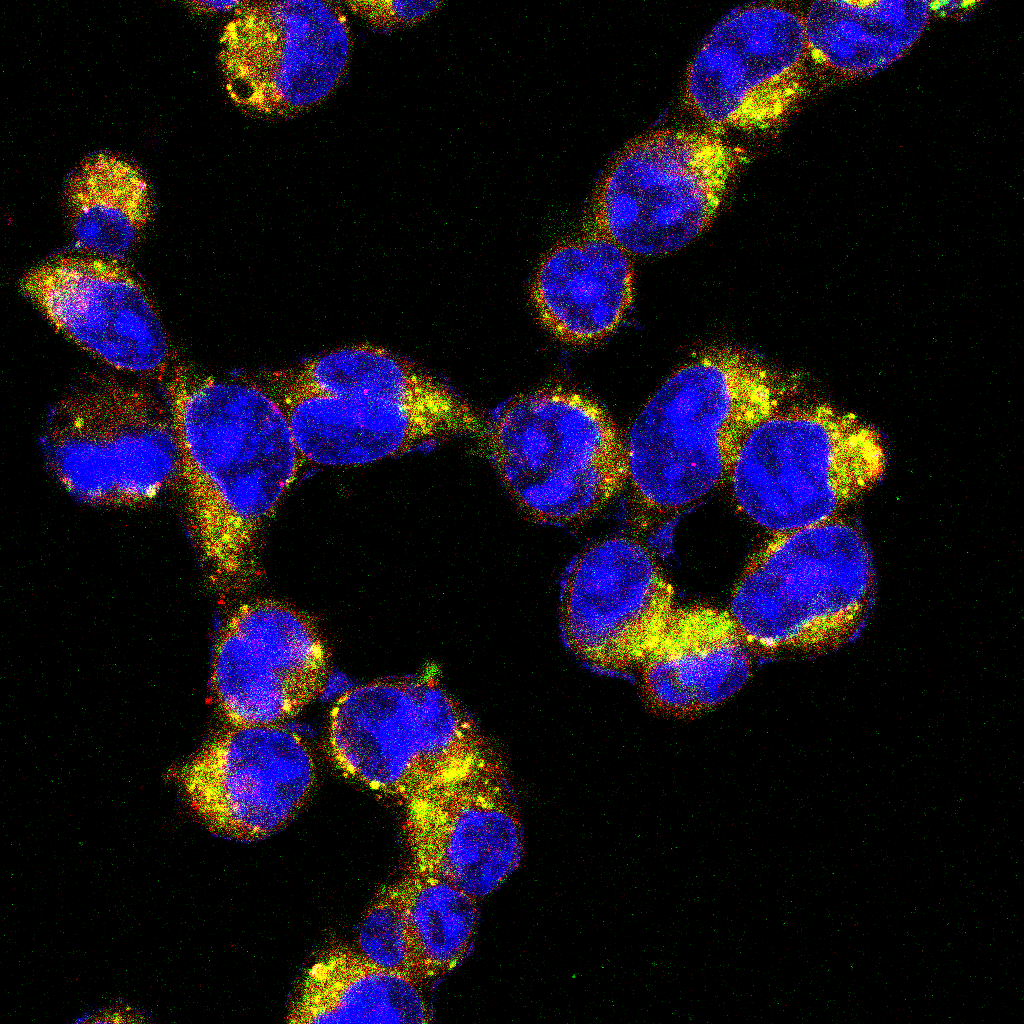

Supplement: Supplementary file 12 — Source data Fig. 7 [file 44318_2024_359_MOESM12_ESM.zip › Figure 7/Fig 7C/ATG/ATG-merge.tif]

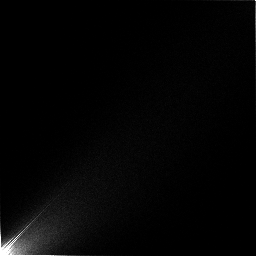

Supplement: Supplementary file 12 — Source data Fig. 7 [file 44318_2024_359_MOESM12_ESM.zip › Figure 7/Fig 7C/ATG/Pearson correlation.tif]

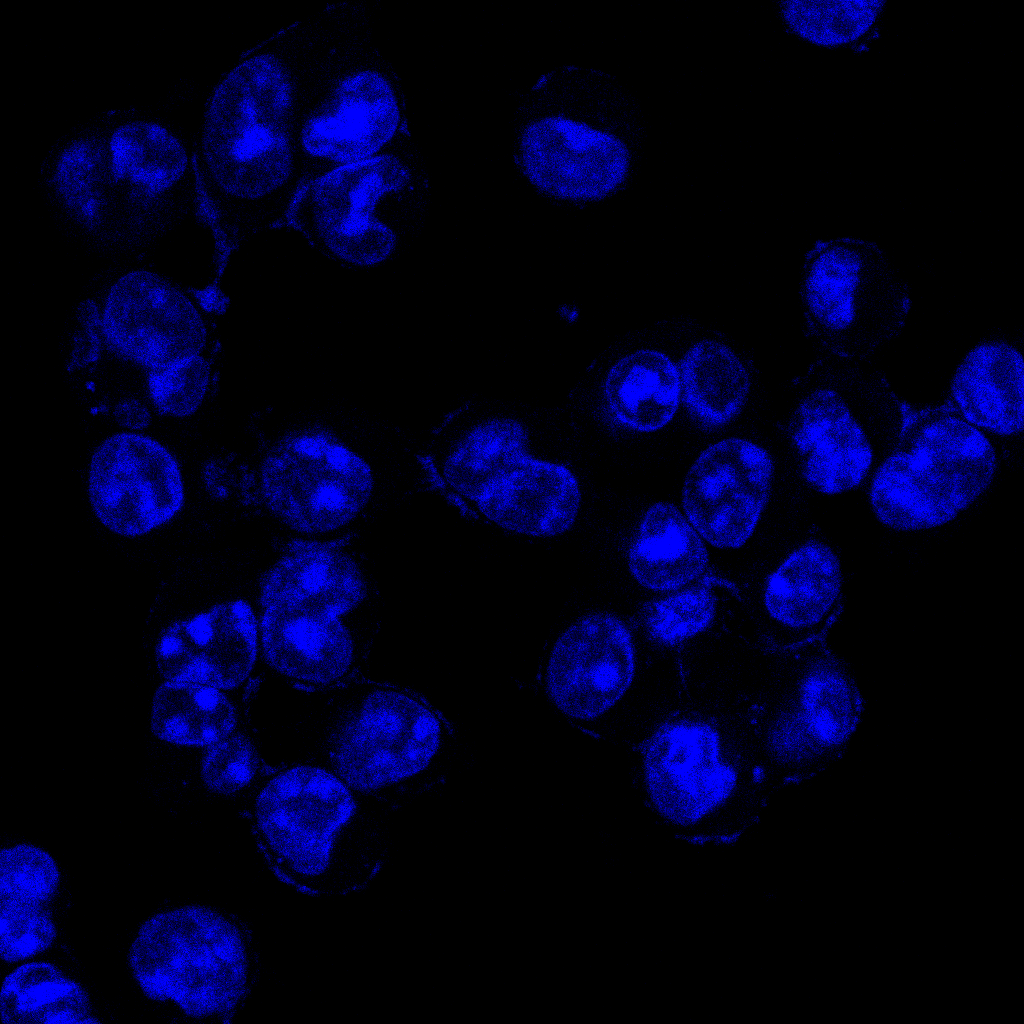

Supplement: Supplementary file 12 — Source data Fig. 7 [file 44318_2024_359_MOESM12_ESM.zip › Figure 7/Fig 7C/C/C-Hoechst.tif]

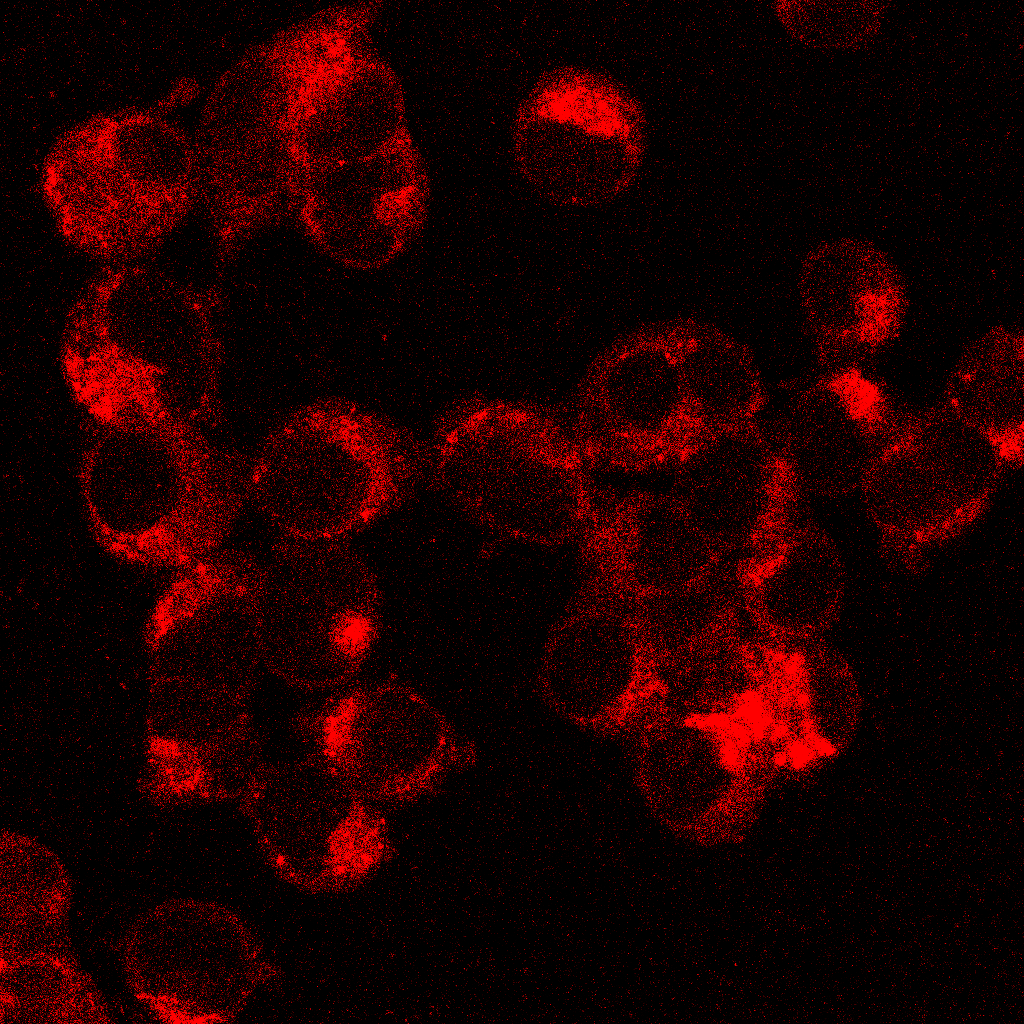

Supplement: Supplementary file 12 — Source data Fig. 7 [file 44318_2024_359_MOESM12_ESM.zip › Figure 7/Fig 7C/C/C-LAMP1.tif]

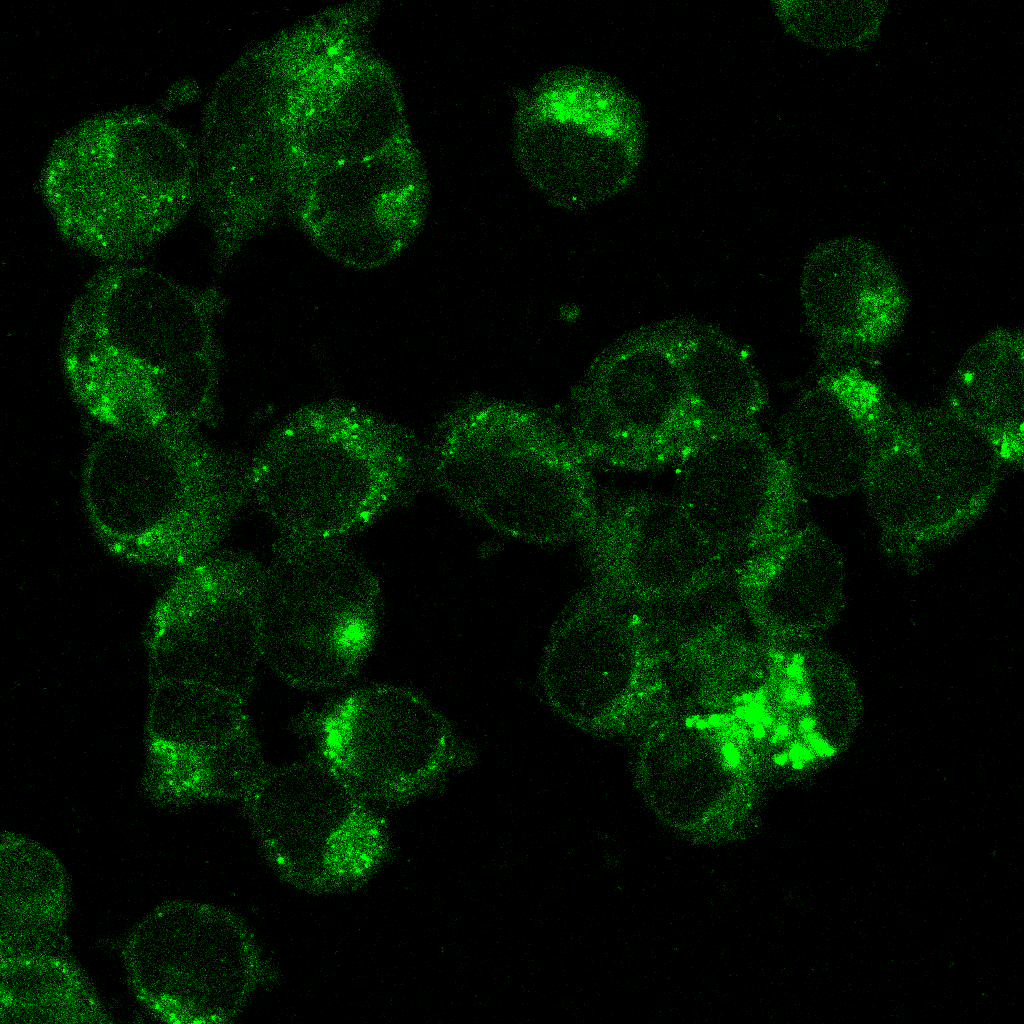

Supplement: Supplementary file 12 — Source data Fig. 7 [file 44318_2024_359_MOESM12_ESM.zip › Figure 7/Fig 7C/C/C-mTOR.tif]

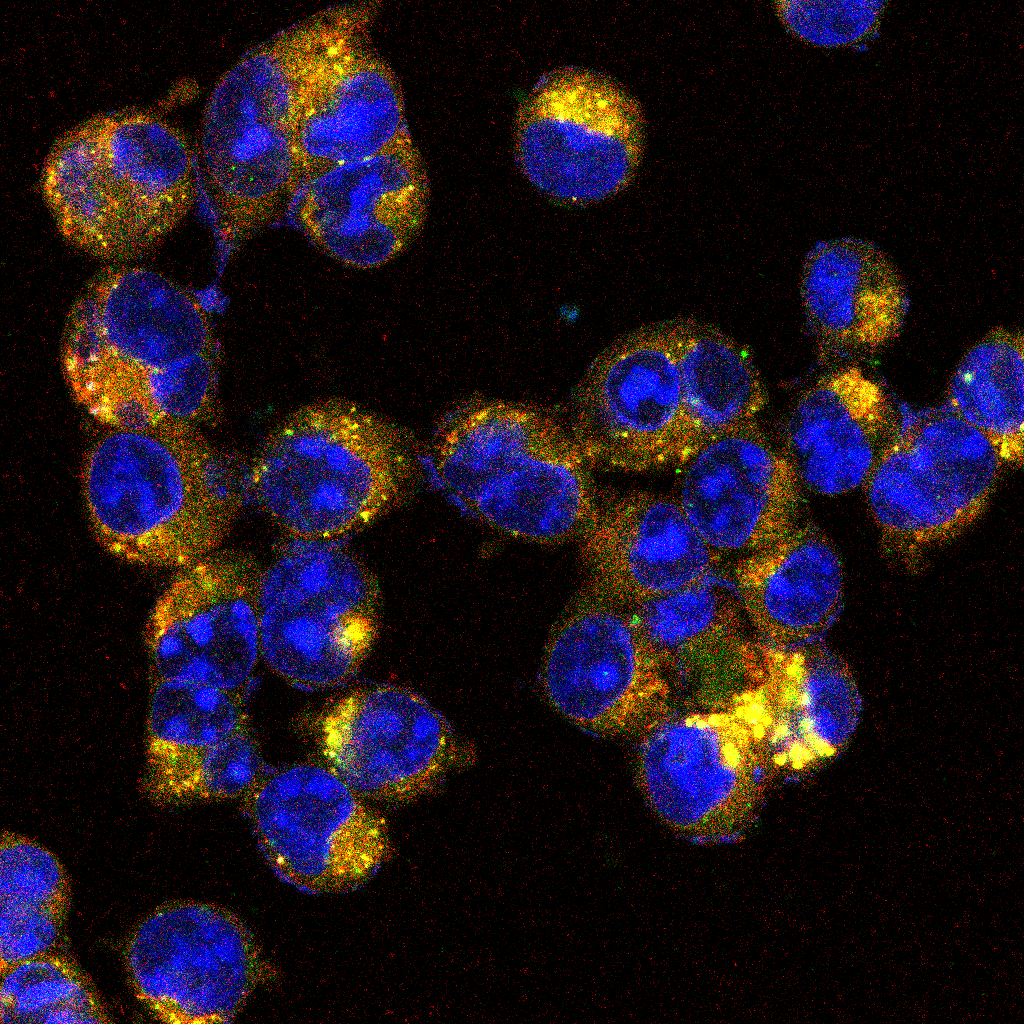

Supplement: Supplementary file 12 — Source data Fig. 7 [file 44318_2024_359_MOESM12_ESM.zip › Figure 7/Fig 7C/C/C-merge.tif]

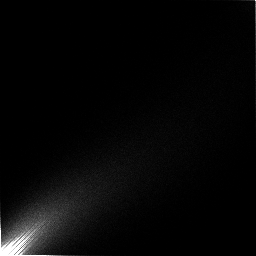

Supplement: Supplementary file 12 — Source data Fig. 7 [file 44318_2024_359_MOESM12_ESM.zip › Figure 7/Fig 7C/C/Pearson correlation.tif]

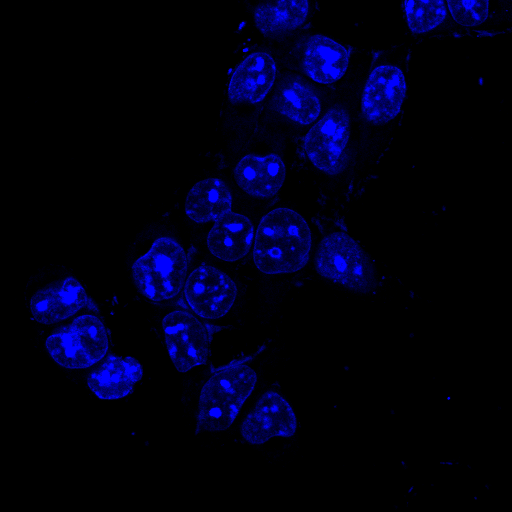

Supplement: Supplementary file 12 — Source data Fig. 7 [file 44318_2024_359_MOESM12_ESM.zip › Figure 7/Fig 7C/N/N-Hoechst.tif]

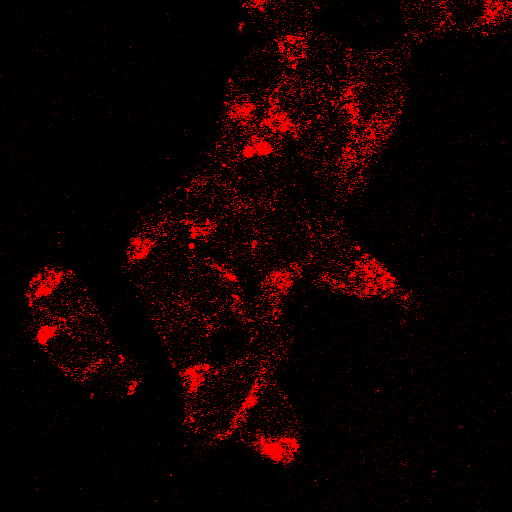

Supplement: Supplementary file 12 — Source data Fig. 7 [file 44318_2024_359_MOESM12_ESM.zip › Figure 7/Fig 7C/N/N-LAMP1.tif]

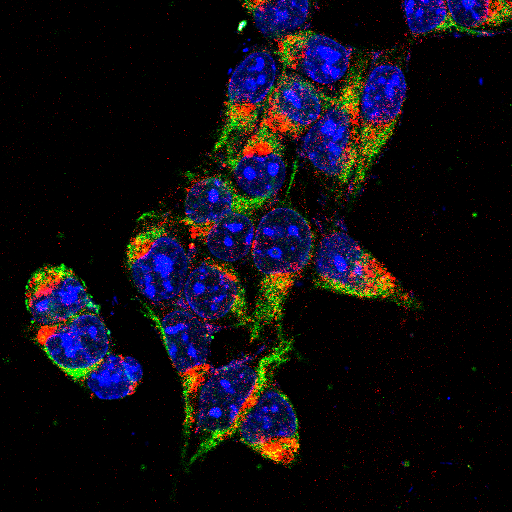

Supplement: Supplementary file 12 — Source data Fig. 7 [file 44318_2024_359_MOESM12_ESM.zip › Figure 7/Fig 7C/N/N-mTOR-merge.tif]

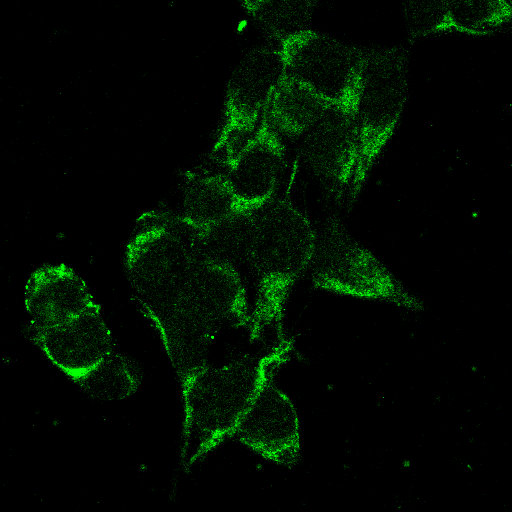

Supplement: Supplementary file 12 — Source data Fig. 7 [file 44318_2024_359_MOESM12_ESM.zip › Figure 7/Fig 7C/N/N-mTOR.tif]

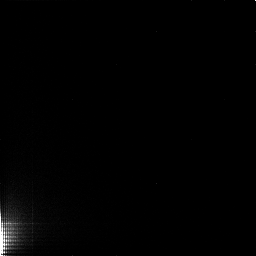

Supplement: Supplementary file 12 — Source data Fig. 7 [file 44318_2024_359_MOESM12_ESM.zip › Figure 7/Fig 7C/N/Pearson correlation.tif]

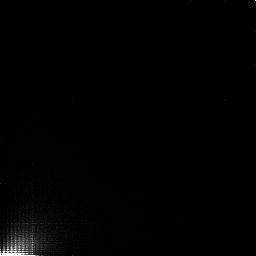

Supplement: Supplementary file 12 — Source data Fig. 7 [file 44318_2024_359_MOESM12_ESM.zip › Figure 7/Fig 7C/TM/Pearson correlation.tif]

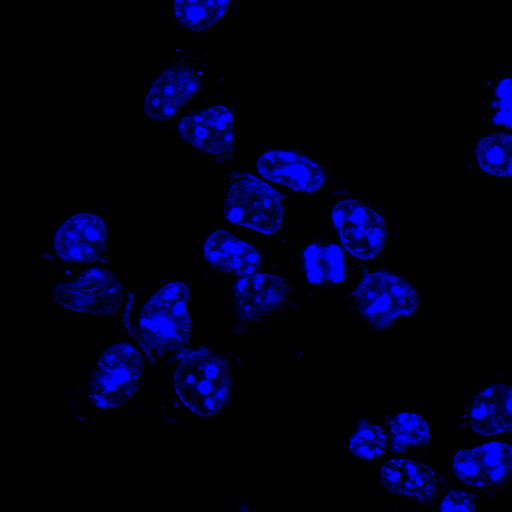

Supplement: Supplementary file 12 — Source data Fig. 7 [file 44318_2024_359_MOESM12_ESM.zip › Figure 7/Fig 7C/TM/TM-Hoechst.tif]

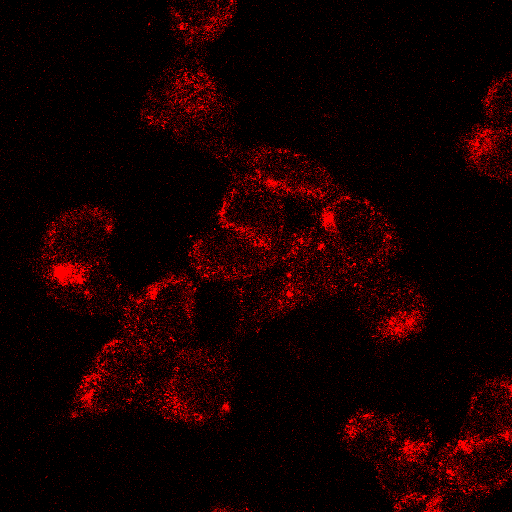

Supplement: Supplementary file 12 — Source data Fig. 7 [file 44318_2024_359_MOESM12_ESM.zip › Figure 7/Fig 7C/TM/TM-LAMP1.tif]

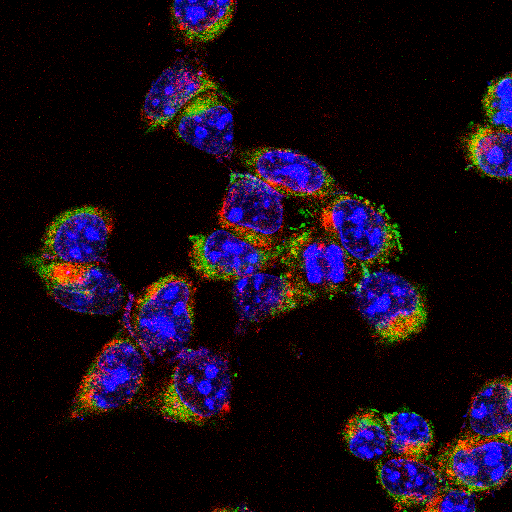

Supplement: Supplementary file 12 — Source data Fig. 7 [file 44318_2024_359_MOESM12_ESM.zip › Figure 7/Fig 7C/TM/TM-mTOR-merge.tif]

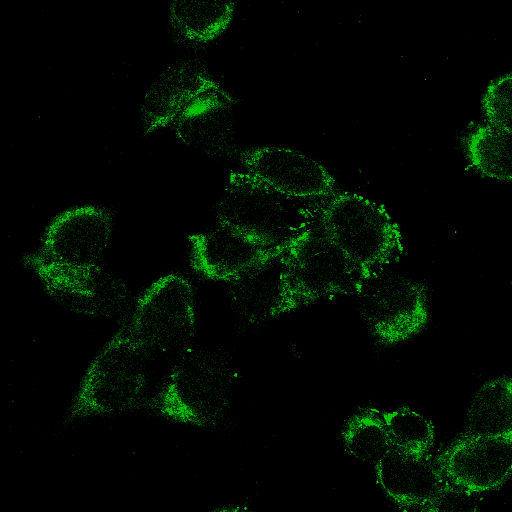

Supplement: Supplementary file 12 — Source data Fig. 7 [file 44318_2024_359_MOESM12_ESM.zip › Figure 7/Fig 7C/TM/TM-mTOR.tif]

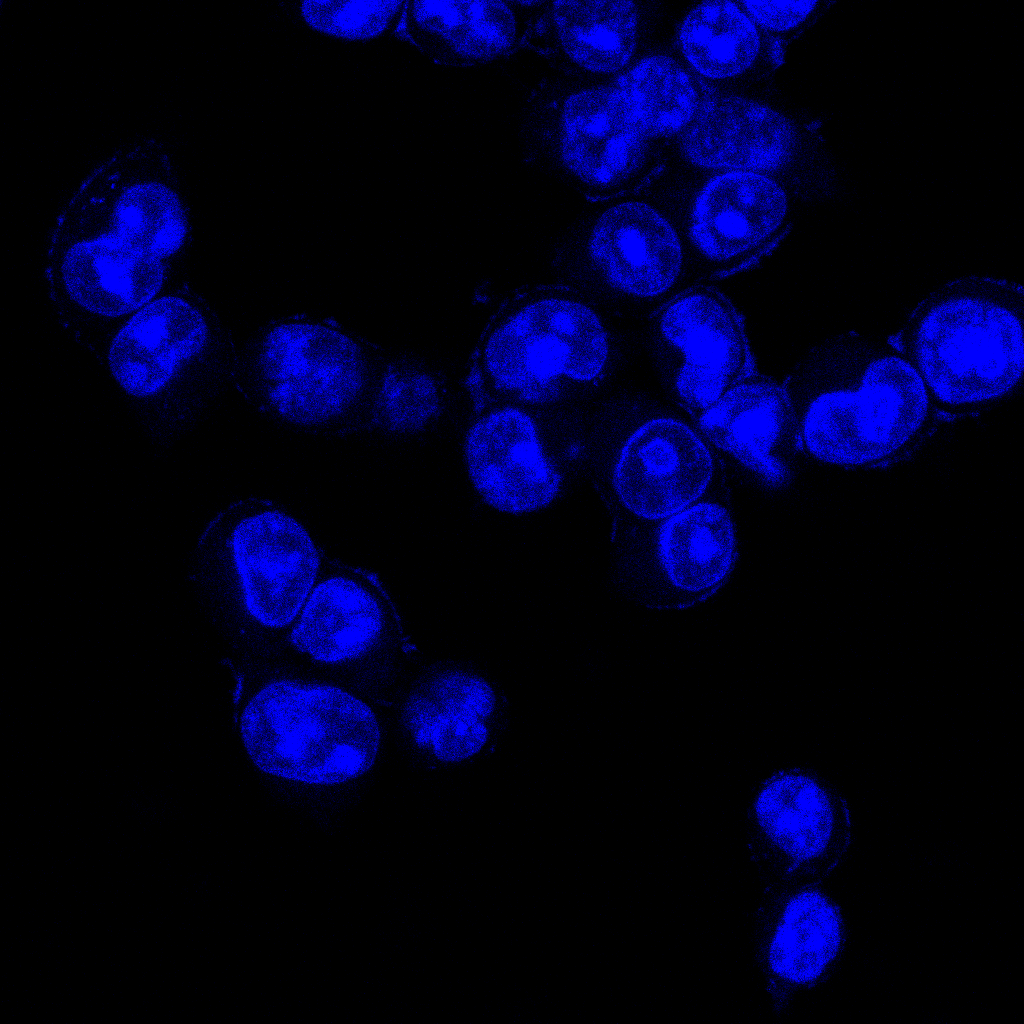

Supplement: Supplementary file 12 — Source data Fig. 7 [file 44318_2024_359_MOESM12_ESM.zip › Figure 7/Fig 7C/Vector Ctrl/Ctrl-Hoechst.tif]

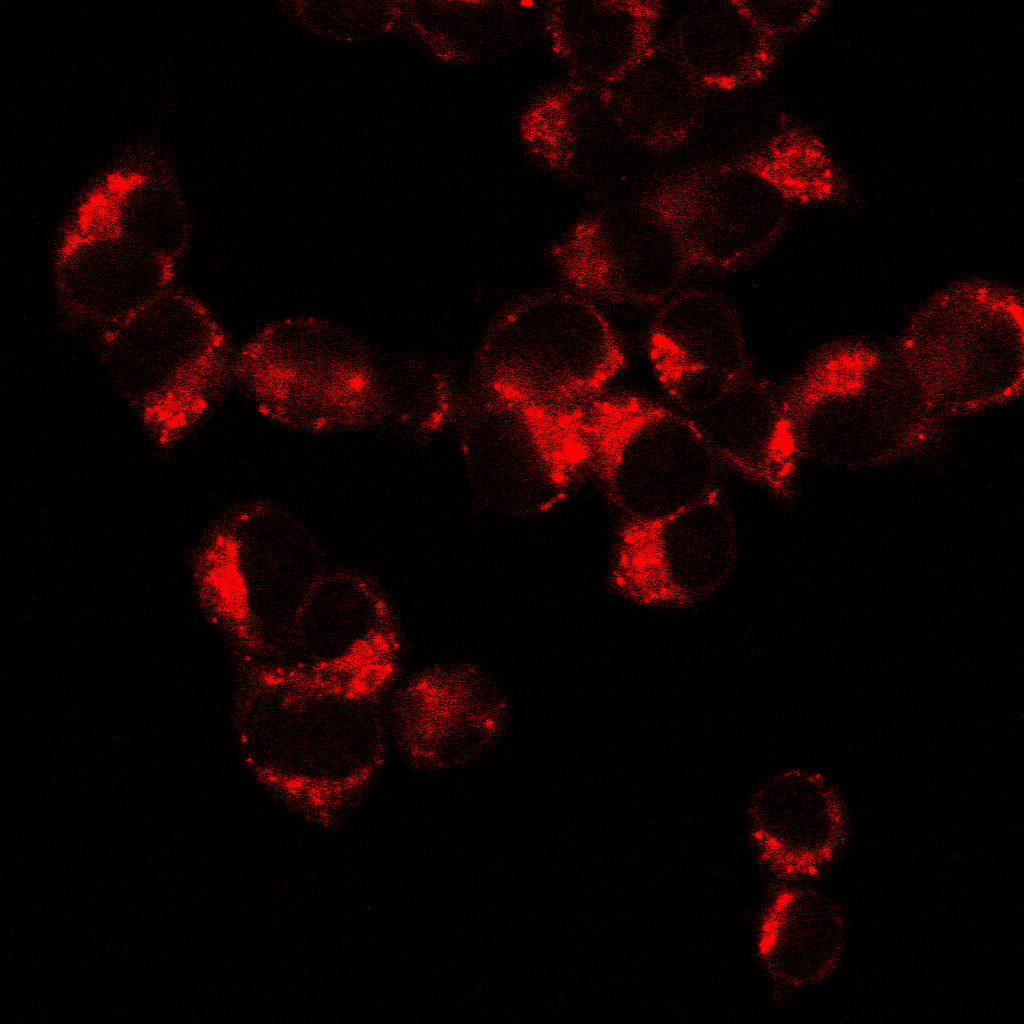

Supplement: Supplementary file 12 — Source data Fig. 7 [file 44318_2024_359_MOESM12_ESM.zip › Figure 7/Fig 7C/Vector Ctrl/Ctrl-LAMP1.tif]

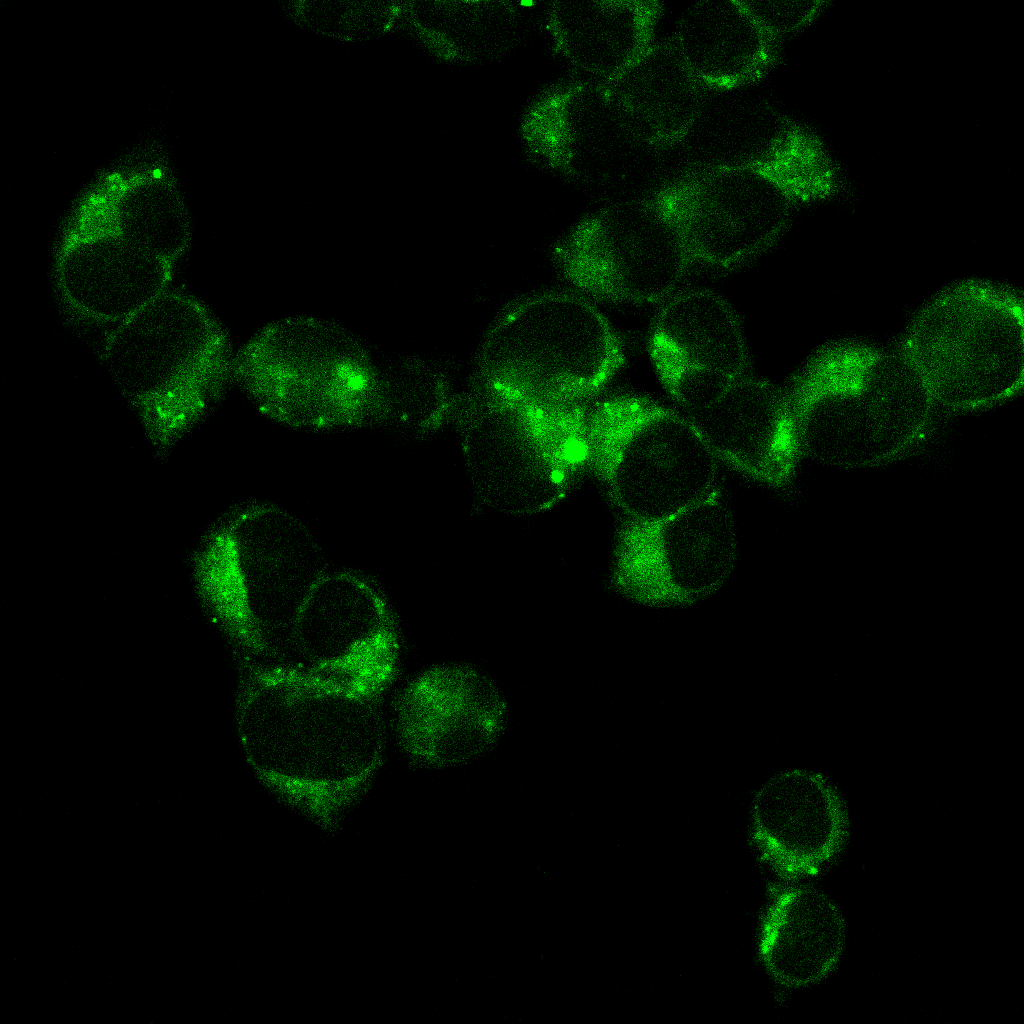

Supplement: Supplementary file 12 — Source data Fig. 7 [file 44318_2024_359_MOESM12_ESM.zip › Figure 7/Fig 7C/Vector Ctrl/Ctrl-mTOR.tif]

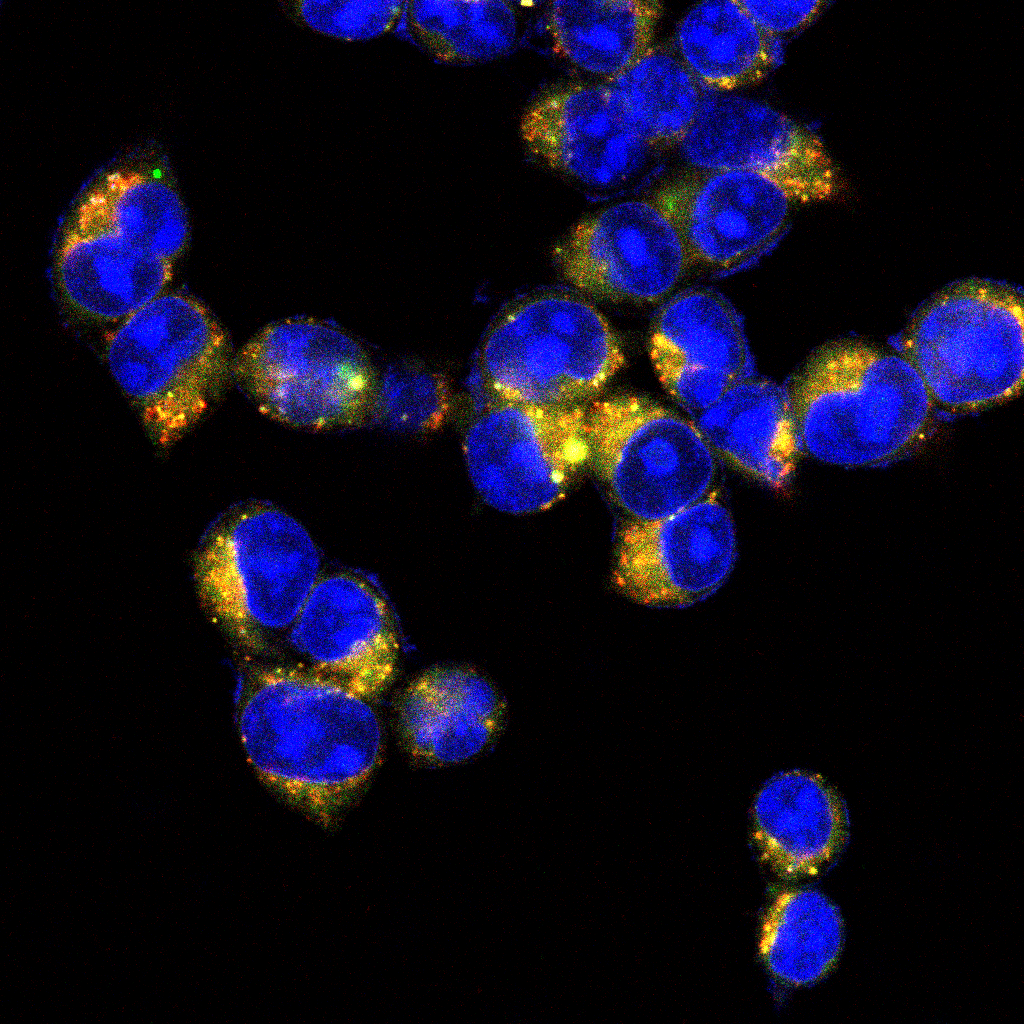

Supplement: Supplementary file 12 — Source data Fig. 7 [file 44318_2024_359_MOESM12_ESM.zip › Figure 7/Fig 7C/Vector Ctrl/Ctrl-merge.tif]

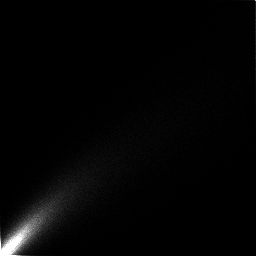

Supplement: Supplementary file 12 — Source data Fig. 7 [file 44318_2024_359_MOESM12_ESM.zip › Figure 7/Fig 7C/Vector Ctrl/Pearson correlation.tif]

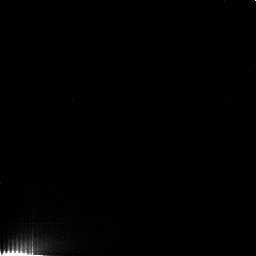

Supplement: Supplementary file 12 — Source data Fig. 7 [file 44318_2024_359_MOESM12_ESM.zip › Figure 7/Fig 7C/hSPAR/Pearson correlation.tif]

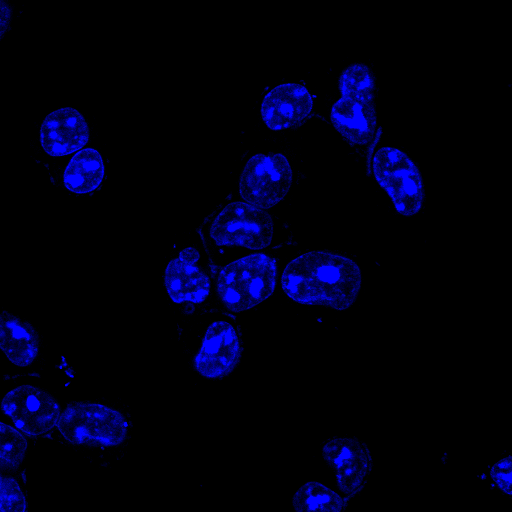

Supplement: Supplementary file 12 — Source data Fig. 7 [file 44318_2024_359_MOESM12_ESM.zip › Figure 7/Fig 7C/hSPAR/hSPAR-Hoechst.tif]

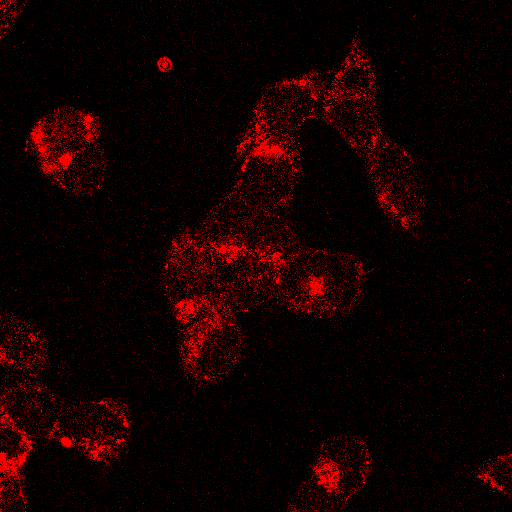

Supplement: Supplementary file 12 — Source data Fig. 7 [file 44318_2024_359_MOESM12_ESM.zip › Figure 7/Fig 7C/hSPAR/hSPAR-LAMP1.tif]

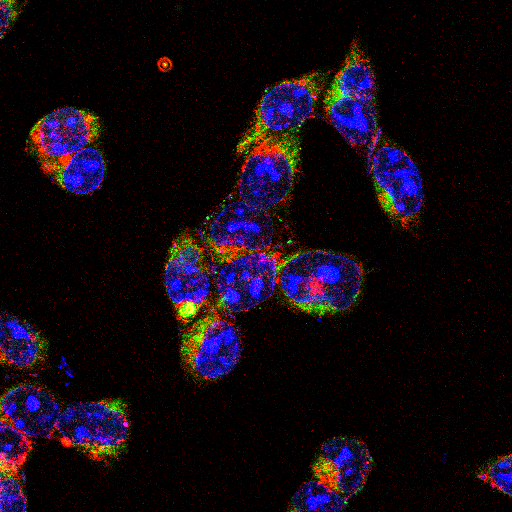

Supplement: Supplementary file 12 — Source data Fig. 7 [file 44318_2024_359_MOESM12_ESM.zip › Figure 7/Fig 7C/hSPAR/hSPAR-mTOR-merge.tif]

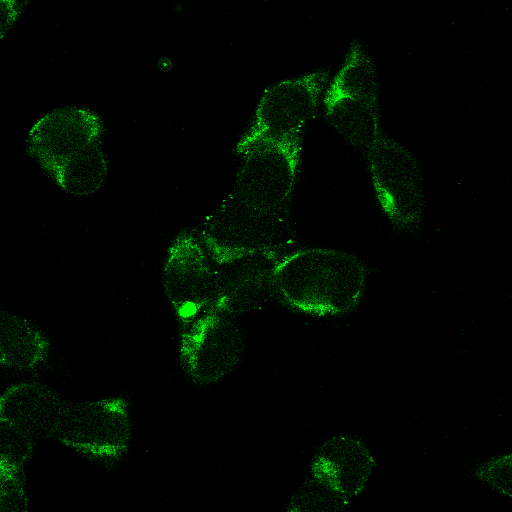

Supplement: Supplementary file 12 — Source data Fig. 7 [file 44318_2024_359_MOESM12_ESM.zip › Figure 7/Fig 7C/hSPAR/hSPAR-mTOR.tif]

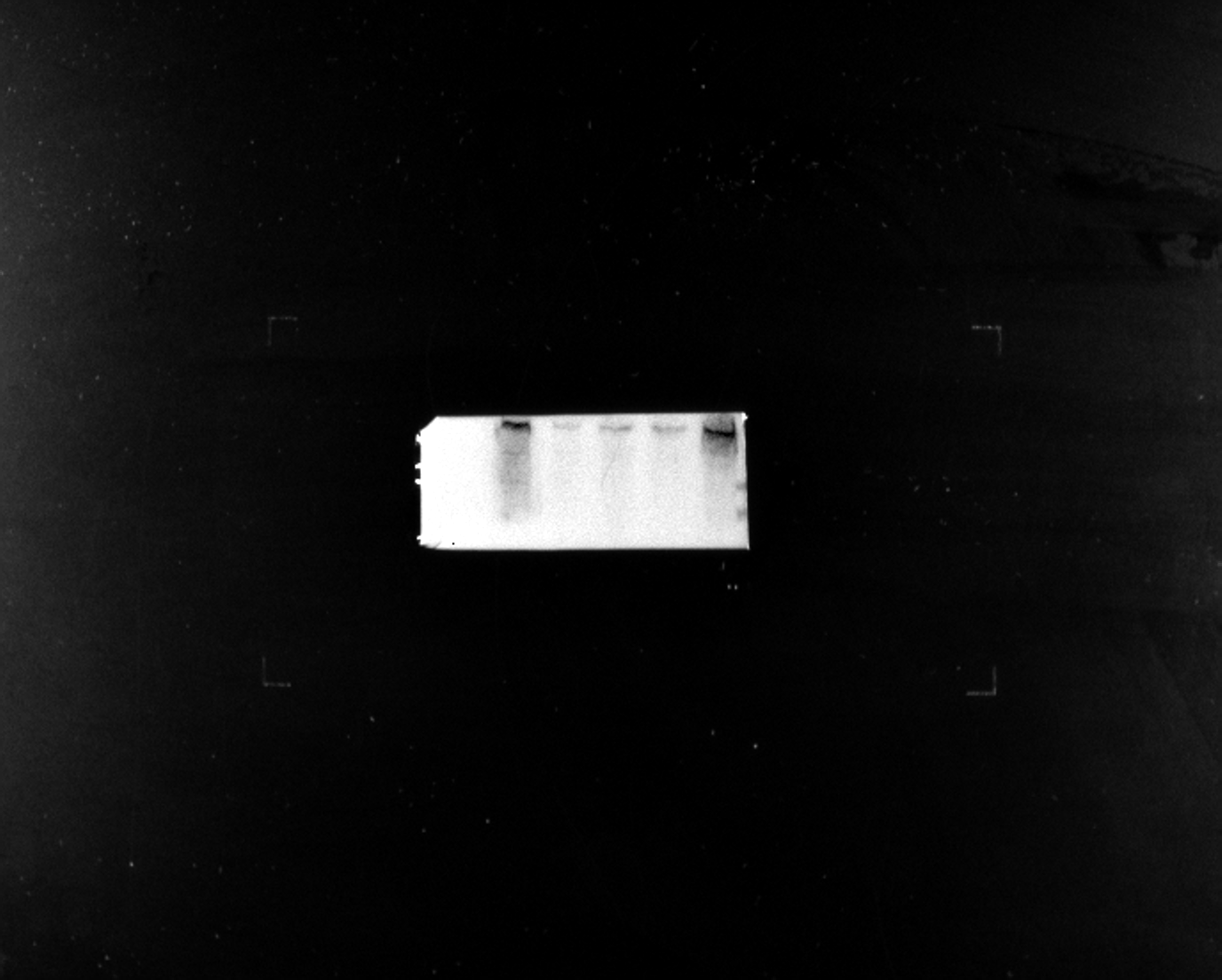

Supplement: Supplementary file 12 — Source data Fig. 7 [file 44318_2024_359_MOESM12_ESM.zip › Figure 7/Fig 7D/1-p-mTOR-merge.Tif]

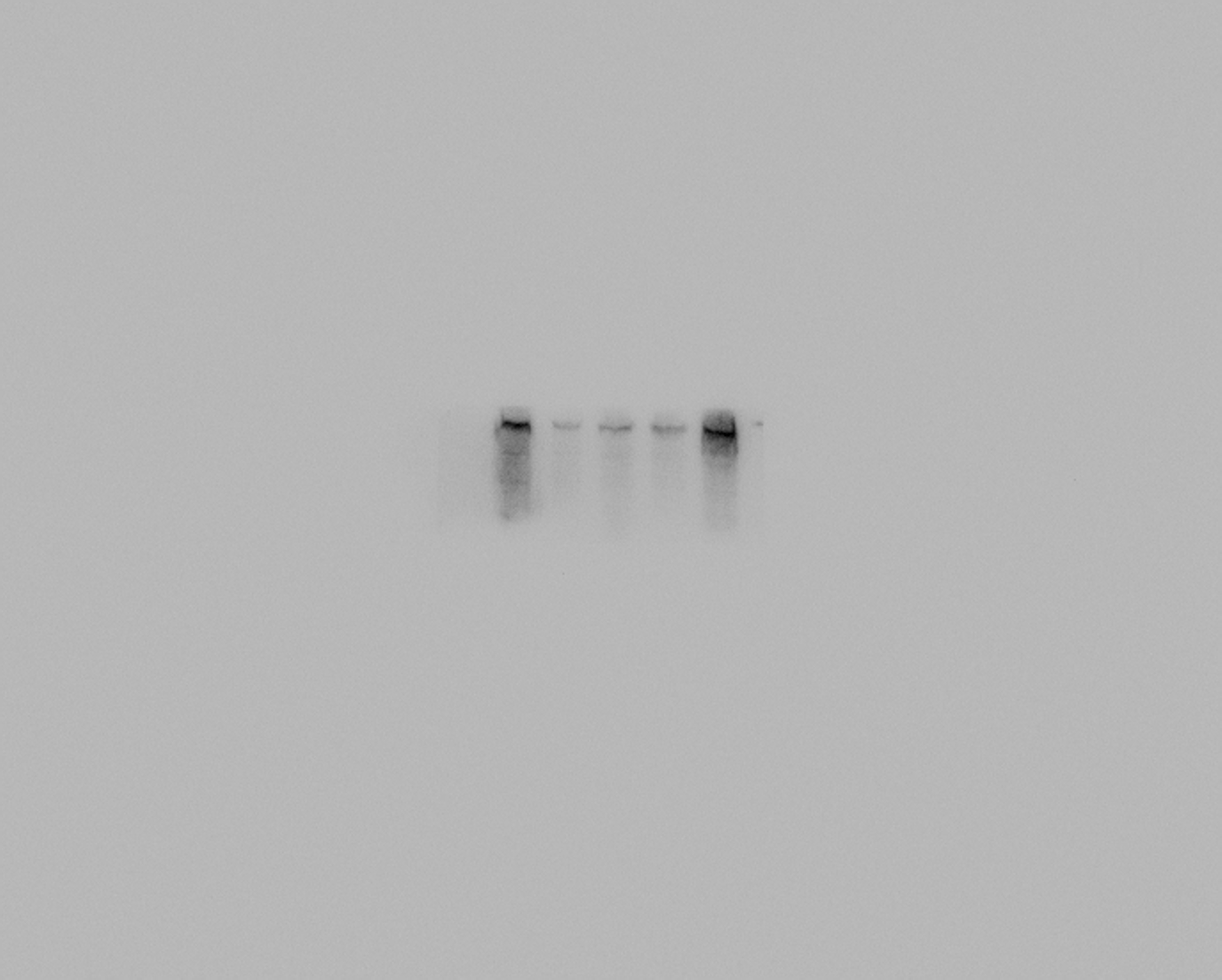

Supplement: Supplementary file 12 — Source data Fig. 7 [file 44318_2024_359_MOESM12_ESM.zip › Figure 7/Fig 7D/1-p-mTOR.Tif]

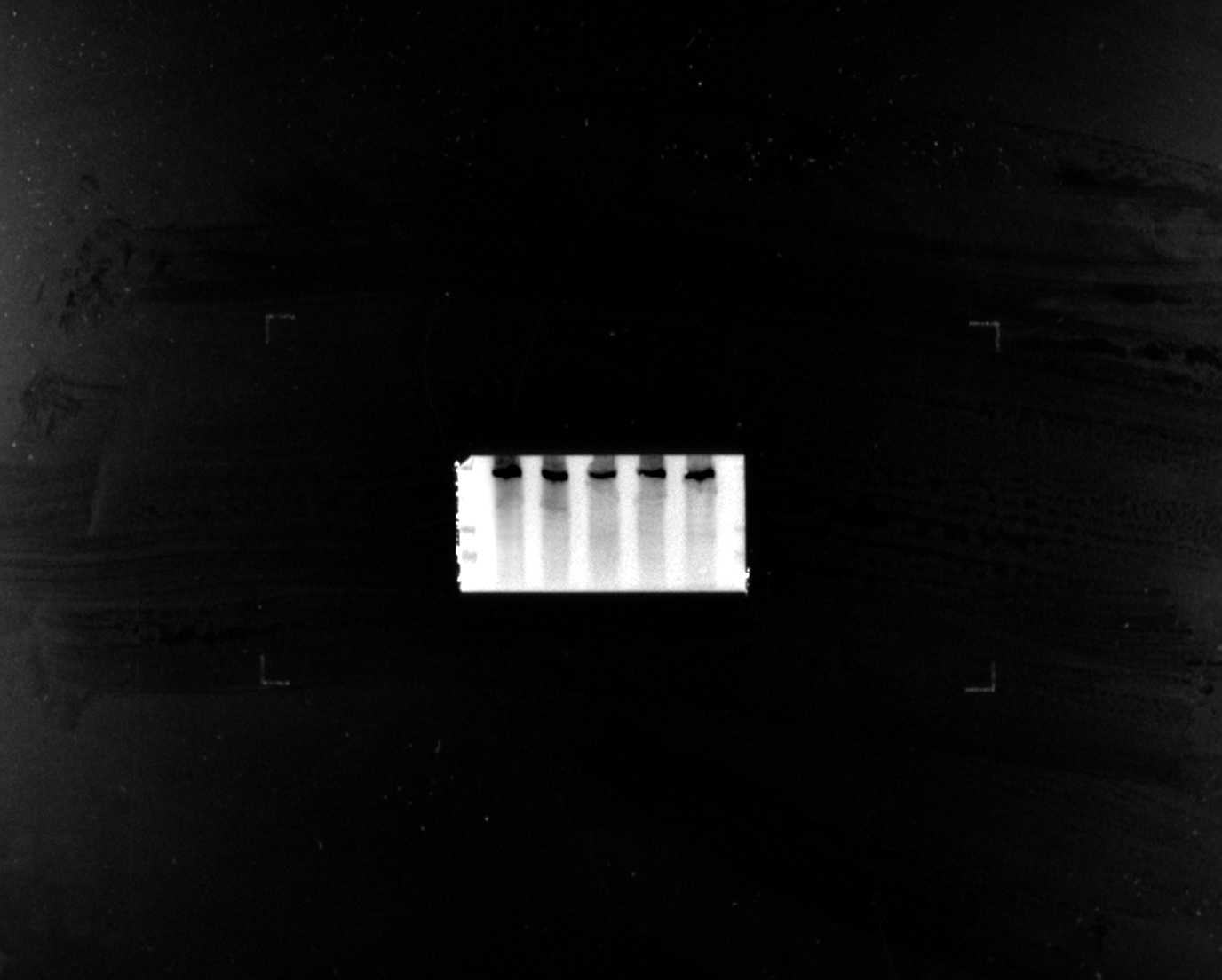

Supplement: Supplementary file 12 — Source data Fig. 7 [file 44318_2024_359_MOESM12_ESM.zip › Figure 7/Fig 7D/2-mTOR-merge.Tif]

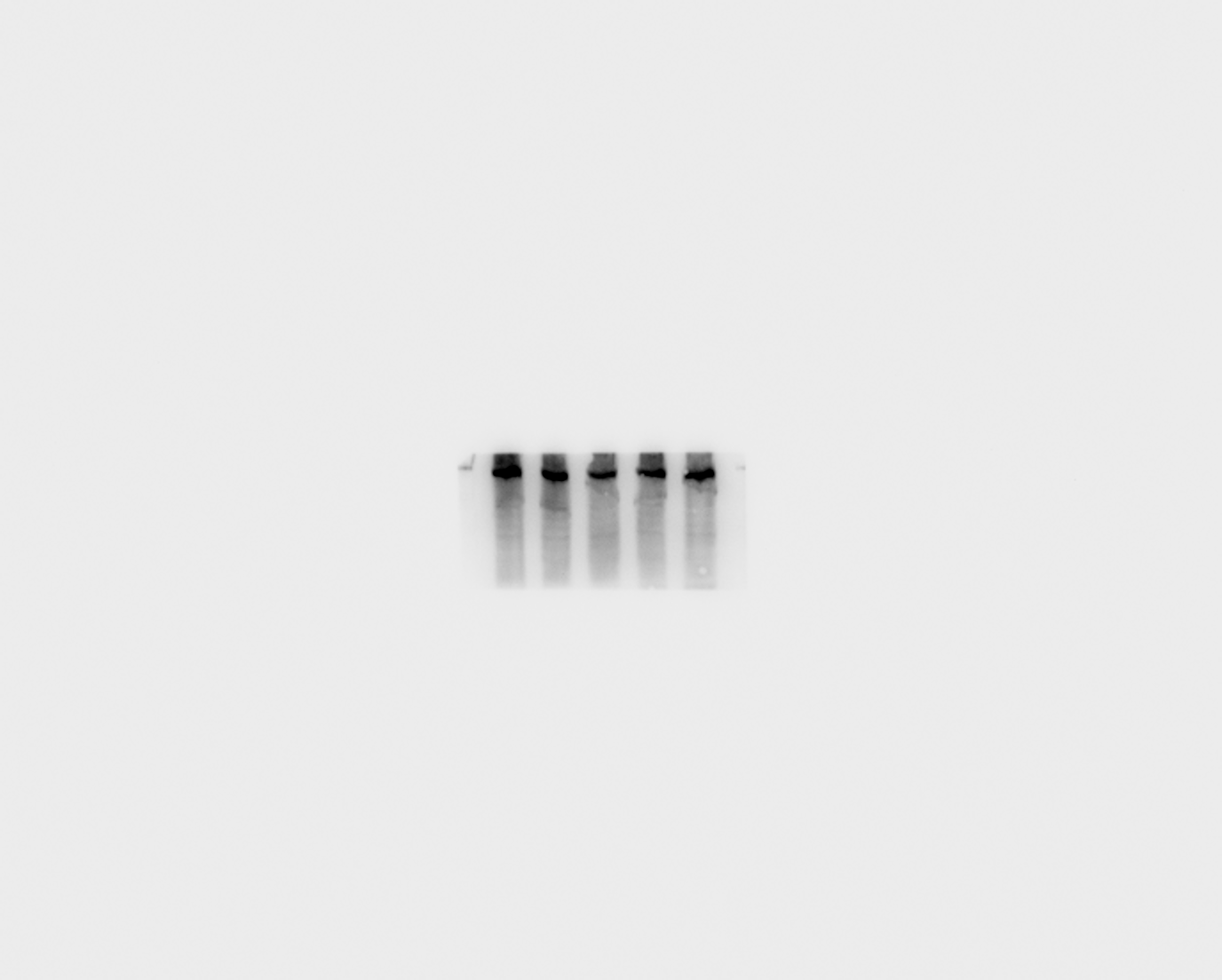

Supplement: Supplementary file 12 — Source data Fig. 7 [file 44318_2024_359_MOESM12_ESM.zip › Figure 7/Fig 7D/2-mTOR.Tif]

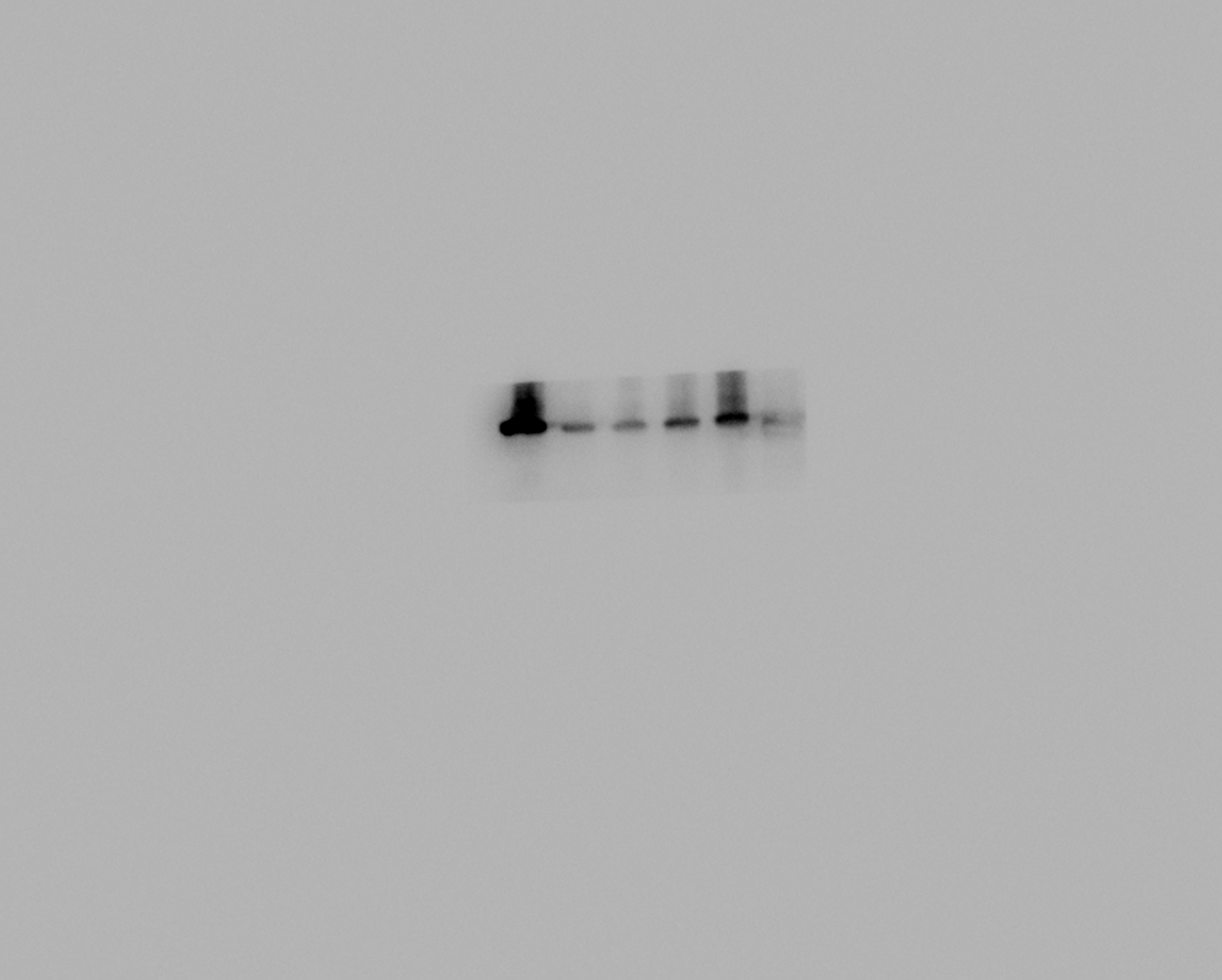

Supplement: Supplementary file 12 — Source data Fig. 7 [file 44318_2024_359_MOESM12_ESM.zip › Figure 7/Fig 7D/3-p-S6K.Tif]

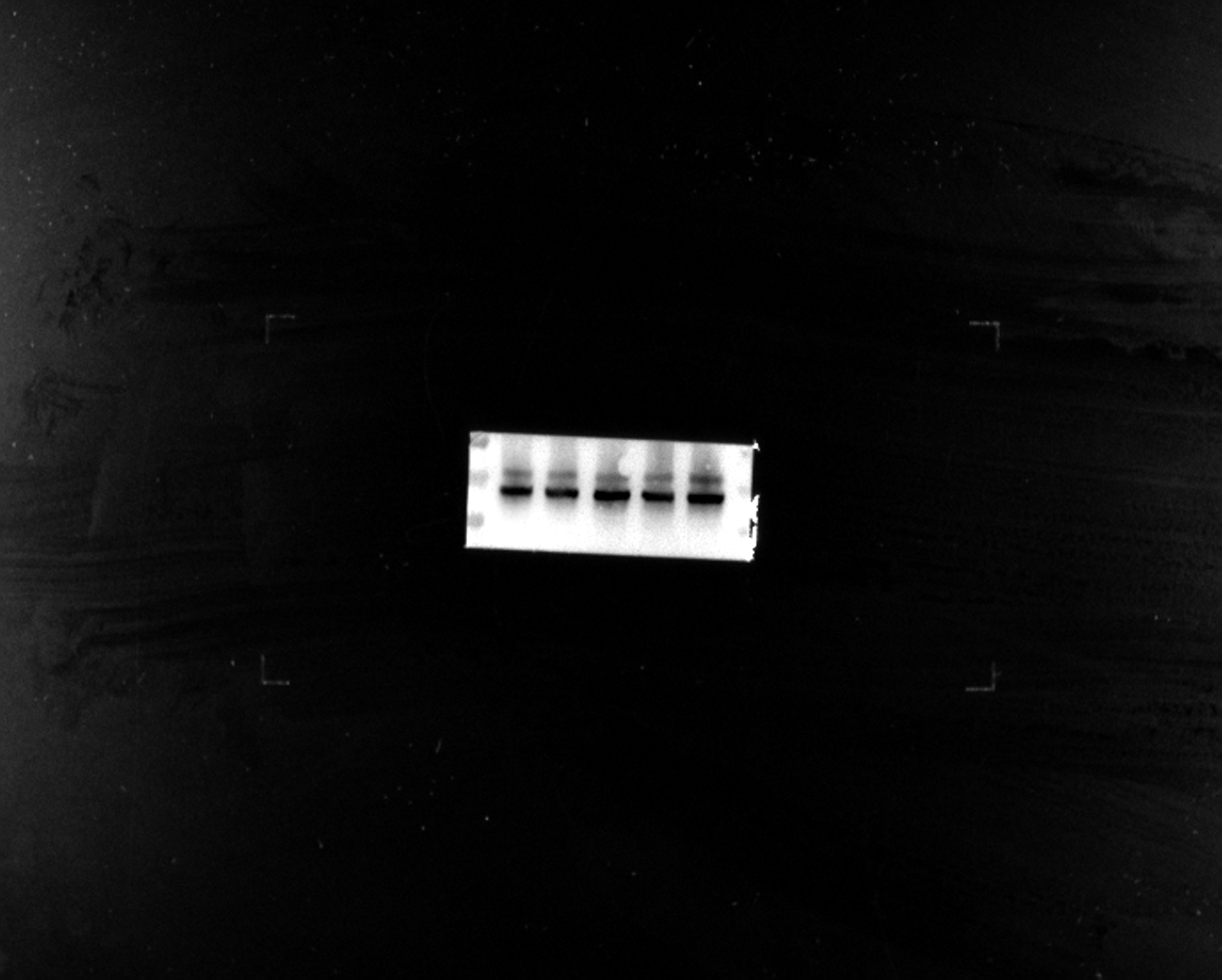

Supplement: Supplementary file 12 — Source data Fig. 7 [file 44318_2024_359_MOESM12_ESM.zip › Figure 7/Fig 7D/4-S6K-merge.Tif]

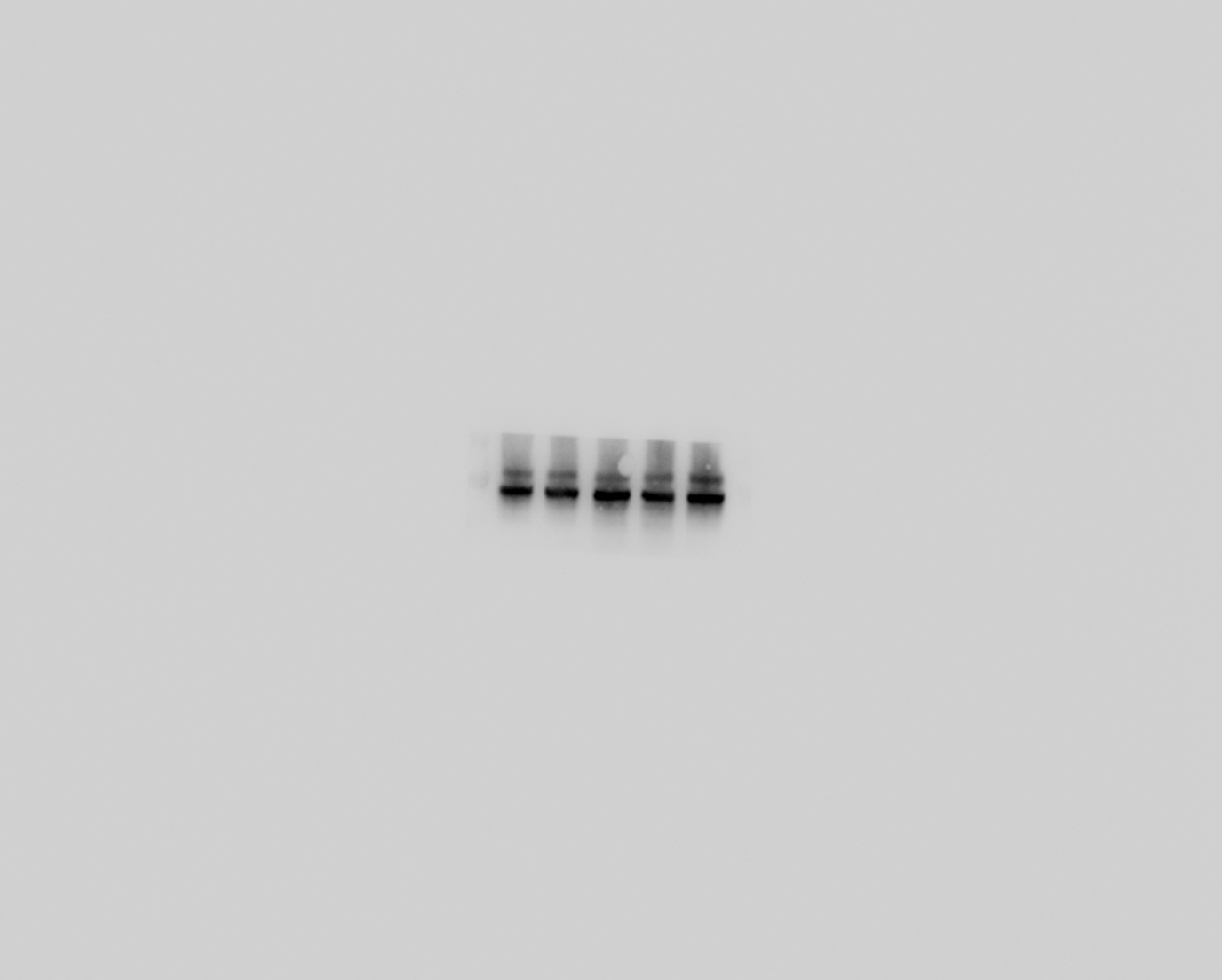

Supplement: Supplementary file 12 — Source data Fig. 7 [file 44318_2024_359_MOESM12_ESM.zip › Figure 7/Fig 7D/4-S6K.Tif]

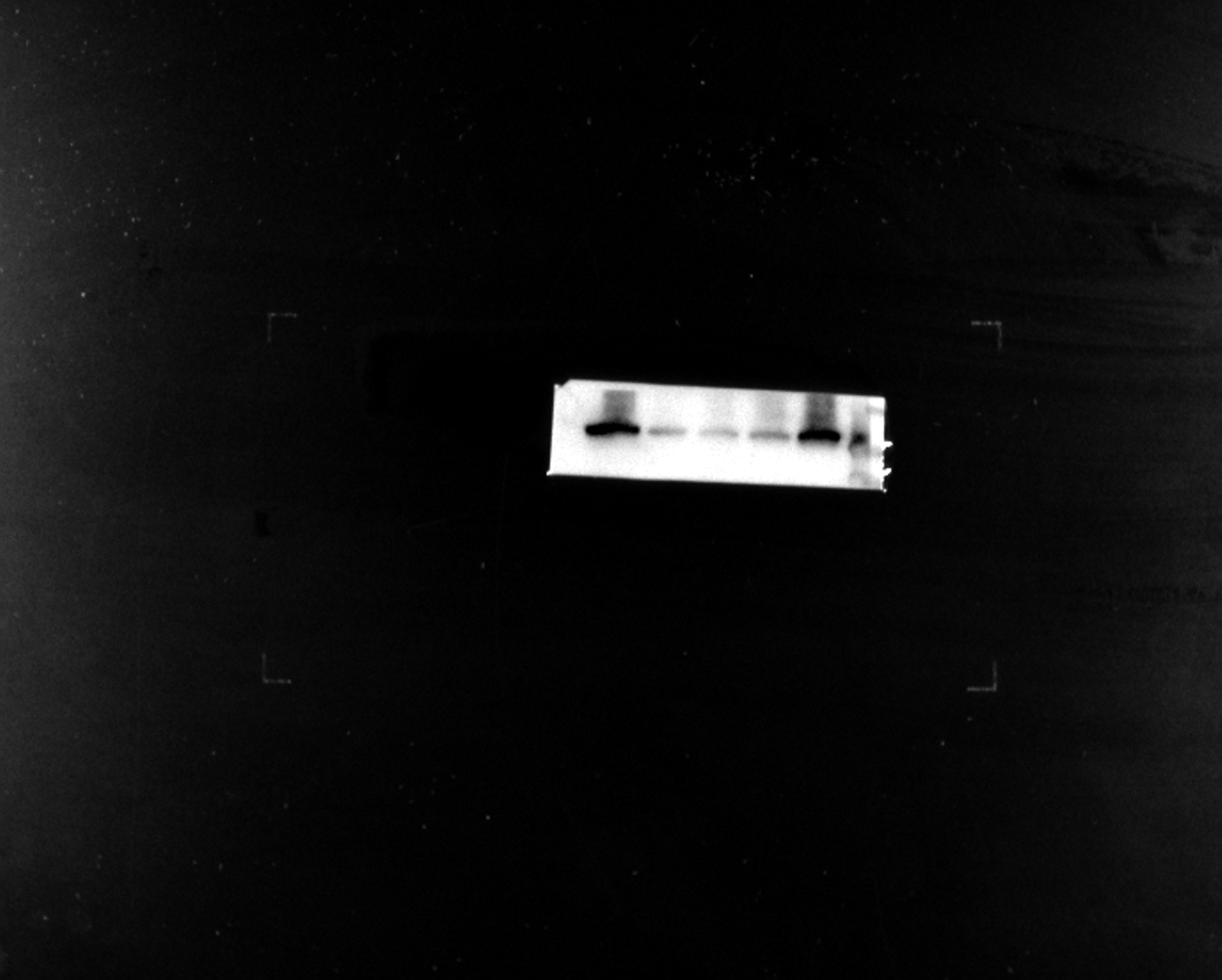

Supplement: Supplementary file 12 — Source data Fig. 7 [file 44318_2024_359_MOESM12_ESM.zip › Figure 7/Fig 7D/5-p-S6-merge.Tif]

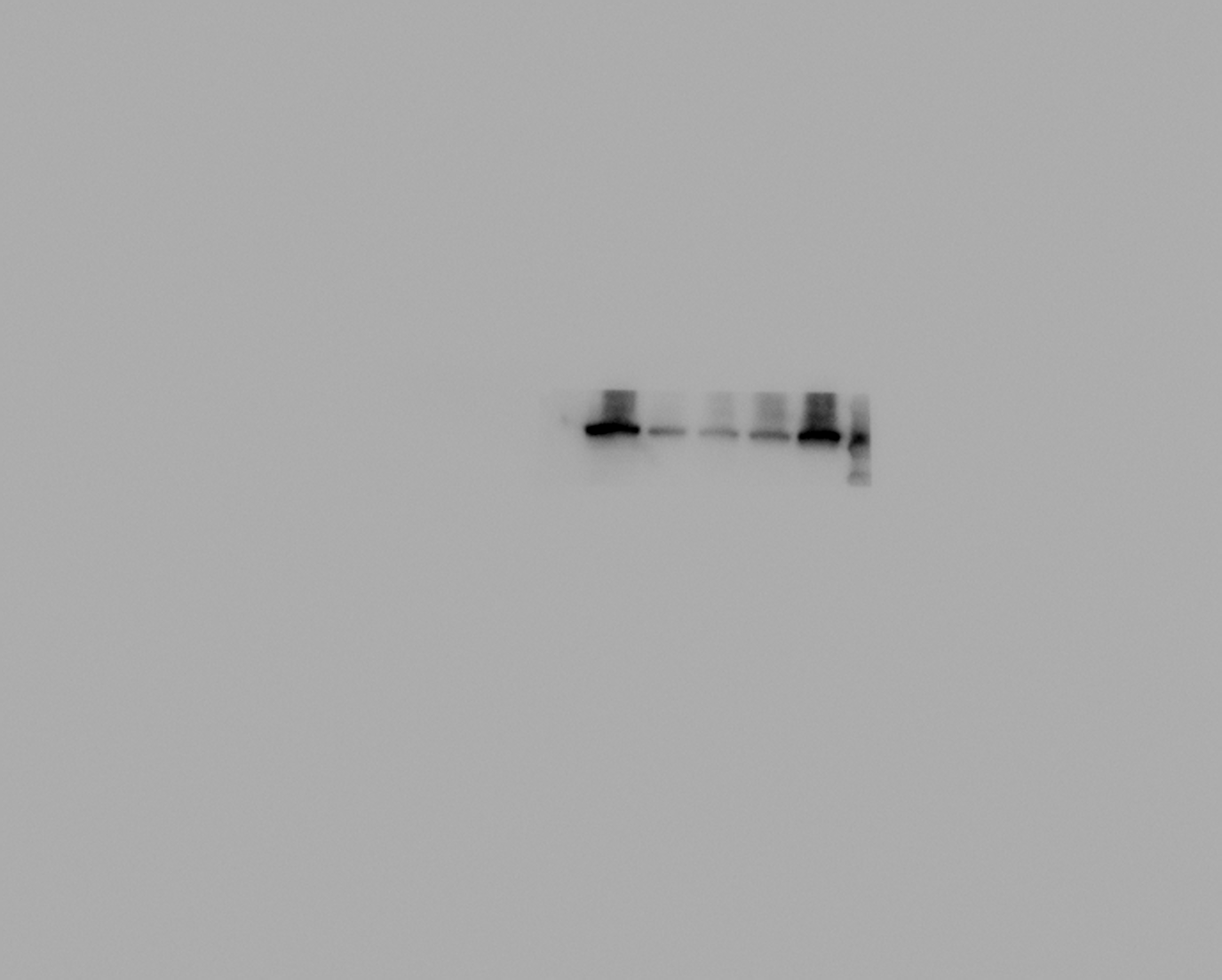

Supplement: Supplementary file 12 — Source data Fig. 7 [file 44318_2024_359_MOESM12_ESM.zip › Figure 7/Fig 7D/5-p-S6.Tif]

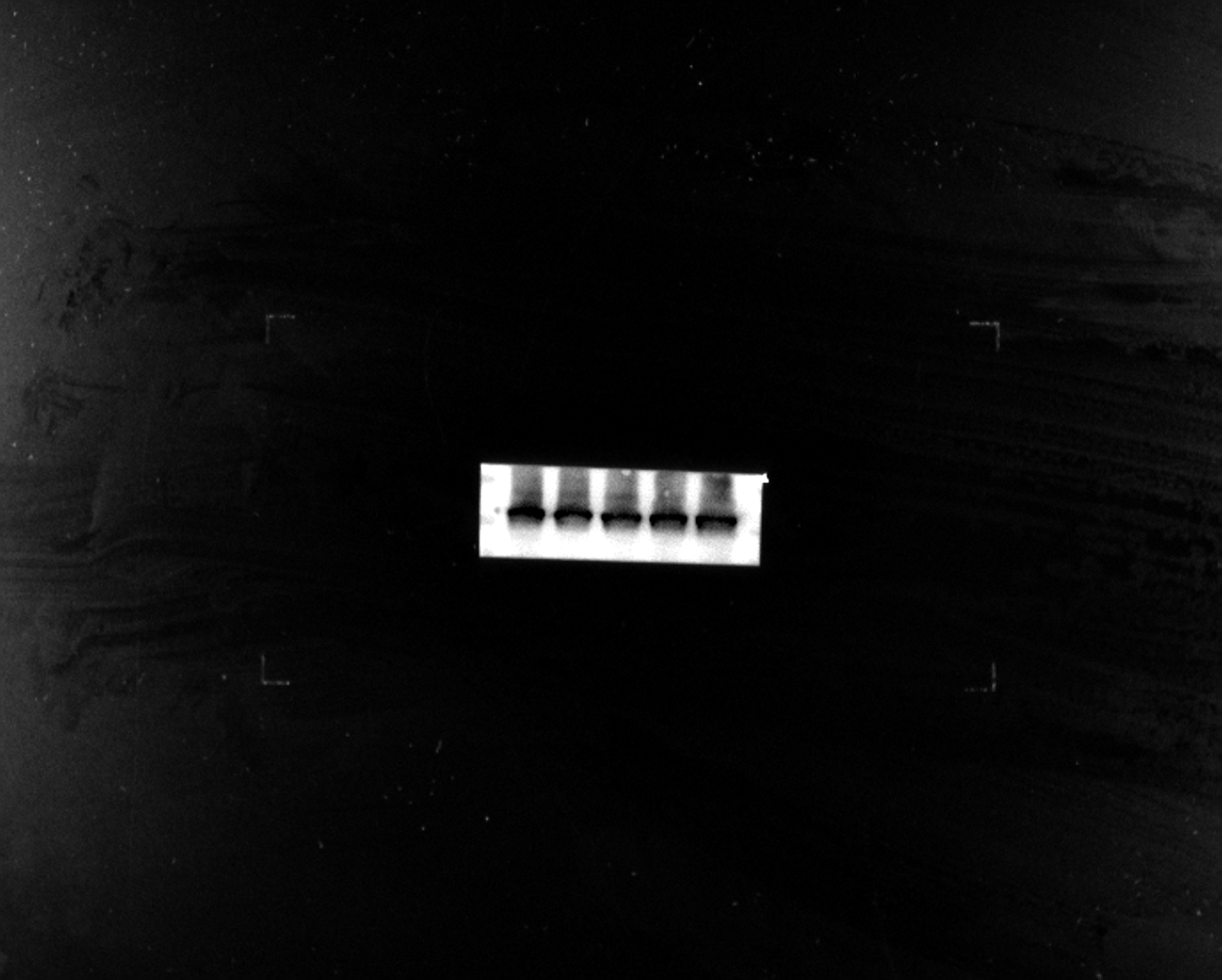

Supplement: Supplementary file 12 — Source data Fig. 7 [file 44318_2024_359_MOESM12_ESM.zip › Figure 7/Fig 7D/6-S6-merge.Tif]

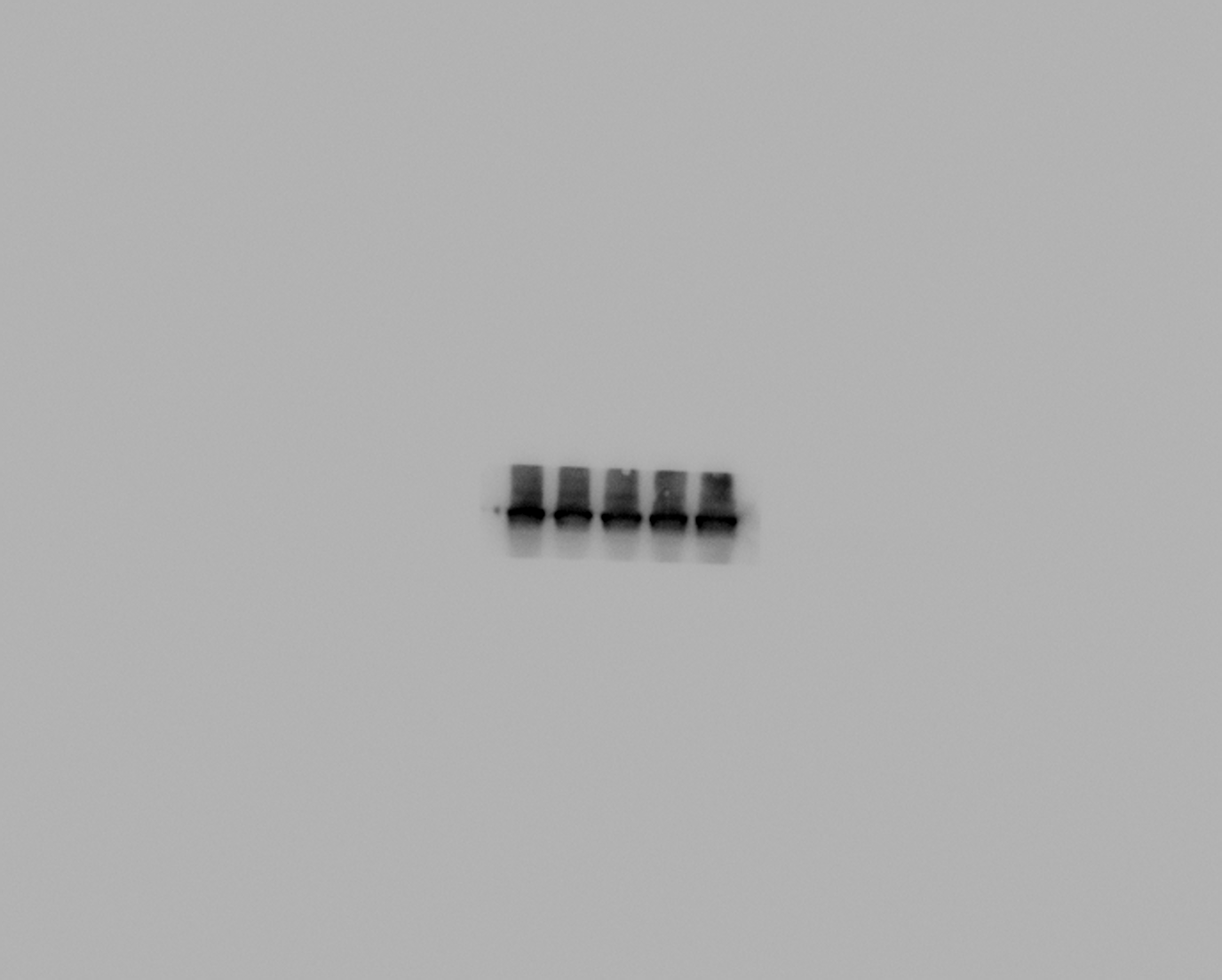

Supplement: Supplementary file 12 — Source data Fig. 7 [file 44318_2024_359_MOESM12_ESM.zip › Figure 7/Fig 7D/6-S6.Tif]

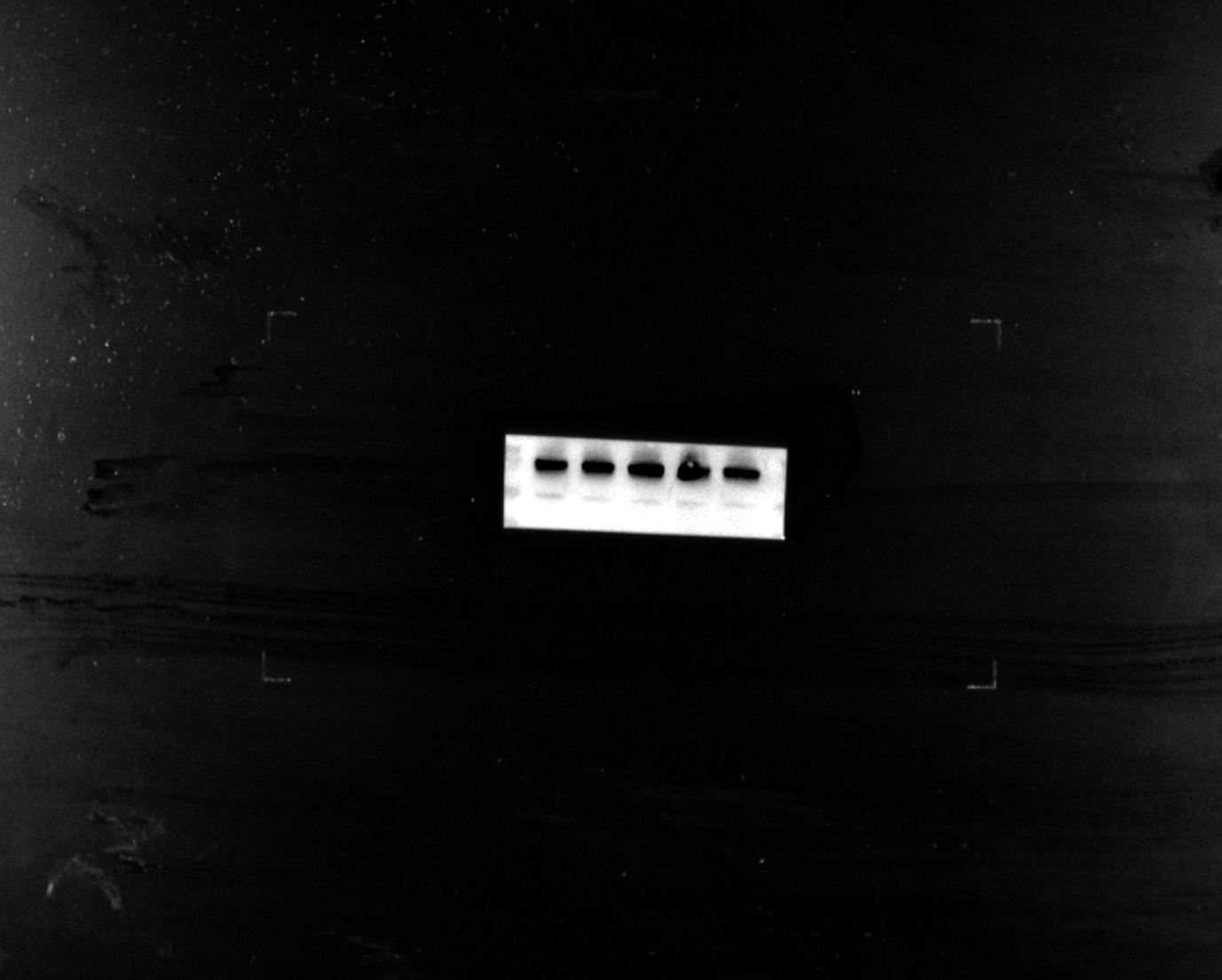

Supplement: Supplementary file 12 — Source data Fig. 7 [file 44318_2024_359_MOESM12_ESM.zip › Figure 7/Fig 7D/7-GAPDH-merge.Tif]

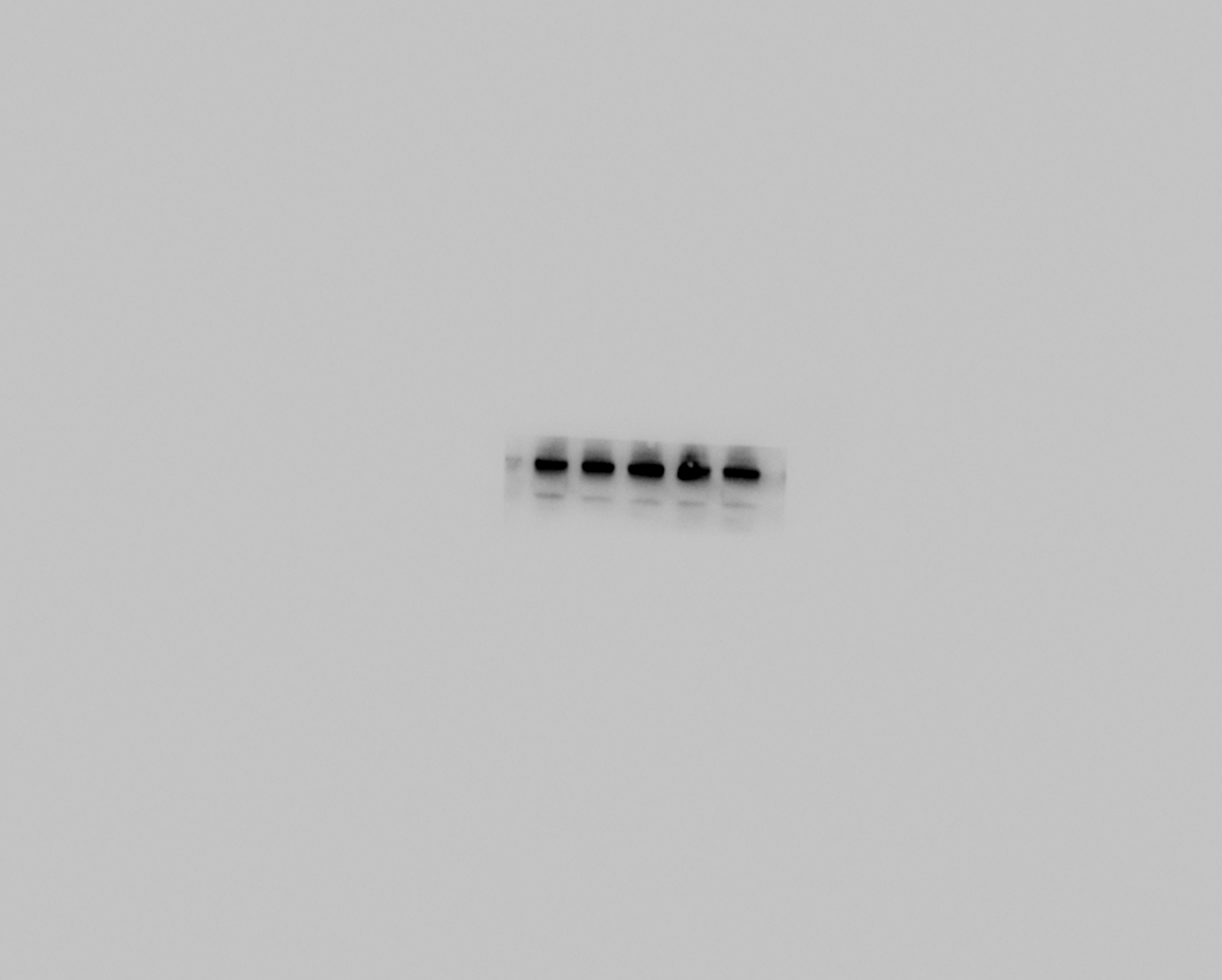

Supplement: Supplementary file 12 — Source data Fig. 7 [file 44318_2024_359_MOESM12_ESM.zip › Figure 7/Fig 7D/7-GAPDH.Tif]

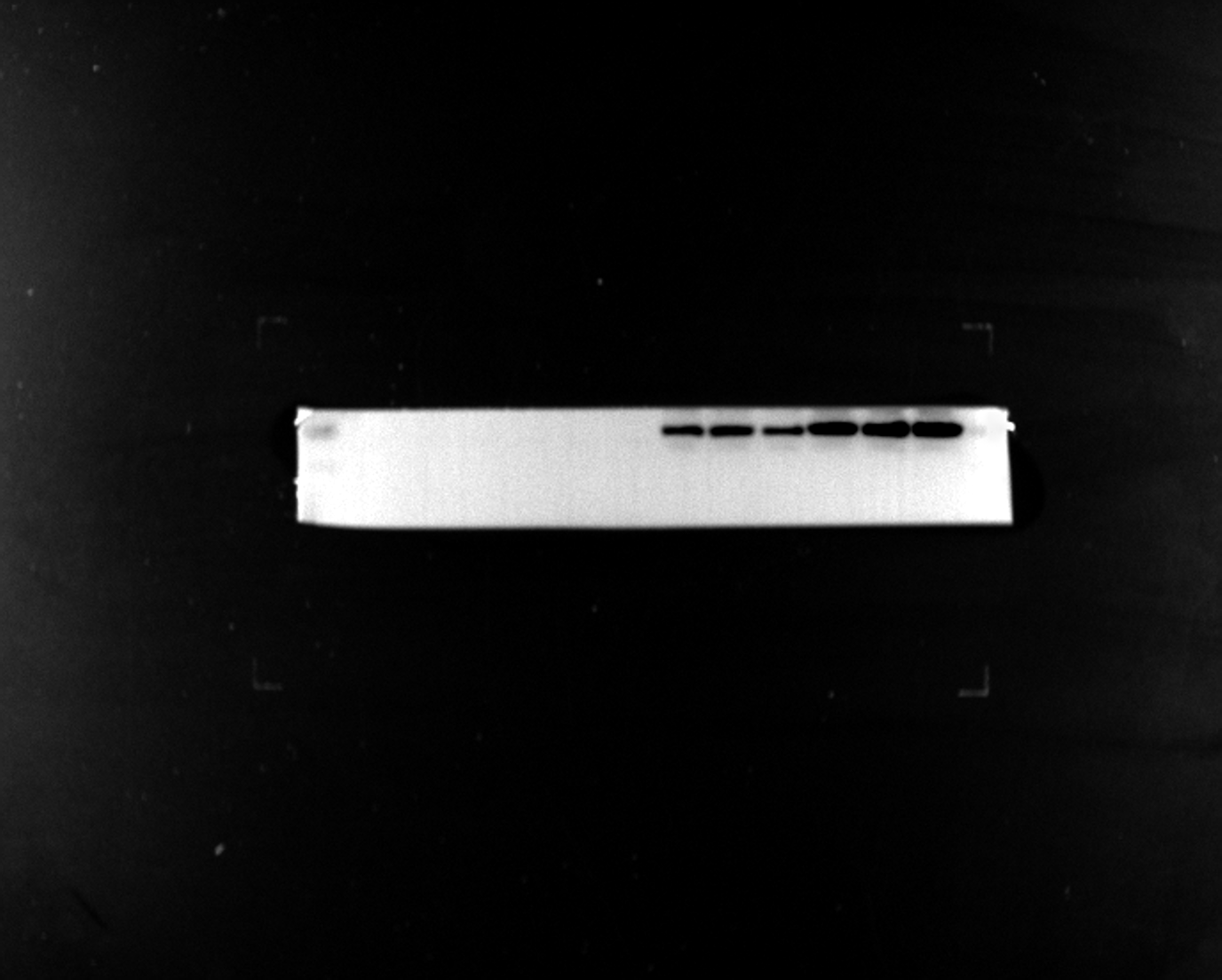

Supplement: Supplementary file 12 — Source data Fig. 7 [file 44318_2024_359_MOESM12_ESM.zip › Figure 7/Fig 7O/9-TAT-merge.Tif]

Fig 7D

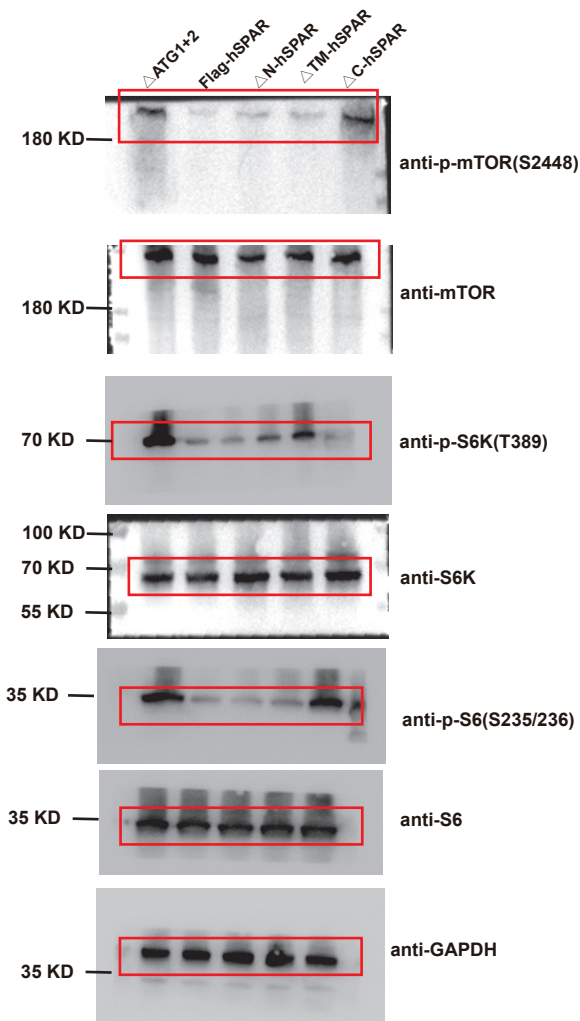

Supplement: Supplementary file 12 — Source data Fig. 7 [file 44318_2024_359_MOESM12_ESM.zip › Figure 7/Fig 7D/Fig. 7D.pdf]

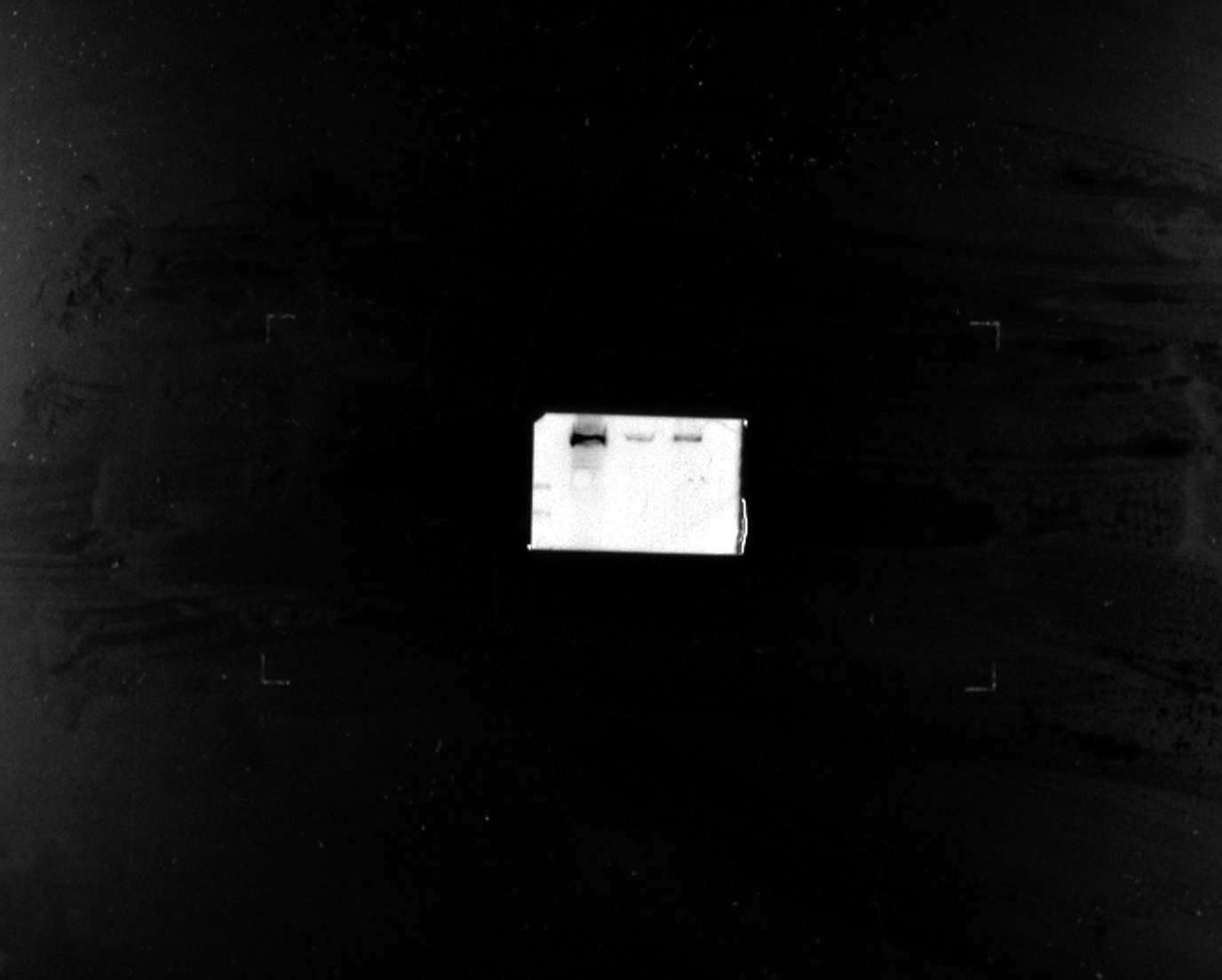

Supplement: Supplementary file 12 — Source data Fig. 7 [file 44318_2024_359_MOESM12_ESM.zip › Figure 7/Fig 7E/1-p-mTOR-merge.Tif]

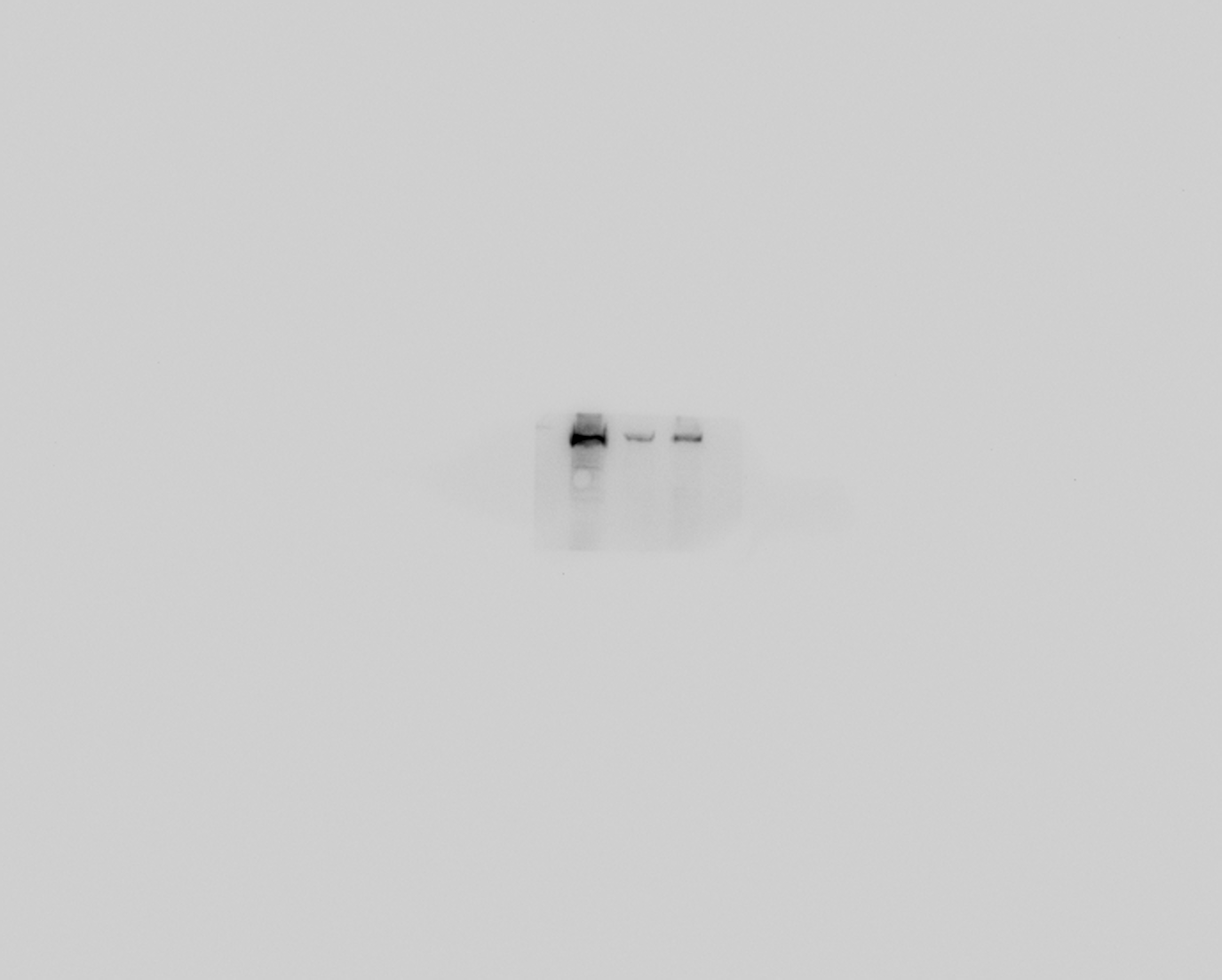

Supplement: Supplementary file 12 — Source data Fig. 7 [file 44318_2024_359_MOESM12_ESM.zip › Figure 7/Fig 7E/1-p-mTOR.Tif]

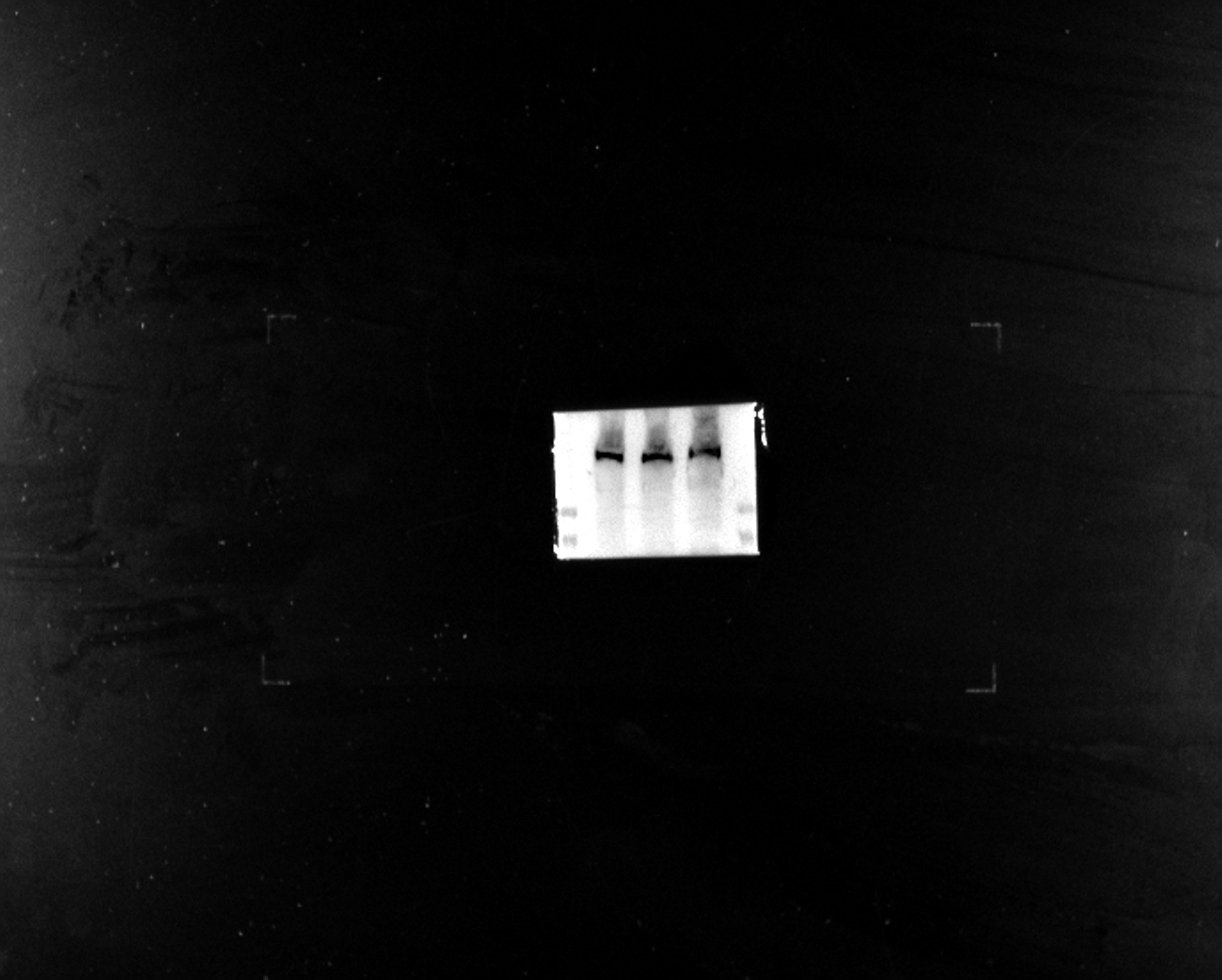

Supplement: Supplementary file 12 — Source data Fig. 7 [file 44318_2024_359_MOESM12_ESM.zip › Figure 7/Fig 7E/2-mTOR-merge.Tif]

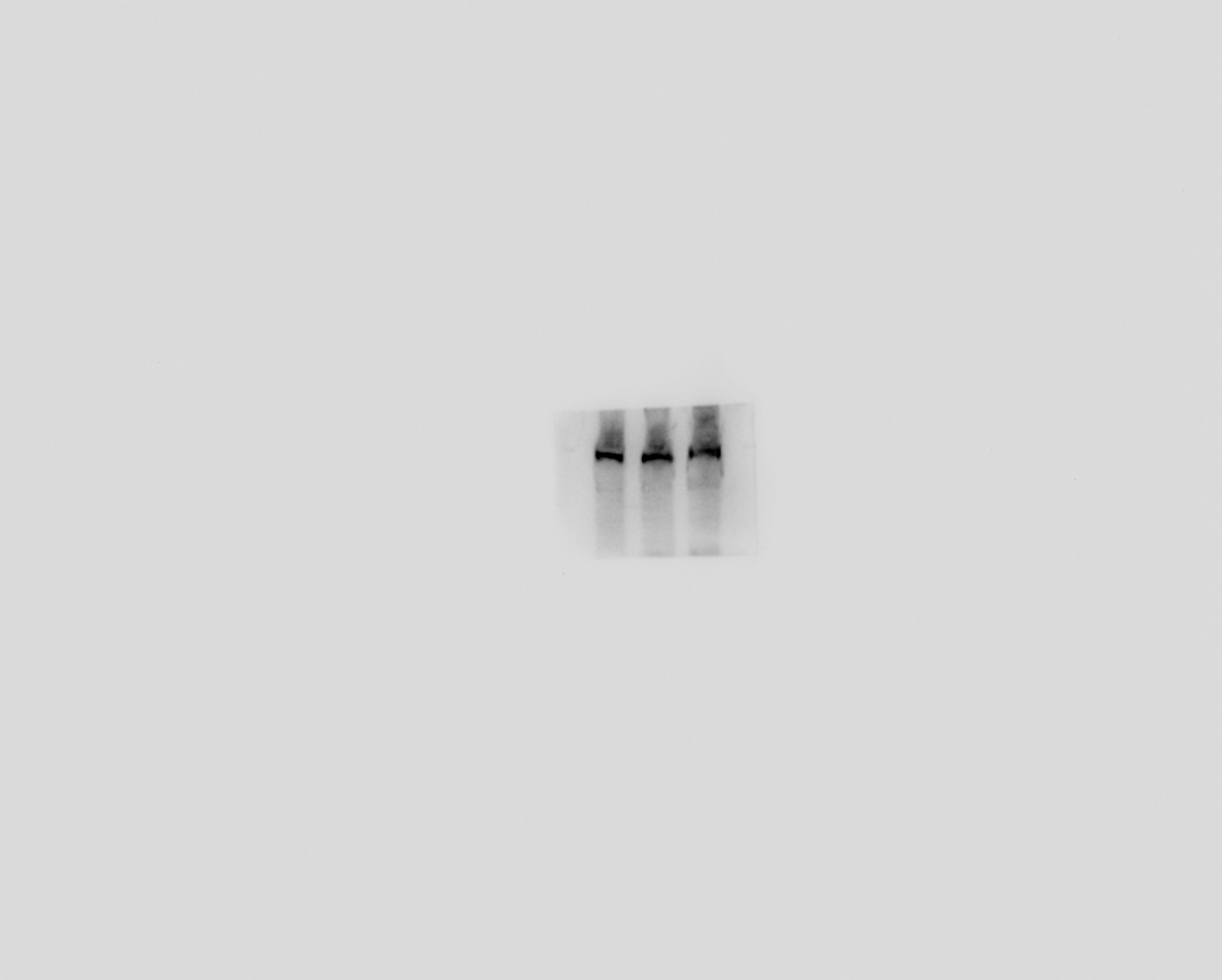

Supplement: Supplementary file 12 — Source data Fig. 7 [file 44318_2024_359_MOESM12_ESM.zip › Figure 7/Fig 7E/2-mTOR.Tif]

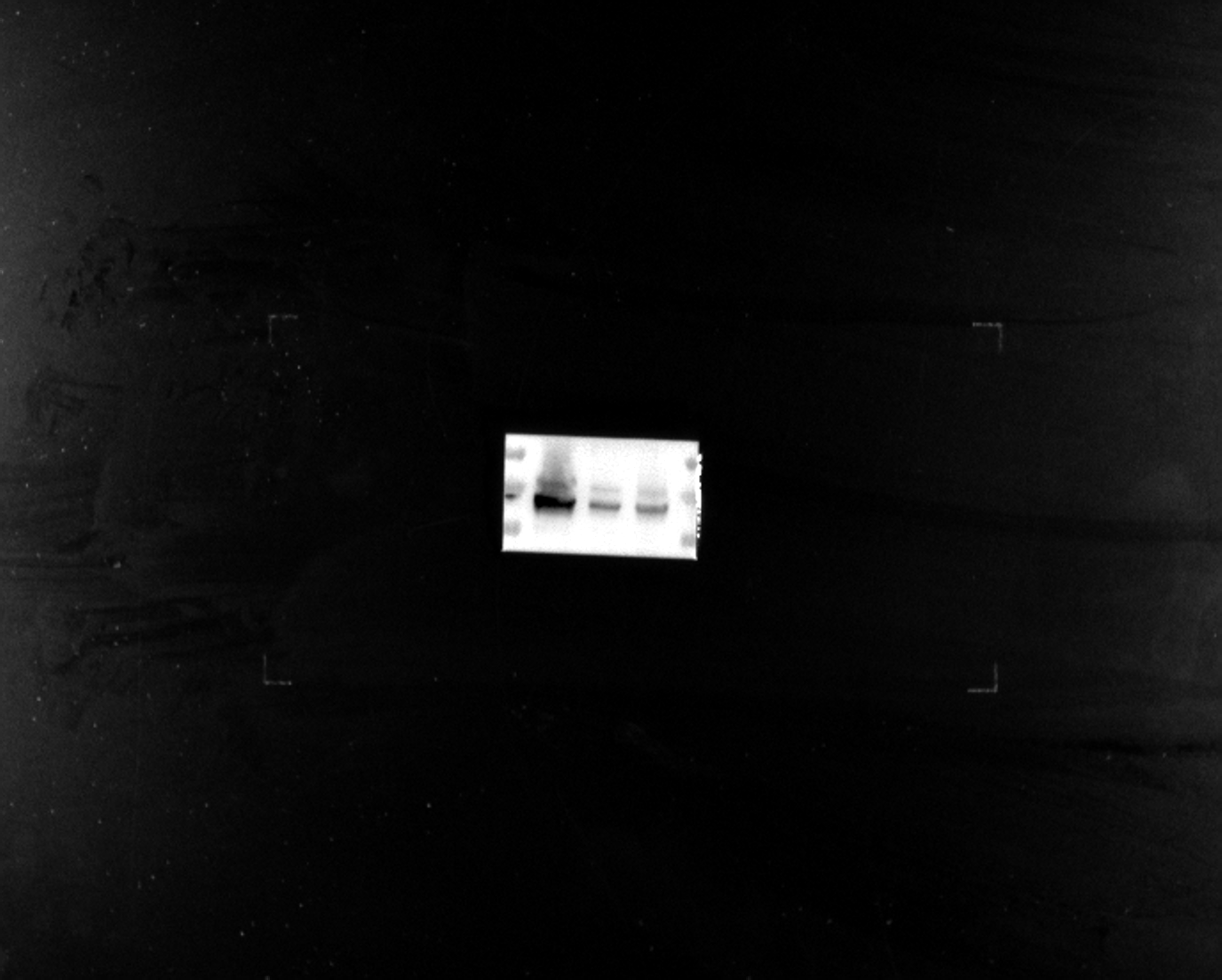

Supplement: Supplementary file 12 — Source data Fig. 7 [file 44318_2024_359_MOESM12_ESM.zip › Figure 7/Fig 7E/3-p-S6K-merge.Tif]

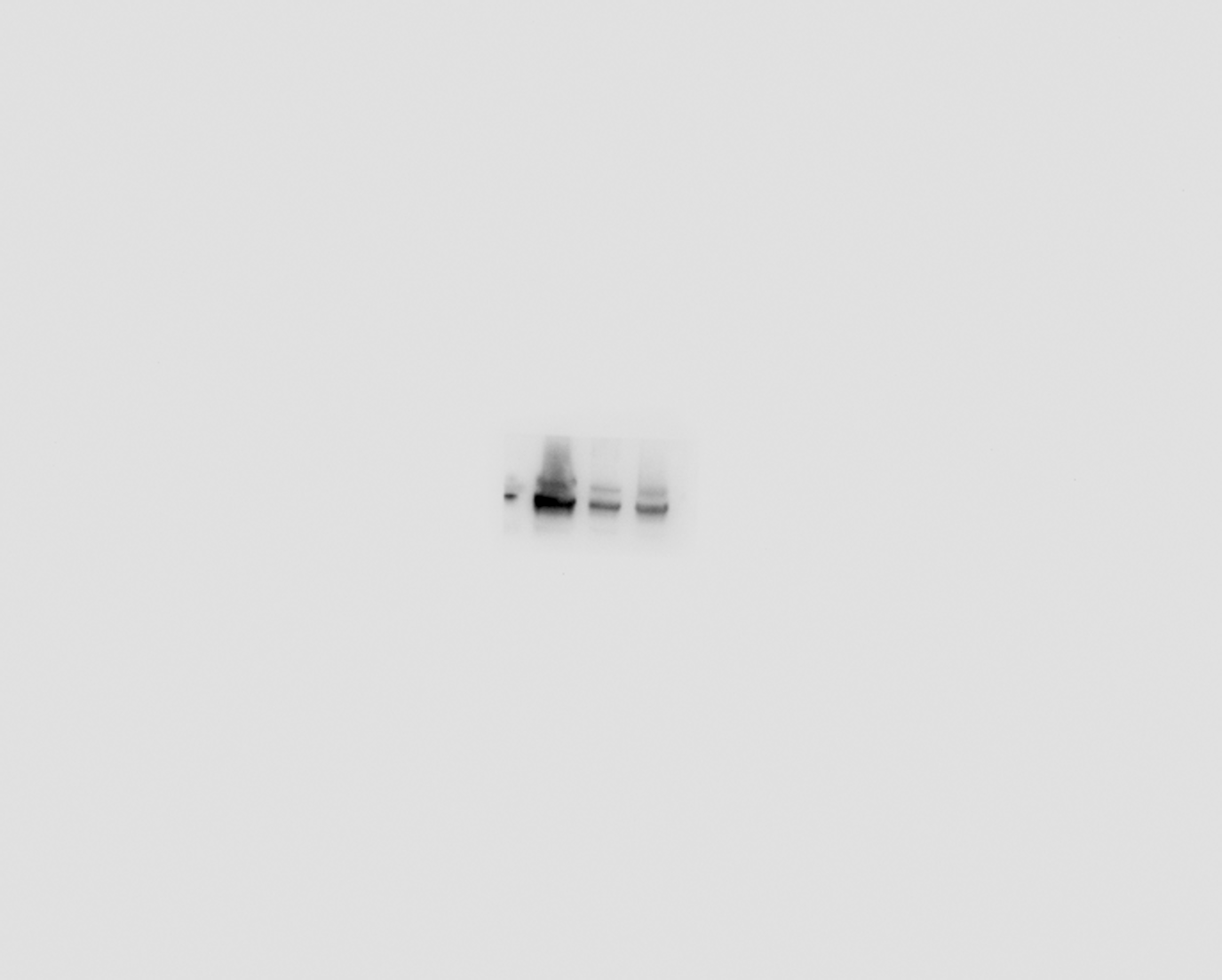

Supplement: Supplementary file 12 — Source data Fig. 7 [file 44318_2024_359_MOESM12_ESM.zip › Figure 7/Fig 7E/3-p-S6K.Tif]

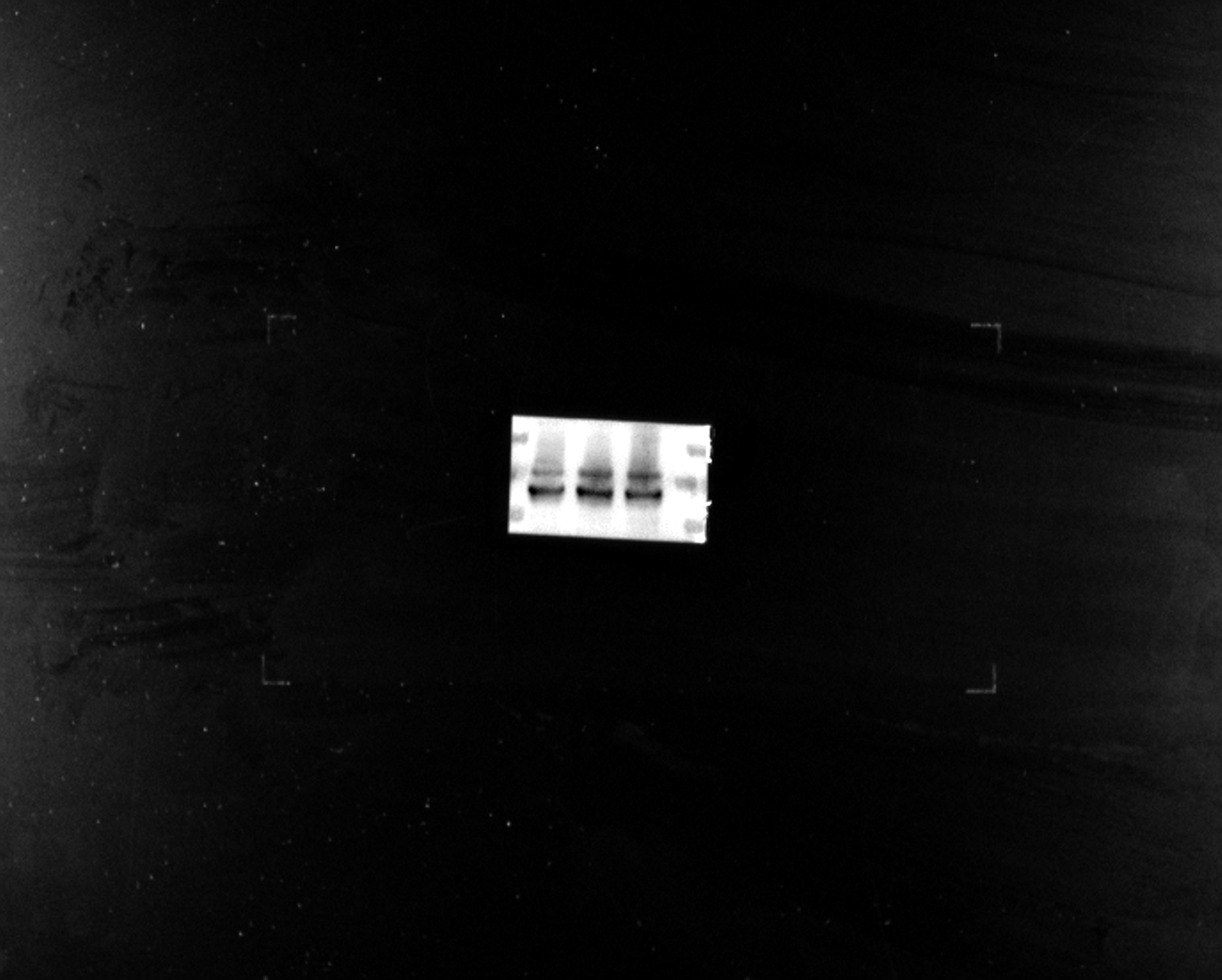

Supplement: Supplementary file 12 — Source data Fig. 7 [file 44318_2024_359_MOESM12_ESM.zip › Figure 7/Fig 7E/4-S6K-merge.Tif]

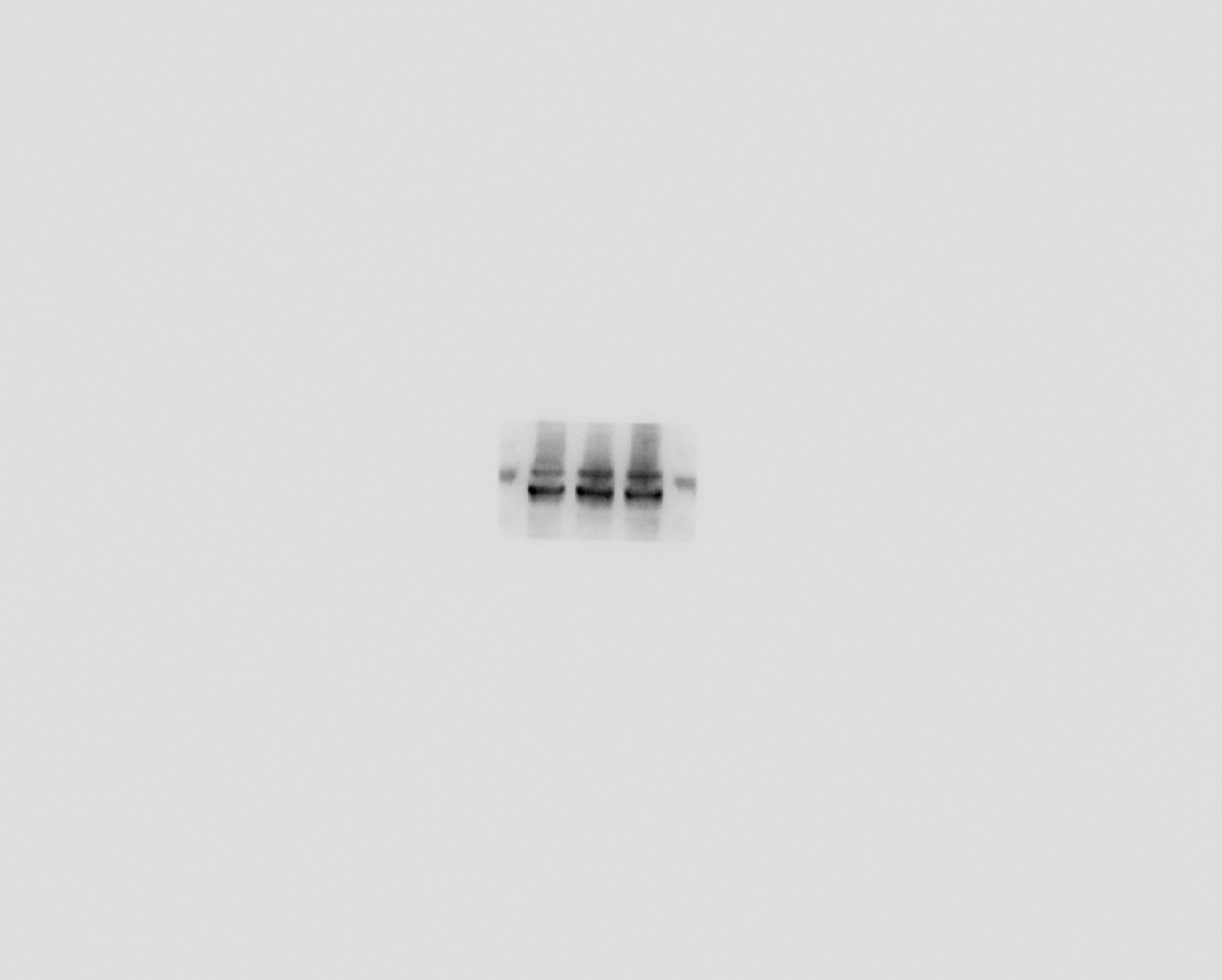

Supplement: Supplementary file 12 — Source data Fig. 7 [file 44318_2024_359_MOESM12_ESM.zip › Figure 7/Fig 7E/4-S6K.Tif]
