# Supplementary figures and images for: Micropeptide hSPAR regulates glutamine levels and suppresses mammary tumor growth via a TRIM21-P27KIP1-mTOR axis (part 7 of 7)
Source: EMBO J. 2025 Jan 28;44(5):1414–41. doi: 10.1038/s44318-024-00359-z (PMC11876615; doi:10.1038/s44318-024-00359-z)

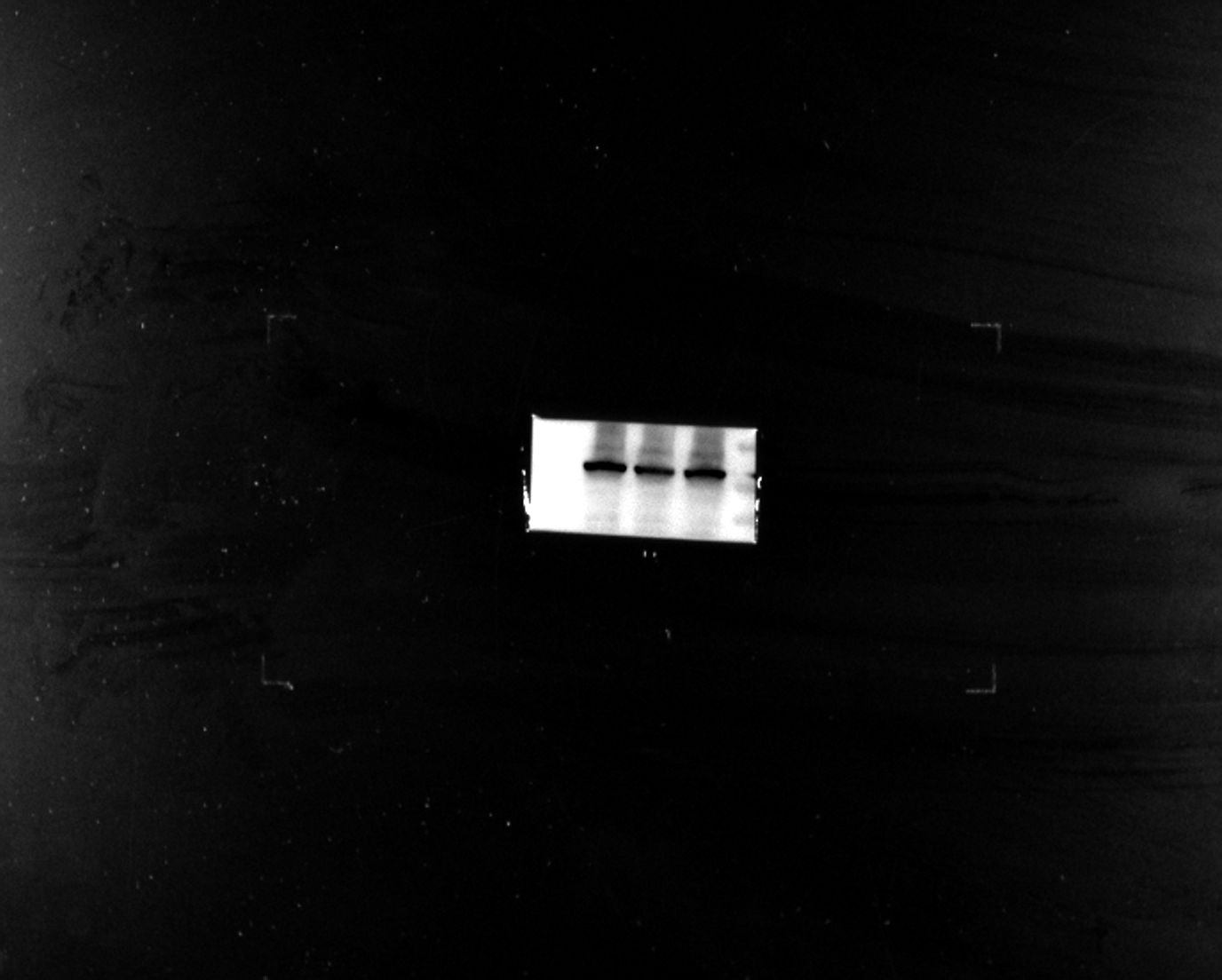

Supplement: Supplementary file 12 — Source data Fig. 7 [file 44318_2024_359_MOESM12_ESM.zip › Figure 7/Fig 7E/5-S6-merge.Tif]

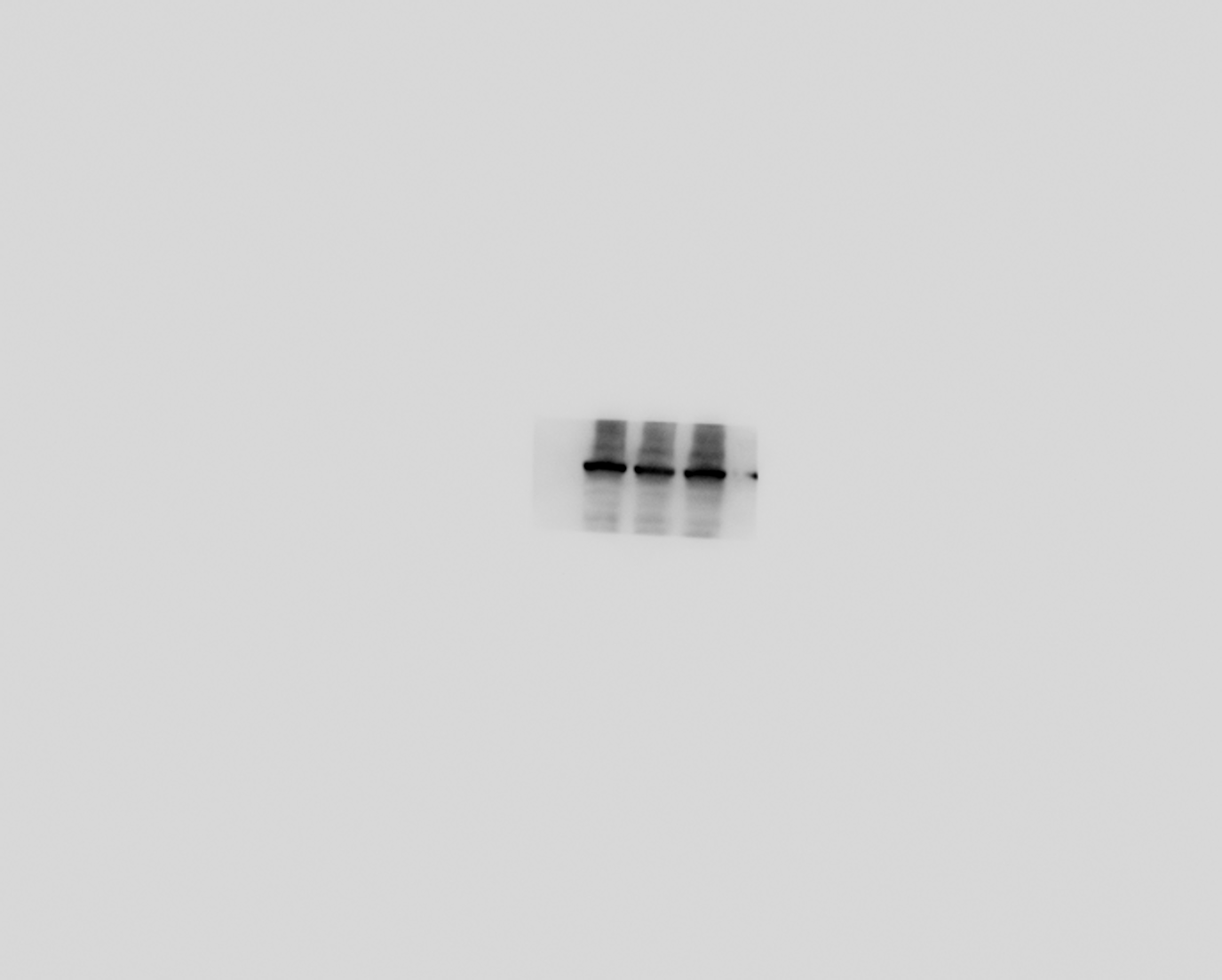

Supplement: Supplementary file 12 — Source data Fig. 7 [file 44318_2024_359_MOESM12_ESM.zip › Figure 7/Fig 7E/5-S6.Tif]

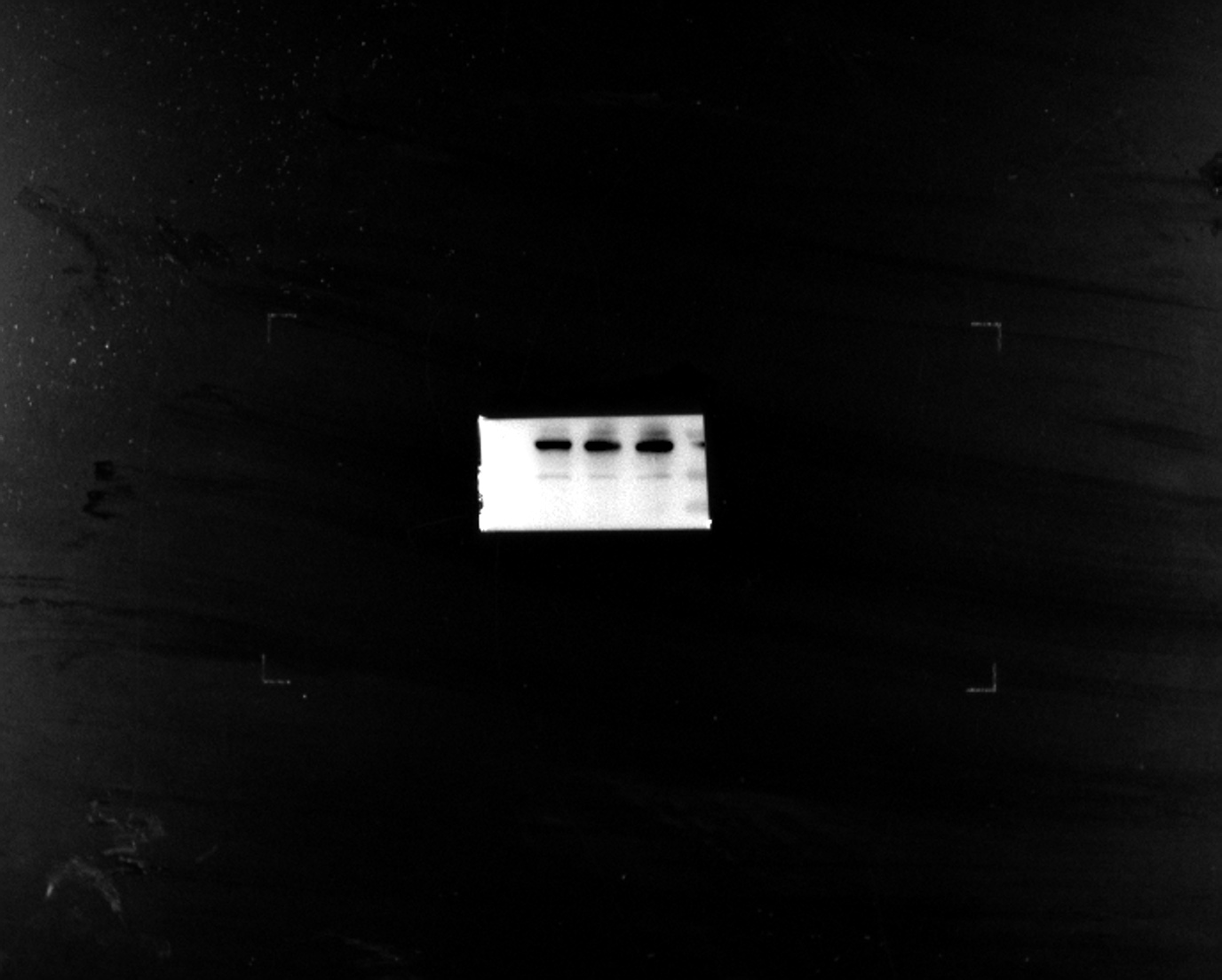

Supplement: Supplementary file 12 — Source data Fig. 7 [file 44318_2024_359_MOESM12_ESM.zip › Figure 7/Fig 7E/6-GAPDH-merge.Tif]

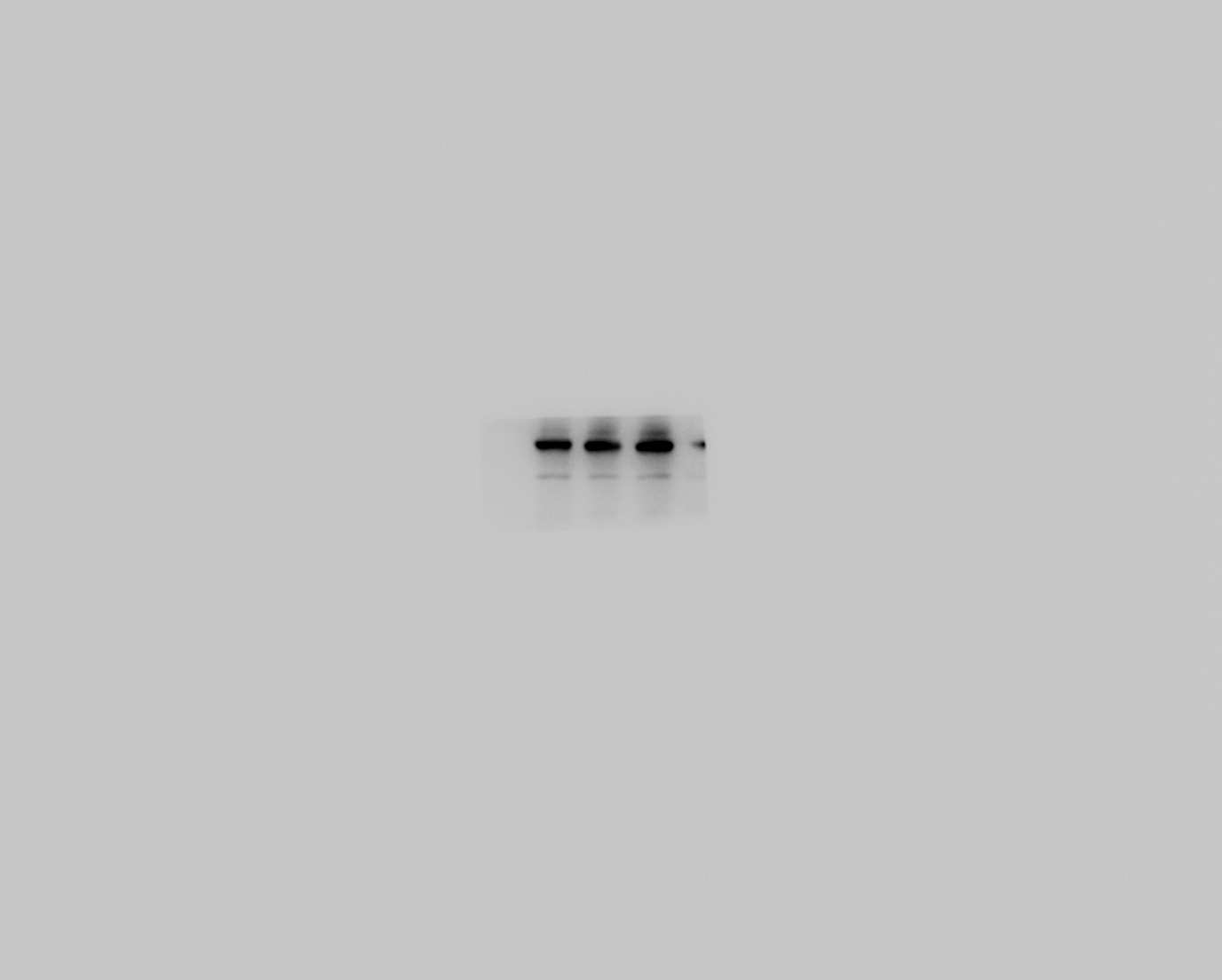

Supplement: Supplementary file 12 — Source data Fig. 7 [file 44318_2024_359_MOESM12_ESM.zip › Figure 7/Fig 7E/6-GAPDH.Tif]

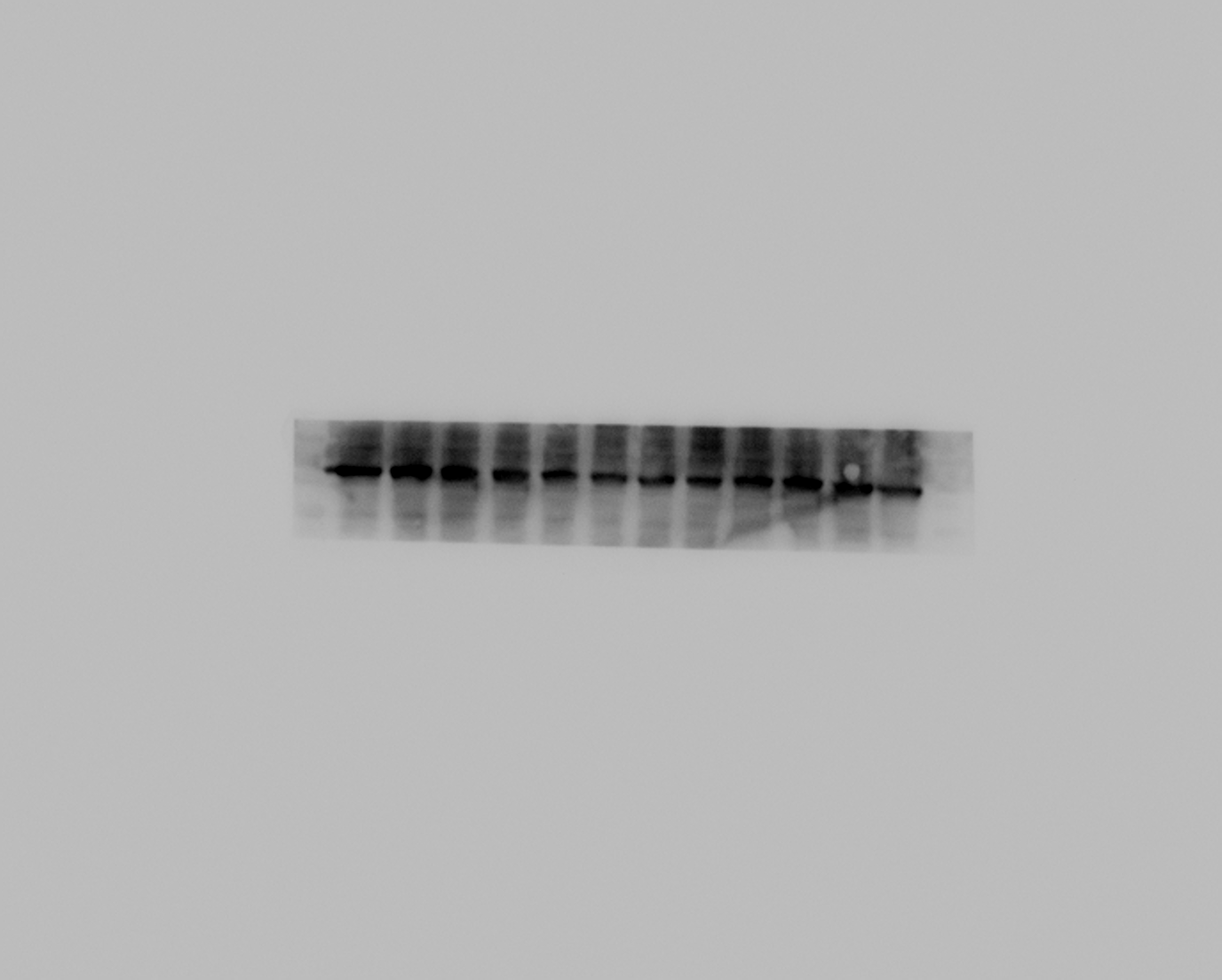

Supplement: Supplementary file 12 — Source data Fig. 7 [file 44318_2024_359_MOESM12_ESM.zip › Figure 7/Fig 7O/8-S6.Tif]

Fig 7E

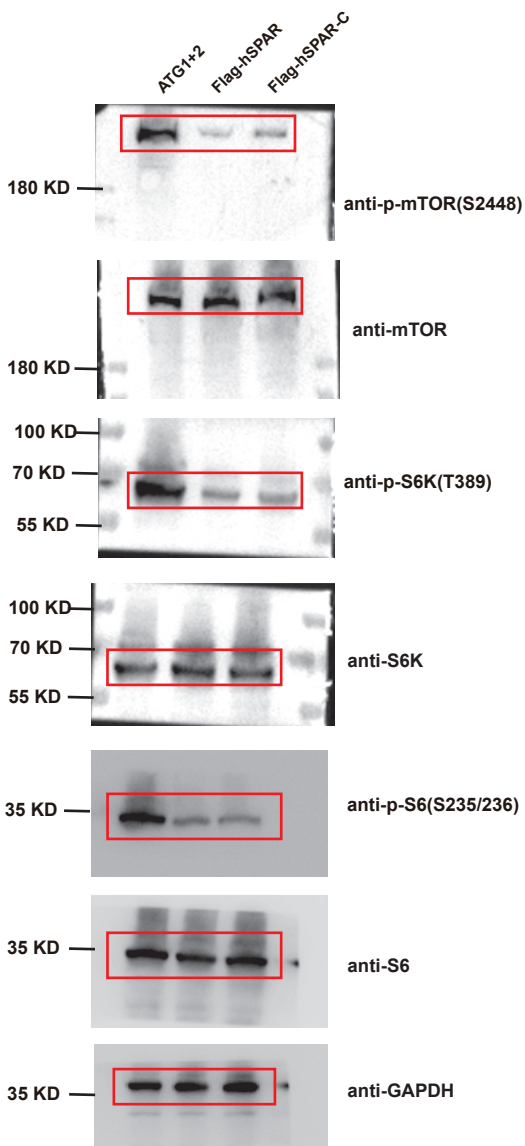

Supplement: Supplementary file 12 — Source data Fig. 7 [file 44318_2024_359_MOESM12_ESM.zip › Figure 7/Fig 7E/Fig. 7E.pdf]

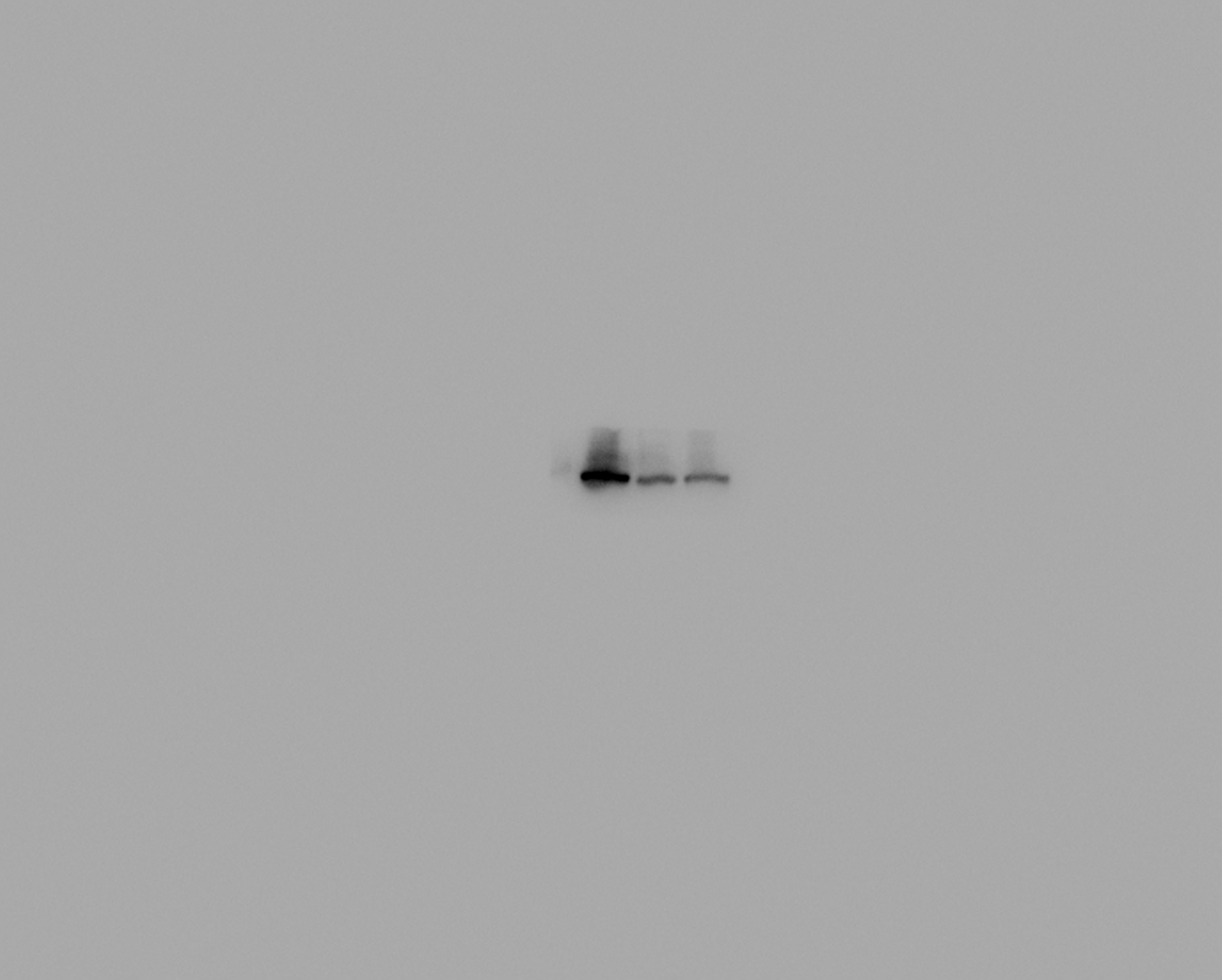

Supplement: Supplementary file 12 — Source data Fig. 7 [file 44318_2024_359_MOESM12_ESM.zip › Figure 7/Fig 7E/p-s6-1.Tif]

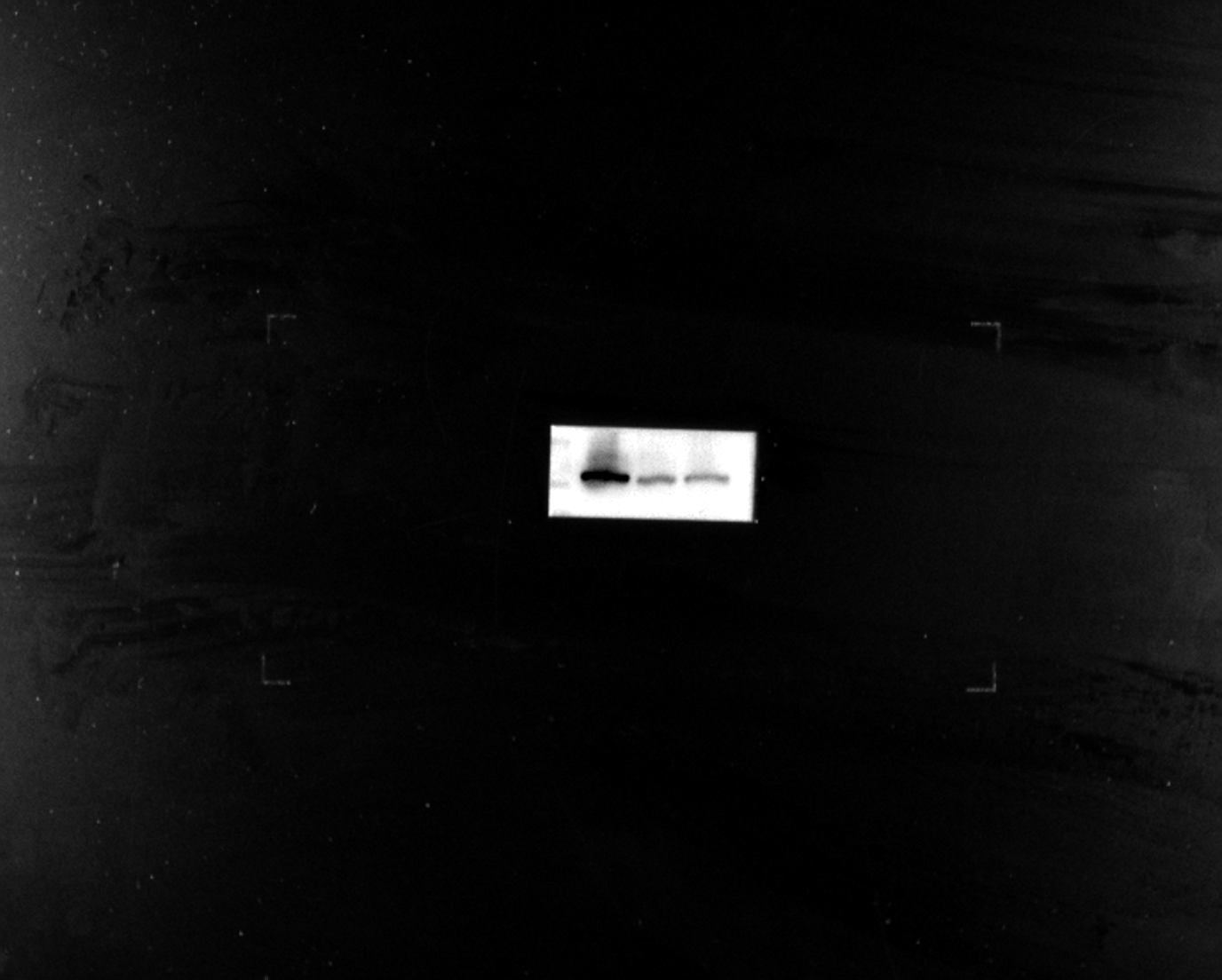

Supplement: Supplementary file 12 — Source data Fig. 7 [file 44318_2024_359_MOESM12_ESM.zip › Figure 7/Fig 7E/p-s6-2.Tif]

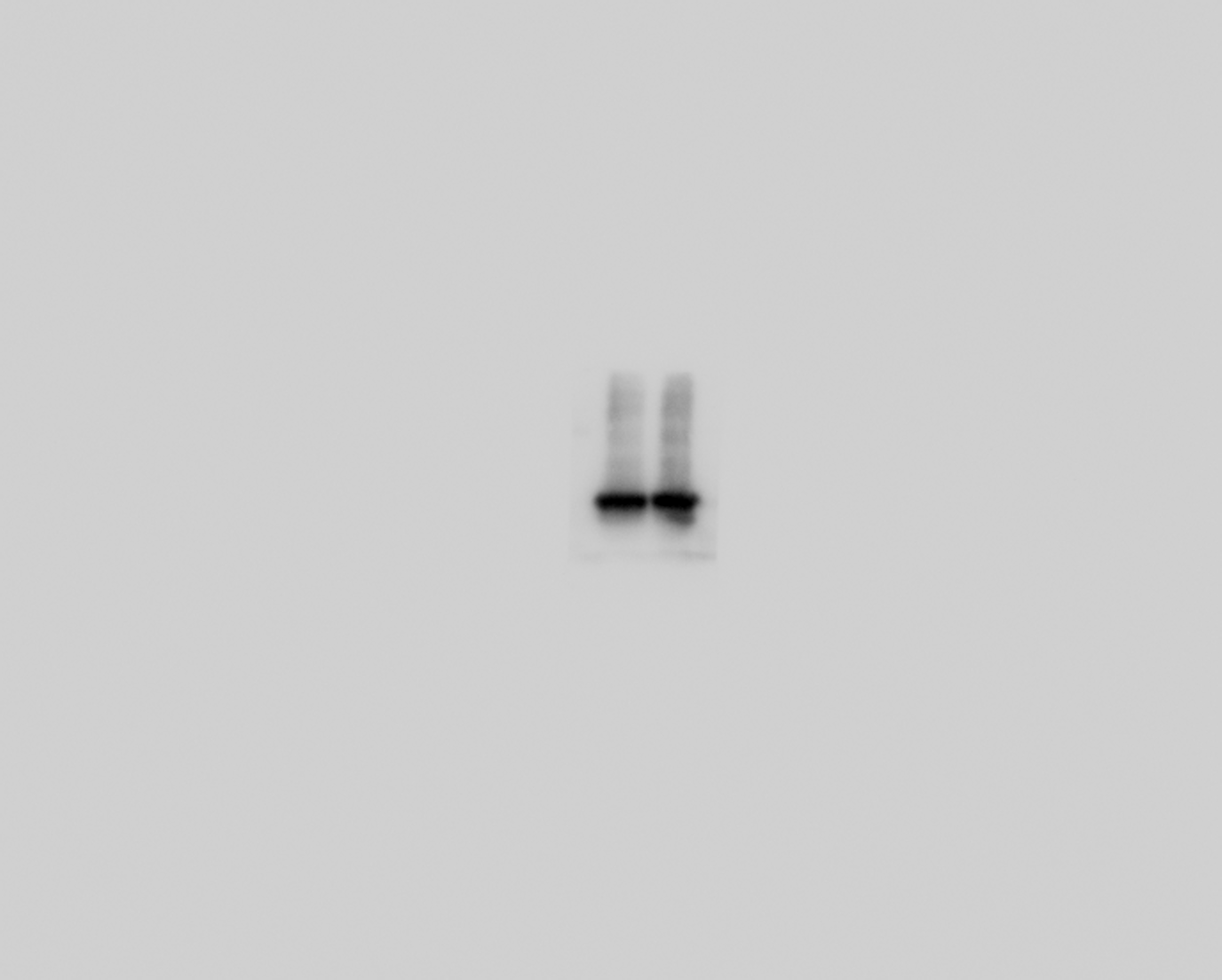

Supplement: Supplementary file 12 — Source data Fig. 7 [file 44318_2024_359_MOESM12_ESM.zip › Figure 7/Fig 7F/1-S6-1.Tif]

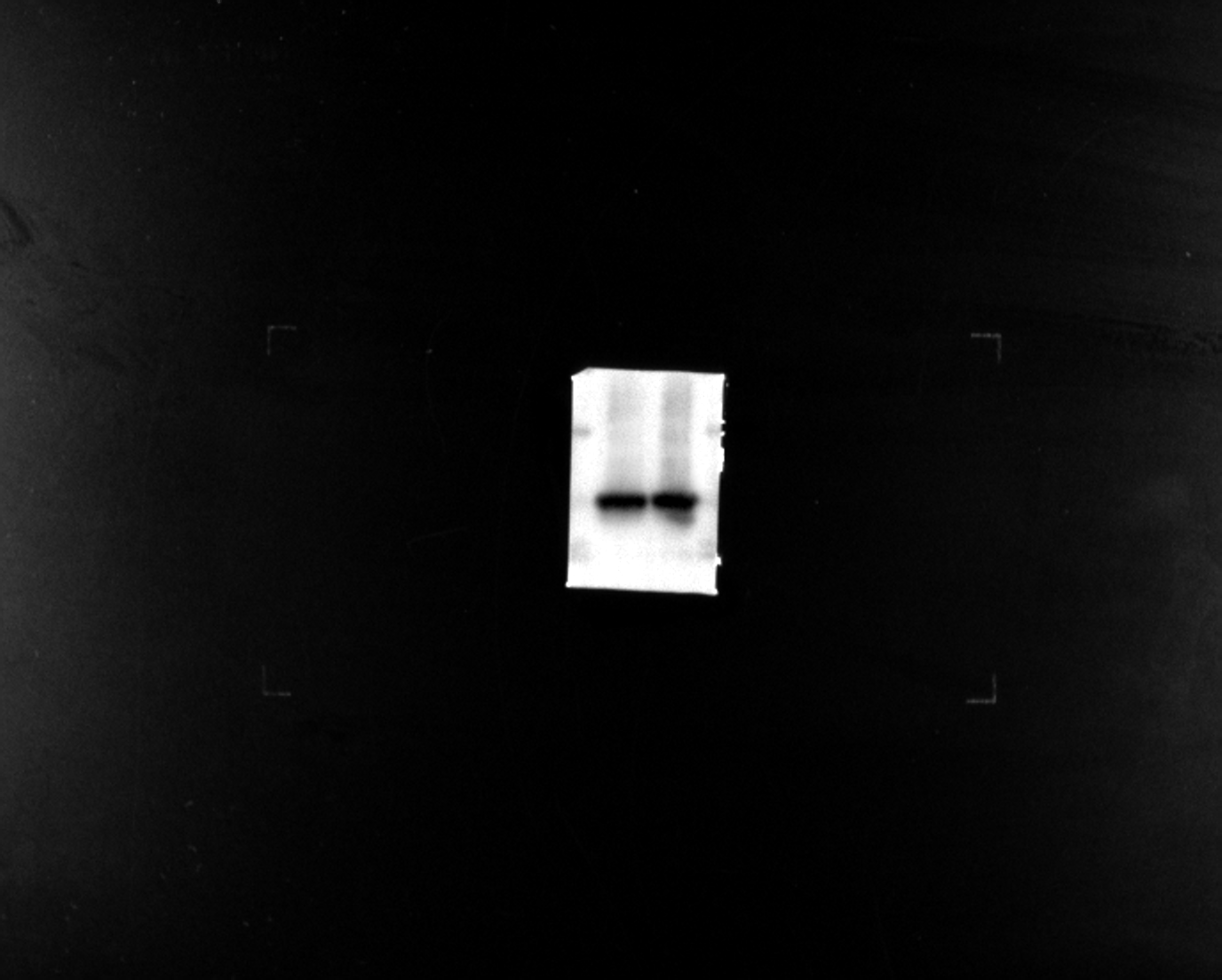

Supplement: Supplementary file 12 — Source data Fig. 7 [file 44318_2024_359_MOESM12_ESM.zip › Figure 7/Fig 7F/1-S6-2.Tif]

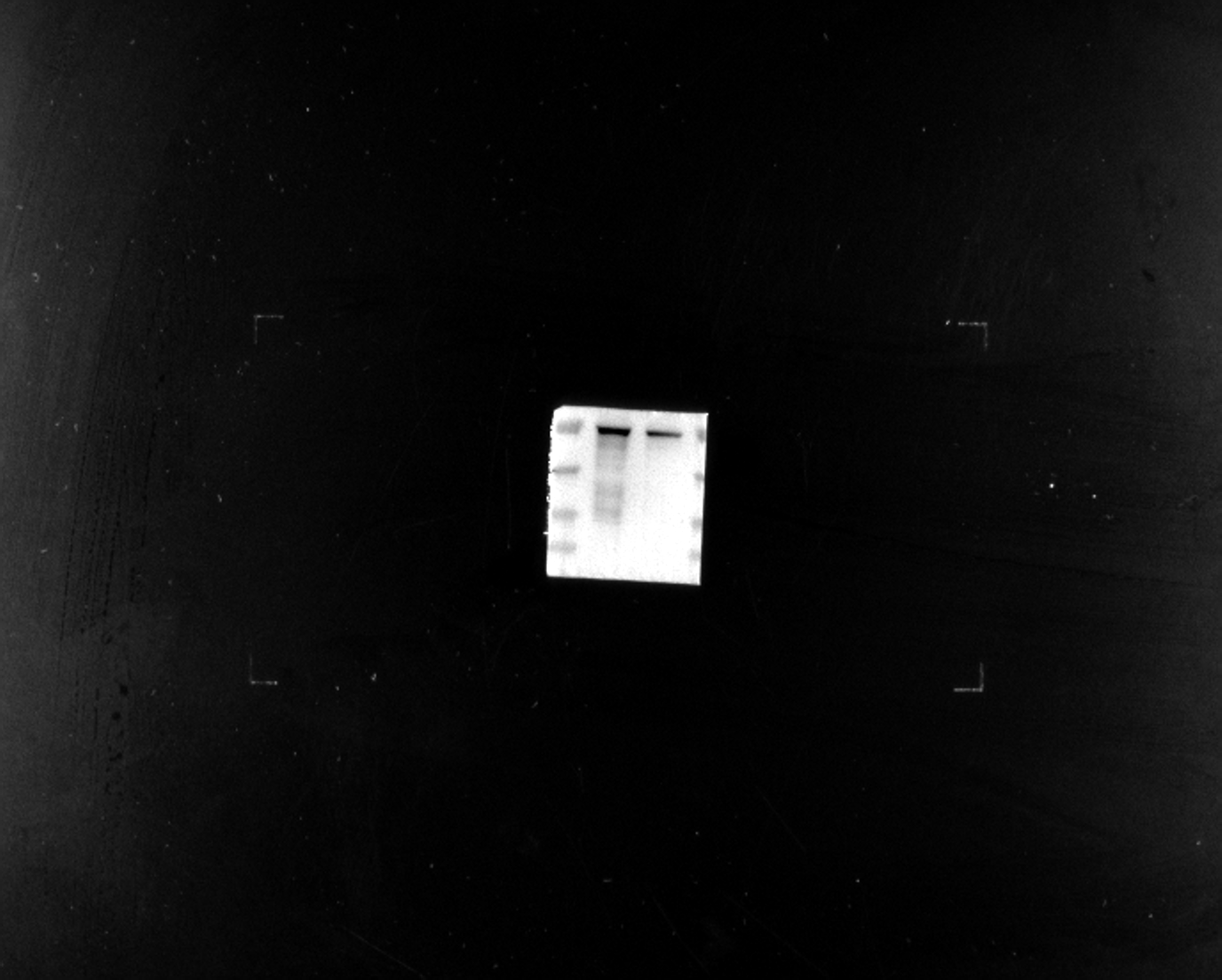

Supplement: Supplementary file 12 — Source data Fig. 7 [file 44318_2024_359_MOESM12_ESM.zip › Figure 7/Fig 7F/1-SLC38A2-merge.Tif]

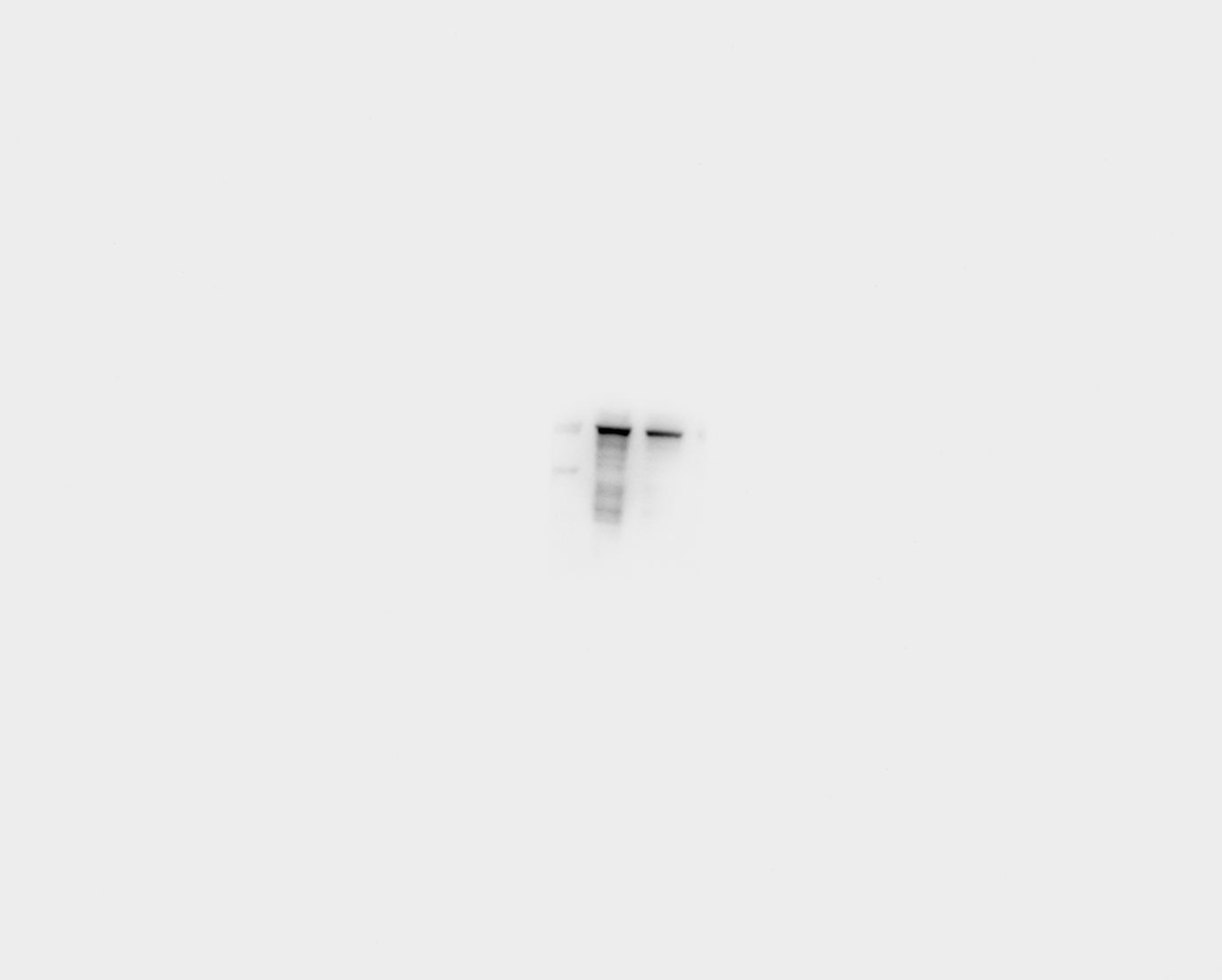

Supplement: Supplementary file 12 — Source data Fig. 7 [file 44318_2024_359_MOESM12_ESM.zip › Figure 7/Fig 7F/1-SLC38A2.Tif]

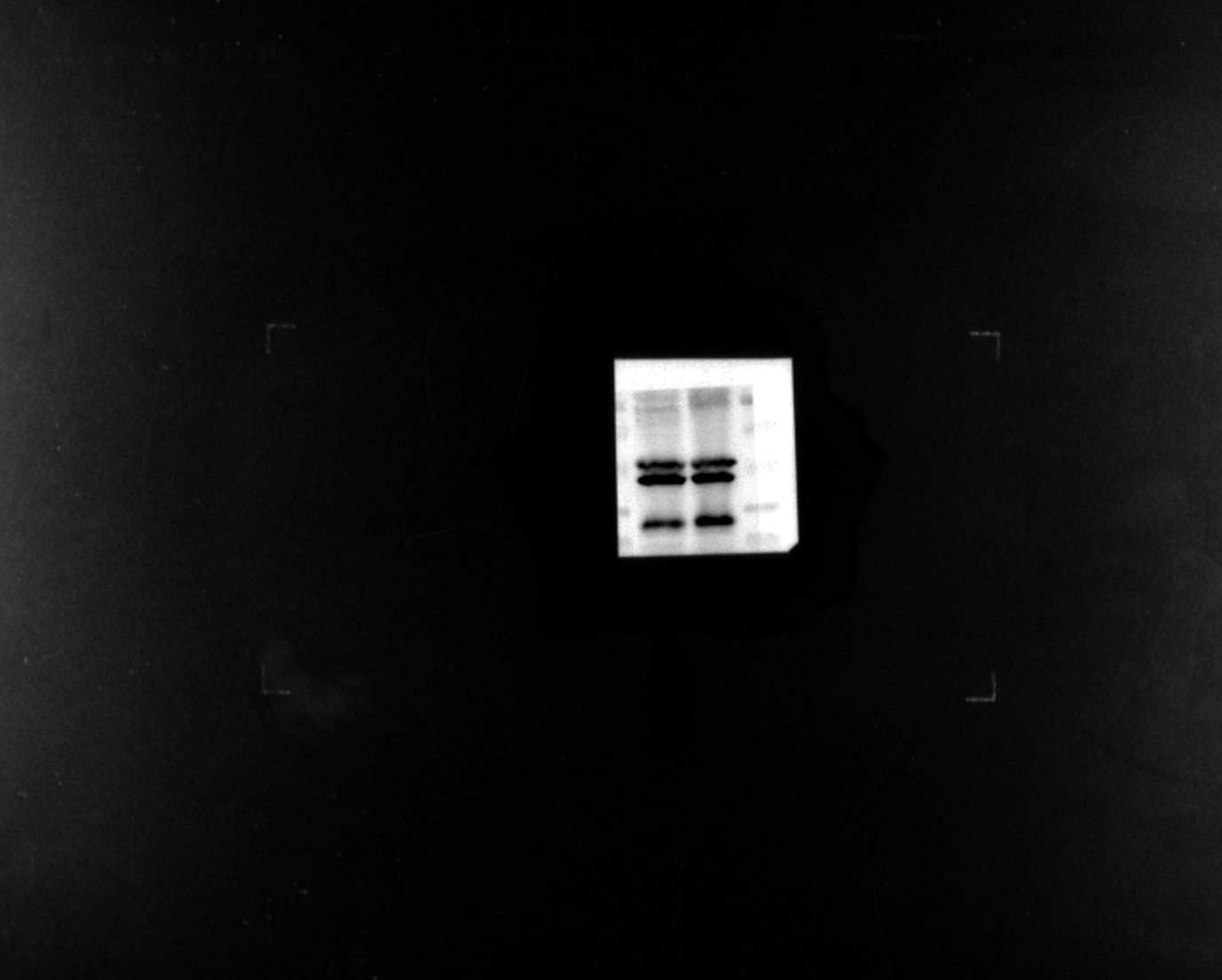

Supplement: Supplementary file 12 — Source data Fig. 7 [file 44318_2024_359_MOESM12_ESM.zip › Figure 7/Fig 7F/2-p27-merge.Tif]

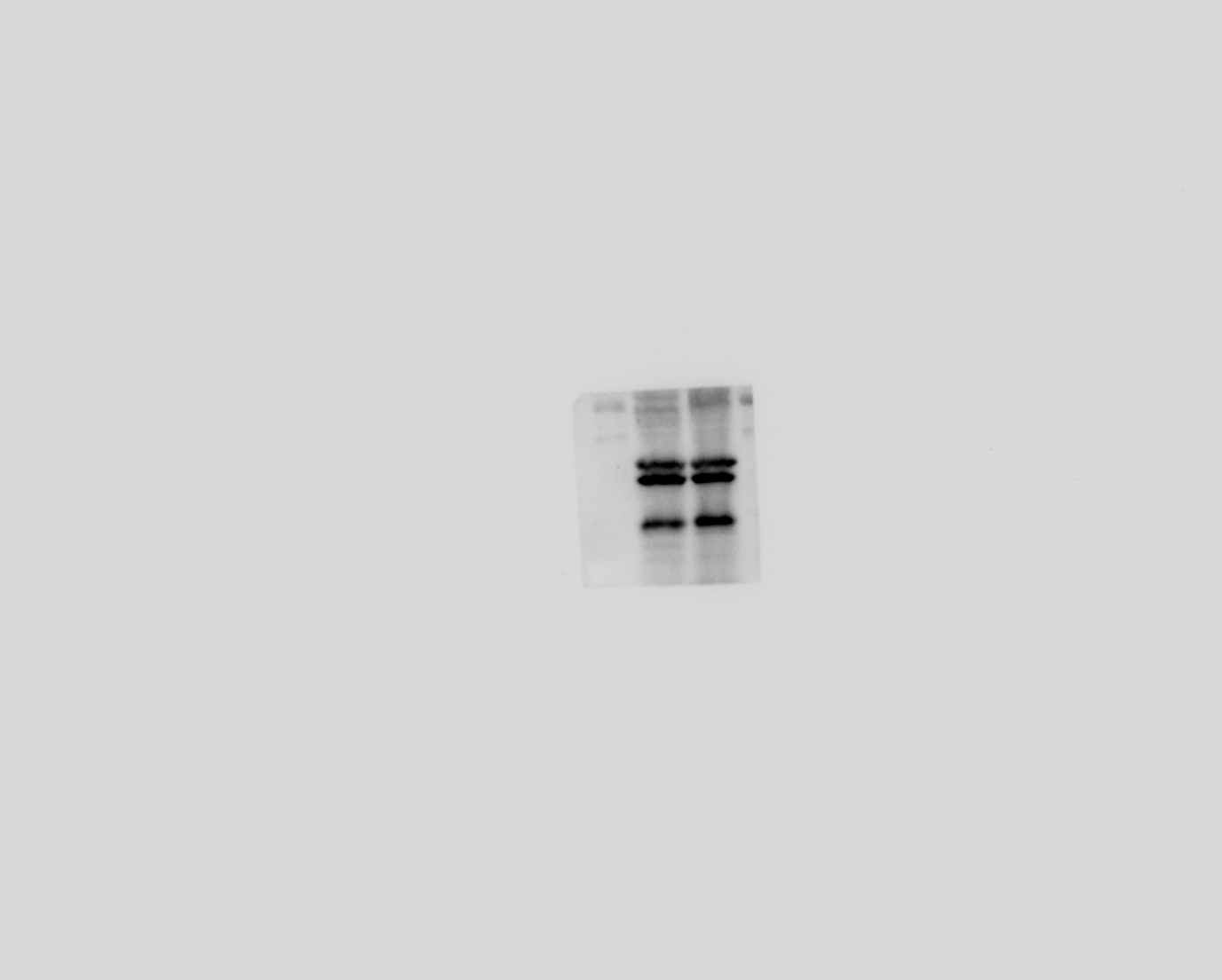

Supplement: Supplementary file 12 — Source data Fig. 7 [file 44318_2024_359_MOESM12_ESM.zip › Figure 7/Fig 7F/2-p27.Tif]

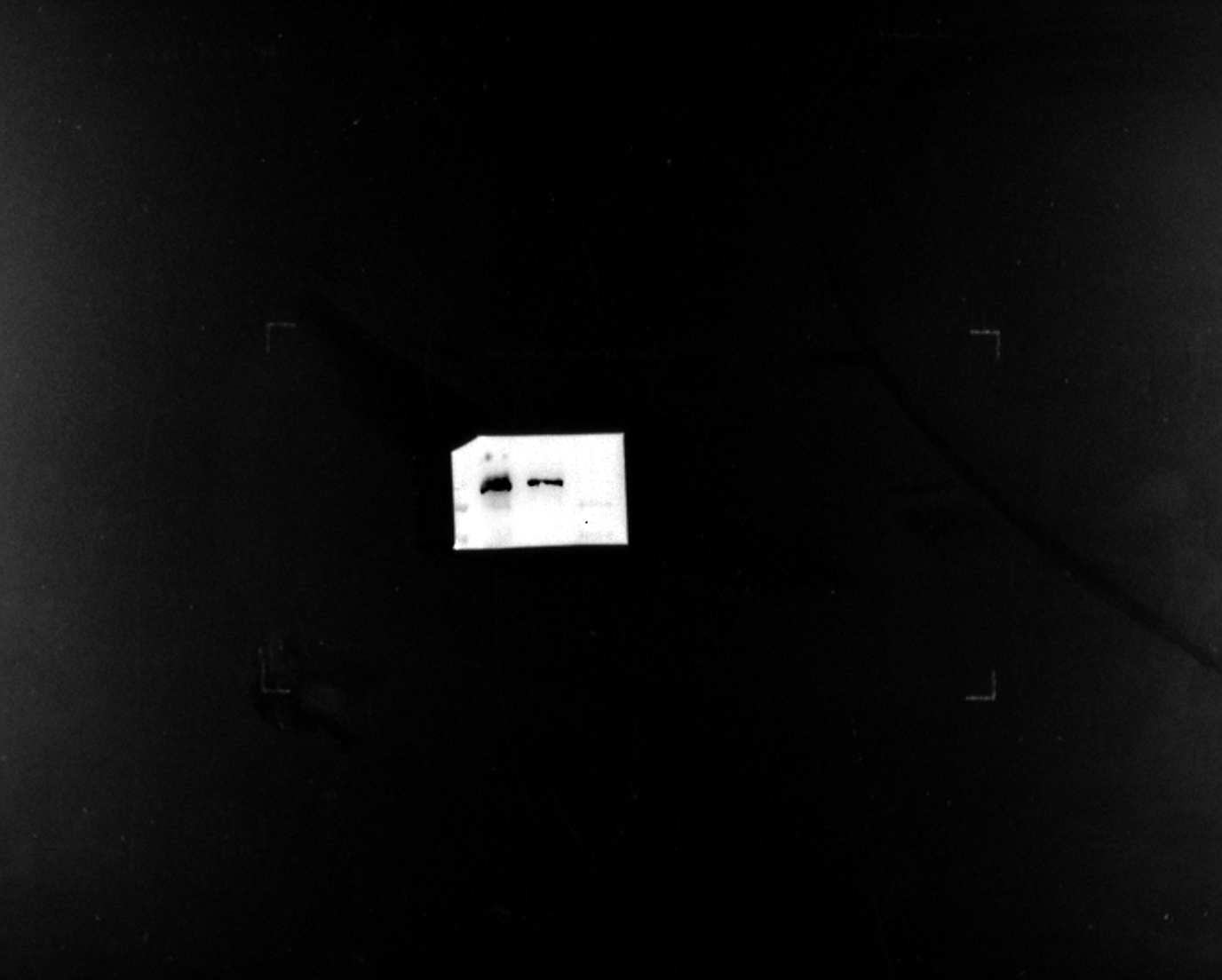

Supplement: Supplementary file 12 — Source data Fig. 7 [file 44318_2024_359_MOESM12_ESM.zip › Figure 7/Fig 7F/3-p-mTOR-merge.Tif]

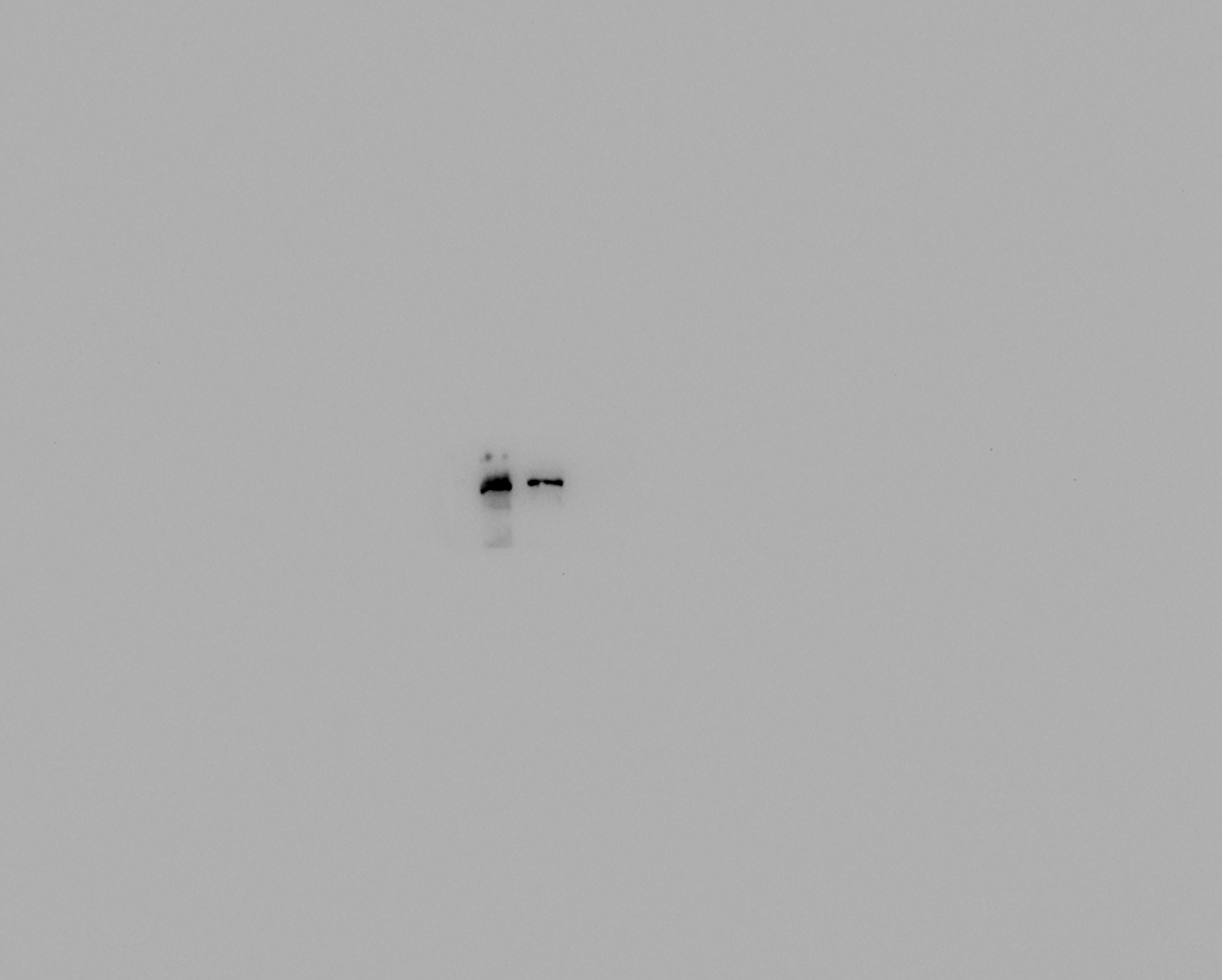

Supplement: Supplementary file 12 — Source data Fig. 7 [file 44318_2024_359_MOESM12_ESM.zip › Figure 7/Fig 7F/3-p-mTOR.Tif]

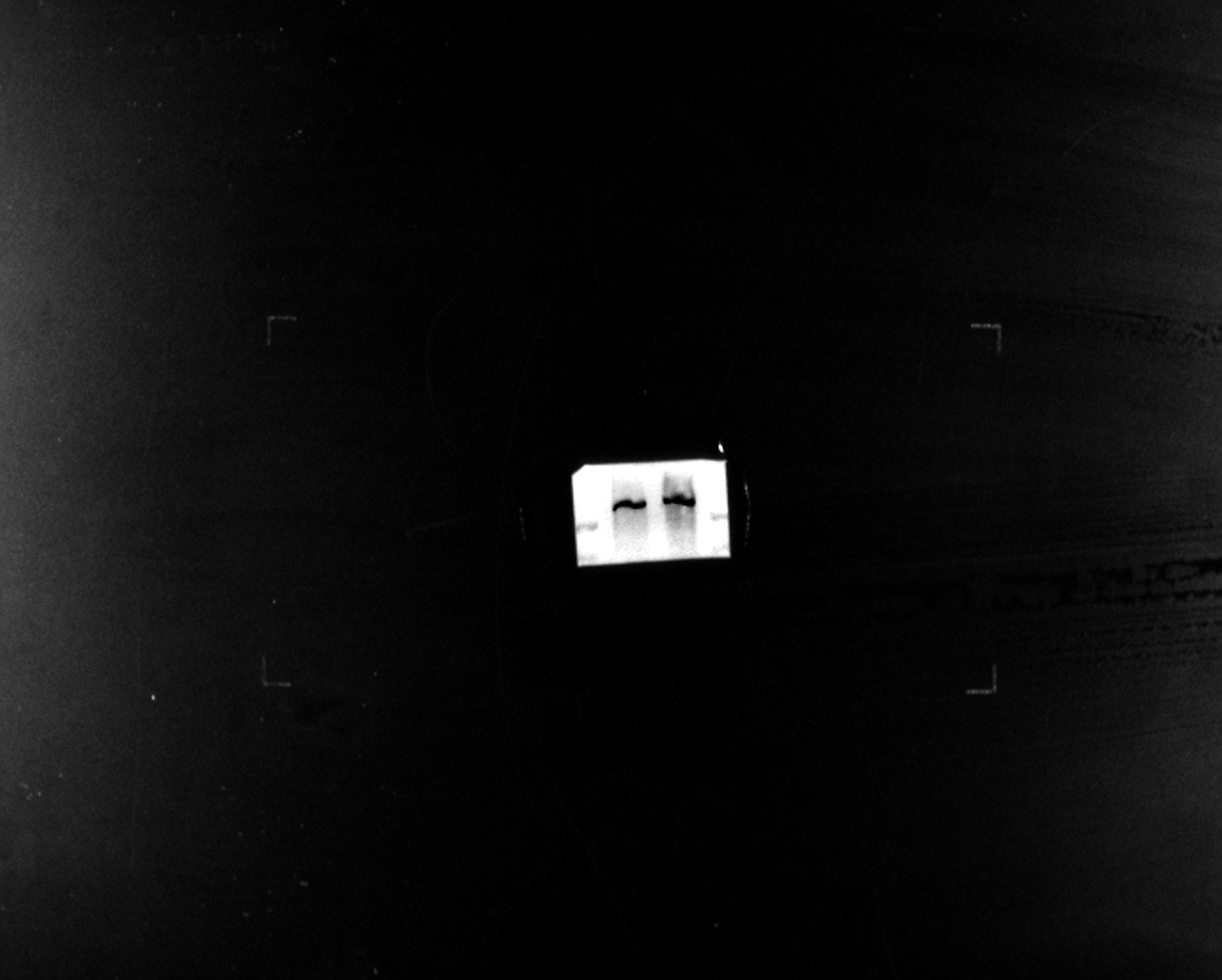

Supplement: Supplementary file 12 — Source data Fig. 7 [file 44318_2024_359_MOESM12_ESM.zip › Figure 7/Fig 7F/4-mTOR-merge.Tif]

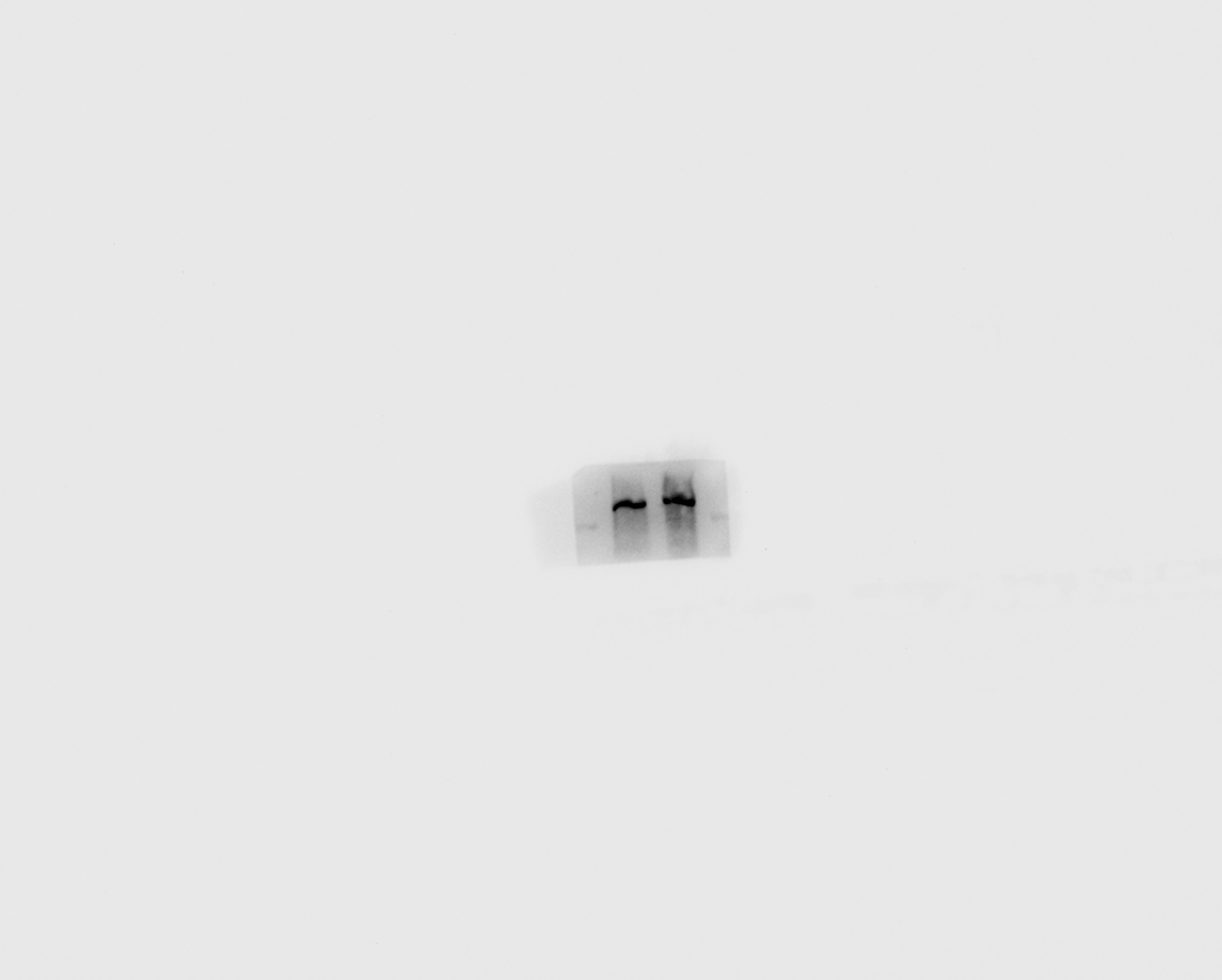

Supplement: Supplementary file 12 — Source data Fig. 7 [file 44318_2024_359_MOESM12_ESM.zip › Figure 7/Fig 7F/4-mTOR.Tif]

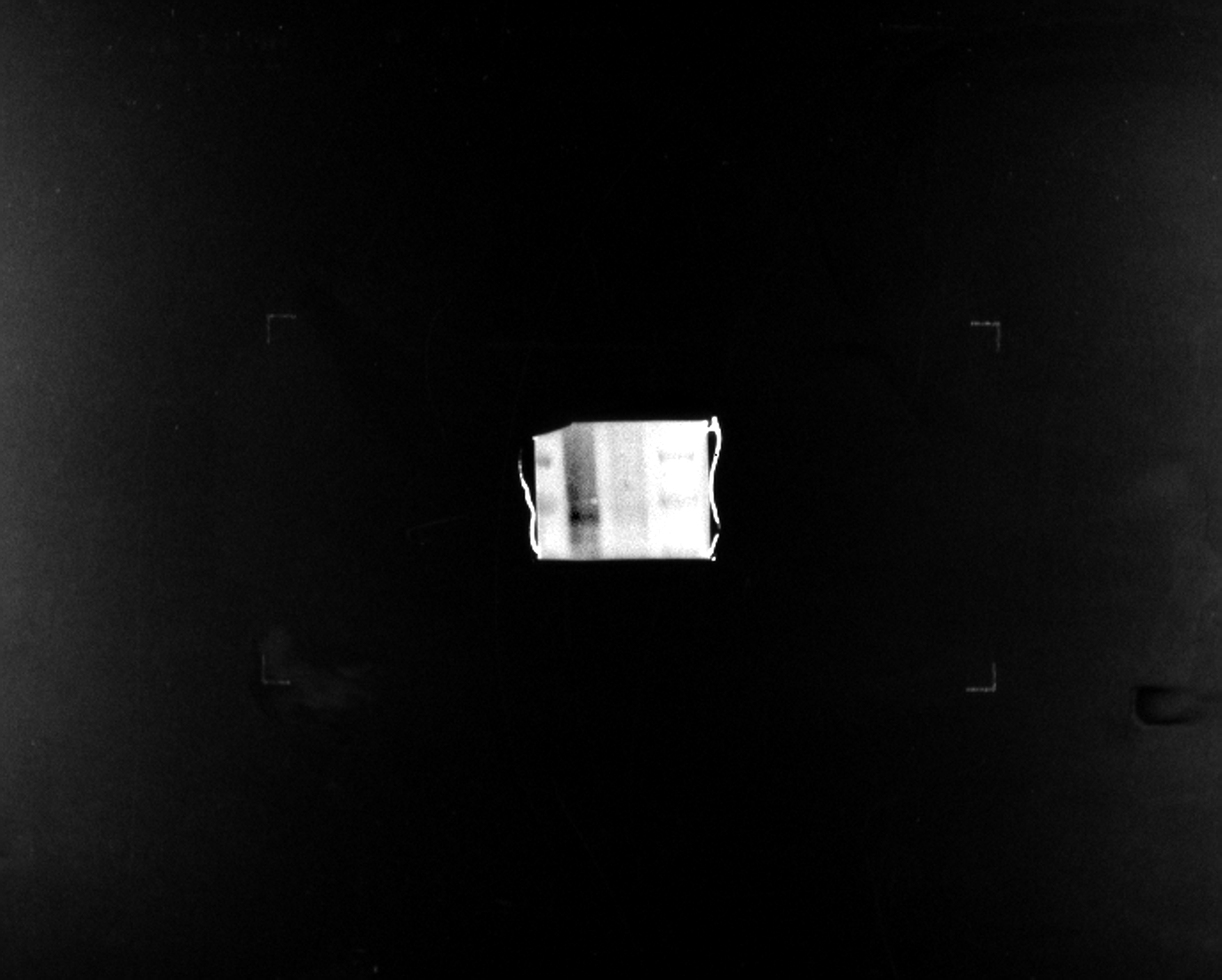

Supplement: Supplementary file 12 — Source data Fig. 7 [file 44318_2024_359_MOESM12_ESM.zip › Figure 7/Fig 7F/5-p-S6K-merge.Tif]

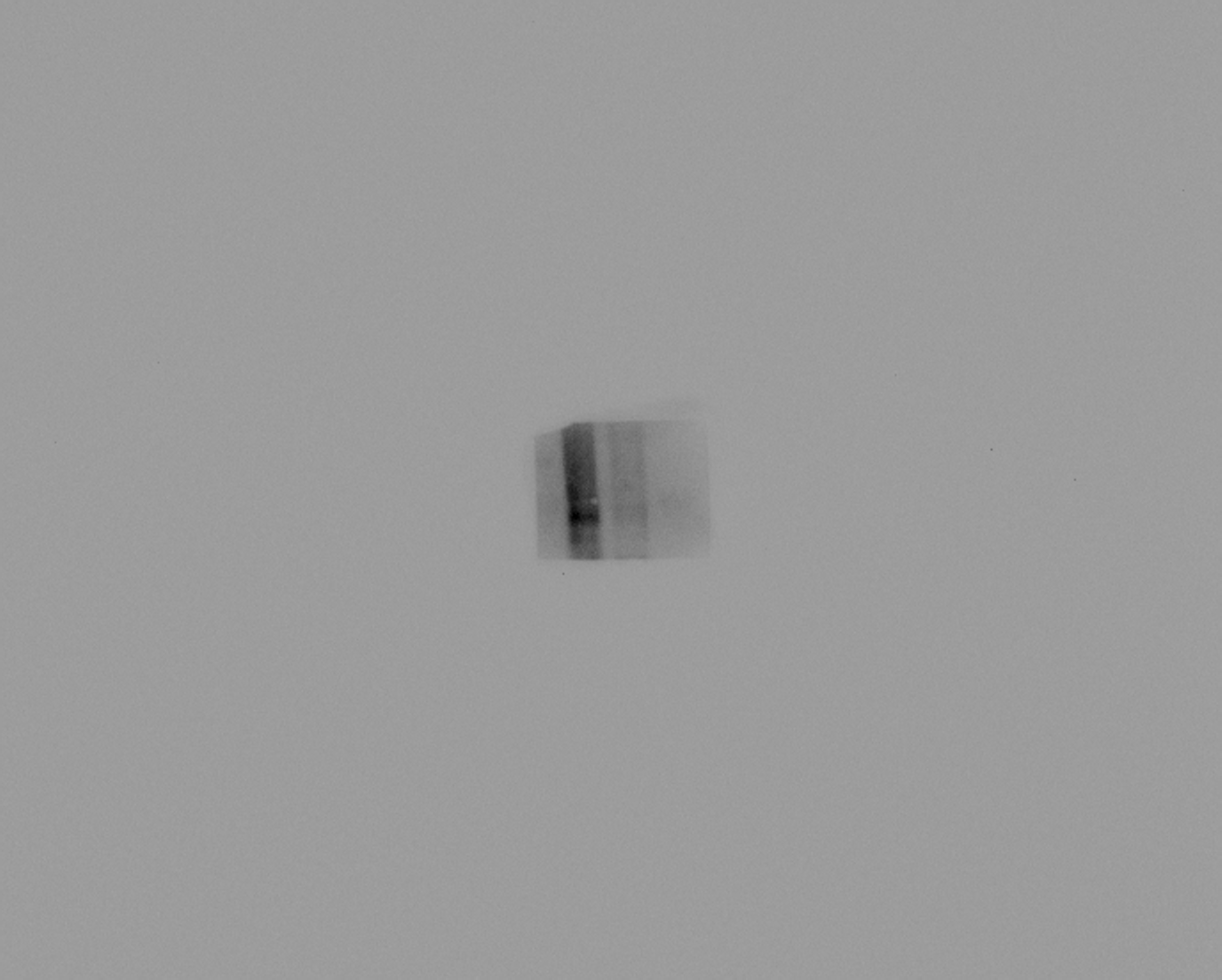

Supplement: Supplementary file 12 — Source data Fig. 7 [file 44318_2024_359_MOESM12_ESM.zip › Figure 7/Fig 7F/5-p-S6K.Tif]

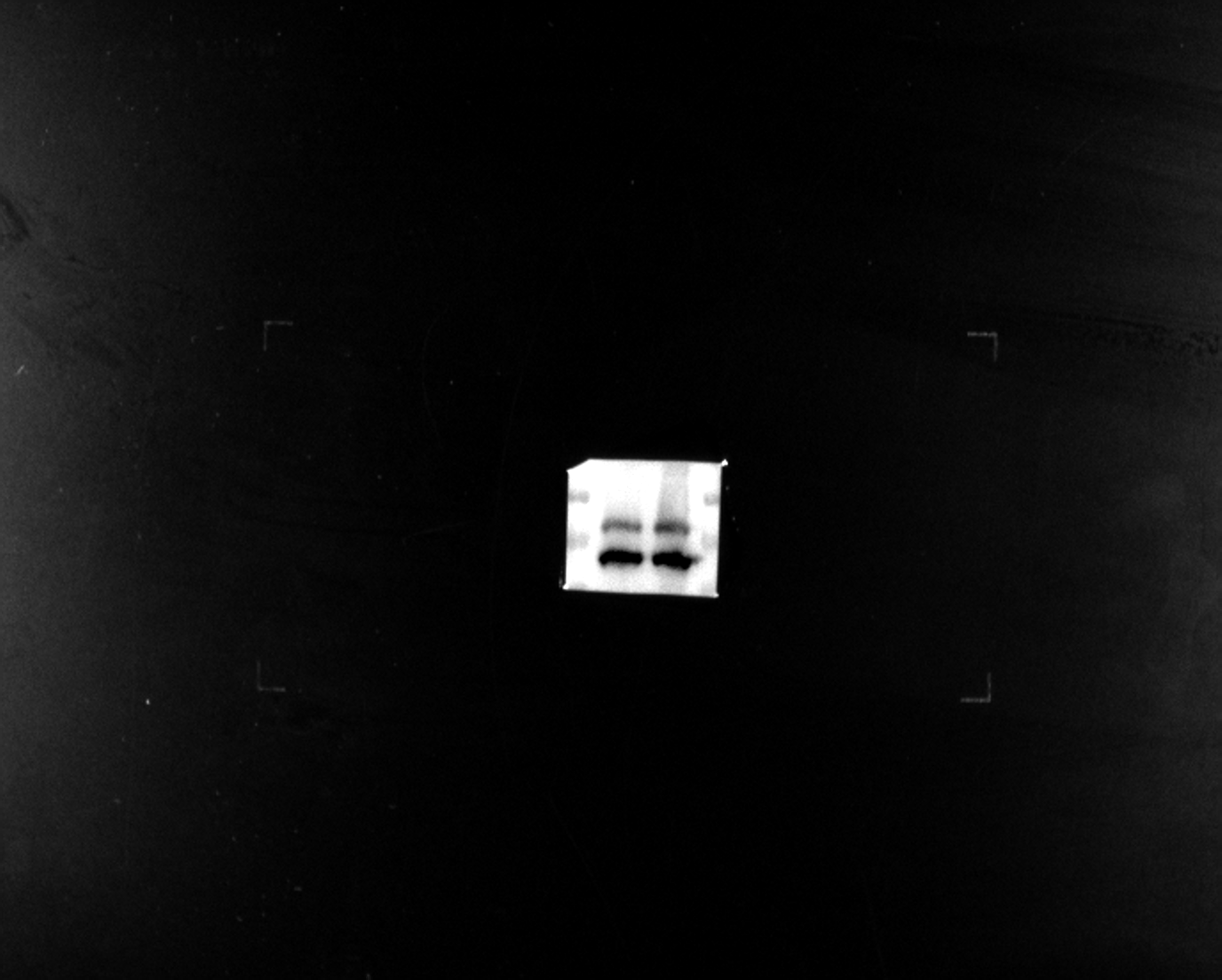

Supplement: Supplementary file 12 — Source data Fig. 7 [file 44318_2024_359_MOESM12_ESM.zip › Figure 7/Fig 7F/6-S6K-merge.Tif]

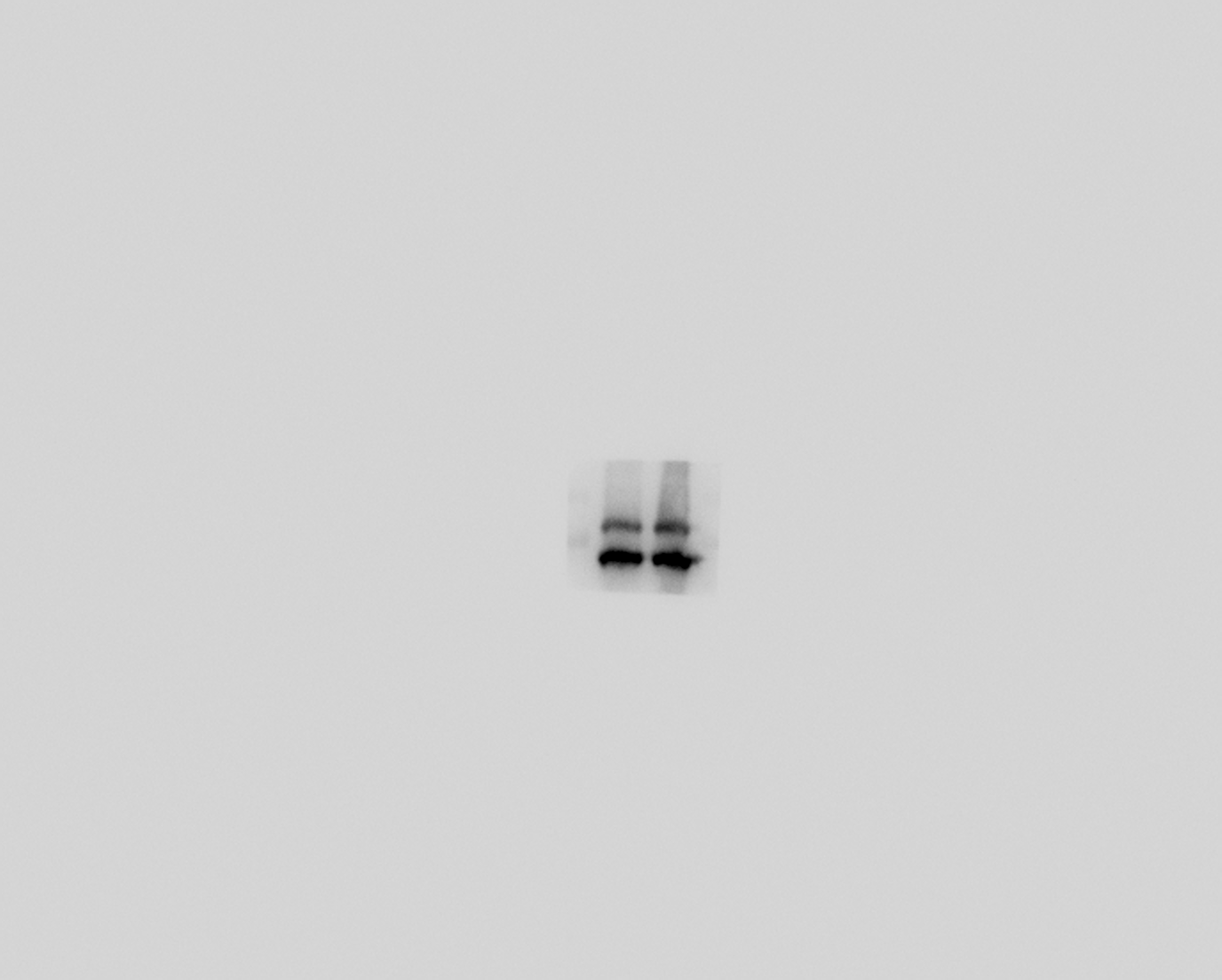

Supplement: Supplementary file 12 — Source data Fig. 7 [file 44318_2024_359_MOESM12_ESM.zip › Figure 7/Fig 7F/6-S6K.Tif]

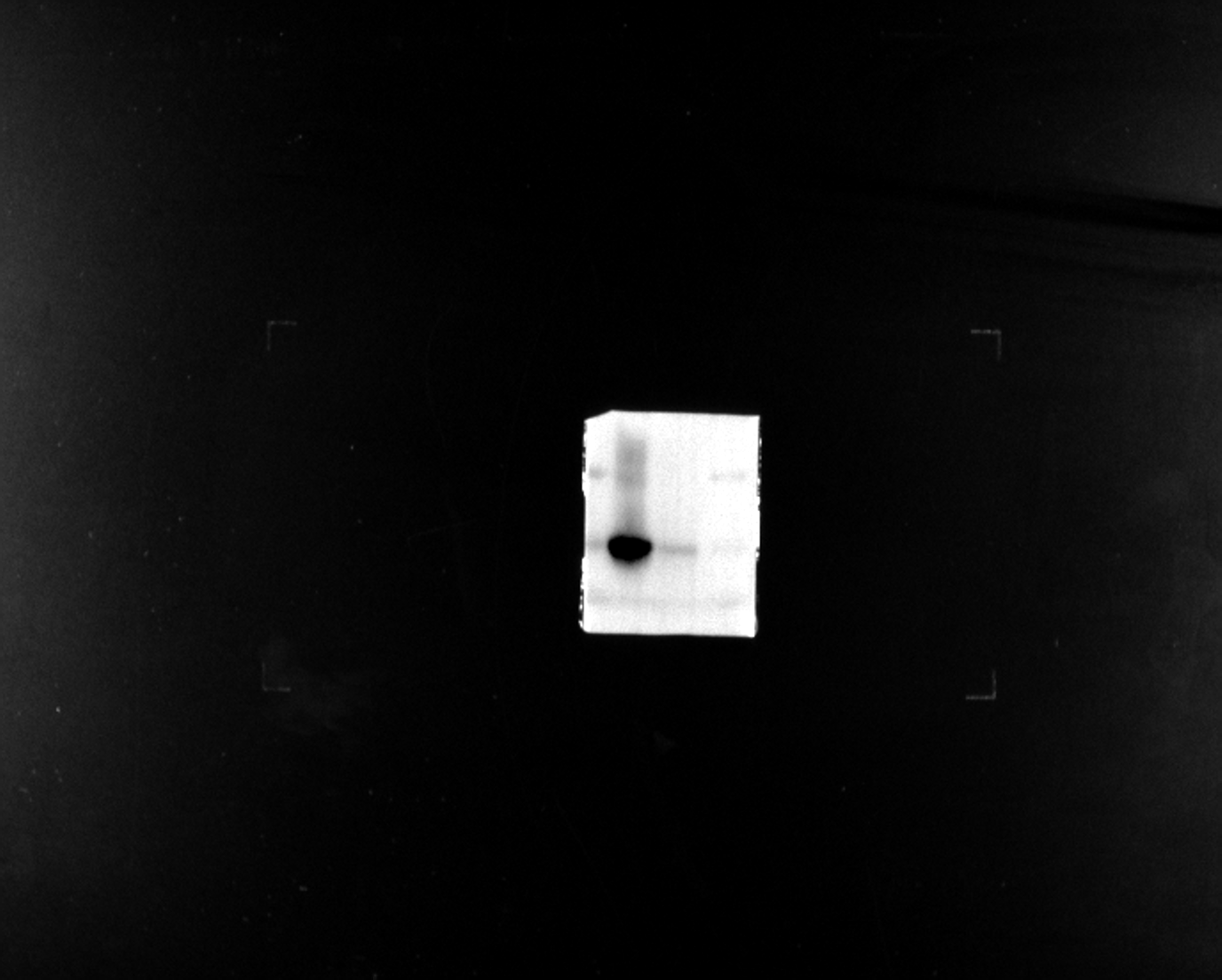

Supplement: Supplementary file 12 — Source data Fig. 7 [file 44318_2024_359_MOESM12_ESM.zip › Figure 7/Fig 7F/7-p-S6-merge.Tif]

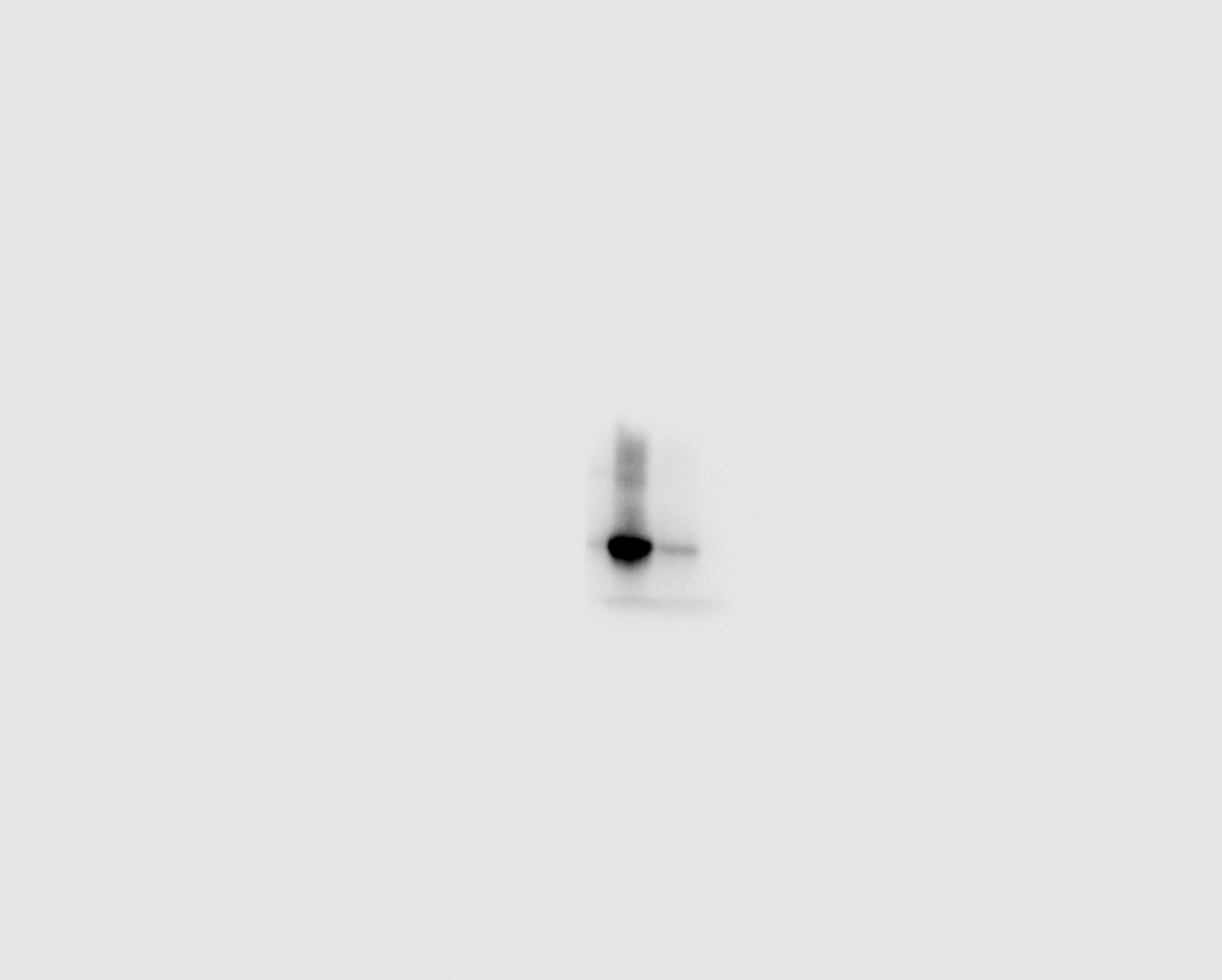

Supplement: Supplementary file 12 — Source data Fig. 7 [file 44318_2024_359_MOESM12_ESM.zip › Figure 7/Fig 7F/7-p-S6.Tif]

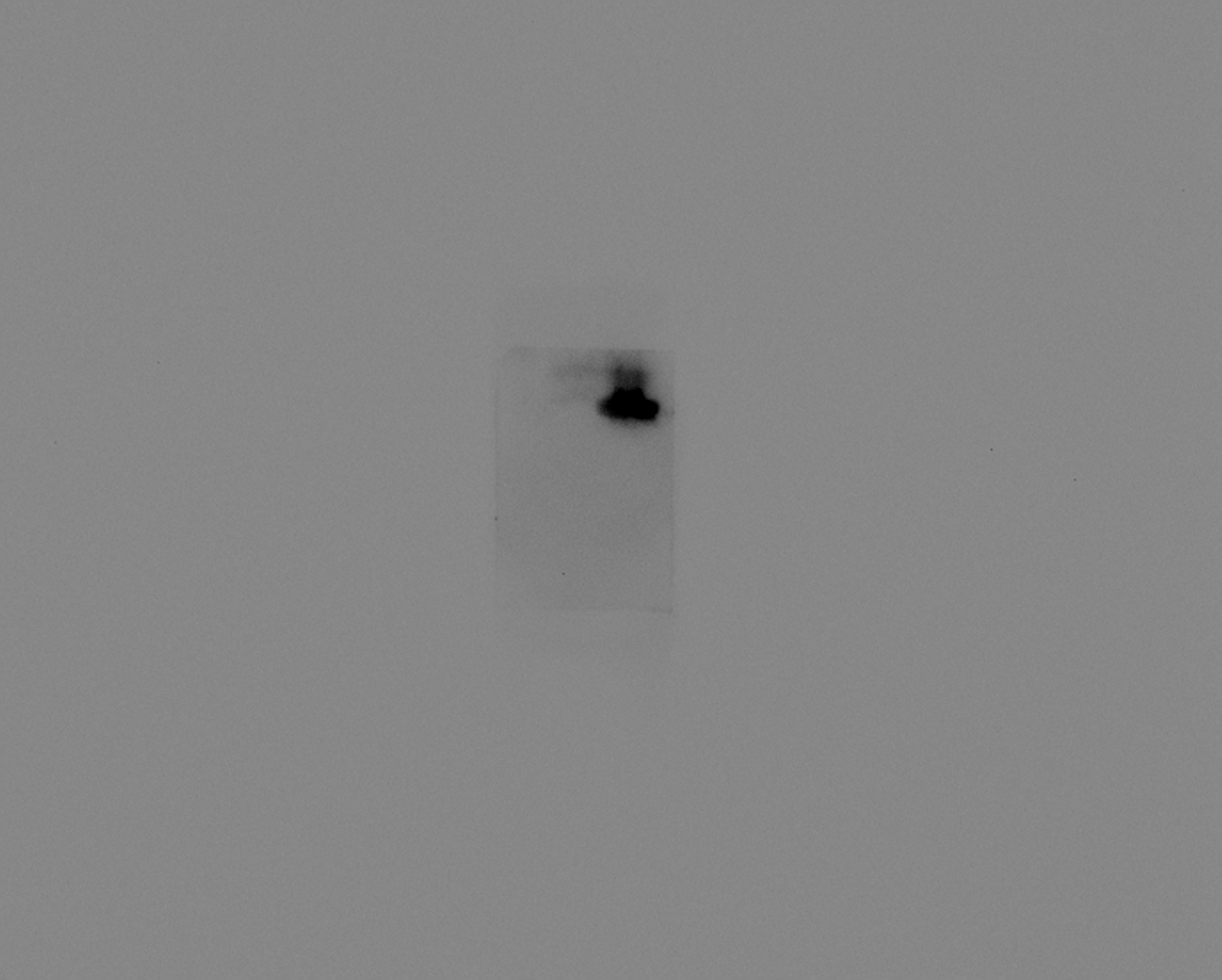

Supplement: Supplementary file 12 — Source data Fig. 7 [file 44318_2024_359_MOESM12_ESM.zip › Figure 7/Fig 7F/8-TAT.Tif]

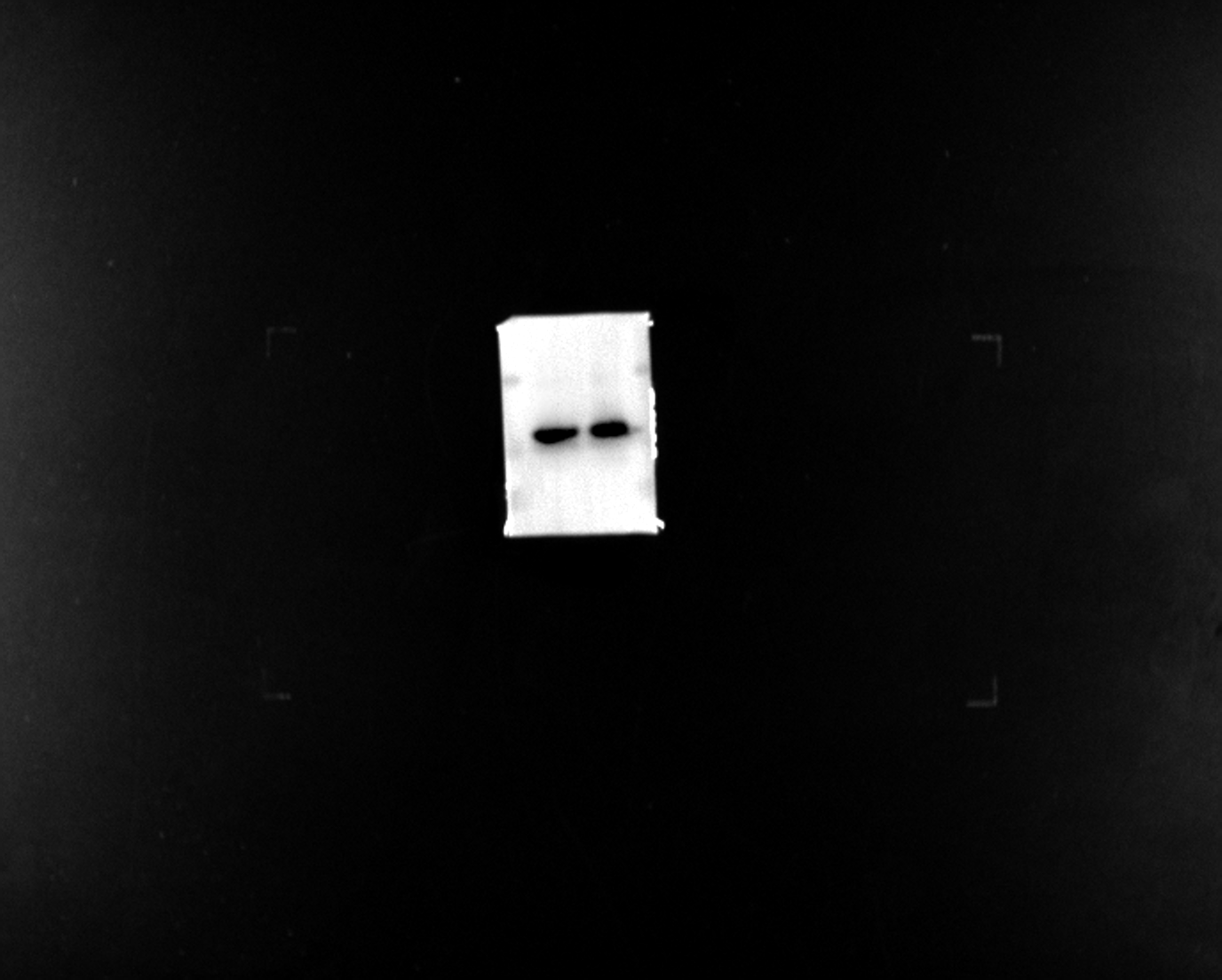

Supplement: Supplementary file 12 — Source data Fig. 7 [file 44318_2024_359_MOESM12_ESM.zip › Figure 7/Fig 7F/9-GAPDH-merge.Tif]

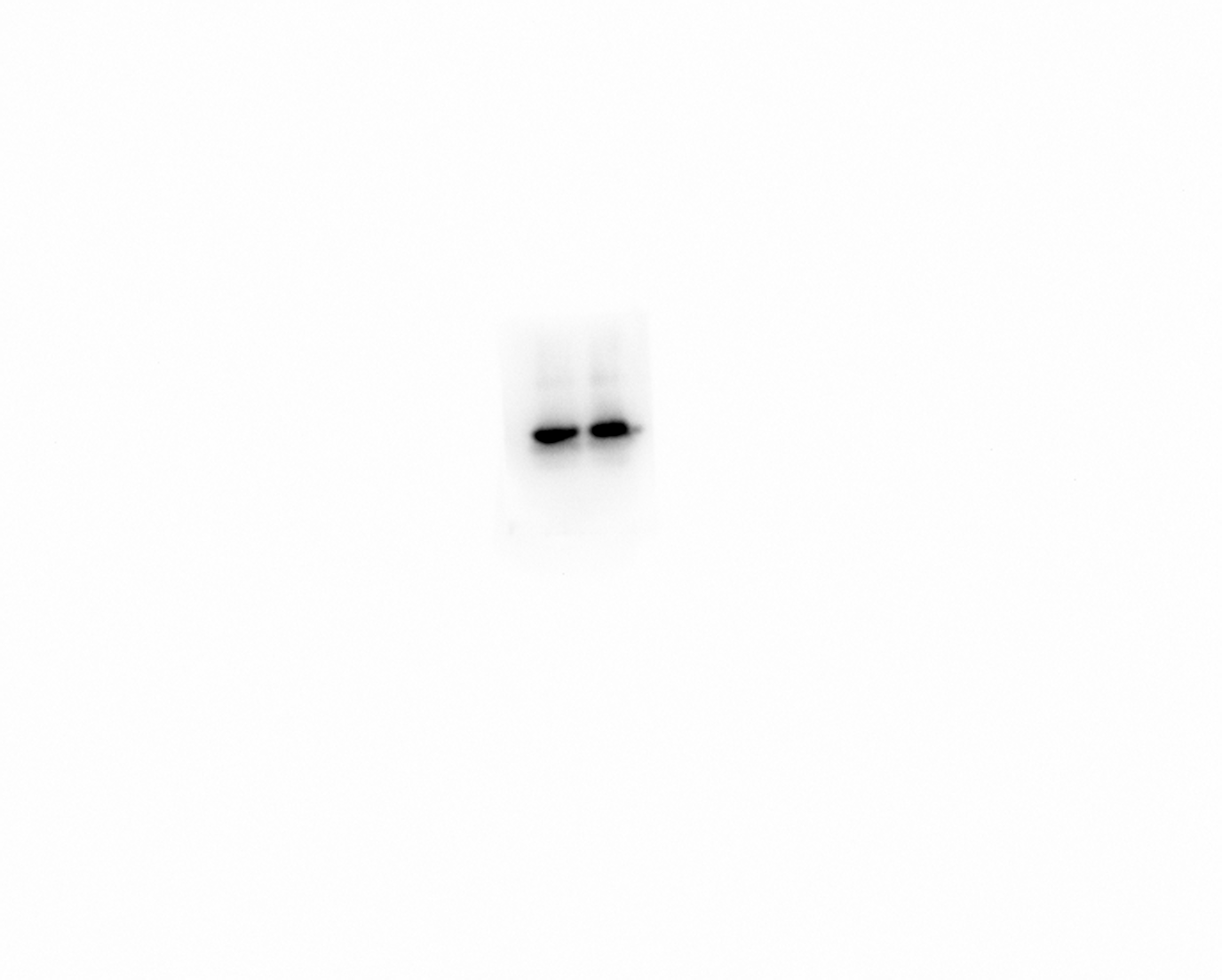

Supplement: Supplementary file 12 — Source data Fig. 7 [file 44318_2024_359_MOESM12_ESM.zip › Figure 7/Fig 7F/9-GAPDH.Tif]

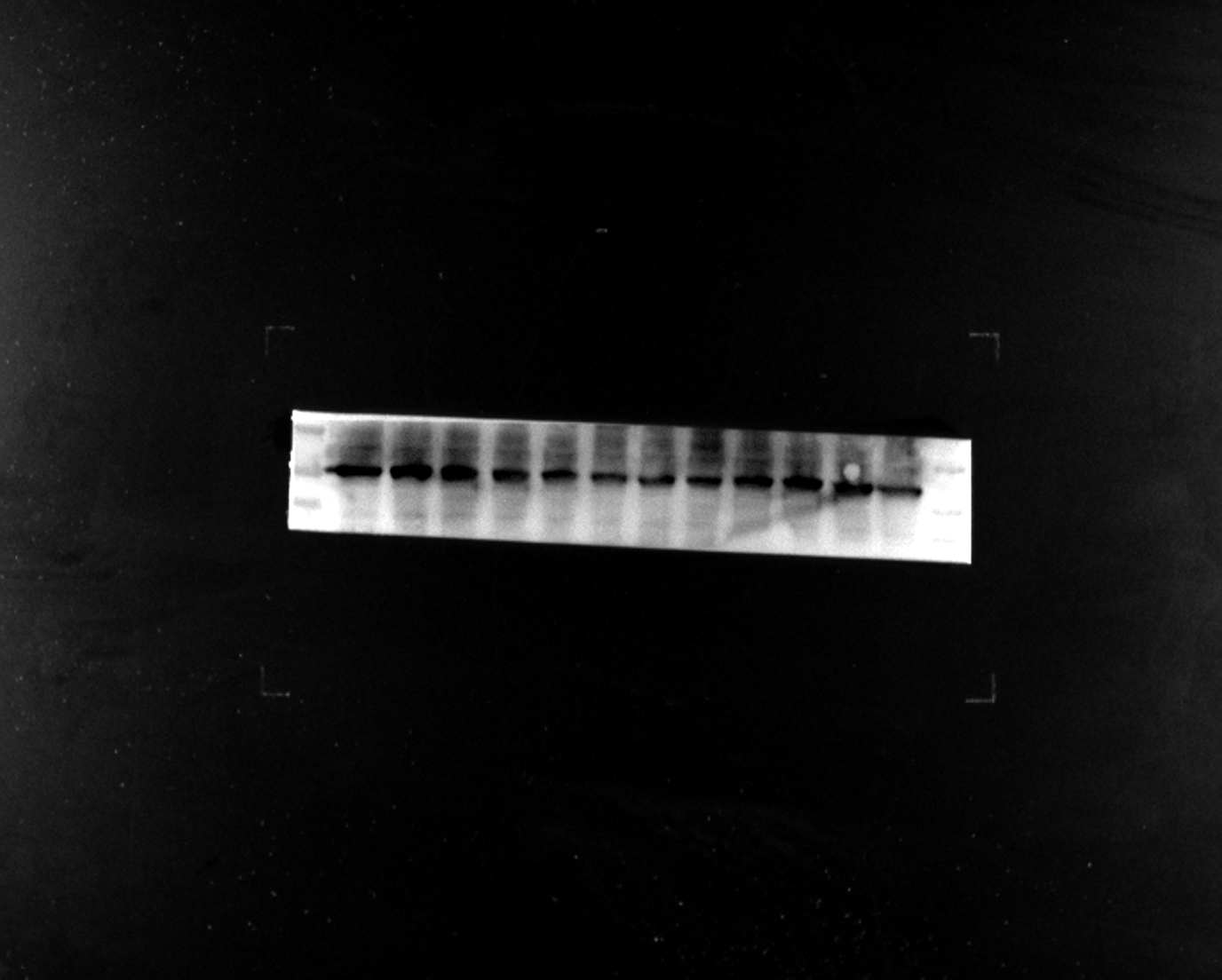

Supplement: Supplementary file 12 — Source data Fig. 7 [file 44318_2024_359_MOESM12_ESM.zip › Figure 7/Fig 7O/8-S6-merge.Tif]

Fig 7F

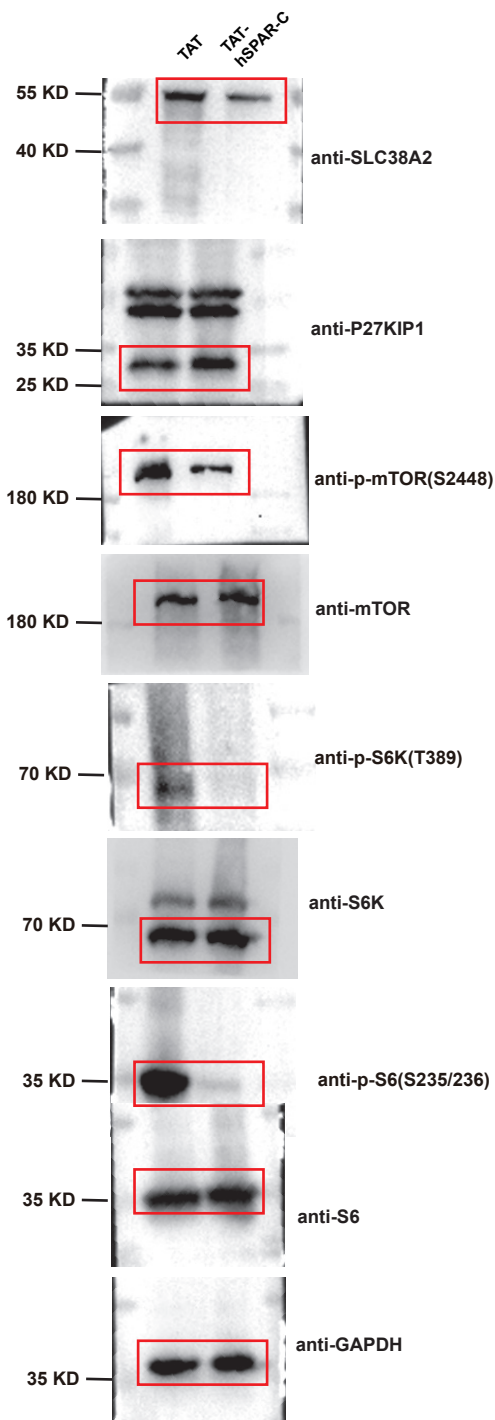

Supplement: Supplementary file 12 — Source data Fig. 7 [file 44318_2024_359_MOESM12_ESM.zip › Figure 7/Fig 7F/Fig. 7F.pdf]

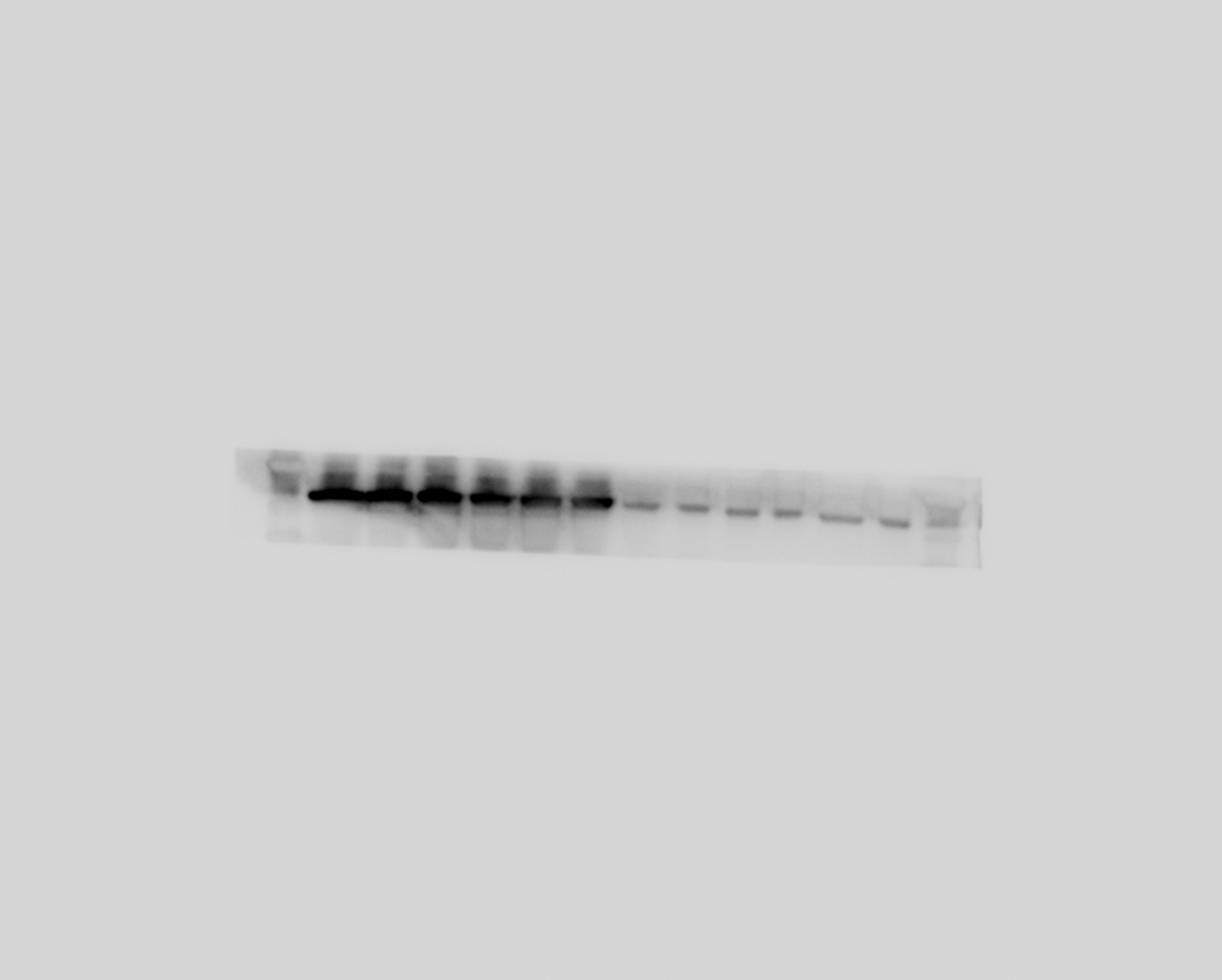

Supplement: Supplementary file 12 — Source data Fig. 7 [file 44318_2024_359_MOESM12_ESM.zip › Figure 7/Fig 7O/7-p-S6.Tif]

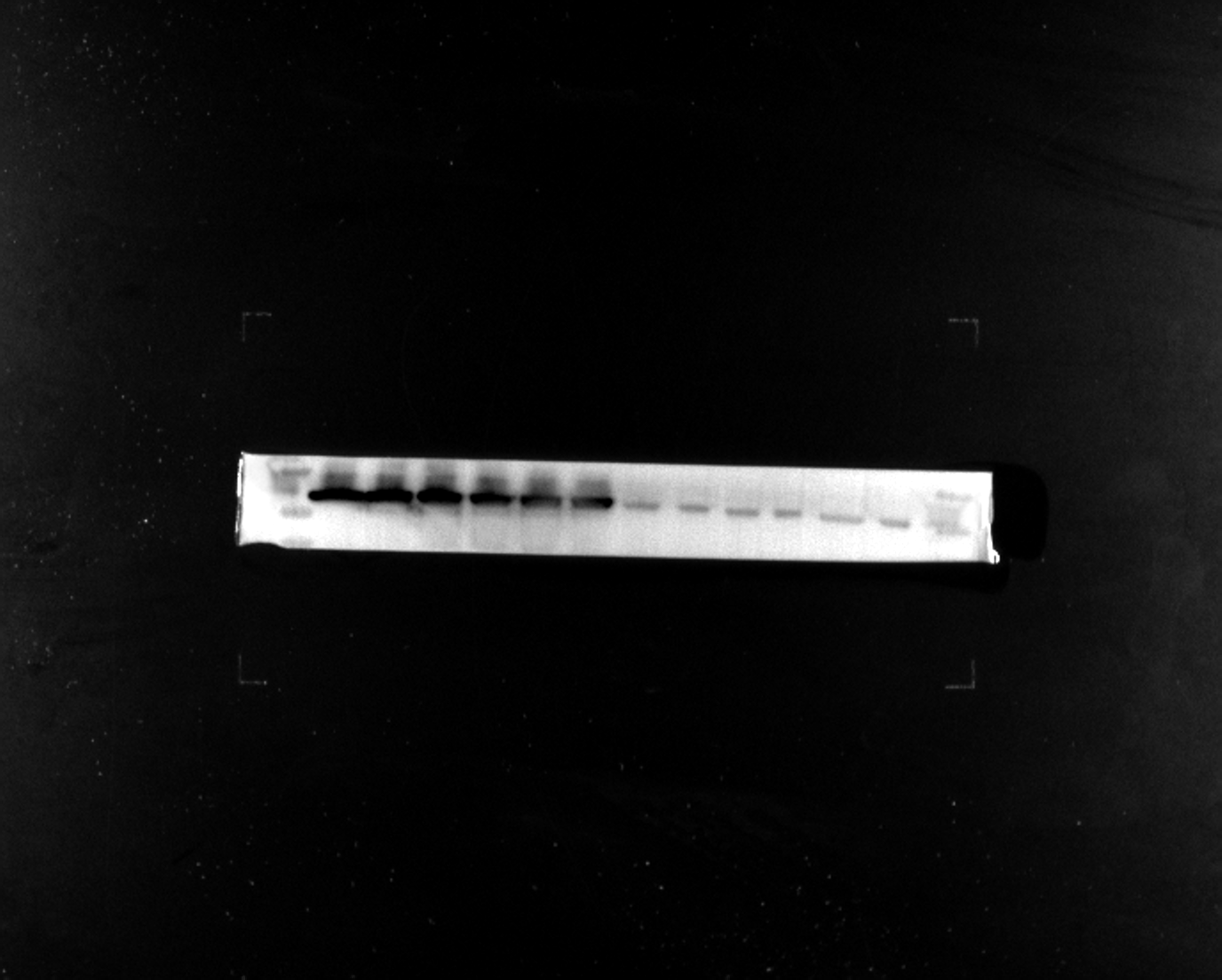

Supplement: Supplementary file 12 — Source data Fig. 7 [file 44318_2024_359_MOESM12_ESM.zip › Figure 7/Fig 7O/7-p-S6-merge.Tif]

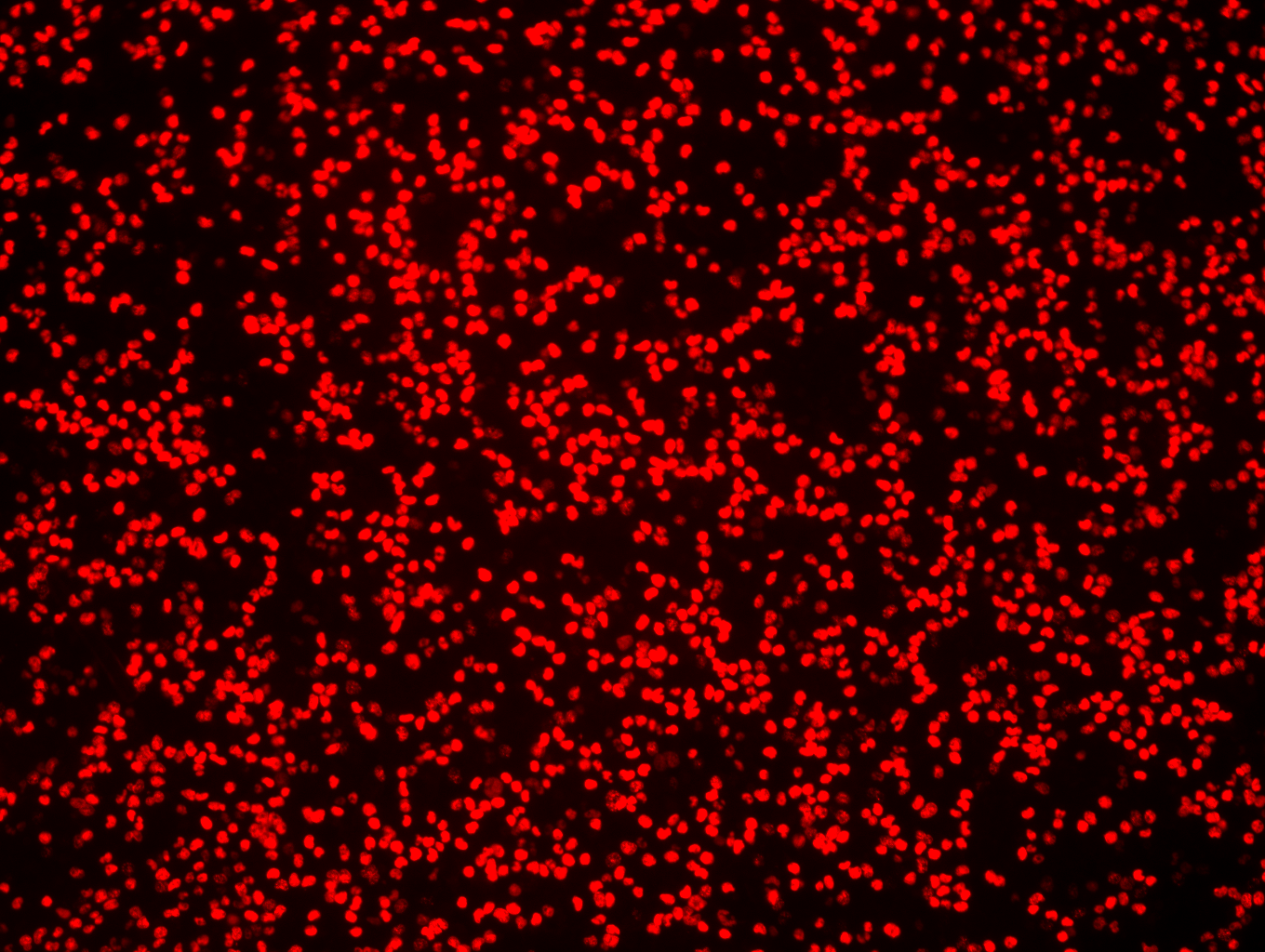

Supplement: Supplementary file 12 — Source data Fig. 7 [file 44318_2024_359_MOESM12_ESM.zip › Figure 7/Fig 7G/ctrl vector/Edu.tif]

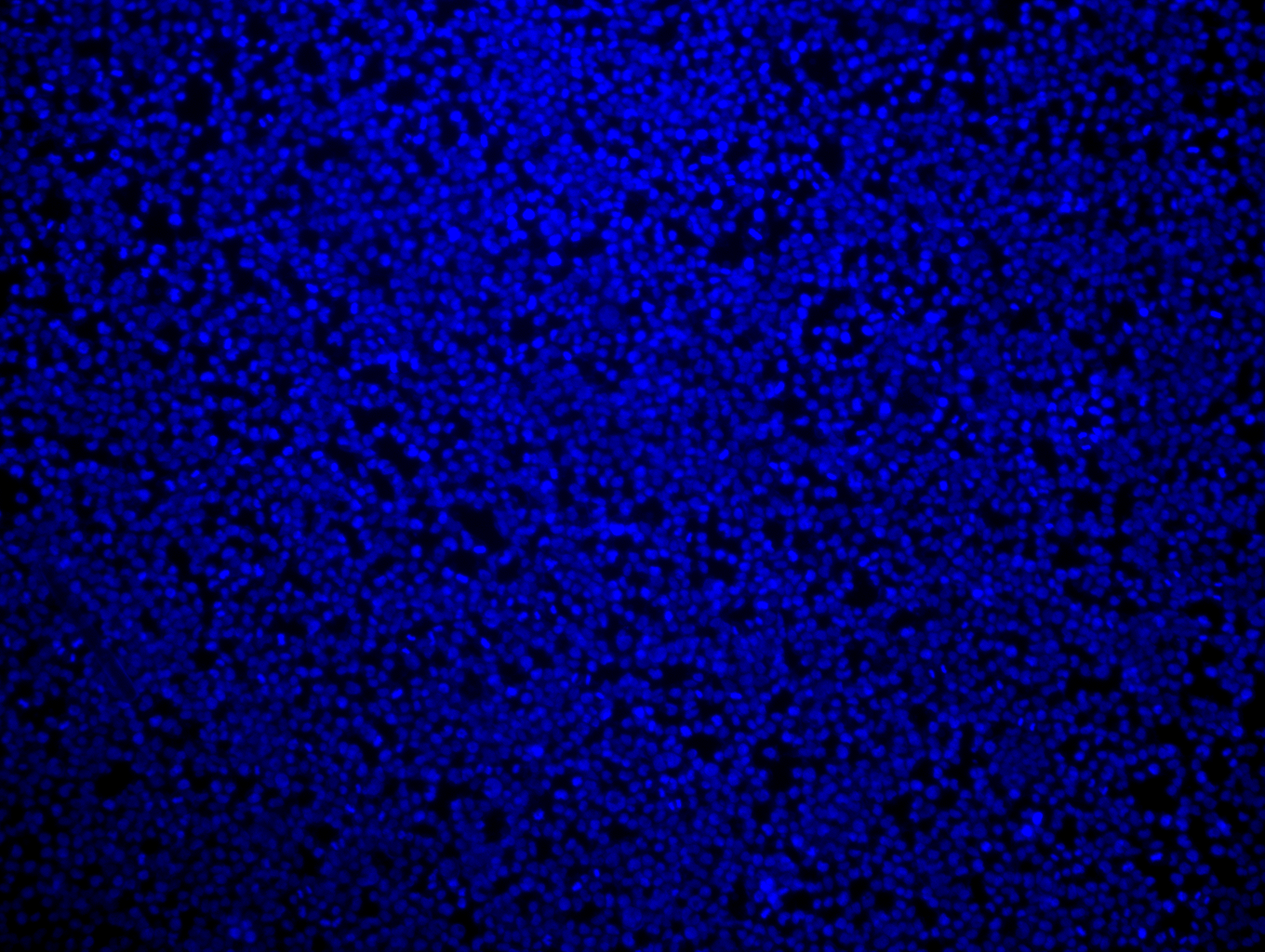

Supplement: Supplementary file 12 — Source data Fig. 7 [file 44318_2024_359_MOESM12_ESM.zip › Figure 7/Fig 7G/ctrl vector/Hoechst.tif]

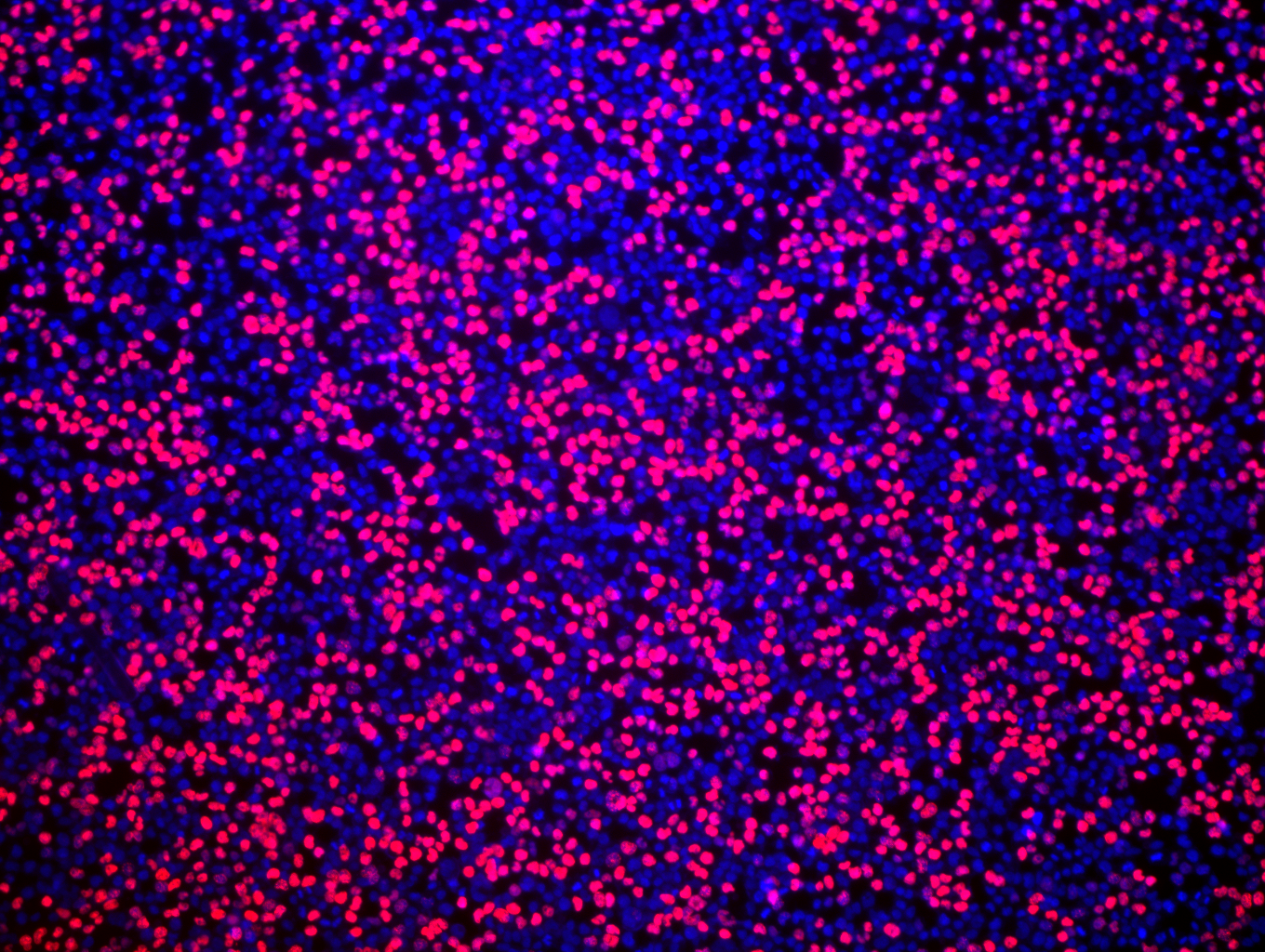

Supplement: Supplementary file 12 — Source data Fig. 7 [file 44318_2024_359_MOESM12_ESM.zip › Figure 7/Fig 7G/ctrl vector/merge.tif]

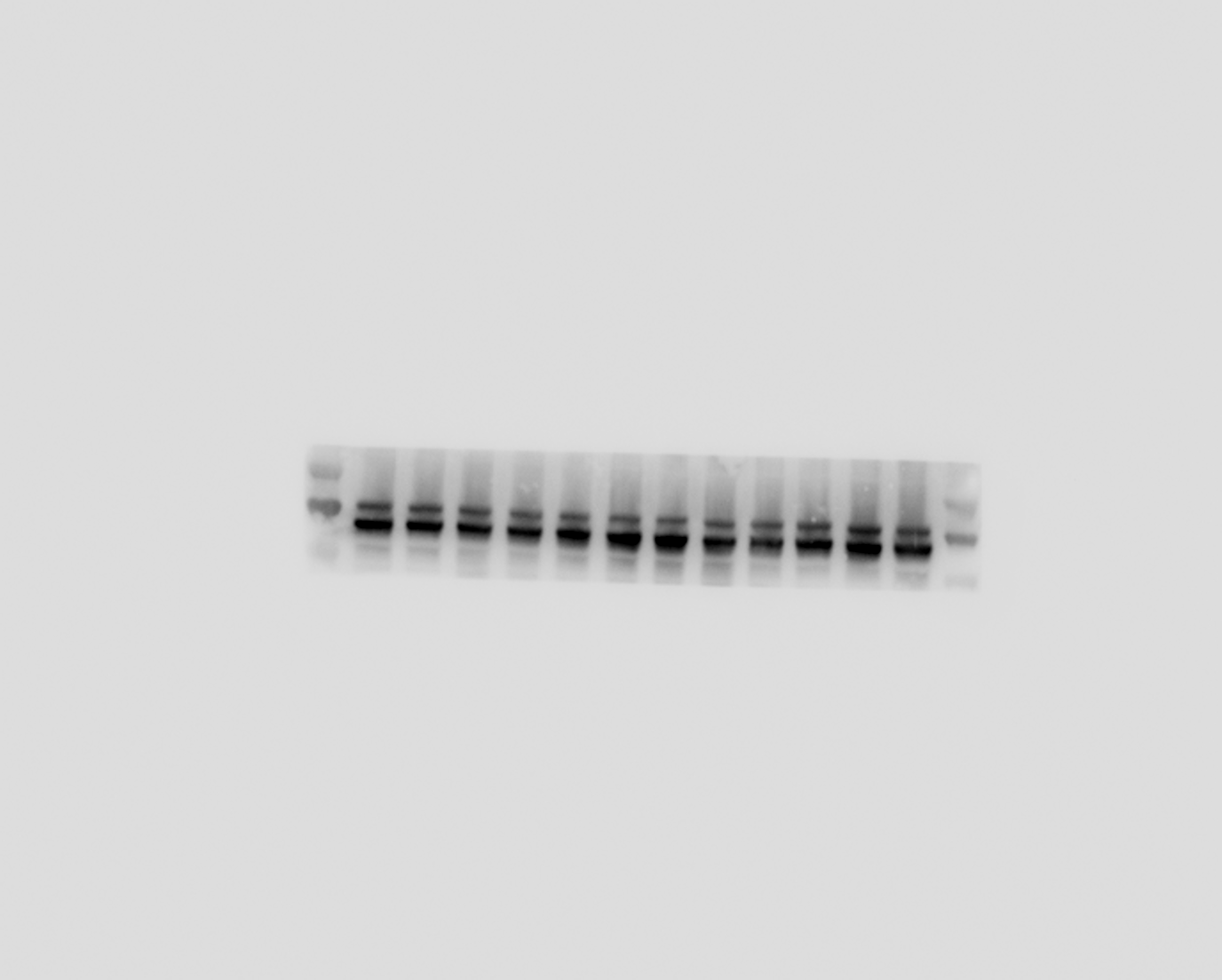

Supplement: Supplementary file 12 — Source data Fig. 7 [file 44318_2024_359_MOESM12_ESM.zip › Figure 7/Fig 7O/6-S6K.Tif]

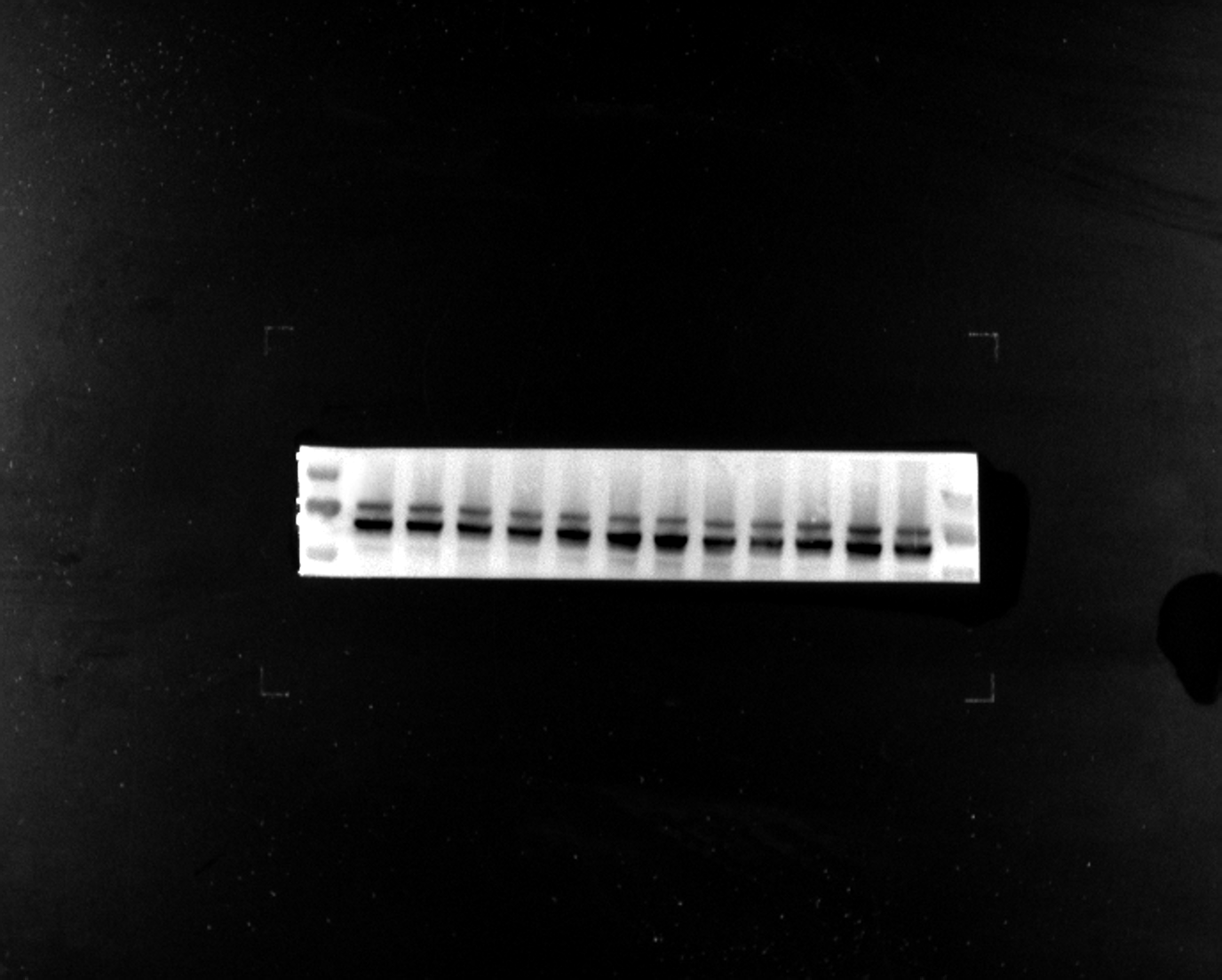

Supplement: Supplementary file 12 — Source data Fig. 7 [file 44318_2024_359_MOESM12_ESM.zip › Figure 7/Fig 7O/6-S6K-merge.Tif]

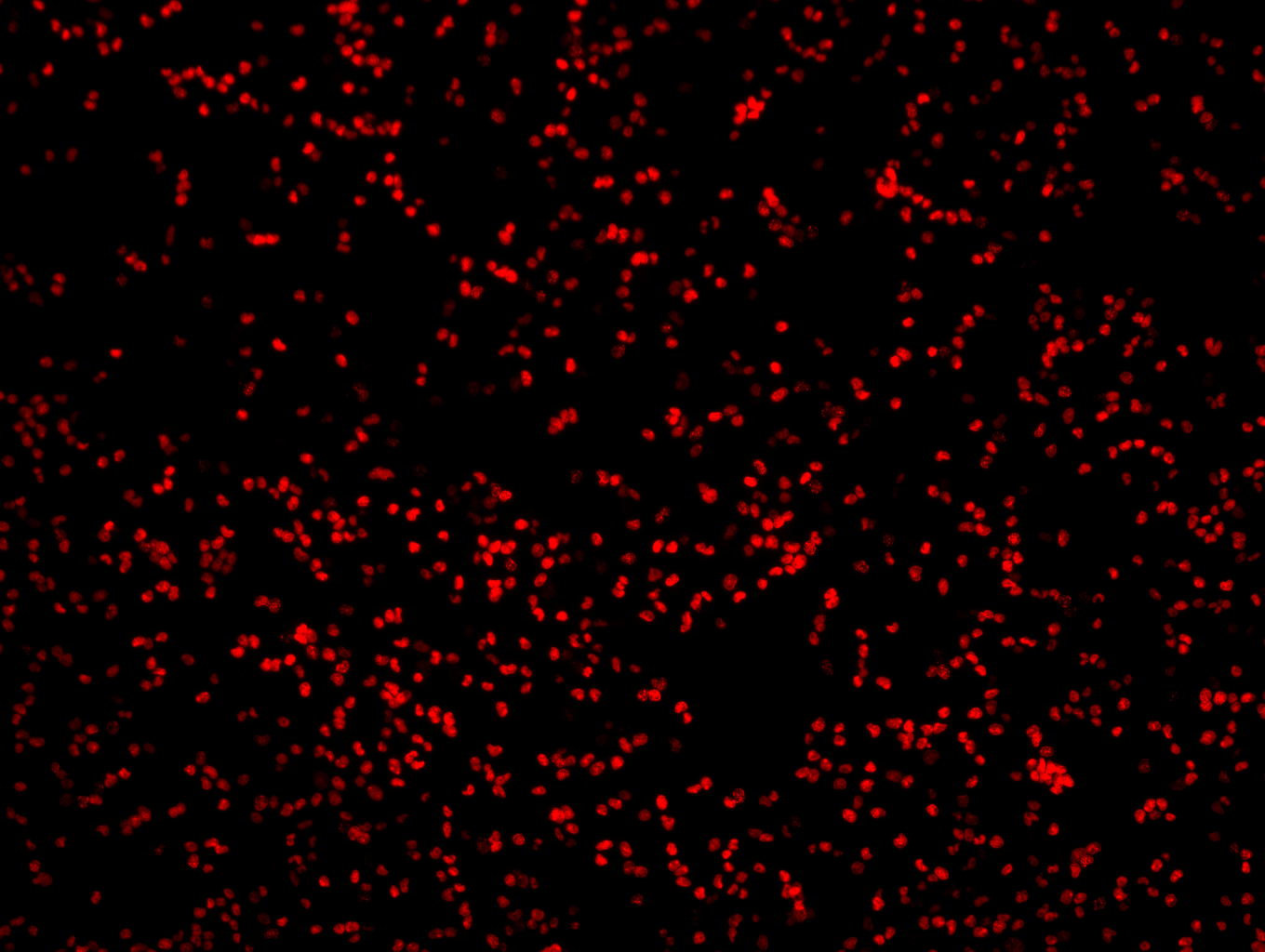

Supplement: Supplementary file 12 — Source data Fig. 7 [file 44318_2024_359_MOESM12_ESM.zip › Figure 7/Fig 7G/hSPAR/Edu.tif]

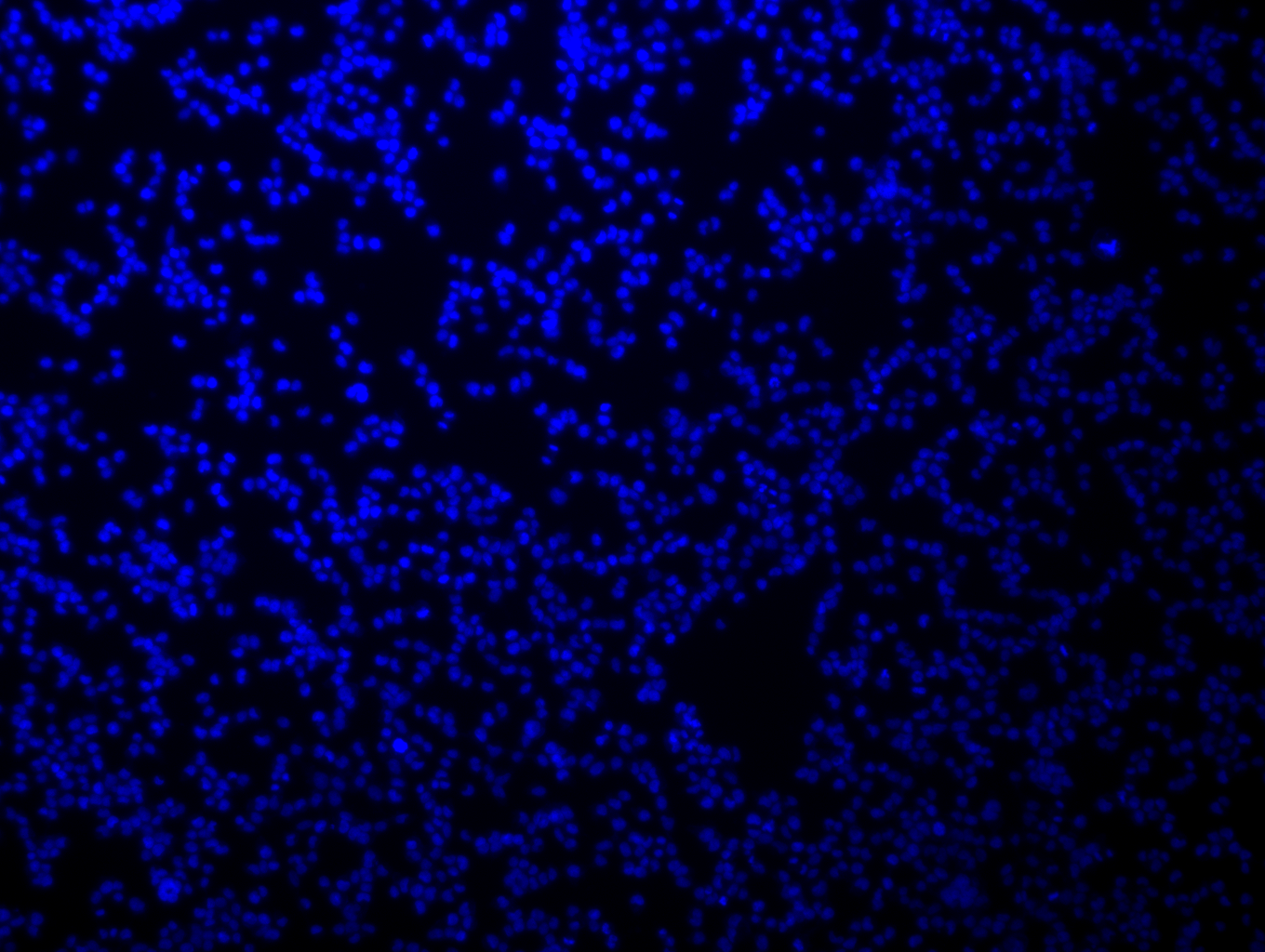

Supplement: Supplementary file 12 — Source data Fig. 7 [file 44318_2024_359_MOESM12_ESM.zip › Figure 7/Fig 7G/hSPAR/Hoechst.tif]

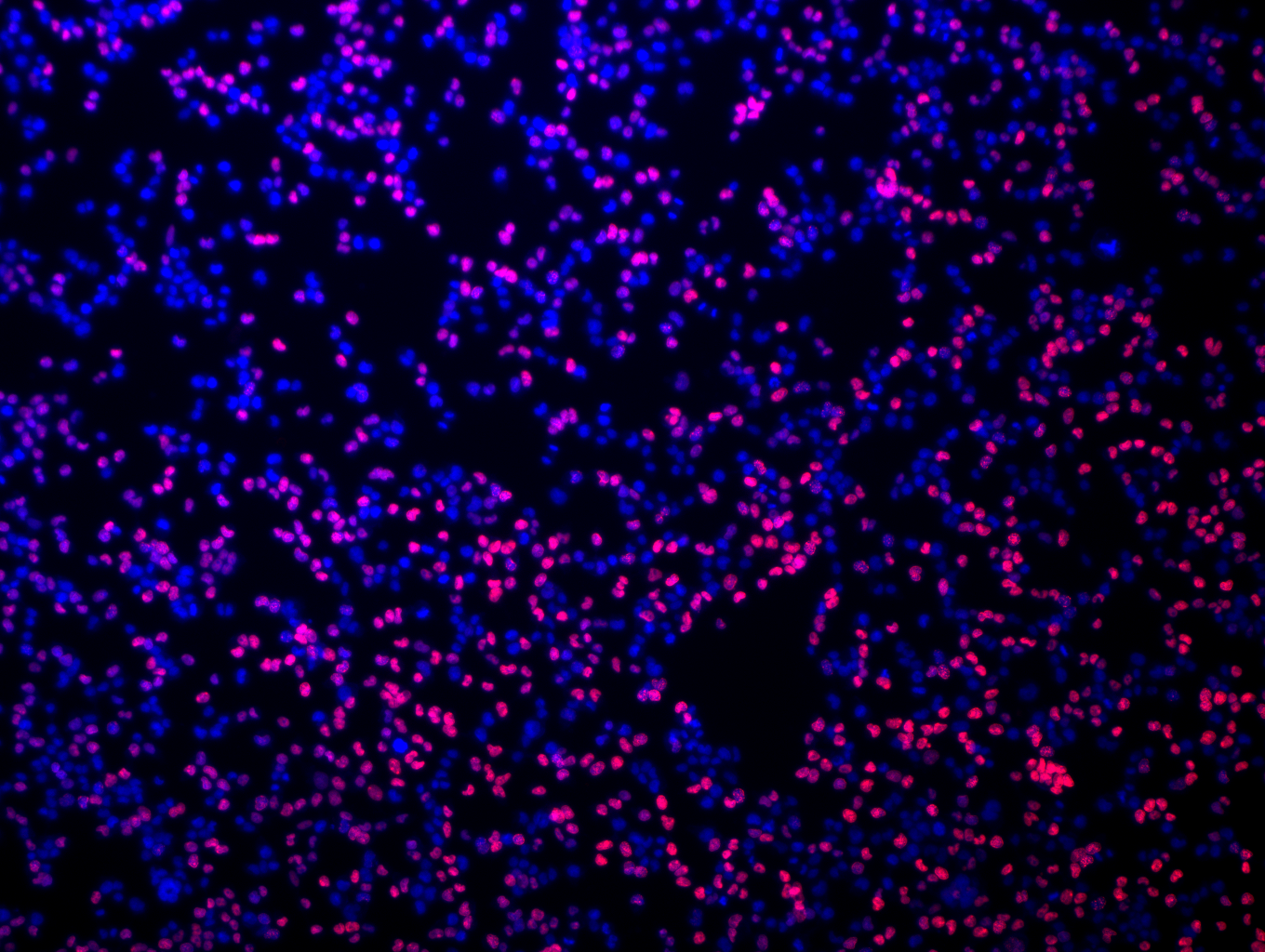

Supplement: Supplementary file 12 — Source data Fig. 7 [file 44318_2024_359_MOESM12_ESM.zip › Figure 7/Fig 7G/hSPAR/merge.tif]

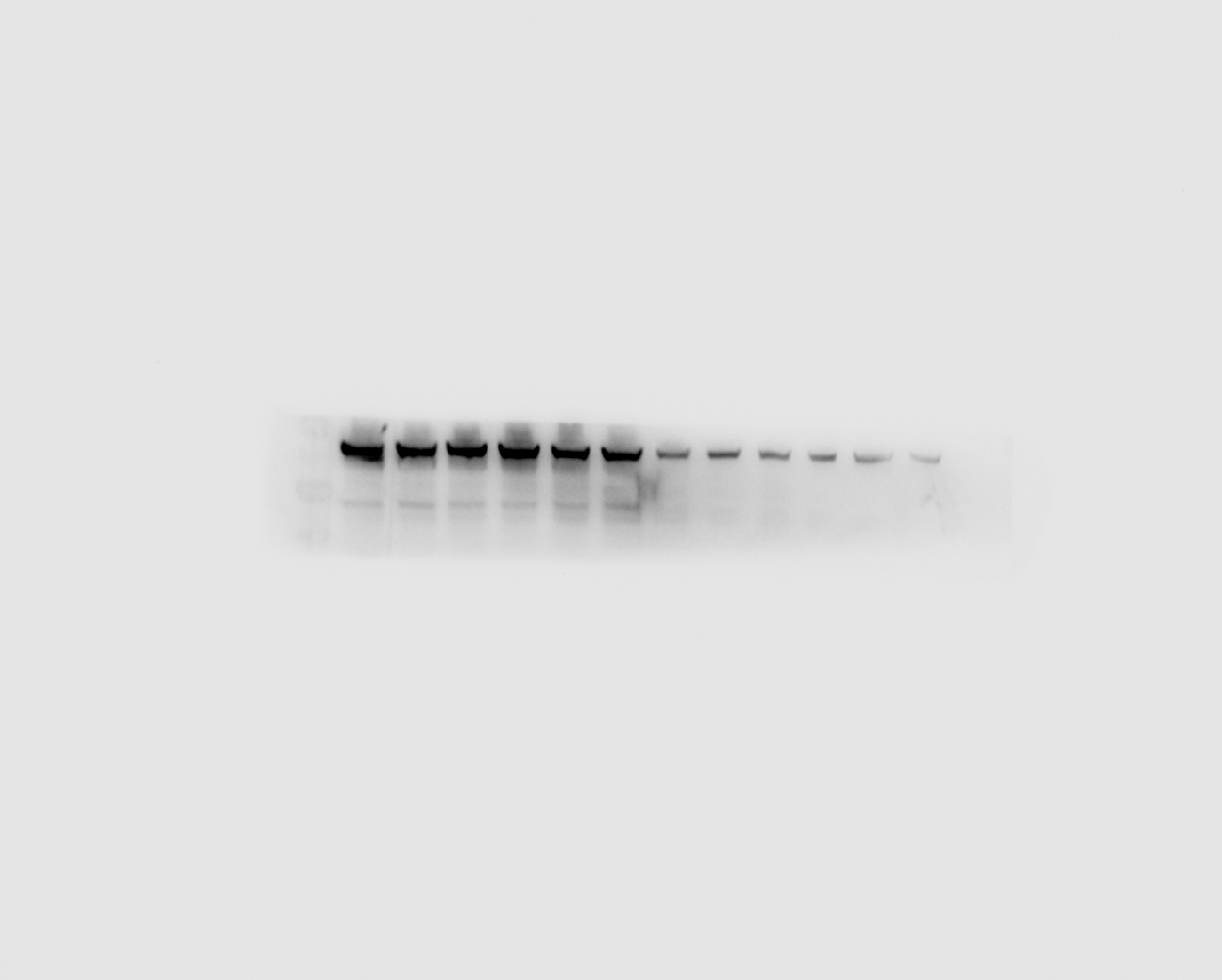

Supplement: Supplementary file 12 — Source data Fig. 7 [file 44318_2024_359_MOESM12_ESM.zip › Figure 7/Fig 7O/5-p-S6K.Tif]

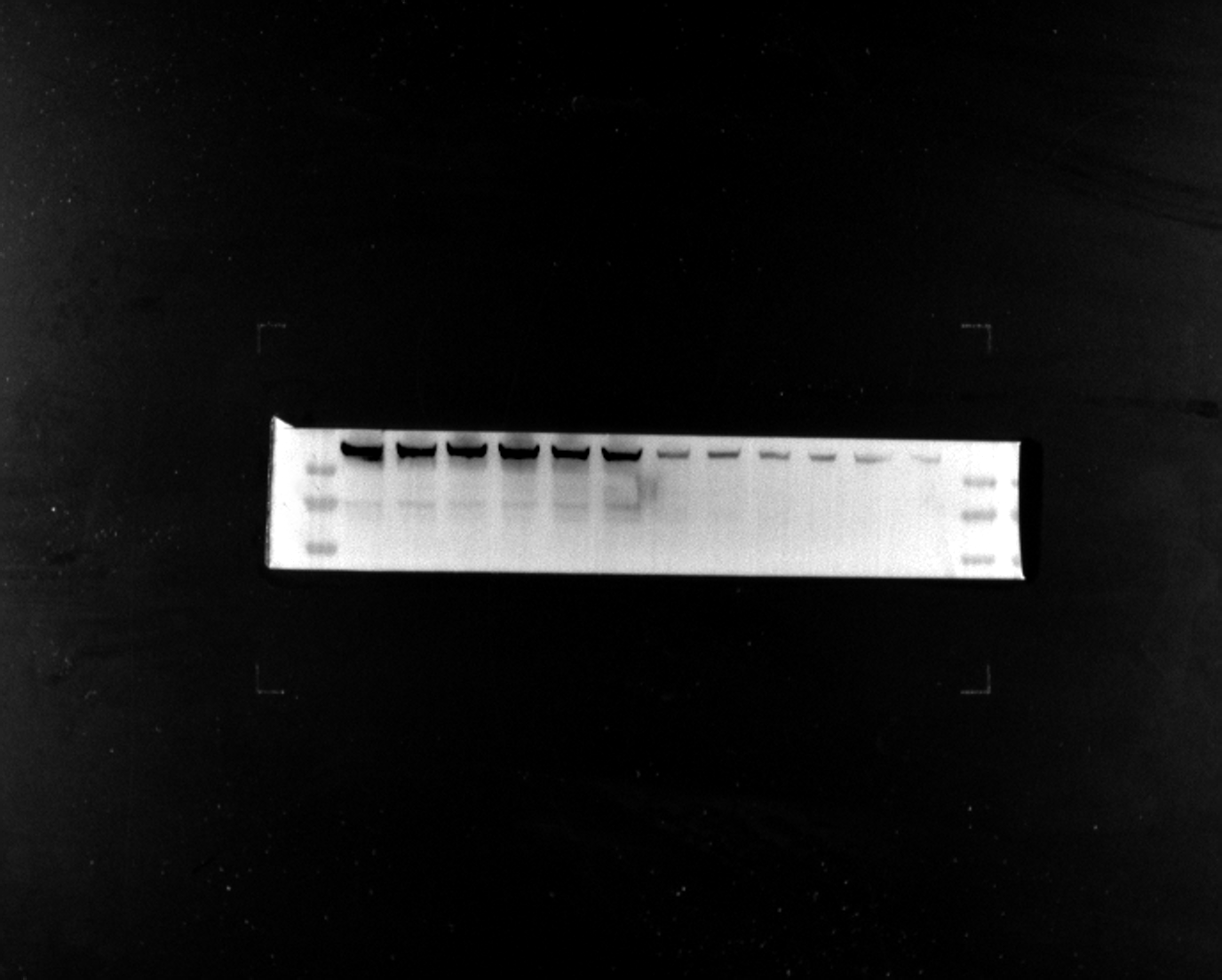

Supplement: Supplementary file 12 — Source data Fig. 7 [file 44318_2024_359_MOESM12_ESM.zip › Figure 7/Fig 7O/5-p-S6K-merge.Tif]

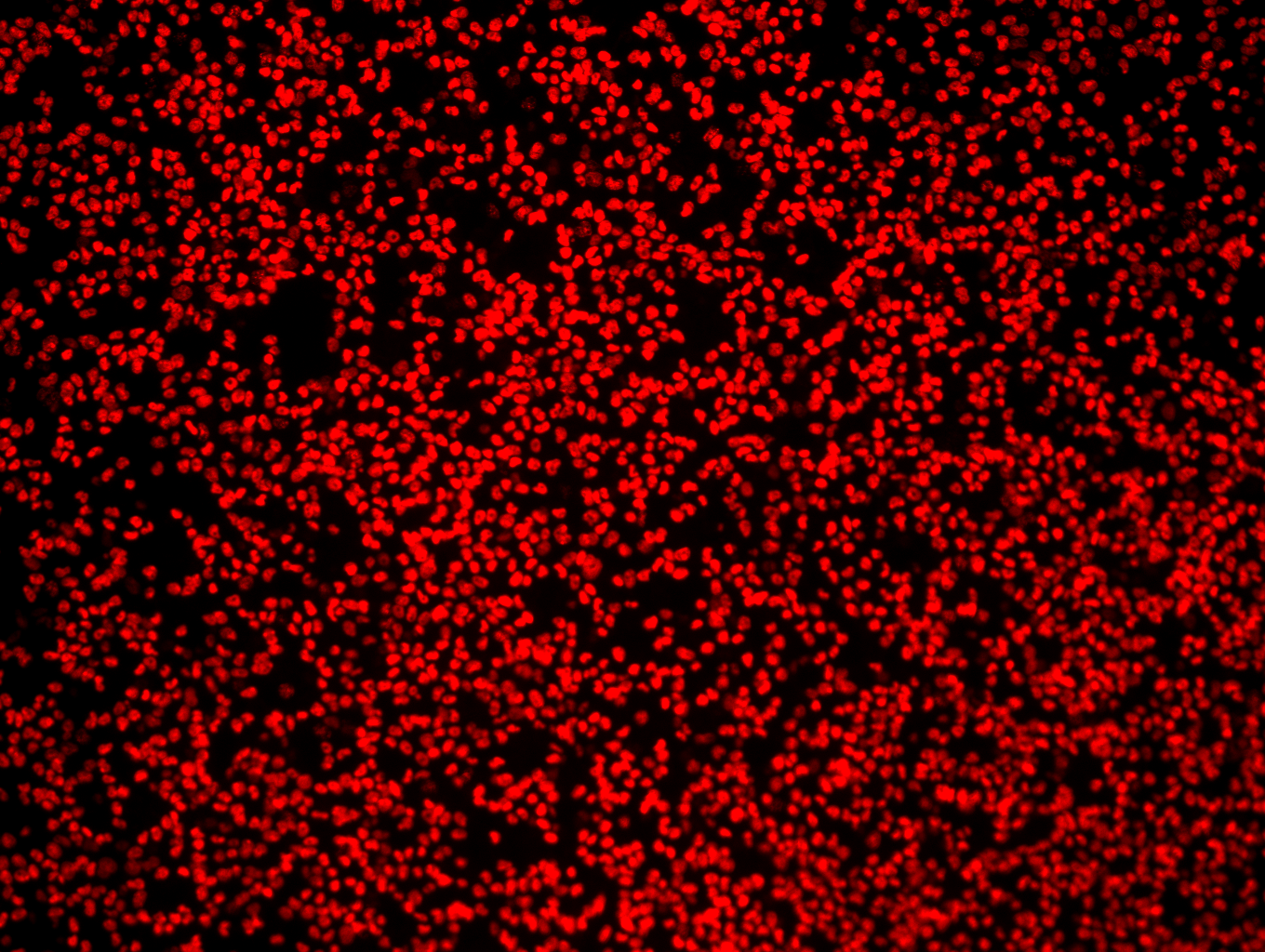

Supplement: Supplementary file 12 — Source data Fig. 7 [file 44318_2024_359_MOESM12_ESM.zip › Figure 7/Fig 7G/hSPAR-ΔC/Edu.tif]

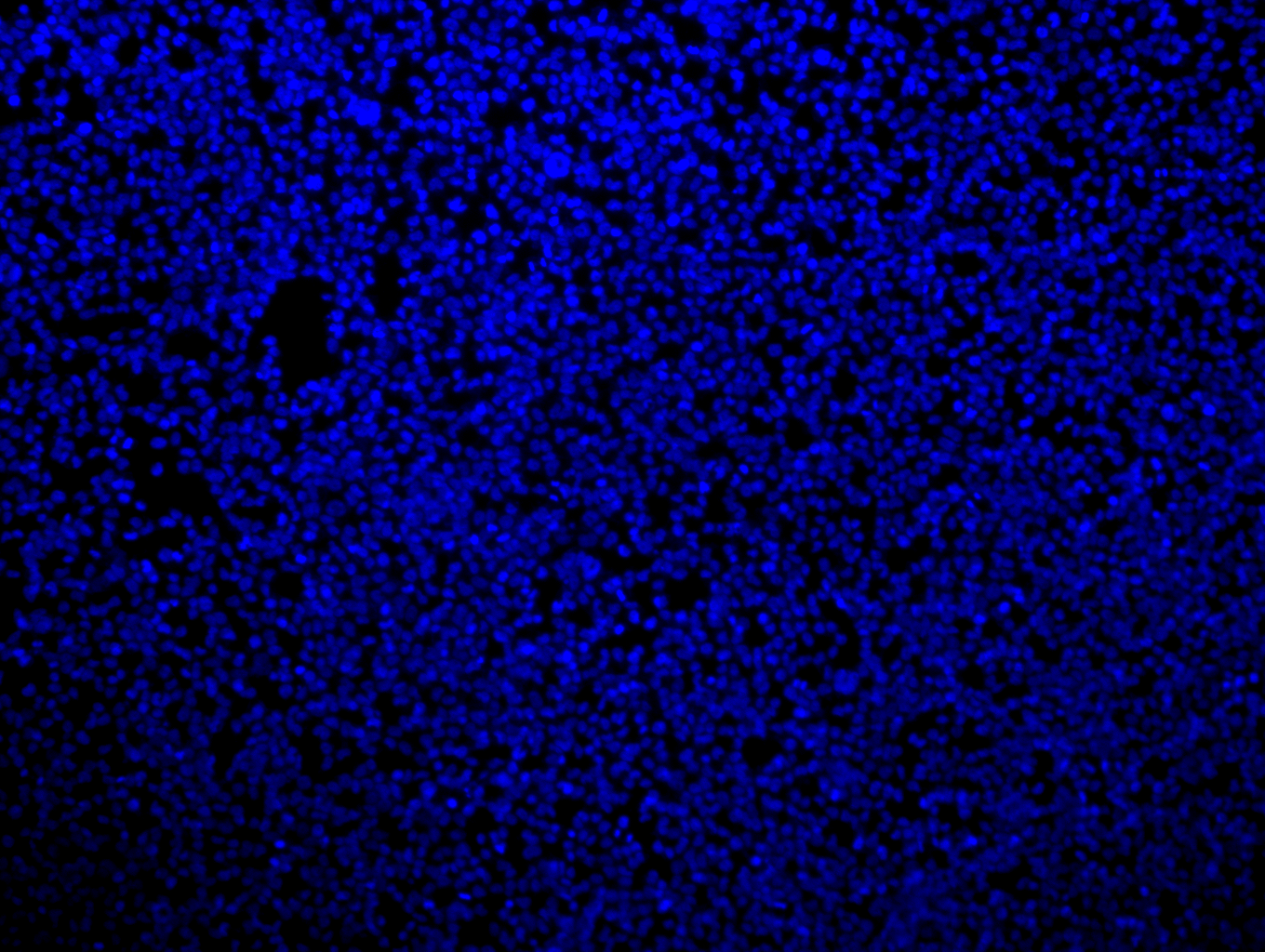

Supplement: Supplementary file 12 — Source data Fig. 7 [file 44318_2024_359_MOESM12_ESM.zip › Figure 7/Fig 7G/hSPAR-ΔC/Hoechst.tif]

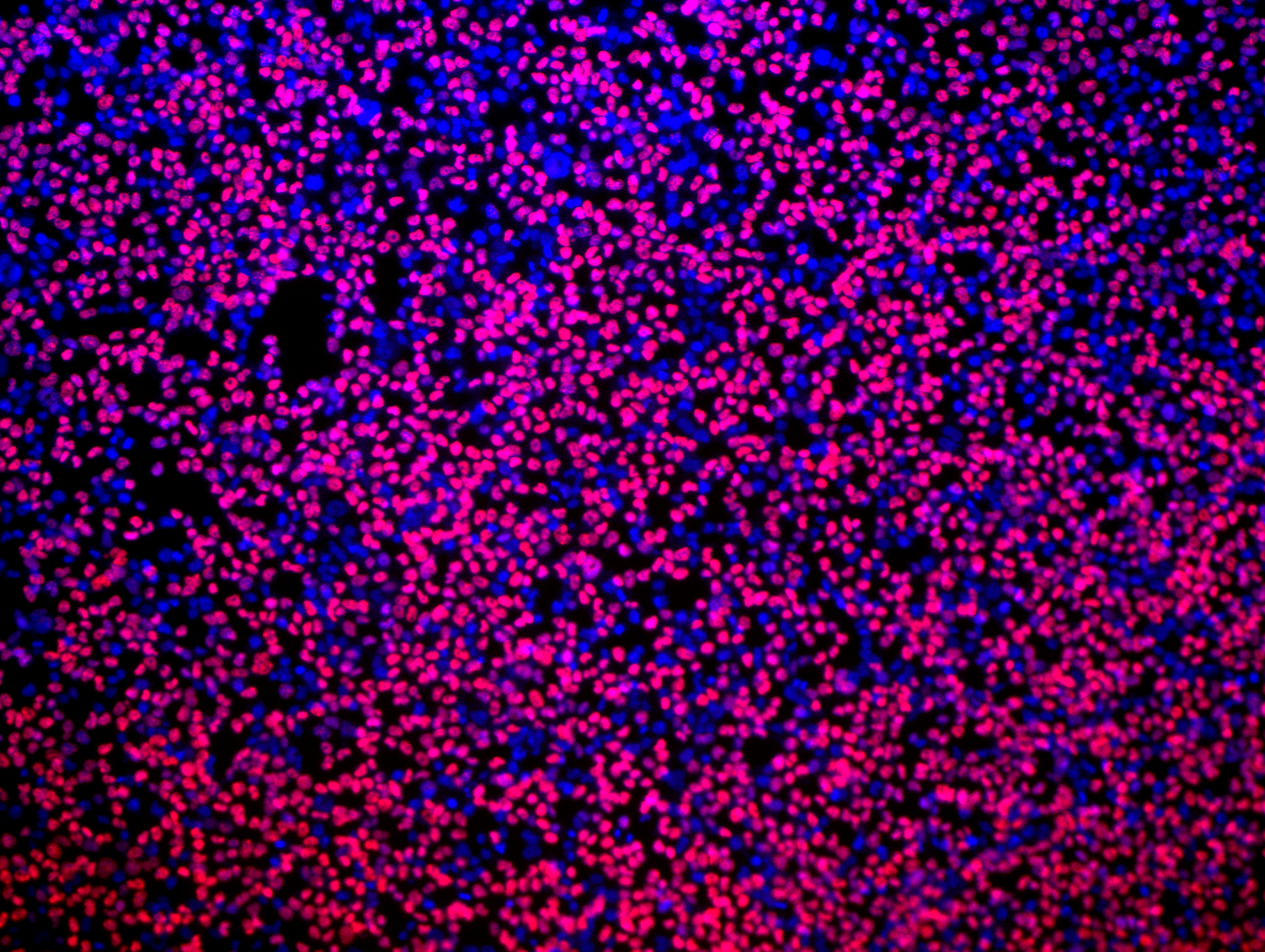

Supplement: Supplementary file 12 — Source data Fig. 7 [file 44318_2024_359_MOESM12_ESM.zip › Figure 7/Fig 7G/hSPAR-ΔC/merge.tif]

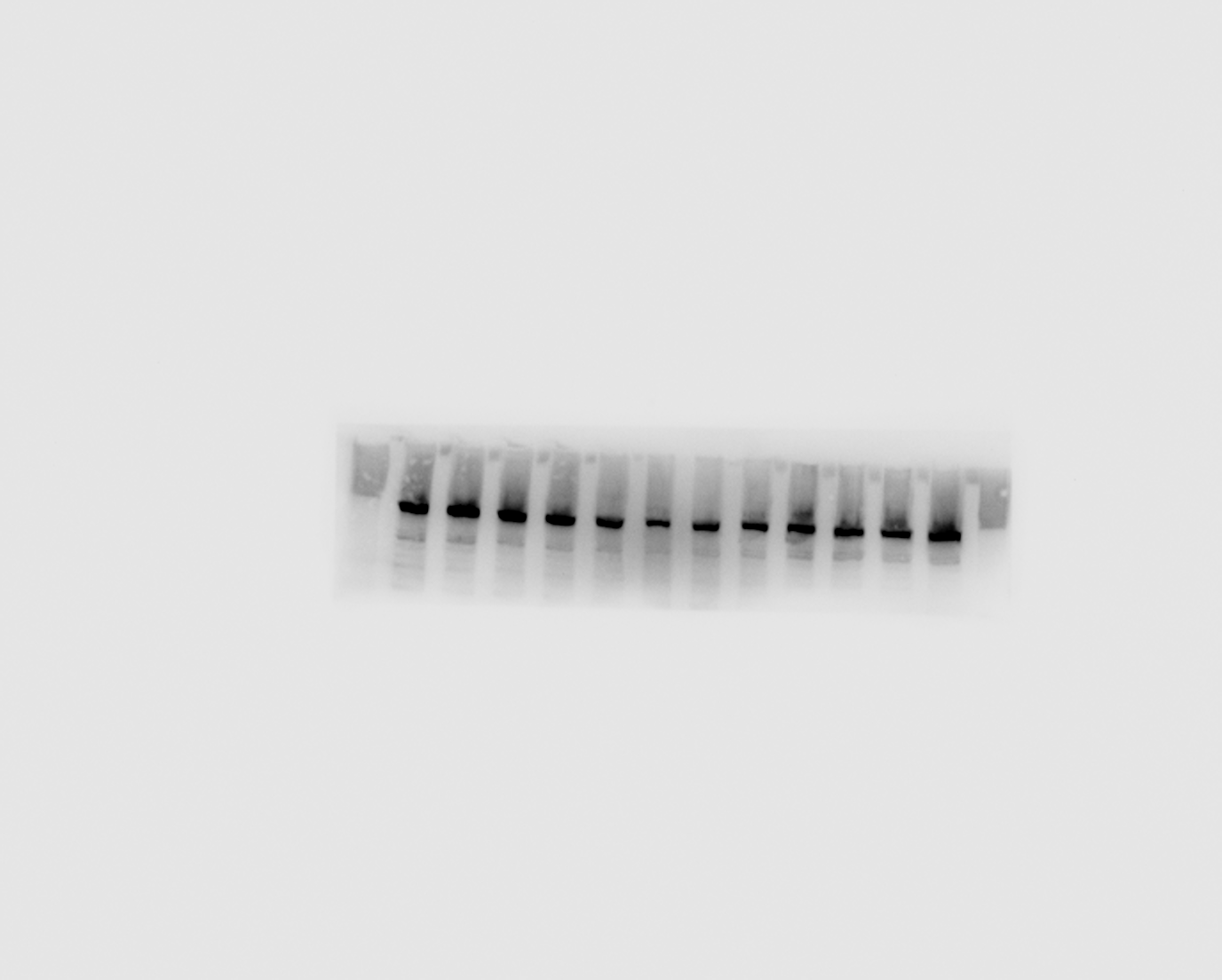

Supplement: Supplementary file 12 — Source data Fig. 7 [file 44318_2024_359_MOESM12_ESM.zip › Figure 7/Fig 7O/4-mTOR.Tif]

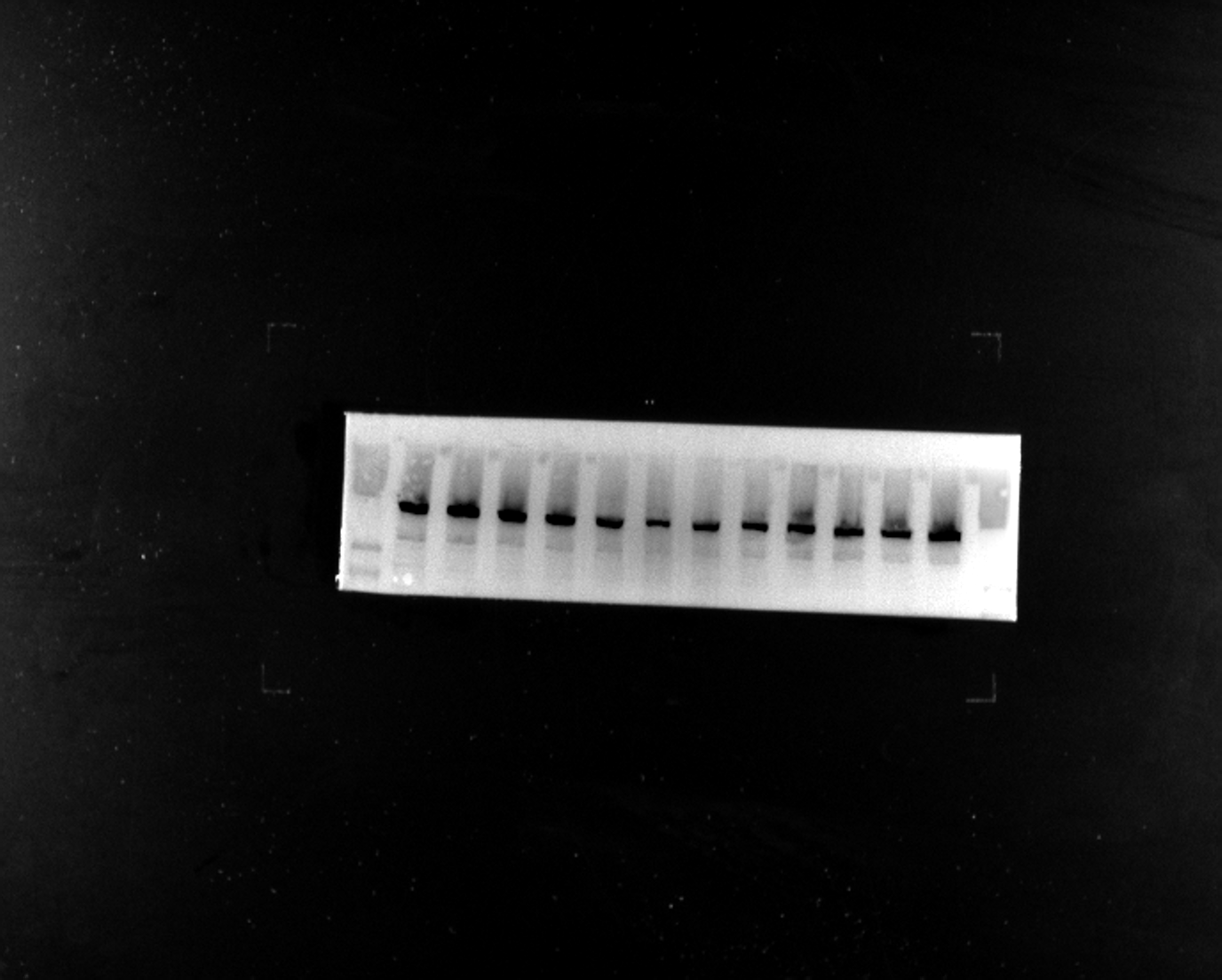

Supplement: Supplementary file 12 — Source data Fig. 7 [file 44318_2024_359_MOESM12_ESM.zip › Figure 7/Fig 7O/4-mTOR-merge.Tif]

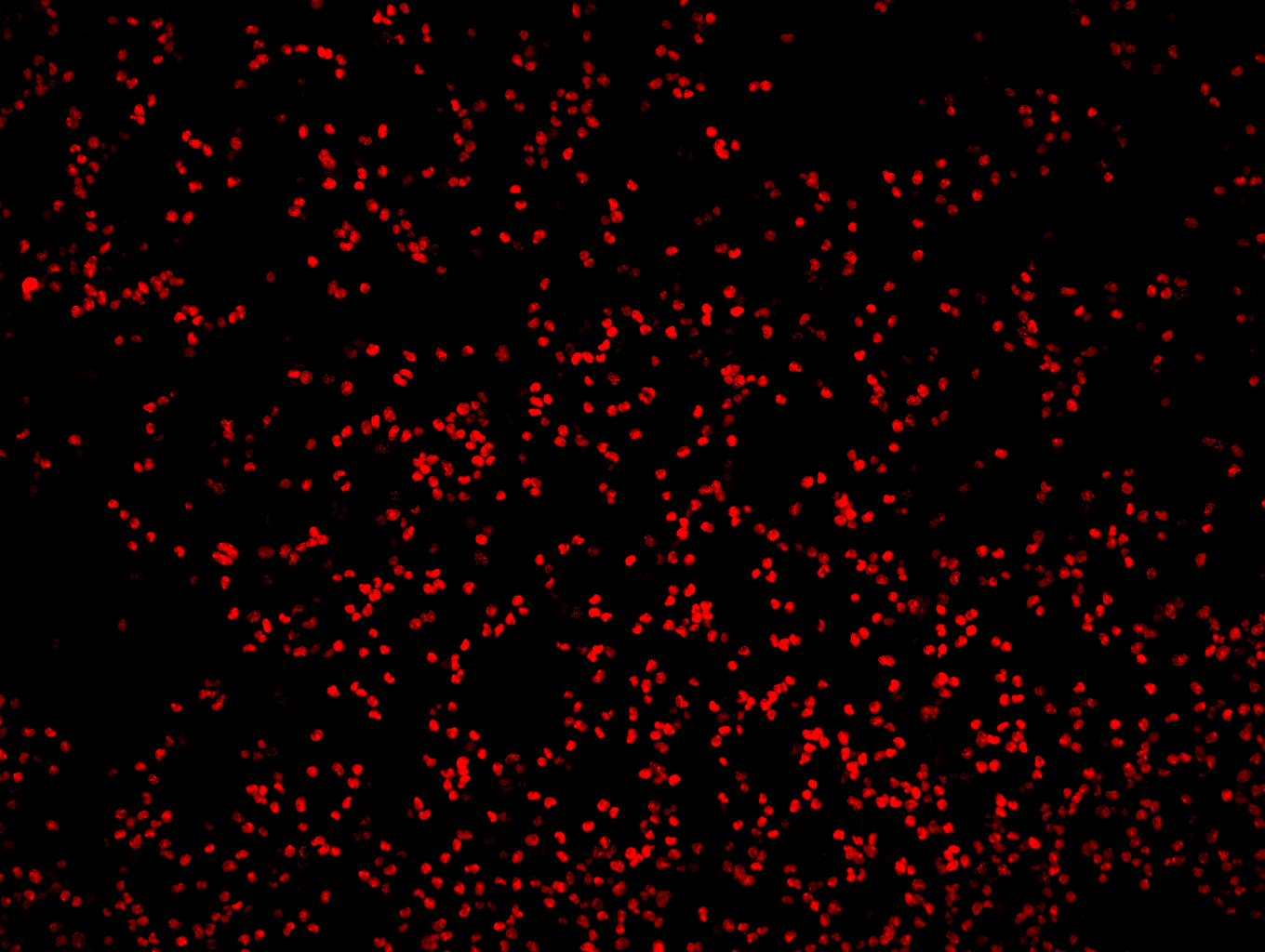

Supplement: Supplementary file 12 — Source data Fig. 7 [file 44318_2024_359_MOESM12_ESM.zip › Figure 7/Fig 7G/hSPAR-ΔN/Edu.tif]

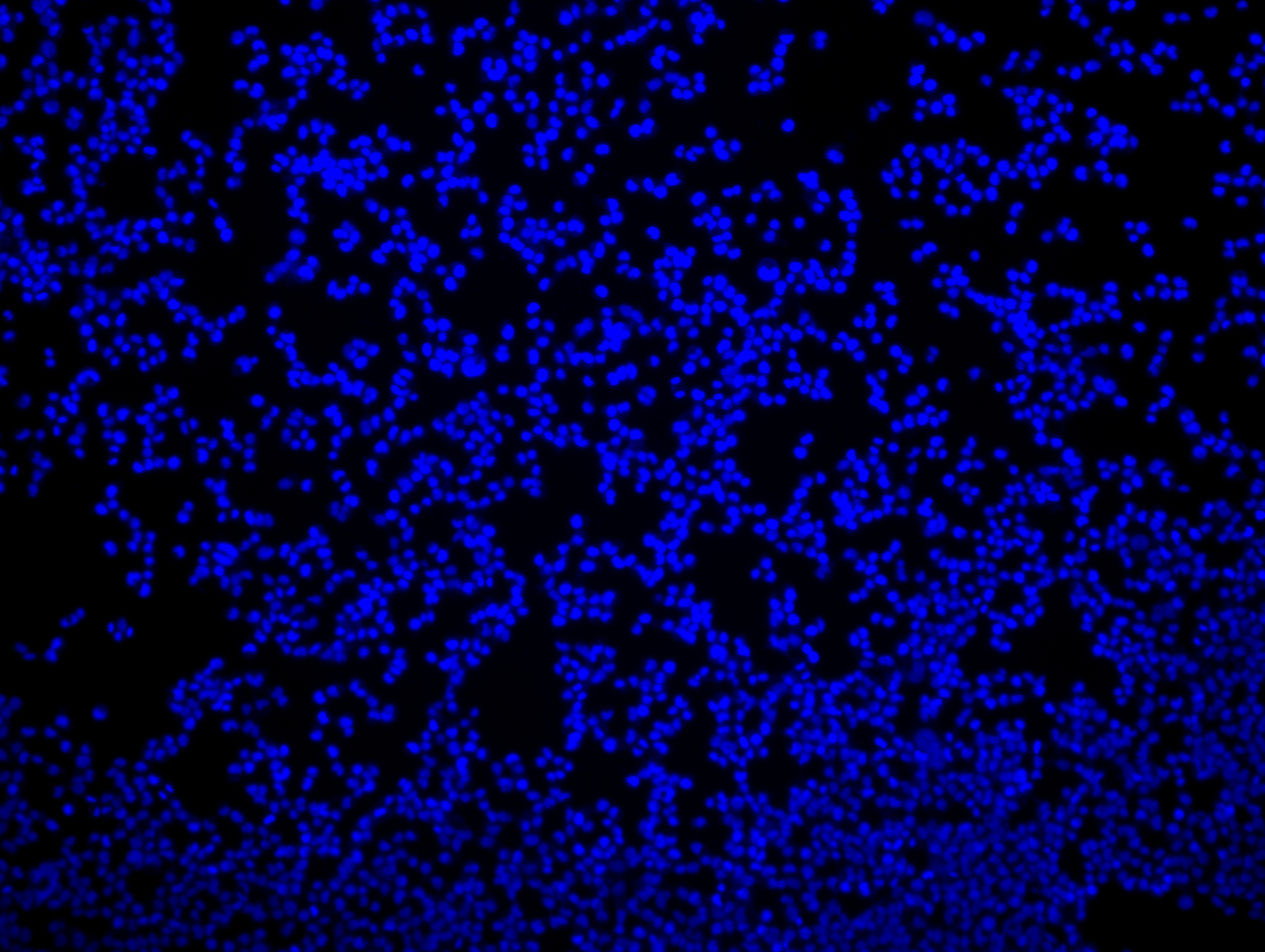

Supplement: Supplementary file 12 — Source data Fig. 7 [file 44318_2024_359_MOESM12_ESM.zip › Figure 7/Fig 7G/hSPAR-ΔN/Hoechst.tif]

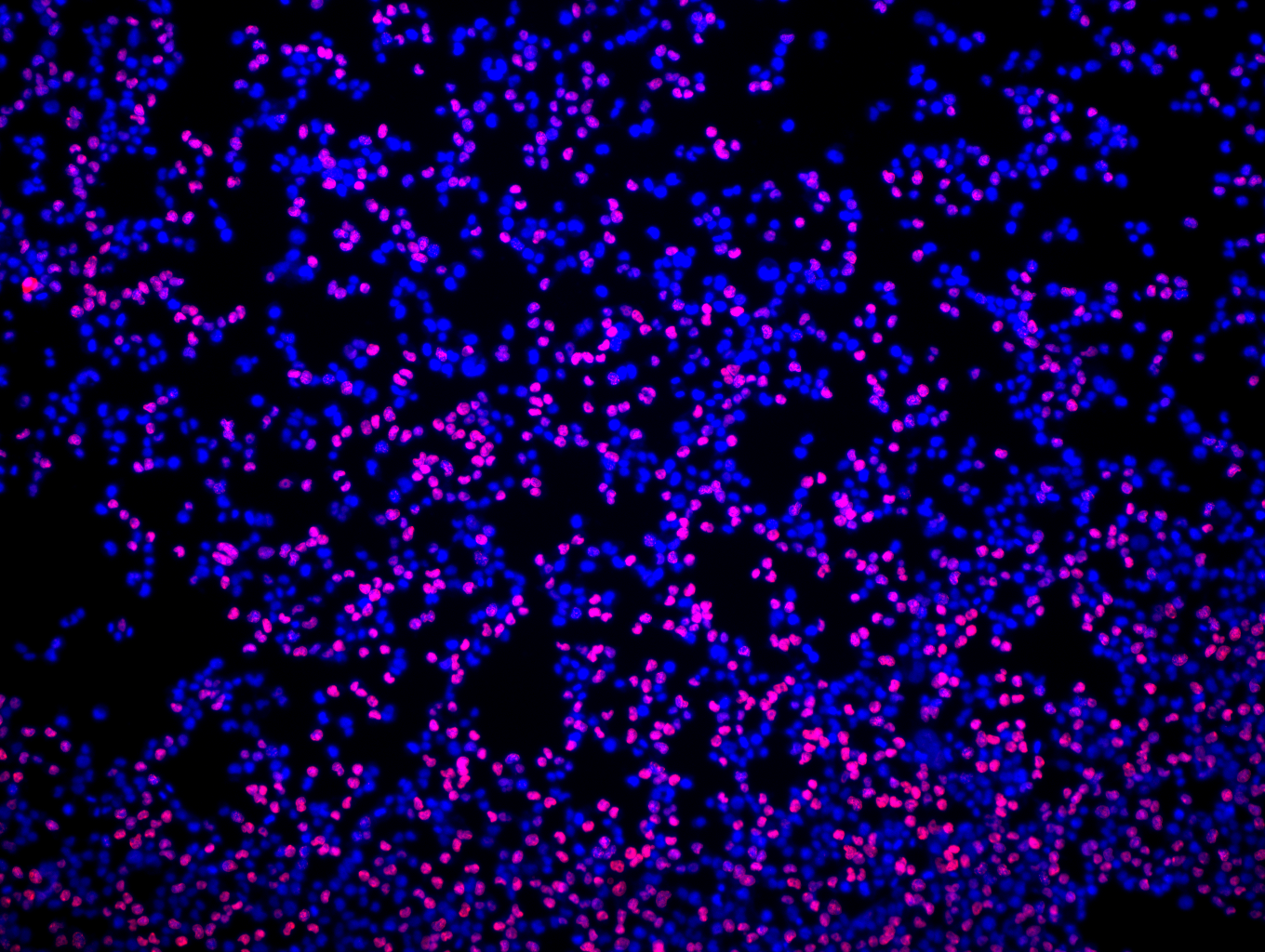

Supplement: Supplementary file 12 — Source data Fig. 7 [file 44318_2024_359_MOESM12_ESM.zip › Figure 7/Fig 7G/hSPAR-ΔN/merge.tif]

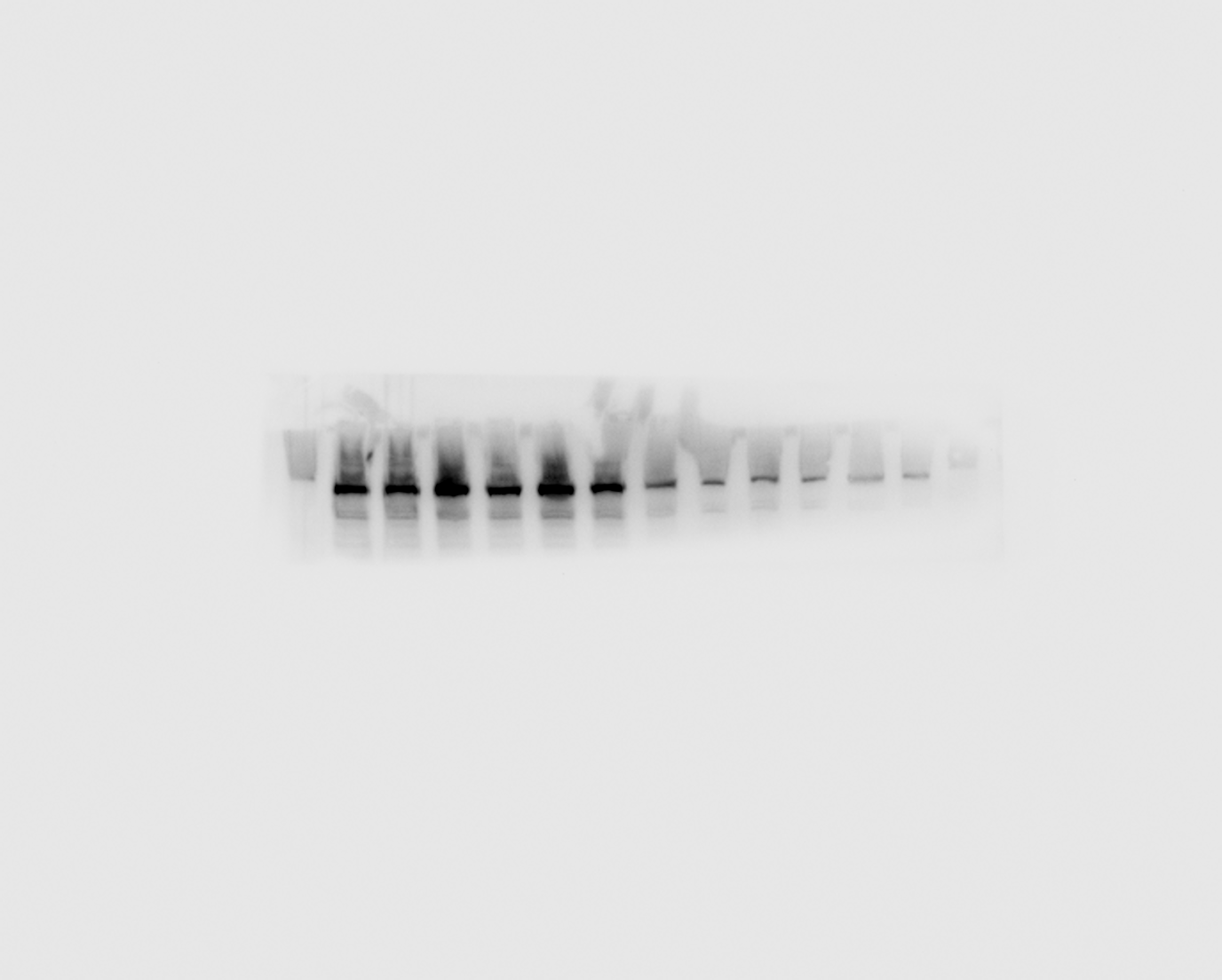

Supplement: Supplementary file 12 — Source data Fig. 7 [file 44318_2024_359_MOESM12_ESM.zip › Figure 7/Fig 7O/3-p-mTOR.Tif]

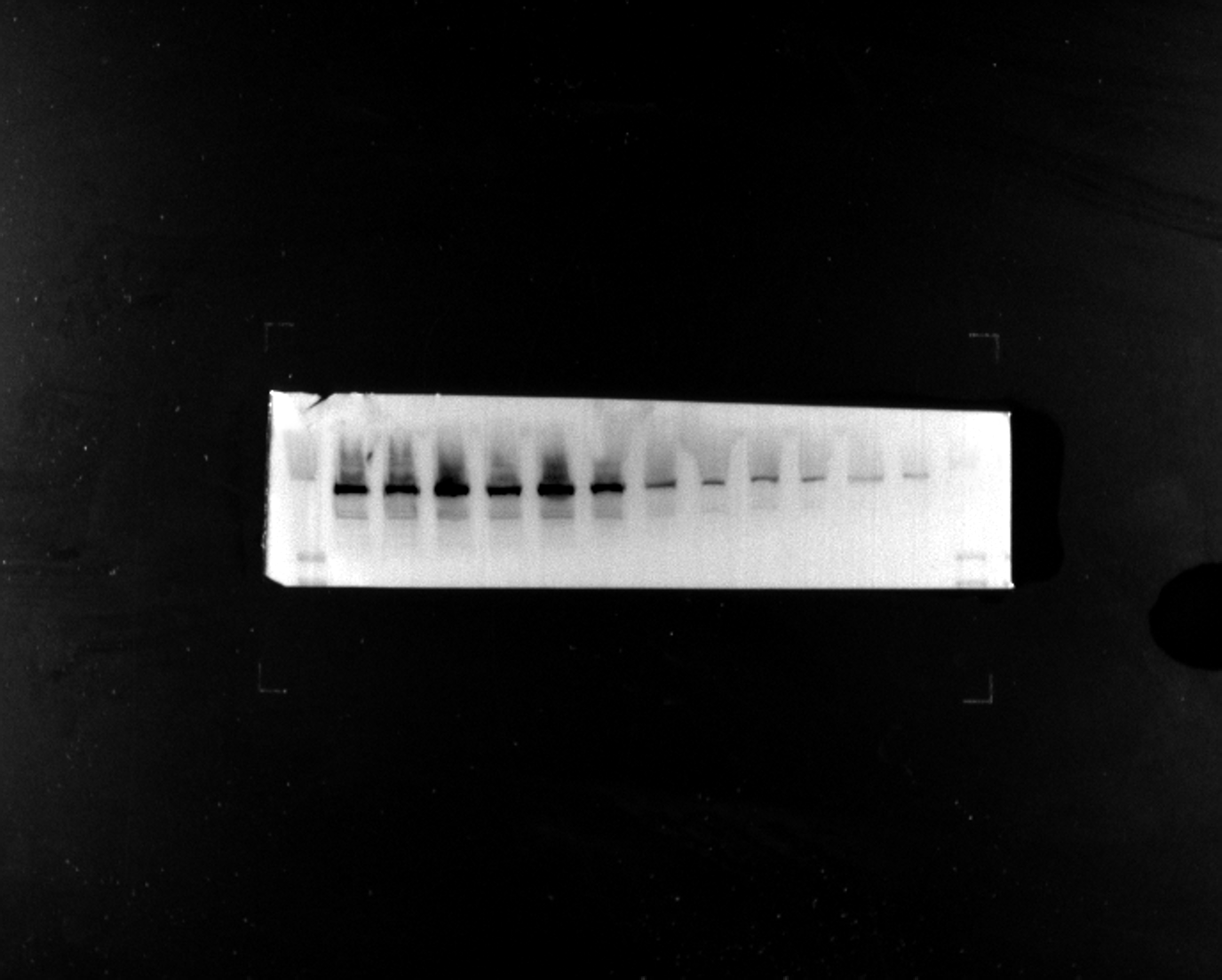

Supplement: Supplementary file 12 — Source data Fig. 7 [file 44318_2024_359_MOESM12_ESM.zip › Figure 7/Fig 7O/3-p-mTOR-merge.Tif]

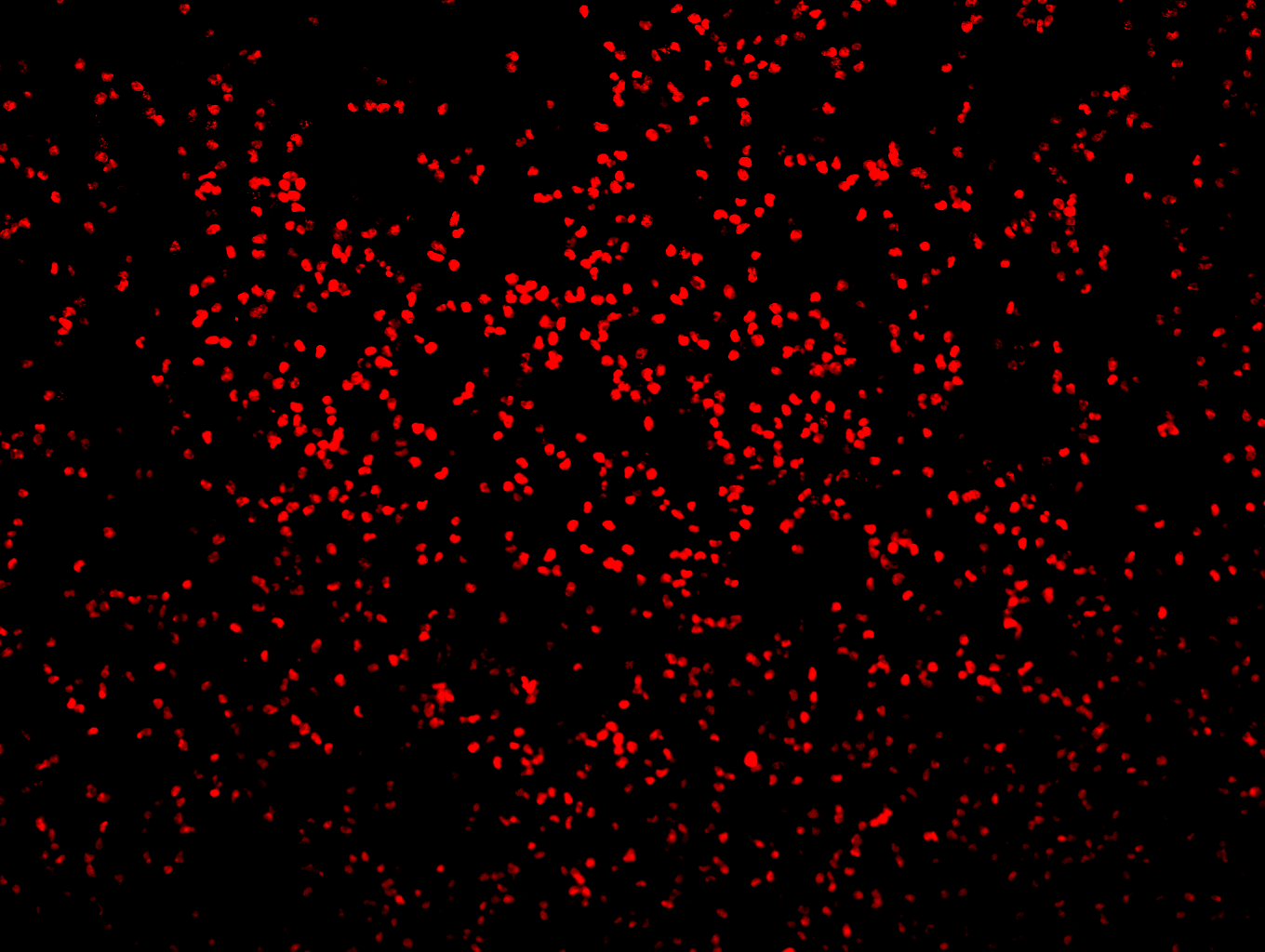

Supplement: Supplementary file 12 — Source data Fig. 7 [file 44318_2024_359_MOESM12_ESM.zip › Figure 7/Fig 7G/hSPAR-ΔTM/Edu.tif]

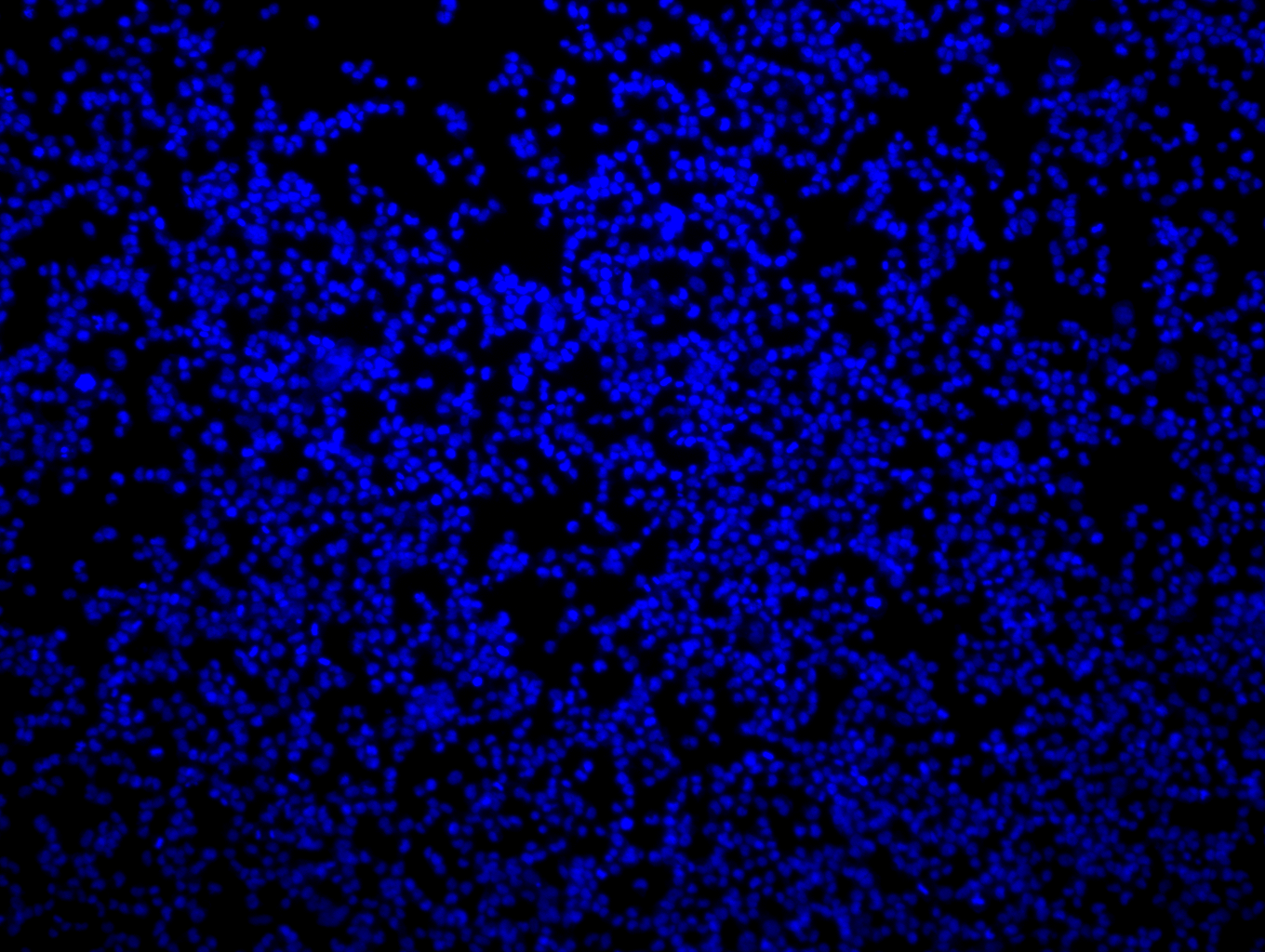

Supplement: Supplementary file 12 — Source data Fig. 7 [file 44318_2024_359_MOESM12_ESM.zip › Figure 7/Fig 7G/hSPAR-ΔTM/Hoechst.tif]

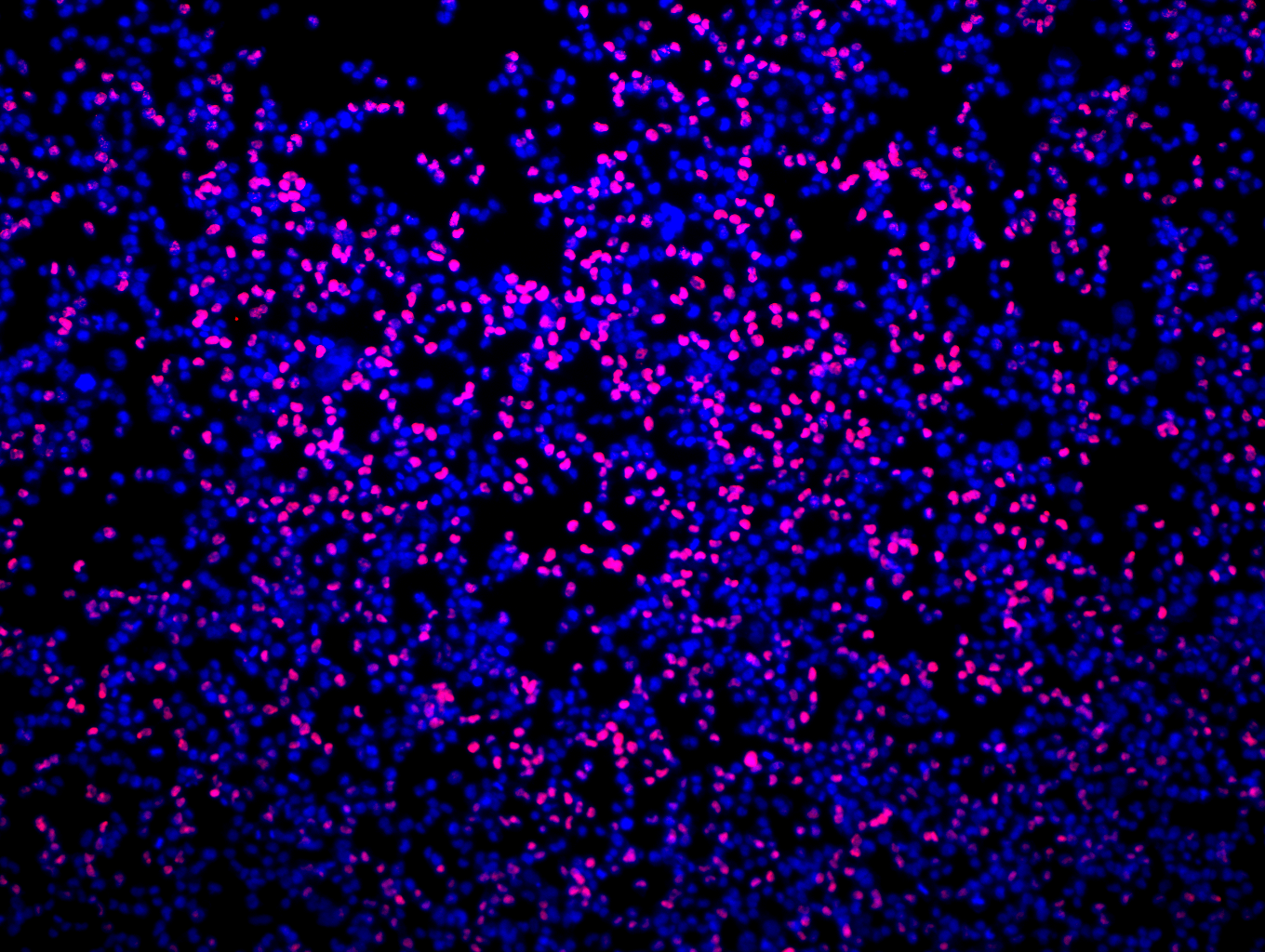

Supplement: Supplementary file 12 — Source data Fig. 7 [file 44318_2024_359_MOESM12_ESM.zip › Figure 7/Fig 7G/hSPAR-ΔTM/merge.tif]

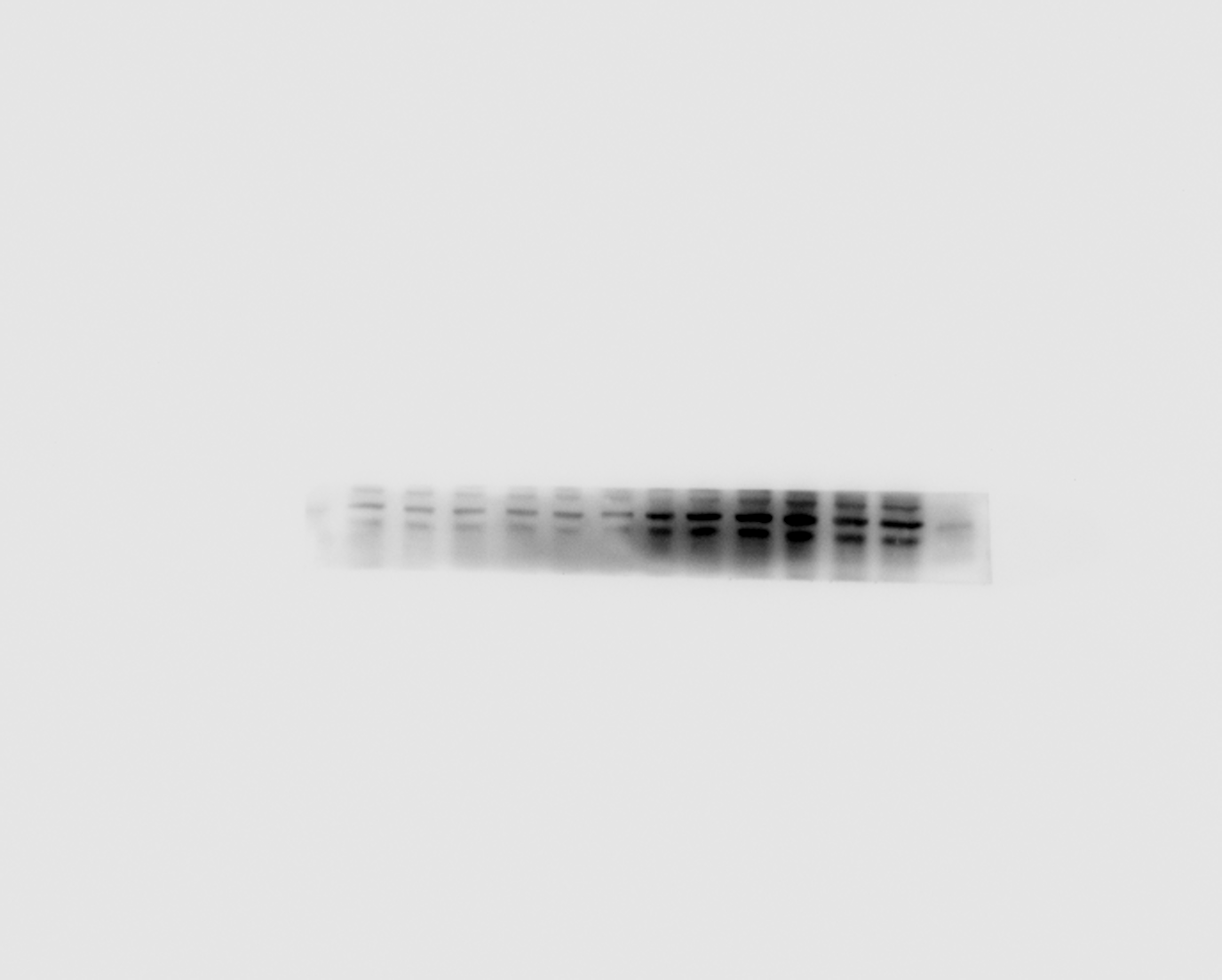

Supplement: Supplementary file 12 — Source data Fig. 7 [file 44318_2024_359_MOESM12_ESM.zip › Figure 7/Fig 7O/2-p27.Tif]

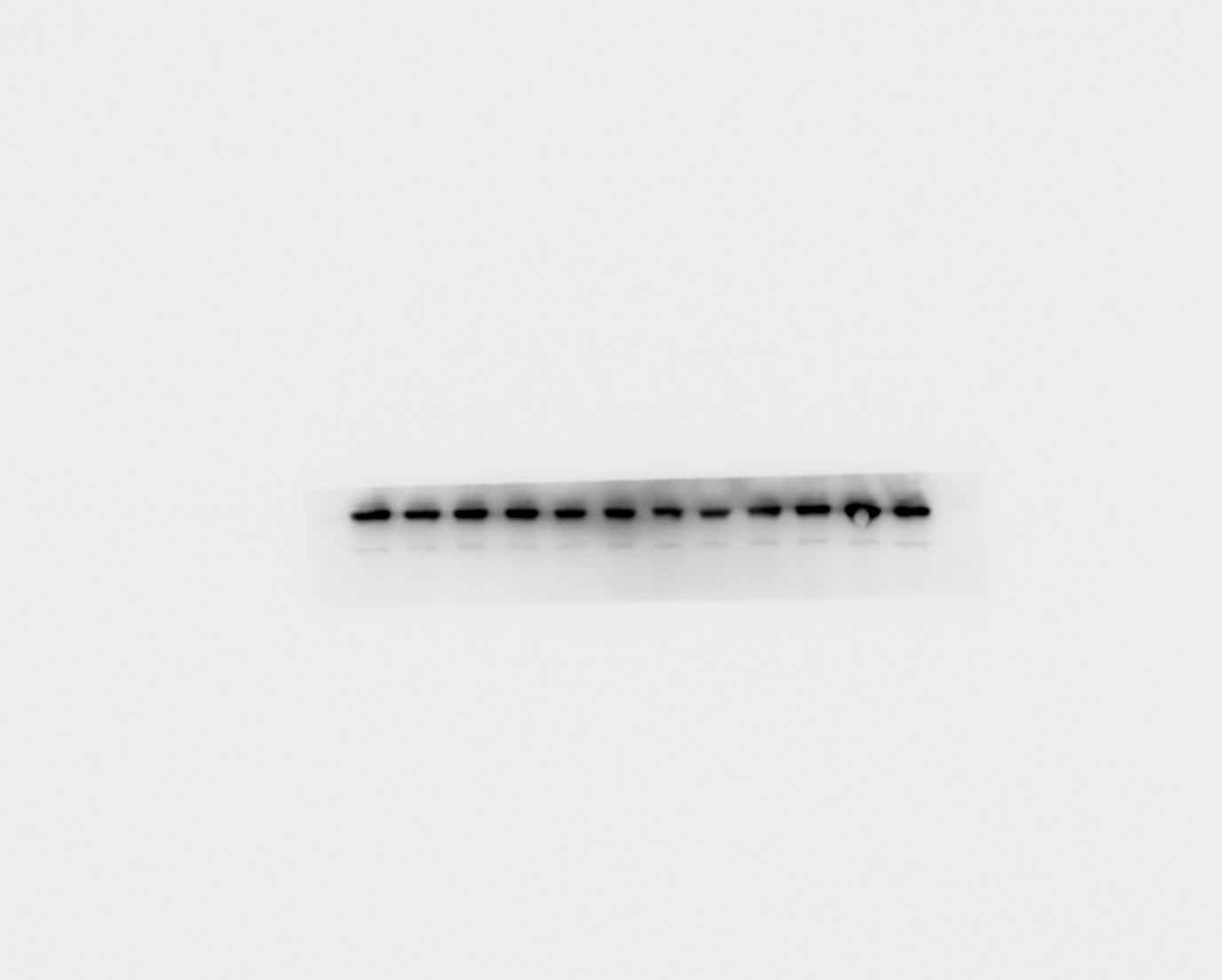

Supplement: Supplementary file 12 — Source data Fig. 7 [file 44318_2024_359_MOESM12_ESM.zip › Figure 7/Fig 7O/10-GAPDH.Tif]

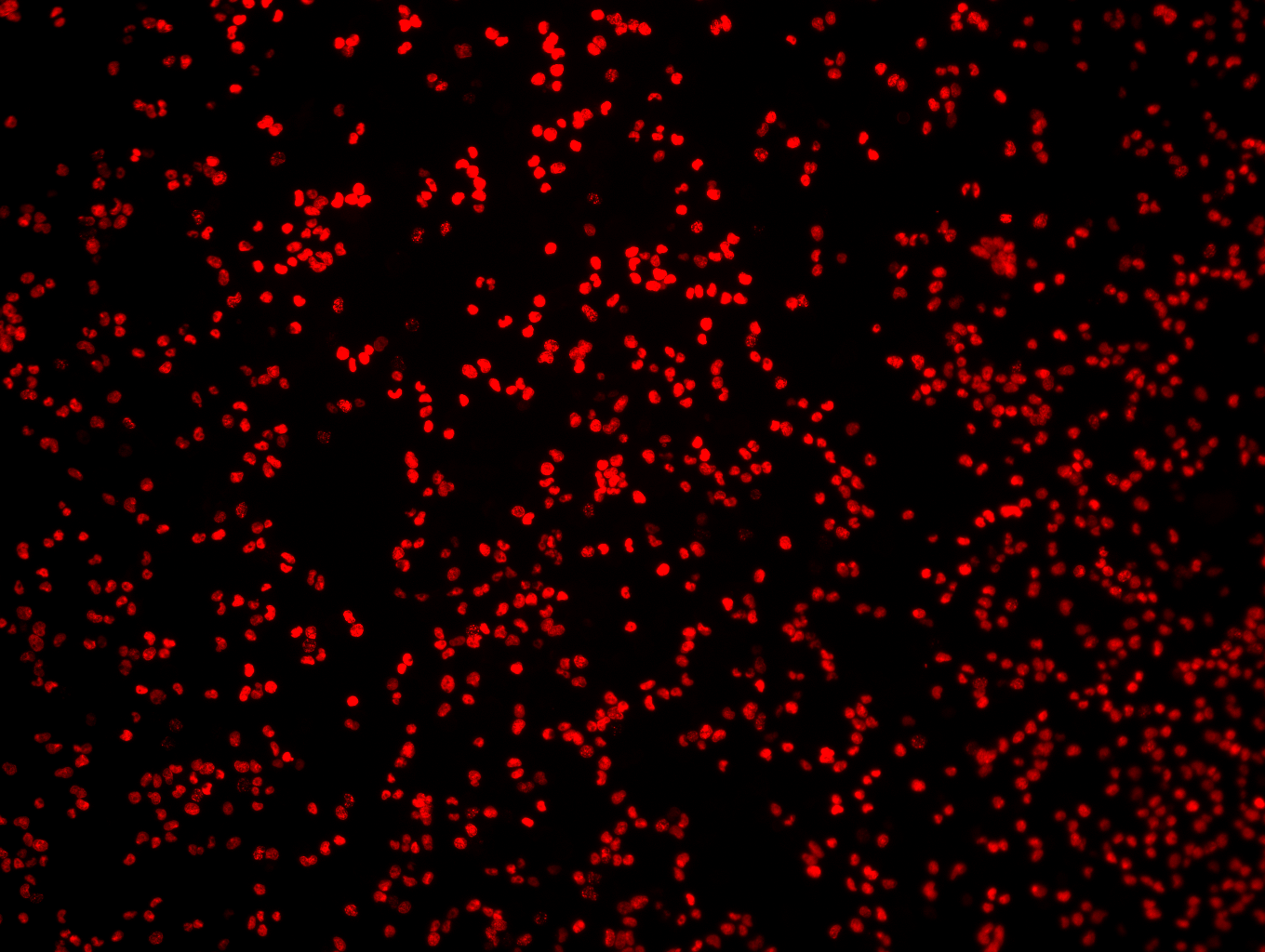

Supplement: Supplementary file 12 — Source data Fig. 7 [file 44318_2024_359_MOESM12_ESM.zip › Figure 7/Fig 7G/hSPAR_C/Edu.tif]

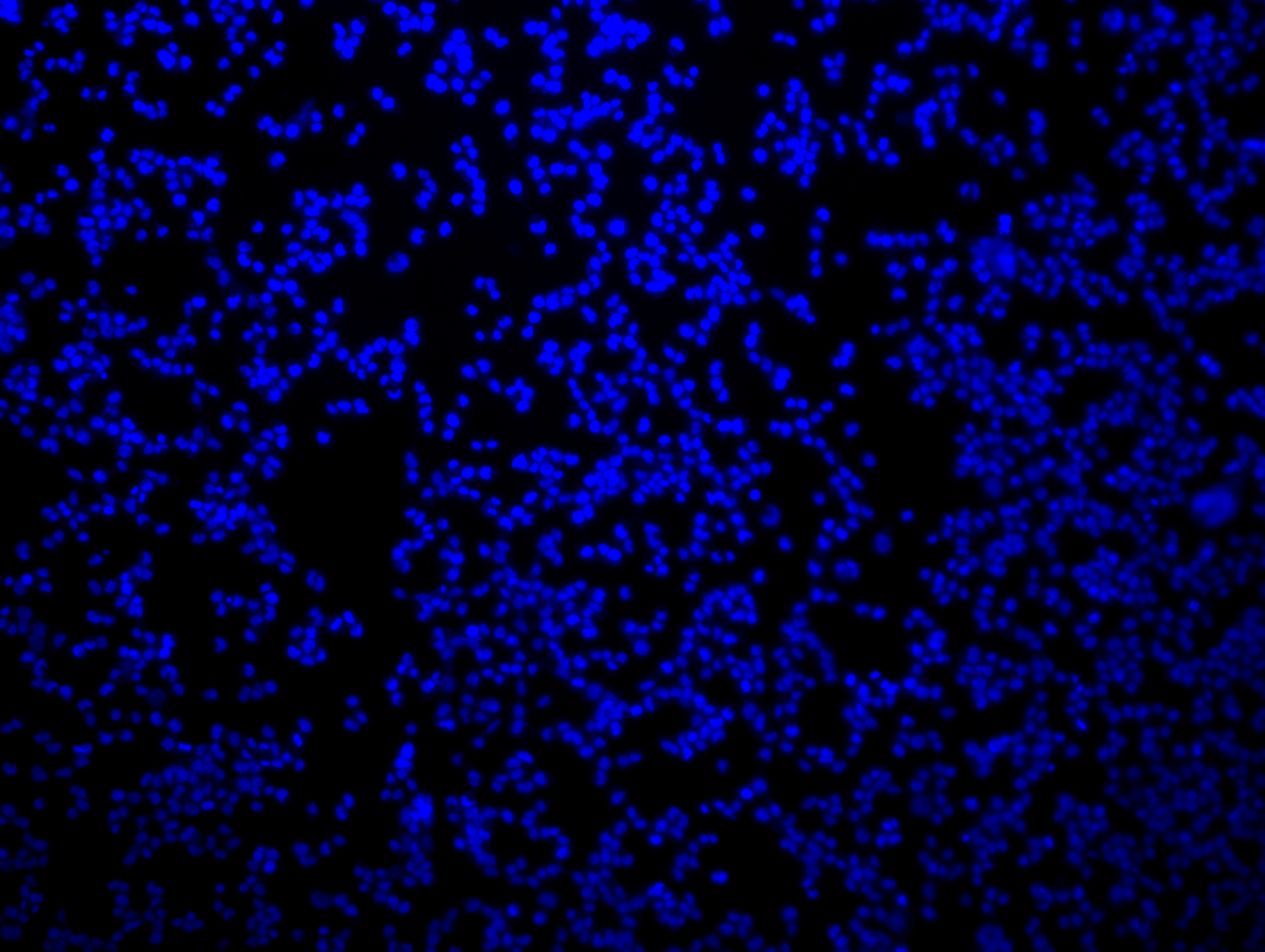

Supplement: Supplementary file 12 — Source data Fig. 7 [file 44318_2024_359_MOESM12_ESM.zip › Figure 7/Fig 7G/hSPAR_C/Hoechst.tif]

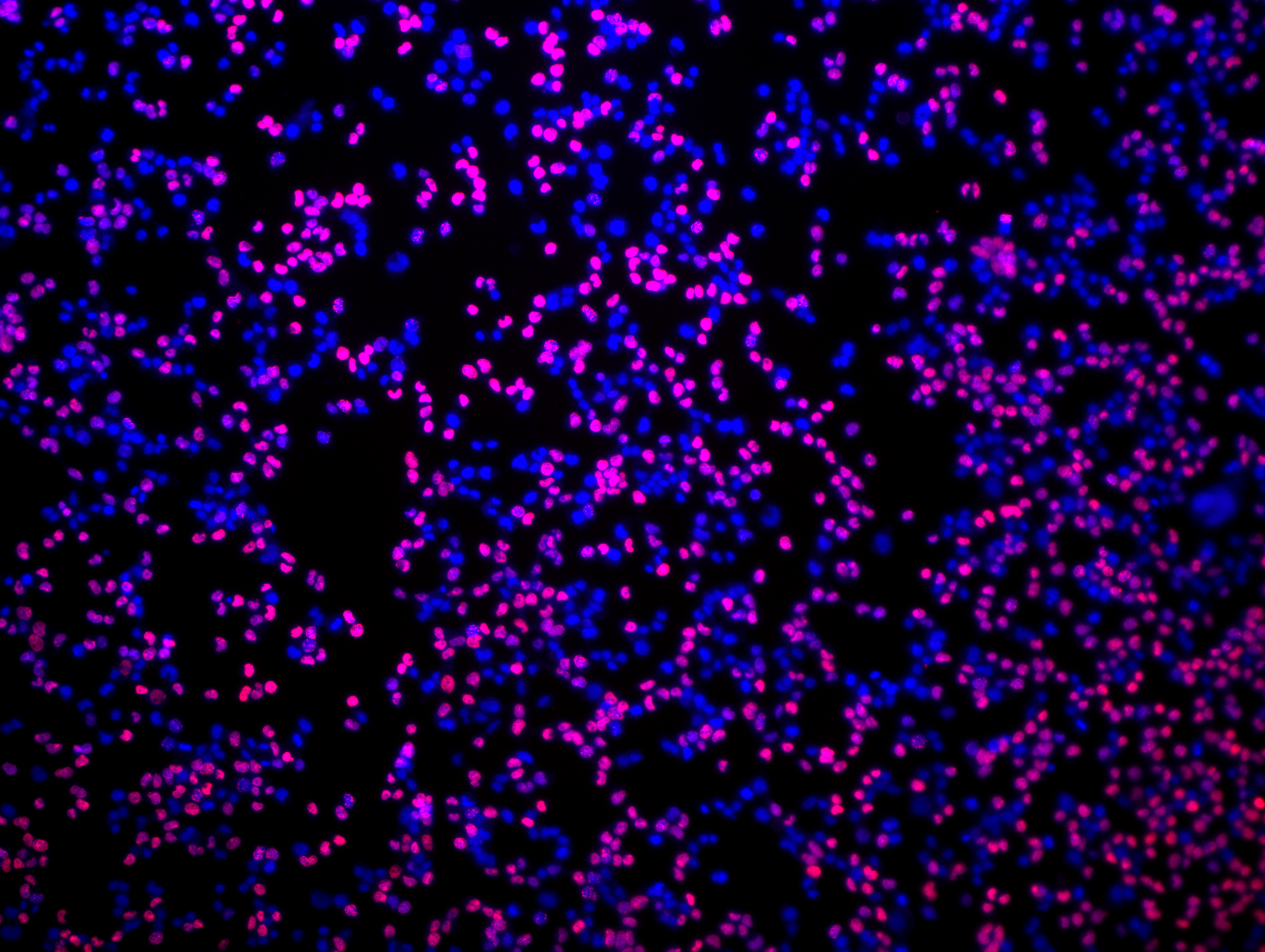

Supplement: Supplementary file 12 — Source data Fig. 7 [file 44318_2024_359_MOESM12_ESM.zip › Figure 7/Fig 7G/hSPAR_C/merge.tif]

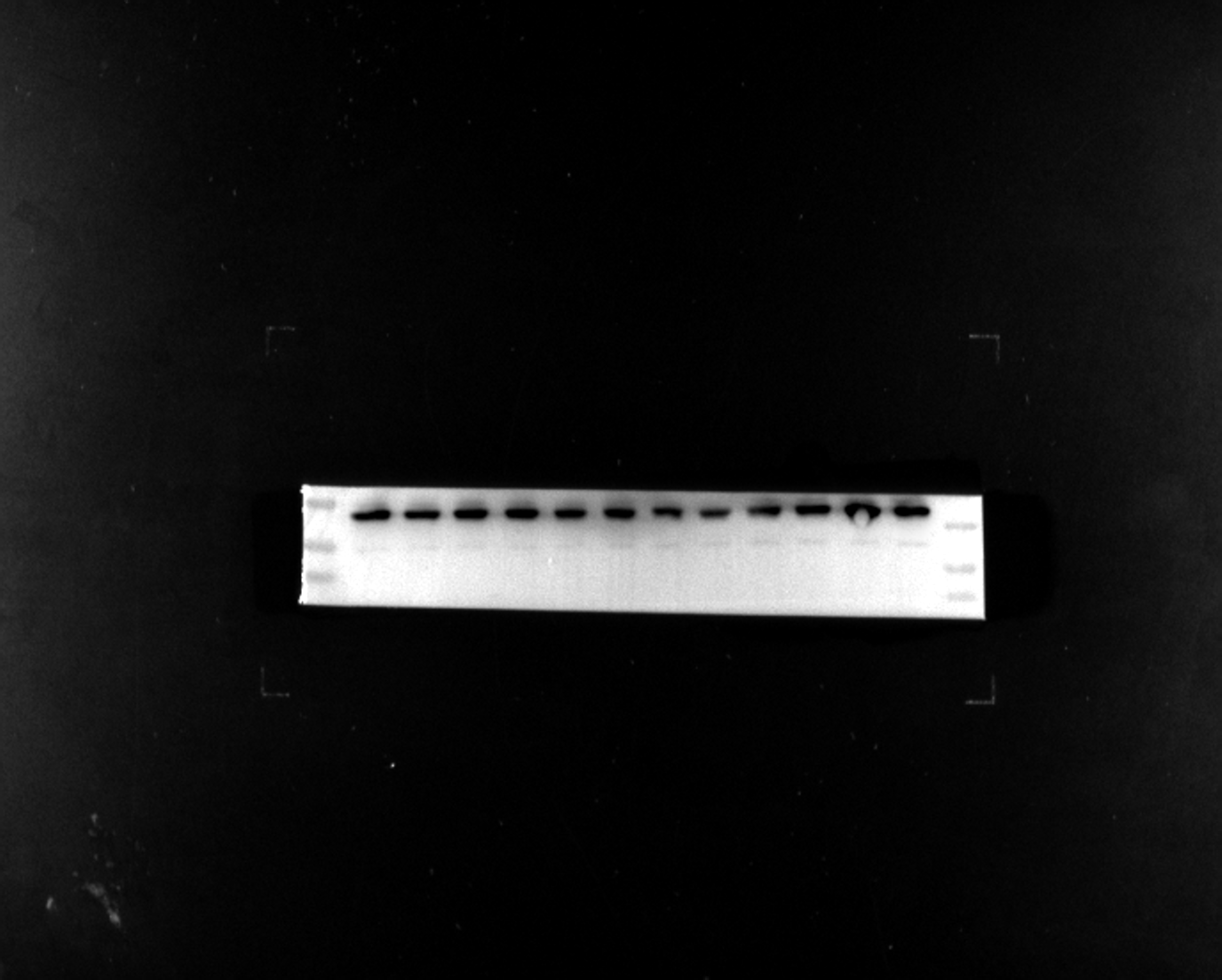

Supplement: Supplementary file 12 — Source data Fig. 7 [file 44318_2024_359_MOESM12_ESM.zip › Figure 7/Fig 7O/10-GAPDH-merge.Tif]

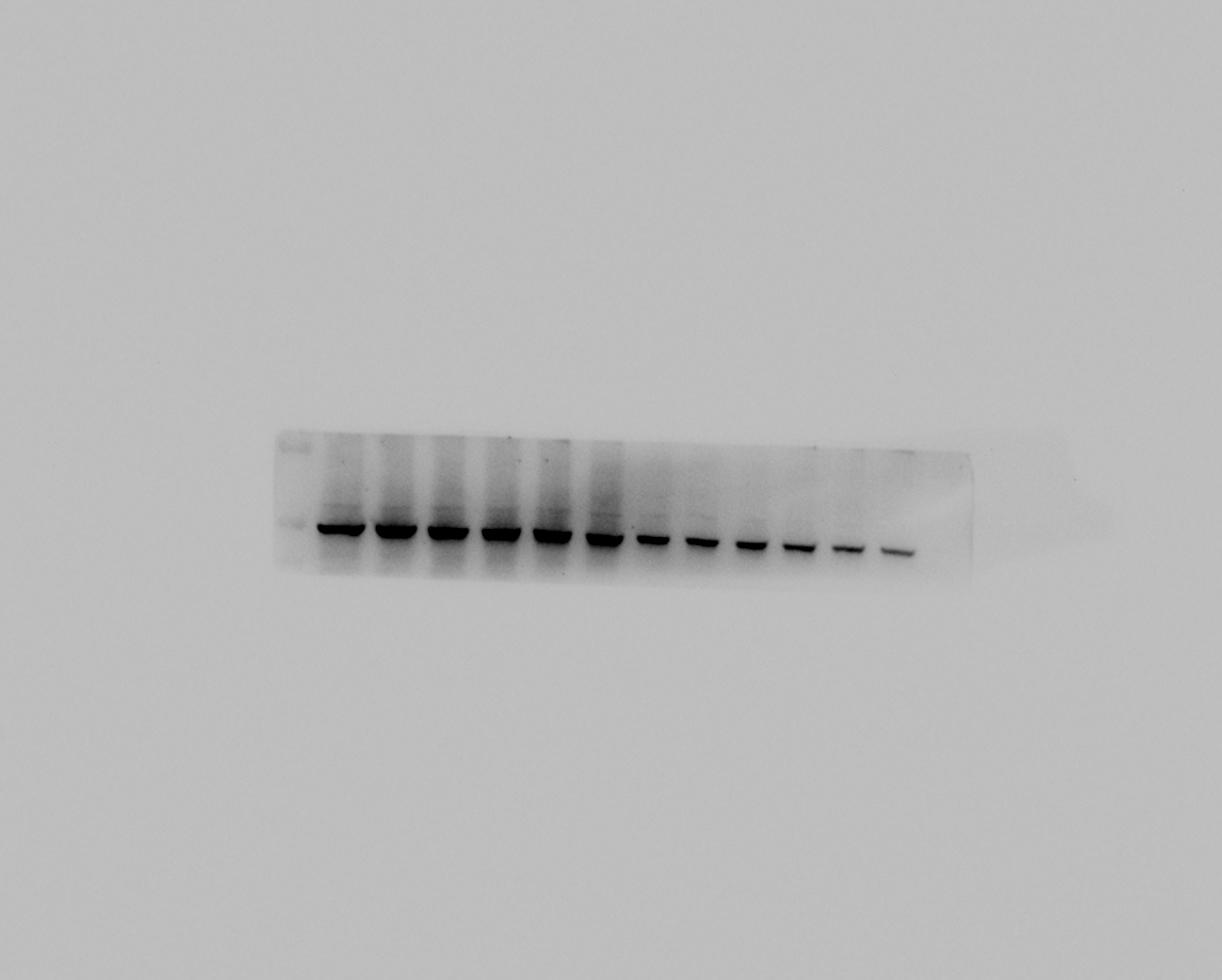

Supplement: Supplementary file 12 — Source data Fig. 7 [file 44318_2024_359_MOESM12_ESM.zip › Figure 7/Fig 7O/1-SLC38A2.Tif]

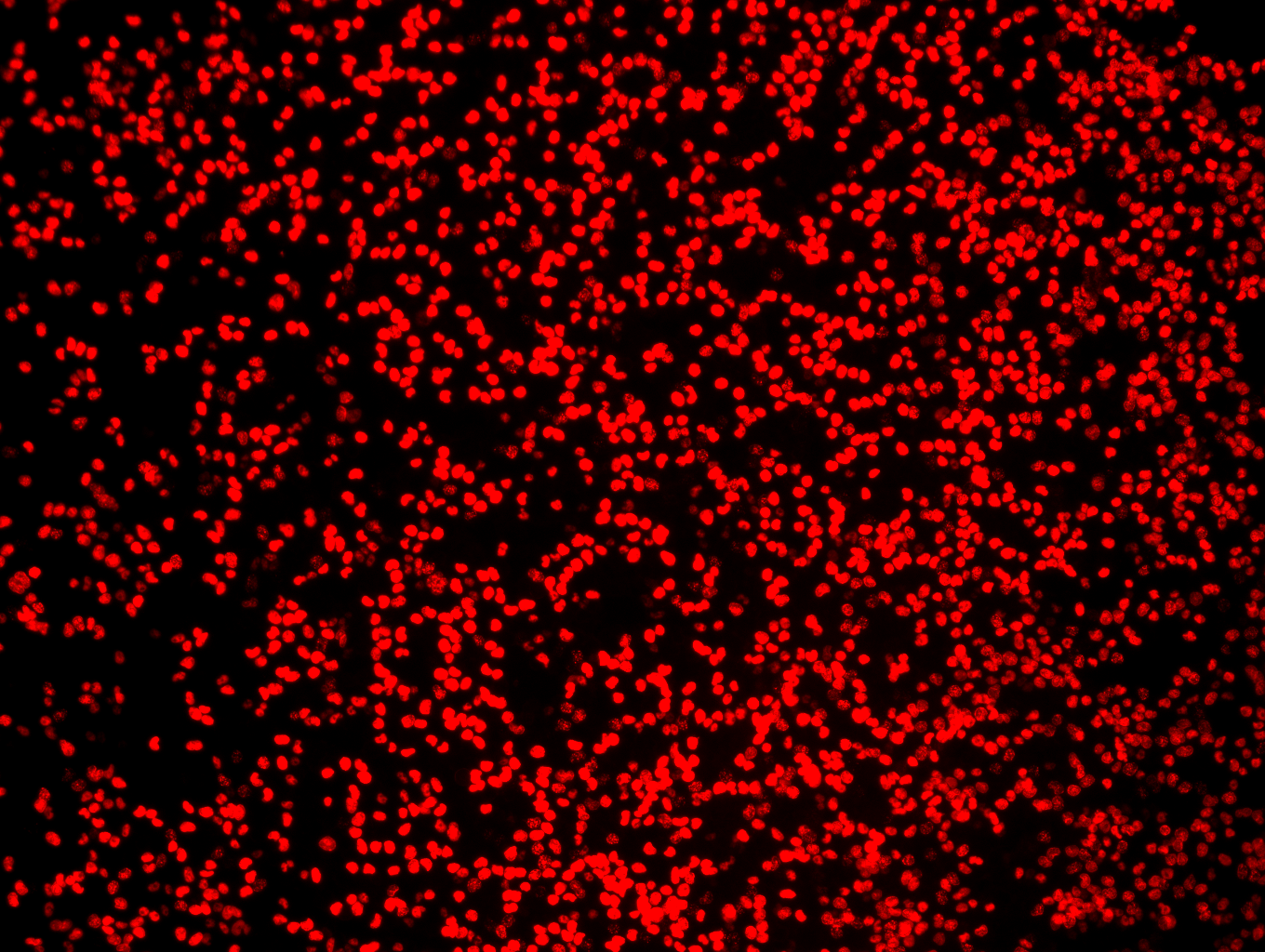

Supplement: Supplementary file 12 — Source data Fig. 7 [file 44318_2024_359_MOESM12_ESM.zip › Figure 7/Fig 7G/ΔATG1+2/Edu.tif]

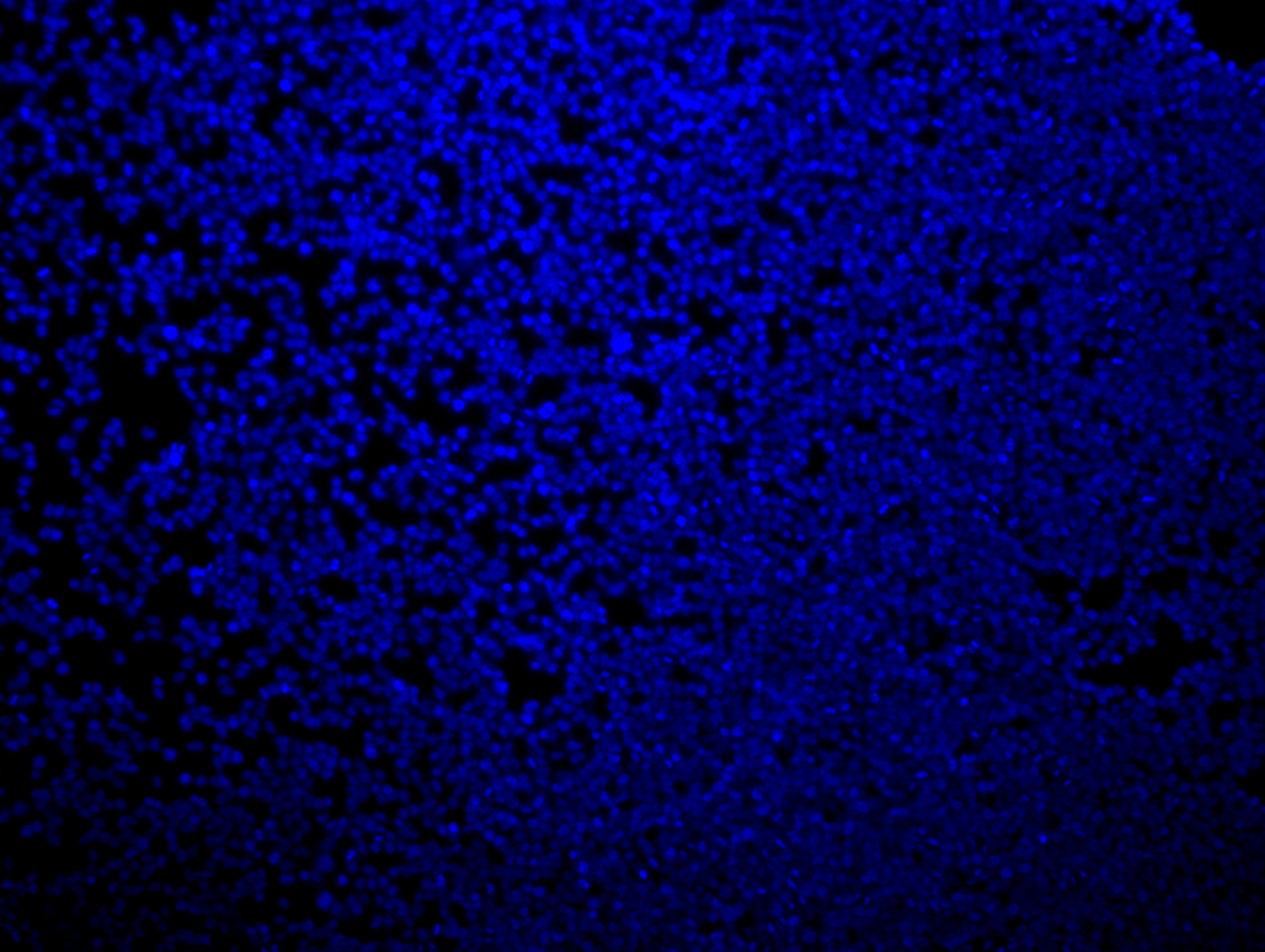

Supplement: Supplementary file 12 — Source data Fig. 7 [file 44318_2024_359_MOESM12_ESM.zip › Figure 7/Fig 7G/ΔATG1+2/Hoechst.tif]

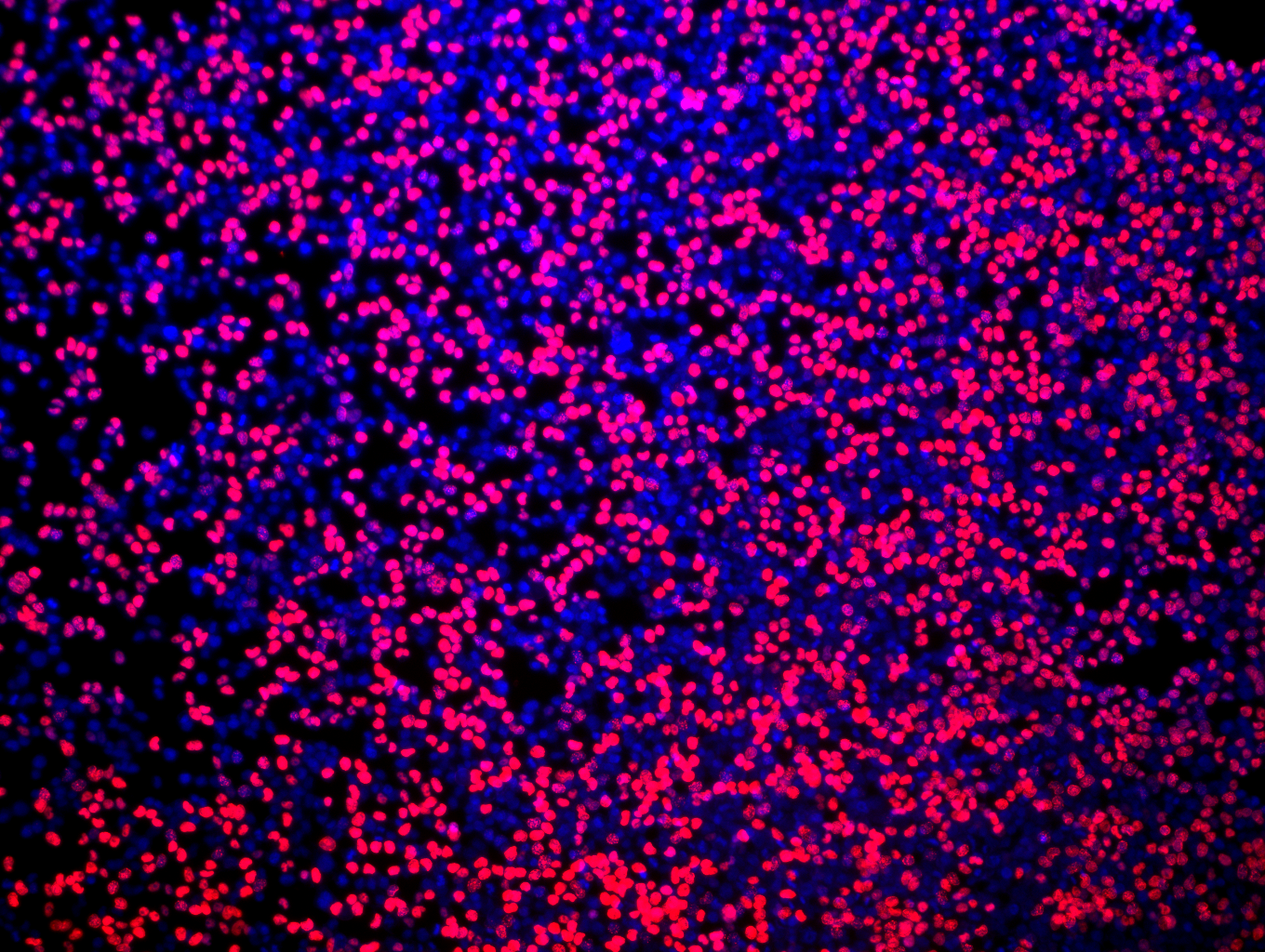

Supplement: Supplementary file 12 — Source data Fig. 7 [file 44318_2024_359_MOESM12_ESM.zip › Figure 7/Fig 7G/ΔATG1+2/merge.tif]

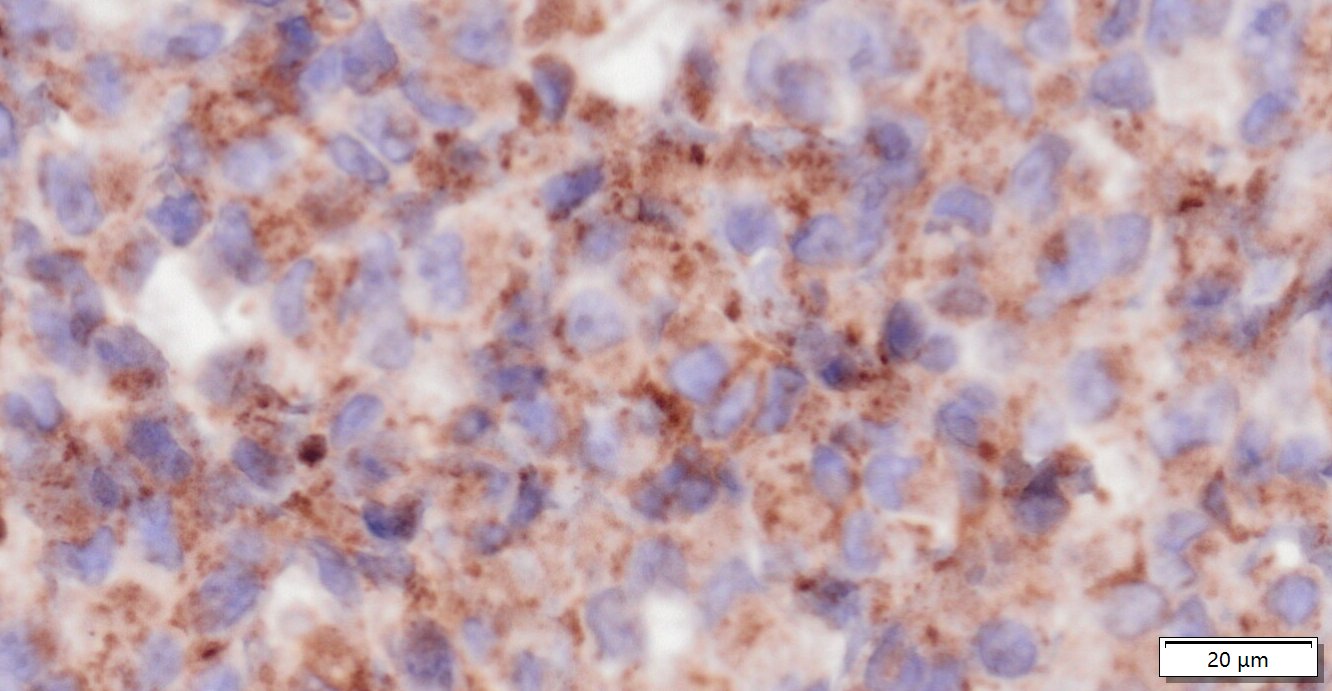

Supplement: Supplementary file 12 — Source data Fig. 7 [file 44318_2024_359_MOESM12_ESM.zip › Figure 7/Fig 7N/TAT-hSPAR/#6.jpg]

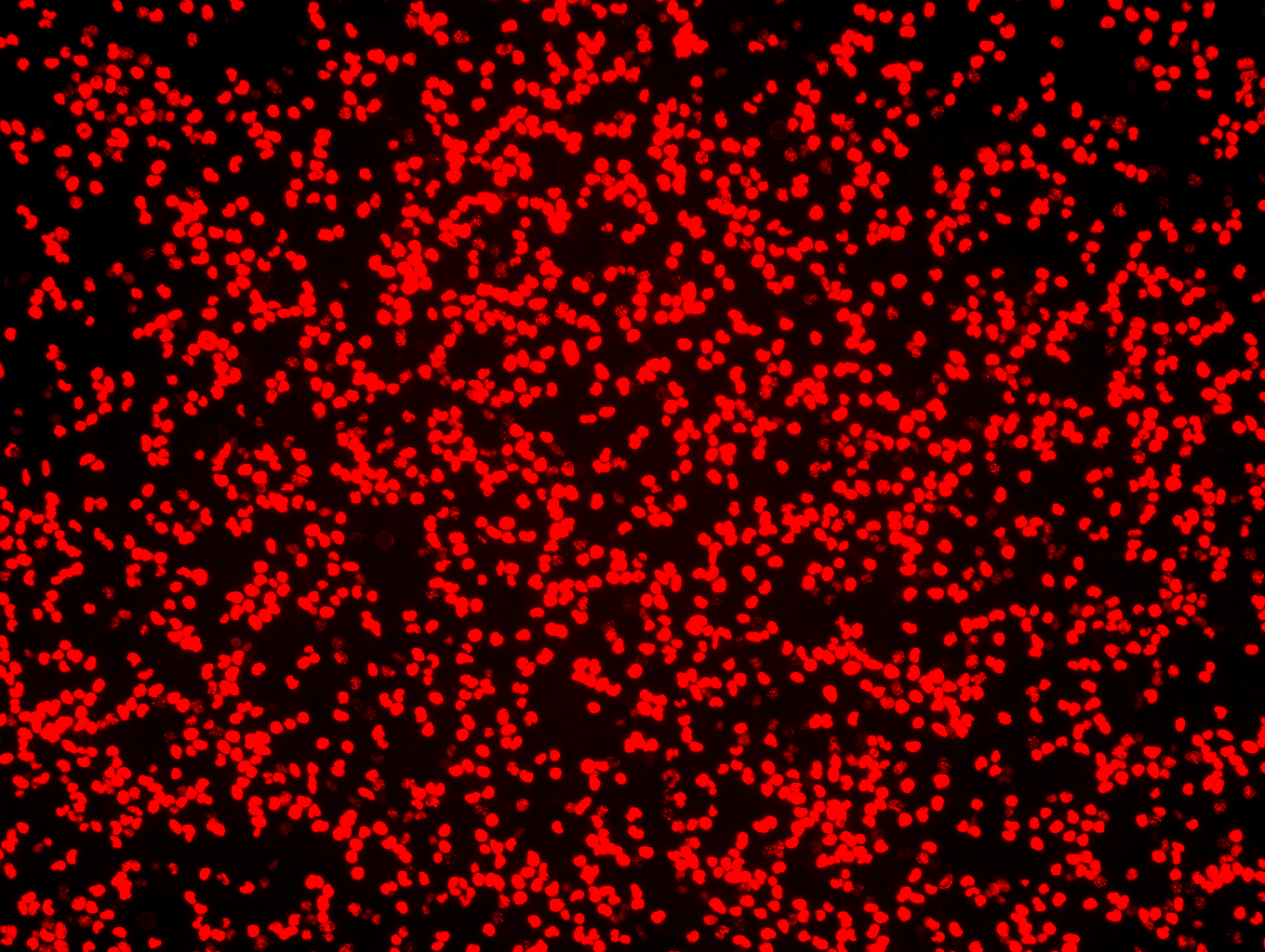

Supplement: Supplementary file 12 — Source data Fig. 7 [file 44318_2024_359_MOESM12_ESM.zip › Figure 7/Fig 7H/TAT/Edu.tif]

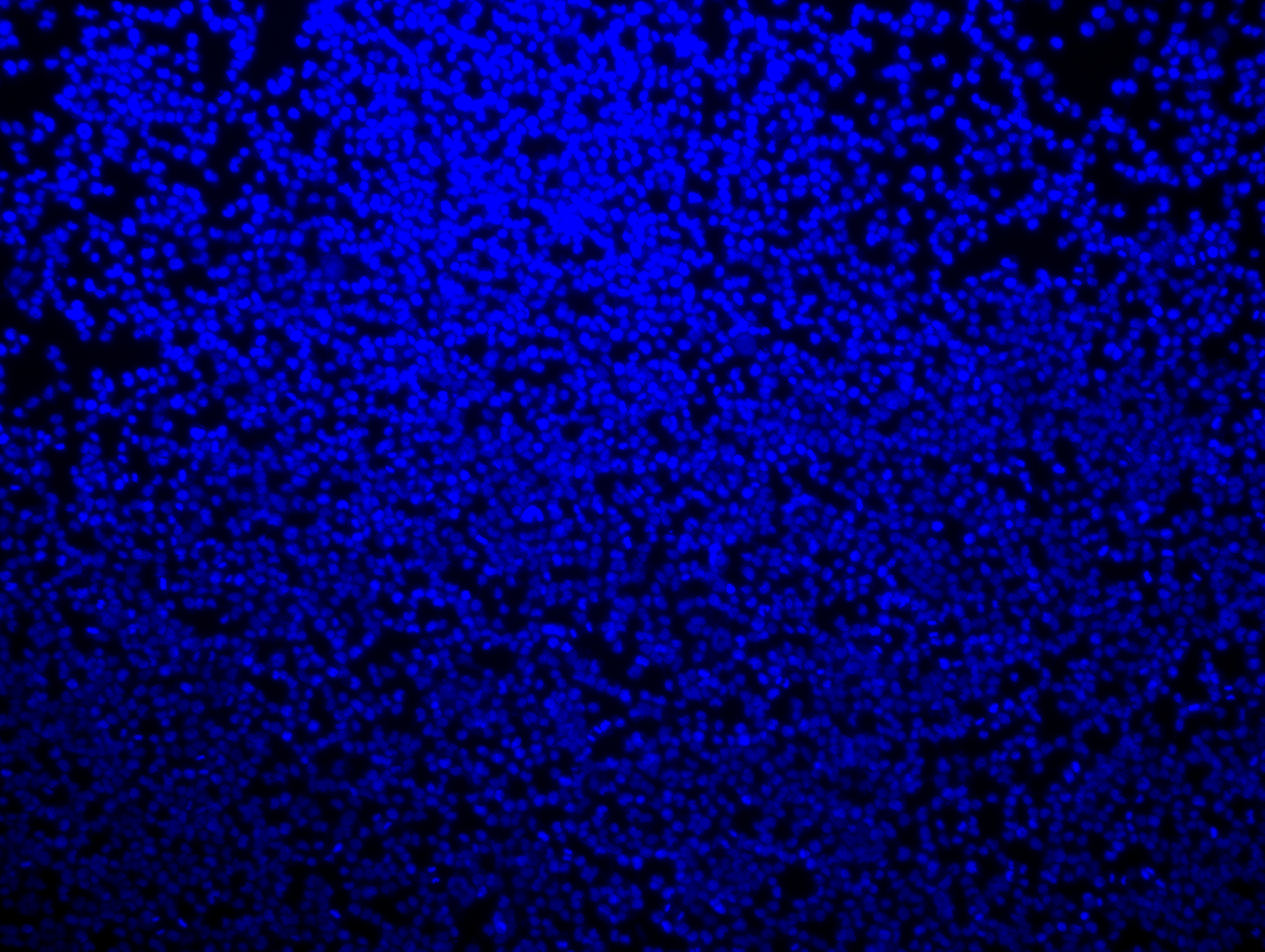

Supplement: Supplementary file 12 — Source data Fig. 7 [file 44318_2024_359_MOESM12_ESM.zip › Figure 7/Fig 7H/TAT/Hoechst.tif]

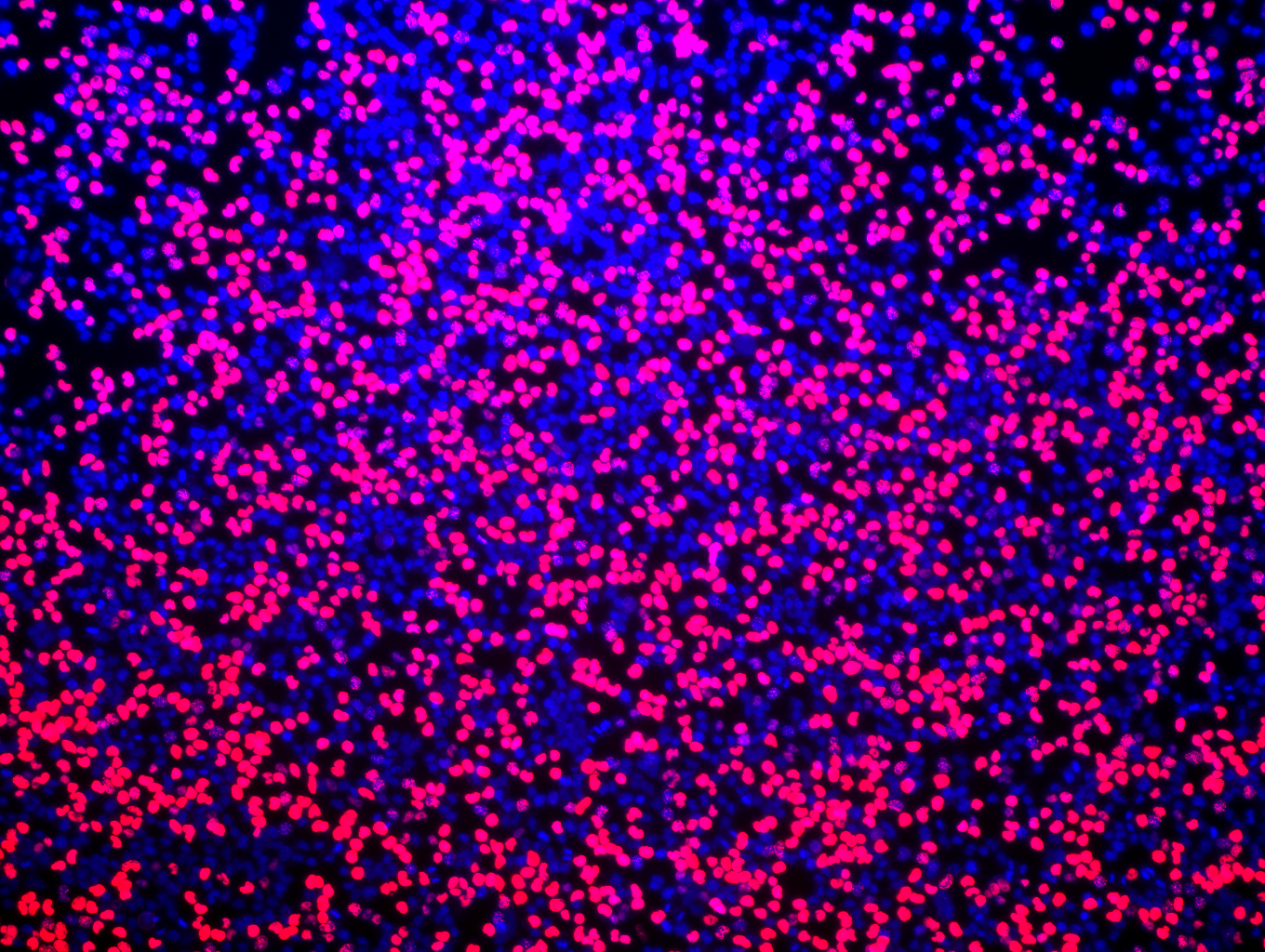

Supplement: Supplementary file 12 — Source data Fig. 7 [file 44318_2024_359_MOESM12_ESM.zip › Figure 7/Fig 7H/TAT/merge.tif]

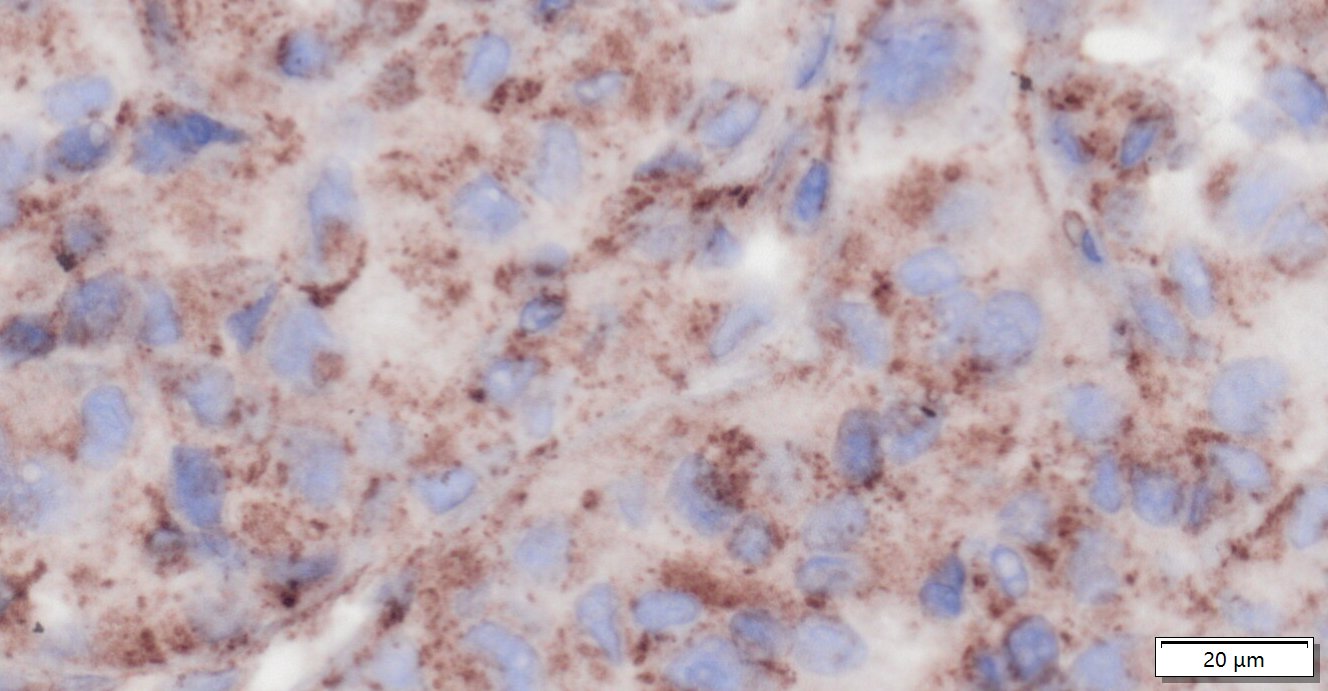

Supplement: Supplementary file 12 — Source data Fig. 7 [file 44318_2024_359_MOESM12_ESM.zip › Figure 7/Fig 7N/TAT-hSPAR/#5.jpg]

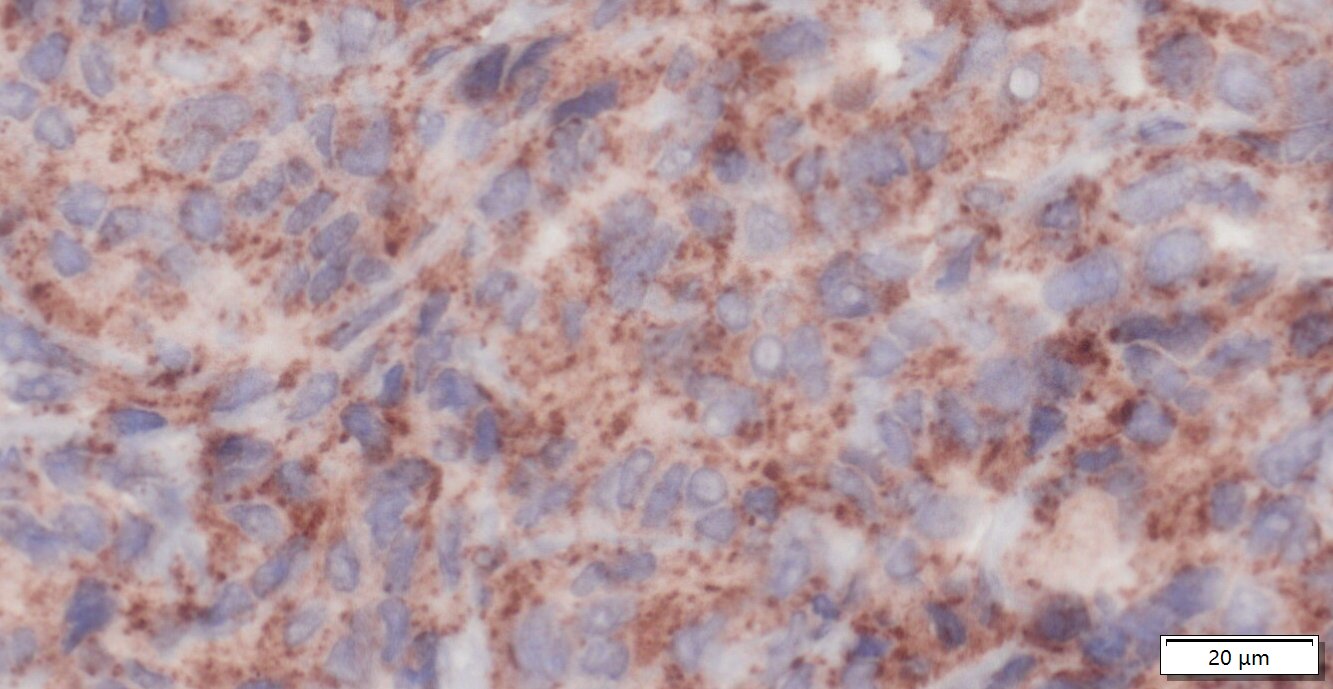

Supplement: Supplementary file 12 — Source data Fig. 7 [file 44318_2024_359_MOESM12_ESM.zip › Figure 7/Fig 7N/TAT-hSPAR/#4.jpg]

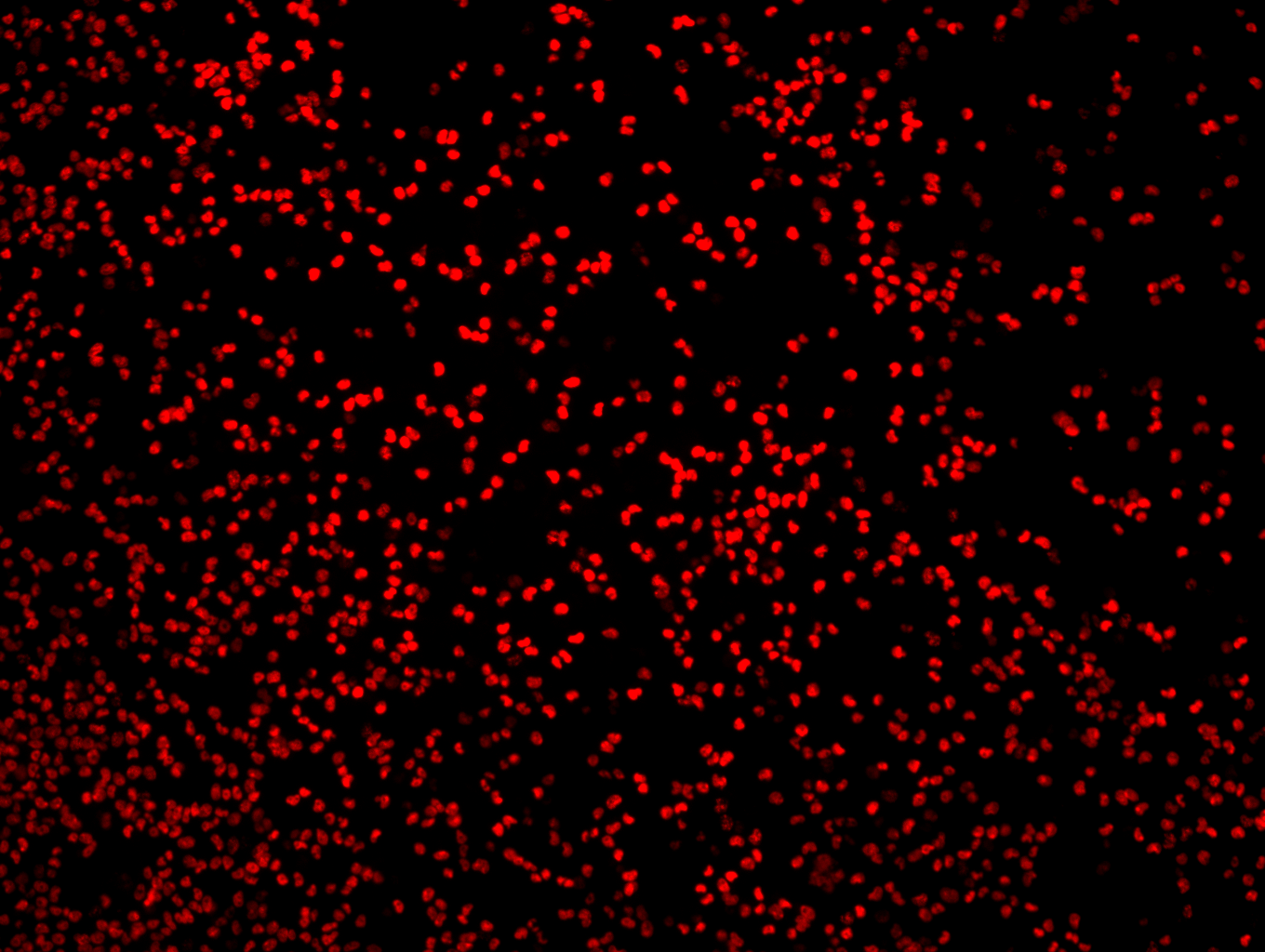

Supplement: Supplementary file 12 — Source data Fig. 7 [file 44318_2024_359_MOESM12_ESM.zip › Figure 7/Fig 7H/TAT-hSPAR_C/Edu.tif]

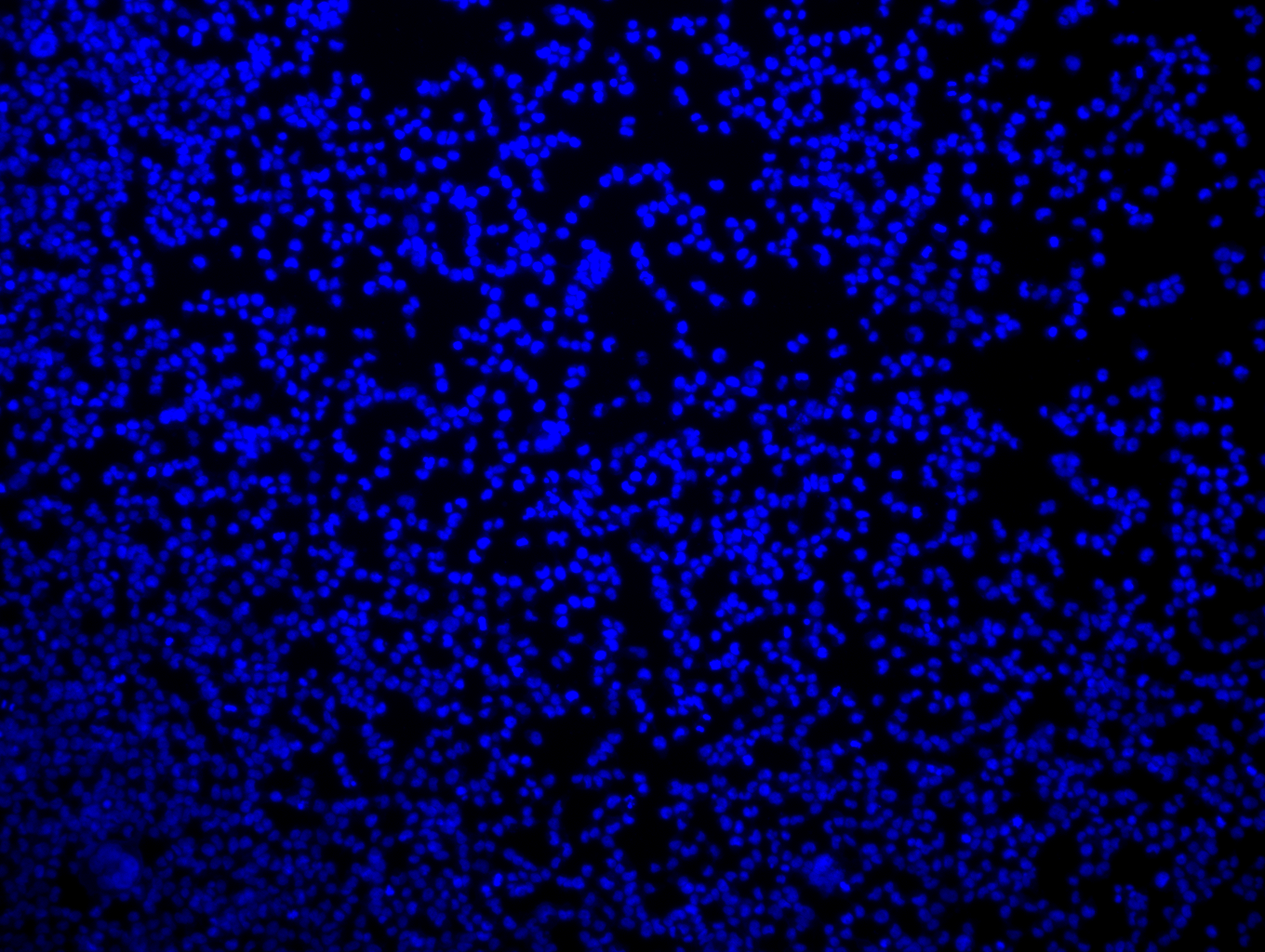

Supplement: Supplementary file 12 — Source data Fig. 7 [file 44318_2024_359_MOESM12_ESM.zip › Figure 7/Fig 7H/TAT-hSPAR_C/Hoechst.tif]

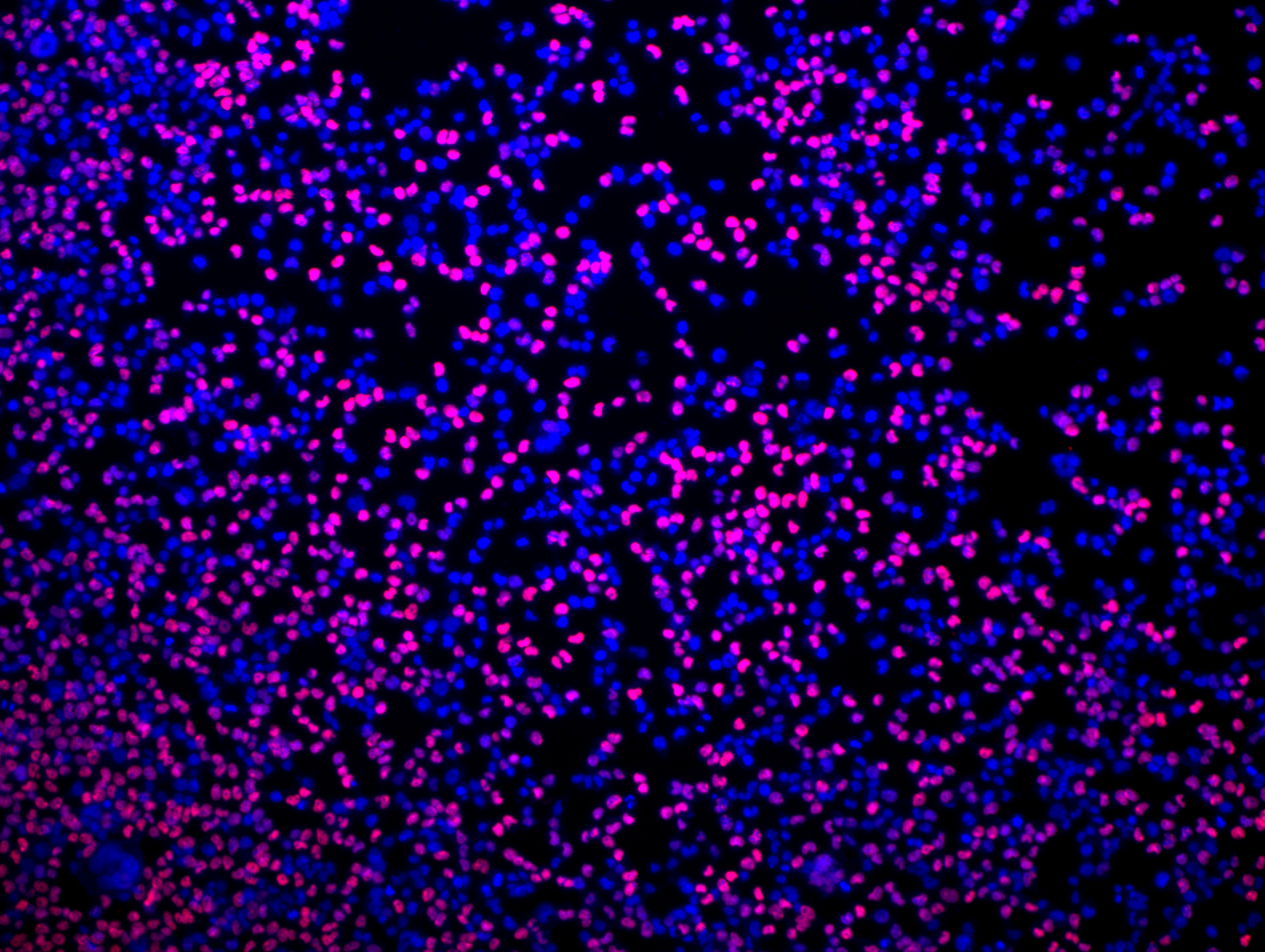

Supplement: Supplementary file 12 — Source data Fig. 7 [file 44318_2024_359_MOESM12_ESM.zip › Figure 7/Fig 7H/TAT-hSPAR_C/merge.tif]

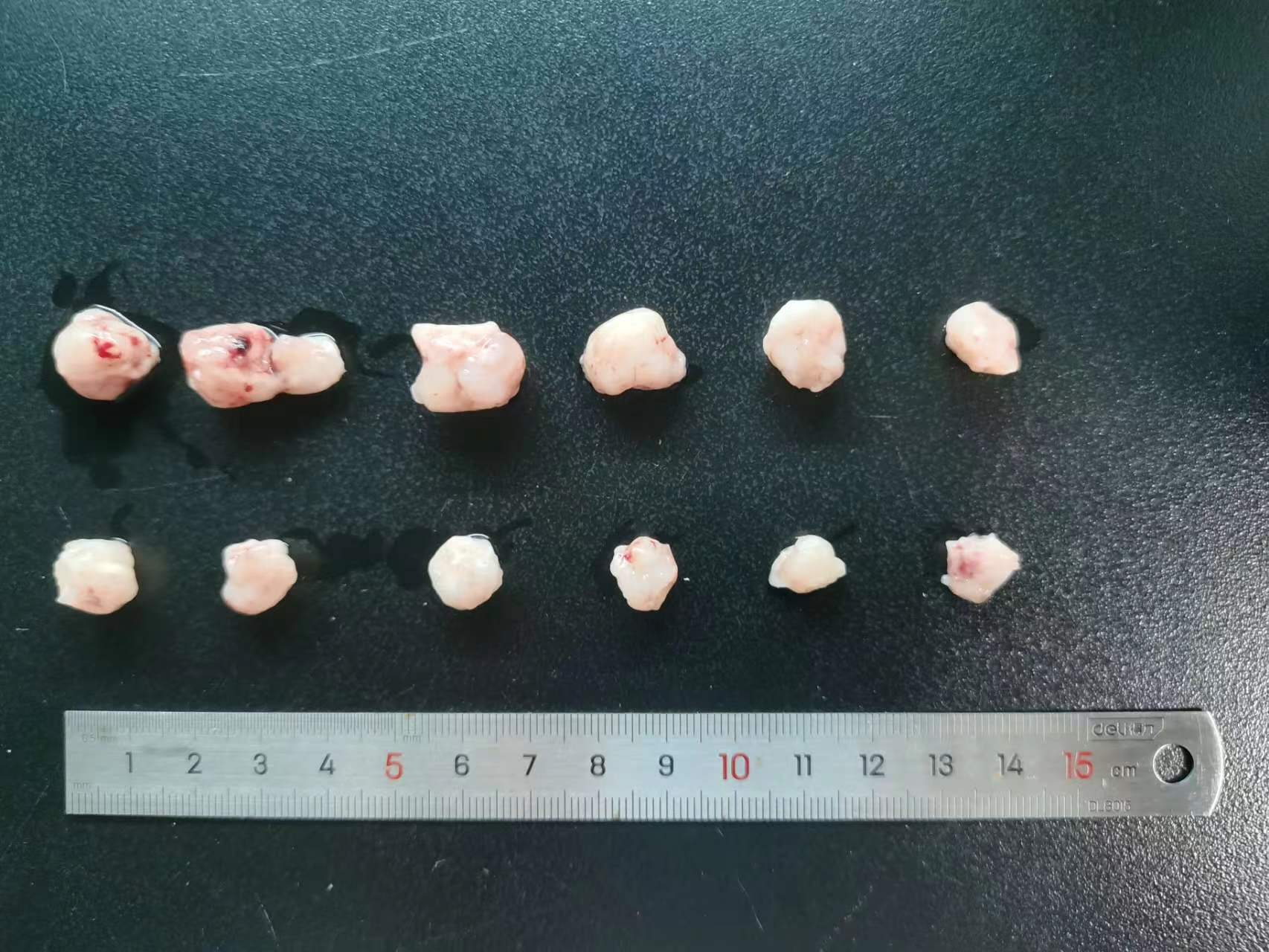

Supplement: Supplementary file 12 — Source data Fig. 7 [file 44318_2024_359_MOESM12_ESM.zip › Figure 7/Fig 7I/Fig 7I.jpg]

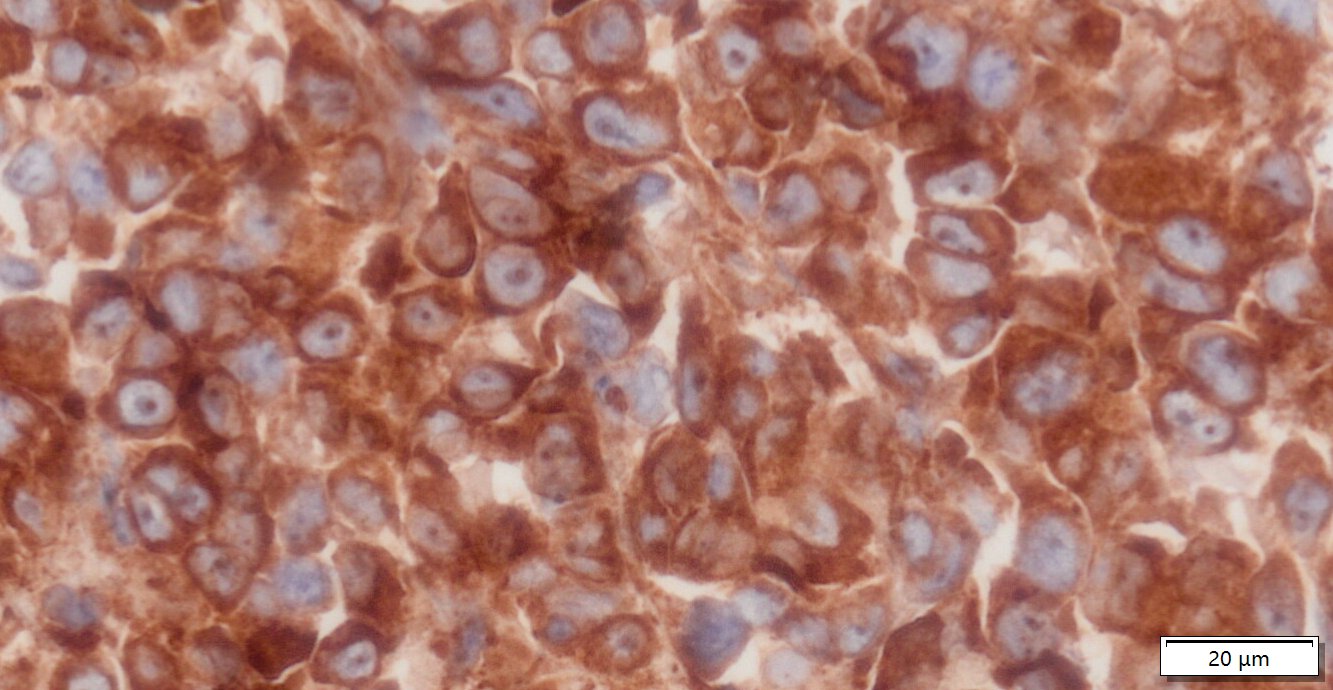

Supplement: Supplementary file 12 — Source data Fig. 7 [file 44318_2024_359_MOESM12_ESM.zip › Figure 7/Fig 7M/TAT ctrl/#1.jpg]

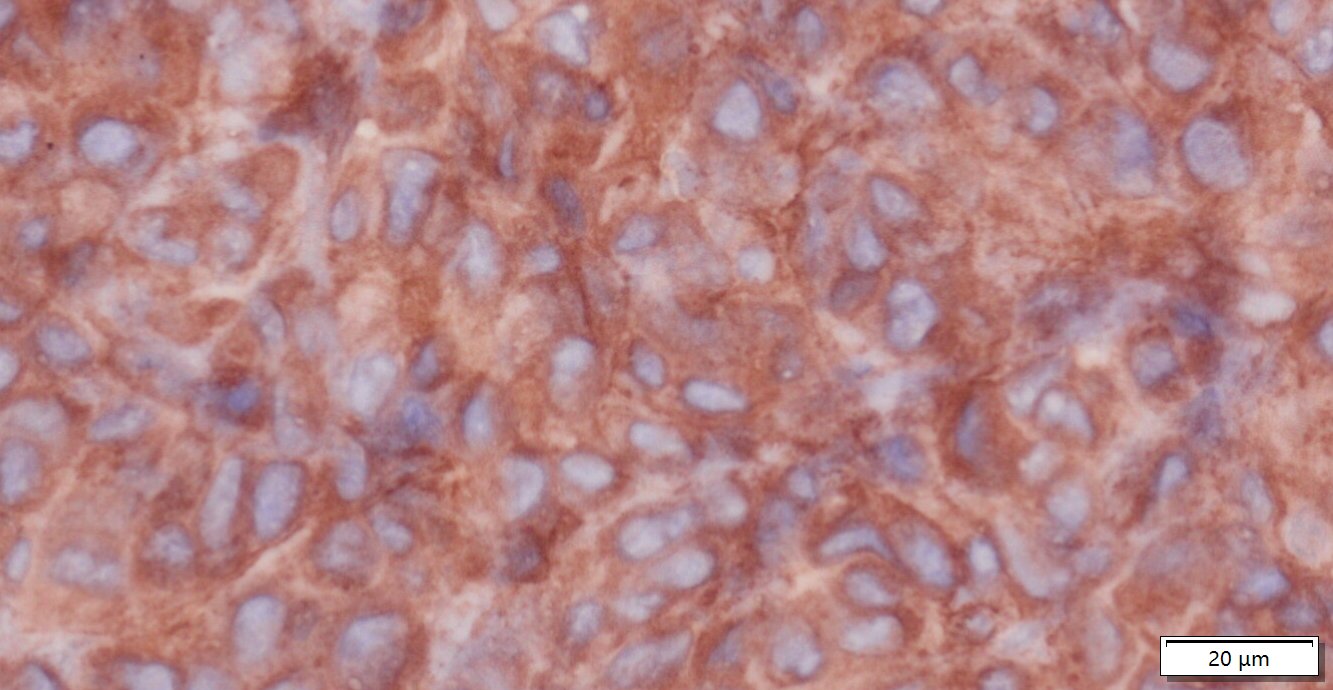

Supplement: Supplementary file 12 — Source data Fig. 7 [file 44318_2024_359_MOESM12_ESM.zip › Figure 7/Fig 7M/TAT ctrl/#2.jpg]

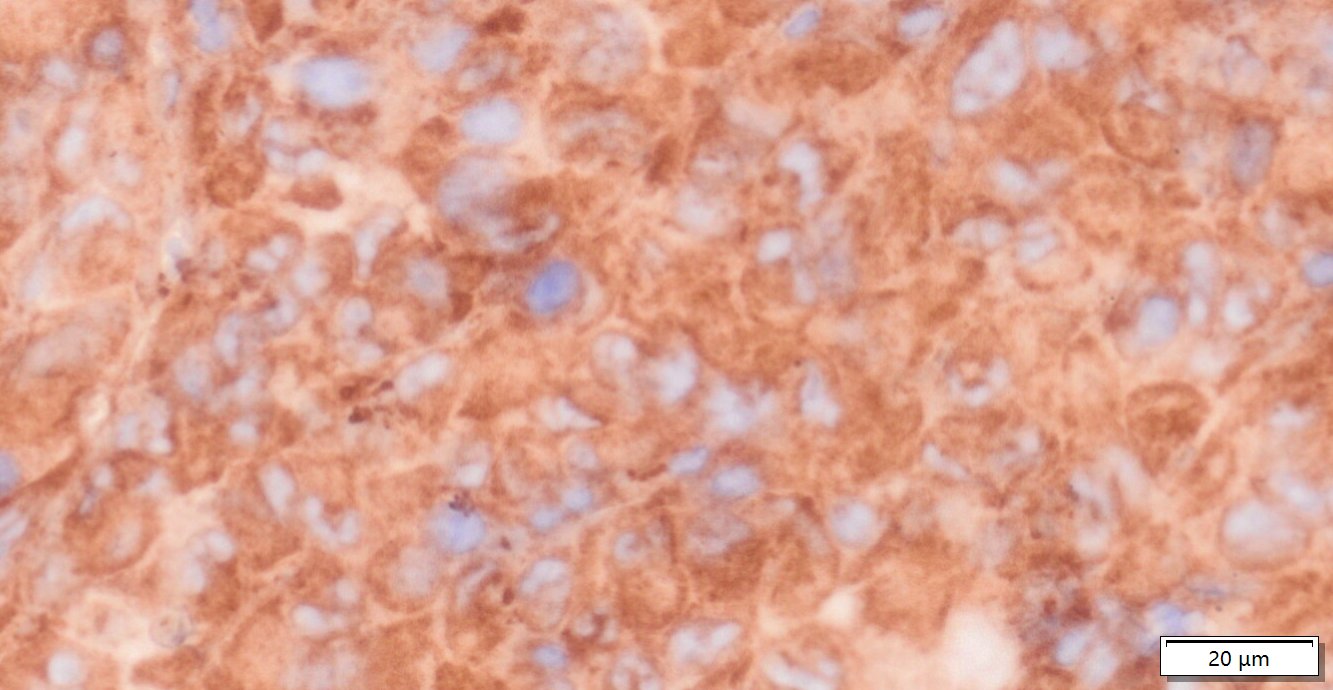

Supplement: Supplementary file 12 — Source data Fig. 7 [file 44318_2024_359_MOESM12_ESM.zip › Figure 7/Fig 7M/TAT ctrl/#3.jpg]

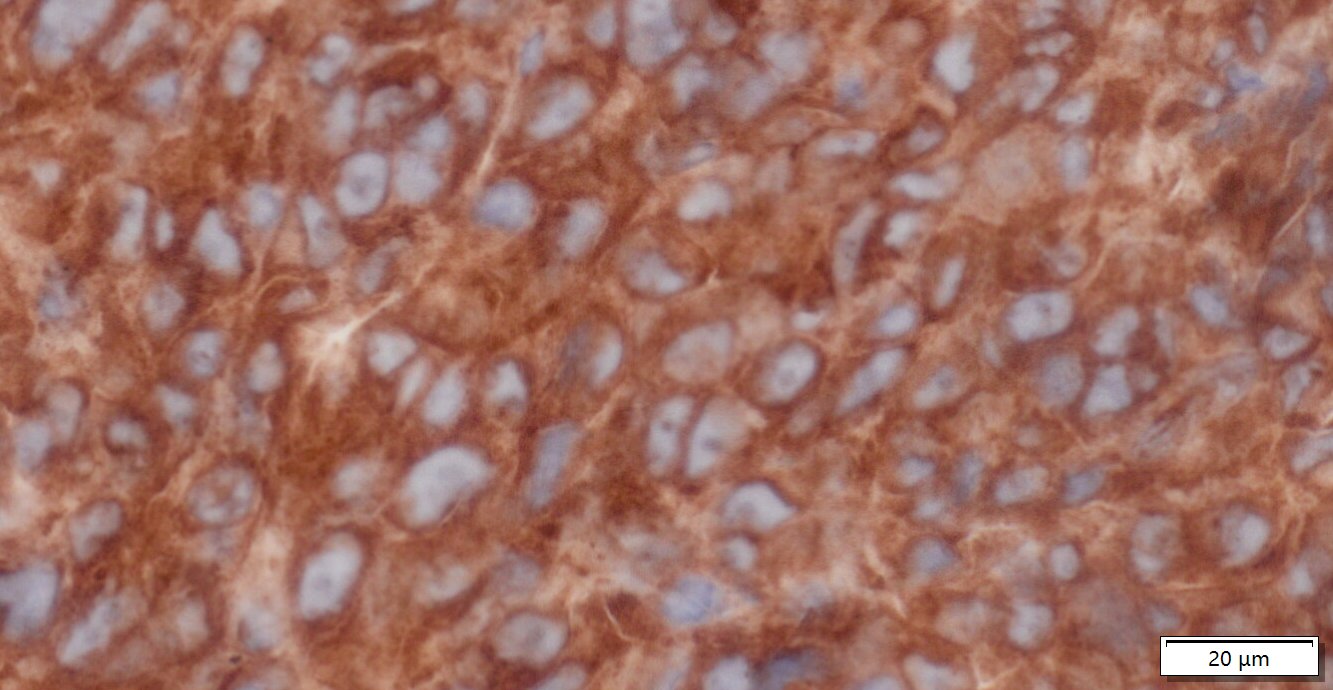

Supplement: Supplementary file 12 — Source data Fig. 7 [file 44318_2024_359_MOESM12_ESM.zip › Figure 7/Fig 7M/TAT ctrl/#4.jpg]
