# Supplementary material for: Micropeptide hSPAR regulates glutamine levels and suppresses mammary tumor growth via a TRIM21-P27KIP1-mTOR axis
Source: EMBO J. 2025 Jan 28;44(5):1414–41. doi: 10.1038/s44318-024-00359-z (PMC11876615; doi:10.1038/s44318-024-00359-z)
Supplement: Supplementary file 14 — Expanded View Figures [file 44318_2024_359_MOESM14_ESM.pdf]

Expanded View Figures

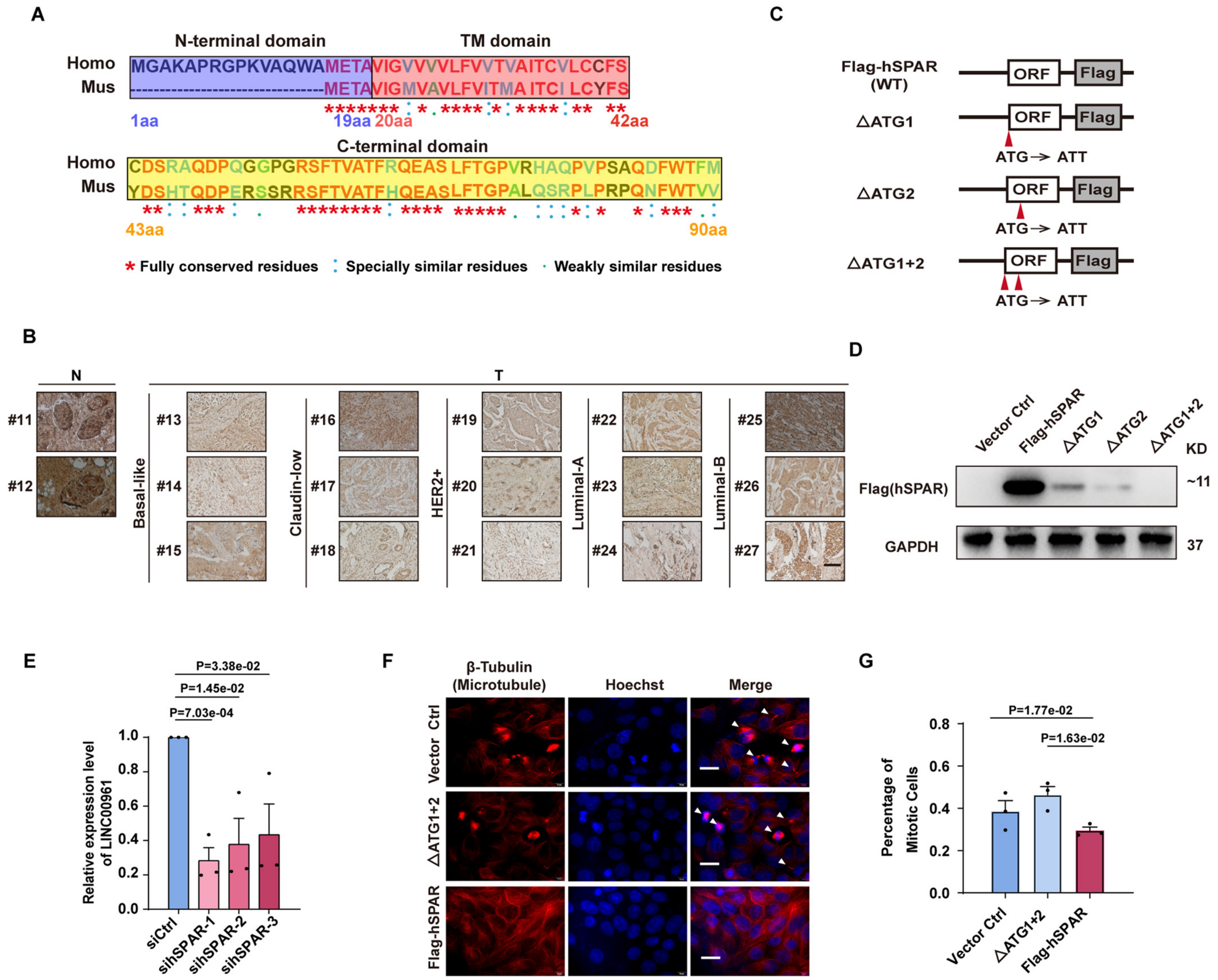

Figure EV1. Related to Fig. 1. The micropeptide hSPAR acts as a tumor suppressor independently of its parental lncRNA *LINC00961* in breast cancer.

(**A**) The fully conserved residues are indicated by red '*', the specially similar residues are indicated by blue ':', and the weakly similar residues are indicated by green '.'. N-terminal domain is highlighted with blue box, TM domain is highlighted with red box, C-terminal domain is highlighted with yellow box. (**B**) The expression levels of hSPAR were detected by immunohistochemistry in the breast cancer tissues (including triple-negative (Basal-like and Claudin-low), HER2 + , Luminal-A and Luminal-B) (*n* = 15 independent biological samples) and non-tumoral tissues (*n* = 2 independent biological samples). Scale bar, 20 μm. (**C**) Diagram of Flag-tagged hSPAR and three translation defective constructs (ΔATG1, ΔATG2, ΔATG1 + 2). (**D**) Immunoblotting against Flag in extracts from MDA-MB-231 cells transfected with Vector Ctrl, ΔATG1, ΔATG2, ΔATG1 + 2, or Flag-hSPAR. GAPDH, loading control (*n* = 3 independent biological samples). (**E**) Knockdown efficiency of *LINC00961* analyzed by qPCR in MDA-MB-231 cells (*n* = 3 independent biological samples). Data are presented as the mean ± SEM and analyzed using one-way ANOVA with Dunnett' multiple comparisons test. (**F**) Immunofluorescence staining of MDA-MB-231 cells transfected with the indicated constructs for β-Tubulin (red). Nuclei were stained with Hoechst (blue). The white arrow heads indicate cells undergoing division (*n* = 3 independent biological samples). Scale bar, 10 μm. (**G**) Quantification of the cell number in mitosis phase from panel (**F**) (*n* = 3 independent biological samples). Data are presented as the mean ± SEM and analyzed using one-way ANOVA with Dunnett' multiple comparisons test. Source data are available online for this figure.

© The Author(s)

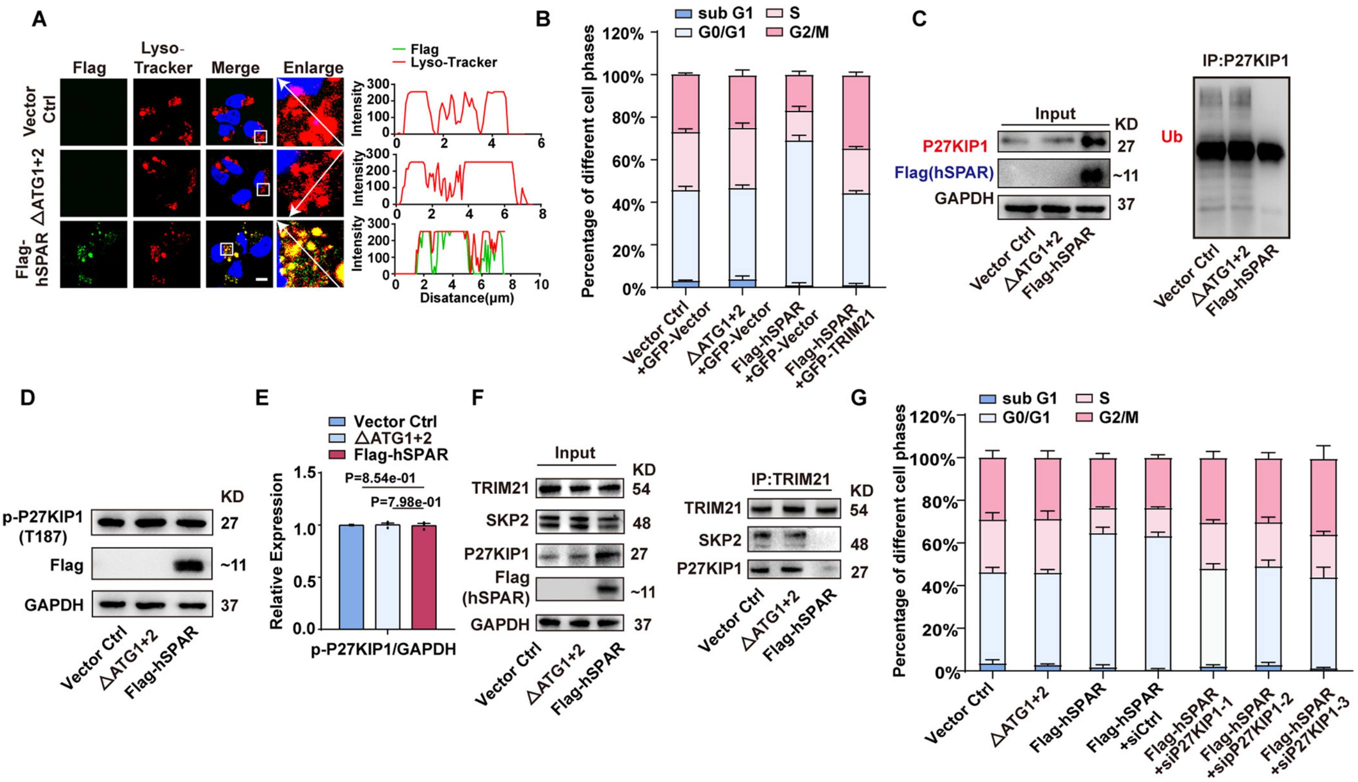

Figure EV2. Related to Figs. 2, 3, and 4. Lysosome localized hSPAR triggers G1-S arrest in MDA-MB-231 cells in TRIM21- and P27KIP-dependent manner.

(**A**) Co-immunofluorescence staining of Flag (green) and Lyso-Tracker (red) in MDA-MB-231 cells transfected with the indicated constructs. Nuclei were stained with Hoechst (blue). The graphs display the fluorescence intensity (arbitrary units) of Flag and Lyso-Tracker over the distance from adjacent image (depicted by the arrows). Scale bar, 5 μm ($n = 3$ independent biological samples). (**B**) Changes of cell ratio at different cell cycle phases in the presence of indicated controls and Flag-hSPAR with or without GFP-TRIM21 ($n = 3$ independent biological samples). Data are presented as the mean ± SEM. (**C**) Changes of the ubiquitination level of P27KIP1 were detected by Co-IP and immunoblotting from MDA-MB-231 cells transfected with Vector Ctrl, ΔATG1 + 2 or Flag-hSPAR. Left, immunoblotting of inputs. Right, immunoblotting using antibody against ubiquitin following IP of P27KIP1 ($n = 3$ independent biological samples). (**D**) Immunoblotting against p-P27KIP1(T187), Flag and GAPDH in extracts from MDA-MB-231 cells transfected with Vector Ctrl, ΔATG1 + 2, or Flag-hSPAR ($n = 3$ independent biological samples). (**E**) Quantified relative levels of p-P27KIP1(T187)/GAPDH from panel (**D**) ($n = 3$ independent biological samples). Data are presented as the mean ± SEM and analyzed using one-way ANOVA with Dunnett' multiple comparisons test. (**F**) Interaction of TRIM21 with SKP2 and P27KIP1 detected by Co-IP and immunoblotting from MDA-MB-231 cells transfected with the indicated constructs. Left, immunoblotting of inputs. Right, immunoblotting using antibodies against TRIM21, SKP2 and P27KIP1 following IP of TRIM21 ($n = 3$ independent biological samples). (**G**) Changes of cell ratio at different cell cycle phases in the presence of indicated controls and Flag-hSPAR with or without P27KIP1 knockdown ($n = 3$ independent biological samples). Data are presented as the mean ± SEM. Source data are available online for this figure.

 © The Author(s)

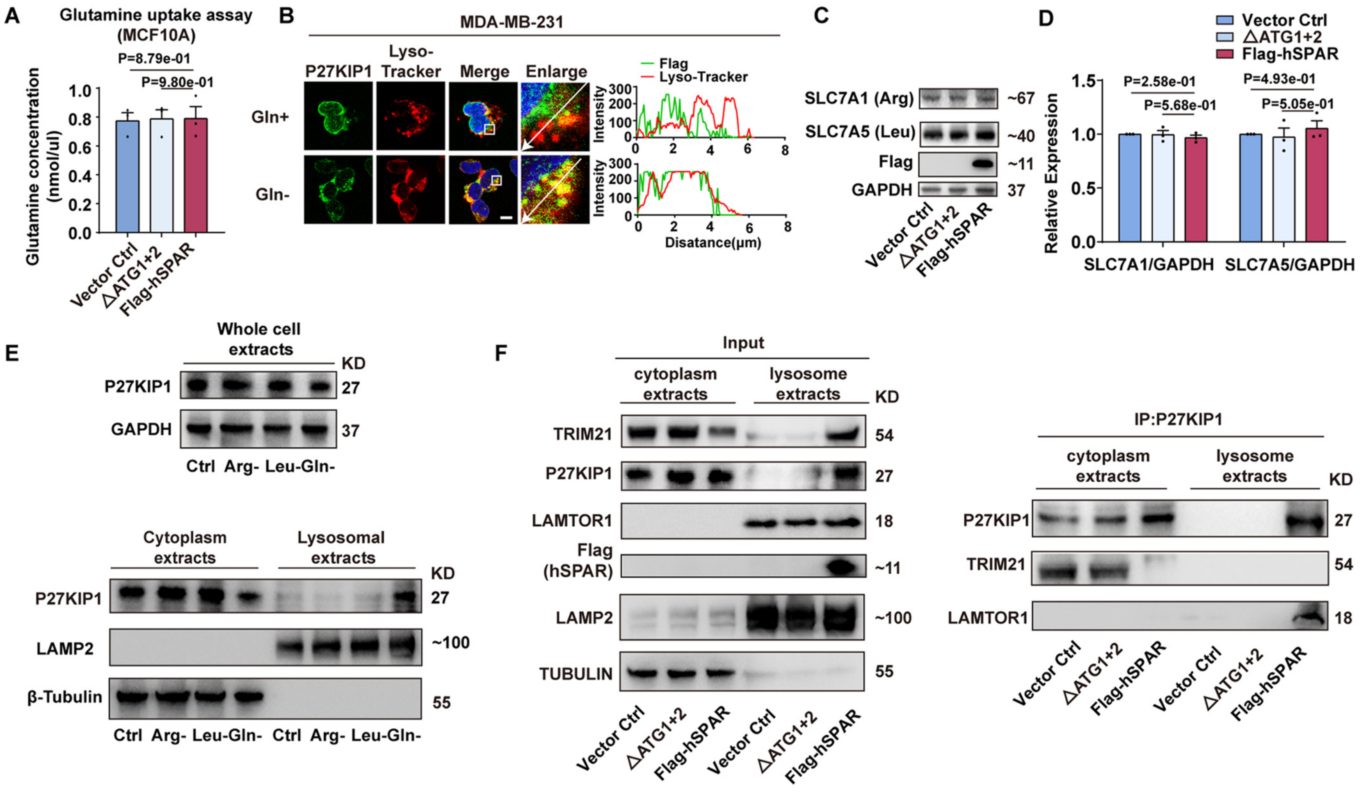

Figure EV3. Related to Figs. 5 and 6. The arginine and leucine not associated with the hSPAR-TRIM21-P27KIP1-mTOR pathway in MDA-MB-231 cells.

(**A**) Levels of glutamine in MCF10A cells transfected with Vector Ctrl, ΔATG1 + 2 or Flag-hSPAR ($n = 3$ independent biological samples). Data are presented as the mean ± SEM and analyzed using one-way ANOVA with Dunnett' multiple comparisons test. (**B**) Co-immunofluorescence staining of P27KIP1 (green) and Lyso-Tracker (red) in MDA-MB-231 cells cultured with or without glutamine. Cells were permeabilized with digitonin to remove the soluble P27KIP1. Nuclei were stained with Hoechst (blue). The graphs display the fluorescence intensity (arbitrary units) of P27KIP1 and Lyso-Tracker over the distance from adjacent image (depicted by the arrows). Scale bar, 5 µm ($n = 3$ independent biological samples). (**C**) Immunoblotting against SLC7A1, SLC7A5, Flag and GAPDH in extracts from MDA-MB-231 cells transfected with Vector Ctrl, ΔATG1 + 2, or Flag-hSPAR ($n = 3$ independent biological samples). (**D**) Quantified relative levels of SLC7A1/GAPDH and SLC7A5/GAPDH from panel (**C**) ($n = 3$ independent biological samples). Data are presented as the mean ± SEM and analyzed using one-way ANOVA with Dunnett' multiple comparisons test.
(**E**) Immunoblotting of whole-cell extracts (upper panel), cytoplasmic (lysosome components removed) and lysosomal extracts (lower panel) prepared from MDA-MB-231 cells cultured with or without arginine, leucine or glutamine against P27KIP1, GAPDH, LAMP2 (lysosomal marker) and β-Tubulin (cytoplasmic marker) ($n = 3$ independent biological samples). (**F**) Changes of interaction between P27KIP1 and TRIM21, P27KIP1 and LAMTOR1 were detected by Co-IP and immunoblotting in the indicated fractions extracts from MDA-MB-231 cells after transfection with the indicated constructs. Left, immunoblotting of inputs. Right, immunoblotting using antibodies against P27KIP1, TRIM21 and LAMTOR1 following IP of P27KIP1 ($n = 3$ independent biological samples). Source data are available online for this figure.

© The Author(s)

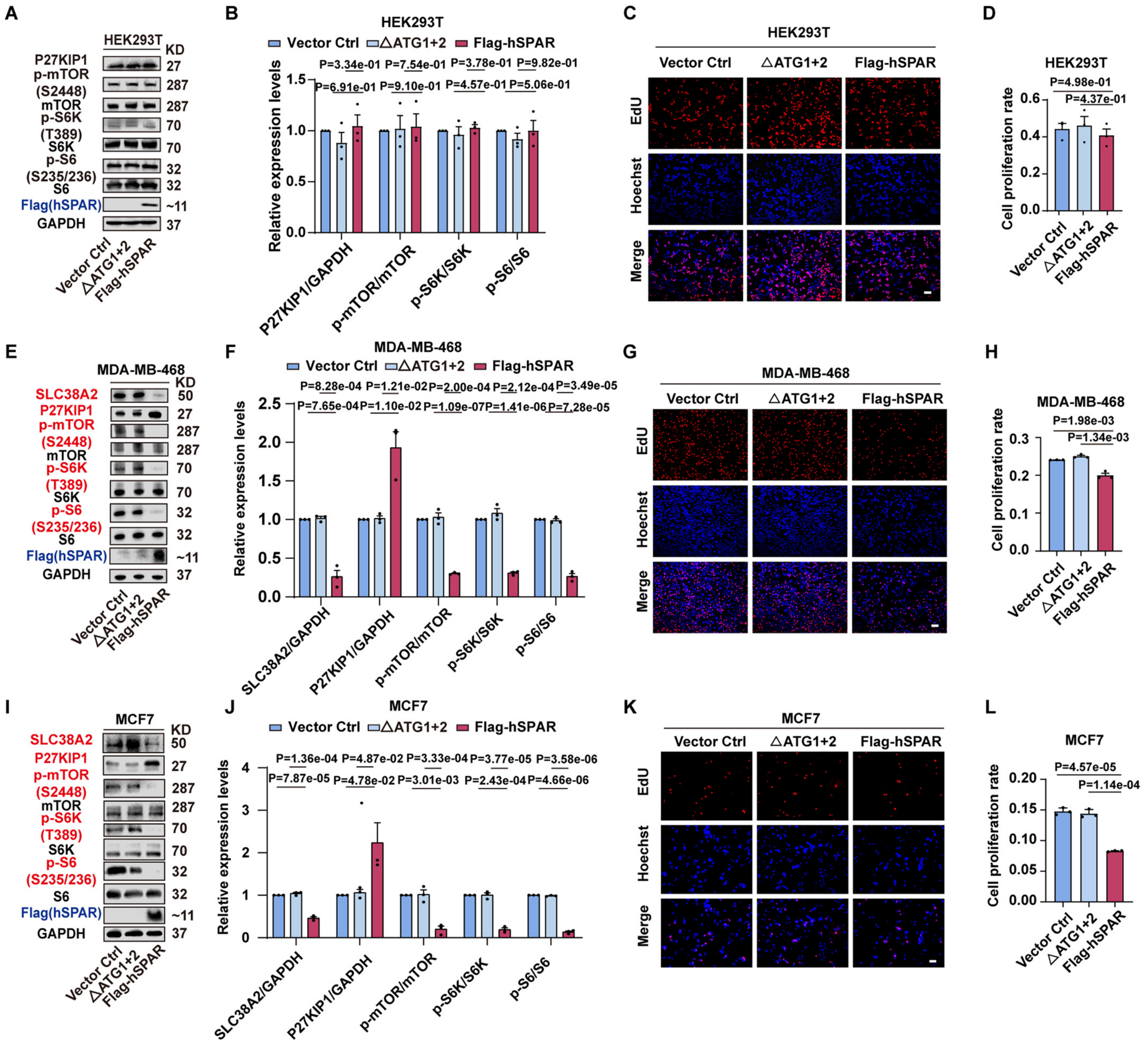

Figure EV4. Related to Fig. 6. hSPAR inhibits P27KIP1-regulated mTOR signaling and cell proliferation in MDA-MB-468 and MCF7 cells, but not in HEK293T cells.

(**A**) Immunoblotting against P27KIP1, p-mTOR, mTOR, p-S6K, S6K, p-S6, S6, Flag and GAPDH for extracts from HEK293T cells transfected with Vector Ctrl, ΔATG1 + 2 or Flag-hSPAR ($n = 3$ independent biological samples). (**B**) Quantified relative levels of P27KIP1/GAPDH, p-mTOR/mTOR, p-S6K/S6K and p-S6/S6 from panel (**A**) ($n = 3$ independent biological samples). Data are presented as the mean ± SEM and analyzed using one-way ANOVA with Dunnett' multiple comparisons test. (**C**) Representative images of EdU assay HEK293T cells transfected with Vector Ctrl, ΔATG1 + 2 or Flag-hSPAR ($n = 3$ independent biological samples). Scale bar, 50 µm. (**D**) Quantification of cell proliferation rate from panel (**C**) ($n = 3$ independent biological samples). Data are presented as the mean ± SEM and analyzed using one-way ANOVA with Dunnett' multiple comparisons test. (**E**) Immunoblotting against SLC38A2, P27KIP1, p-mTOR, mTOR, p-S6K, S6K, p-S6, S6, Flag and GAPDH for extracts from MDA-MB-468 cells transfected with Vector Ctrl, ΔATG1 + 2 or Flag-hSPAR ($n = 3$ independent biological samples). (**F**) Quantified relative levels of SLC38A2/GAPDH, P27KIP1/GAPDH, p-mTOR/mTOR, p-S6K/S6K and p-S6/S6 from panel (**E**) ($n = 3$ independent biological samples). Data are presented as the mean ± SEM and analyzed using one-way ANOVA with Dunnett' multiple comparisons test. (**G**) Representative images of EdU assay MDA-MB-468 cells transfected with Vector Ctrl, ΔATG1 + 2 or Flag-hSPAR ($n = 3$ independent biological samples). Scale bar, 50 µm. (**H**) Quantification of cell proliferation rate from panel (**G**) ($n = 3$ independent biological samples). Data are presented as the mean ± SEM and analyzed using one-way ANOVA with Dunnett' multiple comparisons test. (**I**) Immunoblotting against SLC38A2, P27KIP1, p-mTOR, mTOR, p-S6K, S6K, p-S6, S6, Flag and GAPDH for extracts from MCF7 cells transfected with Vector Ctrl, ΔATG1 + 2 or Flag-hSPAR ($n = 3$ independent biological samples). (**J**) Quantified relative levels of SLC38A2/GAPDH, P27KIP1/GAPDH, p-mTOR/mTOR, p-S6K/S6K and p-S6/S6 from panel (**I**) ($n = 3$ independent biological samples). Data are presented as the mean ± SEM and analyzed using one-way ANOVA with Dunnett' multiple comparisons test. (**K**) Representative images of EdU assay MCF7 cells transfected with Vector Ctrl, ΔATG1 + 2 or Flag-hSPAR ($n = 3$ independent biological samples). Scale bar, 50 µm. (**L**) Quantification of cell proliferation rate from panel (**K**) ($n = 3$ independent biological samples). Data are presented as the mean ± SEM and analyzed using one-way ANOVA with Dunnett' multiple comparisons test. The hSPAR-regulated proteins shown by immunoblotting are marked by red text. Source data are available online for this figure.

© The Author(s)

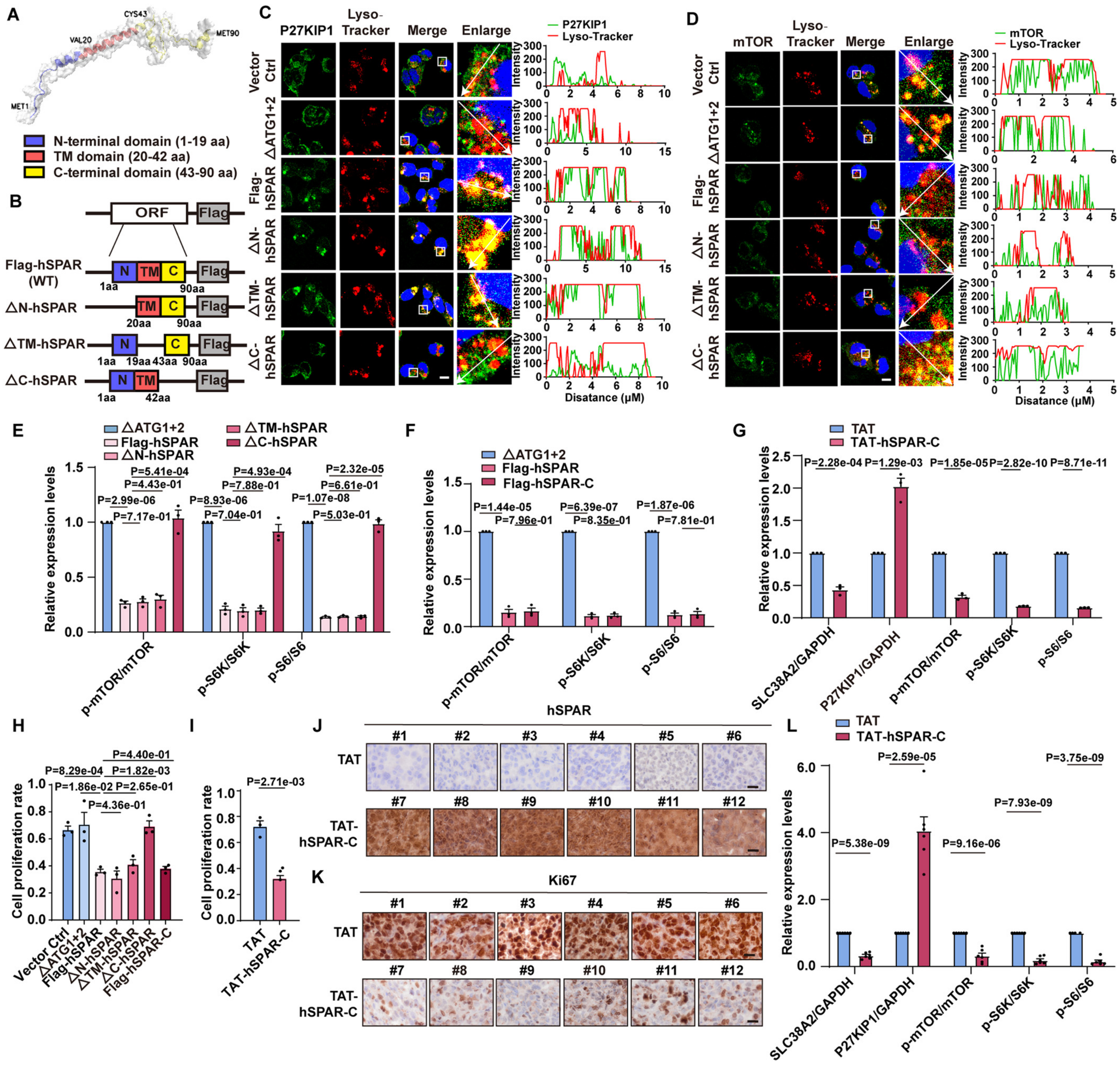
A
CYS43
VAL20
MET90
MET1
N-terminal domain (1-19 aa)
TM domain (20-42 aa)
C-terminal domain (43-90 aa)
B
ORF
Flag
Flag-hSPAR (WT)
△N-hSPAR
△TM-hSPAR
△C-hSPAR
1aa
90aa
20aa
19aa 43aa 90aa
42aa
C
P27KIP1
Lyso-Tracker
Merge
Enlarge
Vector Ctrl
△ATG1+2
Flag-hSPAR
△N-hSPAR
△TM-hSPAR
△C-hSPAR
Intensity
Disatance (μM)
D
mTOR
E
△ATG1+2
Flag-hSPAR
△N-hSPAR
△TM-hSPAR
△C-hSPAR
Relative expression levels
P=5.41e-04
P=4.43e-01
P=2.99e-06
P=7.17e-01
P=4.93e-04
P=7.88e-01
P=8.93e-06
P=7.04e-01
P=2.32e-05
P=6.61e-01
P=1.07e-08
P=5.03e-01
p-mTOR/mTOR
p-S6K/S6K
p-S6/S6
F
Flag-hSPAR-C
P=1.44e-05
P=7.96e-01
P=6.39e-07
P=8.35e-01
P=1.87e-06
P=7.81e-01
G
TAT
TAT-hSPAR-C
P=2.28e-04
P=1.29e-03
P=1.85e-05
P=2.82e-10
P=8.71e-11
SLC38A2/GAPDH
P27KIP1/GAPDH
H
Cell proliferation rate
P=4.40e-01
P=8.29e-04
P=1.82e-03
P=1.86e-02
P=2.65e-01
P=4.36e-01
Vector Ctrl
I
P=2.71e-03
J
hSPAR
#1 #2 #3 #4 #5 #6
#7 #8 #9 #10 #11 #12
K
Ki67
L
P=2.59e-05
P=3.75e-09
P=7.93e-09
P=5.38e-09
P=9.16e-06

Figure EV5. Related to Fig. 7. SPAR-C inhibits mTOR signaling, cell proliferation, and tumors growth in MDA-MB-231 xenograft mice.

(**A**) hSPAR is composed of an N-terminal domain, a transmembrane (TM) domain and a C-terminal domain as predicted by Alphafold2. (**B**) Diagram of Flag-tagged full-length hSPAR and three hSPAR domain deletion variants. (**C**) Co-immunofluorescence staining of P27KIP1 (green) and Lyso-Tracker (red) in MDA-MB-231 cells transfected with the indicated constructs. Cells were permeabilized with digitonin to remove the soluble P27KIP1. Nuclei were stained with Hoechst (blue). The graphs display the fluorescence intensity (arbitrary units) of P27KIP1 and Lyso-Tracker over the distance from adjacent image (depicted by the arrows). Scale bar, 5 µm ($n = 3$ independent biological samples). (**D**) Co-immunofluorescence staining of mTOR (green) and Lyso-Tracker (red) in MDA-MB-231 cells transfected with the indicated constructs. Cells were permeabilized with digitonin to remove the soluble mTOR. Nuclei were stained with Hoechst (blue). The graphs display the fluorescence intensity (arbitrary units) of mTOR and Lyso-Tracker over the distance from adjacent image (depicted by the arrows). Scale bar, 5 µm ($n = 3$ independent biological samples). (**E**) Quantified relative levels of p-mTOR/mTOR, p-S6K/S6K and p-S6/S6 from panel (Fig. 7D) ($n = 3$ independent biological samples). Data are presented as the mean ± SEM and analyzed using one-way ANOVA with Dunnett' multiple comparisons test. (**F**) Quantified relative levels of p-mTOR/mTOR, p-S6K/S6K and p-S6/S6 from panel (Fig. 7E) ($n = 3$ independent biological samples). Data are presented as the mean ± SEM and analyzed using one-way ANOVA with Dunnett' multiple comparisons test. (**G**) Quantified relative levels of SLC38A2/GAPDH, P27KIP1/GAPDH, p-mTOR/mTOR, p-S6K/S6K and p-S6/S6 from panel (Fig. 7F) ($n = 3$ independent biological samples). Data are presented as the mean ± SEM and analyzed using two-tailed Student's *t* test with Welch's correction. (**H**) Quantification of cell proliferation rate from panel (Fig. 7G) ($n = 3$ independent biological samples). Data are presented as the mean ± SEM and analyzed using one-way ANOVA with Dunnett' multiple comparisons test. (**I**) Quantification of cell proliferation rate from panel (Fig. 7H) ($n = 3$ independent biological samples). Data are presented as the mean ± SEM and analyzed using two-tailed Student's *t* test with Welch's correction. (**J**) Immunohistochemistry of TAT-hSPAR-C detected by immunohistochemistry with the anti-hSPAR antibody in the xenografts from panel (Fig. 7I) ($n = 12$ independent biological samples). Scale bar, 20 µm. (**K**) Immunohistochemistry of cell proliferation marker Ki67 in the xenografts from panel (Fig. 7I) ($n = 12$ independent biological samples). Scale bar, 20 µm. (**L**) Quantified relative levels of SLC38A2/GAPDH, P27KIP1/GAPDH, p-mTOR/mTOR, p-S6K/S6K and p-S6/S6 from panel (Fig. 7O) ($n = 12$ independent biological samples). Data are presented as the mean ± SEM and analyzed using two-tailed Student's *t* test with Welch's correction. Source data are available online for this figure.

 © The Author(s)
