# Supplementary material for: Micropeptide hSPAR regulates glutamine levels and suppresses mammary tumor growth via a TRIM21-P27KIP1-mTOR axis
Source: EMBO J. 2025 Jan 28;44(5):1414–41. doi: 10.1038/s44318-024-00359-z (PMC11876615; doi:10.1038/s44318-024-00359-z)
Supplement: Supplementary file 12 — Source data Fig. 7 [file 44318_2024_359_MOESM12_ESM.zip › Figure 7/Fig 7O/Fig 7O.pdf]

Fig 7O

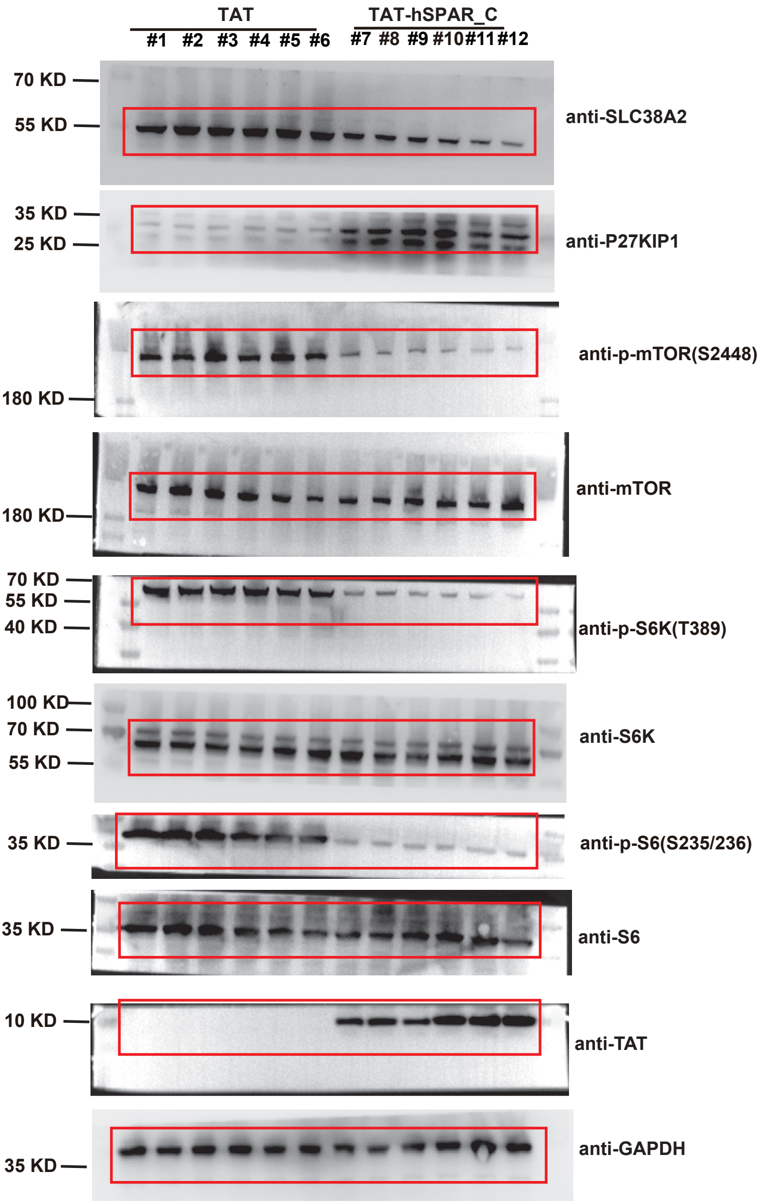
TAT
TAT-hSPAR_C
#1 #2 #3 #4 #5 #6 #7 #8 #9 #10 #11 #12
70 KD
55 KD
anti-SLC38A2
35 KD
25 KD
anti-P27KIP1
180 KD
anti-p-mTOR(S2448)
180 KD
anti-mTOR
70 KD
55 KD
40 KD
anti-p-S6K(T389)
100 KD
70 KD
55 KD
anti-S6K
35 KD
anti-p-S6(S235/236)
35 KD
anti-S6
10 KD
anti-TAT
35 KD
anti-GAPDH
