# Supplementary material for: Micropeptide hSPAR regulates glutamine levels and suppresses mammary tumor growth via a TRIM21-P27KIP1-mTOR axis
Source: EMBO J. 2025 Jan 28;44(5):1414–41. doi: 10.1038/s44318-024-00359-z (PMC11876615; doi:10.1038/s44318-024-00359-z)
Supplement: Supplementary file 13 — EV and Appendix Figure Source Data [file 44318_2024_359_MOESM13_ESM.zip › EV Figure Source Data.zip!/EV_Figure_1.zip!/EV Figure 1/EV Fig 1D/EV Fig 1D.pdf]

EV Fig 1D

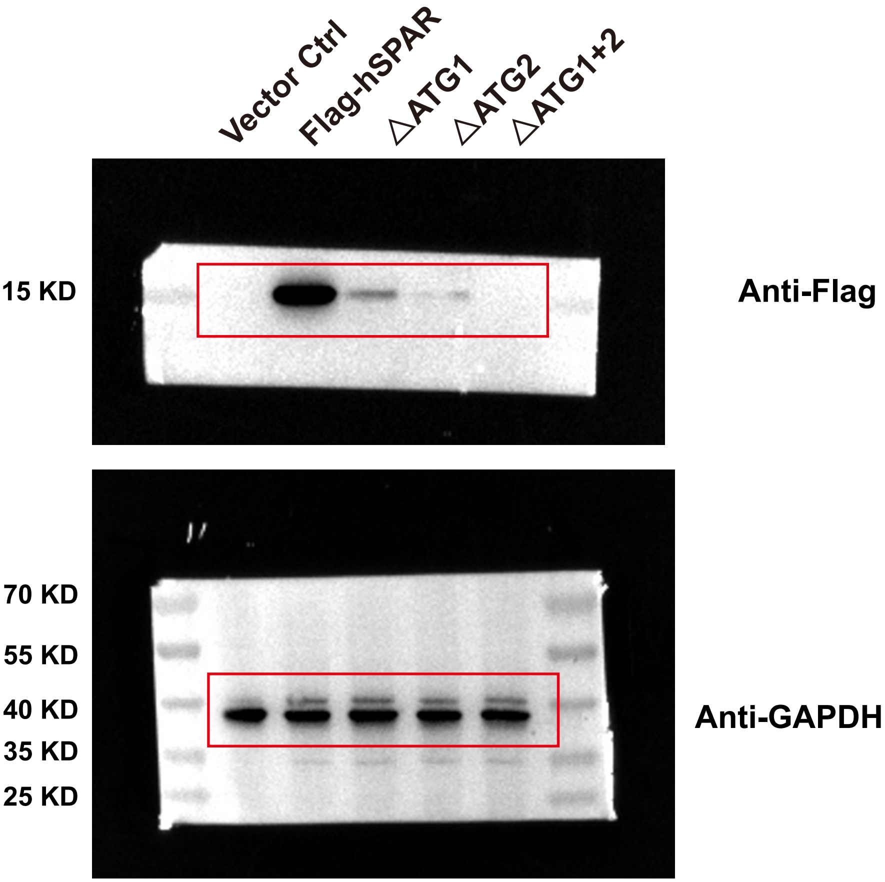
Vector Ctrl
Flag-hSPAR
△ATG1
△ATG2
△ATG1+2
15 KD
Anti-Flag
70 KD
55 KD
40 KD
35 KD
25 KD
Anti-GAPDH
