# Supplementary material for: Micropeptide hSPAR regulates glutamine levels and suppresses mammary tumor growth via a TRIM21-P27KIP1-mTOR axis
Source: EMBO J. 2025 Jan 28;44(5):1414–41. doi: 10.1038/s44318-024-00359-z (PMC11876615; doi:10.1038/s44318-024-00359-z)
Supplement: Supplementary file 13 — EV and Appendix Figure Source Data [file 44318_2024_359_MOESM13_ESM.zip › EV Figure Source Data.zip!/EV_Figure_2.zip!/EV Figure 2/EV Fig 2D and 2E/EV Fig 2D/EV 2D.pdf]

EV 2D

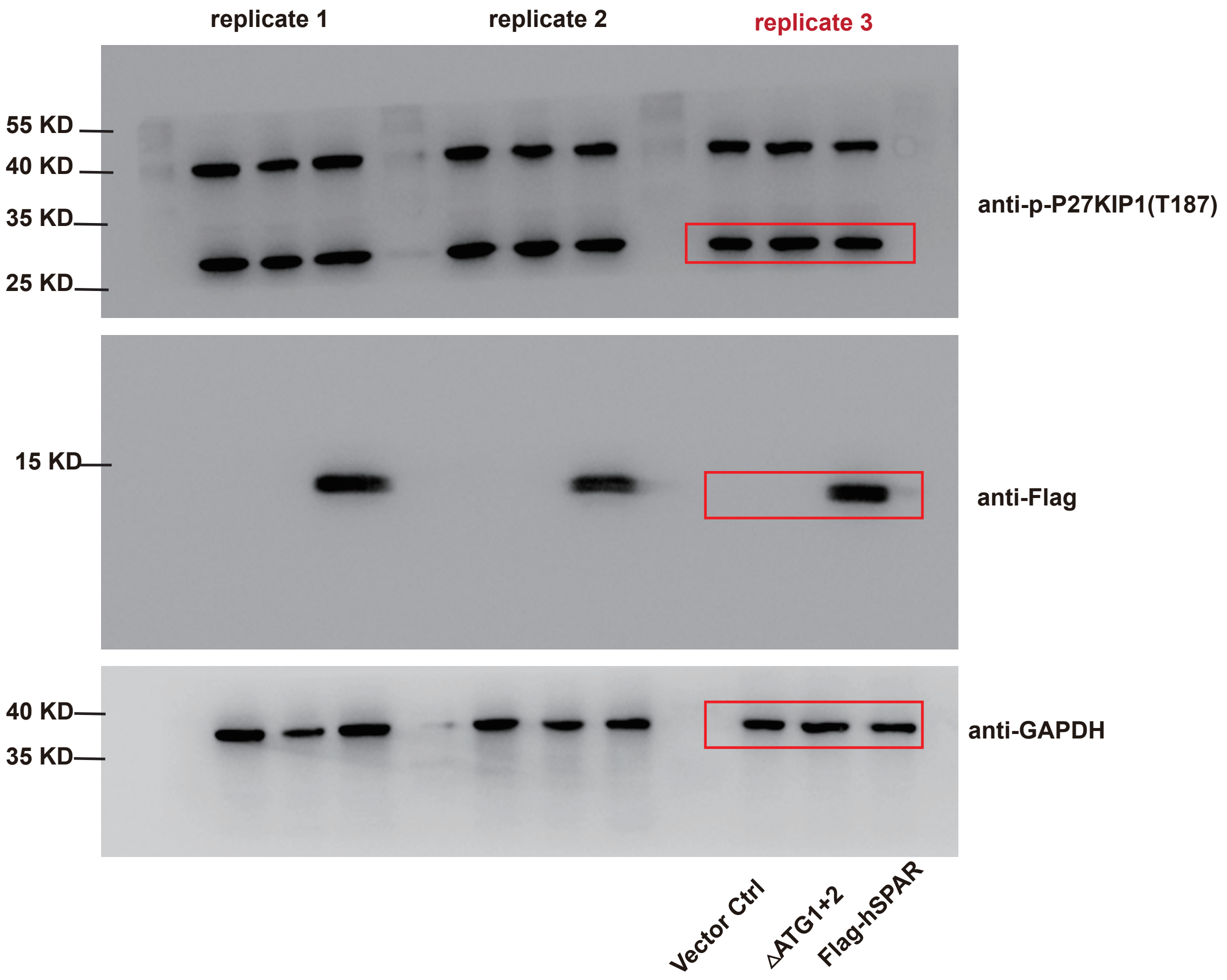
replicate 1
replicate 2
replicate 3
55 KD
40 KD
35 KD
25 KD
anti-p-P27KIP1(T187)
15 KD
anti-Flag
40 KD
35 KD
anti-GAPDH
Vector Ctrl
△ATG1+2
Flag-hSPAR
