# Supplementary material for: Micropeptide hSPAR regulates glutamine levels and suppresses mammary tumor growth via a TRIM21-P27KIP1-mTOR axis
Source: EMBO J. 2025 Jan 28;44(5):1414–41. doi: 10.1038/s44318-024-00359-z (PMC11876615; doi:10.1038/s44318-024-00359-z)
Supplement: Supplementary file 13 — EV and Appendix Figure Source Data [file 44318_2024_359_MOESM13_ESM.zip › EV Figure Source Data.zip!/EV_Figure_2.zip!/EV Figure 2/EV Fig 2F/EV 2F.pdf]

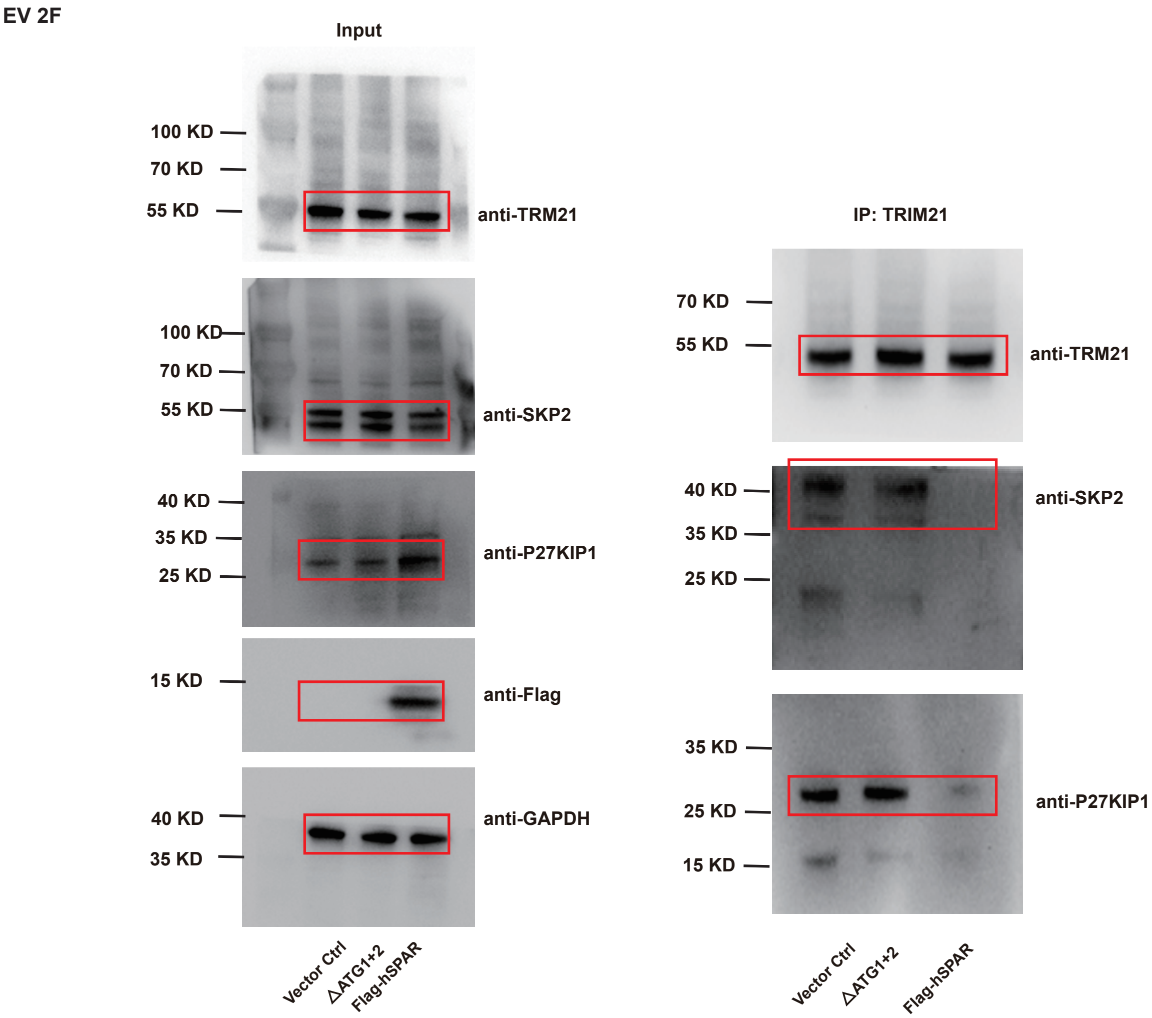
EV 2F
Input
100 KD
70 KD
55 KD
anti-TRM21
100 KD
70 KD
55 KD
anti-SKP2
40 KD
35 KD
25 KD
anti-P27KIP1
15 KD
anti-Flag
40 KD
35 KD
anti-GAPDH
Vector Ctrl
△ATG1+2
Flag-hSPAR
IP: TRIM21
70 KD
55 KD
anti-TRM21
40 KD
35 KD
25 KD
anti-SKP2
35 KD
25 KD
15 KD
anti-P27KIP1
Vector Ctrl
△ATG1+2
Flag-hSPAR
