# Supplementary material for: Micropeptide hSPAR regulates glutamine levels and suppresses mammary tumor growth via a TRIM21-P27KIP1-mTOR axis
Source: EMBO J. 2025 Jan 28;44(5):1414–41. doi: 10.1038/s44318-024-00359-z (PMC11876615; doi:10.1038/s44318-024-00359-z)
Supplement: Supplementary file 13 — EV and Appendix Figure Source Data [file 44318_2024_359_MOESM13_ESM.zip › EV Figure Source Data.zip!/EV_Figure_3.zip!/EV Figure 3/EV Fig 3C and 3D/EV Fig 3C/EV 3C.pdf]

EV 3C

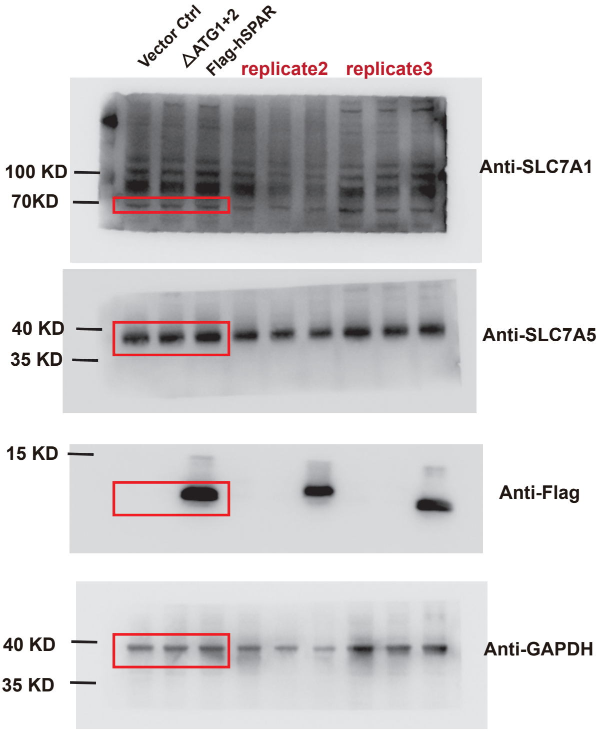
Vector Ctrl
ΔATG1+2
Flag-hSPAR
replicate2
replicate3
100 KD
70KD
Anti-SLC7A1
40 KD
35 KD
Anti-SLC7A5
15 KD
Anti-Flag
40 KD
35 KD
Anti-GAPDH
