# Supplementary material for: Micropeptide hSPAR regulates glutamine levels and suppresses mammary tumor growth via a TRIM21-P27KIP1-mTOR axis
Source: EMBO J. 2025 Jan 28;44(5):1414–41. doi: 10.1038/s44318-024-00359-z (PMC11876615; doi:10.1038/s44318-024-00359-z)
Supplement: Supplementary file 13 — EV and Appendix Figure Source Data [file 44318_2024_359_MOESM13_ESM.zip › EV Figure Source Data.zip!/EV_Figure_3.zip!/EV Figure 3/EV Fig 3E/Fig EV3E.pdf]

Fig EV3E

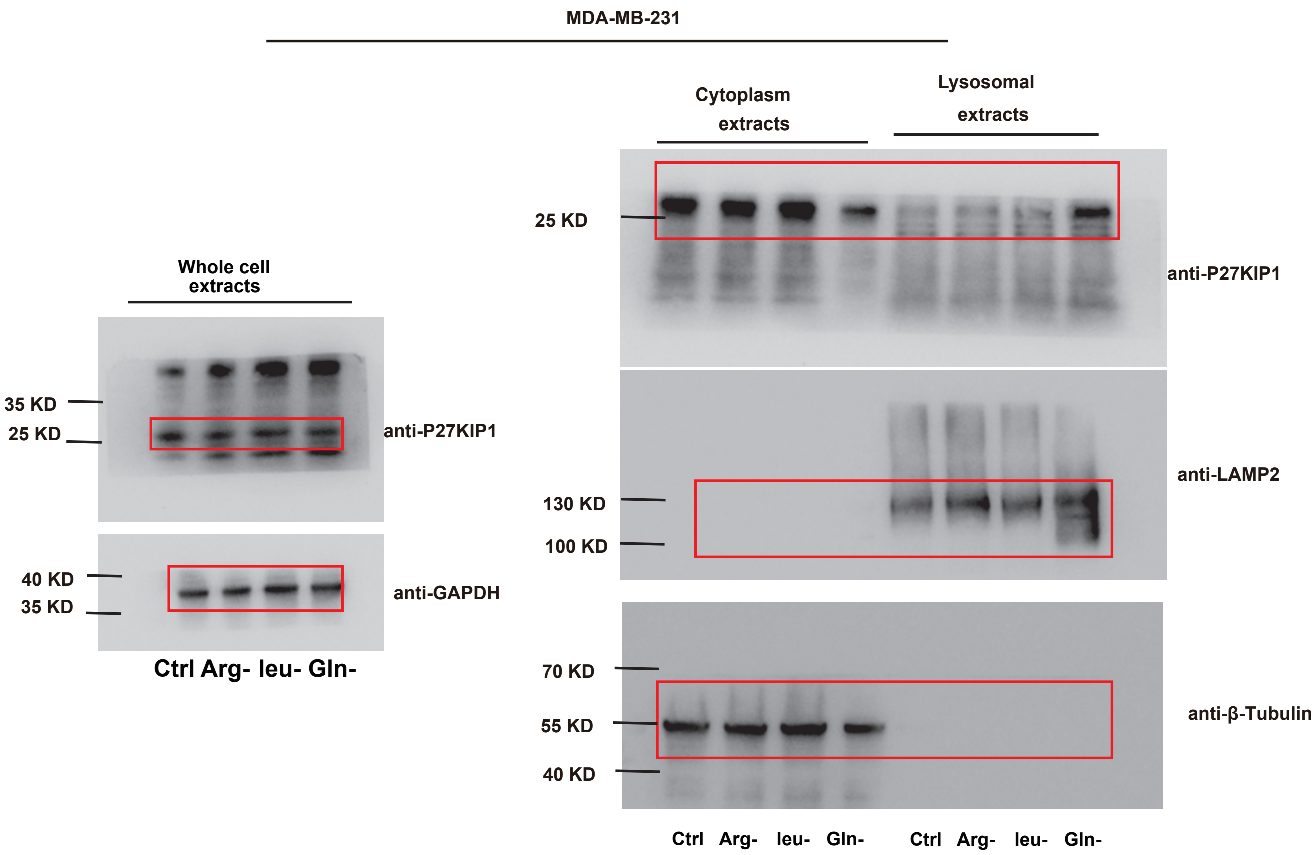
MDA-MB-231
Whole cell extracts
35 KD
25 KD
anti-P27KIP1
40 KD
35 KD
anti-GAPDH
Ctrl Arg- leu- Gln-
Cytoplasm extracts
Lysosomal extracts
25 KD
anti-P27KIP1
130 KD
100 KD
anti-LAMP2
70 KD
55 KD
40 KD
anti-β-Tubulin
Ctrl Arg- leu- Gln- Ctrl Arg- leu- Gln-
