# Supplementary material for: Micropeptide hSPAR regulates glutamine levels and suppresses mammary tumor growth via a TRIM21-P27KIP1-mTOR axis
Source: EMBO J. 2025 Jan 28;44(5):1414–41. doi: 10.1038/s44318-024-00359-z (PMC11876615; doi:10.1038/s44318-024-00359-z)
Supplement: Supplementary file 13 — EV and Appendix Figure Source Data [file 44318_2024_359_MOESM13_ESM.zip › EV Figure Source Data.zip!/EV_Figure_3.zip!/EV Figure 3/EV Fig 3F/Fig EV3F.pdf]

Fig EV3F

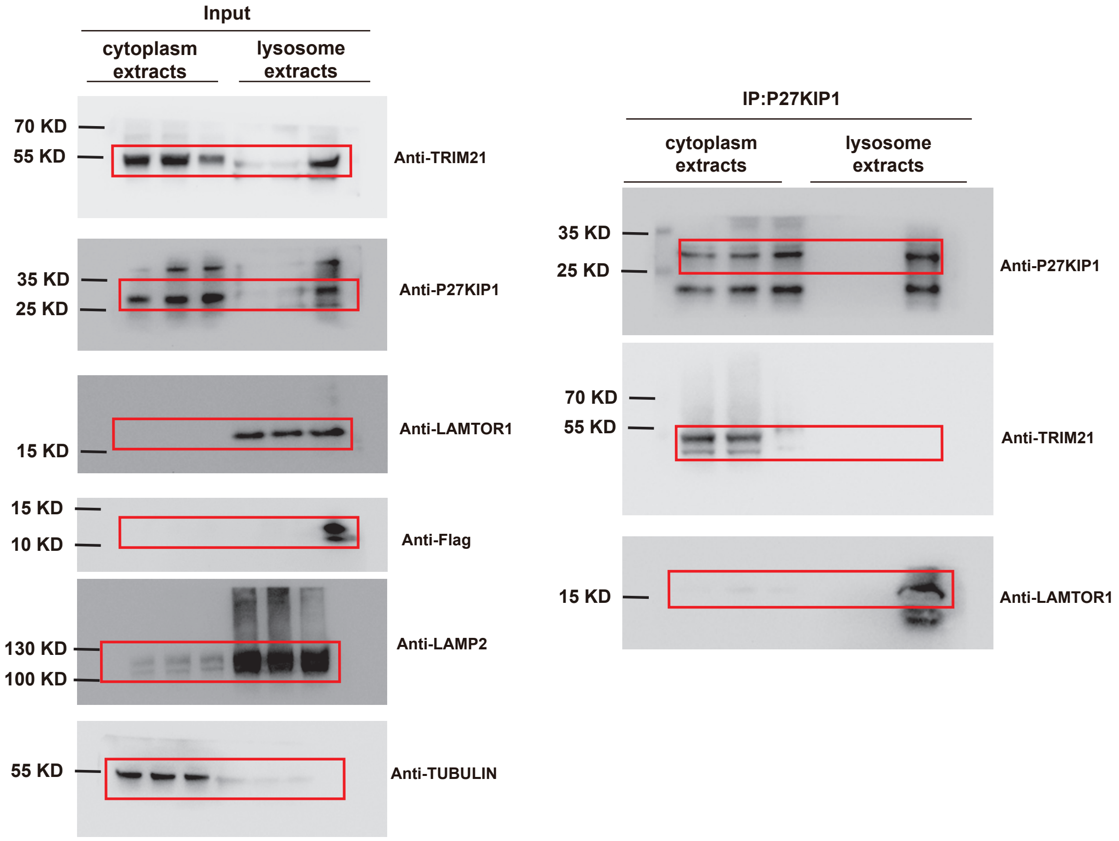
Input
cytoplasm extracts
lysosome extracts
70 KD
55 KD
Anti-TRIM21
35 KD
25 KD
Anti-P27KIP1
15 KD
Anti-LAMTOR1
15 KD
10 KD
Anti-Flag
130 KD
100 KD
Anti-LAMP2
55 KD
Anti-TUBULIN
IP:P27KIP1
cytoplasm extracts
lysosome extracts
35 KD
25 KD
Anti-P27KIP1
70 KD
55 KD
Anti-TRIM21
15 KD
Anti-LAMTOR1
