# Supplementary material for: Micropeptide hSPAR regulates glutamine levels and suppresses mammary tumor growth via a TRIM21-P27KIP1-mTOR axis
Source: EMBO J. 2025 Jan 28;44(5):1414–41. doi: 10.1038/s44318-024-00359-z (PMC11876615; doi:10.1038/s44318-024-00359-z)
Supplement: Supplementary file 13 — EV and Appendix Figure Source Data [file 44318_2024_359_MOESM13_ESM.zip › EV Figure Source Data.zip!/EV_Figure_4.zip!/EV Figure 4/EV Fig 4I and 4J/Fig EV4I/Fig EV4I.pdf]

Fig EV4I

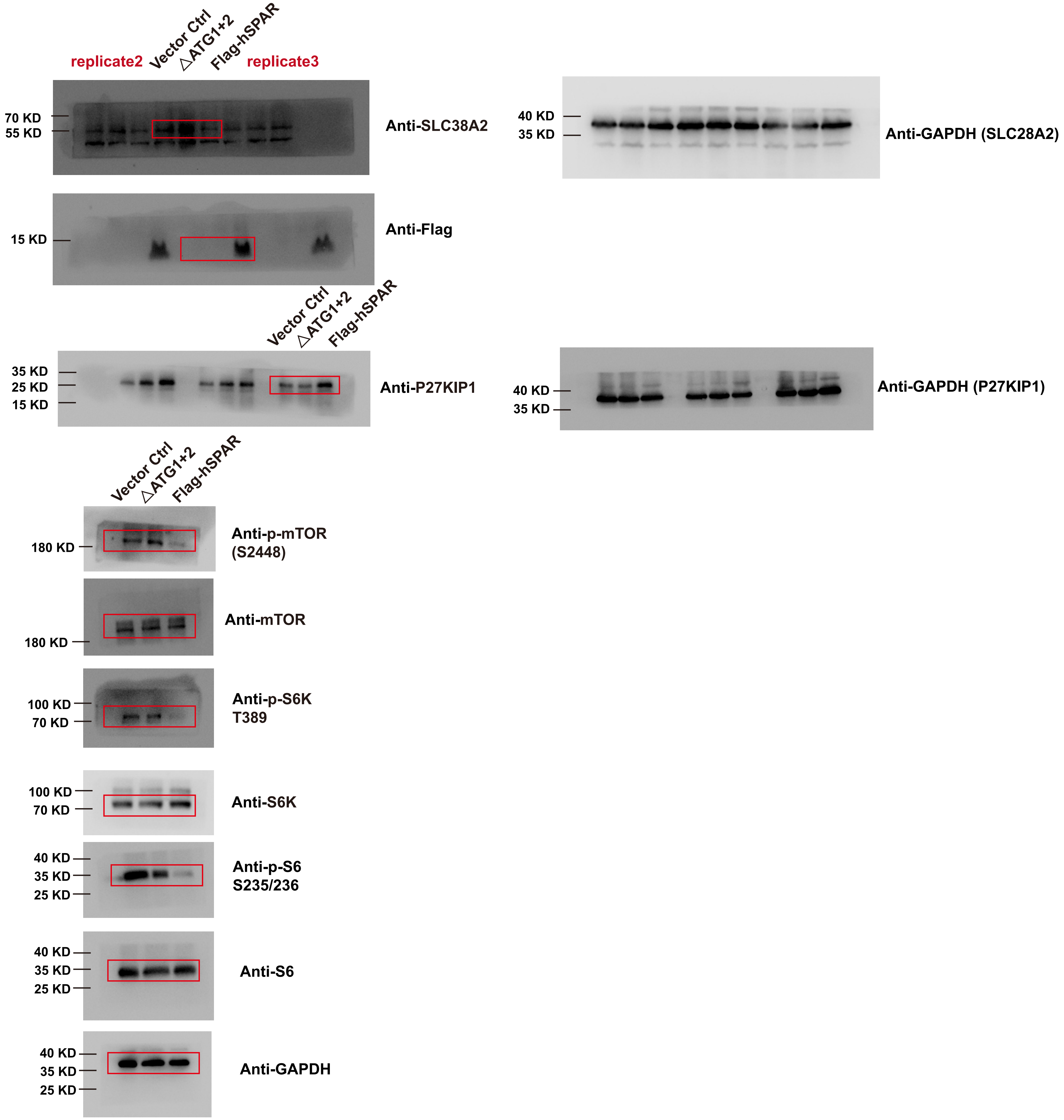

replicate2
Vector Ctrl
△ATG1+2
Flag-hSPAR
replicate3
70 KD
55 KD
Anti-SLC38A2
40 KD
35 KD
Anti-GAPDH (SLC28A2)
15 KD
Anti-Flag
Vector Ctrl
△ATG1+2
Flag-hSPAR
35 KD
25 KD
15 KD
Anti-P27KIP1
40 KD
35 KD
Anti-GAPDH (P27KIP1)
Vector Ctrl
△ATG1+2
Flag-hSPAR
180 KD
Anti-p-mTOR (S2448)
180 KD
Anti-mTOR
100 KD
70 KD
Anti-p-S6K T389
100 KD
70 KD
Anti-S6K
40 KD
35 KD
25 KD
Anti-p-S6 S235/236
40 KD
35 KD
25 KD
Anti-S6
40 KD
35 KD
25 KD
Anti-GAPDH
